# Core-genome-mediated promising alternative drug and multi-epitope vaccine targets prioritization against infectious *Clostridium difficile*

**DOI:** 10.1371/journal.pone.0293731

**Published:** 2024-01-19

**Authors:** Sara Aiman, Qurrat ul Ain Farooq, Zhongjie Han, Muneeba Aslam, Jilong Zhang, Asifullah Khan, Abbas Ahmad, Chunhua Li, Yasir Ali

**Affiliations:** 1 Faculty of Environmental and Life Sciences, Beijing University of Technology, Beijing, China; 2 Department of Biochemistry, Abdul Wali Khan University Mardan, Mardan, Pakistan; 3 Department of Biotechnology, Abdul Wali Khan University Mardan, Mardan, KP, Pakistan; 4 School of Biomedical Sciences, Chinese University of Hong Kong, Hong Kong, Hong Kong; The Islamia University of Bahawalpur Pakistan, PAKISTAN

## Abstract

Prevention of *Clostridium difficile* infection is challenging worldwide owing to its high morbidity and mortality rates. *C*. *difficile* is currently being classified as an urgent threat by the CDC. Devising a new therapeutic strategy become indispensable against *C*. *difficile* infection due to its high rates of reinfection and increasing antimicrobial resistance. The current study is based on core proteome data of *C*. *difficile* to identify promising vaccine and drug candidates. Immunoinformatics and vaccinomics approaches were employed to construct multi-epitope-based chimeric vaccine constructs from top-ranked T- and B-cell epitopes. The efficacy of the designed vaccine was assessed by immunological analysis, immune receptor binding potential and immune simulation analyses. Additionally, subtractive proteomics and druggability analyses prioritized several promising and alternative drug targets against *C*. *difficile*. These include FMN-dependent nitroreductase which was prioritized for pharmacophore-based virtual screening of druggable molecule databases to predict potent inhibitors. A MolPort-001-785-965 druggable molecule was found to exhibit significant binding affinity with the conserved residues of FMN-dependent nitroreductase. The experimental validation of the therapeutic targets prioritized in the current study may worthy to identify new strategies to combat the drug-resistant *C*. *difficile* infection.

## 1.0. Introduction

*Clostridium difficile* is a gram-negative anaerobic bacterium causes pseudomembranous colitis and life-threatening diarrhea [1, 2]. *C*. *difficile* is associated with hospital-acquired infections with high rates of morbidity and mortality worldwide, leading to epidemic in certain parts of the world [3]. *C*. *difficile* infections (CDIs) mainly develop in elderly individuals because of disruption of the protective colon microbiota after antimicrobial therapy [4, 5]. Multiple factors contribute to the increasing number and severity of CDIs. The main risk factors associated with CDIs include prior antibiotic treatment, which leads to disrupted intestinal flora [6, 7]. Hospitalized patients are at maximum risk after the first month of antibiotic therapy [8]. Other risk factors include proton-pump inhibitors (PPIs), chemotherapy [9], and lack of response to toxins, mostly in older individuals. Individuals colonized with *C*. *difficile* spores are likely to serve as infection reservoirs that contaminate the environment with *C*. *difficile* spores [8]. Bacteria with a pathogenic locus (Paloc) expressing a binary toxin (toxin A = enterotoxin, toxin B = cytotoxin) are toxigenic strains, that cause colonic mucosal damage, resulting in actin filament degradation [10, 11]. Mutations in the toxin repressor gene (tcdC) have been reported to produce 15 times more toxin A and B hypervirulent strains i.e. BI/NAP027 and ribotype 027, that exhibit resistance to fluoroquinolones [12–15]. Multi-component biofilm development is also a substantial contributor to *C*. *difficile* drug resistance. The multi-layered biofilm is a dense matrix composed of DNAs, proteins, and polysaccharides [16].

The frequent occurrence of CDIs has increased the rate of emergence of multiple antibiotic-resistant (MAR) and hypervirulent strains during the last two decades [17, 18]. The rapid increase in the MAR bacterial strains is a major global health concern in the 21^st^ century. Currently, it is estimated that over 700,000 annual deaths are caused by MAR pathogens worldwide, and death toll predicted to reach up to 10 million by 2050 [19]. *C*. *difficile* is classified as an urgent threat according to 2019 Antibiotic Resistance Report of the Centers for Disease Control and Prevention (CDC) [19, 20]. *C*. *difficile* resistant is reported to commonly prescribed antibiotics, including erythromycin, penicillin, tetracycline, clindamycin, lincomycin, cephalosporins, aminoglycosides, clindamycin, gentamicin, imipenem, moxifloxacin, rifampicin, and fluoroquinolones [21, 22]. Multiple studies have reported an increase of up to 90% in the antibiotic resistance of *C*. *difficile* in the past decade [23]. Worldwide surveillance reported that the irrational use of antibiotics majorly contributes to the increase in *C*. *difficile* resistance which led to the occurrence and reoccurrence of CDIs. The high rate of CDI recurrence is a serious problem; approximately 25% of treated individuals develop recurrence, and approximately 40% to 60% of individuals experience subsequent recurrence after their first recurrence of CDI [24, 25]. Antibiotic resistance of *C*. *difficile* results in suboptimal clinical outcomes, leading to CDI treatment failure. Current preventive measures are insufficient to treat CDIs, and there is no commercially available vaccine that provides protection against CDIs. Therefore, novel therapeutic strategies are required to combat CDIs, and prevent its subsequent recurrence.

Microbial pan-genomics has sparked the interest of the scientific community owing to the availability of whole genome sequence data of pathogenic strains [26–28]. The core proteome data of pathogenic strains provide primary information to identify novel therapeutic targets in pathogenic species. Combined bioinformatics, chemo-informatics, and immunoinformatic techniques have rapidly increased the identification of potential drug and vaccine targets against pathogenic strains [29–31]. Advancements in genomics and proteomics have facilitated the identification of suitable targets for functional medicines. Subtractive proteomics and vaccinomics is a novel approach that facilitates the identification of potential therapeutic targets against specific pathogenic strains [32]. The purpose of this work was to use comparative proteomic techniques to identify therapeutic drug and vaccine candidate proteins by focusing on the core proteome of *C*. *difficile* drug-resistant strains. Reverse vaccinology and immunoinformatic strategies were implemented for the prediction of immunodominant epitopes from the prioritized vaccine candidates to design a highly immunogenic vaccine construct. The efficacy of proposed vaccine construct was evaluated via immune-simulation analysis. Subtractive proteomics approach was utilized to predict potential drug target proteins. Further, pharmacophore-based modeling, virtual screening, molecular docking, and molecular dynamic simulation analyses were performed to identify novel inhibitors against the top-prioritized drug targets. *In silico* ADME studies were done to investigate the pharmacokinetic properties of the lead inhibitory compounds.

## 2.0. Methodology

**Fig 1:**
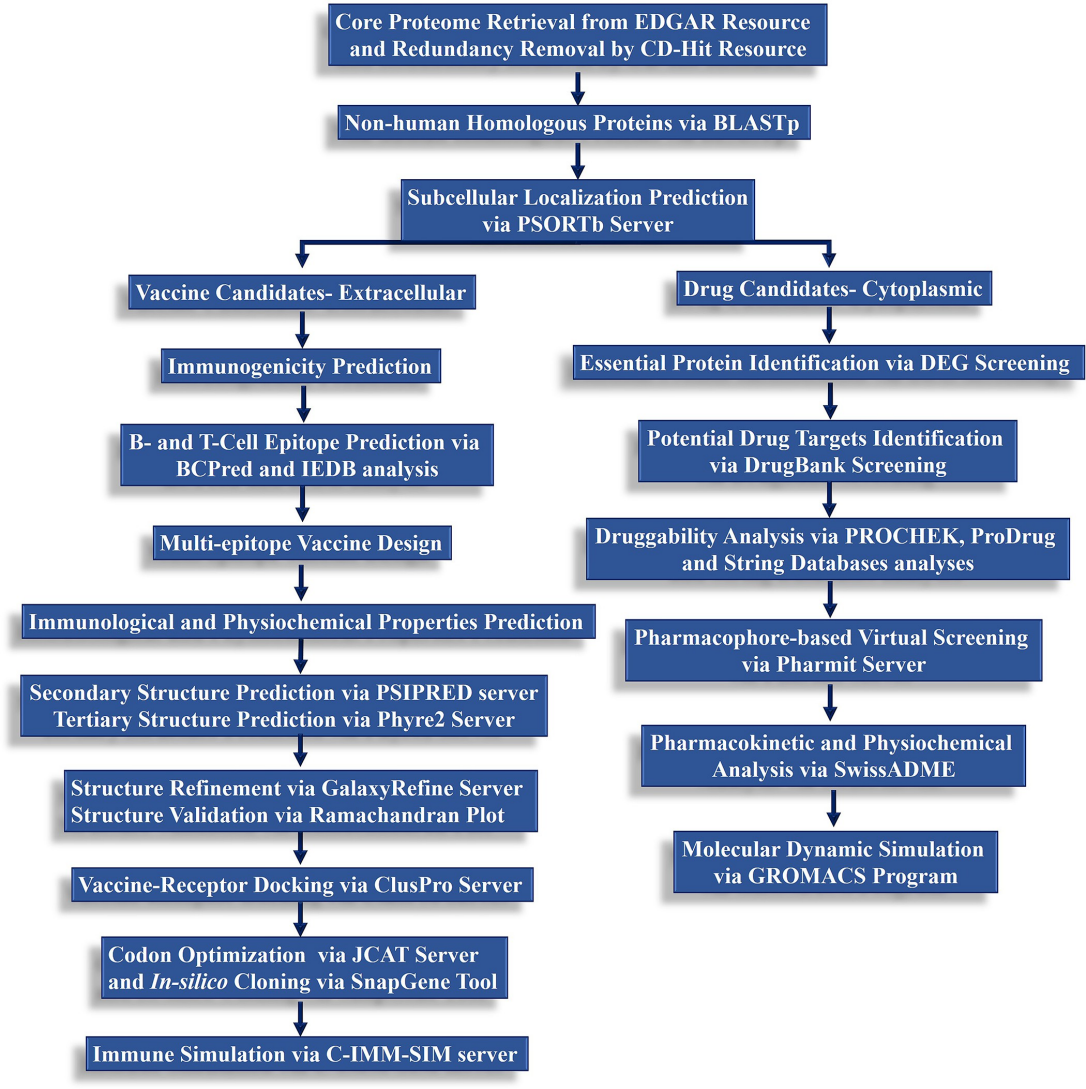
The methodological layout to prioritize drug and vaccine targets in the core proteome of *C*. *difficile* via comparative and subtractive proteogenomic analysis, immunoinformatics approaches, and druggability analyses.

All the steps and methodological layout of the current study is summarized in Fig 1.

### 2.1 Core proteome retrieval

The core proteome of *C*. *difficile* (NCBI Taxonomy ID: 1496) was retrieved from EDGAR version 3 web resource (http://edgar3.computational.bio) [33]. The genome of the *C*. *difficile* strain 630 was selected as a reference genome. Paralogous sequences were determined using the standalone version of CD-HIT resource [34] with a similarity index of 60% [35]. Redundant sequences were discarded to acquire non-paralogous sequences for further analysis. Besides, sequences with less than 100 amino acid residues were also discarded. The PSORTb v3.0.2 web tool (http://www.psort.org/psortb) was used for determining the subcellular localization of non-paralogous pathogen proteins [36].

### 2.2 Reverse vaccinology

#### 2.2.1 Vaccine candidates’ identification

Outer membrane and extracellular proteins were further prioritized based on their allergenicity, antigenicity, and toxicity parameters. The allergenicity assessment of human non-homologous pathogen proteins was performed by AllerTOP v2.0 webserver (http://www.ddg-pharmfac.net/AllerTOP) [37]. VaxiJen v2.0 webserver (http://www.jenner.ac.uk/VaxiJen) was used to evaluate the antigenicity level of the prioritized bacterial proteins with a threshold of >0.4 [38]. ToxinPred2 webserver (https://webs.iiitd.edu.in/raghava/toxinpred2/) was utilized to determine the toxicity of the prioritized bacterial proteins [39].

#### 2.2.2 Assessment of B-cell, MHC-I, and MHC-II epitopes

The prioritized bacterial proteins were further subjected to multiple immunoinformstics tools to predict the potential B- and T-cell epitopes that can induce strong immune responses in the host immune system. T-cell epitopes are represented by major histocompatibility complex (MHC) molecules. MHC class I epitopes are recognized by CD8+ and MHC class-II epitopes are recognized by CD4+ T cells [40]. The Immune Epitope Database (IEDB) analysis resource was used for the prediction of T-cell epitopes in the prioritized pathogen proteins [41]. Stabilized Matrix Method (SMM) prediction approach was utilized to identify 9-mer MHC-I epitopes [42] and the NetMHCIIpanEL 4.1 prediction algorithm was used to predict 15-mer MHC-II epitopes [43]. We used the entire collection of human HLA reference set. Top binding overlapping epitopes were prioritized with an IC_50_ value of <200 nM [35, 44]. ABCPred webserver (http://codes.bio/abcpred/) was used to predict B-cell epitopes with a cut-off value of >0.5, and other default parameters. The server detects linear B-cell epitopes that induce humoral immune responses and stimulate B lymphocytes [45]. The effective synthesis of a vaccine construct is affected by the variations in the distribution and expression of human HLA alleles among different ethnic groups and various regions of the world [46]. The population coverage of the prioritized MHC-I and MHC-II epitopes was determined by the IEDB population coverage tool. The tool calculates population coverage based on the distribution of HLA-binding alleles of each prioritized epitope in multiple global regions [47]. The IEDB epitope conservancy tool was employed to assess the conservation of the prioritized MHC-I and MHC-II epitopes among various *C*. *difficile* strains.

#### 2.2.3 Multi-epitope-based vaccine engineering

Overlapping B- and T-cell epitopes were prioritized and further examined for their allergenicity, antigenicity, and toxicity parameters. The overlapping epitopes were also screened against human epitopes to avoid autoimmune responses. The non-homology threshold was set at an E-value >0.05 [48]. The top-lead overlapping epitopes were used to design a multi-epitope chimeric vaccine, using different adjuvant and linker peptide sequences, to enhance the immunogenicity of the designed vaccine construct [49]. The Pan DR Helper T Epitope (PADRE) sequence was also incorporated in the vaccine construct to avoid polymorphisms of HLA-DR molecules in case of different populations [50]. The “HEYGAEALERAG” and “GGGS” linkers were used to conjugate the prioritized overlapping epitopes [42]. The prioritized overlapping B- and T-cell epitopes in the designed vaccine model ensure the generation of both cell-mediated and humoral immune responses against the antigenic peptide vaccine construct [44]. EAAAK linker was used to connect the adjuvant i.e., β-defensin at the N-terminus of the multi-epitope chimeric vaccine construct. A strong immunostimulatory adjuvant enhances the immunogenicity of the designed vaccine construct and activates long-term innate and adaptive immunity against pathogens [51].

#### 2.2.4 Immunological and physicochemical properties assessment

The designed multi-epitope vaccine construct with various combinations of epitopes was subjected to immunogenic analysis. The AlgPred webserver (https://webs.iiitd.edu.in/raghava/algpred2/) was used to assess the allergenic behavior of the designed vaccine construct [47]. The VaxiJen v2.0 [38] and ANTIGENpro (http://scratch.proteomics.ics.uci.edu) [52] webservers were used for antigenicity assessment of the vaccine construct. These servers evaluate the antigenicity based on the principal amino acid properties using auto cross covariance (ACC) transformation [38] and 10 fold cross validation of the peptide sequence against known protein data files [53]. Likewise, the solubility of the vaccine construct was calculated by SOLpro webserver [54]. The ProtParam program on the ExPASy server (https://web.expasy.org/protparam/) was used to determine the various physicochemical parameters of the vaccine construct [55].

#### 2.2.5 Secondary and tertiary structures prediction, refinement, and validation

Secondary structure prediction of the designed vaccine construct was performed using PRISPRED 4.0 (http://globin.bio.warwick.ac.uk/psipred/) [56] and SOPMA (http://www.ibcp.fr/predict.html) [57] servers. The servers use position-specific scoring matrices to predict the transmembrane topology, transmembrane helix, and recognition of folds and domains in the protein sequence. The tertiary structures of the multi-epitope vaccine constructs were generated by Phyre2 web tool (http://www.sbg.bio.ic.ac.uk/phyre2) [58] and further refined by the GalaxyRefine web program (http://galaxy.seoklab.org/refine) [59]. Subsequently, the refined tertiary structure was validated by the ERRAT tool, Ramachandran plot of PROCHECK suite [60] (https://saves.mbi.ucla.edu/), and ProSA-Web [61] server (https://prosa.services.came.sbg.ac.at).

#### 2.2.6 Vaccine-receptor docking analysis

ClusPro is a protein-protein docking server (https://cluspro.org) that was used to study molecular interactions between the designed vaccine construct with human HLA and TLR receptors to determine receptor-vaccine interactions [62]. The refined vaccine constructs were docked against HLA-A*11–01 (PDB ID: 5WJL), MHC-II allele HLA DRB1*04–01 (PDB ID: 5JLZ), TLR2 (PDB ID: 6NIG), and TLR4 (PDB ID: 3FXI) receptor molecules. The vaccine-receptor docked complex with the lowest docking score was prioritized for immunological validation. The molecular interactions between the residues in the vaccine-receptor complex were determined using the PDBsum programme (https://www.ebi.ac.uk/thornton-srv/databases/pdbsum/) [63].

#### 2.2.7 *In Silico* immune simulation

The C-ImmSim web program (http://www.cbs.dtu.dk/services/C-ImmSim-10.1/) was used for the computational immune simulations of the multi-epitope vaccine to assess the immunogenic potential of the designed vaccine [64]. The server employs multiple machine learning methods to predict the potential stimuli of the host immune system and provides information on humoral and cellular responses to the antigen [65]. The standard clinical protocol calls for a four-week interval between two vaccine doses [66]. We followed the protocol previously used by Aslam et al., 2021 to carry out the immune simulation of the designed chimeric vaccine construct [44]. The simulation was run for 1, 84, and 168 hours with the default settings. The immune system was simulated for a thousand times using the HLA-A*0101 and A*0201, HLA-B*0702 and B*3901, HLA-DRB1*0101, and DRB1*0401 antigens.

#### 2.2.8 *In Silico* codon optimization and *in-silico* restriction cloning

The prioritized multi-epitope vaccine construct was subjected to codon optimization and *in-silico* restriction cloning. Java Codon Adaptation Tool (JCAT) (http://www.prodoric.de/JCat) server was utilized for reverse translation of the peptide sequence to cDNA. The codons were optimized to achieve the maximum expression of vaccine in the bacterial expression system [67]. The maximum possible expression potential of the cloned vaccine gene was calculated by the codon adaptation index (CAI) and percentage GC content. The optimal CAI value reported for favorable transcriptional and translational efficacy is 0.8–1, while the optimum GC content is 30%-70% [42, 68]. The optimized prioritized vaccine gene was computationally cloned in the *E*. *coli* vector using the Snapgene (https://www.snapgene.com/) software. The Addgene server (https://www.addgene.org/) was used to retrieve the *E*. *coli* plasmid pET28a_TIAL1 for computational restriction cloning [69].

#### 2.3 Subtractive proteomics and druggability analysis

The human proteome (Taxonomy ID: 9606) and the human-gut proteome (retrieved from TiD database [70]) were searched for homologs of the non-paralogous pathogenic protein sequences using the standalone BLASTp tool [71], with an E-value cut off of 10^−4^, bit score ≤100, query coverage ≤35%, and sequence identity ≤35%. In-house python-based tool was used to filter out homolog protein sequences based on set parameters. The pathogen-specific protein sequences were further screened against DEG (Database of Essential Genes) [72] via BLASTp to identify the pathogen essential proteins involved in different metabolic pathways. Human non-homologous pathogen essential proteins in the cytoplasmic regions were subjected to druggability analysis to identify potential drug targets and further used to identify potent inhibitors against *C*. *difficile* infections. The DrugBank database was screened to identify novel drug target in the shortlisted proteins via BLASTp parameters set at an E-value cut off of 10^−4^, Bit score <100, query coverage ≤35%, and sequence identity ≤35%. At the threshold values, the non-hit proteins were selected as potential drug targets. Protein Data Bank (PDB) database [73] was curated to determine experimentally validated 3D-structures of the shortlisted *C*. *difficile* proteins. The 3D-structures of the shortlisted *C*. *difficile* proteins were predicted by the SWISS-MODEL web tool [74] with <90% sequence homology with the PDB database entries. 3D-structural validation was carried out using the PROCHECK suite of programs [60]. Putative active sites in these protein structures were determined using the PockDrug server [75]. STRING database v10.5 [76] was used to determine the protein-protein interaction (PPI) and hub proteins identification. Proteins with a high average node degree (K≥5) were considered as hub proteins. The hub proteins are of critical importance for the survivability of pathogens.

#### 2.3.1 Pharmacophore-based virtual screening

The Pharmit webserver [77] was utilized for designing pharmacophore model, using the crustal structure of putative nitroreductase in complex with fmn (Flavin Mononucleotide) (cd3205) acquired from *C*. *difficile* 630 at 1.35 Å resolution (PDB: 3GFA). A pharmacophore is a spatial arrangement of essential features of an interaction within a molecular structure. The server is based on state-of-the-art sub-linear algorithm to screen millions of compounds in a large number of compound databases i.e., MolPort, ZINC, PubChem, and ChEMBL. Pharmit uses AutoDock Vina scoring function to evaluate pharmacophore models, molecular structures, and energy minimization [77, 78]. The pharmacophore model was constructed based on 8 pharmacophore features (2 hydrogen donors, 5 hydrogen acceptors, and 1 hydrophobic). The resultant hit compounds list was further minimized to obtain the top most significant molecules among the millions of compounds available in the Pharmit repository.

#### 2.3.2 Molecular docking and ADME profiling

The drug-like behavior of the top 10 hit compounds was evaluated by Lipinski’s rule of five [79]. Molecular docking analysis determines the binding orientations and affinities of the drug-like compound with the receptor protein [80]. The top 10 hit drug-like compounds were subjected to molecular docking analysis with the target protein via CB-Dock server [81]. CB-Dock improves the accuracy of the predicted binding site of the target protein and binding poses of the query ligand using a novel curvature-based cavity detection approach (CurPocket) and performs docking via the AutoDock Vina program [81]. Protein-ligand interaction analysis and visualization were performed by Discovery Studio Visualizer v.4.5 Client version (Accelrys, San Diego, CA, USA). The ADME (Absorption, Distribution, Metabolism, and Excretion), physicochemical, and pharmacokinetic properties were examined using SwissADME web resource [82].

#### 2.3.3 Molecular dynamic simulation

The highly effective ligand prioritized after ADME profiling was subjected to molecular dynamic (MD) simulations. MD simulation was performed to evaluate the stability, flexibility, hydrogen bond interaction, and inhibitory potential of small drug-like compounds. The GROMACS 2019.2 software was used for MD simulation [83]. The parameters used for simulations were GROMOS96 43a1 force-field, solvation with TIP4P water model, cubic boundary box type calculated through the buffer and NVT and NPT ensemble for 100 ns trajectory at 1 bar pressure and 300K temperature [50]. Finally, the trajectory analyses were calculated for root-mean-square distance (RMSD), root-mean-square fluctuation (RMSF), radius of gyration (Rg), and hydrogen bonds to study the interaction pattern of ligand and dynamic fluctuation in protein [84].

## 3.0 Results

### 3.1 Core proteome retrieval

The core proteome of *C*. *difficile* retrieved from EDGAR yielded a total of 42,700 protein sequences. The removal of redundant or paralogous sequences resulted in 2,113 non-paralogous pathogen core proteins (S1 File).

### 3.2 Subcellular localization

PSORTb categorized the non-paralogous pathogen proteins into their corresponding regions, including the cytoplasm, periplasm, intermembrane space, outer membrane, and extracellular spaces (Table 1). Cytoplasmic proteins were regarded as the best drug candidates, whereas surface proteins were prioritized for model vaccine constructs prioritization.

**Table 1:** Subcellular localization of *C*. *difficile*-specific non-redundant proteins.

S.No	Cellular localization	Number of Proteins
1.	Cytoplasmic	1226
2.	Cytoplasmic Membrane	502
3.	Extracellular	17
4.	Cell-wall	27
5.	Unknown	341

### 3.3 Reverse vaccinology

#### 3.3.1 Vaccines candidates identification

Core proteins located on the outer membrane and extracellular regions of cells were prioritized for the vaccinomics study. The antigenicity of the prioritized proteins was predicted by the Vaxijen v2.0 webserver with 79–89% accuracy [38]. Four proteins (CD630_18220, CD630_27870, CD630_16310, and CD630_10170) were prioritized based on immunological parameters (Table 2). These proteins were subjected to B- and T-cell epitopes prediction for *in silico* chimeric vaccine designing. Vaccine candidate proteins were selected based on antigenicity, allergenicity, and toxicity parameters.

**Table 2:** Vaccine candidate proteins finalized based on antigenicity, allergenicity, and toxicity parameters.

Protein IDs	Protein Names	Allergenicity	Antigenicity VaxiJen v2.0 (Threshold >0.4)	Toxicity
CD630_18220	thiol peroxidase	Non-Allergen	0.5455	Non-Toxin
CD630_27870	cell surface protein	Non-Allergen	0.4669	Non-Toxin
CD630_16310	superoxide dismutase	Non-Allergen	0.4338	Non-Toxin
CD630_10170	multidrug family ABC transporter ATP-binding protein/permease	Non-Allergen	0.4630	Non-Toxin

#### 3.3.2 MHC-I, MHC-II, and B-cell epitopes prediction

The B- and T-cell epitopes were predicted from vaccine candidate proteins to design potential multi-epitope vaccine constructs against *C*. *difficile*. The lead MHC-I and MHC-II epitopes for the prioritized proteins were selected based on an IC_50_ values <200 nM. BCpred scores greater than 0.8 and 75% specificity were used to predict overlapping B-cell epitopes. Overlapping lead epitopes were selected from each prioritized vaccine candidate protein based on non-allergenic, non-toxic, and high antigenic nature. Overall, 12 such epitopes were prioritized to design a highly immunogenic vaccine construct (S1 and S2 Tables). The main objective was to determine key overlapping epitopes capable of eliciting cell-mediated and humoral immune responses. Overlapping epitopes were scanned against human peptides to eliminate homologous epitopes based on the E-values. Conservation of the prioritized epitopes was ensured to be 100% in multiple *C*. *difficile* strains. The conserved epitopes are assumed to provide broader protection against various *C*. *difficile* strains [85]. The IEDB results revealed that the prioritized epitopes showed >97% coverage of the worldwide populations; however, the population coverage of some epitopes was high in various regions of the world (S1 Fig and S3 Table).

#### 3.3.3 Multi-epitope chimeric vaccine designing

**Fig 2:**
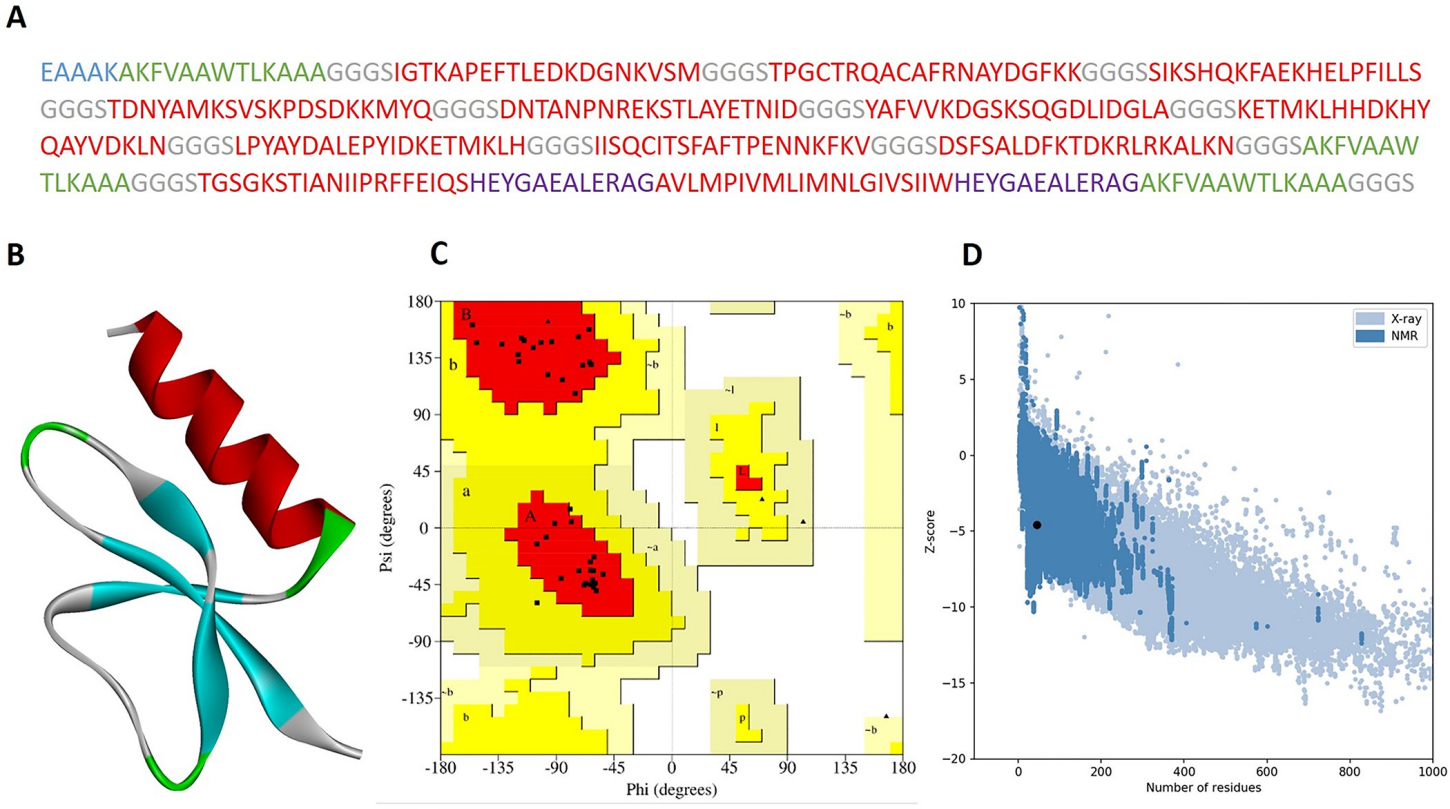
Vaccine design, 3D-structure prediction, and validation. (A) Multi-epitope chimeric vaccine construct. (B) Refined 3D-structure of the vaccine construct. (C) Ramachandran plot with 94.7% of residues in the favored region of the plot. (D) ProSA-web plot representing the z-sore (-4.57) of the refined vaccine construct.

A multi-epitope-based chimeric vaccine was designed using the prioritized human non-homologous overlapping immunodominant epitopes. The GGGS and HEYGAEALERAG linkers were used to join the selected epitopes. These linkers are responsible for the structural stability to the vaccine construct and allow each epitope to independently perform its protective role in human body after the vaccine administration [86]. The epitopes were linked to beta-defensin peptide sequence at the N-terminus using EAAAK linkers to enhance immunogenic responses. The consequences of HLA-DR variation in various groups have been countered by incorporating PADRE peptide sequences into the vaccine design. It has been previously reported that PADRE peptide-containing vaccine models provide enhanced immunity and robust cytotoxic T lymphocyte (CTL) responses against antigens [87] (Fig 2A).

#### 3.3.4 Immunogenicity and phycicochemical properties assessment

The immunological properties determined a highly antigenic, non-allergenic, and non-toxic nature of the designed vaccine construct. Antigenicity scores of 0.882658 and 1.0433 calculated using ANTIGENpro and VaxiJen 2.0, respectively, indicate the substantial antigenic nature of the designed chimeric vaccine construct. An AlgPed score of -0.47191405 indicated that the designed chimeric vaccine construct exhibited non-allergenic behavior. The solubility score of 0.619589 predicts high solubility of the chimeric vaccine upon expression. The ProtParam web tool was employed to predict various physiochemical properties of the chimeric vaccine. The optimal molecular weight of the vaccine construct was calculated to be ~43 KDa which indicates easy purification of the vaccine. The grand average of hydropathicity (GRAVY) value was -0.376 and aliphatic index was 66.56, indicating hydrophilic nature of the chimeric vaccine construct. The Instability Index score of 32.70 predicts the stability of the construct. Immunological and physicochemical properties of the construct indicate the capabilities of the designed vaccine construct to elicit significant immunogenic responses.

#### 3.3.5 Secondary and tertiary structures prediction, refinement, and validation

The secondary structure elements of the chimeric vaccine depicted 36.94% α-helices, 12.50% β-sheets, 29.17% coils, and 21.39% extended strands (S2 Fig). The presence of α-helical coil-coiled domains in the multi-epitope chimeric vaccine construct is critical in order to enable proper protein folding based on standard protein structures, and confers an effective humoral immunity in response to a specific pathogen [88]. The tertiary structures of the chimeric vaccine constructs were predicted by Phyre2 web tool, refined using the GalaxyRefine server (Fig 2B), and further validated by Ramachandran plot. The ERRAT quality score was calculated 100%, Ramachandran plot revealed that 94.7% of the residues appeared in the core region of the plot (Fig 2C), and the z-sore of -4.57 of the refined vaccine constructs determined by ProSA-web plot represented a high-quality 3D structure of the vaccine construct (Fig 2D). The high structure validation scores of the proposed vaccine indicated a prediction with a topology of greater accuracy.

#### 3.3.6 Vaccine-receptor docking analysis

**Fig 3:**
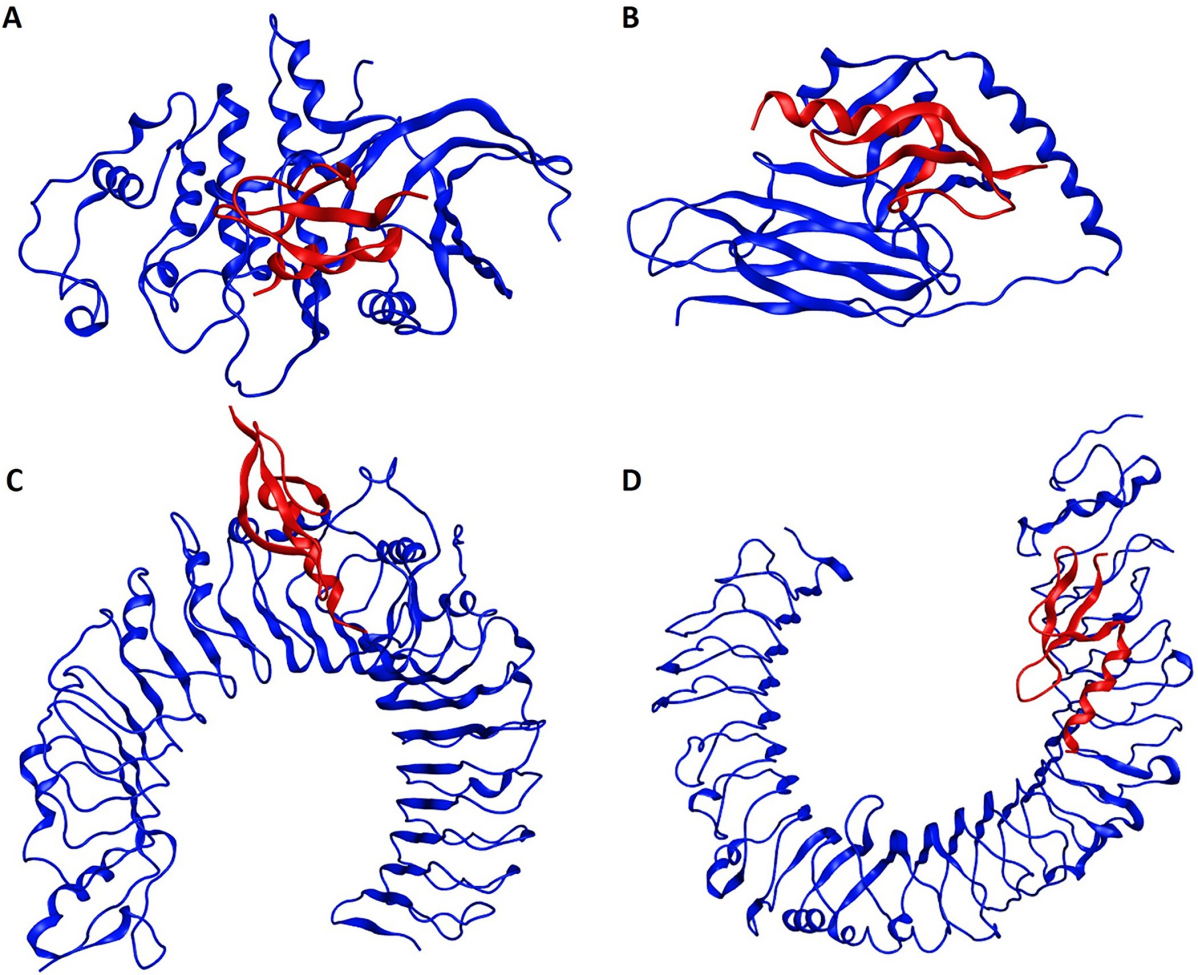
Molecular docking of the multi-epitope chimeric vaccine construct with the human HLA and TLR immune receptors. The blue color represents receptor molecule and the red color represents vaccine construct. (A) Vaccine construct and HLA (PDB ID: 5WJL) docking complex with a global energy of -780.2 kcal/mol. (B) Vaccine construct and HLA (PDB ID: 5JLZ) docking complex with a global energy of -1029.4 kcal/mol. (C) Vaccine construct and TLR2 (PDB ID: 6NIG) docking complex with a global energy of -949.6 kcal/mol. (D) Vaccine construct and TLR4 (PDB ID: 3FXI) docking complex with a global energy of -751.9 kcal/mol.

Molecular docking analysis was carried out to evaluate the binding promiscuity of the designed vaccine with human HLA and TLR immune receptors. ClusPro webserver was used for rigid body protein-protein docking based on billions of confirmations sampling, RMSD-based clustering, and structural refinement based on energy minimization. The docking analysis revealed that the vaccine construct exhibited lowest binding energy of -780.2 kcal/mol with HLA-A*11–01 (PDB ID: 5WJL) and -1029.4 kcal/mol with MHC-II allele HLA DRB1*04–01 (PDB ID: 5JLZ), -949.6 kcal/mol with TLR2 (PDB ID: 6NIG), and -751.9 kcal/mol with TLR4 (PDB ID: 3FXI) (Fig 3). The docking results demonstrated that the vaccine design was capable of developing several molecular interactions with HLA and TLR receptors binding sites. The lowest docking energy scores showed the highest binding affinity of vaccine construct with human immune receptors. These findings speculate the capability of vaccine to trigger potent immune responses when administered to a human host. The molecular interactions between the residues of the vaccine-MHC-I complex demonstrated 12 hydrogen bonds, 5 salt bridges, and 146 non-bonded interactions. Vaccine-MHC-II complex showed 7 hydrogen bonds, 3 salt bridges, and 148 non-bonded interactions. The interactions between vaccine-TLR2 complex revealed 7 hydrogen bonds, 3 salt bridges, and 148 non-bonded contacts, whereas 9 hydrogen bonds, 1 salt bridge, and 117 non-bonded interactions were observed between the residues of vaccine-TLR4 complex (S4 Table and S3 Fig).

#### 3.3.7 Computational immune simulation

**Fig 4:**
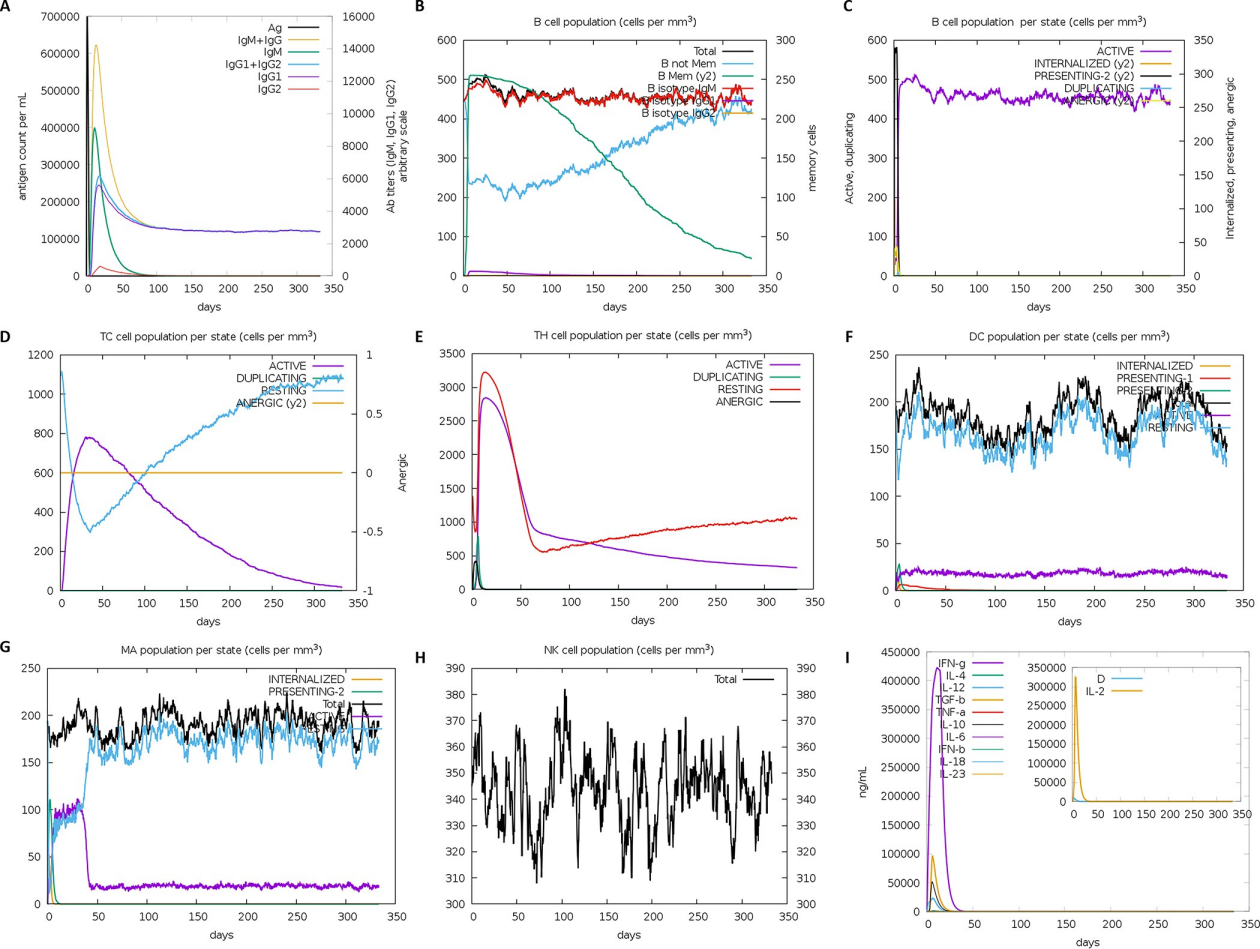
*In-silico* immune simulation of the chimeric vaccine peptide determined by C-ImmSim server. (A, B) A significant rise of the B-cell populations and high levels of immunoglobin antibodies, with a reduction in antigen levels. (C) The rising B-cell population with repeated antigen exposure. (D, E) The increase in helper and cytotoxic T-cell population with repeated antigen exposure. Dendritic cell, macrophage, and natural killer cell populations increase during immunization period. (I) Increased cytokine concentrations following repeated antigen exposure.

The immune simulation results predicted a substantial increase in the primary and secondary responses caused by the prioritized multi-epitope chimeric vaccine construct. Theoretically, this pattern is consistent with the generation of a real-time immune response. The primary simulated responses showed elevated IgM levels. The simulation of secondary and tertiary immune responses showed momentous escalation of B-cells population and increase in the levels of IgG1, IgG2, IgM, and IgM+IgG antibodies were observed, while antigen levels decreased (Fig 4A and 4B). These results suggest immunological memory generation, as evidenced by an increase in memory B-cell population and isotype switching. As a result, repeated exposures to chimeric antigen led to a precipitous drop in antigen levels (Fig 4C). After repeated antigen exposure, the number of cytotoxic (TC) and helper (TH) cells increased, and associated memory responses were predicted to initiate (Fig 4D and 4E). Additionally, macrophage, dendritic cell, and natural killer cell populations were stimulated and kept at high levels throughout the immunization period (Fig 4F–4H). Moreover, higher levels of an interleukin like IL-2 and a cytokine like IFN-y were observed (Fig 4I). These results indicated that the proposed vaccine construct may elicit significant immune responses against the pathogen.

#### 3.3.8 Codon optimization and *in-silico* cloning

The top-ranked vaccine construct was subjected to codon optimization and *in silico* restriction cloning to evaluate the expression potential of the proposed vaccine model. JCAT revealed that the GC content of the optimized cDNA sequence of the prioritized vaccine construct was predicted to be 67.96% with a significant CAI value of 0.95 (S5 Table and S4 Fig). These values are in the optimum range i.e. GC content 30%-70% and CAI index of 0.8–1.0 [67], suggesting that the vaccine construct had a high expression potential. The codon sequence of the proposed vaccine was successfully inserted into the pET28a_TIAL1 vector to ensure heterologous cloning and expression of the vaccine in the *E*. *coli* expression system (S5 Fig).

### 3.4 Subtractive proteomics and druggability analysis

**Fig 5:**
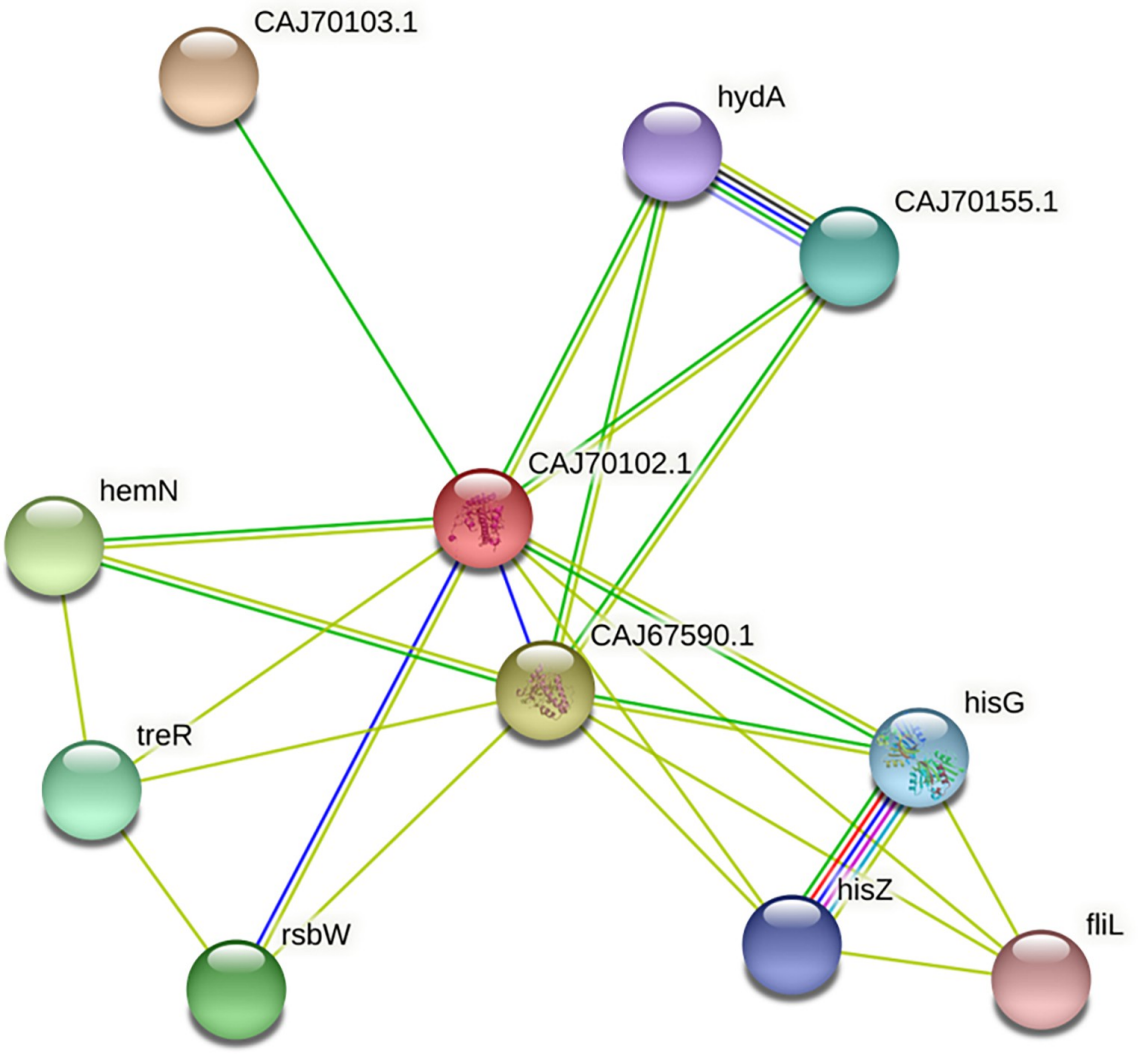
Protein-protein interaction of pathogen protein CD630_32050 (putative nitroreductase) with PDB ID: 3GFA acquired from STRING database. Red color indicated the hub protein.

The non-paralogous proteins were subjected to additional screening against proteins from the human proteome and the human gut microbiome, which resulted in 610 non-homologous hits (S2 File). The proteins homologous to human and human gut microbiome proteins were discarded as they might cause immuno-metabolic disorders in humans. Screening of DEG resource yielded 385 *C*. *difficile* core proteins, showing significant sequence similarity with the essential proteins in the database (S3 File). These human non-homologous and *C*. *difficile* essential proteins might be promising drug and vaccine targets against *C*. *difficile*-mediated infection. Based on the subcellular localization (Table 1), the cytoplasmic proteins were prioritized as suitable drug targets. Finally, 184 proteins were prioritized as suitable drug candidate proteins during downstream druggability analysis. These proteins showed no homology with the DrugBank database entries. The PDB database was manually screened for the availability of 3D structures of these proteins. Among the 184 proteins, the putative nitroreductase in complex with fmn (cd3205) structural information was available (PDB: 3GFA). The PPI analysis via STRING v10.5 database predicted the putative nitroreductase as a hub protein with the node degree (K) ≥5 (Fig 5), representing the high number of molecular interactions. Among the rest of the proteins targets the top 100 proteins were prioritized based on PockDrug probability scores > 0.5 and their 3D structures were designed using the SWISS-MODEL server [74]. The 3D-structure evaluation indicated the high quality of these proteins based on the ERRAT tool, QMEAN Z-scores [89], and RAMPAGE scores [60] (S6 Table). These top drug targets were thought to have promising druggable pockets that could serve as anchors for small drug-like compounds and play critical roles as hub proteins in *C*. *difficile*.

#### 3.4.1 Pharmachophore modeling, virtual screening, and molecular docking

**Fig 6:**
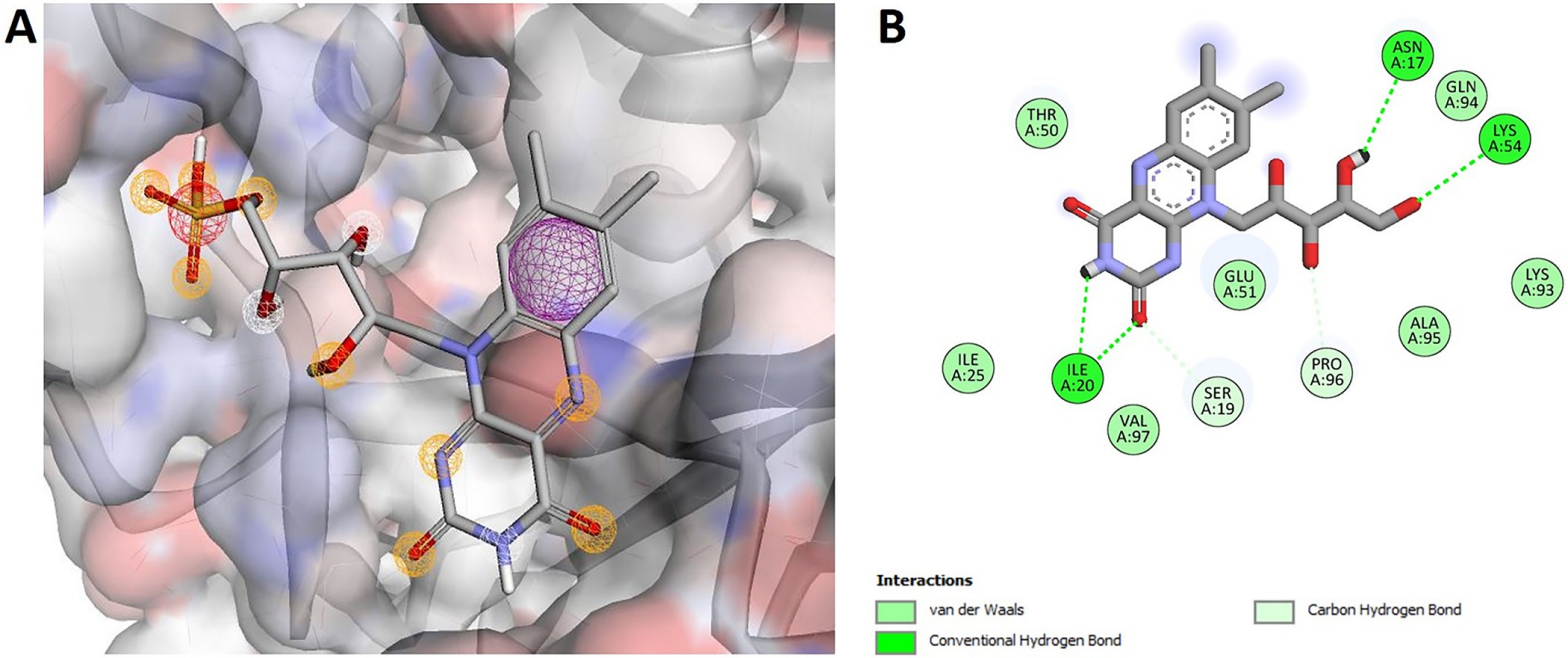
(A) The active site of the protein CD630_32050 was used to create a pharmacophore model. The characteristics are denoted by different colors. White represents a hydrogen-bond donor, yellow represents a hydrogen acceptor, green represents hydrophobic properties, and aromatic represents aromatic features (pink). (B) The molecular interactions of the top hit docked compound (CD630_32050) within the substrate-binding site. The nature of protein-ligand interactions is shown in different colors.

Among the shortlisted drug-target candidate proteins, putative nitroreductase (PDB: 3GFA) was prioritized for pharmacophore-based virtual screening to identify small drug-like compounds. The obtained pharmacophore model based on the 3D-structure of a putative nitroreductase from Pharmit server showed 8 pharmacophore features i.e., two hydrogen donors, five hydrogen acceptors and one hydrophobic feature as shown in Fig 6. The top ten screened molecules were prioritized based on lowest binding energy after being docked repeatedly (S7 Table). The scores and Root Mean Square Distance (RMSD) values of the selected compounds are shown in Table 3. FMN- dependent nitroreductase is the member of a structurally homologous family. The enzyme bounded FMN cofactor mediates the electron transfer from NAD(P)H to proceed the first step of the ping-pong Bi-Bi reaction pathway. The crystal structure of this enzyme shows the binding position of the FMN cofactor is highly conserved i.e., residues 10–25 and 40–55. In the present investigation, this data was used in a pharmacophore-based computational screening procedure. The top hit compound i.e. MolPort-001-785-965 showed interactions with the residues in the conserved region, i.e. Asn17, Ser19, Thr50, Glu51, and Lys54 of the nitroreductase. Besides, the top hit compound was involved in interactions with the flexible region (residues 90–134) (Fig 6A), which is a part of cofactor and substrate binding site [90]. Moreover, the docking analysis showed that all the top 10 compounds exhibited interactions with the residue in the conserved region of the nitroreductase. The top 10 hit compounds were further subjected to molecular docking analysis to re-evaluate the binding conformation of the lead compounds within the active site of the receptor molecule (Fig 6B). These drug-like compounds might have a potential to combat the *C*. *difficile-*mediated infection and may be worthy of consideration in drug discovery and development [91].

**Table 3:** Pharmit scores and RMSD values of the top 10 hit compounds obtained from pharmacophore-based virtual screening using the Pharmit server.

Compounds	(MolPort IDs)	Pharmit Score	RMSD Values
C1	MolPort-044-559-927	-8.57	1.1096
C2	MolPort-044-724-190	-8.57	1.065
C3	MolPort-003-939-021	-8.50	0.843
C4	MolPort-021-783-318	-8.00	0.803
C5	MolPort-039-136-733	-7.72	1.364
C6	MolPort-003-934-329	-7.36	0.807
C7	MolPort-001-785-965	-7.34	0.898
C8	MolPort-004-964-255	-6.92	1.135
C9	MolPort-003-666-643	-6.83	0.955
C10	MolPort-044-561-302	-6.68	0.941

#### 3.4.2 Drug likeliness and ADME analysis

SwissADME provides detailed information on the physicochemical properties, ADME, and medicinal potential of a compound. Drug likeliness is an intricate balance between structural and molecular properties of a compound that shows a certain degree of similarity with a known drug molecule. The drug likeliness of a compound is calculated based on different molecular parameters, such as molecular weight, hydrophobicity, hydrogen bonding, reactivity, electron distribution, pharmacophore entity, bioavailability, molecular stability, and toxicity [92]. Lipinski’s rule of 5 is the most commonly used models to evaluate a therapeutic drug-like compound based on its solubility and permeability [79]. The four major pharmacokinetic parameters of a drug-like compound are absorption, distribution, metabolism, and excretion (ADME). The compound with the best molecular interaction and binding energy with the receptor protein may not be the most suitable drug. A reliable drug should be completely and rapidly absorbed in the gastrointestinal tract, directly distributed towards the respective target, metabolized in a way that does not affect its activity, and ultimately be eliminated without causing any harm [93]. The pharmacokinetic properties of a molecule can be calculated using chemical descriptors as there is a significant relationship between the chemical structure and physicochemical properties.

The pharmacokinetics and pharmacological properties of the top ten Pharmit hits were calculated to evaluate the efficacy of these compounds as drugs (S7 Table). The extremely hydrophobic compounds are poorly soluble in the gastrointestinal tract and solvate fat globules [94]. Based on physicochemical properties, all the compounds exhibited drug-like properties i.e., holding high solubility with the molecular weight of less than 500 Da. According to the ADME results, these compounds showed low gastrointestinal absorption and did not penetrate the blood-brain barrier (BBB). Compounds C3, C4, C5, and C8 are substrates for the p-glycoprotein, which is associated with pumping xenobiotics and detrimental components back into the gut lumen, hence pharmacokinetically decreasing the efficacy of being a potential drug [95]. ADME profiling ensured no mutagenicity or toxicity for all these drug-like compounds. Among these compounds, C1, C2, C3, C4, C5, C8, and C10 showed Lipinski violations, whereas C6, C7, and C9 did not show any Lipinski’s rule violation, making them ideal drug-like molecules based on *C*. *difficile* nitroreductase inhibition. However, the rest of the lead compounds may require extra chemical transformation to have potential drug-like nature (S8 Table).

#### 3.4.3 Molecular dynamic simulations

**Fig 7:**
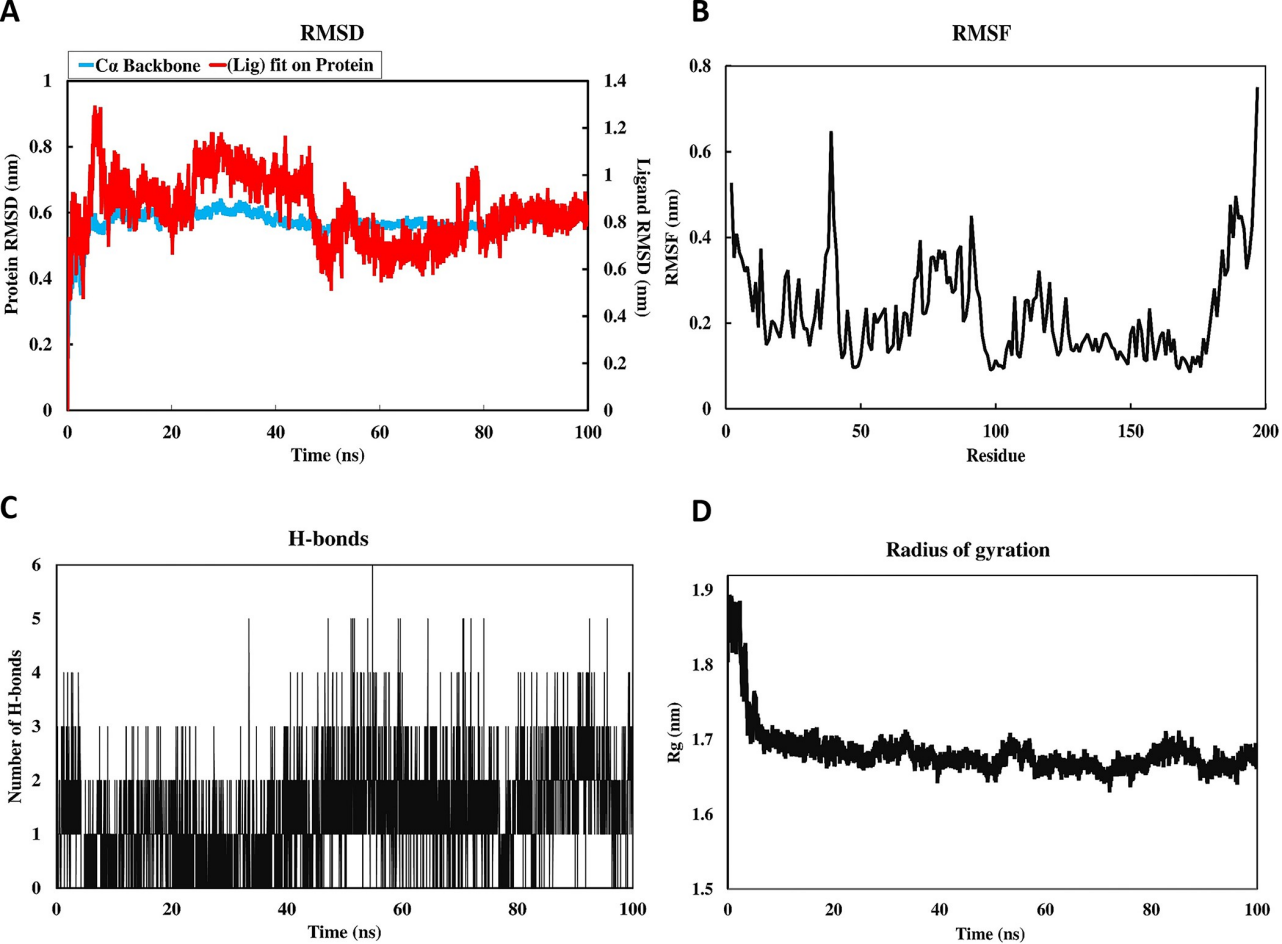
Molecular dynamics (MD) simulation results A) C7-putative nitroreductase RMSD analysis B) RMSF analysis of Cα atoms C) H-bond estimation during 100 ns simulation D) Radius of gyration (Rg) analysis.

The MD simulation analyses were performed for the prioritized nitroreductase-C7 complex docked models to validate the stability of molecular interactions and flexibility of the complex [96]. The RMSD values putative nitroreductase-C7 complex rapidly increased from 0.9 nm in the starting and maintained the equilibrium at 0.6 nm between 25 to 45 ns. The system was observed to gain stability at 0.6 nm after 48 ns which remained constant throughout the remaining simulation time (Fig 7A). In this analysis, it was observed that the complex had some fluctuations in ligand up to 45 ns, while it was observed to be completely stable after 50 ns. According to the RMSF calculations, the complex fluctuated maximally up to 0.62 nm and did not exceed further because of the rigidity of the structure (Fig 7B). Most of the residues in the protein exhibited an RMSF value less than 0.4 nm, indicating that the ligand does not undergo any significant conformational change over the simulation time. Hydrogen bonding between a ligand and receptor is essential for stabilizing the ligand-protein complex. Fig 7C displays the total number of hydrogen bonds formed in the complex, calculated after 100 ns of simulation time. Analysis of the H-bonds revealed that the ligand in the complex had established up to 6 H-bonds. It can be concluded that the ligand bound effectively and tightly to the putative nitroreductase. The Rg enables the measurement of the compactness fluctuations of a ligand-protein complex [42]. The average Rg value of putative nitroreductase was calculated as approximately 1.7 most of the time during the simulation (Fig 7D). This result indicated that the value of Rg remained steady throughout the MD simulation, suggesting stable protein folding. Compared to the MD simulation results of the protein and the protein-ligand complex, the RMSF values indicated that the residues from 90–100 interacting with the C7 were showing lower and smoother fluctuations as compared to the corresponding part of the monomer protein (S6 Fig). The results are in good agreement with the binding modes of the complex structure, ensuring the stability of the complex.

## 4.0 Discussion

The emergence of antibiotic-resistant strains and the recurrence of CDIs represent an urgent threat to the clinical challenges in public health. The treatment options for moderate to severe cases are extremely limited. No commercial vaccine is available, and a limited number of drugs have been proven effective against CDIs. New therapeutic strategies are required to tackle multi-drug resistant strains of *C*. *difficile*. Active vaccination provides the best opportunity to prevent CDIs in high-risk individual. In the recent years, intense research led to the development of experimental vaccines against CDIs, which are currently in clinical trials. Recombinant peptide vaccines and toxoid-based vaccines have been proven promising in healthy adults; however, challenges associated with such vaccination strategy remained in elderly and immunocompromised individuals [97]. Moreover, the peptide sequence variability from different *C*. *difficile* isolates raised the question of their ability to provide broader protection against CDIs. Therefore, more efforts are required to optimize the immunization strategies to enhance the efficacy of vaccines against CDIs. Core genome-mediated analysis is a promising approach for the identification of potential therapeutic targets in *C*. *difficile* as the core genome is conserved specie-wise. In this study, we utilized comparative genome analysis to identify pathogen-specific core drugs and vaccine target proteins. Furthermore, we used immunoinformatics and vaccinomics approaches to design a multi-epitope chimeric vaccine construct that can generate specific immunogenic responses against *C*. *difficile*.

We prioritized four proteins located in the outer membrane and extracellular regions as potent vaccine targets, based on high immunological properties. Highly conserved B-cell, MHC-I, and MHC-II epitopes were determined from the prioritized proteins to design a highly immunogenic vaccine construct against *C*. *difficile* following the reverse vaccinology techniques. Immune enhancers and adaptable adjuvant peptides were used to bind together overlapping B- and T-cell epitopes. To induce a targeted immune response against the pathogen while limiting negative responses towards the host, multi-epitope-based vaccine designing is a unique therapeutic method [98]. Immunological properties unveiled high antigenicity, non-allergenic behavior, and non-toxic properties of the designed vaccine construct. The small molecular weight and hydrophilicity score of the vaccine construct determined highly solubility of the vaccine upon expression. Moreover, the stability score indicates that the expressed vaccine construct will be of high quality and ready for use. The tertiary structural analysis demonstrated a maximum number of residues in the favorable region of the Ramachandran plot, confirming the structural stability of the design vaccine construct. Immune simulation results validated the resemblance to actual cellular immune responses [29]. Strong immunogenic responses were triggered by repeated exposure to the same antigen. The clear development of memory B cells and T cells would last for several months, with the memory in the B cells. After repeated antigen exposure, the consistent high level of Ig production, T-cytotoxic, and T-helper cells indicated a humoral response. The molecular docking investigation further indicated that the design vaccine construct exhibited strong molecular interaction with human HLA and TLR immune receptors. These results indicate that the designed vaccine has the capability of inducing strong immunogenic responses against *C*. *difficile* infections and might be worthy of *in vitro* and *in vivo* investigations to confirm the findings of the current study.

In addition, we employed subtractive genome analysis, druggability analysis, and virtual screening approaches to identify novel inhibitors of *C*. *difficile* drug targets. Cytoplasmic proteins were prioritized as suitable drug candidates and were further scanned against the entries of DrugBank database for potential therapeutic drug targets identification. Non-hit proteins indicate that they have not yet been established as drug targets for *C*. *difficile*. According to the centrality-lethality law, inhibiting the activity of such proteins may be critical for the survival of pathogens [99, 100]. The prioritized druggable proteins were non-homologous to both human and human gut proteomes. In addition, objectives were set using rigorous threshold criteria for fundamental druggability characteristics. Drug designing for such specific targets will only affect the pathogen without disrupting the biological mechanism of the host. Among the prioritized druggable targets, the crystal structure of nitroreductase (i.e. CD630_07560) is available in the PDB database (PDB: 3GFA). Therefore, it was prioritized for the virtual screening of a drug-like molecule to identify novel drug-like compounds that inhibit the activity of this enzyme. FMN- dependent nitroreductases belong to a group of flavoenzymes, that are oxygen-insensitive and are essential for the reduction of nitro compounds in the presence of NAD(P)H [101]. Numerous bacterial species have been shown to contain nitroreductases. The physiological functions of these enzymes are unclear, although nitroreductases are believed to play an important role in the responses to a variety of antibiotics, specific oxidative stress conditions, and environmental chemical threats [102]. The expression of nitroreductase genes might be controlled by the MarRA and SoxRS regulatory systems. Studies have reported that nitroreductases have conserved domains for FMN binding, NAD(P)H electron transfer, and nitroaromatic-substrate interactions. Moreover, nitroreductases are consistent with the enzyme’s ping–pong bi–bi catalytic mechanism, which explains the broader substrate specificity of these enzymes and establishes the groundwork for improving their activity for medicinal applications [103]. Nitroreductase is also involved in antibiotic resistance and bacterial pathogenesis [104]. For instance, nitroreductase has previously been reported in *Enterobacter cloacae* and *salmonella enterica* to be involved in resistance against chloramphenicol [104]; however, no such reports are available concerning *C*. *difficile*.

The top ten hit compounds in this study were riboflavin derivatives, among which C6, C7, and C9 followed all the Lipinski’s rule of five. The results of virtual screening against the MolProt database were refined based on RMSD and energy minimization. Based on the docking scores and ADME-profiling, these compounds showed effective hydrogen-bond interactions with the receptor in the conserved region. Riboflavin is a chemical analog of flavin mononucleotide (FMN) that shows antimicrobial activity against gram-positive bacteria. Riboflavin directly binds to the peptides of the FMN riboswitch and suppresses FMN riboswitch-lacZ receptor gene expression in *Bacillus subtilis*. Targeting the FMN riboswitch to disrupt the expression of receptor genes with riboflavin results in antimicrobial activity against resistant bacteria and may lead to the development of novel antimicrobial drugs [105, 106]. These inhibitors showed strong binding interactions and safe drug profiles. Furthermore, the constant binding free energy along the MD trajectories implies the possibility of antibacterial medication [107]. However, additional *in vivo* and *in vitro* validation experiments should be conducted to determine their pharmacological efficacy, biocompatibility, and role as effective inhibitors.

Antibiotic resistance in bacteria has reached to an extremely dangerous level, and strains resistant to most of the commonly used antibiotics are now reported at alarming rates in many countries around the world [108]. Core genome-mediated analysis in combination with comparative and subtractive genome and/or proteome analysis identifies pathogen specific novel therapeutic drug and vaccine targets against such resistant pathogens. Reverse vaccinology approaches are novel strategies for the designing and developing broad-spectrum potential vaccines with better safety and increased potency. Druggability and virtual screening analyses serve as a starting point for the development of novel inhibitors, targeting pathogen-specific proteins. The *in silico* approaches used in this study could pave the way for identifying novel therapeutic targets among different pathogens and the development of specie-specific potent vaccines and drugs that aid in the elimination of diseases caused by multidrug-resistant pathogens. Experimental and clinical assays are required for further validation of the results acquired in this study.

## 5.0. Conclusions

In this study, the core genome of *C*. *difficile* was utilized to identify potential drug and vaccine targets against *C*. *difficile* infections. Subtractive proteomic analysis was performed to select pathogen-essential proteins that were non-homologous to the human and human gut proteomes. A multi-epitope vaccine was designed utilizing lead overlapping B- and T-cell epitopes from the prioritized four vaccine targets via reverse vaccinology. Highly immunogenic adjuvant and linker peptides were used to conjugate the prioritized epitopes in a vaccine construct that can generate strong host immunogenic response. The *in silico* restriction cloning analysis predicted effective expression potential of the proposed vaccine construct in an *E*. *coli* system. Furthermore, molecular docking and immune simulation analyses revealed that the vaccine has the capability to generate strong cell-mediated and humoral immune responses against *C*. *difficile* through significant binding affinities towards human immune receptors. Comparative genome analysis and multiple *in silico* druggability methods have prioritized several potential drug targets in *C*. *difficile* which have not been previously reported. FMN-dependent nitroreductases was prioritized for pharmacophore-based virtual screening to identify small drug-like compounds. The pharmacokinetic properties based on ADME profiling identified the top hit compounds. Docking studies and molecular dynamic simulation analysis identified potential binding affinities and stable interactions between drug-like compounds and putative nitroreductase which may lead to new strategies in drug development against multidrug-resistant *C*. *difficile* strains. Experimental and clinical assays pursual are required to validate the finding of this study.

## Supporting information

S1 File *Clostridium difficile* non-paralogous proteins. (DOCX)

S2 File *Clostridium difficile* essential proteins non-homologous to the human host. (DOCX)

S3 File *Clostridium difficile* proteins homologous to DEG entries. (DOCX)

S1 Fig Population coverage of MHC-I and MHC-II alleles of the selected epitopes. (TIF)

S2 Fig Secondary structure prediction of vaccine construct. (TIF)

S3 Fig Molecular interactions between receptor proteins (Chain A) and vaccine constructs (Chain B). (TIF)

S4 Fig Codon optimization index graph of vaccine construct determined by JCAT server. (TIF)

S5 Fig *In silico* restriction cloning of the vaccine gene sequence (highlighted in red) into the *E*. *coli* pET28(+) expression vector. (TIF)

S6 Fig The individual RMSF graphs of protein (A) and protein-C7 complex (B).
(TIF)

S1 Table Overlapping B-cell, MHC-I, and MHC-II epitopes prediction using IEDB analysis resource and ABCPred server. (DOCX)

S2 Table Immunogenicity analysis of non-human homologous overlapping epitopes. (DOCX)

S3 Table Prioritized MHC-I and MHC-II epitopes with the respective alleles. (DOCX)

S4 Table Molecular interactions between receptor molecules (Chain A) and vaccine construct (Chain B). (DOCX)

S5 Table Codon optimization of vaccine construct. (DOCX)

S6 Table Druggability analysis of shortlisted non-human homologous pathogenic essential proteins. (DOCX)

S7 Table Top 10 hit molecules obtained from virtual screening using Pharmit server. (DOCX)

S8 Table CB-Dock energy, physicochemical, pharmacokinetics, and medicinal chemistry properties prediction of the molecules obtained from virtual screening using CB-Dock and SwissADME servers. (DOCX)
