# Supplementary material for: Core-genome-mediated promising alternative drug and multi-epitope vaccine targets prioritization against infectious Clostridium difficile
Source: PLoS One. 2024 Jan 19;19(1):e0293731. doi: 10.1371/journal.pone.0293731 (PMC10798517; doi:10.1371/journal.pone.0293731)
Supplement: S1 File — (DOCX) [file pone.0293731.s001.docx]

>CD630_00010 Clostridioides_difficile_630_NC_009089 chromosomal replication initiation protein DnaA

MDIVSLWDKTLQLIKGDLTSVSFNTFFKNIVPLKIHLNDLILLAPSDFNKDILENRYLHL

IEDAISQLSLKKYNIKFVLSEKEVADLNSDSTDLNYRVLYPNLNPKYTFDTFVIGNSNRF

AHAACVAVAESPAKAYNPLFLYGGVGLGKTHLMHAIGHHIVSQKKDSKVVYVSSEKFTNE

LINSIKDDKNEEFRNKYRNVDVLLIDDIQFIAGKERTQEEFFHTFNTLHEANKQIIISSD

RPPKDIPTLEDRLRSRFEMGLITDIQAPDFETRIAILRKKAQLERIDVPNEVMSYIAKNI

KSNIRELEGALTRVVAYSSLSNRVISFDLATEALKDIITTSKNEEINVLRIKEKVSSVFN

LKMEDFNSKKRTRSIAYPRQIAMYLTRELTDLSLPKIGEEFGGRDHTTVIHAHDKVSKDI

EESEEIKTKIDKIISDLKG*

>CD630_00020 Clostridioides_difficile_630_NC_009089 DNA polymerase III subunit beta

LKIICNQKILANRIGIAQKAINGKTTIELLKGILISTEEGQLKLTGYDAEIGIETYVQAE

IIEKGDVVVDARLFGDIIRKLPDSFVEIETDSENNIYINCVNSRFKIKGYAAKEFPKLPE

LNEEDLYSIPQEILKNMIKQTVFAISQDQTKPVLMGELLEIVDRNLNLVAIDGYRLAVKS

CSVDSLTENIKVIIPGKTLIDVNSLLSGEDNVKVGFNEKNAIFIINDTKIITRLLEGDFI

DYKKLLPREHNSRVKLNTKELLNSIERASLLSQSEKNNLIKLSIRDKVMAITSNTEKGNV

YEEVEIDLDGDYLDIAFNSRYFIEGLKNIDNEEIFIEFTTNVNPCIIKPTDDVNYIYLLL

PVRISSNI*

>CD630_00030 Clostridioides_difficile_630_NC_009089 RNA-binding mediating protein

MTEITIESEYIKLDQFLKLAEIASTGGHAKFLIQEGLVTVNDEIELRRGKKIKSGDIVEI

EGTKIKVL*

>CD630_00060 Clostridioides_difficile_630_NC_009089 DNA gyrase subunit A

MEENNKILPIEIAEEMKKSYIDYSMSVIAGRALPDVRDGLKPVHRRILYSMSELNLTPDK

PYRKSARIVGDVLGKYHPHGDTAVYYAMVRMAQDFSTRALLVDGHGNFGSVDGDSPAAMR

YTEAKMSKLSLELLRDIEKETVDFKPNFDESLKEPSVLPARYPNLLVNGSNGIAVGMATS

IPPHNLAEVIDATVYLIDNPECSVDDLIKFVQGPDFPTAAIIMGKESIAEAYRTGRGKVK

VRSRAFIEELPKGKQQIIVTEIPYQVNKAKLVERIAELVKEKRIEGISDLRDESNRNGMR

IVIELKRDANANIVLNNLYKHSQMEDTFSIIMLALVDGQPRVLNLKQILYHYIKHQEDVV

TRRTKFELNKAEARAHILEGLKIALDNIDAVISLIRASKTGQEAKLGLIEKFKLTEIQAQ

AILDMRLQRLTGLERDKIEAEYEDLIKKINRLKEILADERLLLNVIKDEITIIKENYSDE

RRTEIRHAEGEIDMRDLISDEEIAITLTHFGYIKRLPSDTYKSQKRGGRGISALTTREED

FVRHLVTTTTHSRLLFFTNKGRVFKLNAYEIPEGKRQAKGTAIVNLLQLSADEKIATLIP

IDGNDENEYLLLATKKGIVKKTKREEFKNINKSGLIAIGLRDDDELIGVELTDGKQEVLL

VTKEGMSIRFDENDIRYMGRTAMGVKGITLSKEDFVVSMNLCSKGTDVLVVSKNGFGKRT

NIEEYRSQIRAGKGIKTYNISEKTGTIVGADMVNEDDEIMIINSDGVLIRIRVNEISLFG

RVTSGVKLMKTNDEVNVVSIAKINIEEE*

>CD630_00070 Clostridioides_difficile_630_NC_009089 spore protein

MCNKRSCKKGGFLLLGAILGFIFGMFFAPKKGSELRKETKEKFNDVKENPKEVLHETFND

VKERIINLVDDDNNEEDIKISEEDIVISKSFDDEGDVN*

>CD630_00080 Clostridioides_difficile_630_NC_009089 hypothetical protein

MNAWGWQIGAVLVGSSALIVAIYLAKTLNSINKVVEKAYKIVDYNERHIQDIVENASSIS

KGIDDIVYVINKILSIGNIFKIIKRK*

>CDIF1296T_00009 Clostridioides_difficile_ATCC_9689__DSM_1296_strain_DSM1296_CP011968 anti-sigma-B factor antagonist

MSMNIDSNLDSQNKFWNVCLDGELDVSTADKLKEHLHALIEKNMLDVKINLKDLDYIDST

GLGAMIGVLKKLKINEKEIYIVNPKSNVRKIFTITGLDKIFKVEG*

>CD630_00120 Clostridioides_difficile_630_NC_009089 small-molecule-binding protein

LERRCNMNSNERRSKLIDILKESKHPVKGGTLAELLNVSRQVIVQDIALIRARGFEIIAT

PQGYIIYNQPYFTKQIKCKNHKDSKEIYDELKIIVDLGGIIKDVIVNHPTYGQITAELNI

YSKMDIDNFMKKVETNEFKQLSVLTECSHIHTIEAIKEETIEAIVKELKDNGILS*

>CD630_00130 Clostridioides_difficile_630_NC_009089 mechanosensitive ion channel protein

MNFLSLAKTASTNSNITKRTLENMLDNFSDNIPNIIYAIIVFVIGIYISKIVRRMVSKFL

IKYGMSKGVNNFIVYGIYISMLSIISLISLGIIGIQTTSVVAVLGAAGFSIGLAFKEILS

NLGSGMIILFFKPFNIGDYIQGSGVEGTVSDIQIFSTVLKTPDNKTIIIPNFQLTSNNII

NYTHQNKRRIDFSYNISYDSDIDVVKSTLNEIFTNEKRILNDPKPIIGLNSIGNNTMQIV

ARPWVKTDDYWDVYFDVMEKVKNKFDENDIKVPFVPSSLLFNNTGNLNTMDKR*

>CD630_00140 Clostridioides_difficile_630_NC_009089 serine--tRNA ligase

MLDIKRIRENLDDIKKAMERRGEREFDLDAVVELDNKRREILQEVEVMKNELNVNSKKIP

QLIKEGKDVTEEKARLKELSDKIKGIDEKVKEVEAKMEYTLMRIPNVPHPEVPQGETDED

NVQIRTWGEPTKFDFEHKAHWDIGTGLGILDFERAGKITGSRFTLYRGLGARLERSLMNF

FLNTHTAKHGYTEVLPPFMANRNSFIGTGQLPKFEEDMFKIEGLEYFLIPTAEVPVTNIH

ANEILDVAELPIKYCAYTPCFRSEAGSAGRDTRGLVRQHQFNKVELVKFVKPEDSYNELE

SLTHDAETMLQMLGLPYRVVKICTGDLGFTAAFKYDLEVWMPSYNRYVEISSCSNFEDFQ

ARRAGIRFKRDKKSKAEYVHTLNGSGLAIGRCLAAILENYQQADGSVVVPEELRPYMGVD

VIK*

>CD630_00150 Clostridioides_difficile_630_NC_009089 transfer RNA specific adenosine deaminase

MESSFYMKEALKEAYKAYNKKETPIGAIIVKDNQIIARAHNLTETLKDSTAHAEILAIKQ

ASEKLGGWRLTDCDLYVTMEPCIMCSGAIVNSRIKKLIIGTRHVKNSYIEKQHEFKLDYF

NNNNVKVAFDVLQEECSIILQEFFKALRKRD*

>CD630_00160 Clostridioides_difficile_630_NC_009089 DNA polymerase III subunits gamma and tau

MHKALYRAYRPQKFEDVIGQDHIIKTLKNQIYSDNIGHAYLFCGTRGTGKTSTAKIFSRA

VNCLNKINEEPCNECEICESVLKDNTMDVVEIDAASNNSVDDIRELRESVKYSPANAKYK

VYIIDEVHMLSQGAFNALLKTLEEPPSYVIFILATTEPHKIPATILSRCQRYDFKRVTVK

DMTLRMKKICEDEGIDIDDKALNLIARNSQGALRDALSILDQCMSFGESKIDYKDVVELM

GSVNIEQLFELSQCIVEQDTKKSLEILNEFVLWGKDIRNLINDLIDHFRNLMVCKVSSEL

DEIISLPEETIEQLKIQSKNIDINDLIRILNILSITQDDIKSSSNPRVLVEITIMKIAQP

MFDESKEALIKRVENLEKMIELGNFKSEKIGNNKEKEYEVDREIDVKQENVVYEDVKNED

VILIESSWKNILKQIKKDKKMPIYALLSEVKSFNVYSNMLYVIFDDKFDFAKTRLSSQDT

INYLEKTIRDVLNRSFNVKIVLTSEVKDINLEVKEKKDIGEEILKNIVSEEILEIKDSID

ENESK*

>CD630_00170 Clostridioides_difficile_630_NC_009089 DNA binding protein

MAKKGFGGGMMPGGGNMNNLLKQAQKMQENMQKAQQELESKEVEASVGGGAVTVKVNGKK

EVIDITIKPEVVDPDDIEMLQDLVLSAVNQALRNIDDIQASQMSKVTGGMNIPGLF*

>CD630_00180 Clostridioides_difficile_630_NC_009089 recombination protein RecR

MQVYTGPITRLIEEFSKLPGVGRKTAQRLAFHIINMNTNDVEALSKAIIDAKREIRYCSI

CCNITDTDPCSMCSNKSRDSSVICVVEDPRDVAAMERTREFKGQYHVLNGVISPMDGIGP

DMLKIKELIQRLGNQEVKEIIMATNPTIEGEATAMYIARLVKPMGIKVTRIAHGLPVGGD

LEYADEVTISKALEGRREI*

>PCZ31_RS00115 Peptoclostridium_difficile_strain_Z31_NZ_CP013196 hypothetical protein

MKPIMNLEEHNAMKEKRVKKMKSKDKPKYDDFEDISFSNKEKTKKKNVRNKNAEFRNNYK

NYQYDYEEDFYEPI*

>CD630_00200 Clostridioides_difficile_630_NC_009089 hypothetical protein

LYIVIGNEIVDSEELKIIIDKNSEFKVEKDLSKSTKREDVIAYQLSIDLNYLDSLINEQC

NLASLSDEEKFDEYMTLSDELALDLEELMPKYTIINARAYKLDEVYGIVKIILAVAYADL

GHLKLSDVVKRLSRQVD*

>CD630_00210 Clostridioides_difficile_630_NC_009089 pyruvate carboxylase

MLKKFNKILVANRGEIAIRIFRACSELGIKSVGIYSKEDKYGLFRTKADESYLIGEGKGP

IDAYLDMDGIIDLAKRKKVDAIHPGYGFLAENAEFARKCEENGITFIGPSSKVMNMMGDK

INSKKIAKEVNVQTIPGVEKAIRSTEEAKEVANKIGYPVMIKASNGGGGRGMRIVHREED

LELEYETACSESRKAFGEDIIFIEKYIADPKHIEVQILGDNYGNIVHLYERDCSVQRRHQ

KIIEYAPAFSLDDKVRKEICEDAVKLSKHVGYSNAGTLEFLVDANGGHYFIEMNTRVQVE

HTVTEMVTGIDIVQSQILIAQGYSLDSEEINIKSQDDVEIRGYSIQCRITTEDPKNKFMP

DTGKIQVYRTGSGFGIRLDGGNGFTGANISPHYDSLLVKTISWDRTFQGAINKTIRSIKE

LRVRGVKTNVGFLVNVLNNPIFSNGKCSTKFIDENPDLFEITESKDRGTKLLQFIGDVIV

NDNACKEKPLFDALHDPRMDKDGSKSEGSKILFDKLGKSAYIEKIKNDKKLLLTDTTMRD

AHQSLLATRIRTYDLLKAAKPTEKYQKDLFSLEMWGGATYDVAYRFLKESPWRRLQKLRE

EIPSIMFQMLLRASNGVGYKNYPDNVIEEFTKESARQGIDVFRIFDSLNWVENMKPSINT

ALETGKIVEATMCYTGDILDKTKTKYNLEYYIKMAQELESLGADIIAIKDMSGLLKPYSA

YTLVKELKKNVKAPIHLHTHDTSGNGVATCLMASEAGVDIIDAALESMAGLTSQPSLNAI

VEALKNTERDTGIDLFGYDELGKYYKDLRKVYNKFESDLTNSCAEIYNFEIPGGQYTNLK

PQADSLGLVNRFDEVKEKYKEANEVVGDIIKVTPSSKVVGDLAIFMTKNKLDKDNIIEEG

KNLSFPDSVVDYCKGMIGQPEGGIPKDLQEVVLKGEEAITVRPGSLLPAEDFDEIAKYLN

EKYDINANIRNVISYALYPKVYEDYIKHLQHYNDISKLESDVFFYGLNKNEECEVEIEEG

KVLTIRLVEIGEVKENGFRTIGFELNGMVREVEIKDKNFSGKINNVEKADMNDPLQIGAS

IPGKVIKIMVKEEDEVKANQPLIVIEAMKMETIIVAKTDGVIKSIKVKEDDMVEDKQLLM

IMK*

>CD630_00220 Clostridioides_difficile_630_NC_009089 elongation factor G

MKVYDSKMLRNVAVLGHSGCGKTNLIETIAYTANTNKIPKLTDKVNMTYSMGLIPIEYND

YKFNLLDTPGYFDFSGDVVSSLRASDAAIIVIDATAPIQVGTEKSLELTESIPKIMFINK

IDNEKARYKDAIAMLREKYNNKIVPMISPIYKDKNFVKLHNVFENIDDLEGEFKEQAMSV

KEALMELIAETDDQILDKYFNGEELTTEEIQKGIIIGIQRGDIIPVICGSTINNIGTKEI

LDTISSYLEPIFTEESKPFRGLVFKTMVDPFVGKMSYIKITEGVLSKDKDVFNINKNVKE

KIANIYTLRNSELVEIEKAKAGDIVVITKVNSLKTGDTISADKDAEALEKIDFPKPQIYY

AVTPKNKGDEEKVASVLNKLVEEDPTLHWYRNTETKQALLGGQGELHIKTIKNKMKDKFG

VDVELNDLKVPYRETIKGTADVQGKHKKQSGGHGQYGDVKIRFERCESDFEFTEEIFGGS

VPKQYIPAVEKGLKDSMQKGILAGYPVTNIKATLYDGSYHDVDSSEMAFKMAASAAFKKG

MEEAHPILLEPIMKLKITVPEEYMGDVMGDINKRRGKIFGMEPDDKGKQIIFAEAPQAET

FKYAIDLRAMTQGRGYFEMELERYGEVPSQFAEKIIGLATAK*

>CDIF1296T_00085 Clostridioides_difficile_ATCC_9689__DSM_1296_strain_DSM1296_CP011968 transcriptional regulator

VILMATMTDIIEKFIKDLMEEDNSIQIQRNELANLFSCAPSQINYVLTTRFTIDRGYYIE

SKKGGGGYVQIEKIRKSKDGHIRELLNEKIGSQISYKKAKELLEGLKESDLINERELKLI

LYAIDDKSLCMPIYELKEKVRSNILKNIIIGLFSIEE*

>CD630_00240 Clostridioides_difficile_630_NC_009089 activator of protein kinase McsB

MLCQKCNKNKASVYYNKIVNGEKTEMYLCSECAKENTEMNFNLDMPFSMMDIFSNLGFQP

KKELEEKLVCPKCNTTYSEFKNNGRFGCSECYNAFSSQVNPMLQNIHGHIEHTGKAPKKS

FYKISVENEIKELKEDLDRAIKNEEYELAAQFRDKIKYLKGSID*

>CD630_00290 Clostridioides_difficile_630_NC_009089 membrane protein

LIRKVTRILFILLGFTIGITTYLTLMKDFEILTFGKETYGYIAAVVAGIIIAILGYLIEP

WVVNKVKEIAKIVDKELSKYPQTDILLGSMGLIVGFVIAYLLSGLVNRIPIVGGILSLLL

YLFLGYLGMKVALKSKNDLFNVGKLGRLANPIKDKDKDKENKKEVKAIPPKVLDTSVIID

GRIADICKTGFIEGKLIIPAFVLEELRHIADSSDDLKRVRGRRGLDILNIIQKELNIEVE

ISERDFDDIAEVDSKLLKLAQVLNGKVVTNDYNLNKVAQFQGVEVLNINELANAIKPVAI

PGEDMVVQVVKEGKEAQQGVAYLDDGTMIVVDGGRKYMNETIKVLVTSVLQTPAGRMIFA

KPKN*

>CDM120_RS00440 Clostridioides_difficile_M120_NC_017174 AraC family transcriptional regulator

MNRNSFSHEIIEPIDNLDVNFKLFDDSGSYVANHWHNSLEIIYITSGDLQINMEGYTYNL

KANECMFINSGIIHSTRCTYHNTSILLQVPMSFLNKYIPDFKNCYFDFKVNANDNNYKRN

SSKVKLILENMREIKLSSPPAANLQFTSLLFELLFELYTNFKISVGNKNMKKTVLDLSKF

EPVLEYTNINYKSPISINKIAKVAHLQPEYFCRKFKQYMGQTYLEYLNDVRISHIYKDLI

NTNDTLCSILETHGFTNTKLFYRIFKEKFKCTPKHIRKNLPKI*

>CD630_00320 Clostridioides_difficile_630_NC_009089 beta-glucosidase

VYIKGVNLGGWLVLEKWMTSSLFEGTEAEDEYYLPRQLSREAYESRIKTHRSEYITERDF

ATIKSMGFNSVRIPVPYFIFGDCEPFIGCVKELDKAFAWADKYGLSILIDLHTVPGSQNG

FDNGGISGICSWSQNPEYVAFTLNVLERLAKRYGMRHELYGIQILNEPITERMWNIMNVP

NRFKAVDKEMARGSKPNSLEFLRDFYIKAYRVMRPYMREENVIVFHDAFELKAWKDFMRE

EEFKNVVLDTHQYLMLAEADGCEQSIDSYLKYIRENYAKDILQMQKYFPVICGEWSLFNS

YACGIDTNGGQSPLNGIESNIDKLSKDDKRELYRKIAKAQLDAWRNGSGHYYWNYKLLLD

TVNEEGWIGWDSWDLGKCVAQEWYPIEY*

>CD630_00330 Clostridioides_difficile_630_NC_009089 glycoside hydrolase

MIKNPILPGFNPDPCICRKGDDYYLVVSSFEWFPGIPVYHSKDLKNWELYTHILTDETKI

DLKKLPSSKGIWAPCLTYCEEEDLFYIVYGIMNSMNARYFDVDNYLITSKDIKGEWSEPV

YLHSSGFDASIFHDDDGKKWIASLDWETREGYEKPGVICLVEYCTKKKEIVGYPKRIWSG

GTDRGCIEAPHITKRGDYYYIMCAEGGTGYGHSVTMGRAKNIWGPYEKDSMNPIVTSIPG

DFYERHDPDHLKPKYYNPESKLQKSGHGSYIETTSGEVYLVHLTSRPFVPELRCTLGRET

AIQKMKWTKDNWLRMEDESNLAKEYVSESKLEEHLVSSIPSFDDFDSNELGLQYYAPRIS

PLSFADVKSRPGYVRIRGQESRTSLNKVSILARKLTSVYARITTKMEFYPEVHQHSAGLI

MYYDNMNYINLRKYYSETLGQSALSIIHLENGEKTEFLNTRIPIKDIPIYLRLYIQGRKS

YFEWSYDEKNYQRIGKVFDTTKFSDEYCKYGEFTGTFIGLTCADRVKHKHYADFDFFEYI

VDESKDVD*

>CD630_00350 Clostridioides_difficile_630_NC_009089 transcriptional regulator

MKKIGDLRLMMKCCSLYYEDNLNQQEIANQLGISRPTISRILKEAFEQGIVKIQIVDVLK

NDYQKIERSLERKYKLKEVIVVDDKQDALTQKQELARAVSEYLTRVVKENDIIGVSIGTT

LKEIPRYVEKSNCKNVTFIPLLGGIGDNEIDIHANQIAVSLARAFGGDFKLLHAPAVMSD

LSTKEKLCKDEKIKEVLDLIDKTTIAIVGIGNPMSLNSTIMASGYMNEADIEDLKKYNSI

GAICLQAFDKEGKTSILEFNQRVLGVKLEDLKKIKRTIGVASGDEKIEAIKASLKAKFIN

SLAINHSLALKLLEDCD*

>CD630_00360 Clostridioides_difficile_630_NC_009089 acetoin dehydrogenase E1 component subunit alpha

MYMSISKETLLEMYKRMNQARKFEEKVSWFFARGMVHGTTHLSVGQEASSVAAVMALEKG

DLVSLTHRGHSQFIGMGIDLNKMMAELMGKETGFCKGKGGSMHIADIESGNLGANGVVGG

GLTIAPGAALTQQYKKTGKIVLCSFGDGASNEGTFHEGINLSSIWKLPIIFYCENNLYGM

STSIKRHMNIESIATRAASYGIEGISIDGYNPIEVYETVQKAAEKCRRGEGPVLIESRTY

RWLGHSKSDANVYRTKEEIESWKAKDPIEFLKNYLIENNLSNEDELDKIQEFAKQSIEDA

VEFAQNSPNPKIESLLEDVYAD*

>CD630_00370 Clostridioides_difficile_630_NC_009089 acetoin dehydrogenase E1 component subunit beta

MSTRELTYAQAIKEAMSEEMRRDENVIFMGEDIGIYGGAFGVSVGMIDEFGPERVRDTPI

SEAAIAGAAAGAAATGLRPIMEVMFMDFVTISMDAIVNQAAKMRYMFGGKAQVPMVVRCP

GGSGTGSAEQHSQSLEAWFCHVPGVKVVAPSTPADAKGLLKAAIRDNNPVIFVENKLLYR

KKGFVPEDDYVIEIGKADIKREGTDVTVITYGRMLQSVEEAAETLSKENINVEIIDLRTL

YPLDKETIVKSVCKTGRVLICHEAAKTGGLGGEISALITESESFDYLDAPVKRICGKDVP

IPYNPELEKAVVPRVDEIEEAIKSLIVR*

>CD630_00380 Clostridioides_difficile_630_NC_009089 acetoin dehydrogenase E2 component dihydrolipoamide acetyltransferase

MVANKIKATPAARSQARKDNIKLDRLIGSGENGRIHLVDVLNYLKDNKANTTPLARRIAE

DLNIDLETIVGTGYNGKIRKCDVEKLTAKETIVSTNTSKSSEKKELKIENENSSMFNTVE

GIFEKPNPMRATVAKRMSESYFSAPVFTFNIEVDATELKVLRAKLIDTVKESTGVKLTMT

DLIVMAVSKILPNHQALNSAWTDEGIFRYKDVNIAIAVGLDEGLYVPVVKNANKKSLKEI

AKESKELAEKVKTGKLMPADQEGNTFTISNVGMYGITTFTPIINMPSSAILGVGATQDKF

VPVNGEAKIKPIMNLSLTSDHRVIDGTVAAKFLKDLKELLENPLSMLV*

>CD630_00410 Clostridioides_difficile_630_NC_009089 PTS system galactitol-specific transporter subunit IIA

MESMKIISSNLIFKNIEVSNNEDALKFLGQRLFDEQYVKESYIQAVVAREKKYATGLPTE

IYGVAIPHTDIVHVNEPGIAIGILNKPVKFIMMGTDDTEIDVKVVFMLAVKEPQEQLQLL

EKLMTIFQDKNILNNIVDLSEESVSDLLNSKLKN*

>CD630_00420 Clostridioides_difficile_630_NC_009089 PTS system galactitol-specific transporter subunit IIB

MGVTNVAKKILVACGTGVCTSTIAINKLKKALQDIGKLDMVNITQCKVVEVASKAPDYDL

IICTTQVSSSIKTPVINGLPFLTGVGMDKLINDVLEELEL*

>PCZ31_RS00360 Peptoclostridium_difficile_strain_Z31_NZ_CP013196 PTS galactitol transporter subunit IIC

MEGLYMEILNFIVGLGAQVMMPIIICIFGLILGTKLGKSLRAGLTVGVGFIGLNTIIALL

TDNLGPATQQMVKNLGLSLSIIDVGWPAASAIAFASTVGALIIPIGLVVNIVMLITNTTQ

TVDVDIWDYWHFAFTGALVAGATQSVMWGVFAAVANMVIVLVMADLTAPGIEEYLGMPGI

SLPHGFTQAFVPIAIVVNKLLDLIPGINKIEINADTLQKKFGLFGEPLIMGSVIGVIIGI

AAKYDIKGILQLGVTMGAVLILIPKMAALLMEGLLPVSEAAQEFIEKRFKNRGKIYIGLD

SAVGIGHPVTLSVALVLVPLTILIAAILPGNKVLPFADLAVIPFALVLIVPITKGNVFRT

LIIGIIIITSGLLIATNLAPLFTQMALNASFKMPEGATMISSICDGANPLSWVFVKVMNY

KVIGGVVFGVIALGMAIYNRNRIINENKKLSIEE*

>CD630_00450 Clostridioides_difficile_630_NC_009089 sugar-phosphate aldolase

MLLEKLRKEVLQASLDLLNYNLVTLTGGNVSGRDEQTGYIAITPSGMDYRNLTPSDIVIV

DVDGNIIDGKWKASVDLSDHLYIYKHREDINSIIHTHSTYSSCFAILNEPIECASTTLAN

EVGGSVPVAKFSPPTSKKMGKCVIEAIGDKRACLLANHGVIAVGPSVGHALTAAVMLEDS

AKVYYLAKSIGTPVLLPDEEIQRARDVFFNVYGQDK*

>CD630_00480 Clostridioides_difficile_630_NC_009089 2-C-methyl-D-erythritol 2,4-cyclo diphosphate synthase

MRIGLGYDVHKLTEDRKLIIGGVEIPHDKGLLGHSDADVLIHAIMDSILGALALGDIGKH

FPDTDEEYKGADSMKLLEHVYNLITSKGYKIGNIDSTIIAQSPKMAPYIESMRSNISKVL

NTDIDNINIKATTEEGLGFTGAKQGIASQSICLLLLTSQNN*

>CDM68_RS00370 Clostridioides_difficile_M68_NC_017175 proline--tRNA ligase

MKMSKMFMPTLKEIPADAEITSHQLMVRSGMIKKMTSGVYNQLPMGLRVFKKIEQIIREE

LNKKDCQEILCAALLPSELWKESGRWTAMGEEMFRLKDRTEREYCLGPTHEEAFTDIIRQ

EITSYKQLPLNLYQIQVKYRDERRPRFGVMRTKTFTMKDAYSFDADDKGLDKSYQDMFDA

YVSIFDRCGLENSPVQADSGAIGGSTSAEFMVKSEVGEDEVVFCSGCDYAANVERAESCN

LASQKEEMKELEEVHTPGAATIKELEEFLKTSPDKFAKTLVYEADGKTVVVVVRGDREVN

EIKVSNAIGSVIEFALATDDVVRKVTNAEVGFAGPIGINADYVFIDKEIVEQRNIVVGAN

KTEYHIKNANYGRDFEGIVGDFRNVQEGDKCIVCGKPLEIARGVEVGHIFKLGTKYSESM

NANFIDKDGKSKPIVMGCYGIGVERTAAAIIEQHNDEKGIIWPLSVAPYHVVIIPANMKN

EEQISIAENIYNDLQAMGVEVLLDDRDERIGVKFNDSELIGIPMRITVGKNINEGKVEFK

LRHKEDKEIIDIEEINEKVKAEFIRNNVRLGQ*

>CD630_00500 Clostridioides_difficile_630_NC_009089 proline--tRNA ligase

MAKNEKQFVEEITKMEDDFPQWYTDVITKTDLVDYAPVKGFMVVKPYGYALWEKMQEFMD

KKFKETGHKNCYFPLLIPESLLNKEAEHVEGFAPEVAWVTHGGNKKLEERLCVRPTSETI

ICTMYAKWLKSYRELPYLYNQWCSVVRWEKSTRPFLRTSEFLWQEGHTLHETAEEAQEET

IQQLEVYKALCEELLAMPVVAGQKSESEKFAGGERTYTIEAMMHDGKALQSGTSHFLGQH

FTKAFDITFADREGNLANPYHTSWGASTRLIGGLIMTHSDNRGLVLPPRVAPIQVVIVPI

AAKKGNVMETVDKIYADLKAKGVAVEVDDRDNYTTGWKFNEWEMKGVPVRVEIGPKDIEN

NQAMVFRRDTLEKDSMPLEGLADAICDLFDVIHNDMFEKARKHREDNTSIVENMDEFRKA

LEEKPGFIKTMWCGDAECEAKIKEETGATIRCLPFEQENLGHKCVYCGKEADSMVVMAKA

Y*

>CD630_00520 Clostridioides_difficile_630_NC_009089 cysteine--tRNA synthetase

VKVYNTLTRTKEEFVPLEEGKVKMYVCGPTVYNYIHIGNARPFIIFDTLRRYLEYRGYDV

TYVQNFTDVDDKIINRSHEEGISPEEVAAKYIKEYFVDCDGLGIKRATVHPQVTDNIQQI

IEFIKELEDKGYAYAVNGDVYFDTNKFEGYGKLSGQKQEDLEAGARIEVNDQKRHPMDFV

LWKAKKEGEPGWDSPWGEGRPGWHIECSVMSKRYLGETIDIHAGGQDLTFPHHENEIAQS

EARSGKTFSKYWMHNGYININDEKMSKSKGNFFTVRDISKLYDLEIVRFFMLSAHYRNPV

NFSDEMLNQAKAGLERLYNTKEKLEFTLSNLVESPLTEKEVELVKELDDFRQKFIDAMDD

DVNTADAVSVIFELAKLINSNVDENSSLEFAKKCLDEFNELTGVLNIVNKKKDTVLDKDI

EELIQKRTDAKKNKEFQLADDIRQQLLDMGIVLEDTRQGVKWKRI*

>PCZ31_RS00415 Peptoclostridium_difficile_strain_Z31_NZ_CP013196 Mini-ribonuclease 3

VEKDIKMEKTELVTMSPLVLAYLGDTVYETYIREYLIRQNTQRKVNDLHKLAIKYVKAKA

QATIIHEVEIELTEEESKIYKRGRNQKSNTSPKNADIIDYKHATGFEALVGYLYLNNEIE

RLQYIINKGIKIIERDM*

>CD630_00540 Clostridioides_difficile_630_NC_009089 FAD-dependent thymidylate synthase

MKVKLISHTPEPEKVIAMAAKLCYSPVGTDEIEKDLTDESIEKFLNMLLSIGHGSILEHA

SFTFSIEGISRACSHQIVRHRIASFSQQSQRYVKLEQFEYIIPPEIEKIEKAKELFIDSM

KKDQENYDKLVEILFENHYNDLIKNGKNEKTAKRQAEKKAIEDARYVFPNACETKMVFTI

NARSLFNFFEHRCCERAQWEIRNLAVEMLREVKKVAPILFKKTGPSCVNGSCPEGTMTCG

DIVQVREKFKAL*

>CD630_00550 Clostridioides_difficile_630_NC_009089 23S rRNA (guanosine(2251)-2-O)-methyltransferase RlmB

VNNLASIEGRNPVIEAIKSDREIDKILIANSAKEGSIKKIIGMAKDKNIIIQYVDKHKLD

EVSTSHSHQGVIAYASEYKYYELDELIDLAKNKDEDPFFIILDEITDPHNLGSIIRTADA

VGAHGVIIPKRRSVHITPVVAKASAGAVEYMPVCKVTNIVNTIKRLKEEGLWIAAADMDG

ETFYKQNLTGPLGVVIGSEGFGISRLVKQNCDFIVKMPMIGNVTSLNASVAGGILLYEIF

RQRLDKSK*

>CD630_00560 Clostridioides_difficile_630_NC_009089 ribonuclease

MKKNINHYLIIDGYNIINAWDNLKELAKEDLEDSREKLIDDIIEFSEFMGYKTIIVFDAY

NVKNSREKVEKRKHITIVYTREHQTADSYIEKFITSLSKYDDVKVATNDYAEQQMILGKG

ATRMSARELKLELDRSKNKMKEKNIGLRKKIQRNWLEERLDKETLSKLENIRRKR*

>CD630_00570 Clostridioides_difficile_630_NC_009089 RNA polymerase factor sigma-70

MGEIYMLVAKEKSYELVDNCQQDEYNIVLRASEGDKIALEYIITKYRNFVKAKAKSYFLI

GADKEDIIQEGMIGLYKAVRDFDGSKTNSFKCFAEICITRQIITAIKTATRQKHIPLNSY

VSLNKPIYDEESDRTLLDIIATSIVTDPEELIISKEELKNIESKMNELLSDLELEVLELY

LNGKSYQFIADKLKRDVKSIDNALQRVKRKLEKHLENRND*

>CD630_00581 Clostridioides_difficile_630_NC_009089 50S ribosomal protein L33

MRVKVTLACTECKQRNYNTTKNKKNNPDRIELQKYCRFCKKHTTHKETK*

>CD630_00600 Clostridioides_difficile_630_NC_009089 transcription termination/antitermination protein NusG

MSELQEASWYVVHTYSGHENKVKATIEKAVKTRGMEDCIRQVVVPTEEVVETTKTGKEKT

RQRKVYPSYVLVKMIITDESWYVVRNTKGVTGFVGPGSKPVPLSEDEVKAMGIDTTDPKV

VNSDIDFEIGDTVKVSQGPFSGQIGNIEEIDLENREVKVCINAFGKRTLFVIELEGIEKI

*

>CD630_00610 Clostridioides_difficile_630_NC_009089 50S ribosomal protein L11

MAKKVIGQIKLQIPAGKATPAPPVGPALGQHGVNIMGFTKEFNAKTADQAGMIIPVVITV

YQDRSFSFITKTPPAAVLIKKALNLKSGSGEPNKKKVAKMTSAQVREIAELKMPDLNAAS

VEAAMSMIAGTARSMGVVIED*

>CD630_00620 Clostridioides_difficile_630_NC_009089 50S ribosomal protein L1

MAKKGKRYAGALQKVDRTKFYDASEALTLVSDIAGAKFDETVEAHIKLGVDSRHADQQVR

GAVVLPHGTGKTKRVLVFAKGEKAKEAEQAGADFVGAEELVQKIQGENWFDFDIVVATPD

MMGVVGRLGRVLGPKGLMPNPKSGTVTFDVAKAIDEIKAGKVEYRLDKTNIIHVPVGKVS

FGGEKLTENFTALMDAIIKAKPAAAKGQYLRSITVASSMGPGVKINPARTAE*

>CD630_00630 Clostridioides_difficile_630_NC_009089 50S ribosomal protein L10

MRKAIEIKSEVVSEIVEKLQKSSAAVVVDYKGLTVEEVTELRKQMREAGVDYKVYKNTLV

RRAAKEVGIEQFNDELLVGTNAIAFGYDDPVAPARILKGFMDSHPKMKLKMGIVEGAFYD

ESKIVEMANIPSREVLIAKLLGSLKAPVSNFAYLIDAIAKKAEGQEEA*

>CD630_00640 Clostridioides_difficile_630_NC_009089 50S ribosomal protein L7/L12

MTIEQILEAIENMKVLELNELVKAAEEKFGVSASAPVMVAGAAAGGPAAEEKTEFDVVLT

DVGSSKVGVIKAVREITGLGLKEAKEVVDNAPKTVKEGASKEEADQIKEKLEAAGAKVEV

K*

>CD630_00650 Clostridioides_difficile_630_NC_009089 NADP-dependent dehydrogenase

MEKLQGKIAVVTAATKGIGLASAEILAKNGATVYLAARSEELAHEVINKISAEGGCAKFV

YFNAREEETFTSMIEEVVKKEGKIDILVNNFGSTNPSLDKDLVTGDTDNFFDTVNTNLKS

VYLPCKAAIPHMIKNGKGSIVNISSIGSVLPDLSRIAYCVSKAAINSLTQNIATQYAKDN

VRCNAVLPGLIATKAALDNMSPEFIKEFLKHVPLNRIGEPDDIAKAVLFYASDDSSFITG

DLLEVAGGFGLPTPQFADNILG*

>CD630_00680 Clostridioides_difficile_630_NC_009089 30S ribosomal protein S12

MPTINQLVRKSRKALEKKSTAPALQKGYNSLNKKVTDASAPQKRGVCTSVKTVTPRKPNS

ALRKVARVRLTNGIEVSAYIPGEGHNLQEHSVVLIRGGRVKDLPGVRYHILRGTLDTAGV

DKRRQSRSKYGAKRPKEAKK*

>CD630_00690 Clostridioides_difficile_630_NC_009089 30S ribosomal protein S7

MPRKGNIPKREVLPDPMYGSKVVTKLINNLMVDGKKGKSQRIVYDAFAIVAEKTGEEALE

VFNKAMDNIMPVLEVKARRVGGANYQVPIEVRPERRQTLGLRWLVKYTRARGEKGMVEKL

AKEIMDAANNTGASVKKKEDTHKMAEANKAFAHYRW*

>CD630_00720 Clostridioides_difficile_630_NC_009089 30S ribosomal protein S10

MAKNEKIRIRLKSYDHKLLDFSAGKIVETAKKAGSQVSGPVPLPTEKQVVTILRAVHKYK

YSREQFEIRTHKRLIDIANPTPKTVDSLMRLDLPAGVDIEIKL*

>CD630_00730 Clostridioides_difficile_630_NC_009089 50S ribosomal protein L3

MKGILGKKVGMTQIFTDKGVVIPVTAVEAGPMVVTQIKTVDKDGYNAIQIGFEDAKEKAL

NKPKKGHLAAANVLKKHLKEFRVDSVEGYTVGQEIKADVFEAGAKIDVTGISKGKGFQGP

IKRHGQSRGPETHGSRYHRRPGSMGACSYPGRVFKNKKLAGHMGSVKVTVQNLEVVKVDA

DKNLILVKGAIPGAKGSVVTIKEAIKVSK*

>CDIF1296T_00140 Clostridioides_difficile_ATCC_9689__DSM_1296_strain_DSM1296_CP011968 50S ribosomal protein L4

MTNLEKGGITMPKLNVLNVSGQNVGEIELSDSIFGVEVNGHVLYEVVKNQLANKRQGTQS

AKTRAEVRGGGRKPWKQKGTGRARQGSTRSVQWVGGGVAFAPKPRSYKYTLPKKVRRLAM

KSALSSKVQNSEVIVLDALNMDAPKTKEFAQILNNINAAKKALVVIADKNDNVIKSARNI

EGVQTALVNTMNVYDILKYDSFIITTDAVKKVEEVYA*

>CD630_00760 Clostridioides_difficile_630_NC_009089 50S ribosomal protein L2

MAIKKFRPTSPALRQMTVLVSDEITCNQPEKSLLVNLKKNAGRNVHGRITVRHRGGGQKR

KYRIIDFKRDKDGIPAKVATIEYDPNRTANIALLNYADGEKRYILAPVGINVGDTILSGL

GADIKPGNCLALKDMPVGTIIHNIELKPGKGAQLVRSAGVSAQLMAKEGKNALLRLPSGE

MRLVSINCKATIGQVGNIEHGNVVIGKAGRKRHMGIRPTVRGSVMNPNDHPHGGGEGRSP

IGRPSPVTPWGKPALGYKTRKKNKASNKLIVSRRTK*

>CD630_00770 Clostridioides_difficile_630_NC_009089 30S ribosomal protein S19

MSRSTKKGPFVHARLLKKIEAMNASGNKEVIKTWSRSSTVFPQMVENTIAVHDGRKHVPV

YITEDMVGHKLGEFVPTRTFKGHKDDEKSNKRK*

>PCZ31_RS00545 Peptoclostridium_difficile_strain_Z31_NZ_CP013196 50S ribosomal protein L22

MEAKATAKYVRVSPRKAGQICDLVRGKNVDEALAILKFTPRGAASIIAKVVKSAKANAEN

NHEMDTEKLYIASIVANQGPTMKRFMPRAMGRATTIRKRTSHIEVVVKEKNN*

>CD630_00790 Clostridioides_difficile_630_NC_009089 30S ribosomal protein S3

MGQKVNPHGLRVGVIKDWDSRWFATDKKEFGNLLLEDHNIRKFLKKRLYSAGVAKIEIER

SANKIKMDLHVAKPGVVIGRAGAGIEALKAELEKMTKKTIIVNIVEVRSTDKNAQLVAEN

IALAIERRVAFRRAMKQAIQRAMKSGAKGIKVSASGRLGGAEMARTEGYSEGNVPLQTLR

ADIDYGFAEADTTYGKIGIKVWICNGEVLPTRDGVNPREESRKSDRRDNKRDNRRNDRRG

NDRRGNDNRGNYRGQRPQGGSRPQRTENKGN*

>CD630_00800 Clostridioides_difficile_630_NC_009089 50S ribosomal protein L16

MLMPKRVKRRRVHRGSMAGQAHKGNKVTYGEFGLVALEASWITSNQIEAARIAMTRYIKR

GGKVWIKIFPHKPVTRKPAETRMGAGKGSPEYWVAVVKPGRVMFELAGVSEDKAREAMRL

AAHKLPIKCKFVKKEDLEVKGGE*

>CD630_00801 Clostridioides_difficile_630_NC_009089 50S ribosomal protein L29

MKAKELRDLTSEELMNKLNDFKSELFSLRFQLATGQLENTARIKFVKKDIAKVKTVLAER

KLYETRA*

>CD630_00810 Clostridioides_difficile_630_NC_009089 30S ribosomal protein S17

MERGRRKVRIGRVVSDKMDKTIVVAVEEFVRHPLYNKRVKRTKKFKAHDEKNICNIGDRV

KIMETRPLSKDKRFRLVEVVEKVK*

>CD630_00820 Clostridioides_difficile_630_NC_009089 50S ribosomal protein L14

MIQQESRLRVADNSGAKELLCIRVLGGSKRRYGNIGDVIVATVKSATPGGVVKKGKVVKA

VIVRSKQGVRRNDGSYISFDENAAVIIKDDKTPVGTRIFGPVARELRDNEFMKIVSLAPE

VL*

>CDIF1296T_00150 Clostridioides_difficile_ATCC_9689__DSM_1296_strain_DSM1296_CP011968 50S ribosomal protein L24

MMRVKKGDTVVVIAGKDKGKKGSVLKVYPKTSKVLVEGVNVITKHQKPSAMNQQGGIINK

EAPIHISNVMPFDPETGKGVRVRYEVKDGNKVRVSAKSGKEL*

>CD630_00840 Clostridioides_difficile_630_NC_009089 50S ribosomal protein L5

MASRLQEKYMKEVAPALMEKFGYKNVMEIPKLNKIVINMGIGDARENPKGLEKGVEELEM

ISGQKPVITKARKSVANFKLREGMPIGTKVTLRADKMFYFMDKLVSVSLPRVRDFRGVNP

NAFDGRGNYALGVKEQLIFPEIEYDKIDKVRGMDIIFVTTAKTDEEARELLKLLGMPFSK

*

>CD630_00850 Clostridioides_difficile_630_NC_009089 30S ribosomal protein S8

MTMTDPIADMLTRIRNANVVKHETVDVPASNMKKELARILLEEGFIRGYDVIEDGKQGII

RIQLKYGQEGERVITGLKKISKPGMRVYAANHEIPKVLNGLGISVISTSKGILTDKQARK

ENVGGEVICYVW*

>CD630_00860 Clostridioides_difficile_630_NC_009089 50S ribosomal protein L6

MSRIGVKPIIIPAGVEVTIAEGNLVTVKGPKGTLTKQLSAELNIKKEENTIMVERPTDNK

KHRSLHGLTRTLLDNMVVGVNTGFEKKLELKGVGYRAQKQGKKLVMNLGFSHPVEMEDPE

GITVEAPNQTELIVKGIDKQLVGNYAAKIRAWRKPEPYKGKGIKYVDEVIRRKEGKTGKK

*

>PCZ31_RS00600 Peptoclostridium_difficile_strain_Z31_NZ_CP013196 50S ribosomal protein L18

VLKKADKNANRLQRHKRVRRKISGTSQRPRLCVFRSANNIYAQIIDDTKRVTLVAASSLE

AEVKSAVNHTGNKEAAKKVGELVAKKAVEKGITEVVFDRGGYLYHGRIQELAEGAREAGL

KSNTKEGKIHATS*

>CD630_00880 Clostridioides_difficile_630_NC_009089 30S ribosomal protein S5

MLRRKPIDAGQLDLQEKVVEVRRVTKVVKGGRNFRFAALVVVGDENGHVGIGAGKAMEVP

DAIKKAVEDAKKNLIVVPIVGTTIPHEVRGHFGAGNILIMPAVEGTGVIAGGPARAVLEL

AGLKDVRAKSLGSNNPRNMVNATIEGLNSLKTVEDIAKLRGKKVEELLG*

>CD630_00881 Clostridioides_difficile_630_NC_009089 50S ribosomal protein L30

MAKLQIKLVRSVIGTTPNQKKNVEALGLRKREQVVVKEDNAQTRGMINKVSHLLEVTEIA

E*

>CD630_00890 Clostridioides_difficile_630_NC_009089 50S ribosomal protein L15

MKLHELKPAEGAVRAKRRLGRGTATGQGKTAGRGQKGQWSRSGGGVRVGFEGGQMPLARR

LPKRGFNNIFKKVYTEVNVEVLNRFENGTEITAELLKSTKTISKIGKDGIKILGEGNLEK

ALTVKAAKFTASAQEKIEKAGGKAELV*

>CD630_00900 Clostridioides_difficile_630_NC_009089 protein translocase subunit SecY

VLSKLKQAWKIKAVRKKVMYTLMMIVIFRIGTTIPVPGIDTSIIQKMVGGNSLLSLYNMF

TGGAFSNFSLFALGISPYITASIIIQLLTVGFESLAELQKSGEEGKKKINKYTKYTALAL

AVVQALGITLGIVRSALISNSVFFITTVVITLVSASMLVMWIGDKITEKGIGNGSSVIIF

AGIISRIPTDVIKISQQVKSGEVAPWVIVILAVVILLTVTGVTFIQEATRKIPVQYAKRV

VGRKMYGGQSSHIPMKVNQSGVMPIIFASSLLAFPQTIAMFMGPNAQAFVQKYLSMATEQ

GFWTYRSIEILLIIFFSYFYTTVSFNTEDISKNMKNNGGFIPGIRPGEPTMDYLNRILTR

LTLAGATFLAIIAMVPALTTHYMKVNMSLAGTSLLIVVGVALELKRQLESNLVMRSYQGF

LK*

>CD630_00910 Clostridioides_difficile_630_NC_009089 adenylate kinase

MRIILLGPPGAGKGTQAVGIVEKYNIPHISTGDIFRKNIKEGTELGKKAKEYMDQGLLVP

DELTVGLVTDRISQEDCKNGFMLDGFPRNVAQGEHLDIFLKNAGISLDKVVNIEVDKSIL

VSRAVGRRICKSCGATYHVEFNPPKVEGVCDVCQGELYQRADDNEETVSKRIQVYLDETK

PLVDYYSKQGIIADIKGDQAIDKVFEDIVAALGSGK*

>CD630_00930 Clostridioides_difficile_630_NC_009089 50S ribosomal protein L14E/L6E/L27E-like protein

VLSDNLSIGQVVKVSLGRDKGNLFFVVKIINNEYVLIADGKKRKLDKPKLKKVKHLKKYN

FINDEVRKRVVSGQEITDSFLRAELTKLN*

>CD630_00940 Clostridioides_difficile_630_NC_009089 translation initiation factor IF-1

MAKKDVIELEGTVSEALPNAMFKVKLENGHEILCHISGKLRMNFIRILEGDKVNVELSPY

DLTRGRITWRKK*

>CD630_00941 Clostridioides_difficile_630_NC_009089 50S ribosomal protein L36

MKVRPSVKPICEKCKVIKRKGKVMVICENPKHKQKQG*

>CD630_00950 Clostridioides_difficile_630_NC_009089 30S ribosomal protein S13

MARIAGVDLPREKRAEIGLTYIYGIGKATANEILAKAEINPDTRIKDLSEDQVNELRKVI

DDDFLVEGDLRREIALNIKRLRDIKCYRGIRHAKGLPLRGQRTKTNARTRKGPRKTVSRK

KKK*

>CD630_00960 Clostridioides_difficile_630_NC_009089 30S ribosomal protein S11

MAKPKKKVTRIRRRERKNIERGHAHIQSTFNNTIITLTDVHGNAISWASSGQLGFKGSRK

STPFASQMAAETAAKAAMEHGLKSVEVFVKGPGSGREAAIRALQATGLEVTMIKDVTPIP

HNGCRPPKRRRV*

>CD630_00970 Clostridioides_difficile_630_NC_009089 30S ribosomal protein S4

MARYTGASCRQCRREGMKLFLKGDRCYTDKCAIVKRNYAPGQHGQGRKKVSNYGLQLREK

QKVKRIYGVLETQFRNLYERAENMPGKAGENLLSLLERRLDNVVYRMGLASSRKEARQLV

THGHFTLNGNKVDIPSLIVKVGDVIEVKEKSRSSAKFKNLVEVNSRIAPKWLEANVEGMT

AKVVGVPTREDIDLEIAEHLIIELYSK*

>CD630_00980 Clostridioides_difficile_630_NC_009089 DNA-directed RNA polymerase subunit alpha

MIEIEKPKVDIVELSEDYRYGKFVIEPLERGYGITIGNALRRILLSSLPGVAVNAIKIDG

VLHEFSTIPGVKEDVTEIILTLKELSATIDGEGSRTLKIEAQGPCSITGADIICPPDVEI

LSKDLAIATLDDNAKLNMEIFVDKGRGYVSAEENKTENVPIGVLPVDSIYTPVEKVSYHV

ENTRVGQKTDYDKLVLEVWTNGSINPQEGISLAAKVLVEHLNLFIDLTEHVSSVEIMVEK

EEDQKEKVLEMTIEELDLSVRSYNCLKRAGINTVEELANKSEDDMMKVRNLGKKSLEEVI

QKLEELGLGLKPSEE*

>CD630_00990 Clostridioides_difficile_630_NC_009089 50S ribosomal protein L17

MAKYRKLGRETAHRNLMLRNLVTCLLRSGRIETTVTRAKETRRMAEKMITLAKRGDLHAR

RQVLAYVMDETVVNNLFTDLAPKYAERNGGYTRIIKIGPRKGDAAEMAFIELV*

>CD630_01000 Clostridioides_difficile_630_NC_009089 cobalt ABC transporter ATP-binding protein

MDNIVKVNNISFEYITDEAKLKAIDNLSLDVKKGEFVAIIGHNGSGKSTLSKNLNAILMP

TEGNILIDDMDTKEEERLWDIRQTAGMVFQNPDNQIVATIVEEDVAFGPENLGIEPKEIR

RIVEESLKSVGMYDLRDRQPHLLSGGQKQRVAIAGIIAMRPKCIIFDEATAMLDPSGRKE

VMKTIKRLNKEENITVIHITHFMEEAVEADRVVVMEKGKKILEGTPREVFSKIKMLKEIG

LDVPCMTELSSLLIEEGINISSDILTVDEMVMELCQL*

>CD630_01010 Clostridioides_difficile_630_NC_009089 cobalt ABC transporter ATP-binding protein

MSIIVKNLTHIYNEGMPFASKALDDVSFEIKDRDFVGLIGHTGSGKSTLIQHLNGLLKPS

SGEIFINDFNITDKNLNLTEIRKRVGVVFQYPEYQLFEETIDKDIAFGPSNLGLEESEIH

NRVKASMEAVGLDYEGFKDKSPFELSGGQKRRVAIAGVIAMNPEVLILDEPTAGLDPGGR

DEIFNLIKDLHEKKNMTIILSSHSMDDMAKLAKTLIVMNHGSVEFMGTPREVFKSNASKL

KDIGLDIPQVLELALKLREKGFDISEDILTLEEAKQEILKVVRGRGLC*

>CD630_01020 Clostridioides_difficile_630_NC_009089 cobalt ABC transporter permease

MLKDITIGQYYPTSSAIHKLDPRIKLVATIVFMVSIFVVNKFWPYIVVLLCLLAMIKLAN

IPVKYIVKGVKPLKWIILFTFLINIFFLPGDEIWSFGFLAITKQGLRQAIFMAIRLIFLV

VGTSLLTLTTSPIELTDGIERLLNPFKKIGLPVHELAMMMTIALRFIPTLLDETDKIMKA

QMSRGADFESKNLINRAKNLVPLLVPLFVSAFRRADELAMAMEARCYRGGHNRTKMRESV

ISRRDYMACVFQLVYLGAIIATRFIAI*

>CD630_01030 Clostridioides_difficile_630_NC_009089 tRNA pseudouridine synthase A

MRNIKIKIQYNGKNYCGWQKQPDSLGIQGTIERAIYDITKEETSLIGSGRTDSGVHAIGQ

IANFKINSGISIESIPMALNAKLPKDISVIEACEVNDDFHSRYSAKGKTYKYLVYNSKFR

NPILSEISYQVKYELDFDKMCSEAKSLLGTHDFKGFMSSGSSVKDTVRTIYDIDISKKDD

LITFEISGNGFLYNMVRIIVGTLVDMGRGRINEPFLDIIQSKTRSRCGHTAPAQGLFLKK

VHY*

>CD630_01050 Clostridioides_difficile_630_NC_009089 30S ribosomal protein S9

MANVQYYGTGRRKSSVARVRLVAGEGNILVNGRALENYFNYETLIRDVKQPLVLTGNENK

YDVIVKVEGGGFTGQAGAIRHGISRALLKADLDLRPALKKEGFLTRDARMKERKKYGLKA

ARRAPQFSKR*

>CDIF1296T_00176 Clostridioides_difficile_ATCC_9689__DSM_1296_strain_DSM1296_CP011968 germination-specific N-acetylmuramoyl-L-alanine amidase

MEYYGVFISIHIQGDVVRKYIKHIIFSFAMICLVVVSIFEIKNISEDVIKYMPVTNKTII

LDAGHGGIDPGALNKDKSTSEKDINLAITLKLRELIESSGGLVILTREDDSSLYKEENNK

TTRQKYNENLKNRKEIISNSNANMFVSIHLNAFEQSKYYGAQTFYPKDKQDSKALSKCIQ

EELKRVVDKTNNREVKPRDDIYLLKENNIPSVLIECGFLSNEKECKLLTDETYQEKIAWA

IYIGIQKYLS*

>CD630_01100 Clostridioides_difficile_630_NC_009089 hypothetical protein

VLDINSFLNGFFNIILRMSIYDLIDISIVAYIFYKIFMFIKDTRAEQVFKGIIFLLLATQ

LSNTFKLHTVYWISLKALDYGVIAALIIFQPEFRAGLEHIGRAKFNLFGKNVNTSEETLN

RNIEEIVEALYSLSRQKIGALIIMERETRISDIINTGTIIDAEISRQLLINIFIPNTPLH

DGAVVIRDSKVKAAACFLPLTESKDLSKDLGTRHRAGIGVSEVSDCITLIVSEETGGVSI

AKAGKLYRDISRERMMNILRSNLKTNTETRSFFKGGIFK*

>CD630_01120 Clostridioides_difficile_630_NC_009089 phosphate butyryltransferase

MRSFEEVIKFAKERGPKTISVACCQDKEVLMAVEMARKEKIANAILVGDIEKTKEIAKSI

DMDIENYELIDIKDLAEASLKSVELVSQGKADMVMKGLVDTSIILKAVLNKEVGLRTGNV

LSHVAVFDVEGYDRLFFVTDAAMNLAPDTNTKKQIIENACTVAHSLDISEPKVAAICAKE

KVNPKMKDTVEAKELEEMYERGEIKGCMVGGPFAIDNAVSLEAAKHKGINHPVAGRADIL

LAPDIEGGNILYKALVFFSKSKNAGVIVGAKAPIILTSRADSEETKLNSIALGVLMAAKA

*

>CD630_01130 Clostridioides_difficile_630_NC_009089 butyrate kinase

MSKIFKILTINPGSTSTKIAVFDNEDLVFEKTLRHSSEEIGKYEKVSDQFEFRKQVIEEA

LKEGGVKTSELDAVVGRGGLLKPIKGGTYSVSAAMIEDLKVGVLGEHASNLGGIIAKQIG

EEVNVPSYIVDPVVVDELEDVARISGMPEISRASVVHALNQKAIARRYAREINKKYEDIN

LIVAHMGGGVSVGAHKNGKIVDVANALDGEGPFSPERSGGLPVGALVKMCFSGKYTQDEI

KKKIKGNGGLVAYLNTNDAREVEERIEAGDEKAKLVYEAMAYQISKEIGASAAVLKGDVK

AILLTGGIAYSKMFTEMIADRVKFIADVKVYPGEDEMIALAQGGLRVLTGEEEAQVYDN*

>CD630_01140 Clostridioides_difficile_630_NC_009089 hypothetical protein

MITNDKRIRIITGHYGSGKSEFAMNYVVKLRDMVSGKVAIADLDVVNVYFRTREKKELMK

SLGIQPIDSSINAPTLDLPAVSAEVMSPMVDHSYNSVIDLGGDNVGARVIGRFSHLLKEG

DYDMLFVINANREKTQTSEEVIQYIKEIEKSSKLKVTGLINNTHLIRFTTIDDVLRGQKV

AKEVSEKCNIPIRYVACLENLVEQLPKDLEGEIFPIKLYMREDWM*

>CD630_01150 Clostridioides_difficile_630_NC_009089 4Fe-4S binding domain-containing protein

MAKGKVSFNQERCKGCGLCVEACPVKIIQLDSNVINKKGYNPATVFEMEKCIGCASCATM

CPDVVITVERD*

>CD630_01160 Clostridioides_difficile_630_NC_009089 ferredoxin/flavodoxin oxidoreductase subunit alpha

MAKILMKGNEAFGKAAIEAGCKYFFGYPITPQSELPEYLSRELPKIGGAFVQAESEVSAI

NMVYGGAGAGARVMTSSSSPGVALKQEGITYAVGAEVPCVVLNVMRGGPGLGSIQPSQAD

YFMSTRGGGNGDYRTPVFAPATVQEAVDMIMEAFDVADYYRSPVMVVADGMIGQMMEPVE

FRAPEKKRELPPKDWATVGTKGKRKPNVINSLYLEPEVLEDHCWHLQEKFDAMEKNEVQY

EMYKTEDAEFVFAAYGTTSRVVKSAIDILREEGIKAGLIRPKVLWPFPFEAFNQIPNARN

ILTVEMSMGQMVEDVKMAVEGKLPVYFHGRPGGMTPTPAEIVEKAKKIIAGELVAGGAR*

>CD630_01170 Clostridioides_difficile_630_NC_009089 ferredoxin/flavodoxin oxidoreductase subunit beta

MAVVFKKTEGLQDTQTHYCPGCTHGIIHRLVGEVLEELGVLGDAVGVVPVGCSVLGYKYF

NCDTQEAAHGRAPAAATGIKRVHPENTVFTYQGDGDLASIGTAEIVHAAARGEKITTIFV

NNTTYGMTGGQMAPTTLVGQRATTAQSGRNAETQGYPIRVSEMLATLTGAVFVERVAVDT

PAHVRQAKKAIKKAFQVQQAGLGFGIVEVLSTCPTNWGLAPNDALQWLRDNMIPYYPLGN

FKNVEVEEVK*

>CD630_01180 Clostridioides_difficile_630_NC_009089 ferredoxin/flavodoxin oxidoreductase subunit gamma

MSTARVICAGFGGQGVMSMGQLLTYAGMLEGKEVSWLPSYGPEMRGGTANCAVTVSNEPV

GSPLITDDATAAILLNIPAFEKFKDDVIPGGKIIVNSSLIKEKVDRTDVDVYYIPANELA

AELGNDKVANMIMLGAYLKVSDTVDIESVLEAFKKVFGPRKEKFVPLNREALQKGMDATC

SKATN*

>CD630_01190 Clostridioides_difficile_630_NC_009089 phosphoglucosamine mutase

VRKYFGTDGVRGVANTELTCDLAYKLGRAGGFVLAQGDHRVKVVVGKDTRISGDMLEASL

IAGLMSVGCDVITVGIIPTPAVAYLTRKYGADCGVVISASHNPVEYNGIKFFNKNGYKLD

DEIELKIEEYIDDIDKIDCLPIGENVGRKLHEHCAQRDYVDYLKSIISTDFKGLKVVLDC

ANGASYKVAPIVFDELGASVISINSSPDGNNINYKCGSTHPEQLQRAVLEHNADLGLAYD

GDADRLIAVNEKGQIVDGDHIMILSALNLKKNNKLAQDTLVVTVMSNIGLTIAAKENGIN

LSTTAVGDRYVLEDMVKNGYNLGGEQSGHMIFLDYNTTGDGVLSSLILANIILQEKKPLS

EIASIMSQYPQVLVNATIKNENKNKYMEYPEIKTEIERIESILDGNGRVLIRPSGTEPLV

RVMLEGKEEGQIKELATNLANLIQEKLS*

>CD630_01200 Clostridioides_difficile_630_NC_009089 glucosamine--fructose-6-phosphate aminotransferase

MCGIVGYLGSRKAAEVIVEGLSKLEYRGYDSAGVAVNSSNEKELNIRKFKGRLSVLAEDL

EKNPIDGNLGIGHTRWATHGEPSDVNSHPHFNQAKTIAVVHNGIIENYMEIKEELISEGV

KFESQTDTEVIAHLVDKYYEGNLLDAVYKTISKLRGAYALGVICKEHGNELVAVRKDSPL

VVGVGEGENFIASDIPALLKYTRDVYFLENGEVVHLKDENVTVYDSNRNLVEKEVFHVTW

DVEAASKGGYDYFMSKEIHEQPTGVRETLERRLDDNGNIILDSINISKEDLEKINKVYIV

ACGTAYNAGLLGKYAIEKFVNIPVITDIASEFRYSDPFVDENSLVILVSQSGETADTLAV

LRDSKAKGARILSITNVVGSSIARESDDVFYTWAGPEVAVASTKAYTTQITSLYMIALDF

AIKKGTITREFYDSMISKMKEIPSKIQEILDNEEYIKEVAKTVVSSEHAFYLGRGIDYSL

AMEGSLKLKEISYIHAEAFAAGELKHGTIALIEKGTPVIAIATQEKLFEKMVSNMEEVRA

RGAYVVAIAQSHNKDVEKAADKIIYIPNSDDILSPILAVVPMQLLAYHVSVLRGCDVDKP

RNLAKSVTVE*

>CD630_01210 Clostridioides_difficile_630_NC_009089 thiosulfate sulfurtransferase

MTNDFKKEKKEDYFVNLKAISTEVLQEKVQDNAWVIVDTRLNDAYNGWKLDGVKRGGHIK

GAVDFSANWLSVYSDRKDEVLEQALKTKRIDLDKNIVLYDANGKDALVVADYLSKKGYKY

LYKYDIKQWADDENLPMERYKNYQMIVPAFIIKDILDGKIPETFEDSKNIKMIEASWGEE

SYTKGHIPTSVHVNTDIIEPPPTWMLDNDDNLTKFALDYGLTKDDTVIVSSSTPMASYRL

AVILRYIGVKDVRVLNGGTNSWLSAGYELEFISNPKHSCTNFGADIPVNSQLIVTTSELR

QKLKEKNKFILVDNRTWDEHIGKVSGYTYYDKKGRIPGALYGHSGSDSVSLEEYRNIDNT

MRNKYEILEMWDKENIDVNKQLIFMCGSGWRAAEVLTYANVIGVENTSLYSDGWMGWSLD

NSNLIEVGEHK*

>CD630_01240 Clostridioides_difficile_630_NC_009089 stage II sporulation protein D

MKNPLVVLLGFVTCSVLVPSLITLVSYKNVELTEKPESPVSINKTIKKSDIEDKGNKEEK

NTVNYETVNKKAPIINVYNHITGKTEKMDMENYLCGVLAGEMSSEFDIEALKAQSVAART

YVVYKQEHGKSSKHKNAVVCTDYKHCQEYKSYDTLKKLNGEEWIKNKYSKIQEAVRGTKG

QIITYNDKAILPLYFSTSSGKTENSEEVFSAKYPYLKSVESPYDKYSPKFASTLKISNTD

FVKSLRRAYSTIVIDVNNLSKQVSITKRSDAGTVEKIKLGNKELTGKDIRTVFKLNSANF

DIKFGEGYIDFVVKGYGHGVGMSQWGAEGMAEEGYKYYDILSHYYTDTKIKDIY*

>CD630_01250 Clostridioides_difficile_630_NC_009089 cell wall endopeptidase

MKKKLLEKDGFYLSLFVCVCLLAVGGVWFTNNNVDKLASNKGIMENANKDSEEEIHLIEK

DKKDAIPTATDSKQNLEKAKSKEENKSSTTKLNYIGDKVIRGYSEKEPSYSKTLDVWETH

KGVDISCTKGKEVKSLLNGIVVDVFDDEEYGQSVKIKSDNNIVVVYSNLDKNVSVKKEQK

VTEGQSLGTVGSTSQIESEEGIHVHLEAYSGEKSIDPMSLIK*

>CD630_01260 Clostridioides_difficile_630_NC_009089 stage III sporulation protein D

LRSHIEERAIVVAKYILEKNTTVRQTAKTFGVSKSTIHKDVTERLKEINPSLAKEVKNVL

DKNKSERHIRGGLATKLKYEKEHKKM*

>CD630_01290 Clostridioides_difficile_630_NC_009089 sporulation protein

LKGVFLLYLAKVNYSDDDVIQMLSSVLKTIINENTIVVCIGTDRAIGDTLGPLVGTILKN

SNFKYPVYGTLDNPIHALNIYESLDTIKNTHIQGNFLAIDACLGSQSNIGNIQIREGPIL

PGKGVGKKLPQIGNYSIVGIVDKIDENNKLSFNNIRLSFILDLAETIALALLVST*

>CD630_01300 Clostridioides_difficile_630_NC_009089 S-adenosylmethionine synthetase

MARHLFTSESVTEGHPDKICDQISDSILDALLEKDPQSRVACETTVTTGLVLVAGEISTS

AYVDIPKLVRETVREIGYTRAKYGFDCDTCAVITSIDEQSGDIAMGVDEGLESKTGEEIE

EEIEKVGAGDQGIMFGFACNETPELMPLPISLAHKLSRRLTEVRKTGLVDYLRPDGKTQV

TVEYEGSKAVRVHTVLISAQHCETVSNDKIREDLINHVIKEVIPAELLDEETKIYINPTG

RFVIGGPQGDTGLTGRKIIIDTYGGYSRHGGGAFSGKDPTKVDRSAAYAARYVAKNIVAA

GLADKCEIELAYAIGIARPLSIFIDTFGTGKVSEEKLVELVNKHFDLRPGAIIRDLDLRK

PLYKKVAAYGHFGRTDIDLPWERTDKVEQLRKDALGE*

>CD630_01350 Clostridioides_difficile_630_NC_009089 PTS system lactose/cellobiose-family transporter subunit IIA

MEEIVLKIIIHAGNAKSMLYEALDYAKENDFKKADELIENANEEILKAHKVQTELIQKEA

GGDKSDISILLIHSQDHLMTCMSERNLIKEMIMLRKEIQKLK*

>CD630_01360 Clostridioides_difficile_630_NC_009089 PTS system lactose/cellobiose-family transporter subunit IIB

MKRKVYLFCSFGMSTSLLADKMQKVADEHNLPIEVEAFPIAEIDKIVEEKNPDCILLGPQ

VKYMLKELKPKFEAQGKLIDIINEVDYGTMNGEKVLKLAIKLIKSKQA*

>CD630_01370 Clostridioides_difficile_630_NC_009089 PTS system lactose/cellobiose-family transporter subunit IIC

MNRLERILMPLAEKIGKNKLLIAIRDGFLVSSPLLIIGSLFLLVANFPIKGWNEFVGQIF

GPDWAVKLQQPTVATFEVMTLLAVLGIGYSYAKQIDVDPIASAAVAIVAFFIITPFVIPY

TPEGTESVYMVTGIPLGWMGSKGMFVGMITAITSVKLFAAVVKKGWTLKMPDGVPPTVSK

SFAALIPSAIVMVVFFLVKIAFEATPYGNVHEFIFNFLQIPLLKLGNTLSATAIAYVFLH

LFWFFGINGSSVVGAVYNPILKILSAENLAAFQAGTKLPNIITAQFQDMFATFGGAGSTL

SLIIAMLLICKSKRIKSIGKLSILPGIFGINEPLIFGLPIMLNPLLLIPFALVPTVNIII

AYFCMSTGLVPLTNGVQLPWTTPIIFSGFLTTGWQGAVLQLVLLILGIFMYIPFIKILDK

QYLKDEKSNVAEEDDDISFDDLVL*

>CD630_01400 Clostridioides_difficile_630_NC_009089 peptidase

MNIISNKIDELKEDLLSDIIDIVKIPSVKGESENGFPFGEKVGEALNKALEISEKLGFKV

RNLDNYIGYAEHGDSDDYVCVIGHVDVVHEGDGWKHQPYKGEETNGRIYGRGVLDNKGPI

MSALYGLYAIKELNLKLDKSVRIIFGTNEESGFEDIPYYLEKEKAPIMGFTPDCKYPVVY

GEKGMAKIRIKSKINYEEDVYLGFIENMSENVLVTYKELNIENSDTILDIKVKYDFSYKL

KDVLDEIKASFPNSIDIEVISNFNPVYFDKESNLVKKLQLAYERVTSLDGTPVTTNGGTY

AKVMPNIVPFGPSFPGQKGIAHNPDEYMDIEDIILNAKIFANAIYELAKE*

>CD630_01410 Clostridioides_difficile_630_NC_009089 copper homeostasis protein

MLEIIGMSVEDAKIIEDCGADRIELVSALTEGGLTPSFGLIESVVNSVKIPVNVMIRHHA

KSFVYSKEDISIMQKDISVVKEIGANGVVFGVLDKNNNIDEKNLNVLLKCCDNLDVTFHR

AIDESNTIDSVKILKDYDKITNILTSGGKGSIVHNIQMIKNMMLSSNHIKILLGGGLNFN

NIEKIKELTKASNFHFGTAIRINNSPFEDIDRQKLKQLVNIISR*

>CD630_01430 Clostridioides_difficile_630_NC_009089 protein translocase subunit SecA

MSFMDNLFNMADKKELKKFNKTVDIIDSLEPKFESMADSELKNMTNIFKERLANGESIDD

ILPEAFAVVREVSKRVLGLRHYRVQMIGGIVLHQGRIAEMKTGEGKTLVATAPVYLNALT

GKGVHVVTVNDYLAKRDRDQMAKIYEFLGMSVGVIIHGQNPKVRKEQYDCDITYGTNNEY

GFDYLKDNMVIHKEQRVQRGLNYAIVDEVDSILIDEARTPLIISGPGDKSTHLYSDANTF

VLTLKPDDYELEEKDKAVSLTASGIQKAEVYFNVDNITDISHTELYHHINQALRAHVIMK

KDVDYVAKDGEIVIVDEFTGRLMFGRRYSEGLHQAIEAKEGLKIQRESKTLATVTFQNYF

RMYKKLSGMTGTAKTEEEEFKAIYKMDVFQVPTNKLMIREDLPDCVYKSEIGKFNAVAQE

IIERHKVNQPILVGTVSIEKSELLSQILKKKGIKHEVLNAKHHDKEAEIIAQAGRLGAVT

IATNMAGRGTDIVLGGNPDFLTKREMRRNGFKEEIVNRVDTPIEGIPVKGNEILFEAREE

YEKLFEKFKQQTQEEQKQVVEAGGLAIIGTERHESRRIDNQLRGRAGRQGDPGSSRFYIG

LDDDLMRLFGSDRISGIVDKIGLEEDMPIEHRILSKSIEGAQKKVEGKNFGIRKHVLQYD

DVMNKQREIIYAERKRVLEGEDLQEQIQSMTHSIIEEAVTLYTQDKGFDEEGFKEHMYNL

FLPKGSIEIPEIEKLNPVEITEKVYEIAMKIYTSKEEQVGYERMREVERVILLQAVDNHW

IDHIDAMDQLRQGIGLRAVGQQDPVIAYKMEGFDMFDEMNKHIKEDTVRYLFNITIETPV

ERKAVVDVENLSSPSDGTLPTSKTVKKDEKVGRNDLCPCGSGKKYKNCCGR*

>UAB_RS0201575 Clostridioides_difficile_ATCC_43255_NZ_CM000604 peptide chain release factor 2

LMKNIQINEEKMNQQDFWNDNEVAQRVLQENKSLKETLEEYESLKSLLEDIEVLIEIGLE

EDDDSVERDIEKSIESMEEKLSEMKIKTLLNGEYDKNNAILSINAGTGGLDAQDWAQMLL

RMYIRWSESKGYKVKLLDIISDPEAGIKTATILVEGTNAYGYLKSEKGVHRLVRISPFDP

SGKRHTSFASIDVTPELDENIEVEINPSDLKIDTYRASGAGGQHVNTTDSAVRITHIPTG

VVVQCQNERSQHLNKDRAMRLLMAKLIELKELEQKEKIEDIQGKYSQITWGSQIRSYVFQ

PYKLVKDHRTNAEFGNVDSVMNGNIDLFINEYLKMNKIV*

>CD630_01450 Clostridioides_difficile_630_NC_009089 S1 RNA-binding domain-containing protein

LDINQILKKEFNLRDEQINNTLKLIDEGNTIPFIARYRKEMTGEMSDVTLREFYEKLMYL

RNLQSRKDDVVRLIDEQGKLTDEITQNIEKAKTLQEVEDIYAPYKQKKRTRATIAKEKGL

ENLALSILENNLDNIEIEAKNYLDEEKEVLSIEDALKGARDIIAELVSDDAKIRKYIREL

ALREGMIVSKSATDEKSVYDMYYDYSEAVKSMAPHRVLAINRGEKESFLKVKLEINNDKV

LNYIINEYVNDKNFKNKEEIVSSIEDSYKRLIFPSIEREIRNHLTEIAQERAISVFGKNV

KSLLLQPPVKDKVVMGFDPAFRTGCKIAVVDKNGKLLDYTTVYPTDPQNDVEGAKKVLKG

LIEKYDIDIISIGNGTASRESETFVSEMIKEIDSEVQYVIVSEAGASVYSASELANEEHP

DINVSIRGAISIARRLQDPLAELVKIDPKSIGVGQYQHDLNKKRLEEVLDGVVEDSVNSV

GVDLNTASYSLLEHVAGISKAIAKNIIAYREENGDFTSRAQLKKVKRLGPQAFTQCAGFM

RILEGKNPLDNTGVHPESYDICKKMIEIIGYSLDDVKNKNIGEIDEKIKEIGLRELSEKL

EVGQVTLKDIIAEIKKPGRDPREEGIKPILRTDVLKIEDIQEGMTLKGTIRNVVDFGAFV

DIGIKNDGLVHKSEMSNSFVKDPMSIVTVGDIVDVKVIGIDLNKKRVALSMKK*

>CD630_01460 Clostridioides_difficile_630_NC_009089 hydrolase

MIFIKNGKINTITNGIIHGDILIDEGKIIEIGEDLIAPLDVEVIDASNKLVFPGFIDAHT

HLGLWEDGIGFEGADGNEETDPITPQLNPIDGINPMDRTFKEAFEGGITSVCTTPGSANV

MGGQCIAIKTCGKRIDKMVIKNPVASKIAFGENPKSCYGQDDKSPQTRMAIAALLRENLK

KAEEYLEDIDMYESHDDEDCEKPEYDIKMESLIPVLRREIPFKAHAHRADDMFTAIRIAK

EFNLKLTLDHCTEGHLIVDELVEEEFPVIVGPSLSERSKFELRNLTFNTAGILSNAGLDV

CIMTDHPVIPVQYLPICAGIAVKHGMKEEKAIESITINPAKTLGIEDRVGSIEVGKDADL

VIWDNSPLEIQSNVLYTIINGKVVYEKK*

>CDIF1296T_00266 Clostridioides_difficile_ATCC_9689__DSM_1296_strain_DSM1296_CP011968 riboflavin transporter

MQNTAKRGNMISTRTLVVMSILSAISYLLMFIQAPIPGIFPDFLKIDLSDIPAIFGGMSL

GPLVGFGIVVVKNVLQAITATTTGGIGEFANILIGGSYVMILCYSYKRSGDLKGVLIGFV

LGTISMTIIGSLVNYFVMLPLYGQLMGLDAIIGLGSAINPQVHDLFTFVIWMIAPFNVLK

AVIISLVTLPLYKKMGNIIKK*

>CD630_01490 Clostridioides_difficile_630_NC_009089 P-loop ATPase

MAKIYLENENKTREIGYKLGKLLKEGSVICLVGDLGAGKTTMTQSLADSLGIEDYITSPT

FTIINEYEGKIPLYHFDVYRIGSSDEMYDIGYDEYVNSNGICIIEWANLIEDILPKEYLN

IELRYKDEGREMILTPKGEFYKEIVEELIK*

>CD630_01500 Clostridioides_difficile_630_NC_009089 peptidase

MKILGMDTSSMAASVAVVEDDKLICEFTVNNKKTHSQKLMPMIENMLSMSDLSIKDMDLL

AVCIGPGSFTGLRIGMATVKAMAHVNNIPIIAVNSLESLANNINFCDRKICCILDAQKNQ

VYSAKYKFENGEMIELDGVDVVEFDALVDEIVSTNEEFIIVGEAVYKYKDKLENIKNIKI

PSPANNVSKAGSLCSLALNKYNKNIDVHTCYTINPMYIRKSQAEVQYDEKMKRLNNGR*

>CD630_01520 Clostridioides_difficile_630_NC_009089 UGMP family protein

MSDIITLAIESSCDETAASVLKNGREVLSNIISTQIETHKKFGGVVPEVASRKHVENIDI

VVQEALDKANIGFNDIDHIAVTYGPGLVGALLVGLSYAKALAYTLNIPLVGVNHIEGHLS

ANYIEHKDLKPPFITLIVSGGHTHLVEVKDYGKYEILGKTRDDASGEAFDKISRAMNLGY

PGGPIIDNLAKNGNKHAIEFPRAYLEEDSYDFSFSGLKSSVLNYLNGKRMKNEEIVVEDV

AASFQEAVVEVLSTKALKAVKDKGYNIITLSGGVASNSGLRAKITELAKDNGITVKYPPL

ILCTDNAAMIGCAGYYNFINGKTHDMSLNAVPNLKINQ*

>CD630_01530 Clostridioides_difficile_630_NC_009089 4-hydroxyphenylacetate decarboxylase large subunit

MSQSKEDKIRSILEAKNIKSNFQNKENLSEFNEKKASKRAEDLLDVYYNTLSTADMEFPY

WYNREYRKSDGDIPVVRRAKALKAAFSHMTPNIIPGEKIVMQKTRHYRGSFPMPWVSESF

FVAQGEQMREEAKKLASNTADELTKFGSGGGNVTESFGNVVSIAGKFGMRKEEVPVLVKM

AKEWVGKSVEDLGFHYEKMMPDYDLKENLMSTLICMFDSGYTLPQGREVINYFYPLNYGL

DGIIEMAKECKKAVAGNASGDGLIGMDRLYFYEAVIQVIEGLQTWILNYAKHAKYLESIE

TDLEAKKEYSDLVEILEHIAHKQPRTFREALQLTYTIHIASVNEDAISGMSIGRFGQILY

PWYEQDIEKGLITKEEVIELLELYRIKITCIDCFASAGVNGGVLSGNTFNTLSIGGLKED

GSTGANELEELLLEASMRCRTPQPSLTMLYDEKLPEDFLMKAAECTKLGSGYPAWVNNSN

GTTFMMKQFADEGMTVEEARAFALGGCLETSPGCWKQLTLNGKTYSIAGGAGQSAGSGVH

FIANPKILELVLMNGKDYRMNIQVFEPHNKPLDTYEEVIEVFKDYYKQAINVLERANNIE

LDIWRKFDTSIINSLLKPDCLDKGQHIGNMGYRYNATLNVETCGTVTMVNSFAALKKLVY

DDKAFTIEEMKDAILNNFGFKDALEVGNYSMADQVKVDKTGKYDAIYKACLDAPKYGNND

LYADNILKNYEVWLSKVCEEAQSLYAKKMYPCQISVSTHGPQGAATLATPDGRLSGTTYS

DGSVSAYAGTDKNGVYALFESATIWDQAVVQNSQMNLKLHPTTIKGQQGTKKLLDLTRSY

LRKGGFHIQYNVVDSETLKDAQKNPDNYRQLMVRVAGFTQYWCELGKPIQDEVIARTEYE

GV*

>CD630_01540 Clostridioides_difficile_630_NC_009089 4-hydroxyphenylacetate decarboxylase small subunit

MRKHSDCMNFCAVDATKGICRLSKQMINLDDAACPEIKVMPKCKNCKNFVEANDEGIGKC

VGLEKEDWVYSTLNAITCEGHVFNE*

>CD630_01550 Clostridioides_difficile_630_NC_009089 4-hydroxyphenylacetate decarboxylase activating enzyme

MSSQKQLEGMIFDVQSFSVHDGPGCRTTVFLNGCPLSCKWCANPESWTVRPHMMFSELSC

QYENGCTVCHGKCKNGALSFNLDNKPVIDWNICKDCESFECVNSCYYNAFKLCAKPYTVD

ELVQVIKRDSNNWRSNGGVTFSGGEPLLQHEFLHEVLLKCHEVNIHTAIETSACVSNEVF

NKIFNDIDFAFIDIKHMDREKHKEQTGVYNDLILENISNLANSDWNGRLVLRVPVISGFN

DSDENISDIISFMHKNNLVEINLLPFHRLGESKWTQLGKEYEYSDKGDVDEGHLEELQDI

FLDNGIACYVGHETAF*

>CD630_01590 Clostridioides_difficile_630_NC_009089 hypothetical protein

MKFLNVLIVVEDIEKSKKFYYDVLGLKVICDFGENVVLEGNISLQEKKLWLEFINKSDSE

VKFNGNDAELYFEEDNFDTFVERLSTMKDIDYVHLAIEHRWGQRAIRFYDLDGHIIEVGE

TMSSVCRRFLDSGLSIDEVAKRMDVTVEYIESVLE*

>CDM120_RS01340 Clostridioides_difficile_M120_NC_017174 cyclic nucleotide-binding protein

LMKAEKTSIYPIIKNNLNKISIVEYKKGQRFVASDKDLQEVFFIIEGVALVECTTRGGNK

FLVDIVPENEFIGKISYIYEHNLKCDIFAKTNIKLFRFEKNVFEEFYLKPDFIALFHRKC

TRRIYELYKTRMVRELFSCTEVIAYCILLNKQENNVCNIKAIRNFSEIYSISRKSRYHSL

LRLTEKGIIKKMGNTYEILNYDELHRLAFEVKEFLEDE*

>CD630_01690 Clostridioides_difficile_630_NC_009089 MATE family drug/sodium antiporter

LQELFSLKDENKRFYKILLSLCIPIIIQNLISTSVNVIDTIMISSLGETSVASVGVANQF

FFLFNMSLSGITGGAGVFISQFYGKKDVSNIRKVTGLTCVLAIVLSFVFVIPALLTPKPI

IHIFSYDSEVVKLCIDYFSIAVFSYPLIAVSTVFSTGSRGVRNPKLGMICSAFALVTNVI

LNYGFIFGNFGLPALGVKGAALATVIARICELILMITYVYLYKKDYILKFGLKNLKAIDK

IFIKSFSSKSFPIFVNDSVWAIGTVLYSVAYARAGTSAIAASQIATSTGNFFIMTAVCIA

SGASIMLGNELGADHIKRAIEYAKKFSILVFSAGLILGIILILNIPLLLKMFSVSDSLAS

DITKIFFIMGILMALKSFNTLVIIGILRSGGDTKYALFLELGCMWLASIPLTFIAAFKGA

PIFVLVLLTYSEEVVKFIFGVPRALSKKWAINIVKEID*

>CD630_01700 Clostridioides_difficile_630_NC_009089 ABC transporter ATP-binding protein

MIVLSCNNLNKSFGIDSILENISFTVNEGDKIGIIGVNGTGKTTLFKIISGIYGYDSGDI

YTSKDCEIGYLEQNTNFYSDNTILEEVLEVFKNLIEMESYLRELEVKISEESTKTNSPII

EKIMDEYSHKLELFSDLNGYGYKSEAKGVLKGLGFSDNDMDKPISILSGGEKTRVLLGKL

LLKKPTLLLLDEPTNHLDSEAIEWLEVFLKQYKGTVMLISHDRYFLDQSVNRIFEVHNKK

LKVYNGNYSKFVELSKIEKELELKKFEDQQKEIKKQEESIERLKAYGREKHLKRARSKEK

ALDKVDVLDKPEAYRKKARIQFTPSVQSGNDVLQIRDVSMGYGERILFKDLDLDIYRGEK

VALIGANGVGKSTLFKIITNELQPLSGNIKFGTNVHVSYFHQEQKTLNLDNTIIDEIWEN

NTHLTQTTLRNMLGAFLFVDEEVFKKISTLSGGERARVAILKLILSNANLLLLDEPTNHL

DIDSKEVLEEALTNYDGTIFTISHDRYFLNTVVDKILVLDENGITEYLGNYDYYIDKKRQ

IQEMSIIEEKEEKTRTQIKDEKRKEREQREIEKKNRIKRQNIEKEIEKLEIEIEKLDILL

CQEEVYSNPDKAKEVSQEKINLENNLASLYDEWEEFM*

>CD630_01710 Clostridioides_difficile_630_NC_009089 redox-sensing transcriptional repressor Rex

MLGNKNISMAVIRRLPKYHRYLGDLLDRDIQRISSKELSDIIGFTASQIRQDLNNFGGFG

QQGYGYNVEALHTEIGKILGLDRPYNAVLVGAGNLGQAIANYAGFRKAGFEIKALFDANP

RMIGLKIREFEVLDSDTLEDFIKNNNIDIAVLCIPKNGAQEVINRVVKAGIKGVWNFAPL

DLEVPKGVIVENVNLTESLFTLSYLMKEGK*

>CD630_01720 Clostridioides_difficile_630_NC_009089 hypothetical protein

VVFVEELWKKAIEFHGHECPGLAIGFKAALAAREYLGCKQSGDEEIVCISENDACGIDGI

QVVLSCTMGKGNLLLRMTGKSVYHFFNRENGKSIRIYVKSKPFQMDREGYKKYLLESDFE

EIFEITDTKLRLPEKARIFKSLKCEVCGEFASEQYIRIEEGKKVCLDCFNKYERFYEEY*

>CD630_01721 Clostridioides_difficile_630_NC_009089 ferredoxin

MAYKITDACISCGACEAECPVSCISAGDDAYVIDAGSCIDCGSCAGACPVDAPQPE*

>CD630_01740 Clostridioides_difficile_630_NC_009089 carbon monoxide dehydrogenase

MACNTCKMCESADKKLESFVASKDVETAFHRTEDQKVKCGFGLQGVCCRLCSNGPCRVTP

KSPRGICGADADTIVARNFLRAVASGAACYLHVVENTAKNLKNVGITKGVVKGEKTLNQL

AEMFGIESDEKYDKCIKVADKVINDLYKSRDDKMELVEKIAYAPRVKKWKELGIMPGGAK

SEVFDAIVKSSTNLNSDPVDMLVNCLNLGISTGLYGLTLTNLLNDVMLGEPVIRMAPVGF

NVIDPDYINIMITGHQHSTFANFQDRLKDEDVIKLAQSVGAKGFKLVGCTCVGQDLQLRG

EHYQEVFAGHAGNNFTSEAVLSTGAIDIVLSEFNCTIPGLEPIADKYKVKMVCLDDVAKK

ANAEYIGLDRSKLDELSNTLIEKALESYKERRGSIEIDIPKDHGFEQSLTGVSEKNLKAF

LGDSWKPLINLIAEGKIKGVAAVVGCSNMTAGGHDVNTVELTKELIKKDIIVLSAGCSTG

GLENVGLMSPGAEELAGENLKEVCKTLGIPPVLNFGPCLAIGRLEIVATELAAELGIDLP

QLPLVLSAPQWLEEQALADGAFGLALGLPLHLALPPFITGGKLVTEVLTEKLKDLTGGHV

IVNPDPKSSANQLEEIIIDRRKNLGLKDVECNA*

>CDIF1296T_00297 Clostridioides_difficile_ATCC_9689__DSM_1296_strain_DSM1296_CP011968 oxidoreductase, electron transfer subunit

MWNAMHRIFINKDLCTGCKSCVLACMLKHNKDYDMYTLDLENIDNDSRGHIELDSKHNNP

VPILCRHCDEPECVLACMSGAMHKDSESGIVSYDEEKCGSCYMCVMSCPYGLLKPDDRSK

QNILKCDLCKDEEYPRCVANCPSGAIELQKEENDELCSVRS*

>CD630_01760 Clostridioides_difficile_630_NC_009089 oxidoreductase NAD/FAD binding subunit

MNYVVLGASAAGINAVKTLRELDKDSNIVVISKDENVYSRCMLHHVISEHRTLKQINFVD

EDFMEQNNVKWIAGKTVKGIDINKKVVQTEDITVNYDKLLIATGASSAIPPIKNLRDGNF

VYSVRNIDDIYKIKEKAQNSKNVVIIGAGLVGIDALVGLFKYEQLNISVAFMEKYILDRQ

LDEYTASVYENKFKEKGVKFYPSASIQEIVLDNSKNVTGVAFSNGEVLDADMVIVATGVK

PNADFLDGTGIEYDRGIIIDDMCQTTQKDIYAAGDVVGKNAIWPLAVKQGIVAAYNMVGK

DKKIEDEFAFKNSMNFMDIPTISIGMNTPVDDSYKVLTRHGINDYKKFVFKDNVIYGAVI

QGDISYVGVLTYLIKNKVEIYDLENRIFDICYADFFNIKENGEFCYSV*

>CD630_01790 Clostridioides_difficile_630_NC_009089 NAD-specific glutamate dehydrogenase

MSGKDVNVFEMAQSQVKNACDKLGMEPAVYELLKEPMRVIEVSIPVKMDDGSIKTFKGFR

SQHNDAVGPTKGGIRFHQNVSRDEVKALSIWMTFKCSVTGIPYGGGKGGIIVDPSTLSQG

ELERLSRGYIDGIYKLIGEKVDVPAPDVNTNGQIMSWMVDEYNKLTGQSSIGVITGKPVE

FGGSLGRTAATGFGVAVTAREAAAKLGIDMKKAKIAVQGIGNVGSYTVLNCEKLGGTVVA

MAEWCKSEGSYAIYNENGLDGQAMLDYMKEHGNLLNFPGAKRISLEEFWASDVDIVIPAA

LENSITKEVAESIKAKLVCEAANGPTTPEADEVFAERGIVLTPDILTNAGGVTVSYFEWV

QNLYGYYWSEEEVEQKEEIAMVKAFESIWKIKEEYNVTMREAAYMHSIKKVAEAMKLRGW

Y*

>CDIF1296T_00303 Clostridioides_difficile_ATCC_9689__DSM_1296_strain_DSM1296_CP011968 membrane-associated metalloprotease

MSNFILNGIFRTVIVSSLSILMILIFKKNVFKRFSKKFNYYIWMIVVIKLFLPFTYYTFT

INILRSKKHININNINLEGFNNVSTISNNIILYTWIITVVVYLIYTIFKYIKLKNLINDL

SYDVDDGEIVNLYKNILEEFNITKDIKLKYSYEVETPAFFNSCVLLPPHEYKLKELDWIF

RHELMHFKSRDLYLKYVILFLKTVYWFNPFIYIMDKHIDLDCELYCDERVLKNRNSDEKK

DYALTILNAMRKGSNTSNKFIAGLHKQSDIKKRVMNMFNEKYKNGILMALTLCLLSSITF

LKVNSTSIVNLNILPKYDMEKLHVFRGSVTLLEFTYADAPENVKKEHKRMYEAMGLIPKD

SDKISFGVKYYDKLNNLLR*

>CD630_01840 Clostridioides_difficile_630_NC_009089 aspartate carbamoyltransferase

MLKSRNLIQPEDFSIEEIDEILELAQKIIDNPSKYSRICEGKLLATLFYEPSTRTRLSFE

SAMNRLGGRVVGFSEPNSSSASKGETLGDTMRIVSGYVDIIAMRHPQSGAASEAARYTEV

PFINAGDGKNQHPTQTLTDLLTIKSLKGTLESHTIGLCGDLKYGRTVHSLVKAMARYKNT

KFVFIAPEELKMPDYIKEAIKGHAYYETNNLDDVIGSLDVLYMTRVQQERFEDKSEYERL

KNYYILNKAKLEKASKDMLVMHPLPRVNEIDIDVDSDDRAVYFKQAKYGMYVRMALIIKL

LGINED*

>CD630_01850 Clostridioides_difficile_630_NC_009089 dihydroorotate dehydrogenase electron transfer subunit

MYKILENIYIGEDMYRMKVKGNFEGKMGQFYMLRAWDTYPVLSRPISIHDIDEEGITFLY

KVVGEGTQILSNLKVNDNIKLEGPYGNGYAKVDGKVALVGGGIGVAPLYLVAKNIKNCDA

YLGFREDVILEDEYKQVCNKVYTTVGNTFVTDIIDVEKYDYILTCGPTPMMEKLVKMVEG

TKTRIMVSLENHMACGVGACLVCTCKTNGGNKKTCKDGPVFWGEDVIFNG*

>CD630_01860 Clostridioides_difficile_630_NC_009089 dihydroorotate dehydrogenase 1B

MGNLSVKFGSVEFKNPVIMASGTFGFGKEYNEIYDIQKLGGISSKGLTLNKKPGNNGMRV

HETSSGMMNSVGLENPGVQGFIDYELPFFSKLDLVRIANVGGGTLEDYLLGVQMLNDKPI

DIIELNISCPNVKAGGMAFGIKNEVAREVVREVRNITKLPLVIKLSPNAEDIVGMAKVCE

EEGADGVSLVNTFKAMAIDIKNRRPVFENVYAGLSGPAIKPIALRMVHEVCKNVNIPVMG

MGGITKATDAIEFIMAGATCIQVGTANFINPRIGIEIIDGINEFMDREGIKSLDEIRGII

*

>CD630_01880 Clostridioides_difficile_630_NC_009089 2-dehydropantoate 2-reductase

MKNQLNENNKKVVFFGVGAVGATFAEQFFNSKYDFKILCDNERKKRYLEEGFIINGKRYD

FDYVTKDEYKQEADFIIIGLKYNNLKENIKELDGLVGKNTVIMSLLNGVDSEEIIGERFG

IEKMVYSYVTNIDAKKINNNIIHTTNGIIVFGNKDNSEDRKTNIITEVFDDVNIEYTLSK

DIQRDMWWKYMVNIGVNQTSAILGAPYGVFQSSEHLRELAKSAMREVVAIAQAKDISLTE

DDVEHSLHRILEHSKEGRTSMLQDVEAHRLTEVDMFSKNICKLGKKYNIPTPINQTFFYM

IKVIESRF*

>CD630_01890 Clostridioides_difficile_630_NC_009089 LysR family transcriptional regulator

LFEELKTFVAVVEYKNFTKAGEYLNLSQPSVSKHIKNLENYFKVVLINRSIKQKTIFITE

SGQILYKRAKEILNLLNITYHDVSQVSDAITGHLKIGASLTIGEYILPNFLALFSKKYPD

IDVEVFIKNTSIVSSHVKDYILDIGLIEGTCSSPSFIQEYFFEDKMVLALPYKSHLLKDF

SFDKLQNQKWIVREDGSGTRDYLDMFLSVKEIIPKSMMVFGSNYAVKESVRNNLGITIVS

NLVTSLPVLNNELSVIELGSSYNRHFSYIFPKDITLSKAATIFIEELKIFSNLNSI*

>CD630_01900 Clostridioides_difficile_630_NC_009089 membrane protein

MLKKKSSLTLKTIREILPGLFVSVLVGYISIFISILIPKVGAASISIFLGMFVGNLFLNQ

KVFQKGYKFSETDLLSYSIVLLGATLSVSTLIDLKVSGILFIILQMTITIIAALYIGKKL

GFEENFRFLMASGNAVCGSSAIGATVPVVNATDKEKGIAVTIVNVTGIFLMFLLPIISQF

LYSHELVKTSAMIGGTLQSIGQVVASGAIVGENVKDLATIFKIVRVIFLVVVVLVFGHMK

NQSNREILEEEKDEIKKKKVTIPWYVIGFFITCALFSMNIIPQEVSVLCKEISNKLEIIA

LAAIGLKVNVKDLVKQGKEVSLYGLFVGTVQVVSAVVLIKIFI*

>PCZ31_RS01325 Peptoclostridium_difficile_strain_Z31_NZ_CP013196 8-oxoguanine DNA glycosylase

IWYMNVYEKGNGVILEGVTDFDPVHIFECGQCFRWHKQEDGSYTGVAKGRILNVKKENDK

IYLNNTNLKEFNSIWYNYFDLGTDYTEIKNKLKNMDEYLNKATEFGWGIRILRQDGWEML

ISFIISSNNRIPMIQRAIENLSRKFGKYIGEYEGNEYYAFPTPEELNKASQEEIRACQTG

FRDKYIKSTTQAVIENNDEVSEYTNLSTEDCRKELLKFNGVGPKVCDCIALFGMQKYDSF

PVDVWVKRVMQEFYIDEDMSLPKMRTYGIDKFKEMSGFAQQYLFYYARELGIGK*

>CD630_01920 Clostridioides_difficile_630_NC_009089 cardiolipin synthetase 1

MGVIGTIFLFYLIISYLAGAIISVIILLENRDPAKTMSWLLMFIIFPGVGLMIYAISGRN

IRKRKLFKTQKLANNIKEKKLFDTLEKITEIVELEKESIKQNKLLRDEEDGSYRKRVINM

LLKTGMFPFTKNNKVDVFVDGNEKFKRLIEDIREAKDHIHLEYFIIKDSEIGRVLKEELI

KKAKEGIKIRILYDDVGCWRFWFNRKFFREMREVGIEIAAFLPTKFPIIGGKLNYRNHRK

IVVIDGIIGYTGGINIGDEYLGKNDKFGYWRDTHIRIKGISVYMLQMTFLIDWYYTTKEV

LVTKNYFPSVGNVGESMIQVVASGPDSDWEDIHYAYFSAICQARKNVYIETPYFIPDESL

LKAIKSAALSGVDVRIIFPKIADHKIVNIASYSYFEEILRAGGKVYLYNKGFIHSKVVII

DDKIASAGTANMDLRSFMLNFEVNAFIYDEEVIRVMTDDFFEDLSYCEELNLEVFKNRNI

IQKIKESVARLFSPIL*

>CD630_01930 Clostridioides_difficile_630_NC_009089 chaperonin GroES

MKIRPLADRVVIKKVEAEEKTASGIVLPGAAKEQPQIAEVVEVGPGGIVEGKEIKMELTV

GDKVIFQKYSGTEVKIEGQEYTILRQSDVLAVIE*

>CD630_01940 Clostridioides_difficile_630_NC_009089 chaperonin GroEL

MAKEIKFSEETRRALEAGVNKLADTVKVTLGPKGRNVILDKKFGSPLITNDGVTIAKEIE

LEDRFENMGAQLVKEVATKTNDVAGDGTTTATVLAQAIIREGLKNVTAGANPILLRKGIQ

KAVTVAVEELKNQSRIVETQEAISQVASISAGDEEVGKLIAEAMEIVGKDGVITVEESQT

MNTELDAVEGMQFDRGFVSAYMVTDVDKMEAVLNDPYILITDKKISNIQELLPVLEQIVQ

QGKKLLIIAEDVEGEALSTLVVNKLRGTFDVVAVKAPGFGDRRKEMLQDIAILTGAQVIS

EELGYDLKEADLSMLGRASSVKVTKESTTIVDGSGDKKAIEDRVTQIKHQVEQTTSDFDR

EKLMERLAKLAGGVAVVKVGAATEVELKERKLRIEDALNATRAAVEEGIVAGGGTAFVSV

IPAIGTLIESLEGEVKLGAQIVKKALEEPLRQIAINAGLEGAVIVQNVVNSEAETGFDAL

NEKYVNMIEAGIVDPTKVSRSALQNAASIASTFLTTEAAVADLPEKEDAGMPGMGGGMPG

MM*

>CD630_01950 Clostridioides_difficile_630_NC_009089 NCAIR-mutase PurE-like protein

MDLRKLLEEVKNENIDIDFALEKLKDLPYEDLGYANIDHHRELRNGYPEVIYCEGKSDEH

ILGIIDKMNQKSSNILGTRCRKETFLKIKEIYNHAEYEEASKILKIQNHDIENIGKGKIV

IATGGTSDIPVADEAYHTAKFLGNDVDRIYDVGVAGIHRLLNKRHKIDSARVIVAVAGME

GALASVIGGLVDVPVIAVPTSVGYGANFGGLAALLAMLNSCASGISVVNIDNGFGAGYLA

SMINKL*

>CD630_01980 Clostridioides_difficile_630_NC_009089 GMP synthase

MKHELVLVIDFGGQYNQLIARRVRENNVYCEILPCTASIERIKEKNPKGIIFTGGPNSAY

LEDSPTISKEIFELGVPILGICYGIQIMSHVLGGVVRKGNKQEKEYGKTAITYGKSSLFE

GITTNSVWMSHTDLIEKVPEGFTIVANTNDCPVAAMENVERNLYGVQFHPEVEHCLEGDK

ILTNFLYNICKVKGDWTTDSFIEDKIKELKEKIGDKKALCALSGGVDSSVAAVLIHKAIG

DNLTCIFVDHGLLRKNEGNDVERIFREKFDINLIRVNAEDRFLSKLKGVSEPEAKRKIIG

EEFIRVFEEESNKLGKMDFLVQGTIYPDVIESGHGNAATIKSHHNVGGIPEDVDFQEIVE

PLRELFKDEVRKIGLELGIEEGLIFRHPFPGPGLGIRVIGDVTKEKCDILREADAVYMDE

LRKAGLYREIWQAFATLPDVKTVGVMGDERTYAYLVGLRAVTSSDGMTSDWYKMPYDVLE

RISNRIINEVDGVNRVVYDITSKPPGTIEWE*

>CD630_02030 Clostridioides_difficile_630_NC_009089 UvrABC system protein A 1

MKINNYKNQSIITNPKKFENKYQDLPKTPIELLKVVQSLVIHGDQGKLYGISFNKQQSDE

ELLRTIPQMLKRIFEINSNPLTIPRNPKQRLVGMCRDYSLLLVSLLRYRGFEARMRAGFA

NYFESELTYEDHWLVEYYDTLKKRWIRIDAQIDDIQKNYFQINFDTHDVGKTDGFLTGSE

AWIRCQQGHAHPDDFGYNKNWKGWHSVKGNLLHDFNNMIGLELLPWDLWTELSSKKYNQL

TRAEKNLLDEMAEILSSGNIKIEDLNLLIEKLPEDYLKSIFSQLKILGISEIKELGNPLE

LEKKFKFTKSINKSIKNSLCHNKSSIYLKGGRQNNLKDVEVTIPKNQITVITGVSGSGKS

SLAFDTIYEEGKRRYFENLSNGAKLSEQLQKPEFDLLQGLTPTIAIEQKKGSQNPRSTVG

TLTSIWDYLRMLFVSIGKSYCPYCKIPLEKKNNTKNYCPHCQTIFSKINTSTFNANSHTG

ACHDCNGLGFTYQVNPQLIVKDPTISILDGATYYFGKLRGKKPNGNWMVGELYAIAKDKN

IDLDIPWNELPRDFIDAILYGTDDKIYEFSFESKGRESKIRRPASGAINHIQRLFRESSS

ENNTLHQYMNKIPCNTCGGELLCIEARFTTIKGYRFPELTKMTIEQLWNWLCELPNQLQK

NELSLVNDILTELKIRVSYLLKVGLSYISTDRTAPTLSGGELQRVRLSSQLGSELVGLTY

ILDEPSIGLHPRDHNLIIKMIEELRDKGNTVIVVEHDKDTILSADYIIDVGPSAGTKGGF

IIAEGTTQEIIKNPNSITGKYLSTYNKTGSQNKTIPSKWLSLKGCHANNLKNIDVEIPLN

CMCSITGVSGSGKSSLVFHSLLPALEEKLKQKSIPDKNYTEFTGFDAIDDFILMDQTPIG

KSSRSTPATYINIFDEIRSLFAETPQAKQKLLDESYFSFNSKKGQCPNCQGLGKTKIILQ

YMADQWVTCSECQGKRYQKEILSIQYKGKTIADILDMEVAEAKTFFSDCSDIYRKLSLLD

EVGLGYLKLGQNTLGLSGGESQRIKLAKELGTKTKKRMLYILDEPTTGLHFKDIENLLIT

FRKLVNEQHSLLIIEHNTEVIRASDWIIDIGPDSGINGGEIVASGTPDEIKINPNSITGH

FI*

>CD630_02040 Clostridioides_difficile_630_NC_009089 signaling protein

MKLSRYKKNSLVLSIILCSLIISILVITVFNTVKLHNISNKQSKNYLNDVSTQIVMNVDS

KIKFILSDLRIMADFIKQYEGDSRYEYLSKRKTSYKYYDVGIIDLKGKAEFLSGKKFDLK

NTASYKKGIEGKEYSEVIESMGFVLYSVPIFNDNHEVSSILIGVSDKESMNEILNIDNFN

GKGTIEIINSKGKPLFIGKNTELIKDLSHKYKSSGDEPWAQKMLEDFKNDKSGNITITSS

KGVQCLLTYHPITRGLNDWHFLLIVPEDAVLGELNKLNSFTITMTFIITCIIGIITLILY

IIRRKYVNQIENIAYLDSITNGINSTKFSMLVKPLISESPDSTYMMIAMNIKDFKLINDC

FGSEKGNMTLKHLYNILYKNIDENEEFVCRHDADLFYLLIKNRPVLEALELLYKIEADTN

YFNKNRENPYFLRLSVGIYTIENHNEDLITIQDHANTARKSFNKTHQSDFSFYSDIERKR

LISEKEISNLMEKALENKEFFMCLQPKIDIKSGKISGAESLVRWKNSEKGIIYPSDFIPL

FEKSGFICKLDLYILEETCKLISKWIKEGKEVLRISVNVSRQHLNDKLFLEKYKHICNKY

NVPTCLIDLELTESIFLENPEAIDIIEDIHSNGFKCSIDDFGFGYSSLGILKDFKVDIIK

LDRSFFVSKNNIDRGKVVIKSIIELSKRLGMKVTAEGIEELEQVEFLKNVGCDYIQGYVF

SKPLLISDFENFAYENNKIKNIHY*

>CD630_02060 Clostridioides_difficile_630_NC_009089 PTS system fructose-like transporter subunit IIA

MNKLVNVKCSFKIHVNSKEEAIISLVDVVSKEGYLIDKNQFLKDVLKREETLSTYIGHGI

GLPHSQSVGVKNSCITIGKLDTPIEWTEEGEKVDLIFLISVTKDNENNLHLKILSKLARL

LMHESFRNQIRCSDEQTVYNLIKEKIEEED*

>CD630_02070 Clostridioides_difficile_630_NC_009089 PTS system fructose-like transporter subunit IIC

MRILKFLQKHLMTATSYMIPFVVAGGILFALSVTLSGQAAVPETGWLAKLNQIGAAGLAL

FIPILGGYIAFSMADKPGLAPGMIGAYLAKEVNAGFIGGIIAGFIAGFVVLQLKKIKLAP

TMRTLGSIFIYPLVGTLITGGIIVFLIGEPIASFMTWMTNWLNGMSGVSKIPLGGILGGM

IASDMGGPINKVAATFAQTQVDTLPYLMGGVGVAICIPPIGLGLATLLFPKKFSKEERDS

GKASLLMGCVGITEGAIPFATSDPVRVIPCIMAGSIVGNIMAFLLGCLNHAPWGGLIVLP

VVDNRLGYIASVLTGAVVVAVLMKLVKKDVKEDEEIEEDLDESIELIFEEL*

>CD630_02080 Clostridioides_difficile_630_NC_009089 PTS system fructose-like transporter subunit IIB

MKIVAVTSCPSGVAHTYMSAESLELSAKKFGIEIKVETQGSSGIDNELSLKDIDEATCVI

LTNDVEIRNMERFKGKKVVRMSVSEIIKKSDALIKKIKDTFQ*

>CD630_02090 Clostridioides_difficile_630_NC_009089 tagatose 6-phosphate kinase

MKKLPIKTVVQRLLNLQEEGKSATLLGIGPMSPNLLQASFELAKDDDFPLMFIASRNQVD

ADELGGGYVNGWNQETFTKDIKEVADKVGFDGLYYLCRDHGGPWQRDKERNDHLPVDEAM

ELGKKSYLADIEAGFDLLMIDPTKDPFEIGKVIPLDVVLERTVELIEYCENERKRLNLPD

IGYEVGTEETNGGLTSTETYETFITRLKVELDNRGLPMPTFIVGQTGTLTRKTEQVGTFN

FRNAYDLAQMAKKYGVGLKEHNGDYLDDVTLLEHIPSQIIATNVAPQYGTEETRAYLKLA

EVECKLEKEGLVEKTSNIRHVLLVNAIECGRWRKWVVGEQKNLTTEEIFKDEVLSNEILD

IAGHYTFNNDDVKKEIEVLYDNLSKNNIDGQRFVVDHIKRPLRDYAECYNLKGVTTRILN

K*

>CDIF1296T_00419 Clostridioides_difficile_ATCC_9689__DSM_1296_strain_DSM1296_CP011968 hydrolase

LINLIFDVDDTLYNQLTPFYTAYNKVFSSIKDISIEDLYMSSRKYSDEVFHMTENGEMPI

KEMHIYRIMKAFEELGNSITEKDAQSFQDEYIYQQSQITLIPEVERILNFSKERNINLGI

ITNGPSNHQRMKLKQLNIENWVDKSNIFISSEVGFSKPDTNIFRVAENVMNLDRENTYYV

GDSYRNDVLGAKKAGWKSIWLNHRGHEVEELFYKPDFVILEHKDLISLFIKICSYKNM*

>CD630_02130 Clostridioides_difficile_630_NC_009089 spore coat protein

MRNMIENLIKNNTYIDDKVIMLSMLSSAKASANMYLNSALTSSTPELRAIYSASLTQMVE

GHTALTELSINKGWVKPYDKPIQQLTCSYKESQNVID*

>CD630_02170 Clostridioides_difficile_630_NC_009089 nitroreductase

MLEVIKNRHSIRTYIDKNIEEDKITEILKSAMQAPSSKNAQPWEFIIVDDKELLKQLSKS

QHRAKHIEFAPLCIVVLGNRDKFLKPGKWIQDLGACTQNLLLEVTNQGLAACWAGVFPKN

KVVNKVRQTLDLPLKLVPYALISIGYSEEKNEFIDRFDENKIHRNVYKNR*

>CD630_02180 Clostridioides_difficile_630_NC_009089 5-(carboxyamino)imidazole ribonucleotide mutase

MKVAVVMGSKSDYPKLEEGIKLLEKYGIEVVARALSAHRTPEQLSIFLKEIEDDTDVIIA

AAGKAAHLPGVIASQTLIPVIGLPIKSSTMDGLDSLLSIVQMPKGIPVATVTIDLGLNAA

LLALQIMTLKYPKLKEDLKSYREEMAQKVLEDDKNLRG*

>CD630_02190 Clostridioides_difficile_630_NC_009089 phosphoribosylaminoimidazolesuccinocarboxamide synthase

MLLYEGKAKQVYSTDNENEYVVYYKDDATAFNGEKKAEISSKGILNNKISTIIFEMLKEN

NINTHFIKSLSDREMLVKKVEILPLEVIVRNIAAGSICKRVGLEEGVVFDEPIFEISYKN

DAYGDPMLNDDYAVAMKLATREELKFLREETLKINELLKAFFLKLNLKLVDFKIEFGKDS

EGNIILADEVSPDTCRLWDVNTNEKLDKDRFRKDLGDLVEGYTEVLSRMNNK*

>CD630_02200 Clostridioides_difficile_630_NC_009089 amidophosphoribosyltransferase

MCGVLGIYSNKDVTKELYYSLYSMQHRGQESCGLALLDDGEIKYKKDMGLVGDVFKENEL

SKLKGNIGIGHVRYSTAGGSHVSNCQPLVGSCRKRQLAIAHNGNLVNANYLKDMLEEDGY

MFQTNSDTEVILYILARYYKGDIVESLKVTMDYIKGAYALVIMSQEELVAVRDPHGFRPL

VLGKKGDEYIFASENCAIDILGGEVIRDVEPGEIIVVKDGELKSYFYSENYKPVKKSCIF

EHIYFARNDATIDNVNAYEFRIKCGERLAQNETVKADMVVPVPDSGWPGAIGYANASGLK

ISEGLVKNRYVGRTFIKPTQEEREIAVKIKLNPLSTIIKGKSIILVDDSIVRGTTSKQLV

KSLREAGAKEIHLRITSPPVAYSCYYGIDTPNRSKLIASSNNVEEMREYIGCDSLKFLDI

EGMLDATEHKSTFCKACFDGEYPVKKIDKEELLSC*

>CD630_02210 Clostridioides_difficile_630_NC_009089 phosphoribosylformylglycinamidine cyclo-ligase

MLTYKESGVDIDEGNRAVDLIKGKIKGTYDGNVVGDLGNFSGLYSLKDFVGMKEPVLLAS

TDGVGTKLKIAQMMDKHDTVGIDLVAMCVNDLICQGAKPLFFLDYIALGKLVPEHIEKIV

GGIADGCKMSGCALIGGETAEMPGMYGEDDYDLAGFSVGIADKEKIVSGNNVKSGDVLVG

ISSSGVHSNGFSFIRKIFLETYNYKMEQYVEELGMTVGEALLTPTKIYVKLALDVLAKHD

IKAIAHITGGGLIENITRVIPKGLGLDINKKSWEKPPIFKMIEGFNAVDERELHKSFNMG

IGLVLIVDKENADDVVNFINNRENDNADYVDKKYSELLEDKAYIIGEVVDSHEGVELC*

>CD630_02220 Clostridioides_difficile_630_NC_009089 phosphoribosylglycinamide formyltransferase

MLNIGVLISGGGTNLQAVIDGTESGEIKGQVKVVISSKQGAYGLERAKNHNIKAICETDE

DKIIEILKENKIDLVVLAGYLKIISPKLVNEFRNKMINIHPSLIPSFCGAGFYGEKVHQG

VIDYGAKVTGATVHFVDEGADTGPIIMQDVVKVNQDDDAKTLAKRVLEVEHRILKESISL

FCENKLKLQGRRVFINE*

>CD630_02230 Clostridioides_difficile_630_NC_009089 bifunctional phosphoribosylaminoimidazolecarboxamide formyltransferase/IMP cyclohydrolase

MSKRALISVTDKTGVVEFAKELNKLDYEIISTGNTFKTLKENGVNVMQVEDVTNFPEILD

GRVKTLNPYIHGGILYKRDKESHVETVNEHKIHSIDLVAVNLYDFEGTLKAGKSHDEIIE

NIDIGGPSMIRSAAKNYKDVIVVVDIKDYDSIIEKLKTDTMTLEDRKKLSYKAFSTTGRY

DALISSYFAGEVGDTYPDILNLTFQKEQTLRYGENPHQNGFLYSQSNAKNPILNYEQLGG

KELSFNNLNDLHGCLEVMREFKDSEEVVSVAIKHANSCGVGLGKDAFEAYTKCYEADKVS

IFGGIVGITSTIDKATAEKLNEIFLEIVVAYDFEPEALEILKQKKNLRILKLAKIENSLQ

PYEMKYLDGKLLIQDRNNILAEKSENVTKEKPTDAQLKDMEFGMRVVKNMKSNAIAIVKN

GQTLALGCGQTSRIWALKNALENNKDKDFTGAVLASDAFFPFDDCVTLAHEYGISAVVQP

GGSIKDKDSIEACDKYDMVMVFTGIRHFKH*

>CD630_02240 Clostridioides_difficile_630_NC_009089 phosphoribosylamine--glycine ligase

MKILVVGGGGREHAICWKLSKEKNVEKIYCAPGNAGIANVAECVNIGDTNIEELLKFAKE

NEIGLTIVGPEVPLVMGIVDEFEKEGLRVFGPNKKCAQLEGSKAFSKEFMIKHNIPTAKY

KEYTNLEEAISEIDSFGYPVVIKADGLAAGKGVVIPENREDAIATLKEMMSDKKFGAAGD

KIVIEEFLKGIETSILAFVDNDTIVPMASAKDHKKVNNYEQGPNTGGMGTFSPSEIYTEE

LANKVKETVLEKTLEGFKKDGLNFKGILFVGLMITEDGEKVLEYNVRFGDPETQSVLFRL

ETDLHEIMEAILDNKLKDIEINYSDDEAVCVMLTSGGYPDSYEKGKIITGLENLDDDIVV

FHSGTKMFDGNLVTNGGRVIGITAKSTTVKDAAEKVYENIKKINFEGMHYRTDIGR*

>CD630_02250 Clostridioides_difficile_630_NC_009089 phosphoribosylformylglycinamidine synthase

MLNTENKDSMVRRVLVEKREGFDLEAKALKKDLVESLHIDNIENLRILNRYDVEGISEEV

YENAAKTIFSEPNLDVVYYEEIPKLNDERVFAIEFLPGQYDQRGDWAAQCVQIVNQGIRP

AINTAKVYILSGKITDEEFSKIKDYCINPVDSREASLEKPETLKMETEIPTTVEVLDGFI

DLDEKGLRTFVSEKGLAMTLGDLQHVQKYFKDTEKRNPTITEIKVLDTYWSDHCRHTTFM

TEIENVKIEDGKFNDIVKEAYQMYLNSRDNVYVNRHKDICLMDIATVAVKELKKNGKLND

LDESEEINACSINVDVEVDGKMEKYLVMFKNETHNHPTEIEPFGGAATCLGGAIRDPLSG

RSYVYQAMRVTGSADPRTTLEDTLPGKLMQRKITTEAAHGYSSYGNQIGLTTGQVAEVYD

ENFVAKRMEIGAVIAAAPKENVVRERPEAGDVIVLLGGKTGRDGCGGATGSSKEHSEESI

LTCSAEVQKGDAPNERKIQRFFRNKEVAQMIKRCNDFGAGGVCVAIGEIADSLDINLDLV

PKKYDGLDGTELAISESQERMAVAIKKENKDKFIQLAVEENLEATHVATVTDTGYLRMFW

NGKAIVDINREFLDTNGVKQTTDVHVTKVDEENTFFSSNEIVKDVKCSSMKDKFTKVLSD

LNVCSQKGLVEMFDNTIGGNTVLMPFGGKYQATPTQGMVAKIPVLGGETNTSTIMTYGYN

PKVGKWSPFHGALYAVVESVCKLVAIGGNYSTTRLTFQEYFEKLGNNPEKWGKPFSALLG

AFYAQSKFEIPAIGGKDSMSGTFKDIEVPPTLVSFAVDTVDAKKVVSPEFKKADSKVVML

CVNKAENDVVDFEELKRNLDKVRELIHGNKVLSTYALGFAGVGEAISKMAFGNKIGFKFS

EEAEKAFTDDKLFEASYGNIVLELANDDLSMLEGYNYVVLGSTVKEASIFIKGEELALDE

LYKAHCSTLEPIFPTKTEEVKSKIETISYISQGEAKKSSLSIATPRVFIPAFPGTNCEYD

SARAFERAGANASIRVFKNLTYKDIEDSIDTIVNEIKSSQIIMLPGGFSAGDEPDGSGKF

IATVFRNPRVQEAINEFLTQKDGLMLGICNGFQVLIKLGLVPYGEIRVPSESAPTLTYNN

IGRHQAKIARTRISSNKSPWLAQTNVGDIHNIAISHGEGKFVASEDVMRELIANGQVATQ

YVDFNNEATYDIEFNPNGSFYAVEGITSADGRVFGKMGHSERIGEEVYKNIIGEKEQKIF

ESGVKYFR*

>CD630_02260 Clostridioides_difficile_630_NC_009089 lytic transglycosylase

LNEISSLINILALTQLSGSNTTNQCNCGYNNSSGFDMIMMTLLKALSQNNQQTSNGYNLS

SNSEKLFNELDSVANNTVGKFIEVDTKDKSVKSRIENAVEQASKKYNVDANLIKAIIKVE

SDFNPNTVSSAGAKGLMQLMPENCRDLGVTNPFNIEQNIDAGTRHIKEYIDMFGGSIEMG

LMAYNGGPGRMRSRGVESISDLYKMPKETQNYIPKVMKYYRG*

>CD630_02270 Clostridioides_difficile_630_NC_009089 hypothetical protein

MGINIDKLNISPSRSIEVKKTKENNKFSDSLNFVKQSSSLEDIKRNLDGVKKAGERLVLT

QNYGDISRYKNAVKEYLKSVVDNMYSLDKSSSFWEHQYYKNVKIIDNKLEDMTSKLLNEE

KENIDIVSTVDMIQGLLIDMYR*

>CD630_02280 Clostridioides_difficile_630_NC_009089 flagellar motor switch protein FliN

MNSKKEDIYELNFGQLIEEKTGIIEENQNVFNAKVNVAVSIGNITETIKNILDLKEGSII

KLNKNVDEKLDIYANDRLFAYGESIITNETLSVRLSKIEDSDVN*

>CD630_02300 Clostridioides_difficile_630_NC_009089 flagellar biosynthesis protein

MITDLKVIIYEEKKIISNMMELLKEQFDYIIEKDLENLNRLNPKLEDISRELASIEIKRR

QLLGDDVSISEVVENSNDEYLKEIYIDLKHILALAKNQQESNDSLIKKELIFTKKMINFI

KPVDKQTTTYNSYGNIRK*

>CD630_02320 Clostridioides_difficile_630_NC_009089 flagellar hook-associated protein FlgL

MRVSTGMMSSSYLNSLQDNLQRLDKVNRQINTTKEINKLSDNPYKAIKILNSKSEIKTME

TYIENCKDTADWLETTDTSLDQLGNLLADIKKGLVSSGNGSYSDDEIKTISNSTNEKMKE

IANALNATHEGKYIFSGSNTGTPPVECVENADGSVSLKFNTSLNLNKLNDSLSVDVAQGI

SVDYNVKLSSLGFDPTKTDPFEKLNNISKKLANPSDANIKELTTTCLGDMENLIDNTVNV

RSIYGTKANTVDAMKEKNDEGLIQLKDVLSQNDEIDYGEKLVQLKAAELTYQASLQTGGK

LFNVSILDYI*

>CD630_02330 Clostridioides_difficile_630_NC_009089 flagellar assembly factor FliW

MMKITLKKGILGFENLKEYELLDIENEDILKEFNSTEEDCIGFIVVSPFEIIKEYEIVLN

QETIEKLEVKSPNDIMLLNIITVGQTLEESTVNMKAPIVINVRNNCGMQIILQDEEYSIW

HPLLRGDGGC*

>CD630_02340 Clostridioides_difficile_630_NC_009089 carbon storage regulator CsrA

MLVISRKKDEAVLIGDNIEVKVVGVDGNNIKLAISAPNNISILRKEIYEKVKNENIKATN

KNIKILKSLK*

>CD630_02350 Clostridioides_difficile_630_NC_009089 flagellar protein FliS1

MYGENPYNSYKQNAIFMASKEQLLLMLVDGAVKYTKIARGAIIDKNTRKAHRELIRVQDI

FTELMVTLDQNAGQWAKDMYRVYDFVRYELSRANIRKDVQIIDNVLPVIEEIKDTWHEAD

RKSREERSRYK*

>UAB_RS0202440 Clostridioides_difficile_ATCC_43255_NZ_CM000604 flagellar protein FliS

IMELNLAELDKLSKEELLLMLVDGTVKYTNISKEALLNNDYLKAHNELVRVQNIFTELMT

TLDQDAGQWAKDMYKVYEFIKSELAIADENKDIKIIDDILPIVKQIRDTWHEVYNQLKI*

>CDIF1296T_00448 Clostridioides_difficile_ATCC_9689__DSM_1296_strain_DSM1296_CP011968 flagellin subunit

LSDYINEELIKKIKSRNDKDVNYTKEGKIMRVNTNVSALIANNQMGRNVNAQSKSMEKLS

SGVRIKRAADDAAGLAISEKMRAQIKGLDQAGRNVQDGISVVQTAEGALEETGNILQRMR

TLSVQSSNETNTAEERQKIADELLQLKDEVERISSSIEFNGKKLLDGSSTEIRLQVGANF

GTNVAGTSNNNNEIKVALVNTSSIMSKAGITSSTIASLNADGTSGTNAAKQMVSSLDVAL

KELNTSRAKLGAQQNRLESTQNNLNNTIENVTAAESRIRDTDVASEMVNLSKMNILVQAS

QSMLAQANQQPQGVLQLLG*

>CD630_02730 Clostridioides_difficile_630_NC_009089 heat shock protein 90

MEFEKGSISIHTENIFPIIKKWLYSDKDIFIRELISNGCDAVSKHKRLVSLGEISENKSS

DYKITVSVNKGEGTLKFIDNGIGMTEEEIKKYINQVAFSGAEDFFNKYKDKMEESNDIIG

HFGLGFYSAFMVSKKVQIDTLSYTEGATPVRWISEGGTEYEISESDARNDRGTTITLFID

DDSKEFLDEFTVRGIINKYCSFLPVEIYLEDVERLEREAKEAEEKAKKQKEDGKEEIVDA

KVIEPLNDTNPLWLKSPKDCTDEEYKEFYRKVFNVFDEPLFWIHLNVDYPFNLKGILYFP

KLKNEFELTEGKVKLYNNQVFVADNIKEVIPEFLLLLKGVIDCPDLPLNVSRSFLQNDRD

VSKISKHIIKKVADKLKSLCKNEREEYNKFWDDIQIFIKYGCLKDESFYEKVKECILFKT

IDDEYITLQDYLEKCKDKHENKVFYVSDKEQQSQYIKLFKEYDLSAVVLNSSIDTHFISF

MEYKENGVKFNRIDADLSDVLKDKNENKDSEENKEEIAKIEGLFKEAVGERVKNYSVEGL

KNEDTPAMVLVSEQSIRMAEMQSRFAGMDLGMNFEEEKTLVINENSPIIKKLVSLKDDEE

KKDKITLICNQIADLALLSNKELKPDELDSFVQRSNKLMSLLIEL*

>CD630_02740 Clostridioides_difficile_630_NC_009089 iron-containing alcohol dehydrogenase

MNFNYNLPVNILFGRGRINDVGKEVSKYGKKVLIVTGKNSTKKTGLLDKTIDLLKDSKIE

YEVFDRVEQNPLITTIYSGVEIIKSTGCDCVLGLGGGSIMDAAKSIAFSYKNPGDLNDYI

FGIRQGEEALPIILVPTTSGTGSEGNCFAVLTNSETKDKKSIKKNSMYAKASIIDPELMV

TMPKHIIASVGFDALAHNMEAYLSNGRNPLADVQAIYGIELISENLIKVYNDVNDLEAWE

KITLASTIGGMTIGTAGTALPHGMEHPVSGLRDVMHGKGLAALTPTIIESSWDSDIERFG

NISKVLGGTCAEDCSDAIRNFLKKIDLDIKLSDLGIEKSDIDWLTDNCMKISVANIKRHP

KYFNKEQIKEMYHKSL*

>CD630_02760 Clostridioides_difficile_630_NC_009089 AraC family transcriptional regulator

MHAWESIQITLDLIEANLSEEISINELANKANLSPFYYQRLFKRLVNKTVMEYIKLRRLA

RASEYLAEHKNRILDVALNFGFASHETFTRSFKKAYGLTPEKYRANPVKLNHFIKPELIL

NYAMVDEGVPLIADDIVIEVNRKTLSNPRTFVGIEIEVPICQLVGGETTGIAIVEELWMK

LGSQRHNIPNQIPGGNEFAALYMGNAKEGNCMYMAGVEVESGTSVEGYSTFELPVKEYLV

CGFEAENFNELVNSAVFKADKFMERWMKKHNLTTTDFAIEMYYPTTPEAAYLEHWIVPVP

IEQ*

>CD630_02770 Clostridioides_difficile_630_NC_009089 hypothetical protein

MNWQDAFFQDTKPTFEQINEFINNPLWDKLNKTLVSTYNVEPKLEYSKCSMQRGWNVKYK

KRGKSLCTLYPQEGYFKALVIVSESNRVEVDLFINTCCDYIKKIYNEVNFFNGSKWLMIQ

VDSLLVLNDMLELIKFRA*

>CD630_02780 Clostridioides_difficile_630_NC_009089 HxlR family transcriptional regulator

MSINTEKNITKNVEELTCPIRYALDIVGGKWKLPIICMLAVENPIRYSSIKRKLDGITNT

MLAQSLKDLESTGIVHRKQYNEIPPKVEYTLTSKGKSIVPILQQFANWGATNMQEKNTCG

LSCKECRKIK*

>CD630_02790 Clostridioides_difficile_630_NC_009089 hypothetical protein

MKALEFLNKAGVYYLATVDENNQAHVRPLGFVMEYNGKLTFCTSNQKDMFKQLIFNPKVE

LCCIDQNLNTLRILGEAVFVTSKETQAKALEIMPSLGQMYSVGDGKFEIFSIDKAKVSCC

SMSGKKIDAEL*

>CD630_02791 Clostridioides_difficile_630_NC_009089 hypothetical protein

MSKYSSLWEYVKKNNSQSFKLTFEEIKDIAGIEIDHSFLKYKKELNEYGYQVGKISLKEK

TVIFDKIG*

>CD630_02820 Clostridioides_difficile_630_NC_009089 hydrolase

MNFLSDLHTHSIVSGHGYSTLLENINYCKENGIKILGTSEHGPKMPGSPHRWYFHNIQNI

PRIINNIIILRGCEANILDIKGNIDLEPFVIPRLDYLILSFHEAVFSPNTLENNTKALIN

AINKHDNIEILGHLGNPNYPIDYELIIKLAIEKNILIEINNCSIKGVSRNGSSDNCKYIA

TLCKKYGAKIILTSDAHICFDIGNYEYSENILKEINFPDELIMNYPKKLINHFHQKDKLL

DVDYTNI*

>CD630_02840 Clostridioides_difficile_630_NC_009089 PTS system mannose/fructose/sorbose transporter subunit IIA

VIGIIVMTHGSFSEEIIKSCELIAGPAERTAAIKLNRNDNIEDLNKNFVEKLNELDEGDG

VLVLADLLGGSPSNVASLNLKKGGKFHALTGVNLPMLLEALINREGKSLEELAEACIEAG

QTGINNINKVLSSM*

>CD630_02870 Clostridioides_difficile_630_NC_009089 PTS system mannose/fructose/sorbose transporter subunit IIB

MAKITLVRVDHRLIHGQVITKWVKIAQAQKIIIVDDFLGQDEFMADIYKMAAPSGVEVVI

LTAEDAGQAFQNNTLGDKNIFILFKNVDMANKAYKAGLKYEKIQLGGIPNEAGKKMVFTA

VSLGNEDVEQLNELNENGVEIVLQVIPEESSMTYENALKKFK*

>CD630_02880 Clostridioides_difficile_630_NC_009089 PTS system mannose/fructose/sorbose transporter subunit IIC

MESVLMLAIVTGLWYWFAAGLAGYTLFSTLKSPLFIGFSLGLLWGDVTTGMIVGASIEMV

YLGMVAAGGNIPSDKCLAALIAIPVALQTGVNAEVAVSIAVPLGVIGVLVNNLRRTGNAV

LVHKADKYAEEGNTKGIWRCATLYSLIFGFVLRFPIVFVCNFFGADLVQSLLDVIPQWLM

NGLTVMGGILPALGFATTIFTIGKNKFLPMFIIGFFMVQYFEISITAAAIFGVCIALLIT

FMKEDKRVGEV*

>CD630_02890 Clostridioides_difficile_630_NC_009089 PTS system mannose/fructose/sorbose transporter subunit IID

MQEIKNKEVKNGQGPTEVNKTRVLTKKDVTKTYLRWWWTAELSNSFERMQALAVCASFTP

ALEKLYKRKEDLVDALKRHLQFFNTQAIWGGLIHGTVLAMEEEKATEGKIPGEVISGVKN

GLMGPLAGIGDTLDFGTFQTIFLALGASFGAEGSVIGAFFPIMFSILLFCEGYYLFHLGY

SLGRDSIKKILSGGIVNKIIDGASILGMFMMGALSATTVKLSTPLSFDIGGKAIVVQDTL

NMIAPGLLPLGVVFFVYWGMKYKKWTITKLLVILVVLALVGSFIGIF*

>CD630_02900 Clostridioides_difficile_630_NC_009089 hypothetical protein

MSEKMYTEKEYLEGIRGATGERAVWFYLLMKEAEKLGANPDDICKEAIYGFGKMRGQKYN

VADTPGKMAEMLYNSKGQKVFEMELVENTDENGVLKFHHCPLDAAWKEYGLTKEERKEIC

RLACYGDYGRVDCAQGVKLDFAQKCAHDDEVCELVFTKK*

>CD630_02910 Clostridioides_difficile_630_NC_009089 peptidase

MDISRSVDNSLNRTIEFLKELIKIDSQQGEPISQCPFGIGPKKSLDKTLDYCASLGFSVK

NIDNYIGYAEIGEGEELIGIPMHLDIVPPGEGWSVDPFSGAVIDNIIYGRGVIDNKGAVS

MLIHVLKNIEDMYPTINKRIRLIFGTNEETGMKCIKYYLDKGEEIPSMGFTPDAMYPVVN

GEKGRVHIRIEKEIKIDKSKPYIIVSGGTKENVVPSHCTAKIINGIISELTTKGVAVHAS

NPEKGENAISKMVIKIVEDNMDFQHREDIELVSKYLCSDYYGDALGINQYDEVFKNTTLN

LGILKVNEEKIVCELDIRYGKNIVLNNIIDRFKKVFCNGWKIEVIAHKDLHYVDESNLVL

KKLLEAYEEVTDENGYTIAMGGGTYASWFKDMVAFGPKFLAYKTGGHGVDERVPINHIRK

NMEIYTLALIKLLEL*

>CD630_02921 Clostridioides_difficile_630_NC_009089 hypothetical protein

MENLMEYLPLLVPVIILDLILIITALVHVLRHPNYKIGNKAIWIIVVLFISLIGPILYFT

IGRGEE*

>CDIF1296T_00517 Clostridioides_difficile_ATCC_9689__DSM_1296_strain_DSM1296_CP011968 ABC transporter ATP-binding protein

MMNIVSINGLSKGFGNRKIIDNLNFTVPEGSVFGFVGKNGAGKTTTMKMVLGLLKPDSGT

IDVCGEKVTYGKTSSNRHVGYLPDVPEFYNYMRPLEYLSLCGEITGLSKKEIQIRSEELL

SLVGLRNEKRRIGGFSRGMKQRLGIAQALLSRPKLLICDEPTSALDPVGRKEILDIMLKI

KDSTTVIFSTHILSDVERICDHVAILNKGSIALSGTLSEIKSMHGKDRLLLEFASNDEIQ

KFKSSDGIKSLLKDSEETNMEIVLHGKDIKAIQKTVISTLAEMNLCPVKMELIELSLENL

FLEVVK*

>CD630_02940 Clostridioides_difficile_630_NC_009089 bacitracin/multidrug family ABC transporter permease

VREYIAFTKKEFKENLRNYKLFSLIILFLIFGISSPISAKFMPDLIAHFAPTLKVTAAPT

ALDSWTQFFSNISGLGMSLTLIIFCNILSNEYSKGTLVIMLTKGLSRSSIVLSKFSVTVI

IMTIGFWLSFLFAYGYTMYFWPTANLNHIIFSAFNLWLIGIMYISVLILGCVLFRPAFAS

VLLVLVATLLLSLISIPKQIAPYTPNFLMSKNIDLISGKVAMPEFIIPIIITVLISIVCL

LAAVILFNKKPV*

>CD630_02950 Clostridioides_difficile_630_NC_009089 iron-sulfur-binding protein

MEKSKVYFCDLYSNSQNKNVPNNVRRLFDEAGFKDLIEKNDQVAIKLHFGEKGNTTYMSP

VAVRQVVDKVKDCEGKPFLTDTNTLYTGSRTNSVDHLTTAIENGFAYAVVNAPVIIADGL

YSRNYENVKIDKKHFESVKIGGEIYNSSAMIVMSHFKGHEAAGFGGALKNLAMGCASAAG

KQMQHSDVTPVVKEKKCVGCGKCVNSCPTKAISIVDKKAVIDSDVCYGCGECPTVCPTRA

VTIQWESDSDVFVEKMAEYAYGAVSNKKDKVGYITFVMNVTPLCDCVPWSGRPIAHDIGI

LASTDPVAIEQACYDLICKEMGHDVFKHEHPHVNGTRIIDYACEMGMGSKEYELIKL*

>CD630_02960 Clostridioides_difficile_630_NC_009089 hypothetical protein

MKIYYEKENSKKANVNNIPSVFAFNFLRNFLTSKEYEETRKEYFINNLTKSQVNQAMKDL

KWLFREYEGLEVITMESLDGVKTRIVL*

>CDM68_RS01670 Clostridioides_difficile_M68_NC_017175 biotin synthase BioB

MEKYTIKLKNKVLRGKEISYEEALNLISLDTNNKNDFDTLLKSANEIREYFMGRKADLCT

IMNAKSGKCSEDCKFCAQSSHYKTGVEEYSLLDYNEILNRAKEMESKGVHRFSLVTSGKG

MSGKEFNDILNIYEGLRKNTNLKLCASLGIIDYEKAKMLKSAGVTTYHHNVETCRDNFHN

ICTTHTYKDRIKTIKDAKKAGLDVCVGGIIGMNESEEQRLKMAFEIRELNVKSFPINILN

PIKNTPMENYDVLEPMEILKTTAVFRFIIPNVYIRYAGGRLSLKGYDKVGFNGGVNSAIV

GDYLTTVGSGIENDKKMIIEQGFELY*

>CD630_02980 Clostridioides_difficile_630_NC_009089 LacI family transcriptional regulator

LSITIKEIGELAGVSKTTVSKVINNKDENISQATREKILKIMKEKNYVPNKLAQSLVTKK

TNTIGLLIPDIRNPFFTDVSRGVEDKANEEGYNIILCNTDEDAKKEYEGIRTLSERMIDG

IIFAASSNTNWKEANYKDIKIPTVLIDKKISMNKETLKGIVKINNFEGAYIATKHLLDIG

NKKIIYLSGPLQNEIAVDRLEGYKKALIECNLSYNPAYVFEGKYKIEWGQEFIKNLEKID

FDAIFCANDLIAIGVIRGLKERGLSIPNDISVVGFDDIQTSSLISPSLTTVKQPSYDIGY

KASEILINCLRGDKKESFDELIFKPELVIRDSTKESE*

>CD630_03010 Clostridioides_difficile_630_NC_009089 ribose ABC transporter ATP-binding protein

MSNIILKLSNIAKEFPGVRALDNVNFELFHGEVHALLGENGAGKSTMIKILTGAHSKTSG

KFIFEGKEIEHISPDISKKIGINAIYQELTVFDELTVAQNIFMGKEINGKVLTNDKKMNE

EAKKIFDNMGIDINPNSLVKELSIAQKQMVEIARVLSSETKVLIMDEPTSSISKKETEIL

FRLINDLKESGVSIIYISHRMEELFEICDRITIMRDGKTISTLNTKDVSSEEELVNLMID

RKLDQFFPKRKVEIKEEIMRVENLTKNNVFNDISFNIRKGEILGIGGLVGSKRSEIVEAI

LGLRTYDSGKIYLNNEEVKFKTPSDAIENGLGLITEDRKGTGLFLQMSVKENTTMAGLKK

ISKFKSIIDRKKEKEILEKYIEALKIKTPHMNQVIQSLSGGNQQKAIIARWLLLQPDILI

MDEPTRGIDVNAKAEIYNLMGDLVESGVSIIMISSEIPELISMSDRIMVMREGHISGFLE

GEEMVENNVLKLAFGGKINEFNN*

>CD630_03020 Clostridioides_difficile_630_NC_009089 ribose ABC transporter permease

MSSITKHKEEKSSPLLTKLTSVFRDQGSAAVGLVIIFIIMSVASSNFLTLDNLINVGRQI

SINAILAVGMTFVIITGGIDLSVGAVIALVGTFWATTVVNYNAPIWVGMILALTIGTILG

VIKGAIISTQKLPPFIVTLAMLTIISGASFVFTGGRPISVNTDAFKMLGRGYIGPIPIPV

IIMIIVVIAGHFLLKRTNFGRPVHAVGGNEEAARLCGVKVNKVIVKVYALAGLLTALAGI

ILSSRLASGSPTVGDGAELDAIAAVVLGGTNMMGGSGSIVGTCIGVGIIGILGNGLNLLS

VSSYNQMIIKGLVMLFAIWINNIKLKKSAKSK*

>CD630_03030 Clostridioides_difficile_630_NC_009089 acetylornithine deacetylase ArgE

MKKILLDTLNSKKQEYIDYLKELVSIKTEDVGHGILGGLEKEGQEYIEKLANYIGFSVDR

QEMSEELIKKAKNIYKEGNLGHNYQDRYNLICKYSDDLPGKTIVFNGHVDTMPPGDISKW

KYNPYRATEDNGKLYGLGTADMKSGLIASILAVKLIKDSGLNVPGNVKIMSVVDEEGGGN

GTINAVMNGIDGDCCIICEPSEQNLIVAHMGFVFFEVEVKGVSLHCGSKWEGVNAIEKAM

LLLQDIKELEHNWLMIYKHPLLPSPTINLGVINGGTAGSTVPDKCVFNLCVHFLPNIMSY

EQVVNDVTNVIMTRANGDLWLKDNKPNINIYQSGLGFEMDKDSDFVVNAHKILEETLGKK

LEIKGSTAGNDARVMKNLAEIPTLILGPGSIEQCHSIDEYVEIKEYLDSILMYASLILNL

*

>CD630_03040 Clostridioides_difficile_630_NC_009089 hypothetical protein

MKKVLIVGESWVKNITHIKGFDTFVTTHYEEAVKWLKEAIESGGYETVHMPAHVAADSFP

YKLEELNEYDCIILSDIGSNTFLLSNSTFIDCNSNPDRLELIKEYVNNGGALIMVGGYMS

FTGIDAKARFGETAIKDVLPITMIDKDDRVEKPAGIIPEVIDSEHPVLKGIPTEWPKFLG

YNKTVARDNCPVLATIGGDPFVAVGEFGKGKSAIFSSDCAPHWGPIEFTDWKYYNKLWVN

MLDWLTC*

>CDIF1296T_00532 Clostridioides_difficile_ATCC_9689__DSM_1296_strain_DSM1296_CP011968 hypothetical protein

MHYKINELENKYNLYNKVVQKGVFMFGYVKINKMDLTFREYDYYKAYYCGLCKYLKRNHG

EISRFSLNYDITFLIVLLTAVYNPESISTEEVCIVNPFKKKKVITNDITEYAASMNILLT

YYKLEDNLMDDKRIKDKLAYYIYKNKLKLAYEKYPEKAEYIKQQLNELNKLEKDKNINID

EVSSIFGNIMGEVFVYKKDENERNLRMIGFNIGKYIYLLDAYEDLDEDFKKGRYNPFIEY

IDKNDELKEKVKKIIVTSLGFLARGIDNLNLNKNVGIIENIIYSGVYLRYINILESRGGK

NVQ*

>CD630_03070 Clostridioides_difficile_630_NC_009089 chaperone protein

MYSNTYNNENFMRARAFIENSEFKKAYDFLKTLTDKCAEWYYLTGFSAMNIGYYEEGEDF

LKRAKFMEPENSEYSDALRSYTQYRNDYSNRADNYNRRRRNDLDGCCCCCCDDCCCCLGD

DCCENCAKLWCLDSCCECFGGDLITCC*

>CD630_03080 Clostridioides_difficile_630_NC_009089 membrane protein

MINMSIKVAYGGVLLALNVILLTLTNIIPVNTLFIMGLASLLVSIVIMEWGFKSGIAFYI

GSIVLGFIVMASKSQWIVYSLTFGIYGIVKYLIEKDRSIYIEYFMKLVFANIMILILYFL

LRTIVYIPINIFIIGSFEIAFIVYDYVYSSFIGYYNNRLRKMLFKK*

>CD630_03100 Clostridioides_difficile_630_NC_009089 hypothetical protein

MNFNFAKATNSRLMGSLGLIINWIDDENNHFCQYFLLDAEGLGLADYVSLNNPTQEEAYM

EEERLMGGFGSDRVELTKDESLFLVSYFGNKNLYYDKLLPGDKCEYIDIIKNYKTDLTIE

KLYNKICKRVDEEVEFINYMTMRFIAWDRESLKYFSGSDEIANMHITNINGTLLKNVVSD

KGQGRYISEALYEDNDGYYICKIAFCISKCYETGFRINSLLVTDKEPMYDFEVFDEISKS

EFVSVYSVNSPEEFAGVFYRENPFLLKSNMNEGIFFTRFNFNNDHVKENVYIINNDMKAI

YYLMGNKFFIGTYNENDCNYINEISQSNYSDYIKFEEKFFFEQNALYDFAESGSLDFDDF

LE*

>CDM120_RS01965 Clostridioides_difficile_M120_NC_017174 hypothetical protein

MSNKKKKDLDTSYMPYLDPANYADYITNEEIPQGELRNDPQISNGYTKVPNNQNINNLYP

NNINNYPKSSNSPNNLPNTNNTSGNMNYNTSATNNMPNNMNSNMGTPNNMSNNMNSNMGI

PNNMPNNMNSNMGIPNNMPNNMNSNMGIPNNMPNNMNSNMGIPNNMPNNMNSNMGIPNNM

PNNMNSNMGIPNNMYGNMNNNMYGNTGMPNNMNSNMGYNMNNTMGIPNFSCNQNMPPNVL

MMIPGVICHNTMQGMPVVMPSTMPPNIYPTPYGSSNMSMQGIPQATNIEEFDEEEM*

>CDIF1296T_00538 Clostridioides_difficile_ATCC_9689__DSM_1296_strain_DSM1296_CP011968 ArsR family transcriptional regulator

VHSNYIKLIKIIVANLEMIAYNEHMNSYSYIKIIGGLKMREEINDCNCNIVHEEIVTEAK

STMPDEEMLYDLAELFKVFGDTTRVKILYALFANEMCVCDIASLLNMTHSAISHQLRVLK

QARLVKFRREGKTVYYSLDDSHISQIFDCGLNHIRETYK*

>CD630_03130 Clostridioides_difficile_630_NC_009089 K/Mg/Cd/Cu/Zn/Na/Ca/Na/H-transporting P-type ATPase

MEANNSIKKEFILGGLNCAHCAEEINNKVSKLQEVKSSNLNFINKKLTVNIKESFNEDTT

IEKIIDIIDSTEPGLDIQISSKENAASKTSIKKELILGGLNCAHCAEEINNKVSKLKEVE

SSNLNFVNKKLTVNISNNFEEDDVINKIKEIINSTEPGLDIQVGSTDKVKGRTTEKSGAV

NDTNKKELIPLIIGALVYIFGIYQTATGYESQFSNIVFIVAYVIVGGDVLLRAIRNISKG

RVFDENFLMALATVGALAIGELSEAVGVMLFYKVGEYLQGVAVGKSRKSITSLMQIRPDY

ANLKVNSEVKVVSPEEVNVGDIIVVKPGEKVPLDGVVVDGVSMLDTSALTGESVLREVEK

GDEILSGVINKNALLSIEVTKSFGESTVSKILDLVENSSIKKSKTENFISKFSRYYTPIV

VIAALLIAFVPPLVISGEVFSDWLYRGLIFLVVSCPCALVLSIPLSFFSGIGFASKNGIL

IKGSNYLEALRSVDTVVFDKTGTLTKGVFNVTKLNPEGISDEELLEYAAIAEVNSNHPIA

KSILSYYNKKIDLDTIDSYEEIAAYGIRVKHNGNFILAGNEKLMKKENISYSSAKEVGTV

VYIAVDKVYRGYIVISDEVKEDSKNAIRSLKEIGVKEVVMLTGDNEKVAKNIAQELELDT

VYSNLLPNEKVDRLEDLYEGRTEKEKIAFVGDGINDAPVLARADVGIAMGGLGSDAAIEA

ADVVLMTDEPSKISKAIEIANKTNKIVWQNIIFALGVKIIVMILGAGGVATMWEAIFADV

GVALIAVVNAMRAMR*

>CD630_03140 Clostridioides_difficile_630_NC_009089 membrane protein

VSVINSFILQYGLISVFVLIMIEYACFPLPSEVVLPLCGAIAARNHFGFLTILILSIIAG

ILGSIFCYTVGNWGGKSIINKIIEICPKAKKGIFASQDYFNKYSSISVCVCRLIPLCRTY

ISFIAGIAGQNIITFVISSIVGITIWNTSLISIGYIFSESWVRIMSHYNDYKFLVLIIPI

SIIFIGFVIKSFIHKKHHKIRL*

>CDIF1296T_00550 Clostridioides_difficile_ATCC_9689__DSM_1296_strain_DSM1296_CP011968 norq protein

MDIIDNLRVQGVDEKLIEDVLYFRNYYGLEKDLEYRVTKSKTYFYGKDILSMCIAAILEE

ENILLSGPKATGKNLLADNLGEIFNRPQWNTSFHINTDSSTLIGTDTFIDNEVKLRRGSV

YECAINGGFGVFDEINMAKNDAIVVVHSALDYRRIIDVPGYERVNLHPATRFIGTMNYEY

AGTKELNEALVSRFMVIDIPPIEEDKLMMILKNEFSDADEEKLIHFAGIFLDLQLKSQNG

EISSKAIDLRGLMASLKTIRRGLKPTLAINMGLTGKTFDVYEKEMVGDVIKTRIPNKWES

IDVFPISHI*

>CD630_03250 Clostridioides_difficile_630_NC_009089 cobalt ABC transporter substrate-binding protein CbiN

MSAKTKTKTNILLLLLVVALIIFPLLVNSGAEYGGADGQAESEITKINPNYKPWFSSPYE

PPSGEIESLLFSAQAALGAGVIGYILGLQKGKRS*

>CD630_03260 Clostridioides_difficile_630_NC_009089 cobalt ABC transporter permease CbiQ

MLIIDKYAYTNRLSKVNPNKKVAIGVIFLIASMVIQNIFILSGIMILMSILVVCVAGIDL

KNYLKLLRIPMYFLFLSIGITLVNISFNKADLLYSFEFFSFNVGISKASIDMSIHVLFRA

MSCLTCVYFCILTTPFNQLIFFFKKLHLPDTFVELSMLIYRFIFIFLEEFSEIYKSQELR

FGYINLKTSYKSLGILGSMLYKRLMTRYDDMCISLDIKLYDGKFHIVGDNDV*

>CD630_03270 Clostridioides_difficile_630_NC_009089 cobalt ABC transporter ATP-binding protein CbiO

MMFKINNLTYQYEKNTNALLNINMDFSKGNVIGIIGSNGSGKSTLFMNLMGILKPTSGEI

LFKEEKLKYDKRSLYNLRKNVGIVFQDPEKQIFYSKVYDDIAFAMRNIGMDEKTIKERIN

KALVAVNGIDFIDRPVHFLSYGQKKRVAIASVIAMENEIVLLDEPTAGLDPVSTRSIVDI

IKGLNKNNIKIVISSHDMNLMYEICDYIYVLDKGILIDEGKAENVFINENNIIQAGLESP

WLVKVHRNMNLPLFKKEEDLYKYWKERELNTNK*

>CD630_03300 Clostridioides_difficile_630_NC_009089 hypothetical protein

MKLGLCLEGGGAKGAYQAGVVKALYDGGINKFYSISGTSIGAINGYYLYTGNVDNLEKMW

TNIKDIQNGNVKIVNNTVDNSPAIDNLRELDDSNIEEMNFYVNYVEVDNKVVSEKIVDVS

KMPRNEAIISISYSGLLPSNPNATLGFKEQFVKDVQEGIYDGFKLDGGLIRSALIEPLIG

DNVDKIILISTKYNYELPEDIKKVYDEDKIIVVRPNTQFAPKDTLNFDDEFCKTIYQEGY

EIGKNILDRL*

>CD630_03310 Clostridioides_difficile_630_NC_009089 peptidyl-prolyl cis-trans isomerase B

MENKNPIVTIEMENGKEIKIELYPNIAPNTVKNFVSLVNEGYYNGIIFHRVIPGFMIQGG

CPNGTGMGGPGHSIKGEFSGNGFTNNLKHERGVISMARTMAPNSAGSQFFIMHKNSPHLD

GQYAGFGRVIEGMDTVDEIASVRTDSADKPQTPQVMKSVTVETFGIDYSDVEKN*

>CD630_03340 Clostridioides_difficile_630_NC_009089 bifunctional acetaldehyde-CoA/alcohol dehydrogenase

MEKKEKVVEKSNVEVCSPEIVNSVETLRMRLEEIRLAQKEFATFTQEQVDKIFLAASTAA

NQQRIPLAKMAVEETGMGIVEDKVIKNHFASEYIYNAYKDTKTCGVIEKDEAFGFTRIAE

PVGVLSAVIPTTNPTSTAIFKSLIALKTRNGIIFSPHPRAKNCTIEAARVVHDAAVKAGA

PKGLIGWVDVPSIELTNVVMAEADLILATGGPGMVKSAYSSGKPAVGVGPGNVPAIIDES

ADIKMAVSSILVSKSFDNGMICASEQAVIVPEKIYEEVKKEFKYRGAHFLNKEETEKVGK

VVIIDGSLNARIVGQPAHVIAKMADVEVPKTARIIIGEVESVELNEPFAHEKLSPVLAMY

KSKSFEDAVAKAEKLVADGGYGHTSSLYADSINHPERVEKFVNAMKTCRVLVNTPSSQGG

IGDLYNFKLAPSLTLGCGSWGGNSVSENVGVKHLLNIKTVAERRENMLWFRAPEKVYFKK

GCLGVAAREFKDVMDKKKAFIVTDSFLYNNGYTKKLTDLLDEMGIKHTTFFDVAPDPTLA

CAREGAKAMADFQPDLIIAVGGGSAMDAGKIMWVMYEHPEVDFQDLAMRFMDIRKRVYVF

PKMGEKAYFAAIPTSAGTGSEVTPFAVITDQDSGVKYPLADYELMPNMAIIDADMMMEMP

PRLTAASGVDALTHALEAYVSMLRTEPADGLALQAGKIIFEYLPRAYKNGKNDKEAREKM

AMASTMAGMSFANAFLGICHSLAHKLGAFHHVQHGVANALLINEVIKFNCAEAPNKMGAF

SQYRYPDCIQRYAEFASFAGIKGSTDQEKVDNLIKAIDELKAKVGLPKTIKEAGVEESKF

LERLDAMVEQAFDDQCTGANPRYPLMSELKEIYLKVYYGK*

>PCZ31_RS02075 Peptoclostridium_difficile_strain_Z31_NZ_CP013196 hypothetical protein

MLLILGNLNLNLDFDYRIIREENDDVDIFIDINYRSLDIDTDGSNLFNSRIQFPFVRALI

LRLNKNNQCMTIHLLRDIDLFSAFANFEVDYTDSIINIKNQNEKVILNKSIKNSKGALIG

PNFFFSKLIIS*

>CDIF1296T_00565 Clostridioides_difficile_ATCC_9689__DSM_1296_strain_DSM1296_CP011968 ABC transporter ATP-binding protein

MPILETINLGKIYGKKETSVHALKNANLKINKGEFVAIIGPSGSGKSTFLHLVGGLERPS

NGTIKVAGKDICCLSDKELARYRRQKVGFVFQQYNLIPVLNVKENIELPLKLDNKKIDKE

YIEDLINLLGLKERKNHLPNQLSGGQQQRVAIARALSAKPSIILADEPTGNLDSKTTEEV

MDLLKSSIKKYNQTLIIITHNENIARKADRIISIIDGELKLTL*

>CD630_03410 Clostridioides_difficile_630_NC_009089 hypothetical protein

LRGDFMQIYFRVKAVGKRKPMLELTPFDVSDNINCLKEAISEIVSKNVLEYNEKIPEKSI

INFLTNEEIESQADIGKVGFGSIYNENKQSVEKAIEVALQAFEDGIYKVLINEDVIEKLD

SSINLKSGDIFTFIRLTLLAGRMF*

>CD630_03420 Clostridioides_difficile_630_NC_009089 hypothetical protein

MNRYTKIINMMGSYYTKDFEKEKKNVIKVREVKEDTVRKFFLQGDCEVLVVFEDTGKEIL

IDDFSPEEDIKKYLGAKFINKKR*

>CD630_03430 Clostridioides_difficile_630_NC_009089 hypothetical protein

MTSIKNKDILNCIDYSIKNKLFDKLNDVYKSLPVGNCSGCGNCCMESVGINLIEFLNIFC

YLEDRVDLKKSCISKILDYYFEEYTRKNPCPFKDTNNRCLIYEVRPLNCRMFGHWKKEDY

NKNLDNVIEKNNNYRELMKKQYGFEINDEVVNYRIDYCDRFIPNKDYLSKSKRLSFFDEL

MILDSSIYSSGNIDIDFRDRGIVEYFIESLLYRNLAYNVKIRISKEYKIRKRTIDRIKRI

VLV*

>CD630_03450 Clostridioides_difficile_630_NC_009089 GTP pyrophosphokinase

MEYEKWNEILAPYEHAVEELKIKFKNIRKEYLAKGEYSPIEFVTGRTKKISSIISKLKRI

NAKDIETEIDDIAGIRIMCQFVEDIYAIVDLIKVRNDMTIIGEKDYITNYKDSGYRSYHV

IIKYPINSIAGSKEIICEIQIRTLAMNFWATIEHSLKYKYDHYIPETLAERLRRASDAAF

LLDQEMSEIREDIMKAQAMYQMKSIAIRDTLNRIQELYDLGDTHKAMQYQRKLDRTETDK

NITEILELKQEIDILLEQYKDTKVDDKVVQ*

>CD630_03460 Clostridioides_difficile_630_NC_009089 phosphoesterase

MSLYAIGDLHFSTSVNKPMNIFGSNWDGHEKKIIDNWKEVVKEEDMVLVLGDTSWGINLS

EAKKDLDIISKLPGQKILIKGNHDYWWTTVTSLNKLYEDMRFIQTNFYEYKDYAICGGRG

WICPNDVKFDETDEKVYKREEHRLRLSLESARKSGHSKIIVITHYPPTNDKLEESLFTKL

FEEYNVEKVIYGHLHGKESFKMGLKGIRNGVEYTLASCDYTEFNLIKVHD*

>CD630_03500 Clostridioides_difficile_630_NC_009089 HAD-superfamily hydrolase

MNKLNICIDIDGTITSPYHFLPYLNDIFNKNITEAECITHNWEELYGSGIRDVYAEFNNK

YIHSYDEAKIVEGATEVISTLSKGHNLSFVTARHECLTDVTKNWLSRQGFSDIDVYLLGS

DYKVEKARELSCDIFIEDNPLNSVQLADDGVKVILLDTNYNKDVKHQNIVRVSNWIDIEK

IIKQGI*

>CD630_03510 Clostridioides_difficile_630_NC_009089 hypothetical protein

MIDLKIENRKGKIHVKSSEIKDILELRPDFEYVQDISNTINQENILVFDCQLTGNSFDMD

DLDIEEILEELGEEIDESYFSVLFEDVRAYLKDATDEIEADLQDNYLFDNLRCYFDIYNI

NQEFTDFKFVFLVSFKDIKISSLANLAKIVSKRQLIGASKFYS*

>CD630_03520 Clostridioides_difficile_630_NC_009089 transcriptional regulator

MQSLNEIIAENLKKIRKEKHLSLDKIAQLSGVSKSMLSQIEKCEVNPTISTLKKITNGLK

ISFTSLMERQESDIELIQKSDIDHFIEDNGKYISYPIFPFDSKRRFEIFMIEIEEGGNLD

SNAELPGTQEFITVFSGEVTIKINGEDYIVSSGNSIRFKADVSHIYRNSGKGIAKMSMVV

YYI*

>CD630_03530 Clostridioides_difficile_630_NC_009089 hypothetical protein

MNNKKAVFFFCSDLKKDPVASNVIKCCEEIMDLNQTDIVIDDNFVLEFKDKNNNLFHFVK

TKDVISHNYKYYLPILNRHFRDYDFAGVVNWHEGENAPEHILTAHTIGDVPTGEFGNSNP

RYFKNLINAIEDIKNENLLDEFTTLTEATHWSGTTYGEESKLITEYSVPMLDIEIGSSSD

SFNNSIAIQVLAKSLIRVFDCDEPLKTLLCVGGVHFEKSFSDIIKNKEYNISIGHVLPNQ

WIVSGMYDDESGFKKLEKCINSIEGGIDCIVFHDKLKGTYKEQCRKLGEKLNVPVFKHKI

LKNPKDLPIW*

>CD630_03890 Clostridioides_difficile_630_NC_009089 6-phospho-beta-glucosidase

MRKFPEGFLWGGATAANQFEGGWNLGGKGWSVSDVAKAHFDADVKDYKSNNEITTKDIEE

GLAHPEDEVNYPKRHGSDFYHHYKEDIALMAEMGFKTYRMSIAWSRIFPNGDDKEPNEEG

LQFYDDVFDELISYGIEPLVTMSHYEPPLNIVLNYDGWYSRQVINMFVRYVETICERYKN

KVKYWLTFNEVDSMIRHPYTTGGLVRDRFKDKNFEEVIFQAMHHQFVASALATKICHEII

PNSKVGCMLTKLTYYPYTCRPEDVLATQQKMRSIYAYSDTQVFGEYPVYLLSYFKNNNIQ

IVKEEHDDEIMKKYPVDFISFSYYMSSCEAADTTGLDITPGNTLLAVKNPYLEMSEWGWQ

IDSIGLRISLIELYDRYRKPLFIVENGLGAKDILTEDKKVHDQYRINYLKEHFKCMLDAI

IEDGVELWGYTSWGCIDLVSESTKQMSKRYGYIYVDADDYGKGTYNRYKKDSFYWYKKVI

ENNSIDFK*

>CD630_03900 Clostridioides_difficile_630_NC_009089 PTS operon transcription antiterminator

MNYIIKKVLNSSVVLVHDTKDNEFILLGKGIGYGKKTGEYICGSNDNQMFVPVENTKSKQ

FLELMDDIPVDILKITQEIIVEAEKLLNSSFNKNLYLILADHFNFAIERMRKGIKITNRV

FWEIKNYYPNEFKVGMVAIYMVEERLGIQLPEEEAANIAFHFANAMASDGNSYDTIKYAK

VIGEIINIFIFSLNRTLDKKSMHYMRFITHIKFFVERFFSDSMLSSGDDLLFNQMKRSNP

KEMLIALKVRDFLEKKYGKKLTNEEIAFLVVHIARVKQ*

>CD630_03910 Clostridioides_difficile_630_NC_009089 asparagine synthetase

MINLEEKYLTRKDTYMIKILEYNGHIRNWKVLCEELGIDSSLCRDERERAILVEAYKTWG

YDMANHMHGMFAFALWDTVEKKLFCLRDQFGVKPFYYYETENGKLLYGSSIRKIMEQPGF

VKELNEDMLQIYMSFTYVAGENTFFRGVKKLMPGHYLVYQNKTVCIGRYWKPEFHPDNSK

SIEEWTDEIHTTLQKIMPEVKSENETAELFLSGGVDSSYILAMSDIEKTGSCGYEEERFD

ESKLAQQTANILGCKNSRYIITPEEYFASVPYVMYHMEQPTGDASAIVFAIGCKAAAENT

NICYSGEGADEFFGGYNIYHKAECYGENLKTFYIGNTNIMNEGEKRKILKKYNPDVLPIE

VVKGIYEETEELDTLTNMSNVDIQIWLEGDIYFNIDKMSTAVGLEVRMPLTDTRIFDIAS

RIPSEYKVKGKENKIAFRTAASKTLPEEIAFRNKLGFIVPIRIWMADDQYNKDIRDKFNS

EIADKFFNIDEINEIFNDYISGNSDNWRKIWMIYTFLVWYEIYFVKC*

>CD630_03920 Clostridioides_difficile_630_NC_009089 radical SAM family protein

MNILYTVENSIYVNITNTCPCSCVFCIRNEKDEVANSGSLWLEHEPSVDEVKEAFNKYNL

DDYDEIVFCGYGEPLMRINELIDVAKFIKEKSSIKIRINTNGLSDLIHNKKTAILLKDVI

DAVSISLNAPNKEAYNRVTQPKFGEKSFDYMLDFAKDCKKYIKEVAFSVVDEISPEEIEE

SKQLAKKLDIPLRVRHKN*

>CD630_03930 Clostridioides_difficile_630_NC_009089 membrane protein

LEKMNKVTSKKTTYIVTSALFASIICLTIAYILHIPVGGNNGYVHIGDAFIYLAATILPT

NYAIAASAIGAGLADLSTGAAIWVIPTIIIKPILVLFFTSKSDKIINKRNIVASVVAGIV

GLVLYMFAEGIIIGSFTSAFVMSLLGLLQPIGSFIVFIILGMALDKLDFKKRYFN*

>CD630_03940 Clostridioides_difficile_630_NC_009089 D-lactate dehydrogenase

MKILVFGARDYEEPVIKKWSEEHKDVQVDIYPENMTEENVVKAKGYDGISIQQTNYIDNP

YIYETLKDAGVKVIASRTAGVDMIHFDLVNENGLIVTNVPSYSPNAIAELAVTQAMNLLR

KTPLVKKKVCEGDYRWIAELLGTEVRSITVGVIGTGKIGATSAKLFKGLGANVIAFDQYP

NSDLNDILTYKDSLEDLLKEADLITLHTPLLEGTKHMINKDTLAIMKDGAYIVNTGRGGL

INTGDLIEALESGKIRAAALDTFETEGLFLNKKMNPGELTDPEINKLLSMEQVIFTHHLG

FFTSTAIENIVYSSLSSAVEVIKTGTATNRVN*

>CD630_03950 Clostridioides_difficile_630_NC_009089 isocaprenoyl-CoA:2-hydroxyisocaproate CoA-transferase

MLLEGVKVVELSSFIAAPCCAKMLGDWGAEVIKIEPIEGDGIRVMGGTFKSPASDDENPM

FELENGNKKGVSINVKSKEGVEILHKLLSEADIFVTNVRVQALEKMGIAYDQIKDKYPGL

IFSQILGYGEKGPLKDKPGFDYTAYFARGGVSQSVMEKGTSPANTAAGFGDHYAGLALAA

GSLAALHKKAQTGKGERVTVSLFHTAIYGMGTMITTAQYGNEMPLSRENPNSPLMTTYKC

KDGRWIQLALIQYNKWLGKFCKVINREYILEDDRYNNIDSMVNHVEDLVKIVGEAMLEKT

LDEWSALLEEADLPFEKIQSCEDLLDDEQAWANDFLFKKTYDSGNTGVLVNTPVMFRNEG

IKEYTPAPKVGQHTVEVLKSLGYDEEKINNFKDSKVVRY*

>CD630_03960 Clostridioides_difficile_630_NC_009089 2-hydroxyisocaproyl-CoA dehydratase activator

MYTMGLDIGSTASKGVILKNGEDIVASETISSGTGTTGPSRVLEKLYGKTGLAREDIKKV

VVTGYGRMNYSDADKQISELSCHARGVNFIIPETRTIIDIGGQDAKVLKLDNNGRLLNFL

MNDKCAAGTGRFLDVMAKIIEVDVSELGSISMNSQNEVSISSTCTVFAESEVISHLSENA

KIEDIVAGIHTSVAKRVSSLVKRIGVQRNVVMVGGVARNSGIVRAMAREINTEIIVPDIP

QLTGALGAALYAFDEAKESQKEVKNI*

>PCZ31_RS02220 Peptoclostridium_difficile_strain_Z31_NZ_CP013196 2-hydroxyglutaryl-CoA dehydratase

MKMSEKKEARVVINDLLAEQYANAFKAKEEGRPVGWSTSVFPQELAEVFDLNVLYPENQA

AGVAAKKGSLELCEIAESKGYSIDLCAYARTNFGLLENGGCEALDMPAPDFLLCCNNICN

QVIKWYENISRELDIPLIMIDTTFNNEDEVTQSRIDYIKAQFEEAIKQLEIISGKKFDPK

KFEEVMKISAENGRLWKYSMSLPADSSPSPMNGFDLFTYMAVIVCARGKKETTEAFKLLI

EELEDNMKTGKSSFRGEEKYRIMMEGIPCWPYIGYKMKTLAKFGVNMTGSVYPHAWALQY

EVNDLDGMAVAYSTMFNNVNLDRMTKYRVDSLVEGKCDGAFYHMNRSCKLMSLIQYEMQR

RAAEETGLPYAGFDGDQADPRAFTNAQFETRIQGLVEVMEERKNLIEVRYNGSYFI*

>CD630_03980 Clostridioides_difficile_630_NC_009089 oxygen-sensitive 2-hydroxyisocaproyl-CoA dehydratase subunit C

MEAILSKMKEVVENPNAAVKKYKSETGKKAIGCFPVYCPEEIIHAAGMLPVGIWGGQTEL

DLAKQYFPAFACSIMQSCLEYGLKGAYDELSGVIIPGMCDTLICLGQNWKSAVPHIKYIS

LVHPQNRKLEAGVKYLISEYKGVKRELEEICGYEIEEAKIHESIEVYNEHRKTMRDFVEV

AYKHSNTIKPSIRSLVIKSGFFMRKEEHTELVKDLIAKLNAMPEEVCSGKKVLLTGILAD

SKDILDILEDNNISVVADDLAQETRQFRTDVPAGDDALERLARQWSNIEGCSLAYDPKKK

RGSLIVDEVKKKDIDGVIFCMMKFCDPEEYDYPLVRKDIEDSGIPTLYVEIDQQTQNNEQ

ARTRIQTFAEMMSLA*

>CD630_03990 Clostridioides_difficile_630_NC_009089 acyl-CoA dehydrogenase

MLYNKEQELLRKAVRDFVSKELDTLPAEMDKTGVMPKELIKKLADAKFISSNIPEEYGGG

GAGYVSYAIVMEEIARRCASTATFVTAGSSLASLPILYNGTEEQKQKYLKGIATGELIGA

FGLTEPGAGSDAGGQQTTAELVGDHYILNGRKTFITNGPFCDVAIVIAVTDRSKGLRGTS

AFIVESKWDGFSTGAHEDKMGIRGTETSDLIFENVKVPKENLLGKEGQGFKIAMGTLEVG

RIGVAALALGIAQGALDEAVKYTKQRVQFGKPIAKFQNTQFTIADMETKVCAARGLVYDA

AQKRDAGMRVAQESAMAKYYASEIANEVAYKALQLHGGYGFIKDYEIERMYRDARIVSIY

EGTSEVQKMVISSNVLK*

>CD630_04010 Clostridioides_difficile_630_NC_009089 electron transfer flavoprotein subunit alpha

MNDIKDLSSYKNVWIFAEQREGKIAPVVIELLGEGRKLAKEVDAELCAILLGKDVDGLAK

ELITFGADKVYVADDALLEKYTTDAYTKVIKDAIDEIKPEIMLFGATHIGRDLAPRIASR

VGTGLTADCTKLEIDPEDKKIKQTRPAFGGNIMATIICPNHRPQMSTVRPGVMDKAEKDE

TRTGEVIALDYKITQDDIRTTVLETVKTKKDLVSLTDANVIVSGGLGLGGPEGFEMLKKL

ADKLGGVVGSSRAAVDAGWIDHSHQVGQTGTTVKPNLYIACGISGAIQHLAGMQSSDFII

AINKNPAAPILEIADYGVVGDLHEIVPMLIEKLDSVDDLLEAIKA*

>CD630_04020 Clostridioides_difficile_630_NC_009089 sigma-54 dependent transcriptional regulator

MDNNLIGLIEHVENPAILCKESGEIIYCNHLIDSIFSFLDIKKPRNINELDSNFDKTEIL

TDSKKKIAFRELRMTAHIYNMKDNNNENNIVYLFEKSLISDKVIEDIIEHIDEVVVVFNK

DGVIEKMNTVSDEILPFKRTEVLGRNITDLVRQGLVEEPIILNMLKVKKKIYRNIVYPDG

KLIAYTAVPRWDSKGKLTGGVLTGRDISRVIKLESQIKYSDISEDTEYISQSKIMDNIKK

VVKRAAASDSSIFINGESGVGKEIIARTIYKYSSRRDKPFIAINCGAIPNELLESEFFGY

EEGSFTGAKKKGKKGLFEEANGGTIFLDEIGELPMQMQKKLLRVIQENTITRIGGSKPIK

IDVRYISATNISHEDLRNNLKFRQDLYYRLSVIPVKIPPLRERKEDIVPLVNYFLKLYNE

KYNREVEVSPKVIELLEEYSWPGNIRELKNIIERFVVLSAKNVIGEDEFNMLINLDMIDN

ETDDLSPIVVNGIMNLNDAYKIVDQIMISKAINKYGSITKAAEVIGIYPSTIHRKIKSGH

IHV*

>CD630_04030 Clostridioides_difficile_630_NC_009089 fructose-1,6-bisphosphate aldolase

MALITTKEMFKKAYEGGFAIGAFNISDLEQLQGVLKAAKSKNSYVMIQASMSAVKYAGPH

TLVEMVKAASDEIGVDVALHLDHGPNMDAIKTCIDAGFSSVMIDGSHFDFEENVRITKEA

VEYAHSKGVVVEAELGVLAGTEDDVTSDVHKYTQPAEAVEFVERTGVDSLAIAIGTSHGA

FKFKGEAKLRFDILEEIQSKLPGFPIVLHGASAVDQNAVATCNEFGGNIAGAKGVPVDML

RKASSMAVCKINMDTDLRLAMTAAIRKFLAENPKEFDPRKYIGAGRDAIQAVVESKIDDV

LGSANSIN*

>CD630_04040 Clostridioides_difficile_630_NC_009089 hypothetical protein

MPQIKIRGINENDICKISEKMINDLVEAVKCPRDYFEIECIKSVAIRDGKIADVYPFVEV

AWFDRGQEVQDIVARIITDSIRNNLDVESMDLAFTVFEKEKYYENGEHF*

>CD630_04420 Clostridioides_difficile_630_NC_009089 2,4-diaminopentanoate dehydrogenase

MRKVRVGIWGFGAMGIGMANMILKKEGIEIVSVCSRSTSGKSMYDVLGIERGERPEVIIN

KNYEEVFREKSVDVVLLATDSFTKKAFDKIIFLLNRKINVISTAEQMAYPQADDADLAKK

MDEVAKENGVSILGTGINPGFVLDLLVLALSGTCEEVTSIKAKRVNDLSPFGKSVMVEQG

VGVTREEFIKGVEDKTIAGHVGFVESINMIADGLGWKLDKIEQTKEPIMTTVDRKSKYGE

ALAGNVAGCRQCGYGYVNGEVLIEMEHPQQIIPEAEGIKTGDYVSIKGIPNIDLQINPEI

PGGVGTYAMIVNSIPLIINARPGLKTMLDIPVPRAIMGDIRNQIEVELEEESKAN*

>CD630_04430 Clostridioides_difficile_630_NC_009089 2-amino-4-ketopentanoate thiolase subunit alpha

MDAKINDWVIIHNIVLTPEERAAQVPEDTKKVSLEMWVKGFIQSDASIGDLVEVKTITGR

LVKGNLLKINPYYTHDYGKCIPELLQIGIQAKEILFGGVYNE*

>CDIF1296T_00618 Clostridioides_difficile_ATCC_9689__DSM_1296_strain_DSM1296_CP011968 pyridoxal-phosphate dependent enzyme

MNNSTSMKDMSYQAVMGRNNEIMKNAIGLDYSSFEQEGIGFDYEKMMSETGYTLQDIEAI

QSQYAVGNTPLIELKNLTKLARKCAKEGKGARIFVKDEAMNASGSFKARRAATAVYHAKQ

MGYKGVIAATSGNYGAAVASQAAMQGLKCIIVQECYDSNGVGQPEIIEKARKCEAYGAEV

VQLTVGPELFYTFLVLLEETGYFNASLYSPFGIAGVETLGYELAIQFREKYKKDPDIVVC

TNAGGGNLTGTARGLIKAGSINTKVVGASVDLKGLHMASDNQFNKKSFTTGHTGFGIPYC

TWPDRSDVPRSAARPLRYMDRYVLVKQGEVFYTTELLAQLEGIERGPAGNTSLAAAFSLA

QELDEDKAIVVQETEYTGAGKHICPQLTFARENGIDIKFGNPREEIAGVNLILPERPELL

KCVDIDMNKIRKSFIKNCISNNHIDNIDKLSNRDIEFLMEEVKSSRDFVVDVLNNL*

>CD630_04450 Clostridioides_difficile_630_NC_009089 D-ornithine aminomutase S component

MKKREDDFEVRRKHLQELSDDELKERFWSLATQIVEPMIELGKKNTTPSIERSVLLRMGF

SSLEVKPILEGVMERGLIGKGAGHVVYKLAKSKNITVREAGLLLVKGEYWDEVTCLFREG

VESC*

>CD630_04460 Clostridioides_difficile_630_NC_009089 D-ornithine aminomutase E component

MLKENKKLDIDYILKDLDKYKPKRRGWVWREHLENLEMGPFKYKDCTKPLKKSVGLPSSK

YFNNIDPQPSPVITTEIASGRFEDDIRRMRMAAHHGADHLMVIRTAGQSHFDGLIEGTPQ

GIGGVPITRKQVRAQRKALDFIEEEVGRPINYHSYVSGVAGPEVAVMFAEEGVNGAHQDP

QYNVLYRNINMVRSFVDACEAKKIMAFANIAQIDGAHNANATAREAWKVMPELMVQHALN

SIFSEKIGIDKSNICLSTVPPTAPPAPCLKIDLPYAVALRELFSDYKMRAQMNTKYMESS

TREATVTHVLNLLTSVLTRADIQSTITPDEGRNVPWHIYNIEACDTAKQALVGMDGLMDM

IELKDVGELRDKARELKERAVLYMEEISEVGGYFESVEQGFFVDSGNYPERNGDGISRKI

KGGVGEGTVYEREEDYLAPVTAHFGYNNVAQYDENAIDNPSMLIDGCTFENPDKIIYIDE

LDDFDNVENRLKESYEYRNGTKIKPEMEWCADGIVMITMMLPTDKRTAEFAALEFVKKMN

LQEIEVISREVMHESEGTRIEVKGRVPFDIDLNNLVIPEEPKVLTDEEIRADIEEKPMKI

VSATVGEDEHSVGLREIIDIKHGGIEKYGIECHYLGTSVPVEKLVDAAIELNADAILAST

IISHDDIHYKNMKKLHDYCVEKGIRDKVMIACGGTQVTPEIAVEQGIDAGFGRNSKGIHV

ATFLVEKRREMSNK*

>CD630_04470 Clostridioides_difficile_630_NC_009089 reactivating factor for adenosylcobalamine-dependent D-ornithine aminomutase

MENSKVSLKIDVLVAEIGSTTTVVNAFHDINTNNPIFLGQGQAPTTVFEGGDVRNGLSGA

IKDLANKLSVDDIEYNDMFATSSAAGGLKMTVHGLVYDMTVKAAKEAALGAGAVIRQITS

GRIKRTDLNKIKEINPNIILIAGGVDYGERDTAIYNAEMIASMNLGIPVIYAGNVENQEE

IRLIFEDTNYKLYITENVYPKIDLLNIEPTRRIIQSVFEEHITTAPGMKYIKEMVNENIT

PTPGAVMEASKLLYKNIGDLLTLDVGGATTDVHSVTDGSDYINKILVNPEPTAKRSVEGD

LGVYVNMKNIVEVIGKENLQSELSIDIDAVIENYPPIPKSKEEILFVERLTKEAVVKAML

RHSGKIRNIYGTTGKVKIAEGKDLTEVRYIVGTGGALTRLPSRIEILDNMLKYNKNNELL

FPKEKTKILIDNDYIMASMGVLSKKYEEASLKLLLKSLNFEEERLCILG*

>CD630_04480 Clostridioides_difficile_630_NC_009089 ornithine racemase

MYPRLEIDIEKLKHNVKLISQMCHERNIKISFVTKSFCAQKEIVEEICSEGIDHIADSRI

QNLKNLQDINLPKILIRIPMLSELEEVINYCDISFNSELGTIKKLNELCESKNIIHKIVL

MFDLGDLREGYFYEEDFFNNVREIVRLKNIEIIGIATNLTCYGAIIPSRENIGRLVSIAK

RMEEKFGINLEIVSGGNSSSIHLLINNNMPEGITNLRIGESILLGRETAYGENINGTYQD

AFKLICQVVECKEKPSVPIGEIGVDAFRNKPVYEDKGILKRAIIAIGKQDINIDSLIPID

TDIKILGASSDHMILDVSNTKYDYKLGDNLEFLLTYGGIMSSSTSKYVAKKIIV*

>CD630_04490 Clostridioides_difficile_630_NC_009089 NhaA family Na+/H+ antiporter

MKERECRKASLFDAMIPITCLILFLSLGVLVYGSSPHVPLIGAAAVAGLVAVYRLGFKWK

ELELSMFNSIKMAMQAILIIIIIGVLIGTWIVSGVVPCMIYWGLKILSPNIFLVASTLVC

AIVSLATGSSWSTMGTVGVALLGIGQSLGMPIGLIVGSIISGAYFGDKLSPLSETTNLAP

AMAGTDLFTHIKYMLYSTIPSLLICLVIYGVIGMKYSGQALDIKQIELIRSTLDSTFNTL

SPVLLLAPAIVIGLVVFKVPAIPGLIVGAVLGVLFAVVFQGESMNTILDAAQNGYVSSTQ

IQEVDALLSKGGIMNMMSTVSLTICALSLGGILEKTGMLEVVASSLLRLAKGVFGTVLCT

MVTCTITNIIAGEQYLSIVIPGRMYNKEYKKRGIHPKMLSRALEDSGTLTSPLVPWNTCG

AYITATLGVSALTYGPYALLNIINPIVSLVLIACKFKIARIEDEPETCLNFDV*

>CD630_04500 Clostridioides_difficile_630_NC_009089 transcriptional regulator

MTRISNITIAEQSEYCFLVIRKTIDFMVEFSEFSKQSFEKISKYLEERGILSSGAPIVCF

HNMDLAKLDVEVGFPVATYIDGKNEILQRIVPAQKIITAIDLGPYELQDPTLEDLFSWAN

SNGYKLHGDIYYQYLNDINRPASEYLTKMMLPII*

>CD630_04510 Clostridioides_difficile_630_NC_009089 dioxygenase

MKIYDILTRFYIQDIEKAIPFYENLLKEKCSLRFSYKEVGLELAQIGNVLLLSGSDDALK

PFIETKSTFMVDSVDEWRSYLLDNGAVVVRDKKKVPTGYNMTLRHPDGTIIEYVQHTKQD

*

>CDIF1296T_00628 Clostridioides_difficile_ATCC_9689__DSM_1296_strain_DSM1296_CP011968 AraC family transcriptional regulator

MSYKVINVITAIDYIEEHLSEKLDLDIVANAVHYSKYHLHRTFTTSVGLTMHDYIKRRKL

TEAAKLLVFSKKPIIEIALIAGYESQQAFTSIFKAMYKKSPNKYRKEQEFYPLQLRFVLK

KDNLFSKNVLELEKEIKLASMSDIPLWMNLVRLVIDGFPNLQEEEYIYQLEQYILEERAL

ILKLNNVAIANMVFNRETRSIDFFGIHPQYRNSDIAQVFLKKVIEDFLIDTDISITTFRE

GDKADTGHRDMIKKLGFAEAELLVEFGYPTQKFILPCVEERDNRQN*

>CDIF1296T_00630 Clostridioides_difficile_ATCC_9689__DSM_1296_strain_DSM1296_CP011968 beta-lactamase

MKRKKNFIWIAILLVGVVMVMYYVGKKHNDINQKTDKNYIKSELNKSELNKSKIENNNKN

KKEKINMVDYSDCFEGISGGAIFYNTKNKEYNIYNKELIETRRSPCSTFKIVSTLIGLEK

GVINSKESVMGYDGTEYPNKNWNKNLSLEEAFKESCVWYYKKLIDKVDAKSVQNILDDLK

YGNCDISEWEGDLKNGKGHLNGFWLESSLQISPKEQVQTMAKIFEGDTNFKKEHINILRD

IMKIDVNDKNINVYGKTGTGFDEKNKCVDAWFVGMLEREGDTYYFAIKSDDSNKEITGPK

VKEIAINIIKKYYSVRE*

>CD630_04590 Clostridioides_difficile_630_NC_009089 ABC transporter ATP-binding protein

MEILKCENLTKIYGSNQTRVTALNNVNLSVQKGDFVSIVGASGSGKSTLLHLLGGVDRPT

SGKIYVEDTEISSLKEEALAVFRRRKVGLIYQFYNLIPTLDVRKNILLPMLLDKRKVDED

RFSEIVSILGLSDRLNHLPSQLSGGQQQRVSIARSLIYRPAILLADEPTGNLDRKNSEEI

VDLLNLSNKRFNQTILLITHDEKIALEANRIVTMEDGVIVSEKVVKK*

>CD630_04610 Clostridioides_difficile_630_NC_009089 two-component sensor histidine kinase

VKTPRNINSTVVFIIFCLIVTFLLTVILTTITSFNEYNVLSKALGASYEYNPKSTESILK

SLKNSSDKDFKTGNEILSKHGYSKSTFWSKNFFYFLVISFGIISTMATVIYIIKHRTNKQ

KLSRINNLTQYLEEVNLGKESVLLRCEDEFSHLEDEIYKTVGELRCSKEFALKERQTLSD

NLADIAHQLKTPITSMSLMAQLLSENCPDKEIDYINRLTNQISRLERLVSSLLTLSKLDA

STLIFESKLIDVHSLLTYAIEPIEGSLREKHQTFTIEDSPSVEFEIDINWTLEALLNILK

NCSEHIEDGGYIVAYYSQNPLYVEISIEDNGKGFVQEELPYIFNRFYRGKNASKDSIGIG

LALSKSIIERQNGTLHAENCHNGGARFTIKFYT*

>CD630_04630 Clostridioides_difficile_630_NC_009089 TetR family transcriptional regulator

MSELSKRDKEKIQRENEIIDKAEKLFCLNGFDNTTMNELAKEVEYTKRTIYKYFSCKEDL

FFAVVLRGYKRLWDNVKIESAKGKTGFEKIKLSYFAFHKFYSVEPSLLCLMGMIGIVNSE

KSDTDMLFKEKFFSFNKFMFDEIQGMFEIGKNDRSIRHDIEIPTLMYSSIFTLTGFFNLL

SVTGKSYLNNFNIDEEKFIETTLALLIDSIKA*

>PCZ31_RS02485 Peptoclostridium_difficile_strain_Z31_NZ_CP013196 BlaR1 family beta-lactam sensor/signal transducer

MYAPFFIRFLLSTLTLSILIVVILLAKKVFKKHISAKYQYNIGFILLIMLIIPFVPLKYI

NLGNVFNYLYTFNQLGSSTNRTSMIKNSPNSIINNSNALNDFSVSLNQVNFDSLNILLLV

SWIIGILIMIAITIHCNIKIKNMKKSIQILRDDEIVFIFEKCKKDIGINKDFILCKSPII

NTPITFGYFKPHIILPDKSISKLSLKDIKYILLHELQHFKNKDILINYIMCFLQILYWFN

PLVWYAFKEMRIDREIACDISVLKKLDKDSHIEYGQTLINFVDNISRPSNLTLTSDIGGS

KKQIKKRLLKIMDFHNESNTLKFKSILIFTLIALIVFGNIPKLSIMADINEKYRPTNEQT

VYEDLSQYFKGFDGSFVMYDLKSKQYHIYNKEKSKTRISPNSTYKIYSALLGLENGIITR

NNSHIPWDGKHHSNSSWNEDQDLFSAMENSVNWYFQTLDKKTGLKNIQTYLKQIGYGNYD

VSGGISNFWLESSLKISPIEQVELLESFYTNKFKFEDKNIETVKDSLLISKKDGVSLFGK

TGTANINGKDVNGWFIGYVEKNENTYFFATNIQSKDYSNGSSASKITLSILNDKKSIILN

IFTVHSKKFT*

>CD630_04710 Clostridioides_difficile_630_NC_009089 penicillinase transcriptional regulator

MKKLPHISEAEYQVMKIIWKYAPISTTEVIEKLVETSTWSPKTIQTMLLRLVKKGALTYE

KNSRVFVYTPLVKEEEYVATESSSFLNRFYNGTLNSMVLNFLENDKLSEDDIEELREILN

KRTTKGDK*

>PCZ31_RS02510 Peptoclostridium_difficile_strain_Z31_NZ_CP013196 hypothetical protein

INIKSKLSEFIKQNNLFDDSRIISYLPNITIETDKIKTIMINEVVPTNTNDDFYSVDKDA

DYLKTTIPLFDSAGIKVSNINDILDMGIYITNAVKLPKSEYTITRDTIKLHMPILEEEIK

LFKNLEVVMLMGDVAKKSFNMITKKHIKKNVIPSISTYKLRNNELYYDNLRVFPSYIMTG

GNILIEKSKFEMASEDIKKNVRDYKRLILQNNKN*

>CD630_04760 Clostridioides_difficile_630_NC_009089 hypothetical protein

MILNRTELFRGYSLNRFNEICNILSVNKIKYKYKVKNNAYSKKPFFNEITLGNLGQKEDF

SYEYLIYVHKDDYEQAESLINSKLR*

>CD630_04790 Clostridioides_difficile_630_NC_009089 lantibiotic/multidrug family ABC transporter permease

MNALQSELLKYKRTFMGKLIVFFPVSFAAYAFIMQSTLMQNPLSQTTSWAWQSLLALIFN

WWSFLFLPIGFALFATLVAFQEKKAGNYRALRTHNVSPMTLWINKVIAMAVYSFISTLVL

IVVTIITGLILKAGPVPFGQIIGASIVCWVVSLAILPLQLWLATWKGMFLSMGVGALGMI

FGVLAATKPFWIAVPWSWAVRMVCPIINIHPNGTILEAGDPLLNTSVIPIGIVVSLVVFI

VLTALTAAWFNRRDDK*

>CDIF1296T_00655 Clostridioides_difficile_ATCC_9689__DSM_1296_strain_DSM1296_CP011968 lantibiotic ABC transporter permease

MIKLLSSEWLRAKRTAVQWLTLCMPIIFSLCVVFYLTTKSGSTQEFAFEGFFTVWTVFII

PIGVGILAGFIVQEEELAGNFNGFLCVGISRVRLYLGKFLFLLFCLTICTFIATLILCIG

MSIAVPSGATIWLFLSAAGLVVIGTLPLLAIHLWVSFAWGMGASIGISIGGILMAAILGL

TSVGEKVWAFVPWTWPVKLGMLPGIYFIKEAGTISTEAFYSKVMQTASIELIVVAIGLII

FLIGGVIWFKMWEGRKSYE*

>CD630_04810 Clostridioides_difficile_630_NC_009089 two-component response regulator

LSKILIIDDEMDLVMLLEDELKAKGHEVLIAYDGQAGIELSKQEPDLIILDIMMPKMNGF

EVCQAIRDDVLCPIIFLSAKQSETDKIKGLTFGGDDYVTKPFGLRELMARIEANLRREKR

SQYINEENKRSTLYFGKLCLYMKERAIKIDGKNIDLTKIEYDIVELLALHAGQVFSREQI

YEKVWGCDSEGDSSTVVERVKKIRAKFSAVTPEKEYISTVWGIGYKWNKM*

>CD630_04820 Clostridioides_difficile_630_NC_009089 two-component sensor histidine kinase

MKNQSLITQFRHTFIFIIIASIVATVITYVFALYLYIHSLNKDIYPPNYYERQVPRIEKY

INEKNITLLSQSNEEGLKRTIRGDDMLYQVVDNNGNILYGTNPKKLFKTKEELFNNFINK

TVRKGGYIHTVPIKGDNGKIEGAVILFYQVKITFANIRGRFVFAVIIMALFSPFLYIVGF

TRWLSKRFVKNINQPLHLLIDASKKIKEKDLDFEIDYYSDNELGKLCSAFSEMKDELKGS

LSAQWKMEQERVEMVEALAHDLKSPLSIILGYTDALIGNNTDDNEKLHRYLTVIRENTEK

SAALVQKMQYTSDLEKSNIQLNLVPINLPEFLRQKVQDYELQAHQKEVELILKMQGNIQS

PIQIDVDRLTRIFDNIISNSLQYTPSGGNISITVKDEKNCISYEICDSGRGFSSKDLKKA

LDKFYRGDEARQTKGGHSGLGLYIVKQLVEQLGGSVKIENSKSGGACVKFWHSI*

>CD630_04830 Clostridioides_difficile_630_NC_009089 ABC transporter permease

MKNYLSLSIKELKSQKLMTTFIIIAIVLSTIMTTVVGQSIGILQNLRIEQARSFNGDRHV

SFHQLTKNQVDDIKKDDRVYQAGTSITIGSSKIKDSGISVLVKEYDKTGLSNYPKLMKLK

SGHLPKDKNEIALDENTLKLMGIKPRLGVTIPMNLDISLLNDTIPPYNYTANFKLSGILE

DDYTGYVSGIVNGIVGKGTSENLLPKRYILSSLDFKIKQQEKFQEIVNQLAKKINLSHNS

IQYNWIYLNALKIQFEKDENSSNSDGISMIILVSLFVALLVLLASGLVIYNILKISVTKK

IKEYGCLRAIGAEPNQIYKIVILQILILCTVAIPIGAVIGIISSKGITGMVTNILNPDIL

LANDNKEITELIHKNTTAYMFPLVLSTNVSLIFSFISALPSAIYASHVSPKIAMVGSTTK

IKRKIKREKTIKNFERHLAWLNLKRNKGRTIITILSLFMSITVFVALSEFSNVLDVSRSV

SNLKEGDFSLTNEISGFDKSTLDKIAKMKNVNRTSFIKYSEYKQGEIDTDINFENSGEML

KIIGIDEQTLKDLMPSITDSILEDFKNGSICFIKNPIAISTPGVKTRHTNLKPKDSITIN

KKQLDIYSTVDKMFFLQGNGWINGVDVIVYDSVYNTLTNKNKFNQINIYAKDKSKLEQIR

LSIEQICENNPGSHWISYIESDKQLKESFKQIEFLAWAVILFVGLIGTLNIINTTHTNIN

TRTNEIGVKRAIGMSNSSLYKMFLWEGVYYGIFAAIFGSIAGYASAIIINMATIEKLDFT

NIPITSILQATIISVLACIIATLIPLRKVKKMNIIDCIDINL*

>CD630_04840 Clostridioides_difficile_630_NC_009089 ABC transporter ATP-binding protein

MIIKAKQLSKIYGSNNNKVIALNNVNLEINSGEFVSVIGPSGSGKSTLLHILSGLDNPTS

GQVLLDDKDIYKHTEKELSALRRKSFGFVFQQFNLLPVLTASENISMPVLLDKKQPDKGY

LNEISSLLGIADRLNHLPHELSGGQQQRVAIARALIAKPDIIFADEPTGNLDSKSGSEVM

NLLIKTSKQFGKTLVVITHDDRIAKLADRKISIIDGVLMEVK*

>CDIF1296T_00661 Clostridioides_difficile_ATCC_9689__DSM_1296_strain_DSM1296_CP011968 two-component sensor histidine kinase

MDKINNMSLKKAFFVLTLSSLIIASVLTTFAYILLNNLYNSIQDKYLKTPINTGIITIVQ

NNVNQFSDYDKTLLNIINILQLVLPLIFFIGLLLLADIIFYKVKLKTPIEILNKGAMEIS

NNNLDFCLEYNNNDELGNLCNAFEKMRSELNKNNIKMWTMINDRKQLNAAFSHDLRNPLT

VLKGYSNYLTKYIPTGKLSDEKILSTTQLMSEHINRIEYYVENMSNAQRLEDLVVSKSMS

NINKFIENLDENISIIAKQEGKSFTLKNQINNINLFFDENIILRVVENIISNAFRYARNN

VSILIYLEQELLTFVIEDDGIGFSKESLKLALRPFYRDKTLNDSNAHFGMGLYISKILCE

KHGGSISIENNSTNSAKITAKFSTRN*

>CD630_04870 Clostridioides_difficile_630_NC_009089 carbon-nitrogen hydrolase

MNFYKIAVCQMITTENKIENINHAVDMVTEAAINGAKIVVLPEMFNCPYENKYFPKFAEE

YPGETTTILSKLAEKHGIYLVSGSIPELEDGKIYNTCYVFDKNGTLIGKHRKMHLFDIEV

TGKVSFKESDTLTAGNDVTVIDTEYGKVGIAICYDIRFPELSRLMALKGAEIVILPAAFN

MTTGPAHWELSIRMRALDNQIFYVGAAPARNMNASYIAFGNSRISDPWGRIIAQADEKEC

IIYADIDRDLIPDIRQQLPLLKHRRTDLYELNTLK*

>CD630_04890 Clostridioides_difficile_630_NC_009089 phosphoribosylaminoimidazolesuccinocarboxamide synthase

MKKVYQGKTKDVFELDNGNYLLKFKDDVTGKDGVFDPGENSVGLSIDGIGRANLETSVKF

FEILNNAGIKTHYVSANLEEATMEVLPAKVFGNGLEVICRYRAVGSFLRRYGSCVEEGAK

LDCYVEATLKDDDRCDPLITSEGLEALNIMTQAQFDSMKSMTQKISNIVRDTIAEKGLEL

YDIKFEFGFYNDEVILIDEIASGNMRVYKDGVIVDPMDLTALLLDK*

>CD630_04900 Clostridioides_difficile_630_NC_009089 sugar-phosphate dehydrogenase

VKSVRFYGIRDTRVEDVDVPKILEKDDVIIKVKVAGICGSDISKYSKTGPHMVGEILGHE

FSGEVAQVGKEVRSFKIGDRVAVCPAMPCFECDECKKGLYSRCNNVAIIGNKELGGCFAE

YTKVKERNLIKIPDEISYETAAALEPVCIAGHGLFRSEAKVGDTVVVLGTGPIGLFSIQW

AKIFGSTKIIAVDVFDEKLDLAKELGADICINAKEKNIVEEIKRLTDGDGADIVIESAGT

PLTCGQVLLLAKKGGTVLYAGVPYGDVALTREQFEKIVRSELTVKGTWFGNSFPFPGKEW

SAGLYHMQKGDMNVEKLVTHRINLEEAPAYFEKVYKRDIFFGKIMINIDN*

>PCZ31_RS02595 Peptoclostridium_difficile_strain_Z31_NZ_CP013196 PTS mannose transporter subunit IID

MIHMAGIIIATHGNMAKAMLESAELIVGKQENVETLGLNHGDSIDEFSIKLEDGIEKYKD

EGVLVLLDFYGGTPFNTSAIIINKFAKVCELECLTGVNLPMLLELFLNRDTMKLKDLKNL

CEEVGISGIKDVKTVLKL*

>CD630_04920 Clostridioides_difficile_630_NC_009089 PTS system mannose/fructose/sorbose transporter subunit IIB

MSNIALTRIDDRLIHGQVITAWCKITSAKRIVIVDDLVVKDPFIVQVLQMAAPSTVKVEV

HDVESGAEVLKSHNGDENLIVLVKYPKTVLGLVNSGVDLKELNVGGMGAGLGRKSFYKNI

SVSDEEKEIFRELISKDVKCFIQIVPDAKKIDVGTLL*

>CD630_04930 Clostridioides_difficile_630_NC_009089 PTS system mannose/fructose/sorbose transporter subunit IIC

MHISLIQAVLIAIFYYLSWSPWLTYVGFFTWNRPLLAGFVTGIILGDPVQGAIIGAGINM

IYLGFISAGGAQMGDPAFAGYVGTALAIASKLDVSTAMAIAVPLGTVATVLWIGKMTVNS

FFAHWADREVQKGNVDRVAFINIVPPQILLFAMSFIPALLVVYFGPNAIDGMLEVMNDNV

LHVFNVIGAMLPALGIAMNLKLIGNKFTMPFFILGILMAVYFKVDIIVISVIGVILALTI

TSIKYGKDSATS*

>CD630_04940 Clostridioides_difficile_630_NC_009089 PTS system mannose/fructose/sorbose transporter subunit IID

MSDIKNEVSKKTLSKKDVIKSWLRWFFFAQSNYNYERLQSTAFSHSMLPVLKKLYPDKEE

LKQEVETHLAFFNTEPICGCVIHGITIAMEEEKANGADEISGDGMNAIKTGLMGPLAGIG

DTLTQGVITPIVLAVCIGLTEGGASIAGPILFVIAQYIIMTSISFGMWTNGYKYGKKAVE

SILQGGIVNKVIEGASILGTLVMGGLVGRFINLSTPISYTSGDFKFSLQTDLLDKIFPGL

IPLVLTLLVLFALKKGLSPIKIMCILIVVGAITGILGLF*

>PCZ31_RS02630 Peptoclostridium_difficile_strain_Z31_NZ_CP013196 D-alanyl-D-alanine carboxypeptidase

MEDEILKGKIKQLTILALIFIFITPVFAFADTPPVPNSSRAALLIDQETKRILFEKNIDE

KMPLASLSKMMTFLLAIEAVDKNQVKETDMVKIDKSTASVGGSTCKLKDGDEISLGELMQ

GLMLVSGNDAAIAIAKHIGKTEKNFVNMMNKKAEEIGMIDTYYFNPNGLPIYTDPEHKEP

PIENMSTAHDIVTLGKYMYDHYENQVTRITTMQVYNDTKKDFTHYNTNPLLVSVPGVDGI

KTGYTDNAGYCLAFSMMVPKDAKNERNHRLIGVVLGDGNKKNRISSSATLLKYGKDNFHS

KKIAHKGDIIETPCVDGIDDFKITVKVDKDLYGVVSDNENINPKVVFKNMNYPIHKGDIV

GVAKYYNDSGKFVGSVDVKSESNIGCIPLKDKIKIKVAKINKKLEIKNSVCFKA*

>PCZ31_RS02640 Peptoclostridium_difficile_strain_Z31_NZ_CP013196 membrane protein

MNNTIKEWEICFVDIDRILNFSSNAGKAMLQSGGETYRVEETIARICQSFGIDHVDVFAT

PTAVMASVFVDGKLHSAIKRISSRRVDLNLVHEVNSLSRAISINNLDIELCEKILDEISE

DNYYSDLITIFFAGIAAATFSILFGGNVEEFIAAFTVGILTKFVIMYLSKSSLNDFFVNA

IGAVIIASCSILILKLGFIKTLDHLIAGAIMLLVPGLALTNALRDLLDGQLISGLAKLAE

VFFVGVSIAVGMFLVLGLYFKLGGI*

>CD630_05180 Clostridioides_difficile_630_NC_009089 membrane protein

VHLFIEVIAAFFTALSFGVLFNMKGKNLILAGIGGSIAWFSYKFLLNIGVTENLCFFIAT

VCFAIYCELCARIYMTPATTLSVCCLIILVPGYGIYNTMYSVLTNNYIKAVEYGVSTLSC

ASSIALGLVFITTVFRKVNLYGVLTKIKENEKYKKSINKIKQQK*

>CD630_05190 Clostridioides_difficile_630_NC_009089 hypothetical protein

MEKLSDKRRVKAVSMETFVFIVLLAVGFGYVGSIMGAGMMFKVIMSTAHALLLDTVFLIM

AMAVLAGALSALLSEFGVISLVNKIFKGLMRPIWGLPGASIAGVVATYLSDNPAIIPFAK

DKTFTQYFKKYQVPALCNLGTAFGMGLIVTTFMIAQGKEYVLPAIIGNVAAIIGSIISVR

IMLTFTKKYYNYDPKNDTEKQINDKGAKLEEFREIRDGNVFQRTLDAILEGGKLGVEMGM

AIIPGVLVVCTLVMLLTFGPSTDPATGQAVYTGAAYEGIKLLPAIGDKISFIIEPLFGFT

SPEAIAFPVTALGAVGAAISLVPEFIKSGAITPNDIAVFTAMGMCWSGYLSTHIGMMDAL

DARPLAGKAILSHTIGGLCAGICAHFIFMLVG*

>CD630_05210 Clostridioides_difficile_630_NC_009089 two-component response regulator

VEIKPLVLIVEDDKPICKFIKVSLETQNYRCVETDNGGTAISLIHSLDPDLIILDLGLPD

IDGIEVIGRVRACAKTNKIIVVSAREHERDKVEALDGGADDYLTKPFSVTELLARVRVAL

RNKAQQDNINNDAPKSFEVKNLKIDYENHIVSINGEEIHLTPIEYKIIELMSKYSGRVLT

HKFIIDKVWGNYYESENQSLRVFMASIRRKIEKNPAQPEYILTEVGVGYRMADE*

>CD630_05211 Clostridioides_difficile_630_NC_009089 hypothetical protein

MKIFSFLLFVIGICLCIAGFIGHYPVFVGTTLSFLAIAMIAVRQVKTKKKMK*

>CD630_05230 Clostridioides_difficile_630_NC_009089 transcriptional regulator

MIVGIIGPSDSGFKIKDDLKQIDSDLKTKIYVREKVVDTIEVISKCEDECDAIIFTGCAV

YEFIKSKYEISKPHSFVPRSGTSIMKAFWAIKSANIKLDKFSIDVVDRYVVEDALKEFDI

KATSVFCNPFSLEVGESELVEWHIKLFEENKTDIMLTGFGAVYNELKERGYPVFRLQATI

QLIKESYDKVKSEYALSKARFSQIAVEILSLIDYKEKIDNYYSDMIKKSDMDKLVVNYVR

SIQGSLFLLGRNDYVVFVHKGVADNEYNYDKLFNLKREIKDMGFSLSIGIGTGVTAYQAE

NNAHKALRHSIDSKEIGIYLVDEDENIRGPLASENELNYSLMLSDEKVIEISKKTGMSCE

SVAKIIAINENRKSKIYDSKELAEYLNVSERSARRILNKIVNAGLGRICAKETSIGGGRP

KNITEILF*

>CDIF1296T_00684 Clostridioides_difficile_ATCC_9689__DSM_1296_strain_DSM1296_CP011968 peptidase

MLDKNRLINNFMDMVRIDSPSNQELEMSKWLVNYLKERNIDAIIDDAGEKYGGNTGNVIA

YIKGEEGSRPLCLCAHMDQVQPCLGVKPILDGNVVRSDGTTTLGADDKAGIAAILEALEH

VITEKIPHRDIYLCFTICEEAGMHGVKNFNPDNLPCKDMVILDSGGAIGSIAYKAPAQQS

IKISFHGKKAHAGIEPEKGLNAILVASHAISNMHIGRIDSLTTSNIGKIEGGGATNIVTD

KVTLTAEIRSHIPETLEYELNHMEKCCKDAASKFNTTYTFEHNMSYPSFELSRDSHVFKL

SEEAIRQVGIVPNPMVIGGGSDANILANLGYNCAILSLGMYDVHTVNEYVNIDELYDTAK

IVYHMIKL*

>CD630_05290 Clostridioides_difficile_630_NC_009089 membrane protein

MFANLNLKKSSYQLLVMSVIGVIILIGQRISVGTSIVTALPGMVMLILAAMAAMIIKDLF

PKSIFPAFGFATIIGLLLSMPYSPTSEVFLTNTNNINFMAITTPLLAFAGISVGNKIEAL

KEMSWKIVIISLIVFTTIFFACASIAHIVLSIQGKI*

>CD630_05300 Clostridioides_difficile_630_NC_009089 membrane protein

MEINKKHFIQVFLTCLILVVICEFIGQRTIAIGKASIVLFPMLYAVIIGLIITPDLLGKK

IKALKKVVGEKEINIAGEVVGIALVMLGIKYGTTVGPNIDKIIQAGPAFIAQEFGHILAP

IVALPLALMFGMKREAVGATASISREPSLGVISEKYGINSPEGSGVLGTYLMGTVLGTIY

FSVLGSISIYSGLHPYALGMACGVGSGSMMTAAAGSLSTMVPPEMADTILAYAATSNMMS

SITGIIFLVFVSLPFTNFMYKILEPKFSRNKKTKEIKSTEGEC*

>CD630_05310 Clostridioides_difficile_630_NC_009089 DeoR family transcriptional regulator

MKINRLTEIIVILLNKKLVTAKELADRFEVSTRTIYRDIETLSMSGVPVYMTKGKGGGIS

LIEEYSIDKAILSKKDKESLIVALKTLQATKYPEINSVVNKIGSIFGEQNFSNWIEIDFT

EWGSNFNEDDKFTKIKEAILRRNTINFNYVNSLSSQTNRTVEPLKLMYKSKTWYLYGFCK

LKDDFRIFRISRIRNLSIKDEVFSRKIIEEVCLNDSKVIKENTITLKLRFKEKMLFRVFD

DFNKDLITKNEDDTYDVITEFPIGEWIYGYILSFGDNVEVLEPKDVRDNVITRLRELSKI

YSL*

>CD630_05320 Clostridioides_difficile_630_NC_009089 transcriptional regulator

MNYEIVEINEKIVIGISKETTNKDGQAVNDIGELWKKFMGKGIYNAIKGKKNDKTIGLYT

DYQGDFTSPYSFVACCEVNSNSDKEKNLEELNTNNTVGESIISKVIPAGKYAKFVIVGGQ

KEVGDFWFEFWQMDFDRTYISDFEEYQCNTFDTKKQEIHIYIGIK*

>CD630_05330 Clostridioides_difficile_630_NC_009089 chemotaxis protein CheY

MENKILIVDDAIFMRMIIKEALSKSGYNNLIEANDGEEACNIFKYEKPDLMLLDITMPKK

DGLEVLREIKKLDSSAKVVMCSAIGQENMIVEAIKLGALDFIVKPFKTETLVKVVDNALK

*

>CD630_05340 Clostridioides_difficile_630_NC_009089 chemotaxis protein CheY-P phosphatase CheC

IINYLELDDVHIDALREIGNIGSGNAITALASMINSNVEVLIPMVKILEYNEATNLLGGP

ENKVVCILLDMKGDINGMFMFLLDESITQLMLSSLFNKEEAFLDEIEAIEISAIKEIGNI

MASSYVNAIASMLNMTISVSIPDICIDMVGAVLNVPMIRFSDVGDKVLFIENKFKMSDNY

FTSHILMIPEMSSLKKILVRLGLEV*

>CD630_05350 Clostridioides_difficile_630_NC_009089 chemoreceptor glutamine deamidase CheD

MNREIVVNIADMKIAYRPNVLVTYALGSCVGVCLIDKVAGIGGMLHVMLPYSKDAINIEN

KCKFADTGINELIKSMENIGANRIYIKAKIAGGAQMFLGSSNSVIASIGHRNVEAVKKTL

LGLNIPILAEDTGMNYGRTIRFYTETGELLVDSINKGKKKL*

>CD630_05360 Clostridioides_difficile_630_NC_009089 purine-binding chemotaxis protein CheW

MRFPDDPYLDYGLIDEGIKYLKFKVDEQDFGVELEKIIEITGNQEIIFIPELPTYSKGII

NLRGKIIPIIDMRLRLKKDELMTTNCMYIVVTEIKDLVVGFVVDRVDDIVSLEKSKISPP

PRVTIEYSDYFVSGVGELEGKIITLLDLEKLLTDKDEVLIDELINGFDCTIM*

>CD630_05370 Clostridioides_difficile_630_NC_009089 CheY-like chemosensory protein

MKNKQQGTILVVDDSVLMCDLIDKTLTKDNFKVKKAFNAIEAKEFIEEFIPDIILLDIIL

PDNSGFEFCKEVKSNARTADIPIIFITSKNTDTDIVKGFGVGAIDYMVKPFSMTELKARV

IAHLEVKKSQDKLKKINNELESSLEKLNRLVVRDYLTGLYNRRHIINQLMEQRRINKYSE

TSISLILGDIDDFKVINDTYGHEAGDFVLTVISSIIKKNCRQIDTVSRWGGEEFLIMLTN

TPIEGAKILSERIRKEIKEFDFNYKEHKIKCTITLGVVEVNSNISLEENISLADKAMYEG

KNLGKDCSVVSTMKGLKKI*

>CD630_05380 Clostridioides_difficile_630_NC_009089 methyl-accepting chemotaxis receptor

MKREVKLSKLAIVLLIIIVLAVLAGIFSSISSYKAFEKVEKSINDMNNLRTQSQYIKDTT

REISDVLRRYVFTGEKKNKDIYEKELYGKTRESALNKLEKIGLTKSESNKILEAKEISDS

LVPTEQSAIKAVESNDKSKAQSIIFGSDYINSQEKMDSLINEFQEDIVTRSNSKIVEVKK

ELQSLILRTIFILSLLIILTIIEYTVIKLKIVIPINKVEKHFSRIASGDLRTSIDVEESK

SEIGLLVSSVKKMQSMLLNYIDLIDSTLTSIAKGDLRVEIDQEFIGDFKSIKISLESIIN

SFNGDFSEINLSAEQVASASEQVAAGAQALSQGATEQASSVEELASTINEISDGIKKCAD

SASLACEVSDQSASQVGFGSRCMDDMMTSMGEISEKSSEISKIIKTIDEIAFQTNILALN

AAVEAARAGQYGRGFAVVADEVRSLAGKSAKAAQNTAILIEEAIKTVENGVRIAKETANA

LDLVVNGVEKTTNYINGISSAVHSQEVAITQILSGIEQISIVVQMNSATAEESAAASEEM

SAQAQILKELVEKFHLKESEVRESSWV*

>CD630_05400 Clostridioides_difficile_630_NC_009089 chemotaxis protein CheW

MAKDEIIKQVLTFYVNDVIYGIELENVIETIRFQSITYVPCLPTYISGVINLRGRIIPVI

NMHIKYNLPKADYNERTCIIITKVDEYQVGIIVDKVVDVIHIENLNLLETTNSNNTNINK

DIKEIAKVEDNSILILDIRKFLINSI*

>CD630_05420 Clostridioides_difficile_630_NC_009089 hypothetical protein

VERFNNMVIAIGASVGGTEAILEIIKDLPKSTPGIVVVQHMPAIFTYMYAQRLDKQCIMN

VREAKNNDRVEQGNVLIAPGGYQMKLCTDKQGYYVTCEKGERISGHCPSVDVLFDSVAEV

AGKNSIGIILTGMGYDGANGLLKMKENGSFTIGQDENSCIVYGMPMVAFDRGSVMLQLPL

GEISNCLIRHINILKQKD*

>CD630_05430 Clostridioides_difficile_630_NC_009089 hypothetical protein

MKFFKVDTKAKVLRYVFVLTLTIMVVLAIKHDYKYNNLRKDASINLNSNKEAVTVMSNQS

LPKGNTNSKQTLSVSTIGSKTIVKNDEYLESTINMPVITNSNKIVERSTNDKIKNDIFEF

YNKSYKEAKQYLKDNPDEKNKFVANVDFELKKNTDSALSIKVRYYTYSGGAHGFYQDIAY

NVDMRTGKFLELMDLFKDNTKYKEVIDEEIKRQIAELEKKDEENIGIYNFKGIKENQNFY

LQDENLVVYFDLYDITPYAAGIPEFSINKALISSMLKPEYVDLFDLK*

>CD630_05450 Clostridioides_difficile_630_NC_009089 lipoprotein

MKMVKKLILSTIMALVLVLTIGCSNAEEKVKSNFEANLAALEMYKYDEKGISDALTEAEY

KQIQTYNSKDKVSMIAQLTPLYGKENAEKIYETIVKLIQGVEKTVTVESSSKDKITLKVV

SKALDTSKVMSEANTLVKSYVTDEKNYDKKEKEVQDDMTKMIIDLYNKKPTKEVTTMITY

TKKDGKWQPPSNIQKLCEGIYTI*

>CD630_05470 Clostridioides_difficile_630_NC_009089 beta-lactams repressor

MEIKLFDSELKVMEVLWERGESKAGELVKILKEETGWNRNTTYTVIKKLVSKGAIERIEP

NFVCKALISKEEVQKQETETLINKMFGGSSEKFLSAFINKENLSSDEITKLKQIVEKLK*

>CD630_05490 Clostridioides_difficile_630_NC_009089 hypothetical protein

MKIKKSLISAMMVSAMVLGGTGAVFANGNDSSNIALPKFESLIKSGITDPTELEKYGVEH

YTYEEYAKHIQELKDYAKDPNAVKDVSQKDLEETIKKMEQELEKIKTEGLKIMKPITIEN

EDGSQISIAYNYAAID*

>CD630_05500 Clostridioides_difficile_630_NC_009089 membrane protein

MKKGDFIWSGVLICIIAVLAIPSSREVFMEATGAHPYIGGFLKFAVLATMGDLLGARILY

KDWKIPVGVFYRAIVWGIIGVVTTYGMSIYSSGVGQAQVDGMLPFEGIAFANALFKSATM

NILMSPTVFLFHKVMDCFIDKKYEVGKGIKVKLKDVVNIVDWNAYLGFSVLKTIPFFWIP

CHTIVFLFPAEYRVIASAFASIALGLILALANKSKVAV*

>CD630_05510 Clostridioides_difficile_630_NC_009089 spore cortex-lytic protein

MQDGFLTVSIIDATNNRPIQNAVVNIYSMSNGSQSSSTLYQNLRSNESGQVTGLVLPAPD

VDYSLQPSDVRPYSQYIVEAIADGYETVVIEGTQLLATIEARQGVPMSPRTRSKRSFSRQ

SELIFDIGEHTLYGTYPPKIPESNLKPLPPPTGFVVLDNPVVPEFIVVHDGLPEDSSAPN

YWIPFKEYIKNIASSEIYSTWPEQTIYANVIAIISFTLNRVFTEWYRNKGYNFTITSTTA

YDHKFINNRNLFEPINVVVDAIFNTFIKRPPTSRQPLLAQYCDGQKSQCPDQMTQWGSKD

LGDQGYDYESILRYFYGDEIVFERAPIVSGVPVSFPGTTLQVGSSGQYVRTIQNQLNAIS

NSYPAVPKVIEDGIYGTDTENAVKIFQGIFGLPQSGVVDFKTWYEISRVYVATTRIASLN

PLI*

>CD630_05540 Clostridioides_difficile_630_NC_009089 signal peptidase

VGEAVKKEVVEWIKVIVIALVLAFAITRFIVPTIVKGESMYPTLVERDYLIVNRIAYKVG

EPKYKDIIVFKTDLTEENGKKKDLVKRVIGVPGDHVKIQDSKVYVNDKLLDETSYIHNNR

TDGDIDIVVPEGKLFAMGDNREKSLDSRYDEVGLVDEHTILGKVLVRLYPFSKIGTID*

>CD630_05560 Clostridioides_difficile_630_NC_009089 sugar isomerase / endonuclease

MAVGIASLLFNIEEALNICESIKQITHLEIGIDNISECSELCKYKERISKLGLSIGIHLP

MELNTCENIEYIRNSWINFIEKIEFELKGFDLRYFNLHLGYVMTNRVRKNRDKYLGNSVD

FLDKLNTNSYVCIENTYSKGGDFSNIGNISYDFEYIFKRIKNSKICFCYDTGHCLIDEDA

YVKNLKDKIRLIHLSDNDGINDTHVGIGRGILSEEGIKEVLTLDAEYLVLEINYEDIEDT

ISKLNNIVGEG*

>CD630_05570 Clostridioides_difficile_630_NC_009089 uridine kinase

VKKINLKIEGKDKEYSLEENSPGIRLGDIAKEFCDEHKGYITLAVVDNKLKELNCRVKKD

CEINFLDTTNEDGERVYFRVMSFIFVMACREIFWDSRVTIEHSLSDGLYCEVHIDRKLKE

ADVENIKNKMKEIVNNDYVIEKIEVTRNEAISIFENNEMYEKAELLKYKEYDSAKVYKCR

NYIDHFYGYMLPSTGYIKSFDIKLYNGGVIILGPSEEDKTLPMKFVPQPKLSSVYYEAEE

WSRLMGIDKVVSLNKIIENNEYGDIIRTFEALHEKKLSQIADMIKDGNKRVVLIAAPSSS

GKTSFAHRLSIHLRVNNLNPISISLDDYFIDRKHTPLDEYGNYDFESIYAIDLEKFNLDL

KKLLAGEEIDDIRFNFKEGKREYTGKKIKIDSNQPIILEGIHGLNPILTSSIPDEDKFKI

YISALTQINLDDHNRIPTADLRMIRRIVRDYNFRGYSADNTILQWASVRRGEKKNIFPYQ

EEADAIFNSACVYELAVLKRFAKPLLEEIKEDNPAHIEATRLLKFLQYFVELDDTSDIPG

ISILREFIGGSKIVD*

>QAE_RS0202695 Clostridioides_difficile_QCD_23m63_NZ_CM000660 glutaminase A

MNTLDETLLKEIISSNKKYTNYGQVASYIPELKNARRNDLGICIIDSENNLYSAGNCSTK

FTIQSISKPIVLAMALMDNDWEDVFSNVGMEPSGDPFNSIMKLEINDTKKPCNPMINAGA

IVTTSLINGSCLEEKEERMLSFFRKLAKNDNIGINYDVYKSEKMTGDRNRAMAYLLKSDG

FIRGNVEDVLDLYFKQCSIEIDSVDLARIGINLANYGVDIENGEHLMSEMVSRIVKTFMM

TCGMYDASGEFAIKVGIPAKSGVGGGIMASVPGRMGIGVYGPALDKKGNSVAGVKVLEEL

SNKLKLNIFKILRKDFFDNNI*

>CD630_05590 Clostridioides_difficile_630_NC_009089 ArsR family transcriptional regulator

MKEVLVLRDLECIKAIAHPRRIDILKAFDKSPLSAKQLSQLLEEPHAKINYHIKMLYKVG

ILELVEEKIKSGIVEKYYYPSAKNVVIGNRILNFSLDNGEEKEELYISKFENMSEVFYKA

AEEDVLENENIVDYHDISLTHDELVELSDTMKSKIDEILNKRQHNVEGSKYDIAMVIVPT

LEEECPS*

>CD630_05600 Clostridioides_difficile_630_NC_009089 endonuclease IV

MIKLGTHLSIAKGFANAAETAVYIGANTFQFFSRNPRGGNAKEFDEKDIIKFQEIRKENN

FAPLLAHAPYTMNLGGTKDDVYEFATRVIDEDIRRMDSLGIEYMCFHPGSHVGGGVDFGI

DRIVKGLNNAIKGDENITILLETMSGKGTEIGFKFEQLKSIIDKIEHKEKIGVCLDTCHI

FSAGYDIVNDLDGVLEEFDKVIGIERLKTVHLNDSMMPFGDKKDRHAPIGEGKIGLKALI

DFMEHPSLKHLPFFLETPFDDEGHKRELKMIKEILSSK*

>CD630_05610 Clostridioides_difficile_630_NC_009089 aldo/keto reductase ferredoxin

MRNLGNTNMKIKRVGFGGIPIQRITQDDTNLVINELEKQGINFIDSARGYTISEEAIGIA

IEGKRDKFFLATKSMSRDYDSMKRDVEISLNNFKTDFIDLYQFHNVKEEEYDNLFKDKMA

YSALLEAKEQGKIKHIGITSHNLNTIEKAIEDGKFDTIQFPYNIVEGQADEVFKKAHEKG

IGIIVMKPLAGGALDNATLAIKYILSKDYIDVVIPGMESVEQVRQNVAVLENLVLDEKDN

KEIEEIRNSLGKKFCRRCEYCMPCAVGINIPLSFLCEGYYTRYGLKEWAKEKYEVMDVKP

TECIDCGLCESRCPYELPIREMLKTVVEKLG*

>CD630_05620 Clostridioides_difficile_630_NC_009089 RusA-like endodeoxyribonuclease

LIKITIPGRPISKSNFKLHNINGQAWMPSKGKYSKYLAYENMIAGFINQQYQGETVEENL

ITVLKLFFPNKRMGDLHNYPKSICDGIEKSGIIKNDKQLKPVLLFDFIDKDNPRVEIELY

PVSKYDISYNIFEK*

>CD630_05630 Clostridioides_difficile_630_NC_009089 iron-sulfur cluster-binding protein

MDNKELKAFCNLIGLNCVGVAGVDKYDNLEKILKDRQQKGYLTGMEEPIIENRINPRHIM

KDANSIIVCAFPYHINRQEKNKNSNLSKYCHGKDYHIVVKDFLQQICDYISKTVTDFKYK

LFADNGPLVDRYLAYLSGIGYFGINNNIITDEYGSYVFIGYIVNNYKFEEDKPLEKSCFK

CGKCVKYCPGNAILGNYDMNPKKCLSYITQKKGDLSEKEKVVLKNGKKVFGCDICQEVCP

HNVEIPTTHILEFKENIIDYLDYDEIQNISNKEFKRRYGDRAFSWRGRNIIKRNMEIILD

KYDD*

>CDIF1296T_00724 Clostridioides_difficile_ATCC_9689__DSM_1296_strain_DSM1296_CP011968 endonuclease III

MKNVNLKEINEKDVILEEEINKKGMDKKNIIEKEEINKKGIDKKDIIEKKEINKKGIDKK

DIIEKKEINKKGIDKKDIIEKKEINKKEMDKKDIIEKEEINKKGMNKKDIIEKEEINEKG

IDKMIGKKDEIIDEKDIENFMDIEEDKKSIKVSKKSTKKGETEKKTKKSSSNKATQKTSK

ETKSKSKIKNESANEKDVNKILDELEKLYPDAKCELNYGTAFELLIATILSAQCTDVRVN

KVTSELFKKYNTARDFANLSIEEISKEIKSCGLYKSKSQKIKDTSEQLCELYDGEVPDSL

EKLIKLPGVGRKTAGVVLSNAFNHPAIAVDTHVFRVSNRIGIVDEPNPQKTEFALMEAIP

KERWSHSHHVLIFHGRRMCKARNPECASCPIKEDCNYYKELNETK*

>CD630_05660 Clostridioides_difficile_630_NC_009089 tRNA (cytidine(34)-2-O)-methyltransferase

MSLNIVLVEPEIPQNTGNIIRTCAATGSTLHLVKPLGFSLDDKHLKRCGLDYWDIADIQY

YDSFEELQEKYPNSKYFFSTTKAKQSHSDVKYEENCFIVFGKETKGLPEPLLKANMDTCI

RVPMLDVEKARSLNLSNSVAIVVYEALRQIGYPNMR*

>CD630_05670 Clostridioides_difficile_630_NC_009089 hypothetical protein

MKNLKIICDSLSDVPKDLLDKYDIDIVSLTVILEEREYKDRIDISGDEFYKRLREENVYP

KTSQATYAQFKEIFEKYTNEDREILYIAGSATATGTYQSAVMAKNDVDGKIYTYDSNLLC

MGTGLLVIKAGELASEGKSVAEILPVLDELKEKAFVIFSVDTLEYLQKGGRISSTKAAIG

SILNIKPILEIRDGLVSQISQVRGKKNVISKMMELIKSNCGDDLSDQIVYIGYSDDFKEK

EQLSKVMQDELRPKEIRFFQIGVCIGAHAGPGVTGIICLKNK*

>CD630_05710 Clostridioides_difficile_630_NC_009089 hypothetical protein

MEFTREPFILDDDLCLALVDKRISYLMENELKLRNIEIIKTIECKELYESIKYHPDICIC

NLGKGDIIVAPNVYNQYKELLSRYKFNIIKGEANLLNRYPYDIPYNIAIVGDYAIHNFKY

TDKAILDYIENNKLKKINIEQGYSKCSICIVDRTSIITSDKGIWKSMKNTEIECLLIEKG

HINLFEMNYGFIGGCTGLISKDKLAFCGDVKKHPDYERIKSFVESKNKEIVTLSCENLLD

IGSIVPLMTRKER*

>CD630_05720 Clostridioides_difficile_630_NC_009089 sporulation protein

LVEKDNISSMKFLRNNNVNDRLIYTNDIVEVVIDSAKQEVLNKKILNGTKAEQRALNIDE

ISTEITSMIISIMKEKLLKEYILKCYGRTYPEDKNNIYIYSLNIFAKKEIFMRETVFTRV

RNYIEENDFINVEGFIRFRMREFMKYISAIGDIAVEEYLIKKDQDEFIRVLKYFIDTQEE

KIDLLKVHIMEDNTFVLYDKNGNKIDSIDDEEIINMVIRENLNYEDFLISTLLTLCPKKI

EILDLLNNNCSKEIIDTVEAIFENKVSIIMEN*

>CD630_05730 Clostridioides_difficile_630_NC_009089 membrane protein

MDNLIRILILAVIGGFIGYVTNVVAIRLIFRPIEPIKIPILNIEIVGLIPKRRAEIAANV

GEIIQEEFLSMDEILANIITDEDKEEVVRYIKARVKIIIHEKVSFIPSGIKNMIQDYLGE

IIESEVKQSIDELSKNIINKANERIDIQKMVEDKINELDLYELEEIIIRIAKKELKHIEF

LGLVLGFLIGIAQGLITMFI*

>CD630_05740 Clostridioides_difficile_630_NC_009089 threonine--tRNA ligase

MIKVALKDGSIKEFENAISVMDVAKSISEGLARNVVAASVNGEVVGLDHIIDTDCDLNLF

KFEDKEGKEVFRHTSAHILAQAIKRLYPEAKLAIGPSIENGFYYDIDLDHRLVPEDLEKI

EAEMKKIAKEDLKIERFELPRNEALELMKEQGEDYKVELISDLPESEIISFYKQGDFTDL

CRGPHLPSTKKVKAVKLQSVAGAYWRGDENNKMLQRIYGTSFEKNKDLEEYLHLLEEAKK

RDHRKLGKELGLFMIPEEGPGFPMFLPKGMELKNELLKFWREIHRKAGYIEIESPIILNR

KLWETSGHWYHYKENMYTVKIDDEDYAIKPMNCPGGLIYYNSQLHSYRDFPMRVAELGRV

HRHELSGALQGLMRVRAFTQDDSHIFMLPEQIKDEIKGVANLIDGIYKTFGFEYNLELST

RPENSMGSDEEWEAAENGLREALEELGLPYTINEGDGAFYGPKIDFHLKDCLGRTWQCGT

IQLDMQLPRQFDNTYIGQDGEKHRPVMIHRVAFGSIERFIGILIEHYAGKFPVWLSPTQV

KILPISDKFMDYANEVKKELFDKGIRVELDDRAEKIGFKIREAQLEKVPYMLIVGEKEVA

DNNVSVRSRDKGEIGSIKLDEFIASISKEIESRESIIQD*

>CD630_05770 Clostridioides_difficile_630_NC_009089 hypothetical protein

MSYMPTLEQAWEILRKYNKEEFHIRHAQIVSGVMRYYAKEYDPERVEFWEIVGLLHDLDF

EMYPDEHCVKQRELMTELDLDKSIIDSTISHGYGLTGSDVKPEQFMEKVLFAVDELTGLI

GAAAIMRPSKSVSDLELKSVKKKFKDKSFAAGCSRDVIRSGAEMLGWELDKLIQTTIEAM

RSLIPEMAI*

>CD630_05800 Clostridioides_difficile_630_NC_009089 glyceraldehyde-3-phosphate dehydrogenase

MFNELKTKENVYKNLINGKWVESNSRKPIEIYSPIDNSLVGKVQSMTKHEVDEVIKNTKE

SIKVWAEMPVYKRANIFHKAADLLLENIDEIANILVLEIAKDIKSARAEVERTADFLRYT

ADVGKNMEGEAISGDNFPGGTRNKMSYVSRVPLGTVLAISPFNYPVNLSMSKIAPALIGG

NAVVLKPATQGAISALHVVEIMRKAGIPDGVLNTITGRGSEIGDYVVTHKGINFINFTGS

TEVGQHISKISGMVPLLLELGGKDAAIVLEDADLDFAAKNIVSGAYSYSGQRCTAVKRIL

VQESVADKLVGKIKPLVEKLTIGNPMDEVVITPLIDNKATDFVQGLVDDALHKGAKLITG

NVRKNNLFYPTLLDNVNVDMKIAWEEPFGPVLPIIRVKDINQAIEIANQSEYGLQSSVFT

SDIDKAFYIADKLEVGTVQINNKTERGPDHFPFLGVKASGMGTQGVKYSIEAMTRPKAVV

VNVREL*

>CD630_05810 Clostridioides_difficile_630_NC_009089 TetR family transcriptional regulator

MPKGIVLTPEQQAERREKIVAVALSLMAENGFQKTSMREIAVLANMGKSSLYDFFKTKDE

IVVYAVEKEIEKTIQQVHRIIDGEPSPQQCLRKIMLNHLRFPEQYRTVLMWLNTESDYLE

EEYRKRLKDARYAYQDIIKSVIDNGVTTGIFRKTDTALVTRLLINSMLSISYTSRPLDSS

EKMLDETMNIFLNGIMNDGGE*

>CD630_05820 Clostridioides_difficile_630_NC_009089 pyruvate phosphate dikinase

MIYYVLPLEHKQATIEIVGGKGMSLSKLLTAGIPVPDGFHVTTASYQIFVETNHIQSRIN

KLLDGIDSNNTSQLEDVSKKIGELFHNGEMPQEVSDAIKMAYAGLGNISVAVRSSATAED

LPDASFAGQQETYLNIQGEDKVIDSVKRCWASLWTARAIAYRVKNNIKHEIVALAVVVQK

LAFSDSSGIMFTLNPINGRRSEMIINAAWGLGEAVVSSLVTPDTIVVDKDSERIISYEVA

NKEIMTVRTSEGTEETMVPERLRKKYALTRNQVMQLIQLGKKIEKYYQMPMDVEWALEKD

KLYIVQARPITVLPPEWVLPEQDVVYTKGSLAEHLPNPVTPLFATLGLEIVNRASELLWI

DMFGKSAKKLLPKNGAYTVINGYVYLSAKSKPLLIAIKSLSPRSLRRTLMNSVARWETAR

KGYEDAIKQWEEKPLHTMNAHQIMEGIQTIFYAACIYFTRIQLTLPAASISETLFTKLFQ

GAVRRAGITDTSVFLLGFDTIVLQSEKELWGISEWVKQNNSINLYLQNSPAIKIAEDFMS

SVVPVGVSQEVWIEWKNRINKYFKEFGRTAYEFDFAHSTPQETLTPTFESVKTFVEGKGE

SPFLRQTTFEKRRKHAEEEILQHIGGLRKKLFFKLLHWAQETSPMRENAIYLMGMGHPLI

RCMFQEISERFIRGGAISHLDDIYWLTKSELEVLIEQLDKNKSLSDMNGIIPVRKAELKK

YMGYMSPSKLPEKNKKTISQAQQKQKDGKIVLTGIGTSTGVVTAPACVLNSPADFESFQP

GSVLVAVTTTPAWTPLFSSASAIVTDIGGPLSHSSIVAREYGIPAVMATHIATRTIKSGQ

MITVDGLAGTVTFDE*

>CD630_05870 Clostridioides_difficile_630_NC_009089 hypothetical protein

MSLFVGGWSKFRAVEEEDLKVFNEAVGMLKGVDYKPLIVSTQVVAGTNFKFICNATSVTN

PPHNYLAEIIVFKPLPCDEHNATITSINIIK*

>CD630_05880 Clostridioides_difficile_630_NC_009089 hypothetical protein

MRLNVTKQNTIEVENSRKEDVYFVIVDNVFFERQGDVFNSITNNNLNNYDVKILPTDNVF

LELKITSTKDLEPVFIVVEDRQYNHGSIMFKPVLV*

>CDM120_RS03695 Clostridioides_difficile_M120_NC_017174 hypothetical protein

MPEDNKTFLYELKELLSNGEKQIAKAIGQDLILILGGTGCGKSTAINYLAGCEMHKVEDE

DTGVSYITCEDPVADIGSGAVSKTLYPEVIDMKKGFEGMTAKFCDTAGFGDNRGAIHDIC

AAVSLGEVFKDSASICAVIVLIPEADILDSRLTKVLDLFQQLNMCLHKENFKNSITFFIS

KSFLGRTEKQIYNVIKRKYEQLCEASDSNMSDSNSNIKEKAWLFEEMLKDDGKNIHICNP

LASDDREGLLRQVLELEEVKDKISAFQYPISADALLVLDTLIKEIQDNIVSSLEDYVKAY

TEDFISAVGSITMIEEIAKYDDVVEVFKNWLESTTSPTIGRFINELARQSSLFLQDSCMK

LQKNIKENLDIIDALSEYTEHGTSEILLSSVHMEFIIEKTSKKIMEQNFIIIRKNLEKVL

VSYEVQKQVFTLPNRENLMEVANKKDYKYSVNDFNSILTNVTVTQKEELEDSITRLNAYG

EEGNEIIREFISKYILNPFIVKTININDKQGIEIRALIPNLVISSVLEYFKNRKVDDNVS

TFMFFAKNTIYMDDNLGMDFASEKNIIIACNNLDVGSNVKINVSGKSGELVDNSMYGKNG

EDGRDSGNIYLYIKEEIRNYRLTLIANGGNGSKGIDGKNGETGASGSNGADAGYTSSFHG

KMFKGYFAQGTSGGKGEKGGAGGNAGRGGNAGKKGNIYFISDSEEQKNRIINRCDITVRD

GEVGPNGRPGSGGYGGNGGYHGYNTLSFSPSGIDKTVYYTGYFTSWDYKLDSIIKYHIEF

SGGSWNEGKTGPGREYAEYGDRGADGGFNAPNTKISTAVSKIEDNFIKEKYQCYCSEI*

>CD630_05900 Clostridioides_difficile_630_NC_009089 hypothetical protein

MANKLYSEIVNLLEEGRDELRKYDLKEKSILLFLGASGVGKSTCINYLKGCVMEEKTDEE

TGQIYITAKDSAVEIGNGVYSKTLCPEVVDIANRDFSLCDCPGFFDNRGAEYMIAGAMLV

RETISTSSKVKGMVVILDYQSLLDSRHTLLIETGKNLKDMLGDYAKYKENFFFLINKIPK

TLIGNVTEEKIRNILEKAKEDLDSQEKDYDGCKELVYLLNVALSDGVSIKLCNPLDKQPE

KLLNEMKNLPETFEKTDLGLMLTASCKIEIQKAVDVTYKDLEKLLVPYIESVKKGMEDSI

SILSSINSLKDKKIQLDKMYTDLCSRTNNMKELMEFVQELSYGDGVQKVNILEETRLLLE

LNSYCSETHIVGLSELLKSLLKNTLEDTLKDIKYWIAEEEYREEEAKRLEDAEYRIEKIQ

SCTYIKSYEKENVLSIYIPIPKVNLSDLTKIISGYQLNILSSIYMYGDCELKIDCNMEKE

VFSGKNIVLLTEKLHIPEGTYSINVSGKDAEKLVGESSIYDGMNGRNGNNAGTSGDIILS

FKEIEKRGELKLYVDGGRGGNGQDGKKGDTGITGEAGANAKIGSKKESKEFANVKGTKYY

VVKAEKGKQGGTGGTGGNGGNAGLGSKSGQVYLLGKAPLLKSAISKKDGNHGLAGKGGSG

GAGGAGGLGGIRGIYEETIRYIGTSPPMDNSDRNYYYFDRYVFNHALNQKQAADDGEKLN

ALYEEHKDWTISKREDSGSAGAEGKAGHSGDALKEKELLKQEYSLSIDTFFYEFFERFGF

SGIKGYRNLRLLCAVGILEADILELSHIYEIFHAVEKIEMQLNREILQKYYSFEKKNKAV

LSLDDMNTKKEFYKEAKEIYEYLINKLMQSDRIESFVGKNQNFSEWSMILFALEQKTAFY

VQMIEESEIGGNRVTDIRSLTDYLDKTLRELEQGKKDELKEGYFNTYKESILTQIESGNQ

QIEVLKNELENKMLSVNAKMNLLIQEIIKMKEDEQEDIEELKKKQAKLEEQMKTQLILSG

IQSALSLLTAFSGVGQACLGAFDSAKSLKENLSAKEEEYQWTNRMVDFSDFKLDSDGMRF

VLGEQLSEDRKNALDKMTYNLAGVDEERRTKFDQIKEIIVKSIDDDNYADKADDIQRAKE

GLSESEYKEIYTKIQKKEIGMLEANYNNKQQKDKLAALEKSKQAIERQQKTSKIKNALSI

GSSACSLINTAISGVNNCLNVKSSFESKIEVVKEAIKDKENNIKKLDELNTQMVEFKDKT

LKESVIPFVEKFDSTVKNQDIFENEISKLKMKEVLTELSTKFGSLKMENSEEISIIFKKV

QDMMETQIIITDKIDKKKDNLAMGELIYQMSDSGNQMNEYQKKLIHQIELNQVRYLCQVE

AAAFQLWTFPFGSNIVNILNRCHVGITEDENEVINQAKEQNRQIREFLDQDAYQWQKTDN

HILKVALTGSLDGKSRVGFENTYSLYDCNEEEVKKLLTGDEISFEVPLNKNFDAIKYVTM

YANIPNLRRNILEDSLKVKVHIHLYDSGYFSLYDDKKDVYYQYVFPQNYINITHGLDIKY

NDSDSINSIWSSVEDKGIEKFKNAGLNSGRSPYSMAKISLKAHFNRKLSYDSLTNEQKKK

LESYFDKPSKYAWSDSIDILRSLGYIDLKGILSKSWYEIKKDIDSDGEIMKHFSILQVDL

LERIIYEQTSILKENIPNLTIELVGCGIYVESEFNYLKQNDLKCYEERLVENTIIG*

>CD630_05921 Clostridioides_difficile_630_NC_009089 lipoprotein

MNKNNNLVIICMFIGMILGMAIGCAIGISKGNVGITMCYGLIFGMIIGICIGTIIKNSNK

KE*

>CD630_05930 Clostridioides_difficile_630_NC_009089 hypothetical protein

MYERMLDKSNKPCLSQIIEYIGKDGYARLCKLETYLKTQYNLSRELRFPFGNNYGWGYKY

SHKSFHLFYAFFEKGAFTVLIQIGDKQVPAVEKTLVALTPKAKELWVTRYPCGNNGGWIK

YRILEDADLLDIIQYIRAKKSPPKQN*

>CD630_05940 Clostridioides_difficile_630_NC_009089 hypothetical protein

MDKRYLESDVFEAFQERLSYLFNEFDYIYVSFSGGKDSGLLLNLVLDYKRKNNISSKIGV

FHQDFEAQYSLTTEYVEKVFEDNIEDIEPYWVCLPMATRTAVGNYEMYWYPWDDEKEELW

VREMPVKEYIINQKNNPFTYYKYKMRQESLAKQFGRWIRDIHEGAKVVCLLGTRAAESLQ

RYCSIVNKQHGYKGKCWITKEFKNVWTASPIYDWEIEDIWLAHYKFDYDYNKIYDLFYKA

GVAPRKMRVASPFNDSAKESLNLYRILDPDVWVKLVARVQGVNFTSIYGKTKAMGYRSVK

LPYGHTWKSFTYFLLDTLPENIRNSYIKKFSTSIEFWQNTGGGLSEIVIRELLRKGYKIE

LNGVSNHTKDKKSRVIFLDDIPDDTDDIKFTKDIPSWKRMCYCILKNDHMCRFMGFGPTK

EQRIRTEYIKKKYEKIH*

>CD630_05950 Clostridioides_difficile_630_NC_009089 ParB-like nuclease

MEYKSPVYGIIAVPIEKIESNDYNPNAVAPPEMQLLYKSIKEDGYTMPIVCYYNEEKDKY

IIVDGFHRYRVMLENEDIYDRENGLLPVSVIDKSLDERMASTIRHNRARGSHNVDLMSKI

ITELCEIGKSDEWISKHLGMEVDEILRLKQITGLAYLFKDKEFGNAWIPFGSDIEEEN*

>CD630_05960 Clostridioides_difficile_630_NC_009089 hypothetical protein

MGYLKEDKQDYCCEFARPYINRQIYDDTYNPKTALKRGTLFPELDLIESNNYNDWLYANP

KNRVRAKKGGNMY*

>CD630_05970 Clostridioides_difficile_630_NC_009089 spore coat peptide assembly protein CotJB

MSLNRRELLERISEYQFACIELNLYLDNNPRDKKALDSYNRYCDKFTQAVCDYESKYGAL

TNFGYESSEYPWSWISEPWPWDKSFYK*

>CD630_06010 Clostridioides_difficile_630_NC_009089 methylated-DNA-[protein]-cysteine S-methyltransferase 1

MQYISYYHSPIGNILLASDNIGLTGLWFENQKYYAYNLDQEHQEKELPIFEQTKKWLDIY

FSGKEPNFTPSLHMVGTPFQTTVWKILQQIPYGKTITYGEIAYKVAQQKGISKMSAQAIG

GAVGHNGISIIIPCHRVVGTNGSLTGYAGGIDRKIKLLSLEKVSVQHYFIPKKGSAL*

>CD630_06040 Clostridioides_difficile_630_NC_009089 hypothetical protein

MKMPKESIDTIMFAPCGMNCMVCYKHCYHKKPCAGCLNGDTGKPEHCRKCKIKDCVCQKG

LFYCFECFNFPCKLIKNLEKSYNKRYQASLMENSKFVQQYGLEKFMQKQKERYTCSKCGG

IISIHDRECSECQEKVK*

>CD630_06090 Clostridioides_difficile_630_NC_009089 hypothetical protein

MKKKLEDMTLEELWNLFPIFLTTHKECWKNWFKEEQYRLTGILPMQKIVRISHIGSTSVS

TIYAKPIVDILIEIIPDYNMKDIKDLIESNGYICMSESSSRLSLNKGYTVDGFEERVFHL

HIRYVGDNDELFFRDYLIDHSDVAKEYEKLKLDLWKKYEHNRDAYTDSKLDFVREFTEKA

KVLYSNRYN*

>CD630_06100 Clostridioides_difficile_630_NC_009089 two-component sensor histidine kinase

MGTYSLISTSINFILILINIFIAFFLYTLTSYFIDFRNHWIFKIILILGYSIISQMIIWL

SDPVNILFTLFGYFILLIICSKSKLIPIISVVMILYPIIIGLNFLFINNPIYEELIHSKE

YILILSIVQIISALIKLLFWFLIHIFLKNRLVNIKQYLSNKIWIFIDMVCISSFLSILIA

IILPPTNLIPMGSNKNIILGNLPYGTYLIILSAILANIGVILLLQPLIENVKMKIEKQAN

HLKEKYYNLLENQQIQIRKIRHDMNNHFQMIESYLEIEDIAGAKKYFNQLKIGIGTLSGK

QFCKNQALNSILNLRHTSLEENGVDVHFNIDIDNHLGIEPIDLCTIFSNSLDNAIEASLK

IPDLSNRKVVLKARCEKEYFSYLLTNNKVNPINRKQELFISDKKDSNSHGYGIENIREIV

NKYNGKLNISYTESEFSLFLYIRLF*

>CD630_06140 Clostridioides_difficile_630_NC_009089 hypothetical protein

MFFTFIGVISAILLLCTIICGLWIKLNNRNNDSNSHFTFSILSIAFSLLIVVAFLVRN*

>CD630_06150 Clostridioides_difficile_630_NC_009089 TetR family transcriptional regulator

MKADSTKEQLINATIQLLSSHRDVSSITAREIVAKANVNLAMINYYFKSKEELINISIKK

FLKRHMDEYKATHDTKNLAPKQQLREILIAFFDFIGEHSQFIKISVPYILIQEEIVYPFE

ILPLIKAHYENQNNKKTEAEYRTIAYQITSFIQLLFLRSNAFFKYSGIDILNVEQRNGLI

DFQLDLLLVDITK*

>CD630_06160 Clostridioides_difficile_630_NC_009089 MerR family transcriptional regulator

MFKIGEVSKLTQISIRMLRYYDELGILKPAKTDKYTGHRLYSVEQISILQRIVLLRDSKF

SVAEIANIVHNWNDEFVIKELNRKKNEIQKEIKQEQQRINKIDKFIEAINCDKDEIHYNV

VFKKIPSYKIISLREIIPDYQSEGILWEKLSKFIKEEHIEVSRQSNNNIAFYHDEEVKDN

GVDIEVGMVVKKIGKNKSGFIYRETEEIDMMACTMVYGPYENIAGAYESFCYWLDKNSDY

QISGINRQIGHKGEHNEINPENYLTEIQIPLIKV*

>CD630_06170 Clostridioides_difficile_630_NC_009089 membrane protein

MQLYNTPFNTNKHLIYFFTFTLVLNWIFSLTPALLGITNTILGEIIFYLAYTSPAIVGLF

FISTIYPQSAKQDFIYRYLSFKEIRIKWILLTILFFALTFITSLIIGNYFNVKTSDIDWI

HIAVFRPSKIAYMLLASFILSILSQESGWRGYAIDKLLVRFGFIGSSIILGLVCGIWYLG

SYFTPNQIPYNLAQYSLFDAFLFIPNIVLLNFIINFVYINTSRSILSAGLVHMMYYFFNI

QLLLHYPIKLCVIAQYVQIIFGLIFLVYMISSNKFKQKVNSEIEQIKSDDFEFELDW*

>CD630_06180 Clostridioides_difficile_630_NC_009089 LytR family transcriptional regulator

MKIHVEQGINCDDIEITIKCSALDNNIEKIISLLSTPLLDIKGKKSGEIYKLNVCDILYF

ETVGNKTFAYYEDDVFEIDLKIYKLSKLLANTSFSRISKSMILNMDYIKSIKVISHNRMQ

ATLKNNEKVIINRRYIKNIKEKLNI*

>CD630_06190 Clostridioides_difficile_630_NC_009089 hypothetical protein

MDDSLQLLITNVGFPIALSMYLLVRIEGKLNTLSNSIDTLSKNILIFKK*

>CDM120_RS03825 Clostridioides_difficile_M120_NC_017174 hypothetical protein

MEIKKKLVMSFKNVKDKQVSFSIENPKDDLTEENIKSVMDLIVSKNIFSVGGFDLASVVE

AKVVETNTTPYDLVIRIGG*

>CD630_06220 Clostridioides_difficile_630_NC_009089 hypothetical protein

MEVVSTKNLSSIKLKLDAGFDDKGKAVVKSKSFANVKAEALNEDVYAVAEAIASLQENPI

VDILKLDSTSLSK*

>CD630_06240 Clostridioides_difficile_630_NC_009089 transcriptional activator Mor

MKIKVEDIPVQFHAMVEIIGIDKFIEVAKLYGGTNTYIPTYKGLFRYARNRDIVKQFNGV

NHNELAIRYNMCVSNIKRILNENSV*

>CD630_06260 Clostridioides_difficile_630_NC_009089 replicative helicase DnaB-like protein

MGKSKDVEIEKSILGTILLDNKLSYKLDELNENMFMNDICLEIFKIMKELKKENIVIDVA

TVKSKIDRKSLAIKTSDVTNLITWGQNFGLDGHIKILKENLARRSINQNCQNLLHSLNLG

ENIDTCIYKFESNIKEILDKDTYENDDVNSIAGKVLDFLENKKDIGFKFGIKLLDTTIGG

LFKGELTTIAAKSGVGKTALALQIMLNSFKQGKKTLFISREMTSEQVFMRNICRVTGVST

RNMKSKEIDENDWKLIVNAIGDLSENNLIYINDKIDTISAIRKRIRQVKPDLLIVDYVQL

LTSQKSMDKREREVATFSRELKNMTLDFNIPVIQLSQLNDEMKDSRPYGDRPMRDSKAIY

HDSNNVVYIHQLKGSDYEEAVRDIGESEEAVRASEYRGIKMVDLIVAKCRDGQTRHKHFC

YFGDKLHFQELNY*

>CD630_06270 Clostridioides_difficile_630_NC_009089 transcriptional activator Mor

MLELRKIIELLEEHKIEVSIPKELHKYSLLEYLELCQQSGGTYFYIKTLEGALKEARNEL

MIKLFNGKNYMELARMFEMSVINVRHIVRKK*

>CD630_06271 Clostridioides_difficile_630_NC_009089 ferredoxin

MSNKKKKYANILANECVACGSCIKACPRSAISVPCGISAKINRDLCVGCGICEKICPASV

IEIITILKEEEGKCHE*

>CD630_06280 Clostridioides_difficile_630_NC_009089 membrane protein

MNNKKHWYDYLWIFSSIYLILGFVNIIFAWIGLICFFVPLAISIVKGNKAYCNKYCGRGQ

LFNLLGNKLKLSRNRDIPKFIRSKWFRYGFLTFFMVMFANMLFSTYLVFEGSRNFKEVVT

LLWTIKMPWQWAYHGTLVSPWVVQFAFGFYSVMLTSTVLGLITMILFKPRSWCVYCPMGT

MTQMICKAKSKSI*

>CD630_06290 Clostridioides_difficile_630_NC_009089 Crp family transcriptional regulator

MISQNEIDILTHSLPFWDKLTDLQKELLISSANTSHYKKGNPVHCGDSDCIGILIIKSGT

VRTYILSDEGREVTLFRLDDGDVCILSASCILKTITFDVYVDAETDCDIIQISSSVFAKL

STENIHAELFSYKLATERFSDVMWAMQQILFMSFDKRLASFLIDEIAKNGSSTINMTHEQ

IAKYMGSAREVVSRMLKYFAREGIVSLSRGGIKVLDKDKLRSLTL*

>CD630_06310 Clostridioides_difficile_630_NC_009089 transcriptional regulator

MATIDLIVLGIVKQQPKSAYDIQKAVEYRNISRWVKISTPSIYKKVIQLEQKGYIESKTV

KNGKMPEKAVYSLTELGETHFLDLMFDTSSKPINILLDFNAVIVNLNSLTPEKQKLCLNN

IEENVQELKKTIEENISIKTEVPETAKAVLEQQLVLAQALETWINSLKDNTGN*

>CD630_06360 Clostridioides_difficile_630_NC_009089 radical SAM superfamily protein

MIESIPAKQILQKVKFDNTRWFGIDYNMNLYRGCSHGCIYCDSRSTIYNIENFDKVRYKE

NVIEILSKELRSKRKKGVVGIGAMSDTYNPFEKQLCITKQALDLISENHFGVSIDTKSSL

VVRDIPILQKIKKNNSAIVKLTITTANDELSKKIEPYVNPSSVRFEAVKELNDSGIFCGI

LLTPMLPFLTDTKDEIRAIVEKAHKANAKFIYCMYGVTMRSGQREFFYEHLRDISPKLVF

KYQKTYGLNYVCTVQNKDCCEKLLREECARYGILTDMKDIIKAYKRSESYTQIKFF*

>CD630_06370 Clostridioides_difficile_630_NC_009089 acetyltransferase

MIEIKTIEINKKSQIDLLILADPCEEMIDKYLNKGTMYALYDNKELTCIAVVNEISKEIC

ELKNIATYEHFQNMGYASKMIYHLLDVYSKKYSSMVVGTSESGVPFYEKFGFVYSHKIKN

FFVDNYPEPIFEGELQCVDMLYLSYNF*

>CD630_06380 Clostridioides_difficile_630_NC_009089 hypothetical protein

MDSNNNTIKSTVKKGISFGSCLAMIISYTAWKSIPWAIFHGLMSWIYVLYYWVKYA*

>CD630_06390 Clostridioides_difficile_630_NC_009089 hypothetical protein

MKEKLEEKSKIIKTSKEYKRTIIEEKDREWTKKLNDILSRGDFSNEKLDKIKDLIPKEIL

TSENVGEVVTEDGYAKPEYDEVFKSLFTLNNDYDLLANFINDILKDAPYANRNIKQFTQI

KRIIKVETDPTINYIGEKRPRLDILAEDEERNHINIEMQRALEEDYLERAEYYLSRVHGR

KLEEGKEYKEIGKTVGIHILNHVKYNHIEDYVNCLRLTMDGHPDIFSSKTALYFIELPKI

RKSSCIANRVLIWGKLIDNPSHIDIRILSKTDYVIKRALDRLKELGSNEDYLLNLKRGAY

IMNKSRNFKEEIFQEGIEKGIEKGIEKGIAKGKREEKFNIIIKLKNKGYNLSEICDIIDD

LNKSEVEKVYNQN*

>CD630_06410 Clostridioides_difficile_630_NC_009089 membrane protein

MSKQQTGIKSTLISEILRISYCIGATLVNKYFFKGSSIEYLPIIIWVLAMGAQWVYNKKY

DDLIDEASKCILSRVNDIAIKVLFFSVAIVSIFLVTLDINVSNLNIGMSLLVILFIQSLL

KLILFIYFDRKGIYN*

>CD630_06450 Clostridioides_difficile_630_NC_009089 lantibiotic/multidrug family ABC transporter ATP-binding protein

MNYIIETHQLKKIYKDKAVVNAVNIHVKKGEIYGFVGPNGAGKSTVMKMLLNLVKPNSGE

IVMFGKKVAETDFEILKKIGTIIENPYFYENLTAKQNLDLHCEYMGYYNKEHISEVLECV

GLSKQSNKKVSKYSLGMKQRLAIARAILTKPELLILDEPINALDPEGIREMRELFRKLNT

DYGITIFISSHILSEVEQIADTIGIIQDGKLIKEISMSDIHKYQTDYVEVDVDNVELAGY

LLEKEFGIKDFKITSESCIEIYDLRKDVKEISKIFIQNQIGINSIGRKQSSLEDYFFQTT

GTGGKETL*

>CD630_06480 Clostridioides_difficile_630_NC_009089 GntR family transcriptional regulator

VRVVKVKSNLDEILYHKIIESLIRGEYSVGQKILLNDLCEKFEVSRTPVVQAVKMLNKDG

VLTIMTNGKVYVPEYEYDMVKQVCETRTLIETYALEKMMQEEEEVFQQKLDVIKRYSDKC

EEYYRQEKSVELALADLELHKAIVEGANNEILNDVYVGIQGRFIVVNYLIRPLKNRNYEG

TVQDHFEILKFIEDRDTKKAVDKLRNHIQGTIKRFCEDE*

>PCZ31_RS03410 Peptoclostridium_difficile_strain_Z31_NZ_CP013196 prolyl oligopeptidase family protein

MKEVVGSLKKITKERIENTMKKLFTSETFLEYQFISDCQISPDGGYTAFIVKKADIKENG

YTSQVYILNNKTGELKQITSINSVGAYAWEDENTILFPALRNEKVKEAVKNGKQCMSYYA

LSLNGGEAEELFRLPIKGGKLNPIGKGLYAVIDSYDNDRPIVEGLPEEEQQKKINAYNKR

HYEHFKEIPYAVNGEGYISRKRKRLYVYDSNINELKAITAPMFNVVGMKISDGKILYIGQ

EFKDVKGLKNGVYVFDTNTNTNVCILEKDKYIIKGFELYQNQVILNLTDALSYGNGENGD

FYTIDIDTKEMKLLSGHQHHCIGNTVTSDVKMGAGQTTKVDGEYIYYTSTVDMDCIIERI

HIPTGKQEKVTQTGSVDFIDVKDGNIVCVGCLGNGLPEVYTVENGTLCKKTHLNDHILEE

YKISVPEYIESKGSSKWEIQGYVYKPVDYEVGKKYPAVLAIHGGPRLTYGPYFMHEIQVF

TSAGYFVFFCNPRGSEGRGNAFADIRKQFGDIDYIDFMEFTDTVLEKYTDIDKTKLAVEG

GSYGGFMTNWIIGHTNRFAVACAQRSIANWSGMEGTTDIGYYFCKGQTGASHMENHELQW

KQSPLAYADKCVTPTLFLHGEKDYRCYMQEAFQMFSALKIHGCPTKLCLFEGENHELSRS

GRPKQKLQRLVEMLDWFSVYIKKDNIGQ*

>CD630_06500 Clostridioides_difficile_630_NC_009089 peptidase

MEKIKIESSDIFEYIFPHDISCSPDGKHIAYIISNINEEKDCYEHDLYVMDIKTEKQIHM

TQTKDVTSFSWISNTELLFTSKRNKPKAGTTDFYTISIEGGEAKKAFSIPKACSVPVSLG

NKLWLLTTKNPTDSKKSEPDRAVEGVDYWTFTDKPFIRDGENFSQRRRVTLELYQEGENI

TKAITPKFCEVAGIDVSSDKNRILYTGQIYEDCATPFSGLWEYHIDSGETKELVPQGKYQ

ISLAKYIGKDKVMLQASTLDRSITQNHDIFILDLSTSEINMIASPDGMYATLLDVDAVYG

GGRSNKVIGDKFIGARICRTMTEFNEFDTKTGNIRIITKVDAFTSFDIYDNTMYTVMLKD

YELAEIYSIDMTTGTMKKMTAFSKPYLDTHKVSLPEKLTFVAKNKEEVDGFVIPPIDAKE

GEKYPAVLFIHGGPKWAYGYMFTHLKQCVTSKGMYVIYCNPHGGDGYGEKFLEMVERWGY

VDYEHLMEFVDTCIEKYPGIDADRLGVAGGSYGGYMTNWIIGHTDRFKAAVSQRGISNLI

TASLIIDFGDRIMKQTCGDKTPWNHEEVLWNHSPIKYVKNVKTPTLFLHSDRDYRCFMGD

TFQMFTALKQLGVDTEMYLFHGDTHGLSRNGRPSNRIARANAIVDWFERYL*

>CD630_06510 Clostridioides_difficile_630_NC_009089 permease

MSDMLLWFSVVLVLFAIGDLIASKTKAKVSAVFVTLLLFLILFVTKAIPADIIEKAGMTA

AASWSVPMIMFSMGTMLNVKQFIDEWRTVLTAWLGIVAVIVCVSLCIPLFGKSTVLTSIP

VINGALPATTIMTQAALEKGLTLAAATATVVFAIQKFIGTPIASRAALQEANRLLVEYHE

AKSKGIDLANVDTDKKEAENTGTKVKQAFCEKYDKYYSTNVCIFFIALFSYLGYELSEII

HVNYSIVCLVVGVIVTRIGIVPKDILEKGKIKGFINMVVFAAVIPSLAKVSLTDLISLFV

PIVGMFAASIIGIFFMMKVLPGWKIIGSKPLAFGVGFCQMLGFPTTYLISNEVCNAVGET

EEERAYLMSKIMPKLVVGGMACMISIVVAGLMVPML*

>CD630_06550 Clostridioides_difficile_630_NC_009089 beta-lactamase-like protein

MNLAKGLDVLKISSNVLGEDKVMYIPAIYTEDDATLIDTGLPGQGDLIIDALNKSNTSFD

RLKNIIITHHDIDHIGNINYLREKSKNNIKVYAYKSEVSYITGEETPFKLYMLEQMVDKI

DDKMLSMLNVMGLGFKSSYTKVDVSLDNHEKLNLGEEIEVIHTGGHTRGHICLYLKESKV

LIAGDLLQVENGELKPVDVMHSNKQELKDAIKNISNYDIETIVFSHGGLYQRNIIETLKN

LIIE*

>CDIF1296T_00817 Clostridioides_difficile_ATCC_9689__DSM_1296_strain_DSM1296_CP011968 chloride ion channel protein

MIEKFKTLIWIKMIIRIKSVYKTYGGLFFLGLIGIPVGAIIGLIDTIFGTVLLKVTDIRE

TYPMYLIPFLAVVGVVIAYCYFKFGGKSSKGMNLIFEVGHGEEEIIPLRLVPFIISGTWL

THLFGGSAGREGVAVQIGATFSHWVGKRLPIKNASSIFLVTGMAAGFAGLFETPIAAILF

AMEVLVAGSLEYQSLFPAFTASFTASAVSKALGLEKFSFALSSEVVFDLSIFWKLIVLGI

IFGMVGGAFAWCLKLSKRKIGNRLKNPMIRIAIIGVCLSVLFLLFYKGRYSGLGTNLIQN

SFYGGEIYSFDWLLKFILTILTLSAGFQGGEVTPLFSIGASLGVLLAGFFNLPIELVAAL

GYASVFGSATNTFFAPVFIGAEVFGYSYLPYFFVVCAISYIFNMDKSIYSLQKISTKQ*

>CD630_06580 Clostridioides_difficile_630_NC_009089 beta-lactams repressor

MCLKKLSKLELVIMKFIWNLDIKTNSYEIIDYMKEEHNLPEKVALKTLSKLSKKRFLYVQ

ETGKCMYYTVAIKEKAYLEFISRNVQNLLKNNFIRNLLVSFHEEELTEEKIKSLENWVVN

WEEAYV*

>CD630_06641 Clostridioides_difficile_630_NC_009089 hypothetical protein

MGELQKIPGVGKATEKSLIMLGYTTIKSLKDANPAQMYEKECLMRGQHIDRCQLYVYRCA

VYFASTENPEPEKLKWWYWKD*

>CD630_06642 Clostridioides_difficile_630_NC_009089 hypothetical protein

MFESRCGVCCDSCTKKEQVNCTGCPTMEKPFWGGECKVKTCCESKELNHCGECDTFPCDM

LLNKGKDQGFDPMVNIEQCRKWLEETVSN*

>CD630_06660 Clostridioides_difficile_630_NC_009089 lantibiotic/multidrug family ABC transporter permease

MKYLGLEFYKLKRKKIILMTFLFVTVEIMWCIVNSNRAITRNPDMLGGFEYSYMLMSFAS

LNGLFFPILISIITSRISDIEHKGDTWKLLKSSVTSLNSIYLSKFLCSAILVSIPVLMQV

LSIVLFGHFRGVIESISVSLLLKYTLGTILVSIAIIALHLWIATVIPNQMIAITFGMIGS

FIGLTSGLFYQGIRRLFIWSYYLELSPLSYAYDNVLGSSVYRIDMNFSITILVFLIGMLI

YYIGRNHLLKKEV*

>CD630_06680 Clostridioides_difficile_630_NC_009089 two-component response regulator

MIMESIKDKKILVVDDHKELLKMIDEILRKEGFSRVFLASSYEEAVRVFRNVKPDCAILD

VVLPDGDGFSIMRKIRETSKIPVIFLSARGEDEDRLIGLGLGADDYIVKPFLPKELTLRL

VGILNRVYVPIEEEELPVFKLGDSVVVNLNSACVEKDGQEISLTAKEHSLLLKLYENKGR

IVTNDSLCQAIWGDDMYGYENTLMVHVRRVREKIEKTPSTPKHLITVRGLGYKLMIN*

>CD630_06700 Clostridioides_difficile_630_NC_009089 beta-lactams repressor

MTIKKLPQSELKIMKFIWKSDSKVTSRDIVLGMEQKYQWKQTTTLTLLSRLVVKRFLNSQ

KIDKYTHYEVLIKEKEYIGVETRDFFRNIHDSSIKSLLLSLHENINLSKDDILLIEEWIK

NLKEEEKDV*

>CD630_06730 Clostridioides_difficile_630_NC_009089 methyltransferase

MIFTKSNKYDKDFLMKNMMGPNCIKILEELTSKIKLEKGMRILDLGCGKGISSIFLAKEF

DATVFATDLWIEPTENYKRFKEFKLDDKIFPIQAEAHELPYAEGFFDAVISIDSYHYFGN

KEGFLDNHISPLVKEGGILAMAMPGLKEDFVDCIPDELIPFWQDNMNFHSITWWNKLWSE

SESVIVEKCEALNCHDEAWKDWINCDNSYAINDKKMMEVENGKYFNTISLIARTK*

>CD630_06750 Clostridioides_difficile_630_NC_009089 acetyltransferase

LIEGIKFIKAEEKYIYSYWQTFDKIAKERKYLAMDEAFPFEETVEFIKNIINKNLPQLFI

IDLESDNCIGWCDVLPKTEKVGYLGMGILKEYREKGIGSSLLKQIIDLSKEYGYEKIELD

VFKSNSRAIHVYKSLGFVEVNTISSGFTWNDRPVKEEVIQMELTLI*

>CD630_06760 Clostridioides_difficile_630_NC_009089 anti ECF RNA polymerase sigma factor RsiT

MNKNILNNSFPEVPESFHNSLSKTLNSLPEREENYKMINNKTHKLPFRKGLVATLAITLI

LSTTALAVGHIYSLVGKSSTKATYTSIPTTEQVKKDFNFTPNIVSEFSNGYKFKGAYTVN

KKAFDKENNVLGKSKALSFEYKNGNDEISLDTANNVLGKSSDNLKVVDTYKDINISYEAF

AQKFVPEGYKMTEQDKIDEKSGKYTFSVGPNNESDKVEVNKFKFLTWKQDGVYYSFTVQD

SNLSMNDLVKMAHEVIDAK*

>CD630_06840 Clostridioides_difficile_630_NC_009089 ATP-dependent peptidase

MKMLNNFFKKINNPAPVFAQTKMERDNQTDDSLSTKPKTTFRDVAGLDEVKEELFEIVDF

MKSPQKYQKMGAKIPKGVLFYGPPGTGKTLLASAVAGETNSSFFNVTGSEFVEKYVGVGA

KRVRTLFEKARKEAPSIIFIDEIDAVGAKRHLESNNEKDQTLNQLLVEMDGFNKDSNVLI

IGATNRLDLLDEALLRPGRFDRHIHIGAPNYHTRFEILKVHTDDKPIDKSVNLELLAKKT

HGFNGAHLSNIANEAAIFAVRDDSECITSEHFDKALERVIAGLESKNSALVEKEKKIVAY

HEAGHALVSDIVGICPIQKISIVPRGQALGYVLQLPDEDRYIYTKDELIGKIKILLAGKA

SEELIFNHKSTGAKDDLKKVTEIANQMVCEYGMSNLGFMTIDGNDKTFLCDKVQKEANRI

VEICYKETLEMLKDNLEDLHSVSKFLFEKETMTHEELKDLIGKEAVN*

>CD630_06850 Clostridioides_difficile_630_NC_009089 translation initiation factor IF-3

ISKELAINEQIKDKEIRVLSPTGEQLGVMPTKEAQAMANSKNLDLVQISPNANPPVCKIM

DYGKFRYEQARKEKEAKKKQKTIVVKEVRLRPGIEQNDLNTKANNAIKFLKKGDKVKVEL

RFRGRELGHKDIGKEVMLKFLDIIKEFGEPTKAPAFEGNNMVVIIDPKK*

>CD630_06860 Clostridioides_difficile_630_NC_009089 50S ribosomal protein L35

MPKMKTHRGAAKRLKKTGTGKLKRAKAFKKHILTKKSAKTKMNLRKSTLVSDGDAKRIAQ

LLPY*

>CD630_06870 Clostridioides_difficile_630_NC_009089 50S ribosomal protein L20

MARVKKAMNARKKHKKILKLAKGFRGSRSKLYRPANTFVMKALKNAYIGRKLKKRDFRKL

WIQRINAAARMNGISYSRLMNGLKLSGVEVNRKMLSEMAIQDPEGFAKLAEVAKAKLA*

>CD630_06930 Clostridioides_difficile_630_NC_009089 MerR family transcriptional regulator

MFKIGDFSKLSKISIRMLRHYDEIGLLTPSHTNKTNGYRYYSADQLSTTNRIHALKDMGF

GLYSIKEILTEYNDKESLIKYLNIHHSQVKEQLEDTQKKLLKIETTIKRIGGNDIMKNYD

VTIKNFAPKYMMTLRRVIPTYQDEGMLWHQAFLETKDQNVQIEPPKYSKAVFYDTGYKED

YVDVEVQVAVSGKYKDTEHVKFKTVPSVTAATAIVNGNFNQVADACEAIGNWISDNNYDV

DGPMFNIYHVSPGNDSNPDNWVTEVCFPVKKK*

>CD630_06940 Clostridioides_difficile_630_NC_009089 TetR family transcriptional regulator

MKSNDKYARERIIEVTLNLLNEVDDIEEITVRKIAERANVGVGLINYHFKTKDNLLSTAI

GDVMSNIIAELYDDSVYTLRPIEDLKNLLKKLCDTGLHYEKVLPFVLNQCITNGDMQAEL

DIVPMLRKIFGNKKDEMSLRIIALQIILPIQISALSTESFQLYSGINIKNKYERDKFIDI

LIENIIGEDVDVR*

>CD630_06941 Clostridioides_difficile_630_NC_009089 hypothetical protein

MKIITIGSSLITVLLFLSTMICGFWIKNNKVTDASSIKFHMNSAIFTGIFLLISTILLII

YIKK*

>CD630_06960 Clostridioides_difficile_630_NC_009089 cation transport protein

LKTIVRRLILYADGMRPTQIMVSGFAAIIVIGALLLTLPIASQSGESIGLLNALFTATSA

VCVTGLVMVDTATYWSLFGQIVIITLIQIGGLGFMTVATMFSLMARKKIQLRERLLIQES

LNQADLSGLVRLTRFVLIITITIEGIGALVLSTVFIPQFGLSKGIWYSVFHAISAFCNAG

FDLMGSVSGPFTSLNSYVNNFTVSMTVCALIVLGGLGFPVVLDIVRKRRFSKLNVHSKVV

LFSTATLIFVGALFIFLIEFNQKATMADLPLKGKVLSAIFQSVTARTAGFNTLDLATLRE

SSVFVMIILMFIGASPASTGGGIKTTTLAVLIITVRSFLSGKSDIEAFERRLAPSTIKKS

LGIFVISISAVIFGTLIISITQPNFTLVQSAFEVTSALATVGSSLAGTPNLNALGKIIII

IFMFMGRVGSLTLFMAILSGGRRKSQPIRYAEGKIMVG*

>CD630_06970 Clostridioides_difficile_630_NC_009089 K+ conductance regulator

MKQYIVIGCGRFGSSVASTMHLLGHQVMAIDKNEDSVQSISDKVTHSLIVDVTDEQALRS

LGLGNFDVAVVAIGSDIRASIMATLIAKEMGVELIICKAKDELQAKVLYKIGADRVVFPE

RDMGVRVAHNLVSDNILDHIELDPEYSIVEIVTPNSWVGKTLIELELRARYEITVLAIKT

GKNINVTPSPDEELTAGSILVIIGQNTSITAITSGNKGIIRRR*

>CD630_07000 Clostridioides_difficile_630_NC_009089 phenylalanine--tRNA ligase subunit beta

MLVSLKWLRDYVDIDIDVKEFADKMTMTGTKVETIDYYGEEIENILVGKILEIKQHPNAD

KLVVTKVDIGDKVVQIVTGATNISEGDYIPVAVNGSKLPGGVEIKQTDFRGELSDGMMCS

AAELGIDEHYIEEYKRGGIYILDHEDSYELGKDIKDVLGLKDALIDFELTSNRPDCKCMM

GIAREAAATIGTKVKYPEIEVKESDEEIDFKVEIDNPDLCRRYVARMVTDVKIEPSPYWM

QRRLTEAGVRPISNIVDITNFVMLELGQPLHAFDINQVETGRIVVRNAKDGEKLVTLDDV

ERTLDKDMLVITNGEKSLGLAGVMGGANSEITSNTKTVLFESANFKPENIRMTAKKVGIR

SEASSRNEKDLDPNLAEIAANRAAQLVEMLGAGKVLKGVVDVYPNKPEPKKLVVNPQRIN

HLLGVDVPMEQFVGILESLEFKCNLVANDKLEIDVPSFRTDMEQEADVWEEIARIYGFEN

IPSVQLEGNTTAGIKTSKQKFMDALKDNSTAVGLNEILTYSFVSPKGVDKIRVPEGNAKR

NFVKLLNPLGEETSVMRTTLIPNMLDVLSTNVSHKIEEVSAFECGHIFIPQDSELPKEEN

RMCVGMYGKDVDFFTLKGTIETILVNVGFKCYEIEPQDNNTTFHPGRCAKIVYNNKYVGT

LGELHPDVIENYNLGQRVYVAEIDIDFVFDNSDRTKNYVPLPKYPSTSRDIALIVKDDVF

VKQIEDIIKENGQGLVESYKLFDVYKGSQIEAGYKSIAYSITYRSKDKTLTDEDVAKVHD

KILSELSEKLNANLRSN*

>CD630_07010 Clostridioides_difficile_630_NC_009089 cell-division protein

MNKVMVKIHGAEYPMVGDKSEKFMISIADFVDKEMDKITRQNPKLSLSVAAILTALNISD

LLFECSDENEKLIKANEELSKKVGASNEELQLEIKSLKLTIAEKEAENREAETKMKELIE

IIENKKQEIFELSNTTEGSRAELDAYKNKIEELSTQLEEANERATIAENLASEFQNKAYD

LQLKCTGLNNDAKNVE*

>CD630_07040 Clostridioides_difficile_630_NC_009089 metal-dependent phosphohydrolase

MREEALEILFKYLETDRMRKHCYAVEAVMRELAKRLEPEKEEEWAVAGLLHDLDSDIVGR

KPGEICEGHATTTVELLKKENFGDEEMYRAILGHHDGMGVTRTSLMEKAIYAADPITGFI

TAIALVYPDKKLTSVKTKSVIKRMKETRFASNVNRDAMRSIEDIGIPFDEFAELSLNAMK

NISDMLESAENN*

>CD630_07050 Clostridioides_difficile_630_NC_009089 NUDIX family hydrolase

MKLTTICYIEKDDKTLMLYRNKKKDDIHEGKYVGVGGKFEQGETPEECVIREVKEETGLT

LKSLSYKGLITFPKFKDEEDWYMFLYFSDEFEGELSEKDLNDCKEGNLIWVDNDKIFDLN

MWEGDRLFLNWAKTGNIFSAKIVYDNGKLKDYNVSFLD*

>CD630_07060 Clostridioides_difficile_630_NC_009089 Fe-S domain-containing protein

MIDINLEKCVGCGMCESDCLVNAIKVKDDKAKVKNILCINCGHCMAICPTDAIEMQGFDK

NEVIEYNRETFELEPEKLLNFIKFRRSIRQYKDIEVEEEKIKNIVEAGRYTPTGGNRQPI

RYILVKEKLKEVKELAIQGLYNLALDTDDNDPVRSIYKNTFKKMYKRYKENGNDSLFYDA

PLLMVVVGDMSLGGSAYVDGGLAASNMELMAYSQGLGICYNGFFVMASNVEPKIKELLGM

SENEAVITSFILGYPDVKYKRTVNRNTAKFEVR*

>CD630_07080 Clostridioides_difficile_630_NC_009089 peptidase D

MGNVLEGLKPESVFKNFEKISQIPRGSGNEKGISDFLLSFGKNLGLETIQDESLNIIIRK

PATKGYENCPGVVLQGHMDMVCEKEKNVEHDFLKDPIKLRIDGDMIYATGTTLGADNGIA

VAMGMAILEDNTLEHPALEVLVTVNEEDGMNGADALDPSLIKGQYILNMDSEEEGYLLVS

CAGGKTCVVSLPVEYKEVKGDKQGLLVEVTGLLGGHSGMEIVLQRANANKAIARVLSVLN

VDYELASVDGGTKHNAIPREAKCVIAVNKADVESAKKQINDILTAFKHEFTTSDPGMTYS

VAETSVDKVLTKDCKEKVVQMSCLTPHGVQSVSLDIEGLVESSTNFAIIETKESTIEFLT

SVRSSVMSIRDEIADRIRLLAQALGANYDLIAQYPAWEFKKGSKLEKICSETYEKLTGKV

PTVMALHAGLECGLLLDKLPHAEAISIGPDMFDVHTPNEHVSIPSVANVWDYVIEILKSM

NQY*

>CD630_07090 Clostridioides_difficile_630_NC_009089 DNA mismatch repair protein

MNEKSLRVLEYNKIIDLLKKKASSSLGLKYIENLVPNTDFVEVKSMLEETSEAQSIIIKR

GSVGLEGIHDIEDKVKRAYIGASLDPGSLIMIADTLRVARRLRNSLSSSDEEDFNYPIIQ

SLSNSLYVYKDIEDQIYNAIISEVEISDNASSILRDIRRRIAQKNQSIRSKLNSIISSTT

YQKYLQDAIISLRGDRFVVPVKSEYRSQVAGIVHDQSSSGATLFIEPMTIVEMNNELRQL

KLGEQEEIERILSELSAMVGEVSEDLISNQEILGRLDFAFSKGKLSIQMRGIEPTLNEDK

YLNIKNGRHPLLDKKKVVANTIYLGRDFHTLVITGPNTGGKTVTIKTVGLFALMTQSGLH

IPADYGSSMCVYDNVFADIGDEQSIEQSLSTFSSHMTNIVSILQNVTADSLVIFDELGAG

TDPVEGAALAIAVLEDINSVGAKCIATTHYSELKNYALTKSGVENAAVEFDIETLSPTYK

LLIGVPGKSNAFEISRKLGLSDYVISRAKEYINTENIALEDVLQNVEKNRIKAVEDREEA

ERLKEEIEKLKVEYDEKLEKLVSQRDKMIEKAKSEAFSIIRQAKEEVDIIIKELRSLEQE

RASKEKNRKIEELRKELTSSMGSLQPTVKSMIVPKVSNKEIKDLKPGEEVKVITLNQNGS

VVSVDKKRKEAVVQIGIMKMTLPFKSLQKTRKDVSTNVTKSTRNIIRSKSGSVKNEVDLR

GLNLEEAIMEVEKYLDDAYVAGLESVTVIHGIGTGVLKAGLQDILRRNRHVKSQRGGQYG

EGGAGVTIVKLK*

>CD630_07110 Clostridioides_difficile_630_NC_009089 arginyl-tRNA ligase

MQDFKVAISNCLKEKIEDLSKEEIEALIEVPPNKDMGDYAFPCFKLAKVFRKAPNMIASE

LAESIEPSGEITKVIQLGGYVNFFVNKSQLAETVIKKVLDEKENYGHSDFGKDKTVIVEY

SSPNIAKPFHIGHIRTTVIGNALYKIYDSQGYKTIRINHLGDYGTQFGKLIVAFKKWGEK

EVVESNPIPELLKLYVRFHDEAEQHPEMEDEARAWFNKLENGDEEAQELWQWFRNESLKE

FNRVYKLLDIEFDSLAGESFYSDKMNRVIELLEEKNLLKESKGARIVDLEEYKMPPALIT

KNDGSTLYMTRDLAAAIYRKETYDFDKCIYVVGSQQNLHFQQWFKVIELMGYDWAKDLIH

VGFGMVALEEGTMSTRKGRVVFLEDALNQAIDKTKEIILAKNPNAKNVDEISKQVGVGAV

VFQELSNSRIKDYTFSWERTLSFDGETGPYVQYTHARCCAVLRKAEVEVTSDIDYSLLAD

EDSAEVLRVIESFNKNILLALKKNEPHIVTRFMLDLAQAFNKFYHDNPILVENLEIRKAR

LALVLATKQTLENSLKLLGMHAPERM*

>CD630_07120 Clostridioides_difficile_630_NC_009089 hypothetical protein

MNDELLKKVYTSSFSSASRALKDLTIINTKSLIDDKTQRCVYIDENRLRDELIYYRFYGE

KIGNINFLNILLPLILSNTNIQKSEDEVIKLIKKYVIYFKKEELLFDYLLGSVVYNSIMH

NLINNSKIEYVELLQSIKDKIIGFSIELDKSDVVKFQMARIKAIQLIDKYIDLKSEDYDE

ESILLNVLNVLYDVYMEDRTVENEGINSIKKSILSILGEDSKLNEDNIDFIFSMSEYVVK

LRKYKIGVKAYNKSIDPRSLIRLEEGNTIVDPIFNQITVMSKTFNDNILSIKINSKSGIY

ILKFKKV*

>CD630_07121 Clostridioides_difficile_630_NC_009089 hypothetical protein

MELIEKVSINSLSKRDLLLIIKSLEYTNENTQISDFIDLRNNIVKELCFLTDTKEKDFLD

YLEKNSTL*

>CD630_07140 Clostridioides_difficile_630_NC_009089 hypothetical protein

MDFKIEKYLLKKAEELAFITIKHGGEFKLKSYEVPKGGLDVPIKNEVLVKGIKEKTAQDK

LNSMSIADAMIYIIGIDSKFKNNAEYEKFLNALSKDIDLDLKSYMGYMSRKYFEIGEHTD

SLIYIKAFITMYPDDLDAMYNYAIVCQEIAKQYQKDMDDKAMNAFLLEAMAKLEKVIDVD

ENFALGYYHLGYHYYNQGQYLKTKLTWEEALRLGLDADLVAEVQENLGKMDFKVQYEEGY

TLVFQGKFKEGLEKLLPLEEEHMDWWNLLFMIALGYKGMGEIEQAKMYLEKILIIKPNQV

DTIVELGLCEAYKNNLDKAIEYFEQAAKIKEDPEILCNLGMAYLNNGDIDDATYYIERAY

ELNPQDEITIVCLRELGINK*

>CD630_07150 Clostridioides_difficile_630_NC_009089 phosphate butyryltransferase

LIKKLDDILEQLKGGEKIVLSVAAAHDKEVLMAIKDAVERNIITPILVGNEGKIREISKE

IGFDLSGIKIVDKDDIKECAEIAVKLVSSKEADFVMKGLLDTSVILKEVLNKDYGLRTDS

LLSHVMVYELEKYHKLLITTDGGMNIAPDYEQKAKILKNSIKAAKALGMETVKVACLAAK

EKVNPKMQATVDADMLAKACKEGEFGENVIVEGPLAFDLAVSKEASEIKGFKSEVSGDVD

IILVPTIEVGNGIGKAFTYMADSKSAGIIMGAKAPIVLVSRADSHESKLYSIAYGAIVAK

NMK*

>CD630_07160 Clostridioides_difficile_630_NC_009089 bifunctional carbon monoxide dehydrogenase/acetyl-CoA synthase

MDEKMLTIDLNSQKMIAKAREEGVETMYDRKEGFKAQCGFGLQGVCCRICGMGPCRISPK

TPRGLCGADEHTIVGRNFARMVAGGTAAHSDHARDIAHTLALADPNGNYKIRDEAKLITL

AKEWDVETEGRDIYDVAHEVAEIALMEFGKPFGTLRFIKNAPEPRQKIWKEYAIEPRAID

REIATIMHSTHIGCTGDIDSLIHMSLRTSMADGWGGSMIGTRLSDILFGTPVPRRTEANL

AVLEENKVNIILHGHEPALSEMIVLASEEPDLVALAKEVGADGINLAGMCCTGNEITMRH

GVKIAGDFHQQELAIITGAVEAVIVDVQCIFPALARVADCYHTKFVTTSPKAKITGSTYI

EFREEQALDDAKAIVKEAILNFKNRDKSKVLIPELKSGATVGYSVEAVVNQLDRVVNSHI

DPAGTVKPLTDCLKSGVLRGAAGVVGCNNAKGVSNEAHVTIMKELIKNDIIVVTTGCGAS

AAAKFGLMESDAAEKYAGKGLATVCKLVGIPPVLHMGSCVDISRILDLVGAAANYLDMDM

CDLPVVGIAPEWMSEKAVAIGCYVVASGIDTYLGIMPPIAGSSRAVDILTSELKDKVGAT

FTVNTNPKELAATIIEDIEKKRVHFEALVEEKMAEKAEA*

>CD630_07170 Clostridioides_difficile_630_NC_009089 bifunctional carbon monoxide dehydrogenase/acetyl-CoA synthase accessory protein

MKIAITGKGGVGKTTFSSMLSRMFAEDGYRVVAVDADPDANLALALGFPKEVYESIVPIS

EMKKLVSDRTAASVGSFGKMFKMNPKVDDIPENFCKEYNGVRLLTLGTVDSGGTGCVCPE

HVLLKRLCSHLILQNKDVVVMDMEAGIEHLGRGTAQGVDAFIVVVEPGERSLQTYRKVKK

LGHDIGVNKVFVVGNKIRNKEDEEFIIQNLEDGESLGFIYYNQDVIDSDRANQSPYDSSE

TTKEQIKAIKDKLMSLKDK*

>CDIF1296T_00890 Clostridioides_difficile_ATCC_9689__DSM_1296_strain_DSM1296_CP011968 FolD bifunctional protein

MEGMSTKGQIIKGKPVADKISEELIKEVDLLVKEGINPKLTIVRVGARSDDLSYERGALK

RCQNIGITTEVLELAEDITQEEYIDVLKRVNDDKNVNGILCFRPLPKHLNEEVIKYVIAP

EKDVDCFSPINSAKVMEGDKSGFPPCTPTAVVEILKHYNVDLKGSKVTVLGRSMVVGKPV

SMLLLSEHATVTICHSKTKNLSGVAAEADVLIAAIGRAKMVDESFVKDGAVVIDVGINVD

EEGNLCGDVDTNAVLDKVSMITPVPAGVGSVTTSILAKHVVKACKLQNNK*

>CD630_07210 Clostridioides_difficile_630_NC_009089 hypothetical protein

MIISENKPLEEVLGYLKDFDKLVLVGCNQCAATCKSGGEEEVLKMKETLEGEGKKILGYV

MLDPACNLLKSKKDLKALKEETKEADAVLSLACGDGTQTIVKNLKDKPVYPANNTLFIGE

VQRVGEYEEACKACGDCELGWTGGICPVTMCAKGLMNGACGGAKNGKCEVNSENDCAWIK

IYERLEAIGQLDNLAEIRPPKDYSKQNNPRSLSAKKKKEAAANS*

>CD630_07220 Clostridioides_difficile_630_NC_009089 5,10-methylenetetrahydrofolate reductase

MSLLRETLESGKFAVTTEMAPPKGTDLSHLIECAKPLVGRVHAANVTDFQSAVMRATSLA

TCKLLKDAGLEPVIQITGRDRNRIAIQGEMLSAGVFGINNLLALTGDHTSVGDHPQAKGV

FDLDSVGILQTAETLMAGTDMAGNKLKGSPDFYLGASVTPEYSPIEVQLLKMKKKIKAGA

KFFQTQALYDINTMRKFRELTRDMDCKVLAGIVPLKSPGMAKFMTANVPGIFVPDEQIER

LRAAGKENWVSEGIKMAGELIKQLKEEDLCDGVHIMAIGAEENIPAILDAAGL*

>CD630_07230 Clostridioides_difficile_630_NC_009089 bifunctional carbon monoxide dehydrogenase/acetyl-CoA synthase dihydrolipoyl dehydrogenase subunit

MKIVVVGGGPGGYVAAIKASMLGADVTVVEKRRVGGTCLNAGCIPTKALLASSGVLNTVK

EAKDFGIEIDGTVKPNFTAIMERKNKVVNQLISGIEFLFEKRGVNLVNGFGKLIDKNTIE

VTKDDGTVETIKADKIILANGSVPVVPRMFPYDGKVVITSDEVLGLEEIPESMLIVGGGV

IGCEIGQFFRALGTEVTIVEMVDQILLNEDKDVAKQLLRQFKKDKIKVITGIGVQTCEVV

DGKAVATLSNGKVIEAQYALVCVGRRPNLDNSGVEDIGIEMERGKVVVNEHLETNVEGIY

AIGDIIDTPFLAHVASKEGIVAVENALGKTKVVDYRAIPRCVYTEPEVAGVGKTEKQLEA

EGVEYNVGQFDFRGLGKAQAIGHFQGFVKVIADKETDKIIGAAVVGPHATDLLTELSLAV

HLGLTVEQVGDAIHPHPSLSEGLMEALHDVHGECVHSVPKL*

>CD630_07240 Clostridioides_difficile_630_NC_009089 bifunctional carbon monoxide dehydrogenase/acetyl-CoA synthase nickel-inserting subunit

MGYNIAVAGKGGTGKTSLTGLLIDYLVKDKKGPVLVVDADANANINEVLGIEVEATIGEI

REEVNQREKLGNAFPGGMTKAQYLQFRLNSIIEEGEGYDLLVMGRSEGEGCYCFVNGILR

EQVNKISGHYKYLVMDNEAGMEHLSRKVTRHVDTLLLVSDCSRRSIQAVARIRDLAEELK

LSVGRILLIVNKVPNGVMNDGVKEEIEKHNLELIGVVPMDELIYEYDSTGIPLVNLPEDS

KSKVAMKEIFAKLELK*

>CD630_07250 Clostridioides_difficile_630_NC_009089 bifunctional carbon monoxide dehydrogenase/synthase complex subunit delta

MAFKMSTQKYSGKISEVEVGIGEKAIKLGGENVLPFYSFDGEVGNSPKIGIQISDVYPES

WTDSYKELYKDVANCPVEWAKYVEANTQADFICLKFDGSDPNGLDKSVDECADVAKAVIE

AIKLPLVVAGSGNHEKDGKLFEKLAQTLDGHNCLFMSAVEDNYKGVGASAGMAYAHKVGA

ESSVDINLAKQLNVLLTQLGVKGENIVMNVGCSAVGYGYEYVASTMDRIRLAAFGQNDKT

LQMPIITPVAFEVGHVKEAIAPIEDEPDWGCPEERTIAMEVSTAASVLVGGSNAVILRHP

KSIETIKELVNALA*

>CD630_07260 Clostridioides_difficile_630_NC_009089 bifunctional carbon monoxide dehydrogenase/synthase complex subunit gamma

MALKALDIFKLTPKKNCKDCGFPTCMAFSMKVASGAVEVGKCPHMSDDAIAKLSEATAPL

MKALKVGAGASEYELGGETVLFRHEKTLVSRNRYAVSFCTCMSDEAVDAKIANMKKVDYV

RIGEQMKVEMAVLEYCGDKDAYLKLIDKIKGSGLEVAYILACDDAQVVKEAVEVLKDARP

MVYGATKENYKDMIEVVKGASLPLGVKAGSLEELYETVELIQAAGYKELVLDVTGENIKD

TYTNAIQVRRTALKEQDRTFGYPSIVFANRLSNSNPMMEVALSSIFTIKYGSIIVIDDIS

YAKALPLFALRQNIYTDPQRPMRVEPKIYPINNPDENSPVLVTVDFALTYFIVAGDIERS

KVPVWLVIPDAGGYSVLTSWAAGKFGGNSISAFIKESKVEEVTNCKDLIIPGKVAVLKGD

IEDNLPGWNVVIGPEESMELPKFLKGYQEKACQTN*

>CD630_07270 Clostridioides_difficile_630_NC_009089 bifunctional carbon monoxide dehydrogenase/acetyl-CoA synthase methyltransferase subunit

MEKFMIIGERIHCISPSIRKALAERDPAPILKRAKEQLEAGAHYIDFNIGPAERDGEEIM

TWGIKLLQSEFNNVPIALDTANKKAIEAGLKVYDRTNAKPIINSADAGSRFDLIDIAAEY

EAMVIGLCAKEGIPRDNDERMAYCQEILEKGLMLGMEPTDILFDPLCLVIKGMQEKQVEV

LEAIKMMTEMGLLTTGGLSNVSNGCPKHVRPVLDSAFLAMAMANGFSSAIMNPCDPELMK

TVKSCDIINGASLYADSFLELNEGGFAF*

>CD630_07280 Clostridioides_difficile_630_NC_009089 bifunctional carbon monoxide dehydrogenase/acetyl-CoA synthase complex subunit alpha/beta

MNLYNIIFTGSEQALGAAQAMLAEAIEKNGKEHKVAFPDTAYSLPCIYAATGQKMNTLGD

LEGALEVVKSLINRTHLLEHAFNAGLATALAAEVIEALKYSTMDAPYSEPCAGHITDPII

RSLGVPLVTGDIPGVAVVLGECPDAESAAKVIKDYQSKGLLTFLVGKVIDQAIEAGVKMG

LELRVIPLGYDVTSVIHVVSVAVRAALIFGGLTPGDLNGLLEYTANRVPAFVNAFGPLSE

LVVSAGAGAIALGFPVITDQTVLEVPMNLLTQKDYDKIVATSLEARGIKIKVTEIPIPVS

FAAAFEGERIRKSDMFAEFGGNRTEAWELVVKKEATEVEDHKIEIIGPNIDEVDADGVLR

LPLAVIVKIAGKNMQEDFEPVLERRFHYFLNYIEGVMHVGQRDMAWVRISKDAFDKGFRL

EHIGEVLYAKMLDEFESVVDKCEITIITDAEKVSELKGEAIAKYNARDERLASLVDESVD

TFYSCNLCQSFAPAHVCVVTPERLGLCGAVSWLDAKATKELDPTGPCQPIEKGECLDDRT

GVWNSVNETVNQISQGAVESVTLYSILEDPMTSCGCFECICGIMPEANGFVVVNREFASV

TPVGMTFGELASMTGGGVQTPGFMGHGRHFISSKKFAYAEGGPERIVWMPKELKDYVADK

LNATVKEMTGIENFCDMVCDETIADDSEGVLAFLEEKGHPALAMESVM*

>CD630_07290 Clostridioides_difficile_630_NC_009089 glycine cleavage system protein H

MKLLPELKYSKDHEWVKVIDGDVVYIGITDYAQDQLGEILFVETPEVEDTVTKGVDFGVV

ESSKVASDLISPVNGEVLEVNEKLEDEPECINEDPYENWILKVKLADVAELDTLLSDKEY

EAGLE*

>CD630_07300 Clostridioides_difficile_630_NC_009089 iron-sulfur protein

MIKVSFTPNNKEVYCNEGDILLEVARNADIFIDAPCNGNVSCGKCKVKLLNGKVDTEKTR

HITDDEWEQGYILACCTKVISDIEIEVPSKVSSSMHGMKIEGSNKKEDREIFERAKKIIE

EHNLQFKTNIKKKYIEMEEPNLDDNISDVDRLERYVRNNLGYNEIDFRLDILRKMPTVFR

KSDFKVTITYVQKQKKLTIINIEQGNKENSLYGVAIDIGTTSVVVCLVDLYSKEVVDKAS

SGNAQIKYGADVINRIIYSTKKNGLETLHKAIVEETINPLLKTIYERNGIDKEDVVTLVA

AGNTTMTSLFLGVYTDFLRQEPYIPPFLKSPKLMGENVGLFVNDSAYVYLAPSVASYVGG

DITAGVLSAGIWSSEENVLFIDLGTNGEIVFGNKDYMMSCACSAGPAFEGGGISCGMRAS

AGAIEKVIIDKDTLEPTLKIIDECAPVGICGSGIIDLICQMITKGVIDRRGKIYRDLDNK

RVRFNEHEIGEYVLAFKEEFDLENDIVVNEVDIDNFIRAKGAIYSGAYTLVDSLGMDFSI

LDRVYIAGGIGNNLDIENSIIIGLLPDIDREKFTYIGNSSLVGSYLALISKDAKNKLEEI

GNQITYVELSVYPSYMDEFISACFLPHTNIEQFPTAKKLLEE*

>CD630_07310 Clostridioides_difficile_630_NC_009089 radical SAM superfamily protein

MNKNLSEKMKIAFDIKLKNFGNKIEFAYPNQTLALSTTSNQCSLKCAHCNGHYLNNMVPI

EEYEEKVQSRNITSFLLSGGCSYEGDVPINTHINTIKNLKEQGYRLNAHLGLMDKDSIVE

LCKYLDIVSFDLVFDDETIREVYKMKKSKEDYIEVYNTIQEHTEVAPHICIGLKGGQIKG

EYEIIEYLQKNPPNKLTFIVLIPTKGTEYENVEPPELEGVADILCEARINLPDTEINLGC

MRPRGVYRKELDQLSIMCGVNRIVLPSRSAKNKAIEMNMTINECKECCVL*

>CD630_07320 Clostridioides_difficile_630_NC_009089 radical SAM superfamily protein

MIRLSVGTAIELGILNKKSDIPPTTAYIMIGEKCINKCSFCSQSIESSTRKDKLSRVIWP

EFSKEEILDALKAYKGKNIKRICIQSMASEEAHNSVLDFINYISGKIDMPISLSAKLEND

EQIKKFFSAGVNKIGIAIDAANKELYEKIKGNNYDEKLKFITEMSKSYPNKISTHIIVGM

GESHEDIYNLYTYLKENDVTISLFAFTPVRGTKMEKINQPSIESYRRVQLMSYMINKGYP

KEYFKFKNGYLNSIKLDNDILKDINKGYPFEIRGCKDCNRPYYNERPGSTIYNYSRPLNQ

SEIDLAIREINL*

>CD630_07330 Clostridioides_difficile_630_NC_009089 biotin/lipoate-protein ligase

MNQWRVIHNKSYEGAMNMAIDEAIFTAYKKGHNKPTLRFYTWEPACLSIGYFQKLEDEID

LDKCRCMNIDYTRRITGGRAVLHDNELTYSIIIGEDNPLIDKSINLSYRYISEGLVKGLN

LSGIETDNLNRGERISRENLSAACFNAHASYEVTINNKKVIGSAQSRKDGVLLQHGSIIL

DFDVEKLFKLIKTKTPELKERAMKFTAKKASGIENEIGRKIDIDILQKNIVKGLAEQFNV

EFVEGDLTDYEKQLVKELYEKYKNEEYNKKR*

>CD630_07340 Clostridioides_difficile_630_NC_009089 hypothetical protein

MCESSAFICKSNNELEKVMENVVNIDPCDGKIYLTDLLGEQKIIDGIIKEIRLMDHKIII

QEN*

>CD630_07350 Clostridioides_difficile_630_NC_009089 hypothetical protein

MIIAVIDGMGGGIGAQIVSSLREELPTYVEIYALGTNSIATSSMMKAHANKGATGENAIV

VSAKKANIIVAPISVIIPNSMMGEVTCNISEAIADSEALKILLPIMPENVELVGLEGKPL

ALLVKDSVNLIKKEFNIK*

>CD630_07360 Clostridioides_difficile_630_NC_009089 hypothetical protein

MIFDKEIVRYHHGHHDHDHCHEHTHGDICHEHPHDHAHDHDHEHSHEESSESKDEKTLKI

LLVHWINHNETHEEGFREWVEKARAIGKEETAKSIEKAIEYMEEANKMLLEAKKHM*

>CD630_07370 Clostridioides_difficile_630_NC_009089 histidinol-phosphatase

MKDGHIHSPYCPHGSKDDFEKYVQRAMDVGITEMTFTEHFPYANGFRDPAPENDSCMSIE

DLPKYFNDVKKLKEKYEGKIKINVGSEVDFIEGYEEGIKENLNKYGKELEDSLLSVHIIK

IDDDYYCVDYSVEEFQNLIDKLGSIEAVYNKYYETLIKAVNSDLGIHKPKRIGHLNLVRK

FNQVFPYDYKGNTILEELIKLIKEKGYELDYNVSGNRKEYCKEPYIDGYLLELVKKYDIP

LVLGSDSHCAEEINKYDII*

>CDIF1296T_00908 Clostridioides_difficile_ATCC_9689__DSM_1296_strain_DSM1296_CP011968 membrane associated lipoprotein

MKKRLIIMILSVVLVLSSILTIFAYSNIKYNNNNKLIYSNMIDKKTQNSVKEILKENKIN

EKDIDTFIKAVNNYNKLQVKILQNNINISKSGYSSINAKQVPYNLEKLQDNWVKKFPDYM

DVNCRITAFRLFKDFINSNKKFTGDSIDLNVDLDTIMNNKDAKFSTKDVEKFINFFSAIP

AKDTDDTIKISEQIKNEWKKRKISFKNNKNISIINGFLRYPETKNVFIGHTGICIKTKNG

ILFLEKYGVTSPYQVTKFKNKEDVKNYMFNRLKMSEGEIELPDPIIMENDKLMK*

>CD630_07390 Clostridioides_difficile_630_NC_009089 hypothetical protein

MKVKKFKSGIVMALTVMAIGASLTNVSAMELNKSKEFETKYNEMKRDFQEEYGKEDKFNI

TLDDEFTKEEVNGAKGIIKTTNKMNNEVVVLNYFDEMKSGIEFELKPKAEKNAILRDEFQ

QGYGKEDKFKVVLDDEFTKEEVNGAKGIIKTTDKETGEVIIYNYFDELEK*

>CD630_07450 Clostridioides_difficile_630_NC_009089 OmpA/MotB proton channel

MFMLYDEDEKEENNERWLLTYSDLITLLMIFFVIMYSMSNVDAEKYKQLSQSLNSAFGGS

SGVIEGGNSKIEPVVEPGSNDLDTLQNAKFKKVGEEIQKYLNENGMANSVSLRVQDRGLV

ISLKDTILFDTGKAIVKDNSRDKIIQIGKMLNEMNSYMRVEGHTDNMSIKNSEFKSNWDL

SVMRATNVVQLLIDNAGIAPDKLSAVGYGEFRPIAENSSEEGRSKNRRVDIVLVDSKYDN

VENVSKDK*

>CD630_07480 Clostridioides_difficile_630_NC_009089 diguanylate kinase signaling protein

MGMENNLRKNNSIMKKMIFPLIIVMLIQTSLFCATILWGGTIKKLNDNSFDILNERVINR

KNYIQNEMLQRWSNTSETEATINSSVKKVLNEKNALIEDVGNNEEINKDIIKSISNDLIY

LLRKNSVTGAFIILSDKNINDKSIDSKGEISRTGLYIRDLDPKFNPNDNSDLLIERGSSD

IARSFGISMDSYWEPKFSFKSENEQDGDDFFYKPFRAAIDNPNVSSSNLGYWGSKFLLTN

AGDRLMTYTVPLIYEDGTVYGVLGIDLSVNYLRELLPYSEINGNKKGNYILGVDRNNDMA

FENVISNGYSLKEKFWDGLHSNLEQKESYKNIYKIKNKDNECGNGNIYASVQYLDLYNSN

TPFENERWALLGIIEEKSLLSFSKRVEKLVIISIAISLLIGLIGIYIASRLFTKPIVLLA

NKVRNSNPSKPVNLDKINISEIDELSSAIELLSANVADSSSKLSQIIEMVDMPIGAFELY

KNAENVFCTKEFFSVFTTEIKFEEYGYMEKNKFLKKLRGLDKYLQESYETENTYIYKFES

KDKKLKWIRMNIFEDELKVLGVVVDVTKEFIEKRKIEYERDFDILTNLLNRRAFHSQIQE

KFKNKDELKIAAFIMWDLDNLKYINDTYGHDAGDEYIRCAADILKKFISWNGIVSRLSGD

EFYVFIYGYESKDSIRDIINSVKKQMNNTLLKLHDGTEFKIRASAGISWYPDDSTNYDEL

MRYADFAMYQIKNTIKGEVSEFDINIYNKDSFLLSNREELNKLIDGQLVEYAFQPIVDCK

GKVFAYEALMRPKIKTFSSPIDVIRLARSQSKLYQIERITWFKALKSFKKYKEKFGDCKL

FLNSIPNNNLSKVDFEEIEDLYGEYLNRVVLEITENDKINEDFIEKKKAYIDHWNIEIAL

DDFGTGYNGDAVLLYIMPDYVKIDMSIVRGIDKDENRQKILKNLISYSNERNIKVIAEGV

ETKGEMETLINLGVDYMQGYYISKPSFIPEDISDKIKKELKICHENRNDVV*

>CD630_07490 Clostridioides_difficile_630_NC_009089 UvrD/REP type DNA helicase

VVELIITTNIDIRKLNENQLEAVEHIDGPCMILAGPGSGKTRVITYRIANMVVNKNIAPT

RILAISFTKASSIEMKNRALNLSDDIRLNKVTYGTFHSVFFKILRYFERYNLDSIFDEKS

KRMTIKAILKSLNIENADDDENIGQVINEISYVKNELMDKNEFNSEVLTKDEFLKVYNLY

EEQKSKVNKIDFDDMLIRTYYLLLNNKSALEMVRNVYKYILIDEFQDINKVQFEVLKLIC

SPLNNIFAVGDEDQSIYGFRGARPDFLLEFEQYFNNTKKIILDINYRSKSEIVHTANRLI

DKNKNRYEKIIKCSQGDGGSVTYISPHDSEEEALYIAREIIDEIQKDYVEYSDFAVIYRT

NIQSRALVDVFMDMRIPFVVKDSVITIYDHWASQDILAYLRIGINPKSNKDWLRIINKPF

RYISKDSINMVKDEKDFITALINKCNLHPKQVKTINDLEIDLSYLNTLNPKNAISYIRTS

LDYDRYVLDYCSNRKIKTNGLVEILNELESSATNFNTVTEFLEHIDRVKTELTENNKNKQ

TEGVIFTTMHSAKGLEFRNVYIIGVNEGTIPHEKSYDICKEEKKEEQLEEERRLMYVAIT

RAEEKLCISSTLNKYGKKVDKSLFINDIKSPTKKEIDSIDVGDKVYHKKFHEGEIIKKDG

IMFTIKFKDRERILDLKTCLLKNIIYTI*

>CD630_07500 Clostridioides_difficile_630_NC_009089 amino acid family ABC transporter substrate-binding protein

MKVFKKLLSLGLVLGLTLSLVGCSGGGEKTKLEQIKDNGKLVVGTSAEFPPFEFHKVVDG

KDSIKGFDIMLAEEFAKELGVKVEIKDMSFDGLIGALNADQVDIVLAGMSPTPEREKSVD

FSELYYLSRNAVIVKDADIDKVKTEDDLKKLRVGVQAGSIQEEYVVNTLKMTTTKSLKAI

PDLITELKNGNIDAVVTNEAVSLINVKKYDGIKMANTEVGKDVTEGMAAAIKKSDNNKDF

IELLNKKIKELQDGKKIEEFLNEASTEAASN*

>CD630_07510 Clostridioides_difficile_630_NC_009089 amino acid family ABC transporter permease

MSLDFSFLSRFGTSFLEGTGVTVSISLVALCFGFIIGIIICMAKISKSKVLRAISSIYIE

VLRGTPLLVQIYIVWFGLPQLGIRFPMLFGIPSEFIASAFALSVNSGAYVAEILRSGIQS

VDNGQMEASRSLGLNYWSTMRYIIIPQAIKNILPSLANEFITLVKESSIISVIGVVEIMR

TADIVKNAAFRALEPLIVAAAIYFVITFTLSRLVGLLEKKLSVSN*

>CD630_07520 Clostridioides_difficile_630_NC_009089 amino acid family ABC transporter ATP-binding protein

MITIKNLSKSFGDLNVLKNIDLEIAKGEIMVIVGPSGSGKSTFLRCMNLLEIPTGGEIIF

EGKNLVDKKTNIDEVRQNIGMVFQNFNLFPHKTILDNITLAPIKLKKMTKEEAEKKAEIL

LSRVGLLDKKDSYPSQLSGGQKQRIAIARALAMEPDMMLFDEPTSALDPEMVNEVLDVIK

ELAKEGMTMAIVTHEMGFAKEVADRVIFIDGGSILEDNTPEEVFGNPKHERTKAFLAKVL

*

>CD630_07530 Clostridioides_difficile_630_NC_009089 cysteine desulfurase

LEIYLDNSATTKPYQEVIDKMVYALNTEYGNPSSVHRKGVEVEKAIKEVRQDIAKSLGAK

EKEIYFTSGGTECNNTIIRGITSLNKKRKNHIISTNIEHPSVLNTLKDLEEDGFEVTYLE

VGKDGKINIEDLKNAIKSTTCLVSMMHVNNEIGTIQPIGEVGKYLKGLKEKIYFHVDAIQ

SYGKINFRPSKYNIDFMSVSAHKFHGPKGIGFMYIKENNRLKPMLTGGGQEIGIRSGTEN

VPGIYGLGEAVRILNKDLDAVISKVDNLKNILKNEIIDNIEDIKINSPEDGVCHILNVSF

RGTKGEVLLHYLEQKGIYVSTGSACSSKKKGSYVLNAIGLTNEEINGTIRFSLSDMNTEE

EMLEAVKVLKESICDLRSIMKRK*

>CD630_07550 Clostridioides_difficile_630_NC_009089 cell surface protein

MNKKGFTLIELLVVISIIGILVIVAIPALFRNIEKSKAVTCLSNRENIKTQIVIAMAEES

SKGKNEVMKEVLENKDGKYFETEPKCKSGGIYSATFDDGYDGITGIESIAKVYVTCTKHP

DGVEMARDIHQSMKDLIASFSQDPSIIPGASKGNDDFRKYLLDNKYKNGWPTIPDEFKAK

YGLSKDTLYIQPYAYSPTKSDATVVVFANNKTGGNWYTSLVYDYDEGRWYKGKNGISVAG

RSWDVDTDSVKSVKTEIHSKEGWGPLN*

>CD630_07560 Clostridioides_difficile_630_NC_009089 nitroreductase

MNNNFQDNQTINLIQSRRSIRKFTTEQISDEQVNTLLHCAFAAPSGCNKQPWHITVVQDQ

KLLKEISDDTLSRIHEVSNVEINKNFKLFYGAPTVLFISYDESSSWAPYDIGILTGNITT

AAQALGLGSCIIGMVRGLFTPVEQGDIEGLVSVLDKEDVKESESIKMKFDTNKKYRELLD

IPEGYSVPFGIAVGIPDGNLPNAREVVYKVSRV*

>CD630_07580 Clostridioides_difficile_630_NC_009089 pyruvate formate-lyase activating enzyme

MIKGKIHSIETFGTVDGPGIRYILFFQGCPLRCKYCHNRDTWDIKSGKEYTVDEIITDAL

KYTSFMKFSGGGITASGGESTLQPEFLSELFKKAKENDIHTCLDTSGFVDIETIDPVLDN

TDLVLLDLKHMVEEKSIDLTGVGMDKALKLARHLESRNIPVWIRHVLVPGITDDIDNLEK

LGQFVATLKNVERFELLPYHSMGIHKWESLGIDYELKDVPDATKEDIQKASEIISKFGVK

VYNS*

>CD630_07590 Clostridioides_difficile_630_NC_009089 pyruvate formate-lyase

MNAWQGFKTGRWTKEINVREFIQLNYSPYEGNDSFLAGATENTKKLWDEAMVLFKKEREN

GGTLDVDTKTVSGIAAYAPGYLDKELETIVGLQTDAPLKRAVMPYGGIKMVENSCEAFGY

ELDPEIKDIFTKYRKTHNQGVFDVYTPEMRAARKSGIITGLPDAYGRGRIIGDYRRVALY

GVDALIEDKNEQKKSLEVSCMDEEVIRLREEITEQISALNELKKMAESYGFDISKPATNS

KEAVQWLYFGYLGAVKDQNGAAMSLGRTSTFLDIYFERDLKAGIVTEEELQEYMDHFVMK

LRMVKFLRTPDYNNLFSGDPTWVTECIGGMGIDGRTLVTKNSFRMLNTLYTLGPSPEPNL

TVLWSTKLPQGFKDFCSKVSIDTSSVQYENDDLMRAYWGDDYGIACCVSAMRIGKQMQFF

GARVNLAKTLLYAINGGVDEKSGVQVGPRFEPITSEYLDYDEVMSKFEPFTDWLATLYVN

TLNVIHYMHDKYSYEALEMALHDRDIFRTMACGMAGLSVCADSLSAIKHAKVKTIRNEQG

IAVDFEIEGDYPKYGNNDDRVDSIAVELVESFMNKIRKNKTYRNSYPTQSILTITSNVVY

GKKTGNTPDGRRAGAPFAPGANPMHGRDTNGALASLSSVAKLPYEHAQDGISNTFSIVPA

ALGKDMTERINNLSAMMDGYFAQNAHHLNVNVFDRATLEDAMEHPEEYPQLTIRVSGYAV

NFIKLTKEQQLDVINRTFHGKMA*

>CD630_07610 Clostridioides_difficile_630_NC_009089 ATP-dependent RNA helicase

MNITKFEDLPISEGIKKAIAEMGFEEPSPIQAQSIPAILSGKDVIGQAQTGTGKTAAFSI

PILETIDPNNRSLQAVVLCPTRELAIQVSTEIRKLAKYSHGIKTLPIYGGQPIDRQIKSL

KSGVQVVIGTPGRTIDHINRKTLKMDNVKMIILDEADEMLDMGFREDIEMILSKIPEERQ

TTFFSATMPRGILELTKRYQKDPEHIKVVRKELTVSNTKQYYIETRSSNKLEVLCRLVDV

YDPKLSVVFCNTKRKADELVGDLQARGYFADALHGDLKQTQRDIVMDKFRNGTIDILVAT

DVAARGIDVDDVECVFNYDLPQDEEYYVHRIGRTGRAGREGMSFTFVFGKEMRKMKDIER

YTKSKLIKHNIPTITDVEEKKVGTFFAQVKQTIEEGHLTKQLQWLEGFCNDEDYAMVDIA

AALVKLSLGEEMKEEIIEEKPRRERGDRKGGTGAKDGMIRLFINIGRNQRVQAKDIVGAI

AGEVGIPGKVVGTIDIYDKYTFVEIPKKDAKTVIEKMKDIKIKGNKINIEKANKKKK*

>CD630_07700 Clostridioides_difficile_630_NC_009089 anti-sigma F factor antagonist

MVNYSLEHKNLYIEFMCSELDHHVANEIREEIDNLLSVNQVKNVVFNFENINFMDSSGIG

VIIGRYKKISNEGGRVSVINISSRVKKIFDLSGLNKIIGIYDTYEEALSSL*

>CD630_07710 Clostridioides_difficile_630_NC_009089 anti-sigma F factor

MNNIMEVKFSAISENESLARVIVASFAAKLDPTVDELVDIKTAVSEAVTNAIIHGYEEDS

SKFVFLRCEIEGNTIKIIVEDEGYGIENVEKAMEPLYTSKPELDRSGMGFTVMKSFMDDV

EVSSVKDNGTRIEMTKKINVPK*

>CD630_07720 Clostridioides_difficile_630_NC_009089 RNA polymerase sigma factor SigF

MEVTVAREEKKPLLSHEETLELIEKVQNGDEEAKEILISSNLGLVRSVVSRFLNIGYDRE

DLFQLGSIGLIKSIYKFDPKFNVKFSTYAVPMILGEIKRYLRDDGMIKVSRSLKQIAIKA

KMESEALTKKLGREPSIEELAKAIDVEKEDLVMAMEANFNVEYLQGVIHEEEGSPICLID

KISMKGESEEEKVVDNILLKDILGRLDKRERQIIVLRYFEDMTQSEIGEMLNISQVQVSR

IEKKVLSKLKEYIS*

>TW87_RS06500 Clostridioides_difficile_strain_08ACD0030_NZ_CP010888 stage V sporulation protein AC

MDKNYKKYVDQISPKPTYLKNYTLAFIVGGIICMIGQAINDLYMKVGGLDKLGASSATSI

TLIFIGAFLTGLGVYDLIGKRAGAGSIIPITGFANSIVSPAMEYKREGYVLGVGANLFKI

AGPVLVYGIGSSILCGIIYYILRCFR*

>CD630_07740 Clostridioides_difficile_630_NC_009089 stage V sporulation protein AD

MKNKRIGKRTVKLENKPTIISTGTIVGPKEGEGPLKDYFDMIMTDDLYGEKTWELAESKM

VETASQQAIQKAGKKLSDVNYMLGGDLINQIVPASFAARELAIPFLGIYGACSTMAEGLC

IGSMLIDGGFADLVLSGTSSHYCTAERQFRFPLELGNQKPMTAQWTVTGAGSVLLAPNGD

GPKVKYVTVGKVIDEGIDDGNNMGPAMAPAAIDTIYSYFEDTNDDPNSFDIIATGDLGTL

GKQIAEDFLKEKGVDISKVYTDCGIEMFNLKEQDVHCGGSGCGCSATVFAGYIYDKLRKK

EFNKVMLVSTGALLSPTSTLQKQTIPCVAHAVVIVNE*

>CD630_07770 Clostridioides_difficile_630_NC_009089 membrane protein

MKEKAGIRNAIIAAFIITILAELIGPISFKVMGINVTLLSILWAIFIGMAVSPHLLGRVI

PALKKFIGDNEINVSPYLLSLTLYPLGIMFGINAGPKVGILLQAGPALLLQEFGHMGTML

IALPVGVALGLGRSALGGTFSLCRDTALGIIGDKYGLNSPEGIGTLGTYISGSIFGTLLF

SFLAPVGVLLGFHPYALAMASGMGSGSMMGAATASLMNTVPQMSEQILAYSATSGLITAV

TGIYIELFLSLPVANYYYSKVAPVIERFRGRKTRRTSNKL*

>CDIF1296T_00947 Clostridioides_difficile_ATCC_9689__DSM_1296_strain_DSM1296_CP011968 hypothetical protein

MEKTFKTIMEQIKIVLFIYIIICFGQMIALKTQAQDLLIGSVSGFLLVIIAIIIKNFVKK

PNLPGFAWATLVAFVLSLPISPVGDIIATNVGKINFMSTVTPLLAFAGISVGNQLDILKT

LSWKLVVISFIVMTSTYFGSAFISQTVLRLNGML*

>CD630_07790 Clostridioides_difficile_630_NC_009089 amidohydrolase

MNELKAKIKEEIKLLSEEKKTSFEKVSDYLFSNPELAFEEYKSQKALCDLLEENEFNVTK

GVGGLETSFEAVYSNGTNGKTVAFLAEYDALPGMGHACGHNIIGTSSVGAGIILKEIMKK

HNIEGTVKVFGTPAEERVGGKITMIKEGVFNNVDAALILHPSDASMPDDISFAQVNLKFD

FTGKASHAAAFPWEGKSALSGVIALFNSVNSMRLHLKDYARVHGIITDGGSIHNIIPEKS

TAIFNVRALSIEYLNEICEMLKNCAKGAAISTGTNVEIVQLDEIYKEIKNDSELVNIVRQ

NFEVLGEDYVERDLSQGIGSTDTGNLTHEIPAIQAYIKLKENTATHTDEFAVAAGGEEGR

VALIKAIKVLAMCGVDILYSK*

>CD630_07800 Clostridioides_difficile_630_NC_009089 hypothetical protein

MILKQVIEVYDILDKANANGEEVKTYLQGYGEVDVTVKELSSSKGSTDLVKLTIPGKNGK

LKGGDAPTLGILGRLGGIGARPEVIGFVSDGDGALVAIAVAAKLLDMQRKGDILDGDVVI

STHICPDAPTKEHYPTPFMDSPIDMMTMNENEVDSSCDAILSIDTTKGNRVINTRGFAIS

PTVKEGYILKTSDNLLDIMQTVTGKSPFVFPLSIQDITPYGNNLYHLNSILQPAVATDAP

IVGVAITTEMPVAGCATGATNFSDLDSAGRFAIEVAKLFGRNKCDFYDKEEWEMLIKRYG

KLNRFQTFGIQ*

>CD630_07820 Clostridioides_difficile_630_NC_009089 sporulation protein YunB

LNRIVDRKTKNEFRKIIAIFLVILFLSVFIGSFIYIDKTLRPTITVLAETKALELANRSI

NKAVAEMVEGKINYEDLMDIQLDNNGKITMIQANTIMMNEIASAIALEIQDELKKDKTAS

SYIPIGTALGSPILAKYGPKLEVSIEPIGTVSVNFKTEFESSGINQTRHRIYLEAQTQVK

VVIPLITSTKQIKAQIPICETIIVGDVPESYVNIPEKNLGNVLPNTGKNTNK*

>CD630_07840 Clostridioides_difficile_630_NC_009089 N-acetylmuramoyl-L-alanine amidase

MKKYRLLVTCIIAMGIIIIGTSNPVSNFYKTTNLISSGNITNEKADKDENSNKNNKDKNE

DKSNSKSNKDKSSEDKSSDNKNEIQKNKKLLICIDPGHQGKGDSNLEPVAPGSSSKKARV

SSGTEGIATKKPEYVLNLEASLVLKSILESKGYNVIMTRETHDVNISNSERAILANDKKA

DMVVRIHADSLNNSSKTGASILIPEKDGKYTAPIYEESNKCAEFIKQNMEQSGIQINGIV

QRGDLTGFNWSKVPAVLVEMGFMSNYNEDKMMSNPDYQRKMMQCIADGLDAYFK*

>CD630_07850 Clostridioides_difficile_630_NC_009089 ABC transporter ATP-binding protein

MLVVENVSHGFGARTILENVSFRLRKGEHIALVGANGEGKSSFLNIITKKLMPDAGNIKW

SSRATVGYLDQHTVLSKGKTIREVLREAFKHMFDLEQEMIAMYDKMGEASDDEMSKLLEE

TAEIQTILENSGFYMIDAKIQEVANGLGLGEIGLDKDVTDLSGGQRTKVLLTKLLLENPT

ILILDEPTNYLDEEHITWLTKYLQEYENSFVLVSHDIEFINNTCNVIYHMENGELNRYKG

NYDEFVRLNDIKKRQEEQAYDKQVEERKRLEDFVARNKARVATRGMANSRQKQLDKMEIL

ERPKEKIKPTFAFKDARAASKIIFETENLVLGYDEALTKPLNFHLERGKKIALKGMNGIG

KSTLLKTLLGIIKPFEGNVKLGDYLEVGYFEQESSRENSNTPMDEVWSEFPGLTNFEVRQ

ALAKCGLTNEHITSQMRVLSGGEAAKVRLCKVMLKNINFLVLDEPTNHLDVEAKDELKKA

IKEFKGTVLLVCHEPEFYSEIVDDVWNIEDFTTKIV*

>CD630_07870 Clostridioides_difficile_630_NC_009089 metal-binding protein

MGENYKFFNHKDCEFFPCHKTNKPEEFNCLFCYCPLYALGENCGGNFKYTDKGIKDCSSC

ILPHKKDNYNYIMSKFQDLVKITSKK*

>CD630_07890 Clostridioides_difficile_630_NC_009089 hypothetical protein

MDINWENIDNLEDYFITYLLYKESKTVSQISKIRNISSTEASEQLIQAKLKIKEMQKDDF

EASKDILDKFLELDKIKRLDFMDSLDDEKMVYFKRKVFKRILVEKNAEDLIVLIWATGEL

KDDRFLKLLHQLTNHRHSDIRRITYSAIRKIESPSSREVLQKGLYDKNAQTRQYCAKALS

KLGDENSLKILQQLKDKNKNFEKEYVLRAYDEAIKSLENAKNLKK*

>CD630_07900 Clostridioides_difficile_630_NC_009089 NUDIX family hydrolase

MIVRRCAGGVVFYANKVLIVKNDRGEWTLPKGKILGGGLPYESAVQRVKVETGIDAKMID

VAGDTMYEFFSRSRQQEVCNAIMWYVMEACNTDCVLAPEFQEGGFYKVKDALEMLSHHKE

QALVEVSYKKFKELKKLVSEGDDVPAN*

>CD630_07910 Clostridioides_difficile_630_NC_009089 hypothetical protein

MSNYRTLHEFGTDEITIEKSVFIGYAKPIKSEEEALEFINEIKKKHKDATHNVWAYTVGK

NMNIQRYSDDGEPQGTAGIPTLEVIKKEDLRDVAVVVTRYFGGTKLGAGGLVRAYTKGAK

LGLEAGKIIYKVMYQEVKVKIDYTQLGKVQNELMNLGYFIKDTVYEDNVEIVVYSRLEDV

EKLSEKMIDITSGTGKIVLGEEFYLSEQNGEILI*

>CD630_07930 Clostridioides_difficile_630_NC_009089 hypothetical protein

MKKTDDLKKKYFDFNLKNTIIGVFTGFINGVFGSGGGTLLVPILNDIVKVEEHKSHATAL

SIIIFLTTASSVLYVSKGTYDVNLTIKVAVGSILGGIIGAKLLNKVTGKFLRISFGIIMI

IAALRMVF*

>CD630_07940 Clostridioides_difficile_630_NC_009089 NH3-dependent NAD(+) synthetase

MSNIKIQIDKTVEWLINKVNEANAKGLIVGVSGGIDSAVVANLIKKAFPENSMGVIMSIK

SNPQDREDALKVIEGCDIEYLDLDLIEPQSAILDMVVGNLKDKHLYREEYLKMTDANLRA

RVRMSTIYTIANNLGYLVVGTDNAAEIHTGYFTKFGDGGVDILPIANLTKGEVYEWAKEL

GVHEDLINKAPSAGLWEGQTDEDEMGTTYNMIDAVLEGRLDEVPKRDQEIIERLHRLSEH

KRKTPAQPPKF*

>CD630_07950 Clostridioides_difficile_630_NC_009089 DNA-binding regulatory protein

MGRIGNIINRKGKQDAQRAKIFTKHARAIAVAAKEGGADPEYNAALKTAIEKAKADNMPN

DNIDRAIAKGAGAGAGEDYETIVYEGYGPGGVAVIVETLTDNKNRTAGNVRYYFDKNGGN

LGTSGCVSFMFDKKGQILVGLGDGVSEEELMDVALEAGAEDFITEEDGYEIITTPEDFSS

VRDELKAKGYEFISADVKMIPQTTTVLTEESHLKMMNKLVDMLEEDDDVQDIYHNWEVE*

>CDIF1296T_00969 Clostridioides_difficile_ATCC_9689__DSM_1296_strain_DSM1296_CP011968 3-hydroxybutyryl-CoA dehydratase

MESVMENLNNLKVELKDKVCVITINRPKALNALNSDTLRELSQVIDVVSENEAILGVIIT

GEGKVFVAGADIRQMQNYKSEEGRKYAGYAQGIFDKIEALEKTVIAAVNGYALGGGCELA

MSCDIRIASEKAIFGQPEVNLGVIPCFGGTQRLSRLVGTGIAKELIFTGRQVNAEEAKSI

GLINKVVPSDLLLEESMKMMNQIVEKAPIAIRYAKVVINKGIDMDLKNALELEKDIAGLT

FATRDKQEGMNAFIEKRKPVFENK*

>CD630_08020 Clostridioides_difficile_630_NC_009089 CoA-transferase

LKVLNKPLEGVKVIDLTYFVAGPGTSKILADWGADVIKVEPSFGDPGRKTGATMTMPIDD

YNNPFYSTYNSNKRGLSINLKSETGIEIMDKLLSEANVFVSSYRTGALKRLGLDYESLSK

KHPHLIWGQINGFGDFGPAKDNAGFDTVAFWARSGAMLDIAEKDTSPINPPIAFGDATTS

CSLSGGICAALYQQAKTGKGQKVMASLFGQAIWNASSLLASTQFSDEYPKTRKNAISPVI

NSYRCKDGKWIFLSILEHERYFEALCRLFGREDLIKEEKFASSVESKKNATELINILDGE

FAKFTQNEMVEKLTAADIAHEKIQHVNDVVTDEQAIANNYIYEHTSKNGNKTMLASTPVK

FGNIEVNMTCDAPLIGEHSDEILRELGYSDSDIEALIESGIVTMNKEACEIC*

>CD630_08030 Clostridioides_difficile_630_NC_009089 acyl-CoA dehydrogenase

MDFRLTEAQLMLQRVAKEFAENEIAPIAAETDKTGIFPRELFSKMAKIGFNGIGTPVEYS

GSGGADIEKVIVVTEIAKKCAASAAILSIHTIYAQAILKFGTEEQKKKYLPMMAEGGCVG

AFALTEPNAGSDAARAATTAIIDEETDEYVLNGTKCFISGGGQAESLIIFALTDPSKGIK

GMSAIIVDKGTPGFSIGKIEEKMGIHGSETAELIFDNCRVPKSNLLGKEGKGFNIAMTCL

DGARIGVGAQAVGIAEGALEESIKYSKERVQFGKPISALQGIQWYIADMATMVESAKLLV

YYAADLKARGEKHTKEAAMAKYNASRTAREVTNLALQIHGGYGYMKDYPLERMYRDAKIT

EIYEGTSEIHKVVISRAVLG*

>CD630_08040 Clostridioides_difficile_630_NC_009089 electron transfer flavoprotein subunit beta

MNIVVCLKQVPDTNEVKINKETGTLIRDGVPSIINPDDRNALEEALKMKDELGAVIKVIS

MGPPQAKSALKEALAMGADEAYLISDRAFGGSDTWATSTIIAAAIEKVGKYDVIFCGRQA

IDGDTAQVGPEVAEFLGIPQVTYAKEVKVQDDKLLVTRYTETGDYLIEAKMPVLLTAIKE

LNNPRYPSVKGILEAYNNGDAKITVLTLADLDVDTTQIGLKGSPTNVYKSFVPVKDKHNE

IIEGINKKEKAEKLIEILFDLKLV*

>CD630_08050 Clostridioides_difficile_630_NC_009089 electron transfer flavoprotein subunit alpha

MMRAKVNQGINLNDYNGVWVIGEQREGKINPVTIELIGEGRKLADQLGKELAVVIAGYEV

EKEVKELLHYSVDKVYYINDPLLKDFTTDGYAISIANLIERKKPEVVLVGATSIGRDIAP

RIAGKVGTGLTADCTKLEIDSTDNKLLQTRPAFGGNLMATIVCPKNRPQMSTVRPGVMAK

AVRNESETGILEVVTPELTEKMIRTRLVEILPQEKKSVNLTDARIIVSGGRGLKRAEGFE

LIKELADKLGAEIGASRAAVDSGWIEHSHQVGQTGTTVRPELYIACGISGAIQHLAGMSD

SKYIVAINKDAKAPIFSICDYGIVGDLYEIIPEMIESLNR*

>CD630_08060 Clostridioides_difficile_630_NC_009089 sigma-54 dependent transcriptional regulator

MKEQWYKDIFARVLSMTDDGFIVVNTSGVIIDINDKYCDFLGKERKDIIGQNIQSIIPNT

KMLDVMKNKYCEEGAIHHYSGGNTKEKSVIVSRSYVENDNGEVVAGVAQVKFRLQSFDVA

KKLMSEYMELQYYKEQFKDNCGFDKLIGENRDFIELKKTGVKASKTNFPVLLTGETGTGK

EVFARAIHNNSSRSDKPMVSINCAAIPEELLESELFGYDEGAFTGAKKGGKKGKFLVANN

GTIFLDEIGDMPLTMQAKLLRVLQESEIEPVGGLKTIKIDVRVISATRKNLSKMVEEGLF

REDLYYRLNVINIHMMELKDRQDDILLLANYILNKLNVEYKELKVLSDKVKNCFINYTWP

GNIRELQNVIKSAYAVSDDMVIMMCDLPSKMDNISRVAQCNVDSNCSIHEMVENYEKSLI

IDVLRKYNWKCSKAAEVMGIHKSLLYKKIKKYEIELNN*

>CD630_08070 Clostridioides_difficile_630_NC_009089 hypothetical protein

MDQIKRLHELQQKSYLTKEEFEELKNNTYIERFELRDECDNSYIYTFYTNDKCNDIKFIK

SQFIVTLIKIGFLCECGGVFRQKKIVDEYDYGYNAIYKCDSCGKELDKEVENYD*

>CD630_08090 Clostridioides_difficile_630_NC_009089 hypothetical protein

MHEDKNDIFESIDTEDFDNKTEEECLELVRKNGLNLIYIKNQTEEICLEAVRQNCNAIKC

VQNQTEKICIEAVKQDWRMLEFVEEPTEEICMLAINQDGTALKYIENQTEELCLRAVEKN

GAALEYVKEQTEEICIEAVRNSEFGLARVKNQTEKICMEAVKHCSYNLKWVENQTEEICM

EAVRQNGLDLKFVKNQTETICLRAVRQNGMALEFVKEQTVGICLKAVRQYGMALKFVKEQ

TEEICTEAIKQDKRALSFVKGDKEKYKALYDNNEPFAKRYVRNVIEKENRALIKKGEENA

KIKIAGKLYDRGMSFEDISDIVEIEISKLKVSLGVF*

>CD630_08100 Clostridioides_difficile_630_NC_009089 flavodoxin/nitric oxide synthase

LKITVLYQTRSGNTERVAKLIEEGAKKVDGVDVKLMRLDNIDLDYLNESKAVIFGTPTYL

ANTTWEVKKWFDEDSKKVNLAGKLGAVFATCDYICGGPDVAILTIVGHLMVKGMLVYSGG

GSLGKPFIHLGHVHSIEGPELQDEKAIIFGERIATKAKELFA*

>CD630_08120 Clostridioides_difficile_630_NC_009089 universal stress protein A

MNKKKILVPIDGTERSMHSLEFIKGIFKKDEVEVEIMNVKELVFIDGISLAEEIKNSENL

GRRILDKAAEIMGEYDVKVHFTFGYPGDEIIRKAKEDNVDFIVMTKSTKKGLTRMIGSVT

ASVVKQAKCIVMIVPE*

>CD630_08130 Clostridioides_difficile_630_NC_009089 PTS system transporter subunit IIB

MNKKIVAVCESERSLNSLKTAANDLGFDLVYEIQENDNIKNELSIYDIEDASIVLFVTSD

SIESIEKIDRFIDREFYEVDPKYIIEDAKSIVSEILIDLN*

>CDM120_RS04760 Clostridioides_difficile_M120_NC_017174 GntR family transcriptional regulator

MKSLNATEKIIDYIKLNILNGTFKINSKLPSERKIADLFNISRIPVRNAINILCKEGILR

AVPYSSPIVEGFKKIDLFDDGEIYKNHNIQEFYVESLRARQLIESEATRLAILNATSKEI

QKIKYTYLKSIEELDKVSQGLIEECYDADLQFHKEIILASHNPIFIRYYELIPKTISSNQ

YFGFKYRNSLKDMISHHNNIIIAFDSKDSNLGYTSMYNHLEDVIQLFQHDD*

>CDIF1296T_00984 Clostridioides_difficile_ATCC_9689__DSM_1296_strain_DSM1296_CP011968 PTS system transporter subunit IIABC

MDYKNIAQEILLNVGGKENVNEVTHCMTRLRFKVKSASKVNKDKLSKTEGVITVVESMGQ

IQVVIGNKVKKVYDEVIKIVPQSNNVGKKNESQENQGIINSILSAVAGIFTPTIPAIAGV

GMIKGILSVLAMYYMNKNGVDIKETQSYIILNAMADSIFYFMPIILGYTAAKVFNANKII

SMVLGATLCYPTFTALMAGEESVRFLGLAVTKATYTSSVIPIIIAIWALSYVEKVLEKYI

PEIIKIIMVPTLSLVVMLPATLFLFGPIGIYIGNVINFSYKYIYELSPALCGAFVGGLWC

VLVIFGAHRALLPIGISDVAQTGRQNLLAFAGAANFSQAGAALGVFFKTKNQGLKTISMS

ATITALFGITEPAIYGANLRLKKPMVCAVICGAFGGAIMGMGGAYGNAFANQGVLTIPVY

AEAGALGFLSYLGGCAIAFFGSAISTYLVGFEDLEESSNKENSSIKVETKDGVIDITSPV

EGECIELSEVKDDVFASKAMGEGIAVLPTKGVITAPTDCEVASLFPTLHAIGLKLDNGAE

MLIHVGINTVELNGKYFTKHVNQGDLVKKGDKLISFDIDKIKKAGYDVTTPVIVNNTFDF

GQVVSCKSSYVSTNDNIISLVLAGN*

>CD630_08180 Clostridioides_difficile_630_NC_009089 6-phospho-beta-glucosidase

MNTGFPKDFLWGASSSAFQVEGAWDKDNKGKTVADYNSFKKSHLQADTKVASDFYHNYEE

DIELMKELGMKTYRFSISWARIIPDGEGEINQKGLDFYNKIIDKLIECDIEPFVTLYHFD

LPFKLVEKYNGWESRETVYAFERFAKICFKHFGDRVKYWQPHNEQNLIVRVEERINIYDE

TDSWKIDKIRAQMDYNLCLAHALAVNACHEMIKESKIGAAVSSSVTYPLTSKPEDVYAAR

MNDNFKVYYMLDMHHYGEYPGYYMKYLEKRNIVPHMEDGDKEILKKAKMDFIAVNYYRTN

CAEALPEDSQHPFGLREGTVDFSMYGLFKMSMNPNLEASEYGAAIDPSGLRVALNEYWQR

YHLPVIITENGLGAKDILEDGKIHDDYRIDYLRSHINACKLAIEDGVEMIGYCPWSFTDL

LSSSQGFNKRYGLVYINRTDHEVLDLKRIKKDSFYWYKEVIENNGIVK*

>CD630_08210 Clostridioides_difficile_630_NC_009089 two-component sensor histidine kinase

MKMDEQTIILFLLLLSIGLLFLSIFAFSKLGHIYKRLKDIEEILADVEIGDENRKILIKP

CDVMAPLVYQLNEIVYDYENKLLSLKKSDKASKQLMTSLSHDVRTPLTTLIGYLDAVHSG

IVIEQEREEYLEIARRRAYDLKDYIDVLFDWFRLNSDEFTLSIESVEIAELSRNILKDWI

PIFHEKKLNFEIDIPENRLMVNLDPDGYSRVVNNLVQNVLAHSKARQIKITMSEDSRMVL

LRVEDDGVGIARENLPHIFERLYKCDKGRSEKGSGLGLSIVSQMVERMGGQVSVESEIGK

YTVFTVSLPLI*

>CD630_08260 Clostridioides_difficile_630_NC_009089 ferric-uptake regulator

MKFSKQRELILNEILNNPVHPTADYLYENLKKDNPNLSLGTVYRNLAQLTEHGFIRKVSI

PGYPDRFDGRIDNHYHIICEVCGEVYDLESEVLNNLQELISDETDIKITSYNISFKGICN

NCKRCSQVG*

>CD630_08270 Clostridioides_difficile_630_NC_009089 rubredoxin oxidoreductase

MCSEQKFFICETCGNLVGMIQSGGVPIFCCGKPMKELVPNTTDAAVEKHVPVIEVDGNNV

TVKVSSTTHPMTKEHHIAWVYLMTEQGGQRKCLAVDGEPVVKFALNDDDKVISAYAYCNL

HGLWKAEL*

>CD630_08280 Clostridioides_difficile_630_NC_009089 oxidative stress glutamate synthase

MSIYKCSVCGYIYDESKNDKTWDELSEDWECPVCTKGRSYFGKISTVYYEEDEKIAEDIV

EDESKLNTEKEGDLNYLSTYLRRDDEVEKHMDIIHEMAVTGKSIIEPMRTKLPVISWDDI

LIMGAQLNPLPLNEHDEVNTTTIIGKKAKKPMIIENPVYISHMSFGALSKELKIALAKGA

AQNKTAMCSGEGGILPEEKEASYKYIFEYVPNKYSVTEENLKNSDAIEIKIGQGTKPGMG

GHLPGEKVTEEIAKVRNKPVGQDVISPSCFEEIQSKEDLKKLVDELREVSEGRPIGVKIS

AGHIEKDMEFIAYAKPDFVTIDGRGGATGASPKLLKDATSIPTIFALYRARKYIDTHGLD

IDLVITGGLRISTDFAKAIAMGADAVAIASSALMAAACQQYRICGSGKCPVGVATQDEEL

RKRLHIENSANRVANFLNVSLEELKTFARISGHKDIHDLSVDDLYTVNSEISNYTNIQHV

*

>CDM120_RS04840 Clostridioides_difficile_M120_NC_017174 hypothetical protein

MQKNVKKAKVTGSMLAIFGVASVLFSSHAGGGFATGNQETQYYVQYGWTAPLMAILAMII

LTATMREVIIMYNNNDCRNYKDLFCELWRPYPKLEIIWEIYYYLMVLIAVSAVIAGAAAV

FQSIGVNYFVAVFIIGIVLLAFTIFGAMLVSKAATAMTIAILLCTLTIFIVGIKAKAPEI

TEILSNRTSFTPGYFKPILNTFIYAGFQSVVIPTLAGCSRPLLKNSKEATKAMILSFVMN

AIALGLAVTMLMGWYREIIAAGQTTLPTLYVAGQSGNHTIYIIYNVALFLCLMSTGVTTI

FGLVNRFEDHKALSFLSSRMKRRVFTACAIMVVSMLISLTGLSNIVKYGYGYCGYLGLFT

IVIPFLTLGHYKNKKFAKENPEAKWPAELNENIENL*

>CD630_08310 Clostridioides_difficile_630_NC_009089 ATP-dependent RNA helicase

MDFKSLGISENTINILKKSGITTPTPIQKESIKLIKEGKDVIAEAQTGTGKTLAFLLPIF

ENISLDINDIQVLILSPTRELAIQITEEAMKLKESKDVSILAAYGGKDIGSQIKKLKGNI

HMIIATPGRLLDHLNRKTIDLSKLKTFVLDEADQMLLMGFKNEVEAILKETSNKKQTLCF

SATINSQVKKLAYRYTKNPVVVSIQKEEITLNNIKQEVVETTDRKKLDALCKVLDEDNPF

MAIIFCRTKRRVDNLEEALAIRGYNCQKLHSDIAQSKRERIMKSFRNLDIQYLIATDVAS

RGLDISGVSHIYNYDLPETPEDYIHRIGRTGRAGEEGYTCAFIDPKNERMLSEIETAIES

KISRRIIEL*

>CD630_08320 Clostridioides_difficile_630_NC_009089 Re-citrate synthase

MCVISKDRAKEIKIVDTTLRDGEQTAGVVFANREKIMIAEMLSDLGVDQIEVGIPTMGGD

EKNVIKHICSRNLKSDIMAWNRAVIKDVEESISCGVDAVAISISVSDIHIENKLRTSRGW

VLENMAKTVEFAKKNGLYVSVNGEDASRADIDFLTEFINVGKQAGADRFRYCDTVGVMNP

FSIKNAIETLYERTNFDIEMHTHNDFGMATANALAGIAAGANYVGVTVNGLGERAGNAAL

EEVLMALKCVYKCDLNNIDTRKFRGICEYVAQASGRILPTWKPVVGDNMFIHESGIHADG

ALKDPHNYEPFDPSEVNLERKIVIGKHSGRAAVVNKLSEYEMYISPENATKLLNAIRATS

IRLKRSLMDKEILQLYCDILAHEKGTTEEEAVRGSYI*

>CD630_08330 Clostridioides_difficile_630_NC_009089 aconitate hydratase

MGDNIVYKIIKKHIVDGEAVAGSSIGIKIDQTLTQDSTGTMTYLQLEAMGIDKVKTKRSV

AFVDHNMLQQGFENADDHKYIQTVADKYGVYFSKPGNGICHQVFLERFSTPGDTLLGSDS

HTPTAGGVGMMAIGAGGLDVALAMAGGAYYIKAPKVCKVNLVGKLNNMVSSKDIILEVLR

KQTVKGGVGKVYEYGGEGVKSLSVPQRATITNMGAELGATTSIFPSDEKTLEFFKSQGRE

DAWVELKPDADAVYDEEITINLDELKPLAAKPHSPDNVDEVENIGKIKIDQVAIGSCTNS

SYEDLMKVAQILKGNKVHKDVSLVIAPGSRQVMEMIARNGALADIISAGARILENSCGPC

IGMGQSPGTDSVSLRTFNRNFYGRSGTLSAQVYLVSPEVAAVSAIKGVLTDPREFDIKFT

NLDVNEFLIDDSMIIKPADVGSDVEVVRGPNIKPFPLNTELSQSIGGKVILKTEDNITTD

HIMPSNAKLLPFRSNIPYLANYCFNTVDTEFPQRAKDNNGGFIVGGDNYGQGSSREHAAL

APLYLGVKGVIVKSFARIHKANLINSGIIPMEFCDEKDYENISLLDNLEIPNILDNLGSG

ILEVKNTTKGTSFKVKVELSAKEVDVLKAGGKLNYTKNQAN*

>CD630_08340 Clostridioides_difficile_630_NC_009089 Isocitrate dehydrogenase

MYKVTLIPGDGIGPEVAKAMKKVVEATGVEIEWEEVNAGEAVIEEYGTPLPEYIIDSIKK

NKIAIKGPITTPVGKGFRSVNVTLRQALDLYVNLRPIKSFKGIKSRYEDVDLVVVRENTE

DLYAGIEHKIGDYAAESIKIITRSASERIVDFACNYVKDNKRKKVTAIHKANIMKMSDGL

FLDVFREVASKHGVEYDDLIVDAAAMNLVLNPENYDVMVMPNLYGDILSDLGAGLVGGLG

IIPSANIGKDCAIFEAVHGSAPQIAGQNKANPTALIQSSVMMLRYLGEYENAQKIETALE

KVFLEGSKLTVDLGGSASTTEFADEVCKYIV*

>CD630_08350 Clostridioides_difficile_630_NC_009089 MarR family transcriptional regulator

LIKTLDSNILREVGTLSRAVNSINDIKYKELKLQKGQFTFLTRICENPGINLVELSNMLK

VDKATTTKAIQKLIKAGYVDKKQDKFDKRGYNLTPTDKSLEVYELIIEEENRSIEICFDN

FTDEEKQVVTKLLEKMSKNVENEWFKVKR*

>CD630_08360 Clostridioides_difficile_630_NC_009089 acetyltransferase

MIRKANMNDLESIMKIIKSTVEEMKTYNNTQWDENYPLEKDFVSDIKKQDLYIYEVDGEV

AGFICLNYEEPEEYLGLNWSSNKKAMVIHRMAVNPNFRKTGIASKLVDFAEKLAVENNVS

YLKSDTYSINSKMNLLLTKCGFIKIGEMSFLGKEKSFYCYDKIL*

>CD630_08390 Clostridioides_difficile_630_NC_009089 membrane protein

MKYSYKKCIIDSILLMFIVQILRMILNYVLLSQFEFTLENFNIINLISFTLVGLSLILFL

KDNSLYNKVRNRKITEAFEENKNNILIEKCKLILFVVVLSLAIIVTYCTKGYVLFNVTMM

TLSVLIVPIFEELFFREYIWNYLSNFIKSKSKIICITSILSGIYNIGYIDVIRNYVILYN

NSSYTFEVIISKIMIGTVFGIVLGLVKYRFRDVGFCILLRSLFAIFIR*

>CD630_08400 Clostridioides_difficile_630_NC_009089 isomerase/hydrolase

MKFVTFCSSNEEKIGVFNSETNSIYEINSLGLSKLYTDMNDFIENVSTGDLEKIKNNSFE

NAKCYKLEEVKLCSPIVRPKKDIICLGLNYKDHVNEIPDGVIKNVVMPDYPIYFSKRADK

IIGVDDKISLHGDLVEKLDYESELAVIIGKEGINISKEDAYEYIFGYTIVNDISERALQD

KHVQWFRGKSLDTHTSMGPCIVHKEEFEHPLKLDISSVVNGEVRQDSNTEYFIFDIPTII

NDLSRGMTLKPGDIISTGTPAGVAMGMNPQVYLKHGDIVECKVEGIGVLKNIVD*

>CD630_08410 Clostridioides_difficile_630_NC_009089 5-aminoimidazole-4-carboxamide ribonucleotide transformylase

MARELELKYGCNPNQKPSKIYMKNGELPIEVLNGKPGYINFLDAFNSWQLVKELKEATGL

PAATSFKHVSPAGAAVGVPLSDTLKQIYFVDDLELSPLACAYAMARGADRMSSYGDFIAL

SDVCDKETATIIAREVSDGIIAPGYTEEALEILKGKRKGNYNIVKIDENYTPEPIETKDV

YGITFEQGRNEILINEDLLKDIPTDNKIFTDSAKRDLIIALITLKYTQSNSVCYAKDGQV

IGVGAGQQSRIHCTRLAGNKADTWYLRQHPKVLNLKFKKDIGRPDRDNTIDVYLSDDYMD

VLADGIWQNFFEEKPEPLTGEGKRAWLKTLTGVALGSDAFFPFGDNIERAKRSGVSFIAQ

PGGSIRDDNVILTCNKYNIVMAFTKNRLFHH*

>CD630_08430 Clostridioides_difficile_630_NC_009089 group 1 glycosyl transferase

VKILHIITQKPNSTGSGIYLSGMIKGFEKIGHKQAVIAGIDVNDDVNCFPSEVSFYPVKY

NCGELNFPVVGMSDSMPYESTRYKDLNIDMINRLKYQFKVNIDKAMNDFKPELIICHHLY

LLTAFVREMVKDIKVMSICHGTCLRQLNTIDLEKEYIIANIRKLDLIFALHENQKYDIIK

TFGVSESKVVVIGSGYNDDIFYNKNYKIKDDKIKIVFAGKICKSKGLIPFIKAISKLKYS

KDLIEVNFAGTGSDIESYNEIVKLASKSPFKMNFLGKLEQRDLAELFNRSQIFVLPSFYE

GLPVVVLEALSCGTDVITTDILGVKEWIGSEINNSGKIEYVSLPFMEKEGIPKDEELYDF

ENNLYNAIDSKIQSLLNNSNKKTSVDMSKKTWDGLAYRINEVILMDELCLV*

>CD630_08440 Clostridioides_difficile_630_NC_009089 cell wall-binding protein

MRISKKVLALGISALLLSVSFPTSINALDKIENIQGVDKYETAGLISDKQDFTTAVLINA

DSTMVDGIAASGLAGVNNAAILLTNKDDIPEATLQRLNRVTKIYVIGGENSISKDVEKML

LMRRMQVIRIDGVDRVDTSYKIAGEIEKIKNSDKMFLVNGFKDEADAVSVASVAYRDGAP

IILTKDIPSAEEDTDLDPWFGVSPVPVYAIGGESTLSDYIVSRYRATRIGGVDRYQTNKN

VIEKFYNGAKEFYITSGDDLVYALVASPLAKNAPVVLVSNKSDKSILSGASKVTAIGISD

KSIIEQCLDAVKK*

>CD630_08450 Clostridioides_difficile_630_NC_009089 nuclease

MDRKRELKQLYKEMKFDTGVFIIKNDITKKIFLGKSNDIKSKFNSLKFQLGAGSCMIKEL

NNEWKKYGEKAFTFEVLELIKHDDNKTEKDYLEELDILEMVWLEKLKEDNTYEIYSI*

>CD630_08470 Clostridioides_difficile_630_NC_009089 hypothetical protein

MISPIILSSINQNLKEIERNELLETNIESGDYGLALSESDVKDIINSRDNTLKGYGRIEL

DIKVTKQLIENIYTSQYTNVDNYLEAINDMQEIFYYLKNETDDKICDDEVIEILGEFYEK

FSGNMDNVRGEADEFAKKFKFGEV*

>CD630_08500 Clostridioides_difficile_630_NC_009089 NifU-like protein

MREKVEKVLEEKIKPVLQRDGGDVELIDVNENGVVLVRLQGACSGCPGATMTIKAIIENV

LVSEVPGVTQVLGV*

>CD630_08510 Clostridioides_difficile_630_NC_009089 membrane protein

MNYKLILAIVFITSALIFYTIGVFGERKAKILKKKHVIIFWLGFIFDTLGTFTMSNIANS

HTFEVKSALSQNLHSITGLLAIVLMLFHASWATFVLYKDDEEKKKFFHKFSIVVWTIWLV

PYFIGMFIGMAG*

>CD630_08550 Clostridioides_difficile_630_NC_009089 oligopeptide family ABC transporter substrate-binding protein

MKLKKLKVLSLVMILSLMAGCSSGGDKDKKADTPKDGKVLVYGSNDYTSINPALYEHGEI

NSLIFNGLTAHDENNKVVPCLAKDWKFDEATNTYTFNLRDDVKWHDGEKFTANDVKFTIE

TIMNPDNASEIASNYEDITKIDVVNDNTIKITLKAPNTAMLDYLTVGVLPKHALEGKDIA

TDEFNQKPIGTGPFKLEKWDKGQSITLVKNSDYFVKEPGLDKVVFKIVPDDKAKAMQLKS

GELDLAQITPKDMSNFEKDEKNFKVNIMKTADYRGILYNFNSKFFKDKKAKGLPNALSYA

IDRKAIVDSVLLGHGVPAYSPLQMGPYNNPDIEKFEYNPEKAKQEIEKLGWKLGSDGIYE

KEGTKLAFEITAGESDQVRVDMAKICAQQLKEIGVDAKAVVVTETDWANQDAHLIGWGSP

FDPDDHTYKVFGTDKGANYSAYSNPTIDKILQKARETEDKDEKLKLYKQFQVEMTKDMPY

TFIAYIDAIYVGKPNIKGLTPDTVLGHHGVGIFWNIADWTIE*

>CD630_08560 Clostridioides_difficile_630_NC_009089 ABC transporter ATP-binding protein

LEHLLEVNNLSVSFKVEEGEVQAVRNVSFNLKKGETLAIVGESGCGKSVLCKSLMRILPY

NGYIKNGEVLLKSSDLVKKSEKEMEDIRGKNISMIFQDPMTSLNPTISIGKQIAEAVIIH

QGISKSEAKKRAIELIELVGIDNPEKRFKQFPHHFSGGMRQRIVIAIALACNPDVLIADE

PTTALDVTIQAQIIDLIKDLQHKIGLSIIFITHDLGVVATIADRIAVMYAGKIVEIGTVE

DIFYDPRHPYTWGLLGSLPTLDSQDDYLYNIPGMPPNLLNPPKGDAFAIRNKNALKIDYE

KEPPMFKINDTHSAATWLLHPDAPEVDVPVRVNCGRVISNE*

>CD630_08600 Clostridioides_difficile_630_NC_009089 hypothetical protein

MQDKILRDKKANDPWARMTTRNGFSADEIISSFQKAIRRNMVEEACEFAYELYISSPELE

DKLWRRILTISVEDIGMGDPSAVIIINNLNQVRKEYSYADGDRPLFFIHAIRYLCACEKD

RSSDLLKNIIIKGFAMGKVPEVMDVALDKHTYRGLEMGRDSFHFLNEASIVIPEKEVDND

YKERYLKILEKYKQEEVIDTAFKFNHWQY*

>QAE_RS0204355 Clostridioides_difficile_QCD_23m63_NZ_CM000660 PTS sugar transporter subunit IIB

MNILLVCANGASTGVLVEKMKNFCSEHEKLKTKTINIEATSFENLKSYIQANDTDVVLVA

PQIRFKEDEDDVVEACKNYKIAVGLIDTKHYGRMDAPSVMKSAIELYKNR*

>CD630_08620 Clostridioides_difficile_630_NC_009089 PTS system lactose/cellobiose-family transporter subunit IIC

LGGIKMEALQNNLRRFLLPIAQKIEKQRHLQAIKEGMISITPIIIVGSLSMLFMALNNML

PEGSAKTLLSENMDTLLIPNKFTMSLLSIYSAFFIAQALAKKYNLNHVEIGMTAVVAQLV

VCGQVVDGVLDTSYLDAQGLFVSILVALLVVDITKFMNDKNLVIRFPKEVPSVVNKSFRN

LTPMIVCIMLFTAIAAITKNVSGQPLPAIIMNFLAPAISSVDNVFAVTIILFITQLLWFF

GLHGAAITSSIWMPIAATYMAENATLIAAGGDPKYVFTIGFYYGFLQVTGSGITLGLVYL

MSRSKCKSFNSMGKVVILPSLFGINEPVIFGTPIVMNPYMFVPFVFGPVLVGALNFMALK

VGLVGLPIAEPPGFLPPGVAAFLMTLDWKAIVLVFASIILMTLIYYPFFKIMEKEELNKN

AELATTLDDDSFDF*

>CD630_08630 Clostridioides_difficile_630_NC_009089 PTS system lactose/cellobiose-family transporter subunit IIA

MVPEMEEKVFSIISEAGDAKSDVMMSLKEIKKGDYNKAKHLLNSASEKIQTASKYHLELL

SNAMNSEDSGTDFLVVHSEDHYSNALFAHSLVSELVDIFEMMDPRINKN*

>CD630_08640 Clostridioides_difficile_630_NC_009089 maltose-6-phosphate glucosidase

MEKFSVVIAGGGSTYTPEIILMLLDNLDRLPLRAIKLYDNDEERQNKVAKACEILIKEKD

PNIEYLATTCPKEAYTDVDFCLAHIRVGKLEMRELDEKIPLKHGVVGQETCGPGGIAYGM

RSIEGVLENIDYMEKYSPNCWMLNYSNPASIIAEAVRRLRPNSRVINICDMPIGMEHNIA

RIAGLKSRKHMDIRYFGLNHFGWYTSIKDKKGNELLPKLVEHIKEYGFINGEEGMKNDKK

DSWFETNLFTKEIVKTDLTTIPSSYLKYYLFPDYVVNHSDVNYTRANEVIDGREKEVFGS

CALIEKQGHSKGSGLKIGIHAEFIVDLATALAFNTQERMLLIVQNNGAIENIQDDAMVEI

PCIVGKDGYEPLSIGKIPTFQKGLIEQQLAVEKLVVDAWIEKSYNKLWQALMLSKTVPSA

TVAKELLDDLIEANKNYWPKLN*

>CD630_08650 Clostridioides_difficile_630_NC_009089 ADP-ribose binding protein

MKWRDYAADVNLFEDFDKTIKPLTDEQRRKNINTLIAYFSKEVPSKIYNLSNDEIKPRDI

LRGLLNVYPPKEIAPEILNMLHNLLLIECEERELVDVNDIEEIEEGIAIWRGNITNLRAD

AIVNAANNKLLGCLQPLHLCVDNEIHSCAGPRLREDCDKIIKKQGHLEYTGDAKITRGYC

LPAKFVVHTVGPIVSGGQPSKEQEKQLLHCYKSCLNTIKEIDEIKNIVFCGISTGVFGYP

KKEAANLAVSRVRLWLKENPEKNLKVVFNVFTEEEEEKYRRIFK*

>CD630_08660 Clostridioides_difficile_630_NC_009089 hypothetical protein

MKNSFKNCISLRNKFIIISIVFLLILLIVSYIFGKLVYNSSVGLEQLIKNEDIVKIFSER

KDKPLEKLKNYKTSELMIQSSNNYKLESLFITSNIKTRDTMILVHGIGSSYYEMLKVAYR

YLDKGYNVLVYNQRNTGNSGGDNYTFGLYERYDLDSLVKFVKNKFPEGRLGVHGFSMGAG

TAAMHSEINSKDDKVDFYILDSPYSEMKDAIRMGVLEKRIPDILINYVVTCGDLYNKFKS

GFWYSDVKPYESVEKSNVPILFIHGTKDTVCNYQNSKKMYDLVKHDKKDLWLIEGIGHVD

GFEHDSTVYFNKIFKFIDSNVLSDKS*

>CD630_08670 Clostridioides_difficile_630_NC_009089 hypothetical protein

MKNKNNKDKTMLNIKVLRVLGVLLILFDYTGDTLNFYNFFAKPLVSGNLYKTTGTDCIVF

LLFIVECVYLVGRYILKVKINWSEIIENEKGNFINNLLVEKIQEESNIVYNLLNSYVLPH

IYKKARGLMFKTYHMLIYIKEKYYYVNIY*

>CD630_08690 Clostridioides_difficile_630_NC_009089 molybdenum-specific ABC transporter substrate-binding protein

MKKILGILGLVACLTLGTVGCNSNEDKKDNGKQEKTTKSSDSVELNISAAASLKEAMAKI

EEEYKKVDSNVKLTVNYGASGSLQQQIEQGAPCDLFISAGQKQMKVLDEEKLLVSDTMKD

LVKNDLVLISSADSSVSGMKDLTTDKVKKIAVGEAESVPAGKYADEVLTNLNLKDKLKDK

LVFAKDVKEVLAWVQSGNADVGFVYFSDTVNNDKIKVVEKTDEKTHSPITYPVSVIKASK

NVDAAKKFEEFLLSESGQKIFEEFGYKKVE*

>CDIF1296T_01036 Clostridioides_difficile_ATCC_9689__DSM_1296_strain_DSM1296_CP011968 molybdenum ABC transporter permease

MEIVKLIELLVSLFPFKIKEGKMGTDWSPLWISLKTSILSTIITFVIGIFVSYIMANYRG

KWKGLIDGLFTLPLILPPTVVGFFLLLLCGKNGFIGKFLLNFDRTLIFSWTATVISAVVV

SFPMMYRTSRSAFEQIDNNMLSAARTLGLNEWKVFFKIAVPLAWPGIIGGLVLSFARALG

EFGATLMIAGNIPGKTQTMPIAIFFAVEGGDMNKAMMWVMIIVAISIAMILLLNYWSEFQ

QKIIGKRCG*

>CD630_08710 Clostridioides_difficile_630_NC_009089 molybdenum-specific ABC transporter ATP-binding protein

LSLYVDIEKDLSSFKLKVEIKQEKGTLGFLGESGSGKSMTLKCIAGLEKPTRGKIVLNDR

VLFDSEKKINLSTQDRKVGFLFQNYALFPHMTVSQNIELGLLKLSKSEKKEIVARYLDIL

KLNGFEGRYPWQLSGGQQQRVALARALATSPDILLLDEPFSALDHHLRSNMEKELMNMLK

DYKGDILFVTHDIEEAYRVCDDIIVYNKGEGLPKRPKKELFESPKYLIEAKITGCKNISK

LNRLDKNTIYATDWGCELTLNREIGDNIEYVGIREHHIKVLDSNEDLNEKLCFELINIVE

NPFTYTIYVRKTDLSNECVPIQIELEKSKMRFKKGDRIYLDFPQEYLFCFRYNYNKKE*

>CD630_08720 Clostridioides_difficile_630_NC_009089 maltose O-acetyltransferase

MTEKEKMLSGKGYYANDELLVKEREYCKKLTRLFNNTLEDEYEKREDILRQLFGSVGKQI

NVEQNIRCDYGYNIHVGENFFANYDCIFLDVCKIEIGDNVMLAPNVQIYTAYHPIDAQLR

NSGIEYGSPVKIGDNVWIGGGVIITPGITIGDNVVIGAGSVVTKDIPPNTVAVGNPCRVI

KKIEE*

>CD630_08730 Clostridioides_difficile_630_NC_009089 sugar family ABC transporter substrate-binding protein

MINKKRLASLILAGALSISMLTGCSQGGDSGNSKQESNSKDKEVKKIGITQLVEHPALDA

TRTGFVKALEKNGFKDGENIDIDFQNAQNDMPTTQSIASKFASDKKDLIFAISTPSAQAA

FNATKDIPILITAVSDPVAAGLVKTLEKPGTNVSGTSDFVSVDKGLELLKIFAPKAKTIG

VMYNTSEVNSKVQVDALKEYASKNGFKVVEKGITTSNEVNQGISSLVGKIDVLYVPTDNL

VASSMPIVSKIATENKIPVIAAESGPVEKGALACQGINYEKLGYKTGEMAVKILNGESVS

DMPVATSDDTDIIVNEDILKALGMEKPSNENISYVKTKQE*

>CD630_08800 Clostridioides_difficile_630_NC_009089 protease

MKLIWLIVAVLFGIAEMLTPSLTLIWFSVGAVILIFLSSFIESIFLQILIFAVISIAMLV

VATKKIVKKDKGYKSNTNLQAMMSKKGIVTEEISPNNTGLVVVEHETWTAISIDGEKIEK

GSTVEVLKIEGVKLVVKKVDATVNVTNQ*

>CD630_08810 Clostridioides_difficile_630_NC_009089 hypothetical protein

MGTKIVLSIVLIVVVVAISLTCIRVIKQSKVGIIMRLGKFQKVAETGVHFLIPFLDKMAY

VIDLREIVIDFPPQPVITKDNVTMQIDTVVYYKVTDPVRYVFEIANPIAAIENLTATTLR

NIIGELDLDETLTSRDIINVKMRTILDEATDKWGIKVNRVELKNIMPPQDIQVAMEKQMR

AERERREAILQAEGNKSAAILQAEGEKQSAILTAEAKKEAMVRVAEGEKESAILVAEGEA

EAIRQTAIAKAQGEAEMIKRTQMATAEGLKLVFSAMKEADIDNNILALKSMEALEKMAEG

KSTKLVLPSEAVNFLGTFKGIKEVMSDDNKEVLDIKEVLNDNESLKK*

>CD630_08820 Clostridioides_difficile_630_NC_009089 glucose-1-phosphate adenylyltransferase

MKKEMLAMILAGGQGSRLGVFTKRIAKPAVSFGGKYRIIDFVLSNCSNSGIDTVGVLTQY

RPLILNSHIGMGSHWDLDRINGGVYVLQPFMNEKEGNWYNGTAHAIYQNMDFVDTYNPEY

VLILSGDHIYKMDYSKMLKFHKEKGSKATIAVIEVPWDEASRFGIMNTNEDSSIYEFEEK

PSEPKSNLASMGVYIFDWKMLRNYFKEAEKNPEINYDDFGKNLIPKMLEDNVGMYAYPFK

GYWRDVGTIQSLWDANMDIIKSPETLDLADPKWKIYTNTMAMPPQYIGKNANVHRSMIAD

GCRILGEVGNSVLSHGVVVGKGSKVIDSVIMPNVVIGENVTIEKAMIGECATINDNVQIK

NVNNEINVVSEYENIEPRCVLIEGGL*

>CD630_08840 Clostridioides_difficile_630_NC_009089 glycogen synthase

MKVFYVTAECWPFAKTGGLGDVSYALPKELKKEGVDVRVIMPKYSTIPSYLKDQLKEIAV

FSVRVGWRNQYCGLLEMELDGVKFYFIDNEFYFRREDERKSIYGYGDDAERYTFFTDAVL

EAISRIDFYPDVIHINDWHTGMLPLILKERYATLEGYKNIKTMYTIHNLQYQGVFDKHVL

YDILDLPQKYFDNGDIEYYGSINFMKAGINFADKIITVSPTYANEIQTSFYGEQLDGLLR

KESGKLKGILNGIDYDLNDPAKDKDIFVHYDVDSINKKVENKLRLQDILGLKKDSSIPLI

GIVSRLVSQKGFDLIAYMMPELMREDLQIVVLGTGEHQYQSMFNYYDSNFSDKVSARITF

NASLAQQIYAASDMFLMPSLFEPCGIGQMLAMRYGSLPIVRETGGLRDTVTPYNKFTGEG

NGFSFKNYNAHEMFFCLKNAIKVFKDKEKWIKLVENAMKTDNSWKKSAKEYIETYRDICD

*

>CD630_08860 Clostridioides_difficile_630_NC_009089 alpha-amylase

MQSVIEYNSWDKNFKAPFGALKFDEELTICVKVNEGYNIKSISLEINREEEMRTITLNEE

LDNDKLGKCFCGKIEKFDGTGVYFYYFKVDVEMDGQIKTLFYGKNRDNGYSCEYNYSDIN

KYQITVYKDFKVPTWYKEGILYHIFVDRFNNGNRSGKVDNPKKNSFIYGNWEDTPMYIKD

SQGDVIRWDFHGGNLRGIINKLGYLKKLGVSILYLSPIFEASSNHKYDTGDYKKIDPMFG

DEDTFKELIDKAKEKGISIVLDGVFSHTGADSKYFNMYGNYNSLGAYQSKESPYYSWYMF

EEFPQKYKSWWDVKTLPNINELEHSYMDYIIYDNDSVINKWVNMGIKGWRLDVADELPTK

FIRELKKELKKADDDSILIGEVWEDASNKISYGQRRSYLLGEELDSVMGYPFRNNMFSFL

KGEINSYELCNRYMQIKENYPKESFRSNLNLIGTHDVTRAKTELNDDVDLVKLAVAIQMT

FEGVPYIYYGDEAGLCGGVDPDNRRTYPWKNEDEDMLNFYRDVIKIRNKNKLLSSGNTEF

IYTNNDSVFSFIRVNENNDRMLILVNRSENVESISLCIESSFIEEIPIKYSLKNANSTIQ

IENNELKVDLDSKSFRIFRVN*

>CD630_08870 Clostridioides_difficile_630_NC_009089 GntR family transcriptional regulator

MKVDPLTTQVYDYISKKIQNGEYEANQRITESEICKSIGVSRTPAREALTRLAGENLLEK

IPNKGFVVKEFQEKEKLDTYSVIGVLDALAGSSALQNLTESDLVKMEELTEMMAVSIKYK

NYNSYLKLSNEFHDIYITKSDNQVLVNLLNSLRYNFMSKSYTSDNEDELYKMLTYSNNQH

IEVVKSMRENDLENVERVLREHWKTIPIAEMKKEKLL*

>CD630_08880 Clostridioides_difficile_630_NC_009089 arginine decarboxylase

MDSEFANAVELSYECAPLLESLKEYSEKDIACFDVPGHVKNRGVAILNKYLGESLMKMDI

NSSPTMDNVSAPNGIIKNAQDLLAQAYMADEAFFITNGTTQAIHAMILSVIKPGEKVLLP

RNIHKSVINALILCGGIPIFIQPEFDEKLGISLNITLEKVRTEIEKDCNIKALFFLNPTY

YGVCADLESIIELCHKNNVLVLVDEAHGAHFPFHLDLPPSAISLGADMVAVSIHKTGGAL

TQSSALLLNRDNVSFEKVLQSINMLQSTSASYLLMASIDGARVNLVENGEKQLSKALNLS

RYAKSKLNKIDGIKVLSTEILKQKGVKFIDETKLCINVKELNLTGFEVYDLLYKNFSIQV

ELGDSYNILALVSIGTNKSDIDRLVKALSIIAKVYRKESTLNEFNMVQINPIIKLNPREA

FYAPKESVEINSCIDRICGESIMAYPPGIPIIAPGELITEEIMEYIIFLKNSNAYLTDVQ

DKNLDRILVIK*

>CD630_08900 Clostridioides_difficile_630_NC_009089 polyamine aminopropyl transferase

MELWYTEEWTENVRFSIKVNKHLFEGKSQFQRIDVFDSDEFGKFLTIDGLMMVTYKDEFI

YHEMITHVPMATNLNIKKVLVIGGGDGGTVRELSRYPQIEKIDMVEIDKMVVDVSKEYMD

ICSCKLDDKRVSLYFEDGVNFVKCAHDKSYDLIIVDSTDPIGPGEGLFSTDFYKDCYRIL

TDDGILVNQSESPYFDFNAKEMKRANKKLKQIFPISEVYQAHIPTYPSGHWLFGFASKKL

NPVKNQDRNGWEKLSLKTKYYNSDIHLGSFMLPQYVKEMLDEE*

>CD630_08910 Clostridioides_difficile_630_NC_009089 agmatinase

MKNNFYHMNTFMSMDKNYEESNLIVFGVGFDGTTSNRPGARFASSSMRKEFYGLETYSPF

LDLDLEDYNICDYGDLEISVGSTEQVLKEIYQETYKIVRDSKVPFMIGGEHLVTLPAFKA

VHEKYNDIYVIHFDAHTDLREEYNNSKNSHATVIKRIWDIVGDNKIFQFGIRSGTKEEFK

FATEEKHTYMEIGGIDTFENIVNMLNGKNIYLTIDLDVLDASVFPGTGTPEPGGVNYREF

QEIFKIIKNSNINIVGCDIVELSPDYDTTGVSTVIACKILRELCLIISDKIK*

>CD630_08920 Clostridioides_difficile_630_NC_009089 cold shock protein

MKNGIVKWFNNEKGFGFISVEGEDDVFVHFSAIQNDGYKTLEEGEKVSFDITQGNRGPQA

ENVNRI*

>CDIF1296T_01061 Clostridioides_difficile_ATCC_9689__DSM_1296_strain_DSM1296_CP011968 metallo-beta-lactamase superfamily exported protein

MRKIVLKDILFLVTTLSIIVILLLVTNITTNSKIKSTSESYLKSDKNSVRQNGIQRSTKS

STKNTAFKTTILNTGKSDCILVEIGNKVIMIDTGEDKNGKQIVDRLKEKGINTLDYLILT

HLDKDHIGGVDSVLSSVKVKNIIQANYKKDSKQYDEYIDSLKKADIEPVLLKDNMNIVIN

SAEINIHPASKSKYESSNDYSIITNISYGAHKFLFAGDAEEERLSEFINGNTLKYDFVKM

PHHGRYDKLTETFLESISPQYAVITCSEKKEPEEGVLKILERLNIKTFLTSNGEVVINSD

GKTLSVNQ*

>CD630_08970 Clostridioides_difficile_630_NC_009089 hypothetical protein

MAIVDKIFNEIKLMVARLLLGKKYSEYEEKDLVYNTEEEVILITLKRLVFQGNVNEAEEI

LFDKAKSVNSENMQYIAIEFYTMLMEKTDEELEAMNFSKQEVYQGIEDIRKVLNLE*

>CD630_09000 Clostridioides_difficile_630_NC_009089 glycine betaine/carnitine/choline ABC transporter ATP-binding protein

MIEIRNVTKKIGNNVILDDISLVVETGTLVVLIGSSGCGKTTTLKLINKLIKPTSGEIYI

NGKPISQENEIELRRKIGYVIQNTGLFPHLTIKENIELIPRLKKEKSVEEIEKRTLQLLE

MVGLDSDEFLNKYPSELSGGQQQRIGVARAIATDAEIILMDEPFSALDPITRTSLQEQLF

SLQDELKKTIIFVTHDMDEALKIADKICIMKDGRIAQYDTPENILRKPANDFVKDFIGED

RVWDNPEYIKARDIMIKNPIAVNSTRTVTQGIEIMRTSKVDSLLIIDRAKTLKGIVTVKD

MKDIDDKSILLADIMSSEPLHVNEGDNLVEILNVMNRNSVGYIPVISDENKLVGLITRSS

LLSVLSEQFLEMEVSVLG*

>CD630_09010 Clostridioides_difficile_630_NC_009089 glycine betaine/carnitine/choline ABC transporter permease

VFLANFFNFILLQKDKIIELLIQHMSLTVTSILIAIIVGVPLGIIISRISSLRKFVLGFV

NLVQAVPSMALLGLLVPILGIGSKPAIFMVVVYSLLPIVKNTYIGITSIDPVVLESAKGI

GLTRNQTLFKIQFPLALPIIMGGVRISAVTAVGLMTLAAFIGAGGLGYLVFSGVQTVNNN

MILAGAIPSCIIALLVDYLFSKIEVAVTPKGLNPKAPKKNYIALKVISVVLVVSMLFVVF

SSSFSSKKDTITIGSKDYTEQLILGNVYAELVEKNTNLKVKKNLNLGGSSVAFNAIKSGE

LDMYVDYTGTLLVNVMRHAPIKDADEAYNVVKDTMEKENQLTLLDPLGFNNTYTLAMMPE

TAEKYGINTISDLTKYGKEFTFSPTLEFENREDGLVGLSRDYGLKFKDVKAMNGSLRYTA

LDNNESQVIDAFLTDGLIKKFNLKILEDDKNFFVPYYAAPLVREDTLKKYPELEKVMNML

SGKVNEETMRELNYQVDELGKSPEEVAHSFLVKEGLV*

>CD630_09020 Clostridioides_difficile_630_NC_009089 cation efflux protein

MDNYKKVKQVLWIILFANFAVALLKIIIGNQIKSYSMTADGFHSLSDGASNIVGLIGIFF

ASKPKDKNHPYGHKKFEIITSLFISGMLFVIAIKIILSAVLRIANPVVPAITIESLIALI

ITLFINIFVCMYEYRIGTKLNSYVLISDSLHTRSDIFVSLGVLVTLVGVKLGFPVIIESI

VPIIISAFIIYSAYGIFRPSIGILVDRVAVDEDYIKEIVFEFNEVRDVHNIRSRGSKSSI

YIDMHVMVDPFISVEQSHDLTHKIEKQIQEEINENAQVIVHIEPFYSF*

>CD630_09810 Clostridioides_difficile_630_NC_009089 membrane protein

MDGDILMSKILKYKNEMFLFILVVITYLIITKIFFSKTTIFYDLNNTYDVLLDTDTGVLF

NLNVFAISQDNSKHILFSAIISIFAYPIYLFCTSIANPGTTDFNSAYGFGLICLQIITSA

MSITLVFNHIKKIKMQRLTLILLTMIMIFSFPQLFMTLNVERFIYSQFSLIFFIVIANKM

KGKNSYLIELAAIPLFGITISNIYLYFFNMIFEFKLKIGKMLKHLITFILMAYICVVSTK

SYESFMNLGNVIQYDTKFISGEPILEKIAMIIERLLYSVFYFPGASIKKGLFLQNGEVAT

IPVILTLLALCFCVLSVIENSEKRVPKLCMGIIIFNLTLHGIVGYNLVNSSIMAINFSFA

VIILLAYFTKALRKNEKNMYNIFLSLLLVTIVISNVNGFIEILNIGIKSYPV*

>CD630_09820 Clostridioides_difficile_630_NC_009089 UbiA-family prenyltransferase

MKQTNTSIRLLTKISDYIKLMRIKQYIKNFFVFSAIIFSNNILNINLFLNTFIAFVCFCL

MSSSVYALNDAIDMDKDKKHPKKCNRPVASGRISKKSANILFVVLALTSVCLSTVVSFNL

SIILSLYLINNILYSLKLKNIILLDVFSISLGFILRVYAGCVAISVSLSNWIILCTFFLS

LYLALGKRKKEIETLRDDAVEHRKILEDYDIENLNQMMIVILSSTIVCYALYSTSNPEKP

HMIFTTIFVVYGVLRYNYIINTTNENNPTDIVLKDNALKINVILWIITCLIILIF*

>PCZ31_RS04720 Peptoclostridium_difficile_strain_Z31_NZ_CP013196 membrane protein

MSLRIGGNSLGYLHILILSMVPVTELRGAIPIGIAMGLNPIWVYVFSVIGSTLVSIPLIL

TFRHVLQFLRGKKLFKGIADVIDRKINSRMKKLKSVSIIGIILFVGIPLPTTGTWTASAI

ASILKMRIKDAFMGVFLGNLLSGVIVSALSLHII*

>CD630_09850 Clostridioides_difficile_630_NC_009089 MATE family drug/sodium antiporter

MENSNTNLGSESVGKLLFKLATPAIIAQIVNVLYNIVDRIFIGRMENGEVAMAGVGVAFP

IIIIITACSYLIGMGGGPLAAIKMGEQNNDEAEKIMSNSFSVLVILAILLTIGFKIGKEP

LLWMFGASESTIGYSMDYLNIYLIGTVFVQISMGMNTFINTQGFATTGMMTVAIGALINI

ILDPIFIFGFNMGVKGAALATIIAQGVSAIWVLMFLFGKKSILKIKKKYMIPKASIILPV

LGLGISPFIMQSTESLVLIALNSKLQMYGGDLAVGSMAIMSSIMQILMLPNMGVTQGAQP

IISYNYGSGQLDRVKKTFKLCLLSCFTYSTILWLLLMIFPAFFVSIFNKNPQLLSMTSWS

IKIYFAGAFMFGIQIACQQTFLALGKATISLVLALLRKIVLLIPLIFILPTFFNEKLFAV

ILAEPVADITAATITAISFFIFYKCFLSKPKAIKE*

>CD630_09860 Clostridioides_difficile_630_NC_009089 30S ribosomal protein S1

MFIMENDLTMQELLDQQEQVFSKVKVGELTTGKITAVRNDEVQLGLDYGFDGIIPISELN

IEKNQYIEDIYHIGDEITAVITKVSQKDGTITLSKLQLDKRNDFAELQKAYDEHRIITVN

VEKNIDKGVFANYNTYTFFIPISQLDTKFITDTSKFVGLNLEVYIKELDVRKNRLVASHR

DVLQERINKEREERRAQIKAEKEAERARIKQEREEEKARIKAAKEDLFNSLEVGQKRDGK

VTKIMPYGAFVDIGGIEGLAHINNLAWTRVESVEDVVSEGQEVEVYVLDVNKETKKIALA

LKDINNDPWDLIAKEVQIDDVVNAKVLRIIEKGAFVQIKEGVDAYLPISELSDTRVAKVT

NVVNIGDEVKVKILDFKPKTKRMLVSIKEATREPEEDITEYLEVEESLGSIGELFKDKFK

DLEV*

>CD630_09870 Clostridioides_difficile_630_NC_009089 hydrolase

MKKSIMDVHCHTLISGHAHSTFKENVEEASKKNIKYLGISDHGPNMPGGPHPFYFYNLHL

LPREVQGVKILRGIEGNIMDYNGNLDVQEDMLQHLDYIIASLHRPCIASGTKEENTNAIL

KVMDKPKVKIIGHPDDSRYPLDYEPIVKKAKDKNILLEINNSSLSSNSHRTGTWENVSHM

LTLCKTYGARVILGTDSHICYSIGEFENAEKVLEAVDFPDELVINYHEDEIIEFFDINF*

>CD630_09880 Clostridioides_difficile_630_NC_009089 hypothetical protein

LSKNKFIYMNYYDNGYFKKGMTIYMVLALIILATIFIYSTTILYILCIITAVFLFKQGRD

FSNKYEGKVYITLDNHSILINNQCIFSIKSKQSQKFNYKIIEKIEVVKNILNIYTNESNY

KIRLRALSLEDEKKLLNIIDEKMKKFKA*

>CD630_09890 Clostridioides_difficile_630_NC_009089 2-isopropylmalate synthase

MKCGKYKKYDKMQIVNRKWPDNEIFKAPIWCSVDLRDGNQSLPTPMSVNEKVRMFKMLID

TGFKEIEVGFPSASNTEYTFLRKLIDENMIPDDVTIQVLTQSRAHLIEKTFESIRGCKKA

IIHLYNSTSVLQRDVVFNMSKQEIIDIAVEGAKLFNEEVKKYPETEFTFEYSPESFTGTE

MDYALEICEAVIDVWKPTPQKKVIINLPSTVEMATPNVYADQIEWFCKNISCRDSIILSL

HTHNDRGTCTAASELGLLAGADRLEGTLFGNGERTGNMDIVNVGLNLYTQGIDPELDFSN

IDKIIGIYEDCTKLMVHDRHPYAGNLVHCAFSGSHQDAIRKGMIAMKNRDNDYWEVPYLP

IDPHDIGREYKEIIRINSQSGKGGAVYIMETDYGFMIPKNMHSDFGNVVKMESDRIGEEL

SSEAIFNLFKKEYIEVESPYKVKKYKIKSMDELNYENDDSNDTNMIEMTARISYMGNEQR

IVGIGNGPVDSFNNALKQCGMKDYKFRYYWEHALEEGSHSRGVAYVGIEHNNEVYFGVSI

SENINTAAINALMNAINKSYIEEEIKNGDDYDAENISQTC*

>CD630_09910 Clostridioides_difficile_630_NC_009089 3-isopropylmalate dehydratase small subunit

MIANGSVFKFGDNIDTDVIIPARYLNIADYKELATHCMEDIDDKFISKVKKGDIIVATKN

FGCGSSREHAPIVIKESGVSCVIASTFARIFFRNSINIGLPILECEEAANNIDEGDNIEV

DFSTGVIKNITKGKEYKAEPFPEFMQNIILNEGLINSIKANRG*

>CD630_09920 Clostridioides_difficile_630_NC_009089 3-isopropylmalate dehydrogenase

MNCNIAVIKGDGVGPEIIDEGIKVLNKICCKFNHRFDCEYVLAGGCAIDETGEPLPNKTV

EICRKNEAVLLGAVGGPKWDKCKGDKRPESGLLKLRESLGLFANLRPATMYESIKEASPL

RTDIVEKGIDFVVVRELTGGIYFGERGRKIIDGIENAYDVEIYNENEIRRIGKRAFEIAR

NRNKKLISVDKANVLESSRLWRSIMEDLAKEFEDVELSHMYVDNAAMQVVKDPSQFDVIV

TNNIFGDIISDEASMITGSIGMLPSASLREDSFGMYEPIHGSAPDIAGKDIVNPIATILS

VSMMLRHSFNLEEEAKCIEDAVQSVLNKGYRTIDIYNGVGNVVGTRAMGELIVNEI*

>CD630_09930 Clostridioides_difficile_630_NC_009089 thioesterase

MDFLAKYKVLKKQNSSKSCLVCGTQNELGLKADFYELENGELVSICNTKDWHQSYPGRVH

GGMSAAILDETIGRAVSINDDQIWGVTVSLELKYKKPVPTDATIKVVGRITKENRKLFEG

TGEIILPNGDIAVTATGKYMKMPIGQIAEGDFSNEEWFFEESKEKVEYIEL*

>CD630_09940 Clostridioides_difficile_630_NC_009089 serine-pyruvate aminotransferase

MSKKLFIPGPIDVKEEVLQKMATPMIGHRGKDASMLQKSISEKMQKLFYTNNTILLSTSS

GTGLMEGSIRSCTSKKAAVFSCGSFGDRWYKMAVANNVPADIFKVELGEATTPEMVDKVL

STGEYDLITVTHNETSTGIRNPIEEIGEVVKKYEDVIYCVDTVSSAGGIKVEVDKIGIDI

CITSVQKALGLPPGMSICTFSQKAIDRAKQVPFRGVYLDLLAMYEYLIKKNYQYPSTPSL

SHMFALDFQLDNILDEGLDNRFNRHEDMANLVRNWAKKHFQIFTNENHLSNTLTVIENTQ

GISVSNLNSKLQERGFQIANGYGDLKEKTFRISHMGDYTVEDVQELLDNIDDILGFNK*

>CD630_09950 Clostridioides_difficile_630_NC_009089 D-3-phosphoglycerate dehydrogenase

MYNILVTDGIEKEAARKLRELDFNVIEQFYEKDVLGDKLKDVDVLVVRSATKVTKDVIDK

ALEGKKLKLIVRGGVGLDNIDVKYAQANGIKVMNTPNASSISVAELTIGQLFVLARFINT

ANVTMRDGKWEKKKYKGTEINGKTLGLIGFGRIAKEVAKRAELLGMNVIYTDIMGEAQGF

NNYKFCDMEEVLENADFLSLHIPFDKNKGAVITEKEINKMKKGAYLINCARGGLVDEKDL

LKALDEGKLSAAAIDVYEQEPTLNLDLVNHPRVSPTPHIGASTVEAQERIGDEIVNVIQD

FFLDFNNLIGVAL*

>CD630_10010 Clostridioides_difficile_630_NC_009089 nitrate/sulfonate/taurine ATP-binding protein

MEKIKLSIENINKRYDSRIIFRDFNIDFYVNEVNCILGKSGCGKTTLLNIISGIIKNDTN

NLNIKENLNRVGNKLEASYIFQDDRLIDWLTVEENIKIVVNKYYNKTQLNKICDEYLELV

GISDYKKFYPQMLSGGIRQRVNIARAFIYPSKNIIMDEPFKSIDAKNTQLIMDNFRNILR

KEKRTVLFVTHNIEEALFLADRIFILGDSPIRIKKILKNSKELEKNEVLKLI*

>CD630_10020 Clostridioides_difficile_630_NC_009089 Cys-tRNA(Pro)/Cys-tRNA(Cys) deacylase

MVKTNAMRILDSNKIDYKVMSYEVKSEHVDGVEVAHDIGRDVNEVYKTLVTQGVSKNIYV

YVIPVHENLDLKKAAKVAKEKSVEMIHVKDINKLTGYIRGGCSPIGMKKLYKTFVNESAK

NLDTIIVSAGKIGYQIELSPFDLQRLIKVEFVDVIKK*

>CD630_10030 Clostridioides_difficile_630_NC_009089 fumarate hydratase subunit A

LRKIKSEQIVEQVKKLCIEASLYLGEDVLSCIKEKAKSEKSEVGKNILNILVENAEIAKE

KNIPICQDTGMAVFFVEIGQEVLIEGDTLTDAINEGVRQGYEEGYLRKSVVSPINRVNTK

DNTPAVIHYDMVKGDKIKIEFAAKGFGSENMSKMKMLKPSDGLEGIKKFIIDTVSEAGPN

PCPPMVIGVGIGGTVDKCAQIAKKALFRELGEFNKDENIAKLESELLTAINKLGIGPQGL

GGTTTALGLNIETFPTHIAGLPVVVNINCHASRHKKVVI*

>CD630_10040 Clostridioides_difficile_630_NC_009089 fumarate hydratase subunit B

MIKITTPVNEIDIAKLNCGDTISLSGILYTARDAAHKRLIDCINKGEELPFDVYGQGIYY

VGPTPTKPGEVIGAAGPTTSYRMDDLTIPLLERGLRLMIGKGKRSDEVIEGMQKYGAVYL

AAIGGAGAYISNSIKSCEIIAYEDLGAEAIRKIVVEDLKLTVAIDSYGNNIYEQGRAIYE

CK*

>CD630_10050 Clostridioides_difficile_630_NC_009089 NAD-dependent malic enzyme

LMSKDYAKLALEMHEINKGKVSVESKVEIKTKDDLSTAYTPGVAEPCLKIHENQDDVYRY

TSKGNLVAVVSDGSAVLGLGNIGAEASIPVMEGKAILFKQFADVDAFPICLKTNDVDEIV

KSVELMEPVFGGINLEDISSPRCFEIEERLKKSLSIPVFHDDQHGTAIIVAAAIINSIKL

IENKKIEDLEIVINGAGAAGIAIAKILLNMNVKNIILCDRSGALEASIENLNYVQKEMLK

VTNIRNEKGPLKDIIKGKDVFIGVSGPGAVTKEMVESMSEKPIILAMANPTPEIMPEEAK

LGGAFIMGTGRSDFPNQVNNVLAFPGIFRGALDVKAKEINEEMKIAAAYAIANTISDEEI

CPEYILPDVFNKNVVKNVAKAVKEAAIKTKVNRI*

>CD630_10070 Clostridioides_difficile_630_NC_009089 hypothetical protein

MEETKFCQCCAMPMGQTDELYGTNKDGSKSTDYCSYCFKDGEFTADISMEEMIEVCIPHM

LQSNKDMTEDEARTMMNNFFPTLKRWK*

>CD630_10080 Clostridioides_difficile_630_NC_009089 S1 RNA-binding domain-containing protein

LINIGDFNKLTVKRKTEFGYFLDGQTNNTKDDILLHNRLIGKNEINIGDEVNAFIFKDSD

DRTAATLIPPLAKVGDVAHLKVVDNTDIGTFIDMGLPKDILVPFKAKTYPLFRDEKYLFY

IYLDKSKRIAATTDIDSYLLTDHTYNVGDIVTGVVYGFQTNNSAMICVDNKYAGVILHNE

YFTELKAGDVLENLHVIKIYEDGKLGLSPRGNRKDELDTLENKILSYLEGSDGYMRFNDK

SDPKDISILFNSSKKNFKRALGVLMKKGLIYQDEEGTYLK*

>CD630_10090 Clostridioides_difficile_630_NC_009089 GntR family transcriptional regulator

MKIVNKSSNIPLHTQLSSIIREMIETGELKEGDAIMPERELCNIQNVSRMTVNKTIVGLV

TEGLLYRVQGKGTFVAKQKKKYQFSNVKGFTDVMKEKGVNIKTDILSFEMELPDDLVKRK

LGISDNTTNIYKIVRLRYTDGEPFGLEIVYLSEEMCKGLTKGILDNSSLYRVLNEKYGYK

IQKAEQVMEPVILSDEESKLLETDEGALALKLHRNSYNREGSPIEYTISIFRTDKYQYEI

VLSE*

>CD630_10100 Clostridioides_difficile_630_NC_009089 N-acetylglucosamine-6-phosphate deacetylase

MKCLINGKIILKNQILENKVLVFDEKIIDIADSVPKDCEVIDADGKYISPGLIDIHIHGN

MGKDTMDSTDESIETISKSIMRHGVTSFLPTTMTMDKEHVYDALEVIKKAQNRKLEGAQV

LGAHLEGPFINENYKGAQNEKFIINSKYEFIKEYKDVIKVITYAPEKDIDFDFTREIKRC

TDIVLSIGHSNANYDQAKEAINLGVTNVTHMFNAMTGLNHRDPGVVGAALTTNVYSELIA

DTIHINKDLFQFILNNKGKERLILITDSIEAGGLEDGNYSLGGQKVIVKGNEARLENGAL

AGSVLSLNKMVFNFLDNTNLKVNEAINLASLNPATSLGINDKKGSLEIGKDADIAVFDEN

LDCKMTLCLGEVVYKNI*

>CD630_10110 Clostridioides_difficile_630_NC_009089 glucosamine-6-phosphate deaminase

MRVLVCKDYDGMSKKAAEMIAAQIVLKPNSILGLATGSTPVGMYRDLVKKYNDNIVDFSD

VMSFNLDEYYKLPISNDQSYDYFMKENLFNHVNIKPENTHLPNGMADDIEKECMNYEASI

DAAGGIDVQVLGIGRNAHIGFNEPDTKFAKRTHVVELTESTIEANARFFKSREDVPKKAV

SMGIGSILKSKKILLLASGEEKADAVYNTVYGDITPEVPGSILQLHKDTIVIVDEAAASK

LNPKDYKLV*

>CD630_10120 Clostridioides_difficile_630_NC_009089 membrane protein

MKKALRYLLFLIILLLMYFLAVKLNYDFYTQFYTGYMQDFKRYIFINLISSGGIGLLLGT

ELLIREYKKDGSWYIDIPRLLLLCFPSFLLSLMPVFFFMFPVGNIPIIGNFIMLDRIPLN

IIIFNILFGYFLITSFRKK*

>CD630_10150 Clostridioides_difficile_630_NC_009089 two-component sensor histidine kinase

MKEQLIFNITNLVTVVFEAFVIHMFLSDFLGQKEGYIKVVRYAKLGFIICLGFCNLITLN

PKITMPLIFILIFSTSFLYKGNLKTRLFTTVLLSIFFILSEIVVTSIFVLFVKEGFEIML

ENNSIRVLATILSKIVFLLTCKIICLFKKDVHLDMPIKYWLPLFLIPIFSLFLSVSIFDV

SKFFSLESLKFLSLISSVGILYINFIVFYLFKFIIDKTKLSMKYELLEKEIIHKEELRLS

NECYKIIVEQTDSVVFEWNIKENKSFVSQAWTEKFGYNNACKNIFKEIKDKDLVHSEDKA

IFEGFLESIKKKNMHNQAVYRLKKSNGEYIWCRTSITSIYNDENELLRVVGVIVDVDSDI

KKYEELRTRAESDSLTNIYNKGTFEKLVEETIVMNTGDKKDALFIIDLDDFKEINDNFGH

PFGDFVLKTFADKIQTSFGSKDLVGRIGGDEFVVYMQDYVTEVNLHKKAKELNRVLSDNY

TDLSFSFDASVSIGIARYPQDGTSFFELFKNADRALYSIKASGKNSYCLFEEELYVQ*

>CD630_10160 Clostridioides_difficile_630_NC_009089 MerR family transcriptional regulator

MSQVSKNYFTTGEFAKICGINKKTLFHYDDIGLFSPELKKENGYRYYSYHQLSIFGIISS

LREVKMPLKEIKAYIDKRTPNLLIELLEKKTIDIKNEIEKLNNIQALMESTISFTKNACN

IDANTITLKEHEEEYLVKTPVTYKEQFLGDEEENFLYECINFMDNYELSDYGTIGSIIKG

EDIINKDFESYSYLFTKVNKEYKKYPVSIKPKGLYVTAYHKGSYETIYKTYEKLLNFFNQ

NNLRIGDFVYEEYLLYDISVRDSNEYLTQISAEVKM*

>CD630_10170 Clostridioides_difficile_630_NC_009089 multidrug family ABC transporter ATP-binding protein/permease

MKKLIHFLKPYRVLIVVVLIFTFLQTLGTLYIPTLTANIVNNGVVKGDIDYIVKTGLMMM

IVAGITALSAVLVCKVSANLSSGFCRDIREAVFIKSQDLSINDFNNIGTASMITRSTSDI

TLIGQSVFMFIQLVLPAPIITVSGLFLAYSIDKAMTIIIVVVMFLFMLSAFLVGKKLIKL

FKMMQIKMDNMNRVLREVVTGVRVIRAFNRSHFEKKRFDRTAIDYSETAISINKIFAVLM

PIVMLIMNLGIVSIIWFGGMRVSNGNMEIGHIMALVEYCILILFYLIMGVMVFMYIPRAG

ACADRVNQILDIEPEIVDGNGHKDTVSERGHLVFKNVTFSYAQSEEPVLNNITFEAKSGE

VTAIIGSTGSGKSTIANIIPRFFEIQSGEISINGQDIKKIPQKELRDKIGFVPQKAFLFS

GTIEENIRYGKEDASIEEVKHAASIAQADEFISDMEDKYDSFVAQGGNNLSGGQKQRISI

ARALVRKPEVYVFDDSFSALDFKTDKRLRKALKNEIKDSSAIIIAQRISTIMDANQIIVL

NDGKIVGIGKHKDLLENCEVYKQIADSQLSKEELA*

>CD630_10200 Clostridioides_difficile_630_NC_009089 membrane protein

MILIMTFNDIFKSSFIENVSGFSFVDSALALGSAFLVGLFIYMVYKKTYMGIMYSRPFNV

SLVALTMLTTFVILAVTSNVVLSLGMVGALSIVRFRTAIKDPMDLVFLFWSLGSGIVLGA

GLIPLAVMGSIIMGLILIFFSNKTISETPYILMINCNNEDSEDIATDKIKKVFTKYQIKS

KSVTPEKGIELVFEVRMKDGETSLINDLSQVDGVTNAVLVSYNGDYVA*

>CD630_10210 Clostridioides_difficile_630_NC_009089 spore coat protein

MKDKKFTLLISIMIVFLCAVVGVYSTSSNKSVDLYSDVYIEKYFNRDKVMEVNIEIDESD

LKDMNENAIKEEFKVAKVTVDGDTYGNVGIRTKGNSSLISVANSDSDRYSYKINFDKYNT

SQSMEGLTQLNLNNCYSDPSYMREFLTYSICEEMGLATPEFAYAKVSINGEYHGLYLAVE

GLKESYLENNFGNVTGDLYKSDEGSSLQYKGDDPESYSNLIVESDKKTADWSKITKLLKS

LDTGEDIEKYLDVDSVLKNIAINTALLNLDSYQGSFAHNYYLYEQDGVFSMLPWDFNMSF

GGFSGFGGGSQSIAIDEPTTGNLEDRPLISSLLKNETYKTKYHKYLEEIVTKYLDSDYLE

NMTTKLHDMIASYVKEDPTAFYTYEEFEKNITSSIEDSSDNKGFGNKGFDNNNSNNSDSN

NNSNSENKRSGNQSDEKEVNAELTSSVVKANTDNETKNKTTNDSESKNNTDKDKSGNDNN

QKLEGPMGKGGKSIPGVLEVAEDMSKTIKSQLSGETSSTKQNSGDESSSGIKGSEKFDED

MSGMPEPPEGMDGKMPPGMGNMDKGDMNGKNGNMNMDRNQDNPREAGGFGNRGGGSVSKT

TTYFKLILGGASMIIMSIMLVGVSRVKRRRFIKSK*

>CD630_10220 Clostridioides_difficile_630_NC_009089 membrane protein

LLQKVLWKFLKLVVGLFICSVGIVLTINCNLGLSPWDVFHQGLSNHIGITIGTASIIVGS

IVVIADVVLGENVGWGTVFNMLLIGFFMDLLLYSNLIPEADNLFVGIIMLILGLVLLSVG

MVFYMGSGLGSGPRDGLMVAIQKKTGKSLKLIRGTIEVGALIVGFLLGGKVGIGTIISAF

GLGYFTQMVFGLFKLDCSKIKHRFIVDDIKFIKVYMSGEKKAAVQNIVK*

>CD630_10230 Clostridioides_difficile_630_NC_009089 transcriptional regulator

MDIGEKIKRLRTEKQLTQEELANRCELSKGFISQLENNLTSPSIATLIDILEILGTNLRE

FFNEIDDERISFTKEDMFETEDEDLKYKLKWLIPNSQKNEMEPIIITLYPGGQYKEEKPH

EGEEFGYVLAGSIYVHIGEKKNKVKKGESFYFRPKANHYISNEGKTTAKVIWVSTPPSF*

>PCZ31_RS04925 Peptoclostridium_difficile_strain_Z31_NZ_CP013196 ABC transporter permease

MKRKSFLAYPYVVWSAIFVIIPLILVVFFSFTKESGGGYAFTLENYKEVIDPIYMKVFGR

SILLAGGATLICLIVGYPVAYIISKARVSRRGSLILLFILPMWMNFLLRTYAWVAILGKN

GLLNTFLGWFGIQPLAILYTNFAILLGMVYNFLPFMVLPIYTALSKMDNDLINAAHDLGA

NNMTVFRKIIFPLSLPGVMSGITMVFMPAVTTFAISRLLGGGKIMLVGDLIEQQFTVVGD

WNFGSAISIFMMIVILISMSIMSKLEMNLTKKAVGYYFKIKENNF*

>PCZ31_RS04930 Peptoclostridium_difficile_strain_Z31_NZ_CP013196 spermidine/putrescine ABC transporter permease

ILKLKKITSNIYLALVFLFLYAPIFALVLFSFNDSKSMARWNGFTWKWYGQLLQNESIMS

ALYYTIVIAILASVISTIVGTISAIGIHKMRGKSKKLILNVNYLPILNTEIVTAVALMSL

FVFVKMEFGFTTMLLAHIMFCLPYVILSVLPKMKQLPDNIEDAAMDLGATPIYALRKVIL

PQIKPGIVSGFLIAFTMSIDDFIISFFNAGNGVSNLSIEIYGMARRGIKPEINALSTIMF

AVVLGLLLLANKKESIVRGIK*

>CD630_10270 Clostridioides_difficile_630_NC_009089 spermidine/putrescine ABC transporter substrate-binding protein

MIKFKKAISLIAVGMMIVTLFTGCNKATDSTKVLNVYNVGDYIDESLIDKFEKETGIDVQ

YSTYDTNEMMYQKVKSGSTHYDLVFPSDYMVEKMKNEGLLEKLDFKNIPNMKYLDKSFLN

PVYDKTNEYSVAYMWGTLGILYNKKEVKDPMDSWNILWNPKYKGNIMMFDSVRDTIGITL

KKLGYSMNSVNPKEINEAKNLLMKQKDLVLAYVNDEGKDRLLGGEVAMGMLYSGDAVTLM

EQNPDLDYAIPKEGTNKWVDAMCVPTTAQNKKEAELFINFLLDPENAKVNAEYIGYSTPN

TGALKLLDPEITQNPVAYPSKEVLDKCETFEDIGENIKLYDKAWIELKSK*

>CD630_10290 Clostridioides_difficile_630_NC_009089 cell wall anchored protein

MMFKIFNKSLLKLVVVLSIVLTISNFSLSSYASEITSSTDKVLIIYDSKKETAYNRDILN

IMRTLLGRFSSDIELLKLSNYDGEINKNYYSHIFILGINENSYNNDKNTKNLISSLNSYK

GTICWLGYGIENLLEHKKYNLDYVGKTNNIVSVNYRGKSYNLDEHYVFNIVESKDTSNKV

IGSINDTLNKYPYIINDKNLFYVSKLDLDGVLFYIFCDSLNDIFNIKTFDKGRIFVRIED

VHAFREPKNLVEIADYLSSKNIPFTIALIPAYVNPKNHKVITLSESPEIVKAIKYMQDKG

GTVILHGYTHQYKKEEVSGEGYEFWDGKKDEPLKENMKIFVKDRVLNGLRVCIENGIYPL

AFEAPHYAMESEGYKELKKYFSTYMGQHQNNDKKFSTNTYPYIIRDTEEFNIFIPENLGY

IDPEDKFTFQNIKENLDKLSIVRGFSGGFFFHSYLNIEYLKNTIEYLEKQNIEFMNLRDF

NNWVKVDEIQIRNSGDEIIVNYDKDLDEITKSDTRFKSISNISKVLIFIVSISVLIFVII

FIYFKRIDKKKFLK*

>CD630_10300 Clostridioides_difficile_630_NC_009089 family 2 glycosyl transferase

MRDLNVSEYLFVFSLFSIWSLLLINIILAMGGYIFYFKNFDKEIKEIDEYPMISILVPAH

NEAKVIGRTVESLLLLNYPKSKMELIVINDNSSDNSKEILENIKDRYNNYNFTIINTDSL

TGGKGKSNALNIGYTISKGDFIAVYDADNTPDKNALRYLVQTIVMNDELGAVIGKFRTRN

KNKNLLTKFINIETLSFQWMSQAGRWQLFNLCTIPGTNFILRRSIIEEIGGWDSKAIAED

TEISFRIYKLGYKIKLVPQSITWEQEPETVKVWIKQRTRWAKGNIYVLMKYIKNIFKQGR

NKIVFDIAYFFSVYFLFLTSVIISDILFVLSISKLVEISIPINFFLIWILSYLLFIIEVS

ISLTIEKGEATIENIFIVAIMYFTYSQLWLFVAIKGMIEYLKDIIFKREVKWYKTERF*

>CD630_10310 Clostridioides_difficile_630_NC_009089 cell wall anchored protein

MKKFIISIISLVLFFSNISLIYKVNADETKVKNYKFERDITIDGVIGSNSTFFEVNKNWD

IEEVLLHLNFSKSQILNGDVSSLTVLINNVPIKSIKLNAKTNYKNTLEVLVPKDYIIQGY

NEIKIKTYKTISDKICQDDSNTGNWMVIHKESYISIRYKQKKVENSINEYPYPYAEIENN

HKLDTTIVVPDNMTRGETTAVFNLASAFGKITKNDDLKLDVKLYSEMKNWSDDNIIYIGK

PENTAEEILDILSIKEQTLLSSNCIIKQVDSPYNKNKKMMVVIGSNEDDLIKASNLLIEN

RLSNQVLSSSVLVNKETNIKINREQKLNLGHLTLKDLGYSDFLLEGAFNQQALFDVKIPT

GKVLDDGSKIILNLRYSDNLDFEKSLVTVSINDVIVGSKKLDRSHSNNDKLELKIPKDID

NKNYYQVKLTFNLSIKNSNCVTRESNNPWAYVSNNSYLALSTKENETLSFENYPYPFVRD

DEFNDLTVIMPDYSGSQAMTWMFRLGVTLGANINSHNGNINVIRGKEFSDKYKDTNIVVF

GVPHNNSVIKMLNNNLNIKFDKNYSNFISNDKISFIDDYGKNISTIQLIKSPYNNQKNIM

VISSMNEKNLYLGMDYLLNKSKVNDLKGDTLIIDEYGEVEDLAYNLKSKKEVKDSSWNMS

INKTTKVFLMISFITIIVVMILSMLYIKKYKRR*

>CD630_10330 Clostridioides_difficile_630_NC_009089 UDP-N-acetylglucosamine 2-epimerase

MNDIKVMTVFGTRPEAIKVAPLIKELEKRENIKSIVCVTAQHREMLDQVIETFNINVDYD

LDIMEKGQSLNDITCKILNKLPLILNKENPNIILVHGDTTTTLATSLTAFYNKTLVGHIE

AGLRTYDKYSPFPEELNRQLTGIIADMHFAPTNLARKNLISEGKPNNNIFVTGNTAIDAL

KMTIKENYNHPIIDEIGNDRMILLTSHRRENLGKPMKNIFRAIKRIVDDFEDVQIVYPIH

LNPKIRTIADEIFGKFPEKIHIIEPLDVADFHNFLNKSYMIMTDSGGIQEEAPSLGKPVL

VLRDKTERTEGIEAKTLKLVGTNEDRIYNSVSDLLINKDNYVQMSKASNPYGDGNASKYI

VDIIIKKFNCKYLN*

>CD630_10340 Clostridioides_difficile_630_NC_009089 mannosyl-glycoprotein endo-beta-N-acetylglucosamidase

MARKLIKNLGKSKSVKRVKLLFKKIFITVFIVASIVAIFNITKYFEELYKVRDLKSTKIE

YYMDVADEAGDGKVQLSWKALLAIDMVIHDEDLSNIKKKDTLDIGEKFIVEDKNDKGEKV

YKVKKFNKVLSELKFDSSQKSRARKYMKDLEYTYLGNKQLDSSDEKIKFIKKLEDSAIRE

YIDYGILPSITIGQAILESGWGNSKLTKQSNNLFGIKADKAWKGKSVEISTSEHYNEKIV

ASFRSYNSLQDSVKDHSLFLINNKRYRKHGLFEAKDYISQAQALENAGYSTAEDKKGNRI

YAELLIDVIRSYNLQLIDNKVETK*

>CD630_10380 Clostridioides_difficile_630_NC_009089 UvrD-family helicase

MININYREDQIPIINYDNGTMAVPAVPGAGKTFIITNLVAKLLLEQKHKGGKILILTYMN

SAVNNFKGRIRKILEENKIDDTNSYEVMTIHSLAVKIIKEKPEVVMLSEDFNIADDLQKS

IILNECISRFRFEGGERAFRWFLKEQKDERWKEITLEAWERGFFEFAGNAISELKYKGIS

PDKLEEILSRGHKGMLKIILPIYKLYDRKLKQNGLLDYDDILILAEKTLSLDEGLRKKFQ

TRYKYIFEDECQDSNEIQGNIIKIISSENKNLVRVGDINQSITGTFSSSDPKFFKEFINS

ADFCYRMDMSNRSSKDILDLANTLVKYVTKEFRQKECRHALEDMQIKTVPNGMGYKENPN

PEHYNINVKWYESWKIEIEQTARYVKGIKNKYPDKSIGILVPFNEQVTQVAKELRENNLT

FEELGPNSLNKRKVINNIAYIIDFMLHCDDVEKLIVVLNKVFIDTDNEEEIKYFLEELKN

YSTEEIIYNFEFSKLDNDLIEFKEESDIYLSFKRGIDIIKQILEYPIIRLDLLVLFIGKK

LNLEKEDKAVLDYISFYIKYLVSENINMDLEDVYNLLFDVKNRVFNHIIDVVYEINGYEP

EPGSITLCNYHKSKGMEWDCVFLLGLVEYNFPDNINQKFQSDKWYLKEKYKNPMAIIKSE

VEAILKGSISTDYAHKTKIDSINEKIRLLYVGITRAKEMLVLSGSAYRDESDIGNKRKEQ

KPCIYLSRLNQHIIEKRSN*

>CD630_10390 Clostridioides_difficile_630_NC_009089 hypothetical protein

VNDKLKYFSYSQNSINTYKSCPLKFKYKYIDRINWKNDDVGSREYYETLKTGRDFHLICE

RYFSNIPLGIYFNEDDKNSKKFLKWMENIKKVVPIEKGKTYLPEYEVRMTLNGDIIQAKY

DLVVIGNNNIEIWDWKTESKKIDYKHVENRIQTIVYMFLAKEVIPKVLKMDINVKDINMR

YYQPEFDDLPITILYDEEKHEANRNKIQNYISMIKNTNYEEHTYEDNLYNDIEKVYNKEE

RMCYRNKKHCKYCEFNKLCNGKEIDYSILEAEIYGT*

>CD630_10410 Clostridioides_difficile_630_NC_009089 ATP-dependent helicase/nuclease subunit A

VSSPKWTKEQLEVIESRECNLLVAAAAGSGKTAVLVERIIQMITSRENPIDIDKLLVVTF

TNAAASEMRERIGDAIGKALDENPENKHLQNQLVLLNKSSITTIHSFCLDVIKSNFHRIN

LDPNFRIGDQTECAILKQEAIEEVFEDLYEERDEGFLNLVESYAERGGDKEVQDIILGIY

SFAMASPEPKKWLIDSAERFNIDENFDFSQSIWARAILDTVKIEINGLCLNMERALKEVE

SIEELETFAEKLSVEYKKIADISQACNKSWDEAYKKMASMSFENYVKGVKRISKDAPSYI

KESKEKAKTIRDKTKKSLESIVSATFNKDNDSIREEIKYLYNIVKPISSVVLRFEEEYSN

KKREKGIIDFNDIEHFALNILTDVDEKGNIVPSDIAVGYRNKFYEIFIDEYQDSNLVQEV

LLKAVANTETPNRFMVGDVKQSIYRFRQAKPELFLQKYNNYNDKKGSSHRKIMLYKNFRS

REEVVDAVNYIFENIMNENIGEIEYTEKERLNLGANFNVDTDEKSIIGGATEIHLIQKDN

KLDDDIINDKDDRINNKENEIEEEEKLDNIQLEARMVGNIIKDLMKVNEDGKIQKVYDKG

IDGYRPVEFRDIVILLRATSAWAPVFADELMNMDIPTYADVGVGYFDTIEIKTILSLLQI

IDNPMQDIPLISVLKSPIFGFTPEDLIDIRVQSKDKIFYEVLKSTAEYDGFTDSQNENES

EFIPSEECINKSKDFLIKLKEFKEKSMYMSTDEFIWYLYTRTGYYAYVGALPGGSQRQAN

LKVLFERAKQFEETSLKGIFNFVNFIEKLKKSSSDMGSAKTLGENANVVRIMSIHKSKGL

EFPVVICSAMGKNFNTQDFKKSILYHHNLGYGPQFVDYERRISFPSIAKEALKSKINIEN

LSEEMRVLYVAFTRAKEKLIITGSTRNIQDSIKRWSNGIESLDTISQYEILKGKNFLDWI

MPCVLRHRDLSNLLEEVGLDAVFNVEHNSKWYGKLWNKNDILVEKKSDEEKESIEEILEK

IDVNNPDSDYYGEIEEKLNYIYPYEFSTRKPATISVTEIKKIQNNYEEELINTIFEQKVI

LKKPLFIQNEEEREKISGTERGTIVHLVMEVLDLKNVSSVNDIKSQIRGFVSKGIITEKQ

ASIVNPYKIYKFFASNIGKRMLNAEIINREKSIYAQVNMKDIYIYEKLINNDDKKLYDNE

SVMLRGIVDAYFEEDNQIVLVDYKTDFVNEENINQIIEKYKKQLDLYADIIETLTGKSVK

EKCIYLFGVDEAVCY*

>CD630_10420 Clostridioides_difficile_630_NC_009089 nuclease SbcCD subunit D

MRFIHTSDWHLGKSLEGHSRIEEQAKFCEEFIKIVESNEIDMVIIAGDVYDTSNPPAQAE

KLFYQTVSRLANNGQRCVLIISGNHDNPERLSAITPLAHEQGILIYGYPLSATIEAKYKG

FEITYATQGCTKLNINGENIVVATLPYPSEKRLNEVFSSEDEFEKQKNYSEKVGDIFRSL

EENFRSDTINIAVSHIFVIGGESTESERPIQLGGSFLVERKDLPEKAQYTALGHLHKQQK

ASERLNAYYSGSPLQYSKDERAYTKGAYIVDIKAGEKPIIEDVYFNNYKPIEVFKCNGIE

EALDICEQNQDREIWSYFEINTDEIISQNEIKKMKELLKDIIEIKPIITSCYEQESVDIK

EKSMAELFREFYSFSKGVEPKGELMDLFLDIISEEGESADETN*

>CD630_10440 Clostridioides_difficile_630_NC_009089 membrane protein

VLESPNNLIQIIFLIVLLIGSAFFSASETALMSLSKIRIRYMQDEGVKGAKLVSSLIENP

NKLLSSILVGNNVVNIAATSISTSLFIGLMGEKGVALATAVMTVLVLIFGEITPKTIAAN

NSEKVSLLVSKPIKAIIFILRPIVWIFNIITNIIFKLFGITNKGAKSFITEEELKTMVNV

SHEEGVLEMEEREIINNVFEFGDMQAKNAMVQRIDMVAIDMEDSYDEIIQVFKTEKLSRM

PVYEETIDDIVGILNIKDIIFLSDEEIESFDIKNYMREPFFTYEFKKITQLLEEMKLEKS

QMAIVVDEYGGTSGLLTIEDLVEVIVGDIEDEYDEEEDEIQVIKEDEYIVDGSTKIGDVN

ELIGVNLESEEFDSIGGFIIGHLSRLPEENEVIEVDNIRFCIESIEKNRIKKIRIYT*

>CD630_10460 Clostridioides_difficile_630_NC_009089 peptidase T

VKQKVVERFLKYVSFDTTSNSQCENCPSSEGQRVLAKYIVEELKTMGVDDVSLDENSYIM

ATLKGNTDGVDTIGFISHLDTIEDVSGKDIKPRIIENYDGKDIVLNEALNVITYVKDSPE

LEEFKGDDLIVTDGTTLLGSDDKAGIAEIVTAIEYLINHPEIKHGDIKIGFTPDEEIGRG

ADLFDVEKFGAKYAYTLDGGIVGELECENFNAANATITIHGRNVHPGSAKNKMVNAIHIA

AEISEMFPADERPETTEGYEGFWHLNSIGGNVENVSMAYIIRDHCKEKFENRKSIMIENI

EKINKKYDNRVELDLKDSYYNMKEKIEPVMFIVDIAKEAMEELGIKPRLVPVRGGTDGAR

LSFNGLPCPNIFTGGLNFHGKNECIPVSSMEKATKLIVRIAEKYAERV*

>CD630_10470 Clostridioides_difficile_630_NC_009089 cell wall-binding protein

LKIKSKKIIAFALSLGIMLPSCVYADTISKNQNAQVDMNNYLQGRKSPKEEKLIGEDRFD

TAIKISQSGWNNGSERVFLVNSNSLPDALASTPLASKLDAPILLTNKNSIPYNVTEEIKR

LNPAEVILIGSEGAISSSVKESLEDMEVLVSRIGGADREETSLLLTRQLDDTGDLGVSKV

AVVNGYNGLADATSISSPAASDNTAIIYTGKDSIRSEAKNFITQNSTKTYIVGGEYSISK

KLEGQLVNSERLAGTDRKDTNAKVLEKFYGKSSKVNNMYFAKDGSGREADLVDGLAAGVL

ASKTKSPVVLASGSLSSAQKSFIKKVKADKFVQVGGGKNSKPYAEALALQ*

>CD630_10480 Clostridioides_difficile_630_NC_009089 phosphodiesterase

MKIGIMSDTHGSLLYFEKALNVLSDCDVLLHGGDVLYHGPRNDIPEGYNPKKFIETLNKL

ENIVIVKGNCDADVDQMVIEHPIQSPYVMSQFGEIRIILNHGYIESEEEIIDKAKKMGGD

ILVLGHTHVKKLYMDDNLIVINPGSTSIPKDGSHSVAIIDIIKTDDEDELELDINLIDIN

TGNIININD*

>PCZ31_RS05070 Peptoclostridium_difficile_strain_Z31_NZ_CP013196 ABC transporter ATP-binding protein

VGIKKCIRGEDVFSIRNITKKLGKFKLNNINLELKEGDIVGIIGPNGSGKTTLIKIIMGI

IDADEGEIELCNETIENSPISFKNNIGFVYDSLQFYPHLKVKEFRKIVSLFYKNFDRERF

DEYLNKFDIEENMHIENLSKGQSEKLMLSSALSHNAKLLILDEPTAGIDPIVRTEIMQYL

QDFVKNGSSSVIISTHNTDNLIKIADYLVFINRGNQIFTVKKELIEQEYKIIRANKAELE

AIKESIVGVKEYKYYNEALVKVGDSLKVKSLLIEIDKHKVKNPTIEELMYYYVNEVR*

>CD630_10510 Clostridioides_difficile_630_NC_009089 multidrug family ABC transporter permease

MFNLIRKDLIIGISSDGIRNLKYILLFFVFYFFLNSISYYTVSIVVSYLIFINTFECDYE

NDSRIFIRSMPVSIEDIVYSKYLLGVGLIISVTIIVSLLSKLTSLVFFRNMVLNDVFFSV

NIFLAILTILLPLIFKFGYGKMKVCGFIVSILIYFVYGSLLKMISMIVYQVKHFNYSKVG

GVYLSNYVTDIANTKYINLYSMTFLTIIIFIISMYFSIKISKKNKFNY*

>CD630_10520 Clostridioides_difficile_630_NC_009089 hypothetical protein

MKKFIKVSFFILGIFTLFIAINKLVYRDMVSVKDYEINHLEEIVFDFDSRRSRDVGTIVR

FKISNNSKYSYKLNSAKMKFENCVENKKGMNKCSAYVNVDLFNEKNSITERTLKYGIKPN

NQGYVDFVIPKGLNFDYRYFNESGMSVEYKGEYIVNIPMAKGLYLVVGKNNDTWNANIME

*

>PCZ31_RS05085 Peptoclostridium_difficile_strain_Z31_NZ_CP013196 EBSC protein

MSISNVREYFKQFGKEDSILEFEQSSATVELAAEAAGVIPARIAKTLSFKIGDDAILIVT

AGDAKIDNKKYKAEFNCKAKMLTPEEVLEFTGHAIGGVCPFGLKNSIKVYLDDSMKRFDT

VFPACGSSNSAIELTCEEMEKFSKCEKWVDVCKIGKKI*

>CD630_10540 Clostridioides_difficile_630_NC_009089 butyryl-CoA dehydrogenase

MDLNSKKYQMLKELYVSFAENEVKPLATELDEEERFPYETVEKMAKAGMMGIPYPKEYGG

EGGDTVGYIMAVEELSRVCGTTGVILSAHTSLGSWPIYQYGNEEQKQKFLRPLASGEKLG

AFGLTEPNAGTDASGQQTTAVLDGDEYILNGSKIFITNAIAGDIYVVMAMTDKSKGNKGI

SAFIVEKGTPGFSFGVKEKKMGIRGSATSELIFEDCRIPKENLLGKEGQGFKIAMSTLDG

GRIGIAAQALGLAQGALDETVKYVKERVQFGRPLSKFQNTQFQLADMEVKVQAARHLVYQ

AAINKDLGKPYGVEAAMAKLFAAETAMEVTTKAVQLHGGYGYTRDYPVERMMRDAKITEI

YEGTSEVQRMVISGKLLK*

>CD630_10550 Clostridioides_difficile_630_NC_009089 electron transfer flavoprotein subunit beta

MNIVVCIKQVPDTTEVKLDPNTGTLIRDGVPSIINPDDKAGLEEAIKLKEEMGAHVTVIT

MGPPQADMALKEALAMGADRGILLTDRAFAGADTWATSSALAGALKNIDFDIIIAGRQAI

DGDTAQVGPQIAEHLNLPSITYAEEIKTEGEYVLVKRQFEDCCHDLKVKMPCLITTLKDM

NTPRYMKVGRIYDAFENDVVETWTVKDIEVDPSNLGLKGSPTSVFKSFTKSVKPAGTIYN

EDAKTSAGIIIDKLKEKYII*

>CD630_10560 Clostridioides_difficile_630_NC_009089 electron transfer flavoprotein subunit alpha

MGNVLVVIEQRENVIQTVSLELLGKATEIAKDYDTKVSALLLGSKVEGLIDTLAHYGADE

VIVVDDEALAVYTTEPYTKAAYEAIKAADPIVVLFGATSIGRDLAPRVSARIHTGLTADC

TGLAVAEDTKLLLMTRPAFGGNIMATIVCKDFRPQMSTVRPGVMKKNEPDETKEAVINRF

KVEFNDADKLVQVVQVIKEAKKQVKIEDAKILVSAGRGMGGKENLDILYELAEIIGGEVS

GSRATIDAGWLDKARQVGQTGKTVRPDLYIACGISGAIQHIAGMEDAEFIVAINKNPEAP

IFKYADVGIVGDVHKVLPELISQLSVAKEKGEVLAN*

>CD630_10570 Clostridioides_difficile_630_NC_009089 3-hydroxybutyryl-CoA dehydratase

MSTSDVKVYENVAVEVDGNICTVKMNRPKALNAINSKTLEELYEVFVDINNDETIDVVIL

TGEGKAFVAGADIAYMKDLDAVAAKDFSILGAKAFGEIENSKKVVIAAVNGFALGGGCEL

AMACDIRIASAKAKFGQPEVTLGITPGYGGTQRLTRLVGMAKAKELIFTGQVIKADEAEK

IGLINRVVEPDILIEEVEKLAKIIAKNAQLAVRYSKEAIQLGAQTDINTGIDIESNLFGL

CFSTKDQKEGMSAFVEKREANFIKG*

>CD630_10580 Clostridioides_difficile_630_NC_009089 3-hydroxybutyryl-CoA dehydrogenase

MKLAVIGSGTMGSGIVQTFASCGHDVCLKSRTQGAIDKCLALLDKNLTKLVTKGKMDEAT

KAEILSHVSSTTNYEDLKDMDLIIEASVEDMNIKKDVFKLLDELCKEDTILATNTSSLSI

TEIASSTKRPDKVIGMHFFNPVPMMKLVEVISGQLTSKVTFDTVFELSKSINKVPVDVSE

SPGFVVNRILIPMINEAVGIYADGVASKEEIDEAMKLGANHPMGPLALGDLIGLDVVLAI

MNVLYTEFGDTKYRPHPLLAKMVRANQLGRKTKIGFYDYNK*

>CD630_10600 Clostridioides_difficile_630_NC_009089 pseudouridylate synthase

LFKKENQRYNLISYTNEEEMTLKEVLLDKLNFSVRSLSKMKREKSVLVNGVYKKPSLKVY

SGDLIEVKIYEEKANFEPQDLNLQIIYDDFDIIMVNKPPFMVVHPTKSHYDKTIANGISY

YIDNQKENVKIRFVNRLDMNTSGLVIVAKNAYAHHTLSTAMSENKVEKKYITVVDGIIKE

NEGTIDEPIYRPTEDSIKRIIDERGQSSVTHYKVIERLENATVLEVSLETGRTHQIRVHM

AHIGHGIIGDELYGYVDEELINRQALHAYKLEFEQPRTKEKLKFKADIPEDMKELISKLR

*

>CD630_10620 Clostridioides_difficile_630_NC_009089 acyl carrier protein

MFEKIKEIIAEQLGVDNLDEITMEASLMDDLEADSLDAVEIIMALEDEFGIEIPDEEAEN

FKCIGDICKYIEENK*

>CD630_10630 Clostridioides_difficile_630_NC_009089 hypothetical protein

MKYVGLLLTSVGMFLLIAVNFYYNSITLDMQRIEDYVMETNLILEDVAEKESYVSNEKED

YISRLMHVKKGIENSKTSFLIERYKEYKIKSIESLIYTISEEKKDYLDEVDRYNKLGEKE

INKLINKNFLEVTYLSITTYI*

>CD630_10631 Clostridioides_difficile_630_NC_009089 hypothetical protein

MTKLEDNMERFFGGCEGFFGNKMLIIIVIVFLLLCTDILEDLLCDDNIWIWVILIVLLLF

NFDDGCCC*

>CD630_10633 Clostridioides_difficile_630_NC_009089 hypothetical protein

MLDRFFGEEGCFGGGGWWIIVLFFLFLAFEDCWRDIDIMSWIPFLILLLITCSCGGFFDG

EVGCGC*

>PCZ31_RS05155 Peptoclostridium_difficile_strain_Z31_NZ_CP013196 LacI family transcriptional regulator

MKMKGNITIKDVAKQAGVSISTVSRVINDSKPVTDEVKQKVLEVIKETGYIPNPLARSLV

TKKSQLIGVIVPEVSDSFVNEVLNGIEEVAKMYDYDILLANTYSDKEQELKSINLLRAKQ

VEGIVMISWIVEQEHINYIQNCGIPATYISKTARNYDIYTVSTSNEEATFDMTEHLIKKG

HEKIAFIMTSKDDTVLEMERLAGYEKALSNNNIELDKSLIKYGGTDYESGYNSMKELLDD

GIIPHAAFVTGDEAAIGAINAICDAGYKVPEDISVAGFNDVKIARMYRPKLTTVYQPLYD

MGAVAIRMVIKLINKELIENKKIELPYRIVDRESVTERKK*

>CD630_10650 Clostridioides_difficile_630_NC_009089 hypothetical protein

MNIIQYIFIERGFFMKKLDTESLEKMAKQKNIDKDKIEKMADSYKGKSENELMEELIKIG

KNLDGRDEVVSKFKAFLDENQRKKLDNIMEKISDAENQRDTKPAKTKKAKPTKGKSNSPA

PKNTPGSQQKKSKSLFKKTKKSNPHE*

>CDM120_RS05720 Clostridioides_difficile_M120_NC_017174 hypothetical protein

MQDYKKNKRRMTNQPMPTMNEEEVYTEEINSEDMRGFKKSHHHNECNTDNKCDCHDDCKP

CNPCKPNPCNPCKPNPCNPCKPNPCDDNCGCHDHCKCDCEPCEMDSDECFENKCGPECCN

PISPRNFSVSNAVPFAIEANRIFDTMQFQTFTDATGPNGEPLTFETEVVEVFGSVPSAGK

ASVTIEKICLSNDGIVIDTGMTTLEDFDLDPLGDIVGRNCETTFEFAVCGERNAECCRQG

KGKSVAYKQRGLTVAVRNLVLELRGRCGCTEFVALAFPAVRAGGGCKRRVDYVEFTFNTL

SAPICLPADGRAVTLRQEYQTNLTVDCIGKSILKLECNECCEPFYELIIPNDIDLVLCLQ

NTVSTLISEQIVVLASPNPIQPRLVDTFSKVCDFSQCGPNHESGKPSCHR*

>CD630_10690 Clostridioides_difficile_630_NC_009089 histidinol phosphatase/hydrolase

MFYDYHMHSSFSTDGKSTMEEMVKKSIELGLEEICFTDHVDYDVYADDSFSIVYEDYFKS

LETLQSKYKDKISIKKGIEFGVQTQLMDTYKKEAHQYPLDFIICSIHAIDTMDLYLGNYF

KDKTQHEVYENYYLYLYNIVKNYKDYSVLGHLDLIKRYAPYDTILDDRLFSDIIEETLKQ

AIYDGKGIEINTSCYRYNLPDLTPSKYILQMYKDLGGEIITTGSDSHHISQVACEFDYIY

SLLKNMGFKYVSKFNKLKPEFIKL*

>CD630_10710 Clostridioides_difficile_630_NC_009089 hypothetical protein

MFDKKKLDRINELAKKNKEGILSADEIKEREILRKEYLENFRAHFRSRLDSVKVVSPEEY

EQYMKNNKN*

>CDIF1296T_01173 Clostridioides_difficile_ATCC_9689__DSM_1296_strain_DSM1296_CP011968 PTS system transporter subunit IIA

MKKIILASHGDFSKGLLNAVKMIVGDLADCVSSYGLYPGQSASDFALELEKTISEDKECE

YIILSDLYGASVCTAMLRLTNLHNVRLFSGMNLNMVLELLTRFSDELTVEDIDQLVEESR

RGIQSVTLQIKEEEEVF*

>EAA_RS0205150 Clostridioides_difficile_CIP_107932_NZ_CM000659 membrane complex biogenesis protein, BtpA family

ILVYEKKGILSVFKSKKPIIAMIHLKGDTPEDIFERAKKEITIFEENGVDGIMLENYYGN

YYDLERILEYVSKANLSIPYGVNCLNVDTMGFELATKYNASYIQVDSVVGHVKPRDEATL

EEFFKLQRSKCPAYLIGGVRFKYQPVLSENDVEEDLKIGMTRCDAIAVTENATGQETSME

KIELFRKNLGDFPLVIAAGVTLENAKKQLELGDMAIIGSYFKDNYKDFGNVSVEHVKTFM

DEIKKIREEL*

>CD630_10760 Clostridioides_difficile_630_NC_009089 PTS system mannose/fructose/sorbose transporter subunit IIB

MIKLVRVDHRLIHGQVAFTWTKFLSTDCILIASDDLLKDELRMAGLKMAKPSNVKLVMKS

IADSIKALNSGVTDKYNLLILCESVEDVYRLAKEVKAIKSINLGGTKSDDNRENISKAVH

VSKDDIKMIKELDSEGVNVFVQLVPDDDATNVMKLI*

>CD630_10770 Clostridioides_difficile_630_NC_009089 PTS system mannose/fructose/sorbose transporter subunit IIC

MKMEFTQVILITLIAFFAYMHSFVGSTMHNRPIVVAPLVGLALGNLHTGIVIGSTLELVF

MGAFPVGASNPPDFVSGSIIATAFVILTGQDVSAAVVLAVPIATLVLLIDNFLMTVVLTW

GAHIADRYAEEGNIEGVERVQLLFGIGNKLILAIIVGIGFSLGVPVIEKILSFIPSYVTH

GMDVAAGVIPAIGFAMLARMMLNKKTVAFLLLGFILVAYLNITVTGVALFGLAIALIYVN

FVGEKEVIVDDNEF*

>CD630_10780 Clostridioides_difficile_630_NC_009089 PTS system mannose/fructose/sorbose transporter subunit IID

MTTSSKKLETISPDSKITRKDFWKCFRRSLTLDSSWNYERMQNIAYAYMMAPIIRRLYKD

DKEKKSKALKRHLEFMSVTPHISTLLVGISGAMEEENAKNKEFDANSINAVKSSLMGPVS

GIGDSFFWGTLKLIAAGVGIALASQGNIMGPILFLLIINVPHFIIRYICLDKGFKYGTQF

FKDVSGSSIVSKVMEAASMLGLMVIGGMTASNVMLKLSVNVGSGEWAEPIQTYLDQIMPC

MLPAMIFGIMYWLLGKKVKTTTILISVMIICIVLAAIGVV*

>CD630_10790 Clostridioides_difficile_630_NC_009089 LysR family transcriptional regulator

MDFKQLEVFVAVAKHQSFSKAARELFLTQPTVSAHIQNLERELETVLINRSNKVITLTKS

GEILYEHAIYILNNCKRAIYDIKEYSGKIEGIIDIACSSIPETYILPDFMKSFSMSYPDV

KFSISHYDSQYAISEILNERISFGLVGSKINNPQIEYLDLLDDELVLITPSDFKIDNKNN

CIDIGELAYLNFIMRKEGSGTRNLILNTLSKNNFPVSKLNVIAHVESNEAIKEMVRLGLG

VSFISYISAIDYLNAGKIKCYKIKDVDFTRKFFFIYSKKKTFSPLEDKFLNRLCEYFEII

I*

>CD630_10800 Clostridioides_difficile_630_NC_009089 lipoprotein

MKLKRSLVCVTILGIILVGCHKENTKEKNQVASKATQQKTMTKVQNDVNEIMNKDYKYII

KNMGIPYNTFYYIKPKVLKESNTMQDINTSSYMTLVYPKYTGNDELDGSALYVDINENKV

VNVETNSFSSKGISVIDAESGIVIEKSDHEKSAVSLENFRHIDLGEYVGVEDSRINEIVG

DANYDLTAYNHEGSKVVKSYRLKEDNKILKKEVLTISIVDNKIKSIKTIESDKIVKIIKG

TLLE*

>CD630_10840 Clostridioides_difficile_630_NC_009089 multidrug family ABC transporter ATP-binding protein/permease

MIKQFVKYYKPYKKIFTLDLIAAFLFSLCDLVYPMITRNIMDDVVPNKNLRMLVVFAVAL

ILIFIAKAGLNYFMQYWGHVIGVDMQADMRNEVFTHLQRLPNTYFDNNKSGVTMSRIVND

LMDITELAHHGPEDLFISIVMLVGSFFILIDINIPLTLIIFAILPFIIWFAIAKKDKMNI

AFMKSRVTIGDVNATLENSIAGMKVTKSFCTEKEELNKFVRSNKLFRRARQDSYKVMAEY

YSGMNLYMDILEWVVVIAGGYFTYIGKITLGDFAAYILYVKMFIQPMKKLINFTEQYQNG

MTGFKRFIEIMEQDHQKEAKNPIELENVKGDIEIENISFTYEDKTQVLDNLSLSIKAGKT

IALVGPSGGGKTTLCNLLPRFYEFDKGDIKIDGKSIKDVSLKSLRKNIGIVQQDVFLFTG

TIRDNILCGNPNATDEEMIAAAKKARIHDFVETLPDGYDTYIGERGVKLSGGQKQRISIS

RIFLKNPPIIILDEATSALDNVTEREIQESLEELSKDRTNLVVAHRLTTIKNADEIIVLT

DKGIEERGTHEELVNKNGVYSRLHNN*

>CD630_10860 Clostridioides_difficile_630_NC_009089 peptidase

MDIKEITKSYKDYVIKLRREFHENPEKSMEEVRTSKRVKEELDKIGIPYVSAGGTGVIAT

IKGANPGKTVALRGDMDALQVVECTDVEYKSKNEGLMHACGHDGHTSMLLGAAKVLNDIK

DSINGTVKLFFQPGEEVGKGARAMIQDGAMEGVDSVFGIHLWTDVESGTISVEEGPRMAS

ADFFKITVKGRGGHGSLPHQGVDAVLASSAIVMNLQSMVSREVSPLEPLVVSVGVLNSGT

RFNVIASEAVLEGTIRLFNPELRKQIPGILERIAKSTAEAYRADAELEYGYLTPAVINDK

ECSKIATDAAIKLFGEDCITLFEKVTGAEDLAEFMNIAPGALAFVGARNESKGACYPHHH

GCFNIDEDALEIGTALYVQYAVDFLNK*

>CD630_10870 Clostridioides_difficile_630_NC_009089 zinc transporter ZupT

LNNVIFAFLITLLAGLSTGIGSCIAFFAKKTNTKFLSISLGFSAGVMIYVSMIEIFPKAQ

DALTKSMGEKLGSWSTVIAFFIGMAIIAAIDKLIPQEENPHEIKKMENIEEKNIKKNKSL

LRTGIFTAMAIAIHNFPEGLATFISALDDVTIAIPIAIAIAIHNIPEGISVSVPVYYATG

DKKKAFYYSFLSGMSEPLGAIIGYVLLRNFLNDITLGIVFAIVGGIMVFISLDELLPSAR

EYGEHHLSIYGLIAGMGVMAISLLLFK*

>CD630_10890 Clostridioides_difficile_630_NC_009089 VirR-like two-component response regulator RgbR

MIKIAVCEDEKETQLLIEDYLENILKDISIEYEIQKYISGEELLESNLKDIDILLLDIKM

EKLNGMDTARKIREVDNEMEIIFVTSLIDYVQEGYEVRAYRYLLKPIELEELKKHVLTCI

KDIEINKESHITIKNKSNTYKIYLNEIKYIEVQKKDMLIHTINKNFDIKYSLGKIEKELN

PYKFIRCHKSFIVNLRYVENIKPNTAILESGEEVPISRYRYKEVKEKFLKFLGDTIC*

>CD630_11190 Clostridioides_difficile_630_NC_009089 lipoprotein

MKVKRNINRVAIIVALNLSLALMVGCMSPVSDKNLSSSEQISKDSLKEQVEDKKDESIEL

FNNYSQKINDAKGREEVKKDLSQRFSKAIIDSGYSITNSKSDGSFVVSINGAEKPPEKNI

RSLFYSMSEDDKEGKVTIDILCAKEYPEDDKLSESDKYVKFIYNLFKSLTDTNLTEKEIF

SMVEEDFNKGVGVVELPYMKDIHVEVNKVKQSTKVLKLSLVYKFDISNH*

>CD630_11200 Clostridioides_difficile_630_NC_009089 formate acetyltransferase

MSQTNRIEAFRQEYINSKPMICCERARIFTESHKKTEGEAICIRRAKAFLETCKELPIKI

FENELIVGTAGKFRRTGILTPEFSWQWVDKEMDTFDKRTQDPYVISKEQIEFIRKEIFPY

WKGKSLEEVFLARIPEDTAKILVDTGIIDNDSKWRQAVGEVTPDYQDILFVKGYKGIKED

ADKKIKELDISVSENIEKIDFYKSVSIVAQGIMTLAQRYSNLAKEMSKQETDEKRKLELI

KISEICMNVPANPPTNFYEAIQFVWFVQLGGILSENPLALNLGRFDQYMYPYYENDAREG

KITESEAQELIEALWIKLSEWVWTISANTANYFAGYNQFQNLTVGGKKRNGTDGTNDISY

MCLKATESVKTHQPGLSVRVSQGAPDNFVMAVAKLVKQGTGFPAIHSDSAGAQMLLQDGY

DAEDARDWSNCGCVVPHFRKTGQWTSAVNINFAAALEYAMNEGKSRLTGEKMGLDTKNIT

EFTSFEELKDEFLKQLAYLVKSSVIGTTVAQQIHKEMVPRPFLSTCVDGCLDKGVDLSKG

GAKYNIGPVLTGIGLGVVSNSLAAIKKLVFEDKVTTLEELTKALNNDWEGYEELRKLALD

VPKYGNDNDYVDSLAIEVSDFYYTETRKYKDIFGSKFNSAFMGISNYVPTGKIVGATPCG

RKATKPLTEGVSPFVGTDTTSPLAAMKSASKINHDVHTGGTLLNLRLNQDLVETERGLRN

LTSMIKSYFALGGFHVQFNTISNDTLLKAQENPEEYKDLLVRVAGYSTQFVNLSREMQDA

IIARNSHSNF*

>CD630_11210 Clostridioides_difficile_630_NC_009089 pyruvate formate-lyase activating enzyme

MSKKGRVVKVQHFSVNDGDGIRTTIFLEGCKLKCKWCSNPDSWSNIVKLGVMKDKCVSCN

RCIDVCPQNISSLFDRTQINNKCDLCGECIKVCLKDAICIMTEEMSVEEIVEEVEKDFIF

FFESNGGITFSGGEPTLQIDFLRELVDIFYDKGINIAIETCGYFDWNKVNDVFEKIDHIF

VDIKSMDDNIHKEYTGVSNKIILDNICRLSKLNKSMVIRVPIIYGVNDSEENIRNTALFV

KQNVPGGKMELLPYHKFGIDKYKALGLEDYIYEFDEICNNHMLKLKEIVELTGVKIIEYK

*

>CD630_11230 Clostridioides_difficile_630_NC_009089 phosphoesterase

MIIKVSMTVLVLILLYILYKYETQHIETTEYTIFNKKIPKEFDNFKIVQVSDLHNKVFDK

NNKVLIDKIESLNPDVIFITGDLVDGENKNFQVALDLIDNLVEKYEVYHIIGNHEQKSLI

KKYKHLYKDYFKELYSKKIINMENESVRIYRGNSHLNLYGLIIPLECYPYLFANNKKLEL

SDNFVEDNLGKVNENEYNILLAHTPFFFEKYAKWGADLVLAGHVHGGIIRIPFVGGVLSP

NREFFPKYDWGKYEKDNSTMILNKGLGGSKVLIRLNCKPEIVKITLKYEK*

>CD630_11240 Clostridioides_difficile_630_NC_009089 hypothetical protein

MGDTSRFYESVKNKYPKLKDGTRVHLWPKENIIIGPIYDEEDGYKKSIGNVDSLSWKQIV

ILAECDGKNTTDDIINILSNRYRNIHNMKEKVMEFFMFYENVYLTFEDDIQTLSSVFEIT

GNKSYITPLYFTIEIDGDNNTNDYFSEITSLLNCMYEKGCRFIEVIGEDILKNKSMREIF

QYMLDHFDLIVVTKDSFTIDRSLIKELDNYRHKVIWKVYSKNDDTRIKLKEDIKITSLIK

RGHTVVRGDREKAIKIEDMRFKDTKERFSKYGAEWSHLYISSDGSVKSYSFQKDKRIYLG

NIFNNSVEEIFLEMQNKIDKLEIVYQ*

>CD630_11241 Clostridioides_difficile_630_NC_009089 hypothetical protein

MNVNNHKCPECGEHSKKFEIVEENEDCVIFECYTCGTISILKYCENCKEFVFVNIPNNVE

TYEYCCGKCNNKI*

>CD630_11260 Clostridioides_difficile_630_NC_009089 AraC family transcriptional regulator

MEWINKLNQAITYIEENLESGVDYAEAAKIACCSTFHFQRMFSYIAEVPLSEYIRRRRMT

KAAFELQNSNIKILELSEKYGYDSPTSFNRAFQNIHNISPSAARAKGVVLKAYPKMTLSI

YVKGNIEMQYRIVEKKGFRIVGIKESMNMIVEECFEKVPKLWAKCIKNGTIDKLSELINN

EPCGLLGVSVCTNSKGLDYYIAAPTDKPVLEDTHEYLIPSGTWAVFECVSPMPNALAIQE

LQKRIITEWLPSSGYVYANLPDIELYPNGDINAPDYTTEVWIPIQKPS*

>CD630_11270 Clostridioides_difficile_630_NC_009089 membrane protein

MKKNMLYVGIIYFICGILLVLLATFTEFSFEAFIWGLSGAALGPGVCMILQYMYWSKPER

AVDYEEKIKNQRIEMNDERRIMLRDKAGRITNQIMSYVLVILIFIVSILSVFSVMVISKW

VLVVLGLLILFQFICSVVVYNKLDKKL*

>CD630_11290 Clostridioides_difficile_630_NC_009089 dephospho-CoA kinase

MLILGLTGGIGCGKSSLSNIFRNLNIPIVDADIISRKIFEDKLLLEKVFVHFGQSIKNDD

GTLNRKALGKIVFSDEEKLKELNNLTHPRIREKIISEIEKLRKKGENIVVLDAAILVESG

FLDMVDKLLVVTCKQEVQISRIQKRDNCSEQEALSRINSQMSQEEKSKYGDYIIDNSGTI

TELESKAHKFIEYMKENWRE*

>CD630_11300 Clostridioides_difficile_630_NC_009089 lytic transglycosylase-like protein

VNSKKVLILSIFIILFGALLMESKVIHKFLYPKKYSEYVEKYSKEFNLDENIVYSVIKAE

SKFNSSAVSKKEAKGLMQILDITRDWGAEELNLKNVDIFDPETNIRLGCWYLSKLYKEFG

KLDLVIAAYNGGSGNVKKWLENNEYSKDGENLHDIPFKQTSKYVEKVKNNYEHYNKIYGK

KGKN*

>CD630_11320 Clostridioides_difficile_630_NC_009089 heavy-metal transport/detoxification protein

MKKKLLVEGMSCGHCVNHLKTALTEDIDGIEVLDVDLENKCASVDMKDDVSIEKLKEVIA

ELGFELKGIE*

>CD630_11330 Clostridioides_difficile_630_NC_009089 hypothetical protein

MESRGYKEDLKKMILDMAHKELESYVSQKGAKKYFEGNLDKIINNNIKKFNQSIGLNRVY

LKMLKKCAYQESVTSISRTIKKEALFKSKKELFAFSEYLKLDINKRLSYNQILRKISRYI

YYNRSSYAQKYVVFKRGDEEYLLEPEKIKDELISSYRSKTREDMRSIAKLLDIEVEDDYN

AEDIRKKVINYIIKEKLKS*

>CD630_11340 Clostridioides_difficile_630_NC_009089 lactoylglutathione lyase

MKFNFCHNNFNVTNLEKSLDFYKKALGLKEVKRKEAEDGSFILVYLGDGITSHTLELTWL

RDWDRPYNLGDNEFHLALEVEEFDEAKKLHKDLDCICFENESMGIYFIADPDNYWIEILP

KNH*

>CD630_11350 Clostridioides_difficile_630_NC_009089 SH3-domain-containing protein

LLGGVIVVKKAIAALGIGAVAVSVSSINASALEKGTVTASALNIRSGPSSDCDKVAKLYK

GKTVEILEKSNGWYKVRVSSSVVGWGSAKYISTSGSSEGTSSQNNSTSSGTTISGNGKVN

VSSRLNVRSGAGTNYSLVGKANNGDVVKLLEQSNGWYKIKLSNGVTGWASSQYISKTSED

VGTNNSSNSNSTNNSDKKPSSEESIEGKNGKVTSAVSLNVRSGPGTSYSIIGKLNGGDVV

ELKAKSNGWYKVKLSSGTIGWVSASYISETNEDTKEKPNSSSNQNSQSNSNSKPSFTGNS

DKSTAKGSTIVDFAYTLIGIPYQWGASGPDKFDCSGFTQYVFKHSVGVSIPRVSREQANF

GSAISMGNYAPGDLVYFDTDGDGTTNHVGIYVGNSKFIHCSGTQTNPNKVKVDNLTSSYW

SKVLLGARRFV*

>CD630_11370 Clostridioides_difficile_630_NC_009089 electron transport complex protein RnfC

MKLLTFKGGIHPPYRKEYSNTKALEKAQAPKIVYIPLQQHIGAPAKPIVEVGDEVKFGQK

IGEQQGFVSCNVHSSVSGKVIAIEQHEVPGGSAQCVVIENDFKEELHESVQPKGQLEDLS

KEDIVGIIKEAGIVGMGGATFPNHVKVSPPPDSKAEVVILNGAECEPYLTADHRLMVENP

EDVVFGLRALMKVLDVKKGFIGIETNKPDAIEAIQNVAKDYSEIEVVGLQVKYPQGAEKQ

LIYACTGKEVPSGGLPIAAGAVVDNVATAAQIAKSIKTGMPLVERITTITGSCIKEPKNL

ITKVGTLVSEIIEQCGGFKEDKKIGKVIMGGPMMGIAQYTTEIATNKGSSGILCLDEEES

RTPDIQNCLRCGRCTDVCPSFLQPLFISAYSLKDDYDTAEYHRAMDCIECGSCSFICPAR

RPLLQSIRSAKREIGAKRRKQAAQK*

>CD630_11390 Clostridioides_difficile_630_NC_009089 electron transport complex protein RnfG

MNSMVRLGGTLLAISAIAALALGATNQVTAPVIEQRNIQANNELRKAVLPEAKEFKEMKE

DTYKGLGDDLIAEVYEGLDGSEVVGYTLKAKPSGYGGEIEVMVGISSEGQVTGVDIGNMS

ETAGLGAKAKDDAFKGQYKGKTAEPLEVAKGSTTADNQILAISGATITSTAVTTGVNAAI

DVFNSALNK*

>CD630_11410 Clostridioides_difficile_630_NC_009089 electron transport complex protein RnfA

MNLILLFLSIVLVNNVITSQFLGICPFLGVSKKVDTAVGMGVAVTFVLTLASIITYFIQI

LLVKTGTGFLQTIAFILVIASIVQFVEMVIQKMSPSLYQALGVYLPLITTNCAVLGIALV

NVQKSYNLVETIINGFGAGAGFTLAIVIFAGIRERLELADIPEAFKGFPITLISAGLMSI

AFLGFTGLIKL*

>CD630_11420 Clostridioides_difficile_630_NC_009089 electron transport complex protein RnfB

VKMVILTAVLVLGIMGLIFGIVLDFASKKFAVEVDERVEAILGVLPGANCGGCGFPGCGG

LANAIVEGNAPVNGCPVGGADVGAKVGEIMGISAEAGEKQVAKVICKGTCSSAKDKYEYE

GISDCRAANVLNSGAKMCKFGCLGLGTCKDACKFDAISIVDGIAVIDEEKCVNCGKCKEV

CPKGIIITKPESQEVVVECNSKEFGKAVKEKCTAGCIGCGMCVKACKFDAIIFEDKIAKI

DPNKCVGCMQCVAKCPTKVISGDITKKKKVTIDQELCVGCTVCKKQCKFDAIEGELKEKH

KVDADKCVGCHLCMEKCPKKAIKIL*

>CD630_11440 Clostridioides_difficile_630_NC_009089 DNA repair protein RadC

VIYLKKSFNSTIKVKEMAPEERPREKMLAKGVKSLSNAELLAILLRTGNKNKNAIELANY

IINRDIQGIRHLEDMTIEELCNIDGIGLSKSTQIKAALELGSRVASFKPIKYKIMNPWDI

QRYYMDSLRYLKKEVFKAVLLNTKNEIISDVDVSIGTLSSSLVHPREVFKEAIRRSASKI

IVMHNHPSGSVEPSREDKNITSRLIKCGEIIGIEIIDHIIIGDGLYFSFKENMII*

>CD630_11450 Clostridioides_difficile_630_NC_009089 cell shape-determining protein MreB

MAKEKKKEKKGFFSFNKMTKDMGIDLGTANTLVYIKGQGIVVREPSVVAIRDDSKEVLAV

GEEAKKMIGRTPGNIVAIRPMKDGVIADFDITQSMISYFIQKAADKKGVVSPRIAICVPF

GVTEVEKRAIEEAARQAGAKDAFLIEEPMAAAIGAGLKVEEPEGNMVVDIGGGTSEIAVI

SLGGIVTAKSIRIGGDEFDESIVAYVKKEYNLMIGERTAENVKINIGSTFKDDEEINMQI

RGRDLISGLPKTIEICSTEVREALKEPVSSIVDAIKSTLERTPPELASDIMENGIMLTGG

GALLRGLDKLITQETGMSVQIAETPLDCVALGTGKSVEDQEIFEKVLMMNTKN*

>CD630_11490 Clostridioides_difficile_630_NC_009089 septum site-determining protein MinC

MSLREICSQELVEFKGNKRGIIVNIKREAPFEEIQEKIINKLEAYVGFFNGAKISKINSD

CLTDMEILELKEGITSRFDVEFVEDQKIEENSNFPTKYVNTLRSGENIEFEGDVVILNDM

KPGSKVLSKSNTVVMGDINAGAKVVAGGNVFVMGKIEGFVHAGAEGNEFAYVVAGNLNPK

ILQIADNIAEAPDDEENYESESEISPEIAFVSNGRIVIESYLSKLDK*

>CD630_11500 Clostridioides_difficile_630_NC_009089 septum site-determining protein MinD

MSEVIVITSGKGGVGKTTTAANLGTALSLENKKTVVVDADIGLRNLDVVMGLENRIVYDI

VDVVEGTCRLKQALIKDKRFDNLYLLPAAQTRDKNAVSVEQMIDLCEKLKESFEYIIIDC

PAGIEQGFKNAVAGADRAIVVTNPEISAVRDADRIIGLLEANEIKEIRLVINRIRNDMVK

RGDMMDKQDIIEILAIDLLGLVPDDESIIISTNKGEPAILDSKSLAGQAYKNIAKRILNE

EVPLLDLEVEDGFFGRLKKMFSMAK*

>CD630_11510 Clostridioides_difficile_630_NC_009089 cell division topological specificity factor MinE

VLDLFRVFSNEAKTSKSVAKERLKLVLVHDRVDCSPQLLEMIKTDILKVIANYAEIEDDG

LEIKMSKCRGEHDDKPVSALVANIPLKNIKDRCM*

>CD630_11520 Clostridioides_difficile_630_NC_009089 rod shape-determining protein MrdB

LKKININYKSTIKLIKQLDWKLIVTVLAIFIFGLVILSSATHANSTGSYNQLIKQGLAFV

LGIGMIIVILFFDYNLLGRYYKALYIISLILLAIVLLPGIGTVKGGARSWINLGPLDLQT

SEIVKLTFVLSYAKILESKKDKLNTLKEVMPVVVYSLPFIGLLIAQPDLGTGIVFCCMIF

AMLFTAGLSSKLIKRGIIILLVSMPLMYLMMADHQKVRIEAFLNPEDVTLKGNYQVMQSL

IAIGSGGVTGKGLYNGSQNQEDFLPVQDSDFIFAVVGEELGVIGMAVLIILFMIFLLRLL

AIARDAKDFYGTLIVVGVMGMFGYQIIQNIGMTVALIPVTGVTLPFVSYGGSSLLTSLAN

LGLVLNVCMRRKKINF*

>CD630_11530 Clostridioides_difficile_630_NC_009089 methylglyoxal synthase

MNIALVAHDQMKNTMVGFCIGYESILKKYGLYATGTTGKRIMDETELNINRLASGPLGGD

QQIGSLIVTQEIDLVIFLRDPLTSQAHETDIQALIRLCDVYHVPIATNLASAEIFIKALD

RGELSWREVRKSKSQRI*

>CD630_11540 Clostridioides_difficile_630_NC_009089 PadR family transcriptional regulator

MQLNKEVLKGYIDILIVSILEKKDCYGYEIAKQVRERSEFELKEGTMYLALKRMESKNLI

KSYYSNEQSSGGRRKYYNLTNEGKDFLEIKKQEWRFIKKVMNQFLGEVD*

>CD630_11570 Clostridioides_difficile_630_NC_009089 anaerobic nitric oxide reductase flavorubredoxin

MSKVFEVKKDIYFTGVVDEGLKVFDIIMETEFGTTYNSYLIKDEKTVLFDTVKANFKDEF

LSNLSEVTDIAKIDYVVIHHTEPDHAGSLKYLLDINPNIEVYCTKAAKLYLDGQINRPFN

CHVIKDGEILNIGKRNLRFITAPFLHWVDTMFTYIEEDKTLLTCDAFGCHFASVDAEVVN

SEDYLKSAKHYYDCIVKPFAKHVLSAVDKVVGLNIEFDTILTSHGPMLTKDPMAAVKRYV

EWSTEAVNTTNQNQVSIFYLSAYSNTLEMAKKIKEGLDKEGAKAELYDLEDMTLTEMHDT

LVVSKVILLGSPTINKTMVKPMWDLFSVIDPMANQGKIAGVFGSFGWSGEGITMAETLLK

SMSFKMPVESLKKKFFPSEETLKECMAFGAEFAKLVK*

>CD630_11590 Clostridioides_difficile_630_NC_009089 hypothetical protein

MSKIIRVKFKKEGDMIYISHLDLQRLLQRAFRRAEINLSHSQGFNPHPKMSYGNALALGT

ESQGEYVDIEIEEDDLSVEEFLNKVSIQLPDGIDFIKAKEIDRQTPSLSSVIDYGEYLFN

IDLKRPMTKEFVKRKVIDFMNNKEIIITKKNKKGKMVEVDIRPMIRTFDVLNLEDEHITL

TATIATGSKTNLNTNILIPKILEMFELDIDPLDVDILRRDLYVLEDGELVTPM*

>CD630_11600 Clostridioides_difficile_630_NC_009089 ribonuclease G

VKKIVIESLIGSQKTAVLEDERLTELFVEDNLNKKTVSNIYRGIVKKVIPGIEACFVDIG

FKKLAYLQLKKGSTIKSGQDILVQINKEEIGTKGAKLNTEISISGRYIVYIPSNDRTTIS

NKITDEKERFRLKKITKAVNKENLGLIIRTEAQGCTHDEIKKDIEELKLKYENILKEYKL

GIGPKLLYKSLDFATKYVKDNVNDDIESIITNSYDKYSELKSILRGIDKTYVDKLCLEEN

RDVFDLYRIESKIEKLLNKKVWLKSGGYLIIEKTEALTVIDVNTGKFIGTGKLDETVYKT

NLEAAKEIVRQLRIRDIAGIIIIDFIDMHKKKHQKEILHILEEEFNKDKRKAEVLGMTKL

GLVEVARRREKESIDKYYLMSCPCCDGEQTIKSVHYILDSIEKEIMRISEHTVYKNIMVE

FNDFIFEQIKEYYMDIIDKIGEKYNIKISLNANSTLKHNKTNVIFDKIVDNKM*

>CD630_11610 Clostridioides_difficile_630_NC_009089 50S ribosomal protein L21

MYAIVKTGGKQYKVSEGDVLFVEKLEANAGDVVTLNEVLACSKDGELKLGSPVVEGASVQ

AKVVEQGKAKKVIVFKYKAKKDYRRKQGHRQSYTKIVVEKINA*

>PCZ31_RS05525 Peptoclostridium_difficile_strain_Z31_NZ_CP013196 ribosomal protein

MIKVKYYYNDDFLLQGFCLKGHADFAEIGYDIVCAAVTSNAIAVINSLDKLQKIEFEKVV

GEEGHIECIVKDTHVKDAQLLLNHFQLAVKEIKREYQKYKNFKKVGGDSIC*

>CD630_11630 Clostridioides_difficile_630_NC_009089 50S ribosomal protein L27

MLNMNLQLLASKKGVGSSKNGRDSISKRLGVKRFDGQLVTAGSIIVRQRGTKIHPGTNVG

KGSDDTLFALVDGTVKFERKDKKRKKVSIYPVAIAE*

>CD630_11640 Clostridioides_difficile_630_NC_009089 GTPase Obg

LFIDKARIFVKAGNGGNGSVAFRREKYVPAGGPDGGDGGRGASIIFEVDLGLRTLMDFKY

QKKYQAQNGGDGSKGKRAGKNGENLVLKVPAGTVIRDEATGLVLADLKKEGDTAIVAKGG

IGGKGNQHFANAVRQAPAFAKSGTDGEERWITLELKMIADVGLLGFPNVGKSTFLSVVTK

AKPKIANYHFTTLTPNLGVVQTKFGDSFVLADIPGIIEGASEGIGLGHEFLRHVERTKVL

IHIVDISGLEGRDPIEDFDKINDELKLYNEKLSKRPQVVVANKFDILEDESKFEKFKSEL

EGRGYTVFKMSAATRQGIDEVIAYVSKMLKEVEDVELVSEEEMYRPELDIGTEEELSIDI

EDGVYVVTGKALRRIMYSVNFDDMESLQYFQKAMESQGVFDRLREMGIEDGDVVKIYELE

FEFYN*

>CD630_11650 Clostridioides_difficile_630_NC_009089 RNA-binding protein

MLKGKQRAYLRSLANTLKPTTQIGKEGVTESFLEQLDGMLRTRELVKVTILENAGLDTKE

TANAVCEALRAEFVQAIGFKFTIYKRNIEEPKILFPGHEQAKAKTKNNNVTKKGKPTKRA

VR*

>CD630_11670 Clostridioides_difficile_630_NC_009089 integrase/recombinase

LKMATRPIEIDEYKKIMELLHTGFTYSENGVEKRFRKNPKVALALMLEANLGLRISDILR

LKIGNFKGNMLEINEKKTGKLQYRPVNKNIIESINEHARKYNLKSNDYLVNIKTKAIQKQ

LRIICKHLNLYNISTHSFRKLYATTQFEKSNNNLELVKELLNHSSVATTQRYIRVTQQAI

NEASENFFIG*

>QAD_RS0205805 Clostridioides_difficile_QCD_97b34_NZ_CM000657 membrane protein

MKKNKIIAFFAPAIIVGVLVLGIILYPNEAIKSAREGFSIWSNVLVPSLLPFIIGANLIV

DLKIVDIIGFIINPITRFVFNVSGKSALAFAISTVSGYPVGVSLASEFRSNGQISKHEAQ

RLVSFCSTSGPLFIIGSVGTGMFQNSNLGYLMILCHYLGTILVGLFFRNYGNENLPKTKL

DLKTNIKNVINIRNSSGNGFFVLFGNAVFNGVNTLLTVGGFVIVFSVVFKILSLFNVISL

IASVIYLPLSFLGVSKELCQAFVSGLFEITIGCNKVSSITSSPEILRASLASFLIGFSGL

SILAQCCTFLSKTDIDLKLYILSKFAHGVLAAIFIFILYPIANSAVLVSSFADTYNVIYN

NLIWFYYLSYYDTILQIVVIIYLISAVFIAKKHMKTLSTNGKLMRYKNTFFIKKFSTKKI

KLIKLN*

>CD630_11690 Clostridioides_difficile_630_NC_009089 GntR family transcriptional regulator

MFNSIASKKVYEQVIEQIQYKILNGELKKGDKLLSERELSEQMNVSRTSIREAIRVLETM

GVIESRQGEGNFICTNIEKTLIEPLSMIFKLNNGTLEDILELRIILEIEIAKLASKRITS

SEVIELKHIIDEMRVETNKKDNNRVLVLLDQKFHSKLATLSKNYLIQSLFMTASKLFDGF

IEDAREKIIAEPFNENILLKQHEAIYNAVVENDVELACEKAKEHMDFISKNYRKNEN*

>CD630_11710 Clostridioides_difficile_630_NC_009089 electron transfer flavoprotein subunit alpha

VYFMEILTCIKQVPGTTSVEVDETTGVLKRDGVDSKMNPYDLYALETALRIKEDKKANLK

VLSMGPPQAKKVIEESFMMGADEGALISDRRFGGADVLATSYTISQGIKKMGKVDLIICG

KQTTDGDTAQVGPEVAEFLDIPHVTNVTKLIEVKDESIVVEIDMPNDLQVCEIEYPCLIT

VEKDIFQPRLPSFKLKLNTKDREIPVYSLDDFEDKNENNYGLNGSPTQVVRIFPPKPNTD

KNIVRGNADELSFALVNKLEELKLV*

>AEC_RS02000000220890 Clostridioides_difficile_QCD_37x79_NZ_CM000658 FAD-binding oxidoreductase

MYKLIDKKDIDFLIDTCGEENVLVGSDINEDFSHDELGGIEKYPEVLVNVLETEQVSKIM

KYAYKNNIPVTPRGQGTGLVGAAVAINGGIMINLCKMNKILEVDYENLTLTVEPGVLLMT

IGQYVQDRDLFYPPDPGEKSATIAGNINTNAGGMRAVKYGVTRDYVRGLEVVLPNGEIIN

VGGKVVKNSSGYSIKDLLVGSEGTLGIVTKAILKLLPLPKKSISLLIPFPDLSMAIETVP

KIIKSKSIPTAIEFMERDVILAAEEFLGKKFPDNTSDAYLLLTFDGNSTEDIEKEYEKVA

NLCLENGALDVFISDTQERNDSIWSARGAFLEAIKASTTQMDECDVVVPRDKIAEFIRYT

HELQDKLKIRIKSFGHAGDGNLHIYILKDGMDDNTWKIRLKETFDYMYKKSRELSGQVSG

EHGIGYAKKEYLHESNSDAYMMLIKNIKLAFDPKNILIQGKYIRNINLIIFTKQKILN*

>CD630_11750 Clostridioides_difficile_630_NC_009089 acetate kinase

MKILVLNCGSSSLKYQLIDMNNEEVLCIGLVERIGIEGSILKHEKAGRDDKYVVEQPMKD

HKDAIALVLEAVAHPEFGAVKEMKEIDAVGHRVVHAGEKFATSVVITPEVEEALKECIDL

APLHNPANIMGIDACKAILPDVPMVGVFDTAFHQTMPKSSYLYGLPHELYTKYGVRRYGF

HGTSHNYVSQRAAEILGKDIKDLKIVTCHLGNGASIAAVDGGKCVDTSMGFTPLEGLIMG

TRCGDIDPAILPFLMRKEGLDADGLDKLMNKESGVYGMTGISSDFRDIEDAAKNGDERAQ

ATLEAYVKKVQKYIGAYAAEMNGLDVVVFTAGVGENGKAIRADIASNMEFLGMKLDKEAN

DVRGKETVISTADSKVKMLLIPTNEELMIARDTLRLVK*

>CD630_11760 Clostridioides_difficile_630_NC_009089 hypothetical protein

MKVSIEKINRKETDKIDLNFCEKIDTISYCDEIYKLVSPVNLKGKVSKTNKGLYLDIDVN

FTIVDNCSRCLKEVEIPLEYSIQGFLVKEEDYDEDEFEEFDPFIFDGEEIDLIDIIEQTL

DFNVPHKVLCSENCKGLCQVCGANLNEEECSCSEITNDEEYIDPRFAKLKDLFN*

>CD630_11761 Clostridioides_difficile_630_NC_009089 50S ribosomal protein L32

MAVPKRKTSKSNTKMRRAANSKMEATGFVSCPQCHEPKLPHRVCPDCGYYKGKEVVSK*

>CD630_11770 Clostridioides_difficile_630_NC_009089 fatty acid biosynthesis transcriptional regulator

MKKKSKAQRQKELIDMLKTDPFYTDEELSSLFDVSIQTIRLDRMSLNIPELRERVKSIAE

TQSSKVKTLGVKEITGEIIDLSVGRLGISMLEVTQDMIYSKTNTLKDTYIFSLADSLAMA

IIDAPKVIMRVANVKSFKLIEQQDRLIAKAEVYRNIDKKHYVKVVINNKAQEQIFRGKFI

FEELD*

>CD630_11790 Clostridioides_difficile_630_NC_009089 3-oxoacyl-ACP synthase III

MNTKAGILGVGSYLPEQSYDNFHFEKIMDTSDEWISTRTGIKERRFAKESEATSDLASKA

ALKAIECAKLNVEDIELIILATITPDMSLPSTACIVQDAIGAVNATAFDISAACSGFVYG

VTIAKQFVETGCYKNVLVIGAETCSKFLNYDDRTTAVLFGDGAGAAVIGPVNEGGILSTH

MGSDGKGKDCLKVPAGGSRLKASKETVEANLHTIEMAGSDVFKFAVRKMAETSLRALEKA

NLNTTDIDYLVPHQANIRIIQASSKRLELDMKKVYVNIDKYGNMSAASIPVALDEAYREG

KIKKGDNVVLVGFGGGLTWGASVVKWTL*

>CD630_11800 Clostridioides_difficile_630_NC_009089 enoyl-(acyl-carrier-protein) reductase II

MNKICKILNIKYPVIQGGMAWVATASLASAVSNAGGLGIIAAGNAPKEAIKKEIVECKKL

TDKPFGVNVMLMSPFVDDIIDLIIEEKVQVITTGAGNPAKYMDRLKEAGTKVIPVVPTIA

LAQRMEKLGATAVIAEGTEGGGHIGELTTMVLVPQVADAVNIPVIAAGGIVDGRGIAASF

ALGASAVQVGTRFICSEECSVHSNYKNLVLKAKDRDAIVTGRSTGHPVRTLKNKLSKEFL

KMEQNGATPEELDKKGTGALRFATVDGDIEKGSFMAGQSAAMVKEITPCKEIIEAMVNQA

REIMPAIEL*

>CD630_11820 Clostridioides_difficile_630_NC_009089 3-oxoacyl-ACP reductase

MINLTGQVAVVTGGSRGIGKEIAKKLASFGADVVINYTSKEDEALKTKNEIESMGVKCTS

IKCDVSKFDEVNQMIDSVVSEFGKIDILVNNAGITKDGLLMRMKEEDFDRVIDINLKGVF

NCTKAVTKPMMKKKYGRIINMTSVVGIMGNAGQTNYCASKAGVIGFTKASARELASRNIN

INAVAPGFIETDMTKVLSDDVKESTLANIPKKSYGKPEDVANAVAFLVSDMSSYITGQVI

NVDGGMVMQ*

>CD630_11840 Clostridioides_difficile_630_NC_009089 3-oxoacyl-ACP synthase

MNKRVVITGLGCVTPLGTGKEEFWSNIKSGVSGIDKITNFDASTYQTQIAGEVKNFHPEE

YISKKELKRLDKFAQFAIVSAKLAVEDANLDLDKVDRERFGVIIGSGIGGVEAIETQHKI

LLEKGNKRVSSLFVPMMIGNMAAGQVSIFLGAKGPNTNVCTACASGTHSIGDAFKVIQRG

DADIMVAGGSEAAVTGLAFAGFCNMKAMSTRNDDPKTASRPFDKDRDGFVMGEGAGIVIL

EDLEHALARGAKIYAEVVGYGLTADAYHMTTPAENGEGAARSMNMALKDGNVPLEEVDYI

NAHGTSTYYNDLYETMAIKTVFGEKAYDLCVSSTKSMTGHLLGASGAIEAVVCAMSIEDS

FVPPTINIQEVGEDLDLDYVPNQGKEKNIRYALSNSLGFGGHNATIVLKKYV*

>CD630_11870 Clostridioides_difficile_630_NC_009089 hypothetical protein

MMKYKTTGTCATEIEFEVKENKVTNVNFIGGCDGNLKGLKVLVEGMNIEDVIQKLKGIEC

KTKPTSCPDQLSLALENYMNIK*

>CD630_11900 Clostridioides_difficile_630_NC_009089 acyl-CoA N-acyltransferase

MPTITLKNGVDVLIREGVREDAQSIIDFYNEVGGETHFLSFGKDEYKISLEEQENAIESA

KASDNSVKLIAFIDGEIVGIATIDSNQKAKGKHVGVLGIVVKEKYWGIGLGKRLMLDLIE

WCKSNGITKKITFVTNEENYNAIGLYKKVGFEVESILKKECYYNGVYTDLIGMSLLLGI*

>CD630_11920 Clostridioides_difficile_630_NC_009089 stage III sporulation protein AA

MNKLSDEIINSLSTTIREKVEKVSNNNLNIEEIRLRSQKPLILNANSKDYFYNQKTMTLD

LNQQNSYVVTREDVEQTFQIICKYSIHSFMDDIKKGFITLRGGHRVGLVGKAIVEDGQVK

NIKHISSLNIRVSREIIGCSDKILSHIIKGKNQINNTLIISPPQCGKTTLIRDIVRNLSN

GNEDYGFKGLKVALVDERNEIAGAYLGVPQMDVGIRTDIIETCPKDLGITMLLRSMSPNV

IVTDEIGSEKEIKALYTALNGGIGLITTVHGDSIEDIQNRKELNRLLDKELFKKVIILSA

KRGAGTIEKIYDLEEKRWYFAN*

>CD630_11930 Clostridioides_difficile_630_NC_009089 stage III sporulation protein AB

LQIKIIIIAFLIGSSYLIGEQIYKTYTRRHKQLNDLIRVLEILRMDLSFGLYTLEEIFNR

IGGNKEFCFWKFFYQISEGLHNEQSKTLEIIISENIDVLSKETYLGNKEIEELKNLILTL

GKSDIESQQRMIDLSIENLKKQTYETKEDINKKGVLYKKLVTFIGIGICIILI*

>CD630_11940 Clostridioides_difficile_630_NC_009089 stage III sporulation protein AC

MEISLILKVAGVGILISVLNMILEKTDRKDWAGLTTLAGVIIVLGMVITEISDLFNTVRT

MFQLY*

>CD630_11950 Clostridioides_difficile_630_NC_009089 stage III sporulation protein AD

LEIMQLIGIAIISTTLCLVIKKDRPEIANFIAIITGVIILLSVMFKLNFIVDSIQDLANK

ANIPTMYISLIIKLIGIAYLMEFAIQLCKDCGEGNIASKLEFGGKIIVMSMSFPILLSIV

EMVVNIIP*

>CD630_11960 Clostridioides_difficile_630_NC_009089 stage III sporulation protein AE

LLMKKRFLPMIIGFLFTFFFVNIFAIMIFANEVPSSEDKEYGETKNSIDKYIDGQLDKLD

INEIQDYINKEIVINDVNLKSFVKDLISGEKNILDLFNKDGLKILMFDEFKASLKVVAVI

LVLALLSSILKSLENSFSSGAVSQIATYIIFITMVSLTLIGFKDVLQICYDAIDHTVGLM

QVIMPILITFLLLIGFPITSTTLNPIFIGGVTFINVFFKNFLFVSITVAFGILIINNLSK

NIRLKRFFSFVKQINYVSIGAMFTVYLGLVSIQGLYVTSFDKFSVKTAKFAIGNFIPVVG

GFVSDSVDILLSSSQLIKNIFGGIGLILLVGICLLPVIKILSVIVVYKLAAIIVEPVGED

GISNFLNEVANLMIIMLASVIAITVMFFVTVAILTSISVVSQG*

>CD630_11970 Clostridioides_difficile_630_NC_009089 stage III sporulation protein AF

MLEGIKAWIVSVLIGAFIVNIVDMILPSSKIKPYVNLVLNFMFVFIVITPVVGFFSKDMS

LEDRILKSMGNYNKQYVDSTNALAKETGNNSLSKGYEDGLKEVLKLKLDEYGYDLEDIEL

NGANINNIKIKEKNNSAKSNNSSNEENKNNSTKREEENINSNDKENSKQVFKKGTEYGLN

LNEEKLKNDLIKVLDVSIEDIQIDK*

>CD630_11980 Clostridioides_difficile_630_NC_009089 stage III sporulation protein AG

MLKNLNDKDKRKVYSLITIAGICVVSLVLLSCFPSSKSDKQVGKTETNKNAEKQVTKEQE

KDDLESKLTAILSKIDGAGDVDVMVTFESSEEIQPAFNSNNTTETTEEKDAQGGERTVTT

SSENKTMITSNSSDPVVIKTTEPKIKGVIVVASGASDPSVKETLYSAVQTSLQVAGHQVE

IYSK*

>CD630_12000 Clostridioides_difficile_630_NC_009089 hypothetical protein

MEDNKFGQVKISNDVIATIAGLAALEVEGIETTATLTDKLLKNNGVKIQIEEEDVNLDVM

VTIKYGMSIPDTAFKVQENVKNTVETMTGLKVSQVNIHIQGISFKKDKVDKEEAKATKKN

*

>CD630_12010 Clostridioides_difficile_630_NC_009089 N utilization substance protein B

LMKKDRAQKSTTREYIMKLIYQININKEDFETLEDKVDNFLKDNSEHIINRYKELALQYS

KNTNLKLEDTEIEDVIDKKYINTVCKALKENHDKIDELINKHAKNWTVDRMPKVDVSILR

LSVCEILYLDTPNKVSINEAVELAKIYCDDKSPKFINGILGSVVDEIGK*

>CD630_12020 Clostridioides_difficile_630_NC_009089 O-sialoglycoprotein endopeptidase

MKLGNNIIIGIDTSCYTTSIAAISLDKKVIFNEKIMLEVRDNSKGLRQSEAVFQHINNLG

ILSDRIKSFKDKFNVEGVCSSKKPRPVENSYMPVFNVGHNFGKLLSSIYGCRFYETTHQE

NHIEASLLNSKLKNNNKFISVHMSGGTTEILLTSKQDSHHNVCDTNLGKIAKISIKKDDK

SKLYNNFGYNIDIIGGSKDISFGQLIDRVGIKLGYKFPSGKYLDENALNCNLKIESGLKT

SVRDGYMNLSGLENQVNKIINDNGDNTNQKEYISKLVLDSVVRNMFKSLVYLCETYNVNE

VIFAGGVSASKYILRELSMKLRKKHIEAYFTEPQYSTDNAVGCAIIGLNNFLGERV*

>CD630_12040 Clostridioides_difficile_630_NC_009089 exodeoxyribonuclease VII small subunit

MNLTYEEAYKRLESILSELESKNASLDESLSLYEEGISLYKHCNKLLDDAKLKISKFNQL

GIEEDFKIEEE*

>CD630_12050 Clostridioides_difficile_630_NC_009089 geranyltranstransferase

LEFKQCLKEKASFVEKVLKEYMPKEEGYQKTVIEAMNYSLSAGGKRLRPILTLEACKIVG

GNEDEAIPFAIAIEMIHTYSLIHDDLPALDNDDLRRGRPTNHKVYGEAMGILAGDALLNY

AFEVMLAGSINKENPEKYLKAINEIAKGAGIYGMIGGQVVDVESENKQIEKEKLDYIHMN

KTAAMMVGCMRAGATIGGANSEQMEEITKYAKNIGLSFQIVDDILDIVGDEAKLGKKVGS

DIENHKSTYPSLLGLDKSKEIAHNLIDEAKKSIEKLSDDVDFLKGLAEYIIDREY*

>CD630_12060 Clostridioides_difficile_630_NC_009089 membrane protein

MDFFSEIFNNGALGISLIACFLAQFIKIFTGKEKRIELSRILISGGMPSSHSSFVTSLAT

VVGIEKGFNSTDFAIITVLALIIMYDAAGVRRAVGKQATILNQMVADIQHGKHIEQKKLK

ELIGHTPLEVWFGALLGIVTALILM*

>CD630_12070 Clostridioides_difficile_630_NC_009089 1-deoxy-D-xylulose-5-phosphate synthase

MYKYLDKVNSPKNIKNMSIEEMDLLAKDIRKFLVKSVSKTGGHLASNLGVVELTLALHKV

FDSPKDKIVWDVGHQSYVHKIVTGRKDCFVSLRQFNGLSGFPKENESPHDIFDTGHSSTS

ISIATGIACARDIKKENYSVISVIGDGSITGGMALEALNQLGYIDTNMIVILNDNEMSID

KNVGGMSKYLSSIIRNSTVEKMTDEVDKILNVTQTGEILSKTAHRFKDKLMYSFSPQDCS

FFDSLGIRYYGPIDGHNTKELIDILRKAKHKKGPVLLHVITKKGKGYRFAEEQPDKYHGV

SKFDIKTGVTSAKVKSMSISVGEKLVDMANSNENIVAITAAMPSGTGLNLFESAYPKRYY

DVGIAEQHATGFAAGLAKNGMKPYFAVYSSFLQRAYDQVIHDVCITKKPVTFLIDRAGLV

GNDGETHHGMFDLSYLNSIPNIVVMAPKDTREMELMMDLSLKLDCPLAIRYPRGSSYYLD

KGEYGEIVLGKYEVLDDGQDTVILCIGSMVKHALEAKEILSREGINPTIVNARFLKPIDE

GMLKALLKNHKNVVTIEDNIVTGGFGSRINKFIIDNEYNVNILNIAIPEEFVKHGNIDEL

YDFVGLSPKSIADKIRKLVIE*

>CD630_12080 Clostridioides_difficile_630_NC_009089 RNA-binding S4-domain-containing protein

MKKRIDLLLVEQGYFESRERAKKAIMAGLVFVDNQRCDKAGTEVKEDCSIEVKGNPIPYV

SRGGLKLEKAMKNFDLTIDGKVCMDIGASTGGFTDCMLKNGAIKVFSIDVGYGQLAWKLR

QDDRVVCMERTNIRNVTIEDTKQFADFASIDVSFISLKLVLPKAKELVRHDGEVVALIKP

QFEAGREKVGKKGVVREKSTHIEVIKMISDFSVENGFEILGLDFSPIKGPEGNIEYLIHL

RNGNEGYEFDGETYNNKIVEVVEASHNLDK*

>CD630_12090 Clostridioides_difficile_630_NC_009089 DNA repair protein RecN

LILELYMKNCALVEELRLNIDKNLNILTGETGSGKSIIIDALGLCLGDKYDRSFLRKGTD

KGLVEAVFFSDNRYLKKILEENDISMEDDNLLVITRLIYSDGKSTARVNGRTVKVSLLKE

IASTLIDIHGQHQNQALFNKDTHLKFLDLFGENELEEFKIAYKKVYHKYSEVKKALNCLT

ENKDEMQIQREIDLLRFQINEIEAANLNKNEYEDLLKQREVYRNSEKIYNNLNSSYSKLH

NGEYNVIDLIGLASKELNDISKYDSVLSEYSDTVERIMYELQDISGEIRNYKDNIDFEPY

ELEQIELRIDEINNLRRKYGDSIDDIFEYYGKIKDRLDEILNRDERVEQLRSKLMNIEED

LKIKASKLTKARIEVATTLEKILLDELKSLNMKNVMFKVNFEKGPFTLDGVDDIEFMISF

NLGEDIKPIYKVASGGEMSRFMLAFKTILADIDDIDTLVFDEIDTGISGIAAQIVGEKLS

DIAKKKQIICITHLPQIAANADTHYCIEKDTSNNRTFTNVSKLNESQRKNEIARLIAGNN

ITEKTIEHASEIIELAKKC*

>CDIF630_01360 Clostridioides_difficile_630_CP010905 putative N-acetyltransferase

VKSLYKHVYIMADKMAQNITLQFVEEKDLENLENVKLLFTEYSNSLNIDLCFQDFNNELK

TLPGKYKKPSGSLILAFVDENLAGCVALKKLEGKICELKRLYVRNQFRGLKIGKILLEEI

IEEAKKFGYTHMRLDTLPSMKSAQGLYEKFGFYDIEPYTYNPIEGARYMELKL*

>CD630_12120 Clostridioides_difficile_630_NC_009089 diamine N-acetyltransferase

LKLNVRIADINNWHDLVSLSVDKSQLDYIESNALSIAESKFITAWVPVGIYDENSLIGFA

MYGRLEDDRIWLDRFMIDSKYQGKGYGKASLDFLVNHLKNEYNCDELYLSIFEDNKMAIK

LYKDFGFEFNGELDYGGEKVMVLKSN*

>CD630DERM_RS06870 Clostridioides_difficile_strain_630Derm_NZ_LN614756_chromosome_1 sporulation transcription factor Spo0A

MGGFLVEKIKIVLADDNKDFCQVLKEYLSNEDDIDILGIAKDGIEALDLVKKTQPDLLIL

DVIMPHLDGLGVIEKLNTMDIPKMPKIIVLSAVGQDKITQSAINLGADYYIVKPFDFVVF

INRIRELVSNRVTQVEPKPRPVQETQMTRSDFVKNVGNIENVGNIETEITNIIHEIGVPA

HIKGYLYLREAIKMVIDNVELLGAVTKELYPSIAKKFNTTPSRVERAIRHAIEVAWSRGK

VDTINQLFGYTVHNTKGKPTNSEFIAMIADKLRLEHSMVK*

>CD630_12150 Clostridioides_difficile_630_NC_009089 thiamine diphosphokinase

MRVEAPIKVDRKTKKLAKRVESGEIAVINHIDIDEVAANSLVEAKIKLVINAAPSISGRY

PNKGPGILTENNILIIDNVGEELFEELKEGETIEVVDGKIYRKGKFLGAGEVLDKYEVSH

QIKAAYENLAVELDRFIDNTIDYAKKEKGFILGEVEIPKVKTNYANKHVLIVVRGQDYKE

DLSTMLSYIEEVKPVLVGVDGGADALIEFGYTPDVIVGDMDSVSDEALKKASEIVVHAYT

DGRAPGLKRVEELGLDAVVFPAPGTSEDIAMLIAYEYKAELIVAVGTHSNMIDFLEKGRK

GMASTFLVRLKIGSKLIDAKGVNLLYRSKLKIKYIWALIATALFPVLVVASLSPGVQQFI

QLMQLKFRVLLQM*

>CD630_12160 Clostridioides_difficile_630_NC_009089 hypothetical protein

MHINMKYYIVTIGAIFIALGIGMLVGFNLNNNQQLSEQQANIINDLDDKFNILKEKNDKL

DSDLASVNRDYEEAVNFINKNVDKILAGSLNGKSIGIISANENDDYTKNIEDIINKSNGS

IAFNIILKENITNPEKLKEMSTKLGTDVKNANDAVNYIIDTLKKEDASDILTYLQELDVI

KFNFIGDTYLKYDSVVIAGGNDAKDSTKQFEKIEKFVVSKLKSENKYLVEVQNTGVKTSY

VELYSKNKVATIDNIDEGIGSISLAILLQQGNIVGNFGRLDTATSLLPSIK*

>CD630_12180 Clostridioides_difficile_630_NC_009089 glycosyl transferase family protein

VKDYILYTILLLTGLFGTYAVIPLFKNLLINGNVLRPNYKKDMIPVSMGIVFLPMIVING

IILAFFTTEFKDLAYIFMFMFGMISMFFAGILDDVIGNRDVSGLKGHFKSLFKGSLTTGG

FKALFGGFVGLIVSVAISKDIIDIIVNTLIIALSTNLMNLLDLRPGRAIKAYLFIMVIIF

LTLTGFVQVLPLLIVPNVLAYFNYDLKARAMMGDTGSNVLGISIGMLISFGYPFNIRLGW

LIFVVIMHIFTEKYSLTKIIEKNKLLNFIDKLGR*

>CD630_12190 Clostridioides_difficile_630_NC_009089 hypothetical protein

MISKRVGIVESIVSQTETLDDIRVNINGEIQRAYNYPKISGTINIGDEVVLNTTAVELSL

GTGGYHFVITNLNNIESTLTEGGHIMKLRYTPLQIKVDSVEEQESIYHDKFANFKGLEGL

SVVVGTLHSMLTPFAASFKRNNPNKKLVYIMTDGASLPIYLSKNVDTLKEKKLIDSTITI

GNAFGGDYECINIYTALITAKEILKADAVFVSMGPGIAGTGTKYGFTGIEQGSILDAVKK

LEGRAIAIPRISFADKRERHQGISHHSMTVFKEIVNVNVDIPITIYDDEKLNFIKEQINL

NGLDKKHNIIYIDNNKTKEDLDYFNLKVKSMGRNYEQDEAFFKAASTAAYYLMEV*

>CD630_12200 Clostridioides_difficile_630_NC_009089 NUDIX family hydrolase

MVLEEKTISSDRVYTGKVITLKVDTVEIPGQGYQKRELVEVGGAVGIVAITDDNKVVLVK

QFRKPIEKPIFEIPAGKLEKNESPKECAERELKEETGYSAKNIKLIHKFFTSAGFSNEIM

FVYLATGLTPGENNLDADEFLDVYEIELEEAYNMVLKNDVEDAKTSIGLLLVKDMFKN*

>CD630_12210 Clostridioides_difficile_630_NC_009089 membrane protein

LRRTYRKNDFRELNRQIIIIGLLFMMSIVVGSYINKILPGSSNNILNNINPAVEYYNLNI

SIKDTVIQNLKSDAIFMGSIALLSLFVVTIPAVLVAFVLKGMSIGYTINSCILALKFKSI

KMILIILFKNLIIIPCAIILALISLSYFKEMVYEFKKKNRKNMQFLIKRYILNIIIIIAL

SLGLQLILNTASIGIIKFLAK*

>CD630_12230 Clostridioides_difficile_630_NC_009089 phosphopentomutase

MSRVIWIVIDSVGIGALPDAEKFGDSKDVSTLGNIFKEYPDIQIPNMRNLGIGNIDGIDF

FESIKEPIGCFGKCKEMSQGKDTTTGHWEMTGIIVDKPFKTFEHGFSKEIIEEFEKKTGR

KVVGNKPASGTVIIDEYGEHQIKTGDVIVYTSADSVFQIAANEEVIPLEELYNMCKIARE

IMMGDNAVARVIARPFIGKKKGEFVRTSNRRDYSLDPFEPTVLDNIKESGLDVLAVGKIE

DIFNGKGITDAIHTKSNMDGVDETLNYMKQDNKGLIYSNLVDFDSKYGHRRDPEGYKKAL

EEFDSRLPEIMANMREDDILIINADHGNDPTYKGTDHTREYIPVMIYGNKIKKGFNLGVK

DTFADIGATVADILNVKLPKHGSSFKGDLF*

>CD630_12240 Clostridioides_difficile_630_NC_009089 purine nucleoside phosphorylase

MFEKIAQSSKFINSKSNIKPKIGLILGSGLGDLANDIEDPVTIKYSEIPNFPVSTVAGHA

GQLVIGKLEGKEVIAMQGRFHYYEGYSQKEATFPVRVMKELGVEILIVTNAAGGVNKEFK

AGDLMLIRDHINFSGSNPLVGKNDDRFGARFPDMSDAYSSKYVDVVKNCAKECNIDVKEG

VYMFFSGPNYETSAEVRMAQILGADAVGMSTVPEVIVASHSNIGVIGISCITNMAAGILN

QPLSHEEVIETTQKVKSEFMNLVKTTVKNL*

>CD630_12260 Clostridioides_difficile_630_NC_009089 aminodeoxychorismate lyase

MNFKENRLKIAVLIIVILIILAGIFVFIQIGPYDKNNKKDVIIDVPSGASVGKISDILYE

NKLIKNELLFKLLVKVSNKAPSIKSGTYLLNQSYSNNDIISLLVSGKIYQDGIKVTIPEG

ATSKEIIAMLVSKNLGDKATFENLIKKPQEFYDKFPYLKEDGITSLEGFLYPETYYFNSK

KQSEEDILSEMLKVFDSKYTDKFKKKQKELNMTLQEVMEMASIIEKEAVLDKDRPIIASV

FYNRLKVGMPLQSDATIQYIFEERKKIVTYDDLKIDSPYNSYKNKGLPPTPISNPGIKSI

EAALYPEKTDYLYFVAKIDGGNNYSTNYQDHLKYVKEYKEARDKQSKDTKATNKENTKK*

>CD630_12280 Clostridioides_difficile_630_NC_009089 U32 family peptidase

MELNKVELLAPAGDLERLKIAITYGADAVYIGGEIFGMRSAAKNFSKEDMAEGVAFAHER

GKKVFVTVNIIPHNEDFLQLEDYLLELEEIGIDAVIIADPGVLSVIKKVIPNMEIHLSTQ

ANTTNYLSANFWYEHGIKRVVVARELSFDEISEIRAKTPLDMDIEAFMHGAMCISYSGRC

LISNYMTGRDANKGSCAQSCRWQYHLVEEKRPGEYFPIYEDERGTFFFNSKDLCMIEYIP

ELIKSGITSLKIEGRMKTAYYVATVVRAYRMAIDEFYRDPENWKFNPMWMEELKKGSHRH

FTSGFYLNKPTTEDQNYQSASYVRNYDFIGIVRETEDEDGLIVVEQRNKMCVGDEIEVMG

PYKETMFTKIEEMYNEEGEAIESAPHPRQIVKLKLSVKVGKDYMLRKVIEEKVEE*

>PCZ31_RS05865 Peptoclostridium_difficile_strain_Z31_NZ_CP013196 penicillin-binding protein

MSKKKTPFLKKVGKRSWCIFTIILIIYSVLIYRLVDIQVLKGDKYKQSVESQSVEKVELN

SGRGIIYDRNNKKLTDTSKSQVLIVEKEKLNNNYKILELIKKATKMNDLDIYKAVQEQLT

RPIIQIQTKNIDIDKSMKKELEKNGIMVEEKTMRYAKDGLLSHTIGYIKEDDKSGQSGIE

KSMDSVLRNSNEKYISAFKAGDAGNEKSLNILKGSVKTVDNKDKDRHLKTTIDYNIQKKL

EQILNKEENPTAAIISEASTGEILAMCSRPNFDQNDISKSLKGKNGEFENRVIKATYPPG

SVFKMVVLFSALENGVIDENYTYNCTGKTKVGNTNEILRCNKRDGHGFQNLRQAFSNSCN

PAFLDIAMKLGKEKILKSAEKLHLFEKVDIGLDEEKIREAPKNISIRNLAIGQENIEFTP

LQINQMTQIIANNGTFKPLYLYKSLVDNNMNTIKTYKSSKKEELISPYVCTQVKEYMKSV

SRTGTAKDLKDIEGGCGVKTGTAQSSLNKKAIDHGWITGFYPEERPKYVITVLVEGTQKG

NKSATPIFKEICESIK*

>CD630_12420 Clostridioides_difficile_630_NC_009089 hypothetical protein

LFLLLKIRKSGFISIECIISIAILYVAVYLVSTSLYNCYSFISRNISDREMLSTAKKYIE

DEKYRIQNSKYELIEDKIEKNYINGYEINSRIEQILDYYQCYEINIEIKNEFKKLRFNSY

VTRK*

>CD630_12430 Clostridioides_difficile_630_NC_009089 hypothetical protein

MLLENKGSILIITLIIFSIISTVCIMCIGLIYTHNNMFELGYKDIQLKEISLSGIEISVS

NVLSCVEEAIDVSNNEDEFREYFLGNNMIECIKKIKDTSYSSLEELSLKVENNTIYDRGD

FFEIDIESNVREKEFLKAIKVRVKIKNPWTGIDIEDYMDKSNNNKIDDKTFNNYKASENN

EVLNQFKNESENIRNQEVGGFTEEDEEYEEDEEYEEELKQNIIENIKKNFDEKNLVQVYK

YRGV*

>CD630_12440 Clostridioides_difficile_630_NC_009089 hypothetical protein

VTVIELIVSISIILLIVTLSFPKDNLENHKINLFARQLCSDIRYVRRINMFSNYNTYIYY

INQGKHEGYVLRKDGKNIKSIMLPKNAKIARSIEIIKFKSDGSPQKASTIEIYNNRLRKT

ITITPVSGRVLLKEGKYET*

>CDM120_RS06520 Clostridioides_difficile_M120_NC_017174 hypothetical protein

MKHKYGYLLLESVVSLSSMLIMILVLYSIFLSTISLKLKVEDKIELQQQSLEIIKSMEGI

ISNSMGIINVSNYEDTFKKATSIKCRYVDENVNNNEESISNKEIILNERRNKLFVNSLNG

ESSQAGGYEIGDYVDEMYVSINNNGQYVNIKLKLSKRSQKYETEFKIKVWNFSENI*

>CD630_12460 Clostridioides_difficile_630_NC_009089 elongation factor P

MVSAGDFRKGVTFEKDGQPCLVVDFQHVKPGKGAAFVRTKYKNLKTGAIREESFNPSEKF

PKAVIDTRQMQYLYNDGELYYFMDQENFEQIPLNYEQVEDAIKFLKENEVATIRFYQGQP

FQVEAPNFAELEVTDTEPGIKGDTASNVTKAATVETGAVVQVPLFINTGDKVKIDTRTGE

YLSRV*

>CD630_12470 Clostridioides_difficile_630_NC_009089 hypothetical protein

MKHLYEEVAYLKGLAEGLEISAESKEGKMIHKIVDALEVFADAIVTLDEEQEELQDFVES

IDEDLADLEEDIADMEEDLYEEDDDEDDEDFSYIEMECPNCGELVEIDEDLLYDDEVDVV

CPDCKAVILSSEDDCDDDCTCGGCSSCDDRE*

>CD630_12480 Clostridioides_difficile_630_NC_009089 ribonuclease III

MKISKKLLDNIQRFENVINYKFKNKEYILEALTHSSYSNENKKYNFNERLEFLGDSVLGI

VISDYLFNEEANLPEGELTKLRANIVCEDSLSEVANDINLGIHMLLGRGEEATGGRHRTS

ILADAFEAVIAAIYLDGGFESARQFILHHMENIIYDSRKGNIFRDYKTHLQEVLQGNGEN

NIWYRLIEEKGPDHNKRFVMEVGINDDVLGIGEGKSKKEAEQLAAKIALKKKLWEK*

>CD630_12500 Clostridioides_difficile_630_NC_009089 chromosome partition protein

LYLKRLELKGFKSFPVKTDIIFKEGITAIVGPNGSGKSNISDAVRWVLGEQSIKSLRGDK

LEDVIFAGTDTKKPMNYCEVALTIDNSENQLELDFTEVTIRRRAYRNGESEFFLNNKSCR

LKDIKEVFLDTGIGKDGYSIIEQGKVDEILSNNPLSRRKVFDEACGISKYRYKKQEAERN

LSNTKENLERIDDVYIEIENQLKPLFNQQTKAKKYLEISEKLKTLEVNSFIREIEGIEKE

LSEVNEHRKVIEKELNEKEEQKNVVEKKQEDINKEVEVLQDVIEKSVDYINSIKGVISKK

ESQINLIKERIRNFTNEISRKNLEIKDIKEKLNENKQYIKELESNKLSGSEELSTLQENI

KVLEGSKDKQKIKLESLNNEIELLKESIIDILNKKQEFSNKLSTLNANKENMNIRDENIN

SEITELNKNIEIKSSELDTINKEFNMQNENLKNVNNRHKELSINLQDSISEHNKLEDEIQ

KSKYNLNGYNSKLNVYIDMENHYEGFNRGVKEVLKNKNLKGVHGALGQIINVPEKYEKSI

EAALGAYMQNIITDNEFSAKSAINYLKQNNLGRVTFLPLNIIKSNKISLGNLKANTKFIG

IASDLITFDEKYRNIIENILGRTILINNIDEGIKFAKETGHRFKIVTLDGEILNPGGSLT

GGSLKTNGNILSRKRYINEYTEKISNIKNEISHLELKRESLDKDVKNIKNEIDSHESKIK

DLEKSIIIKSTSIKNVESEIESLKGSITKLENEKNDLNSNLNYTLEKSDDVRKDMEELDD

LYNKNKEKIDALNEEIKRYNDLYDKEKSEFDELNLSLVKKTEVYNSIVRDIKRISGENCE

LEEKNKQLEESLNYEEHEIIKLQDSILTEEKEKENLTKQLGDSNRNLETRKIAKDDLKNS

FDEINKELKTIDRQHIELKESLFKVGGRLERLKTSQDTYINKLFEQYDMTLVQALEIKDE

DLDIDRKFLESLKREIRSLGNINIDSIKEYEEIKERYDFYSEQKQDLEESMEEIEKLIHT

LEENMKSEFEIKFEEISKNFKYVYKRLFGGGCGELTILDKENLLESDILITAQPPGKKMK

NLNLLSGGEKALTAISILFAILITKPTPFCILDEIEAPLDDANIFRFGEFLKDLSKETQF

ISVTHRRGTMEAADYIYGVTMQEKAISKVISLKLKEAQEITDII*

>CD630_12510 Clostridioides_difficile_630_NC_009089 signal recognition particle receptor FtsY

VLKKLFGFGKDKEKEIEKKDAEEEIEVEDSVDNLENLEETIFSGLEEEVIDKVEDVEEKN

EESVNDEGTEEIENFEKIEVEMDSNGKEVENISNNDSQEEIIEELENYDEIESKEIEDKE

DKKVNLFERLKQGLTKAKQGITDRIDEVLKSYTKIDEELLEDLEEILITADVGVNTTMDI

IERLRDKIKQKGITEPIKVREELKSIVEDILTNENSTLDIEPAPCIILMVGVNGVGKTTT

IGKLANRYKKDGKKVLLAAADTFRAAATEQLEIWANRTNVDIIKHQEGADPGAVVFDAIK

AAKARKTDVLICDTAGRLHNKANLMNELGKVFKIVDREFPEAKREVLLVVDATTGQNAVV

QAKTFKEVADITGIVLTKLDGTAKGGVVLAVKSEVDVPVKLIGVGESVEDLQDFNAKSFS

DALFGN*

>CD630_12511 Clostridioides_difficile_630_NC_009089 DNA-binding regulator

MNIEKMVEIGLLFEQYKELLTDKQKEIVALYYEEDYSLGEISENLNVSRQGVYDTLKRSE

KILRDYEEKLHLVSKIQEQEKNIKIIKDKIIDIKEDLLHNRDCANLIPKLENIEDVCREM

IK*

>CD630_12530 Clostridioides_difficile_630_NC_009089 30S ribosomal protein S16

MAVKIRLKRMGANKKPFYRIVVADSRAPRDGKFIEEIGYYNPISEPKQVRINDEKAIKWL

ATGAQPTEVVKKLLVKNGVIEKFEASKQAK*

>CD630_12540 Clostridioides_difficile_630_NC_009089 RNA-binding protein

MKELVVDIAKALVDNPDSVVVEEFEDNDGIVLKLTVAQEDMGKVIGKQGRIAKAIRTVVR

SVANRENIKVSLEIV*

>CD630_12560 Clostridioides_difficile_630_NC_009089 transfer RNA (guanine-N(1)-)-methyltransferase

MRFHIMTLFPEIFNSYMDESIMKRAVEKGIIEVHIYNIRDFSNNKHKKVDDYPFGGGAGM

VMTPQPIYDTYKHIITTHNINNPSVIYLTPKGKVYNQSMAKQMSLKEDIILLCGHYEGID

ERIIDLIVTDEISIGDYVLTGGELPALIMIDSISRLIPGVLNQEESFEEESFKDNLLEYP

HYTRPRDFEGLKVPEVLLSGNHKKIDEWRREESIRITKERRFDLYKKSNEK*

>CD630_12570 Clostridioides_difficile_630_NC_009089 50S ribosomal protein L19

MNEMLRAIEQEQLKNEVPNFGPGDTVKVHVRIIEGKRERIQVFEGVVLKRQGGGARETFT

VRKMSFNVGVERTFPVHSPKIEKIEVTRKGKVRRAKLNYLRGRVGKAAKIKEARNK*

>CDIF1296T_01324 Clostridioides_difficile_ATCC_9689__DSM_1296_strain_DSM1296_CP011968 GTPase

VSNEYEEYLMNDNLHINWYPGHMKKTKELVKNNLKLIDVVIELLDARIPFSSKNPDIDRL

VGDKPRVVVLNKSDMADRDKLNQWIEYYKKINIKAIPVDTIKGVGINKIIEECKNVTREK

MSSLKDKGRKERAIRIMIVGVPNVGKSSLINKLTGRKSTQTGDKPGVTKGKQWVRLKGNL

ELLDTPGILWPKFEDQEVALNLAFSRAIKDEILDTETLALRLIEKLMKIEPEKLKARYKL

DCLGETPIETMDMIGHKRGFITGKKELDYTRIATTVLNEFRDGKIGNITLEVPENVKR*

>CD630_12590 Clostridioides_difficile_630_NC_009089 branched chain amino acid transport system carrier protein

MGKTKDVIVFGFALFAMFFGAGNLIFPPYLGIITGPEWLIAFLGFTFADAGLALLAVMAT

AKFDGNVVEMFKRCGIKLGILIGCADILCIGPFLAIPRTGATTYEMGIMPLFGTSIPVLL

FCILFFAISYVLTIRPSKVVDIVGQFLTPALLIALAFIIIKGIISPLGDIVDKPMIPNVF

AEGIGQGYQTMDAFAAIALASVLIVSLNDKGYSTISDKLKMIGKAGVLACGGLALVYGGL

CFLGATVSTMYGTDAVQSQVIVNITEGLLGNVGKAILAVVVSLACLTTSIGLTSATGQYF

SRLTKGKLSYEKIVLAVSVFSAVVASFGVGTIIKIASPILSIVYPPSIVLIILAFFNEKI

KNDNVYKGAVYMSLLVSILTVISSYGVAVPVVNSLPLNSLGFNWVVPVIIAGIIGNFIPS

KSQSNTLGTN*

>CD630_12620 Clostridioides_difficile_630_NC_009089 ribonuclease HII

MQDKSVREIKEIIETLEVEKYMEYIELLRVDERKSVQGLAIKLAKKLDNIRKEEERLETI

NIFENEGYDKGYLYIGGIDEAGRGPLAGPVVASVVVFKKDTKIEGVNDSKKLSEAKRDEL

FEVIKEEALDYGIGIVNNEEIDEFNILNATYMAMKKAINCLKKAPDYLLVDAATIPGIDI

SQNPIVKGDSKSISIAAASILAKVTRDSIMYQYDRVYPEYGFKSHKGYGTKEHYEAIEKY

GITPIHRKSFLKNIL*

>CD630_12640 Clostridioides_difficile_630_NC_009089 hypothetical protein

MSRKAYEEALVELEKFIDERKEIIKSAEDCIDKYIVDRTLPFDYKDKCVEWQQELLDIAE

AQVLEANELSVLLQEKKELEED*

>CD630_12650 Clostridioides_difficile_630_NC_009089 CarD family transcriptional regulator

MYKIGESVMYPKEGACSVNDIVTKKINHEMQKYYELSVIFNSNLKISIPVLNADRIGIRP

VMDGNDVDNFIQSINKTDGVWIFDRKERLKLYQDKFHSGDVFEIVKLIKMLMIQDSSKQL

CSTDKEFLNKAQKFALSELAAAQCKSYTMVLEEMKKHILNSKNTN*

>CD630_12660 Clostridioides_difficile_630_NC_009089 multidrug family ABC transporter permease

MIRLTIANIKRYLKNHTLLINMVMLPIILIFSLNFFINNSGNQSMYFSPVAIVSDSSGKY

EHKLINSSKLKENSFRLNEQDKAMNLLKNNKVSAVFVLDKNFSNSIDKLQRPVVKCFKIE

NGGGSLWAESQIESFITKSLKLKVDKNIDDKLTKTNIIDNKPDKNKGSFLVVFLICYFMY

INAAHLASDLFTLKKSNVLKRLISTDNKDIKIIFSIFLGLFFIQSIVYTFALLCFSIIED

FNLSLNVLMIVLANSFVSTGFVFWVARVFKNESSISLISTFYSLIGLAISISSLIPSMDK

LSFMTNLSKFTPFYWTIDAIKNNGSILINIIALILIGIIFVTAGSLKLRDFAKN*

>CDM120_RS06635 Clostridioides_difficile_M120_NC_017174 ABC transporter permease

MKLYMSVKTMLKGLKSSFILNLIYFLALPLILSWFLGMVTESMFQNPIKTESTPIVIYDK

DNTRLSNHLTKYLKNDLSYILTVKKDDSKAELKLTIPKGYESSLLNEKSNTLNIEKLGTR

DDIAILLQDILDTYHEKFYLNNSQKISSEDFSKLFNKNSIDTSIIGNNVKQSSYEYFALV

SLGFLVIIFIMNNILSNYISESKGLSKRLYSMPITRVQFLIYDFVGLWIYSFIFLLLYVL

FFRIIGITFKGNFAILLLLCALSSYFMTSISTFVNSFFSKKYGTIIVYALLFLQTIFGGI

FSMISDAFTKLTSLSPTYLIGELFSNYETFKTIDSIGNLIFTCLITSTILIALAIVKEKY

KWREVQ*

>CD630_12680 Clostridioides_difficile_630_NC_009089 multidrug family ABC transporter ATP-binding protein

MDIVRVNNITKRFNDKLVLDNISFSVKKGEIFGLIGPNGAGKSTLINIITNLMLPNSGSI

QINDLDLSKDYIKAKSIIGLVPQELAIIETLTPFDNLEYFGAFYGLKGKLLKERIIEALE

VTGLTEVKKKKVKKLSGGMQRRLNIGIALLNHPKILILDEPTVGVDPQSRNHIFNFIKDI

SKKHETTVIYTSHYMEEVEHLCSKIFIMDEGKEIAFGDNDYLKSLVSTNTKLIMEIKNIN

AQLIFDLKNTKGVISVLENNSLLELDIDKKLQLTDILSIIDKNDSKIMKISYEEPSLEDV

FLNLTGKNLRD*

>CDM68_RS20290 Clostridioides_difficile_M68_NC_017175 hypothetical protein

MNRKKLVAFIRYLSLFILVVSYLKNVENTLYPVITMIILFLIIINNQVRFFSLSNENKFI

LISYFLELILIFILCNYTKTFNSIYFVPLILDTSFLIKEKYKYILFSFVIISSLIISLDK

NIYLALESSSILLIITVLSIYIENENLSKLYSQNLYDKLRISKDELKKVNADLETYASSI

EELAILKERNRISREIHDSVGHSLSTTIIQLNAIEKLLKDKPLIYDLVHELREFVTESFQ

DVRRAISELKPVEYENYQSLFKIKELVKSFIKLTNINVKLTISKNTWNLSRNQSIALYRL

IQESLSNSSRHGKATEIRIFITFNPSSFIITISDNGIGCGNIKKGNGLNSISERIYELNG

KVEFDNSNNGFTIRASFPKFSGGDFLNKIKVLIVDDEKLIRKGLKIILSSYNDLEIVGDA

SNGYEALEFCKTNDVDIVLMDIRMKVCDGVLGTRLIKEYNNSITLLILTTFNDDEYIKDA

MKFGASGYLLKDSSDKVLHEGIRSSFFGNIVLDKSVAEKIMTSEKTIKQEYLYDMYNLTE

KEISIIRLIANGLNNKEISQELFLSEGTIKNNITNILAKLELRDRTQLAIFAFKNKIVIE

*

>CD630_12710 Clostridioides_difficile_630_NC_009089 hypothetical protein

MNNKEKGDFGEKVAVNYLLSKGAKILEKNYRLKIGEIDIIAKMEDEIIFVEVKSRSNIKF

GYPCESVSFKKRKKIIGVASYYIIKNNLNNTPIRFDVIEVYLLEKRINHIMNAF*

>AEC_RS0207650 Clostridioides_difficile_QCD_37x79_NZ_CM000658 magnesium chelatase

MLSIINSSNLVGIDSFLVKVEVDVSNGIPSFNIVGLPGKEIKEARERVKSAILNSGYKFP

STRIVVNLSPADIKKEGAFLDLSISIGLLRELIKKDENYIRESMFIGELSLDGKIRKVRK

VRGILPIIMGAKTQNIKRIFIPIENIKESLLVDEIDIIPIKSLKECVDFLNEEIKVDKVS

IMSFLDDKSRKENGELEKDNSYIDCKYTKINNEESKYDEDFKDVKGNYFVKRSAEIAAAG

NHNMFMIGPPGSGKTMIAKRVRTILPDISIEEMIEVSKVYSILGMINESKGIIDKRPFRA

PHHTTTKQSLIGGGMDARPGEIALAHRGILFLDEIAEFDRKILETLRQPIEDGYVNISRV

KYSAKYPCRVLLVAAMNPCPCGYYMSETECRCRSNEIDRYINKISGPLLDRFDIFVEVNS

IKYSDFNSLKQEESSQKIKRRVENARKIQINRFKKDNIKNNSEIKAYNLFKYCKLEKEAS

KTAEMIFNKYNLSSRSYTKLLKMARTIADLEERDLINSQCIIEAFSFRKAYYSYFK*

>CD630_12730 Clostridioides_difficile_630_NC_009089 DNA processing Smf single strand binding protein

VDFMEKRDTYLWLKSIGGITTKTIEIIENEIVNIEDIFDFSEKEIYNLKNISLNIRKNIV

KYRGHAYLENIKELLYKKAIKYICKYDKEYPENLKNIYNAPKLLFYKGDIGLVNNNFNIA

IVGSRKPTAYGINCAKTISCQLSQYGVNIVSGLAIGIDAYSHIGCMSGKSKTIAVLGSGV

DNPLPKQNLHLSNKILENGGLLLSEYNINSTVAPYHFSNRNRIISGLSDGVVVVEAAIKS

GALITVDFALEHGKNVFAIPGNINSQMSRGCHKIIKEGAKLIENIDDILNEYNIFNIIDK

KINQKYDNISLNAKSKQIIEAIKREGNLHIDSICDYTGIEIKYVNSIINELVLNELVVEM

NNKTYSLNV*

>QAE_RS0206240 Clostridioides_difficile_QCD_23m63_NZ_CM000660 GTP-sensing pleiotropic transcriptional regulator CodY

MASEVLQKTRKINKTLQTSGGSSVSFDLLAGALGDVLSSNVYVVSAKGKVLGLHLNDAQD

SSVIEDEYTKQKKFSDEYTQNVLKIDETLENLNGEKILEIFPEEHGRLQKYTTVVPILGS

GQRLGTLVLSRYSNSFNDDDLVIAEYSATVVGLEILRAIGEELEEEMRKKAVVQMAIGTL

SYSELEAVEHIFAELDGKEGLLVASKIADRVGITRSVIVNALRKFESAGVIESRSLGMKG

THIRILNDKLTDEIKKIKKTINNLYFIGLD*

>CD630_12770 Clostridioides_difficile_630_NC_009089 acetyltransferase

MIIKLNSTYHSRVMKYLKKEPEYNLFIIGDIERYGYGNNFLNIWADVGEHGEIKAILLKY

FEFMMFYSDGEYDVEGFYNLLRNTNYEEISGKICAVDALAKRLGLNNLKVVDFCKLQTKK

FLIDNNCNAKVKRIRLGNLKKTVKLYDLIDEFHSTTLENLKNGLRTGRGYCIEINKQVVS

MAKSTSENRTHAMIIGVGTHPKYRAKGLATKCLIKLCSELLRENKIPCLFYDNEEAGKIY

KKLGFENIGKWGIYSK*

>CD630_12780 Clostridioides_difficile_630_NC_009089 Rrf2 family transcriptional regulator

MKLSTKGRYGLKAMFELALNQDNGPVSLKFIAKKQKISDQYLEQIFSSLKKSGLVKSVRG

AQGGYLLSKNAEDITVGDILVVLEGPVALSDCVLDEDVCENSNMCVTKIVWEKMKKGIED

VIDSITLKDMINDYNKNKLENDITNIKK*

>CD630_12790 Clostridioides_difficile_630_NC_009089 cysteine desulfurase

MEKRRLYMDYSATTPIKKEVLDAMMPYLTDYFGNASSFHTFGREAKDALDKAREQVAALI

NAEPSEIYFTAGGSESDNWTLEGIAYANKNKGNHIITSKIEHHAILHTCEYLAKHHGFEI

TYLDVDSEGKVDLKQLEDSIKDTTILISIMFANNEIGTIQPIKEISEIAKKHKILFHTDA

VQATGNIPVDVKELGIDLMSMSSHKIYGPKGVGALYIRKGVRLHNFVHGGAQEKSKRAGT

ENIPAIVGYGKAAELAKENMQNHVETLTRLRNKLIDGVLERIPYTRVNGSLENRLPGNAN

FAFQFIEGEGILLLLDMLGIAGSSGSACTSGSLDPSHVLLAIGLPHEIAHGSLRLTVGDF

TTDDDIDYILENLPKVIERLRSMSPLYDDAKKQGLVK*

>CD630_12800 Clostridioides_difficile_630_NC_009089 NifU family iron-sulfur cluster assembly protein

MQYSDKVMEHFMNPRNMGEIDNASGVGEVGNPTCGDIMKIFLDIDGDVIKDVKFKTFGCG

SAIASSSMATEMIKGKTIKDALELTNKAVAEALDGLPPVKMHCSVLAEQAVKAALIDYAQ

KNNIHIPELDGYVIDDAHDHDVEEEE*

>CD630_12810 Clostridioides_difficile_630_NC_009089 tRNA-specific 2-thiouridylase MnmA

LFMNKKVMIGMSGGVDSSVAAYLLKQQGYDVIGVTMKLWQDDDVVEIEGGCCSLSAVEDA

RRVANKIGIPFYVLNFREVFKEKVIDYFIDEYLEGKTPNPCIACNKHIKFDDFYKKARQI

GCDYVATGHYAKIEKDESTGRYLLKKSVTDKKDQTYALYNLTQEQLEHTLLPIGDYEKDR

VREIAKEMGMAVHNKPDSQEICFVKDNDYANYVKKHSKKRIEEGFFVDTKGNILGKHKGI

LYYTIGQRKGLGITFGKPMFVIDINPINNTIVLGDNEDLFKKELIAKDVNFISIDTLEEP

LRVQAKIRYSAKPSPATIHRVGEDTIKIVFDEAQRAITKGQSVVMYDGDIVVGGGIIEKS

L*

>CD630_12820 Clostridioides_difficile_630_NC_009089 alanine--tRNA ligase

MEKMGLNEIRSKFLEFFESKGHYVANSYSLVPNNDKSLLLINSGMAPLKNYFSGVEVPPS

VRMCTSQKCIRTGDIENVGITARHATFFEMMGNFSFGDYFKRESIKWGWEFVTEWLNIPE

DKIWVTVYEEDDDSYDIWAKEMNFPEERMVRLGKDDNFWEIGTGPCGPCSEIYFDRGEEY

GCDNPDCKPGCDCDRYLEFWNHVFTQFDRDEEGNYSLLENKNIDTGMGLERMGCIMQGVD

TIFEVDTIKSILEAVEKLTGVKYGENPKNDISIRIITDHIRAVTFLVSDGVLPSNEGRGY

VLRRLLRRAARHGKLLGVKELFLQKLIDEVIKVNDKAYPVLVEKESYIKKVVGIEEEKFN

ETIDQGTEILNSYIEVLKNEGKTVLSGQEAFKLYDTYGFPIDLTKEILEEEHLSVDEEAF

NEEMEKQKERARNARGNMDGESWKEDPLSKLESTVDSTFNGYSEIYGEGTIEAIVKDDEL

VQSAEEGDKVSIVLDNTTFYPEGGGQVGDCGLITNENLVLEVLNTKKGANNSIKHIGIIK

SGRISNGDKVKTLVDRETRMSAARNHSATHLLHKALREVLGEHVNQAGSLVTPERLRFDI

THFEAISNEELKVIEEKVNNVILSSLDIKCDIMNIKEAKEKGATALFGEKYGDEVRVVSM

GDYSTELCGGTHLTNTSQVGMFKILSEGGVAAGVRRIEAITGKAVYEYLKERDGIISEVC

VNLKSKEDNLIQRISSLLEENKNLSKELHDMKAKMSLQSVDSIFDSKVEVNGVNLITNKF

EGMDMDTLRETADNLRDKLGSGVVVLANVVDDKVNFVVTATKDVLDKGIHSGNIVREVAK

IAGGKGGGRPNMAQAGASDVSKVDQALSYASEVIKTQVK*

>CD630_12830 Clostridioides_difficile_630_NC_009089 hypothetical protein

LEKDLDYTMKFEGIPEDRMSVGDTIDFVYKALVEKGYNPINQIIGYLLSGDSSYITSHKN

ARAIIKKFERDEILEEVITHYLNRK*

>PCZ31_RS06090 Peptoclostridium_difficile_strain_Z31_NZ_CP013196 hypothetical protein

MRKKIMSLFAIVIMIFVSTASYGFAEEKGKVIFIDMNRTNLGSMMNIPILKNEIEKRGYV

ALMNIRGDQGTDDRRSYASMGAGTRASVTTEDYINFQKVDQDTAKAFEVATGQKAKEIND

LGINRSINENLEYGEYGANLGSLGKTLSENGLKTSVIGNADIIENNQLVKNRNLCLVAMD

QYGRIDSGNIDNINKKDSKMPFGISTDYDKLLDETKKSYKNSDAIFVELGDTYRLDMYRL

NLNDNTYDTMKKNIVKNINKYLEQVFSMVGENDTVYIASAFPSDLDYKNKRRLSPIIKFS

GEGKGLLESATTRKAGIVANLDVGVDILGTFGLENKEMVGRSYTLVNKEDNISYLKSLYE

RIVSISSIRSTIINGFVGVISASWVIAMFAIFFRNYMPHKERVFKVLKEFIKLGIIMPLS

FMLAPLMNFKTPISISLGIFATTLILYLLGRKLFKDNDIKHMGFFATLTILVIVIDSVFG

TYLMQNNIMSYDAVIGARYYGVGNEYEGVTIASAVFGLAVLLHYKKVSKLLVVIFSLIIL

ITSAYPSMGANVGGAISECVAYLLFIMLIYDIKIDFKKAVLLAVSAVVVVFAFAALDILS

GSESHLGMFVQRIFLNGPGEILQTFGRKIQMNMQLAQTSVWVNILLVGIGVIAVLIFKPS

RHFRKIMNNFPILFKGFIASMVGCIITLLVNDSGIVAASTASIYILIPLIIISINMIIFE

KITKKFLLFI*

>CD630_12850 Clostridioides_difficile_630_NC_009089 Holliday junction resolvase-like protein

MLDGRIMGLDVGDKTIGVAVSDLMGLTAQGVKTIKRVGKKKDIEELKAIIKEKQVNKIVS

GLPKNMNGTLGPQGEKVIKFCELVKAETGIDVEFWDERLSTVAAERSLLEADVSRQKRKK

VIDMLAAVIILQGYLDFKINS*

>CD630_12860 Clostridioides_difficile_630_NC_009089 hypothetical protein

MEENIINLIDENGVESQFEIILTLEAEGKEYAILMPLDDEEAEEALIFRIDEDEEGEILI

PLESDEEYETVVAVYTAIMEEEGLNYDEDESNGLN*

>CD630_12870 Clostridioides_difficile_630_NC_009089 Fur family transcriptional regulator

MANTMDLLKDKLKETGFKITPQRRAIVEILLKHDHSHLSSEEIYDLVRVDCPEIGLATVY

RTMQLLDEIGLISKLNLDDGCIRYEISLHKEDCHNHHHLICKNCGKIMEAKEDLLDNIEK

EIQSLYKFKILDHDVKFYGLCDECNGVSDSEE*

>CD630_12880 Clostridioides_difficile_630_NC_009089 membrane protein

MRGKTHCAIGILTAIQTSLIFKIPISLVDILVSATFAVLPDLDKSNSMVSNFILKNNVSK

YIYRIFIYAVNIIIFFISININDNFYLSAIITFVAIIIIEAKLTHTFLRKVFLSLIFILL

AICLYIIEVEIYFTIFCLMLSIFPWLKHRSFSHSIFATIIVYFLLKQIELITNINNLSFY

GTIGYASHMFLGDLFTKQGIPIFYPLSEKKISLGFLTVGGPFSNFIEKSFIFVLIGLIIF

SILKL*

>CD630_12890 Clostridioides_difficile_630_NC_009089 ribonuclease J family protein

MQLFKKNTNKIKVMALGGLNEVGKNMTVVEYKDEIIVIDAGLSFPEDEMLGVDIVIPDIT

YLVKNRDKIKGIFITHGHEDHIGALPYILKKINVPVYGARLSIGLIQVKLKEHKMNNVKL

NVIGPRQVIKLDNMEVEFLKNNHSIPDAYSIAIHTDQGIIYHTGDFKIDLTPIDGDVMDM

HRICELSKKGVLLMLADSTNAEKPGFTMSEKTVGVGLDELFAKGNGRRIIVATFASNIHR

LQQIINTAEKFNRKVAISGRSMVNVVGVAKELGYLDISDDMLIDLNDICKYEDSELVIIT

TGSQGEPMSALARMAFSEHKKVEIKSGDLVIISAHPIPGNEKLISRVINFLFEKGAEVVY

SDIADIHVSGHACQEELKLIHALVRPKFFMPAHGEYRMLKRHAEIAEQLGMDKENIFVMQ

TGDVLELDKNSAKVANRIQTGNILVDGLGVGDVGNIVLRDRKHLSEDGLMIVVVTISKDE

GKVLAGPDIISRGFVYVRESEDLMDGAKDIIKNVLNECEEKNIKEWAYLKNNIKENLKEY

LYQKTKRNPMILPIIMEV*

>CD630_12900 Clostridioides_difficile_630_NC_009089 small acid-soluble spore protein SASP

MDDVSRQNAIKALKQTKMEIAGEYGMNYEDAFEIIENASNKGVLEGYFKKLEKKKNLGQG

ISRHLE*

>CD630_12910 Clostridioides_difficile_630_NC_009089 D-alanyl-D-alanine carboxypeptidase

MKKLASTALAILIALTPLSFSFANNKENADANQLNISSKSAILMDVGSGQILYEKDAHKK

LPPASVTKVMTMLLIVEALDSGKIKLDDEVQVSETASSMGGSQIFLEPGETQKVDTLLKG

IAVASANDACVAMAEHLAGSVEGFVDRMNAKAKELNMNDTHFANTNGLPVANHYTSAHDI

ALMSRELLKHEMISKYLTTWMDKVVVGKKQVTVGLANTNKLIKHYQGATGVKTGFTQEAK

YCLSASAKRGNTHLVAATLGAETSPERFNDASSLLNYGFANYESVKLCSKGDNIATLTLD

KADENKVKLVAKEDLNALIKKGSSKEFEKKIEIVKNPKMPIKKGTVLGKIKICKDKKVIG

EVELINTKDINKASYLQMLQRIIDNMI*

>CD630_12920 Clostridioides_difficile_630_NC_009089 hypothetical protein

MENKNSKCVMVIDENLPMGIISNTAAIMGITLGKHAPETVGPDVIDKTGNSHLGIIDIPV

PILKGNKEIIKDLRKKLYTLEFNDLTVVDFSDVAQSCNLYEEFTQKIASVPEDELQYFGI

AIYGNKKKVNKLTGSMPLLR*

>CD630_12940 Clostridioides_difficile_630_NC_009089 membrane-associated peptidase

MLSMNIGEIVASLVGIAMAISIHEFGHAYSAHLLGDDTAKAYGRMTLNPVRHVDPIGLLA

MFILKIGWAKPVPVNPNNFKNYKIGNLIVSLAGVFCNVLTAILCVIINKYVHMYAINTIA

EYVFLYSIGFAAFNLLPIPPLDGWGVISTFVPYKWNEYLYKFESMSYIILLIALFTGMYR

IILTPIYAVLLKIVYLFV*

>CD630_12950 Clostridioides_difficile_630_NC_009089 segregation and condensation protein A

MKYNIQLQVYEGPLDLLYDLITKHKIDIKDISIIDITKQYLNYLKMLDKMDLEITSEFIT

MASKLLEIKSKYLLYKQKDEEEDPRIELMEKLEEYRKFKVASQDIKENITYVNERFYRNK

EEIIIDDNVDLEDISIEAIKNILPYIFKVKTSQIENANDEKLDKIVRKKIISVEEKILYI

RDIIKEKIEVTFTNIIKSYENDEIIATFLSILELIKEKEIVVVQDIFFDDILIRKSSEC*

>CD630_12960 Clostridioides_difficile_630_NC_009089 segregation and condensation protein B

MKREDIKYIIESVMFAYGEPISIKELNYIINKELSSKEIEIMLNLLIEEYREQNRGIQII

KLENKYQMCTNKDYAEYIKKIIEPKKKKSLSQATLETLTIIAYKQPITKVEIEDIRGVKC

DKVLQTLFENELIREAGRLNKIGKPIIYKTTDEFLKLLNIESLEELPPIENYQEVATNE*

>CD630_12970 Clostridioides_difficile_630_NC_009089 hypothetical protein

MELNELFNFKYLIIAVIFIISVTMLILISHINILIIADIKNKDICLKLNIKYMFNLININ

RQLYPAENSKNNDKKEGMKNNIDSSILLADDLLSIYRLLKKIKIHELYSNINFGTGNIGL

TSSVYVLINTLYGNLFNMIDAEKMYLNVNPDFTKDYVLGNIRIHIRPRIKALFNIIIMIN

KIMNKNKGNKEGDSNESNRFDTESYGNNS*

>CDIF1296T_01365 Clostridioides_difficile_ATCC_9689__DSM_1296_strain_DSM1296_CP011968 sporulation protein YtfJ

MRATGSIQSLMETTLETIKGSIDANTIIGDPIKTDTTVVVPISKVTIGFGIGGGEYSKGY

DDKDREIELKNEKSDTNFAGGSAGAISVQPVAFVVVESGETRIMSLDSNINLVDNILSIT

PRVLEKIQNISQSNKNQDKNNM*

>CD630_12990 Clostridioides_difficile_630_NC_009089 MarR family transcriptional regulator

MSDKELSILLLESMNNFHNLIKIINNERYKKDKVLTERQFFALVKIRKHDKIELKNLSRD

LHVSTSSLCILLNKLVEQEYVYREEDSRDRRNTFYGITKNGEKILDNEILKFVSIISDKM

DCLDIDNKDKLFTSLEESKNIIEQLF*

>CD630_13010 Clostridioides_difficile_630_NC_009089 membrane protein

MFKIFLFSSEQFVSLFIFGLFLYYCPKLTKNILPYSYTVEKIICTLLVIIMALEQLLLIS

SGNYSTLNSLPIGINYICIYLCIAILIFKQYHLFNIFFSWSLVCSVGELIFSKNLGYEFP

SLIYFIFILSKCLIIYADIYMVDVRKFRVNRYALRDNLAICFIYFSFIFLLNTFTNSRYY

YGFLSHSTTAIFTFIFVTSIMYIPALLFNRDTFILEKKKKSK*

>CD630_13011 Clostridioides_difficile_630_NC_009089 hypothetical protein

MGIIESASKLAEMVHLLAVEKGITDIEAWDEAVKEYSKIYEERRNE*

>PCZ31_RS06185 Peptoclostridium_difficile_strain_Z31_NZ_CP013196 mazG nucleotide pyrophosphohydrolase

LKGAFMKLKTISLPELNNLDPTLESTFIKMGEEQGELAECIGKFRNLSGENNDLDEVDII

KKTAKELMDVAQTCVTMMFKLEEQYGINLDEIRKEHIKKLEKRGYIKNMDK*

>CD630_13060 Clostridioides_difficile_630_NC_009089 ribosome maturation factor RimP

MKKNLEATIEEIVTKITDEHGFEMVDVEYVKEAGEYYLRVYIDKEEGISLNECELVSREL

SPILDEKDPIKENYFLEVSSPGLDRALKKDRDFVRYQGRDVDLKLYKPLNGCKQFEGELV

GLTEDNNIKIIANGKEMEFNRKDVAIVRLAIKF*

>CD630_13070 Clostridioides_difficile_630_NC_009089 transcription elongation protein

MNHEFMEALDELVVDRGIDKEILIDTIEQALLTAYKKNFGSAQNVRVEFDRDKGDIKVFS

QRVVVDESDLYDTFLEIELADAREINPNYELGDIIEHEVTPKNFGRIAAQTAKQVVVQRI

REAEREIVYNEFIEKENEIVTGEVARANKNVVYVNLGRIEGIMTQSEQIPFENYKAGQKI

KFYILEVKKTNKGPQIVVSRSHPGLVKRLFEEEVPEIYEGIVQIKSVAREAGSRTKMAVR

SIDEKIDPIGACVGPKGLRVKNIVDELGDEKIDIIKYSEDPAEFISAALSPSKVVRVDID

EDEKSALVIVPDYQLSLAIGKEGQNARLAAKLTNWKIDIKSESQAEQEMLNLENSKEIEA

EDVEAEFIDQEELDTSEDNFSEEE*

>CD630_13071 Clostridioides_difficile_630_NC_009089 hypothetical protein

MILKKVKKVPQRKCIACQDRDSKKELIRIVKNKEGKIFLDPTGRANGRGAYVCKSSECLK

KAIKSKALNKAFKIDVPDEVYDNLLMELEEYEK*

>CD630_13080 Clostridioides_difficile_630_NC_009089 ribosomal protein L7Ae/L30e/S12e/Gadd45

MKNNKEKVLSFLGLATRAGKIVSGDDSTLLDLKKGKVNLILIAEDASNNTKKLFKDKSTF

RNIPYLFFSTKEEIGFAIGKSPRAVVGIKDENFSKKIIELIEI*

>CD630_13090 Clostridioides_difficile_630_NC_009089 translation initiation factor IF-2

VSKTRVYQIAEELNISNEELINKLAELDINVTDKDSVLEGEELELALEMLGEDLSQENGN

VIEIDGKLTVQVLATKLDKSPSEIIMKLMKMGTMATINQEISFEIAALAAKDYGFELTVA

ESDDTEALEIEALMEIEEDKEEDLKPRPPVVTVMGHVDHGKTSLLDAIRKTDVISGEAGG

ITQHIGASEVKINGHKIVFLDTPGHEAFTSMRARGAQVTDIAILVVAADDGIMPQTVEAI

NHAKAAGVPLIVAINKIDKPGANPDKVKQELADQGLLVEDWGGEVIAVPVSAKKKEGIDT

LLEMVLLVAEMEELRANPNKRAVGTVIEAELDKGRGPVATVLVQGGTLTVGDPIVAGVAC

GKVRAMINAKGKRVKTAGPSTAVEILGLSEVPQGGDQFVEVPTDKIARSVAARRQQIVRD

EMLKSTQRLSLDALFSQMSEGSIKDLNIVIKADVQGSVQAVKQSLEKLSNEEVQVKVIHG

GVGAVTESDILLAAASNAIIIGFNVRPVPGAESLGEKENVDIRTYTIIYKAIEDIQAAMT

GMLDPEYVDEETGKAEIREIYKISGVGTVAGCYVTNGKIFRNCKVRLVRDSIIIHEGELA

ALKRFKDDVKEVNSGYECGMSFVNYNDIKEGDIVEAYITKEVERKL*

>CD630_13100 Clostridioides_difficile_630_NC_009089 ribosome-binding factor A

MASYNRTRRIAEEIRKVVSTMLINGVKDPRITSMVSVTDVEVTNDLRYAYVYVSILGGDE

ESTLTGLKSAGGYIRREVGKNIKLRYIPEIVFKLDDSIEKGMYMDSLIKRVNEKNAQQNE

DENYDE*

>CD630_13160 Clostridioides_difficile_630_NC_009089 30S ribosomal protein S15

MINKSEIIKEYGRAEGDTGSPEVQIALLTARINELNGHLKTHKKDHHSRRGLLKMVGRRR

NLLAYLKEKDLEGYRALIAKLGLRK*

>CDM68_RS06575 Clostridioides_difficile_M68_NC_017175 fatty acid-binding protein DegV

LKIKLVCDSLCDIPDEISEKDYIEIVPLTVIFNDREYIEGVDIKKEEFYKKVKEIKQIPK

TSQATYMEFKEVFDKFVTEGYHIICMTGAANASGTFQSAMIAKNDVNENEKEKIHIFDTR

NLSLGSGQYVIKACELLEEGLGFEEIIDELENTRSSVRLLFAPYTLDFLKQSGRVPVATA

LIGNMLNIKPIFFFDNGEAKLVNKVRGIKNIASKLVDIILEMNEGSLEGKIVTIGCGDNL

HDCEILKDEVNKRIKARRVLFTRGGVSICSHTGPDILAISCSN*

>CD630_13180 Clostridioides_difficile_630_NC_009089 polynucleotide phosphorylase/polyadenylase

MFEHKIFKMDFAGRELSVEIGKICEMASGSCIVRYSDSMVMVNTTKSAKPRDGIDFFPLS

VDYEEKLYSVGKIPGGFLKREGKPSEKAILTSRLIDRPIRPLFPKGFRNDVQVVATVLSV

DQDCTPDIVAMIGSSIALSISDIPFNGPTGSVCVGLVDGAFVVNPNAEQREKSSMHLVVS

GTKEAIMMVEAGADEVPDEVMLDAILFAHQEIKKIVEFIEGIVAEVGKEKMPVELYHAGE

EITQLVREFATDKMKKAVQTFEKLERMENMDRVKEETLAHFEETLEDFEDFVGDIEEVLQ

DIIKEEVRKLIVHENVRPDNRKLEEIRPIWCETGMIPRAHGSAIFTRGQTQVLNVATLGA

LGDVQKLDGLDEEENKRYMHHYNFPAYSVGEARPSRGPGRREIGHGALAERALLPVIPSQ

EEFPYAIRLVSEVLSSNGSTSQASVCGSTLSLLDAGVPIKDMVAGIAMGLIKHDGKVAVL

SDIQGMEDHLGDMDFKVAGTEYGITAIQMDIKIDGIDKEILQRALKQAKEGRIHILGEMR

KTISQPKPELSPYAPKIVKMQINPDKIKDVIGPGGKIITKIIDETGVKIDIEQTGEVFIS

GIEIDMIKKAQELINNIVVEPEVGKTYKGKVSRIMNFGAFVEILPGKEGLLHISHIAHER

VAKVEDVLNIGDEVEVKVTEIDEKGRVNLSRKVLLPKPEHKNK*

>CD630_13200 Clostridioides_difficile_630_NC_009089 M16 family peptidase

MYKTKILENGLTIIGEEIPYLKSITLGIWINAGSRIEEAQVSGTSHFIEHMMFKGTKNRT

SKEIASSIDNLGGQINAFTSKECTCYYVKLIDEHIDTGIDVLSDMILNSKFDKNDIDKER

LIILEELKMYEDSPDDLSYDLLVENIYANDGLGMNIIGTKESLYNITRESMLEYLNKYYI

PNNAVISIAGNFNFDDMVEKIKSKFGHWEKKNLSIDISEAKFNPCFISKNKDTEQVNLAM

CLKGIPFENDEEVYSMAVVNNIFGGSISSRLFQKIREEKGLVYSIYSSQTLYRKCGELGI

FASMSTENLQDVYNLIKKEIENIRENYLTEKEISESKEQLKGNYILDLESTSSRMMSTGK

SMLLSKKVKTTDEILECINNVNINSIKKVVDKVFNIENIGTCIVGRDVEKILHLD*

>CD630_13220 Clostridioides_difficile_630_NC_009089 aspartate kinase I

MSILVQKFGGTSVESYEKMNEVCKIVKAYKKNDEELQLVLVVSAMGRKGAPYATDTLINL

CSAVNDEPSKRELDLIMSCGEIISGTILANLLNAQGIDSVFFTGQQAGIITSDEYSNAKI

KYINPKKVKRALDDGKVVVIAGFQGVTDDGEITTLGRGGSDTSAVAIGKALECETVEIYT

DVDGIMTADPRVEPNAKVLSFIDYEEVFQMADKGAKVIHPRAVQLAKSGNITLAIKNTMN

PTFEGTKIGSLCRHLEDNIEYEQERDFKVAVANKDSVAQVKIKSAEEVFTEVLNEIEKKL

ITIDMINFFISEKAFVVEDADIKSLKEILDKFELDYEVNRDCAKVTLICSRIADEMSGIM

SKVVRGLSKAGVSLLQTSDSNMTISCLVSEEDMHTAVHAIHQQFYLK*

>CD630_13230 Clostridioides_difficile_630_NC_009089 protein export-enhancing factor

MKDYREDNERFDKDNCSEESSCEERECIKQFGTNEMPPQPPKDIQCITIIGEIEGHFIGN

PQKKATKYEHIIPMLYSIEESNDVKGVLVVLNTVGGDIEAGLAIAELLNSTSKKVVTLVL

GGSHSIGVPLATAGDYSFIAPTATMIIHPVRTTGLVIGINETFEYFKKMQDRIIQFIIRT

SNIKKDVLEKLMHEKDELVSDVGSVLIGKEAVDYGLIDEVGGLKEALKKLRELIKESEEE

KNND*

>CD630_13260 Clostridioides_difficile_630_NC_009089 30S ribosomal protein S12 methylthiotransferase

MLKIALESLGCSKNLVDAEIMMGILNNKGYKLIGDFEEADVIIVNTCGFIESAKQESIDT

IINFAELKKTGNLKLLIVTGCLAQRYSEELKTEIPEIDAIVGTGSYQNIDKILKELSEIH

QIVSLNDIEFVFNEDLPRYISTPSYMAYLKIGEGCSNNCTYCIIPKLRGKYRSRKFEDII

KEAKKLAESGVKELVVIAQDTTKYGFDLYGKERLSELLEELAKIDGFKWIRVMYSYPESI

TEELIQVIKKYDNICSYFDMPIQHASNNILKLMNRKTTKEDILNKINLIRSNIPDAILRT

TIIVGFPGETEDDFKQLVDFVEEVKFDRLGAFAYSREEDTPADRLPNHIDEEVKIQRRDT

LMMIQQKISEELNDKKIGKTYEVLIEEQIEDNVYTGRTQGDAEEIDSIVYVKSVDNLEVG

EFVSVQINDAMEYDLMGDVLYELA*

>CD630_13270 Clostridioides_difficile_630_NC_009089 CDP-diacylglycerol--glycerol-3-phosphate 3-phosphatidyltransferase

MNLPNKLTLFRIFLIPVFVLIMLLNVPNKFLIACIIFIIASITDALDGKIARKYNLVTDF

GKFMDPLADKLLVISALTCMIEDHLVSSWMVIIIVARELTVSILRAIAAADGKVIAAGNS

GKLKTITQMVSIVFLLLGAQFENVLILNIGEILILIATLLTLYSGWEYLYKNKELFMSSK

*

>CD630_13280 Clostridioides_difficile_630_NC_009089 recombinase A

MSVDQEKLKALNEALGKIEKDFGKGSVMKLGEATSMSIDVISTGAIGLDIAIGIGGLPRG

RIVEVYGPESSGKTTVALSCVASAQKDGGIAAFIDAEHALDPVYAKALGVDVDNLIISQP

DTGEQALEIAEALIRSGAIDIIVIDSVAALVPKAEIDGDMGDSHVGLQARLMSQALRKLT

GSIKKSNCVAIFINQLREKVGIMFGNPETTTGGRALKFYSSVRLDVRKIDTIKQGDKVIG

SRTRVKVVKNKVAPPFKQAEFDIMYGEGISKIGDLLDIAADVDIVKKSGSWYSYNDTKLG

QGRENVKKFLEDNLDLTTEIDEKVRAFYNLNEEHEESGTSVSKEIVEE*

>CDIF1296T_01398 Clostridioides_difficile_ATCC_9689__DSM_1296_strain_DSM1296_CP011968 phosphodiesterase

LGLLTKEVDVIDSVVMIVIGAAVGIIAGYFVRKNISEAKIGQAENLAKEIIDKAHHDSET

VQKEKLLEAKEEIHKWRTEAERENRERRTEVQKYEKRVVQKEEVLDRKLQNLESKEVNLS

EKLKVVEKKEEEVEVIKTQQLEKLESISGITSDKAKEIILTNAERDVRREMSIMIKEIES

QAKEEADKKSREIIGYAIQKCAADHVAETTVTVVSLPNDEMKGRIIGREGRNIRTLETLT

GIDLIIDDTPEAVILSGFDPIRREIARIALEKLIADGRIHPARIEEMVEKARKEVDNIIK

EYGEQAAFETSVHGIHPELIRLLGRLNYRTSYGQNVLKHSIEVAHIAGIMAAEIGADIRL

AKRAGLLHDIGKAVDHEMEGTHVEIGMDLLRRYKESKEVIHAMSTHHGDYEPQTVEAVLV

TAADAISAARPGARRETLEAYIKRLEKLEEIANSYEGVEKSFAIQAGREIRIMVKPEAIN

DEEIHLLARDMTKKIEDELEYPGQIKVSIIRETRAIEYAK*

>CD630_13300 Clostridioides_difficile_630_NC_009089 UvrD/REP type DNA helicase

VSQYININSQLDALKLKEFEKIFYENINEDVKVVPKVTPFKGINTDLLYVKDGRILFIKF

MDTTEDIFFILEEELLEVMNEEYELLKLKMGQKNRNISYNYVYIMPYVEVEETYEFEEFV

NNNIIDKNKLQDIMNKGSLEEYLNDENDEINLNLFLLDICSEYYIINDKLHLNEKFKKIS

FYNDDYKYTATMMEDVQIKDVVSIKYGNTLIEGGSGIGKTAIMLSRAIKLARVYPHHKLV

IFTHTKQLCNELRERIELLYKDNNNLEVHTFSSFIFKLAKKFNLIVDYNMLKNDYEKTFN

NLVKQAQNIIKNKNMFKAIFIDEAESFLEYEIDFIREFLYKTKFIFNVCSCNSLNISNRL

NIFKKLYNGIEFDDKIILSKNYRQAKEIVDFTNKFSNNSNSYINELRPNTEFSTFFYTKA

LRGGNKSVDIIKVSDLDDQISSVIWEIEYLISKKGLDYSEVAIVYPYNKKKLKSGKTIYF

QYMLKKALEEAKIPYICAEDNLTNISKKVGTTIANIYAIKNLEYKAVIVCELEMLYNQTI

NDIEQDYQVNDFVGDLNKVYLAMSRATDYLSIVTTFNEEASDIIRLITESKDI*

>CDM120_RS06980 Clostridioides_difficile_M120_NC_017174 phage tyrosine recombinase

MYSTKILDDYEYETFNVFSTKLNPNTKHDYLSKVILFKEFLKGKELIYATKEDCKNFVDY

IQTKYAKSTCEKIYSYLHSFYNFLKKEGYIDINPFRYVEKPTVTRIKTKDDVLSIQEINK

LIGILPKLNIRDRVIIVCLVTTGCLLNELVSLKWKDLMVDENDNSYVRLGKGRKERVVKL

HPYFFKLLEDYRNYSGLPEVIIPSDDFIFTTQKSNSITDRNVRLIVKKALDLAGLSQYSA

RDFRHSFAAISLRLGADESDVKNQLGWSDKYYAIRYKYVLNFVDSEIVDYLIEKDNLDIN

KKY*

>CD630_13350 Clostridioides_difficile_630_NC_009089 MarR family transcriptional regulator

MNYIVKDTISFLVKTFPKIYSSLYLEDLKKFAPDYNVNKTQLRALVFIKNYGVISMTDLC

SKLNIEKGSLTSMVDDLTDKKYVTRKRDLVDRRKYLIDITEEGDKIATDFMDKLSDGLGE

KLSKLTEEDRKKYLEAINTLQYILNKEEFR*

>QAE_RS0206550 Clostridioides_difficile_QCD_23m63_NZ_CM000660 aspartate aminotransferase

MNYSNRVSAMQASPIRKLVPFAQAAKDKGIKVYHLNIGQPDIKTPKGFFDAVKNFDSEVL

EYATSEGIPELLEALQNYYKTYNMNFEKDELLVTNGGSEALLFTMMAVCDPGDNLLVPEP

FYTNYNGFGQSVNVEVNAVTTKAENGFHLPSKEEILSKVDDKTKAIILSNPGNPTGAIYT

KEELNILAEIAKEKDLWIIADEVYREFVYDGLEYTSCGNLEGVQDRVIIIDSVSKRYSAC

GARIGSIACKNKGLIAQILKLCQGRLCVPTLEQIGAVELYKTPVSYFKEVNEEYKKRRDV

LYNELMKVEGVICKKPTGAFYIVAKLPVENAEDFTIWMLKEFNKDNETVMVCPAEGFYAT

PGLGRDEIRLAYILNEKDLHRAATLLKEGLEQYVALTKIIF*

>CD630_13410 Clostridioides_difficile_630_NC_009089 exodeoxyribonuclease

MKFISWNVNGIRACVGKGFLDFFKEVDADIFCLQETKLQEGQIELDLPGYFQYWNYAERK

GYSGTAIFTKKEPLKVMYGINIEEHDKEGRVITLEFEDFYFVTVYTPNSQSELKRLEYRT

RWEDDFIDYLTKLDNHKPVIVCGDMNVAHKEIDLKNPKNNMKNAGFTKEEREKFSKLLDS

GFIDTYRYFNPDKEGVYSWWSYRFNARKNNAGWRIDYFCASKKLEDRLVSADIHTEILGS

DHCPVELEIK*

>CD630_13420 Clostridioides_difficile_630_NC_009089 ribonuclease

MEYIIFDLEFNQGFDKKLNKTVSNEKCPFEIIQIGAIKLDSKFNIIDTFNSYIKPTIYKD

IHPFISRMTNIKNSDFNDSPTFPEVYNNFIKFISSQDPILCVWGAGDLKELYRNINYHKL

PSNSLPKSYINIQQHASKYFNNPAGKSIGLQNAISILELDEKMSYHNALNDAYYTAKVFI

KIYNPSIVPDIYLYTSIKPKTIRYSNKKRVDYDKLFDEFRKILNRELTKDEKKIINLAYN

MGKTNQFTLENVKQRKNK*

>CD630_13430 Clostridioides_difficile_630_NC_009089 glutamine synthetase

MDSLLYVIEKDKHTNEELREILKSNKNIRFVSLMGVDLGGNATDEKIPVELFLEDIDKFL

ESAIQTDGSSVELYNIATLNNAKVDLMPDKSCHWYVDYNMEYIDEEVGLPVGTLKIPAFL

IHDNKKVCSRGVLQKADKYFKKSMYEIFREYPHVINNIGIDSVDDIEEIMLTAATELEFW

VNTPEDKADLEKLYVSQSLKEQYWKRTHGIIRTCLEKSLIILQKLGVNPEMAHKEVGGIQ

SSISIDGRTNHAMEQLEVSWKFSTPLQAADNELLVRDVIEDVFTSHGLEVTFKAKPIHGV

AGSGGHTHVGVSAKLKDGSIKNLFAPKDLKEDYLSELGYGALMGLLYNYEVLNPIVTASN

DGFNRLVPGFEAPVCIVTSLGHSYEIPSRNRSVLVGLIRDMKNPKTVRFELRSPSPLSNT

YLVIAGCYQTMLDGIKAAAKSGLSTKELEKELSKNVGEESFYLEKDRAYRDENDVFEHYS

LEERNARFGIPPATVYENMKNLEIYASKLKSLKQGDVFTDSIIESFKIGAIDKWQKKLKT

RIIEAGIQKIRSIVKIHTKENMDALDEVVWNSISDLKFNIMKDTLTRESLFTRVREAIEN

KDYQTASDLQIELKRSMEEIQQLYMQYKKNIY*

>CD630_13450 Clostridioides_difficile_630_NC_009089 PadR family transcriptional regulator

MNTQFRKGVLEICVLALISKKDMYGYEIVHNISKVIDVNEGTIYPILRRLTKDLYFETYI

LESNEGPARKYYRITSLGKENLSNLMEEWREFVKAVEILISEDEGGNELE*

>CD630_13470 Clostridioides_difficile_630_NC_009089 hypothetical protein

MNKKLAIFAAVLLVIGIIGTTWSGILVMPSLINFGLEKEAEFKKENKLYQEKVNIDKLDI

AVDNINVTIKKSSSEDVRVTTRGNNEFYKYNVTLKDKTLVVKGERKYENKIKKIKNFDQL

LNTSINSMFSHDYREIIIYVPNNVDINASSISSHLFVYDNVASNTITYKTSYGGFSRAIT

ENKVNRLENLNLISNNNLHLSTKSILGVKNVNIESESLYISSENEDVFINNIEEYIPENV

NIKEKIGRNSNYESEFYLYSDMPIAKFLDIEVPNSKVRLDIPVNKYKFNCDIKSKEAIEE

FNNDEEYDNDSYDYEHNEEHSNKYRNTREIKGLLNKNLSNLEKEYTIKINSNSLEL*

>CD630_13490 Clostridioides_difficile_630_NC_009089 lantibiotic/multidrug family ABC transporter ATP-binding protein

MSNYILQVKGVSKKFKEQMAVDNISLAIKSNSIYGLLGPNGAGKSTLLKMITGILNPTSG

KIMFEGHRWTRKDLSNIGSLIESPAIYENLTARENLKVHTTLLGLPDSRIEEVLETVDLR

DTFKKRSGQFSLGMKQRLGIAIALLNHPKLLILDEPTNGLDPIGIEELRELIRSFPKQGI

TVILSSHILSEVEQVVDEIGIISNGVMGYQGEVSKEQNLEKLFMKVVAENRKRGE*

>CDM120_RS07055 Clostridioides_difficile_M120_NC_017174 lantibiotic ABC transporter permease

MTQLVRIFCSDLIKLKRTFIILMHFCIALIGMGLCLGYYKYSSADDISKIAAYLQVIAIA

FPLLSSIMCSLCIEQEYYSGSYKHMLTSSNPKYLTLISKYIILICLGFGATLVSVLGFKF

GVSSISNEVYFTLDFYMISIMILVGSNLFVYILHLFLSLRFGKGASIGVGIVETLLSAVL

LTGLGARIWPYIPCVWGVRFISIWSSFSSSKTIEYIKVESIKGYQSIGLVCGFVTILAFI

ILCIWFSKWEGKKSEE*

>CDM120_RS07060 Clostridioides_difficile_M120_NC_017174 two-component sensor histidine kinase

ILVGKKFNNKKIVTLRTVFVRYSSLFFAITVVLVSILILSFPILLSLNIILPANYVEKQI

YENKDKIISSKQVTKDLIPDLCEYGVYTLNGKVISGTFNKNESKEVWDLMRGFERRTITS

SKNYIKLSRKNEVCIIRYSIVAEFVSPTLRTYLPKPELLGMIIFSIIFLIEIIILSKLFG

KKLNAEMELLKNTTEKIEQQDLDFVIESSKIREINNVLFSMDKMKLALKDSLEKQWMLEE

NRKEQISALAHDIKTPLTIIHGNTDLLIEINENPELSEYMEYIAKGATQIEKYINTLIEI

SKTETGYILNKEVINVSEFIEDILIQIEALARTQNLNVEFSKQGNLPESITIDKELLFRA

IMNVISNAIDYSPSQSKLYISVSVSNKYLKFVITDCGSGFSKADLSKATGQFYTGDLSRN

SKSHYGMGLYIVNNIVQKHNGILHIENSIKTGGAMVTIEIPIV*

>CD630_13530 Clostridioides_difficile_630_NC_009089 phosphomethylpyrimidine kinase

MQKKVAAINDLSGIGKCSLSVAIPILSALKVQCCPFPTAILSSQTGYPEFTFLDFTDEMV

KYSNVWKNLKVNFDSIYSGFLGSKHQIEIVANFINDYPNAFIVVDPVMGDNGVMYPIFTE

EMRQEIKELVKHSDLTTPNLTEACFLTGNDYTKSDYNRDELIYIAKSVSDLGPSKVVITG

ILEDDNILNLAYDRDNDHVFFTSVKYNNCSYSGTGDIFTSILCGMLVNKHDLGVAVNTAT

DFIYKTINYTSQFDTDRNDGVMFENFLSDLTNI*

>CD630_13560 Clostridioides_difficile_630_NC_009089 CBS domain-containing protein

MNILFFLTPKSEVAYIYEDYTIRQALEKMEYHKYSAIPIISKDGKYVGTITEGDFLWTLK

NDLNLDLKGLEDVPVTDINRKMDNSPVSINADIEDLVIKSLNQNFIPVIDDQDTFIGIIK

RRDVIGYCYEIIRGYKNLANDN*

>PCZ31_RS13970 Peptoclostridium_difficile_strain_Z31_NZ_CP013196 peptidylprolyl isomerase

IHYEGVNYYGKKVLATVGEKEITNIDIENALKSLDPYQAMQFKTEEGKKHLLNDLVNQEL

FFLDAKEEKLDEEEIFKLEMKKIEENVLKQFAINKVLSSVNVTEDEKVKFFEANKSSFSK

PESATAKHILVDSDEKAKEILAQIKSEEISFEDAALKHSSCPSKDMGGDLGTFGRGQMVP

EFEEAVFSMAKGEVSEPVKTQFGYHIIKLEDLQESTESTFDEVKAEVEKSLLYQKQNEVY

GNKINALNAKYGNLVKYND*

>CD630_13580 Clostridioides_difficile_630_NC_009089 membrane protein

MYKEKLFDNYFKFLALLFWPIMWYKWIVISNGTLENMLFTIYAIVAIVFIILYSVFMIKY

KDITQIDFFYRISTLLAFIFTLFSFLIYPKSLFFLYLKIIFTGIYLYYSIVKTLKFKDDE

GVVGIMSSLLLIVITLFY*

>CD630_13590 Clostridioides_difficile_630_NC_009089 hypothetical protein

MNNLDKLFELASQEGIIIHYTTYIAGDLEGLYINKHGIKIISLLSNLKQNSKKLTSILAE

ELGHHFTSLGYYVSSYNDYYTKIIIDKCENKALKWACEFLITEEDIINIINSGITCVYEM

ADILNVDITFFQKRLEFLSLKKQSLQLGNNKYLILTNLPYFYIFDPIS*

>UAB_RS0207695 Clostridioides_difficile_ATCC_43255_NZ_CM000604 XRE family transcriptional regulator

MFAKRLRELRKEFGLTQRELGEKVGVSQRVLGYYETENRFPDEHILNKLADVFNVSVDYL

LGRTLVKENIDTVAAHRKNPHEELPEEAQEQLNDYIEFLLNKYKKNKPYDEQSIFLLF*

>PCZ31_RS13990 Peptoclostridium_difficile_strain_Z31_NZ_CP013196 XRE family transcriptional regulator

MFKNNLKYYRKCKGMTQIQLARKAGITNDYISQIERGIKNPGLLMAKKISSILEENIEEV

FLYSHRTICS*

>CD630_13610 Clostridioides_difficile_630_NC_009089 hypothetical protein

MENKKDILFKETDKRLHNYKYLDIKIKNINLDIKRCENEYSGCGAMVYTEKTSNTYNISS

SVENEVLKREERLRKLKMEKEDIEIEKEKIENALTCLNDIEMEFFNLFYNSKTKNNMTYI

SMKLHLDRTSCYNLKKKMIFKLSEIL*

>CD630_13620 Clostridioides_difficile_630_NC_009089 hypothetical protein

LLKYKEILETIIEILKKNFTESIFIDDESVQGSEGSCFFVSILSVICTPVMLNTNNKDIV

ISIKYLPKPQSKSIRMYEVSDELNKLFNRNIKVTDRKLNITKLEQSIKKEESIYVLNFTL

TLNYLDSVYEEDVVYENMEEINLNLGE*

>PCZ31_RS08230 Peptoclostridium_difficile_strain_Z31_NZ_CP013196 hypothetical protein

LLSYKDILYSFTKELGNNFNEDIFVEGYNIQDNKKSCFFVQILPEVAQTATKKTDIKSFL

VDIKYLPDWKKKKTDLFDILNKLENIFTRNIKVKDRYLTFSKKNGSIEKDEIGNYVQFLI

SINYHEQIYFEEEKHELMEELNMRFKGRSD*

>CD630_13630 Clostridioides_difficile_630_NC_009089 XkdK-like protein

MAIGLPSINISFKELATTVKERSARGIIAMVLKDAKALGLNEIHEKEDIPVDLSAENKEY

INLALMGNVNTPNKLLVYVIEGEADIQTALDFLETKEFNYLCMPKAVEADKTAIKNWIIK

LRDIDKVKVKAVLGKVVGNHEGIINFTTEDVLVGEKKYSVDEFTSRVAGLIAGTPLSQSV

TYTKLSDVVDIPKMTKVDAESKVNKGELILIKEAGAIRIARGVNSLTELTAEKGEMFQKI

KIVDTLDIIHSDIRKVIIDDYIGKVTNSYDNKCLLIVAIKSYLEELEKSALIESDSTVEI

DFEAQKSYLKSKGVDLSYMTLQEIKEANTGSKVFLKAKIKVLDAMEDIDLSIEI*

>CD630_13640 Clostridioides_difficile_630_NC_009089 XkdM-like protein

MANMEARNVMSGTWGELWLDGNKVAEVKKFQAKMEFTKEDIIIAGQMGTDTKYMGYKGKG

SITLYHVSSRMHKLIGEKIKRGSEPRFVAISKLNDPDSYGAERIAVKNIAFDDLTLADWE

VGVKGEIEAPFTFTEYDFLDII*

>CD630_13650 Clostridioides_difficile_630_NC_009089 XkdN-like protein

MSENGLLKNINIVDLLLNADTENLERPSTIVELKRLSTIFGQEFKVMCRALTISKDEEIQ

NTCLKIDENMKTDIDLPEMQMLTIIEGVCDLDGKLLFKNKELMDKFKAPTPKELARKLLL

PGEITNLYRILQDVMGYGKNAVIEEVKN*

>CD630_13670 Clostridioides_difficile_630_NC_009089 XkdP-like protein

MEMWLRQAEDRFRFPVFPSSFSINGKAAVNSSSILKIGEIATFGGVALKSISISSFFPNK

DYTFCDYTGFPSPYDCVNKIEKWMKEGFILRFTITETNINMEVIIEGFSYEERDGTRDVY

FTLDLKEYKRIKIPKVTPKQ*

>CDIF1296T_03821 Clostridioides_difficile_ATCC_9689__DSM_1296_strain_DSM1296_CP011969_plasmid_unnamed phage cell wall hydrolase

VDKCTWSGDYRSPSRTLEFSIIQSASDINFRQIDIPVASTVCFYVDDKELFRGMIIDRSK

DSSNNNIDFTAKDMGFLLLQSEVSYNFKDKLVEDIAKQVFVDNKFPLGNLPKTNVKYTKM

FIGVTGYDTIMSVYTEASKTTKKKYMIESNLDKFNVIEKGVVTLNITFEEGFNLINTNFS

ESMENVKNKVLVVDQYGNKISEKINDSIFKDVGVIMQKVIQQQENSTIDIDGEFKGIEKT

CSLKGYGDITCVTGRGVKVKDSYTKLIGLFYIDTDKHTWQNGDYQIELELNFENIMDEKS

AGQDEQKEDGSEYTGGTEYSAEFTAYCPRKSEGGDKDCRGKKLDPSKKTCAAPMVGTYEK

SYYTKDFLNKHPLMRYGDEIQLVTGVSSRDGTYKVNDNGPAIIIEKDGTYHIDILFGNVE

EANNFGRRKGKIIIGGYSGNVTEKAKTVISEARKHLGKPYKWGGNGPSSFDCSGLMVYCF

KKVNVNLPRTSSQQSKVGKKVEKNNLQAGDLVFFHNPVSHVGLYVGNGEFLHAPQTGDVV

KISKLSNRKDFNTARRVL*

>CDIF1296T_03822 Clostridioides_difficile_ATCC_9689__DSM_1296_strain_DSM1296_CP011969_plasmid_unnamed hypothetical protein

VADPINEFIGIMRQEGKFHNEPSFFIGKIKSKLPDLKIEINNIILEKEDILVDSWMLDRQ

IELFDTETSQEHKHEIKNPFIDTFESGNTVIMFKIGEKFAVVSKLVSLDE*

>CDIF1296T_01436 Clostridioides_difficile_ATCC_9689__DSM_1296_strain_DSM1296_CP011968 hypothetical protein

VANSINEFIGIIRKEGKYYNKPSFFVGKIINKLPDLKVSMNNIVLDKDNLLIDNWLFDRA

TKSFIITQSNNHTHELNESLTNQLDKGDTVIMFKVGEKFAIISKLVSL*

>CDIF1296T_01437 Clostridioides_difficile_ATCC_9689__DSM_1296_strain_DSM1296_CP011968 phage protein

MSNTIFPFIGVPEDYEIPKKEELGLFREVAWNFEKDEPIVENGDFKIIEKNEAIKVWVYK

CIKTNKNEHEIYSNDYGTELTDLIGQKYSKGLTESEASRYIKEALLMNPYILQVDVVNTN

FKKDILGAYIKISTIYGEVEINV*

>CD630_13710 Clostridioides_difficile_630_NC_009089 baseplate assembly protein

MYSDQTYEVIKNRTLENINLDIYKGEGSFLNNMVSGNNLELSKIYLELSKMHKMAFIQDT

YNQFLDKRVNEFGVYRKLGTESNGEVEFIGEKGTVINNGTIISYRDLLFVVIKDVTIGSE

EGDNSPVQALEVGKKYNLPTNCEFKLVDNISGVTKITNTRSFEGGTDIETDEELKERFYK

IQRNQATSGNKAHYEEWALEVDGVYNVKVYPRWDGPGTVKVLIFGKNNQAVDTETIERCQ

QHIDEEKPIGPTITVVTPLPIEISISAVMKLEDGYTLDNVKESFLESINTYFRDIRGEII

YTKVMGILINTTGVHDLSNLLINGSTDNITINEDKIPSVTTVNFSEVENQ*

>CD630_13720 Clostridioides_difficile_630_NC_009089 XkdT-like protein

MKLIDKLPSFDRNYIVEEIQGAYDTELNILKEDIDDTFNQLFVDTATWGLDMWEDILCIE

KKELDFDTRRSNIKAKMRSRGTSTIEVIKSICEAYTKSETDIKVYSDEFTFVLSFIANNC

DYKTLLDCSDMIERVKPAHLLHYLEPIILDKSMVYCGGGMVCSEEVKVHPYFEPIIKCSA

VVNCGAGMLSREEIKVYPLSIKCIENNCKINIAIANDTGVENVVVYPKSEVV*

>CDIF1296T_01439 Clostridioides_difficile_ATCC_9689__DSM_1296_strain_DSM1296_CP011968 phage protein

MKLIDKLPSFYKNYMVEAIQDSYDSELSSLKENIDDTINQMFVNTSTWGLDMWESILCIQ

NNEDLNYETRRSNIKAKMRSLGTTKLKVIKNICEAYTKTDVEVTVLSNEFIFILEFIVNN

CSYNSIVELDKVLENVKPCHLEHKFKMILLNKNELFCGTAINTGETVTVYPWTPSNIEIF

GEITISTGNDRSMEKVILYPKQEAI*

>CD630_13770 Clostridioides_difficile_630_NC_009089 hypothetical protein

MGLRDKFAQSFARSKTMSGPEKKANEIMGKLLLKKAILPIVLMFVIIIAGAMLKINSWVT

LGINLVIAVGAFFYIRNSSKKYQNFKPYVGNLISLEKKGKKEYVAIIKQGKLPVKLQIAY

GGEDLEHVKKNQMVQISYNPDAKIAILVNRQ*

>CD630_13780 Clostridioides_difficile_630_NC_009089 transcriptional regulator

MDTLGERIVYLRKAKNLKQYELEEMLGCDNLSKFERNIRKPNYEILKSIAEIFNVSVDWL

LNGDNLSHKSDLICDSSSNYPLNSINSNEIKLLNNFRKLNDYDKAKIEGMIELKLHEYEK

EKDLGKIEYNKNKDEKIDK*

>CD630_13781 Clostridioides_difficile_630_NC_009089 transcriptional regulator

MDARKKWIPFLGVQVKQRLIELNMTQRELAKKIGVNENYLSAILNGRRTGKKYKSSIYQL

LNIEYSEDD*

>CD630_13782 Clostridioides_difficile_630_NC_009089 hypothetical protein

VNILVCKLDSDGVIECCRAIDDFITALSNIKSLNMERLNTLTKYSSTCSILLKEGNYEGC

TIVYRKMLEELKT*

>CD630_13790 Clostridioides_difficile_630_NC_009089 transcriptional regulator

VDTLGKRIAYLRNSKKLTQRKLMDILKFENLGKYETGDRKPNCDILMSIADYFNVTTDWL

LYGKEKVNVNSSVKEDKEDYLHVTNDEMTILNLYRQLNERDKIKIEGILELKISEYKDLK

KHSSNNNEDKMV*

>CD630_13820 Clostridioides_difficile_630_NC_009089 glycerol kinase

MEKKYVMALDQGTTSSRAILFNKKGEIVKIAQKEFNQIYPKAGWVEHDPMEIWGSQSGVM

REVIETAGIRPEEIASIGITNQRETTVVWSRYTGKPIYNAIVWQCRRTSEICDELKNKGL

EESIKEKTGLLIDAYFSATKVKWILDNVEGAREKAEQGELLFGTIDTWLIWNLTRGKVHV

TDYSNASRTMMYNINDLEWDEDILRELNIPISMLPLVKPSSYVYGHTDERMLSGAKIPIA

GCAGDQQAALFGQNCVEEGTAKNTYGTGCFLLMNTGSNIVKSKHGLLTTIAWGVDGKVTY

ALEGSIFIGGASVQWLRDELKIIESAKDSEMYANRVEDTNGVYVVPAFTGLGAPYWDMYA

RGSILGLTRGAKKEHIVRATLESIAYQTKDVLEAMQNDSKLKLKSLKVDGGASNNNFLMQ

FQSDILNVDIDRPKIVETTALGAAYLAGLSVGFYIGRNEITSKWSVEKEFNPNMSEEKRC

KLYKGWKKAVSRALSWEKEDELDI*

>CD630_13830 Clostridioides_difficile_630_NC_009089 GntR family transcriptional regulator

MDKNYTMPIYQKIALDIANKIYTGEIQEDSVLFGRSVLAGKYNVSPETIRRAVKILEDIG

VVKSIKGKGVIVLSPDKASSFIKKYRDITNISSYKSTLYNLIDTKSNLENEILDTINKIL

DYSNRLEIINPLVPVQFTINSNCKYIGQTAAQTKFWQNTGATIVAIKRGEELIISPGPYI

EFLEGDILLVVGDQHIYNSIPMFLYENEK*

>CD630_13840 Clostridioides_difficile_630_NC_009089 LamB/YcsF family protein

LYKVDLNSDLGESFGTYKIGLDEEVLKYISSANIACGFHAGDPSHMEKTVQLAKKNGVKI

GAHPGFLDLIGFGRREMKITKQEAKDYTKYQLGALMAFASSNGCNIQHVKPHGALYNMAA

KDKELAMGICEAIYEVDKDIILLGLYNSEMINSAKEIGLRFANEVFADRAYDNNGFLVPR

SVEGAVIHDTKHAIDRVVRMVKEGTVETLTGEVIHIKADSICVHGDNPKAIEFVKEIRKR

FELESIEVCPLENIEVCSSENIV*

>CD630_13850 Clostridioides_difficile_630_NC_009089 hypothetical protein

MSTKNITDKTKNKKDVGALIGAAFIMATSAIGPGFLTQTAQFTQDFGPNFSFVILITTIL

FIGAQVNVWRVIGVSGLRGQDIANKIIPGLGYLVAFLVALGGLAFNIGNVGGAALGMNVM

FNMNMTLGTVLSGLIAIFVFMSKNSNSLVDKITKFLALGMIIIVGYVAISNHPPVGEAVS

RMVKPENPKGLIFPIITLLGGSVGGYITFAGGHRLIDGGITGEENIKEITKSSLLGILVA

TMMRVLLFLAILAVVSKGLQLDPENPAASAFKFSAGAIGYKFFGLVLWSAAITSVIGAAY

TSVSFLKTLNPFIDKYEKYFIIAFIAISTLIMAFIGKPATLLILAGALNGLILPITLGIM

LIASKRKDIVGDYKHPTWLLIFGLIVVLISAYTGITSLSSLGALFA*

>CD630_13870 Clostridioides_difficile_630_NC_009089 allophanate hydrolase subunit 2

MGFKVELGGFQTLIQDRGRVGYGQYGVSGCGAMDEYAHRVGNILVGNSEDEASLEVLMLG

PTITFDEYTQIAVTGGDLGAKINGKEIQNWRSYQINPGDVLSFRGVKSGARAYVSIAGGI

DVPLAMGSKSTYTRAKIGGFEGRALKKGDYINTFIQEKDFTINKKLSSKYIPTYSSEIVL

RIVKGPQFDAFSNGEVEKFLSNKYKVTNEIDRMGCRLDGESIKHLNGADIISDGISYGAI

QVPGHGKPIIMLSDRQTSGGYTKIGNVISVDLYKLAQAKPNDVVKFELVDIYEAHRLLRE

QEDKIQDIYKSMKNIRVVKAKVLNDIAV*

>CDIF1296T_01457 Clostridioides_difficile_ATCC_9689__DSM_1296_strain_DSM1296_CP011968 esterase

LIKFKLYISGLYSGNVIFDGDLLIEKLNPFTNKIESLKPISKEENTYYLNLTKIDLKSLF

NNFDVYTKSLVNTDKDTVINNLGETHSKLNEYIWVQRNKKFPLDIIIVDNKIVGFICLSR

ETCTILIMDGYEEYTVLKEWEKTHKNEEIYSIRFGGNYMIDMKDGIKLSTDVYLPDFVDS

TKKAPTILMRTPYGKENDKEIYYKYVQRGYAVVIQDVRGRNESEGKWEPLIHEREDGDST

INWIVSQEWSSGIVGMLGASYLGYVQWAAASSGNKHLKALVSIVTSGSPFIDIPRKGGAF

VSGMLAWAFMVSRNKVDRSKMVRDDWDDVLNIRPIENIPFEALGYRIEFLEEWLKRVEKD

EYWDLMDWHLQKDKINVPALVVSGWYDDNSMGTTEALDVIKDYEKGKRKAILGPWMHNSN

TLRDINGISLGNSSLRYDLDYNYLLWFDKYLKGIENNIDTTAPVEYYSVGSNKWKTEENW

PIINKIDKSMYLISDGNANTSLGNGRLVFDNDLEEKYDSYIYNPKDPSVQLIDMSENEVG

VPNNYKDLEKRSDMLCYTSDAFSEEFTVTGDIKLEFFASSSAKDTDWVIKIMDVDLDGNS

IKLADGILSARFRNSFYKSEFMEEGEIYKFTVITSKISNTFKVGHKIRLDITSSAKNFIF

QNSNTTEGYNSIEYIEAKNTIYHGGKYPSKLILPIENK*

>CD630_13920 Clostridioides_difficile_630_NC_009089 endoribonuclease

MEIKRYEGTGRMSRAVVHNNTVYLCGQTHAEGGVIEQTTEVLAKIEDLLNKYGSDKQHLL

SVTIYLRDMKDFEAMNSVWDAWVEKGFEPARACVEARLAREHLLVEMSVVAATK*

>CD630_13970 Clostridioides_difficile_630_NC_009089 hypothetical protein

MKEILINPIGKICIQGEEVFIKLDKKYVPALNELDDFSHLNVFWWADGFDNPELRSILET

PKPYKSGPDVIGIFATRSPIRPNPIALTAVQIINIDHENGIIKIPYIDANDNSPVIDLKP

YTPSVDRVENPSVPKWCSHWPKSVEKSGEFNWEDEIEF*

>CD630_13980 Clostridioides_difficile_630_NC_009089 peptidase

MCFMLNIKTLCHEINDWVINIRRDLHKTPELGLEEFQTKKKIIKYLNEIGINYIEYKNHT

GITAYINVSPNFETVAIRADIDALPITEELNYSYKSINIGKMHACGHDAHTAILLGTCNI

LFKLKDYLNVNVKFFFQPAEETIGGAQLMIEDGCLENPNVKYIFGLHVNPNINKNLIELK

YNTLNASTDTLQLTVHGSKCHGAYPHQGIDAIVISAHIITALQTIVSRNTNPTDSVVISL

GEIEGGIKENIVCDKVVIRGTLRTLTPETREFSKKRIREICDFTCKTFGGSISVEIEEGY

PALINSNHLVDYVKQNAVELFGEENIILKDSPTLGAEDFSYFLRHCEGAFYHLGCANREK

NITSPLHTSTFDIDEDCLITGVILHVKNVLSF*

>CD630_13990 Clostridioides_difficile_630_NC_009089 muramoyltetrapeptide carboxypeptidase

MLGIIAPSGPLRNTSLEEIKFNLESYGYEVKFSESCSLNYKGYLAGNDDIRARDIEDMFL

DKDVDIIMCLRGGYGTTRILDKINYDIIKQNPKPFIGFSDITGLNLAFYKNCGLLPYHGI

MAANVGKWDKFTYKSLVNALEFKDELYLENPKEEKIYTVCEGKAEGIIMGGNLSLIIATM

GTKYEIDAKDKILFIEEIGEPQYKLDRMLTQLYSSGKLEECNGIIFGDFKDCIEENDLME

LLIEFANKVNKPSIYNLQSGHCIPMITIPLGRMCELDATEKIVKLKK*

>CD630_14000 Clostridioides_difficile_630_NC_009089 hypothetical protein

MSDDRIIDFNELKNKVKDSDVDKFEQYIYNLYFSVMDGKMSMAEFSRKIFDYMRDNNISQ

EKFMKIQKQFMERYGMDTEEVEKQLRNFGIDPSTAGFMSNNTSSKVSTEDLESFKKSAGF

YEKYGEKIQPKSCITTFIKNDLNDINVIIDQEKIMLCSDRKINLMDSELNEFLLEYKNMF

NKKIKVVMCETTNKYDY*

>CD630_14010 Clostridioides_difficile_630_NC_009089 isochorismatase

MDNLLNELEALKSNLDNLPIEKIENYDLSKTALFIIDVNNGFARQGALYSPRVESLIKPI

EMFTKKISNKLNKVIAFTDSHTPKSIELLSYPVHCLENDVESELVDELKSIENLQILPKN

STNGFFALENLDFDNIDNIIIVGDCTDICIYQFAITLKSYFNQHNIEKNIVVPMNLVDTY

DIPNVHPAEILNLVFFNSMIQNGVNVLKEIR*

>CD630_14020 Clostridioides_difficile_630_NC_009089 glycerophosphoryl diester phosphodiesterase

MNIYAHRGFSGKYPENTILAFKKCLDMDIYGIELDVHRTKDGKIVVIHDEKVDRTFNGHG

FVKDFTLRKLKTLNSSFEGYQSNKECKIPTLEEVLILISPTDLILNIELKTDKINYPNIE

KDVLELILKYNMKNRVLISSFNSNSLKNFHKLDPSVKTGLLCYLPINNVVNFAKFLGNSY

LHPPLVLVNESLIELCHKNLLGVNVYTVNEEDDILHCLKLNVDGIFTNYPDIASNLLHSK

QYS*

>CDM120_RS07340 Clostridioides_difficile_M120_NC_017174 peptide transporter

MNKKLPKGAYGEVSGKDYVPYITDKSRTGGNVAVLIIGIILAAIFAASTTYSGMKAGLTV

AAGIPGAIIGSAFVGAFARSKGILGKNLIQGMSSGGESVASGFIFVLPAVILIGSQITFL

EGLAVGVGGVLFGIGVAAIVHNYLIVEEHGKLMYPESMAISETLVASEAGGDSIKYMGIG

FVISGFITVLTGSFLNVANNVMSLVGSKFYKWKFDIEVNPLLLGIGFIVGLEVSLTMFAG

SILSNFGIAPLIGYFTDMAKDGAMVWNNPAMPLNQMDVGAISSSYVKYIGAGMMLCGGII

GAIKLIPTIIASIKETLKAKSSTGEGEEGSSIQMILLLGGVVIGFLAAFLISGGNIVMAI

IGAIISLLLSLLFVIVAGRLTGTIGTSNLPVSGMTIASLVIVTLVFVIMGWTDLEANKSL

LLFGSFIVVAIAIAGGYTQSQKVTYIIGGSKNEMQRYFTIASIVGVIVVVGVILLLSDQL

RATGDNVQFALPQANLMSTLTSGIMSGSLPWVMIIVGVFMAIVLYALNLPIMTIAIGFYL

PIATTSIILVGALIRLFVELVSKTEKEKEVKVSNGISLSSGLVAGGSIIGLIGIILQVTG

VVTPKVPSGFAATNSMAIALLVVLVVLTALPIILSKVKNNEQE*

>CD630_14080 Clostridioides_difficile_630_NC_009089 D-alanine--D-alanine ligase

MKIAVIMGGISSEREVSLNSGKEIYNNLDKNKYEVVKVIIDDKKDIFTKIPEDIDFAILA

LHGKFGEDGCIQSILETMDIPYSGCGPLCSGMCMDKNITKKMLRDSNLPTAPWVLVKSVD

EIDYDEIDNIGYPVFIKPNSGGSSVATFFIHSKDEVEEAVRKGLEVDEFVMIEKYIPGGE

YTSFILNGEVFPTISIKSDSGFFDYEAKYSVEKGAKEEVVYLDEELQKRVNEISETCWKI

FNCKAYVRVDMIISEGIPYVLELNTLPGMTQTSLIPRSAAARGIKYSELLDKLIEYSLN*

>CD630_14090 Clostridioides_difficile_630_NC_009089 competence damage-inducible protein A

LKAEIISVGTEILLGDIVNTNSQFLAKELASLGIEVYHQSTVGDNKQRLLECFDESLKRS

DFVITTGGLGPTGDDMTKETAAEYFGQKLELHKPSLEVLESFFVKTGKKMAENNMKQVYF

PKDAIVLKNNNGTAPGAILKKDGKFIIVLPGPPREMKAMFNESVKPYLQQFTNEMLVSKT

LRLYGIGESNLELEILDIINEQTNPTVALYAKELEVTIRITAKAENEREAFKLIKPVEEK

IKSRVGKYVYTEGDISISEGETALEDAVSKLLVEKNLTIAVAESCTGGLVSSSLINYPGI

SSVFLEGCVTYSNDSKMKRLGVKRETLEEFGAVSEQTAIEMAEGVAKGLKANIGISTTGV

AGPGGGTKEKPVGLVYTAIYINGKTIVKKNIFNGDRRKIRLRATRDLLNELRIQLEKL*

>CD630_14100 Clostridioides_difficile_630_NC_009089 HAD superfamily hydrolase

MIKHIFCDLDGTLYENGTITKEDIVAIEEIEKKGVQFNVATGRIFKQAHNIIKDSLDMNG

YYVCENGSFIYDKDYNVIFKRTIDDNLVKKVIDRFESSDAQLYFKYKGDVIVGSDTTAFR

HYSSDFIVDPDFEKRSSFDNLIGNIGVCSENLEELSRIELYLKSEFSEVLEIYFSGTYTL

NIVPKSVSKRGSIEHVIKTLNVSPDEVATIGDSPNDICMLEGFKYSFAMSKAREDVKQSA

NYVVDSVKSAIDVIMEINS*

>CD630_14130 Clostridioides_difficile_630_NC_009089 membrane protein

MGEKSKGYVFIAIAGLLWATLGLFGKFLMGNGLTSEQVAFTRLFFGFIVLGVYSSIRTPQ

ILKINKKGIIYSVIIGIICQAMFNLCYFKAIDIAGVSIAAVLLYTSPLFLAIFSKICYKE

NITRSKLFSLILCFIGAIMAVTGGRLDFQGLNAFGLLLGVLSAIAYALMPTISKNALKEF

SSSTILVYSFLFGAIFMIPSSRPWEILNYAKDLDVLSCMLMLGIVPAALAYIFYAAGISK

GVELSVAGVVASVELVGSVIIGCTILGESFSLGKLFGVMLMLISAVVALNLSYDEIRIFY

KSNKLKQIEKTESI*

>CDIF1296T_01481 Clostridioides_difficile_ATCC_9689__DSM_1296_strain_DSM1296_CP011968 radical SAM protein

MFFVQAQRTILLEIGDFLDKFKYAFDNKRYHTWNYYLRNTFGEKVFKVSINAGFTCPNID

GSLGYGGCTYCSKEGSGDFAGNPKDNLISQFYNIKEMMLKKWPHAKYIGYFQAYTNTYAP

LEVLKEKYETILELEDVIGLSISTRPDCLPDDVVEYLSELNKRTNLWVELGLQTIHDSTS

KIINRGHDYKTFVEGVEKLKSKNIKVVVHIINGLPGEDYNMMVETAKAVGKMGVDGIKIH

LLHVIKDTPMEKMLQNGMLTLMEQDEYINLVCDQLEILPETMIIHRLTGDGKRDELVGPI

WSLKKWEILNQIDDTLKARNSYQGCKFV*

>PCZ31_RS14295 Peptoclostridium_difficile_strain_Z31_NZ_CP013196 membrane protein

MNFCYNCSIDFGGDFMYTNFNIDFNNFNKKENAIKFMLMGVLLIILGLLCLTFKTLGIKL

ISWTFGIALLFFAYLNLKNINELKRYATKEEIKPSINIQWVLIIACILLFVFPQKIQSIF

SLFLGFYLIFNQLVALVNSKNNPYSKFTTWNIVKILFGICLILSPLFLSRFIVSIMSFFI

ILFGLVLFFSGNTARKY*

>CD630_14180 Clostridioides_difficile_630_NC_009089 MATE family drug/sodium antiporter

MDTKISINENMSLGKRFFKYLAPSVVAMWVFSLYTMVDGIFVSKGVGELALAAVNISMPF

INFIFAVSLLFSTGASTIIAIYLGKKDIKSANEVFSFNLVSIIILSIIILAITFFNLDRL

ALFLGATESTIGMVKDYLGIIIFFNGFFIVSYSLEVIIKTDGFPILATVGVIISALTNII

LDYLFVIEFGWGVKGAGIATGLSQVFSTIFFLIHFLRKNSTLNFSKFRIDFKTLRKIVFI

GFPDSTTELSCGIVVLLFNLSLTKYIGENALIYYSVINYINTLVLMTMMGITQGMQPLTS

FYYGAGNIDNVKKLLKMGIKATIIASVAVFAICMAFSGPIVSLFIHPEETMLFNEGVRVF

KIFSISFLLVGINVIISGFFVSVEKPSISTVISLGRGLVIVVLSLISMILIFGGQGIWMT

TIVSEFICLILSLVFLKKNFSTLDSNLNKVA*

>CD630_14200 Clostridioides_difficile_630_NC_009089 diguanylate kinase signaling protein

MFKEIFLRTFPGFLIIRDSNYRIIFINDNLKNLIKSYMQDNPLGMTNIEIAKKLPDNIAK

FFTDSHNIQLDWEKNYPYDKISNWILEFKKDTTSYWNVLEYKVDVDEKTYIITMANDITK

LYEENKRNLHYSITDPLTGAYNRKYLNDRFDIFIGDYIVLIDLDNFKMINDYEGHNVGDK

ILCDFVSLLNKELINSTSIIRLGGDEFIVIFSSDVDKNYVYSQLEALRENFLKVFSKYKY

LSFSYGVDTVKRNLKLTIVELDKKMYKNKEKNKKKFDKNDY*

>CD630_14220 Clostridioides_difficile_630_NC_009089 MerR family transcriptional regulator

MKDYYKIGEISKIYGIGRDSLMYYEEIGILRPVRDINGYRMYNISDIWKLNLIKEFRSLN

FPMKKIKEYLDDRSIESTKNILNEELDLIDKKIAEFISHKENIIKRLSSIESVIQNTKID

EIEVVYIEKRKALELNADIKRDEDFDFLIQKLQKEYEDRFNILGNNNIGSAFSTEAINKG

IFNEFKSVFCFLESNEKVYNIVFDEGYYVTLNYTGSNSNNKMYMEKVFKFIEENNYKIIA

DPIEIYKIDIHETGIVEEFVTEIQVPITK*

>CD630_14281 Clostridioides_difficile_630_NC_009089 hypothetical protein

MNISRKAMKIIELAQKIANKRGISVEEAWSEAVTEYKNKYEHIA*

>CD630_14290 Clostridioides_difficile_630_NC_009089 acyl-CoA N-acyltransferase

LIVKIDNTLEKEFWQYVSHEESLNLFIIGYVENYGFSSQYQDIWSQVEDGNITSIILKNK

STLIIYSFKNNFNIGEMKNHIKDLDVESISGKKCVIDRLISKYKDFYEKLDNKFCVLKEI

KEIDFSNMKEYKIENAQEKDIDEIGKLLNRSDYKVSKNYIEERKVHLKEGNVRAYFIRND

DTMISTVSTGMETSFLAMVVSVSTDKRYRGKGLASYMVYNLSKELLLEGKVPCLFYNNDV

AGKIYHNIGYKEINEWTILFK*

>CD630_14300 Clostridioides_difficile_630_NC_009089 delta-lactam-biosynthetic de-N-acteylase

VKKDSLKKYIMIGAFALILFGIASINFKSLDKTKTQISSPTLDTHEYDWYFNPREDGKQP

SPIKEADFFKKYGAYYVGNPNEKVIYLSFDAGYESGNTPKLLDTLKKHNAKAQFFVVESY

IKSNPELIKRMEKEGHLVCNHSKSHPSMAGITDFEKFKEEITSVEKAYKDVTGKEMPKYF

RPPMGKFSEQSLKYTQDLGYKSIFWSFAYVDWYEKKQPTHEFAKNKIYSRTHPGAIVLLH

PNSSTNTEILDEVLTHWEKEGYKLKTLDYLNNKK*

>CD630_14310 Clostridioides_difficile_630_NC_009089 DinG family helicase

MDNIISVLNDVVFLDIEVSGLDCLNSEILEVGAVKVKDWKIYTYESLIKNKFEVPVEVFS

VCKNLDKNDLEIANEIELVEDRLVNFVEDSFIICHDLSLKKKFFEYHMPKLKNKFIDLIE

LAVILEPYHKDYSLEYLKNTLTNCNSKVENRALSDAIDIINIVNCLLVKFNNYEKTTLEP

LSFKINSYLKKFNLPTWEWSKFLEEANYDLSNNINIKKEYNIFDSKEEKKKERETLKILN

EEEKNYEELLKYKTIWENKEGFTYEYRPGQYELTKTIRELFRNSEDEEKIACIEAPTGIG

KSVGYLLPAILEARINKKRLLISTDTKELQIQLINKDIPNVLDSLGLNGKVSYGYIKGKN

NYICIDRLEAYIDDYESQNPTKGEILSLIFLKRLVEGGKYGDIEEINYLVFDNFKEISTH

LRNVSCDPNMCRPKKCKKDCLYKNRIEELKEEHITVVNHSLLAKWPYKDEKPLENIIVDE

AHNLTEKGYDFFSSIINSKSLRYLLQEIYPYEFIQNSSFIYKKYSRNMRKIKAFDKFYNV

LKIGREDKQKIARSINLIIEEIDSILNFGNCNEYNNVSNYNLRWELNLQIDEIVGKLKKD

GIDTEISYRAYSEKIKLSCEKIIKNLVSIIIIIYRNIDDDSIDKEADIYKFGKAKTRDLE

DIKIIFEIFLEYDEKDDYARIVEIDKNYNVFEFRVVPLKIADLFEENILSQLEKGIFLSA

TLSLSESMSYFKNTLGIDRVKNVEKIIEPIFDYKNRVSVVGFSDICEYRNSEFPNEMSKI

ISNISKITEGHTLALFNSKDRQEKTYEILKKYLHSFNLEIYADKKGIRHLNDLNRKCVVL

GSKGCFEGVDIPGDGLVCVTLDKLPNLNPKDPLYFTIMKKYGIDYYTINYPQMTIKVKQA

MGRILRSKYDYGCFVIFDVGTNISVLKRLEKDLHDCKISKVNSNEFYTYIRRHLNKSRSL

ILKSVIFDTIKALNVDAKMDNNDVDKDIIKKDINENIRQRAVKGEVYHIDIIKKDMKVKY

FDRNYLINLDIFMREEDKN*

>CD630_14320 Clostridioides_difficile_630_NC_009089 ribonuclease

LAKQKFYAVKKGKNIGVYNTWDECKKQVNGFSGAEYKSFSTFQEAKEYIDGSEKLSFQED

KEFIEAYVDGSYEHSVKMYGSGVVILKNNEVIKTYSEKGKEKTLVSMRNVAGEIEASKIA

MQYCIDNNVQNLILYFDYEGIEKWCTGVWKTNKEGTIAYKNFYDSIKNKLNVKFTKVKAH

SGNKYNEEADKLAKKAIGV*

>CD630_14330 Clostridioides_difficile_630_NC_009089 bifunctional peroxiredoxin/chitinase

VIYMPNLPSLGSKAPDFKANTTNGPIRLSDYKGNWIVLFSHPGDFTPVCTTEFLCFAKYY

DEFKKRNTELIGLSVDSNSSHLAWMYNISLLTGVEIPFPIIEDRDMRIAKLYGMISKPMS

DTSTVRSVFIIDNNQILRTILYYPLTTGRNIPEILRIVDALQTSDRDNIVTPANWFPGMP

VILPYPKNYKELKNRVNSCNKKYSCMDWYLCFVPDNYNDEEVSKKIDNTCSWKKEHTKNI

ENECNCEHEHHDYLNKALDCKQEHKTDIKDDCNHEKKHTKNTNKVHNSKQDKFKDKSCDE

MNFNYDKDESCDKINSSYNKEDSSYEDFYKHNYKNYDYTSEKNTKKIAMKTLKDSKKLVR

PQITDPYNPIVENANCPDINPIVAEYVLGNPTNVDAQLLDAVIFAFAEIDQSGNLFIPYP

RFLNQLLALKGEKPSLKVIVAIGGWGAEGFSDAALTPTSRYNFARQVNQMINEYALDGID

IDWEYPGSSASGITSRPQDRENFTLLLTAIRDVIGDDKWLSVAGTGDRGYINSSAEIDKI

APIIDYFNLMSYDFTAGETGPNGRKHQANLFDSDLSLPGYSVDAMVRNLENAGMPSEKIL

LGIPFYGRLGATITRTYDELRRDYINKNGYEYRFDNTAQVPYLVKDGDFAMSYDDALSIF

LKTQYVLRNCLGGVFSWTSTYDQANILARTMSIGINDPEVLKEELEGIYGQF*

>CD630_14340 Clostridioides_difficile_630_NC_009089 hypothetical protein

VSGLKKILRILVISMMICFAMTFALVLINSVMGVHIGLDYYFLMGSVLTVCLTIGIVLCN

ARKIFDSKAIHKTRTKTVSNNKKQKSHSRDIKRKIS*

>CD630_14350 Clostridioides_difficile_630_NC_009089 hypothetical protein

MNFRDLFEKAVDFIDEKRKSIVAISLSTLGVIALIIVFFLSSNEFSVGNEANELLKIIEK

RQYSIAVDYYTSIEKKFSDSKMERFNKSVSKKINKLLLNSGDKYLDGDISQESFIGLINT

VKELNKISIDVDDLLAQADRVREMYKEENTTYDIAINYISTVSILNGMGSNLDVYKQNIE

TIKESRDVYDSAVKDQKAYKYYEAIEKYNKVLKEDEKYYSMAQNGKEQCIEEMYDYYISR

AEEANNSGDYERALQYIEYLKDDYSDDEKVQSLEKKYKKNLSLYTLTSDDIINVIAKKSG

KDKANLSINSLPQMIKNNKYYYAEVYEYDKLINEVLVSAKTKDLYSYKDGKKDYKVDYGD

GYFRILEDGSYQFGITKDKAKFVLTNTLDEKENKYKKIEILDIEKADRYVKSKKSLEELF

GKYKNIYYYAVVNKGLFRGKEVYAINIYNEKIYAISENGLNEY*

>CD630_14400 Clostridioides_difficile_630_NC_009089 LrgB family transporter

MYNVLQTPVFGIIVTIVFFNLGRYIQRKTSNPICNPLLIAIVGIILFLSISKIPYENYKI

GADSLNFFLGPVTVVLAVPLYKQFELFKKHMFEIIVGIGCGIVISFVSVLIIGKIANSDV

SIINSLIPKSITTPMGISLANSLNGIESITVVAIIFTGIFGGMVASTVFKLGNINHPVAK

GIALGTSAHALGTTKAFELGEIEGAMSGVSIGVSGTITVILIPIIMNFI*

>CD630_14410 Clostridioides_difficile_630_NC_009089 membrane protein

MSEVKNTLQKHPVLRYSTMLFGMLMTSIGINGFLRPAHLLSGGATGIATSINYLTNINVG

LLTFLINIPIFILGFIYLEKEFCISSLVNMIVFSLLLGATQEISNIIPIHDILLQSVYGG

ILSGLGVGVVFRTRSSQGGTDIIAAILKIKKNIEMKDTALAINGLIVLTGSFLFGLDLAL

YTLIGLFLNAYSMSFIKDAMNYQKSVMVMSNEVDLIAEDIMKSLVRGVTFLDAEGAYTHQ

KKKIIYTIVSSNEIPKIKDIALKYDKKAFISVNDVTEVKGRGFKAKDL*

>CD630_14450 Clostridioides_difficile_630_NC_009089 para-aminobenzoate/anthranilate synthase glutamine amidotransferase component II

MILMIDNYDSFVYNLVQYIEELGETVVVKRNNEIKISDIEELNPEVIVLSPGPCSPKEAG

ICIDIVEHFKGKKPILGICLGHQTIGHVFGGDIIKAQQPVHGKVYSINHTNKGVFRGLKN

PLNVTRYHSLIIDSNTVPKELEITAITDKGEIMGIRHKKYLIEGVQFHPEAILSEYGHEM

LKNFITEARERVHV*

>CD630_14460 Clostridioides_difficile_630_NC_009089 Para-aminobenzoate synthase component I

MCNMIREINTKLNSFEIFTIFRNEHDSFILDSAMDKEKLGRYSFISSQPFKVLKYKDTDE

NPLEVLKEELHKYRVVNDTNLPFVGGAVGYLSYDLGNYIENLPRTAVDDIEMPDMYFGFY

NHVIVIDHLVQKTYIATPNIDIELEEKIIDNIEQRILKEEKKGIDSICYEEKEVTPIRLK

SNFTKEEFKNAVQSVREYIRQGDIYQANLTQRFSGETELTSFELYRDLRRFSPAPFGAFL

NFEDAHILSNSPERFIRCVNKRIETRPIKGTRPRGKDKKEDLRLQQELRNSEKDRAELLM

IVDLERNDIGRISKTGSVKVPELFVIEPYANVNHLVSTVVGELKDDKDATDVIKATFPGG

SITGAPKIRAMEIIDELEPTQRNVYTGSIGYIGFNGDMDFNIAIRTIIKNDKKVYFQVGG

GMTWDSDPDEEYQETLDKAKSIMKALRGYYEE*

>CD630_14490 Clostridioides_difficile_630_NC_009089 GTP cyclohydrolase I

MNKVDKEKIQHAVREILEAIGEDPDREGLIETPNRVARMYEEIFSGLSEEPRDHLKVLFA

DEKHEELVLVKDIPFYSCCEHHLVPFFGKAHIAYLPKGGRLTGLSKLARVIDTLAKRPQL

QERITKNAADIIMEELQPYGVLVVVEAEHMCMTMRGVKKPGSKTVTSAVRGIFEKDIASR

AEAMSLITMK*

>CD630_14510 Clostridioides_difficile_630_NC_009089 dihydroneopterin aldolase

MDKILLSNLGFYGYHGVLKEENFLGQKFFVDMELYIDSREAGLSDDINKSVSYAEVYNVV

KDITENKQFNLLEALAENIAEEVLNKFILINGVMVRVRKPEAPVNGIYDYFGVEIRRARD

E*

>CD630_14520 Clostridioides_difficile_630_NC_009089 2-amino-4-hydroxy-6- hydroxymethyldihydropteridine pyrophosphokinase

MNKAYLGIGTNMGDRFDNLSRACELLKNSDSIYKVKESSLYETKPWGYTEQADFLNMCVE

IETEFEPYELLEYCQEIERELHRERIVHWGPRTIDVDVLFFNDVVSTDERLTIPHPRIQD

RAFVLIPLMDLNEELIINEKTIKEHLNLLSAEEREEVKELVGYERKPI*

>CD630_14530 Clostridioides_difficile_630_NC_009089 phospho-2-dehydro-3-deoxyheptonate aldolase

MKENLFNNNSKIEINTNNKKITVKKDKLIIAGPCAIESYEQLLETAKFVKSQGANILRGG

AYKPRTSPNSFQGLKKEGLEILKAVKDEVGMAVITELMDVRDMDELYSISDIIQIGSRNM

QNFTLLSEVGKQNKPVMLKRGIASTITEWIGASEYIAIEGNSNIIMCERGIRTYNDYTRN

TLDLAAVPIIQKETGLPVVVDPSHATGVRYLVKPMSLASFACGADGIMVEVHPDPENALS

DGAQSLCFNEFEDLMKSINNY*

>CD630_14550 Clostridioides_difficile_630_NC_009089 RNA polymerase sigma factor SigA

LSVENKSNKKELKKVTAKTLIEKGKKQGSLTLAEIMEAFSETELDKDQVENLYETLGNLG

IEITETKNYKADIDFSVADDDLSIGHLDEDAEAISHDDSSAIEIETVDLSLPKGISIDDP

VRMYLKEIGKIPLLKPHEEVEFARRMHEGDEIAKQRLVEANLRLVVSIAKRYVGRGMLFL

DLIQEGNLGLIKAVEKFDYTKGYKFSTYATWWIRQAITRAIADQARTIRIPVHMVETINK

LIRVSRQLLQELGRDPKPEEIAKEMEMTEDKVREIMKIAQDPVSLETPIGEEEDSHLGDF

IPDDDAPAPAEAAAYSLLKEQIEDVLGSLNDREQKVLKLRFGLEDGRARTLEEVGKEFDV

TRERIRQIEAKALRKLRHPSRSKKLRDYLD*

>CD630_14560 Clostridioides_difficile_630_NC_009089 SAM-dependent methyltransferase

LKLTDRLLKIASLVSDGKKIADIGTDHGYIPVYLLKEGKVPFAVLADVNKGPLDNAHKEV

IQNNLLDKVDLRLGSGIEILEIGEVEEVIIAGMGGILISELLEAKKEVAHNVEKLILQPM

QAQEELRYYLLNNGYEILEEVLVREDFRIYEIIVAKYTGKNTIIEDEIYYEVGIKLLENK

DSLFNDFIEKKIKTYSSIVNKLEGKNGEAIDKKRKESEVAIKKLENLIK*

>CD630_14570 Clostridioides_difficile_630_NC_009089 hypothetical protein

MLLKSLTRKIEKKYPLNLAEDWDNVGLIVGDFDMDVKKVLVSLEANEDVIDEAISKNIDL

IVTHHPFIFGKINKINSGDLKGRLIQKLIKNDISLYSMHTNFDIAFDGLNDYFMEIMEFG

NSKVLDITKSENLYKLAVYVPHNYSDELRKVLSNSGAGHIGNYSDCTFSIEGEGQFKPLE

GSNPFLGSVNDIETVNEVKIETVVPQKLLGGVISSMLDAHPYEEVAYDLYKLENKGEIFG

LGRISKLDKSMTLESLSRKIKEKLNMKHIRVVGNLSTDITKVAVVTGAGSEFVKKAKRQG

AEVLITGDVKYHEAQDALDIGMCIVDCGHFDTEDIFKNVMKRFLDEFSEIEVIKSNVYLN

PFSTI*

>CD630_14580 Clostridioides_difficile_630_NC_009089 flavodoxin protein

MMKVAVIFHSVCGSTYLLAREYKEALEEMNIEVGIFRVSDEVAKTLPQYYLINSKEYKDE

FESINVIKSGKEILDYDAIFMGSPTYYGNVSGPMKMFMDSFSDIWVGAPLSGKIFGCFAT

AGSQHGGGELALQAMNIFAQHMGMTLLSVPCSVRGGYPAYGILHIAGDNSDIRPNDDDKI

GIRDYLKRLNI*

>PCZ31_RS14520 Peptoclostridium_difficile_strain_Z31_NZ_CP013196 MFS transporter

IRKDDIMFKEMRLKKREMTKEDTVEVLKNGEFGTFSTISENGYPYGVAVNYVYFNDSIYF

HCARNGHKLDNISKNNKVSFLVVANESVIPDKFSTTYSSAIVFGKACTVENEEKKNALVE

IIKKYSKGFFEEGMKYIEKDMNLTTVVKIEIDHISGKASRL*

>PCZ31_RS14525 Peptoclostridium_difficile_strain_Z31_NZ_CP013196 phosphohydrolase

MEIFIMLSKAEIKYFNECASEILSSEKVQLMRTFPHHGNVSCLEHSLSVAYYSYLLCKKL

HLSVDIQSVIRGALLHDFFLYDWHYKGDRKGLHGFTHPREALKNATLFFQINEKETDIIL

KHMWPLTVKPPRYKEAFIVCLLDKFCCLVETLKIHSLLSPYHV*

>CD630_14640 Clostridioides_difficile_630_NC_009089 two-component response regulator

MLKLLIVEDDSTISFGIKYALEQEGFSIDISKDLSSGKEMISSNEYSMILLDVTLPDGTG

YELCQYIRGFSQVPIIFLTACDEEVNIVMGLDIGGDDYITKPFRIRELISRIKAVLRRKG

NTSEENKKILKFGDLSIYTLEARVYKNDKEIFLTSVEYKLLLILIQNKNTVLSRTQILEK

LWDVTYDFVNDNTLTVYIKRLREKIGDDLCEPIYIETVRGIGYKWIGSENSVSI*

>CD630_14650 Clostridioides_difficile_630_NC_009089 two-component sensor histidine kinase

VKIVFLYNPEVKKFLSKYVTLMFIVIIISIGFSIINVSLTKEMVVRNNQAIIGTLSSKYP

NLESQIVDIITQGKSMENIDYGQKILIKYNYDKSIRINSEPIISKLVLETIKINIILVCI

IFILIFVLIVRYFTSIYNDLSDMTKYVYYSSEGKPFDMKNKNQEGQIGLLKTELLKMTTI

LNEKVELLKREKIFLNNTISDISHQLKTPMTSLIMLNDLLYNDVPYEVKIDFLNKIKNQL

NRMDWLIKSMLKLSKVEAKVINFKKDKVKFSELIHRAMLSMKIPMEIKNQKLTIEGNDNV

SYIGDIDWSVEALVNIIKNCVEHTPEFGNIIITYKENPLFSELIIKDEGEGIDKKDIPHV

FKRFYRGRSSSKEDSVGIGLAMSKSIIESQNGDIYVNSEKGKGTEFHIIFHKMYDSD*

>CD630_14680 Clostridioides_difficile_630_NC_009089 iron-sulfur protein

VITISHITAKNMYKSLEERINKFPQGAPPSDTLYKILNVLYTEQEAKLVAQLPIKPFRVK

TAAKIWSVSESEAYRVLDKLASKALILDIEDNKGKKYIMPPPMAGFFEFAMMRTRHDIDQ

KLLAELYYQYMNVEEDFIKDLFYSTETKLGRVYVQEEVLTNDNEVSILDYERATHIIDES

THIGISMCYCRHRMQHVGKACDAPMDICMTFDNVANSLINNKFARRVDKIECKELLHQAY

EHNLVQCGENVRKGVTFICNCCGCCCEAMVAAKRFGNLHPVQTTSFIPNINHENCVKCGK

CITACPIDAISKVKEDGKEYIKIDEDRCLGCGVCVRNCHKNSIMLLKRDEKIITPANSVH

RAVLMAIEKGQLQNLIFDNNALASHRAMGAILSAILKLEPAKKILASKQLKSVYLDKLLS

MNDK*

>CDIF1296T_01539 Clostridioides_difficile_ATCC_9689__DSM_1296_strain_DSM1296_CP011968 penicillin-binding protein

MFKQRLSKLLSSTLVLSMLFTAAPNITFADNTKDNSEKYQSSDIELHDYSKNAESYTKTK

ALAKEKIQTLLSKYGAVSAQYALIDNGKIEISGNGGVYSKQDNKNLNKDNMYSIASISKM

FTTTAVMKLVDDGKLNLDTPVVKYIPEFKMADDRYKEITPRMLLNHSSGLMGSSFKNTIL

LADNDSYGHDNFLKELQKQRLKAKPGAFSVYCNDGFTLAEILVERVSGMSFTNFLDKYIN

NPLNLQNTKTTENSFDSSKLAKAYVPYWEDAVPQDNLNAIGAGGLYSSAENLCTFAQTFM

KNSNGILSPASVKAMENKEYLNGLWPEGEDSILGYGLGWDCVNTYPFNQYNLKALTKGGD

SLLFHSNLIVLPDENMAVAVLSSGGSSQLNEIIGQEILLSALKEKGKIKEIKPDKTFSKP

QQVKMPSSLKENSGLYASSNMIKVDVNDNGTLTVSSPYIENGPEDKYVYIGQDRFVSEKG

NSCLKFVKEKNNITYLNMSSYDDVPGLGQTASLYYVAQKVDDNNISNSVKEVWKKRSGKG

YYLVDEKYTSQSYMFGSVKASFSLSDETPGYIVNTKIMDENNSNAFIEIPGVIGRDLSDI

KLHKENGTEYLSFGTLTYVSEDSITNLPAEKSFTCELESNGYAKWYKIGDDIANKKIEVN

LPQNSAFAVYDDKGVPVNYSLVTKNNRVRLPKGGVIVFLGSPNARFEVTYQDEVNASALT

GTDRYETSIKISQAGWENAENAVLINDSAIADALAATPFAYKKNAPILLTGSSQINEKTL

AELKRLKVKNVYVVGGEASINEKSLDTIKSNNISVSRISGSDRYQTSMNIAKELNNISNI

SKISVVNGEKGLADAVSIGAVSAQNDMPIILTNENSNITEINNVFKNKKIDKSYVIGGEY

TVSKNIESKLQNPQRISGSTRNETNAKVIKEFYKDSKIDNLYVAKNGMNKQDDLIDGLSV

GVLAGKTKSPVMLVGNSLDYNQKELFKTMRFKSVTQIGGNGNENSFKQIKEIA*

>CD630_14700 Clostridioides_difficile_630_NC_009089 rhodanese-like domain-containing protein

MYKNINKKQLKEMMKNEKDVLLLDVRTKDEFKEYKIENAINISLQELINNIDEIYDYKDK

KVVVYCRSGHRSVTACNLLAEEGFEHLYNLNSGIIDYMS*

>CD630_14780 Clostridioides_difficile_630_NC_009089 ferrous iron transport protein FeoA

MNRLKDIKCGETVKVKKLEGEGATRRRIMDMGITRGVNIFIRKVAPLGDPIEVTVRDYEL

SIRKSDAEKIIVE*

>CD630_14790 Clostridioides_difficile_630_NC_009089 ferrous iron transport protein FeoB

MSIKIGLIGNPNCGKTTMFNGLTGSSQYVGNWPGVTVEKKGGKLKGNKDVEIVDLPGIYS

LSPYTLEEVVTRNFMLDDKPDAVINIVDASNIERNLYLTTQVLELGIPTVIALNMMDIVN

KNGDKINIKELSEVIGCPVVEVTAVKGQGIMEAAEKAVELASSNNKLNFKLPFVDESKDA

IEKIEKIIEEKTPYIDVETRWLAIKLFERDENVIQKLNISKTILNSIEEITRNCEDELDD

DSESIITANRYEFISSIISSIIKKNRKGKETVSDKIDKIVTNRILALPIFALIMWGVYYI

AVSSLGTIATDWTNDVLFGEIIQGNVSNFLASLNVAEWLQGLVVDGLIGGVGAVLGFVPQ

IMLLFLLLSILEDCGYMSRVAFIMDRIFRKFGLSGKSFIPMLISSGCGVPGVMSTRTIEN

DRDRKMTIMLTTFIPCGAKIPIIALFAGALFGGASWVAPSMYFLGIAMIIICGIILKKTS

LFAGEPSPFVMELPQYHIPSAKGVLIHMWDRGKAFIIKAGTIIFVACGVIWFLQSFNWSL

QMVDAGDSILASLGNIVAPIFAPLGFGNWQSSVATVTGLVAKENVVGTFGVLFGISDATE

QDPTLLASVASMFTVASAFAFMAFNMLCAPCFAAIGAIKREMGSWKWTWITLGFQTLTAY

IIALLINQVGSLVLGTGGSIAGAIISIFIAVAVVFVVLTYSNKNMKKEKMGKLSYMKN*

>CD630_14800 Clostridioides_difficile_630_NC_009089 hypothetical protein

MNMATFIIAAIVVVLMALAVMYMVKNSKKNGSSCGCGCSGCSSSKSCHSAKR*

>CD630_14830 Clostridioides_difficile_630_NC_009089 sulfonate family ABC transporter ATP-binding protein

MVRSGFLLENIYKKYLVDNKEHLVLDNISLNISSEEITVILGESGCGKTTLLRILAGLEN

ATSGNIYFFNNDKKCTPKVGMVFQESRLMPWLNVSENILLHTEKDNRNKVDLDKYLKMMK

LEKFKNSYPNELSGGMAHRVSIARALSFNPDILLMDEPFAALDYFTRRKMQKEVVNIHKN

TKKGVVFVTHNIEEAMEIAKKIIVFSKNKRIKQFSVEDEYNRDLTKNYYINLKKEILREL

GEF*

>CD630_14860 Clostridioides_difficile_630_NC_009089 hypothetical protein

MKLKRTLFVGLALVLAIGITACTKNNEKPSENTGKNPNESVNNNEGYMNYYSTSYNDYIA

GLNRYSIYDTPESINNQFKDKEYPGNEKYLSDVKAAYKDSRDKIQSFVTSLKKDGKTEDT

ELKKMNDDLIAEGERLVKDIDKRIEKLDKVSDEDMKKGQNDFIKLVHGTEDVGNDIGSGF

RKMIKDMNDRLGITGNNKK*

>AEC_RS02000000221015 Clostridioides_difficile_QCD_37x79_NZ_CM000658 YafY family transcriptional regulator

MSRAERLIELMITINAKRSFTAGELAEEFSVSKRTILRDLQVLESVGFPLYSKVGAAGGY

HVLKERILPPITFSESEVKSIFFAYQSLEYYNDLPFEQETISVLKKFLNCIPNDIQYNIE

NMRRKLVFWTPDRHCSTPLLKELFNIVMNEFTIKIEYSSKQKNSIRTIVPIGLYAMNGLW

YCPAYCIKSESVKEFRADRIVKILSIENLSNKKYKVLSSIHDYLKNMEVGTDYHIKINLT

DEGVKRCETEFLLARGLKILSKGGYIDMYIPKSTLNWVAEYFLTFGKNATIIEPIELKXX

XXXXXXXXXXXXXXXXXXXXXXXXXXXXXXXXXXXXXXXXXXXXXXXXXXXXXXXXXXXX

XXXXXXXXXXXXXXXXXXXXXXXXXXXXXXXXXXXXXXXXXXXXXXXXXXXXXXXXXXXX

XXXXXXXXXXXXXXXXXXXXXXXXXXXXXXXXXXXXXXXXXXXXXXXXXXXXXXXXXXXX

XXXXXXXXXXXXXXXXXXXXXXXXXXXXXXXXXXXXXXXXXXXXXXXXXXXXXXXXXXXX

XXXXXXXXXXXXXXXXXXXXXXXXXXXXXXXXXXXXXXXXXXXXXXXXXXXXXXXXXXXX

XXXXXXXXXXXXXXXXXXXXXXXXXXXXXXXXXXXXXXXXXXXXXXXXXXXXXXXXXXXX

XXXXXXXXXXXXXXXXXXXXXXXXXXXXXXXXXXXXXXXXXXXXXXXXXXXXXXXXXXXX

XXXXXXXXXXXXXXXXXXXXXXXXXXXXXXXXXXXXXXXXXXXXXXXXXXXXXXXXXXXX

XXXXXXXXXXXXXXXXXXXXXXXXXXXXXXXXXXXXXXXXXXXXXXXXXXXXXXXXXXXX

XXXXXXXXXXXXXXXXXXXXXXXXXXXXXXXXXXXXXXXXXXXXXXXXXXXXXXXXX

>CD630_14890 Clostridioides_difficile_630_NC_009089 methionine ABC transporter ATP-binding protein

MISIKNVNKYYGKIQVLKDVSIEIESGEIFGIIGHSGAGKSTLLRCINGLEEYQEGSVLV

SDKEVKSLNEKQMRDLRKELGMIFQHFSLLERKTVFDNVALPLECFGYSKAEIKKRVLEL

LEVVGISEKKNDKPRNLSGGQKQRVAIARALALNPQVLLCDEATSALDPNTTKSILSLLE

DINKKLGITIIVVTHQMEVIKQICGRVAIMENGEVLEVGDTEEIFLRNTKGLRKLIGEES

IILPKGTNIKILFPKDISNEAIITTMARELNIDVSIIFGKLEQFKDDILGSLIINISDKS

GEQVKQYLTSKGIRWEEMINE*

>CD630_14900 Clostridioides_difficile_630_NC_009089 methionine ABC transporter permease

MNSLIDFLTTLFPNALLQTLYMVIVPTIVATILGFILAIILVVTKPDGLKPNSTINSALG

FIVNIFRSFPFMILIVAMIPITRLIVGTSIGETAAIVPITIGAAPFIARIIESSLNEVDK

GLIEAAKSFGATKRQIVFKVMIKEAMPSIVSGITLSIISILGYTAMAGAVGAGGLGNIAL

IYGYQRFDTAVMVYTVIALIILVQIIQGVGNLAYKKLK*

>CD630_14910 Clostridioides_difficile_630_NC_009089 methionine ABC transporter substrate-binding lipoprotein

MKLKKLLSVALVSAIAISAVGCSNKEDKKILVGASSNPHAKILEVAKPLLKEKGYDLEVK

IFDDYVLPNTALDEGSLDANFFQHIPFLEETVKEKGYKLTYTSKVHIEPMGFYSEKVKSL

DELKDGAVIAVPNDATNGARALKLLAKNKLIEVKDGELITKKDITKNPKNIVIKEMNAEQ

LPTVLKDVDGAVINSNYALTANLNPTKDAIVIESSDSPYVNIIACRENNKDSDKIKALSE

AMNSKEVKKFIQDEYKGSIVPAF*

>CD630_14940 Clostridioides_difficile_630_NC_009089 HTH-type transcriptional regulator

LNKDIGAKIKQLRTQKQMTLKDMSEKTNLSIGFLSQLERGLTSVATDSLGKIASVLDVEL

TYFFMKPKEHKRAVLRSYEKEVFDVENSTFIHYHLSSSLKEKTMLPRLIEILPSKSSEEI

CCYVHEGEEFVYVLEGTLTVFLGDEQIEMYPGDTIHYNSEKNNHNWVNYTNKVVKILVVS

IPNPFEKSDAVKEA*

>CD630_14950 Clostridioides_difficile_630_NC_009089 pyrroline-5-carboxylate reductase

VKKIGFIGAGNMASAMIGGIVNSKLVEPNMVIASAYSQGTLDRIDANFGINTTKDSKEVT

RTSDIVIVAVKPDIYDDILEEIKDFIDDNKIIVTIAAGKSIKDIESIIGEDKKIVRTMPN

TPALVNEAMSSLSINKNINKEDLEAVTEVFNSFGNTEVVPEYLIEAVIGASGSAPAYVFL

FIEAIADAAVIAGMPRPQAYKFASQAVMGSAKMVLETGKHPGELKDMVCSPGGTTIEAVK

VLEEEGFRASVIKAICACIEKSKKMSE*

>CD630_14970 Clostridioides_difficile_630_NC_009089 hypothetical protein

MSKELVKDRVIKYLIEDLLVPQDMIDTNVELAEFEEGAEGILDIVVNVKDEEDYYAPVMI

VQCLDEDVELEGEVLQKQIEFLEDVDNITMSGRLVLTNGDAMMYADWRGEEYDTEAALPT

YDIMVKEFHEMEQQAKDLEEHHHHDENCGCGCNHHHEN*

>CD630_14990 Clostridioides_difficile_630_NC_009089 MurJ/MviN family protein

MSKVAKATFYLMIVTIISKILGMGRELVLSSIYGTGLYTESYLTAMNIPNIIFAAIGTAI

VTTFIPMYQDISSKQGEKQALKFLNNVLNIIVGICIVVAILGVIFSKQLVSIFAIGFEGE

RFLLTVKFTKILIKKLWRISKTYFSMQNSMKQEPCDFSHGRFRTGIIFIGITSVMSAFLQ

IKENFIVVGFGSIPYNIVIIISIMLSTIFGPYILPIGAVVAMVVQLLFYMFFVKKTNYKY

LYYLNFKDDSLIKLLALLSPVFIGVAVNQVNSLVDTTLASTLVKGSIPALTYADRLNGFV

TGTFTASIVSVMYPMLSKLSAENNQKKFTSSVKSSINMIIISMIPISVASIFFATPVVRI

IFERGAFDARATQMTATALIFYAVGMTAFGLRDILGKVFYSLQDTKTPMVNGIISVGVNI

VLDLVLIKPMAHGGLALATSSSSIACILLLFLNLKRKVGYFGQDKIIKATLKSVVASLIM

GVLSYFTYKFIFGILGVGTFNEFVSLAISVIVGGGIYTLLMTIFKVEEVDMILNIAKRKL

HLKK*

>CD630_15020 Clostridioides_difficile_630_NC_009089 2-deoxyribose-5-phosphate aldolase

MKHILKTVDHTILKATTTWEDIKILCDEAVDMSVASVCIPPSYVKRASEYLKGKIKICTV

IGFPLGYQTTATKVFEAKDAIENGADEVDMVVNISDIKNKDYDNIGKEIKEIKKAIGDKV

LKVIIETCYLDEDEKIKMCEIVTMSGSDFIKTSTGMGTGGATLEDIKLMKEHVGKNVKIK

AAGGVKSISDAEKFIEAGAERLGTSSICKILKNEDTTDY*

>CD630_15030 Clostridioides_difficile_630_NC_009089 GntR family transcriptional regulator

MKLVLNNEEPIFIQIARAIEDEILSNGIKEEEQVPSTTELSKLYKINPATVLKGINILVD

KNILYKKRGIGMFVSDGAKTIIKEARKENFKHNFVKNLLQEANKLEINREELVDIIINFK

ED*

>CD630_15110 Clostridioides_difficile_630_NC_009089 hypothetical protein

MIDNQKYVILSLELHLFFSRIMKEHALFLEAGFTNKNYNLAMEADHYKKQFEDLLSYTVS

ASNGIIRPDILYSEELVTTLTSVAEQKTEEFTGIEINKNITTRELNLQSGVNPQVGQDLV

NYVAQLNSDAIRLLDGLINFKERVLDGVLSCTIFTSNYPLLLEHIIHEANLYRSYVVDLE

NKIDIESKNAKEIELFWDHIMMEHALFMRGLLDPSEGELINTSNDFAIKFNELIEKTNEM

TDSNIKNITEETLNETVEFKDFKEAGASGIEQCKIKSIILPLLADHVLREANHYIRILES

YKNM*

>CD630_15120 Clostridioides_difficile_630_NC_009089 pantothenate synthetase

MLVKEIKLLRNIIKDWRKHGYSIGLVTTMGFLHEGHQSLIKKAVKENDKVVVSVFVNPTQ

FGPNEDFNSYPRDIDKDFKYCMDSGATVVFNPSPEEMYLKGNCTTINVSGLTDFLCGAKR

PVHFGGVCLVVSKFLNIVTPDKAYFGEKDAQQLAVIKRMVKDLNIDTEIIGCPIIRENDG

LAKSSRNTYLSEEERKSALILNKSLSLAKEELVKGNLNPENIKELITAKINSEHLAKIDY

VEIVDSETLQPVKQIEHSILVAIAVFIGKTRLIDNFTFELNI*

>CD630_15130 Clostridioides_difficile_630_NC_009089 3-methyl-2-oxobutanoate hydroxymethyltransferase

MKNTIQTFKNAKYEGKKLSMLTAYDYSIAKIMDECDINGILIGDSLGMVIKGEENTLSVT

IDEIIYHTKAVKNGVKNALIVSDMPFLSYHVSIEDAVKNAGRLIKEGGAHAVKLEGGSNV

IKQIESIVNAQIPVMGHLGLTPQSVNSFGGFKVQGNTSETARQLIEDAKLIEKAGAFSIV

LEGVPTKIAEMVTNSISIPTIGIGAGINCDGQILVYQDMLGMFGDFVPKFVKQYANIGDI

MKDSIKNYILEVNTGAFPQEKHSFSINESELEKLY*

>CD630_15160 Clostridioides_difficile_630_NC_009089 MerR family transcriptional regulator

LEKQYFTTGEFAKLCGISKQTLIFYDKIGIFSPEYKDKNNYRYYSVYQYDTLDILQSLRE

IGMSLEEIKEYIQNRTPQLCVKMLKEEEKKIREKIKKLRKISSKMQNRINITVEGISKMN

SEEIYISKKFEEYLVLSSDLSNMSNSDFMMELIDFTNYCKSKNLYQGYELGVIVSNENIK

NGDYLSISSFYLKIDKKIKDKKLYVKPEGMYACIKHIGKYEDSYKSYEKLKKYIYENEYK

IIGNSYEESILDFFCESNEDNYMTEISIQVSR*

>CD630_15170 Clostridioides_difficile_630_NC_009089 ferrous iron transport protein FeoB

MGLTHNSTKMSSLKDMFDIDNKEDQFVIALAGNPNTGKSTVFNHLTGLRQHTGNWPGKTV

ATARGNFKYKNTEYALIDLPGTYSLFALSQEEIVARDFICFGNPDAVIVVCDATCLERNL

NLVFQVMELTDKVILCINLIDEARKKGITIDKKLLEDSLGIPVILTAARNGSGMDELLDT

LNDVSFDKYKLNNKPVRYNENIENVVKSIQPELDNIIPGINSRWLGLRLIDGDESIFESM

SNYIDKDSIDAINEVKKKIPDNINKQKIRDEFTKINYDYAKKLSDECCSNVAKKSTDREE

KVDKILTSKIFGLPIMLLLLGTILWITIEGANYPSTLLSNLLLGFEPSISGILNSINCPS

WLNDMLVLGLYRTLAWVISVMLPPMAIFFPLFTLLEDFGYLPRVAFNLDHLFKKACAHGK

QCLTMCMGFGCNAAGVIGCRIIDSPRERLIAILTNNFVPCNGRFPTLIAISTIFFSSVIT

NSFVSSVATALCITLLIILGVIITLLVSYTLSKTLLKGVPSTFTLELPPYRVPQIGRTLY

TSIIDRTIFVLGRAVMVAIPAGVITWIFANIYIGDLSILSHVANFLDPLAKLIGLDGFIL

LAFILGFPANEIVVPILLMAYLATGSMIELDSFSALGQVLREHGWTYLTALNVMLFSLLH

WPCATTLLTIKKETGSLKWTALGFLMPTILAFVVCFLTTTVYNLFI*

>CD630_15180 Clostridioides_difficile_630_NC_009089 ferrous iron transport protein FeoA

MKNLNDIQVKKTVKVEDILSSGNLRERMLALGLTRGAVIDVVRKGPKNNLTVYKIRGSKI

ALRQEESSLILVSDI*

>CD630_15190 Clostridioides_difficile_630_NC_009089 acyltransferase

MFKIKHFNDLSLDEFYEIAKSRYEVFACEQKIFSLNDYDDIDKSSYHIFLKENGLICAYA

RIIPKEYSSYNDVSIGRVLVLSSHRRKGLAKQMMDCAIDFIKVNLHENNITLSAQTYIKN

LYLSCGFKEISEVYDEAGIEHIKMRL*

>CD630_15210 Clostridioides_difficile_630_NC_009089 tyrosine--tRNA ligase

MKSIDEQMRIIMKGVDDLIDEKELREKLIKSEKEGKPMIVKLGLDPSAPDIHLGHTVVLR

KMKQLQDLGHQIVIIIGDFTGKIGDPTGKSKARKALTTEQVLANAKTYEEQIFKVLDKEK

TIVRFNSEWLAKLNFEDVIKLAATITVARMLEREDFKKRYEGQMPISVHEFFYPLMQAYD

SIALEADIELGGTDQRFNLLMGRSLQREFGMESQIVIMMPLIEGLDGKEKMSKSLGNYIG

IDEEAGIMYQKSMEIPDELIIKYYNLVTDVHPDEVNKIESQLKEGSVNPRDIKMNLAREI

VTLYHGEESAKEAEERFKSVFQKGQIPEDIQTIQVKEDGFDLIEVLVSNEIVKSKSEVRR

LASQGGVKVNGEKVEDLSTIVKESELVVQIGKKKFVKIELVK*

>CD630_15230 Clostridioides_difficile_630_NC_009089 transporter

LKKYFGEIGLIFIAIIWGSGFVATQFALDGGLTPLQIITLRFFLAAIIMNLLFFKQIRAN

MGKKLLKAGGILGIFLFLAFTVQTIGLMYTTPSKNAFITAANVVIVPFIGFILYRRKLDK

IGIISSLVALIGIGILSLEADFSINFGDFLTLICSFGFAFHIFFTSEFAKDNNPMALTAI

QFTVAFLMSVVVQTFAGQLKMEAELSGYMGTMYLAVFSTTIGFLFQTICQKRVDGTRTAI

ILSTEAVFGTIFSIIILKELITAKLVIGSILIFVAIITAETKLSFLKSKKVKLKDSEESS

LESI*

>CD630_15240 Clostridioides_difficile_630_NC_009089 rubrerythrin

MKKFVCTVCGYIHEGDAAPAQCPVCKVGADKFEEMKGEMVWADEHRIGVAQGVDAEIIEG

LRANFTGECTEVGMYLAMSRQADREGYPEVAEAYKRIAFEEAEHAAKFAELLGEVVVADT

KENLRVRVDAEYGATDGKLKLAKRAKELGLDAIHDTVHEMCKDEARHGKAFLGLLNRHFG

K*

>CD630_15270 Clostridioides_difficile_630_NC_009089 ABC transporter permease

MSLLKNSLANLKGHKLRVFVALLWIIIGITSVILVSSIGNGFQKEIKKSVNNVNPNKTTI

SFESADNTGLTDDMSIFLKPFNAKDLEELSFVEGVERIAPSRDGFNLDSVYSSQASFDKK

TTYVDVGPVKKDSKINLICGRDFSLDDEKRKVILLTLQSTSEIFENPEDALGHGININGT

IFEIIGVLDDSQQNQAGGFFGGYQDMQFTTSLVPKKAFDTLMSQNSYSNEIYQLDLVSSK

GYNVNEVANNVIAKLYEMHPGINGSYTTPDPTEQTAYLESINSNVNKYVSIITVVAMFVG

GIGVMNIMYVSVMERQREIGIRRAIGAKPRSILFQFLVEAVFITVCGGILGTIVGFAATN

YVSKYIGFEAIPSLNSLFYAIVATILTGVVFGLIPAFKASKLDPIKAIYK*

>CD630_15280 Clostridioides_difficile_630_NC_009089 ABC transporter ATP-binding protein

MLIKLENIQKYYKVGKDELHVLKSLNLEIESGEFVMIMGKSGSGKTTLLNILGFLDVFDE

GRYIFDGTDVTNLSENERSVFRNINIGFVFQQFNLIETLNVYQNVELPLIYNKALKKSNR

EEIVKDKLSSVGLLDKLKQKPLQLSGGQQQRVAIARCLANDPQIIFADEPTGALDSETSR

EIMELLTRLNKQGKTIIMVTHDQDLTKYATKVIRLKDGVFTSEV*

>CDM120_RS08005 Clostridioides_difficile_M120_NC_017174 ABC transporter permease

MSFLKKFKNKRFRNNSFKSNNFRSSSFGSSGFNSKNKGFKNNPFKNKRFKIYAGIAVIIL

LILGVLAYVDSKNTKLQANENFIETYTIPENEKIFINGMVVPKQTKDFNISGDYELSDVN

VTNGQKVNQGDLLFTAKNPTIIAEIDSLKSQLSQYKKQKISLSDIAENRDAIASINAQIT

TLNSQIASLDKKAYDRVTAPFDGTVYLNDQTGNPDQPVSFMTIQGLEFYMKGQASEQDLP

KMKIDQMVNILVFSTDQKLTGRISFISDKPSTPNTEMGAQQNTLSYYDINIAFDNQEGLV

NGFHLQASLEVSNSSFKIPASCVLKDKKHSYVFKDLDGILKKQIVDVASQNDDFAVVRGG

LEQGDIIIKHPTKEMKEGDPVQGDGVTAGSNNNGDTTPNKKVEEVNMDVN*

>CD630_15310 Clostridioides_difficile_630_NC_009089 two-component response regulator

MFKIMVVEDDVSLKNIIAKCLTKWGHDVHQIENLENIIEEFKNYNPELVLLDINLPFYDG

FHWCNEIRKISKVPIIFISSRNSNMDVIMGVNLGADDYIQKPFSVDVLVAKVNALLRRTY

NFVDNNSNQIIHNGVTLDLSTATINYEDNTIELTKNEIKILHELMKYKGQIVSRNKLMKK

LWDNDWFVDDNTLTVNVNRIRSKLNEIGLEDFIETKRGLGYIIS*

>CD630_15320 Clostridioides_difficile_630_NC_009089 ABC transporter ATP-binding protein

MEEILSVENIKKEYGRKGSKHEALRGITFKVYKGEFVGIMGSSGAGKSTLLNIISTIDLP

SSGDIYINGKNTIKMKQNELADFRRDNLGFVFQDSNLLDTLTIKENIMLPLSLKNERVSV

IENRIKEISKELNIESILDKYPGEVSGGQKQRGAVCRAIATKPSLVLADEPTGALDSKSA

RDLLNCLLKLNKDSNKTILMVTHDAISASFCNRILFIKDGIIFTEIVKGESNREFYNKIV

NTVSLIGGVNKNDFI*

>CD630_15340 Clostridioides_difficile_630_NC_009089 gamma-glutamyltranspeptidase

MKFNAYQHKYSSIRNVVYAKNGAVATSTPLASQAGLEILKKGGNAVDAAVATAATLAVVE

PTSNGIGGDAYALVWIEEEKRLYGLNSSGFAPENMELKNYNNMKEMPKYGFGAVTVPGIP

AAWSELNKKYGKLSLMECLSPAINYAREGYVVSPNVAKVWKKSYELYEKELIGEEFKPWF

DTFSKDGKAPEAGDIFICEEQASTLEEIANTQAESFYRGRLADKIDEYSKKFNGAIRKSD

LECFYPTWVEPISTEYKGYKIFEIPPNGHGITVLMALNILKELELEGNIENVEDIHKIIE

SLKLAFADSKTYVTDIEHMKVKIQELLSQEYAKKRSLLIDNKEALYPTAGEPYCGGTVYL

CTADKDGNMVSYIQSNYINFGSGIVIPRTGIALHSRGNNFNLDPKHHNVVKPFKKPYHTI

IPGFLGKEDKAIGPFGVMGAFMQPQGHIQVLTNMIDFGLNPQEALDAPRWQWIKGKEIEV

EPEMPKHIIDSLIEKGHEIKVIHDTVDMGRGQIIFKTEQESYICGTESRCDGHIAVY*

>CD630_15350 Clostridioides_difficile_630_NC_009089 3-mercaptopyruvate sulfurtransferase

LKNIISAKELISKLEKNDNLVVIDCRFDLINRTYGIDSYKKGHIKGSFILDIDKDLSSPT

KEHGGKNPLQDPLILKEKLEKMGVDNDTTIVTYDDGDLNGACRLFFQLKHLGLKNVYVLD

GGITSFVKEGGELEEKVNIPSCTGKEIRPNINNDLVVPMEYVKSKLYKKDTVIIDCRANE

RYQGLVEPAYSKAGHIPSAKNYFCKDLIKSDFENGSLKDIEFLKKFFKDLNNYDEVILSC

GSGISACVNSLALRELDIPHKIYIGSFSDWISYDDNEIKTGQE*

>CD630_15360 Clostridioides_difficile_630_NC_009089 ferredoxin-NADP(+) reductase subunit alpha

MSNKIVSKRQLTDSIYLMEIEAPRVAKSSQPGQFIIIKNDEKGERIPLTIADYDREKGTV

TIVFQTVGASTKKLAMFEENDFVMDFVGPLGQASEFIHEDIEELRNKKILFVAGGVGSAP

VYPQVKWFKEHGLDVDVIIGARTKELIILEDDMKKVAKNVYVSTDDGTYGFNGRVTDLLK

DLVDNQGKKYDQAIVIGPMIMMKFMCQLTKELNIPTIVSLNTIMIDGTGMCGGCRVSVGN

ETKFACVDGPEFDGHLVDFDQAMRRQSMYKTQEGRAMLKLEEGDSHHHSNCGCGGNK*

>CD630_15370 Clostridioides_difficile_630_NC_009089 oxidoreductase

MDAKKVKVPVREQEPAVRATNFDEVCLGYNKEEAMAEANRCLACKKPKCVGGCPVGIDIP

GFITKIKEDDIEGAAKVIAKSSSLPAVCGRVCPQESQCEGVCILGIKSDAVSIGKLERFV

ADWSKENDINLSDTEPKKNQKVAVIGSGPAGLACAGDLAKKGYDVTIFEAMHEPGGVLTY

GIPEFRLPKQAVVQPEIDNIRKLGVKIETNVIVGKTITVDELIEDEGFEAIFIGSGAGLP

MFMNIPGENANGVFSANEFLTRVNLMKAYRDDYDTPISSGKKVAVVGGGNVAMDAARTAL

RLGSESYIVYRRSEKELPARAEEVHHAKEEGIIFNTLTNPKEILVDENGYVKGMVCIRME

LGEPDDSGRRRPIEIEGSEFVLDVDTVIMSLGTSPNPLISSTTKSLDINKKRCLITDENG

QTSKEGVFAGGDAVTGAATVISAMGAGKTAAASIDEYLKAKVNA*

>CDIF1296T_01612 Clostridioides_difficile_ATCC_9689__DSM_1296_strain_DSM1296_CP011968 ABC transporter ATP-binding protein

MVKVVYSRYYGIVVKIKNYGEYKMNILSIRNISKTYCGNIPFKALDKVSLNIEKGEFVSV

MGPSGSGKSTLLNIISTVDRQSEGEVVLDGYDVSKLKGEKLAEFRRKQLGFVFQDFNLID

TLTVGENIMLPLTLEGESIKDMNIQTKSISKFLGIDKILDRKTYEISGGQAQRCAIARAI

INKPAILLADEPTGNLDSKSTDDVLKLFTRINKEQKVTTLMVTHEAYSASYSDRVIFIKD

GCIYTEIKKSESSNSFYSDILAVLSQIGGVR*

>CD630_15410 Clostridioides_difficile_630_NC_009089 MATE family drug/sodium antiporter

MENQQLLGTERISKLLLKYSIPAIIGMLVNSLYNVVDRIFIGNIPGVGPLAITGLGVTMP

IMTIILAFGMLIGIGTTTTISIKLGQGKVEEARKLIGNAMTLSVITGIIIMILGILFANK

ILTLFGASENTLIYAKSYINIILLGTVVNLLSFSLNHSIRADGSPKISAGIMIVGCLTNI

VLDWILIFGFNLGIQGAAIATVTSQALTAILTIGYYISGKSNLRFSKSNLKLDKKLIKAV

FAIGMSPFAMQLAASLVQVISNIALKTHGGDLAIGAMATISSIAMVFLMPIFGINQGAQP

IIGFNYGAEKYDRVKKAYLGSLVVATIILCMGMVVVMLFPEAIIGIFNKDPELMNISVNG

LRIYLLMLPIVGLSVTGTNFIQSIGKAKMAMLLSLLRQVILLIPAVLILPTFLGLQGVWT

AQPVSDFIATVITGIVVFRELKRYTPKTEKLNENERLNEITTE*

>CD630_15430 Clostridioides_difficile_630_NC_009089 FMN-dependent NADH-azoreductase

MKKKLLYINVNSKPEDLSSSKTVARKFINKFMEKNKDFEVEEIDLYKEHIPRLEYQYFEK

RNSIVSEENAKNLDDKDRKELTKIRNLCDQFVSASVYVIAAPMWSLSFPAPLKEYIDCII

QDGKTISFEGNDKPQGILNDIDRSMVYIQSSGGHIPWVLKPVMNKGLNYVESIMKFIGIK

KFDELLVDGTGTSEEERQAAIEKASEKIDGIIDGMKF*

>CD630_15431 Clostridioides_difficile_630_NC_009089 hypothetical protein

MREEKSNEKYDCYWCNQENNFCVEIKDNIVMIDDGTGTLKQAVFIGYKQIQINLNCSHCQ

NLNRIKLNL*

>CD630_15460 Clostridioides_difficile_630_NC_009089 channel-forming hemolysin

MYQFFLKGRDPISSLTHFIGACLSLLATIILVFQSVTLQETSLLMIVSVSVFGLSLIALY

SASSYYHFLKGTPEQELFFRKVDHAMIYVLIAGSYTPICLNFMEKKEGIIFVTAIWIVAF

IGIIIKIFWMDAPRWLSTSIYLLMGWAIVFDINAFNSIPKDCLRLLIMEGVSYSIGAIIY

IIKKPNISPEFGFHEIFHIFIMIGSLFHFLAVLLYVL*

>CD630_15461 Clostridioides_difficile_630_NC_009089 hypothetical protein

MYKYDDYVNLTNDLDLPVEIRNKCVRIVSVSENLEDIIIVHKFKLYVVNEKYIKGIME*

>CD630_15490 Clostridioides_difficile_630_NC_009089 histidinol-phosphate aminotransferase

LREKESIRELRGYEPNHVNCKVKLDANEGSKRLFKYLIKEISDSDIDLNLYPEDSYSDLK

ESIIDYINISGVNKKNLLVGNGSSEIIDLIIHTFVDKDEVILSFSPSFSMYSIYSQINGS

KFIGVESDENLVINIDNVIEKVKENNPKIVIVCNPNNPTGTILKREEIIKLLDSTNSLVV

LDEAYMDFGEESMLSDVFKYDNLIVLRTLSKAFGLAGIRTGYMLSNSSLINSVEKVRPPY

NLNSLSDFIATRALRNKDVVKAYIKEVKEEREVLYEEMIGMGIKAYKSQANFILFYSEIE

NLSQKLIDRGVLIRKFGGKLENYYRVTIGDKEENSMFVGAIRDILKKEK*

>CD630_15500 Clostridioides_difficile_630_NC_009089 imidazoleglycerol-phosphate dehydratase

MRIWKVERNTLETQILVELNIDGSGKAEIDTGIGFLDHMLTLMSFHGKFDLKVICKGDTY

VDDHHSVEDIGIAIGEAFKNALGDKKGIRRYSNIYIPMDESLSMVAIDISNRPYLVFNAK

FDTQMIGSMSTQCFKEFFRAFVNESRVTLHINLLYGENDHHKIESIFKAFARALKEGSEI

VSNEIASSKGVL*

>CD630_15510 Clostridioides_difficile_630_NC_009089 imidazole glycerol phosphate synthase subunit HisH

MNIIVDYGLGNIDSVSRGFRKAGIETKISSDIDEIKQADSLILPGVGAFRDSISALDKLG

LIPIIKEHVSKGKFMIGICLGMQLLYEKSYEYGEYEGLGLIKGSIDKLDISLKVPHMGWN

NLKFNKANDDILKYINEDDYVYFVHSYYANSSNEELIAFSEYEKKIPAIVRKGNVYGIQF

HPEKSGEVGLNILRAYGEMIK*

>CD630_15520 Clostridioides_difficile_630_NC_009089 1-(5-phosphoribosyl)-5-[(5- phosphoribosylamino)methylideneamino] imidazole-4-carboxamide isomerase

MIIFPAIDIKDNKCVRLTQGEFDKVNVYYDNPLEVAYKWKNEGAEYIHIVDLNGARSEFG

VNTKIIEDIANNIDIPIQVGGGVRDKEKVKSLINAGVTRVILGSIAIENLNLVEELVNEY

KEKIVVSIDAKDGKVAVRGWEVVSNVDSLTLCKQLEKIGVQTIVYTDISKDGMLQGPNFD

IYERIAKETSLNVIASGGVTSIEDVKRLKAMNLYGAIIGKALYDKKIDFKEAQQLCLLGE

*

>CD630_15540 Clostridioides_difficile_630_NC_009089 bifunctional phosphoribosyl-AMP cyclohydrolase/phosphoribosyl-ATP pyrophosphatase

MDNKCNNVYSDEVEKFIRSIKFDDKGLVPVVVQEVVSKDVLMLAYMNKDAIKKTLKDKIA

CYFSRSRQELWVKGETSGNTQKVVKMSYDCDVDTILLFVEQTGVACHTGNYSCFYRDLFD

DSSKMELEVQTNILKELYDLINERKNNPVEGSYTNYLFEKGIDKILKKVGEESSEVIIAS

KNTDKSELIYEISDLVYHTLVLMIEKGVEIDEIKKELLKRRK*

>CD630_15550 Clostridioides_difficile_630_NC_009089 amino acid permease

MGSNELKKTLGVSAALSTVVGSVIGAGVFFKPQAVYTLTGGAPGLGILAWLIAGIITITA

GLTAAEVSVAIPKTGGMMVYIKEIYGEKLGFLTGWMQIVLFYPGMMAALGVIFGEQASAL

IGSPSLLLPIAIGIIIIVAGLNMLGSKTGGVIQTVSTICKLIPLILIMVVGFIKGGGNNP

ILTPMVGEGLSLGSVLGQVLIAILFAFDGWMNVGTLAGEMKNPGKDLPKAIIGGLSVVMA

VYFIINLAYLWVLPANELANYASPASAVAEVIFGSMGGKIISVGILISVFGALNGFLLTG

SRVAYTLATDKTLPKYGIFSKLNSAQVPANAIALVSVIASIYALSGQFNLLTDLAVFATW

IFYVLTFIGVMKLRKTHPNIPREYKVPLYPIVPIIAIASGIFVVVNQLCFAGMKTTMISI

GGLVITAIGLPVYAYMTRGVKR*

>CD630_15570 Clostridioides_difficile_630_NC_009089 peptidyl-prolyl isomerase

LNKKLYIGMVGILSLMMVGCNKSLAKVNDVEITKEQYKKTKAVLSATNNYINGQSLDELE

KTLDKKGRNKLENVIISFMVDNELLYQEAKDKGLTPSKSEVDSKYQELEDKMNLNTSYKE

KMDKAGVDKDYLKQEISRDLAIDKNKKAFEDRINISDNDMEAYYTSHKKDFNVEEVSASQ

ILISTLDKNKKEVSKDKKEALKKKADNILTKIKNGESFESLAKKYSDDKATGKNGGQLGY

FTKDDKNAEFTKEVFKLKKNEVSNVFETSYGYHIVKVTDKRERQKSFNECQSLIRESILN

EKYIEHIKKLNEDAKIDR*

>CD630_15580 Clostridioides_difficile_630_NC_009089 ECF RNA polymerase sigma factor CsfV

MFKSLKRTKVEKYIIDNKDSFYRIAYSYTKNEEDALDVVQEAMYKALYSVENIKEVNYIK

TWFYKILVRTSIDFIRKNRKYNNMTDIDLIDETGEYDKYTDLDLRRALEELPIEYKSIII

LRFFEDLKIEEVAIILDENVNTVKTRLYTALKKLKLKIEE*

>CD630_15590 Clostridioides_difficile_630_NC_009089 anti ECF RNA polymerase sigma factor RsiV

MTSKERLNKLKDEYHKIPIPKKLDTIINHEKIENIYREKNKKVNRLRFKVAIAFACIFTV

LVNISPVFADNFSKIPVIGAIVEVITIKNYSLKSENYEAEIDIPKIRGLKDKNLEQRLNS

SFMEDGKRLYHQFQERMEKIQSSKNKGYKSLSLSYSVKNNSKKFLSIEMTKNEIEASSYV

SKVHYTIDKKRQIVLTLPMLFKDDKYIKVISDNIKEQMREQMKKDSTKSYFIDQKKDLPV

EDFKTINKYQDFYFNKNEDLVICFDEYEVAPGYMGAVEFVIPYKVIKDL*

>QAE_RS0207755 Clostridioides_difficile_QCD_23m63_NZ_CM000660 xanthine dehydrogenase accessory protein XdhC

MNIYEQAIRLIENNEDFAFATITSHSGSTPRETGAMMIVKNDSTIFGSVGGGSVEAECIK

HAINVIKNRESMLYKFTLNKSDVAKLGMICGGTGEIQIDFIDSKLKSNIEKFNKRLKENT

SKAYIFGAGHISRDVAVILSLLEFRTVVIDDREEFANHERFPESEVIVLDSFEEIPDFPT

DENSYIIILTRGHLYDSSALEWALKRDAGYIGMIGSRTKIGLTYEKLMKKGFKKEELSRV

HAPIGIKLNAQTPAEIAVCIAAELINCRANKGKIN*

>CD630_15640 Clostridioides_difficile_630_NC_009089 hypothetical protein

MENTVYAKLNQGIDSFLRVAMTLRRREVDIQSISMTVDNMNNSGIKLIVNEEKTSLDSVL

NHMKKLHDIREITVERMRH*

>UAB_RS0208770 Clostridioides_difficile_ATCC_43255_NZ_CM000604 ketol-acid reductoisomerase

MKMARMYYEKDVDLEVLKNKKVAVLGYGSQGHAHAQNLRDNGVHVMIGLYDGSKSAQKAK

EDGFEVKSVAEATKESDLTMMLMPDEKQKKVYEESVKDNLKEGQTLAFAHGFNIHYNQVQ

PPEFVDVVMVAPKGPGHLVRNVFTKGSGVPALFAVYQDYTKKATETVLAYAKGIGATRAG

VLETTFKEETETDLFGEQSVLCGGISELIKLGYKTLVDAGYQKEVAYFECLHEMKLIVDL

IYEGGFERMRYSISDTAEYGDYVSGKRVITDAAKQGMQNVLEDIQNGKFAKAWIKENEEG

RENFLKTREEEYNTEIAEVGRNLRSMMSFLK*

>CD630_15660 Clostridioides_difficile_630_NC_009089 acetolactate synthase large subunit

VRMNGAKVILECLKKEGIDTIFGYPGGAVIPLYDALYDYSDDFKHIRTSHEQGLVHAADG

YARSTNTVGVCFTTSGPGATNAITGIATAFMDSSPMVVISGQVPTSLLGKDSFQEIDITG

ATLSMTKHNYLVRNTKELVPTIKEAFRVANSGRKGPVLVDVPKDLFLAEMDFSGEDYDLC

QIDDYMDYKSDFDLDDETNIKLLNEAIDIIKESKKPVIYAGGGVKSSDSEEILEKFATKI

DTPVLNTLMGLGNIDRKNELSLGMVGMHGSRESNLALSNSDLVIAIGARFSDRVISKSSE

FAKNAKIIHIDIDPSEISKNIESNVSLVGDVKLVLSLLIERVESKNNSNWKEEIKRFRKS

EGVQTYEFHPQNILKKINEKYETLKKPTVVVTDVGQHQMWAAKYWNFKGNKSFITSAGLG

TMGFGLGAAIGTKVGNVDKNVVLVTGDGSFRMNCNELATVANYNVPMLILLLNNRTLGMV

RQWQKLFSNQRYSQTDINENVDYVKLVNAYNIDGYKVSSMEELGKALDMIDFNKPVFLQC

DIDKDYDVYPIVAPNDALENLICN*

>QAE_RS0207780 Clostridioides_difficile_QCD_23m63_NZ_CM000660 manganese catalase

MFKHDKALLKEVKVERPNPQYAVLMQEQLGGANGELKAAMQYLSQSFRIKDPQIKDLFLD

IAAEELSHMEMVAQTINLLNGHDVDYNAVNTGEIETHVLTGLSPVLINSSGAPWTANYVT

VTGDLVADLLSNIASEQRAKVVYEYLYRQIDDKYVKETIDFLLNREEAHNALFRDALNKV

KDTGSNRDFGVTEDSKLYFDLSTPGPNHDTKIDVNPPSFEKPLKNNFIFFMLF*

>CD630_15740 Clostridioides_difficile_630_NC_009089 hypothetical protein

VKFKRLRTSENKLYYKAMELYKISFPFHEQRKSSLQEEILKNKEYQFNLIYDKNQFVGII

LCWETDDFIYVEHFCIFPEMRNNRYGQRALELLNKKGKTIILEIDLPIDEISIYRKAFYE

RVKYKTNNYEHIHPPYHEKFNGHNLIVMSYPEKLSKMEYDKFNHYLRNNVMSF*

>CD630_15750 Clostridioides_difficile_630_NC_009089 hypothetical protein

MEKREKRIIISSLLSVSILMGLTSIYSILNKEDIILTVKGQEQKVSSFKKTVEELLDEQG

VKYNSEDKINPSLDTELKDDMKIKVVKVTKSKKEEIEKIPFDTKHVNDSNLLKGKSKVYQ

EGQEGEKKLVYNLTYHDGKLVKKVLSKEVISKEPTTKIIKYGTKEKVLIASRGANIRGGK

HMKVVATAYAGDTITSTGTTPRWGVIAVDPRVIPYGTKVYIPKLGMTFVAEDCGGAIKGN

RIDIFMNSEGKASNWGRKSIDIYLH*

>EAA_RS0208060 Clostridioides_difficile_CIP_107932_NZ_CM000659 phosphoglucomutase

MDYTKTYEEWIKGSYFDEDTKLELENIKNNEKEIEDRFYKDLEFGTAGLRGIIEAGTNRI

NKYTVRRATFGLANYILENTTKEETSRGVVIAHDNRHKSRQFCIESANTLAACGIKAYIF

DSLRTTPELSFAVRSLNAIAGIVITASHNPPEYNGYKVYWEDGAQVMPEIANAITEKVNS

IHDYSTIPTLTEANKNLVVLLDESQDTKFIEAVKSQIIRKDLVKNVGKSFKIVYTPLCGT

GNVPIRRALKEVGFENIIVVPEEENPDPNFAGLDYPNPEEKKALNRGILLAKEKGADLVI

ATDPDCDRVGVAVKTTTGEYALLTGNQIGGMLTHYIIEGLKENNKLKENPTIIKTIVTSE

FGADIAKANNVDVLNVLTGFKFIGEKIKLFEQNKNRSYVFGYEESYGYLVGTHARDKDGV

VSSLLISEMAAFYYSKGISLYDGLIELYKKYGFFKEQTISLTLKGIEGVEKIKEIISYFR

ENQIDYINSIKVVDKKDYKNGIDNLPKSNVLKYFLEDESWVAIRPSGTEPKLKFYIAVKG

ASDIEADKKIQGLKKYIDDMVEKIKIKYYYFIR*

>CD630_15790 Clostridioides_difficile_630_NC_009089 sporulation-associated two-component sensor histidine kinase

MDTHNKYVNFIKNIPVPFLYCRIVKRQEDIEYRVEYISKGMGKVLQLEEGICDKNILDVL

PVFKSKKYFKELFSNEVDCIKRYIPTLKNWINIKKQIIGDSYIILYFGKIVFDYRQIIDS

FDKKEKVAYIKDEEGIYIDCSENLIPILNNNIKTTKDIFGKNDIEVWGENTGKLFRDDYR

EGVSSKKRFLQNLFEYEETFFMVEKYFLYDEDELLGTIGIVDNIIYSGYSNRNYNSKDLM

KMIEHSIPENMFYKDVYGNYIGFNSGFLNLACMNKEELLGKNSYKISEEEALIDKIFESD

KGVVENKKVVTFELNISMNDENKCIEITKRPFFDSYGSVIGIIGTARDISRRKRLEEEMD

KTRMEFFANLSHELRTPINLISSSLQVIEKKEADLIESNDTLKRNLGIIKQNGNRILRLV

NNVIDFTKMQSGYLDFKPEESDIIAFIEEICMSVADFASQNNIQLTFDTEIEEFSMLFDS

EKLERIILNLLSNGIKYNKKDGKINIFLYVKDNVFNMKISDSGIGIPKEKIDKIFNRFEQ

IDNELSYRVKGSGIGLSLVKSLVELHEGSISLKSQLGIGSEFIVSLPVRSKNNIEKYNHK

REISNELSKKLEIEFSDL*

>CD630_15800 Clostridioides_difficile_630_NC_009089 homoserine dehydrogenase

MENKVKIGVLGYGVVGSGLIDIIDNNKEKRNIEIVGILVNNLEKHKDKKYSNIITNNIDD

IFNKDIDILVEVMGGLEPSLSYIKKALNNKIHVVTANKDLLAECGDELAKLASENKVSIK

FEASVAGGIPVLKPIIESLEGNNIDSINAILNGTTNFILSKMYDENLSYDMALRQAQELG

FAEANPESDVLGYDAARKLSILSTLAYDNRVYWKDVYLEGITDIDEKDIEYAKKLNCKIK

LIGQSKYENDKVSAFVRPVLVEKDNILARIDNEFNAVIVNGDSVGEVSFVGKGAGSLATG

SAVYSDVIDIIDNRVSSIDSFTKDKIQVNKIVREKCGALLRFKKCNKDEILNIVENCLVK

FDILNDDDELAIMVYADSEYEINNSLCLIKDKGYCEKMNKMLKIS*

>CD196_RS08285 Clostridioides_difficile_CD196_NC_013315 hypothetical protein

MENKKYANGGYSEDWYERGESTAKWFQNDREEYERAAYDEDRERRGSNCGCSDSGGNRPR

NCERFRREAEIREREAREAFCESSERKKEALAYECEARKLWEEAEKYWDEYSKYNYKGIE

YLAEAARLFDEGMECEARRNGNNGGNNNNCCHKCHKCNCNCCRK*

>CD630_15820 Clostridioides_difficile_630_NC_009089 histidinol dehydrogenase

MKSIKTSSMKEKVDEKILREQVSSIIEDIRNNKDVALKKYNEKFDRNTRDEFRITKEEIK

EAYKHVDDEFINNLKIAAKNIREFAKAQKLSFENPFEKEIYPGVILGQTNIPIESCLAYV

PGGQYPLFSTALMLIIPAKVAGVKRIVACSPTMKNTEKINPKTLVAMDIAGADEIYATGG

VQAIAAFTYGTEKINPVDIIVGPGNKFVTEAKRQCYGQVGIDFVAGPSEVLIIADETSNP

VYIAADLLAQCEHDLNARGILLTNSLEVAQKVEKNIESMLKDLPTKDISYSSWENNGEII

LVDDMEEAIKISNFYAPEHLEVAVNEYNDICDRLTNYGSLFIGNLSAEVFGDYVSGTNHT

LPTLKASRYTGGVWVGTFIKTCTKQIFNEKAIQSLAPVAEKLAKEEGLYAHAKAAEVRFK

K*

>CD630_15830 Clostridioides_difficile_630_NC_009089 hypothetical protein

MSYFQYQGNSCFYKEYGQGKPIIFLHGNTGSSNMFKALVPLYVENFRCILIDFLGNGQSD

RVSQFSPDIWHDEALQTIALIEHLNCGKVGLIGTSGGAWAAVNTGLERPDLVEAIIADSF

DGRTLNDNFIDNLITGREKSKQDIQARKFYEWCQGKDWENVVNLDTKALLQCANEKRPLF

HKELCKLEMPILFTGSKEDEMCRHNLEEEYKQMATFISKASIHIFSQGGHPAILTNADEF

AQLAKVFFNY*

>CD630_15900 Clostridioides_difficile_630_NC_009089 membrane protein

MARRLFLERIGLIFLGSAILAFGVYNFYYLNNITEGGVLGILLLLKNLFNIQPAIANVVI

DGLLLLVGYKFFGKKFLIYSIVASITFSVLYDLFEAIGPLVPQSQNMLLSTILAGVTVGT

GVGIVVKAGCASGGDDALALVISKTTSLNIGQVYLATDVIVLLLSLFYLSAFDIFYSLIA

VTISGKVIDFIYYHGKSLDMDISNDIVPEC*

>CD630_15910 Clostridioides_difficile_630_NC_009089 potassium-transporting ATPase subunit A

LGGTIFMVQIVIVLAIFMILVIPMGKYLYHIATNQKTFGDRLFDKVDNFIYKVCSIDKKK

EMNWKQYALALLFTNAVMVFIGYIILRTQSMHIFNPSGIKSMEQGLSFNTIISFMTNTNL

QHYSGESGLSYFSQMTVIIYMMFTSAATGYAAAMAFVRGLVGKKKTLGNFYVDLIRITTR

VLLPGALIIGLILVTQGVPQTFAGTETVTTIEGKLQDIARGPVAALESIKHLGTNGGGFF

GSNSSHPFENPTIISNIVEILSMMILPGACVVAFGHMIKNKKQGWVVFGAMSIIFLIGLV

VCFKAESAGNPILSQLGLNQSMGSMEGKEVRFGIAQSSLFTTVTTSFTTGTVNNMHDTLT

PLGGLVPLLNMMLNVVFGGKGVGLMNMLMYAIIAVFLCGLMVGRTPEFLTKKIEGKEMKL

IALLIILHPLLILMFSGLSVAIPAGLEGISNPGFHGLSQVLYEFASSAANNGSGFEGLGD

NTMFWNITTGVVMFFGRYVSIIVLLAISSLLASKKAVNESIGTLRTDNFTFTIVLVLVVL

IVGALTFFPALALGPISEHLVLWH*

>CD630_15950 Clostridioides_difficile_630_NC_009089 serine acetyltransferase

LFKKINKDIEYIMKNDPAARSKIEVFLLYPSVHAMIMHRMAHALYKKKKLFTARLISQIS

RFMTGIEIHPGAKMGEGILIDHGMGVVIGETAEVGNRVTIYQGATLGATGKDTGKRHPTV

GDDVLIGAGTKILGPLNIGSNSKIGANSVVVKDVPNGATVVGIPAKIVKIRNLEPVKKNK

KEVSYEYDELDNVYYI*

>CD630_15951 Clostridioides_difficile_630_NC_009089 ferredoxin

MKANVNQDTCIGCGLCPSICPEVFDMKDDGKSHVIVDEVPSDAEESAAEARESCPVDAID

VH*

>CD630_15970 Clostridioides_difficile_630_NC_009089 hypothetical protein

MIMENNLNTAKRNNTELLQNDSLKVNLQIGVDFTLLSKSLKAFYKKEKDFYCIALAPCNV

SSEKSNRKISIYDMIKEINILIESITDKNELLSEELFTNNLSEFYDESLDEIYIDLKQAY

LYIKKNTDGLNIEDSIEYVFDVDISNDIKAKENPLINFNSLSLVVWSGETKHILQQMNIL

DLEDIHSYSIENNTNITIYENNTLQYKFDEESENIKDSSIDIIEQMLILPDDLNYDKDEV

ENMKNRLAKINIKYLQTLKEKDIKIKLINSNLTDEPEFSDLKYQLPPCWVRSGKTWKDVP

GIYRNNSIVAKIGYSNPSYANVHSSKNLELHETAHAIDKNVLNKKSNSEEFMEVFAQERY

KLYDPKQVAHAYISKFIEEFFAESFVHYYLDEDSKNTLKENCPLTYDFLEKLELNY*

>CD630_15980 Clostridioides_difficile_630_NC_009089 hypothetical protein

VYFTYIIRCKDDSLYTGYTSNIVRRMNEHKLGINSKYTRAKGFEKLEVYFVTNTKSNAMK

LEYYIKKLTRNKKLSIIKNPSILINLIDNKEDYIIGKEIEQLT*

>CD630_16000 Clostridioides_difficile_630_NC_009089 hydroxyethylthiazole kinase

MYNLIKDVKKLNPLVIHYTNNVTINDCANVTLAVGASPLMSFSYEEVEEMVSVANSVVIN

IGTMNSNMLDLFLLAGKAANKYNKPVVLDPVGVFASKARAELTSRLLNEVKFSVVKGNVS

EIKFIGGFNVRGKGVDSFDEEEDSTEIIRKIAEKLECVVVATGKIDIITNGKGTYKINNG

TDKLKGITGTGCMTASLIASFMAVTENILEAATMGVLTMSLSGELANLNNPPIGTFKENL

MNAIYQMDIDALSKNSNIEFLN*

>CD630_16010 Clostridioides_difficile_630_NC_009089 thiamine-phosphate synthase

MIDKESLKKCLKLYLVTDSEMLKGRDFYKCLEDAISSGITTVQLREKNASGREFLRKAMK

LREITKRYGVKFIINDRVDIALICDADGVHVGQSDIDVREVRKLIGNNKILGVSARTLEE

AICAKNDGADYLGVGSIFTTSTKLDAKSASFETVKEIKEKVDMPFVLIGGINLDNIDKLK

CLESDGYAIISAILKAEDISKEVEKWTLKI*

>CD630_16020 Clostridioides_difficile_630_NC_009089 PTS system lactose/cellobiose-family transporter subunit IIB

MIRILLVCVGGMSSTLLVNKMEKDAKKRNIDCKIWAVGEGDIKSELDNFDILLLGPQLRF

MLDDVKSIVGDRAPVSIIDMVNYGTCNGHAVLNSVLEILK*

>CD630_16030 Clostridioides_difficile_630_NC_009089 multidrug family ABC transporter permease

MGMLNILWRSMKWRFKNPISFVVTILQPFLWLVLYSSIANQTMNNININNYTAFILPGII

VLVVFSSCSSGGIINFIMKNSGSFYRVLIAPISRYSIVLGQLLEAILVSFIEVTILCIVS

IFFSVRIESGIGGILLMIVLIFMTAFFLSSLAYSISLLLPNEIVYETIMTAIVLPIFFLS

SALFPIESLSGGLKVAVMLNPFTHVINALRSLIFGETILIGDIMPVILLFLILCCSSFSL

AMWRLKKEMVS*

>CD630_16040 Clostridioides_difficile_630_NC_009089 multidrug family ABC transporter ATP-binding protein

MLAIEIDNLVKEYKNGVKALNGLSFNVNAGEIFSLLGPNGAGKSSLINILTTFYKPTSGN

VTMFGKDLVDNPSWIRTQIACVAQQISIDEHLSLMENMVFQSKMYKVEPQIAKQRIDSLI

DKFDLSSYLKYPTSSYSGGVKRRLDIAMNMVSSPKILFLDEPTVGMDVDSRKSMWDMLLK

IRDEYGTTIFLTTHYLEEAEQLSDNICIMKNGKDLAQGTPSSLRSYIRQNILRITFHNTE

DIKKYKDSIKSTGLVKFMSVRENSIFISVNDSRTAFTLINKWLLEHDIEFDAIEIVEPSL

EDVFLALTSSKKSLKEEWEC*

>CD630_16060 Clostridioides_difficile_630_NC_009089 GntR family transcriptional regulator

MNFELDNTTPIYLQIVKYIKRQIVTGELKPGETIPSRREMALNLKVNLNTVQRAYKEMGD

MNIINTFKNYQSSVTVDENILKNLKLELINESLSVFIEDMKAINVSKEEVLKIIEDKY*

>CD630_16070 Clostridioides_difficile_630_NC_009089 multidrug family ABC transporter ATP-binding protein

MIEIKNVSKTYKRMQGLKIRKIEALKNVSFNIEKGKITALLGINGVGKSTMLKAIAGLIN

IDSGEIRIDGEKINEKVYNKLAFVPDVQSHFSNTTIKETFEFMEIFYSKWNKEKSKEMMD

IFKLDEDEIIDNLSKGNIARVKLILGFCQDPEYILLDEPFTGIDLFKREEFIGVIAQYME

ENQAIIITTHEIVEIESLVDEVVILDEGQIITSFNAEELREREGKSILDKMREVYKNE*

>CD630_16100 Clostridioides_difficile_630_NC_009089 hypothetical protein

LMKDLIRLFEFELKRNIKNYIFIIICCCSFVILNIVKNLNDYNYIIENAVKSEQLTKIGN

TIETVNVTGFSSFRNIMGSTESWFMFGIIACICYAFFIWYRDFNGRSKSIYTLIMLPKNR

INIYISKLLNILFLVYSYTVVLTISLFIASKLLPRHMLGNVTNYGFVQETIYELKMLLPY

SFEILFVEYIFLLIGFVSVVFTSILINKSIYKVSTLISFLFLVIEILVFIISIEFIIPYE

YSDVFTVLYSSINIFVCSLISNKLLKQKIDF*

>CD630_16160 Clostridioides_difficile_630_NC_009089 diguanylate kinase signaling protein

MRNLQKKLMFFLFICIILQITYTTPVYSLCKLYKNNSINNFESIVSNNQLISKHAIQIIL

IMAIICVILIFYIIYDKLNFKIKLQRIAYTDNLTGANTIDKFVIDANKILCKNTQVKYAL

LYIDIDKFKYINDLFGYEVGNEILCNLTKIIKSNIFEEEMFARISADNFIIIMKYIEEED

ITKRLKTIFEELDLFNNNQEEKYKLVLSCGIYFILPEDRDINSIIDRANIPHKMAKGGHK

SSYAFYDNKIHDQEIKEKEMENTMFSSLENKEFIIYLQPKIELNTGEIQGSEALVRWKRP

DKGLIPPNEFIPFFERNGFVINLDLYVLEEVCIYLRKWIDAGINPVTVSVNVSRIHLYCN

NFIETYKNIIDKYNIPAKYIELELTESIIFDNFDILIDIMNNLKKIGFLISMDDFGSGYS

SLNMLKEIPMDILKLDQKFIMETYNSKRSKIIVTKVIEMAKELGMKVISEGVETEEQFKL

LKEVKCDMAQGYLFGKPMPIEEFEHLIVSNLVRG*

>CD630_16170 Clostridioides_difficile_630_NC_009089 GntR family transcriptional regulator

MEWELDNNKPIYIQLVEHLKLKIISGEIKIGSKLETVRALAEDAEVNPNTMQKALTELER

QGLVYSQRTKGRFVTDDKEKIKAMKEEIANVEINTLKVTLEKLGYDRDEMLKLITENLKG

EL*

>CD630_16190 Clostridioides_difficile_630_NC_009089 multidrug family ABC transporter permease

MLGKLLKYELKASGRIFIPLYIAILIVAVFNGIFMNTNILQVQGIGILVLTSLFMALGVL

TIVVTIQRFRKNLLGDEGYLMFTLPVSTSSLILSKCITALIYAVLSFIVAVFTFGVLMLF

GTSGILLPEILDLFNTSFKWISENFLDILLLVVVMFISYSSFILLLYTSISMGQLPKFNK

HRNIVAFASFIAINIVISIVGDAVGSILPNEDTNMVYHLYQSPSFMLAILGSLVVAIALF

FATKFILDKKLNLE*

>CD630_16200 Clostridioides_difficile_630_NC_009089 major facilitator superfamily transporter

MKSYTKAKWSVWGIITFSFVLVLFLRMSTAVVSDNLANELGFNSIQISNIASFCLYAYAF

MQIPAGILIDKYGARKISSLGIIMASLGSILFGLIQSIELAYISRVIVGAGTSVILLCIL

KIQGRWFNKSEFASATAKFSFVGNLGGVLATFPLVFLSELVGWRNSFLLIGIIGVVIGCF

MYIIVRDTPKEYGFNVDTEPYEKSEKVNIVDGIKSVIKNKSTWYNSMIMFSFVGLTSAFI

SLWGVRYIMDVYGVSKSFSAFIVSFFTYGFIFGSIIMDFVFAKIRSSKFNIIKFGAMIDL

FIWIVIVVVYQVKPPIIVLPISFFIMGCIVMSHLQVFNDAKYKNKEIYSGLATSVINTFE

FIGSGIINLIIAISLQVNSYNIVDGYKKGFVVFIVLSIITIVSSHIGVKNDDFKAI*

>CD630_16220 Clostridioides_difficile_630_NC_009089 hypothetical protein

MCKKTYLLVVSMLIAGLLTACSGSPTKDSGQAQDNTKKEANASNNALKDEKNNENLMEQD

FKVPYTDAINIFKDKYKDADIVDLSLERDLNKFVYTVEGVDDNNEYKMKIDANTKDVLED

KTEKLDSEDLNGVARKEKLDLNDIMTPQQAMEIALKEQNGIVKEWSLDKDLDVTFYKIRI

DKDKNEYDIKVDSKKGTVLKVEKED*

>CD630_16230 Clostridioides_difficile_630_NC_009089 oxidoreductase

MSLKSLKIKENLYWVGSLDPDLRVFDIIMYTPYGTTYNSYVLKGTEKTVLFETVKDKHFD

NYIERLNDLNIDFEKIDYIVVSHTEPDHAGSVEKLLDLAKNAKVVASETAIKYLKEIVNK

DFEYVAVTDGDTLSIGDKTLEFFSVPMLHWPDTIYTYIKEDKTLVTCDSFGSHYSNDKIV

NTLDENEEKDYLDALRYYYDCIMGPFKPSMVTAIEKIKDLDIDTVCPGHGPVLTENPRKI

IDLYYNWSVNEQIKLEKEVTICYVSAHGYTKIMAEAIKAYIEKNSNYKVNLFDVIEHKQE

DILAKIAVSQGVLFGTPTILGDALKPIWDILISLNPVLHGGKVASVFGSYGWSGEGIENA

MERISQLRMTAVKPFAVNFKPSNEEIDKLHSYTGKFLDKLNSTFGSKKKTKKFKCVICNE

VFEGDSAPSVCPVCGAKEDQFIEVEEDEVTFRKDTDEYFVIVGNGAAGFYAADAIRKRNK

TCKITMISNEDELTYYRPALSDGINEELGSDFYMEDKDWYDKNNIVVILGTNVDKLDEVN

KTIIVNDGAIKFDKLVIATGSRNFIPPIKGHDLENVFTLRNIKDLYSVKEALEKSKKVVV

IGGGLLGLEAAWEFRLKGLEVVVVEAMDSILSKQLDKEGSKILEQCVRDTGIDVRLGVAV

DGIEGDVKAQKVVFKDGDSVDCDMVVFSIGVRANTQMVQDTSVKIDRGIVVDKTLQTNVK

DIYACGDVAQVGNISLAIWPSSVEMGKIAGANASGDNLTFESEVYPVSLDAMNVKVFSIG

NIQNFDKEISSKDEGQRIYKKLFMKDGSLVGAILINDLSCTVKLIRLISEKGDFEDIMKA

DIL*

>CD630_16290 Clostridioides_difficile_630_NC_009089 hypothetical protein

MQYIQELLDLTQNYWVLATIIGLLSAFIESFIPALPLVAIVTANAAIQGLFIGCLLSWIG

SGLGTTSLFLLISRFNDSKLFNKLRNSKTEKAISWMDKQGFKLLFIAYACPFMPGCLVTI

ASAFGKKDLKDFVPAMLAGKFVMFIVISYVASDIEGFITSPLKIASFILLVFLSWKIGNR

VNKNLENHNYDFHHKKHHNDKDDKMI*

>CD630_16310 Clostridioides_difficile_630_NC_009089 superoxide dismutase

MKKKILIPVIMSLFIISQCITSFAFTPENNKFKVKPLPYAYDALEPYIDKETMKLHHDKH

YQAYVDKLNAALEKYPELYNYSLCELLQNLDSLPKDIATTVRNNAGGAYNHKFFFDIMTP

EKTIPSESLKEAIDRDFGSFEKFKQEFQKSALDVFGSGWAWLVATKDGKLSIMTTPNQDS

PVSKNLTPIIGLDVWEHAYYLKYQNRRNEYIDNWFNVVNWNGALENYKNLKSQD*

>CDM120_RS08485 Clostridioides_difficile_M120_NC_017174 asparagine synthetase B

MDGNISIYYKGDISKKENIKFKIKESLNNINNTRVSSRIEYKSEAKIFDFERFNVIDVIY

GNKPFEKYGKVIVFNGNIYNNHEIKEELIKRGHSFTTGDDIEVLLASYIEFGKNCVHKIK

GMFNFIIYDRENESIFGARDLFGIKPLYYINKESAIIFSSEYKFILEYMKNLNINERSLQ

SYFSFQYVLPEDTMIQGIRLIPAGHYFRVENGILSLKRYNKLEFRSSTKFFYTKNHLGNR

DVDKEEVRNIVVDSIRNHMEEHKEIGTFLSGGIDSSIITTVASQINPNIKSFSAGFSVKG

YSELEVAKKTADKLGIENIQINITQDEYIKALPNVIYSLDDPIADPSEVGLYFLIKEAGK

HVKVALSGEGADELFGGYNIYKEYSTMKSVVNSPTYIKGILGRVSELMPNIKGRNYLYRA

TTPLEKRYIGNAKVFENSEVKRFFFKYKEKNIYEYLLSNLYRDAQKNNYDYISKMQHIDV

NTWLQGDILQKISKLSTAEQVELRVPFLYKDVFDVAKNLRMEQKINKNNTKVLLREAFRE

IVPEHVVQRKKLGFPTPIRVWLKDSLGGIVKETISNSNVDEFIDKKYVIKLLDMHLKGHR

DNSRKIWTIFTFCLWHQLFIEHKNVEY*

>CD630_16330 Clostridioides_difficile_630_NC_009089 serine protease

MICTIKPLKISIKKSTEPLIEFISDTTYKNKRSSYIKEVYFTRYIGVDDSIDKYIEKIKN

IDTYFFNNPTIPYIRLNNLKINFDKEQTDKMLKIFNIYNSSKFNSWDLYNIDFPCKIEND

TLNWTKKIAFKNTLDLFSTSMPNCTSSIIKNFAVKLLCWMDYFLPKLFYNKVKTTISPKI

VYFGNIKRQELFFLYFLSQLGCDILYINPNKDILDLYPECSKFSTLFELSRKTPNILEIP

FERHITNINYSENTNKTSPTTNKPNSKNTLNNTNLTSKKIVQDEEVELSYEDLAKLSASV

VMIVVCNNENKPFKSGSGVVINNEGYILTNLHVVNDGYSFLVRFENDDKVYTSYQIIKYH

SDYDLAVIKVDRKCKPIPVKVSKKPVRGQKIVAIGSPLGLFNTVSDGIISAFRDFETVQM

IQFTAPISSGSSGGALLDMFGNLLGLISAGYDDGQNLNLAVESSLVKIFANNFIEIVN*

>CD630_16360 Clostridioides_difficile_630_NC_009089 tellurium resistance protein

MSINLSKGDKIDLKKSNPGLSNILVGLGWDPVQQSGGGFFKSLFGGGQADIDCDASVFML

NQEGKLSGIKDLIYFGNLKSACKSVLHTGDNLTGEGTGDDEQILVNLDKVPSNIHKLLFV

VNIYNCVDRKQHFGMIENAYIRVEDQGNKKEIAKYNLSDNYSEKTTLIVGAIYRKDGSWQ

FKAIGEGTKDAGLKEVMQNLDRIECAYGI*

>CD630_16390 Clostridioides_difficile_630_NC_009089 tellurite associated resistance protein

MGININSFSKDKNSVEVTENLPTAVQEENFDIMEYTNNKKNELRKSKEVEALTSLIEVEN

PDTILQFGRKASEGVARVSDSLLNTIKLNRNEENSKMLVHLTKIMDKFDLDDFQETKEPN

FVQKLFKKANNAIEMMFQKYETLGGEVEKIQIELEQYERDIALSNKQIGAMLNENFEFYN

ELQKYIVAGEMAIEEMDNEILPFFKQKSETSGDQMDVVNYQELLKVYDMLNQRVYDLRIA

ENIAIQTIPMLRGMQHNNYGLIRKINSAFVVTLPVFKQCLSQAILLKKQELQAKSLKALD

DKTNELLLRNAQNVSTQSAQIARMAGTSSVQIETLEKMYNTIKSGIDETMRIEENNRALI

KDNTKRLEELNTTIIYNK*

>CD630_16420 Clostridioides_difficile_630_NC_009089 hypothetical protein

MRDFSTYSKDDVIFLLKDISNLIEEEGNKERELKIQNGRHYSEMIPIEYEVSEDYLNLYH

EKLRENKDKLAFSIGVMCEKIIKKNGNDVVLVSLARAGTPIGILAKRYIRSKYNISLPHY

TISIIRDKGIDMNAIEYIVKNHPSSKIQFLDGWTGKGTISKELEKACNELNVKFKKHFDS

TLAVLADPAGYSGLYGIREDFLIPSACLNSTVSGLVSRTVLREDLIGKNDFHGAKFYRHL

KDKDESMNYIETIEECFKNQFKNISDEVENWESDIITRDGYFDVLNIKEKYNITDINFIK

PGVGETTRVLLRRVPYKILVKDLNDKSLDHIFILAKEKNVEVEQMDLKAYKCCGIIKNMK

DI*

>CD630_16430 Clostridioides_difficile_630_NC_009089 hypothetical protein

MIVFSDLDRSIIYSNKFLNADSKYANIEIYREKEISYISLDTINLIKQIQYYGMFIPTTT

RTVEQFKRIEFNKYGIYFPWSITSNGGVILKDNEILKSWSEKIDKLKSNYEPIESMIHKF

KDYLNVDGITNFKVAEDTFFYIVVDLSRFNLDSIKEYTNILESKNWKFYVSGRKIYFIPK

EISKENAIKYLTKELGIEYFYAVGDSIMDYGMLNISNKSYVLKHGDINKNEIENSFISSS

FNGMSGTEEILSNILDENCLLNI*

>PCZ31_RS15890 Peptoclostridium_difficile_strain_Z31_NZ_CP013196 membrane protein

MEVIKMKSFVSPDNIWVLWAVVTGWAAFSIYLEQKYNWASKVSGAIIALVGAMLLSNLNI

IPVESVVYDQVWGYVVPLAIALLLYQCNIKKIWKESGRLLIIFLVGSVGTVLGAMIGFLA

LKNVVPDLNIVAAMMTGSYIGGNVNFAAMSGAFDAPGELVSATVVADNLLMALYFFVLIA

IPSIGFFRKHFKHPHVDEMESIGITEGETVAANYWGRKEISLKDIAIAIGSAFIIVAVSV

ELSTWFKAIIPLSNPFLAMLNTLFGNQYLIITTLTMLFATFMPNFFGDIKGAQELGTFLI

YIFFVVVGVPASITLIIQKSPLLLLYCGIMVLINMLVTFIVAKIFKFSLEEAILASNANI

GGPTTAAAMAISKGWSKLVGPILIVGTFGYIVGNYFGLLVGNILI*

>CDM68_RS08640 Clostridioides_difficile_M68_NC_017175 iron ABC transporter permease

MIIIFILINKRFLRRENEKRYLVIGIIILSYFSLFIGAEDINIMHIFAKNQHKLMIFIMS

RVPRLISILIAGVGMSVAGLIMQQISKNKFVSPTTGATIDAAQFGIVICMLLVPTASIFT

KTIIAFVFSLVGTFMFMKIIGKLQFKNIIFVPLVGIMFGNIIGSMTDFIAYKYDLSQNVS

SWMQGDFSMILKGNYEILYITIPLIILAYIYANKFTVVGMGMDFATNLGLSYKRIVNIGL

IIVALVTVCVVVTAGNIPFIGLIVPNIVSLYMGDNIRASIWYTGLLGAIFVLICDIFGRI

IIYPYEISIGLTVGVIGSILFLYLILRRNVNEA*

>CD630_16480 Clostridioides_difficile_630_NC_009089 iron family ABC transporter permease

MKLSLNQKIYFVSILLFISIALFVIYGIDMNHLEYALSQRIPKIFAMVLGGGCIAFTTVV

FQTITNNQILTPSVLGLDSLYVMIQTIIVFIFGSSSSLIINENYNFIINVAMMIGASLLL

YKTLFEKNKNNIFFLILVGMIFGTLFKSATTFIQVMIDPNEFLALQTSIMASLNNINTNV

LLIAFIIIIAIIPFIYDEIKYLDVLSLGKEQAINLGVDFDKAVKKMIILIAILVSISTAL

IGPMTFLGLLLANITREIFKTYKHTYLISGSMLIGMITLIIGQFFIQHVFKFDTTLSVVI

NFIGGIYFIQLLLKGANR*

>CD630_16490 Clostridioides_difficile_630_NC_009089 iron family ABC transporter ATP-binding protein

MIEIKNIFKRYKNKNVVDDVSFSIEKGKITSFIGPNGAGKSTVLSIVTRLIGGDGGEVII

EGKSLTNYSNKELAKKIAILKQSNNITLKLTIRELVGFGRFPYSEGNLTKEDENYIDEAI

EYMKLTDIQHKYLDELSGGQRQRAYIAMVIAQDTEYILLDEPLNNLDMNHSVQMMKVLRS

LCDELDKTIVLVMHDINFASCYSDNIVALKNGKVEKVGRTDEIVNEKVLEDIYEMNFNIK

NINGNRICIYF*

>CD630_16520 Clostridioides_difficile_630_NC_009089 tellurium resistance protein

MAIELKKGQKINLTKKENSDLGEILVNLNWNQKVQKKGFFGSLRSSNIDLDLGCLFEMKN

GVKGAVQALGNAFGSLDNPPFAQLDGDDRTGSNTQGENLRINGNKIKDIKRILIYAFIYE

GVANWSEADGIVTIKQKQDSDLVVKLDEHKNGYNMCSIALIENVNDETFSVEKVVKYFKG

HREMDTEFHWGLKWVAGRK*

>CD630_16530 Clostridioides_difficile_630_NC_009089 lipoprotein

MKFKKLLCLLLCLVLTLAVVGCSKAKDDKKIVVGATLVPGGELLEELKPLIKEKGYTLEV

KNFDDYILPNEALNNGEIDANLFQHEPYLKEAVKAKGYKIMAGKKLYVCPAILYSYKIKS

VDEFKKGDTIAISNNPSSCSKNLRYLESIGLLTLPKGDGLVSPKDIIENPKGIQFKELDI

AQIPSSLPDVTAAFIDTTYAVPAGLDAKKNGIYTAPINDEYANLLAFRTEDKDSEKIKVL

QDVLTSDKARSLIEEKYKGIVIPTF*

>CD630_16550 Clostridioides_difficile_630_NC_009089 Na+/H+ antiporter NhaC-like portein

MRNKKINIIFLTTIMFIMSTVMVFAEEDIDTIALANAEKFGILTLIPPLVAIILAFITKN

VIISLLIGILSGSFIIKASGINVFATFIQAFLDLVDRALVSLADPWNAGIILQVLAIGGV

INLVAKMGGAKAIAEALAKRAKSAKGTQLITWFLGLLVFFDDYANSLIVGPMMRPVADKM

KISREKLAFIIDATAAPVAGLAIISTWIGLEVGLIHDAFESISIDVDAFGIFLNTIPFRF

YNILILAFIVISALLLKEFGPMRKAEIKSRSRKISIDLDEGVEELDDLAPKNGVKLSVWN

AIIPIGTLIIVALASFYYSGYTSIMGGDDKALIQLFTNSPYSFEAIKEAFSASDASRALF

QSALVASLVAIIMAVVKKIFTISEAIDVWIDGMKSLVITGVILILAWSLSSVIKELGTAK

FLIHLLSGSLPPFLLPSLIFGLGAIISFATGTAYGTMGILMPLAIPLAYSLNPDMSYVIV

STSAVLTGAIFGDHCSPISDTTILSSMGAGCNHIDHVNTQMPYAIFTAVITIVFGYIPAG

LGLPIYIVLPVAIAAIFVGIQIIGKKVDEAEIELVE*

>CD630_16570 Clostridioides_difficile_630_NC_009089 bifunctional glycine dehydrogenase/aminomethyl transferase

MNELKRVSLYNIHKELGAKLVEFAGWEMPLEYEGINKEHEKVRKSAGIFDVSHMGEVQIK

GAESEKFIQNLVTNDISTLKINDIIYTPMCYENGGVVDDLLIYKFGEEDYLLVINAGNID

KDVAWIIKQSEGYNVDIKNISSEVSQLAIQGPKAEEILQKITDIDLNSIKFYKSIPSIIV

CGCPCLVSRTGYTGEDGFEIYCKNKYVEIIWNEVLKVGGEDICPAGLGCRDTLRFEAALP

LYGHEINEHISPIEGGLSIFVKTNKESFIGKSILSKEKESGAKRKLVGFEMQGKGMPRNG

YDIRIGDKTVGFVTTGCASPTTGKILGMGIIDSEYAKVGNEIGIAIRKKVVPAVIVKKPF

YKKQYKKDNIILNKENKFSYIPATSEDKSKMLKVVGLNSVDELFSDIPEEVKLKRDLNLE

IGKSELEVSKIVKRLSEENLSLEDLTCFLGAGAYDHYIPSIIKHITSRSEFYTAYTPYQA

EISQGTLQVVFEFQSMIAEITGMEIANASMYDGATAAIEACIMAMNQTRKSKIVVSKTIH

PETLSVLRTYLQYKDCEIVEIDFCNEYGTTDIEKLKASVDKDTACVLIQTPNFFGIIEEM

EEIEKITHENKAMLIMSVDPISLGVLKTPGEIGADIVVGEAQSLGNPLNFGGPYVGFLAS

KSKYTRKMPGRIVGQSLDVEGKIAYVLTLQTREQHVRREKATSNICSNQALNALVASIYM

ATMGKEGFKEVGMQSMKKAHYTYNKLVQTGKYKPIFKGKFFKEFAVQGNLNIETINDKLL

EENILGGYNLEYNYPELKNSTLLCVTEKRSKEEIDKLVGIMEGL*

>CD630_16580 Clostridioides_difficile_630_NC_009089 glycine dehydrogenase subunit 2

MKEYNSLLIDISKKGRKAYSLPKLDIDDIKIEDMIDQDMARQSELNLPEVGELELVRHYT

LLSNKNFGVDTGFYPLGSCTMKYNPKINEDMAALPNFTGMHPYQSSDTAQGSLSLMYDLS

RRLAEITGMDEVTLQPSAGSHGEFTGLMIIKAYHENRGDHKRTKVIIPDSAHGTNPASAA

MANFDVIQIASDKNGAVDINVLKEVLNDEVAALMLTNPSTLGLFEKNIKEIATLVHEAGG

LLYYDGANMNAIMGITRPGDMGFDVVHLNLHKTFSTPHGGGGPGAGPVGVKKELVPFLPI

PVIERKEDKYVLNYDREKSIGKIKNFYGNFGVLVRAYTYILTMGRDGLKEASEMAVLNAN

YIKESIKDDYILPIDTLCKHEFVLGGLPRGEANIKTIDIAKRLLDYGYHPPTMYFPLIIN

EALMIEPTESESIETLDSFIEAMKSVAKEAKEEPELLKTAPHNTLVKRVDDARAVKKPIL

TWSQR*

>CD630_16600 Clostridioides_difficile_630_NC_009089 hypothetical protein

MSEFKNVTAVKKANVYFDGKVSSRVIILPNGERKTLGLMLPGEYTFSTREEEIMEMLAGS

MDVKLPGSNEFVTYKEGQKFNVPSDSSFDLKVNEVVDYCCSYIAD*

>CD630_16640 Clostridioides_difficile_630_NC_009089 transcriptional regulator

MSINTVIAKNLNRLRNERNLSLGQLAELSGVSKVMLSQIEKGDSNPTVNTIWKIASGLNV

PYTAILEQPQNETFIVSKTDIDVQVSENKDYRLYCYYPNTPTRNFELFQMELEEGHSYTS

VGHSEKSQEYIMIIEGQLKLEVNDSIYQLRENDSICFSAESIHTYHNQGEKTLKAVIINY

YPV*

>CD630_16680 Clostridioides_difficile_630_NC_009089 membrane protein

LVNWEIKKLAKSKSMFISLGILVLILMISIFITPVLETESYYIDKEKGRIEDTRSGIDVG

NEKFQNKINVLKQFSNQTDTGEFSKKIELMSKKKLDNLSVNEYKDISFWKVFNYRVTNSL

INVGMLIIISIIISNIYVDEVVTSVKDIILSSKEKKKALKSKIFVALLVPLVIYSVYLMG

IFIITCMQQGVPVNGDLESFRIVDNVVALKTNLSITKYVILNIGIELLMFEGWAMIAIFG

SFISKSSISSISFFIFIISITKIMSVIHILPKKLLSVVSNVNYYDLIFGFNKIIGNYLGD

IMIFKVNIDIVNIAIGLLMAILIGAMSLCFLVIKSDCISR*

>CD630_16720 Clostridioides_difficile_630_NC_009089 two-component sensor histidine kinase

MKWKITRNFIFTIVFVAISVVIINIISILYVISTNSFFKVVDSGNNPEEFARSFEKDLYE

KDGEFKLSKIGAEKLEKSNSWIQVLNDLGEEVYGVNVPKDTPKKYTPFQMVNNYKYIETK

YVNFVLEKHLNKKHLNIIVGIPSRDISRIILTYSQNNIKKTLNKVIIITLVIDSVVALGV

GYLFSRKLTKPISSVLWSIETMANGNYSLYLKDRGIYEEVFKNINMLADTLRVNEVERKE

NEELREEWLANITHDIKTPLASIQGYAEIINDKDYEFEEDEIQEYTEIIYNKSKYIKDLV

DDLNLSTRLKNNTIVLDKKKINLVSLVRNIIIDILNDNRYKNRNIEFESNEDLIEVYIDS

ILFRRAITNLIFNSIVHNSEGTLISVEIVKKDNIEIIIKDNGIGISKSDLKHIFKKYYRG

TNTGEMHKGSGLGMAISKEIIEIHKGKIYVSSEIGIGTKIIIEIKQN*

>CD630_16730 Clostridioides_difficile_630_NC_009089 protein TraX

MDFFKKGISGFTIKILALIFMTFDHIAAFMPQTMQIPIWFHWVGRISAPLFIFMAVEGFY

HTSNRKKYISRLYIWSVIMAIGNQIINNVFSHPEGAIIINNIFSTLFLIAIYLQAIEFIK

KFRKEKEIKYFIIGLLMIIIPIILGIFTVALLFKVTNRVIALFMILVPVPFLVEGGPIWI

ILGIIFYLCRGKKFSLSICYVLMCIFIFTTMSNGDYSLKNSILQNYQWMMIASLPLMLLY

NEEKGKSMKYLFYLYYPIHVYILYILGIYLINGF*

>CD630_16740 Clostridioides_difficile_630_NC_009089 NADPH-dependent FMN reductase

MKVLLINGSPNQYGCTYTALNEITKVLSKHDIKTEILYLGKETIPGCISCASCFETGKCI

RNDKVNELIEDLDNIDGIIIGSPVYFSSATGQLTSFLDRLFFIAGSRMATKLGASVVSCR

RGGASATFDQLNKYFSISNMPIVSSQYWNQVHGFTPEDVMKDEEGLQTMRTLGENMAWLL

KCISAGKKAGIKEPQYEETIMTNFIK*

>CD630_16750 Clostridioides_difficile_630_NC_009089 HxlR family transcriptional regulator

MSIDCISNTDLSETGFSYTLSLISGKYKMIILYCLVEFEVVRYNALKRYINTISYKTLSL

SLKELEADNLIIRTEYPQIPPKVEYSLSERGKSLIPILDAMCEWGERNRP*

>PCZ31_RS16225 Peptoclostridium_difficile_strain_Z31_NZ_CP013196 pyrrolidone-carboxylate peptidase

MKILLTGFDPFGGEPINPAQEAVERVNNNINGAEIIKITIPTVMTKSVEAIDKAIQEHNP

DIVISVGQAGGRFDITPERVAINIDDFRIKDNEGNQVIDTIIKEDGEPAYFSKLPVKAMV

KHMNENKIPASVSNTAGTFVCNHVMYGILYMIDKKYPNIRGGFIHIPYTTSQVIDKKNTP

FMSLEEIVKGLELAIEACIIYKEDVKELVVKFHNKFFSIFIN*

>CD630_16770 Clostridioides_difficile_630_NC_009089 membrane protein

MQNFINISLEIFYALMGFLMIVIAYKSFTTINNNKKYGTSLFWILISLPFIFGRLIPANI

IGIILILSSLLTLSKQVVFAKYEEPDENFGKEQADKLKNKIFIPSLILAFAAVVVAMSLS

NFDNSSQFAIGVGSIIALISALIITKAKPATSVQDGSRLLQQMGPASMLPQLLVALGALF

TQAGVGEVISTMISGVVPADSRLFGVIAYVLGMVIFTMIMGNAFAAFSVITAGIGIPFVL

SQGGNPAIIGALALTAGYCGTLLTPMAANFNIVPAALLECKNDYIVIKYQAPVALVLIIA

HILVMYFLGF*

>CD630_16780 Clostridioides_difficile_630_NC_009089 membrane protein

MIKLIGILIVVIGFILKIDTLFTVLLAGVATGIVAGLDFNQIFTILGDSFVSNRGVSLFI

LTLPVIGVLERYGLKQRAVSLIEKLKRLTTGKVLTIYMIARQIAGALSIRMSGHPQFVRP

LVNPMAQAAGLSNSDELKESDEEAIKALSAASENYGNFYGQNLFAGSSGVLLIASTLTQF

GYNVTGLNIVKASTIMAVIALVVATIQFTLYDKKLNKSHKK*

>CD630_16790 Clostridioides_difficile_630_NC_009089 flavodoxin

MNTIIIYSSKYGCTKDCANILKNKLSDNVTFVDINNNNNNKIELSKFDKIIIGSSIYVGS

VSKKIQVLCNDNVELLNKKQVGIFLCCGFSEQADKYLKSNFPSSLLESANAIGIFGSEAR

LEKMKFLDKLIMKAVSKGNYDSFRISQDNIDNFLINLNS*

>CD630_16800 Clostridioides_difficile_630_NC_009089 FMN-binding protein

MKIRNKATNKKKKYVKISIALFFVFICIIIVIVSNLKPENLVVKDIDINNVKNGIYTGSA

DNNLVKATVSVEVNNGKIQNINILKHDHLLGKPAEKITTSIIKQQSLDVDAITSATYSSN

TIRKAVENALRKGE*

>CD630_16820 Clostridioides_difficile_630_NC_009089 nucleoside hydrolase

MEKRKVIIDCDPGIDDSLAILLALNSPELEVIGITTCCGNVPANIGAENALKTLQMCSSL

NIPVYIGEEAPLKRKLVTAQDTHGEDGIGENFYQKVVGAKAKNGAVDFIINTLYNHEKVS

IIALAPLTNIAKALIKDKKAFENLDEFVSMGGAFRIHGNCSPVAEFNYWVDPHGADYVYK

NLSKKIHMVGLDVTRKIVLTPNIIEFINRLDKKMAKYITEITRFYIDFHWEQEGIIGCVI

NDPLAVAYFIDRSICKGFESYVEVVEDGIAMGQSIVDSFNFYKKNPNAIVLNEVDEKKFM

YMFLKRLFKGYEDIIDSVEGVI*

>CD630_16830 Clostridioides_difficile_630_NC_009089 membrane protein

VKRKICVKTITLIAFGISINIIGAFIAMGLRLPVYLDSIGTIMIASLLGPKYAVVTGVFG

SLISGITFDVYSLYFAPVQISTGLLAGIAFKKNFLRGLKTPLGVLLFAIPTSIISSIISA

FLFGGMTSSGSSYIVQILKVLGLGDVFSVFVTQVFTDYGDKLLAVVLVNLGLNAVPKTLK

VSLTGGK*

>CD630_16840 Clostridioides_difficile_630_NC_009089 radical SAM superfamily protein

MDRYSEITNKNQREIVLLKGFPCIWGKCSFCDYIDDNSNLEEEMNKLNLKVLKNVTGKYG

VLEVINSGSCFELPKDTLEKIKCIIKEKNIKKLFLESHWSYKNRLKEMREYFEIPVVFKI

GVETFDNDFRNNILNKNANFKTPQDVKEYFDSPCIMVGIKGQTKEMIDKDIDIILNTFEK

ATVNVFINNSSSIKRDEELVKWFISKYKFLDENPNIEVLYNNTDFGVGD*

>CD630_16850 Clostridioides_difficile_630_NC_009089 hypothetical protein

MMFDRGKWNKLDYNELLEYMMSIQDIKYRDFNKKLIPGTENIIGIRVPNLRKLSKEISQG

NWKEFLEVAEDTYYEEVRLQGMVIGNINSNFEETLYYVKRFIPKIDNWSVCDGFCSDLKS

VKKYKENMYDILKKYVYSKNPWEIRFALVMFLIYYVDDKHIKEIFEYCNNIQSEEYYVKM

GMAWLLSICFIKCEEETFLYIKNNNLDDFTYNKMLQKIIESNRVDLEKKNIIRSMKRKTR

RC*

>CD630_16870 Clostridioides_difficile_630_NC_009089 hypothetical protein

VLNMNKIVKKSISLMIILTIFIFMLTACEKDEQPDSVDIQTEDKNEIKIDEGNAKVLNIG

KSEIINIDEGDKVDTSTKVENNSTFNISNVELIYNEYDENKKIISSDSKALLDMTLMPGK

VAYIECGHKTFVKGVEVYAYEYEAEGKIVYVNLKENTINIRNNNIKLENSSQYEVLSTSE

LKKVNESNEGITYQVKVKNSSSKDLGNIILKTAEVNENGEYLTVNRVPSYKILKPSEETD

IDILCSTKAKSVEIVGYTYDDIKEKANVDIDLKSHKVKIDK*

>CD630_16880 Clostridioides_difficile_630_NC_009089 two-component response regulator

MKVLIVEDNKILLESVVEELSKHFETEKCEDGEEALYLVNQNIYDLVILDLMLPNINGFD

ILKKMRVNNIDTPVLILTAKETLDDKVEAFTIGANDYLTKPFYMEELVARVYAILRTNGK

IKERNGLEFKSLYLDTLEKRVYIEKEEIKLQNKQFNLLEYFVLNKGSILLKEQIYDRIWG

IDSDATIEIVEVYVSNLRKKLSKYGYDKYIKTKRKVGYIFDDK*

>CD630_16890 Clostridioides_difficile_630_NC_009089 two-component sensor histidine kinase

MINKNVFTSTKNHLIKMYIIVVGSFLIIFSIFIYSYFRGLTYSGIDSEINDELEYIVSQF

KRTSFLNPIRLKDPKDMVYVYEDGRISYYTQNEYFDELLPDRRLDKKNSFFKYTENGYTF

RELNVDVGRYQIQIIRNIDSEMNSLRQLTSVLIIGILISVIITYFVAVYLTRKALIPIET

AWKNQAKFIQDASHELRTPITIVSSKLESMLKSPESTVNDEVETIATAMKETRRLKKMIT

DLLSLTKEDSIVKVNLEEIDLEKLLEEISEDYIDIAEFQEKRFVFNSKLKNKVIITDKNK

LRQLILIFIDNAFKYTKLGDEIALELKEDIEDEVTLLISDTGIGIKKEEIPLIFDRFFRS

ENVRNKDLEGSGIGLSIARMISLNLSIDINVTSDVDIGTTFELSIPKKLK*

>CD630_16900 Clostridioides_difficile_630_NC_009089 thioredoxin

MAKVINTSEFRSSVEGSKGIVVVDFFATWCGPCNMLGPVFAELGEEMKDKARFVKVDIDE

SLEIAQQFNVSTVPTMIIFKDGKPVETLIGFMPKNKIEMQVKSYL*

>CD630_16910 Clostridioides_difficile_630_NC_009089 thioredoxin-disulfide reductase

VSIMRYDIAIIGSGPAGLSAAINAKIRNKTIIMFGNDNLSNKLVKAPSIDNYLGFYDISG

DELKDKFKSHIDSMDISIENKRINNIYAMGEYFTIMSGNDMYEATTVILATGVEYTRPIK

GEEEFLGRGVGYCATCDAPLYRNKKVAVIGYNEESKEEANFLSELTSKTYFIPMYKKDNL

MRSSDNLDDSIEVIHDRPVQIDGDKLVNKVSFKENHIEVDGVFVIKDSTAPSALVPGIEI

DGIHIKVDNNMKTSIDGCFAAGDCVGKPYSYIKAAGQGQIAALNAVYYLDKLKRA*

>CD630_16920 Clostridioides_difficile_630_NC_009089 ArsR family transcriptional regulator

MENLVKIFKALSDETRLNILILVSKRNICQKGISKYLGISDSAVSQHIKILKDVGIITGY

KEGYYVLYHINKESFNICVDFINSMLSNSSESFIDVFDVNTIHLGCSKECKSIKKCCKRR

EK*

>CDIF1296T_01778 Clostridioides_difficile_ATCC_9689__DSM_1296_strain_DSM1296_CP011968 dinitrogenase iron-molybdenum cofactor

MMKVCIPVEENKGLDSKPYGHFGSAPIFVVCDLESGEVKSLDNGDLDHEHGKCQPLKALS

GTAVDAVVVGGIGQGAIIKLNGMGIKVYRAEGDTISANLDLLKNGKLVEFPSDHTCSHDG

CGHH*

>CD630_16940 Clostridioides_difficile_630_NC_009089 hypothetical protein

MNINTVQGDSIEVLLRQLGATRISKVSSTLYFIKFDLGDGWEISYTYNINAKDQYFLQRI

EPYPIGRGLFNDEYEIVSFISKDLKKFLNAKNSSNFKTFVEVTRKVNSIIDNVEELFLNY

NVDGDDLKTLNKELNDILNDIDYIEKHTKKI*

>CD630_16950 Clostridioides_difficile_630_NC_009089 symporter protein

MEFQQIIAICIFLIVMAAIITEKVNRSVAAVGGALLMIIFNILTLDEGLSHIDFNTIGVL

VGMMLFVAVVKNSGLFEYIAIWTAKKAKGDPWKIMICFAIITAILSAVLDNVTTVLLIGP

MTIVITQILGLNPVPFLITQILASNIGGTATLIGDPPNIMIGSAANLSFMDFVINLGPAV

IVILAITIICFRFIYGKELVVNERAKNAILKLDEKKSVKDKPLLIKSLILIAFILFGFMF

HSTIHIDSSVVALTGASIMLLIGKQDVDEIMAGIEWSTILFFMGLFVVVGGLVEVGIINK

LAQALIGLTEGHLVFTMLLILWLSAIVSSFLDNIPFVATLIPLILTMQAEGIDVMPLWWA

TSLGACLGGNGTLIGASANVVLAGIGNKHGHPISFKEYFKIGFPLMIISIIISTVYLIIK

F*

>CD630_16970 Clostridioides_difficile_630_NC_009089 6,7-dimethyl-8-ribityllumazine synthase

MIYEGKLIGKDLKIGIINSRFNEFITSKLLSGAEDCLLRHDVSPENIEIVWVPGAFEIPL

VAQKMAKSGKYDAIICLGCVIRGATSHYDYVCSEVSKGIAKVSLDSELPVIFGIVTTENI

EQAIERAGTKAGNKGYDCAMNALEMANLFKSLN*

>EAA_RS2000000220080 Clostridioides_difficile_CIP_107932_NZ_CM000659 bifunctional 3,4-dihydroxy-2-butanone-4-phosphate synthase/GTP cyclohydrolase II

MFTGIVEEVGILRKITANGKSGKVTILSNKILDGTNLGDSIAVNGVCLTVSNLGKNEFTA

DVMMETIRSTNLGLLNANDKVNLERAMSLSSRFGGHIVTGHVDGKGTICKFEKDENAVLV

SIRPDKKLLSSMILKGSVAIDGVSLTISYLDDEIFKVSIIPHTKINTILLTKNVGDFVNL

ESDVIGKYVNNFMANNYKELNSNSSNHKSNIDKDFLFKNGFKNNKGVNYMFNTIEEAIED

IKNGKMVIVVDDESRENEGDLLMAAEMATPESINFMATYGRGLICLPATEKKFKSLNIPL

MVRENTDTFQTAFTVTIDGADTLTGISAYERAETVKLFCDENSTSKDFKTPGHIFPLIAK

TGGVLVRDGHTEASVDLARLAGFKEIGLICEIMKDDGTMARVDDLMIFKEKHNLKIITIK

DLIEYRKINETTIEKVSSAFLPTKYGNFEIIGYRDTYSNEEHIALTYGNINIENTLVRLH

SECLTGDVFHSLKCDCGLQLESSMKKIVENGSGVLIYMKQEGRGIGLLNKIKAYKLQEEG

YDTVEANLMLGFEEDMRDFYMAAQILKNLNIKSINLLSNNPDKINQLEKYGIKIENRIPI

NEEINDFNKLYLKTKKDKMGHLLDII*

>CD630_17000 Clostridioides_difficile_630_NC_009089 riboflavin biosynthesis bifunctional diaminohydroxyphosphoribosylaminopyrimidine deaminase /5-amino-6-(5-phosphoribosylamino)uracil reductase

VNFVNQKEKDIYYMKKAIELAKNGEGFVNPNPLVGCVIVKDSNIIGKGYHEKFGSNHAEV

NAINSAKQSLKDSTLYVNLEPCSHYGKTPPCVDKIIQNKIKRVVISTLDPNPLVCGNGVK

KLKDNNIDVTVGILEDEARDLNEAFFYHIKNKRPLCIVKSAVSLDGKIATKSLESKWISN

ESSRYLTHKYRNKYQSIMVGINTVLNDNPLLTCRLNQEKVSHPTRIVIDTHLKLPLNSNL

VKDKTSKTIVFTCCKESIKLSMLKENNVETIISPSKNNLVDLEFVMYKLGELNIDSVLVE

GGATLNDSLFRNKLVDKVKLFLSPKIIGGKDAPTFVSGEGINHLSDSTQLTINNVTLIDG

DILIESDVLN*

>CDIF1296T_01789 Clostridioides_difficile_ATCC_9689__DSM_1296_strain_DSM1296_CP011968 thiamine biosynthesis protein ThiC

MLGEICLSVYTIIRVLSKRGKALFLFSKTITLILEDCNMNYTTQMDAARKGIITKEMEIV

SQKEQVDVNELRELIANGQVVIPANKNHKSLSAEGVGKNLRTKINVNLGISRDCKDIEKE

LEKVRVAIDMKAEAIMDLSNYGKTREFREKVVEMSPAMIGSVPMYDAVGYLEKELKDITE

EEFLNVIRQHAIDGVDFITIHAGLTRSVCQKIKNHERLTHIVSRGGSLLFAWMELNNKEN

PIYTNFDKILDICEEYDVTLSLGDACRPGCIKDSTDGVQIQELVVLGELTKRAWERNVQV

MIEGPGHMAIDEIEANVVLEKRLCHGAPFYVLGPLVTDIAPGYDHITSAIGGALACAKGV

DFLCYVTPAEHLRLPNLDDMKEGIIAAKIAAHAGDIAKNVKGAREWDNKMSKARADLDWC

EMFRLAIDPEKAKRYRDESTPTHEDSCTMCGKMCSMRTVKKILNNEELNLI*

>CD630_17021 Clostridioides_difficile_630_NC_009089 thiamine biosynthesis protein ThiS

VKVNGKEIEFEKDLTVIDLLNKYNLKSDRVVVEVNLEIIEESNYNTYVLKDEDIVELISF

IGGG*

>CD630_17040 Clostridioides_difficile_630_NC_009089 thiazole synthase

MDKLVLGGHEFNSRLLVGTGKYGSNNILPEVIKESGSEIITMALRRVDLDNKQENILTYI

PKEMTILPNTSGATNAEEAVRIARISRKMGCGDFIKIEVISDTRYLLPDNEETIKATKIL

ADEGFIVLPYMTPDLYAGRRLIEANAAAVMPLGAPIGSNRGLQMKEMIRIMIDELDIPII

VDAGIGKPSQAMEAMEMGADAVLVNTAIASAGDPVQMARAFKLAVEGGREAYIAKTGNVS

EFANASSPLTGFLGNL*

>CD630_17050 Clostridioides_difficile_630_NC_009089 thiamine biosynthesis protein ThiH

MSFYDVIEKYRDFDFDGYLNNVTDNDVLRSLSKDKLEDFDILNLLSKTAVKHLEDMAQKA

HKLSVQYFGKTVCLYTPMYIANYCVNQCVYCSYNIKSGIKRKKLTMDEIREEGEAISKEG

FKHLLVLTGESSFHSSVEYIGEAIEILREKFPSIGIEVYPMEVEEYKYIVDKGVEGLTVY

QETYDEEIYKRVHIKGPKSNYKFRLDAPERGAKAGMRTLSIGALLGLNDFRKETFFTILH

GKYLKTKYPHIELSYSTPRMRPFKGCFEELVDISDTDLVQAMVCMRLFDPHAAINISTRE

NLEMRSHIIPLGVTKLSAGVSTDVGGHSQDEHDTAQFKINDESTVKDVEKMLNSIGYQHV

FKDWERF*

>CD630_17060 Clostridioides_difficile_630_NC_009089 thiamine-phosphate pyrophosphorylase ThiE2

MYLITNRKLCSEERYLEVIKESILSGVENIIIREKDLEYQELRKLYMKIKTKINCIDFQE

QISDESLKTNINQKECRNKFKVNFIINSNIEFFEKVDCQGIHLPFKLFLNLIENKYNFNE

NKILGLSLHKVEEVDYLEKLIRNQNIKIDYITLSHIYETKCKEGLNPKGIELLKEAKKIT

DIKIIALGGILPSNVKETLKYCDDFAIMSTIMRSKDIKKTISNYNEKLN*

>CD630_17080 Clostridioides_difficile_630_NC_009089 hypothetical protein

MGLKEFDFIEESIDMLRTMSPTLETISDEIEEYFENILDEKNQEYINVTSRIKSESSLRE

KIIRNRYLKKYGEASNLIHNVSDLIGLRIECRFIEDENKIYRLLRRYFNKTDDKINYYNK

ENKNIKLKLSERQPNKQKNGFEIYKIDGVFAYLDREVKFELQIKSLVNVFWSEIEHKIIY

KNNTYLLADKFIKDMMDSIKNNLTMIDNQLLSIYKNFHSGKSFNMKVSKKEIEKLFAKLV

YDAFSEKMNKSIGFVVDFKKPCETILNYSFNKQDISDEVLGNFMLDEFARLNEIVNKDID

FNEQIEFEREPTFDDKFCKDLGNHFRSRLNTEFPWNLFFRILFEIEPYNNTKDFENFVEF

IKENVLLEENREQLLCQFEEDSKLIIEDIYDCIFNSISEIDSVEILYNYNLEKINFTSSE

IINCICREYECYGEYLEEKEKLMNTFKEKIIQIFE*

>CDIF1296T_01797 Clostridioides_difficile_ATCC_9689__DSM_1296_strain_DSM1296_CP011968 molybdopterin-guanine biosynthesis protein

VALMRHFSNIYELSKIILEEAICIEKTGVILAGGRNSRMGRDKAFLELHDKLFIEIAIEA

FKNFDELIIISNNEELYSKYDIKVYNDIVKDVGPIGGIYTALEYAKYDIVTIACDMPYLN

NQIVERIANKMNDKSVISVTNGKLQPLCSGYKKSIINKVSLCIRENDLKLRSFIDKIDKS

YIYFDEEDLFLNVNTVDEYEKLTKV*

>CD630_17100 Clostridioides_difficile_630_NC_009089 NAD(P)-binding protein

MNLLKCDSWAVILNNSDKESKAYKILDELKRNMYKVVAIDEEKKPIEGIDVYECLKDVPH

NIDVVAIIDKQSKMDVILEEVELLDIQNMWFEKGSFSEMMIKKTKDLKLNIEYNLSLYDE

LTR*

>CD630_17110 Clostridioides_difficile_630_NC_009089 hypothetical protein

MLIVTTEKVEGKKISKVLGLVRGSTIRAKHVGKDIGASFKNLVGGELTGYNEMLTEARQI

AIGRMVEDAEAKGANAVIAFRLSSASVMQGAAEMLAYGTAVVLEDDNSILEK*

>CD630_17120 Clostridioides_difficile_630_NC_009089 molybdenum cofactor biosynthesis protein MoaB

MFNVAIITLSDKGYEGKREDITGKKLTEFVENTGAYKVTEYVLIKDDKEMLKENIIRLCN

SNKIDLILTNGGTGFSKRDITPEATKEVLEKEIPGLSEYMRMKSTEITKKAILSRGVSGI

RNNSIIINLPGSPKGAVENLSFIIDVLDHGIEVLRGEATECATKKSE*

>CD630_17160 Clostridioides_difficile_630_NC_009089 permease

MEEEKINYALEQIPENQKRGWVAMFSVLVAIGVDLSSVILGAELAQSMPMKQAILSVIVG

SFFSAILYTTCSLVGSSTSLSTSMITKYVFGEAGAKIFSLVIGVSLLGWFGVQVGFFAQN

AQIIIKDIFNLDVSMQILSLIGGLLMMSTAIYGYKAMEKLSVYSVPFLLVLMMLTIFLAF

RANGISVDDNMKSTMTFAGGVSLSMSIIIVGAIVSPDISRWAKSRRDCALSSFLGIQFGN

AFMIIVSIVLVKCMGTSDIMRIFITLGIAIPGIIVLTLAQWTTNTSNVYSASLSIALVLK

KAPEKVLTIVLGIIATLLAVFGIYEGFIGFLNLLGIVIAPVGGVYTAEYYIVKQELKGFD

KGVLYKPIVKRSIVSWIIGILITYLSTYGFITLTTIAPLDGFIAGFVVQSIIGKVLCSTK

NKQEIDKAV*

>CD630_17170 Clostridioides_difficile_630_NC_009089 hypothetical protein

MRFLTEESVDKIAVGAAVLGTGGGGDPYVGKLVAKQAIKKYGPVKVISLDELDDDALVVP

VSGMGSPVITIEKLLSEVELTTPLEIMEKLLNRKVDVIIPIEIGGINSLMPIAVAAKKGL

PILDADSMGRAFPEAQMVTFYLEGYEASPAVMSDEKGNSAILYPVDGIWSERLARTLTVE

MGGSSSISDYNLSGAQVKKAAIGNTLTIAETIGGFLLENKSDSKGAVENILKELRGYKLF

EGKVIDIKRELKGGFTRGHAFFAGINEYDGEYSILFQNENLIAKKGDTPLCITPDLIAVL

DLETGFPITTERIKYGSRVMVVAFPCNEKWRTEKGIETVGPGYFGYDVEYKTVEELQGK*

>CD630_17210 Clostridioides_difficile_630_NC_009089 hypothetical protein

MKSYIALLRGINISGKNKIIMSELKASFVELGYSEVSTYLNSGNVVFLSDVDDSKVISNT

IRLMIKKQFELEIPVFIISQEELKDILNNAPNWWGDNNKEIYDNLIFMFPDLSYGEFYDE

VGNPKEEYEKVYHYKNAIFWSFSRKDYKKTNWWSKTASSNVSDKITIRTANTVRKIVGMR

*

>CD630_17220 Clostridioides_difficile_630_NC_009089 transcriptional regulator

VAKNTKQTTVHIDETLLKEVKRIGINENKSISQIINESIIDYILVYRENEDKEKNEHDKN

LNETINEFKKEEEKRMQAKSAARAIAEAKKRVLY*

>CD630_17230 Clostridioides_difficile_630_NC_009089 acyltransferase

MNNYLKFATYQLIGFTSSIPRLIKIKKNPDKFSLKEKFEFMQKQAKKSLDIVNIELNIIG

KETLPKEPLLFVVNHSSMLDSFILTASVERPIGCVIADEPVWRNIPIFKEWAKLLRCVYV

NRKNNREGIKSIAQASQNILTGQSMAVFPEGDLTWIKEPNSLVSEFRSGALKIAYKAKCP

IVPLVIKNSKDTYEGYQPIGKINSVPVEVEFLEPIYDHIENPRLKSSVLGENIKNKMINT

IENFRKSNKTFKEF*

>CD630_17240 Clostridioides_difficile_630_NC_009089 hypothetical protein

MIESRCGILCYECGYKEKVNCKGCTEIEKPFWGEQCPVKSCCEDKNLTHCGLCDTFPCEM

LNQFAYDKEQGDDGKRISQCKKWAVLAL*

>CD630_17250 Clostridioides_difficile_630_NC_009089 AraC family transcriptional regulator

MNKIINNILNTCKQYTYILPHKLLRQYIAHYTISIPDTSIKENLTLIPDASGCMIFKFDK

KGIESAFWGATTKTTIVKNDIENVLFRVFVEFRPGGVYYLTGLSQRESTDLKISLEDFNT

LFSLEVNSIFERTSTIKELVEQLDMLFLSYLLKSNIVDMTIPILENARKQNSIMSVKNIS

QISCYSERHLNRIFNNSLGMSVKSYLRLLRINLVLQEIQNNKIPFATLAQDIGYYDQSHF

INDFKSICGVNPTTYIKNLSDFYNEKYKF*

>CD630_17260 Clostridioides_difficile_630_NC_009089 hypothetical protein

MASKQIPRNNLEKFLRIRVLSHTGDKIHIKLPVNFAKRMIENNALDLFNGKDDVVDGKKV

TEILVKAFDYDLTGEVVHLERKNGDIIKVSIVQ*

>CD630_17270 Clostridioides_difficile_630_NC_009089 hypothetical protein

MRGITMENKDFKLTICIFAFVIGVVVLFCCIGLGEFSAEHIIQQNGGIMDTDQYNIYLDQ

SISQYRNFGSILALLGGIGILFDKYIRKY*

>CD630_17280 Clostridioides_difficile_630_NC_009089 hypothetical protein

MATEIFTVKITPLEAQKMIKENLDADLVFSDSYNLGDDKFILITTFEKYYARNNSDAGLI

VVYENTTGKTVIKATSTGSAIGILKIDWGAGGNLLKRVKNILTDYII*

>CD630_17281 Clostridioides_difficile_630_NC_009089 hypothetical protein

MINYMVEIIYSEKISEVENMLKIENIQYEFTSRDGFIFFGFYDMTDAYRFETLCSDEKIR

FKRI*

>CD630_17292 Clostridioides_difficile_630_NC_009089 hypothetical protein

MERLMELIERKFLKDVELLELEDIENVIECKMTQPYNAHYNEYLVKVKNGDEVEEYFVYL

KHR*

>CD630_17300 Clostridioides_difficile_630_NC_009089 ATP-binding protein

MADCNSCPSKGNCNSQSNCSIENNPNNKFGKIIGVMSGKGGVGKSTVTALLANKLNKMGY

KVGILDSDITGPSIPRLMGVKNVKAYSDGSYIYPVENSNNIKVMSINLMIDDENEPVVWR

GPLLGGVVKQFYTDVLWEELDYLLIDMPPGTGDVALTVMQSIPISGIVMVSVPQDLVSMI

VSKAVNMAKKMNINVLGVIENMSYIQCPDCSKKIKLFEGESTEKFLDDLDLELLGELPMT

KEIIDITHNGVTEISDDLDSILTNVVEKIK*

>CD630_17310 Clostridioides_difficile_630_NC_009089 acetyltransferase

MELRPRSAKLEEFDKVIELINYVFRISRSHKPTMMEEFPLLLSKNNIENMIIISEDDKVV

SDVNYLIQDVSIQGNRLKVAAIGGVCTHPDYEKRGYSSKILDKVEEKMFYDGVDIVIISG

TRSLYSRRNCSLVKSFYKYTIKPEDVKIAYEIVEFDETNFEKDNDLDKMIELYNQNSTRF

IRTRDEFQKLLHAATIAWGPIGYKKVFIKENNNIIGYLIIRTIKKEDSTVGEVAEIGLNS

VNVENILKYVANKFGLEYLNYKVHVKNLKDQLKCNGTKSLDYQQGTMKIINFTKLCDSLR

SYFSQYVDFELLKYMEFKQVENKYIIKYKEEELVIENLDKLNKLFFEKNEEQYNEFKHLK

NIYEFATKAFPVDFPWTANLNYQ*

>CD630_17320 Clostridioides_difficile_630_NC_009089 cobalamin-binding protein

MDILDDIAECILNMGVDNIENLVKIAIDKNIDVEDIYEYGLNKGMIGALDKFENKEYYLS

EVIVCTDALNKGINLLKGTGKVKKKSKGVILMSVVEGDTHEIGKNIVKVMVEATGYKVID

LGVNRKSEDIIEEAIKNNVDIIGLSSMMTTTMENMKSVIDELNMIEIDKRPKVIIGGGPV

SMEFAEEIGADGYSSNAPKAVKLINKLIGGEV*

>CD630_17340 Clostridioides_difficile_630_NC_009089 hypothetical protein

MNYNSNKNKYLLKDINLDDIFIDKNIVLKFLGYGNRKAPDTISKIVEREIRNINDILDIK

IYISEVDINKTPKECKKAFAVLYTIGDKIDFKMNDYMENCNMMAGLALDKIGIVCLDYIN

EKIKMYLKGKYDSLKISHEIYPGDKDFEVERQKDIYNYVKNKYNSIEIEINDYHQLSPIK

SVAMLILMGDEENLESRCSKCPRKCF*

>CD630_17360 Clostridioides_difficile_630_NC_009089 HTH-type transcriptional regulator

MNDLNLGEKIAEVRKKQNLSIRDLAKLADVTPSLLSQIERGLANPSVNSLKSIASSLNVP

LFTFFVSEVDKKNLIVRHDNRKKVILPGSKEVIYEILIPDSSGNLEFAIMDLAPNTSSCV

DRITHNGDEIAYVLEGEVKLFMDDDEFTLSKGDSVKVPLGTKHKWQNDSNSESKVIFAVI

L*

>CD630_17370 Clostridioides_difficile_630_NC_009089 divalent ion symporter

MQITTFGAIFGLLIAIILIIKKFQAVYSLMLGAFIGGLVGGANITQTVDFMANGAMNISP

SILRALASGVLAGSLIKTGAVDKISEQIVKIFGEKRALFSIAISTMVLAGVGVNLDVSII

TVAPIGLYIGRKLNYSKLSILLAMLGGGKAGNIISPNPNTIAVADNFSVNLSSVMMANII

PAIIGVVITVILASILINKGNKVQSYEILEQREDLPSLFKSLCGPIIAIFLLFLGNVSPI

VIDPMIALPIGGIVTLIVTGNLNNSREYLAFGLSKMQGVCILLLGTGTIAGIIQMSELQQ

STIGALQFLNMPQFLLAPVSGILMSLATASSTAGATIASSTFHDAIINGGLSPISGASIV

NAGSSVFEQLPHGSLFHTSSGSINMDIGERFKLIPYEALIGIVMTIISTSIQLVL*

>CD630_17400 Clostridioides_difficile_630_NC_009089 glycine/sarcosine/betaine reductase complex component B subunits alpha and beta

MKLELGNIFIKNVEFGEKTEVKDGVLYISSEEIEKIVLEDERIVSVNVELARPGESIRIA

PVKDVIEPRVKIGDESKIFPGIINKVKTVGSGRTHVLLGACVVTCGNIVGFQEGVIDMSG

PTAKYTPFSKTNNICIVIKAKDGIDTHDYEEAARIAGLKIGAYVGEAGREVEPDEVIVYE

TKPLLKQVKEYPDLPTVAYVHMLQSQGLLHDTYYYGVDAKQIVPTFMYPTEIMDGAIISG

NCVAPCDKVTTFHHLNNPVIHDLYKRHGKDLNFIGVILTNENVFLVDKERSSDMVAKLIE

FLGVDGVLVTEEGYGNPDTDLMMNCRKCSEVGANVVLITDEFPGKDGKSQSIADATKEAD

AVVSCGQGNLVVHFPAMEKIIGTLDYVEMMIGGYKGCLNEDGSMDAELQIIIASTIANGY

NHLTARYY*

>CD630_17451 Clostridioides_difficile_630_NC_009089 ferrous iron transport protein

MTVYNLKLGQKGIIDNIAGNEKLMKRLLALGLIDGTEVEVKKIAPLNDPIVIRFRGFDLA

IRKSDAKNINLKNN*

>CD630_17460 Clostridioides_difficile_630_NC_009089 sodium/glutamate symporter

LITLSFDIVQTLTLSIFFFLIGNLLKNKVNFLNNFCIPAPVIGGLLFCFLNLFFKYFNIA

DISISGNLMPNFITFFFTTIGLEISINLIKKGGSVLFRYWILCGVLAFCQNILAITISKI

IKLEPLLGLMCGNVSMEGGHGYSAAFGLTIENLGIEGAVGVGLSAATIGLIMGGILGCPV

AKFLINKYKLKPSSNIDLSIPRYNRNLNRFGSKFFRNKNRIKNNNFSKTITPSVFLEQVL

LIFICINTGEIISRCFYITFNILLPSVVTCMFSAVIFRNLNDKINILELNFKLIDFLKEL

SLGIFLTLSLMNIDLFELSTLLPPILLIVTFQVIFIILFSIFICFRVLGKDFDSAIIISG

LIGHGIGATPNALANMSSLTQKYGDSPKAFLVVPLVSGFLLDAISIPCILFFINILT*

>CD630_17490 Clostridioides_difficile_630_NC_009089 2-hydroxyacyl-CoA dehydratase

MTNLPKQFDSFNEARQKGFINAKELKESGKKMVGVFCTFTPVEIPMAAGATVVGVCGVSE

EPIPDAERVLPRNLCPLIKSSYGHAITDTCPYFYFSDLLIGETTCDGKKKMYEELAKVKP

TYVMHLPNTSKGEFAYKLWKDEMIRLKEEVEKSLGVTITEEDIRTAIKDKNEERELLKEF

YALGKLQPSALTGLELHNVLYQAGFKFDRAELKHSLRKVIDDMKERYEKGECPVQKDKPR

ILITGSPIGGISEKIVKTLEDAGASVVAYELCGAIRSNDLLVDEEIEDVYDALTQKYINI

GCSCMMNNDNRIELLDRIIDEYNVDAVIDVVLQACHTFNIESYRIREFVTKEKNKPFMSL

ETDYSKSDTEQLRTRFEAFVEML*

>CD630_17500 Clostridioides_difficile_630_NC_009089 CoA enzyme activase

MFSIGVDSGSVATKGVLFDGEKIIKKIIIPTGWSPKSTSKQVYELLSSEIDKKDIKKVVG

TGYGRGVMDFADKKVTEITCHTRGIYFLNKNIRTILDVGGQDSKVINLDRDGNVFNFIMN

DKCAAGTGRFLEITSNLLGSDIESIDTLAKGYEPVNISSMCTVFAESEIVSLLAQNISTG

EVAAGILKSIANKSTSMLARGEVIDEVAFTGGLAKSKELVKMIEEILGKKIFIAEDTQII

GALGAAVIGFR*

>CD630_17511 Clostridioides_difficile_630_NC_009089 hypothetical protein

MATTTKNRFYDYVQENLNKYVEVHSYFSSCPLYGEIIEADSLSITLCSRYIDESNPTLNE

TYTLYLPLSSIISIRTL*

>CD630_17520 Clostridioides_difficile_630_NC_009089 TetR family transcriptional regulator

MPKSYSDKEREYIIKRLKEEARLCMEQYGIRKTTVDELVKRVKIPKGTFYLFFQSKELLF

FEVLRDIHDSIQKEILYEINRVDESITCEQLTDIFMKFYRMVDSTSILNLMINGEFEILV

RKLPDSIIEEHFRHDDFEIGEIISCIPNAKNKDIESFSGAFRAVFLTMLYKREVGSNCFE

DALRLMINGLVIQLME*

>CD630_17530 Clostridioides_difficile_630_NC_009089 multidrug family ABC transporter ATP-binding protein

MIKVDDLSFSYTDRDFLQNINFEVGKGEILGFLGPSGAGKSTLQKILIGMITNYGGSVIV

NGVESKRHSNKFYENIGVDFEFPSLYEKLTAIENLKYFGSLYSKKLLSIDELLKSVGLEN

ESNKRVSEYSKGMKSRLNFIKALLHNPDILFLDEPTSGLDPSNSKVMKDIILSEKSKGKT

IILTTHNMLDATELCDRVAFIVNGKISALDTPHNLIMSKGAIKVRYTYFDNGEKTSECFL

NNTANDKNLNMLIEKNKLLSIHSSEPTLNDIFIEITGRNLQ*

>CD630_17540 Clostridioides_difficile_630_NC_009089 multidrug family ABC transporter permease

MRLKNLILGDIKFQFKYGFYFLYLFLSIIYICIINVFPTFMREKIAIIMIYSDPAAMGLF

FMGAIVLLEKSQRVLNSLAVSPVKVSEYILSKVISLGVISSIVAMFIAITLNLDNIIIST

IGTFFSSIIFSLLGLIIASKASSLNQFIVLSIPIEIICFIPPILNVLLDTKSYANLYPFN

ICISLISGDKNFIMINILILISIIIIIYFITYYFICSSWKKVGGVKL*

>CD630_17550 Clostridioides_difficile_630_NC_009089 multidrug family ABC transporter permease

MIKIINAFKQEFEQIKRDAMLFIVCVSPILCGVFIKFGIPLIQNISLNKFYYQLNLEPYF

LMFDLLLAFITPFMFFFASTMVILGEIDDSISRYLIVTPLGKTGYLISRFGIPGILAFII

TMILLIFFSLTKISLLLNLSISLLVLLQGVIISILVISLSSNKLEGMVITKFSGVFMMGI

LAPFFILNKAQYILFFLPTFWISKAFKEDNYVYMFISIVISLIWILLLFKKFSKKIVK*

>EAA_RS0218165 Clostridioides_difficile_CIP_107932_NZ_CM000659 hypothetical protein

MKRILSSTIQVFKQIKSDPMMFAACFTPFVMGALIKFGIPFLDGITDFSLQTYYPIFDLL

LSIMAPVLLCFAFAMITLEEIDDKVSRYFSITPLGKSGYLFTRLGVPSIISAVIAFIVLL

LFSLEKLSIGMTICLALLGSVQAIIVSLMIITLSSNKLEGMAVTKLAALTLLGIPAPFFI

DSYYQFAVGFLPSFWVAKAMQNEAVLYFSIGLMVALVWYYFLIKRLFRKLAG*

>CD630_17551 Clostridioides_difficile_630_NC_009089 HTH-type transcriptional regulator

MKNKIKILREKLGLTQEQLGRLVGTSRQAINAIETGKNEPSIWLAYDISRIFNEPIESVF

LFEESERKSRAQISRGEYYGYKKDKNI*

>CD630_17570 Clostridioides_difficile_630_NC_009089 hypothetical protein

MRLWHKDLIDVLPKNQLVSQWRELLAIKGSIDKKGTPNHLLVNKVLNYSIDEFKFYTKIV

HDEMLKRNYKPNELKYTSILKWKNRNFANDISNEHSLNLENLYDDWHNKMYLKQCLYNLE

EKATCGGIPINEWNILLCKYSKDYELWSGNIMF*

>CD630_17600 Clostridioides_difficile_630_NC_009089 iron-sulfur protein

MIGIYFSGTGNSRYCVEKFLQEYDITANSYSIEDNELLQHINNHENIIFSYPVQYSNVPK

ILKDFIINHSTLWNGKRVFIIATMALFSGDGSGVLARILKKYGAITVGGLHVKMPDSIGD

EKVLKHSLEYNRKLVIKAEKKVTKAAKKLKHGSPPKEGLGFLSHIVGLFGQRLYFINKTN

MYTDKLKINTEECIVCGKCVNLCPMKNLVIKNCMIVANGQCTMCYRCVNNCAKKAITLLG

KKVVEQSNIKKYM*

>CD630_17620 Clostridioides_difficile_630_NC_009089 hypothetical protein

MDKISGKLTVLFEEPFWVGIFERQVGKKYEACRVVFGAEPKEVEVYEFILERFFSLDFGS

IKLEKNVTKDNIGYKRMQRKVKKEQEKETIGTKAQNALKLQYEERKQDRKNLAKNRKEEE

KERLFNLKQEKRKAKHKGH*

>CD630_17630 Clostridioides_difficile_630_NC_009089 membrane protein

VKNFILIYFIVINSIAFFSMYIDKKRAIRNEWRIKEATLMSIAVIGGSIGSMIGMYSFRH

KTKHIKFTFGIPFILFLQFLLVYFYILK*

>CD630_17640 Clostridioides_difficile_630_NC_009089 phenylalanine--tRNA ligase subunit beta

MKFIVEKEVFDKLENVCFGVVVAKGIDNTKEIERISNLLDISIDRVEDYFKDKKVKESEE

IIPYREAFRSLGMNPNKFMSSIEAMTTRVAKNKKLPHINPIVDLGNSISLKYLLPMGAHD

MDFRNDDVYVRFSKKGDKFVPFGETDVELMEEGELIYSVGDMVKTRRWIWRQGEEGKITN

SSKNIFFPIDGFTDANLDKVMSAREELAKLLKEIFNCEIKVGFVDKDNPEMEI*

>CD630_17700 Clostridioides_difficile_630_NC_009089 methylated-DNA-[protein]-cysteine S-methyltransferase 2

MSLVYDVYKSKLGNLYILSEDGFIVSIYIGDEKFNNLKNECIKKGIDIKRSKEDLKEAIS

QIDDYFNGKRKVFNLNIRIEGTEFQKSVYREMLKIPYGETLCYSDIARNINNPKSVRAIG

QASKSNKIPIIIPCHRVVGKKDIGGYMGNHSDLKEILLNLERQS*

>CD630_17710 Clostridioides_difficile_630_NC_009089 monooxygenase

MCDKVIASKYHEKGFNCAESVIKAYNEEFNTDIPVCLGSGLGSGCGVASLCGAVNASNII

IGYVKGRNHEDESTKAKVYAKDLTTTVRKEYGSELCIDLKKDLVACREIMDFAYDSLKET

LKKEL*

>CD630_17720 Clostridioides_difficile_630_NC_009089 hypothetical protein

LKNLNLLINKLYSKNHNEAYKTFLFLENESLKSNITYCFFDSFLEMIDNENSYIRTRGLL

LISANAQWDIDNKIEINIDSILSHIVDKKPSVSRMFIKSIPNITKYKENLINRIKMELSN

ADISIYNNNMKPLVEKDINDTLSNIS*

>CD630_17730 Clostridioides_difficile_630_NC_009089 hypothetical protein

MKEIADAITSEYLSYGINEDISIYEGRFCIYLDKKYRCNGKIYYKMTPPISISFKADIGC

IEEIDNEDDNLALDYDNAILEVHGYKIISITINTLSEFSVEGYINDDCIKSKNSYVEYVD

FNIINLDKIPGKLIKYNDKVYAGRIEFDINDYVVTIDKRYDYRKELKSELKSKSGAIITH

IGRIRRKDGRIFRTNNTINLLDRISTALSFMCGRYVGFCLAKGYRSGNEVYRIWNENQIS

PFRYVPTWSDTLSNYHNMEKYISLMCKKLEDFYYGSAIKSVVDWYIESLGSATMENNIIS

VQIALETLSYVILVEQNKILTDEVFDCNLASKNIRLLLDTCKIPYGKHELNIFDNIIKNK

FDDGVDLVIYLRNSIVHPSRKTHRAVLEVEDIWNIISIGTRYIELVLLFILGYRGEYSNR

LVERCYGEVEVVPWN*

>CD630_17740 Clostridioides_difficile_630_NC_009089 amino acid family ABC transporter substrate-binding protein

MKNILKKVGIFTIMLGLLGGVVGCSKPDNEKDKDASKESKKEVVVGFDNTFVPMGFLDEK

GDTVGFDVDLAKETFKRLGMEVKFQPIDWSMKETELNDSKTVDVLWNGYSITDERKKIVS

YTEPYLQNKQIIVTLSDSKINSKADLKDKEVGTQQGSTALDAVEKDKDFMNSLKGGAPVL

YDTYDKALRDLEIGRTSAVVGDEVLIRYYMGQKGEDKYKVLKDDFGLEDYVVATSKENPE

LCEKINETLKEMKKDGTFDKIYDKWFK*

>CD630_17750 Clostridioides_difficile_630_NC_009089 amino acid family ABC transporter permease

MLQGLEIVIAMFCITLIVSIPLGIGVAFLRLSKNKLVSGITQCYILIMRGTPLLLQMIVI

FYGLPLLGIVFDRFTAGVVAFFLNYAAYFAEIFRGGIQSIDRGQYEASKVLGFDKFTMYK

RVIFPQVFKRILAPISNEVITLVKDTSLVYILGLNDILRISQIAMNREASLLPLFEAGAI

YLIFVAILTKGFELLEKKYSYYR*

>CD630_17760 Clostridioides_difficile_630_NC_009089 amino acid family ABC transporter ATP-binding protein

MLKIKNLNKSFKKNRVLKDISFELEEGQIGVLLGKSGAGKTTILRCINGLEEFDSGEIII

DNEVIKNKRDMAKIRGKIGMVFQNFNLFPHMTVLENIIESPVNVFKVPRKEAEERARELL

RLVDLEDKLNSYPFELSGGQQQRVAIARSCALMPKVLCFDEPTSALDIDTIQRVVNIMNR

LKDKGMTILIITHDVVFSNNVADKIISIKDGIVENVQIKEKIV*

>CD630_17770 Clostridioides_difficile_630_NC_009089 arsenate reductase

MIKLYGYTKCSTVKKAKNWLKENNLEFEDIDMVQNPPSKEELKSIYKTSGYDIKKFFNTS

GMKYRELGLKDIVKTESDDKLLEILVSDGMLIKRPLLLDGKNVLLGFKEDVWKSTLLKED

*

>CD630_17800 Clostridioides_difficile_630_NC_009089 methylase

MKNYTLISPCFFGMEKMLAREITNLGYEIIKTEDGRITYKTDEFGIAKSNMWLRCAERVH

LKIAEFEAKSFDELFENTKRINWSRYIPYGAQFPISKASSIKSKLYSTPDVQAIVKKAIV

ESLKKSYLEDGLLKEDKEKYPIFVFIHKDKVTISIDTTGDALHKRGYREKANKAPIRETL

AAGLIYLTPWKAGRVLVDPMCGSGTILIEAAMIGINMAPGLNREFISEKWRTLDKKIWWD

VRKDAFNKIDNESKFKIYGYDIDEESIDIARENAEIAGVDEYIEFNVGDATQFKSEDEFG

FIITNPPYGERLEDKDSVKQLYKELGYAFRKLKNWSYYLITSYEDFEYEFGQKADKKRKL

YNGMLKTNFFQYPGPKPPRNNK*

>CD630_17810 Clostridioides_difficile_630_NC_009089 lipoprotein

MKVLKLITVVALMMLFVTGCNIKTESPEQLAKIPDYDDTKKVLYDGIDQLLKPDSSMILP

SNTKEVGKINKVDLNSDGIDELVVFEQKEDLSNNVSQVGFVTLSYNGEKYVLGDHFLENG

ESIEYANFYDLDSDGYKEVILLVKSKDKTNMHIYKVVDNEITKIYDLDASWLQNKEDFND

MKVKIGYIDGDDKLDILMLHLNNKTNEMFASVANFDGKMKIKDSVKFENVKNLSELYITL

GNVASSVGNSSAAIKGIVLDIPILKDNNYITQILYMKDGKINKAFSDYDKTITKSYYIPV

DDIDKDKIIEIPIVAGSTGNNKNTYSSKSSATISWYRWNGKEGADSSLIFTSQIYYNYKY

NFKLFIPNNLFDKILVEQEFVGEKAVFKFYYFDYSDRKNLFNIVVESKNKLEDRKNTGTQ

NSIVLQESDEYTFLLVVNNANEMDKLDMTIDALKEYFSLIYG*

>CD630_17820 Clostridioides_difficile_630_NC_009089 two-component response regulator

MVNIILNWRFYFMKEKILILEDEIGIRSFVSINLKREGYEIVEAGTGREAIEKMTTEKDI

TIALLDVMLPDISGIEVCKFIRENFDQVGIIMLTAKAQEDDKIEGFISGADDYIIKPFSI

KELLVRVSALLRRVAKDDSSVKSSEIVSPPFILDIDKRKLFKNGKEIELTPTEFSIVKYL

ISNAKQSLSRDQILDEVWGTNYLYDFKIVDVNIRRIRNKIEDDPSKPKYIQTIWGYGYCF

RKEE*

>PCZ31_RS13230 Peptoclostridium_difficile_strain_Z31_NZ_CP013196 two-component sensor histidine kinase

IVLHFEGLRKKVIKNYFIIIIIMVTLFEGLFMFYIQNYYYDSVKQLLESEIKYADEYNAI

TMETTSFEKKVKNIFDKQPLTKNSEFGISIIDKDKNIILDQYGFKSKEKANYEDVNNALK

DIKTKNLTPYTYRIPDTGEHVMSISLPLKVNNIIEGVVRYTVSLDAIDNAILKQATWLIL

AGIFILIIAILISLKFAETLIKPLRELKKFANELAVGNYNIKLEKMKIVDDEIGDLAQTF

EHMAHEIDKSEKLKEEFISSVSHELRTPLTSIKGWSETLGYESITREELDLGLGIIQDET

ERLIKLVEELLDFSRLSSDRIKLHVDIVDVEGLIVGVVNQLKVKAAEKDISLLFEFENEF

IENIQGDKNRLRQVLINLIQNSFKFTSQGGYIKVVASQDEEITTISVEDNGSGIEKQNLN

KVLDKFFQEDYNKAGSGLGLAISNEIVKLHGGRMKIESEKNVGTKITFNIKNKFAKQA*

>CD630_17840 Clostridioides_difficile_630_NC_009089 tRNA pseudouridine synthase A

MRNIKMIVAYDGSRYKGYQKLGDNNMTIQEKLENVLSKMTNETVEIIGSGRTDMGAHARG

QVVNFRTNCMDSLDKIQKYLYEYLPEDIVVKTVEEVDERFHSRYNVKSKTYMYKIDNNKY

HNPFIRKYATHVSKKLDLDRMRKASEYLVGEHDFTSFASSKSKKKSNVREIYSINIKEDD

NVIEIYVEGNGFLYNMVRIIVGALIDVGLKRRAPQDIKYMLESKDRCQSSDTAPAKGLCL

WKVTY*

>CD630_17850 Clostridioides_difficile_630_NC_009089 argininosuccinate synthase

MKEKVVLAYSGGLDTSIIIPWLKENYEDIDVIAVCGNVGQEDKMEDVYEKALQSGASKAY

VDDISEEFVTETIFKAVKAEAKYEGKYLLGTSLARPIIAKKLVEVAHKEGAKYICHGCTG

KGNDQVRFEATIAALDPTIKVIAPWRIWDIKSREDAIDYAEKHNIKVTATKAKIYSVDAN

LWHVSTEGGDIEHLENEHKKDVYKQCVDPEDACDVAEYVEVYFEKGVPKKVNGEELSPVA

LIHKLNELGCKHGIGVIDIVENRLVGMKSRGIYETPGGTILYEAHNILESATLDKDTLHF

KQMVSYKYGELIYNGLWYCKLRESIDAFMEQTQDNVTGTVKVKLYKGNIKPAGIFTENAL

YDEGISSFGNSELYDHKDAEGFINLFTLPLKIRAMKAGK*

>CD630_17880 Clostridioides_difficile_630_NC_009089 hypothetical protein

LKSTTFVILWTILFVLFGFYVNNKLYDFTEGYKDNISVLEKSIENEDWEKAQKEADSIST

SWNKEKNHWYKVLNHEYFDEIGLRFNILDKAIYTENKLKSLEEVESIKTYLGNIIESVKF

DVNYIF*

>CD630_17890 Clostridioides_difficile_630_NC_009089 hypothetical protein

MTVVLIRSIILYITVLIALRVMGKGEIAEMNCFDLVITLLIAEVASVPMENNNIPIINGV

AAISGLVIMQTLISFLSLKSRKLSSFLSGKPSVLIDKGKIVYKELKKERISIDELLEQLR

IQGYFNLKDVQYAILETDGNLSVVPASSYNSTPPRAFNHLPIPLILDGRIINKNLDIAQK

DTNWLMGILKSNHIETFKDVLICVLDENDKIFIQNKKGD*

>CD630_17910 Clostridioides_difficile_630_NC_009089 hypothetical protein

LLNLKEIREEIIKKYIGDSLISELTEELKNILITDLQSNPFKSNIELSIIPANIEIFKKI

ENILIDSKVNLKMGWERILINYILKDLFELNYEINKSCNEPLMFRVIISIDSYDA*

>CD630_17920 Clostridioides_difficile_630_NC_009089 L-threonine 3-dehydrogenase

LKKILITGALGQIGSELTIKLRNEYGEQNVIASSRRVKEGNPVCESGIFEILDVTDKNRF

FEIAKKYDVDTIIHLASLLSAVAESKPLEAWNLNMNGLINGLEIAKELDCKFFTPSSIAA

FGENSPKNMTPQDTLQRPNTMYGVTKVSGELLCDYYHSKFGVDTRGVRFPGLISYVTPPG

GGTTDYAVDIYYEALKNKRYKSYIAEGTKMDMMYMPDALQSIVDLIEAPADKLIHRNAFN

ITAMSFSPEEIADSIKKYIPDFVIEYDVDPVRQSIADSWPNSLDSSSAVKEWNFKFSYDL

DKMTKDMLEKLSEKGIGK*

>CD630_17940 Clostridioides_difficile_630_NC_009089 hypothetical protein

LFIDEELEGYILTCKISEDFKNIPEYSDEEFYVTVYKDESSDSGYYALLENKEERVVWDG

EVVANNIFNNLWIVVNKVKTG*

>CD630_17950 Clostridioides_difficile_630_NC_009089 hypothetical protein

MEYSYSKMNLKKGDIVEVNLEKQANVILLDHINYVKFKNQRNYDYYGGFAKKNPCRMRVP

NTGTWYLVVNQDGNSGIVNFSINTIQN*

>CD630_17970 Clostridioides_difficile_630_NC_009089 coenzyme A disulfide reductase

VKVLIIGGVAAGTKTAAKIKREMGDNCSVTILNKGDDISYAGCGLPYYVGKIIEDKSSLI

VNTPESFSKLTGAEVRCGVEVTSIDRNAKQVTLKTLSTGEKENLSYDKLVIATGADPIKP

PIPGIDLPGVFFMRTPNDAIKLRDSIENGIKRAVVIGGGFIGLEIAENLSAMGIRVSVVD

MAEHVMPGFDTDFAEYVENHMADKGIMIFTGDQVVGIEGENKVEKLRTKNRAIKTDLVVM

SVGIRPNTGFLKDTGLEFAPNNTIIADEYMLTNDKDIYVVGDCAFVKNSLTNKPTWSPMG

SSANHEGRICAQNISGKSKTYNGVLGTTVVKLPELNAGKTGLSKEIAEKEGYNVCSVTIA

TDDKAHYYPGASNFIIRMVAEKNTRKLLGVQVMGPGAVDKIVDIAATAITLGADLYSLES

MDLAYAPPFSTAIHPFVVAVNVLQNKLNGELDSVLLDEIEDFDSWTKLDVSKAPSIPSLR

YIPVGEINGEIEGLAKDEKILLICAKGKNSYMAQNRLRRFGYTNTKVLEGGILFYPNLKV

MEE*

>CD630_17990 Clostridioides_difficile_630_NC_009089 tellurium resistance protein

MGITLAKGQKVSLTKSNPGLKKVIVGLGWDINKYDGGFDFDLDASAFLTGTDGKVTNDGD

FIFYNNLKHASGAVEYMGDNRTGVGDGDDEQINVDLSKIPQNIAKISFSVTINEAITRRQ

NFGQVENSYIRIYNEETNEELIKYELGEDFSIETAIVVAELYRHNGEWKFNALGSGFEDG

LAGLCKNFGVNIG*

>CD630_18010 Clostridioides_difficile_630_NC_009089 membrane protein

VMLLKKDRITFSEILILIVGCILMAISLNLFFNPHAIAAGGITGLGVVLNSLFGVELWIV

NLLLNVPLFIFAYKILSKKDCFKTVLGIIFLTIALKLTANMATLDITNDMYLAIISGSIL

MGVGQGLIFRINGSTGGTDLMALLLNKYFPTFSIPVLMGIVDCVVVVLSGIVNRQVEIAL

YSTVALYILVKVSDLLIEGFNYSKSFTIISDLSKDISKKIMEDLDRGATILKGEGAYTGE

NKNVLLVVVEKKEVVELKKLVKNVDPNAFIIITDIHEALGNGFKKIE*

>CDM120_RS09425 Clostridioides_difficile_M120_NC_017174 hydrolase

MAKLTLEHIKGNTYYIQLPTIVGVYVDGKDAILIDSGNNKDTARQVLRLLEEHNLIPKLI

INTHSNADHIGGNAYLKNQTKCKIATTKIEGYFTENPILESAFLYGGYPSKALKNKFLLA

KESEVDYIIPSNGKIVDTELEAISLPGHYFEMIGVKTPDNVLFVADSLTPENIITKYHFF

FLLDIDSQFKTLEKLRMLEADFFVPSHSVKTTDIKNLIDINKKKMEEIIDNIKKVCCEPV

MIDKVIEKMCDLYNVKLDANQYVLVGSTIRSYITYLYENNMVEYIFDGGKMMIKVLQ*

>CD630_18040 Clostridioides_difficile_630_NC_009089 LacI family transcriptional regulator

MKKITINDIANLAGVSKSTVSRYLNNKDISDSTKEKIKTIIDEYGYEPNAFAQSLRAKKT

YFIGIITPCLDSFVKSKIMMAIDEELKELKYTSLIINTNRKIRSEIDSISKLASLKVDGI

ILIGTEITKEHKNEIEKLDIPIVVVGQKVDGINSIVNDDYGAGYKMGQYIANKGYKNIVY

LGVDESDISVGLNRKNGVLNGLKDKGYDAKVFYTDFDQETSIQRSGEMLESENPDIIICA

TDNIAIATMKEINKRGKSIPQDISVAGFGGYDVLSIISPKLTTIKFENKNAGKVAANTIV

NLIQERKEPLLKEIKFELIEGESTINKN*

>CD630_18050 Clostridioides_difficile_630_NC_009089 sucrose-6-phosphate hydrolase

MYKEPKYRTILESTKEELNKLRNISLNDKYKPLFHIHPQHGLLNDPNGLAYYNGKYHVFY

QWYPYDATHGMKHWAYVSSDDFVNWNREDVALIPIESYESHGAYSGNAIEVDGKLHMYYT

GNIKYSAEDRYAYQNLAIMNKDGKITKYENNPIVSEIPKGYTGHVRDPKVFKRKDKYFML

LGAQTSDKKGVIIVYESKNSIDWNFKGELNVKNIDEDFGYMWECPDYINIDEKDILIFSP

QGVEPKGFDYQNIYNVVYAIGNMDLDNLTFEIDTMKELEKGFDFYAPQTFIKDSQIILFA

WAGMGEVLYPTDKNKWAHCLTVPRKLNIKNNKLLQMPVDELIKLRYDETSGQNTIKNNIN

IIENNENLYELNINIKNIDSNKFGLELFSSQDEGVKLEFNKLGNIVTLDRSNFKKVFGVE

YGTNRKEYINIDENTNIKVLADRSILEIFINDGEVVFTSRIFAKENSNQIRVYSDKIVEY

EYTKFKLKQGIEL*

>CD630_18060 Clostridioides_difficile_630_NC_009089 fructokinase

MKKVISIGEALIDFIPNQNGGKLKNVSEFRRVAGGAPANVSAVVAKLGGKSSFISKLGKD

AFGDYIIDVLNEVNVNTDYVLRTSKANTGLAFVSLKEDGNRDFSFYRNPSADMLLEADEV

KKEWFNNCHILHFCSVDLIDSPMKLAHKKAIEYALESNSIISFDPNIRLPLWDSEQSCKK

AISEFLPFAHIVKISDEELEFITGENDIEKSLHKLFVGNVELVLYTKGKDGVDAYTKKVK

GMCKGVKVNAIDTTGAGDSYIGSFLYTLLYKQITLNDIKEMKQETLNEYLEFSNYYAARS

TTIKGAISSYATKEEITEFIKNL*

>CD630_18070 Clostridioides_difficile_630_NC_009089 NADPH-dependent FMN reductase

MEVCILMGSPKKKGNTAAILKPFIEELENYNSNIEVIWLYDCKIEPCIACKKCQDNLFDF

GCYCKDDVQKIFKKILMCDLIVFATPIYSWYCTSPMKALLDRLVYGMNKYYGNEKGPALW

AGKPVALITTCGYRPEKGADIWENGMKRYCKHSQLKYIGMLVERDLGKPNFMNKEKEEHS

RQFAKQIYYILDNSIEVDNC*

>CD630_18100 Clostridioides_difficile_630_NC_009089 beta-lactams repressor

MTISKIPQAELKVMKFIWEKNDTVTSKETIEAMERKYGWKQTTTLTLLSRLVKKHFLSAE

KIDRYTHYTIVVGHKEYLSVETKDFLSNIHDNSLQSLISALHDDEVVDKDMLDLFESHFK

NLEEE*

>CDIF1296T_01946 Clostridioides_difficile_ATCC_9689__DSM_1296_strain_DSM1296_CP011968 hypothetical protein

MGDYNKMIELKNNLGISERKLKYPCIYKHFKGKYYATMGLSKAIDDIENICEIYGKENLI

QNRNKYKLVIRHTEREEDIYVYRDLDGNFYHKKEEDTNDLVLYKTLYDDTGIFARPLDMF

LEKVDTDKYVNSIQEYRFEEVYK*

>CD630_18140 Clostridioides_difficile_630_NC_009089 phosphoglycerate mutase

MGNTFYIVRHGQTDWNILGKTQGHGNSDLTPQGIEQAKELSEDIGKYSIDYIFSSDLGRA

MQTAQILGDKLNIEVQKTEALREMGFGVWEGLLIKEIQKDYSDIYATWRNEPHLVNIPEG

ETLKIIKERVDAFIKELNEKYDNKNIILVTHSITLRVMLLSFLESGMENIYRIKQDNTAL

NIVEFKDYGPVIVKMNDTSHIKNHVKINNSALE*

>CD630_18150 Clostridioides_difficile_630_NC_009089 hypothetical protein

MSKVIVVGGGASGMMAALSASKNNNEVILVERNGELGRKLRATGGGRCNFTNNREIEDFF

DKVVSNKKFLYSSFYTFTNKDLISYFESRNLEYKIEEENDHKVYTKNDKSIEVIEVLNKD

LLNHNVKIMYNKKVIDIITEEIALKDDSNKDKSKYLIKGIILDNGDKILGDKVIISTGGV

SYSKTGSDGSMYKILKKHGHTLNKLYPALVPLTIEEKWIKDLQGISMKNVEISCKIKKRK

ISKSGDMLFAHFGITGPCVLIMSSYINKIIEKEKVELNIDFLPNLSTDEISSIIRAFPNK

NVLNNLKQILPQNFLKEIFSLLSLVDKKASDLSKADEIRIIEYIKNMKLTCNGTTGINTG

MVTSGGISVKEINSSTMESKLVKNLFFTGEVIDIDAETGGYNLQIAFSTGYLAGISV*

>CD630_18160 Clostridioides_difficile_630_NC_009089 cytidylate kinase

MGNLVIAVDGPAGAGKSTIAKIVAKKLNINYIDTGAMYRAVTYKCLKSGIDVNNEKEVIQ

IAENSDIDFKDNNIYLDKEVINEEIRTIEVSNNVSNVAKIKEVRQLMVEVQRKIGMKNSV

ILDGRDIGSYVFPDADYKFFLVATPEERGNRRYKELCNKGYNTTLEEVIEDIIRRDEIDS

NREFAPLVKANDALEIDTTGKTIEEVVEEVVSKINL*

>CD630_18170 Clostridioides_difficile_630_NC_009089 acyltransferase

VNFYRFVINIFKGFSKIFFKYEVIGAENIPDRGNIVIASNHKSNLDPIFLAAAIENREIA

AIAKKELFKVKPLGFILKKLHVMPINREKPDVSTIKTILRSVRDGYVLGIFPEGTRIKGD

SFGKAKAGLSVFTIKSKSKVVPVSIISKYKLFSKVIVYIGEPISFEEHFKEKLSNDDHER

ISQEILEVIKQNYFKYSK*

>CDIF1296T_01953 Clostridioides_difficile_ATCC_9689__DSM_1296_strain_DSM1296_CP011968 adenine deaminase

MGNLKRQCDVSSGREKADIVLKNGTIINVFTEELITGDVAIVGDTIVGIGDYKGNVEIDC

SNKYISPGFIDAHMHIESTMVMPIELSKKLLKSGTTTIIADPHELVNVKGASAIDFLLES

TKDIPLNVYIMVPSSVPATSFETNGVGKFSAKDMESYVNNPRILGLGEVMCFNDVINSEN

EILDKLELFKNKVVDGHAPNINGKSLQTYVCAGIENDHECITFDEVYEKLRAGLKILIRE

GSAAKNLKSIVSGMLKHNLPIEEFMFCTDDKHLDDIEKQGHIRWNIKCAIDLGMEPVRAI

KVATYNSAKAYGLRKIGAIGAGYKADIVVLNDLDKMEVDSVYKDGNLVNEEMFSNYNYEI

KDKELLNTVKFKYINKEKIQLKVSEKNYVMEIVPYQILTNKVYESLPCADGYFVPNKEYS

KLCVVERHRMTGNVAIAPLKGFGIKNGAIATTVAHDSHNIIVAGDNDDDILVAINYLKEI

QGGYVIVSNGKVLAHLSLQVAGLISTFTAEEVQEITDNMLEIARKMGVPEYVDPFITLSF

MALPVVPQIRLTDLGLFDVEEFKFI*

>CD630_18220 Clostridioides_difficile_630_NC_009089 thiol peroxidase

MLSIGTKAPEFTLEDKDGNKVSMSDFKGKKVVVYFYPKDNTPGCTRQACAFRNAYDGFKK

EDIQVIGISKDSIKSHQKFAEKHELPFILLSDPDLVAIKAFDVWKEKKMYGKTALGVVRA

TYIIDENGIIEKVFEKAKPDTNAQEILEYLEKQE*

>CD630_18230 Clostridioides_difficile_630_NC_009089 hypothetical protein

MIIMLSPAKNMKNIEVFDRDLSLPCFIDNTKEIVENIKTFAIEDFKNKMKINEKLAVLNK

NRFESIKFDRLGNPAILTYDGIQYKNIEAENFTRKDEEFANSCIRIISGLYGVVKPYDSI

YEYRLEMQTKLRVGEFKNLYEYWGNRIYKELIKEKTAIVNLSSNEYSKSIEKFIKDSDTY

ITCTFKVNKNGILKVESTQAKKARGMMTKYIVKNRIRDIEELKKFNLEGYKYKENLSNNS

EYIFVKE*

>CD630_18250 Clostridioides_difficile_630_NC_009089 O-acetylhomoserine sulfhydrylase

MYNKETICVQGNYKPGNGEPRVLPLYQSTTFKYSSIDQLAELFDLKVDGHIYSRISNPTI

QAFEEKISLLEGGVSSVAVSSGQSANMLAVLNICKSGDSILCSSKVYGGTFNLLGPSLKK

FGIDLISFDLDSSEDEIVELAKENTKVVFAETLANPTLEVIDFEKIANVAKRINVPFIVD

NSLASPVLCNPLKYGANIVTHSTTKYLDGHASSVGGIIVDGGNFNWDNGKFPELVEPDPT

YHGISYTQKFGNAAYATKARVQLLRDYGNCLSPFNAYLTNLNVETLHLRMERHSENALKI

ARFLEKHENVDWINYPGLEDNKYYENAKKYLSRGCSGVLSFGVRGGLENAKKFVEKLQIA

SLVTHVSDVRTCVIHPASTTHRQLTEEQLIASGVLPSLIRLSVGIENVEDLIADLNQALN

F*

>CD630_18260 Clostridioides_difficile_630_NC_009089 homoserine O-succinyltransferase

MALILPKGLPVINKLLDEGIDVIYKEDFKKELKYEENIDTKIAILNLMPIKIDTELDLLR

RIDKTGFNVSVEFIKISTRESKRSCNEYVKEFYKTFDEAKGEYFDGFIITGAPVEQMEFE

EVDYWNELEEIMDYSKRKTKSTLYICWAAQASLYKYYNVKKLPLSQKCFGVFKHKVDKDS

KIVDGFENEFFAPHSRHTTVNIEALKENKELSIVSHSKEAGPYIITNSRDVFVMGHSEYD

KYTLDKEYKRDINKGDKISIPQNYYINDDPSEEPTVKWKKHSELLFRNWIKNYLIQ*

>CD630_18300 Clostridioides_difficile_630_NC_009089 two-component response regulator KdpE

MSKKLILLVEDDKTIRKFISTALLTQDYDVKEAITGKEGISIAVSYSPDVVLLDLGLEDM

DGIEVIKAIRQFSNIPIIVVSAREQDRDKVEVFDAGADDYLTKPFSIVELLARVRVAFRH

SQVEAQQKDDVKSTFEVDKLLIDFDKRKVIVDDVEVHLTPIEYNILSLLAKHHGKVLTHN

FIIKEIWGSVIGNETKSLRVFMATLRRKIEKQPANPRYIITEVGVGYRLNDE*

>QAE_RS0209090 Clostridioides_difficile_QCD_23m63_NZ_CM000660 cation transporter

MYILNLNTRETIEDFRDKFYVAENSYLILSAPKNLKLLKETLDIDEITFNDCLKFDEITK

LDLFDNYDFLSLNTFELRDGEAVIEEVNMYLSDNFILVVVNEEHFLFEFVKNIILKNSQL

EKNPVINLFKINYLILREVIKNGFESLEKVEELILQIEDEMMDNINKNHVSRISDVRGLT

RIIVKNTRPLLYIGDRIVKENIRYLKYSNVKKYNLENFQGIDFGIDKLYSFALSTRELAD

KLLDIYSSRVGEKTNNLITKLTLLTAISAPLTIITGIYGMNFRYMPELNWIYGYPATLFF

MLCIIFVGIIIFKIKNYCKLQMIFN*

>CD630_18320 Clostridioides_difficile_630_NC_009089 phospho-2-dehydro-3-deoxyheptonate aldolase

MIVVLKMGADKNEVKKLIEAIGREGVEVNPIDGTELTVLGLVGDTSKIDAKRIEANKVVE

KVMHVVEPFKKANRKFHPEPSIINVNGMEIGSKKIAMIAGPCSVETEDQIVSIAKDVKKS

GAGFLRGGAFKPRTSPYAFQGLKYDGLDLLKKAKEKTGLPIVTEIMSTQDIDIFEENVDV

IQVGARNMQNFDLLKELGKTNKTILLKRGLSATIEEWLMSAEYIMAGGNENVVLCERGIR

TFETYTRNTLDLSAILAVKKLSHLPVIVDPSHAAGKSWMVDSLSKAAIAVGADGLIIEVH

NDPAHALCDGKQSIKPNEYDELISELKTIASAVGREI*

>UAB_RS0203275 Clostridioides_difficile_ATCC_43255_NZ_CM000604 3-deoxy-7-phosphoheptulonate synthase

MHYVFKNKSDYLKCRRFLDEYKVDYLNLKISNYYLINVFDETLKEWIENKELTFKRTESI

SLENGYLATKNYKEKSIIKVNDIEIGNGRPVFIAGPCSVEKEDTLREIAIKVKESGADVL

RGGAFKPRTSPYEFQGLGKEGVDILYKIGKELDMPVITEILDVRDLDYMIDKVDILQVGT

RNMYNYPLIKELGKVDKPILLKRGLSASVKEFLLSAEYIMLEGNEKVILCERGIRNYDTA

TRNIMDIATIALLKELSHLPIISDPSHATGKRTLISPMVDSSISAGADGVIIETCNNPDA

AWSDGKQSICPKELQSIINRHKKFK*

>CD630_18340 Clostridioides_difficile_630_NC_009089 3-phosphoshikimate 1-carboxyvinyltransferase

MGSLKIYPSKLSGDVKIPPSKSMAHRAVICSSLSNGKSRISNIDFSDDIIATIRAMTSLG

AIIEKKEDILEISGIFSKEGILNRENQLNQPKLTIDCNESGSTLRFLVPISLAFDGVKRF

IGRGNLGKRPLDTYYEIFDRQNIKYSYKENQLDLIISGKLKPDEFRVKGNISSQFITGLL

FILPTLESDSKIIITTELESKGYLDLTLSTIKDFGVEIINNNYKEFIIKGNQTYKARDYK

VEGDYSQGAFYLSADAIGEDISILDLKEDSLQGDSEVVEILSRMGMEILREGNKIKGITN

GLNGTLIDASQCPDIIPVLSVVASLSIGKTTIINAGRLRIKECDRLHAINVELSKLGANI

EEKEDSLIIEGVSKLNGGVEVWSHKDHRIAMTLAIASCRCDKPIILKDFECVSKSYPHFF

KDFKMLGGRIDEWNMGK*

>CD630_18350 Clostridioides_difficile_630_NC_009089 chorismate synthase

MSGIWGNNLKVSIFGESHGNAIGINIDGLPSGIELDLDKIDKEMKRRAPGKNSISTSRNE

SDIPEILSGYFNGRTTGTPLCAIIRNSDTRSKDYGELKNLMRPGHADFTGNVRYSGFNDY

RGGGHFSGRITAPLVFCGAICKQILSQKGIEIGAHIKKIKNIEDMSFDYVNISKQQLSNL

QTLELPLLDLSKEEAMKNTIIDAKNQGDSVGGIIECAVVGINVGLGNPFFDSVESTLSHL

LFSVPAVKGVEFGLGFELADMYGSQSNDEMYYEGNQVKSKTNNNGGIIGGITTGMPIIFK

VAIKPTPSISRQQNTVNIKDKKDDILYIKGRHDPCIVQRAIPVIEAVTAIGIFDLMKGR*

>CD630_18380 Clostridioides_difficile_630_NC_009089 shikimate kinase

MINKTKEKLILIGMPGSGKTTIGKLLAKEYNCSFCDMDDYIIQISQKSIAELFSEGEDIF

RNYETQACRELSISDKTVISTGGGVIKKDVNMEILKETGIIIFIDRPIQKILEDININSR

PLLKNGKDRLYNLYNERINLYKKFSDIEILNDKSLNNAVYNITNAVSENFKFDFKEK*

>CD630_18390 Clostridioides_difficile_630_NC_009089 prephenate dehydrogenase

MNIVIVGLGVIGGSFAKALKKAGYENVFGVDVDLETLKKAEKAKIIKKGCTTGKELFKKA

DLIILSIYPRLVVDFLNNNKNFFKKGTIITDTTGIKETLINDVLQIIPDDIDFIFGHPMA

GREKKGIDFASEQVFNGANYIITPTGRNNIKNLELVENLILEIGFKRVKKLTSQKHDEII

AFTSQLPHVMAVALINSDEEGRDTGKFIGDSYRDLTRIANMNEDLWSELFLGNRDNLLKV

IENFESEVNLVKEAIFNNDKNKLIEYFKKSSIRREALEK*

>CD630_18400 Clostridioides_difficile_630_NC_009089 two-component response regulator

LLKRVRCSYLKKNRLIAKNISIAFIVLFFFSVFTFFYVGNINRVLEYETNDIITVTIAGW

IILSFLFLGIIIYILYSKANSQKTIEKVAYTDFVTGYSNWRKFELDVTNLLKKTSQNNKY

AMVIFDIDKFKAINDIYGHKKGNLILKDIADTLNELTDINETFARVSADNFNILLTYNKK

EDIINIIKKIMANNELVNLSFGIYEIKDKDLSVSVYSDRASLAKSSIKNNSDVNFAFFND

KLREKLLFEDKIEKEMEYALESGQFVMYLQPKYNIKLDKFCGSEALVRWQYTEKEVIYPG

DFIPIFEKNGFIRKIDMYILEQACKEIRSLFDKGISPLPISVNFSRVDFFKKDFIENIVN

ICDRYKIPYSLIEIEITESSMFGDTDTLFNVSRNLQDIGFIVAMDDFGSGYSSVNMLKNI

PLNVIKLDRGFFVDDKDVDKSQIVIKSIVSLIKQLGIRVVAEGIETRSQIEMLKKANCDI

VQGYYFSKPLPIKEFEKLVYKI*

>CD630_18420 Clostridioides_difficile_630_NC_009089 hypothetical protein

MLNSITLILGIIFTIYYFYLKIVFGGISFSEIFLVVGIVLIIYQIFKKKIKEKKVFYRVL

KIIISIFLIVFVVTESLIIFYPKNSLESKSDYLLILGASVKKTTPSTTLKGRLDTALKYL

KINDNCYVVVSGGKGSGEKITEAKAMKDYLIKNGIDKNRIIEEDKSTNTYENFKYSKLKI

EEHSQRKLSDLKVKIVTTDFHVLRSKILAYRNGYKNTSFYASKSKLSFVPTYYTREFFAL

WKTIIFDR*

>CD630_18800 Clostridioides_difficile_630_NC_009089 hypothetical protein

MHENKKNLLDGSIAQYNDTAVPKIPVFAGNDITSEVYYFKPNQVLNAHRHPNGEQIFVFL

KGEGKMKLGEHECDVKNGDTVFVPTGEWHEITNGSNEEMVAVQITKINAGAEYRG*

>CDM120_RS09650 Clostridioides_difficile_M120_NC_017174 SAM-dependent methyltransferase

MKQNKYDDDKFFNKYSKMERSINGLAGAGEWHTFRKMLPNFKGKRVLDLGCGFGWHCQHA

VENGATSAVGIDISEKMLKEARNKTTFDNIKYICMPIEDINFPKDSFDVVISSLAFHYIQ

SFEDICKNISNCLSNKGDFVFSVEHPIFTAHGSQEWHCDKDGNNLHWPVDRYFEEGLRKS

NFLGEEVIKYHKTLTTYLNTLIKTGFEVLEIIEPQPEEKLLDAIPGMKDELRRPMMLLVS

AKKDK*

>CD630_18930 Clostridioides_difficile_630_NC_009089 oligonucleotide binding regulator

MNNQQQEVYKKLDELSIPYEAVNHPPVYTIEEMEELKTLHMEFVVKNLFLRDAKGKEHFL

VVLGKDKKADLKDIRQQIDSKPLSFASEERLQKYLKLTKGAVTPFGILNDKEAVVKVVFD

KDLLKMDKIGIHPNDNTATVFLKFEDMKKLIEENGNEIYYVEV*

>CD630_18950 Clostridioides_difficile_630_NC_009089 hypothetical protein

MNLSKKIEDKRKAKQRAEKVKKAKIATAGVVLGAVTGAVSGVLLAPKSGKETREDIKDAS

QQIAEKINMKTVDVKGKVSEKLEDKKGNFIESKKKIKKYIDNKKSVENSSEEDKIIDIVE

PVSVTEE*

>CD630_18960 Clostridioides_difficile_630_NC_009089 hypothetical protein

MDNTITINISIIYVLLAILACIALVYLIIVLVKLHKILNNVGTMLNENTQNINTTLNNLP

NVVETFGEVGENMKDVTEVVTEVTADFLVTKDSVKSNMDVIVDILTIIKNIFLDKK*

>CD630_18970 Clostridioides_difficile_630_NC_009089 hypothetical protein

MIRTKVVELIATVCRENKPHKWVDENYTPYDKSGKVELMSIEDLNELISSSGRADFLYSS

RLQKLLNEVYINQSRASYISGCGLFWSSYWDILEEKFEEWLYNSYIFFDEDDEYLEGMED

FELECKDVLMDVIETTSIDIYLQMIKRNITNY*

>CD630_18980 Clostridioides_difficile_630_NC_009089 cell wall hydrolase

LKVVIIPGHTLIGKGTGAVGYINESKETRILNDLIVKWLKIGGATVYTGRVDESSNHLAD

QCAIANKQETDLAVQIHFNSNATTSTPVGTETIYKTNNGKTYAERVNTRLATVFKDRGAK

SDVRGLYWLNHTIAPAILIEVCFVDSKADTDYYVNNKDKVAKLIAEGILNKSISNSQGGG

ENKVYENVIVYTGDADKVAAQILHWQLKDSLIIEASSYKQGLGKKVYVVGGEANKLVKGD

VVINGADRYETVKLALQEIDKL*

>CD630_18990 Clostridioides_difficile_630_NC_009089 dCMP deaminase

LNKKLENRCSWQEYFMRLCETVAERGTCDRAYVGAIIVNSENRIVSTGYNGSISGDKHCS

EVGHEMRDGHCIRTIHAEQNALYYCAKEGISVKDCSIYVTHFPCLNCTKAIIQAGIKHIY

YRTGYRIDEYAIKLLQSSNVLYTKL*

>CD630_19000 Clostridioides_difficile_630_NC_009089 hypothetical protein

MGILEGAAMIREIAIKIAKEKGITEQKAWPEAVKEFKEKYELVL*

>CD630_19010 Clostridioides_difficile_630_NC_009089 transcriptional regulator

MLSENIKTIRKSKGLSQQELAIKLNVVRQTISKWEQGLSVPDSDMLISISEVLETPVSTL

LGETIVESEVDNLKVISEKLEIINLQLAQRKNMRRKMFHWLFILLCAVILIVFVVLGRLN

SPYLDWNYTDPETAVFGVAFHAFEWLFVRLVPIVLIGAIIGIFLTRKKI*

>CD630_19040 Clostridioides_difficile_630_NC_009089 ABC transporter permease

MRLNKRSEGYINYVKGVKREKRNVLFYQLLILIGFIVIWELLAYLNVINTFLFSKPSDIY

NLFIQYAFSGQLFKHIGISVYETVLGLVIGTVLGILVAIALWWSEKLSKILDPFLVVLNA

LPKTALAPIIIVWVGAGIEGIVVTAVTISVVVTILSAYNYFMNIDEEKIKMLKSFGATKS

QILFKLILPANTGNLINLTKINIGMAWVGVIVGEFLVSRYGIGYLIVYGSQVFKLDLVMM

GVFVLAICAWAMYAVVNIIEKIYNSR*

>CD630_19280 Clostridioides_difficile_630_NC_009089 membrane protein

LSNIITNLIESIFTNPITILFLLAITVYSVFKIIENNRVYSFVSSKLDEINRDYKTSEFY

KKVRDDYYTYSKENPYADVNITSFIEEVVSDLKHNNLPLLEKIRSIKNSSSISILLGVLG

TFVGLSTMLLCVDTKDIINSLPSTISSMQTAFTTSIFGVVFSLIIGYFTKIKDCEHVLIQ

IMLKSENLLTSEITHFKSERMDLKVEEVKNTIKQISKSIEAIEKFDKISKDLNDFNDEFI

SGIEALKSLLEGSQSSIKTFDQSVRKLDKQFNILNIKFVKLFDKYDNQDNINKEILFDIK

ESSKNIYNATESQFKIRDYIKNINAGFALYERSAQDLLTKLMTHENKISQNQKILLDEKF

TLDDSIKNLSSIIENFSNDLQVKLDMMFENSLDIQDKLDVMFNNSFMNDEVPLDSEELFK

DDVNNVFNPFSEEIYEIEDKEIKVIGEDELNE*

>CD630_19290 Clostridioides_difficile_630_NC_009089 membrane protein

MNNKYRRTVNREFEKSSFWPTFTDLLSTVLMVVILILFSSESISGSVEQDLAKNVNASVE

ETFKKSGIPVKVDKSNGQVTFGEKTMFDVDSDVLKPEAKEMLKMFVPKYIETIYKDYGDY

ISKIVIEGHTDDVGSYIYNLDLSQRRAYSVAKFIVGDEIGDYKYKDKVTKHIIAIGRSKA

ELIKNGDNSVNRDASRRVELKYEININQNK*

>CD630_19310 Clostridioides_difficile_630_NC_009089 LexA transcriptional regulator

MYLDLTEKQVLILEFIKSQIILKGYPPAVREICTAVGLRSTSTVHSHLNKLEKLGYIRKD

PTKPRAIEVLERSKVNDVSGANQEIIELPLVGQITAGEPILAQQNIEEYIPFPASLVKGS

NNFVLRVKGESMINAGILDEDYVVVDKKNTALNSQIVVALINGESATVKRFFKEGNLIRL

QPENDFMEPIMLNDSEVEIVGIVTGVFRVIK*

>CD630_19330 Clostridioides_difficile_630_NC_009089 membrane protein

MIYIIEILKISVINIFCFIGIFIVFGLIFSITENLNNKFIFSSFGKTGIIVTGAIGTFVH

ELSHLIMCLIFMHKINSVKFFRPIESKNDGILGYVSHSYKKDNLYQSIGNFFIGIAPIIG

GTIMIILIFKILLPDSYMKVTQNIDLKLYVSMINNLNINGFIHIILGDISNFIRIMLLTP

SVYSLKYFIFMFLMYSISTHMSLSSADLKNSLNGLSFIVIVIFVISSITYLFGFDNLNIS

SLIVKYNIFISFFMAIGFIFSIVTLIISYILSLVSPFKNFS*

>PCZ31_RS12630 Peptoclostridium_difficile_strain_Z31_NZ_CP013196 amino acid-binding protein

LEEIYIMKLKLLVEEYAVCRLNNDSKIPTWIDTEKFYSITRTDDELSVVCLNNNIPSDVK

SEKEWRILKILGPLDFSLVGILSKLSSLLADNKISIFAISTYDTDYILVKEKDIENACKI

LSCNGYEVE*

>CD630_19350 Clostridioides_difficile_630_NC_009089 stage V sporulation protein S

MEVLKVSSKSNPNSVAGALAGVLRERGVAEIQAIGAGALNQAIKSIAIARGFVAPSGMDL

VCIPAFTDIEIEGDKKTAIKLIIEPR*

>PCZ31_RS12615 Peptoclostridium_difficile_strain_Z31_NZ_CP013196 acetyl-CoA carboxylase carboxyl transferase subunit beta

IDYDKKFLSSKESKYVTISLDDNFKKNSVDDKFWTYCKGCDSHVFRKDIEENSFVCPKCS

RHYGLRARKRINLLIDKGTFMEFNSDVEFQNPLNFPKYKEKVDSYKEKTKESEAVVTGYG

RINGIKTVICVMNPDFMMGSMGSIVGEKITYSIEYAAENNLPIIICSASGGARMQEGMVS

LMQMAKTSQALSKLEEKSLPYISVLTDPTTGGVTASFAMLGDIIISEPNTLIGFAGPRVI

EQTINQKLPEGFQTSEFLLEKGFIDMIVDRRKMKEVLYQILAMHKK*

>CD630_19380 Clostridioides_difficile_630_NC_009089 acetyl-CoA carboxylase biotin carboxylase subunit

LIKKILVANRGDIAVRIIRTCKELGIKTVAIYSEIDKDCFHRYIADESICIGPNNISKSY

NNIENIIYLALKLKCDAIHPGFGFLSENPEFAKQCEDNNIIFIGPTREQMILMGDKSRAR

ETMMELNIPVVPGSESVLKTKEEALEVAREIGYPVMIKASSGGGGKGMRIVRKEEELFSN

FDMASSEALAAFSNSDLYMEKFIENPRHIEVQVFGDKHSNAIHLGDRDCSMQRRNQKVIE

ESLSPYLSDEERQKLHKIAVDIVKGVGYIGAGTIEFIVDKDKNFYFIEMNTRIQVEHPVT

EMVTNLDLIKLQISIANGDKIPFKQEDITFRGHAIECRINAEDSSKNFAPSPGKIESLNL

PGGFGVRFDTFVYAGYTIPPLYDSMIGKLICWAETREECISRIYRALDEIIVEGINTNVE
[truncated: 431,240 more chars]
