# Supplementary material for: Core-genome-mediated promising alternative drug and multi-epitope vaccine targets prioritization against infectious Clostridium difficile
Source: PLoS One. 2024 Jan 19;19(1):e0293731. doi: 10.1371/journal.pone.0293731 (PMC10798517; doi:10.1371/journal.pone.0293731)
Supplement: S2 File — (DOCX) [file pone.0293731.s002.docx]

>CD630_00150 Clostridioides_difficile_630_NC_009089 transfer RNA specific adenosine deaminase

MESSFYMKEALKEAYKAYNKKETPIGAIIVKDNQIIARAHNLTETLKDSTAHAEILAIKQASEKLGGWRLTDCDLYVTMEPCIMCSGAIVNSRIKKLIIGTRHVKNSYIEKQHEFKLDYFNNNNVKVAFDVLQEECSIILQEFFKALRKRD*

>CD630_00160 Clostridioides_difficile_630_NC_009089 DNA polymerase III subunits gamma and tau

MHKALYRAYRPQKFEDVIGQDHIIKTLKNQIYSDNIGHAYLFCGTRGTGKTSTAKIFSRAVNCLNKINEEPCNECEICESVLKDNTMDVVEIDAASNNSVDDIRELRESVKYSPANAKYKVYIIDEVHMLSQGAFNALLKTLEEPPSYVIFILATTEPHKIPATILSRCQRYDFKRVTVKDMTLRMKKICEDEGIDIDDKALNLIARNSQGALRDALSILDQCMSFGESKIDYKDVVELMGSVNIEQLFELSQCIVEQDTKKSLEILNEFVLWGKDIRNLINDLIDHFRNLMVCKVSSELDEIISLPEETIEQLKIQSKNIDINDLIRILNILSITQDDIKSSSNPRVLVEITIMKIAQPMFDESKEALIKRVENLEKMIELGNFKSEKIGNNKEKEYEVDREIDVKQENVVYEDVKNEDVILIESSWKNILKQIKKDKKMPIYALLSEVKSFNVYSNMLYVIFDDKFDFAKTRLSSQDTINYLEKTIRDVLNRSFNVKIVLTSEVKDINLEVKEKKDIGEEILKNIVSEEILEIKDSIDENESK*

>CD630_00210 Clostridioides_difficile_630_NC_009089 pyruvate carboxylase

MLKKFNKILVANRGEIAIRIFRACSELGIKSVGIYSKEDKYGLFRTKADESYLIGEGKGPIDAYLDMDGIIDLAKRKKVDAIHPGYGFLAENAEFARKCEENGITFIGPSSKVMNMMGDKINSKKIAKEVNVQTIPGVEKAIRSTEEAKEVANKIGYPVMIKASNGGGGRGMRIVHREEDLELEYETACSESRKAFGEDIIFIEKYIADPKHIEVQILGDNYGNIVHLYERDCSVQRRHQKIIEYAPAFSLDDKVRKEICEDAVKLSKHVGYSNAGTLEFLVDANGGHYFIEMNTRVQVEHTVTEMVTGIDIVQSQILIAQGYSLDSEEINIKSQDDVEIRGYSIQCRITTEDPKNKFMPDTGKIQVYRTGSGFGIRLDGGNGFTGANISPHYDSLLVKTISWDRTFQGAINKTIRSIKELRVRGVKTNVGFLVNVLNNPIFSNGKCSTKFIDENPDLFEITESKDRGTKLLQFIGDVIVNDNACKEKPLFDALHDPRMDKDGSKSEGSKILFDKLGKSAYIEKIKNDKKLLLTDTTMRDAHQSLLATRIRTYDLLKAAKPTEKYQKDLFSLEMWGGATYDVAYRFLKESPWRRLQKLREEIPSIMFQMLLRASNGVGYKNYPDNVIEEFTKESARQGIDVFRIFDSLNWVENMKPSINTALETGKIVEATMCYTGDILDKTKTKYNLEYYIKMAQELESLGADIIAIKDMSGLLKPYSAYTLVKELKKNVKAPIHLHTHDTSGNGVATCLMASEAGVDIIDAALESMAGLTSQPSLNAIVEALKNTERDTGIDLFGYDELGKYYKDLRKVYNKFESDLTNSCAEIYNFEIPGGQYTNLKPQADSLGLVNRFDEVKEKYKEANEVVGDIIKVTPSSKVVGDLAIFMTKNKLDKDNIIEEGKNLSFPDSVVDYCKGMIGQPEGGIPKDLQEVVLKGEEAITVRPGSLLPAEDFDEIAKYLNEKYDINANIRNVISYALYPKVYEDYIKHLQHYNDISKLESDVFFYGLNKNEECEVEIEEGKVLTIRLVEIGEVKENGFRTIGFELNGMVREVEIKDKNFSGKINNVEKADMNDPLQIGASIPGKVIKIMVKEEDEVKANQPLIVIEAMKMETIIVAKTDGVIKSIKVKEDDMVEDKQLLMIMK*

>CD630_00220 Clostridioides_difficile_630_NC_009089 elongation factor G

MKVYDSKMLRNVAVLGHSGCGKTNLIETIAYTANTNKIPKLTDKVNMTYSMGLIPIEYNDYKFNLLDTPGYFDFSGDVVSSLRASDAAIIVIDATAPIQVGTEKSLELTESIPKIMFINKIDNEKARYKDAIAMLREKYNNKIVPMISPIYKDKNFVKLHNVFENIDDLEGEFKEQAMSVKEALMELIAETDDQILDKYFNGEELTTEEIQKGIIIGIQRGDIIPVICGSTINNIGTKEILDTISSYLEPIFTEESKPFRGLVFKTMVDPFVGKMSYIKITEGVLSKDKDVFNINKNVKEKIANIYTLRNSELVEIEKAKAGDIVVITKVNSLKTGDTISADKDAEALEKIDFPKPQIYYAVTPKNKGDEEKVASVLNKLVEEDPTLHWYRNTETKQALLGGQGELHIKTIKNKMKDKFGVDVELNDLKVPYRETIKGTADVQGKHKKQSGGHGQYGDVKIRFERCESDFEFTEEIFGGSVPKQYIPAVEKGLKDSMQKGILAGYPVTNIKATLYDGSYHDVDSSEMAFKMAASAAFKKGMEEAHPILLEPIMKLKITVPEEYMGDVMGDINKRRGKIFGMEPDDKGKQIIFAEAPQAETFKYAIDLRAMTQGRGYFEMELERYGEVPSQFAEKIIGLATAK*

>CDIF1296T_00085 Clostridioides_difficile_ATCC_9689__DSM_1296_strain_DSM1296_CP011968 transcriptional regulator

VILMATMTDIIEKFIKDLMEEDNSIQIQRNELANLFSCAPSQINYVLTTRFTIDRGYYIESKKGGGGYVQIEKIRKSKDGHIRELLNEKIGSQISYKKAKELLEGLKESDLINERELKLILYAIDDKSLCMPIYELKEKVRSNILKNIIIGLFSIEE*

>CD630_00240 Clostridioides_difficile_630_NC_009089 activator of protein kinase McsB

MLCQKCNKNKASVYYNKIVNGEKTEMYLCSECAKENTEMNFNLDMPFSMMDIFSNLGFQPKKELEEKLVCPKCNTTYSEFKNNGRFGCSECYNAFSSQVNPMLQNIHGHIEHTGKAPKKSFYKISVENEIKELKEDLDRAIKNEEYELAAQFRDKIKYLKGSID*

>CDM120_RS00440 Clostridioides_difficile_M120_NC_017174 AraC family transcriptional regulator

MNRNSFSHEIIEPIDNLDVNFKLFDDSGSYVANHWHNSLEIIYITSGDLQINMEGYTYNLKANECMFINSGIIHSTRCTYHNTSILLQVPMSFLNKYIPDFKNCYFDFKVNANDNNYKRNSSKVKLILENMREIKLSSPPAANLQFTSLLFELLFELYTNFKISVGNKNMKKTVLDLSKFEPVLEYTNINYKSPISINKIAKVAHLQPEYFCRKFKQYMGQTYLEYLNDVRISHIYKDLINTNDTLCSILETHGFTNTKLFYRIFKEKFKCTPKHIRKNLPKI*

>CD630_00320 Clostridioides_difficile_630_NC_009089 beta-glucosidase

VYIKGVNLGGWLVLEKWMTSSLFEGTEAEDEYYLPRQLSREAYESRIKTHRSEYITERDFATIKSMGFNSVRIPVPYFIFGDCEPFIGCVKELDKAFAWADKYGLSILIDLHTVPGSQNGFDNGGISGICSWSQNPEYVAFTLNVLERLAKRYGMRHELYGIQILNEPITERMWNIMNVPNRFKAVDKEMARGSKPNSLEFLRDFYIKAYRVMRPYMREENVIVFHDAFELKAWKDFMREEEFKNVVLDTHQYLMLAEADGCEQSIDSYLKYIRENYAKDILQMQKYFPVICGEWSLFNSYACGIDTNGGQSPLNGIESNIDKLSKDDKRELYRKIAKAQLDAWRNGSGHYYWNYKLLLDTVNEEGWIGWDSWDLGKCVAQEWYPIEY*

>PCZ31_RS00360 Peptoclostridium_difficile_strain_Z31_NZ_CP013196 PTS galactitol transporter subunit IIC

MEGLYMEILNFIVGLGAQVMMPIIICIFGLILGTKLGKSLRAGLTVGVGFIGLNTIIALLTDNLGPATQQMVKNLGLSLSIIDVGWPAASAIAFASTVGALIIPIGLVVNIVMLITNTTQTVDVDIWDYWHFAFTGALVAGATQSVMWGVFAAVANMVIVLVMADLTAPGIEEYLGMPGISLPHGFTQAFVPIAIVVNKLLDLIPGINKIEINADTLQKKFGLFGEPLIMGSVIGVIIGIAAKYDIKGILQLGVTMGAVLILIPKMAALLMEGLLPVSEAAQEFIEKRFKNRGKIYIGLDSAVGIGHPVTLSVALVLVPLTILIAAILPGNKVLPFADLAVIPFALVLIVPITKGNVFRTLIIGIIIITSGLLIATNLAPLFTQMALNASFKMPEGATMISSICDGANPLSWVFVKVMNYKVIGGVVFGVIALGMAIYNRNRIINENKKLSIEE*

>CDM68_RS00370 Clostridioides_difficile_M68_NC_017175 proline--tRNA ligase

MKMSKMFMPTLKEIPADAEITSHQLMVRSGMIKKMTSGVYNQLPMGLRVFKKIEQIIREELNKKDCQEILCAALLPSELWKESGRWTAMGEEMFRLKDRTEREYCLGPTHEEAFTDIIRQEITSYKQLPLNLYQIQVKYRDERRPRFGVMRTKTFTMKDAYSFDADDKGLDKSYQDMFDAYVSIFDRCGLENSPVQADSGAIGGSTSAEFMVKSEVGEDEVVFCSGCDYAANVERAESCNLASQKEEMKELEEVHTPGAATIKELEEFLKTSPDKFAKTLVYEADGKTVVVVVRGDREVNEIKVSNAIGSVIEFALATDDVVRKVTNAEVGFAGPIGINADYVFIDKEIVEQRNIVVGANKTEYHIKNANYGRDFEGIVGDFRNVQEGDKCIVCGKPLEIARGVEVGHIFKLGTKYSESMNANFIDKDGKSKPIVMGCYGIGVERTAAAIIEQHNDEKGIIWPLSVAPYHVVIIPANMKNEEQISIAENIYNDLQAMGVEVLLDDRDERIGVKFNDSELIGIPMRITVGKNINEGKVEFKLRHKEDKEIIDIEEINEKVKAEFIRNNVRLGQ*

>CD630_00520 Clostridioides_difficile_630_NC_009089 cysteine--tRNA synthetase

VKVYNTLTRTKEEFVPLEEGKVKMYVCGPTVYNYIHIGNARPFIIFDTLRRYLEYRGYDVTYVQNFTDVDDKIINRSHEEGISPEEVAAKYIKEYFVDCDGLGIKRATVHPQVTDNIQQIIEFIKELEDKGYAYAVNGDVYFDTNKFEGYGKLSGQKQEDLEAGARIEVNDQKRHPMDFVLWKAKKEGEPGWDSPWGEGRPGWHIECSVMSKRYLGETIDIHAGGQDLTFPHHENEIAQSEARSGKTFSKYWMHNGYININDEKMSKSKGNFFTVRDISKLYDLEIVRFFMLSAHYRNPVNFSDEMLNQAKAGLERLYNTKEKLEFTLSNLVESPLTEKEVELVKELDDFRQKFIDAMDDDVNTADAVSVIFELAKLINSNVDENSSLEFAKKCLDEFNELTGVLNIVNKKKDTVLDKDIEELIQKRTDAKKNKEFQLADDIRQQLLDMGIVLEDTRQGVKWKRI*

>CD630_00550 Clostridioides_difficile_630_NC_009089 23S rRNA (guanosine(2251)-2-O)-methyltransferase RlmB

VNNLASIEGRNPVIEAIKSDREIDKILIANSAKEGSIKKIIGMAKDKNIIIQYVDKHKLDEVSTSHSHQGVIAYASEYKYYELDELIDLAKNKDEDPFFIILDEITDPHNLGSIIRTADAVGAHGVIIPKRRSVHITPVVAKASAGAVEYMPVCKVTNIVNTIKRLKEEGLWIAAADMDGETFYKQNLTGPLGVVIGSEGFGISRLVKQNCDFIVKMPMIGNVTSLNASVAGGILLYEIFRQRLDKSK*

>CD630_00600 Clostridioides_difficile_630_NC_009089 transcription termination/antitermination protein NusG

MSELQEASWYVVHTYSGHENKVKATIEKAVKTRGMEDCIRQVVVPTEEVVETTKTGKEKTRQRKVYPSYVLVKMIITDESWYVVRNTKGVTGFVGPGSKPVPLSEDEVKAMGIDTTDPKVVNSDIDFEIGDTVKVSQGPFSGQIGNIEEIDLENREVKVCINAFGKRTLFVIELEGIEKI*

>CD630_00650 Clostridioides_difficile_630_NC_009089 NADP-dependent dehydrogenase

MEKLQGKIAVVTAATKGIGLASAEILAKNGATVYLAARSEELAHEVINKISAEGGCAKFVYFNAREEETFTSMIEEVVKKEGKIDILVNNFGSTNPSLDKDLVTGDTDNFFDTVNTNLKSVYLPCKAAIPHMIKNGKGSIVNISSIGSVLPDLSRIAYCVSKAAINSLTQNIATQYAKDNVRCNAVLPGLIATKAALDNMSPEFIKEFLKHVPLNRIGEPDDIAKAVLFYASDDSSFITGDLLEVAGGFGLPTPQFADNILG*

>CD630_00720 Clostridioides_difficile_630_NC_009089 30S ribosomal protein S10

MAKNEKIRIRLKSYDHKLLDFSAGKIVETAKKAGSQVSGPVPLPTEKQVVTILRAVHKYKYSREQFEIRTHKRLIDIANPTPKTVDSLMRLDLPAGVDIEIKL*

>CD630_00730 Clostridioides_difficile_630_NC_009089 50S ribosomal protein L3

MKGILGKKVGMTQIFTDKGVVIPVTAVEAGPMVVTQIKTVDKDGYNAIQIGFEDAKEKALNKPKKGHLAAANVLKKHLKEFRVDSVEGYTVGQEIKADVFEAGAKIDVTGISKGKGFQGPIKRHGQSRGPETHGSRYHRRPGSMGACSYPGRVFKNKKLAGHMGSVKVTVQNLEVVKVDADKNLILVKGAIPGAKGSVVTIKEAIKVSK*

>CD630_00760 Clostridioides_difficile_630_NC_009089 50S ribosomal protein L2

MAIKKFRPTSPALRQMTVLVSDEITCNQPEKSLLVNLKKNAGRNVHGRITVRHRGGGQKRKYRIIDFKRDKDGIPAKVATIEYDPNRTANIALLNYADGEKRYILAPVGINVGDTILSGLGADIKPGNCLALKDMPVGTIIHNIELKPGKGAQLVRSAGVSAQLMAKEGKNALLRLPSGEMRLVSINCKATIGQVGNIEHGNVVIGKAGRKRHMGIRPTVRGSVMNPNDHPHGGGEGRSPIGRPSPVTPWGKPALGYKTRKKNKASNKLIVSRRTK*

>CD630_00770 Clostridioides_difficile_630_NC_009089 30S ribosomal protein S19

MSRSTKKGPFVHARLLKKIEAMNASGNKEVIKTWSRSSTVFPQMVENTIAVHDGRKHVPVYITEDMVGHKLGEFVPTRTFKGHKDDEKSNKRK*

>CD630_00790 Clostridioides_difficile_630_NC_009089 30S ribosomal protein S3

MGQKVNPHGLRVGVIKDWDSRWFATDKKEFGNLLLEDHNIRKFLKKRLYSAGVAKIEIERSANKIKMDLHVAKPGVVIGRAGAGIEALKAELEKMTKKTIIVNIVEVRSTDKNAQLVAENIALAIERRVAFRRAMKQAIQRAMKSGAKGIKVSASGRLGGAEMARTEGYSEGNVPLQTLRADIDYGFAEADTTYGKIGIKVWICNGEVLPTRDGVNPREESRKSDRRDNKRDNRRNDRRGNDRRGNDNRGNYRGQRPQGGSRPQRTENKGN*

>CD630_00880 Clostridioides_difficile_630_NC_009089 30S ribosomal protein S5

MLRRKPIDAGQLDLQEKVVEVRRVTKVVKGGRNFRFAALVVVGDENGHVGIGAGKAMEVPDAIKKAVEDAKKNLIVVPIVGTTIPHEVRGHFGAGNILIMPAVEGTGVIAGGPARAVLELAGLKDVRAKSLGSNNPRNMVNATIEGLNSLKTVEDIAKLRGKKVEELLG*

>CD630_00890 Clostridioides_difficile_630_NC_009089 50S ribosomal protein L15

MKLHELKPAEGAVRAKRRLGRGTATGQGKTAGRGQKGQWSRSGGGVRVGFEGGQMPLARRLPKRGFNNIFKKVYTEVNVEVLNRFENGTEITAELLKSTKTISKIGKDGIKILGEGNLEKALTVKAAKFTASAQEKIEKAGGKAELV*

>CD630_00910 Clostridioides_difficile_630_NC_009089 adenylate kinase

MRIILLGPPGAGKGTQAVGIVEKYNIPHISTGDIFRKNIKEGTELGKKAKEYMDQGLLVPDELTVGLVTDRISQEDCKNGFMLDGFPRNVAQGEHLDIFLKNAGISLDKVVNIEVDKSILVSRAVGRRICKSCGATYHVEFNPPKVEGVCDVCQGELYQRADDNEETVSKRIQVYLDETKPLVDYYSKQGIIADIKGDQAIDKVFEDIVAALGSGK*

>CD630_00950 Clostridioides_difficile_630_NC_009089 30S ribosomal protein S13

MARIAGVDLPREKRAEIGLTYIYGIGKATANEILAKAEINPDTRIKDLSEDQVNELRKVIDDDFLVEGDLRREIALNIKRLRDIKCYRGIRHAKGLPLRGQRTKTNARTRKGPRKTVSRKKKK*

>CD630_00970 Clostridioides_difficile_630_NC_009089 30S ribosomal protein S4

MARYTGASCRQCRREGMKLFLKGDRCYTDKCAIVKRNYAPGQHGQGRKKVSNYGLQLREKQKVKRIYGVLETQFRNLYERAENMPGKAGENLLSLLERRLDNVVYRMGLASSRKEARQLVTHGHFTLNGNKVDIPSLIVKVGDVIEVKEKSRSSAKFKNLVEVNSRIAPKWLEANVEGMTAKVVGVPTREDIDLEIAEHLIIELYSK*

>CD630_01000 Clostridioides_difficile_630_NC_009089 cobalt ABC transporter ATP-binding protein

MDNIVKVNNISFEYITDEAKLKAIDNLSLDVKKGEFVAIIGHNGSGKSTLSKNLNAILMPTEGNILIDDMDTKEEERLWDIRQTAGMVFQNPDNQIVATIVEEDVAFGPENLGIEPKEIRRIVEESLKSVGMYDLRDRQPHLLSGGQKQRVAIAGIIAMRPKCIIFDEATAMLDPSGRKEVMKTIKRLNKEENITVIHITHFMEEAVEADRVVVMEKGKKILEGTPREVFSKIKMLKEIGLDVPCMTELSSLLIEEGINISSDILTVDEMVMELCQL*

>CD630_01010 Clostridioides_difficile_630_NC_009089 cobalt ABC transporter ATP-binding protein

MSIIVKNLTHIYNEGMPFASKALDDVSFEIKDRDFVGLIGHTGSGKSTLIQHLNGLLKPSSGEIFINDFNITDKNLNLTEIRKRVGVVFQYPEYQLFEETIDKDIAFGPSNLGLEESEIHNRVKASMEAVGLDYEGFKDKSPFELSGGQKRRVAIAGVIAMNPEVLILDEPTAGLDPGGRDEIFNLIKDLHEKKNMTIILSSHSMDDMAKLAKTLIVMNHGSVEFMGTPREVFKSNASKLKDIGLDIPQVLELALKLREKGFDISEDILTLEEAKQEILKVVRGRGLC*

>CD630_01050 Clostridioides_difficile_630_NC_009089 30S ribosomal protein S9

MANVQYYGTGRRKSSVARVRLVAGEGNILVNGRALENYFNYETLIRDVKQPLVLTGNENKYDVIVKVEGGGFTGQAGAIRHGISRALLKADLDLRPALKKEGFLTRDARMKERKKYGLKAARRAPQFSKR*

>CD630_01140 Clostridioides_difficile_630_NC_009089 hypothetical protein

MITNDKRIRIITGHYGSGKSEFAMNYVVKLRDMVSGKVAIADLDVVNVYFRTREKKELMKSLGIQPIDSSINAPTLDLPAVSAEVMSPMVDHSYNSVIDLGGDNVGARVIGRFSHLLKEGDYDMLFVINANREKTQTSEEVIQYIKEIEKSSKLKVTGLINNTHLIRFTTIDDVLRGQKVAKEVSEKCNIPIRYVACLENLVEQLPKDLEGEIFPIKLYMREDWM*

>CD630_01170 Clostridioides_difficile_630_NC_009089 ferredoxin/flavodoxin oxidoreductase subunit beta

MAVVFKKTEGLQDTQTHYCPGCTHGIIHRLVGEVLEELGVLGDAVGVVPVGCSVLGYKYFNCDTQEAAHGRAPAAATGIKRVHPENTVFTYQGDGDLASIGTAEIVHAAARGEKITTIFVNNTTYGMTGGQMAPTTLVGQRATTAQSGRNAETQGYPIRVSEMLATLTGAVFVERVAVDTPAHVRQAKKAIKKAFQVQQAGLGFGIVEVLSTCPTNWGLAPNDALQWLRDNMIPYYPLGNFKNVEVEEVK*

>CD630_01190 Clostridioides_difficile_630_NC_009089 phosphoglucosamine mutase

VRKYFGTDGVRGVANTELTCDLAYKLGRAGGFVLAQGDHRVKVVVGKDTRISGDMLEASLIAGLMSVGCDVITVGIIPTPAVAYLTRKYGADCGVVISASHNPVEYNGIKFFNKNGYKLDDEIELKIEEYIDDIDKIDCLPIGENVGRKLHEHCAQRDYVDYLKSIISTDFKGLKVVLDCANGASYKVAPIVFDELGASVISINSSPDGNNINYKCGSTHPEQLQRAVLEHNADLGLAYDGDADRLIAVNEKGQIVDGDHIMILSALNLKKNNKLAQDTLVVTVMSNIGLTIAAKENGINLSTTAVGDRYVLEDMVKNGYNLGGEQSGHMIFLDYNTTGDGVLSSLILANIILQEKKPLSEIASIMSQYPQVLVNATIKNENKNKYMEYPEIKTEIERIESILDGNGRVLIRPSGTEPLVRVMLEGKEEGQIKELATNLANLIQEKLS*

>CD630_01210 Clostridioides_difficile_630_NC_009089 thiosulfate sulfurtransferase

MTNDFKKEKKEDYFVNLKAISTEVLQEKVQDNAWVIVDTRLNDAYNGWKLDGVKRGGHIKGAVDFSANWLSVYSDRKDEVLEQALKTKRIDLDKNIVLYDANGKDALVVADYLSKKGYKYLYKYDIKQWADDENLPMERYKNYQMIVPAFIIKDILDGKIPETFEDSKNIKMIEASWGEESYTKGHIPTSVHVNTDIIEPPPTWMLDNDDNLTKFALDYGLTKDDTVIVSSSTPMASYRLAVILRYIGVKDVRVLNGGTNSWLSAGYELEFISNPKHSCTNFGADIPVNSQLIVTTSELRQKLKEKNKFILVDNRTWDEHIGKVSGYTYYDKKGRIPGALYGHSGSDSVSLEEYRNIDNTMRNKYEILEMWDKENIDVNKQLIFMCGSGWRAAEVLTYANVIGVENTSLYSDGWMGWSLDNSNLIEVGEHK*

>CD630_01250 Clostridioides_difficile_630_NC_009089 cell wall endopeptidase

MKKKLLEKDGFYLSLFVCVCLLAVGGVWFTNNNVDKLASNKGIMENANKDSEEEIHLIEKDKKDAIPTATDSKQNLEKAKSKEENKSSTTKLNYIGDKVIRGYSEKEPSYSKTLDVWETHKGVDISCTKGKEVKSLLNGIVVDVFDDEEYGQSVKIKSDNNIVVVYSNLDKNVSVKKEQKVTEGQSLGTVGSTSQIESEEGIHVHLEAYSGEKSIDPMSLIK*

>CD630_01300 Clostridioides_difficile_630_NC_009089 S-adenosylmethionine synthetase

MARHLFTSESVTEGHPDKICDQISDSILDALLEKDPQSRVACETTVTTGLVLVAGEISTSAYVDIPKLVRETVREIGYTRAKYGFDCDTCAVITSIDEQSGDIAMGVDEGLESKTGEEIEEEIEKVGAGDQGIMFGFACNETPELMPLPISLAHKLSRRLTEVRKTGLVDYLRPDGKTQVTVEYEGSKAVRVHTVLISAQHCETVSNDKIREDLINHVIKEVIPAELLDEETKIYINPTGRFVIGGPQGDTGLTGRKIIIDTYGGYSRHGGGAFSGKDPTKVDRSAAYAARYVAKNIVAAGLADKCEIELAYAIGIARPLSIFIDTFGTGKVSEEKLVELVNKHFDLRPGAIIRDLDLRKPLYKKVAAYGHFGRTDIDLPWERTDKVEQLRKDALGE*

>CD630_01400 Clostridioides_difficile_630_NC_009089 peptidase

MNIISNKIDELKEDLLSDIIDIVKIPSVKGESENGFPFGEKVGEALNKALEISEKLGFKVRNLDNYIGYAEHGDSDDYVCVIGHVDVVHEGDGWKHQPYKGEETNGRIYGRGVLDNKGPIMSALYGLYAIKELNLKLDKSVRIIFGTNEESGFEDIPYYLEKEKAPIMGFTPDCKYPVVYGEKGMAKIRIKSKINYEEDVYLGFIENMSENVLVTYKELNIENSDTILDIKVKYDFSYKLKDVLDEIKASFPNSIDIEVISNFNPVYFDKESNLVKKLQLAYERVTSLDGTPVTTNGGTYAKVMPNIVPFGPSFPGQKGIAHNPDEYMDIEDIILNAKIFANAIYELAKE*

>UAB_RS0201575 Clostridioides_difficile_ATCC_43255_NZ_CM000604 peptide chain release factor 2

LMKNIQINEEKMNQQDFWNDNEVAQRVLQENKSLKETLEEYESLKSLLEDIEVLIEIGLEEDDDSVERDIEKSIESMEEKLSEMKIKTLLNGEYDKNNAILSINAGTGGLDAQDWAQMLLRMYIRWSESKGYKVKLLDIISDPEAGIKTATILVEGTNAYGYLKSEKGVHRLVRISPFDPSGKRHTSFASIDVTPELDENIEVEINPSDLKIDTYRASGAGGQHVNTTDSAVRITHIPTGVVVQCQNERSQHLNKDRAMRLLMAKLIELKELEQKEKIEDIQGKYSQITWGSQIRSYVFQPYKLVKDHRTNAEFGNVDSVMNGNIDLFINEYLKMNKIV*

>CD630_01450 Clostridioides_difficile_630_NC_009089 S1 RNA-binding domain-containing protein

LDINQILKKEFNLRDEQINNTLKLIDEGNTIPFIARYRKEMTGEMSDVTLREFYEKLMYLRNLQSRKDDVVRLIDEQGKLTDEITQNIEKAKTLQEVEDIYAPYKQKKRTRATIAKEKGLENLALSILENNLDNIEIEAKNYLDEEKEVLSIEDALKGARDIIAELVSDDAKIRKYIRELALREGMIVSKSATDEKSVYDMYYDYSEAVKSMAPHRVLAINRGEKESFLKVKLEINNDKVLNYIINEYVNDKNFKNKEEIVSSIEDSYKRLIFPSIEREIRNHLTEIAQERAISVFGKNVKSLLLQPPVKDKVVMGFDPAFRTGCKIAVVDKNGKLLDYTTVYPTDPQNDVEGAKKVLKGLIEKYDIDIISIGNGTASRESETFVSEMIKEIDSEVQYVIVSEAGASVYSASELANEEHPDINVSIRGAISIARRLQDPLAELVKIDPKSIGVGQYQHDLNKKRLEEVLDGVVEDSVNSVGVDLNTASYSLLEHVAGISKAIAKNIIAYREENGDFTSRAQLKKVKRLGPQAFTQCAGFMRILEGKNPLDNTGVHPESYDICKKMIEIIGYSLDDVKNKNIGEIDEKIKEIGLRELSEKLEVGQVTLKDIIAEIKKPGRDPREEGIKPILRTDVLKIEDIQEGMTLKGTIRNVVDFGAFVDIGIKNDGLVHKSEMSNSFVKDPMSIVTVGDIVDVKVIGIDLNKKRVALSMKK*

>CD630_01460 Clostridioides_difficile_630_NC_009089 hydrolase

MIFIKNGKINTITNGIIHGDILIDEGKIIEIGEDLIAPLDVEVIDASNKLVFPGFIDAHTHLGLWEDGIGFEGADGNEETDPITPQLNPIDGINPMDRTFKEAFEGGITSVCTTPGSANVMGGQCIAIKTCGKRIDKMVIKNPVASKIAFGENPKSCYGQDDKSPQTRMAIAALLRENLKKAEEYLEDIDMYESHDDEDCEKPEYDIKMESLIPVLRREIPFKAHAHRADDMFTAIRIAKEFNLKLTLDHCTEGHLIVDELVEEEFPVIVGPSLSERSKFELRNLTFNTAGILSNAGLDVCIMTDHPVIPVQYLPICAGIAVKHGMKEEKAIESITINPAKTLGIEDRVGSIEVGKDADLVIWDNSPLEIQSNVLYTIINGKVVYEKK*

>CD630_01490 Clostridioides_difficile_630_NC_009089 P-loop ATPase

MAKIYLENENKTREIGYKLGKLLKEGSVICLVGDLGAGKTTMTQSLADSLGIEDYITSPTFTIINEYEGKIPLYHFDVYRIGSSDEMYDIGYDEYVNSNGICIIEWANLIEDILPKEYLNIELRYKDEGREMILTPKGEFYKEIVEELIK*

>CD630_01520 Clostridioides_difficile_630_NC_009089 UGMP family protein

MSDIITLAIESSCDETAASVLKNGREVLSNIISTQIETHKKFGGVVPEVASRKHVENIDIVVQEALDKANIGFNDIDHIAVTYGPGLVGALLVGLSYAKALAYTLNIPLVGVNHIEGHLSANYIEHKDLKPPFITLIVSGGHTHLVEVKDYGKYEILGKTRDDASGEAFDKISRAMNLGYPGGPIIDNLAKNGNKHAIEFPRAYLEEDSYDFSFSGLKSSVLNYLNGKRMKNEEIVVEDVAASFQEAVVEVLSTKALKAVKDKGYNIITLSGGVASNSGLRAKITELAKDNGITVKYPPLILCTDNAAMIGCAGYYNFINGKTHDMSLNAVPNLKINQ*

>CD630_01530 Clostridioides_difficile_630_NC_009089 4-hydroxyphenylacetate decarboxylase large subunit

MSQSKEDKIRSILEAKNIKSNFQNKENLSEFNEKKASKRAEDLLDVYYNTLSTADMEFPYWYNREYRKSDGDIPVVRRAKALKAAFSHMTPNIIPGEKIVMQKTRHYRGSFPMPWVSESFFVAQGEQMREEAKKLASNTADELTKFGSGGGNVTESFGNVVSIAGKFGMRKEEVPVLVKMAKEWVGKSVEDLGFHYEKMMPDYDLKENLMSTLICMFDSGYTLPQGREVINYFYPLNYGLDGIIEMAKECKKAVAGNASGDGLIGMDRLYFYEAVIQVIEGLQTWILNYAKHAKYLESIETDLEAKKEYSDLVEILEHIAHKQPRTFREALQLTYTIHIASVNEDAISGMSIGRFGQILYPWYEQDIEKGLITKEEVIELLELYRIKITCIDCFASAGVNGGVLSGNTFNTLSIGGLKEDGSTGANELEELLLEASMRCRTPQPSLTMLYDEKLPEDFLMKAAECTKLGSGYPAWVNNSNGTTFMMKQFADEGMTVEEARAFALGGCLETSPGCWKQLTLNGKTYSIAGGAGQSAGSGVHFIANPKILELVLMNGKDYRMNIQVFEPHNKPLDTYEEVIEVFKDYYKQAINVLERANNIELDIWRKFDTSIINSLLKPDCLDKGQHIGNMGYRYNATLNVETCGTVTMVNSFAALKKLVYDDKAFTIEEMKDAILNNFGFKDALEVGNYSMADQVKVDKTGKYDAIYKACLDAPKYGNNDLYADNILKNYEVWLSKVCEEAQSLYAKKMYPCQISVSTHGPQGAATLATPDGRLSGTTYSDGSVSAYAGTDKNGVYALFESATIWDQAVVQNSQMNLKLHPTTIKGQQGTKKLLDLTRSYLRKGGFHIQYNVVDSETLKDAQKNPDNYRQLMVRVAGFTQYWCELGKPIQDEVIARTEYEGV*

>CD630_01690 Clostridioides_difficile_630_NC_009089 MATE family drug/sodium antiporter

LQELFSLKDENKRFYKILLSLCIPIIIQNLISTSVNVIDTIMISSLGETSVASVGVANQFFFLFNMSLSGITGGAGVFISQFYGKKDVSNIRKVTGLTCVLAIVLSFVFVIPALLTPKPIIHIFSYDSEVVKLCIDYFSIAVFSYPLIAVSTVFSTGSRGVRNPKLGMICSAFALVTNVILNYGFIFGNFGLPALGVKGAALATVIARICELILMITYVYLYKKDYILKFGLKNLKAIDKIFIKSFSSKSFPIFVNDSVWAIGTVLYSVAYARAGTSAIAASQIATSTGNFFIMTAVCIASGASIMLGNELGADHIKRAIEYAKKFSILVFSAGLILGIILILNIPLLLKMFSVSDSLASDITKIFFIMGILMALKSFNTLVIIGILRSGGDTKYALFLELGCMWLASIPLTFIAAFKGAPIFVLVLLTYSEEVVKFIFGVPRALSKKWAINIVKEID*

>CD630_01700 Clostridioides_difficile_630_NC_009089 ABC transporter ATP-binding protein

MIVLSCNNLNKSFGIDSILENISFTVNEGDKIGIIGVNGTGKTTLFKIISGIYGYDSGDIYTSKDCEIGYLEQNTNFYSDNTILEEVLEVFKNLIEMESYLRELEVKISEESTKTNSPIIEKIMDEYSHKLELFSDLNGYGYKSEAKGVLKGLGFSDNDMDKPISILSGGEKTRVLLGKLLLKKPTLLLLDEPTNHLDSEAIEWLEVFLKQYKGTVMLISHDRYFLDQSVNRIFEVHNKKLKVYNGNYSKFVELSKIEKELELKKFEDQQKEIKKQEESIERLKAYGREKHLKRARSKEKALDKVDVLDKPEAYRKKARIQFTPSVQSGNDVLQIRDVSMGYGERILFKDLDLDIYRGEKVALIGANGVGKSTLFKIITNELQPLSGNIKFGTNVHVSYFHQEQKTLNLDNTIIDEIWENNTHLTQTTLRNMLGAFLFVDEEVFKKISTLSGGERARVAILKLILSNANLLLLDEPTNHLDIDSKEVLEEALTNYDGTIFTISHDRYFLNTVVDKILVLDENGITEYLGNYDYYIDKKRQIQEMSIIEEKEEKTRTQIKDEKRKEREQREIEKKNRIKRQNIEKEIEKLEIEIEKLDILLCQEEVYSNPDKAKEVSQEKINLENNLASLYDEWEEFM*

>CDIF1296T_00297 Clostridioides_difficile_ATCC_9689__DSM_1296_strain_DSM1296_CP011968 oxidoreductase, electron transfer subunit

MWNAMHRIFINKDLCTGCKSCVLACMLKHNKDYDMYTLDLENIDNDSRGHIELDSKHNNPVPILCRHCDEPECVLACMSGAMHKDSESGIVSYDEEKCGSCYMCVMSCPYGLLKPDDRSKQNILKCDLCKDEEYPRCVANCPSGAIELQKEENDELCSVRS*

>CD630_01760 Clostridioides_difficile_630_NC_009089 oxidoreductase NAD/FAD binding subunit

MNYVVLGASAAGINAVKTLRELDKDSNIVVISKDENVYSRCMLHHVISEHRTLKQINFVDEDFMEQNNVKWIAGKTVKGIDINKKVVQTEDITVNYDKLLIATGASSAIPPIKNLRDGNFVYSVRNIDDIYKIKEKAQNSKNVVIIGAGLVGIDALVGLFKYEQLNISVAFMEKYILDRQLDEYTASVYENKFKEKGVKFYPSASIQEIVLDNSKNVTGVAFSNGEVLDADMVIVATGVKPNADFLDGTGIEYDRGIIIDDMCQTTQKDIYAAGDVVGKNAIWPLAVKQGIVAAYNMVGKDKKIEDEFAFKNSMNFMDIPTISIGMNTPVDDSYKVLTRHGINDYKKFVFKDNVIYGAVIQGDISYVGVLTYLIKNKVEIYDLENRIFDICYADFFNIKENGEFCYSV*

>CD630_01850 Clostridioides_difficile_630_NC_009089 dihydroorotate dehydrogenase electron transfer subunit

MYKILENIYIGEDMYRMKVKGNFEGKMGQFYMLRAWDTYPVLSRPISIHDIDEEGITFLYKVVGEGTQILSNLKVNDNIKLEGPYGNGYAKVDGKVALVGGGIGVAPLYLVAKNIKNCDAYLGFREDVILEDEYKQVCNKVYTTVGNTFVTDIIDVEKYDYILTCGPTPMMEKLVKMVEGTKTRIMVSLENHMACGVGACLVCTCKTNGGNKKTCKDGPVFWGEDVIFNG*

>CD630_01920 Clostridioides_difficile_630_NC_009089 cardiolipin synthetase 1

MGVIGTIFLFYLIISYLAGAIISVIILLENRDPAKTMSWLLMFIIFPGVGLMIYAISGRNIRKRKLFKTQKLANNIKEKKLFDTLEKITEIVELEKESIKQNKLLRDEEDGSYRKRVINMLLKTGMFPFTKNNKVDVFVDGNEKFKRLIEDIREAKDHIHLEYFIIKDSEIGRVLKEELIKKAKEGIKIRILYDDVGCWRFWFNRKFFREMREVGIEIAAFLPTKFPIIGGKLNYRNHRKIVVIDGIIGYTGGINIGDEYLGKNDKFGYWRDTHIRIKGISVYMLQMTFLIDWYYTTKEVLVTKNYFPSVGNVGESMIQVVASGPDSDWEDIHYAYFSAICQARKNVYIETPYFIPDESLLKAIKSAALSGVDVRIIFPKIADHKIVNIASYSYFEEILRAGGKVYLYNKGFIHSKVVIIDDKIASAGTANMDLRSFMLNFEVNAFIYDEEVIRVMTDDFFEDLSYCEELNLEVFKNRNIIQKIKESVARLFSPIL*

>CD630_01940 Clostridioides_difficile_630_NC_009089 chaperonin GroEL

MAKEIKFSEETRRALEAGVNKLADTVKVTLGPKGRNVILDKKFGSPLITNDGVTIAKEIELEDRFENMGAQLVKEVATKTNDVAGDGTTTATVLAQAIIREGLKNVTAGANPILLRKGIQKAVTVAVEELKNQSRIVETQEAISQVASISAGDEEVGKLIAEAMEIVGKDGVITVEESQTMNTELDAVEGMQFDRGFVSAYMVTDVDKMEAVLNDPYILITDKKISNIQELLPVLEQIVQQGKKLLIIAEDVEGEALSTLVVNKLRGTFDVVAVKAPGFGDRRKEMLQDIAILTGAQVISEELGYDLKEADLSMLGRASSVKVTKESTTIVDGSGDKKAIEDRVTQIKHQVEQTTSDFDREKLMERLAKLAGGVAVVKVGAATEVELKERKLRIEDALNATRAAVEEGIVAGGGTAFVSVIPAIGTLIESLEGEVKLGAQIVKKALEEPLRQIAINAGLEGAVIVQNVVNSEAETGFDALNEKYVNMIEAGIVDPTKVSRSALQNAASIASTFLTTEAAVADLPEKEDAGMPGMGGGMPGMM*

>CD630_01980 Clostridioides_difficile_630_NC_009089 GMP synthase

MKHELVLVIDFGGQYNQLIARRVRENNVYCEILPCTASIERIKEKNPKGIIFTGGPNSAYLEDSPTISKEIFELGVPILGICYGIQIMSHVLGGVVRKGNKQEKEYGKTAITYGKSSLFEGITTNSVWMSHTDLIEKVPEGFTIVANTNDCPVAAMENVERNLYGVQFHPEVEHCLEGDKILTNFLYNICKVKGDWTTDSFIEDKIKELKEKIGDKKALCALSGGVDSSVAAVLIHKAIGDNLTCIFVDHGLLRKNEGNDVERIFREKFDINLIRVNAEDRFLSKLKGVSEPEAKRKIIGEEFIRVFEEESNKLGKMDFLVQGTIYPDVIESGHGNAATIKSHHNVGGIPEDVDFQEIVEPLRELFKDEVRKIGLELGIEEGLIFRHPFPGPGLGIRVIGDVTKEKCDILREADAVYMDELRKAGLYREIWQAFATLPDVKTVGVMGDERTYAYLVGLRAVTSSDGMTSDWYKMPYDVLERISNRIINEVDGVNRVVYDITSKPPGTIEWE*

>CD630_02030 Clostridioides_difficile_630_NC_009089 UvrABC system protein A 1

MKINNYKNQSIITNPKKFENKYQDLPKTPIELLKVVQSLVIHGDQGKLYGISFNKQQSDEELLRTIPQMLKRIFEINSNPLTIPRNPKQRLVGMCRDYSLLLVSLLRYRGFEARMRAGFANYFESELTYEDHWLVEYYDTLKKRWIRIDAQIDDIQKNYFQINFDTHDVGKTDGFLTGSEAWIRCQQGHAHPDDFGYNKNWKGWHSVKGNLLHDFNNMIGLELLPWDLWTELSSKKYNQLTRAEKNLLDEMAEILSSGNIKIEDLNLLIEKLPEDYLKSIFSQLKILGISEIKELGNPLELEKKFKFTKSINKSIKNSLCHNKSSIYLKGGRQNNLKDVEVTIPKNQITVITGVSGSGKSSLAFDTIYEEGKRRYFENLSNGAKLSEQLQKPEFDLLQGLTPTIAIEQKKGSQNPRSTVGTLTSIWDYLRMLFVSIGKSYCPYCKIPLEKKNNTKNYCPHCQTIFSKINTSTFNANSHTGACHDCNGLGFTYQVNPQLIVKDPTISILDGATYYFGKLRGKKPNGNWMVGELYAIAKDKNIDLDIPWNELPRDFIDAILYGTDDKIYEFSFESKGRESKIRRPASGAINHIQRLFRESSSENNTLHQYMNKIPCNTCGGELLCIEARFTTIKGYRFPELTKMTIEQLWNWLCELPNQLQKNELSLVNDILTELKIRVSYLLKVGLSYISTDRTAPTLSGGELQRVRLSSQLGSELVGLTYILDEPSIGLHPRDHNLIIKMIEELRDKGNTVIVVEHDKDTILSADYIIDVGPSAGTKGGFIIAEGTTQEIIKNPNSITGKYLSTYNKTGSQNKTIPSKWLSLKGCHANNLKNIDVEIPLNCMCSITGVSGSGKSSLVFHSLLPALEEKLKQKSIPDKNYTEFTGFDAIDDFILMDQTPIGKSSRSTPATYINIFDEIRSLFAETPQAKQKLLDESYFSFNSKKGQCPNCQGLGKTKIILQYMADQWVTCSECQGKRYQKEILSIQYKGKTIADILDMEVAEAKTFFSDCSDIYRKLSLLDEVGLGYLKLGQNTLGLSGGESQRIKLAKELGTKTKKRMLYILDEPTTGLHFKDIENLLITFRKLVNEQHSLLIIEHNTEVIRASDWIIDIGPDSGINGGEIVASGTPDEIKINPNSITGHFI*

>CDIF1296T_00419 Clostridioides_difficile_ATCC_9689__DSM_1296_strain_DSM1296_CP011968 hydrolase

LINLIFDVDDTLYNQLTPFYTAYNKVFSSIKDISIEDLYMSSRKYSDEVFHMTENGEMPIKEMHIYRIMKAFEELGNSITEKDAQSFQDEYIYQQSQITLIPEVERILNFSKERNINLGIITNGPSNHQRMKLKQLNIENWVDKSNIFISSEVGFSKPDTNIFRVAENVMNLDRENTYYVGDSYRNDVLGAKKAGWKSIWLNHRGHEVEELFYKPDFVILEHKDLISLFIKICSYKNM*

>CD630_02130 Clostridioides_difficile_630_NC_009089 spore coat protein

MRNMIENLIKNNTYIDDKVIMLSMLSSAKASANMYLNSALTSSTPELRAIYSASLTQMVEGHTALTELSINKGWVKPYDKPIQQLTCSYKESQNVID*

>CD630_02170 Clostridioides_difficile_630_NC_009089 nitroreductase

MLEVIKNRHSIRTYIDKNIEEDKITEILKSAMQAPSSKNAQPWEFIIVDDKELLKQLSKSQHRAKHIEFAPLCIVVLGNRDKFLKPGKWIQDLGACTQNLLLEVTNQGLAACWAGVFPKNKVVNKVRQTLDLPLKLVPYALISIGYSEEKNEFIDRFDENKIHRNVYKNR*

>CD630_02180 Clostridioides_difficile_630_NC_009089 5-(carboxyamino)imidazole ribonucleotide mutase

MKVAVVMGSKSDYPKLEEGIKLLEKYGIEVVARALSAHRTPEQLSIFLKEIEDDTDVIIAAAGKAAHLPGVIASQTLIPVIGLPIKSSTMDGLDSLLSIVQMPKGIPVATVTIDLGLNAALLALQIMTLKYPKLKEDLKSYREEMAQKVLEDDKNLRG*

>CD630_02200 Clostridioides_difficile_630_NC_009089 amidophosphoribosyltransferase

MCGVLGIYSNKDVTKELYYSLYSMQHRGQESCGLALLDDGEIKYKKDMGLVGDVFKENELSKLKGNIGIGHVRYSTAGGSHVSNCQPLVGSCRKRQLAIAHNGNLVNANYLKDMLEEDGYMFQTNSDTEVILYILARYYKGDIVESLKVTMDYIKGAYALVIMSQEELVAVRDPHGFRPLVLGKKGDEYIFASENCAIDILGGEVIRDVEPGEIIVVKDGELKSYFYSENYKPVKKSCIFEHIYFARNDATIDNVNAYEFRIKCGERLAQNETVKADMVVPVPDSGWPGAIGYANASGLKISEGLVKNRYVGRTFIKPTQEEREIAVKIKLNPLSTIIKGKSIILVDDSIVRGTTSKQLVKSLREAGAKEIHLRITSPPVAYSCYYGIDTPNRSKLIASSNNVEEMREYIGCDSLKFLDIEGMLDATEHKSTFCKACFDGEYPVKKIDKEELLSC*

>CD630_02220 Clostridioides_difficile_630_NC_009089 phosphoribosylglycinamide formyltransferase

MLNIGVLISGGGTNLQAVIDGTESGEIKGQVKVVISSKQGAYGLERAKNHNIKAICETDEDKIIEILKENKIDLVVLAGYLKIISPKLVNEFRNKMINIHPSLIPSFCGAGFYGEKVHQGVIDYGAKVTGATVHFVDEGADTGPIIMQDVVKVNQDDDAKTLAKRVLEVEHRILKESISLFCENKLKLQGRRVFINE*

>CD630_02250 Clostridioides_difficile_630_NC_009089 phosphoribosylformylglycinamidine synthase

MLNTENKDSMVRRVLVEKREGFDLEAKALKKDLVESLHIDNIENLRILNRYDVEGISEEVYENAAKTIFSEPNLDVVYYEEIPKLNDERVFAIEFLPGQYDQRGDWAAQCVQIVNQGIRPAINTAKVYILSGKITDEEFSKIKDYCINPVDSREASLEKPETLKMETEIPTTVEVLDGFIDLDEKGLRTFVSEKGLAMTLGDLQHVQKYFKDTEKRNPTITEIKVLDTYWSDHCRHTTFMTEIENVKIEDGKFNDIVKEAYQMYLNSRDNVYVNRHKDICLMDIATVAVKELKKNGKLNDLDESEEINACSINVDVEVDGKMEKYLVMFKNETHNHPTEIEPFGGAATCLGGAIRDPLSGRSYVYQAMRVTGSADPRTTLEDTLPGKLMQRKITTEAAHGYSSYGNQIGLTTGQVAEVYDENFVAKRMEIGAVIAAAPKENVVRERPEAGDVIVLLGGKTGRDGCGGATGSSKEHSEESILTCSAEVQKGDAPNERKIQRFFRNKEVAQMIKRCNDFGAGGVCVAIGEIADSLDINLDLVPKKYDGLDGTELAISESQERMAVAIKKENKDKFIQLAVEENLEATHVATVTDTGYLRMFWNGKAIVDINREFLDTNGVKQTTDVHVTKVDEENTFFSSNEIVKDVKCSSMKDKFTKVLSDLNVCSQKGLVEMFDNTIGGNTVLMPFGGKYQATPTQGMVAKIPVLGGETNTSTIMTYGYNPKVGKWSPFHGALYAVVESVCKLVAIGGNYSTTRLTFQEYFEKLGNNPEKWGKPFSALLGAFYAQSKFEIPAIGGKDSMSGTFKDIEVPPTLVSFAVDTVDAKKVVSPEFKKADSKVVMLCVNKAENDVVDFEELKRNLDKVRELIHGNKVLSTYALGFAGVGEAISKMAFGNKIGFKFSEEAEKAFTDDKLFEASYGNIVLELANDDLSMLEGYNYVVLGSTVKEASIFIKGEELALDELYKAHCSTLEPIFPTKTEEVKSKIETISYISQGEAKKSSLSIATPRVFIPAFPGTNCEYDSARAFERAGANASIRVFKNLTYKDIEDSIDTIVNEIKSSQIIMLPGGFSAGDEPDGSGKFIATVFRNPRVQEAINEFLTQKDGLMLGICNGFQVLIKLGLVPYGEIRVPSESAPTLTYNNIGRHQAKIARTRISSNKSPWLAQTNVGDIHNIAISHGEGKFVASEDVMRELIANGQVATQYVDFNNEATYDIEFNPNGSFYAVEGITSADGRVFGKMGHSERIGEEVYKNIIGEKEQKIFESGVKYFR*

>CD630_02330 Clostridioides_difficile_630_NC_009089 flagellar assembly factor FliW

MMKITLKKGILGFENLKEYELLDIENEDILKEFNSTEEDCIGFIVVSPFEIIKEYEIVLNQETIEKLEVKSPNDIMLLNIITVGQTLEESTVNMKAPIVINVRNNCGMQIILQDEEYSIWHPLLRGDGGC*

>CD630_02730 Clostridioides_difficile_630_NC_009089 heat shock protein 90

MEFEKGSISIHTENIFPIIKKWLYSDKDIFIRELISNGCDAVSKHKRLVSLGEISENKSSDYKITVSVNKGEGTLKFIDNGIGMTEEEIKKYINQVAFSGAEDFFNKYKDKMEESNDIIGHFGLGFYSAFMVSKKVQIDTLSYTEGATPVRWISEGGTEYEISESDARNDRGTTITLFIDDDSKEFLDEFTVRGIINKYCSFLPVEIYLEDVERLEREAKEAEEKAKKQKEDGKEEIVDAKVIEPLNDTNPLWLKSPKDCTDEEYKEFYRKVFNVFDEPLFWIHLNVDYPFNLKGILYFPKLKNEFELTEGKVKLYNNQVFVADNIKEVIPEFLLLLKGVIDCPDLPLNVSRSFLQNDRDVSKISKHIIKKVADKLKSLCKNEREEYNKFWDDIQIFIKYGCLKDESFYEKVKECILFKTIDDEYITLQDYLEKCKDKHENKVFYVSDKEQQSQYIKLFKEYDLSAVVLNSSIDTHFISFMEYKENGVKFNRIDADLSDVLKDKNENKDSEENKEEIAKIEGLFKEAVGERVKNYSVEGLKNEDTPAMVLVSEQSIRMAEMQSRFAGMDLGMNFEEEKTLVINENSPIIKKLVSLKDDEEKKDKITLICNQIADLALLSNKELKPDELDSFVQRSNKLMSLLIEL*

>CD630_02780 Clostridioides_difficile_630_NC_009089 HxlR family transcriptional regulator

MSINTEKNITKNVEELTCPIRYALDIVGGKWKLPIICMLAVENPIRYSSIKRKLDGITNTMLAQSLKDLESTGIVHRKQYNEIPPKVEYTLTSKGKSIVPILQQFANWGATNMQEKNTCGLSCKECRKIK*

>CD630_02880 Clostridioides_difficile_630_NC_009089 PTS system mannose/fructose/sorbose transporter subunit IIC

MESVLMLAIVTGLWYWFAAGLAGYTLFSTLKSPLFIGFSLGLLWGDVTTGMIVGASIEMVYLGMVAAGGNIPSDKCLAALIAIPVALQTGVNAEVAVSIAVPLGVIGVLVNNLRRTGNAVLVHKADKYAEEGNTKGIWRCATLYSLIFGFVLRFPIVFVCNFFGADLVQSLLDVIPQWLMNGLTVMGGILPALGFATTIFTIGKNKFLPMFIIGFFMVQYFEISITAAAIFGVCIALLITFMKEDKRVGEV*

>CD630_02910 Clostridioides_difficile_630_NC_009089 peptidase

MDISRSVDNSLNRTIEFLKELIKIDSQQGEPISQCPFGIGPKKSLDKTLDYCASLGFSVKNIDNYIGYAEIGEGEELIGIPMHLDIVPPGEGWSVDPFSGAVIDNIIYGRGVIDNKGAVSMLIHVLKNIEDMYPTINKRIRLIFGTNEETGMKCIKYYLDKGEEIPSMGFTPDAMYPVVNGEKGRVHIRIEKEIKIDKSKPYIIVSGGTKENVVPSHCTAKIINGIISELTTKGVAVHASNPEKGENAISKMVIKIVEDNMDFQHREDIELVSKYLCSDYYGDALGINQYDEVFKNTTLNLGILKVNEEKIVCELDIRYGKNIVLNNIIDRFKKVFCNGWKIEVIAHKDLHYVDESNLVLKKLLEAYEEVTDENGYTIAMGGGTYASWFKDMVAFGPKFLAYKTGGHGVDERVPINHIRKNMEIYTLALIKLLEL*

>CDIF1296T_00517 Clostridioides_difficile_ATCC_9689__DSM_1296_strain_DSM1296_CP011968 ABC transporter ATP-binding protein

MMNIVSINGLSKGFGNRKIIDNLNFTVPEGSVFGFVGKNGAGKTTTMKMVLGLLKPDSGTIDVCGEKVTYGKTSSNRHVGYLPDVPEFYNYMRPLEYLSLCGEITGLSKKEIQIRSEELLSLVGLRNEKRRIGGFSRGMKQRLGIAQALLSRPKLLICDEPTSALDPVGRKEILDIMLKIKDSTTVIFSTHILSDVERICDHVAILNKGSIALSGTLSEIKSMHGKDRLLLEFASNDEIQKFKSSDGIKSLLKDSEETNMEIVLHGKDIKAIQKTVISTLAEMNLCPVKMELIELSLENLFLEVVK*

>CD630_02950 Clostridioides_difficile_630_NC_009089 iron-sulfur-binding protein

MEKSKVYFCDLYSNSQNKNVPNNVRRLFDEAGFKDLIEKNDQVAIKLHFGEKGNTTYMSPVAVRQVVDKVKDCEGKPFLTDTNTLYTGSRTNSVDHLTTAIENGFAYAVVNAPVIIADGLYSRNYENVKIDKKHFESVKIGGEIYNSSAMIVMSHFKGHEAAGFGGALKNLAMGCASAAGKQMQHSDVTPVVKEKKCVGCGKCVNSCPTKAISIVDKKAVIDSDVCYGCGECPTVCPTRAVTIQWESDSDVFVEKMAEYAYGAVSNKKDKVGYITFVMNVTPLCDCVPWSGRPIAHDIGILASTDPVAIEQACYDLICKEMGHDVFKHEHPHVNGTRIIDYACEMGMGSKEYELIKL*

>CD630_03010 Clostridioides_difficile_630_NC_009089 ribose ABC transporter ATP-binding protein

MSNIILKLSNIAKEFPGVRALDNVNFELFHGEVHALLGENGAGKSTMIKILTGAHSKTSGKFIFEGKEIEHISPDISKKIGINAIYQELTVFDELTVAQNIFMGKEINGKVLTNDKKMNEEAKKIFDNMGIDINPNSLVKELSIAQKQMVEIARVLSSETKVLIMDEPTSSISKKETEILFRLINDLKESGVSIIYISHRMEELFEICDRITIMRDGKTISTLNTKDVSSEEELVNLMIDRKLDQFFPKRKVEIKEEIMRVENLTKNNVFNDISFNIRKGEILGIGGLVGSKRSEIVEAILGLRTYDSGKIYLNNEEVKFKTPSDAIENGLGLITEDRKGTGLFLQMSVKENTTMAGLKKISKFKSIIDRKKEKEILEKYIEALKIKTPHMNQVIQSLSGGNQQKAIIARWLLLQPDILIMDEPTRGIDVNAKAEIYNLMGDLVESGVSIIMISSEIPELISMSDRIMVMREGHISGFLEGEEMVENNVLKLAFGGKINEFNN*

>CD630_03030 Clostridioides_difficile_630_NC_009089 acetylornithine deacetylase ArgE

MKKILLDTLNSKKQEYIDYLKELVSIKTEDVGHGILGGLEKEGQEYIEKLANYIGFSVDRQEMSEELIKKAKNIYKEGNLGHNYQDRYNLICKYSDDLPGKTIVFNGHVDTMPPGDISKWKYNPYRATEDNGKLYGLGTADMKSGLIASILAVKLIKDSGLNVPGNVKIMSVVDEEGGGNGTINAVMNGIDGDCCIICEPSEQNLIVAHMGFVFFEVEVKGVSLHCGSKWEGVNAIEKAMLLLQDIKELEHNWLMIYKHPLLPSPTINLGVINGGTAGSTVPDKCVFNLCVHFLPNIMSYEQVVNDVTNVIMTRANGDLWLKDNKPNINIYQSGLGFEMDKDSDFVVNAHKILEETLGKKLEIKGSTAGNDARVMKNLAEIPTLILGPGSIEQCHSIDEYVEIKEYLDSILMYASLILNL*

>CD630_03040 Clostridioides_difficile_630_NC_009089 hypothetical protein

MKKVLIVGESWVKNITHIKGFDTFVTTHYEEAVKWLKEAIESGGYETVHMPAHVAADSFPYKLEELNEYDCIILSDIGSNTFLLSNSTFIDCNSNPDRLELIKEYVNNGGALIMVGGYMSFTGIDAKARFGETAIKDVLPITMIDKDDRVEKPAGIIPEVIDSEHPVLKGIPTEWPKFLGYNKTVARDNCPVLATIGGDPFVAVGEFGKGKSAIFSSDCAPHWGPIEFTDWKYYNKLWVNMLDWLTC*

>CD630_03070 Clostridioides_difficile_630_NC_009089 chaperone protein

MYSNTYNNENFMRARAFIENSEFKKAYDFLKTLTDKCAEWYYLTGFSAMNIGYYEEGEDFLKRAKFMEPENSEYSDALRSYTQYRNDYSNRADNYNRRRRNDLDGCCCCCCDDCCCCLGDDCCENCAKLWCLDSCCECFGGDLITCC*

>CDIF1296T_00538 Clostridioides_difficile_ATCC_9689__DSM_1296_strain_DSM1296_CP011968 ArsR family transcriptional regulator

VHSNYIKLIKIIVANLEMIAYNEHMNSYSYIKIIGGLKMREEINDCNCNIVHEEIVTEAKSTMPDEEMLYDLAELFKVFGDTTRVKILYALFANEMCVCDIASLLNMTHSAISHQLRVLKQARLVKFRREGKTVYYSLDDSHISQIFDCGLNHIRETYK*

>CD630_03130 Clostridioides_difficile_630_NC_009089 K/Mg/Cd/Cu/Zn/Na/Ca/Na/H-transporting P-type ATPase

MEANNSIKKEFILGGLNCAHCAEEINNKVSKLQEVKSSNLNFINKKLTVNIKESFNEDTTIEKIIDIIDSTEPGLDIQISSKENAASKTSIKKELILGGLNCAHCAEEINNKVSKLKEVESSNLNFVNKKLTVNISNNFEEDDVINKIKEIINSTEPGLDIQVGSTDKVKGRTTEKSGAVNDTNKKELIPLIIGALVYIFGIYQTATGYESQFSNIVFIVAYVIVGGDVLLRAIRNISKGRVFDENFLMALATVGALAIGELSEAVGVMLFYKVGEYLQGVAVGKSRKSITSLMQIRPDYANLKVNSEVKVVSPEEVNVGDIIVVKPGEKVPLDGVVVDGVSMLDTSALTGESVLREVEKGDEILSGVINKNALLSIEVTKSFGESTVSKILDLVENSSIKKSKTENFISKFSRYYTPIVVIAALLIAFVPPLVISGEVFSDWLYRGLIFLVVSCPCALVLSIPLSFFSGIGFASKNGILIKGSNYLEALRSVDTVVFDKTGTLTKGVFNVTKLNPEGISDEELLEYAAIAEVNSNHPIAKSILSYYNKKIDLDTIDSYEEIAAYGIRVKHNGNFILAGNEKLMKKENISYSSAKEVGTVVYIAVDKVYRGYIVISDEVKEDSKNAIRSLKEIGVKEVVMLTGDNEKVAKNIAQELELDTVYSNLLPNEKVDRLEDLYEGRTEKEKIAFVGDGINDAPVLARADVGIAMGGLGSDAAIEAADVVLMTDEPSKISKAIEIANKTNKIVWQNIIFALGVKIIVMILGAGGVATMWEAIFADVGVALIAVVNAMRAMR*

>CDIF1296T_00550 Clostridioides_difficile_ATCC_9689__DSM_1296_strain_DSM1296_CP011968 norq protein

MDIIDNLRVQGVDEKLIEDVLYFRNYYGLEKDLEYRVTKSKTYFYGKDILSMCIAAILEEENILLSGPKATGKNLLADNLGEIFNRPQWNTSFHINTDSSTLIGTDTFIDNEVKLRRGSVYECAINGGFGVFDEINMAKNDAIVVVHSALDYRRIIDVPGYERVNLHPATRFIGTMNYEYAGTKELNEALVSRFMVIDIPPIEEDKLMMILKNEFSDADEEKLIHFAGIFLDLQLKSQNGEISSKAIDLRGLMASLKTIRRGLKPTLAINMGLTGKTFDVYEKEMVGDVIKTRIPNKWESIDVFPISHI*

>CD630_03270 Clostridioides_difficile_630_NC_009089 cobalt ABC transporter ATP-binding protein CbiO

MMFKINNLTYQYEKNTNALLNINMDFSKGNVIGIIGSNGSGKSTLFMNLMGILKPTSGEILFKEEKLKYDKRSLYNLRKNVGIVFQDPEKQIFYSKVYDDIAFAMRNIGMDEKTIKERINKALVAVNGIDFIDRPVHFLSYGQKKRVAIASVIAMENEIVLLDEPTAGLDPVSTRSIVDIIKGLNKNNIKIVISSHDMNLMYEICDYIYVLDKGILIDEGKAENVFINENNIIQAGLESPWLVKVHRNMNLPLFKKEEDLYKYWKERELNTNK*

>CD630_03300 Clostridioides_difficile_630_NC_009089 hypothetical protein

MKLGLCLEGGGAKGAYQAGVVKALYDGGINKFYSISGTSIGAINGYYLYTGNVDNLEKMWTNIKDIQNGNVKIVNNTVDNSPAIDNLRELDDSNIEEMNFYVNYVEVDNKVVSEKIVDVSKMPRNEAIISISYSGLLPSNPNATLGFKEQFVKDVQEGIYDGFKLDGGLIRSALIEPLIGDNVDKIILISTKYNYELPEDIKKVYDEDKIIVVRPNTQFAPKDTLNFDDEFCKTIYQEGYEIGKNILDRL*

>CD630_03310 Clostridioides_difficile_630_NC_009089 peptidyl-prolyl cis-trans isomerase B

MENKNPIVTIEMENGKEIKIELYPNIAPNTVKNFVSLVNEGYYNGIIFHRVIPGFMIQGGCPNGTGMGGPGHSIKGEFSGNGFTNNLKHERGVISMARTMAPNSAGSQFFIMHKNSPHLDGQYAGFGRVIEGMDTVDEIASVRTDSADKPQTPQVMKSVTVETFGIDYSDVEKN*

>CD630_03340 Clostridioides_difficile_630_NC_009089 bifunctional acetaldehyde-CoA/alcohol dehydrogenase

MEKKEKVVEKSNVEVCSPEIVNSVETLRMRLEEIRLAQKEFATFTQEQVDKIFLAASTAANQQRIPLAKMAVEETGMGIVEDKVIKNHFASEYIYNAYKDTKTCGVIEKDEAFGFTRIAEPVGVLSAVIPTTNPTSTAIFKSLIALKTRNGIIFSPHPRAKNCTIEAARVVHDAAVKAGAPKGLIGWVDVPSIELTNVVMAEADLILATGGPGMVKSAYSSGKPAVGVGPGNVPAIIDESADIKMAVSSILVSKSFDNGMICASEQAVIVPEKIYEEVKKEFKYRGAHFLNKEETEKVGKVVIIDGSLNARIVGQPAHVIAKMADVEVPKTARIIIGEVESVELNEPFAHEKLSPVLAMYKSKSFEDAVAKAEKLVADGGYGHTSSLYADSINHPERVEKFVNAMKTCRVLVNTPSSQGGIGDLYNFKLAPSLTLGCGSWGGNSVSENVGVKHLLNIKTVAERRENMLWFRAPEKVYFKKGCLGVAAREFKDVMDKKKAFIVTDSFLYNNGYTKKLTDLLDEMGIKHTTFFDVAPDPTLACAREGAKAMADFQPDLIIAVGGGSAMDAGKIMWVMYEHPEVDFQDLAMRFMDIRKRVYVFPKMGEKAYFAAIPTSAGTGSEVTPFAVITDQDSGVKYPLADYELMPNMAIIDADMMMEMPPRLTAASGVDALTHALEAYVSMLRTEPADGLALQAGKIIFEYLPRAYKNGKNDKEAREKMAMASTMAGMSFANAFLGICHSLAHKLGAFHHVQHGVANALLINEVIKFNCAEAPNKMGAFSQYRYPDCIQRYAEFASFAGIKGSTDQEKVDNLIKAIDELKAKVGLPKTIKEAGVEESKFLERLDAMVEQAFDDQCTGANPRYPLMSELKEIYLKVYYGK*

>CDIF1296T_00565 Clostridioides_difficile_ATCC_9689__DSM_1296_strain_DSM1296_CP011968 ABC transporter ATP-binding protein

MPILETINLGKIYGKKETSVHALKNANLKINKGEFVAIIGPSGSGKSTFLHLVGGLERPSNGTIKVAGKDICCLSDKELARYRRQKVGFVFQQYNLIPVLNVKENIELPLKLDNKKIDKEYIEDLINLLGLKERKNHLPNQLSGGQQQRVAIARALSAKPSIILADEPTGNLDSKTTEEVMDLLKSSIKKYNQTLIIITHNENIARKADRIISIIDGELKLTL*

>CD630_03890 Clostridioides_difficile_630_NC_009089 6-phospho-beta-glucosidase

MRKFPEGFLWGGATAANQFEGGWNLGGKGWSVSDVAKAHFDADVKDYKSNNEITTKDIEEGLAHPEDEVNYPKRHGSDFYHHYKEDIALMAEMGFKTYRMSIAWSRIFPNGDDKEPNEEGLQFYDDVFDELISYGIEPLVTMSHYEPPLNIVLNYDGWYSRQVINMFVRYVETICERYKNKVKYWLTFNEVDSMIRHPYTTGGLVRDRFKDKNFEEVIFQAMHHQFVASALATKICHEIIPNSKVGCMLTKLTYYPYTCRPEDVLATQQKMRSIYAYSDTQVFGEYPVYLLSYFKNNNIQIVKEEHDDEIMKKYPVDFISFSYYMSSCEAADTTGLDITPGNTLLAVKNPYLEMSEWGWQIDSIGLRISLIELYDRYRKPLFIVENGLGAKDILTEDKKVHDQYRINYLKEHFKCMLDAIIEDGVELWGYTSWGCIDLVSESTKQMSKRYGYIYVDADDYGKGTYNRYKKDSFYWYKKVIENNSIDFK*

>CD630_03930 Clostridioides_difficile_630_NC_009089 membrane protein

LEKMNKVTSKKTTYIVTSALFASIICLTIAYILHIPVGGNNGYVHIGDAFIYLAATILPTNYAIAASAIGAGLADLSTGAAIWVIPTIIIKPILVLFFTSKSDKIINKRNIVASVVAGIVGLVLYMFAEGIIIGSFTSAFVMSLLGLLQPIGSFIVFIILGMALDKLDFKKRYFN*

>CD630_03980 Clostridioides_difficile_630_NC_009089 oxygen-sensitive 2-hydroxyisocaproyl-CoA dehydratase subunit C

MEAILSKMKEVVENPNAAVKKYKSETGKKAIGCFPVYCPEEIIHAAGMLPVGIWGGQTELDLAKQYFPAFACSIMQSCLEYGLKGAYDELSGVIIPGMCDTLICLGQNWKSAVPHIKYISLVHPQNRKLEAGVKYLISEYKGVKRELEEICGYEIEEAKIHESIEVYNEHRKTMRDFVEVAYKHSNTIKPSIRSLVIKSGFFMRKEEHTELVKDLIAKLNAMPEEVCSGKKVLLTGILADSKDILDILEDNNISVVADDLAQETRQFRTDVPAGDDALERLARQWSNIEGCSLAYDPKKKRGSLIVDEVKKKDIDGVIFCMMKFCDPEEYDYPLVRKDIEDSGIPTLYVEIDQQTQNNEQARTRIQTFAEMMSLA*

>CD630_03990 Clostridioides_difficile_630_NC_009089 acyl-CoA dehydrogenase

MLYNKEQELLRKAVRDFVSKELDTLPAEMDKTGVMPKELIKKLADAKFISSNIPEEYGGGGAGYVSYAIVMEEIARRCASTATFVTAGSSLASLPILYNGTEEQKQKYLKGIATGELIGAFGLTEPGAGSDAGGQQTTAELVGDHYILNGRKTFITNGPFCDVAIVIAVTDRSKGLRGTSAFIVESKWDGFSTGAHEDKMGIRGTETSDLIFENVKVPKENLLGKEGQGFKIAMGTLEVGRIGVAALALGIAQGALDEAVKYTKQRVQFGKPIAKFQNTQFTIADMETKVCAARGLVYDAAQKRDAGMRVAQESAMAKYYASEIANEVAYKALQLHGGYGFIKDYEIERMYRDARIVSIYEGTSEVQKMVISSNVLK*

>CD630_04010 Clostridioides_difficile_630_NC_009089 electron transfer flavoprotein subunit alpha

MNDIKDLSSYKNVWIFAEQREGKIAPVVIELLGEGRKLAKEVDAELCAILLGKDVDGLAKELITFGADKVYVADDALLEKYTTDAYTKVIKDAIDEIKPEIMLFGATHIGRDLAPRIASRVGTGLTADCTKLEIDPEDKKIKQTRPAFGGNIMATIICPNHRPQMSTVRPGVMDKAEKDETRTGEVIALDYKITQDDIRTTVLETVKTKKDLVSLTDANVIVSGGLGLGGPEGFEMLKKLADKLGGVVGSSRAAVDAGWIDHSHQVGQTGTTVKPNLYIACGISGAIQHLAGMQSSDFIIAINKNPAAPILEIADYGVVGDLHEIVPMLIEKLDSVDDLLEAIKA*

>CD630_04040 Clostridioides_difficile_630_NC_009089 hypothetical protein

MPQIKIRGINENDICKISEKMINDLVEAVKCPRDYFEIECIKSVAIRDGKIADVYPFVEVAWFDRGQEVQDIVARIITDSIRNNLDVESMDLAFTVFEKEKYYENGEHF*

>CD630_04420 Clostridioides_difficile_630_NC_009089 2,4-diaminopentanoate dehydrogenase

MRKVRVGIWGFGAMGIGMANMILKKEGIEIVSVCSRSTSGKSMYDVLGIERGERPEVIINKNYEEVFREKSVDVVLLATDSFTKKAFDKIIFLLNRKINVISTAEQMAYPQADDADLAKKMDEVAKENGVSILGTGINPGFVLDLLVLALSGTCEEVTSIKAKRVNDLSPFGKSVMVEQGVGVTREEFIKGVEDKTIAGHVGFVESINMIADGLGWKLDKIEQTKEPIMTTVDRKSKYGEALAGNVAGCRQCGYGYVNGEVLIEMEHPQQIIPEAEGIKTGDYVSIKGIPNIDLQINPEIPGGVGTYAMIVNSIPLIINARPGLKTMLDIPVPRAIMGDIRNQIEVELEEESKAN*

>CD630_04470 Clostridioides_difficile_630_NC_009089 reactivating factor for adenosylcobalamine-dependent D-ornithine aminomutase

MENSKVSLKIDVLVAEIGSTTTVVNAFHDINTNNPIFLGQGQAPTTVFEGGDVRNGLSGAIKDLANKLSVDDIEYNDMFATSSAAGGLKMTVHGLVYDMTVKAAKEAALGAGAVIRQITSGRIKRTDLNKIKEINPNIILIAGGVDYGERDTAIYNAEMIASMNLGIPVIYAGNVENQEEIRLIFEDTNYKLYITENVYPKIDLLNIEPTRRIIQSVFEEHITTAPGMKYIKEMVNENITPTPGAVMEASKLLYKNIGDLLTLDVGGATTDVHSVTDGSDYINKILVNPEPTAKRSVEGDLGVYVNMKNIVEVIGKENLQSELSIDIDAVIENYPPIPKSKEEILFVERLTKEAVVKAMLRHSGKIRNIYGTTGKVKIAEGKDLTEVRYIVGTGGALTRLPSRIEILDNMLKYNKNNELLFPKEKTKILIDNDYIMASMGVLSKKYEEASLKLLLKSLNFEEERLCILG*

>CD630_04510 Clostridioides_difficile_630_NC_009089 dioxygenase

MKIYDILTRFYIQDIEKAIPFYENLLKEKCSLRFSYKEVGLELAQIGNVLLLSGSDDALKPFIETKSTFMVDSVDEWRSYLLDNGAVVVRDKKKVPTGYNMTLRHPDGTIIEYVQHTKQD*

>CD630_04590 Clostridioides_difficile_630_NC_009089 ABC transporter ATP-binding protein

MEILKCENLTKIYGSNQTRVTALNNVNLSVQKGDFVSIVGASGSGKSTLLHLLGGVDRPTSGKIYVEDTEISSLKEEALAVFRRRKVGLIYQFYNLIPTLDVRKNILLPMLLDKRKVDEDRFSEIVSILGLSDRLNHLPSQLSGGQQQRVSIARSLIYRPAILLADEPTGNLDRKNSEEIVDLLNLSNKRFNQTILLITHDEKIALEANRIVTMEDGVIVSEKVVKK*

>CD630_04840 Clostridioides_difficile_630_NC_009089 ABC transporter ATP-binding protein

MIIKAKQLSKIYGSNNNKVIALNNVNLEINSGEFVSVIGPSGSGKSTLLHILSGLDNPTSGQVLLDDKDIYKHTEKELSALRRKSFGFVFQQFNLLPVLTASENISMPVLLDKKQPDKGYLNEISSLLGIADRLNHLPHELSGGQQQRVAIARALIAKPDIIFADEPTGNLDSKSGSEVMNLLIKTSKQFGKTLVVITHDDRIAKLADRKISIIDGVLMEVK*

>CD630_04870 Clostridioides_difficile_630_NC_009089 carbon-nitrogen hydrolase

MNFYKIAVCQMITTENKIENINHAVDMVTEAAINGAKIVVLPEMFNCPYENKYFPKFAEEYPGETTTILSKLAEKHGIYLVSGSIPELEDGKIYNTCYVFDKNGTLIGKHRKMHLFDIEVTGKVSFKESDTLTAGNDVTVIDTEYGKVGIAICYDIRFPELSRLMALKGAEIVILPAAFNMTTGPAHWELSIRMRALDNQIFYVGAAPARNMNASYIAFGNSRISDPWGRIIAQADEKECIIYADIDRDLIPDIRQQLPLLKHRRTDLYELNTLK*

>CD630_05190 Clostridioides_difficile_630_NC_009089 hypothetical protein

MEKLSDKRRVKAVSMETFVFIVLLAVGFGYVGSIMGAGMMFKVIMSTAHALLLDTVFLIMAMAVLAGALSALLSEFGVISLVNKIFKGLMRPIWGLPGASIAGVVATYLSDNPAIIPFAKDKTFTQYFKKYQVPALCNLGTAFGMGLIVTTFMIAQGKEYVLPAIIGNVAAIIGSIISVRIMLTFTKKYYNYDPKNDTEKQINDKGAKLEEFREIRDGNVFQRTLDAILEGGKLGVEMGMAIIPGVLVVCTLVMLLTFGPSTDPATGQAVYTGAAYEGIKLLPAIGDKISFIIEPLFGFTSPEAIAFPVTALGAVGAAISLVPEFIKSGAITPNDIAVFTAMGMCWSGYLSTHIGMMDALDARPLAGKAILSHTIGGLCAGICAHFIFMLVG*

>CD630_05560 Clostridioides_difficile_630_NC_009089 sugar isomerase / endonuclease

MAVGIASLLFNIEEALNICESIKQITHLEIGIDNISECSELCKYKERISKLGLSIGIHLPMELNTCENIEYIRNSWINFIEKIEFELKGFDLRYFNLHLGYVMTNRVRKNRDKYLGNSVDFLDKLNTNSYVCIENTYSKGGDFSNIGNISYDFEYIFKRIKNSKICFCYDTGHCLIDEDAYVKNLKDKIRLIHLSDNDGINDTHVGIGRGILSEEGIKEVLTLDAEYLVLEINYEDIEDTISKLNNIVGEG*

>CD630_05590 Clostridioides_difficile_630_NC_009089 ArsR family transcriptional regulator

MKEVLVLRDLECIKAIAHPRRIDILKAFDKSPLSAKQLSQLLEEPHAKINYHIKMLYKVGILELVEEKIKSGIVEKYYYPSAKNVVIGNRILNFSLDNGEEKEELYISKFENMSEVFYKAAEEDVLENENIVDYHDISLTHDELVELSDTMKSKIDEILNKRQHNVEGSKYDIAMVIVPTLEEECPS*

>CD630_05800 Clostridioides_difficile_630_NC_009089 glyceraldehyde-3-phosphate dehydrogenase

MFNELKTKENVYKNLINGKWVESNSRKPIEIYSPIDNSLVGKVQSMTKHEVDEVIKNTKESIKVWAEMPVYKRANIFHKAADLLLENIDEIANILVLEIAKDIKSARAEVERTADFLRYTADVGKNMEGEAISGDNFPGGTRNKMSYVSRVPLGTVLAISPFNYPVNLSMSKIAPALIGGNAVVLKPATQGAISALHVVEIMRKAGIPDGVLNTITGRGSEIGDYVVTHKGINFINFTGSTEVGQHISKISGMVPLLLELGGKDAAIVLEDADLDFAAKNIVSGAYSYSGQRCTAVKRILVQESVADKLVGKIKPLVEKLTIGNPMDEVVITPLIDNKATDFVQGLVDDALHKGAKLITGNVRKNNLFYPTLLDNVNVDMKIAWEEPFGPVLPIIRVKDINQAIEIANQSEYGLQSSVFTSDIDKAFYIADKLEVGTVQINNKTERGPDHFPFLGVKASGMGTQGVKYSIEAMTRPKAVVVNVREL*

>CDM120_RS03695 Clostridioides_difficile_M120_NC_017174 hypothetical protein

MPEDNKTFLYELKELLSNGEKQIAKAIGQDLILILGGTGCGKSTAINYLAGCEMHKVEDEDTGVSYITCEDPVADIGSGAVSKTLYPEVIDMKKGFEGMTAKFCDTAGFGDNRGAIHDICAAVSLGEVFKDSASICAVIVLIPEADILDSRLTKVLDLFQQLNMCLHKENFKNSITFFISKSFLGRTEKQIYNVIKRKYEQLCEASDSNMSDSNSNIKEKAWLFEEMLKDDGKNIHICNPLASDDREGLLRQVLELEEVKDKISAFQYPISADALLVLDTLIKEIQDNIVSSLEDYVKAYTEDFISAVGSITMIEEIAKYDDVVEVFKNWLESTTSPTIGRFINELARQSSLFLQDSCMKLQKNIKENLDIIDALSEYTEHGTSEILLSSVHMEFIIEKTSKKIMEQNFIIIRKNLEKVLVSYEVQKQVFTLPNRENLMEVANKKDYKYSVNDFNSILTNVTVTQKEELEDSITRLNAYGEEGNEIIREFISKYILNPFIVKTININDKQGIEIRALIPNLVISSVLEYFKNRKVDDNVSTFMFFAKNTIYMDDNLGMDFASEKNIIIACNNLDVGSNVKINVSGKSGELVDNSMYGKNGEDGRDSGNIYLYIKEEIRNYRLTLIANGGNGSKGIDGKNGETGASGSNGADAGYTSSFHGKMFKGYFAQGTSGGKGEKGGAGGNAGRGGNAGKKGNIYFISDSEEQKNRIINRCDITVRDGEVGPNGRPGSGGYGGNGGYHGYNTLSFSPSGIDKTVYYTGYFTSWDYKLDSIIKYHIEFSGGSWNEGKTGPGREYAEYGDRGADGGFNAPNTKISTAVSKIEDNFIKEKYQCYCSEI*

>CD630_05900 Clostridioides_difficile_630_NC_009089 hypothetical protein

MANKLYSEIVNLLEEGRDELRKYDLKEKSILLFLGASGVGKSTCINYLKGCVMEEKTDEETGQIYITAKDSAVEIGNGVYSKTLCPEVVDIANRDFSLCDCPGFFDNRGAEYMIAGAMLVRETISTSSKVKGMVVILDYQSLLDSRHTLLIETGKNLKDMLGDYAKYKENFFFLINKIPKTLIGNVTEEKIRNILEKAKEDLDSQEKDYDGCKELVYLLNVALSDGVSIKLCNPLDKQPEKLLNEMKNLPETFEKTDLGLMLTASCKIEIQKAVDVTYKDLEKLLVPYIESVKKGMEDSISILSSINSLKDKKIQLDKMYTDLCSRTNNMKELMEFVQELSYGDGVQKVNILEETRLLLELNSYCSETHIVGLSELLKSLLKNTLEDTLKDIKYWIAEEEYREEEAKRLEDAEYRIEKIQSCTYIKSYEKENVLSIYIPIPKVNLSDLTKIISGYQLNILSSIYMYGDCELKIDCNMEKEVFSGKNIVLLTEKLHIPEGTYSINVSGKDAEKLVGESSIYDGMNGRNGNNAGTSGDIILSFKEIEKRGELKLYVDGGRGGNGQDGKKGDTGITGEAGANAKIGSKKESKEFANVKGTKYYVVKAEKGKQGGTGGTGGNGGNAGLGSKSGQVYLLGKAPLLKSAISKKDGNHGLAGKGGSGGAGGAGGLGGIRGIYEETIRYIGTSPPMDNSDRNYYYFDRYVFNHALNQKQAADDGEKLNALYEEHKDWTISKREDSGSAGAEGKAGHSGDALKEKELLKQEYSLSIDTFFYEFFERFGFSGIKGYRNLRLLCAVGILEADILELSHIYEIFHAVEKIEMQLNREILQKYYSFEKKNKAVLSLDDMNTKKEFYKEAKEIYEYLINKLMQSDRIESFVGKNQNFSEWSMILFALEQKTAFYVQMIEESEIGGNRVTDIRSLTDYLDKTLRELEQGKKDELKEGYFNTYKESILTQIESGNQQIEVLKNELENKMLSVNAKMNLLIQEIIKMKEDEQEDIEELKKKQAKLEEQMKTQLILSGIQSALSLLTAFSGVGQACLGAFDSAKSLKENLSAKEEEYQWTNRMVDFSDFKLDSDGMRFVLGEQLSEDRKNALDKMTYNLAGVDEERRTKFDQIKEIIVKSIDDDNYADKADDIQRAKEGLSESEYKEIYTKIQKKEIGMLEANYNNKQQKDKLAALEKSKQAIERQQKTSKIKNALSIGSSACSLINTAISGVNNCLNVKSSFESKIEVVKEAIKDKENNIKKLDELNTQMVEFKDKTLKESVIPFVEKFDSTVKNQDIFENEISKLKMKEVLTELSTKFGSLKMENSEEISIIFKKVQDMMETQIIITDKIDKKKDNLAMGELIYQMSDSGNQMNEYQKKLIHQIELNQVRYLCQVEAAAFQLWTFPFGSNIVNILNRCHVGITEDENEVINQAKEQNRQIREFLDQDAYQWQKTDNHILKVALTGSLDGKSRVGFENTYSLYDCNEEEVKKLLTGDEISFEVPLNKNFDAIKYVTMYANIPNLRRNILEDSLKVKVHIHLYDSGYFSLYDDKKDVYYQYVFPQNYINITHGLDIKYNDSDSINSIWSSVEDKGIEKFKNAGLNSGRSPYSMAKISLKAHFNRKLSYDSLTNEQKKKLESYFDKPSKYAWSDSIDILRSLGYIDLKGILSKSWYEIKKDIDSDGEIMKHFSILQVDLLERIIYEQTSILKENIPNLTIELVGCGIYVESEFNYLKQNDLKCYEERLVENTIIG*

>CD630_05940 Clostridioides_difficile_630_NC_009089 hypothetical protein

MDKRYLESDVFEAFQERLSYLFNEFDYIYVSFSGGKDSGLLLNLVLDYKRKNNISSKIGVFHQDFEAQYSLTTEYVEKVFEDNIEDIEPYWVCLPMATRTAVGNYEMYWYPWDDEKEELWVREMPVKEYIINQKNNPFTYYKYKMRQESLAKQFGRWIRDIHEGAKVVCLLGTRAAESLQRYCSIVNKQHGYKGKCWITKEFKNVWTASPIYDWEIEDIWLAHYKFDYDYNKIYDLFYKAGVAPRKMRVASPFNDSAKESLNLYRILDPDVWVKLVARVQGVNFTSIYGKTKAMGYRSVKLPYGHTWKSFTYFLLDTLPENIRNSYIKKFSTSIEFWQNTGGGLSEIVIRELLRKGYKIELNGVSNHTKDKKSRVIFLDDIPDDTDDIKFTKDIPSWKRMCYCILKNDHMCRFMGFGPTKEQRIRTEYIKKKYEKIH*

>CD630_06160 Clostridioides_difficile_630_NC_009089 MerR family transcriptional regulator

MFKIGEVSKLTQISIRMLRYYDELGILKPAKTDKYTGHRLYSVEQISILQRIVLLRDSKFSVAEIANIVHNWNDEFVIKELNRKKNEIQKEIKQEQQRINKIDKFIEAINCDKDEIHYNVVFKKIPSYKIISLREIIPDYQSEGILWEKLSKFIKEEHIEVSRQSNNNIAFYHDEEVKDNGVDIEVGMVVKKIGKNKSGFIYRETEEIDMMACTMVYGPYENIAGAYESFCYWLDKNSDYQISGINRQIGHKGEHNEINPENYLTEIQIPLIKV*

>CD630_06260 Clostridioides_difficile_630_NC_009089 replicative helicase DnaB-like protein

MGKSKDVEIEKSILGTILLDNKLSYKLDELNENMFMNDICLEIFKIMKELKKENIVIDVATVKSKIDRKSLAIKTSDVTNLITWGQNFGLDGHIKILKENLARRSINQNCQNLLHSLNLGENIDTCIYKFESNIKEILDKDTYENDDVNSIAGKVLDFLENKKDIGFKFGIKLLDTTIGGLFKGELTTIAAKSGVGKTALALQIMLNSFKQGKKTLFISREMTSEQVFMRNICRVTGVSTRNMKSKEIDENDWKLIVNAIGDLSENNLIYINDKIDTISAIRKRIRQVKPDLLIVDYVQLLTSQKSMDKREREVATFSRELKNMTLDFNIPVIQLSQLNDEMKDSRPYGDRPMRDSKAIYHDSNNVVYIHQLKGSDYEEAVRDIGESEEAVRASEYRGIKMVDLIVAKCRDGQTRHKHFCYFGDKLHFQELNY*

>CD630_06280 Clostridioides_difficile_630_NC_009089 membrane protein

MNNKKHWYDYLWIFSSIYLILGFVNIIFAWIGLICFFVPLAISIVKGNKAYCNKYCGRGQLFNLLGNKLKLSRNRDIPKFIRSKWFRYGFLTFFMVMFANMLFSTYLVFEGSRNFKEVVTLLWTIKMPWQWAYHGTLVSPWVVQFAFGFYSVMLTSTVLGLITMILFKPRSWCVYCPMGTMTQMICKAKSKSI*

>CD630_06370 Clostridioides_difficile_630_NC_009089 acetyltransferase

MIEIKTIEINKKSQIDLLILADPCEEMIDKYLNKGTMYALYDNKELTCIAVVNEISKEICELKNIATYEHFQNMGYASKMIYHLLDVYSKKYSSMVVGTSESGVPFYEKFGFVYSHKIKNFFVDNYPEPIFEGELQCVDMLYLSYNF*

>CD630_06390 Clostridioides_difficile_630_NC_009089 hypothetical protein

MKEKLEEKSKIIKTSKEYKRTIIEEKDREWTKKLNDILSRGDFSNEKLDKIKDLIPKEILTSENVGEVVTEDGYAKPEYDEVFKSLFTLNNDYDLLANFINDILKDAPYANRNIKQFTQIKRIIKVETDPTINYIGEKRPRLDILAEDEERNHINIEMQRALEEDYLERAEYYLSRVHGRKLEEGKEYKEIGKTVGIHILNHVKYNHIEDYVNCLRLTMDGHPDIFSSKTALYFIELPKIRKSSCIANRVLIWGKLIDNPSHIDIRILSKTDYVIKRALDRLKELGSNEDYLLNLKRGAYIMNKSRNFKEEIFQEGIEKGIEKGIEKGIAKGKREEKFNIIIKLKNKGYNLSEICDIIDDLNKSEVEKVYNQN*

>CD630_06450 Clostridioides_difficile_630_NC_009089 lantibiotic/multidrug family ABC transporter ATP-binding protein

MNYIIETHQLKKIYKDKAVVNAVNIHVKKGEIYGFVGPNGAGKSTVMKMLLNLVKPNSGEIVMFGKKVAETDFEILKKIGTIIENPYFYENLTAKQNLDLHCEYMGYYNKEHISEVLECVGLSKQSNKKVSKYSLGMKQRLAIARAILTKPELLILDEPINALDPEGIREMRELFRKLNTDYGITIFISSHILSEVEQIADTIGIIQDGKLIKEISMSDIHKYQTDYVEVDVDNVELAGYLLEKEFGIKDFKITSESCIEIYDLRKDVKEISKIFIQNQIGINSIGRKQSSLEDYFFQTTGTGGKETL*

>PCZ31_RS03410 Peptoclostridium_difficile_strain_Z31_NZ_CP013196 prolyl oligopeptidase family protein

MKEVVGSLKKITKERIENTMKKLFTSETFLEYQFISDCQISPDGGYTAFIVKKADIKENGYTSQVYILNNKTGELKQITSINSVGAYAWEDENTILFPALRNEKVKEAVKNGKQCMSYYALSLNGGEAEELFRLPIKGGKLNPIGKGLYAVIDSYDNDRPIVEGLPEEEQQKKINAYNKRHYEHFKEIPYAVNGEGYISRKRKRLYVYDSNINELKAITAPMFNVVGMKISDGKILYIGQEFKDVKGLKNGVYVFDTNTNTNVCILEKDKYIIKGFELYQNQVILNLTDALSYGNGENGDFYTIDIDTKEMKLLSGHQHHCIGNTVTSDVKMGAGQTTKVDGEYIYYTSTVDMDCIIERIHIPTGKQEKVTQTGSVDFIDVKDGNIVCVGCLGNGLPEVYTVENGTLCKKTHLNDHILEEYKISVPEYIESKGSSKWEIQGYVYKPVDYEVGKKYPAVLAIHGGPRLTYGPYFMHEIQVFTSAGYFVFFCNPRGSEGRGNAFADIRKQFGDIDYIDFMEFTDTVLEKYTDIDKTKLAVEGGSYGGFMTNWIIGHTNRFAVACAQRSIANWSGMEGTTDIGYYFCKGQTGASHMENHELQWKQSPLAYADKCVTPTLFLHGEKDYRCYMQEAFQMFSALKIHGCPTKLCLFEGENHELSRSGRPKQKLQRLVEMLDWFSVYIKKDNIGQ*

>CD630_06500 Clostridioides_difficile_630_NC_009089 peptidase

MEKIKIESSDIFEYIFPHDISCSPDGKHIAYIISNINEEKDCYEHDLYVMDIKTEKQIHMTQTKDVTSFSWISNTELLFTSKRNKPKAGTTDFYTISIEGGEAKKAFSIPKACSVPVSLGNKLWLLTTKNPTDSKKSEPDRAVEGVDYWTFTDKPFIRDGENFSQRRRVTLELYQEGENITKAITPKFCEVAGIDVSSDKNRILYTGQIYEDCATPFSGLWEYHIDSGETKELVPQGKYQISLAKYIGKDKVMLQASTLDRSITQNHDIFILDLSTSEINMIASPDGMYATLLDVDAVYGGGRSNKVIGDKFIGARICRTMTEFNEFDTKTGNIRIITKVDAFTSFDIYDNTMYTVMLKDYELAEIYSIDMTTGTMKKMTAFSKPYLDTHKVSLPEKLTFVAKNKEEVDGFVIPPIDAKEGEKYPAVLFIHGGPKWAYGYMFTHLKQCVTSKGMYVIYCNPHGGDGYGEKFLEMVERWGYVDYEHLMEFVDTCIEKYPGIDADRLGVAGGSYGGYMTNWIIGHTDRFKAAVSQRGISNLITASLIIDFGDRIMKQTCGDKTPWNHEEVLWNHSPIKYVKNVKTPTLFLHSDRDYRCFMGDTFQMFTALKQLGVDTEMYLFHGDTHGLSRNGRPSNRIARANAIVDWFERYL*

>CD630_06550 Clostridioides_difficile_630_NC_009089 beta-lactamase-like protein

MNLAKGLDVLKISSNVLGEDKVMYIPAIYTEDDATLIDTGLPGQGDLIIDALNKSNTSFDRLKNIIITHHDIDHIGNINYLREKSKNNIKVYAYKSEVSYITGEETPFKLYMLEQMVDKIDDKMLSMLNVMGLGFKSSYTKVDVSLDNHEKLNLGEEIEVIHTGGHTRGHICLYLKESKVLIAGDLLQVENGELKPVDVMHSNKQELKDAIKNISNYDIETIVFSHGGLYQRNIIETLKNLIIE*

>CD630_06730 Clostridioides_difficile_630_NC_009089 methyltransferase

MIFTKSNKYDKDFLMKNMMGPNCIKILEELTSKIKLEKGMRILDLGCGKGISSIFLAKEFDATVFATDLWIEPTENYKRFKEFKLDDKIFPIQAEAHELPYAEGFFDAVISIDSYHYFGNKEGFLDNHISPLVKEGGILAMAMPGLKEDFVDCIPDELIPFWQDNMNFHSITWWNKLWSESESVIVEKCEALNCHDEAWKDWINCDNSYAINDKKMMEVENGKYFNTISLIARTK*

>CD630_06870 Clostridioides_difficile_630_NC_009089 50S ribosomal protein L20

MARVKKAMNARKKHKKILKLAKGFRGSRSKLYRPANTFVMKALKNAYIGRKLKKRDFRKLWIQRINAAARMNGISYSRLMNGLKLSGVEVNRKMLSEMAIQDPEGFAKLAEVAKAKLA*

>CD630_06930 Clostridioides_difficile_630_NC_009089 MerR family transcriptional regulator

MFKIGDFSKLSKISIRMLRHYDEIGLLTPSHTNKTNGYRYYSADQLSTTNRIHALKDMGFGLYSIKEILTEYNDKESLIKYLNIHHSQVKEQLEDTQKKLLKIETTIKRIGGNDIMKNYDVTIKNFAPKYMMTLRRVIPTYQDEGMLWHQAFLETKDQNVQIEPPKYSKAVFYDTGYKEDYVDVEVQVAVSGKYKDTEHVKFKTVPSVTAATAIVNGNFNQVADACEAIGNWISDNNYDVDGPMFNIYHVSPGNDSNPDNWVTEVCFPVKKK*

>CD630_06940 Clostridioides_difficile_630_NC_009089 TetR family transcriptional regulator

MKSNDKYARERIIEVTLNLLNEVDDIEEITVRKIAERANVGVGLINYHFKTKDNLLSTAIGDVMSNIIAELYDDSVYTLRPIEDLKNLLKKLCDTGLHYEKVLPFVLNQCITNGDMQAELDIVPMLRKIFGNKKDEMSLRIIALQIILPIQISALSTESFQLYSGINIKNKYERDKFIDILIENIIGEDVDVR*

>CD630_07000 Clostridioides_difficile_630_NC_009089 phenylalanine--tRNA ligase subunit beta

MLVSLKWLRDYVDIDIDVKEFADKMTMTGTKVETIDYYGEEIENILVGKILEIKQHPNADKLVVTKVDIGDKVVQIVTGATNISEGDYIPVAVNGSKLPGGVEIKQTDFRGELSDGMMCSAAELGIDEHYIEEYKRGGIYILDHEDSYELGKDIKDVLGLKDALIDFELTSNRPDCKCMMGIAREAAATIGTKVKYPEIEVKESDEEIDFKVEIDNPDLCRRYVARMVTDVKIEPSPYWMQRRLTEAGVRPISNIVDITNFVMLELGQPLHAFDINQVETGRIVVRNAKDGEKLVTLDDVERTLDKDMLVITNGEKSLGLAGVMGGANSEITSNTKTVLFESANFKPENIRMTAKKVGIRSEASSRNEKDLDPNLAEIAANRAAQLVEMLGAGKVLKGVVDVYPNKPEPKKLVVNPQRINHLLGVDVPMEQFVGILESLEFKCNLVANDKLEIDVPSFRTDMEQEADVWEEIARIYGFENIPSVQLEGNTTAGIKTSKQKFMDALKDNSTAVGLNEILTYSFVSPKGVDKIRVPEGNAKRNFVKLLNPLGEETSVMRTTLIPNMLDVLSTNVSHKIEEVSAFECGHIFIPQDSELPKEENRMCVGMYGKDVDFFTLKGTIETILVNVGFKCYEIEPQDNNTTFHPGRCAKIVYNNKYVGTLGELHPDVIENYNLGQRVYVAEIDIDFVFDNSDRTKNYVPLPKYPSTSRDIALIVKDDVFVKQIEDIIKENGQGLVESYKLFDVYKGSQIEAGYKSIAYSITYRSKDKTLTDEDVAKVHDKILSELSEKLNANLRSN*

>CD630_07010 Clostridioides_difficile_630_NC_009089 cell-division protein

MNKVMVKIHGAEYPMVGDKSEKFMISIADFVDKEMDKITRQNPKLSLSVAAILTALNISDLLFECSDENEKLIKANEELSKKVGASNEELQLEIKSLKLTIAEKEAENREAETKMKELIEIIENKKQEIFELSNTTEGSRAELDAYKNKIEELSTQLEEANERATIAENLASEFQNKAYDLQLKCTGLNNDAKNVE*

>CD630_07050 Clostridioides_difficile_630_NC_009089 NUDIX family hydrolase

MKLTTICYIEKDDKTLMLYRNKKKDDIHEGKYVGVGGKFEQGETPEECVIREVKEETGLTLKSLSYKGLITFPKFKDEEDWYMFLYFSDEFEGELSEKDLNDCKEGNLIWVDNDKIFDLNMWEGDRLFLNWAKTGNIFSAKIVYDNGKLKDYNVSFLD*

>CD630_07060 Clostridioides_difficile_630_NC_009089 Fe-S domain-containing protein

MIDINLEKCVGCGMCESDCLVNAIKVKDDKAKVKNILCINCGHCMAICPTDAIEMQGFDKNEVIEYNRETFELEPEKLLNFIKFRRSIRQYKDIEVEEEKIKNIVEAGRYTPTGGNRQPIRYILVKEKLKEVKELAIQGLYNLALDTDDNDPVRSIYKNTFKKMYKRYKENGNDSLFYDAPLLMVVVGDMSLGGSAYVDGGLAASNMELMAYSQGLGICYNGFFVMASNVEPKIKELLGMSENEAVITSFILGYPDVKYKRTVNRNTAKFEVR*

>CD630_07080 Clostridioides_difficile_630_NC_009089 peptidase D

MGNVLEGLKPESVFKNFEKISQIPRGSGNEKGISDFLLSFGKNLGLETIQDESLNIIIRKPATKGYENCPGVVLQGHMDMVCEKEKNVEHDFLKDPIKLRIDGDMIYATGTTLGADNGIAVAMGMAILEDNTLEHPALEVLVTVNEEDGMNGADALDPSLIKGQYILNMDSEEEGYLLVSCAGGKTCVVSLPVEYKEVKGDKQGLLVEVTGLLGGHSGMEIVLQRANANKAIARVLSVLNVDYELASVDGGTKHNAIPREAKCVIAVNKADVESAKKQINDILTAFKHEFTTSDPGMTYSVAETSVDKVLTKDCKEKVVQMSCLTPHGVQSVSLDIEGLVESSTNFAIIETKESTIEFLTSVRSSVMSIRDEIADRIRLLAQALGANYDLIAQYPAWEFKKGSKLEKICSETYEKLTGKVPTVMALHAGLECGLLLDKLPHAEAISIGPDMFDVHTPNEHVSIPSVANVWDYVIEILKSMNQY*

>CD630_07140 Clostridioides_difficile_630_NC_009089 hypothetical protein

MDFKIEKYLLKKAEELAFITIKHGGEFKLKSYEVPKGGLDVPIKNEVLVKGIKEKTAQDKLNSMSIADAMIYIIGIDSKFKNNAEYEKFLNALSKDIDLDLKSYMGYMSRKYFEIGEHTDSLIYIKAFITMYPDDLDAMYNYAIVCQEIAKQYQKDMDDKAMNAFLLEAMAKLEKVIDVDENFALGYYHLGYHYYNQGQYLKTKLTWEEALRLGLDADLVAEVQENLGKMDFKVQYEEGYTLVFQGKFKEGLEKLLPLEEEHMDWWNLLFMIALGYKGMGEIEQAKMYLEKILIIKPNQVDTIVELGLCEAYKNNLDKAIEYFEQAAKIKEDPEILCNLGMAYLNNGDIDDATYYIERAYELNPQDEITIVCLRELGINK*

>CD630_07170 Clostridioides_difficile_630_NC_009089 bifunctional carbon monoxide dehydrogenase/acetyl-CoA synthase accessory protein

MKIAITGKGGVGKTTFSSMLSRMFAEDGYRVVAVDADPDANLALALGFPKEVYESIVPISEMKKLVSDRTAASVGSFGKMFKMNPKVDDIPENFCKEYNGVRLLTLGTVDSGGTGCVCPEHVLLKRLCSHLILQNKDVVVMDMEAGIEHLGRGTAQGVDAFIVVVEPGERSLQTYRKVKKLGHDIGVNKVFVVGNKIRNKEDEEFIIQNLEDGESLGFIYYNQDVIDSDRANQSPYDSSETTKEQIKAIKDKLMSLKDK*

>CDIF1296T_00890 Clostridioides_difficile_ATCC_9689__DSM_1296_strain_DSM1296_CP011968 FolD bifunctional protein

MEGMSTKGQIIKGKPVADKISEELIKEVDLLVKEGINPKLTIVRVGARSDDLSYERGALKRCQNIGITTEVLELAEDITQEEYIDVLKRVNDDKNVNGILCFRPLPKHLNEEVIKYVIAPEKDVDCFSPINSAKVMEGDKSGFPPCTPTAVVEILKHYNVDLKGSKVTVLGRSMVVGKPVSMLLLSEHATVTICHSKTKNLSGVAAEADVLIAAIGRAKMVDESFVKDGAVVIDVGINVDEEGNLCGDVDTNAVLDKVSMITPVPAGVGSVTTSILAKHVVKACKLQNNK*

>CD630_07210 Clostridioides_difficile_630_NC_009089 hypothetical protein

MIISENKPLEEVLGYLKDFDKLVLVGCNQCAATCKSGGEEEVLKMKETLEGEGKKILGYVMLDPACNLLKSKKDLKALKEETKEADAVLSLACGDGTQTIVKNLKDKPVYPANNTLFIGEVQRVGEYEEACKACGDCELGWTGGICPVTMCAKGLMNGACGGAKNGKCEVNSENDCAWIKIYERLEAIGQLDNLAEIRPPKDYSKQNNPRSLSAKKKKEAAANS*

>CD630_07230 Clostridioides_difficile_630_NC_009089 bifunctional carbon monoxide dehydrogenase/acetyl-CoA synthase dihydrolipoyl dehydrogenase subunit

MKIVVVGGGPGGYVAAIKASMLGADVTVVEKRRVGGTCLNAGCIPTKALLASSGVLNTVKEAKDFGIEIDGTVKPNFTAIMERKNKVVNQLISGIEFLFEKRGVNLVNGFGKLIDKNTIEVTKDDGTVETIKADKIILANGSVPVVPRMFPYDGKVVITSDEVLGLEEIPESMLIVGGGVIGCEIGQFFRALGTEVTIVEMVDQILLNEDKDVAKQLLRQFKKDKIKVITGIGVQTCEVVDGKAVATLSNGKVIEAQYALVCVGRRPNLDNSGVEDIGIEMERGKVVVNEHLETNVEGIYAIGDIIDTPFLAHVASKEGIVAVENALGKTKVVDYRAIPRCVYTEPEVAGVGKTEKQLEAEGVEYNVGQFDFRGLGKAQAIGHFQGFVKVIADKETDKIIGAAVVGPHATDLLTELSLAVHLGLTVEQVGDAIHPHPSLSEGLMEALHDVHGECVHSVPKL*

>CD630_07250 Clostridioides_difficile_630_NC_009089 bifunctional carbon monoxide dehydrogenase/synthase complex subunit delta

MAFKMSTQKYSGKISEVEVGIGEKAIKLGGENVLPFYSFDGEVGNSPKIGIQISDVYPESWTDSYKELYKDVANCPVEWAKYVEANTQADFICLKFDGSDPNGLDKSVDECADVAKAVIEAIKLPLVVAGSGNHEKDGKLFEKLAQTLDGHNCLFMSAVEDNYKGVGASAGMAYAHKVGAESSVDINLAKQLNVLLTQLGVKGENIVMNVGCSAVGYGYEYVASTMDRIRLAAFGQNDKTLQMPIITPVAFEVGHVKEAIAPIEDEPDWGCPEERTIAMEVSTAASVLVGGSNAVILRHPKSIETIKELVNALA*

>CD630_07360 Clostridioides_difficile_630_NC_009089 hypothetical protein

MIFDKEIVRYHHGHHDHDHCHEHTHGDICHEHPHDHAHDHDHEHSHEESSESKDEKTLKILLVHWINHNETHEEGFREWVEKARAIGKEETAKSIEKAIEYMEEANKMLLEAKKHM*

>CDIF1296T_00908 Clostridioides_difficile_ATCC_9689__DSM_1296_strain_DSM1296_CP011968 membrane associated lipoprotein

MKKRLIIMILSVVLVLSSILTIFAYSNIKYNNNNKLIYSNMIDKKTQNSVKEILKENKINEKDIDTFIKAVNNYNKLQVKILQNNINISKSGYSSINAKQVPYNLEKLQDNWVKKFPDYMDVNCRITAFRLFKDFINSNKKFTGDSIDLNVDLDTIMNNKDAKFSTKDVEKFINFFSAIPAKDTDDTIKISEQIKNEWKKRKISFKNNKNISIINGFLRYPETKNVFIGHTGICIKTKNGILFLEKYGVTSPYQVTKFKNKEDVKNYMFNRLKMSEGEIELPDPIIMENDKLMK*

>CD630_07390 Clostridioides_difficile_630_NC_009089 hypothetical protein

MKVKKFKSGIVMALTVMAIGASLTNVSAMELNKSKEFETKYNEMKRDFQEEYGKEDKFNITLDDEFTKEEVNGAKGIIKTTNKMNNEVVVLNYFDEMKSGIEFELKPKAEKNAILRDEFQQGYGKEDKFKVVLDDEFTKEEVNGAKGIIKTTDKETGEVIIYNYFDELEK*

>CD630_07450 Clostridioides_difficile_630_NC_009089 OmpA/MotB proton channel

MFMLYDEDEKEENNERWLLTYSDLITLLMIFFVIMYSMSNVDAEKYKQLSQSLNSAFGGSSGVIEGGNSKIEPVVEPGSNDLDTLQNAKFKKVGEEIQKYLNENGMANSVSLRVQDRGLVISLKDTILFDTGKAIVKDNSRDKIIQIGKMLNEMNSYMRVEGHTDNMSIKNSEFKSNWDLSVMRATNVVQLLIDNAGIAPDKLSAVGYGEFRPIAENSSEEGRSKNRRVDIVLVDSKYDNVENVSKDK*

>CD630_07500 Clostridioides_difficile_630_NC_009089 amino acid family ABC transporter substrate-binding protein

MKVFKKLLSLGLVLGLTLSLVGCSGGGEKTKLEQIKDNGKLVVGTSAEFPPFEFHKVVDGKDSIKGFDIMLAEEFAKELGVKVEIKDMSFDGLIGALNADQVDIVLAGMSPTPEREKSVDFSELYYLSRNAVIVKDADIDKVKTEDDLKKLRVGVQAGSIQEEYVVNTLKMTTTKSLKAIPDLITELKNGNIDAVVTNEAVSLINVKKYDGIKMANTEVGKDVTEGMAAAIKKSDNNKDFIELLNKKIKELQDGKKIEEFLNEASTEAASN*

>CD630_07520 Clostridioides_difficile_630_NC_009089 amino acid family ABC transporter ATP-binding protein

MITIKNLSKSFGDLNVLKNIDLEIAKGEIMVIVGPSGSGKSTFLRCMNLLEIPTGGEIIFEGKNLVDKKTNIDEVRQNIGMVFQNFNLFPHKTILDNITLAPIKLKKMTKEEAEKKAEILLSRVGLLDKKDSYPSQLSGGQKQRIAIARALAMEPDMMLFDEPTSALDPEMVNEVLDVIKELAKEGMTMAIVTHEMGFAKEVADRVIFIDGGSILEDNTPEEVFGNPKHERTKAFLAKVL*

>CD630_07560 Clostridioides_difficile_630_NC_009089 nitroreductase

MNNNFQDNQTINLIQSRRSIRKFTTEQISDEQVNTLLHCAFAAPSGCNKQPWHITVVQDQKLLKEISDDTLSRIHEVSNVEINKNFKLFYGAPTVLFISYDESSSWAPYDIGILTGNITTAAQALGLGSCIIGMVRGLFTPVEQGDIEGLVSVLDKEDVKESESIKMKFDTNKKYRELLDIPEGYSVPFGIAVGIPDGNLPNAREVVYKVSRV*

>CD630_07610 Clostridioides_difficile_630_NC_009089 ATP-dependent RNA helicase

MNITKFEDLPISEGIKKAIAEMGFEEPSPIQAQSIPAILSGKDVIGQAQTGTGKTAAFSIPILETIDPNNRSLQAVVLCPTRELAIQVSTEIRKLAKYSHGIKTLPIYGGQPIDRQIKSLKSGVQVVIGTPGRTIDHINRKTLKMDNVKMIILDEADEMLDMGFREDIEMILSKIPEERQTTFFSATMPRGILELTKRYQKDPEHIKVVRKELTVSNTKQYYIETRSSNKLEVLCRLVDVYDPKLSVVFCNTKRKADELVGDLQARGYFADALHGDLKQTQRDIVMDKFRNGTIDILVATDVAARGIDVDDVECVFNYDLPQDEEYYVHRIGRTGRAGREGMSFTFVFGKEMRKMKDIERYTKSKLIKHNIPTITDVEEKKVGTFFAQVKQTIEEGHLTKQLQWLEGFCNDEDYAMVDIAAALVKLSLGEEMKEEIIEEKPRRERGDRKGGTGAKDGMIRLFINIGRNQRVQAKDIVGAIAGEVGIPGKVVGTIDIYDKYTFVEIPKKDAKTVIEKMKDIKIKGNKINIEKANKKKK*

>CD630_07820 Clostridioides_difficile_630_NC_009089 sporulation protein YunB

LNRIVDRKTKNEFRKIIAIFLVILFLSVFIGSFIYIDKTLRPTITVLAETKALELANRSINKAVAEMVEGKINYEDLMDIQLDNNGKITMIQANTIMMNEIASAIALEIQDELKKDKTASSYIPIGTALGSPILAKYGPKLEVSIEPIGTVSVNFKTEFESSGINQTRHRIYLEAQTQVKVVIPLITSTKQIKAQIPICETIIVGDVPESYVNIPEKNLGNVLPNTGKNTNK*

>CD630_07850 Clostridioides_difficile_630_NC_009089 ABC transporter ATP-binding protein

MLVVENVSHGFGARTILENVSFRLRKGEHIALVGANGEGKSSFLNIITKKLMPDAGNIKWSSRATVGYLDQHTVLSKGKTIREVLREAFKHMFDLEQEMIAMYDKMGEASDDEMSKLLEETAEIQTILENSGFYMIDAKIQEVANGLGLGEIGLDKDVTDLSGGQRTKVLLTKLLLENPTILILDEPTNYLDEEHITWLTKYLQEYENSFVLVSHDIEFINNTCNVIYHMENGELNRYKGNYDEFVRLNDIKKRQEEQAYDKQVEERKRLEDFVARNKARVATRGMANSRQKQLDKMEILERPKEKIKPTFAFKDARAASKIIFETENLVLGYDEALTKPLNFHLERGKKIALKGMNGIGKSTLLKTLLGIIKPFEGNVKLGDYLEVGYFEQESSRENSNTPMDEVWSEFPGLTNFEVRQALAKCGLTNEHITSQMRVLSGGEAAKVRLCKVMLKNINFLVLDEPTNHLDVEAKDELKKAIKEFKGTVLLVCHEPEFYSEIVDDVWNIEDFTTKIV*

>CDIF1296T_00969 Clostridioides_difficile_ATCC_9689__DSM_1296_strain_DSM1296_CP011968 3-hydroxybutyryl-CoA dehydratase

MESVMENLNNLKVELKDKVCVITINRPKALNALNSDTLRELSQVIDVVSENEAILGVIITGEGKVFVAGADIRQMQNYKSEEGRKYAGYAQGIFDKIEALEKTVIAAVNGYALGGGCELAMSCDIRIASEKAIFGQPEVNLGVIPCFGGTQRLSRLVGTGIAKELIFTGRQVNAEEAKSIGLINKVVPSDLLLEESMKMMNQIVEKAPIAIRYAKVVINKGIDMDLKNALELEKDIAGLTFATRDKQEGMNAFIEKRKPVFENK*

>CD630_08030 Clostridioides_difficile_630_NC_009089 acyl-CoA dehydrogenase

MDFRLTEAQLMLQRVAKEFAENEIAPIAAETDKTGIFPRELFSKMAKIGFNGIGTPVEYSGSGGADIEKVIVVTEIAKKCAASAAILSIHTIYAQAILKFGTEEQKKKYLPMMAEGGCVGAFALTEPNAGSDAARAATTAIIDEETDEYVLNGTKCFISGGGQAESLIIFALTDPSKGIKGMSAIIVDKGTPGFSIGKIEEKMGIHGSETAELIFDNCRVPKSNLLGKEGKGFNIAMTCLDGARIGVGAQAVGIAEGALEESIKYSKERVQFGKPISALQGIQWYIADMATMVESAKLLVYYAADLKARGEKHTKEAAMAKYNASRTAREVTNLALQIHGGYGYMKDYPLERMYRDAKITEIYEGTSEIHKVVISRAVLG*

>CD630_08060 Clostridioides_difficile_630_NC_009089 sigma-54 dependent transcriptional regulator

MKEQWYKDIFARVLSMTDDGFIVVNTSGVIIDINDKYCDFLGKERKDIIGQNIQSIIPNTKMLDVMKNKYCEEGAIHHYSGGNTKEKSVIVSRSYVENDNGEVVAGVAQVKFRLQSFDVAKKLMSEYMELQYYKEQFKDNCGFDKLIGENRDFIELKKTGVKASKTNFPVLLTGETGTGKEVFARAIHNNSSRSDKPMVSINCAAIPEELLESELFGYDEGAFTGAKKGGKKGKFLVANNGTIFLDEIGDMPLTMQAKLLRVLQESEIEPVGGLKTIKIDVRVISATRKNLSKMVEEGLFREDLYYRLNVINIHMMELKDRQDDILLLANYILNKLNVEYKELKVLSDKVKNCFINYTWPGNIRELQNVIKSAYAVSDDMVIMMCDLPSKMDNISRVAQCNVDSNCSIHEMVENYEKSLIIDVLRKYNWKCSKAAEVMGIHKSLLYKKIKKYEIELNN*

>CD630_08070 Clostridioides_difficile_630_NC_009089 hypothetical protein

MDQIKRLHELQQKSYLTKEEFEELKNNTYIERFELRDECDNSYIYTFYTNDKCNDIKFIKSQFIVTLIKIGFLCECGGVFRQKKIVDEYDYGYNAIYKCDSCGKELDKEVENYD*

>CD630_08090 Clostridioides_difficile_630_NC_009089 hypothetical protein

MHEDKNDIFESIDTEDFDNKTEEECLELVRKNGLNLIYIKNQTEEICLEAVRQNCNAIKCVQNQTEKICIEAVKQDWRMLEFVEEPTEEICMLAINQDGTALKYIENQTEELCLRAVEKNGAALEYVKEQTEEICIEAVRNSEFGLARVKNQTEKICMEAVKHCSYNLKWVENQTEEICMEAVRQNGLDLKFVKNQTETICLRAVRQNGMALEFVKEQTVGICLKAVRQYGMALKFVKEQTEEICTEAIKQDKRALSFVKGDKEKYKALYDNNEPFAKRYVRNVIEKENRALIKKGEENAKIKIAGKLYDRGMSFEDISDIVEIEISKLKVSLGVF*

>CD630_08180 Clostridioides_difficile_630_NC_009089 6-phospho-beta-glucosidase

MNTGFPKDFLWGASSSAFQVEGAWDKDNKGKTVADYNSFKKSHLQADTKVASDFYHNYEEDIELMKELGMKTYRFSISWARIIPDGEGEINQKGLDFYNKIIDKLIECDIEPFVTLYHFDLPFKLVEKYNGWESRETVYAFERFAKICFKHFGDRVKYWQPHNEQNLIVRVEERINIYDETDSWKIDKIRAQMDYNLCLAHALAVNACHEMIKESKIGAAVSSSVTYPLTSKPEDVYAARMNDNFKVYYMLDMHHYGEYPGYYMKYLEKRNIVPHMEDGDKEILKKAKMDFIAVNYYRTNCAEALPEDSQHPFGLREGTVDFSMYGLFKMSMNPNLEASEYGAAIDPSGLRVALNEYWQRYHLPVIITENGLGAKDILEDGKIHDDYRIDYLRSHINACKLAIEDGVEMIGYCPWSFTDLLSSSQGFNKRYGLVYINRTDHEVLDLKRIKKDSFYWYKEVIENNGIVK*

>CD630_08270 Clostridioides_difficile_630_NC_009089 rubredoxin oxidoreductase

MCSEQKFFICETCGNLVGMIQSGGVPIFCCGKPMKELVPNTTDAAVEKHVPVIEVDGNNVTVKVSSTTHPMTKEHHIAWVYLMTEQGGQRKCLAVDGEPVVKFALNDDDKVISAYAYCNLHGLWKAEL*

>CD630_08310 Clostridioides_difficile_630_NC_009089 ATP-dependent RNA helicase

MDFKSLGISENTINILKKSGITTPTPIQKESIKLIKEGKDVIAEAQTGTGKTLAFLLPIFENISLDINDIQVLILSPTRELAIQITEEAMKLKESKDVSILAAYGGKDIGSQIKKLKGNIHMIIATPGRLLDHLNRKTIDLSKLKTFVLDEADQMLLMGFKNEVEAILKETSNKKQTLCFSATINSQVKKLAYRYTKNPVVVSIQKEEITLNNIKQEVVETTDRKKLDALCKVLDEDNPFMAIIFCRTKRRVDNLEEALAIRGYNCQKLHSDIAQSKRERIMKSFRNLDIQYLIATDVASRGLDISGVSHIYNYDLPETPEDYIHRIGRTGRAGEEGYTCAFIDPKNERMLSEIETAIESKISRRIIEL*

>CD630_08400 Clostridioides_difficile_630_NC_009089 isomerase/hydrolase

MKFVTFCSSNEEKIGVFNSETNSIYEINSLGLSKLYTDMNDFIENVSTGDLEKIKNNSFENAKCYKLEEVKLCSPIVRPKKDIICLGLNYKDHVNEIPDGVIKNVVMPDYPIYFSKRADKIIGVDDKISLHGDLVEKLDYESELAVIIGKEGINISKEDAYEYIFGYTIVNDISERALQDKHVQWFRGKSLDTHTSMGPCIVHKEEFEHPLKLDISSVVNGEVRQDSNTEYFIFDIPTIINDLSRGMTLKPGDIISTGTPAGVAMGMNPQVYLKHGDIVECKVEGIGVLKNIVD*

>CD630_08410 Clostridioides_difficile_630_NC_009089 5-aminoimidazole-4-carboxamide ribonucleotide transformylase

MARELELKYGCNPNQKPSKIYMKNGELPIEVLNGKPGYINFLDAFNSWQLVKELKEATGLPAATSFKHVSPAGAAVGVPLSDTLKQIYFVDDLELSPLACAYAMARGADRMSSYGDFIALSDVCDKETATIIAREVSDGIIAPGYTEEALEILKGKRKGNYNIVKIDENYTPEPIETKDVYGITFEQGRNEILINEDLLKDIPTDNKIFTDSAKRDLIIALITLKYTQSNSVCYAKDGQVIGVGAGQQSRIHCTRLAGNKADTWYLRQHPKVLNLKFKKDIGRPDRDNTIDVYLSDDYMDVLADGIWQNFFEEKPEPLTGEGKRAWLKTLTGVALGSDAFFPFGDNIERAKRSGVSFIAQPGGSIRDDNVILTCNKYNIVMAFTKNRLFHH*

>CD630_08510 Clostridioides_difficile_630_NC_009089 membrane protein

MNYKLILAIVFITSALIFYTIGVFGERKAKILKKKHVIIFWLGFIFDTLGTFTMSNIANSHTFEVKSALSQNLHSITGLLAIVLMLFHASWATFVLYKDDEEKKKFFHKFSIVVWTIWLVPYFIGMFIGMAG*

>CD630_08560 Clostridioides_difficile_630_NC_009089 ABC transporter ATP-binding protein

LEHLLEVNNLSVSFKVEEGEVQAVRNVSFNLKKGETLAIVGESGCGKSVLCKSLMRILPYNGYIKNGEVLLKSSDLVKKSEKEMEDIRGKNISMIFQDPMTSLNPTISIGKQIAEAVIIHQGISKSEAKKRAIELIELVGIDNPEKRFKQFPHHFSGGMRQRIVIAIALACNPDVLIADEPTTALDVTIQAQIIDLIKDLQHKIGLSIIFITHDLGVVATIADRIAVMYAGKIVEIGTVEDIFYDPRHPYTWGLLGSLPTLDSQDDYLYNIPGMPPNLLNPPKGDAFAIRNKNALKIDYEKEPPMFKINDTHSAATWLLHPDAPEVDVPVRVNCGRVISNE*

>CD630_08600 Clostridioides_difficile_630_NC_009089 hypothetical protein

MQDKILRDKKANDPWARMTTRNGFSADEIISSFQKAIRRNMVEEACEFAYELYISSPELEDKLWRRILTISVEDIGMGDPSAVIIINNLNQVRKEYSYADGDRPLFFIHAIRYLCACEKDRSSDLLKNIIIKGFAMGKVPEVMDVALDKHTYRGLEMGRDSFHFLNEASIVIPEKEVDNDYKERYLKILEKYKQEEVIDTAFKFNHWQY*

>CD630_08650 Clostridioides_difficile_630_NC_009089 ADP-ribose binding protein

MKWRDYAADVNLFEDFDKTIKPLTDEQRRKNINTLIAYFSKEVPSKIYNLSNDEIKPRDILRGLLNVYPPKEIAPEILNMLHNLLLIECEERELVDVNDIEEIEEGIAIWRGNITNLRADAIVNAANNKLLGCLQPLHLCVDNEIHSCAGPRLREDCDKIIKKQGHLEYTGDAKITRGYCLPAKFVVHTVGPIVSGGQPSKEQEKQLLHCYKSCLNTIKEIDEIKNIVFCGISTGVFGYPKKEAANLAVSRVRLWLKENPEKNLKVVFNVFTEEEEEKYRRIFK*

>CD630_08710 Clostridioides_difficile_630_NC_009089 molybdenum-specific ABC transporter ATP-binding protein

LSLYVDIEKDLSSFKLKVEIKQEKGTLGFLGESGSGKSMTLKCIAGLEKPTRGKIVLNDRVLFDSEKKINLSTQDRKVGFLFQNYALFPHMTVSQNIELGLLKLSKSEKKEIVARYLDILKLNGFEGRYPWQLSGGQQQRVALARALATSPDILLLDEPFSALDHHLRSNMEKELMNMLKDYKGDILFVTHDIEEAYRVCDDIIVYNKGEGLPKRPKKELFESPKYLIEAKITGCKNISKLNRLDKNTIYATDWGCELTLNREIGDNIEYVGIREHHIKVLDSNEDLNEKLCFELINIVENPFTYTIYVRKTDLSNECVPIQIELEKSKMRFKKGDRIYLDFPQEYLFCFRYNYNKKE*

>CD630_08720 Clostridioides_difficile_630_NC_009089 maltose O-acetyltransferase

MTEKEKMLSGKGYYANDELLVKEREYCKKLTRLFNNTLEDEYEKREDILRQLFGSVGKQINVEQNIRCDYGYNIHVGENFFANYDCIFLDVCKIEIGDNVMLAPNVQIYTAYHPIDAQLRNSGIEYGSPVKIGDNVWIGGGVIITPGITIGDNVVIGAGSVVTKDIPPNTVAVGNPCRVIKKIEE*

>CD630_08810 Clostridioides_difficile_630_NC_009089 hypothetical protein

MGTKIVLSIVLIVVVVAISLTCIRVIKQSKVGIIMRLGKFQKVAETGVHFLIPFLDKMAYVIDLREIVIDFPPQPVITKDNVTMQIDTVVYYKVTDPVRYVFEIANPIAAIENLTATTLRNIIGELDLDETLTSRDIINVKMRTILDEATDKWGIKVNRVELKNIMPPQDIQVAMEKQMRAERERREAILQAEGNKSAAILQAEGEKQSAILTAEAKKEAMVRVAEGEKESAILVAEGEAEAIRQTAIAKAQGEAEMIKRTQMATAEGLKLVFSAMKEADIDNNILALKSMEALEKMAEGKSTKLVLPSEAVNFLGTFKGIKEVMSDDNKEVLDIKEVLNDNESLKK*

>CD630_08820 Clostridioides_difficile_630_NC_009089 glucose-1-phosphate adenylyltransferase

MKKEMLAMILAGGQGSRLGVFTKRIAKPAVSFGGKYRIIDFVLSNCSNSGIDTVGVLTQYRPLILNSHIGMGSHWDLDRINGGVYVLQPFMNEKEGNWYNGTAHAIYQNMDFVDTYNPEYVLILSGDHIYKMDYSKMLKFHKEKGSKATIAVIEVPWDEASRFGIMNTNEDSSIYEFEEKPSEPKSNLASMGVYIFDWKMLRNYFKEAEKNPEINYDDFGKNLIPKMLEDNVGMYAYPFKGYWRDVGTIQSLWDANMDIIKSPETLDLADPKWKIYTNTMAMPPQYIGKNANVHRSMIADGCRILGEVGNSVLSHGVVVGKGSKVIDSVIMPNVVIGENVTIEKAMIGECATINDNVQIKNVNNEINVVSEYENIEPRCVLIEGGL*

>CD630_08840 Clostridioides_difficile_630_NC_009089 glycogen synthase

MKVFYVTAECWPFAKTGGLGDVSYALPKELKKEGVDVRVIMPKYSTIPSYLKDQLKEIAVFSVRVGWRNQYCGLLEMELDGVKFYFIDNEFYFRREDERKSIYGYGDDAERYTFFTDAVLEAISRIDFYPDVIHINDWHTGMLPLILKERYATLEGYKNIKTMYTIHNLQYQGVFDKHVLYDILDLPQKYFDNGDIEYYGSINFMKAGINFADKIITVSPTYANEIQTSFYGEQLDGLLRKESGKLKGILNGIDYDLNDPAKDKDIFVHYDVDSINKKVENKLRLQDILGLKKDSSIPLIGIVSRLVSQKGFDLIAYMMPELMREDLQIVVLGTGEHQYQSMFNYYDSNFSDKVSARITFNASLAQQIYAASDMFLMPSLFEPCGIGQMLAMRYGSLPIVRETGGLRDTVTPYNKFTGEGNGFSFKNYNAHEMFFCLKNAIKVFKDKEKWIKLVENAMKTDNSWKKSAKEYIETYRDICD*

>CD630_08900 Clostridioides_difficile_630_NC_009089 polyamine aminopropyl transferase

MELWYTEEWTENVRFSIKVNKHLFEGKSQFQRIDVFDSDEFGKFLTIDGLMMVTYKDEFIYHEMITHVPMATNLNIKKVLVIGGGDGGTVRELSRYPQIEKIDMVEIDKMVVDVSKEYMDICSCKLDDKRVSLYFEDGVNFVKCAHDKSYDLIIVDSTDPIGPGEGLFSTDFYKDCYRILTDDGILVNQSESPYFDFNAKEMKRANKKLKQIFPISEVYQAHIPTYPSGHWLFGFASKKLNPVKNQDRNGWEKLSLKTKYYNSDIHLGSFMLPQYVKEMLDEE*

>CD630_08910 Clostridioides_difficile_630_NC_009089 agmatinase

MKNNFYHMNTFMSMDKNYEESNLIVFGVGFDGTTSNRPGARFASSSMRKEFYGLETYSPFLDLDLEDYNICDYGDLEISVGSTEQVLKEIYQETYKIVRDSKVPFMIGGEHLVTLPAFKAVHEKYNDIYVIHFDAHTDLREEYNNSKNSHATVIKRIWDIVGDNKIFQFGIRSGTKEEFKFATEEKHTYMEIGGIDTFENIVNMLNGKNIYLTIDLDVLDASVFPGTGTPEPGGVNYREFQEIFKIIKNSNINIVGCDIVELSPDYDTTGVSTVIACKILRELCLIISDKIK*

>CD630_08920 Clostridioides_difficile_630_NC_009089 cold shock protein

MKNGIVKWFNNEKGFGFISVEGEDDVFVHFSAIQNDGYKTLEEGEKVSFDITQGNRGPQAENVNRI*

>CD630_09000 Clostridioides_difficile_630_NC_009089 glycine betaine/carnitine/choline ABC transporter ATP-binding protein

MIEIRNVTKKIGNNVILDDISLVVETGTLVVLIGSSGCGKTTTLKLINKLIKPTSGEIYINGKPISQENEIELRRKIGYVIQNTGLFPHLTIKENIELIPRLKKEKSVEEIEKRTLQLLEMVGLDSDEFLNKYPSELSGGQQQRIGVARAIATDAEIILMDEPFSALDPITRTSLQEQLFSLQDELKKTIIFVTHDMDEALKIADKICIMKDGRIAQYDTPENILRKPANDFVKDFIGEDRVWDNPEYIKARDIMIKNPIAVNSTRTVTQGIEIMRTSKVDSLLIIDRAKTLKGIVTVKDMKDIDDKSILLADIMSSEPLHVNEGDNLVEILNVMNRNSVGYIPVISDENKLVGLITRSSLLSVLSEQFLEMEVSVLG*

>CD630_09020 Clostridioides_difficile_630_NC_009089 cation efflux protein

MDNYKKVKQVLWIILFANFAVALLKIIIGNQIKSYSMTADGFHSLSDGASNIVGLIGIFFASKPKDKNHPYGHKKFEIITSLFISGMLFVIAIKIILSAVLRIANPVVPAITIESLIALIITLFINIFVCMYEYRIGTKLNSYVLISDSLHTRSDIFVSLGVLVTLVGVKLGFPVIIESIVPIIISAFIIYSAYGIFRPSIGILVDRVAVDEDYIKEIVFEFNEVRDVHNIRSRGSKSSIYIDMHVMVDPFISVEQSHDLTHKIEKQIQEEINENAQVIVHIEPFYSF*

>CD630_09860 Clostridioides_difficile_630_NC_009089 30S ribosomal protein S1

MFIMENDLTMQELLDQQEQVFSKVKVGELTTGKITAVRNDEVQLGLDYGFDGIIPISELNIEKNQYIEDIYHIGDEITAVITKVSQKDGTITLSKLQLDKRNDFAELQKAYDEHRIITVNVEKNIDKGVFANYNTYTFFIPISQLDTKFITDTSKFVGLNLEVYIKELDVRKNRLVASHRDVLQERINKEREERRAQIKAEKEAERARIKQEREEEKARIKAAKEDLFNSLEVGQKRDGKVTKIMPYGAFVDIGGIEGLAHINNLAWTRVESVEDVVSEGQEVEVYVLDVNKETKKIALALKDINNDPWDLIAKEVQIDDVVNAKVLRIIEKGAFVQIKEGVDAYLPISELSDTRVAKVTNVVNIGDEVKVKILDFKPKTKRMLVSIKEATREPEEDITEYLEVEESLGSIGELFKDKFKDLEV*

>CD630_09890 Clostridioides_difficile_630_NC_009089 2-isopropylmalate synthase

MKCGKYKKYDKMQIVNRKWPDNEIFKAPIWCSVDLRDGNQSLPTPMSVNEKVRMFKMLIDTGFKEIEVGFPSASNTEYTFLRKLIDENMIPDDVTIQVLTQSRAHLIEKTFESIRGCKKAIIHLYNSTSVLQRDVVFNMSKQEIIDIAVEGAKLFNEEVKKYPETEFTFEYSPESFTGTEMDYALEICEAVIDVWKPTPQKKVIINLPSTVEMATPNVYADQIEWFCKNISCRDSIILSLHTHNDRGTCTAASELGLLAGADRLEGTLFGNGERTGNMDIVNVGLNLYTQGIDPELDFSNIDKIIGIYEDCTKLMVHDRHPYAGNLVHCAFSGSHQDAIRKGMIAMKNRDNDYWEVPYLPIDPHDIGREYKEIIRINSQSGKGGAVYIMETDYGFMIPKNMHSDFGNVVKMESDRIGEELSSEAIFNLFKKEYIEVESPYKVKKYKIKSMDELNYENDDSNDTNMIEMTARISYMGNEQRIVGIGNGPVDSFNNALKQCGMKDYKFRYYWEHALEEGSHSRGVAYVGIEHNNEVYFGVSISENINTAAINALMNAINKSYIEEEIKNGDDYDAENISQTC*

>CD630_09910 Clostridioides_difficile_630_NC_009089 3-isopropylmalate dehydratase small subunit

MIANGSVFKFGDNIDTDVIIPARYLNIADYKELATHCMEDIDDKFISKVKKGDIIVATKNFGCGSSREHAPIVIKESGVSCVIASTFARIFFRNSINIGLPILECEEAANNIDEGDNIEVDFSTGVIKNITKGKEYKAEPFPEFMQNIILNEGLINSIKANRG*

>CD630_09920 Clostridioides_difficile_630_NC_009089 3-isopropylmalate dehydrogenase

MNCNIAVIKGDGVGPEIIDEGIKVLNKICCKFNHRFDCEYVLAGGCAIDETGEPLPNKTVEICRKNEAVLLGAVGGPKWDKCKGDKRPESGLLKLRESLGLFANLRPATMYESIKEASPLRTDIVEKGIDFVVVRELTGGIYFGERGRKIIDGIENAYDVEIYNENEIRRIGKRAFEIARNRNKKLISVDKANVLESSRLWRSIMEDLAKEFEDVELSHMYVDNAAMQVVKDPSQFDVIVTNNIFGDIISDEASMITGSIGMLPSASLREDSFGMYEPIHGSAPDIAGKDIVNPIATILSVSMMLRHSFNLEEEAKCIEDAVQSVLNKGYRTIDIYNGVGNVVGTRAMGELIVNEI*

>CD630_09930 Clostridioides_difficile_630_NC_009089 thioesterase

MDFLAKYKVLKKQNSSKSCLVCGTQNELGLKADFYELENGELVSICNTKDWHQSYPGRVHGGMSAAILDETIGRAVSINDDQIWGVTVSLELKYKKPVPTDATIKVVGRITKENRKLFEGTGEIILPNGDIAVTATGKYMKMPIGQIAEGDFSNEEWFFEESKEKVEYIEL*

>CD630_09950 Clostridioides_difficile_630_NC_009089 D-3-phosphoglycerate dehydrogenase

MYNILVTDGIEKEAARKLRELDFNVIEQFYEKDVLGDKLKDVDVLVVRSATKVTKDVIDKALEGKKLKLIVRGGVGLDNIDVKYAQANGIKVMNTPNASSISVAELTIGQLFVLARFINTANVTMRDGKWEKKKYKGTEINGKTLGLIGFGRIAKEVAKRAELLGMNVIYTDIMGEAQGFNNYKFCDMEEVLENADFLSLHIPFDKNKGAVITEKEINKMKKGAYLINCARGGLVDEKDLLKALDEGKLSAAAIDVYEQEPTLNLDLVNHPRVSPTPHIGASTVEAQERIGDEIVNVIQDFFLDFNNLIGVAL*

>CD630_10010 Clostridioides_difficile_630_NC_009089 nitrate/sulfonate/taurine ATP-binding protein

MEKIKLSIENINKRYDSRIIFRDFNIDFYVNEVNCILGKSGCGKTTLLNIISGIIKNDTNNLNIKENLNRVGNKLEASYIFQDDRLIDWLTVEENIKIVVNKYYNKTQLNKICDEYLELVGISDYKKFYPQMLSGGIRQRVNIARAFIYPSKNIIMDEPFKSIDAKNTQLIMDNFRNILRKEKRTVLFVTHNIEEALFLADRIFILGDSPIRIKKILKNSKELEKNEVLKLI*

>CD630_10040 Clostridioides_difficile_630_NC_009089 fumarate hydratase subunit B

MIKITTPVNEIDIAKLNCGDTISLSGILYTARDAAHKRLIDCINKGEELPFDVYGQGIYYVGPTPTKPGEVIGAAGPTTSYRMDDLTIPLLERGLRLMIGKGKRSDEVIEGMQKYGAVYLAAIGGAGAYISNSIKSCEIIAYEDLGAEAIRKIVVEDLKLTVAIDSYGNNIYEQGRAIYECK*

>CD630_10150 Clostridioides_difficile_630_NC_009089 two-component sensor histidine kinase

MKEQLIFNITNLVTVVFEAFVIHMFLSDFLGQKEGYIKVVRYAKLGFIICLGFCNLITLNPKITMPLIFILIFSTSFLYKGNLKTRLFTTVLLSIFFILSEIVVTSIFVLFVKEGFEIMLENNSIRVLATILSKIVFLLTCKIICLFKKDVHLDMPIKYWLPLFLIPIFSLFLSVSIFDVSKFFSLESLKFLSLISSVGILYINFIVFYLFKFIIDKTKLSMKYELLEKEIIHKEELRLSNECYKIIVEQTDSVVFEWNIKENKSFVSQAWTEKFGYNNACKNIFKEIKDKDLVHSEDKAIFEGFLESIKKKNMHNQAVYRLKKSNGEYIWCRTSITSIYNDENELLRVVGVIVDVDSDIKKYEELRTRAESDSLTNIYNKGTFEKLVEETIVMNTGDKKDALFIIDLDDFKEINDNFGHPFGDFVLKTFADKIQTSFGSKDLVGRIGGDEFVVYMQDYVTEVNLHKKAKELNRVLSDNYTDLSFSFDASVSIGIARYPQDGTSFFELFKNADRALYSIKASGKNSYCLFEEELYVQ*

>CD630_10170 Clostridioides_difficile_630_NC_009089 multidrug family ABC transporter ATP-binding protein/permease

MKKLIHFLKPYRVLIVVVLIFTFLQTLGTLYIPTLTANIVNNGVVKGDIDYIVKTGLMMMIVAGITALSAVLVCKVSANLSSGFCRDIREAVFIKSQDLSINDFNNIGTASMITRSTSDITLIGQSVFMFIQLVLPAPIITVSGLFLAYSIDKAMTIIIVVVMFLFMLSAFLVGKKLIKLFKMMQIKMDNMNRVLREVVTGVRVIRAFNRSHFEKKRFDRTAIDYSETAISINKIFAVLMPIVMLIMNLGIVSIIWFGGMRVSNGNMEIGHIMALVEYCILILFYLIMGVMVFMYIPRAGACADRVNQILDIEPEIVDGNGHKDTVSERGHLVFKNVTFSYAQSEEPVLNNITFEAKSGEVTAIIGSTGSGKSTIANIIPRFFEIQSGEISINGQDIKKIPQKELRDKIGFVPQKAFLFSGTIEENIRYGKEDASIEEVKHAASIAQADEFISDMEDKYDSFVAQGGNNLSGGQKQRISIARALVRKPEVYVFDDSFSALDFKTDKRLRKALKNEIKDSSAIIIAQRISTIMDANQIIVLNDGKIVGIGKHKDLLENCEVYKQIADSQLSKEELA*

>CD630_10230 Clostridioides_difficile_630_NC_009089 transcriptional regulator

MDIGEKIKRLRTEKQLTQEELANRCELSKGFISQLENNLTSPSIATLIDILEILGTNLREFFNEIDDERISFTKEDMFETEDEDLKYKLKWLIPNSQKNEMEPIIITLYPGGQYKEEKPHEGEEFGYVLAGSIYVHIGEKKNKVKKGESFYFRPKANHYISNEGKTTAKVIWVSTPPSF*

>CD630_10300 Clostridioides_difficile_630_NC_009089 family 2 glycosyl transferase

MRDLNVSEYLFVFSLFSIWSLLLINIILAMGGYIFYFKNFDKEIKEIDEYPMISILVPAHNEAKVIGRTVESLLLLNYPKSKMELIVINDNSSDNSKEILENIKDRYNNYNFTIINTDSLTGGKGKSNALNIGYTISKGDFIAVYDADNTPDKNALRYLVQTIVMNDELGAVIGKFRTRNKNKNLLTKFINIETLSFQWMSQAGRWQLFNLCTIPGTNFILRRSIIEEIGGWDSKAIAEDTEISFRIYKLGYKIKLVPQSITWEQEPETVKVWIKQRTRWAKGNIYVLMKYIKNIFKQGRNKIVFDIAYFFSVYFLFLTSVIISDILFVLSISKLVEISIPINFFLIWILSYLLFIIEVSISLTIEKGEATIENIFIVAIMYFTYSQLWLFVAIKGMIEYLKDIIFKREVKWYKTERF*

>CD630_10330 Clostridioides_difficile_630_NC_009089 UDP-N-acetylglucosamine 2-epimerase

MNDIKVMTVFGTRPEAIKVAPLIKELEKRENIKSIVCVTAQHREMLDQVIETFNINVDYDLDIMEKGQSLNDITCKILNKLPLILNKENPNIILVHGDTTTTLATSLTAFYNKTLVGHIEAGLRTYDKYSPFPEELNRQLTGIIADMHFAPTNLARKNLISEGKPNNNIFVTGNTAIDALKMTIKENYNHPIIDEIGNDRMILLTSHRRENLGKPMKNIFRAIKRIVDDFEDVQIVYPIHLNPKIRTIADEIFGKFPEKIHIIEPLDVADFHNFLNKSYMIMTDSGGIQEEAPSLGKPVLVLRDKTERTEGIEAKTLKLVGTNEDRIYNSVSDLLINKDNYVQMSKASNPYGDGNASKYIVDIIIKKFNCKYLN*

>CD630_10410 Clostridioides_difficile_630_NC_009089 ATP-dependent helicase/nuclease subunit A

VSSPKWTKEQLEVIESRECNLLVAAAAGSGKTAVLVERIIQMITSRENPIDIDKLLVVTFTNAAASEMRERIGDAIGKALDENPENKHLQNQLVLLNKSSITTIHSFCLDVIKSNFHRINLDPNFRIGDQTECAILKQEAIEEVFEDLYEERDEGFLNLVESYAERGGDKEVQDIILGIYSFAMASPEPKKWLIDSAERFNIDENFDFSQSIWARAILDTVKIEINGLCLNMERALKEVESIEELETFAEKLSVEYKKIADISQACNKSWDEAYKKMASMSFENYVKGVKRISKDAPSYIKESKEKAKTIRDKTKKSLESIVSATFNKDNDSIREEIKYLYNIVKPISSVVLRFEEEYSNKKREKGIIDFNDIEHFALNILTDVDEKGNIVPSDIAVGYRNKFYEIFIDEYQDSNLVQEVLLKAVANTETPNRFMVGDVKQSIYRFRQAKPELFLQKYNNYNDKKGSSHRKIMLYKNFRSREEVVDAVNYIFENIMNENIGEIEYTEKERLNLGANFNVDTDEKSIIGGATEIHLIQKDNKLDDDIINDKDDRINNKENEIEEEEKLDNIQLEARMVGNIIKDLMKVNEDGKIQKVYDKGIDGYRPVEFRDIVILLRATSAWAPVFADELMNMDIPTYADVGVGYFDTIEIKTILSLLQIIDNPMQDIPLISVLKSPIFGFTPEDLIDIRVQSKDKIFYEVLKSTAEYDGFTDSQNENESEFIPSEECINKSKDFLIKLKEFKEKSMYMSTDEFIWYLYTRTGYYAYVGALPGGSQRQANLKVLFERAKQFEETSLKGIFNFVNFIEKLKKSSSDMGSAKTLGENANVVRIMSIHKSKGLEFPVVICSAMGKNFNTQDFKKSILYHHNLGYGPQFVDYERRISFPSIAKEALKSKINIENLSEEMRVLYVAFTRAKEKLIITGSTRNIQDSIKRWSNGIESLDTISQYEILKGKNFLDWIMPCVLRHRDLSNLLEEVGLDAVFNVEHNSKWYGKLWNKNDILVEKKSDEEKESIEEILEKIDVNNPDSDYYGEIEEKLNYIYPYEFSTRKPATISVTEIKKIQNNYEEELINTIFEQKVILKKPLFIQNEEEREKISGTERGTIVHLVMEVLDLKNVSSVNDIKSQIRGFVSKGIITEKQASIVNPYKIYKFFASNIGKRMLNAEIINREKSIYAQVNMKDIYIYEKLINNDDKKLYDNESVMLRGIVDAYFEEDNQIVLVDYKTDFVNEENINQIIEKYKKQLDLYADIIETLTGKSVKEKCIYLFGVDEAVCY*

>CD630_10480 Clostridioides_difficile_630_NC_009089 phosphodiesterase

MKIGIMSDTHGSLLYFEKALNVLSDCDVLLHGGDVLYHGPRNDIPEGYNPKKFIETLNKLENIVIVKGNCDADVDQMVIEHPIQSPYVMSQFGEIRIILNHGYIESEEEIIDKAKKMGGDILVLGHTHVKKLYMDDNLIVINPGSTSIPKDGSHSVAIIDIIKTDDEDELELDINLIDINTGNIININD*

>PCZ31_RS05070 Peptoclostridium_difficile_strain_Z31_NZ_CP013196 ABC transporter ATP-binding protein

VGIKKCIRGEDVFSIRNITKKLGKFKLNNINLELKEGDIVGIIGPNGSGKTTLIKIIMGIIDADEGEIELCNETIENSPISFKNNIGFVYDSLQFYPHLKVKEFRKIVSLFYKNFDRERFDEYLNKFDIEENMHIENLSKGQSEKLMLSSALSHNAKLLILDEPTAGIDPIVRTEIMQYLQDFVKNGSSSVIISTHNTDNLIKIADYLVFINRGNQIFTVKKELIEQEYKIIRANKAELEAIKESIVGVKEYKYYNEALVKVGDSLKVKSLLIEIDKHKVKNPTIEELMYYYVNEVR*

>PCZ31_RS05085 Peptoclostridium_difficile_strain_Z31_NZ_CP013196 EBSC protein

MSISNVREYFKQFGKEDSILEFEQSSATVELAAEAAGVIPARIAKTLSFKIGDDAILIVTAGDAKIDNKKYKAEFNCKAKMLTPEEVLEFTGHAIGGVCPFGLKNSIKVYLDDSMKRFDTVFPACGSSNSAIELTCEEMEKFSKCEKWVDVCKIGKKI*

>CD630_10540 Clostridioides_difficile_630_NC_009089 butyryl-CoA dehydrogenase

MDLNSKKYQMLKELYVSFAENEVKPLATELDEEERFPYETVEKMAKAGMMGIPYPKEYGGEGGDTVGYIMAVEELSRVCGTTGVILSAHTSLGSWPIYQYGNEEQKQKFLRPLASGEKLGAFGLTEPNAGTDASGQQTTAVLDGDEYILNGSKIFITNAIAGDIYVVMAMTDKSKGNKGISAFIVEKGTPGFSFGVKEKKMGIRGSATSELIFEDCRIPKENLLGKEGQGFKIAMSTLDGGRIGIAAQALGLAQGALDETVKYVKERVQFGRPLSKFQNTQFQLADMEVKVQAARHLVYQAAINKDLGKPYGVEAAMAKLFAAETAMEVTTKAVQLHGGYGYTRDYPVERMMRDAKITEIYEGTSEVQRMVISGKLLK*

>CD630_10570 Clostridioides_difficile_630_NC_009089 3-hydroxybutyryl-CoA dehydratase

MSTSDVKVYENVAVEVDGNICTVKMNRPKALNAINSKTLEELYEVFVDINNDETIDVVILTGEGKAFVAGADIAYMKDLDAVAAKDFSILGAKAFGEIENSKKVVIAAVNGFALGGGCELAMACDIRIASAKAKFGQPEVTLGITPGYGGTQRLTRLVGMAKAKELIFTGQVIKADEAEKIGLINRVVEPDILIEEVEKLAKIIAKNAQLAVRYSKEAIQLGAQTDINTGIDIESNLFGLCFSTKDQKEGMSAFVEKREANFIKG*

>CD630_10600 Clostridioides_difficile_630_NC_009089 pseudouridylate synthase

LFKKENQRYNLISYTNEEEMTLKEVLLDKLNFSVRSLSKMKREKSVLVNGVYKKPSLKVYSGDLIEVKIYEEKANFEPQDLNLQIIYDDFDIIMVNKPPFMVVHPTKSHYDKTIANGISYYIDNQKENVKIRFVNRLDMNTSGLVIVAKNAYAHHTLSTAMSENKVEKKYITVVDGIIKENEGTIDEPIYRPTEDSIKRIIDERGQSSVTHYKVIERLENATVLEVSLETGRTHQIRVHMAHIGHGIIGDELYGYVDEELINRQALHAYKLEFEQPRTKEKLKFKADIPEDMKELISKLR*

>CDM120_RS05720 Clostridioides_difficile_M120_NC_017174 hypothetical protein

MQDYKKNKRRMTNQPMPTMNEEEVYTEEINSEDMRGFKKSHHHNECNTDNKCDCHDDCKPCNPCKPNPCNPCKPNPCNPCKPNPCDDNCGCHDHCKCDCEPCEMDSDECFENKCGPECCNPISPRNFSVSNAVPFAIEANRIFDTMQFQTFTDATGPNGEPLTFETEVVEVFGSVPSAGKASVTIEKICLSNDGIVIDTGMTTLEDFDLDPLGDIVGRNCETTFEFAVCGERNAECCRQGKGKSVAYKQRGLTVAVRNLVLELRGRCGCTEFVALAFPAVRAGGGCKRRVDYVEFTFNTLSAPICLPADGRAVTLRQEYQTNLTVDCIGKSILKLECNECCEPFYELIIPNDIDLVLCLQNTVSTLISEQIVVLASPNPIQPRLVDTFSKVCDFSQCGPNHESGKPSCHR*

>CD630_10790 Clostridioides_difficile_630_NC_009089 LysR family transcriptional regulator

MDFKQLEVFVAVAKHQSFSKAARELFLTQPTVSAHIQNLERELETVLINRSNKVITLTKSGEILYEHAIYILNNCKRAIYDIKEYSGKIEGIIDIACSSIPETYILPDFMKSFSMSYPDVKFSISHYDSQYAISEILNERISFGLVGSKINNPQIEYLDLLDDELVLITPSDFKIDNKNNCIDIGELAYLNFIMRKEGSGTRNLILNTLSKNNFPVSKLNVIAHVESNEAIKEMVRLGLGVSFISYISAIDYLNAGKIKCYKIKDVDFTRKFFFIYSKKKTFSPLEDKFLNRLCEYFEIII*

>CD630_10840 Clostridioides_difficile_630_NC_009089 multidrug family ABC transporter ATP-binding protein/permease

MIKQFVKYYKPYKKIFTLDLIAAFLFSLCDLVYPMITRNIMDDVVPNKNLRMLVVFAVALILIFIAKAGLNYFMQYWGHVIGVDMQADMRNEVFTHLQRLPNTYFDNNKSGVTMSRIVNDLMDITELAHHGPEDLFISIVMLVGSFFILIDINIPLTLIIFAILPFIIWFAIAKKDKMNIAFMKSRVTIGDVNATLENSIAGMKVTKSFCTEKEELNKFVRSNKLFRRARQDSYKVMAEYYSGMNLYMDILEWVVVIAGGYFTYIGKITLGDFAAYILYVKMFIQPMKKLINFTEQYQNGMTGFKRFIEIMEQDHQKEAKNPIELENVKGDIEIENISFTYEDKTQVLDNLSLSIKAGKTIALVGPSGGGKTTLCNLLPRFYEFDKGDIKIDGKSIKDVSLKSLRKNIGIVQQDVFLFTGTIRDNILCGNPNATDEEMIAAAKKARIHDFVETLPDGYDTYIGERGVKLSGGQKQRISISRIFLKNPPIIILDEATSALDNVTEREIQESLEELSKDRTNLVVAHRLTTIKNADEIIVLTDKGIEERGTHEELVNKNGVYSRLHNN*

>CD630_10870 Clostridioides_difficile_630_NC_009089 zinc transporter ZupT

LNNVIFAFLITLLAGLSTGIGSCIAFFAKKTNTKFLSISLGFSAGVMIYVSMIEIFPKAQDALTKSMGEKLGSWSTVIAFFIGMAIIAAIDKLIPQEENPHEIKKMENIEEKNIKKNKSLLRTGIFTAMAIAIHNFPEGLATFISALDDVTIAIPIAIAIAIHNIPEGISVSVPVYYATGDKKKAFYYSFLSGMSEPLGAIIGYVLLRNFLNDITLGIVFAIVGGIMVFISLDELLPSAREYGEHHLSIYGLIAGMGVMAISLLLFK*

>CD630_11240 Clostridioides_difficile_630_NC_009089 hypothetical protein

MGDTSRFYESVKNKYPKLKDGTRVHLWPKENIIIGPIYDEEDGYKKSIGNVDSLSWKQIVILAECDGKNTTDDIINILSNRYRNIHNMKEKVMEFFMFYENVYLTFEDDIQTLSSVFEITGNKSYITPLYFTIEIDGDNNTNDYFSEITSLLNCMYEKGCRFIEVIGEDILKNKSMREIFQYMLDHFDLIVVTKDSFTIDRSLIKELDNYRHKVIWKVYSKNDDTRIKLKEDIKITSLIKRGHTVVRGDREKAIKIEDMRFKDTKERFSKYGAEWSHLYISSDGSVKSYSFQKDKRIYLGNIFNNSVEEIFLEMQNKIDKLEIVYQ*

>CD630_11260 Clostridioides_difficile_630_NC_009089 AraC family transcriptional regulator

MEWINKLNQAITYIEENLESGVDYAEAAKIACCSTFHFQRMFSYIAEVPLSEYIRRRRMTKAAFELQNSNIKILELSEKYGYDSPTSFNRAFQNIHNISPSAARAKGVVLKAYPKMTLSIYVKGNIEMQYRIVEKKGFRIVGIKESMNMIVEECFEKVPKLWAKCIKNGTIDKLSELINNEPCGLLGVSVCTNSKGLDYYIAAPTDKPVLEDTHEYLIPSGTWAVFECVSPMPNALAIQELQKRIITEWLPSSGYVYANLPDIELYPNGDINAPDYTTEVWIPIQKPS*

>CD630_11270 Clostridioides_difficile_630_NC_009089 membrane protein

MKKNMLYVGIIYFICGILLVLLATFTEFSFEAFIWGLSGAALGPGVCMILQYMYWSKPERAVDYEEKIKNQRIEMNDERRIMLRDKAGRITNQIMSYVLVILIFIVSILSVFSVMVISKWVLVVLGLLILFQFICSVVVYNKLDKKL*

>CD630_11290 Clostridioides_difficile_630_NC_009089 dephospho-CoA kinase

MLILGLTGGIGCGKSSLSNIFRNLNIPIVDADIISRKIFEDKLLLEKVFVHFGQSIKNDDGTLNRKALGKIVFSDEEKLKELNNLTHPRIREKIISEIEKLRKKGENIVVLDAAILVESGFLDMVDKLLVVTCKQEVQISRIQKRDNCSEQEALSRINSQMSQEEKSKYGDYIIDNSGTITELESKAHKFIEYMKENWRE*

>CD630_11340 Clostridioides_difficile_630_NC_009089 lactoylglutathione lyase

MKFNFCHNNFNVTNLEKSLDFYKKALGLKEVKRKEAEDGSFILVYLGDGITSHTLELTWLRDWDRPYNLGDNEFHLALEVEEFDEAKKLHKDLDCICFENESMGIYFIADPDNYWIEILPKNH*

>CD630_11350 Clostridioides_difficile_630_NC_009089 SH3-domain-containing protein

LLGGVIVVKKAIAALGIGAVAVSVSSINASALEKGTVTASALNIRSGPSSDCDKVAKLYKGKTVEILEKSNGWYKVRVSSSVVGWGSAKYISTSGSSEGTSSQNNSTSSGTTISGNGKVNVSSRLNVRSGAGTNYSLVGKANNGDVVKLLEQSNGWYKIKLSNGVTGWASSQYISKTSEDVGTNNSSNSNSTNNSDKKPSSEESIEGKNGKVTSAVSLNVRSGPGTSYSIIGKLNGGDVVELKAKSNGWYKVKLSSGTIGWVSASYISETNEDTKEKPNSSSNQNSQSNSNSKPSFTGNSDKSTAKGSTIVDFAYTLIGIPYQWGASGPDKFDCSGFTQYVFKHSVGVSIPRVSREQANFGSAISMGNYAPGDLVYFDTDGDGTTNHVGIYVGNSKFIHCSGTQTNPNKVKVDNLTSSYWSKVLLGARRFV*

>CD630_11420 Clostridioides_difficile_630_NC_009089 electron transport complex protein RnfB

VKMVILTAVLVLGIMGLIFGIVLDFASKKFAVEVDERVEAILGVLPGANCGGCGFPGCGGLANAIVEGNAPVNGCPVGGADVGAKVGEIMGISAEAGEKQVAKVICKGTCSSAKDKYEYEGISDCRAANVLNSGAKMCKFGCLGLGTCKDACKFDAISIVDGIAVIDEEKCVNCGKCKEVCPKGIIITKPESQEVVVECNSKEFGKAVKEKCTAGCIGCGMCVKACKFDAIIFEDKIAKIDPNKCVGCMQCVAKCPTKVISGDITKKKKVTIDQELCVGCTVCKKQCKFDAIEGELKEKHKVDADKCVGCHLCMEKCPKKAIKIL*

>CD630_11450 Clostridioides_difficile_630_NC_009089 cell shape-determining protein MreB

MAKEKKKEKKGFFSFNKMTKDMGIDLGTANTLVYIKGQGIVVREPSVVAIRDDSKEVLAVGEEAKKMIGRTPGNIVAIRPMKDGVIADFDITQSMISYFIQKAADKKGVVSPRIAICVPFGVTEVEKRAIEEAARQAGAKDAFLIEEPMAAAIGAGLKVEEPEGNMVVDIGGGTSEIAVISLGGIVTAKSIRIGGDEFDESIVAYVKKEYNLMIGERTAENVKINIGSTFKDDEEINMQIRGRDLISGLPKTIEICSTEVREALKEPVSSIVDAIKSTLERTPPELASDIMENGIMLTGGGALLRGLDKLITQETGMSVQIAETPLDCVALGTGKSVEDQEIFEKVLMMNTKN*

>CD630_11500 Clostridioides_difficile_630_NC_009089 septum site-determining protein MinD

MSEVIVITSGKGGVGKTTTAANLGTALSLENKKTVVVDADIGLRNLDVVMGLENRIVYDIVDVVEGTCRLKQALIKDKRFDNLYLLPAAQTRDKNAVSVEQMIDLCEKLKESFEYIIIDCPAGIEQGFKNAVAGADRAIVVTNPEISAVRDADRIIGLLEANEIKEIRLVINRIRNDMVKRGDMMDKQDIIEILAIDLLGLVPDDESIIISTNKGEPAILDSKSLAGQAYKNIAKRILNEEVPLLDLEVEDGFFGRLKKMFSMAK*

>CD630_11640 Clostridioides_difficile_630_NC_009089 GTPase Obg

LFIDKARIFVKAGNGGNGSVAFRREKYVPAGGPDGGDGGRGASIIFEVDLGLRTLMDFKYQKKYQAQNGGDGSKGKRAGKNGENLVLKVPAGTVIRDEATGLVLADLKKEGDTAIVAKGGIGGKGNQHFANAVRQAPAFAKSGTDGEERWITLELKMIADVGLLGFPNVGKSTFLSVVTKAKPKIANYHFTTLTPNLGVVQTKFGDSFVLADIPGIIEGASEGIGLGHEFLRHVERTKVLIHIVDISGLEGRDPIEDFDKINDELKLYNEKLSKRPQVVVANKFDILEDESKFEKFKSELEGRGYTVFKMSAATRQGIDEVIAYVSKMLKEVEDVELVSEEEMYRPELDIGTEEELSIDIEDGVYVVTGKALRRIMYSVNFDDMESLQYFQKAMESQGVFDRLREMGIEDGDVVKIYELEFEFYN*

>AEC_RS02000000220890 Clostridioides_difficile_QCD_37x79_NZ_CM000658 FAD-binding oxidoreductase

MYKLIDKKDIDFLIDTCGEENVLVGSDINEDFSHDELGGIEKYPEVLVNVLETEQVSKIMKYAYKNNIPVTPRGQGTGLVGAAVAINGGIMINLCKMNKILEVDYENLTLTVEPGVLLMTIGQYVQDRDLFYPPDPGEKSATIAGNINTNAGGMRAVKYGVTRDYVRGLEVVLPNGEIINVGGKVVKNSSGYSIKDLLVGSEGTLGIVTKAILKLLPLPKKSISLLIPFPDLSMAIETVPKIIKSKSIPTAIEFMERDVILAAEEFLGKKFPDNTSDAYLLLTFDGNSTEDIEKEYEKVANLCLENGALDVFISDTQERNDSIWSARGAFLEAIKASTTQMDECDVVVPRDKIAEFIRYTHELQDKLKIRIKSFGHAGDGNLHIYILKDGMDDNTWKIRLKETFDYMYKKSRELSGQVSGEHGIGYAKKEYLHESNSDAYMMLIKNIKLAFDPKNILIQGKYIRNINLIIFTKQKILN*

>CD630_11750 Clostridioides_difficile_630_NC_009089 acetate kinase

MKILVLNCGSSSLKYQLIDMNNEEVLCIGLVERIGIEGSILKHEKAGRDDKYVVEQPMKDHKDAIALVLEAVAHPEFGAVKEMKEIDAVGHRVVHAGEKFATSVVITPEVEEALKECIDLAPLHNPANIMGIDACKAILPDVPMVGVFDTAFHQTMPKSSYLYGLPHELYTKYGVRRYGFHGTSHNYVSQRAAEILGKDIKDLKIVTCHLGNGASIAAVDGGKCVDTSMGFTPLEGLIMGTRCGDIDPAILPFLMRKEGLDADGLDKLMNKESGVYGMTGISSDFRDIEDAAKNGDERAQATLEAYVKKVQKYIGAYAAEMNGLDVVVFTAGVGENGKAIRADIASNMEFLGMKLDKEANDVRGKETVISTADSKVKMLLIPTNEELMIARDTLRLVK*

>CD630_11761 Clostridioides_difficile_630_NC_009089 50S ribosomal protein L32

MAVPKRKTSKSNTKMRRAANSKMEATGFVSCPQCHEPKLPHRVCPDCGYYKGKEVVSK*

>CD630_11770 Clostridioides_difficile_630_NC_009089 fatty acid biosynthesis transcriptional regulator

MKKKSKAQRQKELIDMLKTDPFYTDEELSSLFDVSIQTIRLDRMSLNIPELRERVKSIAETQSSKVKTLGVKEITGEIIDLSVGRLGISMLEVTQDMIYSKTNTLKDTYIFSLADSLAMAIIDAPKVIMRVANVKSFKLIEQQDRLIAKAEVYRNIDKKHYVKVVINNKAQEQIFRGKFIFEELD*

>CD630_11800 Clostridioides_difficile_630_NC_009089 enoyl-(acyl-carrier-protein) reductase II

MNKICKILNIKYPVIQGGMAWVATASLASAVSNAGGLGIIAAGNAPKEAIKKEIVECKKLTDKPFGVNVMLMSPFVDDIIDLIIEEKVQVITTGAGNPAKYMDRLKEAGTKVIPVVPTIALAQRMEKLGATAVIAEGTEGGGHIGELTTMVLVPQVADAVNIPVIAAGGIVDGRGIAASFALGASAVQVGTRFICSEECSVHSNYKNLVLKAKDRDAIVTGRSTGHPVRTLKNKLSKEFLKMEQNGATPEELDKKGTGALRFATVDGDIEKGSFMAGQSAAMVKEITPCKEIIEAMVNQAREIMPAIEL*

>CD630_11820 Clostridioides_difficile_630_NC_009089 3-oxoacyl-ACP reductase

MINLTGQVAVVTGGSRGIGKEIAKKLASFGADVVINYTSKEDEALKTKNEIESMGVKCTSIKCDVSKFDEVNQMIDSVVSEFGKIDILVNNAGITKDGLLMRMKEEDFDRVIDINLKGVFNCTKAVTKPMMKKKYGRIINMTSVVGIMGNAGQTNYCASKAGVIGFTKASARELASRNININAVAPGFIETDMTKVLSDDVKESTLANIPKKSYGKPEDVANAVAFLVSDMSSYITGQVINVDGGMVMQ*

>CD630_11840 Clostridioides_difficile_630_NC_009089 3-oxoacyl-ACP synthase

MNKRVVITGLGCVTPLGTGKEEFWSNIKSGVSGIDKITNFDASTYQTQIAGEVKNFHPEEYISKKELKRLDKFAQFAIVSAKLAVEDANLDLDKVDRERFGVIIGSGIGGVEAIETQHKILLEKGNKRVSSLFVPMMIGNMAAGQVSIFLGAKGPNTNVCTACASGTHSIGDAFKVIQRGDADIMVAGGSEAAVTGLAFAGFCNMKAMSTRNDDPKTASRPFDKDRDGFVMGEGAGIVILEDLEHALARGAKIYAEVVGYGLTADAYHMTTPAENGEGAARSMNMALKDGNVPLEEVDYINAHGTSTYYNDLYETMAIKTVFGEKAYDLCVSSTKSMTGHLLGASGAIEAVVCAMSIEDSFVPPTINIQEVGEDLDLDYVPNQGKEKNIRYALSNSLGFGGHNATIVLKKYV*

>CD630_11900 Clostridioides_difficile_630_NC_009089 acyl-CoA N-acyltransferase

MPTITLKNGVDVLIREGVREDAQSIIDFYNEVGGETHFLSFGKDEYKISLEEQENAIESAKASDNSVKLIAFIDGEIVGIATIDSNQKAKGKHVGVLGIVVKEKYWGIGLGKRLMLDLIEWCKSNGITKKITFVTNEENYNAIGLYKKVGFEVESILKKECYYNGVYTDLIGMSLLLGI*

>CD630_12050 Clostridioides_difficile_630_NC_009089 geranyltranstransferase

LEFKQCLKEKASFVEKVLKEYMPKEEGYQKTVIEAMNYSLSAGGKRLRPILTLEACKIVGGNEDEAIPFAIAIEMIHTYSLIHDDLPALDNDDLRRGRPTNHKVYGEAMGILAGDALLNYAFEVMLAGSINKENPEKYLKAINEIAKGAGIYGMIGGQVVDVESENKQIEKEKLDYIHMNKTAAMMVGCMRAGATIGGANSEQMEEITKYAKNIGLSFQIVDDILDIVGDEAKLGKKVGSDIENHKSTYPSLLGLDKSKEIAHNLIDEAKKSIEKLSDDVDFLKGLAEYIIDREY*

>CD630_12090 Clostridioides_difficile_630_NC_009089 DNA repair protein RecN

LILELYMKNCALVEELRLNIDKNLNILTGETGSGKSIIIDALGLCLGDKYDRSFLRKGTDKGLVEAVFFSDNRYLKKILEENDISMEDDNLLVITRLIYSDGKSTARVNGRTVKVSLLKEIASTLIDIHGQHQNQALFNKDTHLKFLDLFGENELEEFKIAYKKVYHKYSEVKKALNCLTENKDEMQIQREIDLLRFQINEIEAANLNKNEYEDLLKQREVYRNSEKIYNNLNSSYSKLHNGEYNVIDLIGLASKELNDISKYDSVLSEYSDTVERIMYELQDISGEIRNYKDNIDFEPYELEQIELRIDEINNLRRKYGDSIDDIFEYYGKIKDRLDEILNRDERVEQLRSKLMNIEEDLKIKASKLTKARIEVATTLEKILLDELKSLNMKNVMFKVNFEKGPFTLDGVDDIEFMISFNLGEDIKPIYKVASGGEMSRFMLAFKTILADIDDIDTLVFDEIDTGISGIAAQIVGEKLSDIAKKKQIICITHLPQIAANADTHYCIEKDTSNNRTFTNVSKLNESQRKNEIARLIAGNNITEKTIEHASEIIELAKKC*

>CD630_12120 Clostridioides_difficile_630_NC_009089 diamine N-acetyltransferase

LKLNVRIADINNWHDLVSLSVDKSQLDYIESNALSIAESKFITAWVPVGIYDENSLIGFAMYGRLEDDRIWLDRFMIDSKYQGKGYGKASLDFLVNHLKNEYNCDELYLSIFEDNKMAIKLYKDFGFEFNGELDYGGEKVMVLKSN*

>CD630_12200 Clostridioides_difficile_630_NC_009089 NUDIX family hydrolase

MVLEEKTISSDRVYTGKVITLKVDTVEIPGQGYQKRELVEVGGAVGIVAITDDNKVVLVKQFRKPIEKPIFEIPAGKLEKNESPKECAERELKEETGYSAKNIKLIHKFFTSAGFSNEIMFVYLATGLTPGENNLDADEFLDVYEIELEEAYNMVLKNDVEDAKTSIGLLLVKDMFKN*

>CD630_12260 Clostridioides_difficile_630_NC_009089 aminodeoxychorismate lyase

MNFKENRLKIAVLIIVILIILAGIFVFIQIGPYDKNNKKDVIIDVPSGASVGKISDILYENKLIKNELLFKLLVKVSNKAPSIKSGTYLLNQSYSNNDIISLLVSGKIYQDGIKVTIPEGATSKEIIAMLVSKNLGDKATFENLIKKPQEFYDKFPYLKEDGITSLEGFLYPETYYFNSKKQSEEDILSEMLKVFDSKYTDKFKKKQKELNMTLQEVMEMASIIEKEAVLDKDRPIIASVFYNRLKVGMPLQSDATIQYIFEERKKIVTYDDLKIDSPYNSYKNKGLPPTPISNPGIKSIEAALYPEKTDYLYFVAKIDGGNNYSTNYQDHLKYVKEYKEARDKQSKDTKATNKENTKK*

>CD630_12480 Clostridioides_difficile_630_NC_009089 ribonuclease III

MKISKKLLDNIQRFENVINYKFKNKEYILEALTHSSYSNENKKYNFNERLEFLGDSVLGIVISDYLFNEEANLPEGELTKLRANIVCEDSLSEVANDINLGIHMLLGRGEEATGGRHRTSILADAFEAVIAAIYLDGGFESARQFILHHMENIIYDSRKGNIFRDYKTHLQEVLQGNGENNIWYRLIEEKGPDHNKRFVMEVGINDDVLGIGEGKSKKEAEQLAAKIALKKKLWEK*

>CD630_12500 Clostridioides_difficile_630_NC_009089 chromosome partition protein

LYLKRLELKGFKSFPVKTDIIFKEGITAIVGPNGSGKSNISDAVRWVLGEQSIKSLRGDKLEDVIFAGTDTKKPMNYCEVALTIDNSENQLELDFTEVTIRRRAYRNGESEFFLNNKSCRLKDIKEVFLDTGIGKDGYSIIEQGKVDEILSNNPLSRRKVFDEACGISKYRYKKQEAERNLSNTKENLERIDDVYIEIENQLKPLFNQQTKAKKYLEISEKLKTLEVNSFIREIEGIEKELSEVNEHRKVIEKELNEKEEQKNVVEKKQEDINKEVEVLQDVIEKSVDYINSIKGVISKKESQINLIKERIRNFTNEISRKNLEIKDIKEKLNENKQYIKELESNKLSGSEELSTLQENIKVLEGSKDKQKIKLESLNNEIELLKESIIDILNKKQEFSNKLSTLNANKENMNIRDENINSEITELNKNIEIKSSELDTINKEFNMQNENLKNVNNRHKELSINLQDSISEHNKLEDEIQKSKYNLNGYNSKLNVYIDMENHYEGFNRGVKEVLKNKNLKGVHGALGQIINVPEKYEKSIEAALGAYMQNIITDNEFSAKSAINYLKQNNLGRVTFLPLNIIKSNKISLGNLKANTKFIGIASDLITFDEKYRNIIENILGRTILINNIDEGIKFAKETGHRFKIVTLDGEILNPGGSLTGGSLKTNGNILSRKRYINEYTEKISNIKNEISHLELKRESLDKDVKNIKNEIDSHESKIKDLEKSIIIKSTSIKNVESEIESLKGSITKLENEKNDLNSNLNYTLEKSDDVRKDMEELDDLYNKNKEKIDALNEEIKRYNDLYDKEKSEFDELNLSLVKKTEVYNSIVRDIKRISGENCELEEKNKQLEESLNYEEHEIIKLQDSILTEEKEKENLTKQLGDSNRNLETRKIAKDDLKNSFDEINKELKTIDRQHIELKESLFKVGGRLERLKTSQDTYINKLFEQYDMTLVQALEIKDEDLDIDRKFLESLKREIRSLGNINIDSIKEYEEIKERYDFYSEQKQDLEESMEEIEKLIHTLEENMKSEFEIKFEEISKNFKYVYKRLFGGGCGELTILDKENLLESDILITAQPPGKKMKNLNLLSGGEKALTAISILFAILITKPTPFCILDEIEAPLDDANIFRFGEFLKDLSKETQFISVTHRRGTMEAADYIYGVTMQEKAISKVISLKLKEAQEITDII*

>CDIF1296T_01324 Clostridioides_difficile_ATCC_9689__DSM_1296_strain_DSM1296_CP011968 GTPase

VSNEYEEYLMNDNLHINWYPGHMKKTKELVKNNLKLIDVVIELLDARIPFSSKNPDIDRLVGDKPRVVVLNKSDMADRDKLNQWIEYYKKINIKAIPVDTIKGVGINKIIEECKNVTREKMSSLKDKGRKERAIRIMIVGVPNVGKSSLINKLTGRKSTQTGDKPGVTKGKQWVRLKGNLELLDTPGILWPKFEDQEVALNLAFSRAIKDEILDTETLALRLIEKLMKIEPEKLKARYKLDCLGETPIETMDMIGHKRGFITGKKELDYTRIATTVLNEFRDGKIGNITLEVPENVKR*

>CD630_12620 Clostridioides_difficile_630_NC_009089 ribonuclease HII

MQDKSVREIKEIIETLEVEKYMEYIELLRVDERKSVQGLAIKLAKKLDNIRKEEERLETINIFENEGYDKGYLYIGGIDEAGRGPLAGPVVASVVVFKKDTKIEGVNDSKKLSEAKRDELFEVIKEEALDYGIGIVNNEEIDEFNILNATYMAMKKAINCLKKAPDYLLVDAATIPGIDISQNPIVKGDSKSISIAAASILAKVTRDSIMYQYDRVYPEYGFKSHKGYGTKEHYEAIEKYGITPIHRKSFLKNIL*

>CD630_12650 Clostridioides_difficile_630_NC_009089 CarD family transcriptional regulator

MYKIGESVMYPKEGACSVNDIVTKKINHEMQKYYELSVIFNSNLKISIPVLNADRIGIRPVMDGNDVDNFIQSINKTDGVWIFDRKERLKLYQDKFHSGDVFEIVKLIKMLMIQDSSKQLCSTDKEFLNKAQKFALSELAAAQCKSYTMVLEEMKKHILNSKNTN*

>CDM120_RS06635 Clostridioides_difficile_M120_NC_017174 ABC transporter permease

MKLYMSVKTMLKGLKSSFILNLIYFLALPLILSWFLGMVTESMFQNPIKTESTPIVIYDKDNTRLSNHLTKYLKNDLSYILTVKKDDSKAELKLTIPKGYESSLLNEKSNTLNIEKLGTRDDIAILLQDILDTYHEKFYLNNSQKISSEDFSKLFNKNSIDTSIIGNNVKQSSYEYFALVSLGFLVIIFIMNNILSNYISESKGLSKRLYSMPITRVQFLIYDFVGLWIYSFIFLLLYVLFFRIIGITFKGNFAILLLLCALSSYFMTSISTFVNSFFSKKYGTIIVYALLFLQTIFGGIFSMISDAFTKLTSLSPTYLIGELFSNYETFKTIDSIGNLIFTCLITSTILIALAIVKEKYKWREVQ*

>AEC_RS0207650 Clostridioides_difficile_QCD_37x79_NZ_CM000658 magnesium chelatase

MLSIINSSNLVGIDSFLVKVEVDVSNGIPSFNIVGLPGKEIKEARERVKSAILNSGYKFPSTRIVVNLSPADIKKEGAFLDLSISIGLLRELIKKDENYIRESMFIGELSLDGKIRKVRKVRGILPIIMGAKTQNIKRIFIPIENIKESLLVDEIDIIPIKSLKECVDFLNEEIKVDKVSIMSFLDDKSRKENGELEKDNSYIDCKYTKINNEESKYDEDFKDVKGNYFVKRSAEIAAAGNHNMFMIGPPGSGKTMIAKRVRTILPDISIEEMIEVSKVYSILGMINESKGIIDKRPFRAPHHTTTKQSLIGGGMDARPGEIALAHRGILFLDEIAEFDRKILETLRQPIEDGYVNISRVKYSAKYPCRVLLVAAMNPCPCGYYMSETECRCRSNEIDRYINKISGPLLDRFDIFVEVNSIKYSDFNSLKQEESSQKIKRRVENARKIQINRFKKDNIKNNSEIKAYNLFKYCKLEKEASKTAEMIFNKYNLSSRSYTKLLKMARTIADLEERDLINSQCIIEAFSFRKAYYSYFK*

>CD630_12810 Clostridioides_difficile_630_NC_009089 tRNA-specific 2-thiouridylase MnmA

LFMNKKVMIGMSGGVDSSVAAYLLKQQGYDVIGVTMKLWQDDDVVEIEGGCCSLSAVEDARRVANKIGIPFYVLNFREVFKEKVIDYFIDEYLEGKTPNPCIACNKHIKFDDFYKKARQIGCDYVATGHYAKIEKDESTGRYLLKKSVTDKKDQTYALYNLTQEQLEHTLLPIGDYEKDRVREIAKEMGMAVHNKPDSQEICFVKDNDYANYVKKHSKKRIEEGFFVDTKGNILGKHKGILYYTIGQRKGLGITFGKPMFVIDINPINNTIVLGDNEDLFKKELIAKDVNFISIDTLEEPLRVQAKIRYSAKPSPATIHRVGEDTIKIVFDEAQRAITKGQSVVMYDGDIVVGGGIIEKSL*

>CD630_12870 Clostridioides_difficile_630_NC_009089 Fur family transcriptional regulator

MANTMDLLKDKLKETGFKITPQRRAIVEILLKHDHSHLSSEEIYDLVRVDCPEIGLATVYRTMQLLDEIGLISKLNLDDGCIRYEISLHKEDCHNHHHLICKNCGKIMEAKEDLLDNIEKEIQSLYKFKILDHDVKFYGLCDECNGVSDSEE*

>CD630_12890 Clostridioides_difficile_630_NC_009089 ribonuclease J family protein

MQLFKKNTNKIKVMALGGLNEVGKNMTVVEYKDEIIVIDAGLSFPEDEMLGVDIVIPDITYLVKNRDKIKGIFITHGHEDHIGALPYILKKINVPVYGARLSIGLIQVKLKEHKMNNVKLNVIGPRQVIKLDNMEVEFLKNNHSIPDAYSIAIHTDQGIIYHTGDFKIDLTPIDGDVMDMHRICELSKKGVLLMLADSTNAEKPGFTMSEKTVGVGLDELFAKGNGRRIIVATFASNIHRLQQIINTAEKFNRKVAISGRSMVNVVGVAKELGYLDISDDMLIDLNDICKYEDSELVIITTGSQGEPMSALARMAFSEHKKVEIKSGDLVIISAHPIPGNEKLISRVINFLFEKGAEVVYSDIADIHVSGHACQEELKLIHALVRPKFFMPAHGEYRMLKRHAEIAEQLGMDKENIFVMQTGDVLELDKNSAKVANRIQTGNILVDGLGVGDVGNIVLRDRKHLSEDGLMIVVVTISKDEGKVLAGPDIISRGFVYVRESEDLMDGAKDIIKNVLNECEEKNIKEWAYLKNNIKENLKEYLYQKTKRNPMILPIIMEV*

>CD630_12960 Clostridioides_difficile_630_NC_009089 segregation and condensation protein B

MKREDIKYIIESVMFAYGEPISIKELNYIINKELSSKEIEIMLNLLIEEYREQNRGIQIIKLENKYQMCTNKDYAEYIKKIIEPKKKKSLSQATLETLTIIAYKQPITKVEIEDIRGVKCDKVLQTLFENELIREAGRLNKIGKPIIYKTTDEFLKLLNIESLEELPPIENYQEVATNE*

>CD630_13010 Clostridioides_difficile_630_NC_009089 membrane protein

MFKIFLFSSEQFVSLFIFGLFLYYCPKLTKNILPYSYTVEKIICTLLVIIMALEQLLLISSGNYSTLNSLPIGINYICIYLCIAILIFKQYHLFNIFFSWSLVCSVGELIFSKNLGYEFPSLIYFIFILSKCLIIYADIYMVDVRKFRVNRYALRDNLAICFIYFSFIFLLNTFTNSRYYYGFLSHSTTAIFTFIFVTSIMYIPALLFNRDTFILEKKKKSK*

>CD630_13090 Clostridioides_difficile_630_NC_009089 translation initiation factor IF-2

VSKTRVYQIAEELNISNEELINKLAELDINVTDKDSVLEGEELELALEMLGEDLSQENGNVIEIDGKLTVQVLATKLDKSPSEIIMKLMKMGTMATINQEISFEIAALAAKDYGFELTVAESDDTEALEIEALMEIEEDKEEDLKPRPPVVTVMGHVDHGKTSLLDAIRKTDVISGEAGGITQHIGASEVKINGHKIVFLDTPGHEAFTSMRARGAQVTDIAILVVAADDGIMPQTVEAINHAKAAGVPLIVAINKIDKPGANPDKVKQELADQGLLVEDWGGEVIAVPVSAKKKEGIDTLLEMVLLVAEMEELRANPNKRAVGTVIEAELDKGRGPVATVLVQGGTLTVGDPIVAGVACGKVRAMINAKGKRVKTAGPSTAVEILGLSEVPQGGDQFVEVPTDKIARSVAARRQQIVRDEMLKSTQRLSLDALFSQMSEGSIKDLNIVIKADVQGSVQAVKQSLEKLSNEEVQVKVIHGGVGAVTESDILLAAASNAIIIGFNVRPVPGAESLGEKENVDIRTYTIIYKAIEDIQAAMTGMLDPEYVDEETGKAEIREIYKISGVGTVAGCYVTNGKIFRNCKVRLVRDSIIIHEGELAALKRFKDDVKEVNSGYECGMSFVNYNDIKEGDIVEAYITKEVERKL*

>CD630_13180 Clostridioides_difficile_630_NC_009089 polynucleotide phosphorylase/polyadenylase

MFEHKIFKMDFAGRELSVEIGKICEMASGSCIVRYSDSMVMVNTTKSAKPRDGIDFFPLSVDYEEKLYSVGKIPGGFLKREGKPSEKAILTSRLIDRPIRPLFPKGFRNDVQVVATVLSVDQDCTPDIVAMIGSSIALSISDIPFNGPTGSVCVGLVDGAFVVNPNAEQREKSSMHLVVSGTKEAIMMVEAGADEVPDEVMLDAILFAHQEIKKIVEFIEGIVAEVGKEKMPVELYHAGEEITQLVREFATDKMKKAVQTFEKLERMENMDRVKEETLAHFEETLEDFEDFVGDIEEVLQDIIKEEVRKLIVHENVRPDNRKLEEIRPIWCETGMIPRAHGSAIFTRGQTQVLNVATLGALGDVQKLDGLDEEENKRYMHHYNFPAYSVGEARPSRGPGRREIGHGALAERALLPVIPSQEEFPYAIRLVSEVLSSNGSTSQASVCGSTLSLLDAGVPIKDMVAGIAMGLIKHDGKVAVLSDIQGMEDHLGDMDFKVAGTEYGITAIQMDIKIDGIDKEILQRALKQAKEGRIHILGEMRKTISQPKPELSPYAPKIVKMQINPDKIKDVIGPGGKIITKIIDETGVKIDIEQTGEVFISGIEIDMIKKAQELINNIVVEPEVGKTYKGKVSRIMNFGAFVEILPGKEGLLHISHIAHERVAKVEDVLNIGDEVEVKVTEIDEKGRVNLSRKVLLPKPEHKNK*

>CD630_13200 Clostridioides_difficile_630_NC_009089 M16 family peptidase

MYKTKILENGLTIIGEEIPYLKSITLGIWINAGSRIEEAQVSGTSHFIEHMMFKGTKNRTSKEIASSIDNLGGQINAFTSKECTCYYVKLIDEHIDTGIDVLSDMILNSKFDKNDIDKERLIILEELKMYEDSPDDLSYDLLVENIYANDGLGMNIIGTKESLYNITRESMLEYLNKYYIPNNAVISIAGNFNFDDMVEKIKSKFGHWEKKNLSIDISEAKFNPCFISKNKDTEQVNLAMCLKGIPFENDEEVYSMAVVNNIFGGSISSRLFQKIREEKGLVYSIYSSQTLYRKCGELGIFASMSTENLQDVYNLIKKEIENIRENYLTEKEISESKEQLKGNYILDLESTSSRMMSTGKSMLLSKKVKTTDEILECINNVNINSIKKVVDKVFNIENIGTCIVGRDVEKILHLD*

>CD630_13230 Clostridioides_difficile_630_NC_009089 protein export-enhancing factor

MKDYREDNERFDKDNCSEESSCEERECIKQFGTNEMPPQPPKDIQCITIIGEIEGHFIGNPQKKATKYEHIIPMLYSIEESNDVKGVLVVLNTVGGDIEAGLAIAELLNSTSKKVVTLVLGGSHSIGVPLATAGDYSFIAPTATMIIHPVRTTGLVIGINETFEYFKKMQDRIIQFIIRTSNIKKDVLEKLMHEKDELVSDVGSVLIGKEAVDYGLIDEVGGLKEALKKLRELIKESEEEKNND*

>CD630_13270 Clostridioides_difficile_630_NC_009089 CDP-diacylglycerol--glycerol-3-phosphate 3-phosphatidyltransferase

MNLPNKLTLFRIFLIPVFVLIMLLNVPNKFLIACIIFIIASITDALDGKIARKYNLVTDFGKFMDPLADKLLVISALTCMIEDHLVSSWMVIIIVARELTVSILRAIAAADGKVIAAGNSGKLKTITQMVSIVFLLLGAQFENVLILNIGEILILIATLLTLYSGWEYLYKNKELFMSSK*

>CD630_13280 Clostridioides_difficile_630_NC_009089 recombinase A

MSVDQEKLKALNEALGKIEKDFGKGSVMKLGEATSMSIDVISTGAIGLDIAIGIGGLPRGRIVEVYGPESSGKTTVALSCVASAQKDGGIAAFIDAEHALDPVYAKALGVDVDNLIISQPDTGEQALEIAEALIRSGAIDIIVIDSVAALVPKAEIDGDMGDSHVGLQARLMSQALRKLTGSIKKSNCVAIFINQLREKVGIMFGNPETTTGGRALKFYSSVRLDVRKIDTIKQGDKVIGSRTRVKVVKNKVAPPFKQAEFDIMYGEGISKIGDLLDIAADVDIVKKSGSWYSYNDTKLGQGRENVKKFLEDNLDLTTEIDEKVRAFYNLNEEHEESGTSVSKEIVEE*

>CDIF1296T_01398 Clostridioides_difficile_ATCC_9689__DSM_1296_strain_DSM1296_CP011968 phosphodiesterase

LGLLTKEVDVIDSVVMIVIGAAVGIIAGYFVRKNISEAKIGQAENLAKEIIDKAHHDSETVQKEKLLEAKEEIHKWRTEAERENRERRTEVQKYEKRVVQKEEVLDRKLQNLESKEVNLSEKLKVVEKKEEEVEVIKTQQLEKLESISGITSDKAKEIILTNAERDVRREMSIMIKEIESQAKEEADKKSREIIGYAIQKCAADHVAETTVTVVSLPNDEMKGRIIGREGRNIRTLETLTGIDLIIDDTPEAVILSGFDPIRREIARIALEKLIADGRIHPARIEEMVEKARKEVDNIIKEYGEQAAFETSVHGIHPELIRLLGRLNYRTSYGQNVLKHSIEVAHIAGIMAAEIGADIRLAKRAGLLHDIGKAVDHEMEGTHVEIGMDLLRRYKESKEVIHAMSTHHGDYEPQTVEAVLVTAADAISAARPGARRETLEAYIKRLEKLEEIANSYEGVEKSFAIQAGREIRIMVKPEAINDEEIHLLARDMTKKIEDELEYPGQIKVSIIRETRAIEYAK*

>QAE_RS0206550 Clostridioides_difficile_QCD_23m63_NZ_CM000660 aspartate aminotransferase

MNYSNRVSAMQASPIRKLVPFAQAAKDKGIKVYHLNIGQPDIKTPKGFFDAVKNFDSEVLEYATSEGIPELLEALQNYYKTYNMNFEKDELLVTNGGSEALLFTMMAVCDPGDNLLVPEPFYTNYNGFGQSVNVEVNAVTTKAENGFHLPSKEEILSKVDDKTKAIILSNPGNPTGAIYTKEELNILAEIAKEKDLWIIADEVYREFVYDGLEYTSCGNLEGVQDRVIIIDSVSKRYSACGARIGSIACKNKGLIAQILKLCQGRLCVPTLEQIGAVELYKTPVSYFKEVNEEYKKRRDVLYNELMKVEGVICKKPTGAFYIVAKLPVENAEDFTIWMLKEFNKDNETVMVCPAEGFYATPGLGRDEIRLAYILNEKDLHRAATLLKEGLEQYVALTKIIF*

>CD630_13420 Clostridioides_difficile_630_NC_009089 ribonuclease

MEYIIFDLEFNQGFDKKLNKTVSNEKCPFEIIQIGAIKLDSKFNIIDTFNSYIKPTIYKDIHPFISRMTNIKNSDFNDSPTFPEVYNNFIKFISSQDPILCVWGAGDLKELYRNINYHKLPSNSLPKSYINIQQHASKYFNNPAGKSIGLQNAISILELDEKMSYHNALNDAYYTAKVFIKIYNPSIVPDIYLYTSIKPKTIRYSNKKRVDYDKLFDEFRKILNRELTKDEKKIINLAYNMGKTNQFTLENVKQRKNK*

>CDM120_RS07055 Clostridioides_difficile_M120_NC_017174 lantibiotic ABC transporter permease

MTQLVRIFCSDLIKLKRTFIILMHFCIALIGMGLCLGYYKYSSADDISKIAAYLQVIAIAFPLLSSIMCSLCIEQEYYSGSYKHMLTSSNPKYLTLISKYIILICLGFGATLVSVLGFKFGVSSISNEVYFTLDFYMISIMILVGSNLFVYILHLFLSLRFGKGASIGVGIVETLLSAVLLTGLGARIWPYIPCVWGVRFISIWSSFSSSKTIEYIKVESIKGYQSIGLVCGFVTILAFIILCIWFSKWEGKKSEE*

>CDM120_RS07060 Clostridioides_difficile_M120_NC_017174 two-component sensor histidine kinase

ILVGKKFNNKKIVTLRTVFVRYSSLFFAITVVLVSILILSFPILLSLNIILPANYVEKQIYENKDKIISSKQVTKDLIPDLCEYGVYTLNGKVISGTFNKNESKEVWDLMRGFERRTITSSKNYIKLSRKNEVCIIRYSIVAEFVSPTLRTYLPKPELLGMIIFSIIFLIEIIILSKLFGKKLNAEMELLKNTTEKIEQQDLDFVIESSKIREINNVLFSMDKMKLALKDSLEKQWMLEENRKEQISALAHDIKTPLTIIHGNTDLLIEINENPELSEYMEYIAKGATQIEKYINTLIEISKTETGYILNKEVINVSEFIEDILIQIEALARTQNLNVEFSKQGNLPESITIDKELLFRAIMNVISNAIDYSPSQSKLYISVSVSNKYLKFVITDCGSGFSKADLSKATGQFYTGDLSRNSKSHYGMGLYIVNNIVQKHNGILHIENSIKTGGAMVTIEIPIV*

>PCZ31_RS13970 Peptoclostridium_difficile_strain_Z31_NZ_CP013196 peptidylprolyl isomerase

IHYEGVNYYGKKVLATVGEKEITNIDIENALKSLDPYQAMQFKTEEGKKHLLNDLVNQELFFLDAKEEKLDEEEIFKLEMKKIEENVLKQFAINKVLSSVNVTEDEKVKFFEANKSSFSKPESATAKHILVDSDEKAKEILAQIKSEEISFEDAALKHSSCPSKDMGGDLGTFGRGQMVPEFEEAVFSMAKGEVSEPVKTQFGYHIIKLEDLQESTESTFDEVKAEVEKSLLYQKQNEVYGNKINALNAKYGNLVKYND*

>CD630_13610 Clostridioides_difficile_630_NC_009089 hypothetical protein

MENKKDILFKETDKRLHNYKYLDIKIKNINLDIKRCENEYSGCGAMVYTEKTSNTYNISSSVENEVLKREERLRKLKMEKEDIEIEKEKIENALTCLNDIEMEFFNLFYNSKTKNNMTYISMKLHLDRTSCYNLKKKMIFKLSEIL*

>CD630_13970 Clostridioides_difficile_630_NC_009089 hypothetical protein

MKEILINPIGKICIQGEEVFIKLDKKYVPALNELDDFSHLNVFWWADGFDNPELRSILETPKPYKSGPDVIGIFATRSPIRPNPIALTAVQIINIDHENGIIKIPYIDANDNSPVIDLKPYTPSVDRVENPSVPKWCSHWPKSVEKSGEFNWEDEIEF*

>CD630_13980 Clostridioides_difficile_630_NC_009089 peptidase

MCFMLNIKTLCHEINDWVINIRRDLHKTPELGLEEFQTKKKIIKYLNEIGINYIEYKNHTGITAYINVSPNFETVAIRADIDALPITEELNYSYKSINIGKMHACGHDAHTAILLGTCNILFKLKDYLNVNVKFFFQPAEETIGGAQLMIEDGCLENPNVKYIFGLHVNPNINKNLIELKYNTLNASTDTLQLTVHGSKCHGAYPHQGIDAIVISAHIITALQTIVSRNTNPTDSVVISLGEIEGGIKENIVCDKVVIRGTLRTLTPETREFSKKRIREICDFTCKTFGGSISVEIEEGYPALINSNHLVDYVKQNAVELFGEENIILKDSPTLGAEDFSYFLRHCEGAFYHLGCANREKNITSPLHTSTFDIDEDCLITGVILHVKNVLSF*

>CD630_14010 Clostridioides_difficile_630_NC_009089 isochorismatase

MDNLLNELEALKSNLDNLPIEKIENYDLSKTALFIIDVNNGFARQGALYSPRVESLIKPIEMFTKKISNKLNKVIAFTDSHTPKSIELLSYPVHCLENDVESELVDELKSIENLQILPKNSTNGFFALENLDFDNIDNIIIVGDCTDICIYQFAITLKSYFNQHNIEKNIVVPMNLVDTYDIPNVHPAEILNLVFFNSMIQNGVNVLKEIR*

>CD630_14020 Clostridioides_difficile_630_NC_009089 glycerophosphoryl diester phosphodiesterase

MNIYAHRGFSGKYPENTILAFKKCLDMDIYGIELDVHRTKDGKIVVIHDEKVDRTFNGHGFVKDFTLRKLKTLNSSFEGYQSNKECKIPTLEEVLILISPTDLILNIELKTDKINYPNIEKDVLELILKYNMKNRVLISSFNSNSLKNFHKLDPSVKTGLLCYLPINNVVNFAKFLGNSYLHPPLVLVNESLIELCHKNLLGVNVYTVNEEDDILHCLKLNVDGIFTNYPDIASNLLHSKQYS*

>CD630_14090 Clostridioides_difficile_630_NC_009089 competence damage-inducible protein A

LKAEIISVGTEILLGDIVNTNSQFLAKELASLGIEVYHQSTVGDNKQRLLECFDESLKRSDFVITTGGLGPTGDDMTKETAAEYFGQKLELHKPSLEVLESFFVKTGKKMAENNMKQVYFPKDAIVLKNNNGTAPGAILKKDGKFIIVLPGPPREMKAMFNESVKPYLQQFTNEMLVSKTLRLYGIGESNLELEILDIINEQTNPTVALYAKELEVTIRITAKAENEREAFKLIKPVEEKIKSRVGKYVYTEGDISISEGETALEDAVSKLLVEKNLTIAVAESCTGGLVSSSLINYPGISSVFLEGCVTYSNDSKMKRLGVKRETLEEFGAVSEQTAIEMAEGVAKGLKANIGISTTGVAGPGGGTKEKPVGLVYTAIYINGKTIVKKNIFNGDRRKIRLRATRDLLNELRIQLEKL*

>CD630_14130 Clostridioides_difficile_630_NC_009089 membrane protein

MGEKSKGYVFIAIAGLLWATLGLFGKFLMGNGLTSEQVAFTRLFFGFIVLGVYSSIRTPQILKINKKGIIYSVIIGIICQAMFNLCYFKAIDIAGVSIAAVLLYTSPLFLAIFSKICYKENITRSKLFSLILCFIGAIMAVTGGRLDFQGLNAFGLLLGVLSAIAYALMPTISKNALKEFSSSTILVYSFLFGAIFMIPSSRPWEILNYAKDLDVLSCMLMLGIVPAALAYIFYAAGISKGVELSVAGVVASVELVGSVIIGCTILGESFSLGKLFGVMLMLISAVVALNLSYDEIRIFYKSNKLKQIEKTESI*

>CD630_14180 Clostridioides_difficile_630_NC_009089 MATE family drug/sodium antiporter

MDTKISINENMSLGKRFFKYLAPSVVAMWVFSLYTMVDGIFVSKGVGELALAAVNISMPFINFIFAVSLLFSTGASTIIAIYLGKKDIKSANEVFSFNLVSIIILSIIILAITFFNLDRLALFLGATESTIGMVKDYLGIIIFFNGFFIVSYSLEVIIKTDGFPILATVGVIISALTNIILDYLFVIEFGWGVKGAGIATGLSQVFSTIFFLIHFLRKNSTLNFSKFRIDFKTLRKIVFIGFPDSTTELSCGIVVLLFNLSLTKYIGENALIYYSVINYINTLVLMTMMGITQGMQPLTSFYYGAGNIDNVKKLLKMGIKATIIASVAVFAICMAFSGPIVSLFIHPEETMLFNEGVRVFKIFSISFLLVGINVIISGFFVSVEKPSISTVISLGRGLVIVVLSLISMILIFGGQGIWMTTIVSEFICLILSLVFLKKNFSTLDSNLNKVA*

>CD630_14200 Clostridioides_difficile_630_NC_009089 diguanylate kinase signaling protein

MFKEIFLRTFPGFLIIRDSNYRIIFINDNLKNLIKSYMQDNPLGMTNIEIAKKLPDNIAKFFTDSHNIQLDWEKNYPYDKISNWILEFKKDTTSYWNVLEYKVDVDEKTYIITMANDITKLYEENKRNLHYSITDPLTGAYNRKYLNDRFDIFIGDYIVLIDLDNFKMINDYEGHNVGDKILCDFVSLLNKELINSTSIIRLGGDEFIVIFSSDVDKNYVYSQLEALRENFLKVFSKYKYLSFSYGVDTVKRNLKLTIVELDKKMYKNKEKNKKKFDKNDY*

>CD630_14310 Clostridioides_difficile_630_NC_009089 DinG family helicase

MDNIISVLNDVVFLDIEVSGLDCLNSEILEVGAVKVKDWKIYTYESLIKNKFEVPVEVFSVCKNLDKNDLEIANEIELVEDRLVNFVEDSFIICHDLSLKKKFFEYHMPKLKNKFIDLIELAVILEPYHKDYSLEYLKNTLTNCNSKVENRALSDAIDIINIVNCLLVKFNNYEKTTLEPLSFKINSYLKKFNLPTWEWSKFLEEANYDLSNNINIKKEYNIFDSKEEKKKERETLKILNEEEKNYEELLKYKTIWENKEGFTYEYRPGQYELTKTIRELFRNSEDEEKIACIEAPTGIGKSVGYLLPAILEARINKKRLLISTDTKELQIQLINKDIPNVLDSLGLNGKVSYGYIKGKNNYICIDRLEAYIDDYESQNPTKGEILSLIFLKRLVEGGKYGDIEEINYLVFDNFKEISTHLRNVSCDPNMCRPKKCKKDCLYKNRIEELKEEHITVVNHSLLAKWPYKDEKPLENIIVDEAHNLTEKGYDFFSSIINSKSLRYLLQEIYPYEFIQNSSFIYKKYSRNMRKIKAFDKFYNVLKIGREDKQKIARSINLIIEEIDSILNFGNCNEYNNVSNYNLRWELNLQIDEIVGKLKKDGIDTEISYRAYSEKIKLSCEKIIKNLVSIIIIIYRNIDDDSIDKEADIYKFGKAKTRDLEDIKIIFEIFLEYDEKDDYARIVEIDKNYNVFEFRVVPLKIADLFEENILSQLEKGIFLSATLSLSESMSYFKNTLGIDRVKNVEKIIEPIFDYKNRVSVVGFSDICEYRNSEFPNEMSKIISNISKITEGHTLALFNSKDRQEKTYEILKKYLHSFNLEIYADKKGIRHLNDLNRKCVVLGSKGCFEGVDIPGDGLVCVTLDKLPNLNPKDPLYFTIMKKYGIDYYTINYPQMTIKVKQAMGRILRSKYDYGCFVIFDVGTNISVLKRLEKDLHDCKISKVNSNEFYTYIRRHLNKSRSLILKSVIFDTIKALNVDAKMDNNDVDKDIIKKDINENIRQRAVKGEVYHIDIIKKDMKVKYFDRNYLINLDIFMREEDKN*

>CD630_14320 Clostridioides_difficile_630_NC_009089 ribonuclease

LAKQKFYAVKKGKNIGVYNTWDECKKQVNGFSGAEYKSFSTFQEAKEYIDGSEKLSFQEDKEFIEAYVDGSYEHSVKMYGSGVVILKNNEVIKTYSEKGKEKTLVSMRNVAGEIEASKIAMQYCIDNNVQNLILYFDYEGIEKWCTGVWKTNKEGTIAYKNFYDSIKNKLNVKFTKVKAHSGNKYNEEADKLAKKAIGV*

>CD630_14330 Clostridioides_difficile_630_NC_009089 bifunctional peroxiredoxin/chitinase

VIYMPNLPSLGSKAPDFKANTTNGPIRLSDYKGNWIVLFSHPGDFTPVCTTEFLCFAKYYDEFKKRNTELIGLSVDSNSSHLAWMYNISLLTGVEIPFPIIEDRDMRIAKLYGMISKPMSDTSTVRSVFIIDNNQILRTILYYPLTTGRNIPEILRIVDALQTSDRDNIVTPANWFPGMPVILPYPKNYKELKNRVNSCNKKYSCMDWYLCFVPDNYNDEEVSKKIDNTCSWKKEHTKNIENECNCEHEHHDYLNKALDCKQEHKTDIKDDCNHEKKHTKNTNKVHNSKQDKFKDKSCDEMNFNYDKDESCDKINSSYNKEDSSYEDFYKHNYKNYDYTSEKNTKKIAMKTLKDSKKLVRPQITDPYNPIVENANCPDINPIVAEYVLGNPTNVDAQLLDAVIFAFAEIDQSGNLFIPYPRFLNQLLALKGEKPSLKVIVAIGGWGAEGFSDAALTPTSRYNFARQVNQMINEYALDGIDIDWEYPGSSASGITSRPQDRENFTLLLTAIRDVIGDDKWLSVAGTGDRGYINSSAEIDKIAPIIDYFNLMSYDFTAGETGPNGRKHQANLFDSDLSLPGYSVDAMVRNLENAGMPSEKILLGIPFYGRLGATITRTYDELRRDYINKNGYEYRFDNTAQVPYLVKDGDFAMSYDDALSIFLKTQYVLRNCLGGVFSWTSTYDQANILARTMSIGINDPEVLKEELEGIYGQF*

>CD630_14450 Clostridioides_difficile_630_NC_009089 para-aminobenzoate/anthranilate synthase glutamine amidotransferase component II

MILMIDNYDSFVYNLVQYIEELGETVVVKRNNEIKISDIEELNPEVIVLSPGPCSPKEAGICIDIVEHFKGKKPILGICLGHQTIGHVFGGDIIKAQQPVHGKVYSINHTNKGVFRGLKNPLNVTRYHSLIIDSNTVPKELEITAITDKGEIMGIRHKKYLIEGVQFHPEAILSEYGHEMLKNFITEARERVHV*

>CD630_14680 Clostridioides_difficile_630_NC_009089 iron-sulfur protein

VITISHITAKNMYKSLEERINKFPQGAPPSDTLYKILNVLYTEQEAKLVAQLPIKPFRVKTAAKIWSVSESEAYRVLDKLASKALILDIEDNKGKKYIMPPPMAGFFEFAMMRTRHDIDQKLLAELYYQYMNVEEDFIKDLFYSTETKLGRVYVQEEVLTNDNEVSILDYERATHIIDESTHIGISMCYCRHRMQHVGKACDAPMDICMTFDNVANSLINNKFARRVDKIECKELLHQAYEHNLVQCGENVRKGVTFICNCCGCCCEAMVAAKRFGNLHPVQTTSFIPNINHENCVKCGKCITACPIDAISKVKEDGKEYIKIDEDRCLGCGVCVRNCHKNSIMLLKRDEKIITPANSVHRAVLMAIEKGQLQNLIFDNNALASHRAMGAILSAILKLEPAKKILASKQLKSVYLDKLLSMNDK*

>CDIF1296T_01539 Clostridioides_difficile_ATCC_9689__DSM_1296_strain_DSM1296_CP011968 penicillin-binding protein

MFKQRLSKLLSSTLVLSMLFTAAPNITFADNTKDNSEKYQSSDIELHDYSKNAESYTKTKALAKEKIQTLLSKYGAVSAQYALIDNGKIEISGNGGVYSKQDNKNLNKDNMYSIASISKMFTTTAVMKLVDDGKLNLDTPVVKYIPEFKMADDRYKEITPRMLLNHSSGLMGSSFKNTILLADNDSYGHDNFLKELQKQRLKAKPGAFSVYCNDGFTLAEILVERVSGMSFTNFLDKYINNPLNLQNTKTTENSFDSSKLAKAYVPYWEDAVPQDNLNAIGAGGLYSSAENLCTFAQTFMKNSNGILSPASVKAMENKEYLNGLWPEGEDSILGYGLGWDCVNTYPFNQYNLKALTKGGDSLLFHSNLIVLPDENMAVAVLSSGGSSQLNEIIGQEILLSALKEKGKIKEIKPDKTFSKPQQVKMPSSLKENSGLYASSNMIKVDVNDNGTLTVSSPYIENGPEDKYVYIGQDRFVSEKGNSCLKFVKEKNNITYLNMSSYDDVPGLGQTASLYYVAQKVDDNNISNSVKEVWKKRSGKGYYLVDEKYTSQSYMFGSVKASFSLSDETPGYIVNTKIMDENNSNAFIEIPGVIGRDLSDIKLHKENGTEYLSFGTLTYVSEDSITNLPAEKSFTCELESNGYAKWYKIGDDIANKKIEVNLPQNSAFAVYDDKGVPVNYSLVTKNNRVRLPKGGVIVFLGSPNARFEVTYQDEVNASALTGTDRYETSIKISQAGWENAENAVLINDSAIADALAATPFAYKKNAPILLTGSSQINEKTLAELKRLKVKNVYVVGGEASINEKSLDTIKSNNISVSRISGSDRYQTSMNIAKELNNISNISKISVVNGEKGLADAVSIGAVSAQNDMPIILTNENSNITEINNVFKNKKIDKSYVIGGEYTVSKNIESKLQNPQRISGSTRNETNAKVIKEFYKDSKIDNLYVAKNGMNKQDDLIDGLSVGVLAGKTKSPVMLVGNSLDYNQKELFKTMRFKSVTQIGGNGNENSFKQIKEIA*

>CD630_14790 Clostridioides_difficile_630_NC_009089 ferrous iron transport protein FeoB

MSIKIGLIGNPNCGKTTMFNGLTGSSQYVGNWPGVTVEKKGGKLKGNKDVEIVDLPGIYSLSPYTLEEVVTRNFMLDDKPDAVINIVDASNIERNLYLTTQVLELGIPTVIALNMMDIVNKNGDKINIKELSEVIGCPVVEVTAVKGQGIMEAAEKAVELASSNNKLNFKLPFVDESKDAIEKIEKIIEEKTPYIDVETRWLAIKLFERDENVIQKLNISKTILNSIEEITRNCEDELDDDSESIITANRYEFISSIISSIIKKNRKGKETVSDKIDKIVTNRILALPIFALIMWGVYYIAVSSLGTIATDWTNDVLFGEIIQGNVSNFLASLNVAEWLQGLVVDGLIGGVGAVLGFVPQIMLLFLLLSILEDCGYMSRVAFIMDRIFRKFGLSGKSFIPMLISSGCGVPGVMSTRTIENDRDRKMTIMLTTFIPCGAKIPIIALFAGALFGGASWVAPSMYFLGIAMIIICGIILKKTSLFAGEPSPFVMELPQYHIPSAKGVLIHMWDRGKAFIIKAGTIIFVACGVIWFLQSFNWSLQMVDAGDSILASLGNIVAPIFAPLGFGNWQSSVATVTGLVAKENVVGTFGVLFGISDATEQDPTLLASVASMFTVASAFAFMAFNMLCAPCFAAIGAIKREMGSWKWTWITLGFQTLTAYIIALLINQVGSLVLGTGGSIAGAIISIFIAVAVVFVVLTYSNKNMKKEKMGKLSYMKN*

>CD630_14830 Clostridioides_difficile_630_NC_009089 sulfonate family ABC transporter ATP-binding protein

MVRSGFLLENIYKKYLVDNKEHLVLDNISLNISSEEITVILGESGCGKTTLLRILAGLENATSGNIYFFNNDKKCTPKVGMVFQESRLMPWLNVSENILLHTEKDNRNKVDLDKYLKMMKLEKFKNSYPNELSGGMAHRVSIARALSFNPDILLMDEPFAALDYFTRRKMQKEVVNIHKNTKKGVVFVTHNIEEAMEIAKKIIVFSKNKRIKQFSVEDEYNRDLTKNYYINLKKEILRELGEF*

>CD630_14890 Clostridioides_difficile_630_NC_009089 methionine ABC transporter ATP-binding protein

MISIKNVNKYYGKIQVLKDVSIEIESGEIFGIIGHSGAGKSTLLRCINGLEEYQEGSVLVSDKEVKSLNEKQMRDLRKELGMIFQHFSLLERKTVFDNVALPLECFGYSKAEIKKRVLELLEVVGISEKKNDKPRNLSGGQKQRVAIARALALNPQVLLCDEATSALDPNTTKSILSLLEDINKKLGITIIVVTHQMEVIKQICGRVAIMENGEVLEVGDTEEIFLRNTKGLRKLIGEESIILPKGTNIKILFPKDISNEAIITTMARELNIDVSIIFGKLEQFKDDILGSLIINISDKSGEQVKQYLTSKGIRWEEMINE*

>CD630_15020 Clostridioides_difficile_630_NC_009089 2-deoxyribose-5-phosphate aldolase

MKHILKTVDHTILKATTTWEDIKILCDEAVDMSVASVCIPPSYVKRASEYLKGKIKICTVIGFPLGYQTTATKVFEAKDAIENGADEVDMVVNISDIKNKDYDNIGKEIKEIKKAIGDKVLKVIIETCYLDEDEKIKMCEIVTMSGSDFIKTSTGMGTGGATLEDIKLMKEHVGKNVKIKAAGGVKSISDAEKFIEAGAERLGTSSICKILKNEDTTDY*

>CD630_15120 Clostridioides_difficile_630_NC_009089 pantothenate synthetase

MLVKEIKLLRNIIKDWRKHGYSIGLVTTMGFLHEGHQSLIKKAVKENDKVVVSVFVNPTQFGPNEDFNSYPRDIDKDFKYCMDSGATVVFNPSPEEMYLKGNCTTINVSGLTDFLCGAKRPVHFGGVCLVVSKFLNIVTPDKAYFGEKDAQQLAVIKRMVKDLNIDTEIIGCPIIRENDGLAKSSRNTYLSEEERKSALILNKSLSLAKEELVKGNLNPENIKELITAKINSEHLAKIDYVEIVDSETLQPVKQIEHSILVAIAVFIGKTRLIDNFTFELNI*

>CD630_15210 Clostridioides_difficile_630_NC_009089 tyrosine--tRNA ligase

MKSIDEQMRIIMKGVDDLIDEKELREKLIKSEKEGKPMIVKLGLDPSAPDIHLGHTVVLRKMKQLQDLGHQIVIIIGDFTGKIGDPTGKSKARKALTTEQVLANAKTYEEQIFKVLDKEKTIVRFNSEWLAKLNFEDVIKLAATITVARMLEREDFKKRYEGQMPISVHEFFYPLMQAYDSIALEADIELGGTDQRFNLLMGRSLQREFGMESQIVIMMPLIEGLDGKEKMSKSLGNYIGIDEEAGIMYQKSMEIPDELIIKYYNLVTDVHPDEVNKIESQLKEGSVNPRDIKMNLAREIVTLYHGEESAKEAEERFKSVFQKGQIPEDIQTIQVKEDGFDLIEVLVSNEIVKSKSEVRRLASQGGVKVNGEKVEDLSTIVKESELVVQIGKKKFVKIELVK*

>CD630_15280 Clostridioides_difficile_630_NC_009089 ABC transporter ATP-binding protein

MLIKLENIQKYYKVGKDELHVLKSLNLEIESGEFVMIMGKSGSGKTTLLNILGFLDVFDEGRYIFDGTDVTNLSENERSVFRNINIGFVFQQFNLIETLNVYQNVELPLIYNKALKKSNREEIVKDKLSSVGLLDKLKQKPLQLSGGQQQRVAIARCLANDPQIIFADEPTGALDSETSREIMELLTRLNKQGKTIIMVTHDQDLTKYATKVIRLKDGVFTSEV*

>CD630_15320 Clostridioides_difficile_630_NC_009089 ABC transporter ATP-binding protein

MEEILSVENIKKEYGRKGSKHEALRGITFKVYKGEFVGIMGSSGAGKSTLLNIISTIDLPSSGDIYINGKNTIKMKQNELADFRRDNLGFVFQDSNLLDTLTIKENIMLPLSLKNERVSVIENRIKEISKELNIESILDKYPGEVSGGQKQRGAVCRAIATKPSLVLADEPTGALDSKSARDLLNCLLKLNKDSNKTILMVTHDAISASFCNRILFIKDGIIFTEIVKGESNREFYNKIVNTVSLIGGVNKNDFI*

>CD630_15340 Clostridioides_difficile_630_NC_009089 gamma-glutamyltranspeptidase

MKFNAYQHKYSSIRNVVYAKNGAVATSTPLASQAGLEILKKGGNAVDAAVATAATLAVVEPTSNGIGGDAYALVWIEEEKRLYGLNSSGFAPENMELKNYNNMKEMPKYGFGAVTVPGIPAAWSELNKKYGKLSLMECLSPAINYAREGYVVSPNVAKVWKKSYELYEKELIGEEFKPWFDTFSKDGKAPEAGDIFICEEQASTLEEIANTQAESFYRGRLADKIDEYSKKFNGAIRKSDLECFYPTWVEPISTEYKGYKIFEIPPNGHGITVLMALNILKELELEGNIENVEDIHKIIESLKLAFADSKTYVTDIEHMKVKIQELLSQEYAKKRSLLIDNKEALYPTAGEPYCGGTVYLCTADKDGNMVSYIQSNYINFGSGIVIPRTGIALHSRGNNFNLDPKHHNVVKPFKKPYHTIIPGFLGKEDKAIGPFGVMGAFMQPQGHIQVLTNMIDFGLNPQEALDAPRWQWIKGKEIEVEPEMPKHIIDSLIEKGHEIKVIHDTVDMGRGQIIFKTEQESYICGTESRCDGHIAVY*

>CD630_15370 Clostridioides_difficile_630_NC_009089 oxidoreductase

MDAKKVKVPVREQEPAVRATNFDEVCLGYNKEEAMAEANRCLACKKPKCVGGCPVGIDIPGFITKIKEDDIEGAAKVIAKSSSLPAVCGRVCPQESQCEGVCILGIKSDAVSIGKLERFVADWSKENDINLSDTEPKKNQKVAVIGSGPAGLACAGDLAKKGYDVTIFEAMHEPGGVLTYGIPEFRLPKQAVVQPEIDNIRKLGVKIETNVIVGKTITVDELIEDEGFEAIFIGSGAGLPMFMNIPGENANGVFSANEFLTRVNLMKAYRDDYDTPISSGKKVAVVGGGNVAMDAARTALRLGSESYIVYRRSEKELPARAEEVHHAKEEGIIFNTLTNPKEILVDENGYVKGMVCIRMELGEPDDSGRRRPIEIEGSEFVLDVDTVIMSLGTSPNPLISSTTKSLDINKKRCLITDENGQTSKEGVFAGGDAVTGAATVISAMGAGKTAAASIDEYLKAKVNA*

>CDIF1296T_01612 Clostridioides_difficile_ATCC_9689__DSM_1296_strain_DSM1296_CP011968 ABC transporter ATP-binding protein

MVKVVYSRYYGIVVKIKNYGEYKMNILSIRNISKTYCGNIPFKALDKVSLNIEKGEFVSVMGPSGSGKSTLLNIISTVDRQSEGEVVLDGYDVSKLKGEKLAEFRRKQLGFVFQDFNLIDTLTVGENIMLPLTLEGESIKDMNIQTKSISKFLGIDKILDRKTYEISGGQAQRCAIARAIINKPAILLADEPTGNLDSKSTDDVLKLFTRINKEQKVTTLMVTHEAYSASYSDRVIFIKDGCIYTEIKKSESSNSFYSDILAVLSQIGGVR*

>CD630_15410 Clostridioides_difficile_630_NC_009089 MATE family drug/sodium antiporter

MENQQLLGTERISKLLLKYSIPAIIGMLVNSLYNVVDRIFIGNIPGVGPLAITGLGVTMPIMTIILAFGMLIGIGTTTTISIKLGQGKVEEARKLIGNAMTLSVITGIIIMILGILFANKILTLFGASENTLIYAKSYINIILLGTVVNLLSFSLNHSIRADGSPKISAGIMIVGCLTNIVLDWILIFGFNLGIQGAAIATVTSQALTAILTIGYYISGKSNLRFSKSNLKLDKKLIKAVFAIGMSPFAMQLAASLVQVISNIALKTHGGDLAIGAMATISSIAMVFLMPIFGINQGAQPIIGFNYGAEKYDRVKKAYLGSLVVATIILCMGMVVVMLFPEAIIGIFNKDPELMNISVNGLRIYLLMLPIVGLSVTGTNFIQSIGKAKMAMLLSLLRQVILLIPAVLILPTFLGLQGVWTAQPVSDFIATVITGIVVFRELKRYTPKTEKLNENERLNEITTE*

>CD630_15490 Clostridioides_difficile_630_NC_009089 histidinol-phosphate aminotransferase

LREKESIRELRGYEPNHVNCKVKLDANEGSKRLFKYLIKEISDSDIDLNLYPEDSYSDLKESIIDYINISGVNKKNLLVGNGSSEIIDLIIHTFVDKDEVILSFSPSFSMYSIYSQINGSKFIGVESDENLVINIDNVIEKVKENNPKIVIVCNPNNPTGTILKREEIIKLLDSTNSLVVLDEAYMDFGEESMLSDVFKYDNLIVLRTLSKAFGLAGIRTGYMLSNSSLINSVEKVRPPYNLNSLSDFIATRALRNKDVVKAYIKEVKEEREVLYEEMIGMGIKAYKSQANFILFYSEIENLSQKLIDRGVLIRKFGGKLENYYRVTIGDKEENSMFVGAIRDILKKEK*

>CD630_15550 Clostridioides_difficile_630_NC_009089 amino acid permease

MGSNELKKTLGVSAALSTVVGSVIGAGVFFKPQAVYTLTGGAPGLGILAWLIAGIITITAGLTAAEVSVAIPKTGGMMVYIKEIYGEKLGFLTGWMQIVLFYPGMMAALGVIFGEQASALIGSPSLLLPIAIGIIIIVAGLNMLGSKTGGVIQTVSTICKLIPLILIMVVGFIKGGGNNPILTPMVGEGLSLGSVLGQVLIAILFAFDGWMNVGTLAGEMKNPGKDLPKAIIGGLSVVMAVYFIINLAYLWVLPANELANYASPASAVAEVIFGSMGGKIISVGILISVFGALNGFLLTGSRVAYTLATDKTLPKYGIFSKLNSAQVPANAIALVSVIASIYALSGQFNLLTDLAVFATWIFYVLTFIGVMKLRKTHPNIPREYKVPLYPIVPIIAIASGIFVVVNQLCFAGMKTTMISIGGLVITAIGLPVYAYMTRGVKR*

>CD630_15570 Clostridioides_difficile_630_NC_009089 peptidyl-prolyl isomerase

LNKKLYIGMVGILSLMMVGCNKSLAKVNDVEITKEQYKKTKAVLSATNNYINGQSLDELEKTLDKKGRNKLENVIISFMVDNELLYQEAKDKGLTPSKSEVDSKYQELEDKMNLNTSYKEKMDKAGVDKDYLKQEISRDLAIDKNKKAFEDRINISDNDMEAYYTSHKKDFNVEEVSASQILISTLDKNKKEVSKDKKEALKKKADNILTKIKNGESFESLAKKYSDDKATGKNGGQLGYFTKDDKNAEFTKEVFKLKKNEVSNVFETSYGYHIVKVTDKRERQKSFNECQSLIRESILNEKYIEHIKKLNEDAKIDR*

>CD630_15660 Clostridioides_difficile_630_NC_009089 acetolactate synthase large subunit

VRMNGAKVILECLKKEGIDTIFGYPGGAVIPLYDALYDYSDDFKHIRTSHEQGLVHAADGYARSTNTVGVCFTTSGPGATNAITGIATAFMDSSPMVVISGQVPTSLLGKDSFQEIDITGATLSMTKHNYLVRNTKELVPTIKEAFRVANSGRKGPVLVDVPKDLFLAEMDFSGEDYDLCQIDDYMDYKSDFDLDDETNIKLLNEAIDIIKESKKPVIYAGGGVKSSDSEEILEKFATKIDTPVLNTLMGLGNIDRKNELSLGMVGMHGSRESNLALSNSDLVIAIGARFSDRVISKSSEFAKNAKIIHIDIDPSEISKNIESNVSLVGDVKLVLSLLIERVESKNNSNWKEEIKRFRKSEGVQTYEFHPQNILKKINEKYETLKKPTVVVTDVGQHQMWAAKYWNFKGNKSFITSAGLGTMGFGLGAAIGTKVGNVDKNVVLVTGDGSFRMNCNELATVANYNVPMLILLLNNRTLGMVRQWQKLFSNQRYSQTDINENVDYVKLVNAYNIDGYKVSSMEELGKALDMIDFNKPVFLQCDIDKDYDVYPIVAPNDALENLICN*

>EAA_RS0208060 Clostridioides_difficile_CIP_107932_NZ_CM000659 phosphoglucomutase

MDYTKTYEEWIKGSYFDEDTKLELENIKNNEKEIEDRFYKDLEFGTAGLRGIIEAGTNRINKYTVRRATFGLANYILENTTKEETSRGVVIAHDNRHKSRQFCIESANTLAACGIKAYIFDSLRTTPELSFAVRSLNAIAGIVITASHNPPEYNGYKVYWEDGAQVMPEIANAITEKVNSIHDYSTIPTLTEANKNLVVLLDESQDTKFIEAVKSQIIRKDLVKNVGKSFKIVYTPLCGTGNVPIRRALKEVGFENIIVVPEEENPDPNFAGLDYPNPEEKKALNRGILLAKEKGADLVIATDPDCDRVGVAVKTTTGEYALLTGNQIGGMLTHYIIEGLKENNKLKENPTIIKTIVTSEFGADIAKANNVDVLNVLTGFKFIGEKIKLFEQNKNRSYVFGYEESYGYLVGTHARDKDGVVSSLLISEMAAFYYSKGISLYDGLIELYKKYGFFKEQTISLTLKGIEGVEKIKEIISYFRENQIDYINSIKVVDKKDYKNGIDNLPKSNVLKYFLEDESWVAIRPSGTEPKLKFYIAVKGASDIEADKKIQGLKKYIDDMVEKIKIKYYYFIR*

>CD630_15790 Clostridioides_difficile_630_NC_009089 sporulation-associated two-component sensor histidine kinase

MDTHNKYVNFIKNIPVPFLYCRIVKRQEDIEYRVEYISKGMGKVLQLEEGICDKNILDVLPVFKSKKYFKELFSNEVDCIKRYIPTLKNWINIKKQIIGDSYIILYFGKIVFDYRQIIDSFDKKEKVAYIKDEEGIYIDCSENLIPILNNNIKTTKDIFGKNDIEVWGENTGKLFRDDYREGVSSKKRFLQNLFEYEETFFMVEKYFLYDEDELLGTIGIVDNIIYSGYSNRNYNSKDLMKMIEHSIPENMFYKDVYGNYIGFNSGFLNLACMNKEELLGKNSYKISEEEALIDKIFESDKGVVENKKVVTFELNISMNDENKCIEITKRPFFDSYGSVIGIIGTARDISRRKRLEEEMDKTRMEFFANLSHELRTPINLISSSLQVIEKKEADLIESNDTLKRNLGIIKQNGNRILRLVNNVIDFTKMQSGYLDFKPEESDIIAFIEEICMSVADFASQNNIQLTFDTEIEEFSMLFDSEKLERIILNLLSNGIKYNKKDGKINIFLYVKDNVFNMKISDSGIGIPKEKIDKIFNRFEQIDNELSYRVKGSGIGLSLVKSLVELHEGSISLKSQLGIGSEFIVSLPVRSKNNIEKYNHKREISNELSKKLEIEFSDL*

>CD196_RS08285 Clostridioides_difficile_CD196_NC_013315 hypothetical protein

MENKKYANGGYSEDWYERGESTAKWFQNDREEYERAAYDEDRERRGSNCGCSDSGGNRPRNCERFRREAEIREREAREAFCESSERKKEALAYECEARKLWEEAEKYWDEYSKYNYKGIEYLAEAARLFDEGMECEARRNGNNGGNNNNCCHKCHKCNCNCCRK*

>CD630_15830 Clostridioides_difficile_630_NC_009089 hypothetical protein

MSYFQYQGNSCFYKEYGQGKPIIFLHGNTGSSNMFKALVPLYVENFRCILIDFLGNGQSDRVSQFSPDIWHDEALQTIALIEHLNCGKVGLIGTSGGAWAAVNTGLERPDLVEAIIADSFDGRTLNDNFIDNLITGREKSKQDIQARKFYEWCQGKDWENVVNLDTKALLQCANEKRPLFHKELCKLEMPILFTGSKEDEMCRHNLEEEYKQMATFISKASIHIFSQGGHPAILTNADEFAQLAKVFFNY*

>CD630_15950 Clostridioides_difficile_630_NC_009089 serine acetyltransferase

LFKKINKDIEYIMKNDPAARSKIEVFLLYPSVHAMIMHRMAHALYKKKKLFTARLISQISRFMTGIEIHPGAKMGEGILIDHGMGVVIGETAEVGNRVTIYQGATLGATGKDTGKRHPTVGDDVLIGAGTKILGPLNIGSNSKIGANSVVVKDVPNGATVVGIPAKIVKIRNLEPVKKNKKEVSYEYDELDNVYYI*

>CD630_16040 Clostridioides_difficile_630_NC_009089 multidrug family ABC transporter ATP-binding protein

MLAIEIDNLVKEYKNGVKALNGLSFNVNAGEIFSLLGPNGAGKSSLINILTTFYKPTSGNVTMFGKDLVDNPSWIRTQIACVAQQISIDEHLSLMENMVFQSKMYKVEPQIAKQRIDSLIDKFDLSSYLKYPTSSYSGGVKRRLDIAMNMVSSPKILFLDEPTVGMDVDSRKSMWDMLLKIRDEYGTTIFLTTHYLEEAEQLSDNICIMKNGKDLAQGTPSSLRSYIRQNILRITFHNTEDIKKYKDSIKSTGLVKFMSVRENSIFISVNDSRTAFTLINKWLLEHDIEFDAIEIVEPSLEDVFLALTSSKKSLKEEWEC*

>CD630_16070 Clostridioides_difficile_630_NC_009089 multidrug family ABC transporter ATP-binding protein

MIEIKNVSKTYKRMQGLKIRKIEALKNVSFNIEKGKITALLGINGVGKSTMLKAIAGLINIDSGEIRIDGEKINEKVYNKLAFVPDVQSHFSNTTIKETFEFMEIFYSKWNKEKSKEMMDIFKLDEDEIIDNLSKGNIARVKLILGFCQDPEYILLDEPFTGIDLFKREEFIGVIAQYMEENQAIIITTHEIVEIESLVDEVVILDEGQIITSFNAEELREREGKSILDKMREVYKNE*

>CD630_16200 Clostridioides_difficile_630_NC_009089 major facilitator superfamily transporter

MKSYTKAKWSVWGIITFSFVLVLFLRMSTAVVSDNLANELGFNSIQISNIASFCLYAYAFMQIPAGILIDKYGARKISSLGIIMASLGSILFGLIQSIELAYISRVIVGAGTSVILLCILKIQGRWFNKSEFASATAKFSFVGNLGGVLATFPLVFLSELVGWRNSFLLIGIIGVVIGCFMYIIVRDTPKEYGFNVDTEPYEKSEKVNIVDGIKSVIKNKSTWYNSMIMFSFVGLTSAFISLWGVRYIMDVYGVSKSFSAFIVSFFTYGFIFGSIIMDFVFAKIRSSKFNIIKFGAMIDLFIWIVIVVVYQVKPPIIVLPISFFIMGCIVMSHLQVFNDAKYKNKEIYSGLATSVINTFEFIGSGIINLIIAISLQVNSYNIVDGYKKGFVVFIVLSIITIVSSHIGVKNDDFKAI*

>CD630_16230 Clostridioides_difficile_630_NC_009089 oxidoreductase

MSLKSLKIKENLYWVGSLDPDLRVFDIIMYTPYGTTYNSYVLKGTEKTVLFETVKDKHFDNYIERLNDLNIDFEKIDYIVVSHTEPDHAGSVEKLLDLAKNAKVVASETAIKYLKEIVNKDFEYVAVTDGDTLSIGDKTLEFFSVPMLHWPDTIYTYIKEDKTLVTCDSFGSHYSNDKIVNTLDENEEKDYLDALRYYYDCIMGPFKPSMVTAIEKIKDLDIDTVCPGHGPVLTENPRKIIDLYYNWSVNEQIKLEKEVTICYVSAHGYTKIMAEAIKAYIEKNSNYKVNLFDVIEHKQEDILAKIAVSQGVLFGTPTILGDALKPIWDILISLNPVLHGGKVASVFGSYGWSGEGIENAMERISQLRMTAVKPFAVNFKPSNEEIDKLHSYTGKFLDKLNSTFGSKKKTKKFKCVICNEVFEGDSAPSVCPVCGAKEDQFIEVEEDEVTFRKDTDEYFVIVGNGAAGFYAADAIRKRNKTCKITMISNEDELTYYRPALSDGINEELGSDFYMEDKDWYDKNNIVVILGTNVDKLDEVNKTIIVNDGAIKFDKLVIATGSRNFIPPIKGHDLENVFTLRNIKDLYSVKEALEKSKKVVVIGGGLLGLEAAWEFRLKGLEVVVVEAMDSILSKQLDKEGSKILEQCVRDTGIDVRLGVAVDGIEGDVKAQKVVFKDGDSVDCDMVVFSIGVRANTQMVQDTSVKIDRGIVVDKTLQTNVKDIYACGDVAQVGNISLAIWPSSVEMGKIAGANASGDNLTFESEVYPVSLDAMNVKVFSIGNIQNFDKEISSKDEGQRIYKKLFMKDGSLVGAILINDLSCTVKLIRLISEKGDFEDIMKADIL*

>CD630_16310 Clostridioides_difficile_630_NC_009089 superoxide dismutase

MKKKILIPVIMSLFIISQCITSFAFTPENNKFKVKPLPYAYDALEPYIDKETMKLHHDKHYQAYVDKLNAALEKYPELYNYSLCELLQNLDSLPKDIATTVRNNAGGAYNHKFFFDIMTPEKTIPSESLKEAIDRDFGSFEKFKQEFQKSALDVFGSGWAWLVATKDGKLSIMTTPNQDSPVSKNLTPIIGLDVWEHAYYLKYQNRRNEYIDNWFNVVNWNGALENYKNLKSQD*

>CD630_16330 Clostridioides_difficile_630_NC_009089 serine protease

MICTIKPLKISIKKSTEPLIEFISDTTYKNKRSSYIKEVYFTRYIGVDDSIDKYIEKIKNIDTYFFNNPTIPYIRLNNLKINFDKEQTDKMLKIFNIYNSSKFNSWDLYNIDFPCKIENDTLNWTKKIAFKNTLDLFSTSMPNCTSSIIKNFAVKLLCWMDYFLPKLFYNKVKTTISPKIVYFGNIKRQELFFLYFLSQLGCDILYINPNKDILDLYPECSKFSTLFELSRKTPNILEIPFERHITNINYSENTNKTSPTTNKPNSKNTLNNTNLTSKKIVQDEEVELSYEDLAKLSASVVMIVVCNNENKPFKSGSGVVINNEGYILTNLHVVNDGYSFLVRFENDDKVYTSYQIIKYHSDYDLAVIKVDRKCKPIPVKVSKKPVRGQKIVAIGSPLGLFNTVSDGIISAFRDFETVQMIQFTAPISSGSSGGALLDMFGNLLGLISAGYDDGQNLNLAVESSLVKIFANNFIEIVN*

>CD630_16490 Clostridioides_difficile_630_NC_009089 iron family ABC transporter ATP-binding protein

MIEIKNIFKRYKNKNVVDDVSFSIEKGKITSFIGPNGAGKSTVLSIVTRLIGGDGGEVIIEGKSLTNYSNKELAKKIAILKQSNNITLKLTIRELVGFGRFPYSEGNLTKEDENYIDEAIEYMKLTDIQHKYLDELSGGQRQRAYIAMVIAQDTEYILLDEPLNNLDMNHSVQMMKVLRSLCDELDKTIVLVMHDINFASCYSDNIVALKNGKVEKVGRTDEIVNEKVLEDIYEMNFNIKNINGNRICIYF*

>CD630_16550 Clostridioides_difficile_630_NC_009089 Na+/H+ antiporter NhaC-like portein

MRNKKINIIFLTTIMFIMSTVMVFAEEDIDTIALANAEKFGILTLIPPLVAIILAFITKNVIISLLIGILSGSFIIKASGINVFATFIQAFLDLVDRALVSLADPWNAGIILQVLAIGGVINLVAKMGGAKAIAEALAKRAKSAKGTQLITWFLGLLVFFDDYANSLIVGPMMRPVADKMKISREKLAFIIDATAAPVAGLAIISTWIGLEVGLIHDAFESISIDVDAFGIFLNTIPFRFYNILILAFIVISALLLKEFGPMRKAEIKSRSRKISIDLDEGVEELDDLAPKNGVKLSVWNAIIPIGTLIIVALASFYYSGYTSIMGGDDKALIQLFTNSPYSFEAIKEAFSASDASRALFQSALVASLVAIIMAVVKKIFTISEAIDVWIDGMKSLVITGVILILAWSLSSVIKELGTAKFLIHLLSGSLPPFLLPSLIFGLGAIISFATGTAYGTMGILMPLAIPLAYSLNPDMSYVIVSTSAVLTGAIFGDHCSPISDTTILSSMGAGCNHIDHVNTQMPYAIFTAVITIVFGYIPAGLGLPIYIVLPVAIAAIFVGIQIIGKKVDEAEIELVE*

>CD630_16640 Clostridioides_difficile_630_NC_009089 transcriptional regulator

MSINTVIAKNLNRLRNERNLSLGQLAELSGVSKVMLSQIEKGDSNPTVNTIWKIASGLNVPYTAILEQPQNETFIVSKTDIDVQVSENKDYRLYCYYPNTPTRNFELFQMELEEGHSYTSVGHSEKSQEYIMIIEGQLKLEVNDSIYQLRENDSICFSAESIHTYHNQGEKTLKAVIINYYPV*

>CD630_16720 Clostridioides_difficile_630_NC_009089 two-component sensor histidine kinase

MKWKITRNFIFTIVFVAISVVIINIISILYVISTNSFFKVVDSGNNPEEFARSFEKDLYEKDGEFKLSKIGAEKLEKSNSWIQVLNDLGEEVYGVNVPKDTPKKYTPFQMVNNYKYIETKYVNFVLEKHLNKKHLNIIVGIPSRDISRIILTYSQNNIKKTLNKVIIITLVIDSVVALGVGYLFSRKLTKPISSVLWSIETMANGNYSLYLKDRGIYEEVFKNINMLADTLRVNEVERKENEELREEWLANITHDIKTPLASIQGYAEIINDKDYEFEEDEIQEYTEIIYNKSKYIKDLVDDLNLSTRLKNNTIVLDKKKINLVSLVRNIIIDILNDNRYKNRNIEFESNEDLIEVYIDSILFRRAITNLIFNSIVHNSEGTLISVEIVKKDNIEIIIKDNGIGISKSDLKHIFKKYYRGTNTGEMHKGSGLGMAISKEIIEIHKGKIYVSSEIGIGTKIIIEIKQN*

>CD630_16900 Clostridioides_difficile_630_NC_009089 thioredoxin

MAKVINTSEFRSSVEGSKGIVVVDFFATWCGPCNMLGPVFAELGEEMKDKARFVKVDIDESLEIAQQFNVSTVPTMIIFKDGKPVETLIGFMPKNKIEMQVKSYL*

>CD630_17000 Clostridioides_difficile_630_NC_009089 riboflavin biosynthesis bifunctional diaminohydroxyphosphoribosylaminopyrimidine deaminase /5-amino-6-(5-phosphoribosylamino)uracil reductase

VNFVNQKEKDIYYMKKAIELAKNGEGFVNPNPLVGCVIVKDSNIIGKGYHEKFGSNHAEVNAINSAKQSLKDSTLYVNLEPCSHYGKTPPCVDKIIQNKIKRVVISTLDPNPLVCGNGVKKLKDNNIDVTVGILEDEARDLNEAFFYHIKNKRPLCIVKSAVSLDGKIATKSLESKWISNESSRYLTHKYRNKYQSIMVGINTVLNDNPLLTCRLNQEKVSHPTRIVIDTHLKLPLNSNLVKDKTSKTIVFTCCKESIKLSMLKENNVETIISPSKNNLVDLEFVMYKLGELNIDSVLVEGGATLNDSLFRNKLVDKVKLFLSPKIIGGKDAPTFVSGEGINHLSDSTQLTINNVTLIDGDILIESDVLN*

>CDIF1296T_01789 Clostridioides_difficile_ATCC_9689__DSM_1296_strain_DSM1296_CP011968 thiamine biosynthesis protein ThiC

MLGEICLSVYTIIRVLSKRGKALFLFSKTITLILEDCNMNYTTQMDAARKGIITKEMEIVSQKEQVDVNELRELIANGQVVIPANKNHKSLSAEGVGKNLRTKINVNLGISRDCKDIEKELEKVRVAIDMKAEAIMDLSNYGKTREFREKVVEMSPAMIGSVPMYDAVGYLEKELKDITEEEFLNVIRQHAIDGVDFITIHAGLTRSVCQKIKNHERLTHIVSRGGSLLFAWMELNNKENPIYTNFDKILDICEEYDVTLSLGDACRPGCIKDSTDGVQIQELVVLGELTKRAWERNVQVMIEGPGHMAIDEIEANVVLEKRLCHGAPFYVLGPLVTDIAPGYDHITSAIGGALACAKGVDFLCYVTPAEHLRLPNLDDMKEGIIAAKIAAHAGDIAKNVKGAREWDNKMSKARADLDWCEMFRLAIDPEKAKRYRDESTPTHEDSCTMCGKMCSMRTVKKILNNEELNLI*

>CDIF1296T_01797 Clostridioides_difficile_ATCC_9689__DSM_1296_strain_DSM1296_CP011968 molybdopterin-guanine biosynthesis protein

VALMRHFSNIYELSKIILEEAICIEKTGVILAGGRNSRMGRDKAFLELHDKLFIEIAIEAFKNFDELIIISNNEELYSKYDIKVYNDIVKDVGPIGGIYTALEYAKYDIVTIACDMPYLNNQIVERIANKMNDKSVISVTNGKLQPLCSGYKKSIINKVSLCIRENDLKLRSFIDKIDKSYIYFDEEDLFLNVNTVDEYEKLTKV*

>CD630_17120 Clostridioides_difficile_630_NC_009089 molybdenum cofactor biosynthesis protein MoaB

MFNVAIITLSDKGYEGKREDITGKKLTEFVENTGAYKVTEYVLIKDDKEMLKENIIRLCNSNKIDLILTNGGTGFSKRDITPEATKEVLEKEIPGLSEYMRMKSTEITKKAILSRGVSGIRNNSIIINLPGSPKGAVENLSFIIDVLDHGIEVLRGEATECATKKSE*

>CD630_17230 Clostridioides_difficile_630_NC_009089 acyltransferase

MNNYLKFATYQLIGFTSSIPRLIKIKKNPDKFSLKEKFEFMQKQAKKSLDIVNIELNIIGKETLPKEPLLFVVNHSSMLDSFILTASVERPIGCVIADEPVWRNIPIFKEWAKLLRCVYVNRKNNREGIKSIAQASQNILTGQSMAVFPEGDLTWIKEPNSLVSEFRSGALKIAYKAKCPIVPLVIKNSKDTYEGYQPIGKINSVPVEVEFLEPIYDHIENPRLKSSVLGENIKNKMINTIENFRKSNKTFKEF*

>CD630_17300 Clostridioides_difficile_630_NC_009089 ATP-binding protein

MADCNSCPSKGNCNSQSNCSIENNPNNKFGKIIGVMSGKGGVGKSTVTALLANKLNKMGYKVGILDSDITGPSIPRLMGVKNVKAYSDGSYIYPVENSNNIKVMSINLMIDDENEPVVWRGPLLGGVVKQFYTDVLWEELDYLLIDMPPGTGDVALTVMQSIPISGIVMVSVPQDLVSMIVSKAVNMAKKMNINVLGVIENMSYIQCPDCSKKIKLFEGESTEKFLDDLDLELLGELPMTKEIIDITHNGVTEISDDLDSILTNVVEKIK*

>CD630_17310 Clostridioides_difficile_630_NC_009089 acetyltransferase

MELRPRSAKLEEFDKVIELINYVFRISRSHKPTMMEEFPLLLSKNNIENMIIISEDDKVVSDVNYLIQDVSIQGNRLKVAAIGGVCTHPDYEKRGYSSKILDKVEEKMFYDGVDIVIISGTRSLYSRRNCSLVKSFYKYTIKPEDVKIAYEIVEFDETNFEKDNDLDKMIELYNQNSTRFIRTRDEFQKLLHAATIAWGPIGYKKVFIKENNNIIGYLIIRTIKKEDSTVGEVAEIGLNSVNVENILKYVANKFGLEYLNYKVHVKNLKDQLKCNGTKSLDYQQGTMKIINFTKLCDSLRSYFSQYVDFELLKYMEFKQVENKYIIKYKEEELVIENLDKLNKLFFEKNEEQYNEFKHLKNIYEFATKAFPVDFPWTANLNYQ*

>CD630_17320 Clostridioides_difficile_630_NC_009089 cobalamin-binding protein

MDILDDIAECILNMGVDNIENLVKIAIDKNIDVEDIYEYGLNKGMIGALDKFENKEYYLSEVIVCTDALNKGINLLKGTGKVKKKSKGVILMSVVEGDTHEIGKNIVKVMVEATGYKVIDLGVNRKSEDIIEEAIKNNVDIIGLSSMMTTTMENMKSVIDELNMIEIDKRPKVIIGGGPVSMEFAEEIGADGYSSNAPKAVKLINKLIGGEV*

>CD630_17530 Clostridioides_difficile_630_NC_009089 multidrug family ABC transporter ATP-binding protein

MIKVDDLSFSYTDRDFLQNINFEVGKGEILGFLGPSGAGKSTLQKILIGMITNYGGSVIVNGVESKRHSNKFYENIGVDFEFPSLYEKLTAIENLKYFGSLYSKKLLSIDELLKSVGLENESNKRVSEYSKGMKSRLNFIKALLHNPDILFLDEPTSGLDPSNSKVMKDIILSEKSKGKTIILTTHNMLDATELCDRVAFIVNGKISALDTPHNLIMSKGAIKVRYTYFDNGEKTSECFLNNTANDKNLNMLIEKNKLLSIHSSEPTLNDIFIEITGRNLQ*

>CD630_17550 Clostridioides_difficile_630_NC_009089 multidrug family ABC transporter permease

MIKIINAFKQEFEQIKRDAMLFIVCVSPILCGVFIKFGIPLIQNISLNKFYYQLNLEPYFLMFDLLLAFITPFMFFFASTMVILGEIDDSISRYLIVTPLGKTGYLISRFGIPGILAFIITMILLIFFSLTKISLLLNLSISLLVLLQGVIISILVISLSSNKLEGMVITKFSGVFMMGILAPFFILNKAQYILFFLPTFWISKAFKEDNYVYMFISIVISLIWILLLFKKFSKKIVK*

>EAA_RS0218165 Clostridioides_difficile_CIP_107932_NZ_CM000659 hypothetical protein

MKRILSSTIQVFKQIKSDPMMFAACFTPFVMGALIKFGIPFLDGITDFSLQTYYPIFDLLLSIMAPVLLCFAFAMITLEEIDDKVSRYFSITPLGKSGYLFTRLGVPSIISAVIAFIVLLLFSLEKLSIGMTICLALLGSVQAIIVSLMIITLSSNKLEGMAVTKLAALTLLGIPAPFFIDSYYQFAVGFLPSFWVAKAMQNEAVLYFSIGLMVALVWYYFLIKRLFRKLAG*

>CD630_17720 Clostridioides_difficile_630_NC_009089 hypothetical protein

LKNLNLLINKLYSKNHNEAYKTFLFLENESLKSNITYCFFDSFLEMIDNENSYIRTRGLLLISANAQWDIDNKIEINIDSILSHIVDKKPSVSRMFIKSIPNITKYKENLINRIKMELSNADISIYNNNMKPLVEKDINDTLSNIS*

>CD630_17730 Clostridioides_difficile_630_NC_009089 hypothetical protein

MKEIADAITSEYLSYGINEDISIYEGRFCIYLDKKYRCNGKIYYKMTPPISISFKADIGCIEEIDNEDDNLALDYDNAILEVHGYKIISITINTLSEFSVEGYINDDCIKSKNSYVEYVDFNIINLDKIPGKLIKYNDKVYAGRIEFDINDYVVTIDKRYDYRKELKSELKSKSGAIITHIGRIRRKDGRIFRTNNTINLLDRISTALSFMCGRYVGFCLAKGYRSGNEVYRIWNENQISPFRYVPTWSDTLSNYHNMEKYISLMCKKLEDFYYGSAIKSVVDWYIESLGSATMENNIISVQIALETLSYVILVEQNKILTDEVFDCNLASKNIRLLLDTCKIPYGKHELNIFDNIIKNKFDDGVDLVIYLRNSIVHPSRKTHRAVLEVEDIWNIISIGTRYIELVLLFILGYRGEYSNRLVERCYGEVEVVPWN*

>CD630_17740 Clostridioides_difficile_630_NC_009089 amino acid family ABC transporter substrate-binding protein

MKNILKKVGIFTIMLGLLGGVVGCSKPDNEKDKDASKESKKEVVVGFDNTFVPMGFLDEKGDTVGFDVDLAKETFKRLGMEVKFQPIDWSMKETELNDSKTVDVLWNGYSITDERKKIVSYTEPYLQNKQIIVTLSDSKINSKADLKDKEVGTQQGSTALDAVEKDKDFMNSLKGGAPVLYDTYDKALRDLEIGRTSAVVGDEVLIRYYMGQKGEDKYKVLKDDFGLEDYVVATSKENPELCEKINETLKEMKKDGTFDKIYDKWFK*

>CD630_17760 Clostridioides_difficile_630_NC_009089 amino acid family ABC transporter ATP-binding protein

MLKIKNLNKSFKKNRVLKDISFELEEGQIGVLLGKSGAGKTTILRCINGLEEFDSGEIIIDNEVIKNKRDMAKIRGKIGMVFQNFNLFPHMTVLENIIESPVNVFKVPRKEAEERARELLRLVDLEDKLNSYPFELSGGQQQRVAIARSCALMPKVLCFDEPTSALDIDTIQRVVNIMNRLKDKGMTILIITHDVVFSNNVADKIISIKDGIVENVQIKEKIV*

>CD630_17800 Clostridioides_difficile_630_NC_009089 methylase

MKNYTLISPCFFGMEKMLAREITNLGYEIIKTEDGRITYKTDEFGIAKSNMWLRCAERVHLKIAEFEAKSFDELFENTKRINWSRYIPYGAQFPISKASSIKSKLYSTPDVQAIVKKAIVESLKKSYLEDGLLKEDKEKYPIFVFIHKDKVTISIDTTGDALHKRGYREKANKAPIRETLAAGLIYLTPWKAGRVLVDPMCGSGTILIEAAMIGINMAPGLNREFISEKWRTLDKKIWWDVRKDAFNKIDNESKFKIYGYDIDEESIDIARENAEIAGVDEYIEFNVGDATQFKSEDEFGFIITNPPYGERLEDKDSVKQLYKELGYAFRKLKNWSYYLITSYEDFEYEFGQKADKKRKLYNGMLKTNFFQYPGPKPPRNNK*

>PCZ31_RS13230 Peptoclostridium_difficile_strain_Z31_NZ_CP013196 two-component sensor histidine kinase

IVLHFEGLRKKVIKNYFIIIIIMVTLFEGLFMFYIQNYYYDSVKQLLESEIKYADEYNAITMETTSFEKKVKNIFDKQPLTKNSEFGISIIDKDKNIILDQYGFKSKEKANYEDVNNALKDIKTKNLTPYTYRIPDTGEHVMSISLPLKVNNIIEGVVRYTVSLDAIDNAILKQATWLILAGIFILIIAILISLKFAETLIKPLRELKKFANELAVGNYNIKLEKMKIVDDEIGDLAQTFEHMAHEIDKSEKLKEEFISSVSHELRTPLTSIKGWSETLGYESITREELDLGLGIIQDETERLIKLVEELLDFSRLSSDRIKLHVDIVDVEGLIVGVVNQLKVKAAEKDISLLFEFENEFIENIQGDKNRLRQVLINLIQNSFKFTSQGGYIKVVASQDEEITTISVEDNGSGIEKQNLNKVLDKFFQEDYNKAGSGLGLAISNEIVKLHGGRMKIESEKNVGTKITFNIKNKFAKQA*

>CD630_17880 Clostridioides_difficile_630_NC_009089 hypothetical protein

LKSTTFVILWTILFVLFGFYVNNKLYDFTEGYKDNISVLEKSIENEDWEKAQKEADSISTSWNKEKNHWYKVLNHEYFDEIGLRFNILDKAIYTENKLKSLEEVESIKTYLGNIIESVKFDVNYIF*

>CDM120_RS09425 Clostridioides_difficile_M120_NC_017174 hydrolase

MAKLTLEHIKGNTYYIQLPTIVGVYVDGKDAILIDSGNNKDTARQVLRLLEEHNLIPKLIINTHSNADHIGGNAYLKNQTKCKIATTKIEGYFTENPILESAFLYGGYPSKALKNKFLLAKESEVDYIIPSNGKIVDTELEAISLPGHYFEMIGVKTPDNVLFVADSLTPENIITKYHFFFLLDIDSQFKTLEKLRMLEADFFVPSHSVKTTDIKNLIDINKKKMEEIIDNIKKVCCEPVMIDKVIEKMCDLYNVKLDANQYVLVGSTIRSYITYLYENNMVEYIFDGGKMMIKVLQ*

>CD630_18050 Clostridioides_difficile_630_NC_009089 sucrose-6-phosphate hydrolase

MYKEPKYRTILESTKEELNKLRNISLNDKYKPLFHIHPQHGLLNDPNGLAYYNGKYHVFYQWYPYDATHGMKHWAYVSSDDFVNWNREDVALIPIESYESHGAYSGNAIEVDGKLHMYYTGNIKYSAEDRYAYQNLAIMNKDGKITKYENNPIVSEIPKGYTGHVRDPKVFKRKDKYFMLLGAQTSDKKGVIIVYESKNSIDWNFKGELNVKNIDEDFGYMWECPDYINIDEKDILIFSPQGVEPKGFDYQNIYNVVYAIGNMDLDNLTFEIDTMKELEKGFDFYAPQTFIKDSQIILFAWAGMGEVLYPTDKNKWAHCLTVPRKLNIKNNKLLQMPVDELIKLRYDETSGQNTIKNNINIIENNENLYELNINIKNIDSNKFGLELFSSQDEGVKLEFNKLGNIVTLDRSNFKKVFGVEYGTNRKEYINIDENTNIKVLADRSILEIFINDGEVVFTSRIFAKENSNQIRVYSDKIVEYEYTKFKLKQGIEL*

>CD630_18060 Clostridioides_difficile_630_NC_009089 fructokinase

MKKVISIGEALIDFIPNQNGGKLKNVSEFRRVAGGAPANVSAVVAKLGGKSSFISKLGKDAFGDYIIDVLNEVNVNTDYVLRTSKANTGLAFVSLKEDGNRDFSFYRNPSADMLLEADEVKKEWFNNCHILHFCSVDLIDSPMKLAHKKAIEYALESNSIISFDPNIRLPLWDSEQSCKKAISEFLPFAHIVKISDEELEFITGENDIEKSLHKLFVGNVELVLYTKGKDGVDAYTKKVKGMCKGVKVNAIDTTGAGDSYIGSFLYTLLYKQITLNDIKEMKQETLNEYLEFSNYYAARSTTIKGAISSYATKEEITEFIKNL*

>CD630_18070 Clostridioides_difficile_630_NC_009089 NADPH-dependent FMN reductase

MEVCILMGSPKKKGNTAAILKPFIEELENYNSNIEVIWLYDCKIEPCIACKKCQDNLFDFGCYCKDDVQKIFKKILMCDLIVFATPIYSWYCTSPMKALLDRLVYGMNKYYGNEKGPALWAGKPVALITTCGYRPEKGADIWENGMKRYCKHSQLKYIGMLVERDLGKPNFMNKEKEEHSRQFAKQIYYILDNSIEVDNC*

>CD630_18140 Clostridioides_difficile_630_NC_009089 phosphoglycerate mutase

MGNTFYIVRHGQTDWNILGKTQGHGNSDLTPQGIEQAKELSEDIGKYSIDYIFSSDLGRAMQTAQILGDKLNIEVQKTEALREMGFGVWEGLLIKEIQKDYSDIYATWRNEPHLVNIPEGETLKIIKERVDAFIKELNEKYDNKNIILVTHSITLRVMLLSFLESGMENIYRIKQDNTALNIVEFKDYGPVIVKMNDTSHIKNHVKINNSALE*

>CD630_18160 Clostridioides_difficile_630_NC_009089 cytidylate kinase

MGNLVIAVDGPAGAGKSTIAKIVAKKLNINYIDTGAMYRAVTYKCLKSGIDVNNEKEVIQIAENSDIDFKDNNIYLDKEVINEEIRTIEVSNNVSNVAKIKEVRQLMVEVQRKIGMKNSVILDGRDIGSYVFPDADYKFFLVATPEERGNRRYKELCNKGYNTTLEEVIEDIIRRDEIDSNREFAPLVKANDALEIDTTGKTIEEVVEEVVSKINL*

>CD630_18170 Clostridioides_difficile_630_NC_009089 acyltransferase

VNFYRFVINIFKGFSKIFFKYEVIGAENIPDRGNIVIASNHKSNLDPIFLAAAIENREIAAIAKKELFKVKPLGFILKKLHVMPINREKPDVSTIKTILRSVRDGYVLGIFPEGTRIKGDSFGKAKAGLSVFTIKSKSKVVPVSIISKYKLFSKVIVYIGEPISFEEHFKEKLSNDDHERISQEILEVIKQNYFKYSK*

>CD630_18220 Clostridioides_difficile_630_NC_009089 thiol peroxidase

MLSIGTKAPEFTLEDKDGNKVSMSDFKGKKVVVYFYPKDNTPGCTRQACAFRNAYDGFKKEDIQVIGISKDSIKSHQKFAEKHELPFILLSDPDLVAIKAFDVWKEKKMYGKTALGVVRATYIIDENGIIEKVFEKAKPDTNAQEILEYLEKQE*

>QAE_RS0209090 Clostridioides_difficile_QCD_23m63_NZ_CM000660 cation transporter

MYILNLNTRETIEDFRDKFYVAENSYLILSAPKNLKLLKETLDIDEITFNDCLKFDEITKLDLFDNYDFLSLNTFELRDGEAVIEEVNMYLSDNFILVVVNEEHFLFEFVKNIILKNSQLEKNPVINLFKINYLILREVIKNGFESLEKVEELILQIEDEMMDNINKNHVSRISDVRGLTRIIVKNTRPLLYIGDRIVKENIRYLKYSNVKKYNLENFQGIDFGIDKLYSFALSTRELADKLLDIYSSRVGEKTNNLITKLTLLTAISAPLTIITGIYGMNFRYMPELNWIYGYPATLFFMLCIIFVGIIIFKIKNYCKLQMIFN*

>CD630_18350 Clostridioides_difficile_630_NC_009089 chorismate synthase

MSGIWGNNLKVSIFGESHGNAIGINIDGLPSGIELDLDKIDKEMKRRAPGKNSISTSRNESDIPEILSGYFNGRTTGTPLCAIIRNSDTRSKDYGELKNLMRPGHADFTGNVRYSGFNDYRGGGHFSGRITAPLVFCGAICKQILSQKGIEIGAHIKKIKNIEDMSFDYVNISKQQLSNLQTLELPLLDLSKEEAMKNTIIDAKNQGDSVGGIIECAVVGINVGLGNPFFDSVESTLSHLLFSVPAVKGVEFGLGFELADMYGSQSNDEMYYEGNQVKSKTNNNGGIIGGITTGMPIIFKVAIKPTPSISRQQNTVNIKDKKDDILYIKGRHDPCIVQRAIPVIEAVTAIGIFDLMKGR*

>CD630_18380 Clostridioides_difficile_630_NC_009089 shikimate kinase

MINKTKEKLILIGMPGSGKTTIGKLLAKEYNCSFCDMDDYIIQISQKSIAELFSEGEDIFRNYETQACRELSISDKTVISTGGGVIKKDVNMEILKETGIIIFIDRPIQKILEDININSRPLLKNGKDRLYNLYNERINLYKKFSDIEILNDKSLNNAVYNITNAVSENFKFDFKEK*

>CD630_18390 Clostridioides_difficile_630_NC_009089 prephenate dehydrogenase

MNIVIVGLGVIGGSFAKALKKAGYENVFGVDVDLETLKKAEKAKIIKKGCTTGKELFKKADLIILSIYPRLVVDFLNNNKNFFKKGTIITDTTGIKETLINDVLQIIPDDIDFIFGHPMAGREKKGIDFASEQVFNGANYIITPTGRNNIKNLELVENLILEIGFKRVKKLTSQKHDEIIAFTSQLPHVMAVALINSDEEGRDTGKFIGDSYRDLTRIANMNEDLWSELFLGNRDNLLKVIENFESEVNLVKEAIFNNDKNKLIEYFKKSSIRREALEK*

>CD630_18800 Clostridioides_difficile_630_NC_009089 hypothetical protein

MHENKKNLLDGSIAQYNDTAVPKIPVFAGNDITSEVYYFKPNQVLNAHRHPNGEQIFVFLKGEGKMKLGEHECDVKNGDTVFVPTGEWHEITNGSNEEMVAVQITKINAGAEYRG*

>CDM120_RS09650 Clostridioides_difficile_M120_NC_017174 SAM-dependent methyltransferase

MKQNKYDDDKFFNKYSKMERSINGLAGAGEWHTFRKMLPNFKGKRVLDLGCGFGWHCQHAVENGATSAVGIDISEKMLKEARNKTTFDNIKYICMPIEDINFPKDSFDVVISSLAFHYIQSFEDICKNISNCLSNKGDFVFSVEHPIFTAHGSQEWHCDKDGNNLHWPVDRYFEEGLRKSNFLGEEVIKYHKTLTTYLNTLIKTGFEVLEIIEPQPEEKLLDAIPGMKDELRRPMMLLVSAKKDK*

>CD630_18930 Clostridioides_difficile_630_NC_009089 oligonucleotide binding regulator

MNNQQQEVYKKLDELSIPYEAVNHPPVYTIEEMEELKTLHMEFVVKNLFLRDAKGKEHFLVVLGKDKKADLKDIRQQIDSKPLSFASEERLQKYLKLTKGAVTPFGILNDKEAVVKVVFDKDLLKMDKIGIHPNDNTATVFLKFEDMKKLIEENGNEIYYVEV*

>CD630_18990 Clostridioides_difficile_630_NC_009089 dCMP deaminase

LNKKLENRCSWQEYFMRLCETVAERGTCDRAYVGAIIVNSENRIVSTGYNGSISGDKHCSEVGHEMRDGHCIRTIHAEQNALYYCAKEGISVKDCSIYVTHFPCLNCTKAIIQAGIKHIYYRTGYRIDEYAIKLLQSSNVLYTKL*

>PCZ31_RS12630 Peptoclostridium_difficile_strain_Z31_NZ_CP013196 amino acid-binding protein

LEEIYIMKLKLLVEEYAVCRLNNDSKIPTWIDTEKFYSITRTDDELSVVCLNNNIPSDVKSEKEWRILKILGPLDFSLVGILSKLSSLLADNKISIFAISTYDTDYILVKEKDIENACKILSCNGYEVE*

>CD630_19390 Clostridioides_difficile_630_NC_009089 acetyl-CoA carboxylase, biotin carboxyl carrier protein

MNINEIKELLKAIDSTNLEYVKLESSDLRLEVSKKAQSTSSPVLSVQQESVVDLSLEKPVVNDTPVTSNENLSVVVAPLMGTFYDSPSPDADSFVKVGDVVEEGDTLCILEAMKLMNEITSEIKGEIIEVLVSNEELVEYNQPLFKIKPL*

>CD630_19420 Clostridioides_difficile_630_NC_009089 transfer RNA (guanine-N(7)-)-methyltransferase

VRRRRKKGADEKLLSYTKYVLRDDIDKLKGKWNLKFRNDNPIHVEFGTGKGKFITTLAKQNPDINYIAMELKEEVLLKAVEKADASNLNNILFLWGDVSNILDYFEAKELSRIYINFCDPWPKNRWSKRRLTHSGFLEMYNRVLEDDGEIHFKTDNEKLFEFSLNEIAANNWLLKNISLDLGNSEYENNVTTEYEDKFMSQGMRIFRCEAKKRN*

>CD630_19470 Clostridioides_difficile_630_NC_009089 ABC transporter ATP-binding protein

MKITHNSLAVEAKNIIKEYKIGNTTTRVLKEVSLQVMKGEFVSIMGQSGSGKSTLLYILGGLDTPTSGKVYMNGADISHFNDEKMSIIRRRNIGFVFQFYNLIPNLNVEENIMLPLLLDGKNLKDYKNQLDEILDIVGLTDRRKHTPRELSGGQQQRVAIARALIGKPEILFADEPTGNLDSKTGIEIIDLLNKINRDNGQTIIMVTHSPEAAKSSSRTITVSDGLIV*

>CD630_19520 Clostridioides_difficile_630_NC_009089 LysR family transcriptional regulator

MNIKLELYKVFNAVVNNKSFSLAAKELFMSQPAVSQSIKQLEEQLDTLLFYRNNKGVKLTPEGKILSEHVTTALKLISSGEDRINKFKKLEYGSLKIGVGDTAARFFLLKYLEIFHKKYPHIHVSTINRTSRELISLLKDGNIDIAIINMPIEDDTLNIVECIEIHDIFVCANDYIEYKGRKISLEELNTLPLIMLENKANSRLYVNEYFLSKGIKLNPDIELGSHELLLEFAYINLGVSCVIEEFSIDYLENEKLFKLDIKEPIPKRNIGYCHLKDISLSLATKEFLSMISNNI*

>CD630_19550 Clostridioides_difficile_630_NC_009089 ABC transporter ATP-binding protein

MSILVTNNLVKHYGKGETKVKALNGVSIEIERDTFTAIVGTSGSGKSTLLNIIGGLDNPTSGDVIIKGKNISKIGKKDLTVFRRRNIGFIFQNYSLMPVLNTYDNIALPVTLDKGNHIDHEYIEMLMKTLGIWDKRLKFPSELSGGQQQRVAIARALANKPALILADEPTGNLDSKTTMEVVCLLKESSAKFHQTILMVTHNENIAQVCDSIIHIEDGVVVNTGGEVL*

>CD630_19630 Clostridioides_difficile_630_NC_009089 methyltransferase

METKQYLIDFYNTYDEDSRLALKHGMVEFLTTMHYIDKYIKSGDCVLEIGAATGRYSHTLARQGYDVDAVELVEHNIEVFHKNTQSNENTSITQGNAMDLSVFPDNKYDITLLLGPLYHLYNKEDKQQALHEAIRVTKPGGVVFAAYVISDGCLIDEGFHRGNIDVSEYIEKGLIDPQTFAAKSEPKDLFELVRKENIDDLMSAFNVTRLHYVASDGLALYMREAVDSMNDDAFALYLKYHLATCEREDLVGVTSHAIDIFRK*

>CD630_19730 Clostridioides_difficile_630_NC_009089 pyridoxal phosphate-dependent transferase

MLNETKELLKDYYGIDDDTFKLSQEIMEEIKDKFEEIKEIREYNQYKVLKAMQESKLSDMHFNWTTGYGYNDIGREKIEEIYSKVFNTEDALVRPIIVNGTHALTLCIQGIVRPGDEILSVTGRPYDTLEGVIGIREEKGSLKEYGVTYDDVDFLEDGNLDLEGIKSKINDRTKLVMIQRSKGYSWRKSLSISDIKEAIEVIKSVKPEAIVMVDNCYGEFLDTKEPTDVGADVMAGSLIKNPGGGLALTGGYIAGRKDLIELISYRMTSPGIGKECGLTFGTTRNVLQGFFLAPYIVSQAVMGAIFCSRAFEKLGYDVLPKYDDLRSDIIQCIRLNNADEVISFCEGIQEAAPVDSYVKPVPWDMPGYESEVIMAAGAFIQGSSIELSADAPIRPPYNVYFQGGLTFDHSKMGTLKAIEFIKKLKK*

>CD630_19760 Clostridioides_difficile_630_NC_009089 DNA mismatch repair protein MutL

MKNIINILDDLTINKIAAGEVVERPSSVVKELIENSIDAGANKISIDIIDGGKSLIKITDNGIGIPSSEVEKSFLRHATSKIKKIDDLYDLYSLGFRGEALASISAVSKLEMTTKTKDEIIGTKIYVEGGKIISKEPIGFTNGTTIIIKDIFFNTPARQKFLKSTHAETINISDLINKLAIGNPNIQFKYTNNNKQMLNTPGDGKLVNTIRSIYGKEITENIIDVEFKCNHFKMNGYIGNNNIYRSNKNLQHIYINKRFVKSKIIIDAITESYKSIIPIGKHAVCFLNIEVDPSCIDVNIHPNKLEIKFEKEQEVYIELRDFLKVKLIHSNLIGKYATYSDKKTQPRIAINSREKSTDYKLRNNDLLESTPKNSNITKGKDEVIEVVTLSSEKPINEFQSVSEVLNASVEDDVKNINYLSEDSANDNIQEEFQVDGIKNEGNYYLGDSIKDSEEEYLCSSKRKFSLYGYSVIGVVFNTYIILSKDDSMYLLDQHAAHERILYERYMEKFYRQDINMQILLDPVVIEVSNVDMLQIENNLELFMKFGFELEIFGNNHIMVRCVPTIFGVPETEKFILQIIDNIEEITSNYDLKGERFASMACRSAIKANDKIYDIEIKSLLEQLEKCENPFTCPHGRPIMVEISKTEIEKMFKRIM*

>CD630_19780 Clostridioides_difficile_630_NC_009089 ABC transporter ATP-binding protein

VMLTQMNFYHNKKVKVSIENVSKTYKDIEVLKDISIDVYEGEFVSILGPSGCGKSTIFNIITKLTDYDSGKVNINGKYSYMYQKDLLLPYKTIIDNVSLPLILKKEKKSKARSMVKPYFEVFGLSGYEDKYPSELSGGMRQRANFLRTFINSNDIMLLDEPFGALDSLTKTSMQKWLLDVKKKVNSTILLITHDIDEAIMLSNRIYVISKKPSIVKKEFVIDSRKLNEDNLENIIKLKKEIISLL*

>CD630_20190 Clostridioides_difficile_630_NC_009089 drug resistance transporter

MTNSFKKDDNRKKITVLFVVIAMTFMATLDSSIINVALPVLASKLNVSLASIEWVIASYSIIICSTLLFFGRLGDIIGKSRVFQVGTILFTSASLLCGLSNSLTLLIVCRFIQGIGASAYMANNHGIITELFPKESRGKALGILVTAVAIGNMVGPSVGGFILSIFDWNVIFFINIPIGLIVIFLNTKFLPNSKKSSENMDKTGAILQFLGTTLFFSALISAQQTGLLNPYILIALLLSIIFIILFLILEKKHPQPLLDLEIFRNFKFSLNLICALTSFICIASSSILIPFYLQSTMKLPPIQAGLFMILSPLILAIFSPIFGNISDKIKSEKIILIGLLVMSFGFFLMSRLKESSALILFVIYILIISIGQAIFQPANNALIMSSCSRSKLGVVGSINSLVRNLGQVIGITISTTLLYNFMSIKAGYRVNDYVINNDKIFVFGMRNVYIIVTLVCLIGAILIGFYLFKYNKNEQ*

>CD630_20200 Clostridioides_difficile_630_NC_009089 chaperone protein

MDVEKMTLRVQKSLNEAYNEAVKNHNQQVDVIHLFSALINQEDGLIPNIIEKMNISIDSLRNTVNFEIDKLPKVYGEGADSQGVSATRKINEVLIKAESISKEFKDSYISVEHVMLAMMETESKSAVGKILKQYNINKNDFLNILSQVRGSQRVETQDPEGTYDALARYGTNLVDLAKKNKLDPVIGRDEEIRRIIRILSRRTKNNPVLIGEPGVGKTAIVEGLAERIVRGDVPEGLKDKIVFSLDMGALIAGAKYRGEFEERLKAVLKEVQSSDGKIILFIDEIHTIVGAGKTEGSMDAGNLIKPMLARGELNCIGATTFDEYRKYIEKDKALERRFQPVIAEEPTVEDTISILRGLKERFEIHHGVRIHDNAIVAAAKLSHRYIQDRFLPDKAIDLIDEAGAMIRSEIDSLPTELDVVRRKLFTLETEREALLKENDDKSKSRLDDIQKEIAELKSKNDEMTAKYEKEKSQILDIKNLKAQLDEAKGKAEKYEREYDFNKAAEVKYGEIPKLEEQIKQYEENMNDGSENSLLKEEVTEEEISSIVSKWTGIPVTKLVEGEREKLLKLEDELHKRVIGQDEAVTAVSNAVIRARAGLKDERKPIGSFIFLGPTGVGKTELAKTLARNLFDSEDNIIRIDMSEYMEKHAVSRLVGPPPGYVGYEEGGQLTEAVRRAPYSVILFDEIEKAHEDVFNMFLQILDDGRLTDNKGKTVDFKNTLIIMTSNIGSNYLLEAGGNITETTNNLVMNEMKHRFKPEFLNRVDDIIMFKPLDQENIKKIIDIFMKDLKNRLKEKDITIEVTNSAKDVMVREGYDPVYGARPLKRYIGNTLETIIAKKLIAGDVYNGCTIVIDGKDENIEVLVK*

>CD630_20240 Clostridioides_difficile_630_NC_009089 multidrug family ABC transporter ATP-binding protein

MISMDAITIKNLNKTYKDFSLQDISFSVPKGSVMGFVGENGAGKTTTLKAILNLISYDSGNIEIFGLDSKKNEKEIKEQIGVVFEGSNFHENLNTDHVSKIMSKIYKNWNDTLFKDYLKKLRVPDNKLIKEFSKGNKMKLSIAVALSHKPKLLILDEATSSLDPIVREEILDIFLDFIQDEEHSIILSSHITSDLDKIADYITFIHKGKIVFSENKDELIDTMGVLKCKPSDFDNLSREDYSYYRKSQFGYEVLLKDKHRFISRHPNCIVDNTSIEEIMLFYVRGDK*

>CD630_20310 Clostridioides_difficile_630_NC_009089 acetylornithine aminotransferase

MNNDNTISKWNEYFIDTYNQPNFVIDYGEGSCFFDTNGNKYIDFTSGYGVSSLGYSNSNLKNALKEQVDKLLHTSNLYFNEPVLCSGEKIINSSGMAKVYFCNSGTEANETAFKIARKYSSDKYGNGRGTIISLKDSFHGRTMMSLMATGMDKYHKYFYPLPEGFKYVERNNIEDLKNNLDSTVCAIILEAIQGEGGVNVLEKDYVLEIVKICQEKDIVVIFDEVQCGIGRTGKLFGYEYFDVKPDIVTVAKGLGAGIPVGGVLVNKKLSKVLGKGDQGTTFGGNLLAMVAASVVLDEISKDGFYNEVLEKGNYIRKSIESFNNKVVLKTKGIGLMIGIETNIESSIIEEKARKKGLLILTAGKNVLRFLPPLTISYKEIDEALEILKDILLEIN*

>CD630_20480 Clostridioides_difficile_630_NC_009089 RpiR family transcriptional regulator

MEETNEMKDSKHLISNIQSQYTRLSKGQKLIAQYILNNYDKVAFMTACKLGETVGVSESTVVRFANALGYSGYPKLQAALQELIKNKLTTVQRVEMAHDYSDDFAILNKVLKSDIDNIRSTLEEIDERAFKEASNKLLRARKIYILGMRSSFVVAQYLGFYLDIILDNVHIIRMDMGDAFEQIVRINEEDVIVAISFPRYSKKSYQIVNYAKEKGAHVISLTDSLFAPVASLADNTLLVKSNMASFVDSLVPALSISNALAISVGMKEKEDIKQHFDDLEQIWKRYSVYE*

>CD630_20590 Clostridioides_difficile_630_NC_009089 glutamine--tRNA ligase

MSNETNSSNFIKNIIINDLETGKHDSIITRFPPEPNGYLHIGHAKSICLNFGLAKEFNGKANLRFDDTNPLKEDVEYVESIKEDVKWLGFDWNELNFASNYFDEMYKRALILIKKGKAYVCDLTQEEMREYRGTLTEPGKESPHRNRTIEENLDLFERMKNGEFKDGEKTLRAKIDMSSPNINLRDPIIYRISHSTHHNTGDKWCIYPMYAFAHPIEDAIEGITHSICTLEFEDQRPLYDWFVKECEMENIPRQIEFARLNINNTVMSKRKLKQLVDEGIVDGWDDPRVPTISGIRRKGYTAEALRNFCSEIGVSKVNSTVDSQMLDYFLRENLQPKAPLTMGVLRPLKLIITNYPEDKIEMLEIENNAKDESQGKRLVPFSRELYIEQDDFMEEPVKKYFRFFPGNEVRLKGAYFVKCTDVIKDENGNVVEIHGTYDPETKSGSGFTGRKVKSTIHWVDAKSAIPCEFRLFEPLILDDIPENEGKHFLEQINPNSLEILQGFVEPTQIKDAKPFDKFQFVRNGFFSIDNKYTTDEKFVFNRIVPLKSSFKPGK*

>CD630_20680 Clostridioides_difficile_630_NC_009089 ABC transporter ATP-binding protein

MLQVTGVGLRFGDKELFKDVNLKFTKGNCYGIIGANGAGKSTFLKILSGEIEPNTGSISITDKERMSVLKQDHFEYEEETVLNVVIRGHERLWNIMKEKDALYMKEDFNEEDGIKAAELEGEFAELDGWDAETNAEKILMGLGITKDMHYKQMKELVGGEKVKVLLAQSLFGKPEILLMDEPTNHLDFKSINWLNNFIMDLEESIVIIVSHDRHFLNQICTNIVDVDFGKIQMYVGNYDFWYESSQLALQLAKDQNKKTEEKIAQLKEFIARFSSNASKAKQATSRKKQLEKLEVEDIQPSRRRYPYVGFTPAREIGNEVLEVHNLTKTIDGVKVLDNVSFRLDRDDKVVFMGDEIATTALFNIVMGELEPDSGEYKWGVTTSQDYLPKNHNKFFDGVEYSLVDWLRQFSEEKSESFIRGFLGRMLFSGEEALKEAQVISGGEKVRCMLSKLMLSNANVLVLDDPTNHLDLESITSVNKGLEKFPGVLLFTSHDHEFISTIANRIIEITPNGIMDRKMDFDEYLESKEIQDQLAKMYGKDK*

>PCZ31_RS11780 Peptoclostridium_difficile_strain_Z31_NZ_CP013196 hypothetical protein

MVMQVQEKILELLYTINEDVVSIKNDMSEVKQEMTVMKEDMSEVKQEMTVMKEDMSEVKQEMTVMKEDMSEVKQEINVLKEDMSEVKQEMTVMKEDMSEVKQEITVMKEDMSEVKQEINVLKGNMSEVKQEMIVMKEDMSEVRQEINIMKEDMSEVRQEINTMKEDMSEVRQEMTVMKEDMSEVKQEINVLKEDMSEVRQEINIMKEDMSEVRQEMTVMKEDTSEVKQEINVMKKDMSEVRQEITVMKEDVSEVKSEVNFMQNKINNINEDMNGIKDEVSIANEKLYGIEIKIDSLESEDKSMKEIQIEQNNILGSLLHNSEINKATHDNIEHNIAYIKGDTNSIKEDIAEIRRDLNLVELATSKNWSDIVKLKSVK*

>CD630_20780 Clostridioides_difficile_630_NC_009089 S-adenosylhomocysteine deaminase

MIAIKSKYLISSADEIYTNSAVVVENDIIKDILPNEEVEIKYKNIEEIIDKSDAIIMPGFVNGHMHQYGVLSRGIPANVHFTDFEGFLNDYWWPFIENRIGLKEVKATTKASAIELIESGVVAFCDTLEAPNTEEGTLIEQGKILEEIGMKAILSLESCERISFENGLRCLDENSNLIKWSRENSKLINGIMCTHTSFTCSDRFIKKAKEDAKKLNAPMQFHLCESIYEPNYAEKHFGKKAVDYYNDLDILDETVLASQCVKVNDEEIDILKEKGVKVVHMPLSNCEVGGGFSPVPKMIKKGIKVALGTDGYINDFFEVMRGAFLMHKSVEEDASVMPANLVFRMATEHGAYVLGLQNSGKIAVGNKADIIVMEDEFKTPVTLDNIFDQIVVQGKKEFISNVYIDGRHILKEKQLVDLDKKAIVKEMKEVACEFWKF*

>CD630_20810 Clostridioides_difficile_630_NC_009089 xanthine dehydrogenase iron-sulfur binding subunit XdhC

LIIKCTINNKEKELSIRPDDFLADTLRENGYVSVKKGCDTGSCGLCTVMVNDKAVLSCNYLTARVEGQHITTLEGVLDEAKKFGDFLADEGADQCGFCAPGFIMMVLAMKRELQNPTEEEVIDYVNGNLCRCTGYKGQLRAIHKYLNT*

>CD630_20820 Clostridioides_difficile_630_NC_009089 amidohydrolase

MMDILIKNAIIVTVNKEREVIFDGALVVKDNKIADIGNSKEIESKYTDVKKIIDAKGKVLFPGFINTHNHLFQTLLKGLGDDMVLKDWLETMTFPAANYLEPKDTYDAAMLGCIEGLRSGITTMVDYMYPHSKPGLCDGIIDAYKELGIRGILGRGCMNTGAQFGVHPGIMQDVETVEKDVRRLFEKHHNTENGRIKIGVAPAAIWSNSQEMLEMLWRVVKEYDDALFTVHISETPFDREAAKELHGQYDIDVLEKLGILGPNVLMVHCVYLTEKDMELTKKYDMKVSHNTASNMYLSSGVAPVPEMLKKGITVSLGVDGAASNNSQDMLELMKLTALQHKVNKCDPLAMSAEKVLELATIDGARAIGMEDEIGSLEIGKKADLLIFNPMLSPKAIPMHNPVSTLVYSSSMKNIESVIVDGNIIMEDSKILTANEEKALKDAQDTAERLCVRGTIKNRMEGHKWNSLY*

>CD630_20830 Clostridioides_difficile_630_NC_009089 D-hydantoinase

MGTILRGGTIITSDKTYISDLRIEDEKIVEIGNNLEINGDKVIDATGKIVIPGGIDTHTHFDMNAGSITTADNFETGTRAAIAGGTTTILDFAEANEGENLLQGVEAYHKKASGNCYCDYGFHMTITCLNKDTFNHMEKLISDGIVSFKMYMAYDGMKVDDGTIYRVLKKARDLGCIVGFHCENGDLLDVLIEENIQNGNLEPKYHPLTRPNIVEKESVSRLADITKLSNSKSYVVHLSCRESLETVKMAREKNIDMIVETCPQYLLLEDNLYNKDKFEGAKYVMSPPLRKKEDINYLWEGLSSGDIQTVGTDHCSFNFKGQKDLGIDDFSKIPNGAPGVEHRLALLYTYGVLENRISANKFVEVTSTNAAKIFGMYPKKGEIAVGSDADIVILNINKEETISYKTQKQNVDYTPYEGFKVKCKVEDVFLRGNHVVQSCNVKEHPTGQYIKRKITK*

>CD630_20840 Clostridioides_difficile_630_NC_009089 peptidase

MLNEQRKQEVTEICQKLIQNPSSSGNEEGVVKAIEESFEKLGFDSWSRDRYGNIVGCIKGNKPGKKILFDGHIDTVPVPDASKWSVPPFEGKIVDGKIYGRGTSDMKGQYTAMMSAVAYFAKDTNRDFAGELYVAGVVHEEIFEGVSAREISKAVQPDYVVIGESSELNLKIGQRGRGEIVVETFGKPAHSANPEKGVNAVYKMANVIQKIQQLVPPTHPVLGDGILVLTDIKSSPYPGASVVPDYCKATFDRRLLVGETREGVLAPIQALLDEMMKEDPELNAKVSYAVEKADCYTGNTIESERFFPGWLYDEEDEFVQAAYKGLKEAGIDSEITQYSFCTNGSHYAGEAGIKTIGFGPSKENLAHTIDEYIEQEQLFIGTEGYYGILKSVYGK*

>CD630_20950 Clostridioides_difficile_630_NC_009089 cell division GTPase

MENFNKNAKIIGIGAEGINIINEVEEKIKANMDIEKININQEIEKEYVRSLLDGVDILFLIYSSEDKHIRDIIKAVSYMSNERRVLSIGMDCSEKENKEDLELGREFKINNDSIFKFVDLMNIMVESISDSCMINIDITDLKEAIVGDKGIKYSFEEFEDTKSYSEIADILFDRMEYIGEEFISKKGIVFVEGSSEFSIMELNDLISNIQSKVEESYEVIFSLYIKENLNGNIRVGLLYN*

>CD630_21020 Clostridioides_difficile_630_NC_009089 Na(+)/H(+) antiporter

MEASIHTIASNNLLIIFAIVSITGIICSKLSEIIKVPDVVLYLLVGILIGPSFLKFIDIRGFQIENQLILTFGSAFILYLGGKEISLKVLRNVKISVFLLATLGVVISAFIMQQVIGFTFGISAMTALLAGTIIASTDPATLVPIFNQVKIKDRVKQTVISESAFNDATGAILTSAVLAVILSNKFSLGENIYELGTMVIVGVLVGLITGILLLKLVNDKPYGIFKDFAPIISVISVVVAYELSTKLGGSGYMSCFIVGIITGNKKNFKIWLSQKSYDADFYVAETLGTICRMAIFIILGSQVNLAQLSKYFMPSVITVLVLMFIARPLCVLVCTLVDREAKWSKSEILFMMWVRETGVIPAALCGIISAMKVPGYEVISSVVFMTILITLVIQGSTTKLVAKKLGLLEEEVINISEKVSTF*

>CD630_21050 Clostridioides_difficile_630_NC_009089 multidrug family ABC transporter ATP-binding protein

MLKVINVSKSYKNKKVVSSVSFDVNDGELFGFIGHNGAGKTTTIKAIVGIHDFEEGEVLINSKSIKKQPVECKKEMAYIPDNPDLYESLTGIQYLNFIADIFEVAKDKREELIKYYSNKFELNKALGDLISSYSHGMKQKLAIISALIHSPKILILDEPFVGLDPKASFILKEIMREFCDKGGCIFFSTHVLEVAEKICDKIAIIKDGNIIAYGTTEEVKGSNSLESIFMELIEK*

>CD630_21060 Clostridioides_difficile_630_NC_009089 ATP-dependent RNA helicase

MNTFEQLKISSTLIDGLKKQDITSPTEVQSLVIGNIIQNKDLLINSQTGTGKTLAYLLPIFEKIDTSKRETQALILAPTHELVMQITNQVELLAKNAELSVTSLALIGEVNIQKQIKNIKAVKPHIVIGSCGRVLDLIKQKKLKSHNIKTIVLDEVDNLLNGKNITCIEDIIRTTLRDRQIIGCSASLTDSTIKICDKLMKEFEIIKTKEKSQINPNINHSYLLGEIRDKFTFLRKALAATNPKKAIVFVNNEKNIEVLVSKLNYHNYKAIGIFGNMEKEDRKNAINKFKLGKAKILITTDLSARGLDIVDVSHVFNLDFPKSKNEYLHRCGRTARGNRSGNTISIITKKELDIIKDLQKEFNIVITPKTLQNGELIDIIK*

>CD630_21160 Clostridioides_difficile_630_NC_009089 GTP-binding protein BipA

MSQKHKIINIAVIAHVDAGKSTLVDAFLSQSGVFRKNEVVKDCVMDSNDLEKERGITIYSKNCAINYEDYKINIVDTPGHSDFSSEVERVMKTVDTVILLVDASEGPMPQTRFVLQKSLEFGLKPILFINKIDKKDQRAEEVVNEVFDLFVDLNATDEQCEFPIIYGIAKQGIAKLEMDDDSEDLSPLFKTIVNHVEAYPNYDNEPLQFQISALAYDDYVGRLGIGRIYKGTLKNNTQVAICREDSVVSKGKVSKLSVYEGLKQVEVDEATSGEIVVIAGIPDISIGETICDLDSPLPMEMIKIEEPTLSMNFLVNDSPFVGKSGKFVTTRHLKDRLEKELEVNVGLKVEPLDTTDGYKVSGRGELHLSILLENMRREGYEVGVSKPEVLMHKEDGKLMEPIERVVVNCPEVYSGTIINELNMRKGMMESMSIEGDYVKIEFLAPTRGLLGYRSEFINATRGEGTLVRSFEKFEEFKGEIPSRGNGVLIAQGPGVTMGYSLNALSDRAVMFVDPGVEVYEGMIIGMNSRKDDMVVNPCKNKKMSNVRASGSDDAIKLSPPRIFTLEEALEFIEDDELVEITPDSIRLRKRFLNEHDRLRYNKSRQGK*

>CD630_21170 Clostridioides_difficile_630_NC_009089 thioredoxin reductase

MVDIIVIGAGPAGLTSAIYAMRAGLSVTVFEKNIYGGQVASTSEVENYPAVQKISGVEFSNNIYNQAVAQGVDIQFDEVEEINLEGKVKVVKTSSGEHKAKAVILANGVERRKLGCAGEQEFTGRGVSYCATCDGAFFKDKEVAIVGGGNTALEDALFLANNCTKVYLIHRRDSFRGEEVLEKSVKARENIEILYSHGVEKIEGEKTVSKIEVKNLKTEEKRTIDVSGIFIAIGLKPNNKMFENVLDLDEGGYIISDESCTTSVEGVYVAGDSRTKFLRQIITAASDGAIAAVQAANYINVE*

>CD630_21190 Clostridioides_difficile_630_NC_009089 homoserine kinase

MLEIIVPATSANIGPGFDCLGIALNIYNKFYVEEIESGLEIEGCEDAYKNENNLVYTSMKYFFDRVKPEKIPAGIKIKIQSEVPICRGLGSSASCIVAGVIAANALSGANLDKNQLLNIASEIEGHPDNVAPAILGNMIVSVTDNENIHYDIIKIPEELKFCAMIPNFKLSTEKARGVLPKEIPYSDGVFNVSRVALLISALLNKNFDLLKVACQDKLHQDYRGTLIENYNDIVEKSEQLNSIGVFLSGAGPTIMSLIKENDDSFVDNMKNYLQKLKSDWEIKELCCDSNGAVLNII*

>CD630_21250 Clostridioides_difficile_630_NC_009089 acetyltransferase

MSKPIDITIRPIRREDAQAFNEIRRTYSVMRNTLALMSDRVDKTEAMLASLGENDYMFVAETNNNGEKDVIGFAGLHVNSSPRLRHSAEIGISVAENYQGRGVGKKLMEQLLDIADNWIMLTRVGLEVIVDNEKGLNLYKKLGFEIEGTKKYAVIRDGKFEDVYIMGRYNKNLI*

>CD630_21290 Clostridioides_difficile_630_NC_009089 membrane-associated peptidase

MTIIAALILFSIIVLIHELGHFIFAKRSGIKVNEFSIGMGPKIYSVKKDTEYSIRALPIGGYVSMEGEDEEQISPNSFGNKSILQRFSTIVAGPIFNIILAAILLVPVFLYIGSPTTKLGKIMPDTPAQAVGLQVGDKINKINGNSVKTWDEVANIINTSSGGELKLSITRDGSDKVVNVTPKNNNGKYEIGIQPQREKDFLGSIVNACKTTVDMTKQMLTFLGQMITGRVPGGIGNAVAGPVGVIGMVSDAARTGIINVVYLAAVISLNLGIVNLLPIPALDGWRILMLLLEAVRGGKKLDPNKEGMINVVGFGALMLFMLFITYKDILRLFQ*

>CD630_21350 Clostridioides_difficile_630_NC_009089 phosphatidate cytidylyltransferase

MLTRIIASLALVPLFLFVVYGGIPLYIAETAIVYIALHEFYKAFKIKDVHPIFIIGYLFSIYLAVKNIFNLPLEYTYAVIFILFLASIIYMLMGKNNVIDVSITFLGVFYIGVFLDFIIITINGFEKGSIYVWLIFVISFMTDIFAYFSGYLLGKHKLIPKVSPKKTIEGAIGGIIGSTLCCILFGYLFGIDLLQLAIIGSIGSVIAQLGDLFASSIKRYVGIKDYGKIIPGHGGILDRFDSVILVAPFVYSAIKFFIR*

>CD630_21360 Clostridioides_difficile_630_NC_009089 undecaprenyl pyrophosphate synthetase

MNNNIIYDIDLNNIPTHIAIIMDGNGRWAKARFLPRTAGHKAGVETIRDIVKECSKLGVKHLTLYAFSTENWKRPKLEVDTLMNLLSTYLRNEIAELHQNNVKVTAIGDISALPKTCIRELNSAKELTKNNTGVNLNLALNYGSRADIKNALIDIVKNCESGKIDINNIDEDIIKNYLSTKSIPDPDLVIRTSGEQRLSNFLLWEVAYSEFYFTDIHWPDFKKEELQKAIYVYQRRDRRFGGLK*

>CD630_21400 Clostridioides_difficile_630_NC_009089 30S ribosomal protein S2

MSVISMKQLLEAGVHFGHQTRRWNPKMAKYIFTERNGIYIIDLQKTVKKVEEAYKFTKEVAETGKPILFVGTKKQAQDAIKDEAERCGMYFVNERWLGGMLTNHKTIKTRINKLRELEKMEEEGVFNVLPKKEVIKLRAEKEKLEKYLGGIKDMPELPGAMFVVDPRKENIAIQEAHRLGIPVVGIVDTNCDPEQLDFAIPGNDDAIRAVKLITGAMATAVIEGRQGAEEEVAEDQE*

>CD630_21420 Clostridioides_difficile_630_NC_009089 transporter

MNCKWISKIDERKKCHREADSSGYCIFHKENKSDEEIQLMMDTLHKEEISEFNGFVFENEFNAEEILTYNYKILDFSESIFKQKANFKKYIFKKNIIFNYTEFRDKVLFNGCVFLENCDFNRTIFSKHYINDRIFEKVKFKGPDLVVNKVENFPRMDGIIFSMCTKFVLKNVEYGKSEYEHGKINYRIARNQATKIGEYEMIGFYYYKERIYSSKIMKRSNYPTFSDYLVEKFFDQIARYTTGYGEKPWNILLVIIAIISVFALLYLFVGIESSNSTLVALDINNIGDYSLSEIFKMYMDLWYFSMATFSTVGYGDMVATSLIGKALAGIEVFFGVTIGAIWASVIIKRMIR*

>CD630_21480 Clostridioides_difficile_630_NC_009089 serine/threonine protein kinase

LNFIGNRYEVVNSDDVLEINKIYKARDVFYRKNVLIKVIKHNNYICEDFVSNLIDESTALDEINSPYILKIIDVGIHCTEETTLYYVVSEDFNGIGLDELILGNYLHLEAIVNIMIQVLKALEMIHRNHSYHGSLKSSSIIVDTEYNIKICDFGITKANNGVNTRSYGNRRYLCPHQLCINYTDKESDFFATGLILFESIFKKLPFGESNSEEKMLQSIDKGLDWNSIKAINGNTELINVIKRLLGRNNKYTRANDIIIDLSKVMYEKAYIEVEDEKEEEQKQDKVIQVKKEYKSKMRKKLHKKLVVAGLAIVMISMIIISSII*

>CD630_21540 Clostridioides_difficile_630_NC_009089 [FeFe] hydrogenase H-cluster maturation GTPase HydF

MSLNSTPQSVRVHIGLFGKRNAGKSSIINAITNQSAAIVSDIAGTTTDPVFRPMEILPIGPCVLIDTAGLDDVGELGELRIGKSLDVLEKTDIALLVVDCQIGISQEDLSLIEKFNDKNIPHILILNKIDTIKNQSEILNLTKNKVKCPVVSVSSTDKIGIENLKNEIIKVLPKDSTEFKLVSDLIEPNDLVVLVVPIDKAAPKGRLILPQQQVIRDILDNGAISIVTKEDSLKETLSNLGKKPKLVITDSQVFPQVDKDTPKDIPLTSFSILFARQKGDLKELINGAYALENLKDGDKILMAEGCTHHRQTDDIGTVKIPNMIRKKTGKNITFEFSSGVSFTEDINKYALVVHCGACMMNRAGMLSRIEKAKSFNVPIVNYGVLIAYVKGILERSLELFNY*

>CD630_21550 Clostridioides_difficile_630_NC_009089 [FeFe] hydrogenase H-cluster radical SAM maturase HydE

MTKNKIKYFIDKLYSINSLSFDELLYLIENIDNEEYNFEQNLRNYLYKQASDMRNKHYGNRVYLRGLIELTNYCKNNCYYCGIQSSNKNIIRYRLSLDEILECCDIGYKIGYRTFVLQGGEDAYFTDDKICEIVSSIKNKYEDCAITLSLGEKSYDSYKKYFLCGADRYLLRHETATDSHYKKLHPPNIELRTRKECLKNLKEIGYQVGAGFMVESPFQNNEDLVRDLLYLKELNPHMVGIGPFIPHHDTIFKDYKHGDLEKTLLMLSITRLLLPKVLLPATTALASINPSGRNAGLLAGCNVIMPNLSPQEFRKQYSLYDNKAFTGQEACEYHQSLEENIKSLGLEVDYSRGDNIEWRRL*

>CD630_21580 Clostridioides_difficile_630_NC_009089 4-aminobutyrate aminotransferase

LPMSEITSQMISTEEKKYVAKTQKIPYYPVAFKSGDGAMLYDYEGNEYVDFLASAGSANVGHGNKEISQAVKEQMDDITQYTLAYFHSDPPVKLAEKLVEIAPGDNDKKVLYSATGSACIDAAIKLARGYTGRTKIISMCESYHGSTYGAISISALSTNMRRKMGPLLPEVYHFHYPDKNRTAKECLDEIEYAFAHYLPAEEVAAIFIEPIAGDAGIIVPPVEWVQGLSKICKENGILLVSDEIQQGMGRTGKWFGIENFGVEADLIVLGKSVGGGLPLGAVVGRTEIMQSLDAPAHLFTLAGNTTVCVAALKSIEIIEKENLLQKSIEMGDYIKAGFEKLKEKYDIIGEIRGIGLSIGVDIVKGKGSNEKHPDATAKICYRCIQTGLIMIFLGQSTLRVQPPLVITKEQVDKAMNIIDSAIDDYLNGRIGDEVYEVTQGW*

>CD630_21620 Clostridioides_difficile_630_NC_009089 GCN5-related N-acetyltransferase-like protein

MEQQKDYIELYENEELIEIREARMDDLDTIAKFNYNLAKETEGKELDMDVLTKGVKALLLDERKGKYHVYTVFDKVVAQIMYTYEWSDWRNGNFLWIQSVYVDKEYRRKGIFNYLFNYIKNICDKDENIVGMRLYVEKENINAKATYESLNMYECDYNMYEYEVIHS*

>CD630_21670 Clostridioides_difficile_630_NC_009089 acyl transferase/acyl hydrolase/lysophospholipase

MWGYMYMKNSNFNILAFDGGGLKGALSISILERIVKEYPNLLNNINMFGGTSTGSLIALGLAYGVSPKEIKELYSIENSKYIFNKSYAEILRPKYENKNLKEVLLSIFPEELELKDLNKLVMIPSFYIGNEENAWKPVFYNNMPNSFTKTSKVVDVAMASSAAPVFFPTYNRHVDGGIIATDPSLACIIHAIDSGFRLKNTRLFSIGTGYVYNSIKADTTEWGAIDWIINKEPDLPIISITLEGNSQMSQLFSQKLLGDNYYRLNPKMEKDVAMDDCDALEYLMSLGENCDIKDAFGWIKKKSFYRN*

>PCZ31_RS11270 Peptoclostridium_difficile_strain_Z31_NZ_CP013196 cNMP-binding regulatory protein

MQQGSDYLKKFLEKHIDLLKKIPLFEGIKSDELEEMLECLGVVDKTYPKDSIIFSVGSEVTSIGIMLKGSAHIIKEDIEGNRNIVAELLPGDLFGEVFACTRLHKSNVTVTTTSSCEVLFIKFKSVTGICSSACVFHNRLVENMLQLIAEKNILLHNKIELLSRKTTREKLLMYFSKQIEQTGSHQFTIPFSRNEMADFLCVDRSSMSRELGKMRDEGLLSFNKNKFEIFYFQNKLDI*

>CD630_21780 Clostridioides_difficile_630_NC_009089 oxidoreductase Fe-S subunit

MKSLFKFNKELCTACGACLLACIDQNDNNIDNGELFRHVFTIEEGENIEYFSLACIHCNEPKCLGVCPKNCFKKEEGFIVLDNQNCIGCKLCEKACEYGALIYGNDGKANKCNGCIERYKCGMDSPCVMVCPTGALKFNK*

>CDM68_RS11195 Clostridioides_difficile_M68_NC_017175 formate dehydrogenase

MYNKYNNLYLLLGKENVNMDSYRQLKAKIPCKETGIEIKRTLCDICSPGVHCGIDAYVKDGKVIKIEGTKEHPMNKGLLCTKGLCNREYIYREDRIKTPLKRIGKRGEGKFKPISWDEAYREIAEKLNRVKSEYGAKHVAFFTGYGKWYRPFLQRFASSFGSPNYGTESSSCSTSAQMAGNVATGRNFTMQDMENAKTYLGWGFNPYYSKFLNSVNLIKLKEKGLKFIIVDPRITPTTQKLADLHLRIKPGTDGALALGMANLFIQKDLIDREYIEKHVHGFNEYKEYVKLFDLDTVSRITGVSKDNIVKAVKMLSQGPFCINESAAPIVHHQNGFQNYRAMMSLSAITGNYDRIGGNIPKQDYFAGKIGGFQTLEKEFVKEVLPKNINERIGGERFPLWNEMVTQFQAMDLPRQIEEKTPYEIRALFCHGINARMFPNSGRLFKALNSVDFFVNIDLFMTDTSKYADILLPACTSFEREECKSYPGGYITYTKKVIDKLYDSKSDVEILSDLANVMNIDDDLLKAGYEASIRYMFKNTCVDVDKLKESDLPLKCKDFKPYIVGSYTREGYDTSTSRFELKSTIIEKYKDFGLDALPTYRGFNDDIDNEYTHLLTSGARISNALHSRLHDIKLSRILKNEASCDISILDAKELDVISGDYIKITTKTGSLDVKVNITDRVLYRNIHMYHGYREADVNSIVCDNFDPYSGFPAYRSCKCKIEKMNRQMEGYNEKFI*

>CD630_21810 Clostridioides_difficile_630_NC_009089 alpha/beta hydrolase

MTYFNYNDKQCYYNEIGDGTPLLFLHGNTASSKMFNDIIDFYKDEYKVILIDFVGHGKSQMVDKFSADLWFDEAMQVICFLEAMNYKKVNIIGSSGGALVALNVALERPDLVNKVIADSFEGEVPLESFVQNVKIEREASKQDDGAKAFYIYNQGENWEKVVDNDTEAIFEHYKTIGKFFHKPLETMQPEVLLTGSREDEFVSLISSDFFENTFSLLLEKIKNGKMYLFDKGGHPAILSNGLDFSNVAKKFLEE*

>CD630_21830 Clostridioides_difficile_630_NC_009089 ATP-dependent RNA helicase

MKYTFEKFKLNEKILKSLKSLGYNIPSRVQREVIPKLLKGQNLVVRSKTGSGKTASFAIPLCENINVDYNNIQALIVVPTRELALQVKDEISDIGRLKKVRCSAIFGKQSIKDQIAELKQRVHIVVATPGRILDHINRGSIKLENVKYLVIDEADKMFNKGFVEQMEKILLNLPKEKIVSLFSATIDEEIKYICEKYMLDYSVINIEENESDTNQKTRQIDDKIIKANGREKYILLKELIYSENPKSVIIFCNTKEKVSKLYNKMSKEGFLIRELHADLSQERRIFVIKDFKNQKFNILVSSDVASRGIHIDDISLVINYDVPQDKENYIHRIGRTGRKGNSGKAITIVTEKDEKYIENIETYIGYKINELTDIEQSRITHGKVLFEEYSKGILKKVSKRKKVDKNSYKSKDIKLNIESEVVKLYLNAGKKKKIRVLDIVGAFSNIKGITNDDIGVIEVQDLCSYVDILNYKGDLILKKYKEIPIKKKMVKVKRDIR*

>CD630_21900 Clostridioides_difficile_630_NC_009089 cyclomaltodextrinase

MSNKNKENITLSNQIKGTIQNSLTKEAILHIPMSNYAYGYDRETLHIRLRTKKNEAKRVILRIGDQYVWDKGGAGGGNLNASGVGWSGGTNIVMSKEVETELFDYWIAKCKPLNKRSRYGFIIEGQEEKLLFTEKRIIELGNENDEKELCEIGNFFGYPYLNYIDIPKVPNWVKDTVWYQIFPDRFANGDPSINPEGVDKWGAIPTRDNFTGGDLQGVIEHLDYLSDLGINGIYFCPITIGKTNHRYDTVDYMEVDPTLGDKETLKKLIEEAHKRNIKIMLDAVFNHIGYYSKQWQDVVQNKENSRYKNWFYIKDMSKIDTPIEQIDEKNIPYETFGCEKYMPKLNTENSEVIKYLLDVGKYWIQEFDIDAWRLDVSNEVDHVFWRKFREEVKKVKPDIYILGEIWHGSLPWLMGDQFDSVMNYLMSEAMKKFFCTDEINAEEFKYMINDVMVSYPIQVSEVIFNLLGSHDTTRILTYANGNIDRFKLAYMFMFVQTGCPCIYYGDEIGMEGELSSISEGQRKCMEWNEEKWNKDIFDFMKKIIRLRKENKELRSISNEWVLADKENGTIILKKESISIIINNSSKNIILTLPDYLANKKVKDLYEEEIVDLKEELMLEKYKFIILK*

>CD630_21940 Clostridioides_difficile_630_NC_009089 ROK protein

MMSNKQGSNIKDVKIKNKLIILKLLSTNTPMSRVDLAKSTGLTKMTLSNLVTELINENMIREIESSTLNAHSSGRKPILLDISNISPCICGILIRRNICKVIISDLKGNIVKQLSETYPKCLSNDVLKDIIKRMFTKIMKINKRDIIGIGISSIGPVDDINGTILNPPDFGNVSNLNIVDFIKEMSDLPIFLINDANSGALAEKMYGLGKNISNYIYLHIMNGIGAGLVLENKLHTGNLGQSGEIGHTSINFSGPLCSCGNNGCLDYYTSVSNLIKRIESLSHIYPNSPLINCKDFSLVKLIDEANNKDSLAMFLLDEFCTYVSYALVNTLTLIDCSSIIIGYDFNIPGTFIENTLLKKLTASASFSKYKKISVRHSNFGANAPLIGSIAIVSYNFFNGSINFY*

>CD630_21950 Clostridioides_difficile_630_NC_009089 ferritin

MISAKMEKLLNEQINHELYSAYLYLSMSSYLEAEGLKGFSNWFYVQYKEETDHAMFFYKYLHNVGGKVQLDAIPMPDSDFTSAMDILERTLAHEKKVTALINNLAAVANSESDFRTSQFLLWFISEQAEEETNCEDNIKRVKLAGEGGLFFVDQEFANRTYAAPTNPPVTI*

>CD630_21991 Clostridioides_difficile_630_NC_009089 4Fe-4S ferredoxin

MKRGVDMNLKINAERCKSCEYCVISCKKGALKISSKINKEGYAHVEVDESKCILCGICYQVCPDNVFEIIKEEACQEA*

>CD630_22040 Clostridioides_difficile_630_NC_009089 esterase

VAFIECKFKSKYTGGETDIIVILPVANGKELFSGKEIYDFEGKFKTLYLFHGLEDDQSSWIRYTNVEHYAKNKNIALVIPRVETSFYTDMAYGHQYFTFVSEELPKFVRAIFPLSDKREDNFVAGMSMGGYGALKLALSKPKEFSAVASFSGSPDMIHELENQQIETNADILFHDFGRPEDAKNSENDLLYILKNLKERNEDIPKIYQFCGDRDFLYKNNQMFKKYADSLGVDVEYEECVGGHEWRLWNLWIKKFIDIIG*

>CD630_22050 Clostridioides_difficile_630_NC_009089 membrane protein

MKINWNTKYTTIAIYTFIIAASSIIFYLVSSQIDVFSNNLDAIFTTLQPFIIGFAIAYLLNFILKFYEDRIFIKSEKLKKLKQSSKRGLGLLLTYATATLILYLFMHFVLPQVIESIVGLANDIPMYVNNATKLIDKLMTDLNLDEQYFNLAVDKWNEFVTYIIKFVTDLIPILGSMLKNVASSIWNIVLGLIVSVYLLIDKEKFYGLSKKITYAVFTEKQAARILELTHRSNYTFGRFLGGKILDSFIIGILTFVILTLVKMPYTLLISVIIGITNIIPFFGPLFGAIPSTLIVMFVSPIKAFWLLLIILIIQQIDGNIIGPKILGDSIGISAFWILFSLLVAGKLLGFIGMVIGVPMFAVIYSIIKDIVESKLDKKGLPTDTSDYM*

>CD630_22060 Clostridioides_difficile_630_NC_009089 aldehyde dehydrogenase

MDIKELVKMQRKYYNTGKTRDISFRIEQLKKLKLVVSQNEEKILLALKKDLNKSDFEGFMTEVGMFYSELNFAIKNIRKWSKIKRVKSSMVNFPSISKVVPQPYGVTLIMSPWNYPFQLALIPLVWSLAAGNCVILKPSEYSASTSSVVKDIVEDTFSKEYVAVVQGSQEESEKLLLERFDYIFFTGSTNVGKIIMKSASEHLTPITLELGGKSPCIILKDANIDLTAKRLTWGKLINAGQTCVAPDYVLVHEDRKNELIEKIKYYTNKYFGDNPCNNEQFPKIINQKHFNRILSLIDKDKIVYGGNYNKETLKIEPTIVDNVNWDDNIMKEEIFGPIFPILTYKDLDEVIQKIIQMPNPLALYIFTKNKYLENKLLEMIPAGGCCINDTVTHIATNYLPFGGIGESGMGSYHGKAGFDTFTHYKSVLKKLNLDVPIRYAPYDNKLIKVLKKIM*

>CD630_22090 Clostridioides_difficile_630_NC_009089 GTP-binding protein HflX

MDMAKKGITVGLNINNKSEDFNELMIELENLCSACDIDVVGSITQNAKQVNRAFYIGTGKVEEILNLIKKENIEIVIFYNELSTSQLKNLEEKLNCEIIDRTALILDIFAQRAKTREAKLQVEVASLKYMLPRLIGSNENLGRQSGGVGTKNRGSGEKKLELDRRRIEEKITSLNRELDDLKFQRETQRSMRRKSNLPNVALVGYTNAGKSSIMNKLVDIFKNSEEKKVFEKNMLFATLETSVRNIVLANNKEFLLSDTVGFVSNLPHDLVKAFRSTLEEACEADVLLHVIDISNPSYKSHIKVTEDTLKQIGADGIPMIHVYNKIDLIDVEVLDRILDSIDKEGIFVSVKKDINIDKMIKCICDSIFKDYVRCKFLIPYDKGHVVSYFNENTSIINTEYREDGAILDVECSNIEYNKYKKYALE*

>CD630_22100 Clostridioides_difficile_630_NC_009089 multidrug family ABC transporter ATP-binding protein/permease

MSRMGRGPMGKSMGAGQKANDFKGTMRKLIAYLSKFKISIILVIVFAIGSASFSIVGPKILGKATTKIFEGLVSKVSGGNVGIDFNAIGKILTFLLFLYLISALFSFIQGFIMSGISQKVSYNLRKEISAKLDRLPMKYFDTKTHGEILSRITNDIDTLNQSLNQSMTQLITSVTTMIGVLIMMLSISGIMTLVAVLILPISMFVISRIIKKSQKYFRYQQEYLGNVNGQVEETYSGQTIVKAFNREDEVIEEFDKLNDSLYNSAWKSQFLSGIMQPLMMFIGNLGYVMVSILGGWLAIKKTIEVGDIQSFIQYVRNFTQPMTQIAQVANLLQSTAAASERVFEFLEEEEEVQIVENAVSIDGLEGKIDFENVNFGYNPNKTIINDFSVNVKPGQKVAIVGPTGAGKTTIVKLLMRFYDVNSGSILIDGHNIKDFNRSELREMFGMVLQDTWLFSGSIMENIRYGKLNATDEEVIEAAKSAHVHRFIKTLPDGYKMKLNEEASNVSQGQKQLLTIARAILADPKILILDEATSSVDTRTEVLIQKAMDNLMEGRTSFVIAHRLSTIRDADMILVMNEGDIVEQGNHEELLKKGGFYANLYNSQFEEDEAM*

>CD630_22310 Clostridioides_difficile_630_NC_009089 anaerobic sulfite reductase subunit C

VIRDLNTKKVMKNAYRITKTKYETSLRVRVPGGLIDPESLAVVSKIASEYGNGQVHITTRQGFEILGINMEDMEEINKIIQPVIEKMNINQSEKNAGYPAAGTRNIAACIGNKVCPKAQYNTTEFAKRIEKAIFPNDLHFKVALTGCPNDCIKARMNDFGIIGMALPIYEKDRCVNCGACVKKCSKISVGALKTENNKVVRDKDKCIGCGECVLNCPTNAWTRDEKKYYRLAIMGRTGKKNPRLAEDFLLWVDEDSIIKIILNTYRYVEKYIDKDAPGGKEHIGYIIDRTGFMEFKKWALEGVELPEITKMYENIYWSGIKYL*

>CD630_22390 Clostridioides_difficile_630_NC_009089 SSS family Na+/solute symporter

MVGFTWIDFAILVVYLLAVLFAGLLFSKKEMKGKEFFKGDGTIPWWVTSVSIFATLLSPISFLSLAGNSYGGTWILWFAQLGMFIAIPLTIKFFLPLYSRLEIDTAYEYLEMRYESKGLRVLGALMFIIYQIGRMSIIMYLPSIVLAGLTGIPVNVLIIVMGVIAIIYSYTGGLKAVLWTDFIQGMVLIVGVTGTLFYLIASINGGFGTVMDTLTTGHKFLASNEVMFDKNILSTSAFIIFVGAGLNTFSSYVSSQDIVQRFTTTTDIKQLNKMTYGNGVLSMGLATVFYLIGTCLFIYYTQNPDLAQTVQQDQVFASYIAYELPVGITGILLAAIYAASQSTLSTGLNSVATSWTLDIQTIITKDISMEKQTKIAQYISLGVGILSIAVAIVLANGEIKSAYEWFNSFMGLVLGVLAGIFVLGAFCKKATKMGAYVGFIVSAILVVYLKYRMPEVTSWAYSLITITTSVVVGTIVSAIEAKVKAKVSLPKAETTVYYEK*

>CD630_22400 Clostridioides_difficile_630_NC_009089 N-acetylneuraminate lyase

MRTTDMKGIYSALLVSFDKEGNINEKGLRQIIRHNIDVCKVDGLYVGGSTGENFMLSTDEKKRIFEIAKDEVKEEIKLIAQVGSVNLKEAVELAKFTTDLGYDAISAVTPFYYKFDFEEIKHYYNTIINSVDNRLIIYSIPFLTGVDMSLDQFGELFENEKIIGVKFTAADFYLLERMRKTFPNKLIFAGFDEMMLPATVLGVDGAIGSTFNVNGVRARQIFELTKNEKISEALEVQHVTNDLITDILGNGLYQTIKLLLEEQGVEAGYCRQPMKEATDEMKSRAKEIYRKYF*

>CD630_22460 Clostridioides_difficile_630_NC_009089 subtilisin-like serine protease

MEKSYCIIYQGDIESALQENGINRYMVLNSQLAVIYVPLDFDETILNNIIQVAWWEESEPMSSLIEITNNVNNGETITTAAETDYIYENPYNDITGRGILLAVIDSGIDYLHPDFINDDGTSKVLYLWDQEANTNPPPEGFIFGSEFTRSELNIAINRNDGSLSQDNIGTGTLVSGILVGNGRINSQYRGITTESDLIVVKLKSYTDTYYAGRINYSVSDFLAAITYVTNIARTENKPLIINLTIGVKSSAVATTSILDTFNILSSAGVVVVSGAGNQGNTDIHYSGRFSSVGEVQDVIIQDGDDYALDITLNTNGPDKVGAQIISPSGEVSHDIRYSPDFYIYRGKFNLENTTYAMRFIYPYITSGKENLEIRLRDIKPGVWILRLTSELIINGEYDIYLPNKNLIAPDTRFLDPDSVATITMYAASDDVITVGTFNNKTDSMWIGSSKGPIRGRGIKPDIVASGVDIISTYKNGTYNTGTGTGVSSSIVTGVLALLMEYLEKQDNVPRLSLFTQVLKTYLILGATKLEIYTYPNVSQGYGILNLKNTIQQIANTL*

>CD630_22470 Clostridioides_difficile_630_NC_009089 subtilisin-like serine protease

VIIINYELIVKYNGDILRLEEELGVSVEILNSSYAIITSSNEEDVNILLTYPEIEFIEKPFILQTQDVQSFSSTGITGFKNRTGLTGKGTIIGIIDSGIDYTLPVFRDSDGRSKILYYWDQSIQGNPPEGFREGTLYTNEDINNAIDGSMYIPISTTSLHGTHVAGICATIASDARIIVVRVGNIQTDIFSRSTEFMRAIKFILDRALELRMPVTLNISYGSNEGSHRGTSLFEQYIDDMCLFWKNNIVVAAGNNADKGGHKRIRLQNNITEEVEFIVGEGERILNINIWPDFVDDFSVHLVNPSNNQTQAISLTSGEIRNTLGETRITGYFYPIAPYSLTRRVTLQLSSNTQITPGLWKIVFEPIDIVTGNVNIYLPTSEGLNRNTRFLIPTQELTVTVPGTASRVITVGSFNSRTDIVSIFSGEGDTQLGVFKPDLLAPGEDIVSFLPGGTSGALTGTSMATPHVTGVCSLFMEWGIVNGNDLFLYSQKLRALLLKGARRLSNQSYPNNSSGFGFLNLSDIDLYTLSNINQDLETEDMGYRSINKSFKDEENSYKFIDGYNMQIHNDLENEIYISKNASRQSGILSGIDIVHTPEFEEELAGLGMSQRFFKISDSLGVLSINNTDYSSIQRVLQLPSIIRTVSTTKMTLLGEINRGTFGGVVATEEMGVNFFKNNPNINITGRGTLISIADTGIDYLHPDFIYPDGTSKIVYLWDQTKEGTPPDGFYIGTEYTREDINRAIAENDPSLSQDEVGQGTMLSGICAGLGNVNSEYAGIAEDSELIIIKLGKIDDFYNSAMLFAASQYAYKKAFELGRPLVINMSLGTSSLAGLTNRSNSEKAFFTRGLCITAGAGNEGNTQTHTSGIIPYVGGSVEVELELNEDEEELSLELWLNRPDKADVIIVSPTGEESKSVGISNYNKVTGLFDLEGTEYSITYIYPTTFSGQQFTNVTLKNAKRGVWKIRLVGVYIITGRYNLYLPNRELLKSGTRFREVDPFYTINYPAIQDDLITIGAYNTINGSLWQSSSRGPTIEDRLKPDIVAPGVNIIAAYPGNTYATITGTAAASAHAAGAAAMYFQYTFVDGRYPNQAYVQKIKTFMQAGARKDSNTVYPNTNSGYGLLDVRGMFDVLR*

>CD630_22490 Clostridioides_difficile_630_NC_009089 ATPase

MIKKEISNFRGSSDYVVSPELMASVNVAIALEKPLLIKGEPGTGKTMLAQAISNELKKDLVIWNIKSTTKAQEGLYVYDTVQRLYDSQFGGEGVDDISKYIKYGKLGEAFSSNQQVILLIDEIDKADLEFPNDLLWELDKMEFYINETKETVRAKQRPIVIITSNAEKELPDAFLRRCIFHYIEFPDRDMMEEIVKVHFDKVEEHLLEQVMTTFYWIRSLKDIQKKPSTSELIDWIQALTLSGMPIEKIEKEVPFAGILLKNNEDIESMQRHL*

>CD630_22560 Clostridioides_difficile_630_NC_009089 PTS system transporter subunit IIC

LEINYKKRNFKSFAKRKNIEISVQRYLIDALSYMALGLFSSLLIGTIFNTLGDNFNISLFTEVISPLAKQVTGPAIAVAIAYGLQAPPLVLFSCALVGACGNELGGPVGAFVATLFAVEVGKLVSKETKIDILVTPAVTILIGVLVAELIGPAVSAFMTSFGNIIMQATTMQPIIMGALVSALVGIALTLPISSAAICMMLSLGGLAGGAATVGCCCQMVGFAVMSFRENGVGGLVAQGLGTSMLQVPNIIKNWKIWIPPTVASILLGPLSTTIFKMENIPIGSGMGTCGLVGQFGTVTAMQSAGKGGISMWVGILLLNFILPAIVTLLISEFMRKKGLIKPEDLKLDV*

>CD630_22590 Clostridioides_difficile_630_NC_009089 hypothetical protein

MIRKKSELQVEVKENLNGGIGKVKLENIIQNEELKGKGRLFKRVALPVGSSIGVHDHTTDFEVYYILKGKGKVFDNGEFVEVNEGDVVYTADGEKHSIENIGEEDLEFVALVLYM*

>CD630_22660 Clostridioides_difficile_630_NC_009089 oxidoreductase

MNESYWFLNSSPKEYNKLGENIKTDCLIVGGGITGLTTAYLLAKEGKKVVLVEADKIGYGTSGRNTGKVTCQHDIFYSKIEKKYGLDKAKSYYNANNEALNLVKQIIEENNIKCDFKRETSFVFTEKEDTIKNIKDEYRTCKKIGINCEYHETIENIPLDIKGAISFTNQGQFNPKKYIDGLAKAAVNLGLKIYENTPVVDLEKGKICRVKTREDNIIEAENVIISSHSPWYDGLNFYFAKEYAERAYLMVAVLENKLADGMFISIDDPSITFRQYNDGSENLLIFGGGDHKVGQGGTEKEIFDDLEHYGKEVFKVKDFKGKWSAQDNMSFDNVPYIGYINKREDNIYVATGFSKWGITNGTAAGIIIKDLIINNNSDYKDTFNPSRLGSYFSKDFIKENANVAINYVSGKLKIGSGDMPKNNGEGKIVNIDGKRYGVYKDDNGDFYIVDTTCTHLGCELNFNSEEKTWDCPCHGSRFDYKGNILEGPALKPLKLYGHGDNDVNPKLL*

>CD630_22700 Clostridioides_difficile_630_NC_009089 1-phosphofructokinase

MIYTVTLNPSIDYIVKLDELKTGSTNRVKEEYVYPGGKGINVSRILKELGNDNTSLGFISGFTGEYIIRTLEEKELKTDFIKIKSGFSRINVKIKALKETEINGQGPNIDDEDIDTLYKKLDKLNQDDILILAGSIPSTLDEKLYENIMARLEKKNIKVVVDATKNLLLNVLKYKPFLIKPNNDELEELFGVKLNSIEDMVKYARSLKEMGAINVLVSMGKGGALLITEEEEVLISDVPKGKVKNSVGAGDSMVAGFISGYLNTGKYDYALKLGAASGSATAFSYDLAKREYIDKLVNEISVKKF*

>CD630_22830 Clostridioides_difficile_630_NC_009089 PTS operon transcription antiterminator

MELRDKLVNIINTENKKDPLTDVQIAKFLSTTRENITNLRKELNISNSRQRRYPYLKSAISAILQKNKNISISEITRELMTEGFNISRRVVEELLPKESLEMDVVEESEEESNDPFETLIGNKGSLRNAVEQAKSAILYPPKGLPTLIIGESGVGKSLFSRHMYEFAKQKKIVKESSNFVVFNCADYSDNPQLLLSLLFGYKKGAFTGAEYDTPGLVEEADEGVLFLDEIHRLPPKGQEILFSILDRGKFRRLGETNAERKVSIMLIGATTENVETNLLLTFRRRIPMLITLPPLHDRLLKEKIDLIYNIFQQECNRINAKIFVDKNVIEILALKKFSGNIGQLQNMIQVLCARAFMKFINSKDEDSESIVVVDINEVLKLKDSFKDAAFQEIEYTEIRKYLKNAIFIPFNLEESSVKGSLLSDEYILPEDIYKTIEKKYYDLKSLDIEDIDIENILWTFILNRFADLKFNLDSNEEIFSMSDLNSFVSDSLVKLVKELMNDIVEKNMNLEVNKNMFKYLAIHLEEAIKRIKLNQKIINVNLEKIKIDFSKEYEISKSFAIKIEESIGIKISDDEIGFITLYIKSALKNEVKKDKVGLIIISHGRIATETVNVVKELLGVKFPVAIDMPLDEKPINIYNKAVELSKIIDQGKGILFLVDIGSLTNIGQIVNKRTNISTKTIDRVDLLMALEATRKVSMGEEELDEIFFSLLKDRMGYNYELEKHIDKSNAIITLCLTGEGTAKYISKTLEEKYDNTKCYQMSALDENLFCKIENLKETNNILAIVGTINPKIPGINFIPYNKELFKNLDIYLSKSKQQDDRPVKSYERMLDEDLVIFEPDIYFKKDLLEYVCSILINKEYVEKDYLDSVLHREEMLPTYSKGAIGVPHGDSSTVNSTRFVFVKLKNPIDWGVGNVNFIFMPVFQANDKEIVKNVLEILKDAEFMNNANKCFDKDAFRRIIFDKFKHL*

>CD630_23120 Clostridioides_difficile_630_NC_009089 molybdenum-like ABC transporter permease

LKNKFFTTLLTGFFAIVFLFIVSPLLMLIIKGISYVPICLKSVEVQYAITLSIKTSLISTIVCLLLAIPVAYFLHITKLPFKKLIIQIINLPMSLPHLVSGIALLLLFGRMGIGDSIYKIFKLDFIFTKQGIVLAQVFVNLPLTIKILHTSLNESNEKMIFVARTLGCNSWEAFRFIILPNLKTGIISATVMTWSRALGEFGAVAMIAGSTRMKTEIIPTSIYLNMSTGDIDIAIGIAVILIFISLTCLMLFEIFFNREVDKN*

>CD630_23140 Clostridioides_difficile_630_NC_009089 hypothetical protein

MNIFDILRDYLLVQVDKYNLNMDMISIVSKSLSSKEAIGNTKRKDFPIIVGKEIMLEADFKGAKGQAFTSTPSTFEGSLKDILSLDLHDNPHDRSLFIASLNAVMKYLGKTDRTIHCKNNEPEVCAKKFPEFIKMEFGNPKVAIIGYQPAIIDNIKDFFETRVLDLNPEFVDTIQYNVKIEDGIRDYEDVISWADLVICTGSTLCNNSIINFLSLNKPVYYYGTTIAGASNILGLKRLCFCSK*

>CD630_23240 Clostridioides_difficile_630_NC_009089 sugar-phosphate dehydrogenase

VKAAVLHGTNDMRFEDIEIKPCESDEVKIKVMAAGICGSDPPRVLKHWKYPVPAIPGHEFSGVIAEVGKDVKNVKVGDRVVAIPFIPCNECEYCKRGLFSLCDDHGMLGAKSFGAFAEYVNIKATNVLPIGDMDFEDAAMIEPLAVAMHGVLNIGVQVGDTVAVMGSGTMGQLVIQGLKIAGAGTIIAVDISDNKLRESKELGADIIINAKDINPVEKIKELTGGKGVDIALECAGSKITQEQCLLITKKKSKIGFLGIAYSDITLSEEAFENIFRKELELKGFWNSYSAPFPGQEWTKGINLVNEGKIKLKEMVSHRFSLEDTYKAFEMIRDRKEEFNKILILPQGVEK*

>CD630_23330 Clostridioides_difficile_630_NC_009089 PTS operon transcription antiterminator

LKKKKITSRQKKIILMIVENSKKNIPITISEIAGTLELSSRTVLRDMSGIEKWFDENDFNFVKKPGVGLILEENIENQNFIIELLEEEKIEKEYSKEERNLIILSKLLVSNEPVKSYYFAKILKVSEGVLNNDFALASKWLERFDIELVRKPGLGVYLKGQEKNFREAYVNLIYDSFNEKEILDMVRNISENIQTDKAIEILSENRLLNLMDRCIIRKVEATLTKKLSDLDVNLADSAYIGLVVHISLALQRIKNGENITMDKEFLKELSITEEFKLAKEIVKGMETDFNMDIPVDEVGYITMHIRGAKQRSSSNHKALNLDDIEIMEITNKMIDLAEDEFKISLKNDERLFKDLANHLGPSINRLNMGLEIRNPLLDEIKSKYSYAYDGVEKISRIIKDKLNINSIPESEIGYIAMHFASAIEKNLMMNTNINIVVACPTGIGTSRFLSTKIENKFPNLNILETISAINIDEEYLKEKDVDLIVSTVELNTSLNYICVGPFMSLDDEQIIKEKIKSIAQNKLINLNAKNDTKSKNKVYEQITESMNIGKDILQFLEEIRFEKFESKDLSELIEDSSRIFAKSSEDIISIKESLKERLKISIPYIEESKILLLHCMSERIDIMKLSIIKLENRIVLDSNEEIDNVVFMLLPKNSPSYQRQIMSEISGSLIDNIIFTNKINKFSIEEMALEVKDIVFNFYTNRLKAFIDK*

>CD630_23360 Clostridioides_difficile_630_NC_009089 toxic anion resistance protein

MNNLDDIPVMPTLTLDPFGEESNTNDIDNSDLLMKKDEKDPEEEKLSESERKMVKEFAEKIDITNTNMILQYGAGAQKKVASFSETALKSVKTRDLGEVGDMLTNLVTDLKAFSADEKEQSGFLGIFKKANNKISNLKTKYDSAEVNVDKVSKELQKHQVKILKDIAMLDKMYELNLAYSKELTMYIIAGKQKLKDMKEYEMPKLREKARLSGSTEDAQSVNDMVSLCDRFEKKIHDLELTRMVSLQMAPQIRLVQNSNNLMAEKIQSTIVNTIPLWKNQIVLALGISHLNQAMKAQREVSNMTNELLLKNAETLKMGTIETAKESERGIVDIDTIKKTNQSLISTIDEVVKIQHDGRIKRQEAEVELSKIENELKSKLLEFTVK*

>CD630_23420 Clostridioides_difficile_630_NC_009089 succinate-semialdehyde dehydrogenase

MEKAVENFEDLSKEYINGYIERARKAQREFECYTQEQVDKIVKIVGKVVYYNAEYLAKLAVEETGMGVYEDKVAKNKSKAKVIYNNLKDKKSVGIIDIDRETGITKVAKPVGVVAAITPCTNPIVTPMSNAMFALKGRNAIIITPHHKAIGCSTKTVEMINEELEKIGAPENLIQILDQQSRENTRNLISSADVVIATGGMGMVKAAYSSGKPALGVGAGNVQCIIDRDVDIKEAVPKIIAGRIFDNGIICSGEQSVIVAEEMFDKIMDEFKNNKGFIVRDKVQKEAFRNAMFVNKSMNKDAVGQSVHTIAKIAGVEIPEDTKIIVIEADGPGEEDIIAKEKMCPVISAYKYKSFEEGVAIAKANLNVEGKGHSVSIHSNTVKNIEYAGENIEVSRFVINQCCATSAGGSFFNGLAPTNTLGCGSWGNNSISENLDYKHLINISRIAYYMPENEVPTDEELWG*

>CD630_23470 Clostridioides_difficile_630_NC_009089 Xaa-Pro dipeptidase

MSRVKNVVELLETKGVDALYLTKKTNVNYISGFPDEEAYAVICKDGNFLVTDSRYMELAEKVCKDFEIINWHNFDRSVAKAVKSVCDKVGIKKLGFERTNIVFDKYEELKNLIEKDNGELIPTENIVETLRYVKDKDEIKNTRKACEIADKALEELIPHIKAGVSEIELATKLEYFMKMNGAQNIGFETILISGAKTSLLHGKPSDKIIEKGDFVLIDYGAMYNGYISDTTRTFIVGGASEKQLEIYNLVKEAQNVGVENMKAGVHATIPDAEIRKVVKKYEDYYYQGIGHGVGRDVHEEPFIGNYGDKIIEEGCIITMEPGIYFPGWGGVRIEDTVLITKNGPERLTKFPKDLMILDK*

>CD630_23550 Clostridioides_difficile_630_NC_009089 thioredoxin 2

MLDLDKATFEEEVLNAEGFVFVDFWSEGCEPCKALMPDVHKLAETYGDKIKFCKMDTTKARRLAIKQKVLGLPTMAIYKDGEKVDEVTKDDATVPNIENMIKKYL*

>CD630_23560 Clostridioides_difficile_630_NC_009089 thioredoxin reductase

MENVYDLVIIGSGPAGLAAGLYGARAKLKTLILEKDKTGGQIVITHEIANYPGSVPNATGPSLIARMVEQCKEFGAEMLRDNIVDTELDGDIKVLKGEKAEYRAKAVIIGTGATPRKIGCPGEKELTGKGVSYCATCDADFFEDFEVFVVGGGDSALEEAMYLTKFARKVTIVHRRQGFRCAKSVEEKAKANPKIEFLLDTVIEEIKGDGILESVVFKNKVTGETHEYFADEEDGTMGVFVFVGLDAQTDLFKGKVDMDEKGYIITDEDMRTNIPGVFAAGDCRSKTLRQVVTATNDGAIASIVAEKYIDEKFGN*

>CD630_23590 Clostridioides_difficile_630_NC_009089 HAD superfamily hydrolase

MYKLIALDIDGTILNTQKRITPEVFESIQEAKRAGAKVVITTGRPLPGVKELLNQLNLTDEGDYVICFNGAIIQEVKSEKIIHDVEMSLDDFDFIYNNVCKKYKTKIHINTMTNLITPNETPGKYTLHEAKLNNIEVKYIQKDKIDESIKICKIMIVDEPERLEEIIQQLPKNLFNKYTIVRSAPFYLEFLGKTTNKGTALKTLCTNLNIPIENAIAVGDEENDQHMIKYAGLGVAMGNARNSIKEIADYVTDTNNENGVAKVINKYILNKAI*

>CD630_23610 Clostridioides_difficile_630_NC_009089 nitrate/sulfonate/taurine ATP-binding protein

MKLSVRDINKTFVNNRVHTKVLEDISMDIDDGQFVCLLGPSGCGKTTLLTIIGGFQKSERGDVFINDKRVKKPGIDRAFIFQNYALFPWKTIRGNVLFPMKQQKIPKEKREEMLEELLVMSDLKGKEKLFPHQLSGGMKQRVAMIRALACNPEVLLMDEPLGAVDFQMRQNLQEELERIWIKKKITALMVTHDVDEAVYMSDRVIVMSRDKGRIIDDINIDIPRPRIRGSQKYEEYKNKLTDTLSKCYEV*

>CD630_23710 Clostridioides_difficile_630_NC_009089 L-aspartate oxidase

MNLEQDVLIVGSGVSGLYCALNLDKSLNVLVVSKSTIENNNTYLAQGGISTARNIDDIESFVEDTMKAGQYKNRVEAVQVLADESIENVGQIVEYGMPLDKENGEIDYTREGAHSVNRIVHSKDNTGEVVFKTLLKEAKTRENITLIEDAYLLDILKDGNKCIGARIFKSKKEIHVFSKIVVLATGGIGGLFKNSTNQRHLTGDGIAIALRNNIKIENLEYIQIHPTAFYEENNEGRRMLISESLRGEGAKLLNKNKERFVDELLPRDVVSKAIFEQMEKDKLPYVYLDATHLDSEYLINRFSFIYNECLARGTDITKECIKVSPAQHYFMGGIHVDLDSKTSMENLYAVGEISCTGVHGANRLASNSLLEGLVFSKRAAKNINSVIDNVKVKFIDVPDMDIDIEQVKKENKILVIKEIERTSEDFGDELFDY*

>CDIF630_02613 Clostridioides_difficile_630_CP010905 putative CstA-like carbon starvation protein

MYTKTKKFYKWGDIMTTFLIGLAILLIGGALYGAYCEKVFGPDDRKTPALAQSDGVDYVPMKKWKNSLIELLNIAGTGPILGPIQGILFGPIAFILIPIGCVFGGALHDYMSGMISIREKGAQMPSLISRFLGNKVFQVYNIFLCLLMLLVGAVFIYTPGDLVVTQILNMKSTINNPVVWIVYGLIFLYYLCATLFPIDKIIGKVYPIFGAILLLSAAGVGIGIFTQGYDLANLSLANWKGIHPDGIPLIPTFFVTVACGIVSGFHSTQATLIARSVSNEKEGKTTFYNMMILEGLIAMIWAAAAMGIYNKGIPKELVGSPDVIGLVARDLLGSIGGIIAIIGVIVLPITSGDTALRSLRLMLADYFHYDQKEKKHRVILSICIFIPVIAILIFAKLSASGFNILWRYFSWSNQTIAIFAFAMITVYLIIKEKNYIISLIPGMFYSFVIFSYIFNAQIGFNLNINISYVLAAIFTVLYAILTVRSGRKLKSKADTKLAD*

>CD630_23780 Clostridioides_difficile_630_NC_009089 membrane protein

LMGKISLKSMIRLFSGFFIFAISSVLMINAHVGLMPWDVLHQGLSIKLGITIGQASIMVGVVIVILDAVFGENIGWGTLLNMTFIGIFIDLVIFSGVIPHASNTYIGVFMVVIGIILAAIASFLYLGVCLGSGPRDGLMIALQKKTNKSVRLVRTILEILALVVGWLLGGSVGIGTLVSALGLGYVLQIVFRIFKFDTKLLKHRFIIDDIREWKEKKSNEHKCKSSIVIKNEQN*

>UAB_RS0213145 Clostridioides_difficile_ATCC_43255_NZ_CM000604 indolepyruvate oxidoreductase subunit

MKQLMTGNEAIARGAYEAGVKYASAYPGTPSTEILENIATYKDAIVAEWAPNEKVALEAAIGGSIAGARTMASMKHVGLNVAADPIFTYAYTGVNGGMVLITADEPGMHSSQNEQDNRMYAKFAKIPLFEPSTSQEAKDMIKEAFEVSEKYDTPVLYRVTTRLCHSKGLVECYDREEVEIKEYVKNAKKMVTVPANAQIRRGVVEERVECYDREEVEIKEYVKNAKKMVTVPANAQIRRGVVEERMEILKKFSNETDLNYYEINDTKIGVIASGMCCNFAKEVFGKNASYMKLGFTNPLPYEKIKEFAEKVDKIYVIEENDPFIEEQIKAYGIDCIGKDVIPPYGEMTPDVLRKAIFGKTNDTIEYKSELVTPRPPSFCAGCPHRGFFYELGKRKNLIVGGDIGCYTLGFAPPYNGIDYVVCMGSAFGTAHGAQKVLNMKDDNEKRLVGVLGDSTFFHTGINGLLDVVYNRGNSISVILDNRITGMTGHQENPGSGYTLQGAKTKEVDIEGLVKACGIEHVRVINPNNLKEVNEALDWALAIEDEPSVIITRWPCVLKKFSKEDIEEFNNPFKTKCKVDHDKCIGCKLCLKTGCPALSFDKENKLSNIDRNQCVGCGVCAQVCPKQAIVKEEK*

>CD630_23820 Clostridioides_difficile_630_NC_009089 pyridoxal phosphate-dependent transferase

MSQSMVGKHAMWPKENDVIFSISGRAQAAEKAFGMDNVINATIGALMDDSGKLITMKTVYEEYKALDNCEIGAYAALEGQPDYLEAVKKVFFRDYLPEGHIRVLASPGGSGAIKLAVWNYTNEGDEVLTSDWFWSPYVSIAEEANRKVVNYQLFDENRRFNFESFKEKFVNIAEKQGRVFTIINTPAHNPTGYSVADDEWDKILDLSKEVAKDKDKKIIFFVDSAYIDFAGDDDVCRKFFKKFSNLPENVLVLVGFSMSKGFTAYGMRMGAIICISSSEDVAEEFHYSCVHSCRANWSNCNRSAMAVLSNIVNDPKKFKEYEDEKEIYKNMLTRRADVFVKEAERVGLEILPYIAGFFVSIPCDNPKEVCEELTKHNLFAVPLKMGLRFAVCAVSEDKCKKAPSIIKEALESLEVKINN*

>CD630_23870 Clostridioides_difficile_630_NC_009089 alpha-hydroxy acid dehydrogenase

MDYNDLLKSARENFNGTCKVCKICNGLACAGDVPGMGGKGSGSSFIENRKSLEKIKINMRVIHNVSKPDTSIELFGRKMSSPIFAAPVSGTILNMGGKVSEKEYIEPVVRGCSNSGIYAMVGDTNVDTFLLDNLDVLKDNRGNGIVFIKPWNNSKIIDKIRLSEEAGAFAVGVDLDACGLINNQFQENPFSPKTIDEIRELVESTRLPFIIKGIMTVDDALMAVESGASAIIVSNHGGRVLDYTPGTCEVLPDIAKSVKGKITILVDGGVRTGVDVVKMLGLGADAVLMGRPFVTASFGGGLDGVEFFIEKVRNELCETMILTGCQNVKDIDGRVIWNLL*

>CD630_23940 Clostridioides_difficile_630_NC_009089 DNA polymerase III subunit epsilon

MKEYVVVDLETTGLDPYKGCEIIEIGITEIKNEQIVKNYSRLIKPKGIISSFITELTHISNEMVENEEPLELVLPRFRKYIGDRTIIAHNAKFDLKFLNYYLRMLDLEPINNYICTVELLKKCKSYKGKNKKLETACAYYNIENINAHRADSDTLATAKLFLKIKDEY*

>CD630_23950 Clostridioides_difficile_630_NC_009089 hypothetical protein

MENITSYFTTILCVFICLSSVFIFTQLARVFINKKKINQKIKSRNGFRYDRDFIEARREEIHIKDNNNNKNKSNNKKLKEEKVFKYDNGDLYKGEFVDGKKNGFGIYIFSSKEKYEGLWKDDKMHGIGKYTYRDGSIYTGEFKYGLKNGLGKLTYPNNDIYKGYFLDNKKNGKGVLYKNDGNKQAGIWEDDEQCKSLDFKDLNNNKINNVYQNRLNNNKLKV*

>CD630_23960 Clostridioides_difficile_630_NC_009089 hypothetical protein

MGFREVKIEELQFNPFTKIGKEWLLITAGDSEKFNTMTASWGGVGVYWGKNVVTIYIRPQRYTKEFVDSNDTFTVAFFDETYREALNICGTISGRDINKIEKAGLTPYFVDDTVAFEEANMIIVCKKLYHDNMPPENFDAKENDKKWYPEKDYHTMYISEIIKVLVKE*

>CD196_RS12325 Clostridioides_difficile_CD196_NC_013315 MFS transporter

MGEKENGIKFGKLMFQLVIIAISWELVYIIPFIQYTLYDPILKALECTNTQLGFLLTIYGLGNIFGAPIGGWLADRFDYRKIILGSVFLNGVVSFLFAFNMNYPFAVATWVGCAITSLVMNYPSMVKILRVIGKDNQGKVYGFNEAMVGVSGVVMGAIFLYIYTCFSTPVLGMRWVMISLGILSIVMCPVLWFVIKDVDIREDKEEEASEKMSAGDFMTVLKSPNTWLVGISIFCVYSFTVTMSYFTPYITSVLGGSVALSGALAIIRQHGLKLFGAPFGGYCADKVKSPTKVLLPIYVFGIAVIILFLVLPASTPMTIFIALTFVVGILGYMGKGIYYAVQDEVKVPVKYSATTIGIAAALGFSPDVFQFALIGHWIDTYGNKGYTYTFIFQIAILIIGILSCLYILKVKKKRESKLEA*

>CD630_24360 Clostridioides_difficile_630_NC_009089 divalent cation transporter

VGYMDRKLDASQDLLYEVKSLIDNNKVLELRELIEEYHIIDIFDIMENLEEDMKIQLFEVLPLDMASSILEEGSVEFFISILSKLDVEHSKNILELMSLGDMADKLSELEEEEREHIINLLNQENADYVKELLFYDEDSAGGTMTTGYISINKDMTALEAIDHMREEAEEAETIYYIYVVDDEEKLVGVLSLRELIIARDANIVEDLMSENIISVYVDEDREEAVRLVSKYNLIAIPVVDRQEKLKGIITVDDIIDVMEEEATEDMYKFAGSSEHEREVAEKENPTLREQIISALRGRLPWLIITLVGGLLASLILSNLDYIMNPVYAPLVFFIPVVIGMGGNIGTQSSSVTVITLSNKDLNFSNVVREGIVGIITGLLCSIITGIVIYFVMRKLDIVLIVSISLFINMVLGATIGAFMPVLLKKMDADPSTVSSPIISTALDITGIAVYFIITTALLSKIV*

>CD630_24380 Clostridioides_difficile_630_NC_009089 cytidine deaminase

VDNIELLRLAEDARQHSYAPYSGFRVGAALLTKSGKVYTGCNIECASLGGTNCAERTAIFKAISEGDKDIYKIAIASDNSENNEQTYPCGICRQVIIEFGSDIKIITGYTKGEVFEHSIKDLLPNYFSGNDLK*

>CD630_24390 Clostridioides_difficile_630_NC_009089 diacylglycerol kinase

MKPEKTRQGIIKAFNAAIEGILYTFKFERNMKIHYLGSVAVLIISLFFNFSKLEMIMLLMSICLVVVAEMFNTAIEKAVDLVTDEYHVLAKIAKDVAAGGVLVAALNSVVVGYILFYDKLTDISGILIYKIRESELHITLICILLVLIAVVVVKALTSTGTPLKGGMPSGHAALAFAIATAITLMTERVVASTLAYIMAVLVAQSRIEGKIHTFWETIAGALLGVLIAILVFQLGMFYN*

>CD630_24400 Clostridioides_difficile_630_NC_009089 rRNA maturation factor YbeY

MDLILDDRQDKLEVSEELIEKIKDIIIECLDYEGYDDNYEVSLSFVDNKEIHELNREYRGVDRVTDVLSFPLLSDDFEDVELEEESLGDIVVSLERALEQSIEYNHSFEREVCFLICHSMFHLLGYDHDTDENTKEMREKEEHILNKLNITRE*

>CDIF1296T_02562 Clostridioides_difficile_ATCC_9689__DSM_1296_strain_DSM1296_CP011968 PhoH-like protein

MKIIVIKIVIIVKIIIAITTNKIKLKILGGLIQLIIQKKFTIADGNFERELFGNFDENVKLIEKTLNIDVILREGNIILIGEEKNVDSALKLMNELHQTVSNGKHLDKQSISYSLSLLLEGSEQKIKELEGTIVITQRGKAVQPKTLGQKEYIKLIENNDITFGVGPAGTGKTYLAVAMAVKAFKRDEVSRIILTRPAVEAGESLGFLPGDLKDKVDPYLRPLYDALFEMLGADKFNKYLERGTIEVAPLAFMRGRTLDNSFIILDEAQNTTSEQMKMFLTRLGFGSKAVVTGDVTQTDLPQNKKSGLIQATEILKGVPGIGSIMLTDRDVVRHELVQRIIRAYEKHDKREEFKKEERKKAKMQEKTFKRK*

>CD630_24470 Clostridioides_difficile_630_NC_009089 histidine triad (HIT) protein

MDCIFCKIANGEIPSTKVYEDDRVLAFNDLNPVAPYHILVVPKKHYDSLIDIPDKEMDIVSHIHVVINKIAKEKGFDQTGFRVINNCGSDGGQEVKHLHYHILAGKKLPNYEAGQN*

>CD630_24480 Clostridioides_difficile_630_NC_009089 MiaB-like tRNA modifying protein

LKKVAFYTLGCKVNQYETEAMLELFEKDGYEQVNSEEYADVYVINTCTVTHMSDRKSRQYIRRVKKKNPDAIIAVVGCYSQVSPEEILDIEEVNLVMGTNDRRKIVEEIKKINSSKKVSTVDDIMKVKAFEEIEISQTNGKTRAFMKIQDGCDRFCTYCIIPYARGRVRSRDIDSIVDEVKKLANNGYKEVVLTGIHVASYGKDLKDRDIKLLDVIKQINQIEKIERIRLSSVEPILFTDEFVNEVLKMDKVCPHYHLSLQSGCDETLKRMNRRYTTLEYKTIVDRLRSKMPDVAITTDVIVGFPGETNEEFKKTYEFLKEIELSQMHIFKYSPRKGTPAATMENQVDPQMKHFRSEQLLNLSKVNFNKFATKFIGRELDVLFEQNIEGNKYEGLTSNYIRVVVESDKNIQGQILKVKINDVKDEYVEGILL*

>CD630_24500 Clostridioides_difficile_630_NC_009089 50S ribosomal protein L11 methyltransferase

MNNWIEVTIKTTTEAVEPITNILYEQGAGGAVIEDPKDFLFQKKNELDWDYVEEEVFKKNEEDDVLIKTYVSEEKNVMEFVEIIKQKVLGLKDFGIDIGEGSVSLYQVNEADWANAWKAYYKPTKVGQRVVVKPTWEDYAMQEGDLIIELDPGMAFGTGTHETTSMCIRELEKYVNKDSKVFDIGCGSGILAIAAAKLGAKEVVAVDLDEVAVKVAKENVLENKVEKSVSVMHGNLTDVIKDKADVIVANIIADIIKILAKDVQNFMKEDAIFISSGIILDKVEEVKESLIENGFEIVEVQKLGEWSAIVSKLKK*

>CD630_24560 Clostridioides_difficile_630_NC_009089 sugar family ABC transporter ATP-binding protein

MSEVILKNISKLYSNGFNAVKNINIDIKDKEFIVLVGPSGCGKSTTLRMIAGLEEISEGELYIGDKLVNDIEPKDRDIAMVFQNYALYPHLSVYENMAFALKLRKLPKDEIDKKVKEAAKILDLLPLLNKKPKTLSGGQRQRVALGRAIVRNPKVFLMDEPLSNLDAKLRTAMRTEITKLHQQLGTTFIYVTHDQVEAMTMADRIVVMKDGVVQQIATPQDVYDYPANIFVAGFIGAPQMNFIDVILIEENDEIYAQNEHIKLKLNKEKHYDLIKDNYINNEVVIGIRPEDIHVEDTFIKSNPDTCFKSRIEIKELMGAETYAHLKLGENTITIRFDSKNRINVGDDLILSVDNSRVHIFDKETTLAIR*

>CD630_24590 Clostridioides_difficile_630_NC_009089 ROK family glucokinase

MYYIGVDIGGTGIQAGVVDNYGKIIFRSECKTVIEKGFEGILNDIKIMIYKLLEDNKLTMSDIKSIGFGVPGFINKEGLVTCVNLKWNKKAFNKELKRRFPDVEIHGENDATVAALGEAKFGSMKGANVGVLYTLGTGVGGGIVINQKVFSGAHGLGSEIGHQIIGENYFNCNCGNNGCVETFCSATAIIKYSQKLIEEGEKSRILDLAEGNLENVNAKMVFDAYRENDLVAIKVINRFKEYLAKTFANTINSLDPEIISIGGGISKSSDIILDGIEDLVRKFVLYKTEDIATITCATLGSDAGIIGAAFL*

>CD630_24670 Clostridioides_difficile_630_NC_009089 elongation factor 4

VDNKQSRTRNFSIIAHIDHGKSTLADRLIQQTGLVSERDMKSQLLDNMDLERERGITIKLQNIRLMYKAKDGNEYYLNLIDTPGHVDFNYEVSRSLAACEGALLVVDAAQGVEAQTLANVYLAIDQDLEILPIINKIDLPSARPEEVKNEIEDLIGLDSSEAPLISAKTGLNIEDVLEDIVKNVPPPKGDNEAPLKALIFDSYYDAYKGVVAYVRVFEGTVKKGMTIKMMNTNKKFEVTEVGVMAPGQTELSELSAGDVGYIAASIKDIRSCRVGDTITDSNNPTEEPLPGYKKATPMVYCGIYPGEGEKYENVRDALEKLQVNDAALEYEAETSAALGFGFRCGFLGLLHMEIMQERLEREFNLDIITTAPSVIYRVTKMDGEVVMIQNPANLPEPSEIKMIEEPIVKGDIIVPKDYVGVVMELCQERRGNMLNMEYIDERRVMLHYDLPLNEVVYDFFDALKSRTRGYGSLDYEVKGYVASTLVKLDILINKEQVDALSFIVHETRAFPRGKAMCEKLKGEIPRHQFAIPIQAAVGNKVIARETISALRKDVLAKCYGGDISRKKKLLEKQKEGKKRMRQIGSVEVPQKAFMSVLKLDE*

>CD630_24700 Clostridioides_difficile_630_NC_009089 germination protease

MISVRTDLALEASEMCEKSQEGSSIPGVKIETKELENCIVTKVEVIDRQGAEIMNKDIGKYITLESNLMKFDDDESREEMISYLKEELVDIFGQDKNKKTLVIGLGNRNITSDALGPKSVSKTLVTRHLFKNYNKDYDDDFTEVSALSPGVMGVTGIETSEIVKSLVEKVKPDRVVAIDALASRKMERVNSTIQISTAGISPGGGVGNTRKSLTKETLGVDVIAIGVPTVVDAATLTIDVLDMAIDNLIAQSEETESFYEMLKKLKEEEKYHLIKDSLDPYDKNLIVTPKDIDDTIENLSIIISEGLNRSLHPGRLV*

>CD630_24880 Clostridioides_difficile_630_NC_009089 PTS system fructose-like transporter subunit IIA

MGTNIFNKEYVFLNVDAKSKVEVLKFISKKAKDLNLAEDESLVYEGLMAREEQFTTNLGESIAIPHTKNDAIENPAVVVLKFNEDVVWNEGEDKVKLAISLLMPGKSKENIHLKLLSSLSRKLINKEFKDSLLKSDNVEEISNSINEALGL*

>CD630_24910 Clostridioides_difficile_630_NC_009089 mannose-6-phosphate isomerase

MEPLFLKPIFMDRIWGGTALKDKFNYEIDSPTTGECWAISSHKNGDCLIENGKYKGKKLSELWNKNRELFGNTPGDKFPLLTKILDANDNLSVQVHPNDEYAKKNENGELGKTECWYVIDCSDDAEIIIGHNAKSHKEFVNMVNNNEWDKLLRKVNIKKGDFFYVPSGTIHAICKGTLILETQQNSDTTYRVYDYDRTDNSGNKRELHVQKSIDVTNVPHINFDTDYKIVSTSDFKCTTFVSNEFFSVYKLDVFGKCNFTHNTPFSLYSVLDGNGKLIHNSVEYNLKKGMHFILPNDFGDFSFEGNLEIICSHI*

>CD630_25020 Clostridioides_difficile_630_NC_009089 pyridoxal phosphate-dependent transferase

MKLKINKAIEMQLKKSYAIESGHSEDVFEIDCGEGINTVSYSNKAVEAFNALKFDMIRGYPHSIALKDNIVDYWKDFIALDTDRICLADGSIHVIYLLNRLFIEKGDKVLGYSPQFSEYETDIKMHGATYDYVLLKKEDNFKFNEKEFIEKINPEYKVIYIDNPNNPTGQIIPLSSIENIVREAAKYDIAVMVDEAYGEYMPKENSAVKLLNNYDNVIALKTFSKGFGLAGLRAGYAVLPEQLVSPIKKISTPYEVSEISRSIAANLLDDVQFIEELKEKTKDIKNQLLIPWKNLNIAETSDTVSIMTVEHKNKDIDLQQEFAKLKIRVISGSDFTGLDKNFIRFRMPEEKELPEVIKAFQIIDNIE*

>CD630_25060 Clostridioides_difficile_630_NC_009089 major facilitator superfamily transporter

METQSNNKKGNLIIAIVMTGAFISSLSQTLLSTALPNIMSDFKITADIGQWLTTIYLLIAGIIVPTTAYLINRFSTRKLFITSMSIFSIGCIIALFSNNFSTMLIARVLQAMGSGSLMPLLQVIILYLCPEEKRGAAMSLVGITVGFAPAIGPTLSGWLVDSFGWHSLFLFLSPIAILDVILSFILLRNVGETQKLKLDIPSIVLSSLGFGGLLIGFTNQGNYGWTNIATYLPILIGIMSLILFTLRQLKSKEPFLELRVFKNKPFLISTILIMIVYASMMSATLMIPLYVQSVRGFSALSSGSLMLPGAILMVVLNPIAGRHLDKYGPHALSILGTGCLLLGTLSFAFLGRDTSLIHVSLMYCIRMIGISMVLMPLTTWGIKTLDRELISHATAINNTLRQISGAIGSAILITIMTSATKKAHMSSNMLSNIHGIDVAFSIAATLAFTGLIVSICFIKRHQIIRS*

>CD630_25080 Clostridioides_difficile_630_NC_009089 AraC family transcriptional regulator

MNIINKIFWQEDRIGMITNNLEADTHSHCMLQLFLGIEDSIEITVNEKLVKCNCIIVDKNISHSFSARKKVYYSAIIEPTSIYAEQLTSKMNDFGYWICDNNGLEKLRQQGTFLIDNSSKEQYLRFMEMLNNYLNIPITLKHYDDRITELLNLLRTCNCDNHTISSFADKVSLSASRLSHLFKEQIGIPLKSYILFHQMECAFRELLSGKNVTEASMTAGFDTPSHFAGTVKRMMGMPVSLSLKDSEFLKVY*

>CD630_25220 Clostridioides_difficile_630_NC_009089 ribosomal silencing factor RsfS

MTVEQMTKIAYDAIEDKLGQDTVIINIGKVSSLCDYFIITTASSQRQVKAIADNVEDELAKLGLEPRGKEGQGTQTWVLLDYGDIMVHVFNEENRGFYNLEKLWKDAPYIDIDTLA*

>CD630_25240 Clostridioides_difficile_630_NC_009089 nicotinic acid mononucleotide adenylyltransferase

MRSENLVKMAELKNTEKLEKFNRHKGKIKIGILGGTFDPIHYAHLATAEFIRDKYDIDKIIFIPSGNPPHKLCITTDKYDRYNMTLLATESNEDFLVSKVEIERKKRTYTIDTLKYLKKKYKNADIYFITGADAICSVEEWKDVKKNFELATFIAATRPGISLLRSQETIEKLTKKYNADIITVYVPSLDISSTYIREQLNEGKSIRYLVPENVENYLYENKLYQYGDD*

>CD630_25340 Clostridioides_difficile_630_NC_009089 ABC transporter ATP-binding protein

MKILYTENLSKHYGKGESLVRALDNVDLEINEGEFVAIIGKSGSGKSTLLHMIGGLDIPTSGKVYIDNKNIFTLKEEELAVFRRRKIGFIFQSYNLIPSLNVWENVVLPIGLDGREVDESFIKELLKSLGLENKHDVLPNTLSGGQQQRVAIARALATRPAIILADEPTGNLDSKTSDEVMSILKSMSKKYSQTLVMITHDDSIAQMADRVIFIEDGRVSKVGDKND*

>CD630_25390 Clostridioides_difficile_630_NC_009089 ribonuclease Z

MIDLTLLGCGGNVPMPNRFLSSVFINYKGRKILIDCGEGTQVSMKLKKCGFKDIDLICITHLHGDHIFGLLGLLSTIGNSGRTSDLTIVGPVGIVDCIRSMRNLVEYVPYTLKIIENPQGNFSLDNKVLRNLEISTISLEHSIECLGYSFNFRRNPKFDIDKATKNEVPKILWNKLQEGQNIVLDSKQYTPDMVLGELRKGVKISLTTDTRPIESIPDFIKDSDLFICEAMYGDDLDISKAVRNKHMTFREAANLAKLGNVKQLLLTHFSPSLDIPSMYLENATNVFENTILGEDRLSLRLNFDE*

>CD630_25400 Clostridioides_difficile_630_NC_009089 coenzyme A disulfide reductase

MRVIIIGGVAAGMSAAAKLKRIKPEYEVVVYEKTEIVSFGACGLPYFVGGFFNDADELLARTPEKLREAGIDLNIFREVVEVDSESKKIKVKNIKTDEIYEDYYDKLMIAAGARSIMPPIKNIKLKNVSTLKSLYDGEYLKKLLSNEDNKRVTIIGAGFIGLEAVEACKKLGKDVDVIQLEDRILPQVFDKEMTDVLEEEINRHNVNLHLDEMVVELCGEDKVEKVITNKGEIDTDVVIIATGVRPNTEFLSNTNIKMLKNGAIVVDEYGRTSVEDIYSAGDCATIKNIVSNENVYVPLATGANKLGRIVGENLAGREVSYQGSLSSSCIKIMDMEAASTGITEKQAKDLGINVKSKFISDYNQTHYYPGRNKIYVKLIYDADTKVILGGQVAGFKDAVQRANVLAAAIFGKMTTNQLGMLDLCYAPPFARTWDVLNVAGNVSK*

>CDIF1296T_02671 Clostridioides_difficile_ATCC_9689__DSM_1296_strain_DSM1296_CP011968 hypothetical protein

MSLNPWNIFRRDEMKTLKVFFCSTKYLILVFIFFINTTFISSATSDKILDLSFKKIETDLSSKITYEDTGVRIETDSSKSDKERYLYIYQNIKENWSMYNNFYIEIQNKNKSSQRINLSIQSKNMLEFRLKEGSEVFLEGKNIIYSDKIKEGCIEVPGEFEGKIYVNFNSLINEESNVVLDSNMLSNIVSWGITFIPSDEEHNIVIIKKISLLSEEKLRFLNNIKIIGDEEVQIPVLGQSISQYEVLGLKSDSKIKYSLMGKQDNVSISQKGKLTLSNKAKPGQIILQVNVDDKFKIGKKITLTESWSVNKKDKDGVPYTLVYPEQSPTVQDMKKINFMNNIITFVRILFVSLVIICFGIYLYWKKCSKTK*

>PCZ31_RS09270 Peptoclostridium_difficile_strain_Z31_NZ_CP013196 ATP-dependent DNA helicase RecG

IYLLDLYKDVQYVKGIGPKKADKLNKLGIFTLKDLLYYFPRQFEDRNNLKKIAQLEDGEKVTIKAVISSINTFSPKEGMTLTKIDVKDETGSAKLVFFNKSYIKNTFRPGDSILVFGKVKKKFNNLELTSCELEYLTNSPKNTCRFMPVYQLTYGVTNKEIMSIIRTVLEDKELIIQEYMPQRIIEKYRLCSIDFAVRNIHSPSSKESLKIALYRIVFEELLILQLGLFVFKSGRNKEDGIKFETSKDLKKIISALPFKLTKAQNRALDEIIQDMNLEKIMNRLVQGDVGSGKTVVALLALANCVLNGYQGALMAPTEILAGQHYISLTESLKDFGINVGLLIGSLAKKQKDTVLEQIKNNEIDILIGTHALIEDKVEFNNIGLVITDEQHRFGVMQRSKLSLKGANPDILVMTATPIPRTLALILYGDLDISIIDELPPGRQPIETIAIEKSKRDRAYNNLVRREVESGRQVYIVCPLVEESEAIEAKSAVELVEELRAEYFHDLRLGLLHGKMKSSEKDEVMRLFKDKEIDILVSTTVIEVGVNVPNATLMIIENAERFGLAQLHQLRGRVGRGSHKSYCVLIYDSKTDVCRQRMAIMEETNDGFKISEKDLEIRGPGEFFGTRQHGLPELKVANLFKHIKILKLAQQEARYILGEDNNLQLKENMALKKEIIDKFKDTLKEISLN*

>CD630_25690 Clostridioides_difficile_630_NC_009089 alpha-mannosidase

MKKTKVHVVPHSHWDREWYFTTSRSKIYLMKDFQDILDILEEKEDFKYFTMDAQASLLDDYLKWRPQDEYRIRDLVQRRRLVIGPWYTQTDQLVISGESIIRNLYYGIDRCNEFGEPMRVGYVPDSFGQSAQMPQIYRGFGIDSSLFWRGVSDDMVDTTEFIWKGSDGSKVLAVQIPFGYYYGGNIPEDDSDLKEYLKDIIGKLKVKASTNNVYFPNGFDQAPIRKNLPDILKKANEIDNENEYEISDIENYIDSVRNERNEFTELKGEFLNAKHMRIHKSIFSSRSDLKIMNNKLENYITNVLEPLLTLSYSLGNEYPHLVIKDIWKLMFENAAHDSIGSCNSDTTNEDVYTRYKQARDLSINLLDLHMRLISTQIKNNNEEITLTVFNGFPEVRNEVVEFDTYIPGQDFVIKNKNGKILNYIIKEKEDITNYILTQTIRLNPSKKIYIPKTVYRAKIALSVKDIPPMGYTQLVFELDTKNKCEIETSNCKEIENKYYKITANKNGSLEILDKESNKTYKNQAVIEDNGDDGDSYNYSPPRKDIYVSSLDSVSSVEVLKSNIQEEMILKYSLTIPTSLEERAIGKVSVKMPVTMKITLEPKEQIIKFNVNIKNNQALSHRVRVLFNTEIASKFSIADQQFGIIQRPTVLEKDLALWKRDNYSWQEKPITIEPMQSFVTLEDGQRGIALITGCVREYQIVGDNLDTIALTLFRSFGYMGRENLLYRPGRASGEKIIATPDAQLLKELNFDFGLYIYNNKFDEANVANTAKRFLTPIQIYEYADFLNGRLIFAFNDEKQIYENEFSLMNVNNSNFTVSAIKKEEKGDGIVVRIFNGMKNEDAKGSLNINKNVNDAYSAMLDEIYDNTSKLKVNTKTVEVNSLSHCKVQTIVIK*

>CD630_25720 Clostridioides_difficile_630_NC_009089 nitroreductase

MELQDTIFKRQSVRKFKNQDVSDEDILKMIKAAGAAPSGKNIQNWHFVVIKRRDLMEKIADVITKKQQEILVEMDKVSVDKANRFRKFVKNFTLFYLKAPVLVLVFTKVYNPSGYYELELIDAPKETIDKLFIRNPGMQSLGAAIENFTLSAIELGYGSCWLTSQNYAADEIEAVLEAETGFEKGEYFLGAMLALGVPEDNLKSPSKKPVEEICTFIK*

>CD630_25740 Clostridioides_difficile_630_NC_009089 thiamine pyrophosphokinase

MKICIVLNGEIEDYEVTRDIIIKECYDCIICADGGANHTYKMEIMPDYILGDLDSVEEEKINFYKNKGVKFEKFPSKKDETDTELCLFLAKTLKANHIDFFGALGGRIDHTLANIKLLYYLKEDGIYSRILSDKEEMYIVENEEISLYGNPGDTISVIAINGDAKGVTLTGLEYPLDNYYMKYSVPIGISNVMLSNSCKIKVEQGCVLVVRNL*

>CD630_25760 Clostridioides_difficile_630_NC_009089 ribosome biogenesis GTPase

MLEGKIIKGISGFYYVDTYNGIYECKARGIFRKQKITPLVGDRVKISIVDEDEKKGILEEIDSRDTELIRPPIANVDKALIVFAIKNPKPNLSLLDRFIVLAEKENLETVIILTKADLDDNDTLETVKNIYELSGYKVIPVSNITKLNIDKVKEELKENVVVFAGPSGVGKSSLLNEIDENFKLQTGVVSDKIKRGKHTTRHAELLKLEFGGMVADTPGFSSLALEDIEEVELKDYFIEFDKFNDCKFGSKCIHENEPNCAIKEAVTNGEISKERYDSYIQLLHEIRQNNSRRY*

>CD630_25770 Clostridioides_difficile_630_NC_009089 3-oxoacyl-ACP reductase

MKKKTVLITGGARGIGKAMSKAFAKEGYNVLVNFNKSENEAKELYTILNEKNFSVKLFKANISNREDVEDMVDYCIKEFGGLDVLVNNAGVSQDKLFTDITDEDWDNMMNINLKGSFYCSQVALKYMISEKKGNIINISSIWGISGASCEVHYSITKAGIIGMTKALAKEVGPSNIRVNSIAPGVINTDMLSGYNEEDIDALVEETPLMRLGTPEDIANCAIFLASDKSNFITGQVISPNGGFVI*

>CD630_25790 Clostridioides_difficile_630_NC_009089 phosphatase

MVYSCASHIGKIRKNNEDYCEGEVIDTEHGPIGIFAIADGMGGHKKGEVASKLAVENIIDFLKENLLQHDNVKIDYIDDILKQAYNNVNSIVHKKSMEDIEFEGMGTTLVTAIVYNNVLYVANVGDSRCYLLTDEKFDKITIDHSVVEELMMAHVITEEEARRHPQRNRITRAIGTDDMVVVDIFKKEIKKSDIILLATDGLTGFIDDEDIRDLILDYEYERTSNISEELISMANDVSGKDNVSVIVIKV*

>CD630_25840 Clostridioides_difficile_630_NC_009089 methionyl-tRNA formyltransferase

MKIVFMGTPDIAVPCLQKIIDENYEILGVVTQPDKPKGRGKKLGMSPVKELAIENNIPVYQPVKARDKEFIDKIKSLNPDVIVVVAFGQILPKEILEIPKLGCINVHVSLLPKYRGAAPINWVIINGEEKTGVTTMYMDEGLDTGDMILKTEVNLDENITAGELHDKMMNIGAETLKETLRLIEEGNAPREVQNHEEFSYAPIMNKSLGNIDFSKSAREIHNLVRGVNPWPSAYTTYNDVIMKVWKTKVLDEKSTKDVGTIIDVSKDGIKVSTIDNVLLIEEIQMPNKKRMLVGEYIKGNTIETGLVLG*

>CD630_25850 Clostridioides_difficile_630_NC_009089 peptide deformylase 2

MALRQIVQIGEPVLRKKSKKVEKIDEKIIQLLDDMAETMYDADGVGLAAPQVGILKRVVVIDIGEELIELINPEIIETSGEQIDEEGCLSVVGEAGNVRRPNYVKVRALNRNGETIELEGEELLARAFCHEIDHLDGILFVDKIEK*

>CD630_25860 Clostridioides_difficile_630_NC_009089 primosomal protein N

MKKYAKVIVRSNTIYTDNLFTYQIPVFLSDVIKIGHRILVPFGKGNKPTEAFVFQFTDSLDEKIKIKEIIDILDENPIFRKEDLELVYWMKNRYLCTYIECINLIYPKGYKLNNYKVVLLGESLSGLNDIELKEKISTLSDKNREIVEKVIDSKGKIKVDKLKYIPNLNSSLYTMNKNGIIKLCWEYKNHKNEKKVCYISLSLESDKIDDYIEQNKINVGSKQKEILSFLKNNENVEINDLLDLLNASKQSINSLSKKKLITLEFKDYYREPKSIYKSVLKSIKLNNEQQEAVDEIKSNMFVDDKKTYLIHGVTGSGKTEVYMEIIEFALNQGLDSIFLVPEIALTPQTIDRLKIRFGDLVGVFHSKLSEGEKHDVYKAVKAGKVRVLIGARSALFAPFNSLGLIIIDECHESSYKSEKNPKFNAIEVARFMALKNNITLILGSATPSIEEYYRAKSGEYKLINIKSRANDKPLPNIEVVDMKDELDKGNRSIFSMKLQKEIRYAIEENNQVILFLNRRGYANFVSCRKCGYVFQCENCDISLTYHKKSNTGRCHYCGYEKEIPKECPECKSTYVKPFGVGTQKIEEELKYIFPDIKTLRMDKDTTSKKGALDEILNKFKDKEADVLIGTQMLSKGLDFENVTLVGILSADMILNFPDFKSFETTFQLITQVSGRAGRADKEGKVVLQTYDTEHYAIKHAIEYDYEGFYEDEIKIRKAFGYSPFNNMLSVVVSGEDERLVIKNIKNMHASLIYLLEKRGINDLGFILGPNPCSISKINQNYRWQILFKDENIEINLLKGIIKYICITKRDLIFDKNINVSIDINPNSVL*

>CD630_25910 Clostridioides_difficile_630_NC_009089 low specificity L-threonine aldolase

MRFIDLRSDTVTMPTDEMREAMAKAPVGDSIFRDDPTVNKLEELAAAKVGKEDAIFLPSGTFGNQLALFTHCLRGQEIIIGKGYHIVTHEVGAPAVIAGVQLRTIDEDESGALNPELVEKAIRRDDIHEPQTGLICVENAYSGGTVVSLDNLREIKKIAEKHNLPVHLDGARLFNAALTLGVEAKEIAKCCDSVMFCVSKGLAAPMGSILAGSKEFIAKARRKQKVMGGGMRQVGIVAAAGIIAIEKMTLRLNEDHENAKYLACELNKIDGIEVNNVNPDISMVFFKMSEDIIKEDVLIKEFFERNIKINKMENGEYRFVTHVDITKEDLDYVLKTLKELIGVS*

>PCZ31_RS09075 Peptoclostridium_difficile_strain_Z31_NZ_CP013196 RNA pseudouridine synthase

MFYFLKVKVGDIMDEVKEFLVLEEEEEVRLDIYLAEQLGDMSRSYIQKIIKDGKVKVNNKVEKARYLVKEEDNIVIEIPEPKVLEVLPQDIPIDIVYEDDDVLIINKPQDMVVHPAPGNYENTLVNGILYHCKDKLSSINGVIRPGIVHRIDKDTSGLLMIAKNNYAHNFLAEQLKEHTITREYEFICYGVVKEDKITVDKPIGRNPKDRLKMAVVKDGKNAITHFEVVERFDKFTHMRARLETGRTHQIRVHALSINHPLLGDYVYGPKETKFKLKGQTLHAKKLGFIHPTTKEYIEFNSELPEYFKEVIKKIK*

>CD630_26130 Clostridioides_difficile_630_NC_009089 M24 family peptidase

MKAQRLNAVLEQMKKDDISQMLVSDPTSIFYLTGVLIHPGERLLALYLNLNGNNKLFINELFPVSEDLGVEMVWFNDTQNPVEIITEHIDKNATMGVDKNWPARFLLNLIELGGGSKFVNSSYIIDTLRMCKDEEEKELMRIASKLNDKAMEQLKATVSGELTEKQLVGKLSKIYEDLGTDGFSFDPIIGFGPNGANPHGEPGNALVKPGDAIILDIGCIKDNYCADMTRTVFYKEIPEKGREIFEIVLEANKRAEAIVKPGVRFCDIDAAARDYITEKGYGQYFTHRTGHSIGLEVHDKGDVSSINTDTVQPGMIFSIEPGIYLPGEFGVRIEDLVLVTEDGCEILNKHDKEICVVG*

>CDIF1296T_02743 Clostridioides_difficile_ATCC_9689__DSM_1296_strain_DSM1296_CP011968 hypothetical protein

MYTVVNGVHEVLNNILGGIRIMLRERFVVRNHHNPHTSGCCGTGGEGCCGGDNHEHSHQHSHSHGHEHEHVNGVGHDHNHDDGFSGTHEHSHEHTHTHGHEHSDGHTHEHK*

>CDIF1296T_02745 Clostridioides_difficile_ATCC_9689__DSM_1296_strain_DSM1296_CP011968 isoleucyl-tRNA synthetase

VIYMAKFKPLVDSSVKQAEAQVFDYWKDINILEKTLEKGKDDPSFVFYEGPPTANGNPGVHHVLSRTLKDSVCRYKTMSGYQVKRKAGWDTHGLPVEIQVEKELGLTSKQQIEEYGIAEFNQKCRESVFSFEKQWRIMTERMAYEVDLDNPYITLDNNYIESVWWILNKFNKEGYIYEGHKILPYCPRCGTGLASHEVAQGYKEVKTNTVIAKFKKKDADEYFLAWTTTPWTLPSNVSLTVNADVDYLKVKKGDEVYYVSKPLADKVLGEDYEVLEEMKGKDLEGLEYEQLMPFVEVDKKAFFITVGDYVTTEDGTGIVHTAPAFGEDDYNTGKRYDLPVIQPVDETGKFTSTPWEGRFVMEDGLDVEIIKWLASENKLYSKEKVVHNYPHCWRCQTPLVYYAKPSWYIEMTKLKDKLIENNNGVKWFPSFVGEKRFGNWLENLNDWAISRTRYWGTPLPIWRCECGHTDSVGSRAELAEKAIEDVNPETVELHRPYVDDIHLKCEKCGKPMTRVTEVIDCWFDSGAMPFAQHHYPFENKENFDQLFPADYISEGIDQTRGWFYSLLAVSTFVLGKAPYKSVLVPDLVLDKDGKKMSKSRGNTVNPMELFDQYGADALRWYLLYVSPPWTPTKFDMDGLKEIQSKFIGTMKNVYNFFTLYANTDDINPTEFFVEYKDRPELDRWILSKFNNLMKDVEENLAIFELNKTLRMIQDFINDDLSNWYIRRSRRRFWATELTEDKKAVYNTTYELLHGLCRAIAPFAPYMSEEMYRNLTGEVSVHLAEYPKCNEELVDTKLEEKMDLAKNLVTLGRASREVERIKVRQPLQKVLVDGKFEDTISDVVDLIKEELNVKEVIFAKDLDEYMNFSLKPNFKEAGPLLGSKMNLFVGALSKLNAHETANKLEKGETLTVDLDGEAFEFNKDLVLIGITAKEGFNVSVENNLFVILDTKLTEELINEGYAREFISKVQQLRKSNNFEVLDNIVIEYCGDDEIAKAVDHFNEYIKSETLALEINRVDDKSLEEQNLNDHMTGIKVIKK*

>CD630_26320 Clostridioides_difficile_630_NC_009089 GTP-binding protein Der

MSISRPVVAVVGRPNVGKSTIFNKFAGKRISIVENTPGVTRDRIFAEVEWLDKYFTLVDTGGIEPDSEDIILSQMRNQAMLAMDMSHVILFIVDGKAGITAADKEIAQLLRKTKKPVILVVNKIDSQSQFDNIYDFYELGFGTPFAVSGANSMGFGDLLDEIVENFPAGLDTEYEEDIIRVAITGKPNAGKSSILNKILGEERVIVSPIAGTTRDAIDTYFEKNGQKFLLIDTAGLRRKSKIYETIEKYSVIRAMSAVDRADVVLIVIDALEGVTEQDTKVAGIAHDEGKGCIFVINKWDLIEKDNKTMSNYTKDIKEKFPFMMYAPIVFVSAKTNQRMNKILDTVEYVSNEHSKRISTSALNEVIGEAVMLNQPPSDKGRRLKIYYGTQTDIRPPKITLFINDKDLTHFSYQRYLENKIRENFGFEGTSIKFEYRQKNKK*

>CD630_26390 Clostridioides_difficile_630_NC_009089 hypothetical protein

MMKDYINIKNVQDIKEYVNLSGNCEELDEKLNSVNLVITCKNIDAKNREDMLKVCKECDLTFENLTSNSQIHSDIVNIVNKDTIGKRRDGDALITNLEKVPLLLFTADCVPISIIDAKNKAIGLAHAGWRGTFSNIGHKTIKLMSECYKTVPEDLVCIIGPSIGPCCYEVSEDLVEKFNTILTNNDEKFYIIKEGSYYLDLWKINEYMLRCSGVKKENIINLNLCTSCRADKFHSYRKHNKASERIGTVLQIK*

>CD630_26420 Clostridioides_difficile_630_NC_009089 sporulation sigma factor SigG

MQVNKVEICGVNTSELPVLKNKQMKELLLQIKNGDEEARQQFVRGNLRLVLSVIKKFNNRGENIDDLFQIGCIGLIKAIDNFDLSQNVRFSTYAVPMIIGEIRRYLRDNNPIRVSRSLKDIAYKALQVRERLIRTNSKEPTVSEIAKELELEVESVVMALDAIQDPISLFDPVYQDNGDAIFVMDQVQDKKDTDENWLQEISLKEAIKKLNSREKLVLDLRFYKGRTQIEVADEIGISQAQVSRIEKNALKNMRKYV*

>CD630_26540 Clostridioides_difficile_630_NC_009089 phospho-N-acetylmuramoyl-pentapeptide- transferase

MMLGITELTYTALIAFLIVIIIGPIFIPMLRKFKFGQTVRDDGPQTHLAKNGTPTMGGIIMIVAILITGLTRVKVSHDMAVGLICIAGFGFIGFLDDFIKIKLKRSLGLKAYQKIILQVALSFYVAFYQYTSSSSASQLMIPFTDFVINVGILYIPIMMFIIVAIVNAVNLTDGLDGLASGVTLIVSVFFMLFASSIAGNTEVAVLAAATVGACLGFLGFNSYPARVFMGDTGSMALGGAVVAFSVLTNSVLIIPIIGGIYFAEALSVLIQVGYFKATRKRFFKMAPIHHHFEQCGWPETRVVFIFWIITVVLAWISIIAVF*

>CD630_26600 Clostridioides_difficile_630_NC_009089 M16 family peptidase

MEKIVNDILKEEVYYEKLQNGLDVYFMPKRGFMKKYAILATNYGSNDLEFVPIGEDKKIRVNEGIAHFLEHKMFEQPDGGDAFDKFSKLGVNANAFTNFTMTAYLFSATENFYESLEHLIDYVQTPYFTDENVEKEKGIIAQEIKMYNDDPDWNVYFNCLKAMYVNYPARIDIAGTVDSIYKITKEELYKCYNTFYNPGNMALFVVGDLDVEKVIDVTKKSNNYKVDRLSKSIERFYPEEPESVKEKEVIEKFPISMPMFNIGFKDSNVGLKGKELLRKEIVTDILVGMLFKKGSKLYEDLYMQGLINENFGAGFSSQVDYAFSIIAGDSKEPKKVKEIILDYIEKSKKEGLSKEEFERTKKKKIGSFIKCFDSINFIGNSFISYVFKDINLLDYLDIIKDITFEEVEERLKEHFKEEYCVISIVEPK*

>CD630_26620 Clostridioides_difficile_630_NC_009089 GTP-binding protein YchF

MKLGIVGLPNVGKSTLFNAITQAGAESANYPFCTIDPNVGVVSVPDERLNKLQELYNSEKIVPTAIEFCDIAGLVRGASKGEGLGNKFLSHIREVDAIVHVVRCFEDENVVHVDGSVDPLRDIETINLELIFSDIEILERRINKTQKAAKADKTLGSELDLLKSIMSTLEESKCVRTMEFTEDEQTFVNSLDLLTSKPVIYASNVSEDDLADNGENNKYVQQVKAFAETEDAEVVVVCAQIEAEISELDSAEEKKEFLETLGLEQSGLDKLIKSSYALLGLISFLTAGPKEVRAWTIKVGSKAPQAGGKIHSDIERGFIRAETIAFNDLVEHGTMAAAKEKGLVKLEGKEYIVKDGDVILFRFNV*

>CD630_26700 Clostridioides_difficile_630_NC_009089 ABC transporter ATP-binding protein

LKKEESLLKVEGLKKYFEVKSGLFDKEIKCVKAVDGISFDLKKGETLAIVGESGCGKSTAGRTILRLIEKTEGKVEFNGVDIYKLEKEELRKLRPKMQMIFQDPYSSLNPRLNVEQIISEAVIEHNLVPKNEIKDNILNVVESCGLSKYHLNRKPGEFSGGQRQRIGIARALALNPEFIVCDEPVSALDVSIQSQIINLLMDLQDKYKLSYLFISHDLSVVNHIAHRVCVMYLGSIIEIATKGELYLHSQHPYTKALMSAVPIQDPTIKKNRIILKGDIPSPTNPPSGCKFHTRCPYCKEICKKEIPVLKEISRNHFVACHLV*

>PCZ31_RS04475 Peptoclostridium_difficile_strain_Z31_NZ_CP013196 peptide ABC transporter ATP-binding protein

MNREKILEIKNLKQYFHLDKSTTVKAVDDISFDIYKGEIFGLVGESGSGKSTTGKTIIRLHESTGGEVIYKGNCISDKKTYKFIKKDVNKSMQIIFQDSTSSLNPRMTIADIISEPLKIQGICKNKTDRMNKVYEMLKLVGLDRSYANKYPSDFSGGQRQRIGIARALSVDPEFIIADEPIASLDVSIQAQIVNLFKKLQQEKNLTCLFIAHDLSMVRHISDRIGVMYNGKLVELADSNELYNNPIHPYTKSLLSAIPVPDPRYAKSRNRIEYNSNGYDCSNEKSLSWIEVSDGHFVYSSKSEINKYQQNLKVV*

>CD630_26710 Clostridioides_difficile_630_NC_009089 ABC transporter ATP-binding protein

MSKALIEVKDLKVYFHTDKGIVKSVNEVSFNINEGETIGIVGESGCGKSVTAMSLMKLLPTSKIEGGEIIFRGKDILKMNEDELMGIRGNEISMIFQEPMTSLNPAFTIGSQIIEGIMIHQDLSKEEAKKKVIDMIKLVEIPRAEEIYNSYPHELSGGMRQRIMIAMALSCNPKLLIADEPTTALDVTIQAQILDIMKNIKEKLNTSIMMITHDLGVVAEMCDKVLVMYAGKIIEVAEVVELFKNPKHPYTIGLLKSKPVLGKNKDKRLYSIPGQVPNPIGMPDSCYFSDRCEKVCDKCRTQIPPLIELNSGHSIACWLYKKEEI*

>CD630_26760 Clostridioides_difficile_630_NC_009089 acetoacetyl-CoA thiolase 2

MKDVVIVSAVRTPIGSFGGVFKNTSAVQLGTIAVKEAISRVGLNLSEIDEVIIGNVLQTGLGQNVARQIAINAGIPNSVPSYTVNKLCGSGLKSVQLAAQSITSGENDVVIAGGTENMSQAPYIVPTARFGSKMGNITMVDSMLTDGLIDAFNQYHMGITAENIATKFEFTREMQDKLALESQNKAENAIKNNRFKEEIVPVDVLIRRGKIETIDKDEYPKLGMTFEGLSKLKPAFKKDGTVTAGNASGINDGAAMLILMSQQKADELGIRPLAKIKSYASAGVEPEVMGTGPIPATRKALKKAGLSINDIDLIEANEAFAAQALAVKNELQIDSSKLNVNGGAIALGHPIGASGARILVTLIYEMQKRKVETGLATLCIGGGQGISMVVSR*

>CD630_26780 Clostridioides_difficile_630_NC_009089 succinyl CoA:3-oxoacid CoA-transferase subunit B

MDKLEMQEYIANRVSKELKDGAVVNLGIGLPTKVANYIPDNVNVILQSENGFLGLGSAEDGNSSDETIVNAGGQPVTILPGGCFFDSATSFGIIRGGHVDITVLGALQVDKYGNIANYMIPGKMVPGMGGAMDLVTGAKKVIVAMEHTSKGSAKILNNCTLPLTATNAVDLIVTEMGVMEVTSDGILLKEINPAFTLDDVISATEAPLILSDSLNGKISVV*

>CD630_26890 Clostridioides_difficile_630_NC_009089 lipoprotein

LKKVVIIGGGPAGMIAASTACEKGYDVTLIEKNHKLGKKLAITGKGRCNITNACEIEELIENVPTNGKFLYSAFYTFTNDDVISMFNNLGVKTKTERGKRVFPESDKAFDIVNALERQLKSKKVNILLNSKVEKIISKNNKIEKVILNDKKEIKCDSVVVATGGLSYPLTGSTGDGYKFAISQGHTIIDTKPSLIGIEVQESFTKDLEKLSLRNVEIRVFNSKQKKVYSDFGELEFTRFGLDGPIIKSASCRMKDTRKENYTILLDLKPALDEEKLDKRVQKDFQKYTNKKFEKALDDLLPKKLIPIIINLSEINPNTVVHQISREQRKNLVHLLKNLKFTVKRYRPIEEAIITSGGVKVNEINSSTMESKLVEGLFFAGEVIDIDAYTGGFNLQIAFSTGYLAGFNC*

>CD630_26920 Clostridioides_difficile_630_NC_009089 threonine-phosphate decarboxylase

MSTNHGANLYSLSSKYGFSKEEFMDFSSNINPFGTSSLAKQYIVNNIDMVSMYPDPDYIDLKTSISNYCKCSIDNIVLGSGATELISSFIHTINPKQALLLSPAYSEYEKELSKINCSIEKYFAKEEDNFHINLENLIKTINAKDYDLVVICNPSNPTGFAFTKVEVREILKNTDSFLMIDETYVEFTDTDTYSCTQLVDDYSNLFVIRGTSKFFSTPGIRLGYGLISNTNVKNEINKNLDLWNINIIASKMGEIMFSDLDFISNTISLMNTERDYLLKELKNIKSLDIYNTKGNFILCKIKTKELTAKSLREQLLPQKIIIRDCCSFKGLDEYFFRVCILKPNENKLLISSLKSIFK*

>CD630_26960 Clostridioides_difficile_630_NC_009089 pyridoxal phosphate-dependent transferase

MSKYNFDRVIDRVGTDCVKWDFRTNCSTKAQKDGLPFWIADMDFECAEPIIEALRKKVEHKIFGYSSNDSDKYFNAVCDWYKRRFNWEINRKDIIFSPGVVPAVAILVRILTNSNEGVIVQKPVYYPFEAKIKSNNRDVVNNPLIYENGTYRMDYDDLEEKAKCSNNKVLILCSPHNPVGRVWREDELKKVVEICKKYDLWIIADEIHSDLIRKGFKHTPLQSLCPEYKDKIVTCIAPSKTFNLAGMQLSNIIVNNDELKKKYQEEVTAVGVATSPNPFAIVATIAAYNESEDWLNELNDYLDNNIQFIDEYLKENLPKVKLVYPEGTYLAWLDFTAYGLNEVELEDLMFKKANVLFDEGYIFGKEGIGFERINVACPQSLLKECMDRLKTIFENL*

>CD630_27030 Clostridioides_difficile_630_NC_009089 pentapeptide repeat-containing protein

MTKIIKVQKPKFTGELEIIENLEDILEDIFNDEKIFNKIIENEVISDLDSVRVSFESCIFRNVSFENCNLKKIDLLDVVFENCNLSNTFFDGGSIYRVEFKNSKLIGVRFDDCILKNVLFKDVLGKYSNFSFSKFKIVSILDSNFENSVFQEVKGDNLVFEYTNLKKANFNGTKLSGIDFTTNNIEGIEIGIDDIRGAIFDVSQAIDLTKLMGIIIK*

>CD630_27050 Clostridioides_difficile_630_NC_009089 amidohydrolase

MSQLLIKNAYLITMNADREVYENGSILIENNIIKEVGKFDINIVDKDAEIYDAKGKILMPGLVNTHVHLSQQLGRGLADDVVLLTWLRERIWPYESSFNYEDSLISSTACCVELIKSGVTTFLESGGQYVDAMVEAVDKTGLRACLAKSVMDTGDGLPEAWQKTTDEEINTQLDLFHKFNNTSNERIKIWFALRTIFNTTEELLVRTKELADKYNTGIHMHIAEIADEISFVKQNNGVGTVEYLDKLGVLGPNLLAAHTVWLTHKEIDLFRLHDVKVSHDPGSAMKVVLGFASIPEMLEKGIPVSIGTDGAPSNNRMDLMRDMYLTSLIHKGRTLNPTVVPAEEVLEMATINGAKCALLEKEIGSLEVGKKADMIILNPDTIHCLPMHNPIGNIVYSMTSENVDSTICDGKWLMKERKVLVVDESELLNKVKKQASKIKDKAEVKIPSKFKIIK*

>CD630_27080 Clostridioides_difficile_630_NC_009089 shikimate 5-dehydrogenase

MEISGRTGLFALIGTPVGHSKSPVMYNYSFKKLDLDYRYLAFDITVDKVKEALLAIKTFNIKGANVTMPCKSAVTEYMDELSPAARIIGACNTIVNDNGKLVGHITDGVGYVRNLKENGVEVKGKKITIMGAGGAATAIQVQCALDGAREISIFNPKDDFYKRAEQTVENIKKDVPECVVNLYDLEDTNKLYEEIESSDILTNATLIGMKPYDNETNIKDTSVLRKDLVVTDVVYNPKKTKMIEDAEANGCKAIGGLGMLLYQGAEAFNLYTGLEMPVEEVNELCFK*

>CD630_27140 Clostridioides_difficile_630_NC_009089 UDP-glucose 4-epimerase

MAVLVAGGAGYIGSHTAIELLESGYEVVIVDNLSNSNSIVVDRIKELSKKPVKFYNIDIRNKDEMHIVFKENNIESIIHFAALKAVGESVEKPIEYYSNNLISTLNLFELMREYGVKKFVFSSSATVYGDPHTCPILEDFPLSVTNPYGRTKLMIEQMLVDISKADKSLDIALLRYFNPVGAHKSGRIGEEPNGVPSNLMPYITKIAVGKLKELSVYGNDYPTHDGTGVRDYIHVLDLAAGHVKALQKLEENPGLVVYNLGTGKGYSVLDLVKAFSKASGKEIPYKIVGRRAGDVAMCYADSSKAEKELGWKAKYELEEMCEDSWRWQSMNPNGYEE*

>CDM68_RS13985 Clostridioides_difficile_M68_NC_017175 sporulation and cell division repeat protein

VKVLSENNIIRNTVLNVTASFLKQESKINEKLDGVLEKKFEKVEFNEAKYAELLKFNILFYKTLARNTEPLIGKWIVDKYIPEIDELEKDLELTTAKCRKYVNKAMKDGLDSLKANDLNSFLAYDKMELSERRRRLEKDYKVLNLYKDLLNISLRKISLEKKECGDLFLKNQADAKRELKREIIFCVNKILASNKDVVPNNVEENNEQDRVEIKAAEIEDLQLGKDNSENDMLLNEIKNSKVKHESKSNDIESKFIDKLNSLNNESISQTIHNEYKKLCGLEELHSVEGYGFGKEIIKDFACATVVLEFLKRRNRDLIEGAMRLTIIGEFGPENFKEFMDYVIKNKTEISEDTWNKGHSLIKDNYSELENHEIASKRTRRNKDIDIEEYIYMIKNADKDICFRSSISIEDDAEEETKEEVDSNSQDIGDVVEDKDITDKEYDSNKEDIIEPENKKSKKKAKLFGFIKKDNEEVEQEEENLNDISPDIILDKPVENHQVKSEEIEQNELKEIKQEETSQHIEEERSVKIEKPINNNLDEKVSSNNESKLEKESKNLEDKKAKETKEEKEIKEEKLENEKSVVIPIKKKENSNKKSKNSSKDKYIENKKEINNYISDKEDSLDDEEVVSKKSRLKETIIAVVIVAIVGVGYFITVGNNKKNDKEDIPKSSTQQQANNKLTEEEKKAQAEKEKKEAEEKAKAEKEAQEKAQAEKKAKEMEAYKDGKGVYYTVYAGSLKVEKTAKETAKEYEAKGISSTIIQENGYYKIKIGDYSQYGEAQEKCNELAKKSIDTYIAMYDKYYDYKLEELKESAPSLSAEELKQKYEDLRSELKNKSGYREYVKHLDKLYEEIVEGA*

>CD630_27390 Clostridioides_difficile_630_NC_009089 aspartate--tRNA ligase

LETLKGLKRTHYCGELREKNINEEVVLMGWVQKKRNLGGLVFVDLRDTSGLCQIVFDTDVDKEAFEKAEKLGAEFVIAVKGKVCERQSKNPNMPTGDIEIFATELRLLNKSETPPIYIKDDDDVSEALRLKYRYLDLRKPSMQRNLKLRHKVMNITRNYLSNNRFCEIETPFLIAPTPEGARDYLVPSRVNPGKFYALPQSPQLYKQLLMVSGMDRYFQIVKCFRDEDLRADRQPEFTQIDCEMSFVEQEDVMSMIEGLLEAIFKEVLDVELALPLPKMTYAEAMSKYGSDKPDTRFGYELTDISDVVCNCGFKVFADATQPGKSVRGINVKGKADDFTRKQISSLEEHAKTYRAKGLAWMKVGQEGVTSPIAKFFNEEEMNAILTRMNAEVGDLLLFVADKNSIVFDALGQVRLEVANRLNLLDKNVYNLLWVTEFPVFEEDEETGTFSAMHHPFTSPMDEDLDKLEEGDKSSLRAKAYDIVLNGYEIGGGSVRISNSDVQSRMFKALGFTEERANEKFGYLLEAFKYGTPPHAGLAFGLDRLVMLLAGTDNIREVIAFPKNQNAVCPMTNAPTLAEDEQLEELSIKVDIKDNE*

>CD630_27400 Clostridioides_difficile_630_NC_009089 histidine--tRNA ligase

MLTKAPRGTKDITPKEAYKWRYVENKFREICALYGYEEMVTPIFEHTELFKRSVGDTTDIVQKEMYSFKDKGDREITLKPEGTAGVVRAFIENKLYADTQPTKLFYVTPCFRYERPQAGRQRQFHQFGIEALGSDTPSMDAEIIALAVQFFNEVGLNDLVVSINSVGCPVCRKEYNALLKEYLDSKADILCDTCNERREKNPMRVIDCKNPTCKENIKDIPFIADHLCDDCKSHFDKLQEYLKEMNINFVIDKTIVRGLDYYRKTAFEIISNDIGAQSTVCGGGRYDGLVEQLGGPKGISGIGFGLGIERLLLTLEGNGIEIENPQSTDIFIVTIGEEANTRSFKLLKDLRQNHISADKDHIERSVKAQFKYSDKINSKFTIVIGDDELKNDTATLKNMKTSEQTTVKLSTLVEELKQKL*

>CD630_27470 Clostridioides_difficile_630_NC_009089 ubiquinone biosynthesis protein

LRISYRNLKRYREIGYVLIKYGFSFIVERLNIEGIAYKIPLFDPPEEIKNMTTGERMKRVLEELGPTYIKIGQILSTRKDLLDQDIIDEISKLRDDVEKFDSNIAIDIFKEEVGLSIEEIFLEFKEEPIAAASIGQVYEGVLKTGEEVIVKIQRPNIEKIIKSDLEILRTIANTLKDLKKDFNLDLVQMIEEFQTQLMRELDYTFEAINATKFSRIFKNSDEVYIPKVYSEYNTKKILVMEKVNGTKLSDVEKIRRLGYNTKTIVEIGVRSFFTQVLSHGFFHADPHPGNIFVVAKNKIAYIDFGMIGIIDNKTLNQLNEIALAGVEKNVDKIIYLLIEMDALNGEADIKGLRQDLLYLIHYYYDISIEKINVTDILNELFRFFRQYKIVMPAQFVTLAKTVITLEGTSRTLNTDFSFGSMGKEFMKHHYKSKFNPKNVVLSSRQNVEEILLDIKTIPKQLKAILKNIERNNIKMQIEDVKMTRLENCIIELTSQISLSLVLASIIVGSSLIIASPNIENNIWIKFTAIAGFFISFIIGLCLVIRSIRSKYKKD*

>CD630_27480 Clostridioides_difficile_630_NC_009089 acyltransferase

MIRDYLEDKPLIDESVFVAKSADVIGNVKIGKDSSIWYNAVVRGDEGPITIGENTNIQDCSIVHGDTETIIGNNVTVGHRSIVHGCKISDNVLIGMGSIILDNAEIGEYTLIGAGTLITSNKKFPPGVLIMGSPGKVVRELTEEDKKYIDESYEWYLEAAQNQKY*

>CD630_27490 Clostridioides_difficile_630_NC_009089 radical SAM family protein

MSMIHKFSMNGYNIVLDVNGGAVHVLDDVAYDLLDFYKEKSKEEILEILKSKYQEEKINEAYEEILNLEKEGLLYTEDTYQYHPSFVHREPVVKALCLNVAHDCNLKCKYCFAAQGDFGGEKELMSFEVGKAAIDYLIANSGSRKNLEIDFFGGEPLMNFEVVKQLVDYGRSVEKDYNKNIRFTITTNGVLLNDEIIDYINENMHNVVLSLDGRKEVNDNMRPTLNDKGSYDITLPRFKKLVEKRSKDKYYYIRGTFTRDNLDFSKDVMHFADLGFKLTSVEPVVGDESNPYALREEDLPKIFEEYEKFAVEYADRQLQGDGFKFFHFMIDLNQGPCVIKRITGCGAGNEYLSVTPNGDIYPCHQFVGNEEFKMANIFDEEIVLPENLKNMFREAHVYTKEECKQCWNKFYCSGGCHANAINFNSDISKPYELGCEMQRKRTECSIMIQAKLMLEGATN*

>CD630_27660 Clostridioides_difficile_630_NC_009089 LytR family transcriptional regulator

LSKLKKFVILLAFLVVIFPISVYGYFYYKLSAIHDSSISSDLLDNNDHKNEDGIINILLMGTDARPNEDSSRSDAMMILTIDNKHNDIKLTSLARDSYVDIPGHGKQKLTHAYAYGQADLLIQTIEENFNIDIQNYACVNFESFMYIIDAIGGVEVTVEKGEIRELNKFIPETYKWNKSDDKGSIQYIRNSGKQTLNGYQALSFARIRHNDTAFARDGRQRQIIQAIIKKTETLPVTKYPGLLDAVLPYVKTNMKPNAILSLGAQVLKMGDLNIKQFEFPIDDEIHSTGGIYGKAGWVLRFDPDTLDILHDFIFNDIEFKQ*

>CD630_27740 Clostridioides_difficile_630_NC_009089 family 2 glycosyl transferase

MKKNLVSIITPMYNSEKFIEATIKSVLNQTYQEWEMLIIDDCSTDNSPNIVKSYMQQDSRIKCIKTETNKGVSNARNLALSKATGQFIAFLDSDDQWNSSKLEKQVNFMLENDYVISFTSYELMDENDKKLNKVIKVPPNVDYRRLLKGNILGCLTVVIDKSKLDFEIRMSGVRHEDYVLWLSILKKGHIAHGINEVLALYRKSSNSLSGNKIKAAMWTWNIYRNIEKIPLYKAIYYFINYGINGIKKS*

>CD630_27780 Clostridioides_difficile_630_NC_009089 glycosyl transferase family protein

LIPKKIHYVWFGGPKGNIENICINSWKEKLPEYEIVEWNEKNFDIEKEIKGNKFLEECYKRKLWAFISDYTRIKVLYEQGGVYMDTDMQILKDITPLLENNRLICGYEDDREYINGAIIGVEKGHPFLKDLLEYYEKEVLTSSLFTIPKIMTHLMEKNYKKIDPNNYEEGIRVYDKEYFYPFGFKEDFTPECITENTFGIHWWGKSWAKKRNYFLESKHLTGVNKIWKCCKIFASNTLRS*

>CDIF1296T_02918 Clostridioides_difficile_ATCC_9689__DSM_1296_strain_DSM1296_CP011968 phosphomannomutase/phosphoglycerate mutase

MDYKNNYEMWLNSPYFDEQTKNELLSIKDDEKEIQDRFYKNLEFGTGGLRGIIGAGTNRINIYTVRRATLGVLNYIMKTQGEEGKQKGIVIAHDSRYMSREFCIEVAKTLSAYGVKAYIFEELKPTPELSFAVRYLKCAMGIVITASHNPKEYNGYKVYDSDGGQICIDMANDIIAEVNKIDDYSTIKSIDFKEALSKNLITILDNEVDDEFIKAVKKQVLRQNIIDEYGKKLKIIYTPIHGTGNKPVRKVLNECGFENVMVVKEQELPDSNFSTVKYPNPEEKSVFNIAIEMAKNNGTDLIIGTDPDCDRVGIVVKDSSGEYVVLNGNQVGSLLVRYILESLVEENKLPKNNPTIIKTIVTSELGAKIAKAYNVDCLNTLTGFKFIGEKIKAFEESNDRSFIMGYEESYGYLIGTHARDKDGVVSSLMICEMAAYYSSKGMNLYEALIDTYNKFGYYKEDLKSVTLKGIDGIKKIKEMMLYFRSVKIDNVADVKVDKILDYKDSVDDLPKSDVLKFLLEDGSWIAIRPSGTEPKIKFYFGANSDNQEDVEFKLNNLISYILNVVDSI*

>CD630_27820 Clostridioides_difficile_630_NC_009089 cell wall binding protein

MIKKISTILSLVLLISISSTIGVFADANPKRELIEGSIPEISTELNKRAFKDSKEVILVNEESIVDSISATPLAYSKNAPIVVTKSKNLGRVTRNYLKELGPEKVTIVGGLKAVSKDAERNIEKMGMKVERIRGKDRYDTSLKIAREMYRTVGFDEAFLLSSTTGLENAISVYSYAAKSGMPIIWAKDEGFEEQIDFLKGKNLKKIYALGDSKEFIAEIDSNLKNIEGIKQINKSSTNVDLIKKFYDEKDIKKIYTARLDFGSRSDVNEYISLGVVSAKENMPILICSDNLSRAQDKFLKDSNINDVVEVGYTVGDYSLFKSIFNLTFLSCIVLILLLLLITFRALRYESK*

>CD196_RS14155 Clostridioides_difficile_CD196_NC_013315 DUF5009 domain-containing protein

MGIGIECNKKINKSIDKTRIKKNNLNSNSNSNSNSNSNSNSNSNSNSNSNSNSNSNSNSNSNSNSNSNSNSNSNSNSNSNSNSNSNSNSNSNSNSNSNSNSNSNSNSNSNSNSNSNSNSNSNSNKISNNVVDSKLTNSRIKSIDIIRGLSIALMIVCNNPGTWMRMYPQLRHAVWHGVTLADFAFPFFVISLGVTIPISINSKLKNNKSTLSIILSIFKRSILLILFGFFLNYLGNPDLNSVRILGVLQRMGLVYFVTSLVYLLLKKLNVGSTATIITFLCIATFIIVGYYILAKPYGFELEGSLAQLVDLHFFKGHLYKPEFEPDGFLTSIVAISSGMLGCTMGCVLLKENIGEYKKFFKILVMSIILLIGAFIFNQYFPFNKRLWSSSFVLLMAGSYGMLLSIFYFICDIKNKSKIFTPIIALGSSPIFTYMCLEILSHVFWNVPKLTNKVDYPTTLVEWTTYELITPWAGTTWDSLIFSLLYVLFWVIVMSIMYKKKIFIKI*

>CD630_27870 Clostridioides_difficile_630_NC_009089 cell surface protein

MRKYKSKKLSKLLALLTVCFLIVSTIPVSAENHKTLDGVETAEYSESYLQYLEDVKNGDTAKYNGVIPFPHEMEGTTLRNKGRSSLPSAYKSSVAYNPMDLGLTTPAKNQGSLNTCWSFSGMSTLEAYLKLKGYGTYDLSEEHLRWWATGGKYGWNLDDMSGSSNVTAIGYLTAWAGPKLEKDIPYNLKSEAQGATKPSNMDTAPTQFNVTDVVRLNKDKETVKNAIMQYGSVTSGYAHYSTYFNKDETAYNCTNKRAPLNHAVAIVGWDDNYSKDNFASDVKPESNGAWLVKSSWGEFNSMKGFFWISYEDKTLLTDTDNYAMKSVSKPDSDKKMYQLEYAGLSKIMSNKVTAANVFDFSRDSEKLDSVMFETDSVGAKYEVYYAPVVNGVPQNNSMTKLASGTVSYSGYINVPTNSYSLPKGKGAIVVVIDNTANPNREKSTLAYETNIDAYYLYEAKANLGESYILQNNKFEDINTYSEFSPCNFVIKAITKTSSGQATSGESLTGADRYETAVKVSQKGWTSSQNAVLVNGDAIVDALTATPFTAAIDSPILLTGKDNLDSKTKAELQRLGTKKVYLIGGENSLSKNVQTQLSNMGISVERISGSDRYKTSISLAQKLNSIKSVSQVAVANGVNGLADAISVGAAAADNNMPIILTNEKSELQGADEFLNSSKITKSYIIGGTATLSSNLESKLSNPTRLAGSNRNETNAKIIDKFYPSSDLKYAFVVKDGSKSQGDLIDGLAVGALGAKTDSPVVLVGNKLDESQKNVLKSKKIETPIRVGGNGNESAFNELNTLLGK*

>TW87_RS17190 Clostridioides_difficile_strain_08ACD0030_NZ_CP010888 cell wall-binding repeat 2 family protein

MKVNKRVLSIGLTISLIMAGAPNINALSSIEKIQGKDRYETSALIADKQIYDTIILVNTDNSIVDGLSASGLSGVAKAPIMLVQRDKIPTDVEKRLKDVKNAYVIGTEDTIGKSVQNQLKNKGIEVKRIGGEDRIKTSYLIAKEISAIKPVNDGDKVFLVNGYTGEADAMSVSSVAARDGVPVILTDGKSIPFKVDGVQCYSLGSEEIMSNELVSKTNSVRIAGKDRFETNKKVIQRFYKGTKKFYVSQGYKLVDAVAGSPLAKDKPIVLVNDGSDKSVLRGADEVTSLGGMDKKVVDQCISSASDKNTMPTITANDVEISVGDKFDNSMLNIVATDYYGNDLKANIKGNVDINKAGTYVLNISVVDNLGQKSEISVNVKVVVNASTKDSNSYEFKAMVSNEMYDLVNSYRKEKGKKSLRELDSLAGMANAWSKYMEDKKVFAHEIDGKNAAEVFFGFGARSGENIAYLPMNVKSVYTSKDAKEMAESIFDLWKKSSKYNENMLKEEFYSFGFGMHVSSKGEVNATMEFLNS*

>CD630_28000 Clostridioides_difficile_630_NC_009089 membrane protein

LRGGFFMGKSNYIFKGLGYAYIITLAVLLVYNLFLTFTDIGGDNITMVSSFITTISAAIGGFYTSKHMKEKGLMYGLLVGLLYIVCIFLTVFLAQEKFVFEVGMIYKLLLISAAGGIGGVLGVNFK*

>CD630_28050 Clostridioides_difficile_630_NC_009089 Holliday junction ATP-dependent DNA helicase RuvB

MQGFEDENRIITSTMKMEDIDIENSLRPKTLEDYLGQEKSKEQLSIFIEAAKSRNEQLDHVLLYGPPGLGKTTLASIIANEMGVNLRITSGPAIERAGDLAAILTNLNENDVLFIDEIHRINRSVEEVLYPAMEDFCLDIIIGKGPSARSIRLDLPKFTLIGATTRAGMLTNPLRDRFGVICKLDYYTVDELSKIVLRSSSILDAEIQSNGALELAKRSRGTPRIANRLLKRVRDFAQVRADGKITDKVAKDALELLGVDSLGLDFVDEKLLMTIIEKFRGGPVGLDTLAASIGEDRNTIEDVYEPYLLQLGFINRGPRGRVAMPLAYEHLKIPYPNEK*

>UAB_RS0215400 Clostridioides_difficile_ATCC_43255_NZ_CM000604 2-hydroxy-3-oxopropionate reductase

MEGYKMKLGFIGLGIMGKPMAKNLLKDGFNLLVYDINKSAVDELISCGAKYASVLEMGQECDIVFTILPNGTIVQDILFGMDGLAKTLKEGSIVVDMSSVTPTESILCANKLKDMGLEFIDSPVSGGEPKAIDGTLAFMAGGKEEIYKKVEPFFNIMGSSSILIGDNGSGSVTKLTNQVIVNLTIAAVSEAFVLAAKAGADPEKVYKAIRGGLAGSTILDVKIPMIMNRDFKPGGKISINLKDIKNVMQTAHNLDVPLPMTSQLLEIMQTLKVHGHLEDDHSGIAQYFEKLAGVEIKKHNN*

>CD630_28170 Clostridioides_difficile_630_NC_009089 multidrug family ABC transporter ATP-binding protein/permease

MKKSKGFKKTIGRLLPFVKKYKFSFIIAIICIISAATMNALAPKTEGLIITQLTKDVISIAKGVPGASVNFDYVTKILVILACIYFANAIFTYTSSFLLTNAIQNTMRDLRNEVENKIRRLPISYFDSNSFGDVLSRISNDVDTISNALQQSFMQIVNSILVIILALSMMFTINIYMALIALFIIPISYFVSKFVVKKSQSRFSLQQNALGKLNGKVQEMYTGFNEIKLYGKEEDSIKEFKKVNQELCENGFKAQFISSMMNPMVSLVTYFGIAAVAVVGSIYAVSGGITVGNLQAFVRYIWQINQPLSQMTQLSTVIQSSFAAIERVFEILDEEEEIPDVENPVKIENVKGNVTFEHVNFGYGENSTLIEDLNAEVKSGQMVAIVGPTGAGKTTLINLLMRFYDVKKGAIKIDGVDIRDMKRKDLRSMFGMVLQDTWLFNGTIFENIEYGRFGATKEEIIQAAKVANVHHFITTLPDGYNMFLNEEASNISLGEKQLLTIARAFISDPSILILDEATSSVDTRLELMLQKAMRNLMNGRTSFVIAHRLSTIRNADLILVMNNGSIIEQGNHDELMEKGGFYEKLYNSQFADKESE*

>CD630_28180 Clostridioides_difficile_630_NC_009089 multidrug family ABC transporter ATP-binding protein/permease

MKLILSYLKNYKLLIVLNILAIFSFALVELGIPTIIAKIIDNGIANQNIAYIKQMGIVIVIISIIGVVGSILLGYCSAKVSTSVTRDIRNDIFEKSQEFSHTEYNKFGISSMITRTTNDAFQIQQFVNILLRTALLTPVMFIISIIMTIRTSVELSIVLAISVPFIIIGVAIIAKVSQAISSKQQKGLDKLNLISRENLTGIRVIRAFGNDDYETERFEKTNTYYANVSKKLFKLMSITQPAFFLLLNIAVLAVFWISSEKINIGELQVGQLVAFLEYLFHAMFSIMLFSMVFIMYPRAEVSANRIKELLNEEPLIKNPENGIKDTENKGIIEFDNVTFTYPDGEASVLKDISFTAKTGETVAFIGSTGSGKSTLINLIPRFYDVTEGSIKINGVDIREYDLKALRKKIGFIPQKSLLFTGSIANNIRFGKHKAGESELEYSAKVAQAYEFISKKPRKFDELISEGGANVSGGQKQRLSIARAIIRRPEIYIFDDSFSALDFKTDAILRAKLKKETKDAIVLIVAQRISSIIDADKIIVLNEGQVVGMGTHKELLKNCEIYYEIATSQLKKEELE*

>CD630_28280 Clostridioides_difficile_630_NC_009089 aspartate aminotransferase

VLHMISNKMQTLVANSSVIRAMFEEGKKLSDIYGEENVFDFSIGNPSVEPPETIKAVINDILNEESPNLVHGYMNNSGYEDVRDAIAEHINKKDGLNLTRENLIMTCGAAGGLNIILKTLLNPGDEVIAFAPYFGEYKNYTENYDGKLIEVPTNIETFEPDLDALKNAITPKTRALIINTPNNPTGVIYSEELLKNLGELLDSKQKEFNTSIYLISDEPYREIIYDGAKVPCVLKYYRNSFIGYSYSKSLSLPGERIGYIVANGQMDDFDDVMSSLNVANRILGFVNAPSLFQRVIARSLDAEVDVNIYKKNFDLLYNSLIDMGYSCVKPNGTFYLFPKAPIEDDKKFCNDAKQFNLLLVPGSSFGCPGHFRVSYCVSYDKVKSSLPAFEKLAKLYNLK*

>CD630_28320 Clostridioides_difficile_630_NC_009089 cation efflux protein

MFSKILVKTFIRDSENVQNTDVRNKYGYVAGVVGILSNLLLFVIKVFIGMLTSSIAIMADAFNNLSDMASSAITMIGFKLASKPADKEHPFGHGRIEYLSALIVAFMVMLVGLQFVKSSIERIVNPIPVKFEVIPLILLIASIMIKIWLSRFNKFMGNKIDSSALKAVSLDALGDVFTSSCVVISFIVARFTNFPIDGYVGIVVSLVILYAGFSLVKDTINPLLGEAPDEEMVNSIIELLLSYKYIIGTHDLIIHNYGVGRCIASIHAEIPSNIDIMEIHEIIDTAEREISEKLDIYLVIHMDPICLEDKEVMSAKKELEEILKKNSLVKSMHDFRIVGKGSKKNLIFDIVVNPSEFSKNMSEDDLKEDITKLVKEINPEYNCVIVVDKDFI*

>CD630_28330 Clostridioides_difficile_630_NC_009089 calcium-transporting ATPase

MRYYNKPTKEVLKYLKTNPEIGLDDNEVEERKLRYGLNEFTIKEGRTFWDELGESLTEPMILILIGAAVISSFVGELHDALGILGAIFIGISIGIITEGKSKKAAHALSKLTENIEVKVLRNGKIIKISKNDLVPGDIVYIETGDMIPADGRLIQSINLKLREDMLTGESDDVAKNADAVLDMEVVYSKTEIIEQDAIPAKQVNMVFGGTLVAYGRGIMVVTHTGDKTEMGKIAQNLSNEDQQTPLQIKLGKLGAKIAGISGIIATLLCMFMIIQMQRKGMLILDTSSVLSFLQSLEPAKNAYMVCIALIVATVPEGLPTMINITLAITMQKMAKINALVTKKEACETIGSVSVICSDKTGTLTQNKMMVEVAYVDGKYISGGEYQSNSYFEQNCIVNSTADIEKEDNSFKYIGSATECALLLYHNDKNYNEMRKQTYLISQIPFSSEEKKMSTLIRQEDSDILLSKGAPEVLLKKCSYVQQGKNIVPITPKVEKSILDEIKKLQIKSMRTLGFAYKKMSNSKTEVAMTSEGELNLIGNSRSYMKEDNLVFSGFVGIVDPLREGVKDSIDKAFNAGVDVKMLTGDNINTATAIGNELGLLNDGKKAVEATYIDVLTDKELREEIKGISIVARSKPDTKMRIVSALQKSGEVVAVTGDGINDAPALSQADVGIAMGISGTEVSKNAADIILTDDSFSTIVEGIKWGRGIYENFQRFIQFQLTVNIVAFIIAIISQLTGKDMPFTTIQLLWVNIIMDGPPALALGLEPVRDYVLKRKPINRHSGIIARSMFVNIIINAILIITIVFTQSAFNILGATSEEQGTVIFSLFAFSALFNALNCREFGLNSTIPNFFKNKLALQIIVVTGIIQIIFTQVFQSFFNSVSLDFDMWIKIILFASTILLSNEFVKLILRTMKNSRTMNFNSKN*

>CD630_28410 Clostridioides_difficile_630_NC_009089 amidohydrolase

MKAKIYYNGNIITMEDSICGDAILIKDKIIKKIGTKEEVFALKNKDTEIIDLQGKTLMPSFIDSHSHLIAFATTLKLVPLEDATSFKDIVKKIQDFKESNNIKKGDWIIGFSYDNNFLEENKHPDKSVLDSASSENPILISHASGHMGVANTLGLKQLGVTNETRDPEGGHIGRVEGSEEPNGYLEENAFFNVASKIKQPSSNEIFNSIEKAQNIYLSYGITTAQEGLMEENQFNILKAMANQNKLKMDVVGYVNLKKSKSVVDNNREFIKKYINRFKIGGYKIFLDGSPQGKTAWLSRPYENSDDGYCGYPIYKDEEVEKFIDISLKEKMQLLTHCNGDAAADQLIDAFEKVLNLKEQSSENNIRPVMIHAQTVRADQIDDMKVINMIPSYFVAHTYYWGDIHIKNLGEDRAFKISPLKTTIEKGLIYTLHQDTPVIAPNMLETVWCAVNRITKKGIQIGENEKISPLDALKGVTINAAYQYFEEDKKGSIKEGKLANLIILDENPLTIDPMKIKDIKVLQTIREGEVLYSLK*

>CD630_28530 Clostridioides_difficile_630_NC_009089 D-alanine--poly(phosphoribitol) ligase subunit 1

MKIIEGIKKYSNTDRTALMCNGDKLSYKDLNEYSDAISVFLKDVYKEEDTPIVIYGNKENMIMACMIGALKSGRAYVPLDISFPIDRVFEVTKEIKPKVLFNFSDERNFGDINVIDMDKLNYIINEYQGKSLDKENWVKDDENAYILFTSGSTGKPKGVQISSNNLDSFSDWISPYLNIDGSEKVIMNQPAYSFDLSVTTIYPGLIHGATLFSISKDVLADYKELFRQFSISDIAVWVSTPSFAGVCITEKEFNSKMLPNLESMIFIGEALSKNLTKELMSRFPNTRIINGYGPTEATVGVSVNDMTQKAIDDEKSLPVGYPMSNCKIKILDEDGNELKENEKGEIIIIGPSVSKGYFNNKEKTDEVFFYDEIDGVKWRAYKTGDMGYLLDGNIYYCGRKDFQIKLNGFRIEIEDIENNLRKVHNVKNAVVLPVYKDEKIAYLKGIVELNEKNDLSNIKNGMIIKKELGKYIPSYMIPRNISIISEFPTNINGKIDRKKLMEEI*

>CD630_28550 Clostridioides_difficile_630_NC_009089 NAD(P)-binding oxidoreductase

MMGKIRFGIVGTSNIANIFLRAASRVKDFELVAVYSRNLEKAREFGTLYGASIFFDDLEQMAKSKDIDAVYIASPNALHSKQTITCLKHKKHVLCEKSLASNLKEVKSMIKTAKDNNVLLMEAMRITCVPNFKAVKENLYKIGKIRRFFGSYCQYSSRYDKYKNGIIENAFKKELSNGALMDIGVYCIHPMVNLFGAPKTVKAVSHILQTGVDGEGSAVFQYDDMDAIIQYSKVADSYIPSEIQGEEGSIIIEKLNLFEKAIIKYRDGREEDISIPKEKAEEKPREIEGMYYELVEFISLINNNKIESNINSHENSIIVMEIMDEIRRQSSIVYPADSI*

>CD630_28570 Clostridioides_difficile_630_NC_009089 HAD superfamily hydrolase

MQKVEGIIFDMDGVLFDSERISLEFWMETFEKYGYTMTKEIYTSVMGRNRKGIIEGLTDIYDSSVPIIDLYDEKTKNMIEFMERKGAPIKLGVNELISFLKENGYKMAVATSTKRERAVKRLAKANLKDYFDAIVCGDDVVNSKPNPEIFLKAAKKINVNPKNCIVIEDSPMGVEAAYNGGIRCINVPDLKEPDEQIKSQSHKILENLLEVREYLKSLNSKECHNL*

>CD630_28590 Clostridioides_difficile_630_NC_009089 D-aminoacylase

LIYDLILKNGFIIDGTGNPGFYGDIAIKDNLIAKIDSKINSNINKEIDCCGKVMTPGFIDPHVHEEIVAILDGKFEKFLKQGVTTTINGNCGHSITPYSSENVYEYMYKNGLLLEEEKKYLIDKNKCWNNFTEYCDLISKSGISVNMGFLLGHGTIRWSVMGGSKDRTPTEKEKNEITDIINDGMKSGAFGISTGLAYIPSKYADIDELVDIARQIKEYDGIYTSHIRDYIGRYNAVKEAIEVGQKSGARVQVSHLSPVEIEAFDEILKARYNGVEIMVDTVPRSSGHCMKKKRVIQFIMAISSSLFELGIDGVMDALRNEEGRTLILKEAFILGDRGSIILLNTKDINMEKKSIREIATQKGIDEDKLLLDLLLDGDEELIFCLGGMYRADFPDKLHDDKIIDNPFVMVGSDCLFSVGGDMSWFELQRRGAFPIFFNMYRKSGVRLEEIVRRVTSLPARQFKIKNRGILKEGLIADIAVIDMDNYSYSRSEDIDFSKPQLLANGVEYVIVNGKIALENGEITENKCGEVLKR*

>CD630_28640 Clostridioides_difficile_630_NC_009089 hydrolase

MYYVYVNDINIAVYDLNPSAKKTVLFIHGWPLGHKIFEYQTNILPKLGYRTVSIDLRGFGKSDATSGGYTYSQLADDIYKVVHAIGLKDFTLVGFSMGGAIVLRYMSLFNGYGVSKLVLAAAAAPSFVQRPPEFPYGMTREDVNKLIAQACTDRPEMVTDFGEKVFASNPPESFRRWFNDIGFSASGIGTIGTAVSLRDEELFNDLKCVRVPTGIFHGKLDEICPYEFAVFMNEKIEDSILYTFEYSGHAIFYDELKLFNQEFLQFLEE*

>CD630_28650 Clostridioides_difficile_630_NC_009089 bacterioferritin

MSDAHYDMHKRNGYSSDEPYPEIKVLGPNKYYAELLMDDYAGVSSEFTSVNQYLYHNFDLDETHRELSEMWINISITEMLHMEILAKTIRLLGGNPVYRGSTSSCGAYWNGGFVCYGNSICNRLKLDLHLEHVAINNYYKDISLIEDPYIKAILNRIILDEKLHVSLFEKAIEKYCK*

>CD630_28680 Clostridioides_difficile_630_NC_009089 oxidoreductase

MNQEYVKGNPIFTNINKVPRQFPYLTDDIDTDVIIVGGGVTGCICAYYLAKNNIKSVILEKGRIAHGSTSVTTSLLQYELDDNLIDLTEVMTLKDALKAYNLCTSALEELDTFIELYGNKCDYAKRDTLLYTANKLEVKAIKEEYNLRKENGFDVEYIDESTNPFSFDLKSGLIAKNGGRELDPYKYSHHLIDVSLKNGLQVYENTEVKKVDLSNDKVTAEVSYGHKVHGKKLIVATGYNTSLFTKRNFATKSNTFNIATKPLKNIASWKNNILIRDNCDPYNYLRTTKDNRLIIGGEDVSFDDIENETLANEKYDILEQRLKSMFKDIKDIEVEYKYCGCFASTLDNLGFIGPDNKNNNLWYCLGYGANGILFAILGGIMLSELYLGKQNKNMKLFKVDRFDK*

>CD630_29561 Clostridioides_difficile_630_NC_009089 V-type ATP synthase subunit F

MYKVGVVGDKDSIMGFLALGIDIFPAYDSDEIKKSIHKLVEDEYAIIYITEQASLLAKESIAKYKDYQLPAIIVIPGIGGSMGLGMNEVRESSKRAIGADILFKE*

>CD630_29590 Clostridioides_difficile_630_NC_009089 V-type ATP synthase subunit K

MEFLNALGNGQFLAISGAAIAALFAGMGSAKGTSIAGQAAAGVVTEDPSKFGQLLVLQLLPGTQGLYGFIIAFLILSKAGIIGGDAIPTSAQGLQLLMAGLPIGLVGLVSGIAQGKAAAAGVGIVAKRPEELGKAITFAAIVETYALLGLLVSFLAYNGIAIG*

>CD630_29630 Clostridioides_difficile_630_NC_009089 hypothetical protein

MIDGKEVKQESGQKNLKFKIAIVVGVLFVAYIGMAIFFRNHFYFGTTINGVKASGKTVEQVNDLLASSTNSYTLNLEERNNKKEQIKAKDIDLKFTPDDKVQKIKDSQSAIGWIGGIFKSKEYNNMITLSYNKDLLEKRFNALSCFDSKNVVEPKSAYPKYDSTKNAYVIVPEVLGNKVKKDDFYKLVQNSINSGNTSISLDESKVYEAPNNTKDSEKLKQAIKTLDKYVGVVVTYDFSDRKETLDGSEINKWLKVDSEKNYDITFDNDAMVEYTRGLSKKYSTFGDSRPFKTATGKDITISGGIYGWLIDKKKEAEALVDVVKAGKNVTREPIYIQKAVSRNKDDIGNTYVEISLSGQHMWFFKNGEMLVSTDVVTGDLARGFATPSGIYPLNYKARNVSLTGQGYSSPVSYWLPFNGNIGIHDATWRNSFGGSIYKSSGSHGCVNTPFSKAKKIYENIEPGTPIILY*

>CD630_29670 Clostridioides_difficile_630_NC_009089 dipicolinate synthase subunit B

MRLEGLTIGVGFTGSFCTYDKIFIELENLVKEGANVHTIFSDVSQNIDCRFGNSEEFMKKAYELTGNKPIVTIEEAEPFGPKGIADIIIIAPCTGNTAAKLANGITDSPVLMAAKGHLRNDKPLVISISTNDALSFNFKNIGILLNSKNIYFVPFGQDNCKAKPNSMIAHTELIIPTIELALENKQLQPVIKSPHQ*

>CD630_29850 Clostridioides_difficile_630_NC_009089 ABC transporter ATP-binding protein

MKILTVNNLSKVYGKKIIFNALNDINFSIEDGEFVGIMGPSGSGKTTLLNMISTIDKPTTGTMELKGKNPLLLRGEELALFRRRELGFVFQDFNLLDTLTIGENIVLPLTLDKVSVKEQDERLNEVSTILGIKDLLGKRTFEVSGGQAQRTAIARALINNPSILLADEPTGNLDSKSSKVVMELFQKINKENKVTTMMVTHDPLAASYCSRILFIKDGSIYNEIYKGSSREQFYQEIMDVLTLLGGDN*

>CD630_29890 Clostridioides_difficile_630_NC_009089 sulfonate family ABC transporter substrate-binding protein

MKLKKLLIGITILTLATASLAGCAKKNNGEKLSEINLTYVKSPLNVPSIIQKQDDLFGKEFKKDNIKVNFHEITTGPEQTQALAAGEIDFLHALGGTSALIAASNGVELKILNTYSRSPKGFMILTNNNSIKSAADLVGKKVAGPKGTILHQVLISALDKEGLSMDDVEFVNMGIPEASAALSDGSVDAALIAGPAALKAMKSGSKLVANGEGLVDGIIVTAVSTDFAEKHPEIVERFMKVEKETLEYVNNNFDEAMEKVAKEVDLSLEETKELYAWYDFSLDITDKDISSLEDTQDFLIKNKLQEKKVNIKELIYNAK*

>CD630_29960 Clostridioides_difficile_630_NC_009089 hypothetical protein

MKTISKQELEKTLEKHYLWLKNNNAGEKADLKELRLENMDLRGYCLSNIDFSWSDLINLNLEKADLENSIFNNSYLSDCSLKNSILKNADLSGASFRFCDLSNSDIRGANLENSNLEYATLNGVISDESTRFFKLYCPETGAFIGYKKCFNFRMVQLLIPSDAKRVSATSNACRCDKAKVLSITSVDGKESFKSARAYADENFIYRVGETIVAEDFNENRWKESTTGIHFFLTREEAIGYL*

>CD630_29970 Clostridioides_difficile_630_NC_009089 iron family ABC transporter ATP-binding protein

MLKTNNLSVGYNNKVVISNINVEVKNGEILCLLGSNGAGKTTLLRSLSKLISPIKGEIYLNGVNINCISRKALSKKMALVLTNRLLGDLMTVQDIVNIGRYPYTGFFGSLSKKDLIMVDEALESVDALHLKKRYFDELSDGEKQKVLVARALVQEPEIIILDEPTTHLDIKHRLELINILKKLSKEKSISVILSLHEIDIALKSCDKVALIKNNKVIAYGQPEDVVDEDIINSLYELDDKNFNSLLGSVEISNKSKNEVFIIGGGGKATPIYRAFTKKGIGLYSGIIHENDIDYEIGRTMGIKMFTENPFEPISDESFDLAIRNLNDSKIIIDTGFSVGETNKRNIDIIKEALKLDKKVYSFRNRDESKKYYDSLDNKIEHIDKVSQIINSADINNLL*

>CD630_30010 Clostridioides_difficile_630_NC_009089 hydrolase/isomerase/hydratase

MKFVSFTEGENECKRTGVFSKDELFIIDVNSLNLSRNFKDLNELIQNVSSEDIIKLKTIINESTFYNYDVFKRERCTVHVPIEKPIHDILCVGVNYKDHLEETQTHFDDSFEEPQKTVYFTKRVTKAIGPEDEIDGHFEINNQLDYEVELGVVIGKTGVNIRKEEVEDYIFGYTVINDISARKLQKEHVQWFRGKGLDTFTSLGPCILHKSAVPFPIKLKVCSRVNGEERQSSNTGLFISDVSDIVSELSKGMTLEAGDIIATGTPSGVGMGFSPPRYMSSGDVVECEIESIGILKNYIK*

>CD630_30020 Clostridioides_difficile_630_NC_009089 D-galactate dehydratase/altronate hydrolase

MKFYGYKRPDGRVGIRNHILILPTSVCASDTTRIIASQVQGAVTFNNQNGCSQVHSDQQLTMDVMAGYAANPNVYGVIVVSLGCENCQNDLVVDAIKERTNKPIKTLVIQEEHGTLKTIEKAVRYAREMAQEASLLRKEEFPISELILGTECGGSDPTSGLAANPLIGELSDKLVDLGATSILSETTEFIGAEHILARRAVNEEVKEKILHIVHRYENSLKLVGEEVREGNPSPGNIAGGLTSLEEKSLGCIHKGGHRQISEVYDYAKQIDKKGLVIMDTPGNDASSVAGMVAGGAQIVVFSTGRGTPSGNPIAPVIKITGNKITFANMEDNMDFDASPVIYGPQTMEELTDDLLNMVVDVANGKQSKAESLGYTEMAIARVCNYV*

>CD630_30070 Clostridioides_difficile_630_NC_009089 hypothetical protein

VKLTKLNIKKYRAPIYKYALANVNLRSAKSTNSSIITVIPQGAKMEVLDEEDDWIKVMYNSQEGYVYKDLVSVSEYAWSNLNLREDKSTTSNIITVIPEKSRVEVLQVDGDWSKVVYDDKIGYVFNYFLSIDGNKPNELDYKYFYTDMIKFVNENNIKSTSDYLIVTDLRNKYTYIFKKDNGGWGQLYKWQCTIGKPETPTITGIFYISGRKPSFGTDEYSVKYATRIKGGYYYHSVLYDSTGSYIIDGRLGEALSHGCIRLSTENAKWIYDNIPDTTTVIIH*

>CD630_30090 Clostridioides_difficile_630_NC_009089 pyridine nucleotide-disulfide oxidoreductase

MKKTFDAIIIGFGKGGKTLAGDLANRGLKVALIEKSNKMYGGTCVNVACIPTKSLENSANSVKTKNINSWDEVQAEYEKAIDKKETLITKLREANYNKLNSNENVTIFTGMGTFIDEKTVQVKTENEIYELVADNIFINTGSRPFIPNIKGIENKNIVYDSESLMNLRTLPKKMTIIGAGFIGLEFAGIYSSFGAEVTILNSNNGILPNEDVEDSEEIIKLLAKRNVKIVNNANIKEIKEVSELAIVEYEVDGKSKELTSNMILVATGRKANTEGLGLENAGIELNERGFIKVSETLKTNKEHIWAIGDINGGPQFTYISLDDYRIVINQLFGDKTRTTNDRKNIPNSIFISPAFSRVGLNVKQAKEKGYEVLVAKMPVEAIPRAKQIGKADGFIKIVIDKKSNKILGASMICENSSEIIHLIQLAVDLEVEYTYLRDRVYAHPTMTEALNDILSPNMIKEV*

>CD630_30100 Clostridioides_difficile_630_NC_009089 cytochrome C assembly protein

LQNVNLFLVFIEGIVSFFSPCILPILPIYLSILSNSSVENLKEGKTSFIGSSLFKNTIFFALGISTTFFILGSSVKVLSMFFNENKDLIMFIGGIIIIIMGLFYMGIIKSSILNREKRFNVKFKEMKAITAFILGFTFSFGWTPCIGPILASVLVMVSSSSNHLSANLLIAVYTIGFILPFIITAMFYSKLFKTIDKIKSNMEIIKKIGGIILIVSGILMMVNGFGSISKHFNTSQNSKIESKQEENKRENSTDKEENSDGNDSQKDSNNDNNDKGSNDEDRIKSIDFTLTDQYGKTHKLSDYEGKVVFLNFWATWCPPCKEEMPYIEQLYKDYNKNNDDVVILGVASPNLGREGSREHVVNFLKDQGYTFPVVLDEDGALAYQYGINAFPTTFIIDKEGYVTQYIPGAMDKATMASFIENQRNK*

>CD630_30120 Clostridioides_difficile_630_NC_009089 alpha-mannosidase

MIKAHIVNHTHWDREWYFTSGDALVLSEQLFTDVIDELERNPDVSFVLDGQLSILDDYVQLHKEKIEIIKKLIKEDRLHIGPWFTQTDAFFARGESILRNLMIGIFESKKYGKYMKIGYLPDTFGFNAQMPILLKHVGLDNIFFWRGIHLGKQVQSPYFKWKSLNGESYIYAVNMPHGYGTGMLLEPSLKYVNGRLDPAIDFIKSYTDVDEVLIPSGNDQLNIIGDFKNKIKAINDIGKYSYITSSYQEFLKYIKSIESLESYRGEFREPVLARIHKSIGSSRMDIKLACDKLENKLIKRIEPLLVIAKKSKIEISNQLLINTWKKLLEGQAHDSLAGCVTDTVTDDILHRIREANEICDSIENTIVKKISEGLKLSKNDILVFNTEAKRFNGYKEIQVVSDSKNIYFKDNLDATIIEETYVKPRENVLEETPAGNIFIEEPGYYILKVRINVQLPALGYKVVSFELSDKEMVSLDKSNDTFISNDNYKIIYEDGSLNLQLKDGTYISNFLTLKDSGNAGDTYDFSPLKNDEDIKLSFDKVYTEKALGYEKMVVKGTTLLPLTLEDRKNKYLNGKLNVKVSIVLSKENPLMDVKLCVDNTIYNHRLRVHIKTDIKDNKNIASLPFGYITRENGVLDNWEDIYSEMPIDLEPLESNITLTNQNRSCTVFTRGIKEYQHIEDEIALTLLATTDELGKPDLLYRPGRASGDTTKKGHIRIKTDKAQMLKKLEFSFAIYMDSKSFDELEIANLTHNYLRECVNYQLQDYNFFLYRIDNKIQKSIVNKTVSRELEIISLPENYLISACYPSYYSKDKFIIRVENPSSNRMILDEQIFKNRNGKIVNAIEDVEEEQVYEIAPFDVISILLDL*

>CD630_30130 Clostridioides_difficile_630_NC_009089 PTS system mannose-specfic transporter subunit IIC

MEGNVMLKKIVSELKKHVLTGISYMIPLVIAGAMIMAISRVGGSFYNIPDIWDAKYAESASSIVRLLHDLDGFGGTALGLMFPVIAAFIGFSIADKLAIVPGLVGGMIAKDIGAGFLGALAVGLIAGYTCLFIKNHVKLPKSAASIVPIFIVPVFGTLITVVLINYVIGIPFATLNTGLENWLNGLSGTNQILMAAIIGAMMAVDLGGPVNKAALTTSLALLTSGIYAPNTAAMVGIVIPPLGLGLATILAKQKYNKQLREAGKSSLIMGLIGVSEGAIPFAVESPLKVIPSCVIGTAIASAMAVGLGSVNATPISGFYGWFTVENWPMYVLSIAVGTVIVAGLVIVLRGNPVIEDDEFEDDDFDEAEWES*

>CD630_30180 Clostridioides_difficile_630_NC_009089 oxidoreductase

MKTKEEYLKEVRNISEEYFRKGEFFCSEAVLQTINDALGQPLSPEITKLASGFPIGLGKAQCLCGAISGGEMALGIVYGRVHGEAMNPKMFEHAKGLHDYIKKEYGATCCRAITKKWDGDNFMSPERKEHCIKITGQVTEWVADKLIEDEKLNIK*

>CD630_30250 Clostridioides_difficile_630_NC_009089 ferredoxin, iron-sulfur domain-containing protein

MNDELKSILYNKGVDIVRFVDISEFPINQTQGFSKAILFCIGLSKKFITDIYNNLPTDSDEFLEKEEKVEELADWISKYIQNKGYRAYSQSEKNNLEHGYFEKAYINPEMQSGISPLPHKTIANISGIGFMGKNNLFVTEEYGCAFSMCTVLTDAPISVERYPLIDSKCMDCNVCVENCPAKAIHGNEWTLPGKRESIIDVSKCFCVLKCMMSCPWSLRYANQK*

>CD630_30360 Clostridioides_difficile_630_NC_009089 major facilitator superfamily transporter

MSSTVKKKGKFPMGFYICSTTFSFERAAYYASKYLIYIFLTTAIVHGGLGIDKGQAAIMQANLVAFTYLAPIIGGYISDRWIGARYTIPIGMLIMGVGYYLGSIATTVSMVNAMIILVSIGTAFFKGNVSAVNGQLFDSQEELDTAFSVQYSFVNIGSFIGTIAVGILYLKTFAKNGVLGFSQCFFIAAVLCVIGAIWFIYGWRFLGNAGKRPFKEGVVAEKVEEKDKSPLTSMDKKRIWAIILISFFSVIFWVFWYLTYLAVYDYGAAFVNMNVGGFDVPLAWFDSLNSLVCIVLGPVLGALWFKLASRPQGDMSLFKKTGLGLIFLGLAFLMLVGAEFSRGVGAPETAKASILWIIMFGILLSLGEMLFSPLGNSFVSKYAPKKLLGVLMGVWTFATFIAGKGYGYIYAFTLKFDMIKVYTIIPVILFVAAILLFLSDKTLSRLVEDDK*

>CDIF1296T_03118 Clostridioides_difficile_ATCC_9689__DSM_1296_strain_DSM1296_CP011968 regulatory protein

LSKLFFLLRMVMFLFKIDDYIMYGMTGVCKVMDITNERFTNGIKKEYYVLSPIYSNNTVIKIPVDNEKVPMRKLLSKINVLSLINDIPNMDTSWIDNEKLRSEQFKKILRGGKCEELIKLVRSIDNNREYVKSIGKKTHQADDNIMKEAERLLNEEFATILDISPNEVSSYISSHIPQ*

>CD630_30410 Clostridioides_difficile_630_NC_009089 proline iminopeptidae

MKITEGYMPFKGFKTYYRIVGENTEGKKPLVLLHGGPGSTHNYFEVLDKIAESGRQVIMYDQIGCGNSFVEGHPELFNADTWIEELIELRKHLGLDEIHLLGQSWGGMQAIWYAIEYKPKGIKSYILSSTLSSAKLWEKEQKRRISYMSEVDQKALLDAVNTGDYSSKEYNDALERFMEMYCAGEVTEDSPECLRRPKKSGSEAYIVGWGQNEFSPTGTLSGYEFTDRLHEIKEPCLVTSGAIDLCSPYIAKTMYDRIPNSKWELFEYSRHMPFVEENEKYIKVLTEWLNAND*

>CD630_30460 Clostridioides_difficile_630_NC_009089 N-acetylmuramic acid 6-phosphate etherase

MLIKTLENLVTEGRNKNTLQIDKEDTLGIIELINNEDKTVAYAVEEQKESIAKAVNIIVDRMKQGGRLFYIGAGTSGRIGILDATECPPTYGVDFELVQAIIAGGNQAIFKAIEGAEDDKELGKQDIIDRGVTSKDVICGIAASGRTPYVIGAMEYAKELGCAVLSITMNPNSEMSKKADLPINIIVGAEVIMGSTRMKSGTAQKMVCNMLTTASMVKMGKVYSNLMVDVKTSNEKLVERAKRIIMIATNVKYDVAEKFLEEADNSVKLAIFMIKSGLDKDSAKSILDRQEGYISEALKSIEKL*

>CDM68_RS15380 Clostridioides_difficile_M68_NC_017175 PTS sugar transporter subunit IIB

MRVLFVCSSGMSSAIAVNALQKEGAKNGIDIDVLAVGTQEFEDEVKNGWDIAMVAPQVRHRFDYLKAFADEAGVPCALIQAQAYSPLGGPKLLKQVQELLSKY*

>CD630_30540 Clostridioides_difficile_630_NC_009089 two-component response regulator

MYRILLVEDDIDLSKEIALALEKWGFKVELIDDFEVVLDEFIDKKPDVVLLDVNLPLYNGFYWCEKIRAISNVPLIFLSSRDSDMDLIMGINNGADDYITKPFSIEILVTKINGIIRRVYNYSDSNSILYCEDLMFDVGKGIIKHKYKDKSIELTKNEIKILTLLLKNKNRVVSRESLMMTLWDNDEFVTDNALTVNMNRLRSKVKELGFDDFIKTKKGIGYIIQC*

>CD630_30560 Clostridioides_difficile_630_NC_009089 ABC transporter ATP-binding protein

MKEILKIKNISKDYGIKGFKTNVLKNISLTVNEGDFIAIMGPSGAGKTTLLNLMSTLDKQTSGEIILDGINISKVKNNELSKLRREKIGFIFQDYNLLDNMKLMDNIALPLALGKKKSKEIEAKVFSIAKKFGLENHLDKYPYQLSGGQKQRGAAARSLITNPTVIFADEPTGALDSKSAYELLESLEKINRENNATIIMITHDPLTASYSNEVYMINDGNIKCKLNKGNSRKEFYGKIMDMLASMGGEM*

>CD630_30640 Clostridioides_difficile_630_NC_009089 xylose isomerase

MSEIFKGIGQIKFEGVKSDNELAFRYYNPEQVVGNKTMKEHLRFAMSYWHTLCGEGNDPFGVGTVERPWNNITDPIEIAKIKVDAGFEFMSKMGIEYFCFHDRDIAPEGRDLEETNKILDEIVEYIKVNMEKTGIKLLWGTANMFGNPRFVHGASTTCNADVYAYAAAQVKKAMEITKYLGGENFVFWGGREGYETLLNTNTELEMDNFARFLQMAVDYAKEIGFTGQFLIEPKPKEPTKHQYDFDTATVLGFLRKYNLDKYFKMNIEANHATLAGHTFQHELNIARINNVLGSIDANQGDLLLGWDTDQFPTNIYDATLAMYEVLKQGGIAPGGFNFDSKVRRASFEVEDLFLAYIAGMDTFAKGLLIAHKLLEDEVFENFTKERYASFSEGIGKDIVEGKVGFKELESYALQMPVIKNKSGRQEMLEAILNRYIYEVDTISNK*

>CD630_30650 Clostridioides_difficile_630_NC_009089 xylulose kinase

LEYVLGVDIGTSGTKTVLFDKLGNTIKSCTYEYPLIQEKSGWAEQDANDWWKAVVESIREVVQSSNISSECIKGIGLSGQMHGLVMLDNEGKTLRNSIIWCDQRTVKECEEITDLVGEERLIEITANPALTGFTASKILWVRNNEPDIYKNTNKILLPKDYIRYKLTGEYATEVSDASGMQLLDIRKRDWSDEVLEKLNIDKNLLGKVYESQEITGYVTRDVASLTGLKEGTIVVGGAGDQAAGAIGNGIVKDGVVSSTIGTSGVVFAYTKEPKIDKEGRVHTFCHAIPNTWHVMGVTQGAGLSLKWFKDNFCQSEVEVSNSLGEDVYEIINNQVSQVPTGCNGLLYLPYMMGERTPHLDPYARGVFFGLSPIHSKKEIARSIMEGVSYSLKDCMDIIENLNIDVNEVRASGGGGKSKVWRQMQADMFNQDVYTINSSEGPALGVAILALVGAGIYENIQKACDAIIKTSTKLEPISENVDTYNIYHRLYKRIYKSLKDDFKLLDEVVNAKINN*

>CD630_30660 Clostridioides_difficile_630_NC_009089 transcriptional regulator

LVTDKYTIREMNERLVLEQIIKNGPISRASIASTIGLNKATISAITKKLIDESLVHEIGIGNSTHSGGRKPILLVFNKCAGISLSMDIGYDYIFSSLSYLDGTIINSKKLTDIQVSKDNVIQLIDEIINSYNISKIDTPYKVIGLTLAIHGITCENKVLFTPYYNLNEIDLYSILSKKYDFPIHIENEANLTALAENTFSTVHNSLLSLSIHSGFGSGIIINNKLYSGRNGMSGEIGHTIIMPNGKLCPCGNRGCLEQYCSEKKVFEQLSSLENIPKIDSDIVKQLYYEDNQNAKKVIHEFCSYLTIAINNAITTYAPEIIYLNSQIISDIPEILQITKDMLVSSFNKGINIEISSLGSEASLYGGSAVNIKSFLNIQNLTLINEI*

>CD630_30920 Clostridioides_difficile_630_NC_009089 amino acid permease

MEKGIKLGFWSIVLLGINAIIGSGIFGLPGDAYTDIGPASILVLVFCMLLAVSIALCFAEAGSWFDTDGGPYLYAKEAFGDFVGFEVGFMKWIVSMIAWATMANFFAVTLSSVWPQAAEPLIKNIIIGILVVGLGIINFMGMKQSKHLNNIMTIGKLLPIVLFIAVGLFFIKGSNFTPFVIIQKGQSASSAFVAVSITLFYAFTGFESLAVAAKDMENPKKNVPKALVMVMFVVSVIYMLILGISIGVLGNGLAGSATPVADAAIKMLGPIGGYIITIGTIVSVGGINIASSIFTPRSAAALVEQGLMPKSIRKTNKNGAPYIAIIVSVIGTLLIAWSGSFTTLSQISVVSRFIQYIPTCLAVLILRKKYAGKDVNFRIPGGAIIPIFAVIISILLLIKAGIDEPMKIVWGLGGMIIVVPVYFYMTKVYSKKYNDVEVK*

>CD630_30950 Clostridioides_difficile_630_NC_009089 6-phospho-beta-glucosidase

MSFKKDFLWGGATAANQCEGAWNLDGKGPSCSDMCTGGSQKVSKRITRVIEKGTFYPSHEAIDFYHRYKEDIALFAEMGFKVFRFSIAWTRIFPTGMEKEPNEAGLKFYENVIDECLKYNIEPLITISHYEIPFAITEKYNGWVSRDVIDLYMNYCETIFKRYKGKVKYWLTFNEINSATMPMGGYLSQGILNEGTTDFINQVDIPQLRFQGLHHQFVASAKAVKLAHEIDSNYQVGCMQIFATMYPYTCNPDDAVKTQRDSRVMNYFCGDVQVRGEYPTYMNRYFGENNIEIKMEKGDLEILKEGCVDFYTFSYYMSTCVSSDSKEDNTSGNILGGVKNPYLKSSEWGWQIDPEGLRYALNEIYDRYRIPMMVVENGLGAYDKKESNGVINDDYRIEYLKAHIEQMKEAVEDGVDLFGYTPWGCIDLVSASTGEMAKRYGFIYVDKYDDGSGDLSRIKKKSFDWYKQVIESNGERL*

>CD630_30980 Clostridioides_difficile_630_NC_009089 PTS operon transcription antiterminator

MIIEKILNNNVIITTDENHKEIVVMGRGLAYKKRTGEHISKDKIDKIFKLSDPNISDKFKELIADIPIRYMELSDEIILYAKEKLGKRLNDSIYISLTDHMYTAIERAREGVSVKNVLLWDIKRFYKSEFKIGLEALDYIEKKFEIRLSEDEAGFIALHIVNAQMDQSIKTIYEITQIIQEISNMVKYHYRIVFDEDSVYYHRFITHLKYFAERIVSNNLHENNEDDLLNVIKVKYKNAYKFIEKLDEFIHKKYNYDLTDEEKLYLTIHVERVVSKSIKSDINPNN*

>CD630_31020 Clostridioides_difficile_630_NC_009089 peptidase

MIVNKELIIDYLKSNQENIINDIRSLVEIPSIRDESTTDINQPFGIEIRNAFDKLIQIAKDKDFVVKDFDGYAVHIEYGEGEEVVGVLNHIDVVPIYNKELWKSKPFKVCQKDNYLYGRGVNDNKGPLIGILYALLFLRELNEKPKRKIRLIVGGAEETTWECMEHYFSVNEQPKFAFSPDGNFPIVNGEKGILYFNLRKKIDKDKFRNHNLVDIKSNKEDGFVCDKIEAVFKTNDKKDLVESLAYYTEIEELDEGKVLVRYTGERALSRNPHRSYNCAFSLAKDLEKIKKLNDKGVIIRDILNSYFTDDNHGKKLGMYKEDVDMGVSTICIMSIFLEKNELNMKIDFRYPKGISWEFITNRINEIGKKENLIVDIYKDLKLLYVKPDSELINKLSNAYKQGFGKEAELFTKGAASYARVLKNGVAFGPTIEGDNPNSHQANENISIDTLYKAIEVYIYALYSLAFQ*

>CD630_31040 Clostridioides_difficile_630_NC_009089 HAD superfamily hydrolase

MRYKLICTDMDGTLMGKGFEVSEENIKALKEAMEKGIKIALVTGRPYNAMKYFTSVLGDDIYIISTNGTYFKLLGYEYKKVLSKEALKKIYTIGEKYNLNKHFKGCKIVISNNEIGEEHPYRLINSKNKEEDRIEIIENASCETLLEKADNEILKCILFSENVDSLREAKEEFKKQEDLEVVSSGKINFEVMSKGTSKGIAVKKFCDILGIDSEEVICIGDNENDISMIKFAGLGIAMGNATDEVKSMADFVTDTNVNDGVAKALRKILS*

>CD630_31130 Clostridioides_difficile_630_NC_009089 aminobenzoyl-glutamate utilization protein B

MIGSISINEIDKKRFKLDDLSKKIWENPEKAFKEFKACENTANFLRSEGFDVEVGVGGLATAIRASFGSGKPVIGFMGEFDALPGLNQKVSTKQEAFELGAYGHGCGHNLLCTAHVGAVVGLKREMIENNLSGTIVFYACPGEEQLTGKGFMARGGAFEGLDLAINFHPNKISEATIGTSTAVNSVKFHFKGKTAHAGSDPQNGRSALDAVELTNVGANYLREHVTSDVRIHYTITDGGVAPNIVPDKASVWYYTRALSREAVESTYERLVKVAKGAAMMTETEVEVEFLGGCYNTLNNHVLANLVSECMNEIKQEPWTREELDFAAELDRQSPQQYKAMISKYNLPEGTHLYYGGGNVTNFNSYGSTDIGDVMHIVPTAYFFTGCTNLGAPGHSWQFTSCAGSSIGEKGMIYASKIMAMFGAKILNNPEIAKKAKEEFDKSMNGKTYKSPIPDDIPTP*

>CD630_31190 Clostridioides_difficile_630_NC_009089 UDP-glycosyltransferase, MGT subfamily

MSKIVFFSIPAYGHTNPTIEVVRELVDRGNEVLYYSFNEFKDKIEGAGAKFICCDKYLPELLPGDEKKIGKDFPGLIEMIVDTTISLDEKVCRELKDFNPDCIVSDSLSFWGKLFAQKLNIPYVCSTTTFAFNKHTAKLMKQNFIEIIRMIFGVRRINKKIKLLQRKGYEVKNFISIVSNDNDTDTIVYTSKEFQPMVETFSSKYSFVGPSVSKLIIEPKERKRKLIYISLGTINNKNVSFYKNCISAFKDSDVDVIMSVGRSTDIKSLGNIPNNFEVKNSVEQIAILQKTDVFVTHSGMNSVNESLYYGVPMVLFPQHSEQRMVAKRVVHLGAGIMLKEDKSESIKKAVFQVINDNEYKENATKLSKSFYNAGGSKKAADVILQIICNSRQVDI*

>CD630_31210 Clostridioides_difficile_630_NC_009089 flavodoxin/nitric oxide synthase

MILYFSGTGNSRYVARKIAQELNDELISLNQLIKDEKTDELISADKPFIFVCPTYAWRLPIVVTDFIKETKFLGSNRVYFVMTCGGDTAKSINYIQKLCKYKEWQLKGMAEIKMPENYIALFSTTDKETSKQMIKEADKQIYRIISDIRNENEFETITPSGLGGTIKSGIINTVFYKTIISAKGFHYTDKCIGCGKCVELCPLNNINLKNKKPVWKNNCTHCMACICGCPTEAIEYKNKTQNRERYYLE*

>CD630_31230 Clostridioides_difficile_630_NC_009089 Fe-S binding domain-containing protein

MNMKYQHIIEVDKELCIGCGLCKNDCPVNNIIIENKKSVIKKQDCLMCGHCAAICPTKAITLTGFDEPPIELTNKPKLDSDELLMAIKSRRSIRKFKDKEVSSEIIKQIIEAGRYTPSAKNSQDVSYIVLDNKKSIYENEAVKFFRKIKPIANIAIKYSKEVEIDDNFFFKHAPIAIMIITKDKISGSLAASNMALMAESYGLGVLFSGYFSDVANNSPKLKKLLGLKRSNHVLTTLVIGYPDVKYRRTAQKEVATVRYL*

>CD630_31240 Clostridioides_difficile_630_NC_009089 6-phospho-beta-glucosidase

LTMSFKNDFLWGGATAANQCEGGYNEDERGLANVDVCPTGKDRTAVITGKLKMFDFDDEHYYPAKTGIDMYHNYKEDIKLFAEMGFKVYRMSIAWSRIFPKGDEETPNEKGLQFYENIFKECKKYKIEPLVTITHFDCPMHLVKKYGAWRNRKMIGFYEKLCNVIFRRYKGLVKYWLTFNEINMILHAPFMGAGICFEEGEDVEKIKYQAAHHELVASAIATKIAHEVDSNNLIGCMFAAGSVYPYSCNPNDVWEATKLDRENYFFVDVQSKGKYPNYALKYMEQKGITPEMEPGDIELLNKYTVDFISFSYYNSRCVRTDENADDMAEGNIFTSAKNPYLSYSQWGWPIDPLGLRITLNHVYDRYEKPLFIVENGLGAKDIADENGYVEDDYRIDYLREHIKAMHDAVSIDGVDLLGYTTWAPIDLVSAGTGEMEKRYGFIYVDRDNSGNGSLRRMKKKSFEWYKKVIASNGEDLY*

>CD630_31350 Clostridioides_difficile_630_NC_009089 fructose-1-6-bisphosphate aldolase

MLINMKEMLKVAQENQFAVPAFNIGSGQILKAVVQSANEKNAPVILAIHPNELSFLGDSFVASCIEEANKSKVPMVIHLDHGENKEQILRAIRCGFTSVMIDGSHLPYEENVAISREIVEIAKGLNVSVEGELGTIGTTGTSSEGGTDEIIYTDSKLAKDFVEKTGVDTLAIAIGTAHGIYPKGFKPELKLDLLKEIREVVDIPLVLHGGSSNPDEEIAQAVKLGVCKINISSDVKSAYYKKCRELLEQNPSLYEPDTIYPPCIKSAREVIEFKMNLFNAIDKLKCFYKNKN*

>CD630_31710 Clostridioides_difficile_630_NC_009089 2,3-bisphosphoglycerate-independent phosphoglycerate mutase

MMKKPVALIIMDGFGYNKDVKGNAIAESKTPNLDRIKKEYPNTLINASGLDVGLPDGQMGNSEVGHTNIGAGRIVYQDLTRITKSIKDGDFFTNKVLCEAMDNAKENSLHVMGLLSDGGVHSHIDHLKAIIKMAKDKGVQKVYVHAFTDGRDTDPQSALEYAKEVQASMDEIGVGEFATVSGRYYAMDRDKRWERVELAYNAMVRGIGEKANSIEEAIQNSYDDGKNDEFIMPTVIMKDDKPVGSIKENDSIIFFNFRPDRARQITRALVCEEFDGFKREDIKNFFVCLTEYDITIENVHIAFGPQSLANTLGEYLAKNGKTQLRAAETEKYAHVTFFFNGGVEEPNKGEERLLIPSPKVATYDLKPEMSAYELTDKALDKLGEDKFDFIVLNFANPDMVGHTGSIEAAIKAVETVDTCVGKLIDKIVELGGSAIITADHGNAEYMLDPETGKTVTAHSINPVPFIVVGQEYESAKLLDGGRLSDIAPTILDMMKLEKPEEMTGHSLISK*

>CD630_31730 Clostridioides_difficile_630_NC_009089 phosphoglycerate kinase

MSMLNKKTIEDIDVCGKKVLVRCDFNVPLQDGVITDENRLNGALPTIQYLISKGAKVILCSHLGKPKGEAKPELSLAPVAKRLSEMLGKEVVFAADDNVVGENAKKATEKMENGDVVLLENTRYRKEETKNEENFSKELASLAEIFVNDAFGTAHRAHCSTVGAGEFLQERVCGYLIQKELKFLGEAVANPVRPFTAILGGAKVSDKLAVINELLEKVDNLIIGGGMAYTFLKAQGYEVGTSLLEIDKVEYAKEMMEKAKNKGVNLLLPVDVVMADHFAPDATPIVTEDANVKEDYMGLDMGPKTIANFVKTIKESKTVVWNGPMGVFEFENFANGTLSVARAMAELTDATTVIGGGDSAAAVNQLGFGDKMTHVSTGGGASLEFLEGKELPGIAALDNK*

>CD630_31810 Clostridioides_difficile_630_NC_009089 chlorohydrolase/aminohydrolase

MILVGNGRLISQDSLNPYMEDGCVVINDNLIEDIGTTEEMKAKYPVHEFIDAGKKIIMPGLINSHMHIYSSFARGMAVPGKPSENFLEILNNLWWRLDKQLTLEDTKYSAYATYIECIKNGVTTVYDHHASPNAIEGSLFTISDVAKDLGIRTCLCYEVSDRDGGNTIQQGINENVNFIKYAQKDDTDMIKGMFGLHAAFTLSDDTLNKCASEMAGLDTGYHVHVAEGLADLQYNVDKYGKRVLERFNDFGVLKEKTIAVHCIYISAEEISFLNKTNTFVVHNPESNMGNAVGCSPAIELLKRGVTVGLGTDGYTSDMFESLKVANIIHKHHLKDPRVGFEESQTMLFDNNRKIAKAYYKNDLGVLKKGAYADVIVVDYIPHTPLTENTIGGHTIFGMSGRSVDTTIINGKVLMKDRKLINIDEESILAKSRELSQNLWNRI*

>CD630_31820 Clostridioides_difficile_630_NC_009089 D-aminoacylase

VRTLIKHGLIVDGNKTPAYEGDILIENEKILKISQDLNEEADKVIDAKGRVICPGFIDTHSHSDLVILVNPYNEVKIRQGITTEVLGQDGISMAPLPQEHISSWRKNLAGLDGESDEIDWKYETTENYLKMMDYNGVGLNETYLVPHGNVRMEAMGLEDRPATKEEIQKMCEITERELKAGAIGLSTGLIYIPCAYSLTEEIIEMCKVVAKYDGVFVVHQRSEADTILTSMEEIIEIGKQSGVKVHFSHFKVCGKANWKYIPQVIELLEKAEKEGIRVSFDQYPYAAGSTMLGVVLPPWAHSGGTDKLIERLSDENERAKMKKDIANGIEGWDNFIEFAGIDQIFVTSVKTEKNKDTIGKSLLEIGKMRGKDPLDATFDLLKEEENAVGMVDFYGLEEHIIGFMKRDEQNVCTDGLLAGKPHPRAYGSFPKILGRYVRELNVLTIEEAVYKMTKKAATSFSIKDRGELKEGYFADIVIFDKDTVSGCDDFINSMQYPTGIDYVIINGNCVVEEGKYNKIKAGKVLKN*

>PCZ31_RS03555 Peptoclostridium_difficile_strain_Z31_NZ_CP013196 aminoacylase

MFDVKIVNGIIVDGTGNSRYKSDVGIVGDKIMAIGDLSQKEAKETIDATGKFVSPGFIDFHTHSDLSLVYDKYTRSRIHTGVTTDVIANCGIGVAPIREEKKQELIDYLGTRIIGTIPTKLELHWNTMQEYFDYLTENSPAVNVVAYVAQGPVRINEMGFSKEPATLEQLKNMKLEVRKAMEAGCVALSSGLVYLPGAYTKKEEMAELCKELIPYNGYYISHIRDEGDEEMEALDEAIYIAKTAGVPLHISHLKVMGHKNFGTIDEVFKKLDEAEADGLEVTFDCYPYTAGMTSLGALLPPWAFEGGVENMVKRLEVQENRDRIIKELEEGIPGWQCFYQLAGGWNGVVLASVMTEANKYVEGKTLMEVAKINGENPFDTFFRLLIEEKSKIQVVVHTQGQEDTDKVVCHPKSCIGSDSMDLSTEGLLSLGKPHPRAFGTFGRIFSYYVREKGMLTFEEAVKKITYLSAKRLGIYKERGLLKENYFADIVVFDPDTIEDKATYSNPKQYTVGVEYVLVNGKIALAGGKQTDVCAGRVIKNPLSIAK*

>CD630_31840 Clostridioides_difficile_630_NC_009089 diaminopropionate ammonia-lyase

MREIKWKLNNLPKSEEKEKGIEFLKDEEIAKAKAFHESFPQYEKTPLVKLDNLAKSLGVSGIYVKDESYRFGLNAFKVLGGSYSMGRYLAQRLDTDISELGYDKLTSKEIKEKLGEITFFTATDGNHGRGVAWTANKLGQKSVVLMPKGSSEFRLNKIKGEGADASITDLNYDDAVRLANDYAEADDHGVMVQDTAWDGYEEIPAWIMQGYGTMAKEAIEQLKEYGVDRPTHVFVQAGVGSLAGAVQGYVASIYDECPITVVVEADEADCYYKSAEAGDGKPRFVGGDMPTIMAGLACGEPNTIGFEVLKNYSSAFVSAPDWVSAKGMRILGNPLRGDEKVISGESGAVTTGALVSILESEDLKDLREALKLDENSKVLLISTEGDTDPDKYRDIVWNGECQSK*

>CD630_31950 Clostridioides_difficile_630_NC_009089 UvrABC system protein A

MKEIKINGAKIHNLKNIDVSIPKDKLVVATGVSGSGKSSLMFDIVFEEGRKQYLQSLGILAGIDSEDKFDNIQGLAPTIAVQQNIIRQSNPRSTVGTRTNILNMLTLLYSVEGQIMCTMCDTPVSDNLICNNCGYEEERLRPSYFSYNSSDGMCMKCSGHGAYFEINIEKLVLDKHDTLKQVLDRAKITPGYLRVFSKKFNDYLDIQFLQLPEEIRNEVLYGHYENGKKSSSLSKVFHNRYKRGEDLNGVYTMTACSDCNGFKIGEEARRVLLNGKHIGELGKMTILEIDDFLKLLINQGNLNTFGTNLVNDILSKTQHLIESRLGHLSLYREMSTLSGGEMQRLFLNSHLDSEMDSLIYVLDEPTAGLHESEKIDILKSLKKLKDLGNTVIVVEHDKNTIEMAEHIIDIGPKAGVEGGQLVYQGDLEGLLQSDKSLTGQYLSGKYPMPNRTSNKNITHTDKIPCITIQNANTNNLKNVTVSLPLGAMVGISGKSGSGKSSLISDTLLPLLRSQFNNHTINNQINSSESEIGEEDDYAVVETIADRLIGTKHISGYSEISQSPIGKNMNSTPASYIGIWDKIRTLFAGQPESLEQGFTAGHFSFNSKGACPKCSGSGYEKVWLGNNLSIDHICSECQGKRFNDESLSIKYKNKNIHDILNMSVSESVHFFKDIPNIVSHLNVLEQIGMGYIKLGQPTPTLSGGESQRIKLAKEIGKKRNGNILYVLDEPTTGLSLYDTSKLIQLLDELVENGNSVIVVEHDIDVLNVCDWIVELGPEGGDKGGYIIAEGSPKTLKENPKSITGRYL*

>CD630_32010 Clostridioides_difficile_630_NC_009089 multidrug family ABC transporter ATP-binding protein

MIKVGELKFSYGKDKQILHGLNFEVKEGEIFGFLGPNGSGKSTTQKILNGVLKGYNGHVSIFGKEVKAYTESLYQKIGVLFEFPYLYTNLSAIDNLEYFSSFYPKNQRRDIYELLDLLEFKKEFINKPVSSYSKGMKQRISMARALVSNPKLLFLDEPTSGLDPSGAVLFRKIIEEERKKGTTIFLTTHNMLDADLMCNNVAFIADGKIMVIDKPKNLKMKNSNNKVEVEFVYNGNRDIEIVDIQELESGITFKYDEILSVHSKEPTLEEVFIKCTGRMLV*

>CD630_32030 Clostridioides_difficile_630_NC_009089 two-component response regulator

MAKILVVEDEKRMQNIIVEYMQKGGYTCITADDGVEALTILKSNNIDLMILDIMMPYLDGFSVCRVSREMTNIPIIILTAKGEEEDKLKGYEYGADDYITKPFSPKVLLAKVNALLRRYTTDIPKNSLSLGKIFIMVASRQVYVEDKLIDLTYKEFELLRLFMENPNQVFSREKLLNCIWGYDFEGNTRTVDTHIKTLRKKLGSEGHHIVTLIRSGYKFEVKE*

>CD630_32040 Clostridioides_difficile_630_NC_009089 membrane protein

MDFISSILQGLLSILFNFTGDFGISIVLITLIVKLVLMPLSLKQRFSVKKQQELAEKMEYIKEKYKNDTKELEKQIQVSSVESMKSMLGCLIVLVQMPVVYALYHVCLDMTKEFTTIIIPWIANLGTFDNLFILPCIYTLIMLASNLVNYIPYLKINSQVKFNKQMAVVTTITSLILTVRTPVAIGLYFITSAIYSLIEDICFRIYFNRKNRIVTK*

>CD630_32050 Clostridioides_difficile_630_NC_009089 nitroreductase

MISDSISKRRSIRKYKNQSISHETIEKIIEAGINAPSSKNRQPWRFVVITEKEKESMLKAMSKGIQNEINDNGLLPGSRQHIAGANYTVEIMKQAPVTIFILNILGKSPLEKLSPEERFYEMANMQSIGAAIQNMSLTAVELGLGSLWICDVYFAYRELCEWLNTDSQLVAAISLGYPDEEPSRRPRLQLSDVTEWR*

>CD630_32150 Clostridioides_difficile_630_NC_009089 glycine betaine/carnitine/choline ABC transporter ATP-binding protein

MTPIIQFKNIKKQYNDKTIIDNLNLDIEKGEFLTVIGSSGSGKTTLLKMINGLILPDGGNILINQTDIKNEDLIKLRRRIGYCVQGSVLFPHMTVEENISYVPNLLSKKNKLEVKSAVNKWMEIVGLPNDMKVRYPSELSGGQQQRVGIARALASSPEILLMDEPFGAVDEITRKQLQKEIKEIHKKTGVTIIFITHDIYEALILGTKTLVLNHGVVQQYDTPENILNTPANQFVDQLLNIRKSIIDDDNLKE*

>CD630_32170 Clostridioides_difficile_630_NC_009089 ABC transporter ATP-binding protein

MNNEFIILKGCKENNLNNISLKIPKRKITIFTGVSGSGKSSIVFETIAKESQRQLNERFSTFVRSFLPKYGEVKADCIENLSTPIIIDQSRLGGNSRSTLGTITDVNSFLRALYSRFGSEYIGNANMFSFNNIDGMCPHCHGLGKKLVPNMNQILDMNKSLNEGAILLSGFGVGSWHWNIFAESGFFDINKRICDYSEVELEKFLHGKAEKIKIENAGQTNMTYEGLMVKFNRLYLSREGEISEATKKKLSKLLIEDRCPICDGRRLNERVYRSLINGYNIADLTSMQIDELAEVIKSIDEPEAQPLIKGIIEKLNSIIDIGLGYLTLDRETSSLSGGESQRIKMVKYLNSNLVDLMYIFDEPSVGLHPKDVYKLNNLLKKLRDKGNTIIVVEHDPDVIKIADHIVDVGPKAGKYGGEVVYEGSYENLLTSGTLTGNALSKFLPIKESVREHSGYLEVKNCNKNNLKNVSIKMPKGVLTLVTGVAGAGKSTLIKDEFLKQNPSAVLIDQSPVSANSRSSLATYSGIMNNIRSIFSKTNGVNASLFSSNSEGACDNCKGSGIVEMNLAFMESIKSTCNVCEGKKFKKEVLEYRFQGKNIIEVLEMSVLEAIEFFNLKQIKTKLQCIEKMGIGYLTLGQTLDTLSGGECQRLKLASELNKESSVYILDEPTTGLHMADIENFISIIEEIVDNGNTVIIIEHNIDVIKRADWIIELGPEGGTKGGRVIFEGIPKQLCNSKISLTAKYIV*

>CD630_32190 Clostridioides_difficile_630_NC_009089 Hsp33-like chaperonin

MRDYVLRATSGNGQVRAFVATTRNTVEEARRLHETTKVATAALGRTLTATSIMGLMMKNDSDKLTVIIKGGGPIGTIIATSDSKGMVKGYVGNPQVEVEDYPNGKLNVAAAVGTEGVVKVIKDLGLREPYNGTYPLVSGEIAEDFTYYFAVSEQTPSVVALGVLTKEDEVEFAGGFIVQLMPDAEEETIAKLEENVAKLPSITNMLKEGKSPEDILNIVLDGLEPKILDTCEVGFMCECSKERVKTALVAIGKKSLAQIIEEDKKAEVGCQFCNKKYMYSEEELLEILKEM*

>CD630_32520 Clostridioides_difficile_630_NC_009089 hypothetical protein

MKVTFNPSGVCCREMIFEVDENNVIVDAEFIGGCNGNLSGLRSLIIGQNALEVADKLNGIDCGGKGTSCPDQLSKAIRQSI*

>CD630_32530 Clostridioides_difficile_630_NC_009089 folylpolyglutamate synthase

MKYEEALEYISQTNKFGIRLGLENIGKLLELLGNPQETLNIIHVAGTNGKGSVCSFVSNILRECGYKVGLYTSPYLETFTERIRVNGQNIPQDDVARIIELIKEKIEIMVKEGYAYPTEFEVVTAMAFYYYSEQKVDFVALEVGLGGRYDATNIITKSLVSVIVSISLDHTGILGDTIEKIAYEKAGIIKENGVVLVYDQTDEAKDVIKSVCKEKKAKYIEVDFDDINIKKSDINSQIYDCTVMKETYRDLEIKLIGEHQINNSILAISVIKYLKDINKLANINEESIRKGLINTKWPGRIEKIKENPIFIIDGAHNEDGAKSLAKALDKNFKGRKLTLLIGMLEDKDIDSVLEILLPHFNKVITTTPSNPRAINSDILREKVLKYVDDVTSKHEIEDAVNYTLETSSEDDIIISAGSLYMIGTVRTLVKKL*

>CD630_32540 Clostridioides_difficile_630_NC_009089 peptidase

MNNKVKVAVLDTGIDKEHDYLKDNLVGGIAFECMHDYIFISDKFDDEDGHGTACASIIKKEYEDVELFVIKILRKDGITNIKVLEEALRYLLDTNIRLINLSLSVIGVESVKGLFEVCYELFRKGKIIVCSLANDFDLSYPAMFNNVIGVRGFTLDGDDSFWYNKKYDVQCVMDSNSYISCDINNSYRLPPKCNSYLSAKLTGKIAKILSEKPGITISDLNDKLESLATRNHWDSCDFDECSRIVNFKLDLYDKENALFMKVADVVRDCLDIEENNEKLFKGSLFNKEIGLVYDNCFNLLKKLEHRFDIKFNYMDISKYDLVSIYTLTELVERYKNL*

>CD630_32610 Clostridioides_difficile_630_NC_009089 phosphate ABC transporter ATP-binding protein PstB

MELIDKIKMSVKDLDLFYGDKQALKKINMDIKENKVTALIGPSGCGKSTFIRTLNRMNDLIEDVTIKGNISVDGEDIYTSDDVINLRTKVGMVFQKPNPFPMSIYDNVAYGPRTHGLRDKKQLDKIVEESLKGAAIWDEVKDRLKSSALGLSGGQQQRICIARAIAMRPEVILMDEPTSALDPISTLKVEELIEDLKKDYTIVIVTHNMQQAARISDETAFFLNGEVIEFSDTKTMFTTPVDKRTEDYITGRFG*

>CD630_32820 Clostridioides_difficile_630_NC_009089 pyruvate formate-lyase

MARGTFERTKKLREESINAEPHISIERAVLMTEAYKKYEGSVEIPVLRALSFKHYIENRTLSINDGELIVGEKGDSPNGAPTYPEICCHTMEDLEVMHNRDIINFSVSEEARKIHKEEIIPFWKKRQTRDKIINAMTPEWLAAYEAGMFTEFMEQRAPGHTVCGDTIYKKGFLDLKKDIEARLKELDFLNDLDAYNKKADLEAMAIACDAMVILGKRYAEKARQMAEEETDEAKKKDLLLIAETCDVVPAHKPETYHQAIQMYWFVHIGVTTELNIWDAFTPGRLDQHLNPFYERDVENGILDRDRAQELLECLWVKFNNQPAPPKVGITLKESSTYTDFANINTGGINPDGQDGVNEVSYIILDVMDEMKLIQPSSNVQISKKTPQKFLKRACEISRKGWGQPAFYNTEAIVQELMEAGKTIEDARLGGTSGCVETGCFGKEAYVLTGYMNIPKILELTLNNGYDPISKKQIGIETGDPRNFQSYEELFEAFKKQLHYMIDIKIEGNAVIENICAKHMPCPLMSTIVDDCIEKGKDYQRGGARYNTRYIQGVGIGTITDSLTAIKYNVFDKKKFDMDTLLKALDVNFEGYEAILNLVSNKTPKYGNDDDYADEIMQEIFNAYYNEVTGRPTVCGGEYRVDMLPTTCHIYFGEIMGASPNGRLCAKPVSEGISPEKGGDTNGPTAVIKSCAKMDHIKTGGTLLNQRFAPSVVQGEKGLDNMANLVRAYFNMDGHHIQFNVFDKNVLLEAQKNPQDYKDLIVRVAGYSDHFNNLSRTLQDEIIGRTEQTF*

>CD630_32830 Clostridioides_difficile_630_NC_009089 pyruvate formate-lyase

MNPLVINLQKCSIHDGPGIRSTVFFKGCPLECVWCHNPESQTYTKQVLYNEERCSKCEACINICPHKAIYKGETKICLDQDKCEFCETCLDYCVNNAREIVGQEYSVRDLVKEIEKDRIFYEESGGGVTLSGGEVMAQDMDFICGVINMCKSKGIHVAIDTCGYAKSENYERVAKCADLFLYDIKLIDEDKHIKFTGKSNDLILKNVKILSELGANINIRIPLIVGVNVDDENLEVKKMIEFLKPLNIQAVSLLPYHNIGKHKYDKIYKKYEGEELQRPSEEKLEEIKRLFEASNFNTKIGG*

>CD630_32840 Clostridioides_difficile_630_NC_009089 serine protease, HrtA family

MSRRKKGISLVILVAIISSILSSFLTIILVKDNLVSKSTGSSTPIVVNDDGKSQNIYQAVAEKATPSVVGITTTSVDTSNMFAIPTETQGVGTGIIVDSNGYILTNSHVISDGQATSVNVLFNDGSTTSGKVVWFDQQLDLAIVKVDKTGLTPAEFADSDKVKVGDISIAIGNPLGLDFQKTVTQGIISGLDRTIQTEKTNMTGLLQTDASINAGNSGGPLLNQKGQVIGINTAKASQAEGLGFAIPINTAKSIVEEVIKNGKYEKVTLGIKGTDVSNYEAATGTKLSTDKGVYVAEVISGSSAEKAGVKVGDIITKVGDTDITGMNDLNKKLYTFSKGASTKITVNRGGKAVTINVNF*

>CD630_32860 Clostridioides_difficile_630_NC_009089 sodium:phosphate symporter

LNIIISLMGGLGLFLYGMNLMGEGLQKSAGTKLKKIIKLLTSNLFMGVLVGTGVTAVIQSSSATTVMVVGFVNAGIMTLKQAIGVIMGANIGTTVTAQLVSFDLTGMAPVALGVGIILYLFGNKPRIKNIAEILIGFGILFTGMDFMKMAVEPLRDYQGFTDLLVTFGRYPLLGLLLGFGITAIIQSSSASMGMLVALAAEGLVPLSAALPILYGQNIGTCVTSLLSSIGANKNARRAAMMHLIFNVLGTVIFLIFLNKPVVSMVTSWDPSNVARQIANTHTLFNIISVLILLPFTNLIIKLAIKLVPDRAGDIDEDETKTIKYIDDRMIETPSIALANTIKEALRMGEKAKESLNASMEALVEHSTEKIDKTYRRERLINDLQKAILNYLLKLSKAPLDDDSREVVDTLFNTVNDIERIGDHAKNIAELSQVAIDSNISFSEEGQSELDVMYNRVVSAYTYALESMRTDNVDLACKVIKIEEQVDIMEKSCRANHMYRLNNNLCSIENGVIYLDVISNLERISDHAVNIAQQVIAKRLGND*

>CD630_32880 Clostridioides_difficile_630_NC_009089 hypothetical protein

MDYIGIENITPYENTYEFSVYEYDDEITLGSEKLYVCELRVVLIKVNSLYVERLHKSVEAMVLVKNLKKDLDKTLVVNKIKNFVLDEIWVENLVKENIEVIFVES*

>CD630_32960 Clostridioides_difficile_630_NC_009089 pilus assembly ATPase

LPMLIPEKLAKKYNVFPVEFQDDNLVVEIEDEDIYALQDLRLATGKEIILKKEKKELISEKIEKYYNSFELDEDYAKKLFENLLEKAIKENASDIHIEPFKEHLIVRMRVDGELKEVSKFLVDVYPSLSTVIKLKASMDITEKRLPQDGRVDIKLGGNLIDIRVSSIPTVYGEKIVLRILNRDTFLKDKLELGFSEEAIKSINRIINKRAGILLVTGPTGSGKTTTVYSLLNDLKGINKNIMTIENPVEYKMDGISQIQVNSKVGLSFDVGLRAILRQDPDIIMVGEIRDAETAKIAVRAAITGHLVISTLHTNDAVSSIARLLEMQIPPYLLNASLIGVISQKLVRKVCNHCSHEIMIKDNFSGDVNTKVAVGCEKCNDKGYFGRTAIYEILEINDDIKTCIRNMEDSSTIKDVAQNNGMITFEDSCKRLINEKITTLEECLVVNEMI*

>CD630_32990 Clostridioides_difficile_630_NC_009089 major facilitator superfamily transporter

MKQKWIVLIIICIGVFMSTLDGSILNIANPTIAADFKINMSQIQWVVTAYMLVVTATMLFFGKLGDKVGSNRLYTLGFFIFTIGSFLCSMSNNLSTLISSRIFQAVGASILMATGLGIVSNAFPANEKGKAIGITGAVVGIGNMSGPVIGGIILEHFGWPSIFIINIPIGIIAVFLGIKFLPKPVLDEQNKSFDIPGLLLFASCTTLILLAMNEKGNTRLYLGITALIIFLLLALREVKFEQSFIDLPLFKNRNFTVGNIIGVACYFPQMAVSFLLPFYLEQLKNLSPMMAGYVMTVHPLIMVLIAPIAGSLSDKHGAKNILTASFSFMTISLVGMALLKADSPLYLLIVCLVIFGLGLGAFSSPNNSSILADVPPQKQGYGGSFLATIRNLSFALGTAFFSSFFAQSLTYNQKFKSHTSAYVIASNQSYWIAASVCFIGLILTVFFMRKTDKSIS*

>CDIF1296T_03417 Clostridioides_difficile_ATCC_9689__DSM_1296_strain_DSM1296_CP011968 ribosome biogenesis GTP-binding protein YsxC

MKIRSSEITMSAVNKSQYPAEGIPEIALAGRSNVGKSSIINTLLNRRNFARTSQTPGKTRTINFYLINNEFYFVDLPGYGYAKIAKSEKEKWGGIMERYLESRQELCSIFLLVDIRHEPTADDKLMYEWIKHFGYNCVVIATKADKISRGQYQKHISIIRKKLQMESSEKVIPVSSLKKTGVEELWEEIVNQYNQHGYEITVD*

>CD630_33040 Clostridioides_difficile_630_NC_009089 ATP-dependent protease ATP-binding subunit ClpX

MSKYEEKRQLKCSFCGKNQDQVRRLIAGPNVYICDECVELCDEIIQEEIEDTIDEDTTSLPKPKEMMEILNDYVIGQEKAKKALSVAVYNHYKRIYSKKSSSKDIEIQKSNILLLGPTGSGKTLLAQTLARTLNVPFAMADATSLTEAGYVGEDVENILLKLIQAADFDIEKAERGIIYIDEIDKIARKSENPSITRDVSGEGVQQALLKILEGTVANVPPQGGRKHPHQEFLKIDTTNVLFILGGAFDGLEKIIQKRGGDKTLGFGAKIESKKELDLGKLYEKVLPEDLLKYGIIPEFIGRIPVLATLELLDEDALMQILQEPKNALVKQYKKLLELDDVELEFEEGALRAIAKKAIERNTGARGLRSIVESVMMETMFEVPSRDNIKKVIVTEKSVNEDSVNPIIVLKDQEESA*

>CD630_33130 Clostridioides_difficile_630_NC_009089 oxidoreductase Fe-S subunit

MNYFVIADPDKCIGCRTCMIGCVVAHSDEDIFYQNLDEINFNPKLSVIKTKEVSAPIQCRHCEDAPCAKACPNGGIVRVGNTIKINEENCIGCKTCMLACPIGAIDIVTLKDVDEGKLCFRERMTANKCDFCIESPEGPACVNVCPTKAFTIVKDEDIDSKVKNKRKLAIL*

>CD630_33140 Clostridioides_difficile_630_NC_009089 iron hydrogenase

VTFMIINIDKDLCTGCRECSKVCPVNAIEGEEGKPQEINLDRCVMCGQCVQTCKSYASVIDEGFEFLQEKKQEREIPESINEPIFAAYNVCDIDKVKKALSDPNVFTMVQCAPAVRVALGEDFGYYLGYLGAGKMAAALRALNFDRVYDTNFAADLTIMEEGSELIKRVTEGGTLPMFTSCCPAWVKFMEKNYPKLTNHLSSCKSPQQMGGAIFKTYGAEINNVDASKIFSVSIMPCTCKKYECDREEMDSSGYRDVDVVLTTRELAYLIKDMGIDFKNLREEKFDSPLGSYSGAGTIFGVTGGVMEAAIRTGYELITGESIPDVEVKQVRGENGFRKSTIKIGDLDLRVGVVSGLKNVIPVLEDLEKGKLDVDFIEVMTCPVGCVSGGGQPKILLEEYKELAYENRTKATYVHDEHLAIRKSHENPDIKKLYDEYLVEPLGEKSHHLLHTTYCIGKEVAK*

>CD630_33150 Clostridioides_difficile_630_NC_009089 oxidoreductase Fe-S subunit

MKNKIGSFVIADPNKCTGCRACEVACFTMHNQHNNVGYTVGTVDIPVIPRLYLVKGDNFCMPIQCRHCEDAPCLNSCPQKAIVKENNIMSVNEEKCIGCKTCLLACPFGAIDLLPQYEDGKEVEQVILDENKKIAYKCDLCKDNEKIACISACPQKALKLVTPIDDKKAKNRQAALSLLLTNK*

>CD630_33500 Clostridioides_difficile_630_NC_009089 family 2 glycosyl transferase

MITISLCMIVKNEEEVIGRCLECVKDIVDEIIIVDTGSTDSTKEIVSKYTDMVYDFEWIDDFSAARNFSFSKASKDYTMWLDADDIILEADREKLLKIKESLDTSIDIVMAKYNVSFDENGNPTLSYYRERLFKRSSNHKWVDPIHEVVPLFGEVFYSDIAISHKKLHRQDPLRNLRIFEKMISEGKTLEPRHQFYYSRELYYNARYKEAIEGFTKFLDSSRGWIEDCISACRDLATCYYLINDEKSALYSLFRSFEFDEPRAEICCDIGKHMFDRQKYKEAIFWYKVALTRDKNDTNGGFKSNDCYGYIPSIQLSVCYDRLGESDKAIYYHEKTKEIKPNDSAVLHNENYFSKFKNS*

>CD630_33590 Clostridioides_difficile_630_NC_009089 ABC transporter ATP-binding protein

MSLLKVTNLSQCFMDKSLYEKANFDLFKGEHIGVVGQNGTGKSTLIKILLGEVVPDSGEIKWQPNINIGHLDQYAEINRDTTISLYLHTAFEELYKIEKEMNLLYQKSAISENEQYLIKASDYQEQLIANNFYSIDNEINKIANGLGLDSIGMNRVVRELSGGQRAKVILAKLLLSNHDVLLLDEPTNFLDKEHVEWLSNYLNTFNGAFIVVSHDFDFLEKISTGILDIEFGMIKKYHGKYSEFLKQKSRLREDYIRRYQAQQKKIEKEETFIRKNKAGVNSKIARGRQKQLDKIERIAPPSFTGKPNIQFSEIEISAQNALTITNLEVGYYYSLLPKLNFSVDGGQKIVITGFNGIGKSTLLKTLVKDIPRISGDFQFSEQVKIGYYEQDLKWENPDKTPLQIVADKYPKLNTKEIRRHLARCGVKEEHVSRSVSTLSGGEQSKVKLCCMMLSPCNFLILDEPTNHFDAETKDALQNALKQFRGSIILVSHEEKFYKGWIDKVFNIEKQLV*

>CD630_33670 Clostridioides_difficile_630_NC_009089 hypothetical protein

MKISSQYRSQYSFRYESNINNTRINESMVKKNETVGKDTYLSNIMKQKQELNDRIRDLKYRQEVYTKKINDAIKNLCKSEIRETTNNFSNIEIGIKNSIIEEKNKSTMLDENSTYLNTNDEKESLITKESNEKIEEEILNDEKLEELEQKKDYKEDSNKKEKVSEDLSLVGKTREELENMLKNFINLTQEEIMKLESRIEKLDKNAEEYKQNSKTNIFDKTDEQKKHINVLI*

>CD630_33980 Clostridioides_difficile_630_NC_009089 NUDIX family hydrolase

MREEVSAGGVVLFGNTILLLRKFNGDWVLPKGKVEEGENNQEAALREVSEETGVKADILKYLGEIHYTFKENWDENRAVHKTVFWYLMQAKNMDTIPQKEEGFIDAKFIHLDRVVDLARYDDEKEIIKVALQEIKKRLKKN*

>CD630_34050 Clostridioides_difficile_630_NC_009089 iron-only hydrogenase electron-transferring subunit HymA-like protein

MCDFISHNKQLFDELDIFIDSLATKEGALIQVLHEAQGIFGYLPKEVQLHVARKLGVAPAKVYGVVTFYSYFTTEPVGKYKISVCLGTVCFVKGADKILSAFEKQLGIKVGETTSDFKFSLEGLRCLGACGLAPVVTVNGKVYGKVKPDQVSEILDTYRELELNC*

>CD630_34070 Clostridioides_difficile_630_NC_009089 iron-only hydrogenase catalytic subunit HymC-like protein

MSLVNLTINGKHVSAPSGTSILDAAKLINIKIHNLCHLHMNEIDKLDTCASCRVCMVETERGLVPACGTVIKEGMKVQTNSAKALNARRTIVELLLSDHPQDCFVCEKNGDCELQTIAADLGVRKIRYQGSKSFAGKDTSTKSLVKDHSKCILCRRCETVCNDIQTVGALSGVNRGFNTLVSTFFNSDMVETECTFCGQCISVCPTGALTEVDNVPKLWDVLNKKEKTIVVQVAPAVRVAIGEEFGLEPGSISTGKMVAALKALGFEHVFDTNFGADFTIMEEATEFIERIQKGENLPILTSCCPAWVNFLEHNYPDKLNLASSCKSPQGMFGSIAKNYYAPKILGINPEELYVVSVMPCVAKKYEASREELSESGILDVDLSITTRELAKMIKEAAIDLPNLQDQDFDNPLGKSTGAASIFGASGGVLEAALRTSYEKITNKTLDNVNFTNVRGLKGIREASIDVDGTTVNVCIVNTLKNARKIMDKVRSGECKYHIIEVMACPGGCVGGAGQPYHHGNTEIVDRRANALYEIDRNKAIRKSHENPDLQAIYKDFFGEPNSDVAHKYLHTHYFDKSCVYGECPQECACEEAK*

>CD630_34080 Clostridioides_difficile_630_NC_009089 DNA mismatch repair ATPase MutS

MGIEYFSKLLESSTEKELLLSKKSNNISTARLISFLIVIAGFAIRFYNKNIVGIFVGVLSIIIFIALLVIHSKVKEEETYFKSKSEVLNKYIKRFGDGWKEFKIDGKEYLKDENSQAKDLDLFGRASLYQYICVANTSYGKNFLAKYLWNENPDENVILERQRAIKELLSKDDFSIHIQTLSNIIGKEQKNNSDSSIESFIEYGENKNVYIPKWMHIFTWGLPTATILSFIFCMLGFLPVLPVFLLFIIQLGFSGFGYPRLMQTLAPLFSFSRSIQVYEKMFEVLEKETFESAYLKELQDKLSKGSGVSRGVKQLNSIGNAVNLRYNQILYIIACGVLMWNYHCAEALERWKGIYGNQIRDWFESIGEFEALISLTVVSHVKENTCFPVIKYEDTPRLKVEEVYHPLIAEKSVIANSIQLNSQTCIITGSNMSGKTTFLRSIGVNLVLAYAGAPVCAKSFDATCMAIFTSMRIQDDVSQGISTFYAEILRIKSMIQYSLKELPMLVLVDEIFKGTNSADRIIGASEAVKKLSKPWVISMVTTHDFELCDLSGSGDVEVVNYHFSEYYVDDKIHFDYKIKDGRCKTTNAKQLMRMAGIL*

>CD630_34110 Clostridioides_difficile_630_NC_009089 excinuclease ABC subunit A

MEDKIIIRGAKEHNLKNIDLELPRDKFIVFTGLSGSGKSSLAFDTIYAEGQRRYVESLSAYARQFLGQMEKPNVEYIEGLSPAISIDQKTTSKNPRSTVGTVTEIYDYLRLLFARVGDVYCPTCGEKISQMTIQEIVDKMLDFPDRTKLQILSPIVRGQKGTHKKVFDNIKKEGFVRVRVNGENYEVSDDIILNKNQKHNIEVVVDRIVIKDGIESRLADSIETAVKLSDGLVIAQIIDGEEILFSTKFACPEHGIGIEELSPRMFSFNAPFGACDVCNGLGESKEVDPELVVPNKDLSIKQGAVAAWGSTGVNDDTYYSKMIKSLAEHFNVSLTTPFKDLPDDFVEELLYGKDNIIVEFTYESKFGGTRNYKSYFEGVIVNLERRYRETNSDYMRDKIEEYMAERPCPKCKGMRLKKEVLSVLVDGKNIMEVTNLSVNELIDFMENINLTEKQRFIAHEIIKEIKGRAMFLRDVGLDYLNLSRKAGTLSGGEAQRIRLATQIGSALVGVLYVLDEPSIGLHQRDNDRLITTLRHLTDLGNTLIVVEHDEDTMREADYVIDIGPGAGIHGGEIVAQGTLDEIIENENSITGQYLSGKKQILLPETTREGNGSFIEIVKASENNLKNIDVKFPLGKFTCITGVSGSGKSTLINDILYKGVASKVNRLKQRAGKHKEIKGIESIDKVINIDQSPIGRTPRSNPATYTGVFDFIRDIFASTNEAKARGYKKGRFSFNVKGGRCEACKGDGIIKIEMHFLPDVYVPCEICKGERYNRETLQVKYKDKTISDILDMNVEEAVEFFENIPNIKRKLETLMDVGLSYIKLGQPSTQLSGGEAQRIKLAAELSKRPTGKTLYILDEPTTGLHMADVDKLIQVLQRLADTGNTIIVIEHNLDVIKTCDYVIDLGPEGGDKGGTIVATGTPKEVSKVDGSYTGQFLKKYFS*

>CD630_34120 Clostridioides_difficile_630_NC_009089 excinuclease ABC subunit B

MDFKIKSDFKPTGDQPEAIKSIVDSINRNEKFSTLLGVTGSGKTFTMANIIQQVKKPTLIMAHNKTLAAQLYSEFKEFFPDNAVEYFVSYYDYYQPEAYVAHSDTYIEKDASINDEIDKLRHSATASILERRDTIIISSVSCIYGLGDPKDYKELMLSIRPGMQRDRDDVIKRLIEIQYERNDINFTRGTFRVRGDILEIFPASNDEKAIRIEFFGDEVDRITEIDYVTGKIVGTRNHVVIFPASHYVTTPERIEKAIVEIENELQEQIKFFKENDRLLEAQRIEQRTKYDIEMLKEIGFCQGIENYSRHITGRSEGERPYTLMDFFPDDYLIIVDEAHVTIPQVRGMYAGDRSRKTSLIENGFRLPSALDNRPLNFQEFEGNINQMLFVSATPGPYEIQHSETIAEQIIRPTGLLDPIVEVRPINNQIDDLVGEITKTIEKNERVLITTLTKKMSEDLTNYLKEIGIKVKYLHSDIVTLERTEIIRDLRLGKFDVLVGINLLREGLDIPEVSLIAILDADKEGFLRSETALIQTIGRAARNENGRVIMYADRITDSMQNAIDETKRRRDIQNLYNEEHNIIPKTIQKNIRDSIEATKVAEEEVVYGISDTDDKDEIRANIDKLKSEMMEAAQNLQFERAAELRDKVKQLEEKLEK*

>CD630_34130 Clostridioides_difficile_630_NC_009089 nucleotide pyrophosphatase

MTKVIVLSVDSLFEKDLEFVKNLPNFKSILENCSIVKNISCVYPTLTYPCHTSMVTGVYPKKHGIYHNEKFDPNKENKDWYWYSKDIKAKTIIDVAKENNLTTSSVLWPVMGANPNIDYNIAEIWAPSREDDPRETFERSSSKVIMDGIYNRHCHYIDWKLEPNMDLFGVNCSVDIIKEYKPDLMLIHLATLDHTRHNYGLFNDEVDKALKMNDKWLGDIIQATKDAGTYKDTNFIILGDHGHLRVDKVINPNVLLKSNGFIKVENGEVKDFDAYVNSSGISAQIIVNNLDRLTELRELLEEFKEELFIENIFTKEEASNLGLEGDFEMVIEGLEGISFGNDFEGSIIKDSDIKDYKFAVATHGHLPTKGNRPPFIAFGPNIKGGVVIEEGDLRAHAATILKMFNLKLDGVEKEAFDFIM*

>CD630_34240 Clostridioides_difficile_630_NC_009089 cobalt-precorrin-3B C(17)-methyltransferase

MIYVVGIGPGSKDTMTLEAIKAIEDSEAIVGYKTYIKLIEEFIQDKEIIQNGMRQEVDRCKQAVEEAKKGKKVAVVSSGDAGIYGMAGLILELISKEDEDIKVKVIPGVTASIGAAAILGAPIMHDFCHISLSDLMTPWEVIEKRLRLAAEADFVICLYNPRSKGRSEHLSKAFKIMGEFKGGDTPVGVVKDVGREKEEKFVCTFENMDFEKVDMTTMVIIGNKSTYINGELMITPRGYTV*

>CD630_34320 Clostridioides_difficile_630_NC_009089 threonine-phosphate decarboxylase

MKDLGHGANVDEMARLYGKNPKEIIDFSSNINPNVLPNLERYILKGLEECRNYPDINYTNLRENISKYIDINPDFIIPGNGATEVIYLLMKSIKKKLAIINPTFSEYRRSAELNNLDIIDLELDLENNFKLNIDIIKENIKRFDSLFICNPNNPSGNVQDLKELVHLLDKHNKVLIIDETFMEFVEDESKYSLVKYIESNKNIFIIKAVTKFFGMPGLRLGYGLTSNTEIMNKIYEHKEPWTINSFADILSNFIFEDKEYIKNSKEYYIEERKYMLQELRNIRNIKVYDTDANFILIKIYKKTTKELKKDLFKQGNILVRDASNFIGLDDSFIRVAIKSHEDNKILIENMKNLLGD*

>CD630_34360 Clostridioides_difficile_630_NC_009089 cobalamin biosynthesis phosphoglycerate mutase CobC

MIRLILIRHALTNDNKKGRLSGHINSCISEEGKLQINKITRYLSNENIDKVYTTPSTRTKDTVEKISKLKLLEIEEKEALREISFGDFEGRTFEEIKIKYPNEFEKMIREGNNYRYPNGESLIDSYKRVAKEIDNIILENNSNLDTKTILICSHAGTIRNIITHLISGSYKYHWNFKIDNASITVLEIDGGFAVIDKMNFTDFI*

>CD630_34370 Clostridioides_difficile_630_NC_009089 adenosylcobinamide-GDP ribazoletransferase

MKRFILILQFLTRIPIKLNVGFDDEFYKSIVYFPLVGFVIGILSYLIGWISMLLFEPFIASIIITLAGVLITGGLHIDGLGDTFDAIYSYRDKEKMLEIMKDSRLGTNSLLAIMFVLLLKVGFVYDIISNNSLWVIIFMPMIARLGVMLLTYKTVTPREKGMGNLFIGKLTTSMLITAIIYTLLIVALITKFIFLLPNIVLIKVLGSIIVVFVFIILFKKHIYKKIDGVTGDILGCGIELSELVYLIYIYLLIFMFF*

>CD630_34400 Clostridioides_difficile_630_NC_009089 glycoside hydrolase-type carbohydrate-binding protein

MNILQNENLIIESSSFGAELTKIFSKKYDKNILWDGNKKYWGRQSPILFPIVGKLFDNETIIEENLYSMTQHGFARDMDFEIVDKGDTFVTYKLTDNESTLKKYPYSFELIINYTLNDNSIEVEWIVKNTDSKDIFFSIGGHPAFNLPFYKQNDFSKHYLEFKSKNDVEKINLNGSFTDDIIPIGKLKNLQLNPEVFKNDALIYTNIDEVSICNNDGSKYVTISMEDFPLVGIWTPYYSETNSTAPFLCIEPWYGLADSINSNKIYKDKKFINKLSKGKVFTASYNINIH*

>CD630_34410 Clostridioides_difficile_630_NC_009089 pyridoxal phosphate-dependent transferase

MKYNFNKVLNRKEGNCRKWSNHVIKEKFGLNEDAIPMDLADIDFECAPAIKESILKRASVGDYSYTFIGDDFYDAIINWNKRRFDVYIEKDWIKLTFGTVSTLHYIVQAYTKEGEGVLINTPAYDPFAEAVVNNNRKLCCSSLKLDNNRYYLDLEDMENQMKYENIRLLIFCSPQNPSGRIWTKEELYQVSELCLKYNVILVSDEIHRDIIFKDYRFVSLWNAHPEIYKNSIICVSPNKGFNLGGLKTSYVLIRDVKIRETLLERLKSNSITSPNVFAIPAIVAAYNESEEWLDAMTSYVEENFEIVYDFFETNIPKAKVMKSDSSFLAWIDVREVFRNEEESKEFFRHANLTMVVGSYFVKDGDGFIRINVGCPRETLNEALNRIKKTYISMYC*

>CD630_34490 Clostridioides_difficile_630_NC_009089 phosphosugar isomerase

MFKLEEQKLKDLGAIITTNEIKQQPELWLETYEIYKSNKEKLSRFIDTISNNHGQFRVIFTGAGTSAYIGNSILPYLKNKNDIRKYIFEAIPTTDIVSNPYDYLKKDIPTLLISFARSGNSPESLAALNLGNKIVDNFYHLAITCNPEGELAKMTKNDENNYLLLMPSKSNDEGFAMTGSFSCMMLSAMLIFDSLEDDVEKSYINAIIEMGRNVIDRKDEIHELINKDFDRVVYLGSGGLGGLTQEAQLKLLELTAGKISTVYDSPMGFRHGPKSFIDENTLVFEFVSNCLYTRKYDLDVLEEIKRDKIAKFTCAVSVENENNFSGTKFEFKEKYNKLPDVYLAMPYILFAQTIALFVSVKVGNKPDTPSATGTVNRVVKGVTIYEY*

>CD630_34510 Clostridioides_difficile_630_NC_009089 1-phosphofructokinase

MITVITFNPSIDRMYRVNNINIGEVQRVVSKNATAGGKGLNVAKVCKILKENPLAMGFLGGFNGEFIKNELRKLDIQNKFTKIEQETRNCLNIIEDNKVSTEFLEKGPIVEKSDLERFENDIEEVLKNTKILVASGSYCENMPIDYYEKIGNICRANNIKFILDTSGEALKIALKSKPYLIKPNIDEIRHLLGINIESREEIILSGKKLIEMGAENVCISLGKDGMVYLNEDSVYDVKVPVVKCINTVGSGDSTVAGFSVGILRGYKIENLLKFANACGISNALNMETGFVDLGEIDKYQDLVEVKKLS*

>CD630_34550 Clostridioides_difficile_630_NC_009089 carboxy-terminal protease

MYLVISKKKAIFLGVILVIITAMVTSAFQLTLGNKVVISKELYEDYKKYDKLLGLESIIKQDFYKKVSDTDLVNGAAKGLFLGTNDKYSGYYTKDEMENLINDSEGSYVGVGMYIGASKDGGLVVVPMKDSPAEKAGVKSGDKLVKVNGKSVSYKNSDEAVRMMKGKKGKTVELTILREDKQLNFKVKTDQIIEKSIESKVIDNDLGYIEITQFISSTYTDFDKALKELKAKNIKGLVIDLRNNPGGMLDICKEVADELIGEGTIVYTKDNKGNTEYLKSDKEKLGLPIVVLTNGESASAAEILTAAIVDNKEGISVGTTTFGKGLVQSVVRLKDGTGYKLTTAQYFTPNGDYINEKGIKPTIEEKDENKQLDVATKWLREQIDK*

>CD630_34650 Clostridioides_difficile_630_NC_009089 hypothetical protein

MMDKTAKEIMTTDVIVAKQDDSIADVANMLIAEKIGGLPVVDSENRVVGIISETDILKKEKYIEAPLYINLLQGLIFLDDLKKVEKDIKQVAAYKVGELMSKDIIKVHEDDKFDDVANVMIKKSINRVPVVDDDNKLKGIICRYDIIKALYNE*

>CD630_34660 Clostridioides_difficile_630_NC_009089 holo-ACP synthase

MNIFDIGVDIIEIDRIRKAVDKNNRFLEKIFTDREIEYFNSKNFKAESIAGNFAAKEAISKSIGTGIRVFNFKDIEVLRDEMGKPIVKTYNNLAKMCIDYNVLEIKVSISHSKDYAIANAITIIKD*

>CD630_34700 Clostridioides_difficile_630_NC_009089 ATPase subunit alpha

MNLKPEEISSIIKQQIKNYENKVELTDTGSVLTVGDGIASVYGLEKAMSGELLEFPGEIYGMALNLEEEVVGAVILGDDSEIKEGDIVKRTGRIVEVPVGEALIGRVVNSLGQPIDGKGPIAYTKTRPVESEAPGIIDRRSVYEPLQTGIKSIDSMIPIGRGQRELIIGDRQTGKTSIVIDTILNQKGKDVICIYVAIGQKRSTIAQLVSSLEKGGALDYTIVVSATASESAPLQYIAPYAGAAMGEEFMYNGKHVLIVYDDLSKQAVAYREMSLLLRRPPGREAYPGDVFYLHSRLLERAAKLSDELGGGSMTALPIIETQAGDVSAYIPTNVISITDGQIYLQPELFYSGVRPAVDPGISVSRVGGSAQIKAMKKVAGTLKLAYSQYRELAAFSQFGSDLDEDTKKRLAQGERIVEILKQGEHQPIKVENQVMIIYAVINNHLEDIPIDNIARFESELYAFVDNNYPEISRKILGGEDFTHDLTDAINEFKEKFVVEV*

>CD630_34770 Clostridioides_difficile_630_NC_009089 dCMP deaminase

MRPSWDEYFMEIAEVVKKRSTCIRRQVGAVIVRDKQILTTGYNGSPRNLEHCENIGCKRQELNIPSGERHELCRALHAEQNAIIQAAHNGISVDGATLYVTTRPCVLCAKMCINAGILKIVYEGDYPDDMSTELLKEAGIELIKF*

>CD630_34790 Clostridioides_difficile_630_NC_009089 uracil phosphoribosyltransferase

MSKVVETNHPLIQHKLTLMRDKNTGSKDFRELLTEIAMLMGYEITKDIPLKDVEIETPIQKTSSKVVAGKKLAIIPILRAGLGMVDGLVSLMPAAKVGHVGLYRDPETLKPVEYYCKLPQDIGERDIIVVDPMLATGGSAVAAIDLLKSKGAKSIKLANLVAAPEGIAEVQKYHDDVDIYVASVDERLNEHGYIIPGLGDAGDRLFGTK*

>CD630_34810 Clostridioides_difficile_630_NC_009089 protein-tyrosine phosphatase reductase

MNILIVCTGNTCRSPMAEAILRKAIKESGRSIEEYSISSAGISTANGMGASENSIEVLKEIGIDLSNHRSKVITKKLIDESDIILTMTKSHKEILVQAVPKCKEKVYTFKGFANKNEEDISDPFGGNLDIYRSTMREIMYSVNEIVKKI*

>CD630_34830 Clostridioides_difficile_630_NC_009089 zinc/iron permease

MILKVTIIGLLAGVIGTGLGGVISAIFKREVDKYLSFFMGLSGGIMLAVVVFDLMKESMDKMGIINTVIFTFVGALITMYIKTKLDVSGNMASGYLIFISILLHNLPEGLAIGSSFMSTESLGITLAIVIGLHNIPEGLAMALGLVCNKMKLSKVILFTVIAGLPMGLGSFLGVYFGGVFTSLIGVFLATAGGTMMYVVLEEIFPHSKSVYCIIGFLLGTMIVNYI*

>CD630_34840 Clostridioides_difficile_630_NC_009089 peptide chain release factor 1

MLKKLEVLEDTYKDLSEKIGDPDVINDQKVWQKYIKEHADLEPIVMKYREYKSVLDSIKESKEILQEESDEELRELAKMELAEMEEKVAPLEEEIKILLLPKDPNDDKNVIVEIRGGAGGDEAALFAGDLFRMYSRYAERRRWKIELLSASDTGVGGYKEVSFMIKGKGAYSRLKYESGVHRVQRIPSTESGGRIHTSTSTVAVLPEVEDVEVEINPNDLRIDVFRSSGNGGQSVNTTDSAVRVTHIPTGEVVSCQDGKSQLKNKEQALKILKARLYDKALAEQHKDIAAERKSQVGTGDRSERIRTYNFPQGRISDHRINLTLYKLDAFLDGDIDEMIDALITVDQTEKMTAI*

>CD630_34880 Clostridioides_difficile_630_NC_009089 UTP--glucose-1-phosphate uridylyltransferase

MQVKVKKAVIPAAGLGTRFLPATKAQPKEMLPIVDKPTLQYIIEEAVASGIEEILIITGRNKKSIEDHFDKSVELELDLEKKGKKELLEVVQNISNMINIHYIRQKEPKGLGDAIYCARHFIGDEPFAVMLGDDIVDNDVPCLKQLTDAYEEYRTTILGVQKVNQEDTNKYGIIEAKNIEGRVYKVKDMVEKPESGKAPSNIAILGRYIITPEIFDILKDLPPGKGGEVQLTDALKILSKKEAMYAYNFEGKRYDVGDKLGFLEATVDFALKKEDLKEDFIKYLKHVCSEFDKNNNVLYNNTDEKIDLIEVNEARELEIQK*

>CD630_35010 Clostridioides_difficile_630_NC_009089 transcription-repair coupling factor

MNDVFLYPLQNSKEYKDIINCIKNTKGSLLVNGLLPVQKPHISYSIFNDLSRQMIFITSSDLEAKKVYEDLSFYMEDKVEYLGFQDIYFYHLDAKDRNEEAKKLKVLLKLANKEKIILVTSIEAVLRKYIPKQVLLDSVSHYKVGDSLDLEKLTEKLVSLGYERVSKIEGFGQFSIRGGIIDVFSLEYTNPIRMELFDDEIDSIRTFDVYSQKSIDKLQQFSITPSREFIYPEKTTDALVKLKKETTKNTDEGTFQNIDYISSKTYFEGVENYIDYIYPEENKSIFTYLADDAIVFINDITRLKERCENYINEFRENYKLNLERGLAIKNQGKLLYHYTDLEYLVKDKSVVLNSLLPKSINNFSIKSIINFESREVPTFNGKVDLLVEELNRLKYNGYKIILATNTLERANKLGKDLLDKGLETTISKDRDIEIKSSQVIIVPAHINSGFQYKSIKFVVITDNEMIGVYKRASKTSNKKVKKGKKIESFLDLSVGDYVVHENSGVGRYTGIEQITVNAIKKDYMKIVYQGGDNLYVPIDQMDKVQKYIGAEVEKVKLNKLGTNEWTKAKAKVKKEIEDMTKDLIELYAKREKIQGYKFSKDTPWQAEFESLFPYQETEDQLKAIEETKKDMESSKVMDRLVCGDVGYGKTEVAIRSIFKACMDQKQVAVLVPTTILAQQHYNTFKERFENYPLRVEVLSRFKTPKQQKQIIEDAKKGLVDILIGTHRIISKDINLPNLGLVVIDEEQRFGVKHKEALKKIKSTVDVLTLSATPIPRTLHMSLSGIRDMSVIEEPPQERHPVITYVTESKESVIQDEIERELSRGGQVFFVYNRVEHIEEMASMIQKLVPDARVAVAHGRMTSKSLENIILGFLNKDYDVLVCTTIIETGMDISNANTMIIYDADKMGLAQLYQLRGRVGRSSRQGYAYLLYEKDKTLSEIAEKRLKAIREFTEFGSGFKIAMRDLEIRGAGNILGSQQHGHMAVIGYDLYVKMLNDAIKKVKGEPIVEEIDVEIDLSVNAYIPDNYIKDELIKIEMYKKIASIENKEDMLDIQEELEDRFSDIPKPVQTLLTIAYIKSLCKILKIEKIRQLKDEILLVPITKYRTKQKIGYNIVTELEELLEKMCKVK*

>CD630_35040 Clostridioides_difficile_630_NC_009089 type IV prepilin peptidase

MDIIIVNLYVFIVGIVFGSFFNVCIFRIPEGISISRPPSHCMSCNTRLKPIDLIPVLSYLMSGRKCRYCYEKISSRYAIVELITGLLFLTVFMLYGISTSTIYYLVLVSLLIIITFIDIDHFIIPDKILVFGAIFSMIFNLLFKEIPVKDSILGVFICGGSVWIIVLLIEFVIKKECMGGGDIKLFAMLGLYMGVKNGLLTALLSVYVGAVYGICVIIISRIKGKEYNSVIPYGPFISIGALITILCGKQLLELYFSMVI*

>CD630_35050 Clostridioides_difficile_630_NC_009089 twitching motility protein PilT

MYIYDLLEQGIRLNASDIHITVGTNPVARVKGGFVKLSEQILTSEVTMQMAKDIAGESMFKVIEEHGEADFSASLKTGERFRVNAYRQKGNYAIAIRTITAEIPTFEKLGLPESIKSFTEKHKGLVLVTGPTGSGKSTTLASMINIINEKQQKHIITLEDPIEYVHHHKQSLVNQREVGTDTESFHSALRAILRQDPDVILIGEMRDPETVSIALTAAETGHLVFSTLHTVGAAKTIDRIVDMFQPSQQQQIKTQLSTVCEGVVSQQLLPTADGKGRIAAIELMFATPAIKNLIREGKTYQIPNMIQTGVKSGMKTMDQDLMELYKNGKITKDMALSRCTDQEFMTRMIGGVNYNGYYNR*

>CD630_35120 Clostridioides_difficile_630_NC_009089 type IV pilus transporter system

VAKKVRIGDKLVEKGYITEEQLKWALSEQKNSGKRLGEFLVQEGLIDSNLLISVLKELLDIESIFLEGTEIDTLATKMVPENICKRYTVFPFKIDGNKICLAMSDPQDREAVQDVRRMSGKDVEIFISSTEDINKAIGHAYAHSEINKAMTEYNKNRTGGVRETVILEEDVNAAPIVRLVNNILENAVRMEASDIHIEQSENYMRVRFRIDGMLREYMRMNSAPYKAVISRIKIMSDINISEKRIPQDGRIYLKVDNKPIDFRVSTMPTNRDEKIAMRVLDKSNFMVSKEVLGIDEHGSKIYDELINTPYGLILVVGPTGSGKTTTLYSMLNQLNTENRNLLTIEDPIEYELPGVNQSQINEKAGLTFASGLRAFMRQDPDIIMVGEIRDTETAEIAIRASLTGHLVLSTLHANTAVGAISRLLDMDVESFLITSSVLGVISQRLTRKICEHCKVSYEADIGEKKALGIDVNESVTIYRGKGCERCNNTGYKGRLGIFEMLEITPEIKELIDSSANQREILKMARKQGMVSLKEDIVKKVLNGKTTVEEMIRIILMTD*

>CD630_35150 Clostridioides_difficile_630_NC_009089 bifunctional N-acetylglucosamine-1-phosphate uridyltransferase/glucosamine-1-phosphate acetyltransferase

MNFKAIILAAGKGTRMKSKYPKVIHKVCGKEMVNHIIDVSKKSGVKDTVVILGHEADVVKEKLAEEIIIAMQTEQLGTGHAVKMAKEYINDEDTIVVLCGDTPLIKEETLKRLFEYHIENKYHATVLTTRVGNPTGYGRIIRDKKGDLLKIVEQKDANSEEKMISEINSGIYCFNGKSLREALDLLNNNNSQGEYYLTDTAKIMRDKGLKVGAFAGSTIEELMGVNSRVELSKAEEIMRRRINESHMVNGVTIIDTNSTYIESDVMIGNDTIIYPGVMLQGKTRIGSDCIIGMNSSITNSEIGDGTEIKNSTIIDSKVGENSTVGPYAYLRPKSDLGNNVKIGDFVEVKNAIIEDGSKASHLSYIGDAHVGKNVNIGCGVVFVNYDGKNKFKSIVKDNAFIGSNSNLVAPVVVEEKGYIATGSTITHDVPDGALAIARERQVIKEGWVEKKNQKDDQSK*

>PCZ31_RS19055 Peptoclostridium_difficile_strain_Z31_NZ_CP013196 LacI family transcriptional regulator

IMKFKRTERIGAIVKILSDNPNKIYTLSYFTNQFNAAKSTISEDLLVVKNVFEKLHLGKVITISGAAGGVKYIPKTSIAENAEFLMELCERICDKSRILSGGFLYLIDLIYDPTIAAKIGKIFASNIEYVDADYVVTMETKGIPMALMTAKAMNLPLVIIRKDIKVSEGPTLSMTYVSGNSSKVESMSLPRKALKPDSKVIIIDDFMRGGGTIKGMVDLMNEFGAEVIGTGVFISTTNPSEKMVKDYISLIQLDVNGDKIVVEPNLKTFKDEYRNEDLDEEEDSEFEFEIDED*

>CD630_35200 Clostridioides_difficile_630_NC_009089 cation efflux protein

METRYEEANKITIQSILWNVVLTIIKVIAGVIGNSSAMIADGLHSASDIISSIGVLIGNYVSSRPGDREHNYGHEKAETLVSFVLSILLIFVSITIGIEAIKSLFNLDALSVPSILPLVVSVISILIKEYQYRITIKVAKKINSPALKADAWHHRSDALSSVAAFIGIGGSILGFKPLDPIASVVVAIFVAKVGISILISSVNELMDVSVDEEEIKELKFIVADTEGVKNLGDIKTRKHGAMAYVDLTICVDENLTVKQGHDIATKLEKHIIKHMEFVKGITVHVEPCTNCQGNKCNN*

>CD630_35230 Clostridioides_difficile_630_NC_009089 rRNA small subunit methyltransferase A

MDRLSSHNATKEVVQKYNFKFSKSLGQNFLIDSNIIDKILSGARITRGDNIIEVGPGIGTLTREMGKIAEKVVAIEIDRNLIPILKDTLSDLDNTEVVNQDILKVDIQELVKDKLNGGPVKLVANLPYYITTPIVMKFLEEDIPVTDIVVMVQKEVADRMNAIPGTKDYGALSIAVQYYCDTEIVAKAPRHMFIPQPNVDSTVIGLHVRDKRKYDVHNEDIFFKTVKASFGQRRKTLLNSLGGLGFLNKDEIREILKEANIDEKRRGETLSIEEFSVLSNIINTKVSSK*

>CD630_35300 Clostridioides_difficile_630_NC_009089 iron family ABC transporter ATP-binding protein

MSFLQVKNVGKSYGQVKVLKDISIDIEKGEFICLLGPSGCGKSTLLRIIAGLEDKHGGKIIINDKDMTNSPPESRNFGIVFQSYALFPNMNVYKNIAFGLENKNISKSNIDKKVKEVLEVVELSGYEKKYPSQLSGGQQQRVALARAIALEPDFLLLDEPLSALDAKVRLKLREQIRSLHRKLGITTIMVTHDQEEALCLADKMVVMNRGEIIQVGTPKEVYKNPETPFVADFIGTINFIDDGINKIAIRPEDIKVESNRDSKDKDIKVGEILDIEFRGFNYRITVEYRSKQMKLDVVSKVAEQMKLCIGSKINFKIPKEGIVQYKSEGCA*

>CD630_35390 Clostridioides_difficile_630_NC_009089 deoxyribonuclease

MLFDSHAHLNDESFDEDRDELIGSLKDKGVDLVVNPGADIETSITAIELAKKYDFIYSAVGVHPHDVSKLDDTAIETLRKLATENEKVVAIGEIGLDYYYDYSPREEQKEWFKKQIELANELKLPIIIHDRDAHGDTFEIIKNTKNPEIGCVLHCYSGNVELAREYVKMGCYISIPGTVTFKNNKKTREVVREIPLERLFIETDSPYMSPEPHRGKRNNPSQVSFVADKIAQEKGISYEEVCRVTKENAKKFFNIK*

>CD630_35400 Clostridioides_difficile_630_NC_009089 methionine--tRNA ligase

MSKPSFYVTTPIYYPSGGLHIGHTYSTVAADTIARFKRFCGYDVKFLTGTDEHGEKIQKKAIEQGMSEIEYLDGMIKDIKALWNTMDISYDDFIRTTEKRHTDIIQKIFTKLYEQGDIYKGEYEGRYCTPCESFWTESQLLEGNKCPDCGRETYLVKEESYFFRLSKYEDRLKELFKDDSFCFPAARKNEMVANFLDKGLEDLSVTRTTFDWGIKVPFDEKHVIYVWVDALCNYITALGYMTDNDEEFKKYWPANVQIVGKEIVRFHTIIWPALLMALGLEVPKQVFGHGWILFADDKMSKSKGNVVYPEPIIERYGIDTLKYFLLREFAFGQDGSYTHRNFVTRINYDLANDLGNLISRTVAMVEKYNNGIIPTAKVSTDFDADLKEQAVSTRENFEAEMDKMQFHEALESVWKLVRRTNKYIDETMPWALAKDETKKGELDTVLYNLCESIRIIATLINPIMNETANKIYEHIGIKGQDDITTWESTKTFGLIGENVKVFKGEPLFPRLDVEKEIEELTKMFSGKPPVEEKPLEHKEEITIDDLDKIELRVGKIISCEKHPKANKLLVSQVKIGPETRQIVSGIAEYYKPEDLVGKEVTVVCNLKPVKLRGVESQGMILAAGDDGEPYVLPFTQGAKDGCEVR*

>CD630_35430 Clostridioides_difficile_630_NC_009089 nicotinate phosphribosyltransferase

MRNLTLLTDLYQLTMLNGYFEKNIHEDIVVFDMFFRKNACDGGYTIVCGIDQVVEYIDNLHFSDEDLEYLKNLNLFSDKFLKFLKEFKFTGDIYAVEEGTIMFPNEPLITVKAPLYQAQLIETALLTIVNFQSLIATKASRVCFAAQGDPVFEFGLRRAQGPDAGIYGARAAVVGGCAGTANVLAGKMFDIPIIGTQAHSWVQKFDNELEAFQAYADVYPDKCLLLVDTYDVLNSGVPNAIKVFKNISEKGYKPMGIRLDSGDLAYLSKEAKKQLDNAGFSDISITASNDLDEYTITSLKAEGATINSWGVGTKLITSFDSPSLGGVYKLAASCEKGVLEPKIKISENPEKINNPGYKKVIRIYNEDNKAEADLIMLHDEVIDESKPLEIFHPTYTWKTKVFTNYKVKELLKPLYIKGRCKYNKKAVLEIKNHVQYELSTIWEQYKRLSKPHIYKVDLSRNLWYLKTQMIDSKKVL*

>CD630_35440 Clostridioides_difficile_630_NC_009089 AsnC/Lrp family transcriptional regulator

MDVTDYRIIEILQDDGRISMKDLGKIVGLTSPAVSERVKRLEESGVIEGYKAIVNPDSLGRVIKAFIHISLPSNGYTEFIESAAKDPRIVECHHITGDDCLLLKVIVKDMYELENVIDTIKKIGSTKTSVILSTPIQAKSIL*

>CD630_35450 Clostridioides_difficile_630_NC_009089 hypothetical protein

MEIGKIIIESLKREDTHKIFTELDNKHELEKIIPKISCMKSVGECKYHVVNCFEHSINALKELEIVLNDKDFFPIHLRKHVSNYLNTYIEEGINKLHVLKLGTFLHDIGKPDSMTLDETGRVHFTNHEKIGAQIIDNMGIKLELSTETYRLISKYVRYHMILLSLYKKNDLSRKELINVFNLVDEDTIGVIMLGYADIVSTVKLLGKSGEVSVLKTYMEYILTNYLYKSNYTHV*

>CD630_35520 Clostridioides_difficile_630_NC_009089 lysine--tRNA ligase

MKNNQQSNEEAQIQEDLSEVLQVRRDKLKKLQESGRDPFKESRYDRTHYSMDIKDNFDSLEGKTTKIAGRIMSKRIQGKAGFIDIQDQEGRIQSYVRLDAIGEEEYSVFSTYDIGDIVGIEGEIFKTKKGEISVKAKSVVLLCKSLQVLPEKYHGLKDQELRYRQRYVDLIVNPEVKNAFLIRTKALKALRAYLDDRGFLEVETPILNTIAGGANARPFITNHNTLHIPMYLRIANELYLKRLIVGGFDKVYEMGRMFRNEGMDLKHNPEYTAIELYQAYADYTDMMEITENVIAHMAEVATGSMIVNYQGTEINFTPPWKRMSMEDCVKEYSGVDFSTINTDEEALEVAREKGIEIKPGMRRGEVINAFFEEFGEDKLIQPTFITHHPVEVSPLSKRNVEDPRRTDRFEAFANKWELANAFSELNDPIDQKGRFIDQLRKRELGDDEAFEMDEDFLKALEVGLPPTGGLGIGIDRVIMLLTNSPSIRDVLLFPTMKLIDNNSNKEEEN*

>CD630_35550 Clostridioides_difficile_630_NC_009089 pantothenate kinase

MLLVFDVGNTNMVLGIYKGDKLVNYWRIKTDREKTSDEYGILISNLFDYDNVNISDIDDVIISSVVPNVMHSLENFCIKYCKKQPLIVGPGIKTGLNIKYDNPKQVGADRIVNAVAGIEKYGAPSILVDFGTATTFCAISEKGEYLGGTIAPGIKISSEALFQSASKLPRVELAKPGMTICKSTVSAMQSGIIYGYVGLVDKIISIMKKELNCDDVKVIATGGLAKLIASETKSIDYVDGFLTLEGLRIIYEKNQE*

>CD630_35590 Clostridioides_difficile_630_NC_009089 ATP-dependent zinc metalloprotease FtsH

MYERRASILNKLLKGAGFYLLVFIIIVGIVQFSGKPTEKIKDLKFSEVYRELTDENISRLYFVNQTSVEGTIKDTNTKFKSYVPTEVMGNKLADEVLDQAKAGKLTFGGEAKPSTPWFVEMLPTLLLIFFMVIIWFVFMNQSQGGGGKVMSFGKSKAKVHKDDEKTRVTFKDVAGLDEEKEDLQEVVDFLKNPKKYIELGARIPKGMLMVGPPGTGKTYLSRAVAGEAGVPFFSISGSDFVEMFVGVGASRVRDLFEQAKKSAPAIIFIDEIDAVGRKRGAGLGGGHDEREQTLNQLLVEMDGFGVNQGIIIMAATNRPDILDPALLRPGRFDRQVVVGTPDVKGREAIFKVHSRNKPLSDDVKMDVLARRTPGFTPADIENLMNEAAILTARKREKKIKMETIEEAITKVIAGVAKKSKVISEKERRLTAYHEGGHAVCAHVLEEVSPVHQVTIVPRGRAGGFTMQLPVEDKFYATKNEMKENIVVLLGGRVAEELVLKDVSTGASNDLERVTATARSMVTKYGMSSKLGPMSFDSDDEVFLGNSFSSKRNYSEEVAFEIDQETKRIVDGAYDKTRSILQENMDRLEYVAQALLIYETLDAEQFVKAFNKELPLNEIENAVTEENSSKEVEEQLTIKLEKDEEERNNVIDINKNLEDKSDKDK*

>CD630_35600 Clostridioides_difficile_630_NC_009089 tRNA(Ile)-lysidine synthase

MIFDKVLSTINKHNLIQKGDKIVLGLSGGPDSVCLLHVLNRLKKDFNIEIYAAHLNHQIRGIEAQKDALYVSKLCEDMGIIFFVKSINVPKYCENEGLSLEEGARKLRYEMFYEIKDKIKANKIAIGHNLNDQAETVMMRIMRGTGLKGLKGIDYIRDNCIIRPILDVERNEIEEYCEAYNLNPRIDKTNLENIYTRNKIRLDLLPYMKDNFNSNVIESIVRMSNSLKSDNDYIEKEAEAKFREVSNIKEKGFVEINLDDFVCLHDAIKVRVLRNSIKHILGDTNFVDQRHIEDIMSLEDNSKVNKMLTLPRNIFVYRKKDSIILTNEEIVNEEIEFYYNVPSNGFIKIKELKQIIETQVMSIDRYKSMKLDNSSKGFDFNKVKGGIVIRSRRQGDKIKLAMGSKKVKDLFIDLKIPREERCKIPIITDSEGIICVGDYKISENYKIDENTKEVLKINFNKL*

>AEC_RS0219720 Clostridioides_difficile_QCD_37x79_NZ_CM000658 methionine gamma-lyase

MSNNINQDLETKIIHWGHSADPTTGALATPICQTATFAAKTVEHFEELCMTWGYVYTRECNPTLTELEAKLAMLENAESAISSTSGMGAITSTILALVKSGDHIVSSDGIFSHTKLFMSELLSKFGVEVTFVDAVNPQNVKEAMRPNTKIVYIESPLNPSLDLVDIKTIAEIAHENKSLAIVDSTFGTPIVQRPIDLGADLVIHSLTKFINGHGDTLGGAVAGSKELIDLVRWPSLCCFTGASLPPMSAWMILRGMKTLDMRMKKHCENGLAVAEFLEEEENVELVKYPALKSHPQYELCKTQMNGLGGGVVSFKLKDGINGLTRDQASRKLMNSLELATIATSLGEEHTLVQMNGENLIRIAVGLESSNDIINDFKQANKKIKIKLHE*

>CD630_35850 Clostridioides_difficile_630_NC_009089 ABC transporter ATP-binding protein

MAFLKIEDLCKVYGKNENKVTALDHVSLTIEKGEFTAIIGSSGSGKSTLLHSIAGVDVPTSGKIYLEGQDVYGQSNEKLAIFRRRQVGLIYQFHNLIPTLNVVENITLPILMDKRKVNQERLNDLLELLGLKERKTHLPNQLSGGQQQRVAIGRALMNAPAVMLADEPTGSLDSKNGQEIIQLLKESHSKYHQTLIIVTHDENIALQADRIICISDGKVVRDERKVNR*

>CD630_35860 Clostridioides_difficile_630_NC_009089 two-component sensor histidine kinase

MFRNREFRKFAILFLLITILTVALGFAISIMTGILSIVSATTFGIAFFVFTKNRYKSIAQISEQINLVLHNANHLYIAESDEGELSILQSEITKMTLRIREQNYALKKEKEHLADSLADIAHQLRTPLTSVTLILSLLENTSDEDERKELIRETEELLIRMDWLITSLLKLSRLDAGIVVFQKEQIDVNNLISSALHQLLIPMELHNITLHIDIPKGVRILGDLNWLSEAIQNIFKNCMESVGDNGKIDIICEDNFLFTQLTIHDNGAGFKKEDLPCLFNRFYRGKNSSTAGYGIGLALCKTIIMRQGGTITAQNHPQGGAIFVIRFPK*

>CD630_35970 Clostridioides_difficile_630_NC_009089 S-adenosyl-L-methionine dependent methionine synthase

MENNLIGREDISINQSEVLRYLQYRGQEIDKDLKYTIDECVAITKNKINPRYLSRIYPIKINKDNVVELEGTNLTLESKDVYELLKECNECVIMAATIGIDIEKEIRRHSYSNLTKGLIIDSCGTTAIEEVCDIVQNNLEKVFLKQDKFITMRYSPGYGDLPIEKNIDILNVLNAQKQIGLTITSNGIMIPRKSVVAIIGISNTRINKSKKEKSCENCKNYRNCTYRKGANSCGNKTISEK*

>PCZ31_RS19495 Peptoclostridium_difficile_strain_Z31_NZ_CP013196 S-ribosylhomocysteinase

ILKYNGGLNNGKSRSFKLDHTKVKAPFVRKCSVLDGVKGDKVTKFDLRFLQPNVESFGTAAMHGLEHLLATYLRDTLDGVIDLSPMGCRTGFYLILWGDVDAKTVKIGLEEALKKVLESDKMPAATAIECGNYRDLSLFGAKEYAKDVLDKGFSLNIYGE*

>CD630_36020 Clostridioides_difficile_630_NC_009089 ATPase

MSIQKLLSKARKAIQDFDMIQENDKIAVGLSGGKDSLTLLHILKSYQRFSPQNFELIAITLNTGGVDNSPLDKLCKEINVPFYEFQTDIKEIVFDIRQEKNPCALCANLRRGALNDNAKKLGCNKVALGHHKDDAIETFLMSMFYEGRVNCFSPKTYLDRQDLTVIRPMVYIEEYMTKKISKDSNYPIITNPCPANGHTRREYIKNLIANLNKEMPDFKRNVFGALNNSEKLFIWDKEKIKNFK*

>CD630_36040 Clostridioides_difficile_630_NC_009089 hypothetical protein

MKSILIMGGSDFIGSALAKRLIKCGYQIDILTNGKKEIDYNGFKEHLICDRKVRKDMENIITGRKYDYIYDMTAYTKEDVSNLIDFISMDNLKKYIVLSAGAVYKDSGRNIKEENEKGENENWGKYGLNKKEAEDFIINSPIPYIIIRPTYIYGENNNLYREYYFFEKIEKNEKIPVPKGKQVSNQFIYIGDLVKVLESIMKNPHVREAYNVTNPQLISWDDLIYTCGEIIGKEPIIKYVDMEKVEFRERTYFPFRNIDFNLDINKLIEHGLYIPNVLLKEGLTATYKWFSANKPKMHDRKMNKVESVMQIV*

>CD630_36080 Clostridioides_difficile_630_NC_009089 ABC transporter ATP-binding protein

MLEIKNLSFNVESNNEELGIINDVSLSFERGKLIVITGPNGGGKSTIAKLIMGIEKATSGQIILDGEDITNLSITERAKKGIGYAFQQPPRIKGMTVENLLTLAHGKPLSTDVCCQYLTDVGLCSKDYLNREVDNSLSGGEMKRIEIATLFARDLKVSIFDEPEAGIDLWSFGKLNESFKKIHEESNQTIIIISHQERILELADEIIVLQDGSVKSHGTKEAILPEIMCQVNSSCELMKDMN*

>CD630_36210 Clostridioides_difficile_630_NC_009089 hypothetical protein

MIDIHKNIYDNKLFEELKIDCKKCFGLCCVALYFSASDGFPIDKESGKPCINLQLDFKCSVHNSLMKRGFKGCTAYDCFGSGQKVAQVTYKGIDWMQSSELTNQMSEVFLIMRQLHEMLWYLKEASVLNISDTIKSKIDLIIEETEKITNMGPEQIINLDIISHRTKVNLLLSQASESVMGKVKSFIKTSTLKNMKKLSKNIDLIGADLRKINLIGADLRGRFLIAANLRNTDLSGANLIGADLRDCDIRGANLENSIFLTQLQVNTAKGDSSTKLPASLIRPKYWEK*

>CD630_36430 Clostridioides_difficile_630_NC_009089 dihydroorotase

VKIDILIKCGKTVDKSIIDIAILDDKIIEVKNQIDDSKYQAKQIINLDGEKFISAGWIDIHTHCYERLDLYKDYPDEVGIKSGVTTVVDAGTTGALDIGKFYEDTKKYKTNVYALINIAKQGITSQDELSSMMNIDEYELKRAVKKYKDFVVGIKARMSKSVVISNDVEPLKVAKRIKNELNLPMMVHFGSSPPTIEDIFDYMEKGDILTHIYNGKPNGILRGNEVKKEIIEARERGIILDVGHGTESFSMDIAMKSKDAGIFPDTISTDIYIKNRINGPVYNLSTTMEKFIYMGYSLEDIIDKVTKNAADAISLKNKGLIKEGYDADLTIFDVVNEEKELQDSLNKSVITSTSIKPRAVVVNGEYLNIGKSIGENE*

>CD630_36450 Clostridioides_difficile_630_NC_009089 PTS system lichenan-specific transporter subunit IIC

MEKFASFLERKIMPVAAKISNQRHMRAVRAGIIATLPLTIVGSFFTILLNIPIDAYMDFIAPFKAILDIPFRYTVGFLSLYATFGIASSLAKSYDLDTTGVGMLAVMAFLVSTIVPTQVVEPVGGVIEAGRWMDMAKLNSPSLFGAIVTSLITVEIYRFMKEKNITIKMPAGVPVEVSNSFTALLPTAVILMLFWVIRHILGFDISVALSAMLSPLKGFLSGNSLGGGLLTVFLITLFWVLGIHGPAIMGPVIRPIWDMSIAENITAFTEGVSAHAMPNIFTEQFLQWYVWIGGAGATLALVVMFMFSKSQYLKSLGRLGFLPGLFNINEPIIFGAPIVMNPILGIPFIIGPIIMTILSYVLTITGVIPMMVARLPFAMPAPIAAVMSTNWSILAGILVLINFVISFAVYYPFFKVFEKQQLQREQEEMSENL*

>CD630_36540 Clostridioides_difficile_630_NC_009089 DNA replication protein DnaC

MNEDKIRKILAKYAKRRDDNELLLEHRKNEVYNRIPEIKSIDDEISKIGLSLAKIVLLNPKSKDEIVKKTKENIESLKVKKERLLAESNIPLDYLEIKFQCISCKDKGFLPNGEKCSCLKQEIVNEAYKMSNLDRILSQENFSNFNLNIFSPKKGSDGEISPRENMLNNLSICENFVHDFKKDNSENLLFYGSTGLGKTYMCNCIAKELLDKGNVVIYQTSFRILDILEDYKFRRDTNNQISEDNYKNLFDCDLLIIDDLGTELNNSFTSGEIFNIVNTRLVAGKKIIISTNLTPSQIGNTYTQRTLSRILDKFRILEFTGDDLRWERFK*

>CD630_36640 Clostridioides_difficile_630_NC_009089 aminotransferase

MAVKYAKRMQGLQGSEIRELLKLTQQPQIISFAGGMPAPELFPVEEMKKVSVAVLEENGRSAMQYTTTEGYEPLREKIAARMNDKNKTNVNKDDILVTSGSQQGLDFAGKVFIDEGDVILCESPSYIGAINAFKSYQPKFIDVPTDSDGMIMEELEKILETTDRIKMIYVIPDFQNPTGRTWPLERRKKFMEIVNKFEIPVIEDNPYGDLRFEGETLPSLKSMDTKGLVIFLGTFSKIFCPGYRLGWTCASPEILSKFNFAKQGADLQASTISQMEVSKFMDMYDLDAHVDKIKAVYVKRRDVMLKTMEEEFPEGLVFTHPEGGLFTWVELPSNLNAKELMPKCLDKNVAYVAGGGFFPNGGRENTFRLNYSNMPEEKIIEGIKNIAAVLKEAMGVEA*

>CD630_36690 Clostridioides_difficile_630_NC_009089 hypothetical protein

MKNKKIMLVLSILSISIFAVGCTNAQNGSDSSKKETKSNTNVEQPKEENNTEKEVPNNKAKPTPKEETKAQSATIYSFDVDKTDLIENKVDLNKIDENTLFEELQKLKVVPESAKLNSFTTKDIDGVKTGILDVSSDFTKSNLGSDAETLMLDSVARTYIKNMNVEQIKITVDGSNYESGHIVLEEGDYLK*

>CD630_36700 Clostridioides_difficile_630_NC_009089 cysteine desulfurase

MIYLDNAATTYPKPERVYNAVLDCMKNYCANPGRAGHKLAMRAAREIYDTRENIAKLFNVSNPMNIVFTSNATDSLNLAIKGVLQEGDHVITTSMEHNSVIRPIKALEKRGIENTVVKCDYEGFLDYEDLEKSIKSNTKLIVTTHASNVCGTLIDIKKVGEIAKKHNILFLVDASQTAGVYDIDVNECNIDMLAMPGHKCLFGPQGTGILYVREGLNLNILKEGGTGSKSEEIVQPELFPDKYESGTHNTPGIAGLNQGILFIFERGINNIRQHEEELCQYMIDKLEEVPDIKIYGPKDSKKRASVIALNIGDMDSGEVTFLLDSDYNIATRSGIHCSPLAHTTLGTLKQGAVRFSIGYFNTKDEIDKAVEALKKISKNK*

>CD630_36720 Clostridioides_difficile_630_NC_009089 sporulation initiation inhibitor

MGKVIAVFNQKGGVGKTTTNVNLSASLGTLGKKILVLDLDPQGNTTSGYGINKNEVENTIYEIMLDGLHIKEAIISTEFENIDVVPSATELSGAEIELTSKTNREYILKNSIKAVIDEYDYIFLDCPPSLGMLTINCLTAVDSVLIPIQCEYYALEGVSQLMETIKLVKSRLNADIEIQGVVLSMFDGRANLSIQVVEEVKKYFKGSVYTTLIPRNVRLAEAPSHGKPVIYYDKRCRGSVAYLELAEEFIDLEEEEW*

>CD630_36750 Clostridioides_difficile_630_NC_009089 tRNA uridine 5-carboxymethylaminomethyl modification protein GidA

MIKFEAGKYDVIVVGAGHAGCEAALATARMGYKTLIITMSLDSIALMPCNPSIGGTGKGQLVKEIDALGGQMGLNIDKTYIQSRMLNTAKGPAVHSLRAQADKFKYHEEMKKTLEDEPNLDIAMDEVVEILHEGNVVIGVGTKLGCSFKSKAVILATGVYLNSKIYMGEVAFYEGPNALGYAKYLTDSLVELGLRMRRFKTGTPARVHRDSIDFSVMSLQEGDEKVTPFSFMNENIEKKQEPCYLTRTTEETQKVILDNLKRSAMYSGVIESTGPRYCPSIEDKVVRFSDKTSHQLFIEPEGLNTKEMYIQGISTSLPFEVQLDMYKTIKGLENCKIMRPAYAIEYDCVDPTQLKISLEIKGVENLFSAGQFNGTSGYEEAAAQGLMAGINAVRKIEGKEPFVLDRSEAYIGVLLDDLVTKGTNEPYRMMTSRAEYRLYLRQDNADMRLTQKGYDIGLVKKDRYERFLNKKAAVEKEFERLKNERVTPKEVNSLLEEKGATPIKVGISLYEFLKRPEVTYELLEELGKGAGDDVSREVKEQCVIITKYEGYIEKQLKQIDQFKKLENKKLDEKINYSSIEGLRLEARQKLDDIKPISIGQASRISGVSPADISVLLIYLEQIRRTRGGKGE*

>CD630_36760 Clostridioides_difficile_630_NC_009089 tRNA modification GTPase TrmE

LFIDDTIAAIATAPGEGGIGILRISGEKALKVAEEIFKSMSGKSIEEYNKRTLIYGNIVDNENIIDEVLLAYMKGPNSYTGEDVIEINCHGGFISVKKILELILSKDVRLAEAGEFTKRAFLNGRIDLSQAEAVIDVIKAKTDIAHEVAQNQLEGSLSKKIRELRDKVTEILAHVEVAIDYPEEDIEHITYQTLKEKTDELKKDIKKLYDTAESGKILREGLKTVIVGKPNVGKSSLLNSILGENRAIVTDIPGTTRDVIEEFVNIKGIPLKIVDTAGIRDTDDIVEKIGVEKSKESFTSADLIVMVLDASRKLSEEDIEILEKLKDKQTIVLLNKNDLKQEIEEEKILKYVENNSIIKISALQQEGIEELQDKIESMVYKGSIKNNSSLVVTNSRHKDALSKAYKSATDALIALEQSMPFDFVEVDLKNIWDYLGYINGDTVTEDLLDNIFHNFCIGK*
