# Supplementary material for: Core-genome-mediated promising alternative drug and multi-epitope vaccine targets prioritization against infectious Clostridium difficile
Source: PLoS One. 2024 Jan 19;19(1):e0293731. doi: 10.1371/journal.pone.0293731 (PMC10798517; doi:10.1371/journal.pone.0293731)
Supplement: S3 File — (DOCX) [file pone.0293731.s003.docx]

>CD630_00060 Clostridioides_difficile_630_NC_009089 DNA gyrase subunit A

MEENNKILPIEIAEEMKKSYIDYSMSVIAGRALPDVRDGLKPVHRRILYSMSELNLTPDKPYRKSARIVGDVLGKYHPHGDTAVYYAMVRMAQDFSTRALLVDGHGNFGSVDGDSPAAMRYTEAKMSKLSLELLRDIEKETVDFKPNFDESLKEPSVLPARYPNLLVNGSNGIAVGMATSIPPHNLAEVIDATVYLIDNPECSVDDLIKFVQGPDFPTAAIIMGKESIAEAYRTGRGKVKVRSRAFIEELPKGKQQIIVTEIPYQVNKAKLVERIAELVKEKRIEGISDLRDESNRNGMRIVIELKRDANANIVLNNLYKHSQMEDTFSIIMLALVDGQPRVLNLKQILYHYIKHQEDVVTRRTKFELNKAEARAHILEGLKIALDNIDAVISLIRASKTGQEAKLGLIEKFKLTEIQAQAILDMRLQRLTGLERDKIEAEYEDLIKKINRLKEILADERLLLNVIKDEITIIKENYSDERRTEIRHAEGEIDMRDLISDEEIAITLTHFGYIKRLPSDTYKSQKRGGRGISALTTREEDFVRHLVTTTTHSRLLFFTNKGRVFKLNAYEIPEGKRQAKGTAIVNLLQLSADEKIATLIPIDGNDENEYLLLATKKGIVKKTKREEFKNINKSGLIAIGLRDDDELIGVELTDGKQEVLLVTKEGMSIRFDENDIRYMGRTAMGVKGITLSKEDFVVSMNLCSKGTDVLVVSKNGFGKRTNIEEYRSQIRAGKGIKTYNISEKTGTIVGADMVNEDDEIMIINSDGVLIRIRVNEISLFGRVTSGVKLMKTNDEVNVVSIAKINIEEE*

>CD630_00140 Clostridioides_difficile_630_NC_009089 serine--tRNA ligase

MLDIKRIRENLDDIKKAMERRGEREFDLDAVVELDNKRREILQEVEVMKNELNVNSKKIPQLIKEGKDVTEEKARLKELSDKIKGIDEKVKEVEAKMEYTLMRIPNVPHPEVPQGETDEDNVQIRTWGEPTKFDFEHKAHWDIGTGLGILDFERAGKITGSRFTLYRGLGARLERSLMNFFLNTHTAKHGYTEVLPPFMANRNSFIGTGQLPKFEEDMFKIEGLEYFLIPTAEVPVTNIHANEILDVAELPIKYCAYTPCFRSEAGSAGRDTRGLVRQHQFNKVELVKFVKPEDSYNELESLTHDAETMLQMLGLPYRVVKICTGDLGFTAAFKYDLEVWMPSYNRYVEISSCSNFEDFQARRAGIRFKRDKKSKAEYVHTLNGSGLAIGRCLAAILENYQQADGSVVVPEELRPYMGVDVIK*

>CD630_00150 Clostridioides_difficile_630_NC_009089 transfer RNA specific adenosine deaminase

MESSFYMKEALKEAYKAYNKKETPIGAIIVKDNQIIARAHNLTETLKDSTAHAEILAIKQASEKLGGWRLTDCDLYVTMEPCIMCSGAIVNSRIKKLIIGTRHVKNSYIEKQHEFKLDYFNNNNVKVAFDVLQEECSIILQEFFKALRKRD*

>CD630_00160 Clostridioides_difficile_630_NC_009089 DNA polymerase III subunits gamma and tau

MHKALYRAYRPQKFEDVIGQDHIIKTLKNQIYSDNIGHAYLFCGTRGTGKTSTAKIFSRAVNCLNKINEEPCNECEICESVLKDNTMDVVEIDAASNNSVDDIRELRESVKYSPANAKYKVYIIDEVHMLSQGAFNALLKTLEEPPSYVIFILATTEPHKIPATILSRCQRYDFKRVTVKDMTLRMKKICEDEGIDIDDKALNLIARNSQGALRDALSILDQCMSFGESKIDYKDVVELMGSVNIEQLFELSQCIVEQDTKKSLEILNEFVLWGKDIRNLINDLIDHFRNLMVCKVSSELDEIISLPEETIEQLKIQSKNIDINDLIRILNILSITQDDIKSSSNPRVLVEITIMKIAQPMFDESKEALIKRVENLEKMIELGNFKSEKIGNNKEKEYEVDREIDVKQENVVYEDVKNEDVILIESSWKNILKQIKKDKKMPIYALLSEVKSFNVYSNMLYVIFDDKFDFAKTRLSSQDTINYLEKTIRDVLNRSFNVKIVLTSEVKDINLEVKEKKDIGEEILKNIVSEEILEIKDSIDENESK*

>CD630_00210 Clostridioides_difficile_630_NC_009089 pyruvate carboxylase

MLKKFNKILVANRGEIAIRIFRACSELGIKSVGIYSKEDKYGLFRTKADESYLIGEGKGPIDAYLDMDGIIDLAKRKKVDAIHPGYGFLAENAEFARKCEENGITFIGPSSKVMNMMGDKINSKKIAKEVNVQTIPGVEKAIRSTEEAKEVANKIGYPVMIKASNGGGGRGMRIVHREEDLELEYETACSESRKAFGEDIIFIEKYIADPKHIEVQILGDNYGNIVHLYERDCSVQRRHQKIIEYAPAFSLDDKVRKEICEDAVKLSKHVGYSNAGTLEFLVDANGGHYFIEMNTRVQVEHTVTEMVTGIDIVQSQILIAQGYSLDSEEINIKSQDDVEIRGYSIQCRITTEDPKNKFMPDTGKIQVYRTGSGFGIRLDGGNGFTGANISPHYDSLLVKTISWDRTFQGAINKTIRSIKELRVRGVKTNVGFLVNVLNNPIFSNGKCSTKFIDENPDLFEITESKDRGTKLLQFIGDVIVNDNACKEKPLFDALHDPRMDKDGSKSEGSKILFDKLGKSAYIEKIKNDKKLLLTDTTMRDAHQSLLATRIRTYDLLKAAKPTEKYQKDLFSLEMWGGATYDVAYRFLKESPWRRLQKLREEIPSIMFQMLLRASNGVGYKNYPDNVIEEFTKESARQGIDVFRIFDSLNWVENMKPSINTALETGKIVEATMCYTGDILDKTKTKYNLEYYIKMAQELESLGADIIAIKDMSGLLKPYSAYTLVKELKKNVKAPIHLHTHDTSGNGVATCLMASEAGVDIIDAALESMAGLTSQPSLNAIVEALKNTERDTGIDLFGYDELGKYYKDLRKVYNKFESDLTNSCAEIYNFEIPGGQYTNLKPQADSLGLVNRFDEVKEKYKEANEVVGDIIKVTPSSKVVGDLAIFMTKNKLDKDNIIEEGKNLSFPDSVVDYCKGMIGQPEGGIPKDLQEVVLKGEEAITVRPGSLLPAEDFDEIAKYLNEKYDINANIRNVISYALYPKVYEDYIKHLQHYNDISKLESDVFFYGLNKNEECEVEIEEGKVLTIRLVEIGEVKENGFRTIGFELNGMVREVEIKDKNFSGKINNVEKADMNDPLQIGASIPGKVIKIMVKEEDEVKANQPLIVIEAMKMETIIVAKTDGVIKSIKVKEDDMVEDKQLLMIMK*

>CD630_00220 Clostridioides_difficile_630_NC_009089 elongation factor G

MKVYDSKMLRNVAVLGHSGCGKTNLIETIAYTANTNKIPKLTDKVNMTYSMGLIPIEYNDYKFNLLDTPGYFDFSGDVVSSLRASDAAIIVIDATAPIQVGTEKSLELTESIPKIMFINKIDNEKARYKDAIAMLREKYNNKIVPMISPIYKDKNFVKLHNVFENIDDLEGEFKEQAMSVKEALMELIAETDDQILDKYFNGEELTTEEIQKGIIIGIQRGDIIPVICGSTINNIGTKEILDTISSYLEPIFTEESKPFRGLVFKTMVDPFVGKMSYIKITEGVLSKDKDVFNINKNVKEKIANIYTLRNSELVEIEKAKAGDIVVITKVNSLKTGDTISADKDAEALEKIDFPKPQIYYAVTPKNKGDEEKVASVLNKLVEEDPTLHWYRNTETKQALLGGQGELHIKTIKNKMKDKFGVDVELNDLKVPYRETIKGTADVQGKHKKQSGGHGQYGDVKIRFERCESDFEFTEEIFGGSVPKQYIPAVEKGLKDSMQKGILAGYPVTNIKATLYDGSYHDVDSSEMAFKMAASAAFKKGMEEAHPILLEPIMKLKITVPEEYMGDVMGDINKRRGKIFGMEPDDKGKQIIFAEAPQAETFKYAIDLRAMTQGRGYFEMELERYGEVPSQFAEKIIGLATAK*

>CD630_00360 Clostridioides_difficile_630_NC_009089 acetoin dehydrogenase E1 component subunit alpha

MYMSISKETLLEMYKRMNQARKFEEKVSWFFARGMVHGTTHLSVGQEASSVAAVMALEKGDLVSLTHRGHSQFIGMGIDLNKMMAELMGKETGFCKGKGGSMHIADIESGNLGANGVVGGGLTIAPGAALTQQYKKTGKIVLCSFGDGASNEGTFHEGINLSSIWKLPIIFYCENNLYGMSTSIKRHMNIESIATRAASYGIEGISIDGYNPIEVYETVQKAAEKCRRGEGPVLIESRTYRWLGHSKSDANVYRTKEEIESWKAKDPIEFLKNYLIENNLSNEDELDKIQEFAKQSIEDAVEFAQNSPNPKIESLLEDVYAD*

>CD630_00370 Clostridioides_difficile_630_NC_009089 acetoin dehydrogenase E1 component subunit beta

MSTRELTYAQAIKEAMSEEMRRDENVIFMGEDIGIYGGAFGVSVGMIDEFGPERVRDTPISEAAIAGAAAGAAATGLRPIMEVMFMDFVTISMDAIVNQAAKMRYMFGGKAQVPMVVRCPGGSGTGSAEQHSQSLEAWFCHVPGVKVVAPSTPADAKGLLKAAIRDNNPVIFVENKLLYRKKGFVPEDDYVIEIGKADIKREGTDVTVITYGRMLQSVEEAAETLSKENINVEIIDLRTLYPLDKETIVKSVCKTGRVLICHEAAKTGGLGGEISALITESESFDYLDAPVKRICGKDVPIPYNPELEKAVVPRVDEIEEAIKSLIVR*

>CD630_00380 Clostridioides_difficile_630_NC_009089 acetoin dehydrogenase E2 component dihydrolipoamide acetyltransferase

MVANKIKATPAARSQARKDNIKLDRLIGSGENGRIHLVDVLNYLKDNKANTTPLARRIAEDLNIDLETIVGTGYNGKIRKCDVEKLTAKETIVSTNTSKSSEKKELKIENENSSMFNTVEGIFEKPNPMRATVAKRMSESYFSAPVFTFNIEVDATELKVLRAKLIDTVKESTGVKLTMTDLIVMAVSKILPNHQALNSAWTDEGIFRYKDVNIAIAVGLDEGLYVPVVKNANKKSLKEIAKESKELAEKVKTGKLMPADQEGNTFTISNVGMYGITTFTPIINMPSSAILGVGATQDKFVPVNGEAKIKPIMNLSLTSDHRVIDGTVAAKFLKDLKELLENPLSMLV*

>CDM68_RS00370 Clostridioides_difficile_M68_NC_017175 proline--tRNA ligase

MKMSKMFMPTLKEIPADAEITSHQLMVRSGMIKKMTSGVYNQLPMGLRVFKKIEQIIREELNKKDCQEILCAALLPSELWKESGRWTAMGEEMFRLKDRTEREYCLGPTHEEAFTDIIRQEITSYKQLPLNLYQIQVKYRDERRPRFGVMRTKTFTMKDAYSFDADDKGLDKSYQDMFDAYVSIFDRCGLENSPVQADSGAIGGSTSAEFMVKSEVGEDEVVFCSGCDYAANVERAESCNLASQKEEMKELEEVHTPGAATIKELEEFLKTSPDKFAKTLVYEADGKTVVVVVRGDREVNEIKVSNAIGSVIEFALATDDVVRKVTNAEVGFAGPIGINADYVFIDKEIVEQRNIVVGANKTEYHIKNANYGRDFEGIVGDFRNVQEGDKCIVCGKPLEIARGVEVGHIFKLGTKYSESMNANFIDKDGKSKPIVMGCYGIGVERTAAAIIEQHNDEKGIIWPLSVAPYHVVIIPANMKNEEQISIAENIYNDLQAMGVEVLLDDRDERIGVKFNDSELIGIPMRITVGKNINEGKVEFKLRHKEDKEIIDIEEINEKVKAEFIRNNVRLGQ*

>CD630_00500 Clostridioides_difficile_630_NC_009089 proline--tRNA ligase

MAKNEKQFVEEITKMEDDFPQWYTDVITKTDLVDYAPVKGFMVVKPYGYALWEKMQEFMDKKFKETGHKNCYFPLLIPESLLNKEAEHVEGFAPEVAWVTHGGNKKLEERLCVRPTSETIICTMYAKWLKSYRELPYLYNQWCSVVRWEKSTRPFLRTSEFLWQEGHTLHETAEEAQEETIQQLEVYKALCEELLAMPVVAGQKSESEKFAGGERTYTIEAMMHDGKALQSGTSHFLGQHFTKAFDITFADREGNLANPYHTSWGASTRLIGGLIMTHSDNRGLVLPPRVAPIQVVIVPIAAKKGNVMETVDKIYADLKAKGVAVEVDDRDNYTTGWKFNEWEMKGVPVRVEIGPKDIENNQAMVFRRDTLEKDSMPLEGLADAICDLFDVIHNDMFEKARKHREDNTSIVENMDEFRKALEEKPGFIKTMWCGDAECEAKIKEETGATIRCLPFEQENLGHKCVYCGKEADSMVVMAKAY*

>CD630_00520 Clostridioides_difficile_630_NC_009089 cysteine--tRNA synthetase

VKVYNTLTRTKEEFVPLEEGKVKMYVCGPTVYNYIHIGNARPFIIFDTLRRYLEYRGYDVTYVQNFTDVDDKIINRSHEEGISPEEVAAKYIKEYFVDCDGLGIKRATVHPQVTDNIQQIIEFIKELEDKGYAYAVNGDVYFDTNKFEGYGKLSGQKQEDLEAGARIEVNDQKRHPMDFVLWKAKKEGEPGWDSPWGEGRPGWHIECSVMSKRYLGETIDIHAGGQDLTFPHHENEIAQSEARSGKTFSKYWMHNGYININDEKMSKSKGNFFTVRDISKLYDLEIVRFFMLSAHYRNPVNFSDEMLNQAKAGLERLYNTKEKLEFTLSNLVESPLTEKEVELVKELDDFRQKFIDAMDDDVNTADAVSVIFELAKLINSNVDENSSLEFAKKCLDEFNELTGVLNIVNKKKDTVLDKDIEELIQKRTDAKKNKEFQLADDIRQQLLDMGIVLEDTRQGVKWKRI*

>CD630_00550 Clostridioides_difficile_630_NC_009089 23S rRNA (guanosine(2251)-2-O)-methyltransferase RlmB

VNNLASIEGRNPVIEAIKSDREIDKILIANSAKEGSIKKIIGMAKDKNIIIQYVDKHKLDEVSTSHSHQGVIAYASEYKYYELDELIDLAKNKDEDPFFIILDEITDPHNLGSIIRTADAVGAHGVIIPKRRSVHITPVVAKASAGAVEYMPVCKVTNIVNTIKRLKEEGLWIAAADMDGETFYKQNLTGPLGVVIGSEGFGISRLVKQNCDFIVKMPMIGNVTSLNASVAGGILLYEIFRQRLDKSK*

>CD630_00600 Clostridioides_difficile_630_NC_009089 transcription termination/antitermination protein NusG

MSELQEASWYVVHTYSGHENKVKATIEKAVKTRGMEDCIRQVVVPTEEVVETTKTGKEKTRQRKVYPSYVLVKMIITDESWYVVRNTKGVTGFVGPGSKPVPLSEDEVKAMGIDTTDPKVVNSDIDFEIGDTVKVSQGPFSGQIGNIEEIDLENREVKVCINAFGKRTLFVIELEGIEKI*

>CD630_00610 Clostridioides_difficile_630_NC_009089 50S ribosomal protein L11

MAKKVIGQIKLQIPAGKATPAPPVGPALGQHGVNIMGFTKEFNAKTADQAGMIIPVVITVYQDRSFSFITKTPPAAVLIKKALNLKSGSGEPNKKKVAKMTSAQVREIAELKMPDLNAASVEAAMSMIAGTARSMGVVIED*

>CD630_00650 Clostridioides_difficile_630_NC_009089 NADP-dependent dehydrogenase

MEKLQGKIAVVTAATKGIGLASAEILAKNGATVYLAARSEELAHEVINKISAEGGCAKFVYFNAREEETFTSMIEEVVKKEGKIDILVNNFGSTNPSLDKDLVTGDTDNFFDTVNTNLKSVYLPCKAAIPHMIKNGKGSIVNISSIGSVLPDLSRIAYCVSKAAINSLTQNIATQYAKDNVRCNAVLPGLIATKAALDNMSPEFIKEFLKHVPLNRIGEPDDIAKAVLFYASDDSSFITGDLLEVAGGFGLPTPQFADNILG*

>CD630_00680 Clostridioides_difficile_630_NC_009089 30S ribosomal protein S12

MPTINQLVRKSRKALEKKSTAPALQKGYNSLNKKVTDASAPQKRGVCTSVKTVTPRKPNSALRKVARVRLTNGIEVSAYIPGEGHNLQEHSVVLIRGGRVKDLPGVRYHILRGTLDTAGVDKRRQSRSKYGAKRPKEAKK*

>CD630_00690 Clostridioides_difficile_630_NC_009089 30S ribosomal protein S7

MPRKGNIPKREVLPDPMYGSKVVTKLINNLMVDGKKGKSQRIVYDAFAIVAEKTGEEALEVFNKAMDNIMPVLEVKARRVGGANYQVPIEVRPERRQTLGLRWLVKYTRARGEKGMVEKLAKEIMDAANNTGASVKKKEDTHKMAEANKAFAHYRW*

>CD630_00720 Clostridioides_difficile_630_NC_009089 30S ribosomal protein S10

MAKNEKIRIRLKSYDHKLLDFSAGKIVETAKKAGSQVSGPVPLPTEKQVVTILRAVHKYKYSREQFEIRTHKRLIDIANPTPKTVDSLMRLDLPAGVDIEIKL*

>CD630_00730 Clostridioides_difficile_630_NC_009089 50S ribosomal protein L3

MKGILGKKVGMTQIFTDKGVVIPVTAVEAGPMVVTQIKTVDKDGYNAIQIGFEDAKEKALNKPKKGHLAAANVLKKHLKEFRVDSVEGYTVGQEIKADVFEAGAKIDVTGISKGKGFQGPIKRHGQSRGPETHGSRYHRRPGSMGACSYPGRVFKNKKLAGHMGSVKVTVQNLEVVKVDADKNLILVKGAIPGAKGSVVTIKEAIKVSK*

>CDIF1296T_00140 Clostridioides_difficile_ATCC_9689__DSM_1296_strain_DSM1296_CP011968 50S ribosomal protein L4

MTNLEKGGITMPKLNVLNVSGQNVGEIELSDSIFGVEVNGHVLYEVVKNQLANKRQGTQSAKTRAEVRGGGRKPWKQKGTGRARQGSTRSVQWVGGGVAFAPKPRSYKYTLPKKVRRLAMKSALSSKVQNSEVIVLDALNMDAPKTKEFAQILNNINAAKKALVVIADKNDNVIKSARNIEGVQTALVNTMNVYDILKYDSFIITTDAVKKVEEVYA*

>CD630_00760 Clostridioides_difficile_630_NC_009089 50S ribosomal protein L2

MAIKKFRPTSPALRQMTVLVSDEITCNQPEKSLLVNLKKNAGRNVHGRITVRHRGGGQKRKYRIIDFKRDKDGIPAKVATIEYDPNRTANIALLNYADGEKRYILAPVGINVGDTILSGLGADIKPGNCLALKDMPVGTIIHNIELKPGKGAQLVRSAGVSAQLMAKEGKNALLRLPSGEMRLVSINCKATIGQVGNIEHGNVVIGKAGRKRHMGIRPTVRGSVMNPNDHPHGGGEGRSPIGRPSPVTPWGKPALGYKTRKKNKASNKLIVSRRTK*

>CD630_00770 Clostridioides_difficile_630_NC_009089 30S ribosomal protein S19

MSRSTKKGPFVHARLLKKIEAMNASGNKEVIKTWSRSSTVFPQMVENTIAVHDGRKHVPVYITEDMVGHKLGEFVPTRTFKGHKDDEKSNKRK*

>CD630_00790 Clostridioides_difficile_630_NC_009089 30S ribosomal protein S3

MGQKVNPHGLRVGVIKDWDSRWFATDKKEFGNLLLEDHNIRKFLKKRLYSAGVAKIEIERSANKIKMDLHVAKPGVVIGRAGAGIEALKAELEKMTKKTIIVNIVEVRSTDKNAQLVAENIALAIERRVAFRRAMKQAIQRAMKSGAKGIKVSASGRLGGAEMARTEGYSEGNVPLQTLRADIDYGFAEADTTYGKIGIKVWICNGEVLPTRDGVNPREESRKSDRRDNKRDNRRNDRRGNDRRGNDNRGNYRGQRPQGGSRPQRTENKGN*

>CD630_00800 Clostridioides_difficile_630_NC_009089 50S ribosomal protein L16

MLMPKRVKRRRVHRGSMAGQAHKGNKVTYGEFGLVALEASWITSNQIEAARIAMTRYIKRGGKVWIKIFPHKPVTRKPAETRMGAGKGSPEYWVAVVKPGRVMFELAGVSEDKAREAMRLAAHKLPIKCKFVKKEDLEVKGGE*

>CD630_00820 Clostridioides_difficile_630_NC_009089 50S ribosomal protein L14

MIQQESRLRVADNSGAKELLCIRVLGGSKRRYGNIGDVIVATVKSATPGGVVKKGKVVKAVIVRSKQGVRRNDGSYISFDENAAVIIKDDKTPVGTRIFGPVARELRDNEFMKIVSLAPEVL*

>CD630_00880 Clostridioides_difficile_630_NC_009089 30S ribosomal protein S5

MLRRKPIDAGQLDLQEKVVEVRRVTKVVKGGRNFRFAALVVVGDENGHVGIGAGKAMEVPDAIKKAVEDAKKNLIVVPIVGTTIPHEVRGHFGAGNILIMPAVEGTGVIAGGPARAVLELAGLKDVRAKSLGSNNPRNMVNATIEGLNSLKTVEDIAKLRGKKVEELLG*

>CD630_00890 Clostridioides_difficile_630_NC_009089 50S ribosomal protein L15

MKLHELKPAEGAVRAKRRLGRGTATGQGKTAGRGQKGQWSRSGGGVRVGFEGGQMPLARRLPKRGFNNIFKKVYTEVNVEVLNRFENGTEITAELLKSTKTISKIGKDGIKILGEGNLEKALTVKAAKFTASAQEKIEKAGGKAELV*

>CD630_00910 Clostridioides_difficile_630_NC_009089 adenylate kinase

MRIILLGPPGAGKGTQAVGIVEKYNIPHISTGDIFRKNIKEGTELGKKAKEYMDQGLLVPDELTVGLVTDRISQEDCKNGFMLDGFPRNVAQGEHLDIFLKNAGISLDKVVNIEVDKSILVSRAVGRRICKSCGATYHVEFNPPKVEGVCDVCQGELYQRADDNEETVSKRIQVYLDETKPLVDYYSKQGIIADIKGDQAIDKVFEDIVAALGSGK*

>CD630_00950 Clostridioides_difficile_630_NC_009089 30S ribosomal protein S13

MARIAGVDLPREKRAEIGLTYIYGIGKATANEILAKAEINPDTRIKDLSEDQVNELRKVIDDDFLVEGDLRREIALNIKRLRDIKCYRGIRHAKGLPLRGQRTKTNARTRKGPRKTVSRKKKK*

>CD630_00960 Clostridioides_difficile_630_NC_009089 30S ribosomal protein S11

MAKPKKKVTRIRRRERKNIERGHAHIQSTFNNTIITLTDVHGNAISWASSGQLGFKGSRKSTPFASQMAAETAAKAAMEHGLKSVEVFVKGPGSGREAAIRALQATGLEVTMIKDVTPIPHNGCRPPKRRRV*

>CD630_00970 Clostridioides_difficile_630_NC_009089 30S ribosomal protein S4

MARYTGASCRQCRREGMKLFLKGDRCYTDKCAIVKRNYAPGQHGQGRKKVSNYGLQLREKQKVKRIYGVLETQFRNLYERAENMPGKAGENLLSLLERRLDNVVYRMGLASSRKEARQLVTHGHFTLNGNKVDIPSLIVKVGDVIEVKEKSRSSAKFKNLVEVNSRIAPKWLEANVEGMTAKVVGVPTREDIDLEIAEHLIIELYSK*

>CD630_00990 Clostridioides_difficile_630_NC_009089 50S ribosomal protein L17

MAKYRKLGRETAHRNLMLRNLVTCLLRSGRIETTVTRAKETRRMAEKMITLAKRGDLHARRQVLAYVMDETVVNNLFTDLAPKYAERNGGYTRIIKIGPRKGDAAEMAFIELV*

>CD630_01000 Clostridioides_difficile_630_NC_009089 cobalt ABC transporter ATP-binding protein

MDNIVKVNNISFEYITDEAKLKAIDNLSLDVKKGEFVAIIGHNGSGKSTLSKNLNAILMPTEGNILIDDMDTKEEERLWDIRQTAGMVFQNPDNQIVATIVEEDVAFGPENLGIEPKEIRRIVEESLKSVGMYDLRDRQPHLLSGGQKQRVAIAGIIAMRPKCIIFDEATAMLDPSGRKEVMKTIKRLNKEENITVIHITHFMEEAVEADRVVVMEKGKKILEGTPREVFSKIKMLKEIGLDVPCMTELSSLLIEEGINISSDILTVDEMVMELCQL*

>CD630_01010 Clostridioides_difficile_630_NC_009089 cobalt ABC transporter ATP-binding protein

MSIIVKNLTHIYNEGMPFASKALDDVSFEIKDRDFVGLIGHTGSGKSTLIQHLNGLLKPSSGEIFINDFNITDKNLNLTEIRKRVGVVFQYPEYQLFEETIDKDIAFGPSNLGLEESEIHNRVKASMEAVGLDYEGFKDKSPFELSGGQKRRVAIAGVIAMNPEVLILDEPTAGLDPGGRDEIFNLIKDLHEKKNMTIILSSHSMDDMAKLAKTLIVMNHGSVEFMGTPREVFKSNASKLKDIGLDIPQVLELALKLREKGFDISEDILTLEEAKQEILKVVRGRGLC*

>CD630_01030 Clostridioides_difficile_630_NC_009089 tRNA pseudouridine synthase A

MRNIKIKIQYNGKNYCGWQKQPDSLGIQGTIERAIYDITKEETSLIGSGRTDSGVHAIGQIANFKINSGISIESIPMALNAKLPKDISVIEACEVNDDFHSRYSAKGKTYKYLVYNSKFRNPILSEISYQVKYELDFDKMCSEAKSLLGTHDFKGFMSSGSSVKDTVRTIYDIDISKKDDLITFEISGNGFLYNMVRIIVGTLVDMGRGRINEPFLDIIQSKTRSRCGHTAPAQGLFLKKVHY*

>CD630_01050 Clostridioides_difficile_630_NC_009089 30S ribosomal protein S9

MANVQYYGTGRRKSSVARVRLVAGEGNILVNGRALENYFNYETLIRDVKQPLVLTGNENKYDVIVKVEGGGFTGQAGAIRHGISRALLKADLDLRPALKKEGFLTRDARMKERKKYGLKAARRAPQFSKR*

>CD630_01170 Clostridioides_difficile_630_NC_009089 ferredoxin/flavodoxin oxidoreductase subunit beta

MAVVFKKTEGLQDTQTHYCPGCTHGIIHRLVGEVLEELGVLGDAVGVVPVGCSVLGYKYFNCDTQEAAHGRAPAAATGIKRVHPENTVFTYQGDGDLASIGTAEIVHAAARGEKITTIFVNNTTYGMTGGQMAPTTLVGQRATTAQSGRNAETQGYPIRVSEMLATLTGAVFVERVAVDTPAHVRQAKKAIKKAFQVQQAGLGFGIVEVLSTCPTNWGLAPNDALQWLRDNMIPYYPLGNFKNVEVEEVK*

>CD630_01190 Clostridioides_difficile_630_NC_009089 phosphoglucosamine mutase

VRKYFGTDGVRGVANTELTCDLAYKLGRAGGFVLAQGDHRVKVVVGKDTRISGDMLEASLIAGLMSVGCDVITVGIIPTPAVAYLTRKYGADCGVVISASHNPVEYNGIKFFNKNGYKLDDEIELKIEEYIDDIDKIDCLPIGENVGRKLHEHCAQRDYVDYLKSIISTDFKGLKVVLDCANGASYKVAPIVFDELGASVISINSSPDGNNINYKCGSTHPEQLQRAVLEHNADLGLAYDGDADRLIAVNEKGQIVDGDHIMILSALNLKKNNKLAQDTLVVTVMSNIGLTIAAKENGINLSTTAVGDRYVLEDMVKNGYNLGGEQSGHMIFLDYNTTGDGVLSSLILANIILQEKKPLSEIASIMSQYPQVLVNATIKNENKNKYMEYPEIKTEIERIESILDGNGRVLIRPSGTEPLVRVMLEGKEEGQIKELATNLANLIQEKLS*

>CD630_01200 Clostridioides_difficile_630_NC_009089 glucosamine--fructose-6-phosphate aminotransferase

MCGIVGYLGSRKAAEVIVEGLSKLEYRGYDSAGVAVNSSNEKELNIRKFKGRLSVLAEDLEKNPIDGNLGIGHTRWATHGEPSDVNSHPHFNQAKTIAVVHNGIIENYMEIKEELISEGVKFESQTDTEVIAHLVDKYYEGNLLDAVYKTISKLRGAYALGVICKEHGNELVAVRKDSPLVVGVGEGENFIASDIPALLKYTRDVYFLENGEVVHLKDENVTVYDSNRNLVEKEVFHVTWDVEAASKGGYDYFMSKEIHEQPTGVRETLERRLDDNGNIILDSINISKEDLEKINKVYIVACGTAYNAGLLGKYAIEKFVNIPVITDIASEFRYSDPFVDENSLVILVSQSGETADTLAVLRDSKAKGARILSITNVVGSSIARESDDVFYTWAGPEVAVASTKAYTTQITSLYMIALDFAIKKGTITREFYDSMISKMKEIPSKIQEILDNEEYIKEVAKTVVSSEHAFYLGRGIDYSLAMEGSLKLKEISYIHAEAFAAGELKHGTIALIEKGTPVIAIATQEKLFEKMVSNMEEVRARGAYVVAIAQSHNKDVEKAADKIIYIPNSDDILSPILAVVPMQLLAYHVSVLRGCDVDKPRNLAKSVTVE*

>CD630_01300 Clostridioides_difficile_630_NC_009089 S-adenosylmethionine synthetase

MARHLFTSESVTEGHPDKICDQISDSILDALLEKDPQSRVACETTVTTGLVLVAGEISTSAYVDIPKLVRETVREIGYTRAKYGFDCDTCAVITSIDEQSGDIAMGVDEGLESKTGEEIEEEIEKVGAGDQGIMFGFACNETPELMPLPISLAHKLSRRLTEVRKTGLVDYLRPDGKTQVTVEYEGSKAVRVHTVLISAQHCETVSNDKIREDLINHVIKEVIPAELLDEETKIYINPTGRFVIGGPQGDTGLTGRKIIIDTYGGYSRHGGGAFSGKDPTKVDRSAAYAARYVAKNIVAAGLADKCEIELAYAIGIARPLSIFIDTFGTGKVSEEKLVELVNKHFDLRPGAIIRDLDLRKPLYKKVAAYGHFGRTDIDLPWERTDKVEQLRKDALGE*

>CD630_01410 Clostridioides_difficile_630_NC_009089 copper homeostasis protein

MLEIIGMSVEDAKIIEDCGADRIELVSALTEGGLTPSFGLIESVVNSVKIPVNVMIRHHAKSFVYSKEDISIMQKDISVVKEIGANGVVFGVLDKNNNIDEKNLNVLLKCCDNLDVTFHRAIDESNTIDSVKILKDYDKITNILTSGGKGSIVHNIQMIKNMMLSSNHIKILLGGGLNFNNIEKIKELTKASNFHFGTAIRINNSPFEDIDRQKLKQLVNIISR*

>UAB_RS0201575 Clostridioides_difficile_ATCC_43255_NZ_CM000604 peptide chain release factor 2

LMKNIQINEEKMNQQDFWNDNEVAQRVLQENKSLKETLEEYESLKSLLEDIEVLIEIGLEEDDDSVERDIEKSIESMEEKLSEMKIKTLLNGEYDKNNAILSINAGTGGLDAQDWAQMLLRMYIRWSESKGYKVKLLDIISDPEAGIKTATILVEGTNAYGYLKSEKGVHRLVRISPFDPSGKRHTSFASIDVTPELDENIEVEINPSDLKIDTYRASGAGGQHVNTTDSAVRITHIPTGVVVQCQNERSQHLNKDRAMRLLMAKLIELKELEQKEKIEDIQGKYSQITWGSQIRSYVFQPYKLVKDHRTNAEFGNVDSVMNGNIDLFINEYLKMNKIV*

>CD630_01450 Clostridioides_difficile_630_NC_009089 S1 RNA-binding domain-containing protein

LDINQILKKEFNLRDEQINNTLKLIDEGNTIPFIARYRKEMTGEMSDVTLREFYEKLMYLRNLQSRKDDVVRLIDEQGKLTDEITQNIEKAKTLQEVEDIYAPYKQKKRTRATIAKEKGLENLALSILENNLDNIEIEAKNYLDEEKEVLSIEDALKGARDIIAELVSDDAKIRKYIRELALREGMIVSKSATDEKSVYDMYYDYSEAVKSMAPHRVLAINRGEKESFLKVKLEINNDKVLNYIINEYVNDKNFKNKEEIVSSIEDSYKRLIFPSIEREIRNHLTEIAQERAISVFGKNVKSLLLQPPVKDKVVMGFDPAFRTGCKIAVVDKNGKLLDYTTVYPTDPQNDVEGAKKVLKGLIEKYDIDIISIGNGTASRESETFVSEMIKEIDSEVQYVIVSEAGASVYSASELANEEHPDINVSIRGAISIARRLQDPLAELVKIDPKSIGVGQYQHDLNKKRLEEVLDGVVEDSVNSVGVDLNTASYSLLEHVAGISKAIAKNIIAYREENGDFTSRAQLKKVKRLGPQAFTQCAGFMRILEGKNPLDNTGVHPESYDICKKMIEIIGYSLDDVKNKNIGEIDEKIKEIGLRELSEKLEVGQVTLKDIIAEIKKPGRDPREEGIKPILRTDVLKIEDIQEGMTLKGTIRNVVDFGAFVDIGIKNDGLVHKSEMSNSFVKDPMSIVTVGDIVDVKVIGIDLNKKRVALSMKK*

>CD630_01460 Clostridioides_difficile_630_NC_009089 hydrolase

MIFIKNGKINTITNGIIHGDILIDEGKIIEIGEDLIAPLDVEVIDASNKLVFPGFIDAHTHLGLWEDGIGFEGADGNEETDPITPQLNPIDGINPMDRTFKEAFEGGITSVCTTPGSANVMGGQCIAIKTCGKRIDKMVIKNPVASKIAFGENPKSCYGQDDKSPQTRMAIAALLRENLKKAEEYLEDIDMYESHDDEDCEKPEYDIKMESLIPVLRREIPFKAHAHRADDMFTAIRIAKEFNLKLTLDHCTEGHLIVDELVEEEFPVIVGPSLSERSKFELRNLTFNTAGILSNAGLDVCIMTDHPVIPVQYLPICAGIAVKHGMKEEKAIESITINPAKTLGIEDRVGSIEVGKDADLVIWDNSPLEIQSNVLYTIINGKVVYEKK*

>CD630_01490 Clostridioides_difficile_630_NC_009089 P-loop ATPase

MAKIYLENENKTREIGYKLGKLLKEGSVICLVGDLGAGKTTMTQSLADSLGIEDYITSPTFTIINEYEGKIPLYHFDVYRIGSSDEMYDIGYDEYVNSNGICIIEWANLIEDILPKEYLNIELRYKDEGREMILTPKGEFYKEIVEELIK*

>CD630_01520 Clostridioides_difficile_630_NC_009089 UGMP family protein

MSDIITLAIESSCDETAASVLKNGREVLSNIISTQIETHKKFGGVVPEVASRKHVENIDIVVQEALDKANIGFNDIDHIAVTYGPGLVGALLVGLSYAKALAYTLNIPLVGVNHIEGHLSANYIEHKDLKPPFITLIVSGGHTHLVEVKDYGKYEILGKTRDDASGEAFDKISRAMNLGYPGGPIIDNLAKNGNKHAIEFPRAYLEEDSYDFSFSGLKSSVLNYLNGKRMKNEEIVVEDVAASFQEAVVEVLSTKALKAVKDKGYNIITLSGGVASNSGLRAKITELAKDNGITVKYPPLILCTDNAAMIGCAGYYNFINGKTHDMSLNAVPNLKINQ*

>CD630_01700 Clostridioides_difficile_630_NC_009089 ABC transporter ATP-binding protein

MIVLSCNNLNKSFGIDSILENISFTVNEGDKIGIIGVNGTGKTTLFKIISGIYGYDSGDIYTSKDCEIGYLEQNTNFYSDNTILEEVLEVFKNLIEMESYLRELEVKISEESTKTNSPIIEKIMDEYSHKLELFSDLNGYGYKSEAKGVLKGLGFSDNDMDKPISILSGGEKTRVLLGKLLLKKPTLLLLDEPTNHLDSEAIEWLEVFLKQYKGTVMLISHDRYFLDQSVNRIFEVHNKKLKVYNGNYSKFVELSKIEKELELKKFEDQQKEIKKQEESIERLKAYGREKHLKRARSKEKALDKVDVLDKPEAYRKKARIQFTPSVQSGNDVLQIRDVSMGYGERILFKDLDLDIYRGEKVALIGANGVGKSTLFKIITNELQPLSGNIKFGTNVHVSYFHQEQKTLNLDNTIIDEIWENNTHLTQTTLRNMLGAFLFVDEEVFKKISTLSGGERARVAILKLILSNANLLLLDEPTNHLDIDSKEVLEEALTNYDGTIFTISHDRYFLNTVVDKILVLDENGITEYLGNYDYYIDKKRQIQEMSIIEEKEEKTRTQIKDEKRKEREQREIEKKNRIKRQNIEKEIEKLEIEIEKLDILLCQEEVYSNPDKAKEVSQEKINLENNLASLYDEWEEFM*

>CD630_01790 Clostridioides_difficile_630_NC_009089 NAD-specific glutamate dehydrogenase

MSGKDVNVFEMAQSQVKNACDKLGMEPAVYELLKEPMRVIEVSIPVKMDDGSIKTFKGFRSQHNDAVGPTKGGIRFHQNVSRDEVKALSIWMTFKCSVTGIPYGGGKGGIIVDPSTLSQGELERLSRGYIDGIYKLIGEKVDVPAPDVNTNGQIMSWMVDEYNKLTGQSSIGVITGKPVEFGGSLGRTAATGFGVAVTAREAAAKLGIDMKKAKIAVQGIGNVGSYTVLNCEKLGGTVVAMAEWCKSEGSYAIYNENGLDGQAMLDYMKEHGNLLNFPGAKRISLEEFWASDVDIVIPAALENSITKEVAESIKAKLVCEAANGPTTPEADEVFAERGIVLTPDILTNAGGVTVSYFEWVQNLYGYYWSEEEVEQKEEIAMVKAFESIWKIKEEYNVTMREAAYMHSIKKVAEAMKLRGWY*

>CD630_01840 Clostridioides_difficile_630_NC_009089 aspartate carbamoyltransferase

MLKSRNLIQPEDFSIEEIDEILELAQKIIDNPSKYSRICEGKLLATLFYEPSTRTRLSFESAMNRLGGRVVGFSEPNSSSASKGETLGDTMRIVSGYVDIIAMRHPQSGAASEAARYTEVPFINAGDGKNQHPTQTLTDLLTIKSLKGTLESHTIGLCGDLKYGRTVHSLVKAMARYKNTKFVFIAPEELKMPDYIKEAIKGHAYYETNNLDDVIGSLDVLYMTRVQQERFEDKSEYERLKNYYILNKAKLEKASKDMLVMHPLPRVNEIDIDVDSDDRAVYFKQAKYGMYVRMALIIKLLGINED*

>CD630_01920 Clostridioides_difficile_630_NC_009089 cardiolipin synthetase 1

MGVIGTIFLFYLIISYLAGAIISVIILLENRDPAKTMSWLLMFIIFPGVGLMIYAISGRNIRKRKLFKTQKLANNIKEKKLFDTLEKITEIVELEKESIKQNKLLRDEEDGSYRKRVINMLLKTGMFPFTKNNKVDVFVDGNEKFKRLIEDIREAKDHIHLEYFIIKDSEIGRVLKEELIKKAKEGIKIRILYDDVGCWRFWFNRKFFREMREVGIEIAAFLPTKFPIIGGKLNYRNHRKIVVIDGIIGYTGGINIGDEYLGKNDKFGYWRDTHIRIKGISVYMLQMTFLIDWYYTTKEVLVTKNYFPSVGNVGESMIQVVASGPDSDWEDIHYAYFSAICQARKNVYIETPYFIPDESLLKAIKSAALSGVDVRIIFPKIADHKIVNIASYSYFEEILRAGGKVYLYNKGFIHSKVVIIDDKIASAGTANMDLRSFMLNFEVNAFIYDEEVIRVMTDDFFEDLSYCEELNLEVFKNRNIIQKIKESVARLFSPIL*

>CD630_01940 Clostridioides_difficile_630_NC_009089 chaperonin GroEL

MAKEIKFSEETRRALEAGVNKLADTVKVTLGPKGRNVILDKKFGSPLITNDGVTIAKEIELEDRFENMGAQLVKEVATKTNDVAGDGTTTATVLAQAIIREGLKNVTAGANPILLRKGIQKAVTVAVEELKNQSRIVETQEAISQVASISAGDEEVGKLIAEAMEIVGKDGVITVEESQTMNTELDAVEGMQFDRGFVSAYMVTDVDKMEAVLNDPYILITDKKISNIQELLPVLEQIVQQGKKLLIIAEDVEGEALSTLVVNKLRGTFDVVAVKAPGFGDRRKEMLQDIAILTGAQVISEELGYDLKEADLSMLGRASSVKVTKESTTIVDGSGDKKAIEDRVTQIKHQVEQTTSDFDREKLMERLAKLAGGVAVVKVGAATEVELKERKLRIEDALNATRAAVEEGIVAGGGTAFVSVIPAIGTLIESLEGEVKLGAQIVKKALEEPLRQIAINAGLEGAVIVQNVVNSEAETGFDALNEKYVNMIEAGIVDPTKVSRSALQNAASIASTFLTTEAAVADLPEKEDAGMPGMGGGMPGMM*

>CD630_01980 Clostridioides_difficile_630_NC_009089 GMP synthase

MKHELVLVIDFGGQYNQLIARRVRENNVYCEILPCTASIERIKEKNPKGIIFTGGPNSAYLEDSPTISKEIFELGVPILGICYGIQIMSHVLGGVVRKGNKQEKEYGKTAITYGKSSLFEGITTNSVWMSHTDLIEKVPEGFTIVANTNDCPVAAMENVERNLYGVQFHPEVEHCLEGDKILTNFLYNICKVKGDWTTDSFIEDKIKELKEKIGDKKALCALSGGVDSSVAAVLIHKAIGDNLTCIFVDHGLLRKNEGNDVERIFREKFDINLIRVNAEDRFLSKLKGVSEPEAKRKIIGEEFIRVFEEESNKLGKMDFLVQGTIYPDVIESGHGNAATIKSHHNVGGIPEDVDFQEIVEPLRELFKDEVRKIGLELGIEEGLIFRHPFPGPGLGIRVIGDVTKEKCDILREADAVYMDELRKAGLYREIWQAFATLPDVKTVGVMGDERTYAYLVGLRAVTSSDGMTSDWYKMPYDVLERISNRIINEVDGVNRVVYDITSKPPGTIEWE*

>CD630_02030 Clostridioides_difficile_630_NC_009089 UvrABC system protein A 1

MKINNYKNQSIITNPKKFENKYQDLPKTPIELLKVVQSLVIHGDQGKLYGISFNKQQSDEELLRTIPQMLKRIFEINSNPLTIPRNPKQRLVGMCRDYSLLLVSLLRYRGFEARMRAGFANYFESELTYEDHWLVEYYDTLKKRWIRIDAQIDDIQKNYFQINFDTHDVGKTDGFLTGSEAWIRCQQGHAHPDDFGYNKNWKGWHSVKGNLLHDFNNMIGLELLPWDLWTELSSKKYNQLTRAEKNLLDEMAEILSSGNIKIEDLNLLIEKLPEDYLKSIFSQLKILGISEIKELGNPLELEKKFKFTKSINKSIKNSLCHNKSSIYLKGGRQNNLKDVEVTIPKNQITVITGVSGSGKSSLAFDTIYEEGKRRYFENLSNGAKLSEQLQKPEFDLLQGLTPTIAIEQKKGSQNPRSTVGTLTSIWDYLRMLFVSIGKSYCPYCKIPLEKKNNTKNYCPHCQTIFSKINTSTFNANSHTGACHDCNGLGFTYQVNPQLIVKDPTISILDGATYYFGKLRGKKPNGNWMVGELYAIAKDKNIDLDIPWNELPRDFIDAILYGTDDKIYEFSFESKGRESKIRRPASGAINHIQRLFRESSSENNTLHQYMNKIPCNTCGGELLCIEARFTTIKGYRFPELTKMTIEQLWNWLCELPNQLQKNELSLVNDILTELKIRVSYLLKVGLSYISTDRTAPTLSGGELQRVRLSSQLGSELVGLTYILDEPSIGLHPRDHNLIIKMIEELRDKGNTVIVVEHDKDTILSADYIIDVGPSAGTKGGFIIAEGTTQEIIKNPNSITGKYLSTYNKTGSQNKTIPSKWLSLKGCHANNLKNIDVEIPLNCMCSITGVSGSGKSSLVFHSLLPALEEKLKQKSIPDKNYTEFTGFDAIDDFILMDQTPIGKSSRSTPATYINIFDEIRSLFAETPQAKQKLLDESYFSFNSKKGQCPNCQGLGKTKIILQYMADQWVTCSECQGKRYQKEILSIQYKGKTIADILDMEVAEAKTFFSDCSDIYRKLSLLDEVGLGYLKLGQNTLGLSGGESQRIKLAKELGTKTKKRMLYILDEPTTGLHFKDIENLLITFRKLVNEQHSLLIIEHNTEVIRASDWIIDIGPDSGINGGEIVASGTPDEIKINPNSITGHFI*

>CD630_02170 Clostridioides_difficile_630_NC_009089 nitroreductase

MLEVIKNRHSIRTYIDKNIEEDKITEILKSAMQAPSSKNAQPWEFIIVDDKELLKQLSKSQHRAKHIEFAPLCIVVLGNRDKFLKPGKWIQDLGACTQNLLLEVTNQGLAACWAGVFPKNKVVNKVRQTLDLPLKLVPYALISIGYSEEKNEFIDRFDENKIHRNVYKNR*

>CD630_02180 Clostridioides_difficile_630_NC_009089 5-(carboxyamino)imidazole ribonucleotide mutase

MKVAVVMGSKSDYPKLEEGIKLLEKYGIEVVARALSAHRTPEQLSIFLKEIEDDTDVIIAAAGKAAHLPGVIASQTLIPVIGLPIKSSTMDGLDSLLSIVQMPKGIPVATVTIDLGLNAALLALQIMTLKYPKLKEDLKSYREEMAQKVLEDDKNLRG*

>CD630_02190 Clostridioides_difficile_630_NC_009089 phosphoribosylaminoimidazolesuccinocarboxamide synthase

MLLYEGKAKQVYSTDNENEYVVYYKDDATAFNGEKKAEISSKGILNNKISTIIFEMLKENNINTHFIKSLSDREMLVKKVEILPLEVIVRNIAAGSICKRVGLEEGVVFDEPIFEISYKNDAYGDPMLNDDYAVAMKLATREELKFLREETLKINELLKAFFLKLNLKLVDFKIEFGKDSEGNIILADEVSPDTCRLWDVNTNEKLDKDRFRKDLGDLVEGYTEVLSRMNNK*

>CD630_02200 Clostridioides_difficile_630_NC_009089 amidophosphoribosyltransferase

MCGVLGIYSNKDVTKELYYSLYSMQHRGQESCGLALLDDGEIKYKKDMGLVGDVFKENELSKLKGNIGIGHVRYSTAGGSHVSNCQPLVGSCRKRQLAIAHNGNLVNANYLKDMLEEDGYMFQTNSDTEVILYILARYYKGDIVESLKVTMDYIKGAYALVIMSQEELVAVRDPHGFRPLVLGKKGDEYIFASENCAIDILGGEVIRDVEPGEIIVVKDGELKSYFYSENYKPVKKSCIFEHIYFARNDATIDNVNAYEFRIKCGERLAQNETVKADMVVPVPDSGWPGAIGYANASGLKISEGLVKNRYVGRTFIKPTQEEREIAVKIKLNPLSTIIKGKSIILVDDSIVRGTTSKQLVKSLREAGAKEIHLRITSPPVAYSCYYGIDTPNRSKLIASSNNVEEMREYIGCDSLKFLDIEGMLDATEHKSTFCKACFDGEYPVKKIDKEELLSC*

>CD630_02210 Clostridioides_difficile_630_NC_009089 phosphoribosylformylglycinamidine cyclo-ligase

MLTYKESGVDIDEGNRAVDLIKGKIKGTYDGNVVGDLGNFSGLYSLKDFVGMKEPVLLASTDGVGTKLKIAQMMDKHDTVGIDLVAMCVNDLICQGAKPLFFLDYIALGKLVPEHIEKIVGGIADGCKMSGCALIGGETAEMPGMYGEDDYDLAGFSVGIADKEKIVSGNNVKSGDVLVGISSSGVHSNGFSFIRKIFLETYNYKMEQYVEELGMTVGEALLTPTKIYVKLALDVLAKHDIKAIAHITGGGLIENITRVIPKGLGLDINKKSWEKPPIFKMIEGFNAVDERELHKSFNMGIGLVLIVDKENADDVVNFINNRENDNADYVDKKYSELLEDKAYIIGEVVDSHEGVELC*

>CD630_02220 Clostridioides_difficile_630_NC_009089 phosphoribosylglycinamide formyltransferase

MLNIGVLISGGGTNLQAVIDGTESGEIKGQVKVVISSKQGAYGLERAKNHNIKAICETDEDKIIEILKENKIDLVVLAGYLKIISPKLVNEFRNKMINIHPSLIPSFCGAGFYGEKVHQGVIDYGAKVTGATVHFVDEGADTGPIIMQDVVKVNQDDDAKTLAKRVLEVEHRILKESISLFCENKLKLQGRRVFINE*

>CD630_02230 Clostridioides_difficile_630_NC_009089 bifunctional phosphoribosylaminoimidazolecarboxamide formyltransferase/IMP cyclohydrolase

MSKRALISVTDKTGVVEFAKELNKLDYEIISTGNTFKTLKENGVNVMQVEDVTNFPEILDGRVKTLNPYIHGGILYKRDKESHVETVNEHKIHSIDLVAVNLYDFEGTLKAGKSHDEIIENIDIGGPSMIRSAAKNYKDVIVVVDIKDYDSIIEKLKTDTMTLEDRKKLSYKAFSTTGRYDALISSYFAGEVGDTYPDILNLTFQKEQTLRYGENPHQNGFLYSQSNAKNPILNYEQLGGKELSFNNLNDLHGCLEVMREFKDSEEVVSVAIKHANSCGVGLGKDAFEAYTKCYEADKVSIFGGIVGITSTIDKATAEKLNEIFLEIVVAYDFEPEALEILKQKKNLRILKLAKIENSLQPYEMKYLDGKLLIQDRNNILAEKSENVTKEKPTDAQLKDMEFGMRVVKNMKSNAIAIVKNGQTLALGCGQTSRIWALKNALENNKDKDFTGAVLASDAFFPFDDCVTLAHEYGISAVVQPGGSIKDKDSIEACDKYDMVMVFTGIRHFKH*

>CD630_02240 Clostridioides_difficile_630_NC_009089 phosphoribosylamine--glycine ligase

MKILVVGGGGREHAICWKLSKEKNVEKIYCAPGNAGIANVAECVNIGDTNIEELLKFAKENEIGLTIVGPEVPLVMGIVDEFEKEGLRVFGPNKKCAQLEGSKAFSKEFMIKHNIPTAKYKEYTNLEEAISEIDSFGYPVVIKADGLAAGKGVVIPENREDAIATLKEMMSDKKFGAAGDKIVIEEFLKGIETSILAFVDNDTIVPMASAKDHKKVNNYEQGPNTGGMGTFSPSEIYTEELANKVKETVLEKTLEGFKKDGLNFKGILFVGLMITEDGEKVLEYNVRFGDPETQSVLFRLETDLHEIMEAILDNKLKDIEINYSDDEAVCVMLTSGGYPDSYEKGKIITGLENLDDDIVVFHSGTKMFDGNLVTNGGRVIGITAKSTTVKDAAEKVYENIKKINFEGMHYRTDIGR*

>CD630_02250 Clostridioides_difficile_630_NC_009089 phosphoribosylformylglycinamidine synthase

MLNTENKDSMVRRVLVEKREGFDLEAKALKKDLVESLHIDNIENLRILNRYDVEGISEEVYENAAKTIFSEPNLDVVYYEEIPKLNDERVFAIEFLPGQYDQRGDWAAQCVQIVNQGIRPAINTAKVYILSGKITDEEFSKIKDYCINPVDSREASLEKPETLKMETEIPTTVEVLDGFIDLDEKGLRTFVSEKGLAMTLGDLQHVQKYFKDTEKRNPTITEIKVLDTYWSDHCRHTTFMTEIENVKIEDGKFNDIVKEAYQMYLNSRDNVYVNRHKDICLMDIATVAVKELKKNGKLNDLDESEEINACSINVDVEVDGKMEKYLVMFKNETHNHPTEIEPFGGAATCLGGAIRDPLSGRSYVYQAMRVTGSADPRTTLEDTLPGKLMQRKITTEAAHGYSSYGNQIGLTTGQVAEVYDENFVAKRMEIGAVIAAAPKENVVRERPEAGDVIVLLGGKTGRDGCGGATGSSKEHSEESILTCSAEVQKGDAPNERKIQRFFRNKEVAQMIKRCNDFGAGGVCVAIGEIADSLDINLDLVPKKYDGLDGTELAISESQERMAVAIKKENKDKFIQLAVEENLEATHVATVTDTGYLRMFWNGKAIVDINREFLDTNGVKQTTDVHVTKVDEENTFFSSNEIVKDVKCSSMKDKFTKVLSDLNVCSQKGLVEMFDNTIGGNTVLMPFGGKYQATPTQGMVAKIPVLGGETNTSTIMTYGYNPKVGKWSPFHGALYAVVESVCKLVAIGGNYSTTRLTFQEYFEKLGNNPEKWGKPFSALLGAFYAQSKFEIPAIGGKDSMSGTFKDIEVPPTLVSFAVDTVDAKKVVSPEFKKADSKVVMLCVNKAENDVVDFEELKRNLDKVRELIHGNKVLSTYALGFAGVGEAISKMAFGNKIGFKFSEEAEKAFTDDKLFEASYGNIVLELANDDLSMLEGYNYVVLGSTVKEASIFIKGEELALDELYKAHCSTLEPIFPTKTEEVKSKIETISYISQGEAKKSSLSIATPRVFIPAFPGTNCEYDSARAFERAGANASIRVFKNLTYKDIEDSIDTIVNEIKSSQIIMLPGGFSAGDEPDGSGKFIATVFRNPRVQEAINEFLTQKDGLMLGICNGFQVLIKLGLVPYGEIRVPSESAPTLTYNNIGRHQAKIARTRISSNKSPWLAQTNVGDIHNIAISHGEGKFVASEDVMRELIANGQVATQYVDFNNEATYDIEFNPNGSFYAVEGITSADGRVFGKMGHSERIGEEVYKNIIGEKEQKIFESGVKYFR*

>CDIF1296T_00517 Clostridioides_difficile_ATCC_9689__DSM_1296_strain_DSM1296_CP011968 ABC transporter ATP-binding protein

MMNIVSINGLSKGFGNRKIIDNLNFTVPEGSVFGFVGKNGAGKTTTMKMVLGLLKPDSGTIDVCGEKVTYGKTSSNRHVGYLPDVPEFYNYMRPLEYLSLCGEITGLSKKEIQIRSEELLSLVGLRNEKRRIGGFSRGMKQRLGIAQALLSRPKLLICDEPTSALDPVGRKEILDIMLKIKDSTTVIFSTHILSDVERICDHVAILNKGSIALSGTLSEIKSMHGKDRLLLEFASNDEIQKFKSSDGIKSLLKDSEETNMEIVLHGKDIKAIQKTVISTLAEMNLCPVKMELIELSLENLFLEVVK*

>CD630_03130 Clostridioides_difficile_630_NC_009089 K/Mg/Cd/Cu/Zn/Na/Ca/Na/H-transporting P-type ATPase

MEANNSIKKEFILGGLNCAHCAEEINNKVSKLQEVKSSNLNFINKKLTVNIKESFNEDTTIEKIIDIIDSTEPGLDIQISSKENAASKTSIKKELILGGLNCAHCAEEINNKVSKLKEVESSNLNFVNKKLTVNISNNFEEDDVINKIKEIINSTEPGLDIQVGSTDKVKGRTTEKSGAVNDTNKKELIPLIIGALVYIFGIYQTATGYESQFSNIVFIVAYVIVGGDVLLRAIRNISKGRVFDENFLMALATVGALAIGELSEAVGVMLFYKVGEYLQGVAVGKSRKSITSLMQIRPDYANLKVNSEVKVVSPEEVNVGDIIVVKPGEKVPLDGVVVDGVSMLDTSALTGESVLREVEKGDEILSGVINKNALLSIEVTKSFGESTVSKILDLVENSSIKKSKTENFISKFSRYYTPIVVIAALLIAFVPPLVISGEVFSDWLYRGLIFLVVSCPCALVLSIPLSFFSGIGFASKNGILIKGSNYLEALRSVDTVVFDKTGTLTKGVFNVTKLNPEGISDEELLEYAAIAEVNSNHPIAKSILSYYNKKIDLDTIDSYEEIAAYGIRVKHNGNFILAGNEKLMKKENISYSSAKEVGTVVYIAVDKVYRGYIVISDEVKEDSKNAIRSLKEIGVKEVVMLTGDNEKVAKNIAQELELDTVYSNLLPNEKVDRLEDLYEGRTEKEKIAFVGDGINDAPVLARADVGIAMGGLGSDAAIEAADVVLMTDEPSKISKAIEIANKTNKIVWQNIIFALGVKIIVMILGAGGVATMWEAIFADVGVALIAVVNAMRAMR*

>CD630_03270 Clostridioides_difficile_630_NC_009089 cobalt ABC transporter ATP-binding protein CbiO

MMFKINNLTYQYEKNTNALLNINMDFSKGNVIGIIGSNGSGKSTLFMNLMGILKPTSGEILFKEEKLKYDKRSLYNLRKNVGIVFQDPEKQIFYSKVYDDIAFAMRNIGMDEKTIKERINKALVAVNGIDFIDRPVHFLSYGQKKRVAIASVIAMENEIVLLDEPTAGLDPVSTRSIVDIIKGLNKNNIKIVISSHDMNLMYEICDYIYVLDKGILIDEGKAENVFINENNIIQAGLESPWLVKVHRNMNLPLFKKEEDLYKYWKERELNTNK*

>CD630_03340 Clostridioides_difficile_630_NC_009089 bifunctional acetaldehyde-CoA/alcohol dehydrogenase

MEKKEKVVEKSNVEVCSPEIVNSVETLRMRLEEIRLAQKEFATFTQEQVDKIFLAASTAANQQRIPLAKMAVEETGMGIVEDKVIKNHFASEYIYNAYKDTKTCGVIEKDEAFGFTRIAEPVGVLSAVIPTTNPTSTAIFKSLIALKTRNGIIFSPHPRAKNCTIEAARVVHDAAVKAGAPKGLIGWVDVPSIELTNVVMAEADLILATGGPGMVKSAYSSGKPAVGVGPGNVPAIIDESADIKMAVSSILVSKSFDNGMICASEQAVIVPEKIYEEVKKEFKYRGAHFLNKEETEKVGKVVIIDGSLNARIVGQPAHVIAKMADVEVPKTARIIIGEVESVELNEPFAHEKLSPVLAMYKSKSFEDAVAKAEKLVADGGYGHTSSLYADSINHPERVEKFVNAMKTCRVLVNTPSSQGGIGDLYNFKLAPSLTLGCGSWGGNSVSENVGVKHLLNIKTVAERRENMLWFRAPEKVYFKKGCLGVAAREFKDVMDKKKAFIVTDSFLYNNGYTKKLTDLLDEMGIKHTTFFDVAPDPTLACAREGAKAMADFQPDLIIAVGGGSAMDAGKIMWVMYEHPEVDFQDLAMRFMDIRKRVYVFPKMGEKAYFAAIPTSAGTGSEVTPFAVITDQDSGVKYPLADYELMPNMAIIDADMMMEMPPRLTAASGVDALTHALEAYVSMLRTEPADGLALQAGKIIFEYLPRAYKNGKNDKEAREKMAMASTMAGMSFANAFLGICHSLAHKLGAFHHVQHGVANALLINEVIKFNCAEAPNKMGAFSQYRYPDCIQRYAEFASFAGIKGSTDQEKVDNLIKAIDELKAKVGLPKTIKEAGVEESKFLERLDAMVEQAFDDQCTGANPRYPLMSELKEIYLKVYYGK*

>CDIF1296T_00565 Clostridioides_difficile_ATCC_9689__DSM_1296_strain_DSM1296_CP011968 ABC transporter ATP-binding protein

MPILETINLGKIYGKKETSVHALKNANLKINKGEFVAIIGPSGSGKSTFLHLVGGLERPSNGTIKVAGKDICCLSDKELARYRRQKVGFVFQQYNLIPVLNVKENIELPLKLDNKKIDKEYIEDLINLLGLKERKNHLPNQLSGGQQQRVAIARALSAKPSIILADEPTGNLDSKTTEEVMDLLKSSIKKYNQTLIIITHNENIARKADRIISIIDGELKLTL*

>CD630_03940 Clostridioides_difficile_630_NC_009089 D-lactate dehydrogenase

MKILVFGARDYEEPVIKKWSEEHKDVQVDIYPENMTEENVVKAKGYDGISIQQTNYIDNPYIYETLKDAGVKVIASRTAGVDMIHFDLVNENGLIVTNVPSYSPNAIAELAVTQAMNLLRKTPLVKKKVCEGDYRWIAELLGTEVRSITVGVIGTGKIGATSAKLFKGLGANVIAFDQYPNSDLNDILTYKDSLEDLLKEADLITLHTPLLEGTKHMINKDTLAIMKDGAYIVNTGRGGLINTGDLIEALESGKIRAAALDTFETEGLFLNKKMNPGELTDPEINKLLSMEQVIFTHHLGFFTSTAIENIVYSSLSSAVEVIKTGTATNRVN*

>CD630_03990 Clostridioides_difficile_630_NC_009089 acyl-CoA dehydrogenase

MLYNKEQELLRKAVRDFVSKELDTLPAEMDKTGVMPKELIKKLADAKFISSNIPEEYGGGGAGYVSYAIVMEEIARRCASTATFVTAGSSLASLPILYNGTEEQKQKYLKGIATGELIGAFGLTEPGAGSDAGGQQTTAELVGDHYILNGRKTFITNGPFCDVAIVIAVTDRSKGLRGTSAFIVESKWDGFSTGAHEDKMGIRGTETSDLIFENVKVPKENLLGKEGQGFKIAMGTLEVGRIGVAALALGIAQGALDEAVKYTKQRVQFGKPIAKFQNTQFTIADMETKVCAARGLVYDAAQKRDAGMRVAQESAMAKYYASEIANEVAYKALQLHGGYGFIKDYEIERMYRDARIVSIYEGTSEVQKMVISSNVLK*

>CD630_04010 Clostridioides_difficile_630_NC_009089 electron transfer flavoprotein subunit alpha

MNDIKDLSSYKNVWIFAEQREGKIAPVVIELLGEGRKLAKEVDAELCAILLGKDVDGLAKELITFGADKVYVADDALLEKYTTDAYTKVIKDAIDEIKPEIMLFGATHIGRDLAPRIASRVGTGLTADCTKLEIDPEDKKIKQTRPAFGGNIMATIICPNHRPQMSTVRPGVMDKAEKDETRTGEVIALDYKITQDDIRTTVLETVKTKKDLVSLTDANVIVSGGLGLGGPEGFEMLKKLADKLGGVVGSSRAAVDAGWIDHSHQVGQTGTTVKPNLYIACGISGAIQHLAGMQSSDFIIAINKNPAAPILEIADYGVVGDLHEIVPMLIEKLDSVDDLLEAIKA*

>CDIF1296T_00618 Clostridioides_difficile_ATCC_9689__DSM_1296_strain_DSM1296_CP011968 pyridoxal-phosphate dependent enzyme

MNNSTSMKDMSYQAVMGRNNEIMKNAIGLDYSSFEQEGIGFDYEKMMSETGYTLQDIEAIQSQYAVGNTPLIELKNLTKLARKCAKEGKGARIFVKDEAMNASGSFKARRAATAVYHAKQMGYKGVIAATSGNYGAAVASQAAMQGLKCIIVQECYDSNGVGQPEIIEKARKCEAYGAEVVQLTVGPELFYTFLVLLEETGYFNASLYSPFGIAGVETLGYELAIQFREKYKKDPDIVVCTNAGGGNLTGTARGLIKAGSINTKVVGASVDLKGLHMASDNQFNKKSFTTGHTGFGIPYCTWPDRSDVPRSAARPLRYMDRYVLVKQGEVFYTTELLAQLEGIERGPAGNTSLAAAFSLAQELDEDKAIVVQETEYTGAGKHICPQLTFARENGIDIKFGNPREEIAGVNLILPERPELLKCVDIDMNKIRKSFIKNCISNNHIDNIDKLSNRDIEFLMEEVKSSRDFVVDVLNNL*

>CD630_04590 Clostridioides_difficile_630_NC_009089 ABC transporter ATP-binding protein

MEILKCENLTKIYGSNQTRVTALNNVNLSVQKGDFVSIVGASGSGKSTLLHLLGGVDRPTSGKIYVEDTEISSLKEEALAVFRRRKVGLIYQFYNLIPTLDVRKNILLPMLLDKRKVDEDRFSEIVSILGLSDRLNHLPSQLSGGQQQRVSIARSLIYRPAILLADEPTGNLDRKNSEEIVDLLNLSNKRFNQTILLITHDEKIALEANRIVTMEDGVIVSEKVVKK*

>CD630_04840 Clostridioides_difficile_630_NC_009089 ABC transporter ATP-binding protein

MIIKAKQLSKIYGSNNNKVIALNNVNLEINSGEFVSVIGPSGSGKSTLLHILSGLDNPTSGQVLLDDKDIYKHTEKELSALRRKSFGFVFQQFNLLPVLTASENISMPVLLDKKQPDKGYLNEISSLLGIADRLNHLPHELSGGQQQRVAIARALIAKPDIIFADEPTGNLDSKSGSEVMNLLIKTSKQFGKTLVVITHDDRIAKLADRKISIIDGVLMEVK*

>CD630_04900 Clostridioides_difficile_630_NC_009089 sugar-phosphate dehydrogenase

VKSVRFYGIRDTRVEDVDVPKILEKDDVIIKVKVAGICGSDISKYSKTGPHMVGEILGHEFSGEVAQVGKEVRSFKIGDRVAVCPAMPCFECDECKKGLYSRCNNVAIIGNKELGGCFAEYTKVKERNLIKIPDEISYETAAALEPVCIAGHGLFRSEAKVGDTVVVLGTGPIGLFSIQWAKIFGSTKIIAVDVFDEKLDLAKELGADICINAKEKNIVEEIKRLTDGDGADIVIESAGTPLTCGQVLLLAKKGGTVLYAGVPYGDVALTREQFEKIVRSELTVKGTWFGNSFPFPGKEWSAGLYHMQKGDMNVEKLVTHRINLEEAPAYFEKVYKRDIFFGKIMINIDN*

>CDIF1296T_00724 Clostridioides_difficile_ATCC_9689__DSM_1296_strain_DSM1296_CP011968 endonuclease III

MKNVNLKEINEKDVILEEEINKKGMDKKNIIEKEEINKKGIDKKDIIEKKEINKKGIDKKDIIEKKEINKKGIDKKDIIEKKEINKKEMDKKDIIEKEEINKKGMNKKDIIEKEEINEKGIDKMIGKKDEIIDEKDIENFMDIEEDKKSIKVSKKSTKKGETEKKTKKSSSNKATQKTSKETKSKSKIKNESANEKDVNKILDELEKLYPDAKCELNYGTAFELLIATILSAQCTDVRVNKVTSELFKKYNTARDFANLSIEEISKEIKSCGLYKSKSQKIKDTSEQLCELYDGEVPDSLEKLIKLPGVGRKTAGVVLSNAFNHPAIAVDTHVFRVSNRIGIVDEPNPQKTEFALMEAIPKERWSHSHHVLIFHGRRMCKARNPECASCPIKEDCNYYKELNETK*

>CD630_05740 Clostridioides_difficile_630_NC_009089 threonine--tRNA ligase

MIKVALKDGSIKEFENAISVMDVAKSISEGLARNVVAASVNGEVVGLDHIIDTDCDLNLFKFEDKEGKEVFRHTSAHILAQAIKRLYPEAKLAIGPSIENGFYYDIDLDHRLVPEDLEKIEAEMKKIAKEDLKIERFELPRNEALELMKEQGEDYKVELISDLPESEIISFYKQGDFTDLCRGPHLPSTKKVKAVKLQSVAGAYWRGDENNKMLQRIYGTSFEKNKDLEEYLHLLEEAKKRDHRKLGKELGLFMIPEEGPGFPMFLPKGMELKNELLKFWREIHRKAGYIEIESPIILNRKLWETSGHWYHYKENMYTVKIDDEDYAIKPMNCPGGLIYYNSQLHSYRDFPMRVAELGRVHRHELSGALQGLMRVRAFTQDDSHIFMLPEQIKDEIKGVANLIDGIYKTFGFEYNLELSTRPENSMGSDEEWEAAENGLREALEELGLPYTINEGDGAFYGPKIDFHLKDCLGRTWQCGTIQLDMQLPRQFDNTYIGQDGEKHRPVMIHRVAFGSIERFIGILIEHYAGKFPVWLSPTQVKILPISDKFMDYANEVKKELFDKGIRVELDDRAEKIGFKIREAQLEKVPYMLIVGEKEVADNNVSVRSRDKGEIGSIKLDEFIASISKEIESRESIIQD*

>CD630_05800 Clostridioides_difficile_630_NC_009089 glyceraldehyde-3-phosphate dehydrogenase

MFNELKTKENVYKNLINGKWVESNSRKPIEIYSPIDNSLVGKVQSMTKHEVDEVIKNTKESIKVWAEMPVYKRANIFHKAADLLLENIDEIANILVLEIAKDIKSARAEVERTADFLRYTADVGKNMEGEAISGDNFPGGTRNKMSYVSRVPLGTVLAISPFNYPVNLSMSKIAPALIGGNAVVLKPATQGAISALHVVEIMRKAGIPDGVLNTITGRGSEIGDYVVTHKGINFINFTGSTEVGQHISKISGMVPLLLELGGKDAAIVLEDADLDFAAKNIVSGAYSYSGQRCTAVKRILVQESVADKLVGKIKPLVEKLTIGNPMDEVVITPLIDNKATDFVQGLVDDALHKGAKLITGNVRKNNLFYPTLLDNVNVDMKIAWEEPFGPVLPIIRVKDINQAIEIANQSEYGLQSSVFTSDIDKAFYIADKLEVGTVQINNKTERGPDHFPFLGVKASGMGTQGVKYSIEAMTRPKAVVVNVREL*

>CD630_06450 Clostridioides_difficile_630_NC_009089 lantibiotic/multidrug family ABC transporter ATP-binding protein

MNYIIETHQLKKIYKDKAVVNAVNIHVKKGEIYGFVGPNGAGKSTVMKMLLNLVKPNSGEIVMFGKKVAETDFEILKKIGTIIENPYFYENLTAKQNLDLHCEYMGYYNKEHISEVLECVGLSKQSNKKVSKYSLGMKQRLAIARAILTKPELLILDEPINALDPEGIREMRELFRKLNTDYGITIFISSHILSEVEQIADTIGIIQDGKLIKEISMSDIHKYQTDYVEVDVDNVELAGYLLEKEFGIKDFKITSESCIEIYDLRKDVKEISKIFIQNQIGINSIGRKQSSLEDYFFQTTGTGGKETL*

>CD630_06500 Clostridioides_difficile_630_NC_009089 peptidase

MEKIKIESSDIFEYIFPHDISCSPDGKHIAYIISNINEEKDCYEHDLYVMDIKTEKQIHMTQTKDVTSFSWISNTELLFTSKRNKPKAGTTDFYTISIEGGEAKKAFSIPKACSVPVSLGNKLWLLTTKNPTDSKKSEPDRAVEGVDYWTFTDKPFIRDGENFSQRRRVTLELYQEGENITKAITPKFCEVAGIDVSSDKNRILYTGQIYEDCATPFSGLWEYHIDSGETKELVPQGKYQISLAKYIGKDKVMLQASTLDRSITQNHDIFILDLSTSEINMIASPDGMYATLLDVDAVYGGGRSNKVIGDKFIGARICRTMTEFNEFDTKTGNIRIITKVDAFTSFDIYDNTMYTVMLKDYELAEIYSIDMTTGTMKKMTAFSKPYLDTHKVSLPEKLTFVAKNKEEVDGFVIPPIDAKEGEKYPAVLFIHGGPKWAYGYMFTHLKQCVTSKGMYVIYCNPHGGDGYGEKFLEMVERWGYVDYEHLMEFVDTCIEKYPGIDADRLGVAGGSYGGYMTNWIIGHTDRFKAAVSQRGISNLITASLIIDFGDRIMKQTCGDKTPWNHEEVLWNHSPIKYVKNVKTPTLFLHSDRDYRCFMGDTFQMFTALKQLGVDTEMYLFHGDTHGLSRNGRPSNRIARANAIVDWFERYL*

>CD630_06550 Clostridioides_difficile_630_NC_009089 beta-lactamase-like protein

MNLAKGLDVLKISSNVLGEDKVMYIPAIYTEDDATLIDTGLPGQGDLIIDALNKSNTSFDRLKNIIITHHDIDHIGNINYLREKSKNNIKVYAYKSEVSYITGEETPFKLYMLEQMVDKIDDKMLSMLNVMGLGFKSSYTKVDVSLDNHEKLNLGEEIEVIHTGGHTRGHICLYLKESKVLIAGDLLQVENGELKPVDVMHSNKQELKDAIKNISNYDIETIVFSHGGLYQRNIIETLKNLIIE*

>CD630_06840 Clostridioides_difficile_630_NC_009089 ATP-dependent peptidase

MKMLNNFFKKINNPAPVFAQTKMERDNQTDDSLSTKPKTTFRDVAGLDEVKEELFEIVDFMKSPQKYQKMGAKIPKGVLFYGPPGTGKTLLASAVAGETNSSFFNVTGSEFVEKYVGVGAKRVRTLFEKARKEAPSIIFIDEIDAVGAKRHLESNNEKDQTLNQLLVEMDGFNKDSNVLIIGATNRLDLLDEALLRPGRFDRHIHIGAPNYHTRFEILKVHTDDKPIDKSVNLELLAKKTHGFNGAHLSNIANEAAIFAVRDDSECITSEHFDKALERVIAGLESKNSALVEKEKKIVAYHEAGHALVSDIVGICPIQKISIVPRGQALGYVLQLPDEDRYIYTKDELIGKIKILLAGKASEELIFNHKSTGAKDDLKKVTEIANQMVCEYGMSNLGFMTIDGNDKTFLCDKVQKEANRIVEICYKETLEMLKDNLEDLHSVSKFLFEKETMTHEELKDLIGKEAVN*

>CD630_06870 Clostridioides_difficile_630_NC_009089 50S ribosomal protein L20

MARVKKAMNARKKHKKILKLAKGFRGSRSKLYRPANTFVMKALKNAYIGRKLKKRDFRKLWIQRINAAARMNGISYSRLMNGLKLSGVEVNRKMLSEMAIQDPEGFAKLAEVAKAKLA*

>CD630_06940 Clostridioides_difficile_630_NC_009089 TetR family transcriptional regulator

MKSNDKYARERIIEVTLNLLNEVDDIEEITVRKIAERANVGVGLINYHFKTKDNLLSTAIGDVMSNIIAELYDDSVYTLRPIEDLKNLLKKLCDTGLHYEKVLPFVLNQCITNGDMQAELDIVPMLRKIFGNKKDEMSLRIIALQIILPIQISALSTESFQLYSGINIKNKYERDKFIDILIENIIGEDVDVR*

>CD630_07000 Clostridioides_difficile_630_NC_009089 phenylalanine--tRNA ligase subunit beta

MLVSLKWLRDYVDIDIDVKEFADKMTMTGTKVETIDYYGEEIENILVGKILEIKQHPNADKLVVTKVDIGDKVVQIVTGATNISEGDYIPVAVNGSKLPGGVEIKQTDFRGELSDGMMCSAAELGIDEHYIEEYKRGGIYILDHEDSYELGKDIKDVLGLKDALIDFELTSNRPDCKCMMGIAREAAATIGTKVKYPEIEVKESDEEIDFKVEIDNPDLCRRYVARMVTDVKIEPSPYWMQRRLTEAGVRPISNIVDITNFVMLELGQPLHAFDINQVETGRIVVRNAKDGEKLVTLDDVERTLDKDMLVITNGEKSLGLAGVMGGANSEITSNTKTVLFESANFKPENIRMTAKKVGIRSEASSRNEKDLDPNLAEIAANRAAQLVEMLGAGKVLKGVVDVYPNKPEPKKLVVNPQRINHLLGVDVPMEQFVGILESLEFKCNLVANDKLEIDVPSFRTDMEQEADVWEEIARIYGFENIPSVQLEGNTTAGIKTSKQKFMDALKDNSTAVGLNEILTYSFVSPKGVDKIRVPEGNAKRNFVKLLNPLGEETSVMRTTLIPNMLDVLSTNVSHKIEEVSAFECGHIFIPQDSELPKEENRMCVGMYGKDVDFFTLKGTIETILVNVGFKCYEIEPQDNNTTFHPGRCAKIVYNNKYVGTLGELHPDVIENYNLGQRVYVAEIDIDFVFDNSDRTKNYVPLPKYPSTSRDIALIVKDDVFVKQIEDIIKENGQGLVESYKLFDVYKGSQIEAGYKSIAYSITYRSKDKTLTDEDVAKVHDKILSELSEKLNANLRSN*

>CD630_07060 Clostridioides_difficile_630_NC_009089 Fe-S domain-containing protein

MIDINLEKCVGCGMCESDCLVNAIKVKDDKAKVKNILCINCGHCMAICPTDAIEMQGFDKNEVIEYNRETFELEPEKLLNFIKFRRSIRQYKDIEVEEEKIKNIVEAGRYTPTGGNRQPIRYILVKEKLKEVKELAIQGLYNLALDTDDNDPVRSIYKNTFKKMYKRYKENGNDSLFYDAPLLMVVVGDMSLGGSAYVDGGLAASNMELMAYSQGLGICYNGFFVMASNVEPKIKELLGMSENEAVITSFILGYPDVKYKRTVNRNTAKFEVR*

>CD630_07080 Clostridioides_difficile_630_NC_009089 peptidase D

MGNVLEGLKPESVFKNFEKISQIPRGSGNEKGISDFLLSFGKNLGLETIQDESLNIIIRKPATKGYENCPGVVLQGHMDMVCEKEKNVEHDFLKDPIKLRIDGDMIYATGTTLGADNGIAVAMGMAILEDNTLEHPALEVLVTVNEEDGMNGADALDPSLIKGQYILNMDSEEEGYLLVSCAGGKTCVVSLPVEYKEVKGDKQGLLVEVTGLLGGHSGMEIVLQRANANKAIARVLSVLNVDYELASVDGGTKHNAIPREAKCVIAVNKADVESAKKQINDILTAFKHEFTTSDPGMTYSVAETSVDKVLTKDCKEKVVQMSCLTPHGVQSVSLDIEGLVESSTNFAIIETKESTIEFLTSVRSSVMSIRDEIADRIRLLAQALGANYDLIAQYPAWEFKKGSKLEKICSETYEKLTGKVPTVMALHAGLECGLLLDKLPHAEAISIGPDMFDVHTPNEHVSIPSVANVWDYVIEILKSMNQY*

>CD630_07110 Clostridioides_difficile_630_NC_009089 arginyl-tRNA ligase

MQDFKVAISNCLKEKIEDLSKEEIEALIEVPPNKDMGDYAFPCFKLAKVFRKAPNMIASELAESIEPSGEITKVIQLGGYVNFFVNKSQLAETVIKKVLDEKENYGHSDFGKDKTVIVEYSSPNIAKPFHIGHIRTTVIGNALYKIYDSQGYKTIRINHLGDYGTQFGKLIVAFKKWGEKEVVESNPIPELLKLYVRFHDEAEQHPEMEDEARAWFNKLENGDEEAQELWQWFRNESLKEFNRVYKLLDIEFDSLAGESFYSDKMNRVIELLEEKNLLKESKGARIVDLEEYKMPPALITKNDGSTLYMTRDLAAAIYRKETYDFDKCIYVVGSQQNLHFQQWFKVIELMGYDWAKDLIHVGFGMVALEEGTMSTRKGRVVFLEDALNQAIDKTKEIILAKNPNAKNVDEISKQVGVGAVVFQELSNSRIKDYTFSWERTLSFDGETGPYVQYTHARCCAVLRKAEVEVTSDIDYSLLADEDSAEVLRVIESFNKNILLALKKNEPHIVTRFMLDLAQAFNKFYHDNPILVENLEIRKARLALVLATKQTLENSLKLLGMHAPERM*

>CD630_07170 Clostridioides_difficile_630_NC_009089 bifunctional carbon monoxide dehydrogenase/acetyl-CoA synthase accessory protein

MKIAITGKGGVGKTTFSSMLSRMFAEDGYRVVAVDADPDANLALALGFPKEVYESIVPISEMKKLVSDRTAASVGSFGKMFKMNPKVDDIPENFCKEYNGVRLLTLGTVDSGGTGCVCPEHVLLKRLCSHLILQNKDVVVMDMEAGIEHLGRGTAQGVDAFIVVVEPGERSLQTYRKVKKLGHDIGVNKVFVVGNKIRNKEDEEFIIQNLEDGESLGFIYYNQDVIDSDRANQSPYDSSETTKEQIKAIKDKLMSLKDK*

>CDIF1296T_00890 Clostridioides_difficile_ATCC_9689__DSM_1296_strain_DSM1296_CP011968 FolD bifunctional protein

MEGMSTKGQIIKGKPVADKISEELIKEVDLLVKEGINPKLTIVRVGARSDDLSYERGALKRCQNIGITTEVLELAEDITQEEYIDVLKRVNDDKNVNGILCFRPLPKHLNEEVIKYVIAPEKDVDCFSPINSAKVMEGDKSGFPPCTPTAVVEILKHYNVDLKGSKVTVLGRSMVVGKPVSMLLLSEHATVTICHSKTKNLSGVAAEADVLIAAIGRAKMVDESFVKDGAVVIDVGINVDEEGNLCGDVDTNAVLDKVSMITPVPAGVGSVTTSILAKHVVKACKLQNNK*

>CD630_07230 Clostridioides_difficile_630_NC_009089 bifunctional carbon monoxide dehydrogenase/acetyl-CoA synthase dihydrolipoyl dehydrogenase subunit

MKIVVVGGGPGGYVAAIKASMLGADVTVVEKRRVGGTCLNAGCIPTKALLASSGVLNTVKEAKDFGIEIDGTVKPNFTAIMERKNKVVNQLISGIEFLFEKRGVNLVNGFGKLIDKNTIEVTKDDGTVETIKADKIILANGSVPVVPRMFPYDGKVVITSDEVLGLEEIPESMLIVGGGVIGCEIGQFFRALGTEVTIVEMVDQILLNEDKDVAKQLLRQFKKDKIKVITGIGVQTCEVVDGKAVATLSNGKVIEAQYALVCVGRRPNLDNSGVEDIGIEMERGKVVVNEHLETNVEGIYAIGDIIDTPFLAHVASKEGIVAVENALGKTKVVDYRAIPRCVYTEPEVAGVGKTEKQLEAEGVEYNVGQFDFRGLGKAQAIGHFQGFVKVIADKETDKIIGAAVVGPHATDLLTELSLAVHLGLTVEQVGDAIHPHPSLSEGLMEALHDVHGECVHSVPKL*

>CD630_07290 Clostridioides_difficile_630_NC_009089 glycine cleavage system protein H

MKLLPELKYSKDHEWVKVIDGDVVYIGITDYAQDQLGEILFVETPEVEDTVTKGVDFGVVESSKVASDLISPVNGEVLEVNEKLEDEPECINEDPYENWILKVKLADVAELDTLLSDKEYEAGLE*

>CD630_07500 Clostridioides_difficile_630_NC_009089 amino acid family ABC transporter substrate-binding protein

MKVFKKLLSLGLVLGLTLSLVGCSGGGEKTKLEQIKDNGKLVVGTSAEFPPFEFHKVVDGKDSIKGFDIMLAEEFAKELGVKVEIKDMSFDGLIGALNADQVDIVLAGMSPTPEREKSVDFSELYYLSRNAVIVKDADIDKVKTEDDLKKLRVGVQAGSIQEEYVVNTLKMTTTKSLKAIPDLITELKNGNIDAVVTNEAVSLINVKKYDGIKMANTEVGKDVTEGMAAAIKKSDNNKDFIELLNKKIKELQDGKKIEEFLNEASTEAASN*

>CD630_07520 Clostridioides_difficile_630_NC_009089 amino acid family ABC transporter ATP-binding protein

MITIKNLSKSFGDLNVLKNIDLEIAKGEIMVIVGPSGSGKSTFLRCMNLLEIPTGGEIIFEGKNLVDKKTNIDEVRQNIGMVFQNFNLFPHKTILDNITLAPIKLKKMTKEEAEKKAEILLSRVGLLDKKDSYPSQLSGGQKQRIAIARALAMEPDMMLFDEPTSALDPEMVNEVLDVIKELAKEGMTMAIVTHEMGFAKEVADRVIFIDGGSILEDNTPEEVFGNPKHERTKAFLAKVL*

>CD630_07530 Clostridioides_difficile_630_NC_009089 cysteine desulfurase

LEIYLDNSATTKPYQEVIDKMVYALNTEYGNPSSVHRKGVEVEKAIKEVRQDIAKSLGAKEKEIYFTSGGTECNNTIIRGITSLNKKRKNHIISTNIEHPSVLNTLKDLEEDGFEVTYLEVGKDGKINIEDLKNAIKSTTCLVSMMHVNNEIGTIQPIGEVGKYLKGLKEKIYFHVDAIQSYGKINFRPSKYNIDFMSVSAHKFHGPKGIGFMYIKENNRLKPMLTGGGQEIGIRSGTENVPGIYGLGEAVRILNKDLDAVISKVDNLKNILKNEIIDNIEDIKINSPEDGVCHILNVSFRGTKGEVLLHYLEQKGIYVSTGSACSSKKKGSYVLNAIGLTNEEINGTIRFSLSDMNTEEEMLEAVKVLKESICDLRSIMKRK*

>CD630_07560 Clostridioides_difficile_630_NC_009089 nitroreductase

MNNNFQDNQTINLIQSRRSIRKFTTEQISDEQVNTLLHCAFAAPSGCNKQPWHITVVQDQKLLKEISDDTLSRIHEVSNVEINKNFKLFYGAPTVLFISYDESSSWAPYDIGILTGNITTAAQALGLGSCIIGMVRGLFTPVEQGDIEGLVSVLDKEDVKESESIKMKFDTNKKYRELLDIPEGYSVPFGIAVGIPDGNLPNAREVVYKVSRV*

>CD630_07610 Clostridioides_difficile_630_NC_009089 ATP-dependent RNA helicase

MNITKFEDLPISEGIKKAIAEMGFEEPSPIQAQSIPAILSGKDVIGQAQTGTGKTAAFSIPILETIDPNNRSLQAVVLCPTRELAIQVSTEIRKLAKYSHGIKTLPIYGGQPIDRQIKSLKSGVQVVIGTPGRTIDHINRKTLKMDNVKMIILDEADEMLDMGFREDIEMILSKIPEERQTTFFSATMPRGILELTKRYQKDPEHIKVVRKELTVSNTKQYYIETRSSNKLEVLCRLVDVYDPKLSVVFCNTKRKADELVGDLQARGYFADALHGDLKQTQRDIVMDKFRNGTIDILVATDVAARGIDVDDVECVFNYDLPQDEEYYVHRIGRTGRAGREGMSFTFVFGKEMRKMKDIERYTKSKLIKHNIPTITDVEEKKVGTFFAQVKQTIEEGHLTKQLQWLEGFCNDEDYAMVDIAAALVKLSLGEEMKEEIIEEKPRRERGDRKGGTGAKDGMIRLFINIGRNQRVQAKDIVGAIAGEVGIPGKVVGTIDIYDKYTFVEIPKKDAKTVIEKMKDIKIKGNKINIEKANKKKK*

>CD630_07850 Clostridioides_difficile_630_NC_009089 ABC transporter ATP-binding protein

MLVVENVSHGFGARTILENVSFRLRKGEHIALVGANGEGKSSFLNIITKKLMPDAGNIKWSSRATVGYLDQHTVLSKGKTIREVLREAFKHMFDLEQEMIAMYDKMGEASDDEMSKLLEETAEIQTILENSGFYMIDAKIQEVANGLGLGEIGLDKDVTDLSGGQRTKVLLTKLLLENPTILILDEPTNYLDEEHITWLTKYLQEYENSFVLVSHDIEFINNTCNVIYHMENGELNRYKGNYDEFVRLNDIKKRQEEQAYDKQVEERKRLEDFVARNKARVATRGMANSRQKQLDKMEILERPKEKIKPTFAFKDARAASKIIFETENLVLGYDEALTKPLNFHLERGKKIALKGMNGIGKSTLLKTLLGIIKPFEGNVKLGDYLEVGYFEQESSRENSNTPMDEVWSEFPGLTNFEVRQALAKCGLTNEHITSQMRVLSGGEAAKVRLCKVMLKNINFLVLDEPTNHLDVEAKDELKKAIKEFKGTVLLVCHEPEFYSEIVDDVWNIEDFTTKIV*

>CD630_07950 Clostridioides_difficile_630_NC_009089 DNA-binding regulatory protein

MGRIGNIINRKGKQDAQRAKIFTKHARAIAVAAKEGGADPEYNAALKTAIEKAKADNMPNDNIDRAIAKGAGAGAGEDYETIVYEGYGPGGVAVIVETLTDNKNRTAGNVRYYFDKNGGNLGTSGCVSFMFDKKGQILVGLGDGVSEEELMDVALEAGAEDFITEEDGYEIITTPEDFSSVRDELKAKGYEFISADVKMIPQTTTVLTEESHLKMMNKLVDMLEEDDDVQDIYHNWEVE*

>CDIF1296T_00969 Clostridioides_difficile_ATCC_9689__DSM_1296_strain_DSM1296_CP011968 3-hydroxybutyryl-CoA dehydratase

MESVMENLNNLKVELKDKVCVITINRPKALNALNSDTLRELSQVIDVVSENEAILGVIITGEGKVFVAGADIRQMQNYKSEEGRKYAGYAQGIFDKIEALEKTVIAAVNGYALGGGCELAMSCDIRIASEKAIFGQPEVNLGVIPCFGGTQRLSRLVGTGIAKELIFTGRQVNAEEAKSIGLINKVVPSDLLLEESMKMMNQIVEKAPIAIRYAKVVINKGIDMDLKNALELEKDIAGLTFATRDKQEGMNAFIEKRKPVFENK*

>CD630_08030 Clostridioides_difficile_630_NC_009089 acyl-CoA dehydrogenase

MDFRLTEAQLMLQRVAKEFAENEIAPIAAETDKTGIFPRELFSKMAKIGFNGIGTPVEYSGSGGADIEKVIVVTEIAKKCAASAAILSIHTIYAQAILKFGTEEQKKKYLPMMAEGGCVGAFALTEPNAGSDAARAATTAIIDEETDEYVLNGTKCFISGGGQAESLIIFALTDPSKGIKGMSAIIVDKGTPGFSIGKIEEKMGIHGSETAELIFDNCRVPKSNLLGKEGKGFNIAMTCLDGARIGVGAQAVGIAEGALEESIKYSKERVQFGKPISALQGIQWYIADMATMVESAKLLVYYAADLKARGEKHTKEAAMAKYNASRTAREVTNLALQIHGGYGYMKDYPLERMYRDAKITEIYEGTSEIHKVVISRAVLG*

>CD630_08050 Clostridioides_difficile_630_NC_009089 electron transfer flavoprotein subunit alpha

MMRAKVNQGINLNDYNGVWVIGEQREGKINPVTIELIGEGRKLADQLGKELAVVIAGYEVEKEVKELLHYSVDKVYYINDPLLKDFTTDGYAISIANLIERKKPEVVLVGATSIGRDIAPRIAGKVGTGLTADCTKLEIDSTDNKLLQTRPAFGGNLMATIVCPKNRPQMSTVRPGVMAKAVRNESETGILEVVTPELTEKMIRTRLVEILPQEKKSVNLTDARIIVSGGRGLKRAEGFELIKELADKLGAEIGASRAAVDSGWIEHSHQVGQTGTTVRPELYIACGISGAIQHLAGMSDSKYIVAINKDAKAPIFSICDYGIVGDLYEIIPEMIESLNR*

>CD630_08060 Clostridioides_difficile_630_NC_009089 sigma-54 dependent transcriptional regulator

MKEQWYKDIFARVLSMTDDGFIVVNTSGVIIDINDKYCDFLGKERKDIIGQNIQSIIPNTKMLDVMKNKYCEEGAIHHYSGGNTKEKSVIVSRSYVENDNGEVVAGVAQVKFRLQSFDVAKKLMSEYMELQYYKEQFKDNCGFDKLIGENRDFIELKKTGVKASKTNFPVLLTGETGTGKEVFARAIHNNSSRSDKPMVSINCAAIPEELLESELFGYDEGAFTGAKKGGKKGKFLVANNGTIFLDEIGDMPLTMQAKLLRVLQESEIEPVGGLKTIKIDVRVISATRKNLSKMVEEGLFREDLYYRLNVINIHMMELKDRQDDILLLANYILNKLNVEYKELKVLSDKVKNCFINYTWPGNIRELQNVIKSAYAVSDDMVIMMCDLPSKMDNISRVAQCNVDSNCSIHEMVENYEKSLIIDVLRKYNWKCSKAAEVMGIHKSLLYKKIKKYEIELNN*

>CD630_08180 Clostridioides_difficile_630_NC_009089 6-phospho-beta-glucosidase

MNTGFPKDFLWGASSSAFQVEGAWDKDNKGKTVADYNSFKKSHLQADTKVASDFYHNYEEDIELMKELGMKTYRFSISWARIIPDGEGEINQKGLDFYNKIIDKLIECDIEPFVTLYHFDLPFKLVEKYNGWESRETVYAFERFAKICFKHFGDRVKYWQPHNEQNLIVRVEERINIYDETDSWKIDKIRAQMDYNLCLAHALAVNACHEMIKESKIGAAVSSSVTYPLTSKPEDVYAARMNDNFKVYYMLDMHHYGEYPGYYMKYLEKRNIVPHMEDGDKEILKKAKMDFIAVNYYRTNCAEALPEDSQHPFGLREGTVDFSMYGLFKMSMNPNLEASEYGAAIDPSGLRVALNEYWQRYHLPVIITENGLGAKDILEDGKIHDDYRIDYLRSHINACKLAIEDGVEMIGYCPWSFTDLLSSSQGFNKRYGLVYINRTDHEVLDLKRIKKDSFYWYKEVIENNGIVK*

>CD630_08310 Clostridioides_difficile_630_NC_009089 ATP-dependent RNA helicase

MDFKSLGISENTINILKKSGITTPTPIQKESIKLIKEGKDVIAEAQTGTGKTLAFLLPIFENISLDINDIQVLILSPTRELAIQITEEAMKLKESKDVSILAAYGGKDIGSQIKKLKGNIHMIIATPGRLLDHLNRKTIDLSKLKTFVLDEADQMLLMGFKNEVEAILKETSNKKQTLCFSATINSQVKKLAYRYTKNPVVVSIQKEEITLNNIKQEVVETTDRKKLDALCKVLDEDNPFMAIIFCRTKRRVDNLEEALAIRGYNCQKLHSDIAQSKRERIMKSFRNLDIQYLIATDVASRGLDISGVSHIYNYDLPETPEDYIHRIGRTGRAGEEGYTCAFIDPKNERMLSEIETAIESKISRRIIEL*

>CD630_08330 Clostridioides_difficile_630_NC_009089 aconitate hydratase

MGDNIVYKIIKKHIVDGEAVAGSSIGIKIDQTLTQDSTGTMTYLQLEAMGIDKVKTKRSVAFVDHNMLQQGFENADDHKYIQTVADKYGVYFSKPGNGICHQVFLERFSTPGDTLLGSDSHTPTAGGVGMMAIGAGGLDVALAMAGGAYYIKAPKVCKVNLVGKLNNMVSSKDIILEVLRKQTVKGGVGKVYEYGGEGVKSLSVPQRATITNMGAELGATTSIFPSDEKTLEFFKSQGREDAWVELKPDADAVYDEEITINLDELKPLAAKPHSPDNVDEVENIGKIKIDQVAIGSCTNSSYEDLMKVAQILKGNKVHKDVSLVIAPGSRQVMEMIARNGALADIISAGARILENSCGPCIGMGQSPGTDSVSLRTFNRNFYGRSGTLSAQVYLVSPEVAAVSAIKGVLTDPREFDIKFTNLDVNEFLIDDSMIIKPADVGSDVEVVRGPNIKPFPLNTELSQSIGGKVILKTEDNITTDHIMPSNAKLLPFRSNIPYLANYCFNTVDTEFPQRAKDNNGGFIVGGDNYGQGSSREHAALAPLYLGVKGVIVKSFARIHKANLINSGIIPMEFCDEKDYENISLLDNLEIPNILDNLGSGILEVKNTTKGTSFKVKVELSAKEVDVLKAGGKLNYTKNQAN*

>CD630_08340 Clostridioides_difficile_630_NC_009089 Isocitrate dehydrogenase

MYKVTLIPGDGIGPEVAKAMKKVVEATGVEIEWEEVNAGEAVIEEYGTPLPEYIIDSIKKNKIAIKGPITTPVGKGFRSVNVTLRQALDLYVNLRPIKSFKGIKSRYEDVDLVVVRENTEDLYAGIEHKIGDYAAESIKIITRSASERIVDFACNYVKDNKRKKVTAIHKANIMKMSDGLFLDVFREVASKHGVEYDDLIVDAAAMNLVLNPENYDVMVMPNLYGDILSDLGAGLVGGLGIIPSANIGKDCAIFEAVHGSAPQIAGQNKANPTALIQSSVMMLRYLGEYENAQKIETALEKVFLEGSKLTVDLGGSASTTEFADEVCKYIV*

>CD630_08410 Clostridioides_difficile_630_NC_009089 5-aminoimidazole-4-carboxamide ribonucleotide transformylase

MARELELKYGCNPNQKPSKIYMKNGELPIEVLNGKPGYINFLDAFNSWQLVKELKEATGLPAATSFKHVSPAGAAVGVPLSDTLKQIYFVDDLELSPLACAYAMARGADRMSSYGDFIALSDVCDKETATIIAREVSDGIIAPGYTEEALEILKGKRKGNYNIVKIDENYTPEPIETKDVYGITFEQGRNEILINEDLLKDIPTDNKIFTDSAKRDLIIALITLKYTQSNSVCYAKDGQVIGVGAGQQSRIHCTRLAGNKADTWYLRQHPKVLNLKFKKDIGRPDRDNTIDVYLSDDYMDVLADGIWQNFFEEKPEPLTGEGKRAWLKTLTGVALGSDAFFPFGDNIERAKRSGVSFIAQPGGSIRDDNVILTCNKYNIVMAFTKNRLFHH*

>CD630_08560 Clostridioides_difficile_630_NC_009089 ABC transporter ATP-binding protein

LEHLLEVNNLSVSFKVEEGEVQAVRNVSFNLKKGETLAIVGESGCGKSVLCKSLMRILPYNGYIKNGEVLLKSSDLVKKSEKEMEDIRGKNISMIFQDPMTSLNPTISIGKQIAEAVIIHQGISKSEAKKRAIELIELVGIDNPEKRFKQFPHHFSGGMRQRIVIAIALACNPDVLIADEPTTALDVTIQAQIIDLIKDLQHKIGLSIIFITHDLGVVATIADRIAVMYAGKIVEIGTVEDIFYDPRHPYTWGLLGSLPTLDSQDDYLYNIPGMPPNLLNPPKGDAFAIRNKNALKIDYEKEPPMFKINDTHSAATWLLHPDAPEVDVPVRVNCGRVISNE*

>CD630_08710 Clostridioides_difficile_630_NC_009089 molybdenum-specific ABC transporter ATP-binding protein

LSLYVDIEKDLSSFKLKVEIKQEKGTLGFLGESGSGKSMTLKCIAGLEKPTRGKIVLNDRVLFDSEKKINLSTQDRKVGFLFQNYALFPHMTVSQNIELGLLKLSKSEKKEIVARYLDILKLNGFEGRYPWQLSGGQQQRVALARALATSPDILLLDEPFSALDHHLRSNMEKELMNMLKDYKGDILFVTHDIEEAYRVCDDIIVYNKGEGLPKRPKKELFESPKYLIEAKITGCKNISKLNRLDKNTIYATDWGCELTLNREIGDNIEYVGIREHHIKVLDSNEDLNEKLCFELINIVENPFTYTIYVRKTDLSNECVPIQIELEKSKMRFKKGDRIYLDFPQEYLFCFRYNYNKKE*

>CD630_08720 Clostridioides_difficile_630_NC_009089 maltose O-acetyltransferase

MTEKEKMLSGKGYYANDELLVKEREYCKKLTRLFNNTLEDEYEKREDILRQLFGSVGKQINVEQNIRCDYGYNIHVGENFFANYDCIFLDVCKIEIGDNVMLAPNVQIYTAYHPIDAQLRNSGIEYGSPVKIGDNVWIGGGVIITPGITIGDNVVIGAGSVVTKDIPPNTVAVGNPCRVIKKIEE*

>CD630_08820 Clostridioides_difficile_630_NC_009089 glucose-1-phosphate adenylyltransferase

MKKEMLAMILAGGQGSRLGVFTKRIAKPAVSFGGKYRIIDFVLSNCSNSGIDTVGVLTQYRPLILNSHIGMGSHWDLDRINGGVYVLQPFMNEKEGNWYNGTAHAIYQNMDFVDTYNPEYVLILSGDHIYKMDYSKMLKFHKEKGSKATIAVIEVPWDEASRFGIMNTNEDSSIYEFEEKPSEPKSNLASMGVYIFDWKMLRNYFKEAEKNPEINYDDFGKNLIPKMLEDNVGMYAYPFKGYWRDVGTIQSLWDANMDIIKSPETLDLADPKWKIYTNTMAMPPQYIGKNANVHRSMIADGCRILGEVGNSVLSHGVVVGKGSKVIDSVIMPNVVIGENVTIEKAMIGECATINDNVQIKNVNNEINVVSEYENIEPRCVLIEGGL*

>CD630_08840 Clostridioides_difficile_630_NC_009089 glycogen synthase

MKVFYVTAECWPFAKTGGLGDVSYALPKELKKEGVDVRVIMPKYSTIPSYLKDQLKEIAVFSVRVGWRNQYCGLLEMELDGVKFYFIDNEFYFRREDERKSIYGYGDDAERYTFFTDAVLEAISRIDFYPDVIHINDWHTGMLPLILKERYATLEGYKNIKTMYTIHNLQYQGVFDKHVLYDILDLPQKYFDNGDIEYYGSINFMKAGINFADKIITVSPTYANEIQTSFYGEQLDGLLRKESGKLKGILNGIDYDLNDPAKDKDIFVHYDVDSINKKVENKLRLQDILGLKKDSSIPLIGIVSRLVSQKGFDLIAYMMPELMREDLQIVVLGTGEHQYQSMFNYYDSNFSDKVSARITFNASLAQQIYAASDMFLMPSLFEPCGIGQMLAMRYGSLPIVRETGGLRDTVTPYNKFTGEGNGFSFKNYNAHEMFFCLKNAIKVFKDKEKWIKLVENAMKTDNSWKKSAKEYIETYRDICD*

>CD630_08920 Clostridioides_difficile_630_NC_009089 cold shock protein

MKNGIVKWFNNEKGFGFISVEGEDDVFVHFSAIQNDGYKTLEEGEKVSFDITQGNRGPQAENVNRI*

>CD630_09000 Clostridioides_difficile_630_NC_009089 glycine betaine/carnitine/choline ABC transporter ATP-binding protein

MIEIRNVTKKIGNNVILDDISLVVETGTLVVLIGSSGCGKTTTLKLINKLIKPTSGEIYINGKPISQENEIELRRKIGYVIQNTGLFPHLTIKENIELIPRLKKEKSVEEIEKRTLQLLEMVGLDSDEFLNKYPSELSGGQQQRIGVARAIATDAEIILMDEPFSALDPITRTSLQEQLFSLQDELKKTIIFVTHDMDEALKIADKICIMKDGRIAQYDTPENILRKPANDFVKDFIGEDRVWDNPEYIKARDIMIKNPIAVNSTRTVTQGIEIMRTSKVDSLLIIDRAKTLKGIVTVKDMKDIDDKSILLADIMSSEPLHVNEGDNLVEILNVMNRNSVGYIPVISDENKLVGLITRSSLLSVLSEQFLEMEVSVLG*

>CD630_09860 Clostridioides_difficile_630_NC_009089 30S ribosomal protein S1

MFIMENDLTMQELLDQQEQVFSKVKVGELTTGKITAVRNDEVQLGLDYGFDGIIPISELNIEKNQYIEDIYHIGDEITAVITKVSQKDGTITLSKLQLDKRNDFAELQKAYDEHRIITVNVEKNIDKGVFANYNTYTFFIPISQLDTKFITDTSKFVGLNLEVYIKELDVRKNRLVASHRDVLQERINKEREERRAQIKAEKEAERARIKQEREEEKARIKAAKEDLFNSLEVGQKRDGKVTKIMPYGAFVDIGGIEGLAHINNLAWTRVESVEDVVSEGQEVEVYVLDVNKETKKIALALKDINNDPWDLIAKEVQIDDVVNAKVLRIIEKGAFVQIKEGVDAYLPISELSDTRVAKVTNVVNIGDEVKVKILDFKPKTKRMLVSIKEATREPEEDITEYLEVEESLGSIGELFKDKFKDLEV*

>CD630_09890 Clostridioides_difficile_630_NC_009089 2-isopropylmalate synthase

MKCGKYKKYDKMQIVNRKWPDNEIFKAPIWCSVDLRDGNQSLPTPMSVNEKVRMFKMLIDTGFKEIEVGFPSASNTEYTFLRKLIDENMIPDDVTIQVLTQSRAHLIEKTFESIRGCKKAIIHLYNSTSVLQRDVVFNMSKQEIIDIAVEGAKLFNEEVKKYPETEFTFEYSPESFTGTEMDYALEICEAVIDVWKPTPQKKVIINLPSTVEMATPNVYADQIEWFCKNISCRDSIILSLHTHNDRGTCTAASELGLLAGADRLEGTLFGNGERTGNMDIVNVGLNLYTQGIDPELDFSNIDKIIGIYEDCTKLMVHDRHPYAGNLVHCAFSGSHQDAIRKGMIAMKNRDNDYWEVPYLPIDPHDIGREYKEIIRINSQSGKGGAVYIMETDYGFMIPKNMHSDFGNVVKMESDRIGEELSSEAIFNLFKKEYIEVESPYKVKKYKIKSMDELNYENDDSNDTNMIEMTARISYMGNEQRIVGIGNGPVDSFNNALKQCGMKDYKFRYYWEHALEEGSHSRGVAYVGIEHNNEVYFGVSISENINTAAINALMNAINKSYIEEEIKNGDDYDAENISQTC*

>CD630_09910 Clostridioides_difficile_630_NC_009089 3-isopropylmalate dehydratase small subunit

MIANGSVFKFGDNIDTDVIIPARYLNIADYKELATHCMEDIDDKFISKVKKGDIIVATKNFGCGSSREHAPIVIKESGVSCVIASTFARIFFRNSINIGLPILECEEAANNIDEGDNIEVDFSTGVIKNITKGKEYKAEPFPEFMQNIILNEGLINSIKANRG*

>CD630_09920 Clostridioides_difficile_630_NC_009089 3-isopropylmalate dehydrogenase

MNCNIAVIKGDGVGPEIIDEGIKVLNKICCKFNHRFDCEYVLAGGCAIDETGEPLPNKTVEICRKNEAVLLGAVGGPKWDKCKGDKRPESGLLKLRESLGLFANLRPATMYESIKEASPLRTDIVEKGIDFVVVRELTGGIYFGERGRKIIDGIENAYDVEIYNENEIRRIGKRAFEIARNRNKKLISVDKANVLESSRLWRSIMEDLAKEFEDVELSHMYVDNAAMQVVKDPSQFDVIVTNNIFGDIISDEASMITGSIGMLPSASLREDSFGMYEPIHGSAPDIAGKDIVNPIATILSVSMMLRHSFNLEEEAKCIEDAVQSVLNKGYRTIDIYNGVGNVVGTRAMGELIVNEI*

>CD630_09940 Clostridioides_difficile_630_NC_009089 serine-pyruvate aminotransferase

MSKKLFIPGPIDVKEEVLQKMATPMIGHRGKDASMLQKSISEKMQKLFYTNNTILLSTSSGTGLMEGSIRSCTSKKAAVFSCGSFGDRWYKMAVANNVPADIFKVELGEATTPEMVDKVLSTGEYDLITVTHNETSTGIRNPIEEIGEVVKKYEDVIYCVDTVSSAGGIKVEVDKIGIDICITSVQKALGLPPGMSICTFSQKAIDRAKQVPFRGVYLDLLAMYEYLIKKNYQYPSTPSLSHMFALDFQLDNILDEGLDNRFNRHEDMANLVRNWAKKHFQIFTNENHLSNTLTVIENTQGISVSNLNSKLQERGFQIANGYGDLKEKTFRISHMGDYTVEDVQELLDNIDDILGFNK*

>CD630_09950 Clostridioides_difficile_630_NC_009089 D-3-phosphoglycerate dehydrogenase

MYNILVTDGIEKEAARKLRELDFNVIEQFYEKDVLGDKLKDVDVLVVRSATKVTKDVIDKALEGKKLKLIVRGGVGLDNIDVKYAQANGIKVMNTPNASSISVAELTIGQLFVLARFINTANVTMRDGKWEKKKYKGTEINGKTLGLIGFGRIAKEVAKRAELLGMNVIYTDIMGEAQGFNNYKFCDMEEVLENADFLSLHIPFDKNKGAVITEKEINKMKKGAYLINCARGGLVDEKDLLKALDEGKLSAAAIDVYEQEPTLNLDLVNHPRVSPTPHIGASTVEAQERIGDEIVNVIQDFFLDFNNLIGVAL*

>CD630_10010 Clostridioides_difficile_630_NC_009089 nitrate/sulfonate/taurine ATP-binding protein

MEKIKLSIENINKRYDSRIIFRDFNIDFYVNEVNCILGKSGCGKTTLLNIISGIIKNDTNNLNIKENLNRVGNKLEASYIFQDDRLIDWLTVEENIKIVVNKYYNKTQLNKICDEYLELVGISDYKKFYPQMLSGGIRQRVNIARAFIYPSKNIIMDEPFKSIDAKNTQLIMDNFRNILRKEKRTVLFVTHNIEEALFLADRIFILGDSPIRIKKILKNSKELEKNEVLKLI*

>CD630_10050 Clostridioides_difficile_630_NC_009089 NAD-dependent malic enzyme

LMSKDYAKLALEMHEINKGKVSVESKVEIKTKDDLSTAYTPGVAEPCLKIHENQDDVYRYTSKGNLVAVVSDGSAVLGLGNIGAEASIPVMEGKAILFKQFADVDAFPICLKTNDVDEIVKSVELMEPVFGGINLEDISSPRCFEIEERLKKSLSIPVFHDDQHGTAIIVAAAIINSIKLIENKKIEDLEIVINGAGAAGIAIAKILLNMNVKNIILCDRSGALEASIENLNYVQKEMLKVTNIRNEKGPLKDIIKGKDVFIGVSGPGAVTKEMVESMSEKPIILAMANPTPEIMPEEAKLGGAFIMGTGRSDFPNQVNNVLAFPGIFRGALDVKAKEINEEMKIAAAYAIANTISDEEICPEYILPDVFNKNVVKNVAKAVKEAAIKTKVNRI*

>CD630_10170 Clostridioides_difficile_630_NC_009089 multidrug family ABC transporter ATP-binding protein/permease

MKKLIHFLKPYRVLIVVVLIFTFLQTLGTLYIPTLTANIVNNGVVKGDIDYIVKTGLMMMIVAGITALSAVLVCKVSANLSSGFCRDIREAVFIKSQDLSINDFNNIGTASMITRSTSDITLIGQSVFMFIQLVLPAPIITVSGLFLAYSIDKAMTIIIVVVMFLFMLSAFLVGKKLIKLFKMMQIKMDNMNRVLREVVTGVRVIRAFNRSHFEKKRFDRTAIDYSETAISINKIFAVLMPIVMLIMNLGIVSIIWFGGMRVSNGNMEIGHIMALVEYCILILFYLIMGVMVFMYIPRAGACADRVNQILDIEPEIVDGNGHKDTVSERGHLVFKNVTFSYAQSEEPVLNNITFEAKSGEVTAIIGSTGSGKSTIANIIPRFFEIQSGEISINGQDIKKIPQKELRDKIGFVPQKAFLFSGTIEENIRYGKEDASIEEVKHAASIAQADEFISDMEDKYDSFVAQGGNNLSGGQKQRISIARALVRKPEVYVFDDSFSALDFKTDKRLRKALKNEIKDSSAIIIAQRISTIMDANQIIVLNDGKIVGIGKHKDLLENCEVYKQIADSQLSKEELA*

>CD630_10230 Clostridioides_difficile_630_NC_009089 transcriptional regulator

MDIGEKIKRLRTEKQLTQEELANRCELSKGFISQLENNLTSPSIATLIDILEILGTNLREFFNEIDDERISFTKEDMFETEDEDLKYKLKWLIPNSQKNEMEPIIITLYPGGQYKEEKPHEGEEFGYVLAGSIYVHIGEKKNKVKKGESFYFRPKANHYISNEGKTTAKVIWVSTPPSF*

>PCZ31_RS05070 Peptoclostridium_difficile_strain_Z31_NZ_CP013196 ABC transporter ATP-binding protein

VGIKKCIRGEDVFSIRNITKKLGKFKLNNINLELKEGDIVGIIGPNGSGKTTLIKIIMGIIDADEGEIELCNETIENSPISFKNNIGFVYDSLQFYPHLKVKEFRKIVSLFYKNFDRERFDEYLNKFDIEENMHIENLSKGQSEKLMLSSALSHNAKLLILDEPTAGIDPIVRTEIMQYLQDFVKNGSSSVIISTHNTDNLIKIADYLVFINRGNQIFTVKKELIEQEYKIIRANKAELEAIKESIVGVKEYKYYNEALVKVGDSLKVKSLLIEIDKHKVKNPTIEELMYYYVNEVR*

>CD630_10540 Clostridioides_difficile_630_NC_009089 butyryl-CoA dehydrogenase

MDLNSKKYQMLKELYVSFAENEVKPLATELDEEERFPYETVEKMAKAGMMGIPYPKEYGGEGGDTVGYIMAVEELSRVCGTTGVILSAHTSLGSWPIYQYGNEEQKQKFLRPLASGEKLGAFGLTEPNAGTDASGQQTTAVLDGDEYILNGSKIFITNAIAGDIYVVMAMTDKSKGNKGISAFIVEKGTPGFSFGVKEKKMGIRGSATSELIFEDCRIPKENLLGKEGQGFKIAMSTLDGGRIGIAAQALGLAQGALDETVKYVKERVQFGRPLSKFQNTQFQLADMEVKVQAARHLVYQAAINKDLGKPYGVEAAMAKLFAAETAMEVTTKAVQLHGGYGYTRDYPVERMMRDAKITEIYEGTSEVQRMVISGKLLK*

>CD630_10550 Clostridioides_difficile_630_NC_009089 electron transfer flavoprotein subunit beta

MNIVVCIKQVPDTTEVKLDPNTGTLIRDGVPSIINPDDKAGLEEAIKLKEEMGAHVTVITMGPPQADMALKEALAMGADRGILLTDRAFAGADTWATSSALAGALKNIDFDIIIAGRQAIDGDTAQVGPQIAEHLNLPSITYAEEIKTEGEYVLVKRQFEDCCHDLKVKMPCLITTLKDMNTPRYMKVGRIYDAFENDVVETWTVKDIEVDPSNLGLKGSPTSVFKSFTKSVKPAGTIYNEDAKTSAGIIIDKLKEKYII*

>CD630_10560 Clostridioides_difficile_630_NC_009089 electron transfer flavoprotein subunit alpha

MGNVLVVIEQRENVIQTVSLELLGKATEIAKDYDTKVSALLLGSKVEGLIDTLAHYGADEVIVVDDEALAVYTTEPYTKAAYEAIKAADPIVVLFGATSIGRDLAPRVSARIHTGLTADCTGLAVAEDTKLLLMTRPAFGGNIMATIVCKDFRPQMSTVRPGVMKKNEPDETKEAVINRFKVEFNDADKLVQVVQVIKEAKKQVKIEDAKILVSAGRGMGGKENLDILYELAEIIGGEVSGSRATIDAGWLDKARQVGQTGKTVRPDLYIACGISGAIQHIAGMEDAEFIVAINKNPEAPIFKYADVGIVGDVHKVLPELISQLSVAKEKGEVLAN*

>CD630_10570 Clostridioides_difficile_630_NC_009089 3-hydroxybutyryl-CoA dehydratase

MSTSDVKVYENVAVEVDGNICTVKMNRPKALNAINSKTLEELYEVFVDINNDETIDVVILTGEGKAFVAGADIAYMKDLDAVAAKDFSILGAKAFGEIENSKKVVIAAVNGFALGGGCELAMACDIRIASAKAKFGQPEVTLGITPGYGGTQRLTRLVGMAKAKELIFTGQVIKADEAEKIGLINRVVEPDILIEEVEKLAKIIAKNAQLAVRYSKEAIQLGAQTDINTGIDIESNLFGLCFSTKDQKEGMSAFVEKREANFIKG*

>CD630_10580 Clostridioides_difficile_630_NC_009089 3-hydroxybutyryl-CoA dehydrogenase

MKLAVIGSGTMGSGIVQTFASCGHDVCLKSRTQGAIDKCLALLDKNLTKLVTKGKMDEATKAEILSHVSSTTNYEDLKDMDLIIEASVEDMNIKKDVFKLLDELCKEDTILATNTSSLSITEIASSTKRPDKVIGMHFFNPVPMMKLVEVISGQLTSKVTFDTVFELSKSINKVPVDVSESPGFVVNRILIPMINEAVGIYADGVASKEEIDEAMKLGANHPMGPLALGDLIGLDVVLAIMNVLYTEFGDTKYRPHPLLAKMVRANQLGRKTKIGFYDYNK*

>CD630_10840 Clostridioides_difficile_630_NC_009089 multidrug family ABC transporter ATP-binding protein/permease

MIKQFVKYYKPYKKIFTLDLIAAFLFSLCDLVYPMITRNIMDDVVPNKNLRMLVVFAVALILIFIAKAGLNYFMQYWGHVIGVDMQADMRNEVFTHLQRLPNTYFDNNKSGVTMSRIVNDLMDITELAHHGPEDLFISIVMLVGSFFILIDINIPLTLIIFAILPFIIWFAIAKKDKMNIAFMKSRVTIGDVNATLENSIAGMKVTKSFCTEKEELNKFVRSNKLFRRARQDSYKVMAEYYSGMNLYMDILEWVVVIAGGYFTYIGKITLGDFAAYILYVKMFIQPMKKLINFTEQYQNGMTGFKRFIEIMEQDHQKEAKNPIELENVKGDIEIENISFTYEDKTQVLDNLSLSIKAGKTIALVGPSGGGKTTLCNLLPRFYEFDKGDIKIDGKSIKDVSLKSLRKNIGIVQQDVFLFTGTIRDNILCGNPNATDEEMIAAAKKARIHDFVETLPDGYDTYIGERGVKLSGGQKQRISISRIFLKNPPIIILDEATSALDNVTEREIQESLEELSKDRTNLVVAHRLTTIKNADEIIVLTDKGIEERGTHEELVNKNGVYSRLHNN*

>CD630_11290 Clostridioides_difficile_630_NC_009089 dephospho-CoA kinase

MLILGLTGGIGCGKSSLSNIFRNLNIPIVDADIISRKIFEDKLLLEKVFVHFGQSIKNDDGTLNRKALGKIVFSDEEKLKELNNLTHPRIREKIISEIEKLRKKGENIVVLDAAILVESGFLDMVDKLLVVTCKQEVQISRIQKRDNCSEQEALSRINSQMSQEEKSKYGDYIIDNSGTITELESKAHKFIEYMKENWRE*

>CD630_11420 Clostridioides_difficile_630_NC_009089 electron transport complex protein RnfB

VKMVILTAVLVLGIMGLIFGIVLDFASKKFAVEVDERVEAILGVLPGANCGGCGFPGCGGLANAIVEGNAPVNGCPVGGADVGAKVGEIMGISAEAGEKQVAKVICKGTCSSAKDKYEYEGISDCRAANVLNSGAKMCKFGCLGLGTCKDACKFDAISIVDGIAVIDEEKCVNCGKCKEVCPKGIIITKPESQEVVVECNSKEFGKAVKEKCTAGCIGCGMCVKACKFDAIIFEDKIAKIDPNKCVGCMQCVAKCPTKVISGDITKKKKVTIDQELCVGCTVCKKQCKFDAIEGELKEKHKVDADKCVGCHLCMEKCPKKAIKIL*

>CD630_11450 Clostridioides_difficile_630_NC_009089 cell shape-determining protein MreB

MAKEKKKEKKGFFSFNKMTKDMGIDLGTANTLVYIKGQGIVVREPSVVAIRDDSKEVLAVGEEAKKMIGRTPGNIVAIRPMKDGVIADFDITQSMISYFIQKAADKKGVVSPRIAICVPFGVTEVEKRAIEEAARQAGAKDAFLIEEPMAAAIGAGLKVEEPEGNMVVDIGGGTSEIAVISLGGIVTAKSIRIGGDEFDESIVAYVKKEYNLMIGERTAENVKINIGSTFKDDEEINMQIRGRDLISGLPKTIEICSTEVREALKEPVSSIVDAIKSTLERTPPELASDIMENGIMLTGGGALLRGLDKLITQETGMSVQIAETPLDCVALGTGKSVEDQEIFEKVLMMNTKN*

>CD630_11500 Clostridioides_difficile_630_NC_009089 septum site-determining protein MinD

MSEVIVITSGKGGVGKTTTAANLGTALSLENKKTVVVDADIGLRNLDVVMGLENRIVYDIVDVVEGTCRLKQALIKDKRFDNLYLLPAAQTRDKNAVSVEQMIDLCEKLKESFEYIIIDCPAGIEQGFKNAVAGADRAIVVTNPEISAVRDADRIIGLLEANEIKEIRLVINRIRNDMVKRGDMMDKQDIIEILAIDLLGLVPDDESIIISTNKGEPAILDSKSLAGQAYKNIAKRILNEEVPLLDLEVEDGFFGRLKKMFSMAK*

>CD630_11640 Clostridioides_difficile_630_NC_009089 GTPase Obg

LFIDKARIFVKAGNGGNGSVAFRREKYVPAGGPDGGDGGRGASIIFEVDLGLRTLMDFKYQKKYQAQNGGDGSKGKRAGKNGENLVLKVPAGTVIRDEATGLVLADLKKEGDTAIVAKGGIGGKGNQHFANAVRQAPAFAKSGTDGEERWITLELKMIADVGLLGFPNVGKSTFLSVVTKAKPKIANYHFTTLTPNLGVVQTKFGDSFVLADIPGIIEGASEGIGLGHEFLRHVERTKVLIHIVDISGLEGRDPIEDFDKINDELKLYNEKLSKRPQVVVANKFDILEDESKFEKFKSELEGRGYTVFKMSAATRQGIDEVIAYVSKMLKEVEDVELVSEEEMYRPELDIGTEEELSIDIEDGVYVVTGKALRRIMYSVNFDDMESLQYFQKAMESQGVFDRLREMGIEDGDVVKIYELEFEFYN*

>AEC_RS02000000220890 Clostridioides_difficile_QCD_37x79_NZ_CM000658 FAD-binding oxidoreductase

MYKLIDKKDIDFLIDTCGEENVLVGSDINEDFSHDELGGIEKYPEVLVNVLETEQVSKIMKYAYKNNIPVTPRGQGTGLVGAAVAINGGIMINLCKMNKILEVDYENLTLTVEPGVLLMTIGQYVQDRDLFYPPDPGEKSATIAGNINTNAGGMRAVKYGVTRDYVRGLEVVLPNGEIINVGGKVVKNSSGYSIKDLLVGSEGTLGIVTKAILKLLPLPKKSISLLIPFPDLSMAIETVPKIIKSKSIPTAIEFMERDVILAAEEFLGKKFPDNTSDAYLLLTFDGNSTEDIEKEYEKVANLCLENGALDVFISDTQERNDSIWSARGAFLEAIKASTTQMDECDVVVPRDKIAEFIRYTHELQDKLKIRIKSFGHAGDGNLHIYILKDGMDDNTWKIRLKETFDYMYKKSRELSGQVSGEHGIGYAKKEYLHESNSDAYMMLIKNIKLAFDPKNILIQGKYIRNINLIIFTKQKILN*

>CD630_11750 Clostridioides_difficile_630_NC_009089 acetate kinase

MKILVLNCGSSSLKYQLIDMNNEEVLCIGLVERIGIEGSILKHEKAGRDDKYVVEQPMKDHKDAIALVLEAVAHPEFGAVKEMKEIDAVGHRVVHAGEKFATSVVITPEVEEALKECIDLAPLHNPANIMGIDACKAILPDVPMVGVFDTAFHQTMPKSSYLYGLPHELYTKYGVRRYGFHGTSHNYVSQRAAEILGKDIKDLKIVTCHLGNGASIAAVDGGKCVDTSMGFTPLEGLIMGTRCGDIDPAILPFLMRKEGLDADGLDKLMNKESGVYGMTGISSDFRDIEDAAKNGDERAQATLEAYVKKVQKYIGAYAAEMNGLDVVVFTAGVGENGKAIRADIASNMEFLGMKLDKEANDVRGKETVISTADSKVKMLLIPTNEELMIARDTLRLVK*

>CD630_11800 Clostridioides_difficile_630_NC_009089 enoyl-(acyl-carrier-protein) reductase II

MNKICKILNIKYPVIQGGMAWVATASLASAVSNAGGLGIIAAGNAPKEAIKKEIVECKKLTDKPFGVNVMLMSPFVDDIIDLIIEEKVQVITTGAGNPAKYMDRLKEAGTKVIPVVPTIALAQRMEKLGATAVIAEGTEGGGHIGELTTMVLVPQVADAVNIPVIAAGGIVDGRGIAASFALGASAVQVGTRFICSEECSVHSNYKNLVLKAKDRDAIVTGRSTGHPVRTLKNKLSKEFLKMEQNGATPEELDKKGTGALRFATVDGDIEKGSFMAGQSAAMVKEITPCKEIIEAMVNQAREIMPAIEL*

>CD630_11820 Clostridioides_difficile_630_NC_009089 3-oxoacyl-ACP reductase

MINLTGQVAVVTGGSRGIGKEIAKKLASFGADVVINYTSKEDEALKTKNEIESMGVKCTSIKCDVSKFDEVNQMIDSVVSEFGKIDILVNNAGITKDGLLMRMKEEDFDRVIDINLKGVFNCTKAVTKPMMKKKYGRIINMTSVVGIMGNAGQTNYCASKAGVIGFTKASARELASRNININAVAPGFIETDMTKVLSDDVKESTLANIPKKSYGKPEDVANAVAFLVSDMSSYITGQVINVDGGMVMQ*

>CD630_11840 Clostridioides_difficile_630_NC_009089 3-oxoacyl-ACP synthase

MNKRVVITGLGCVTPLGTGKEEFWSNIKSGVSGIDKITNFDASTYQTQIAGEVKNFHPEEYISKKELKRLDKFAQFAIVSAKLAVEDANLDLDKVDRERFGVIIGSGIGGVEAIETQHKILLEKGNKRVSSLFVPMMIGNMAAGQVSIFLGAKGPNTNVCTACASGTHSIGDAFKVIQRGDADIMVAGGSEAAVTGLAFAGFCNMKAMSTRNDDPKTASRPFDKDRDGFVMGEGAGIVILEDLEHALARGAKIYAEVVGYGLTADAYHMTTPAENGEGAARSMNMALKDGNVPLEEVDYINAHGTSTYYNDLYETMAIKTVFGEKAYDLCVSSTKSMTGHLLGASGAIEAVVCAMSIEDSFVPPTINIQEVGEDLDLDYVPNQGKEKNIRYALSNSLGFGGHNATIVLKKYV*

>CD630_11900 Clostridioides_difficile_630_NC_009089 acyl-CoA N-acyltransferase

MPTITLKNGVDVLIREGVREDAQSIIDFYNEVGGETHFLSFGKDEYKISLEEQENAIESAKASDNSVKLIAFIDGEIVGIATIDSNQKAKGKHVGVLGIVVKEKYWGIGLGKRLMLDLIEWCKSNGITKKITFVTNEENYNAIGLYKKVGFEVESILKKECYYNGVYTDLIGMSLLLGI*

>CD630_12050 Clostridioides_difficile_630_NC_009089 geranyltranstransferase

LEFKQCLKEKASFVEKVLKEYMPKEEGYQKTVIEAMNYSLSAGGKRLRPILTLEACKIVGGNEDEAIPFAIAIEMIHTYSLIHDDLPALDNDDLRRGRPTNHKVYGEAMGILAGDALLNYAFEVMLAGSINKENPEKYLKAINEIAKGAGIYGMIGGQVVDVESENKQIEKEKLDYIHMNKTAAMMVGCMRAGATIGGANSEQMEEITKYAKNIGLSFQIVDDILDIVGDEAKLGKKVGSDIENHKSTYPSLLGLDKSKEIAHNLIDEAKKSIEKLSDDVDFLKGLAEYIIDREY*

>CD630_12200 Clostridioides_difficile_630_NC_009089 NUDIX family hydrolase

MVLEEKTISSDRVYTGKVITLKVDTVEIPGQGYQKRELVEVGGAVGIVAITDDNKVVLVKQFRKPIEKPIFEIPAGKLEKNESPKECAERELKEETGYSAKNIKLIHKFFTSAGFSNEIMFVYLATGLTPGENNLDADEFLDVYEIELEEAYNMVLKNDVEDAKTSIGLLLVKDMFKN*

>CD630_12260 Clostridioides_difficile_630_NC_009089 aminodeoxychorismate lyase

MNFKENRLKIAVLIIVILIILAGIFVFIQIGPYDKNNKKDVIIDVPSGASVGKISDILYENKLIKNELLFKLLVKVSNKAPSIKSGTYLLNQSYSNNDIISLLVSGKIYQDGIKVTIPEGATSKEIIAMLVSKNLGDKATFENLIKKPQEFYDKFPYLKEDGITSLEGFLYPETYYFNSKKQSEEDILSEMLKVFDSKYTDKFKKKQKELNMTLQEVMEMASIIEKEAVLDKDRPIIASVFYNRLKVGMPLQSDATIQYIFEERKKIVTYDDLKIDSPYNSYKNKGLPPTPISNPGIKSIEAALYPEKTDYLYFVAKIDGGNNYSTNYQDHLKYVKEYKEARDKQSKDTKATNKENTKK*

>CD630_12480 Clostridioides_difficile_630_NC_009089 ribonuclease III

MKISKKLLDNIQRFENVINYKFKNKEYILEALTHSSYSNENKKYNFNERLEFLGDSVLGIVISDYLFNEEANLPEGELTKLRANIVCEDSLSEVANDINLGIHMLLGRGEEATGGRHRTSILADAFEAVIAAIYLDGGFESARQFILHHMENIIYDSRKGNIFRDYKTHLQEVLQGNGENNIWYRLIEEKGPDHNKRFVMEVGINDDVLGIGEGKSKKEAEQLAAKIALKKKLWEK*

>CD630_12500 Clostridioides_difficile_630_NC_009089 chromosome partition protein

LYLKRLELKGFKSFPVKTDIIFKEGITAIVGPNGSGKSNISDAVRWVLGEQSIKSLRGDKLEDVIFAGTDTKKPMNYCEVALTIDNSENQLELDFTEVTIRRRAYRNGESEFFLNNKSCRLKDIKEVFLDTGIGKDGYSIIEQGKVDEILSNNPLSRRKVFDEACGISKYRYKKQEAERNLSNTKENLERIDDVYIEIENQLKPLFNQQTKAKKYLEISEKLKTLEVNSFIREIEGIEKELSEVNEHRKVIEKELNEKEEQKNVVEKKQEDINKEVEVLQDVIEKSVDYINSIKGVISKKESQINLIKERIRNFTNEISRKNLEIKDIKEKLNENKQYIKELESNKLSGSEELSTLQENIKVLEGSKDKQKIKLESLNNEIELLKESIIDILNKKQEFSNKLSTLNANKENMNIRDENINSEITELNKNIEIKSSELDTINKEFNMQNENLKNVNNRHKELSINLQDSISEHNKLEDEIQKSKYNLNGYNSKLNVYIDMENHYEGFNRGVKEVLKNKNLKGVHGALGQIINVPEKYEKSIEAALGAYMQNIITDNEFSAKSAINYLKQNNLGRVTFLPLNIIKSNKISLGNLKANTKFIGIASDLITFDEKYRNIIENILGRTILINNIDEGIKFAKETGHRFKIVTLDGEILNPGGSLTGGSLKTNGNILSRKRYINEYTEKISNIKNEISHLELKRESLDKDVKNIKNEIDSHESKIKDLEKSIIIKSTSIKNVESEIESLKGSITKLENEKNDLNSNLNYTLEKSDDVRKDMEELDDLYNKNKEKIDALNEEIKRYNDLYDKEKSEFDELNLSLVKKTEVYNSIVRDIKRISGENCELEEKNKQLEESLNYEEHEIIKLQDSILTEEKEKENLTKQLGDSNRNLETRKIAKDDLKNSFDEINKELKTIDRQHIELKESLFKVGGRLERLKTSQDTYINKLFEQYDMTLVQALEIKDEDLDIDRKFLESLKREIRSLGNINIDSIKEYEEIKERYDFYSEQKQDLEESMEEIEKLIHTLEENMKSEFEIKFEEISKNFKYVYKRLFGGGCGELTILDKENLLESDILITAQPPGKKMKNLNLLSGGEKALTAISILFAILITKPTPFCILDEIEAPLDDANIFRFGEFLKDLSKETQFISVTHRRGTMEAADYIYGVTMQEKAISKVISLKLKEAQEITDII*

>CD630_12510 Clostridioides_difficile_630_NC_009089 signal recognition particle receptor FtsY

VLKKLFGFGKDKEKEIEKKDAEEEIEVEDSVDNLENLEETIFSGLEEEVIDKVEDVEEKNEESVNDEGTEEIENFEKIEVEMDSNGKEVENISNNDSQEEIIEELENYDEIESKEIEDKEDKKVNLFERLKQGLTKAKQGITDRIDEVLKSYTKIDEELLEDLEEILITADVGVNTTMDIIERLRDKIKQKGITEPIKVREELKSIVEDILTNENSTLDIEPAPCIILMVGVNGVGKTTTIGKLANRYKKDGKKVLLAAADTFRAAATEQLEIWANRTNVDIIKHQEGADPGAVVFDAIKAAKARKTDVLICDTAGRLHNKANLMNELGKVFKIVDREFPEAKREVLLVVDATTGQNAVVQAKTFKEVADITGIVLTKLDGTAKGGVVLAVKSEVDVPVKLIGVGESVEDLQDFNAKSFSDALFGN*

>CDIF1296T_01324 Clostridioides_difficile_ATCC_9689__DSM_1296_strain_DSM1296_CP011968 GTPase

VSNEYEEYLMNDNLHINWYPGHMKKTKELVKNNLKLIDVVIELLDARIPFSSKNPDIDRLVGDKPRVVVLNKSDMADRDKLNQWIEYYKKINIKAIPVDTIKGVGINKIIEECKNVTREKMSSLKDKGRKERAIRIMIVGVPNVGKSSLINKLTGRKSTQTGDKPGVTKGKQWVRLKGNLELLDTPGILWPKFEDQEVALNLAFSRAIKDEILDTETLALRLIEKLMKIEPEKLKARYKLDCLGETPIETMDMIGHKRGFITGKKELDYTRIATTVLNEFRDGKIGNITLEVPENVKR*

>CD630_12650 Clostridioides_difficile_630_NC_009089 CarD family transcriptional regulator

MYKIGESVMYPKEGACSVNDIVTKKINHEMQKYYELSVIFNSNLKISIPVLNADRIGIRPVMDGNDVDNFIQSINKTDGVWIFDRKERLKLYQDKFHSGDVFEIVKLIKMLMIQDSSKQLCSTDKEFLNKAQKFALSELAAAQCKSYTMVLEEMKKHILNSKNTN*

>CD630_12680 Clostridioides_difficile_630_NC_009089 multidrug family ABC transporter ATP-binding protein

MDIVRVNNITKRFNDKLVLDNISFSVKKGEIFGLIGPNGAGKSTLINIITNLMLPNSGSIQINDLDLSKDYIKAKSIIGLVPQELAIIETLTPFDNLEYFGAFYGLKGKLLKERIIEALEVTGLTEVKKKKVKKLSGGMQRRLNIGIALLNHPKILILDEPTVGVDPQSRNHIFNFIKDISKKHETTVIYTSHYMEEVEHLCSKIFIMDEGKEIAFGDNDYLKSLVSTNTKLIMEIKNINAQLIFDLKNTKGVISVLENNSLLELDIDKKLQLTDILSIIDKNDSKIMKISYEEPSLEDVFLNLTGKNLRD*

>AEC_RS0207650 Clostridioides_difficile_QCD_37x79_NZ_CM000658 magnesium chelatase

MLSIINSSNLVGIDSFLVKVEVDVSNGIPSFNIVGLPGKEIKEARERVKSAILNSGYKFPSTRIVVNLSPADIKKEGAFLDLSISIGLLRELIKKDENYIRESMFIGELSLDGKIRKVRKVRGILPIIMGAKTQNIKRIFIPIENIKESLLVDEIDIIPIKSLKECVDFLNEEIKVDKVSIMSFLDDKSRKENGELEKDNSYIDCKYTKINNEESKYDEDFKDVKGNYFVKRSAEIAAAGNHNMFMIGPPGSGKTMIAKRVRTILPDISIEEMIEVSKVYSILGMINESKGIIDKRPFRAPHHTTTKQSLIGGGMDARPGEIALAHRGILFLDEIAEFDRKILETLRQPIEDGYVNISRVKYSAKYPCRVLLVAAMNPCPCGYYMSETECRCRSNEIDRYINKISGPLLDRFDIFVEVNSIKYSDFNSLKQEESSQKIKRRVENARKIQINRFKKDNIKNNSEIKAYNLFKYCKLEKEASKTAEMIFNKYNLSSRSYTKLLKMARTIADLEERDLINSQCIIEAFSFRKAYYSYFK*

>CD630_12790 Clostridioides_difficile_630_NC_009089 cysteine desulfurase

MEKRRLYMDYSATTPIKKEVLDAMMPYLTDYFGNASSFHTFGREAKDALDKAREQVAALINAEPSEIYFTAGGSESDNWTLEGIAYANKNKGNHIITSKIEHHAILHTCEYLAKHHGFEITYLDVDSEGKVDLKQLEDSIKDTTILISIMFANNEIGTIQPIKEISEIAKKHKILFHTDAVQATGNIPVDVKELGIDLMSMSSHKIYGPKGVGALYIRKGVRLHNFVHGGAQEKSKRAGTENIPAIVGYGKAAELAKENMQNHVETLTRLRNKLIDGVLERIPYTRVNGSLENRLPGNANFAFQFIEGEGILLLLDMLGIAGSSGSACTSGSLDPSHVLLAIGLPHEIAHGSLRLTVGDFTTDDDIDYILENLPKVIERLRSMSPLYDDAKKQGLVK*

>CD630_12800 Clostridioides_difficile_630_NC_009089 NifU family iron-sulfur cluster assembly protein

MQYSDKVMEHFMNPRNMGEIDNASGVGEVGNPTCGDIMKIFLDIDGDVIKDVKFKTFGCGSAIASSSMATEMIKGKTIKDALELTNKAVAEALDGLPPVKMHCSVLAEQAVKAALIDYAQKNNIHIPELDGYVIDDAHDHDVEEEE*

>CD630_12810 Clostridioides_difficile_630_NC_009089 tRNA-specific 2-thiouridylase MnmA

LFMNKKVMIGMSGGVDSSVAAYLLKQQGYDVIGVTMKLWQDDDVVEIEGGCCSLSAVEDARRVANKIGIPFYVLNFREVFKEKVIDYFIDEYLEGKTPNPCIACNKHIKFDDFYKKARQIGCDYVATGHYAKIEKDESTGRYLLKKSVTDKKDQTYALYNLTQEQLEHTLLPIGDYEKDRVREIAKEMGMAVHNKPDSQEICFVKDNDYANYVKKHSKKRIEEGFFVDTKGNILGKHKGILYYTIGQRKGLGITFGKPMFVIDINPINNTIVLGDNEDLFKKELIAKDVNFISIDTLEEPLRVQAKIRYSAKPSPATIHRVGEDTIKIVFDEAQRAITKGQSVVMYDGDIVVGGGIIEKSL*

>CD630_12820 Clostridioides_difficile_630_NC_009089 alanine--tRNA ligase

MEKMGLNEIRSKFLEFFESKGHYVANSYSLVPNNDKSLLLINSGMAPLKNYFSGVEVPPSVRMCTSQKCIRTGDIENVGITARHATFFEMMGNFSFGDYFKRESIKWGWEFVTEWLNIPEDKIWVTVYEEDDDSYDIWAKEMNFPEERMVRLGKDDNFWEIGTGPCGPCSEIYFDRGEEYGCDNPDCKPGCDCDRYLEFWNHVFTQFDRDEEGNYSLLENKNIDTGMGLERMGCIMQGVDTIFEVDTIKSILEAVEKLTGVKYGENPKNDISIRIITDHIRAVTFLVSDGVLPSNEGRGYVLRRLLRRAARHGKLLGVKELFLQKLIDEVIKVNDKAYPVLVEKESYIKKVVGIEEEKFNETIDQGTEILNSYIEVLKNEGKTVLSGQEAFKLYDTYGFPIDLTKEILEEEHLSVDEEAFNEEMEKQKERARNARGNMDGESWKEDPLSKLESTVDSTFNGYSEIYGEGTIEAIVKDDELVQSAEEGDKVSIVLDNTTFYPEGGGQVGDCGLITNENLVLEVLNTKKGANNSIKHIGIIKSGRISNGDKVKTLVDRETRMSAARNHSATHLLHKALREVLGEHVNQAGSLVTPERLRFDITHFEAISNEELKVIEEKVNNVILSSLDIKCDIMNIKEAKEKGATALFGEKYGDEVRVVSMGDYSTELCGGTHLTNTSQVGMFKILSEGGVAAGVRRIEAITGKAVYEYLKERDGIISEVCVNLKSKEDNLIQRISSLLEENKNLSKELHDMKAKMSLQSVDSIFDSKVEVNGVNLITNKFEGMDMDTLRETADNLRDKLGSGVVVLANVVDDKVNFVVTATKDVLDKGIHSGNIVREVAKIAGGKGGGRPNMAQAGASDVSKVDQALSYASEVIKTQVK*

>CD630_12870 Clostridioides_difficile_630_NC_009089 Fur family transcriptional regulator

MANTMDLLKDKLKETGFKITPQRRAIVEILLKHDHSHLSSEEIYDLVRVDCPEIGLATVYRTMQLLDEIGLISKLNLDDGCIRYEISLHKEDCHNHHHLICKNCGKIMEAKEDLLDNIEKEIQSLYKFKILDHDVKFYGLCDECNGVSDSEE*

>CD630_12890 Clostridioides_difficile_630_NC_009089 ribonuclease J family protein

MQLFKKNTNKIKVMALGGLNEVGKNMTVVEYKDEIIVIDAGLSFPEDEMLGVDIVIPDITYLVKNRDKIKGIFITHGHEDHIGALPYILKKINVPVYGARLSIGLIQVKLKEHKMNNVKLNVIGPRQVIKLDNMEVEFLKNNHSIPDAYSIAIHTDQGIIYHTGDFKIDLTPIDGDVMDMHRICELSKKGVLLMLADSTNAEKPGFTMSEKTVGVGLDELFAKGNGRRIIVATFASNIHRLQQIINTAEKFNRKVAISGRSMVNVVGVAKELGYLDISDDMLIDLNDICKYEDSELVIITTGSQGEPMSALARMAFSEHKKVEIKSGDLVIISAHPIPGNEKLISRVINFLFEKGAEVVYSDIADIHVSGHACQEELKLIHALVRPKFFMPAHGEYRMLKRHAEIAEQLGMDKENIFVMQTGDVLELDKNSAKVANRIQTGNILVDGLGVGDVGNIVLRDRKHLSEDGLMIVVVTISKDEGKVLAGPDIISRGFVYVRESEDLMDGAKDIIKNVLNECEEKNIKEWAYLKNNIKENLKEYLYQKTKRNPMILPIIMEV*

>CD630_12960 Clostridioides_difficile_630_NC_009089 segregation and condensation protein B

MKREDIKYIIESVMFAYGEPISIKELNYIINKELSSKEIEIMLNLLIEEYREQNRGIQIIKLENKYQMCTNKDYAEYIKKIIEPKKKKSLSQATLETLTIIAYKQPITKVEIEDIRGVKCDKVLQTLFENELIREAGRLNKIGKPIIYKTTDEFLKLLNIESLEELPPIENYQEVATNE*

>CD630_13090 Clostridioides_difficile_630_NC_009089 translation initiation factor IF-2

VSKTRVYQIAEELNISNEELINKLAELDINVTDKDSVLEGEELELALEMLGEDLSQENGNVIEIDGKLTVQVLATKLDKSPSEIIMKLMKMGTMATINQEISFEIAALAAKDYGFELTVAESDDTEALEIEALMEIEEDKEEDLKPRPPVVTVMGHVDHGKTSLLDAIRKTDVISGEAGGITQHIGASEVKINGHKIVFLDTPGHEAFTSMRARGAQVTDIAILVVAADDGIMPQTVEAINHAKAAGVPLIVAINKIDKPGANPDKVKQELADQGLLVEDWGGEVIAVPVSAKKKEGIDTLLEMVLLVAEMEELRANPNKRAVGTVIEAELDKGRGPVATVLVQGGTLTVGDPIVAGVACGKVRAMINAKGKRVKTAGPSTAVEILGLSEVPQGGDQFVEVPTDKIARSVAARRQQIVRDEMLKSTQRLSLDALFSQMSEGSIKDLNIVIKADVQGSVQAVKQSLEKLSNEEVQVKVIHGGVGAVTESDILLAAASNAIIIGFNVRPVPGAESLGEKENVDIRTYTIIYKAIEDIQAAMTGMLDPEYVDEETGKAEIREIYKISGVGTVAGCYVTNGKIFRNCKVRLVRDSIIIHEGELAALKRFKDDVKEVNSGYECGMSFVNYNDIKEGDIVEAYITKEVERKL*

>CD630_13180 Clostridioides_difficile_630_NC_009089 polynucleotide phosphorylase/polyadenylase

MFEHKIFKMDFAGRELSVEIGKICEMASGSCIVRYSDSMVMVNTTKSAKPRDGIDFFPLSVDYEEKLYSVGKIPGGFLKREGKPSEKAILTSRLIDRPIRPLFPKGFRNDVQVVATVLSVDQDCTPDIVAMIGSSIALSISDIPFNGPTGSVCVGLVDGAFVVNPNAEQREKSSMHLVVSGTKEAIMMVEAGADEVPDEVMLDAILFAHQEIKKIVEFIEGIVAEVGKEKMPVELYHAGEEITQLVREFATDKMKKAVQTFEKLERMENMDRVKEETLAHFEETLEDFEDFVGDIEEVLQDIIKEEVRKLIVHENVRPDNRKLEEIRPIWCETGMIPRAHGSAIFTRGQTQVLNVATLGALGDVQKLDGLDEEENKRYMHHYNFPAYSVGEARPSRGPGRREIGHGALAERALLPVIPSQEEFPYAIRLVSEVLSSNGSTSQASVCGSTLSLLDAGVPIKDMVAGIAMGLIKHDGKVAVLSDIQGMEDHLGDMDFKVAGTEYGITAIQMDIKIDGIDKEILQRALKQAKEGRIHILGEMRKTISQPKPELSPYAPKIVKMQINPDKIKDVIGPGGKIITKIIDETGVKIDIEQTGEVFISGIEIDMIKKAQELINNIVVEPEVGKTYKGKVSRIMNFGAFVEILPGKEGLLHISHIAHERVAKVEDVLNIGDEVEVKVTEIDEKGRVNLSRKVLLPKPEHKNK*

>CD630_13260 Clostridioides_difficile_630_NC_009089 30S ribosomal protein S12 methylthiotransferase

MLKIALESLGCSKNLVDAEIMMGILNNKGYKLIGDFEEADVIIVNTCGFIESAKQESIDTIINFAELKKTGNLKLLIVTGCLAQRYSEELKTEIPEIDAIVGTGSYQNIDKILKELSEIHQIVSLNDIEFVFNEDLPRYISTPSYMAYLKIGEGCSNNCTYCIIPKLRGKYRSRKFEDIIKEAKKLAESGVKELVVIAQDTTKYGFDLYGKERLSELLEELAKIDGFKWIRVMYSYPESITEELIQVIKKYDNICSYFDMPIQHASNNILKLMNRKTTKEDILNKINLIRSNIPDAILRTTIIVGFPGETEDDFKQLVDFVEEVKFDRLGAFAYSREEDTPADRLPNHIDEEVKIQRRDTLMMIQQKISEELNDKKIGKTYEVLIEEQIEDNVYTGRTQGDAEEIDSIVYVKSVDNLEVGEFVSVQINDAMEYDLMGDVLYELA*

>CD630_13270 Clostridioides_difficile_630_NC_009089 CDP-diacylglycerol--glycerol-3-phosphate 3-phosphatidyltransferase

MNLPNKLTLFRIFLIPVFVLIMLLNVPNKFLIACIIFIIASITDALDGKIARKYNLVTDFGKFMDPLADKLLVISALTCMIEDHLVSSWMVIIIVARELTVSILRAIAAADGKVIAAGNSGKLKTITQMVSIVFLLLGAQFENVLILNIGEILILIATLLTLYSGWEYLYKNKELFMSSK*

>CD630_13280 Clostridioides_difficile_630_NC_009089 recombinase A

MSVDQEKLKALNEALGKIEKDFGKGSVMKLGEATSMSIDVISTGAIGLDIAIGIGGLPRGRIVEVYGPESSGKTTVALSCVASAQKDGGIAAFIDAEHALDPVYAKALGVDVDNLIISQPDTGEQALEIAEALIRSGAIDIIVIDSVAALVPKAEIDGDMGDSHVGLQARLMSQALRKLTGSIKKSNCVAIFINQLREKVGIMFGNPETTTGGRALKFYSSVRLDVRKIDTIKQGDKVIGSRTRVKVVKNKVAPPFKQAEFDIMYGEGISKIGDLLDIAADVDIVKKSGSWYSYNDTKLGQGRENVKKFLEDNLDLTTEIDEKVRAFYNLNEEHEESGTSVSKEIVEE*

>QAE_RS0206550 Clostridioides_difficile_QCD_23m63_NZ_CM000660 aspartate aminotransferase

MNYSNRVSAMQASPIRKLVPFAQAAKDKGIKVYHLNIGQPDIKTPKGFFDAVKNFDSEVLEYATSEGIPELLEALQNYYKTYNMNFEKDELLVTNGGSEALLFTMMAVCDPGDNLLVPEPFYTNYNGFGQSVNVEVNAVTTKAENGFHLPSKEEILSKVDDKTKAIILSNPGNPTGAIYTKEELNILAEIAKEKDLWIIADEVYREFVYDGLEYTSCGNLEGVQDRVIIIDSVSKRYSACGARIGSIACKNKGLIAQILKLCQGRLCVPTLEQIGAVELYKTPVSYFKEVNEEYKKRRDVLYNELMKVEGVICKKPTGAFYIVAKLPVENAEDFTIWMLKEFNKDNETVMVCPAEGFYATPGLGRDEIRLAYILNEKDLHRAATLLKEGLEQYVALTKIIF*

>CDM120_RS07060 Clostridioides_difficile_M120_NC_017174 two-component sensor histidine kinase

ILVGKKFNNKKIVTLRTVFVRYSSLFFAITVVLVSILILSFPILLSLNIILPANYVEKQIYENKDKIISSKQVTKDLIPDLCEYGVYTLNGKVISGTFNKNESKEVWDLMRGFERRTITSSKNYIKLSRKNEVCIIRYSIVAEFVSPTLRTYLPKPELLGMIIFSIIFLIEIIILSKLFGKKLNAEMELLKNTTEKIEQQDLDFVIESSKIREINNVLFSMDKMKLALKDSLEKQWMLEENRKEQISALAHDIKTPLTIIHGNTDLLIEINENPELSEYMEYIAKGATQIEKYINTLIEISKTETGYILNKEVINVSEFIEDILIQIEALARTQNLNVEFSKQGNLPESITIDKELLFRAIMNVISNAIDYSPSQSKLYISVSVSNKYLKFVITDCGSGFSKADLSKATGQFYTGDLSRNSKSHYGMGLYIVNNIVQKHNGILHIENSIKTGGAMVTIEIPIV*

>CD630_14310 Clostridioides_difficile_630_NC_009089 DinG family helicase

MDNIISVLNDVVFLDIEVSGLDCLNSEILEVGAVKVKDWKIYTYESLIKNKFEVPVEVFSVCKNLDKNDLEIANEIELVEDRLVNFVEDSFIICHDLSLKKKFFEYHMPKLKNKFIDLIELAVILEPYHKDYSLEYLKNTLTNCNSKVENRALSDAIDIINIVNCLLVKFNNYEKTTLEPLSFKINSYLKKFNLPTWEWSKFLEEANYDLSNNINIKKEYNIFDSKEEKKKERETLKILNEEEKNYEELLKYKTIWENKEGFTYEYRPGQYELTKTIRELFRNSEDEEKIACIEAPTGIGKSVGYLLPAILEARINKKRLLISTDTKELQIQLINKDIPNVLDSLGLNGKVSYGYIKGKNNYICIDRLEAYIDDYESQNPTKGEILSLIFLKRLVEGGKYGDIEEINYLVFDNFKEISTHLRNVSCDPNMCRPKKCKKDCLYKNRIEELKEEHITVVNHSLLAKWPYKDEKPLENIIVDEAHNLTEKGYDFFSSIINSKSLRYLLQEIYPYEFIQNSSFIYKKYSRNMRKIKAFDKFYNVLKIGREDKQKIARSINLIIEEIDSILNFGNCNEYNNVSNYNLRWELNLQIDEIVGKLKKDGIDTEISYRAYSEKIKLSCEKIIKNLVSIIIIIYRNIDDDSIDKEADIYKFGKAKTRDLEDIKIIFEIFLEYDEKDDYARIVEIDKNYNVFEFRVVPLKIADLFEENILSQLEKGIFLSATLSLSESMSYFKNTLGIDRVKNVEKIIEPIFDYKNRVSVVGFSDICEYRNSEFPNEMSKIISNISKITEGHTLALFNSKDRQEKTYEILKKYLHSFNLEIYADKKGIRHLNDLNRKCVVLGSKGCFEGVDIPGDGLVCVTLDKLPNLNPKDPLYFTIMKKYGIDYYTINYPQMTIKVKQAMGRILRSKYDYGCFVIFDVGTNISVLKRLEKDLHDCKISKVNSNEFYTYIRRHLNKSRSLILKSVIFDTIKALNVDAKMDNNDVDKDIIKKDINENIRQRAVKGEVYHIDIIKKDMKVKYFDRNYLINLDIFMREEDKN*

>CD630_14320 Clostridioides_difficile_630_NC_009089 ribonuclease

LAKQKFYAVKKGKNIGVYNTWDECKKQVNGFSGAEYKSFSTFQEAKEYIDGSEKLSFQEDKEFIEAYVDGSYEHSVKMYGSGVVILKNNEVIKTYSEKGKEKTLVSMRNVAGEIEASKIAMQYCIDNNVQNLILYFDYEGIEKWCTGVWKTNKEGTIAYKNFYDSIKNKLNVKFTKVKAHSGNKYNEEADKLAKKAIGV*

>CD630_14330 Clostridioides_difficile_630_NC_009089 bifunctional peroxiredoxin/chitinase

VIYMPNLPSLGSKAPDFKANTTNGPIRLSDYKGNWIVLFSHPGDFTPVCTTEFLCFAKYYDEFKKRNTELIGLSVDSNSSHLAWMYNISLLTGVEIPFPIIEDRDMRIAKLYGMISKPMSDTSTVRSVFIIDNNQILRTILYYPLTTGRNIPEILRIVDALQTSDRDNIVTPANWFPGMPVILPYPKNYKELKNRVNSCNKKYSCMDWYLCFVPDNYNDEEVSKKIDNTCSWKKEHTKNIENECNCEHEHHDYLNKALDCKQEHKTDIKDDCNHEKKHTKNTNKVHNSKQDKFKDKSCDEMNFNYDKDESCDKINSSYNKEDSSYEDFYKHNYKNYDYTSEKNTKKIAMKTLKDSKKLVRPQITDPYNPIVENANCPDINPIVAEYVLGNPTNVDAQLLDAVIFAFAEIDQSGNLFIPYPRFLNQLLALKGEKPSLKVIVAIGGWGAEGFSDAALTPTSRYNFARQVNQMINEYALDGIDIDWEYPGSSASGITSRPQDRENFTLLLTAIRDVIGDDKWLSVAGTGDRGYINSSAEIDKIAPIIDYFNLMSYDFTAGETGPNGRKHQANLFDSDLSLPGYSVDAMVRNLENAGMPSEKILLGIPFYGRLGATITRTYDELRRDYINKNGYEYRFDNTAQVPYLVKDGDFAMSYDDALSIFLKTQYVLRNCLGGVFSWTSTYDQANILARTMSIGINDPEVLKEELEGIYGQF*

>CD630_14450 Clostridioides_difficile_630_NC_009089 para-aminobenzoate/anthranilate synthase glutamine amidotransferase component II

MILMIDNYDSFVYNLVQYIEELGETVVVKRNNEIKISDIEELNPEVIVLSPGPCSPKEAGICIDIVEHFKGKKPILGICLGHQTIGHVFGGDIIKAQQPVHGKVYSINHTNKGVFRGLKNPLNVTRYHSLIIDSNTVPKELEITAITDKGEIMGIRHKKYLIEGVQFHPEAILSEYGHEMLKNFITEARERVHV*

>CD630_14490 Clostridioides_difficile_630_NC_009089 GTP cyclohydrolase I

MNKVDKEKIQHAVREILEAIGEDPDREGLIETPNRVARMYEEIFSGLSEEPRDHLKVLFADEKHEELVLVKDIPFYSCCEHHLVPFFGKAHIAYLPKGGRLTGLSKLARVIDTLAKRPQLQERITKNAADIIMEELQPYGVLVVVEAEHMCMTMRGVKKPGSKTVTSAVRGIFEKDIASRAEAMSLITMK*

>CDIF1296T_01539 Clostridioides_difficile_ATCC_9689__DSM_1296_strain_DSM1296_CP011968 penicillin-binding protein

MFKQRLSKLLSSTLVLSMLFTAAPNITFADNTKDNSEKYQSSDIELHDYSKNAESYTKTKALAKEKIQTLLSKYGAVSAQYALIDNGKIEISGNGGVYSKQDNKNLNKDNMYSIASISKMFTTTAVMKLVDDGKLNLDTPVVKYIPEFKMADDRYKEITPRMLLNHSSGLMGSSFKNTILLADNDSYGHDNFLKELQKQRLKAKPGAFSVYCNDGFTLAEILVERVSGMSFTNFLDKYINNPLNLQNTKTTENSFDSSKLAKAYVPYWEDAVPQDNLNAIGAGGLYSSAENLCTFAQTFMKNSNGILSPASVKAMENKEYLNGLWPEGEDSILGYGLGWDCVNTYPFNQYNLKALTKGGDSLLFHSNLIVLPDENMAVAVLSSGGSSQLNEIIGQEILLSALKEKGKIKEIKPDKTFSKPQQVKMPSSLKENSGLYASSNMIKVDVNDNGTLTVSSPYIENGPEDKYVYIGQDRFVSEKGNSCLKFVKEKNNITYLNMSSYDDVPGLGQTASLYYVAQKVDDNNISNSVKEVWKKRSGKGYYLVDEKYTSQSYMFGSVKASFSLSDETPGYIVNTKIMDENNSNAFIEIPGVIGRDLSDIKLHKENGTEYLSFGTLTYVSEDSITNLPAEKSFTCELESNGYAKWYKIGDDIANKKIEVNLPQNSAFAVYDDKGVPVNYSLVTKNNRVRLPKGGVIVFLGSPNARFEVTYQDEVNASALTGTDRYETSIKISQAGWENAENAVLINDSAIADALAATPFAYKKNAPILLTGSSQINEKTLAELKRLKVKNVYVVGGEASINEKSLDTIKSNNISVSRISGSDRYQTSMNIAKELNNISNISKISVVNGEKGLADAVSIGAVSAQNDMPIILTNENSNITEINNVFKNKKIDKSYVIGGEYTVSKNIESKLQNPQRISGSTRNETNAKVIKEFYKDSKIDNLYVAKNGMNKQDDLIDGLSVGVLAGKTKSPVMLVGNSLDYNQKELFKTMRFKSVTQIGGNGNENSFKQIKEIA*

>CD630_14790 Clostridioides_difficile_630_NC_009089 ferrous iron transport protein FeoB

MSIKIGLIGNPNCGKTTMFNGLTGSSQYVGNWPGVTVEKKGGKLKGNKDVEIVDLPGIYSLSPYTLEEVVTRNFMLDDKPDAVINIVDASNIERNLYLTTQVLELGIPTVIALNMMDIVNKNGDKINIKELSEVIGCPVVEVTAVKGQGIMEAAEKAVELASSNNKLNFKLPFVDESKDAIEKIEKIIEEKTPYIDVETRWLAIKLFERDENVIQKLNISKTILNSIEEITRNCEDELDDDSESIITANRYEFISSIISSIIKKNRKGKETVSDKIDKIVTNRILALPIFALIMWGVYYIAVSSLGTIATDWTNDVLFGEIIQGNVSNFLASLNVAEWLQGLVVDGLIGGVGAVLGFVPQIMLLFLLLSILEDCGYMSRVAFIMDRIFRKFGLSGKSFIPMLISSGCGVPGVMSTRTIENDRDRKMTIMLTTFIPCGAKIPIIALFAGALFGGASWVAPSMYFLGIAMIIICGIILKKTSLFAGEPSPFVMELPQYHIPSAKGVLIHMWDRGKAFIIKAGTIIFVACGVIWFLQSFNWSLQMVDAGDSILASLGNIVAPIFAPLGFGNWQSSVATVTGLVAKENVVGTFGVLFGISDATEQDPTLLASVASMFTVASAFAFMAFNMLCAPCFAAIGAIKREMGSWKWTWITLGFQTLTAYIIALLINQVGSLVLGTGGSIAGAIISIFIAVAVVFVVLTYSNKNMKKEKMGKLSYMKN*

>CD630_14830 Clostridioides_difficile_630_NC_009089 sulfonate family ABC transporter ATP-binding protein

MVRSGFLLENIYKKYLVDNKEHLVLDNISLNISSEEITVILGESGCGKTTLLRILAGLENATSGNIYFFNNDKKCTPKVGMVFQESRLMPWLNVSENILLHTEKDNRNKVDLDKYLKMMKLEKFKNSYPNELSGGMAHRVSIARALSFNPDILLMDEPFAALDYFTRRKMQKEVVNIHKNTKKGVVFVTHNIEEAMEIAKKIIVFSKNKRIKQFSVEDEYNRDLTKNYYINLKKEILRELGEF*

>CD630_14890 Clostridioides_difficile_630_NC_009089 methionine ABC transporter ATP-binding protein

MISIKNVNKYYGKIQVLKDVSIEIESGEIFGIIGHSGAGKSTLLRCINGLEEYQEGSVLVSDKEVKSLNEKQMRDLRKELGMIFQHFSLLERKTVFDNVALPLECFGYSKAEIKKRVLELLEVVGISEKKNDKPRNLSGGQKQRVAIARALALNPQVLLCDEATSALDPNTTKSILSLLEDINKKLGITIIVVTHQMEVIKQICGRVAIMENGEVLEVGDTEEIFLRNTKGLRKLIGEESIILPKGTNIKILFPKDISNEAIITTMARELNIDVSIIFGKLEQFKDDILGSLIINISDKSGEQVKQYLTSKGIRWEEMINE*

>CD630_14950 Clostridioides_difficile_630_NC_009089 pyrroline-5-carboxylate reductase

VKKIGFIGAGNMASAMIGGIVNSKLVEPNMVIASAYSQGTLDRIDANFGINTTKDSKEVTRTSDIVIVAVKPDIYDDILEEIKDFIDDNKIIVTIAAGKSIKDIESIIGEDKKIVRTMPNTPALVNEAMSSLSINKNINKEDLEAVTEVFNSFGNTEVVPEYLIEAVIGASGSAPAYVFLFIEAIADAAVIAGMPRPQAYKFASQAVMGSAKMVLETGKHPGELKDMVCSPGGTTIEAVKVLEEEGFRASVIKAICACIEKSKKMSE*

>CD630_15020 Clostridioides_difficile_630_NC_009089 2-deoxyribose-5-phosphate aldolase

MKHILKTVDHTILKATTTWEDIKILCDEAVDMSVASVCIPPSYVKRASEYLKGKIKICTVIGFPLGYQTTATKVFEAKDAIENGADEVDMVVNISDIKNKDYDNIGKEIKEIKKAIGDKVLKVIIETCYLDEDEKIKMCEIVTMSGSDFIKTSTGMGTGGATLEDIKLMKEHVGKNVKIKAAGGVKSISDAEKFIEAGAERLGTSSICKILKNEDTTDY*

>CD630_15120 Clostridioides_difficile_630_NC_009089 pantothenate synthetase

MLVKEIKLLRNIIKDWRKHGYSIGLVTTMGFLHEGHQSLIKKAVKENDKVVVSVFVNPTQFGPNEDFNSYPRDIDKDFKYCMDSGATVVFNPSPEEMYLKGNCTTINVSGLTDFLCGAKRPVHFGGVCLVVSKFLNIVTPDKAYFGEKDAQQLAVIKRMVKDLNIDTEIIGCPIIRENDGLAKSSRNTYLSEEERKSALILNKSLSLAKEELVKGNLNPENIKELITAKINSEHLAKIDYVEIVDSETLQPVKQIEHSILVAIAVFIGKTRLIDNFTFELNI*

>CD630_15210 Clostridioides_difficile_630_NC_009089 tyrosine--tRNA ligase

MKSIDEQMRIIMKGVDDLIDEKELREKLIKSEKEGKPMIVKLGLDPSAPDIHLGHTVVLRKMKQLQDLGHQIVIIIGDFTGKIGDPTGKSKARKALTTEQVLANAKTYEEQIFKVLDKEKTIVRFNSEWLAKLNFEDVIKLAATITVARMLEREDFKKRYEGQMPISVHEFFYPLMQAYDSIALEADIELGGTDQRFNLLMGRSLQREFGMESQIVIMMPLIEGLDGKEKMSKSLGNYIGIDEEAGIMYQKSMEIPDELIIKYYNLVTDVHPDEVNKIESQLKEGSVNPRDIKMNLAREIVTLYHGEESAKEAEERFKSVFQKGQIPEDIQTIQVKEDGFDLIEVLVSNEIVKSKSEVRRLASQGGVKVNGEKVEDLSTIVKESELVVQIGKKKFVKIELVK*

>CD630_15280 Clostridioides_difficile_630_NC_009089 ABC transporter ATP-binding protein

MLIKLENIQKYYKVGKDELHVLKSLNLEIESGEFVMIMGKSGSGKTTLLNILGFLDVFDEGRYIFDGTDVTNLSENERSVFRNINIGFVFQQFNLIETLNVYQNVELPLIYNKALKKSNREEIVKDKLSSVGLLDKLKQKPLQLSGGQQQRVAIARCLANDPQIIFADEPTGALDSETSREIMELLTRLNKQGKTIIMVTHDQDLTKYATKVIRLKDGVFTSEV*

>CD630_15320 Clostridioides_difficile_630_NC_009089 ABC transporter ATP-binding protein

MEEILSVENIKKEYGRKGSKHEALRGITFKVYKGEFVGIMGSSGAGKSTLLNIISTIDLPSSGDIYINGKNTIKMKQNELADFRRDNLGFVFQDSNLLDTLTIKENIMLPLSLKNERVSVIENRIKEISKELNIESILDKYPGEVSGGQKQRGAVCRAIATKPSLVLADEPTGALDSKSARDLLNCLLKLNKDSNKTILMVTHDAISASFCNRILFIKDGIIFTEIVKGESNREFYNKIVNTVSLIGGVNKNDFI*

>CD630_15370 Clostridioides_difficile_630_NC_009089 oxidoreductase

MDAKKVKVPVREQEPAVRATNFDEVCLGYNKEEAMAEANRCLACKKPKCVGGCPVGIDIPGFITKIKEDDIEGAAKVIAKSSSLPAVCGRVCPQESQCEGVCILGIKSDAVSIGKLERFVADWSKENDINLSDTEPKKNQKVAVIGSGPAGLACAGDLAKKGYDVTIFEAMHEPGGVLTYGIPEFRLPKQAVVQPEIDNIRKLGVKIETNVIVGKTITVDELIEDEGFEAIFIGSGAGLPMFMNIPGENANGVFSANEFLTRVNLMKAYRDDYDTPISSGKKVAVVGGGNVAMDAARTALRLGSESYIVYRRSEKELPARAEEVHHAKEEGIIFNTLTNPKEILVDENGYVKGMVCIRMELGEPDDSGRRRPIEIEGSEFVLDVDTVIMSLGTSPNPLISSTTKSLDINKKRCLITDENGQTSKEGVFAGGDAVTGAATVISAMGAGKTAAASIDEYLKAKVNA*

>CDIF1296T_01612 Clostridioides_difficile_ATCC_9689__DSM_1296_strain_DSM1296_CP011968 ABC transporter ATP-binding protein

MVKVVYSRYYGIVVKIKNYGEYKMNILSIRNISKTYCGNIPFKALDKVSLNIEKGEFVSVMGPSGSGKSTLLNIISTVDRQSEGEVVLDGYDVSKLKGEKLAEFRRKQLGFVFQDFNLIDTLTVGENIMLPLTLEGESIKDMNIQTKSISKFLGIDKILDRKTYEISGGQAQRCAIARAIINKPAILLADEPTGNLDSKSTDDVLKLFTRINKEQKVTTLMVTHEAYSASYSDRVIFIKDGCIYTEIKKSESSNSFYSDILAVLSQIGGVR*

>CD630_15490 Clostridioides_difficile_630_NC_009089 histidinol-phosphate aminotransferase

LREKESIRELRGYEPNHVNCKVKLDANEGSKRLFKYLIKEISDSDIDLNLYPEDSYSDLKESIIDYINISGVNKKNLLVGNGSSEIIDLIIHTFVDKDEVILSFSPSFSMYSIYSQINGSKFIGVESDENLVINIDNVIEKVKENNPKIVIVCNPNNPTGTILKREEIIKLLDSTNSLVVLDEAYMDFGEESMLSDVFKYDNLIVLRTLSKAFGLAGIRTGYMLSNSSLINSVEKVRPPYNLNSLSDFIATRALRNKDVVKAYIKEVKEEREVLYEEMIGMGIKAYKSQANFILFYSEIENLSQKLIDRGVLIRKFGGKLENYYRVTIGDKEENSMFVGAIRDILKKEK*

>CD630_15660 Clostridioides_difficile_630_NC_009089 acetolactate synthase large subunit

VRMNGAKVILECLKKEGIDTIFGYPGGAVIPLYDALYDYSDDFKHIRTSHEQGLVHAADGYARSTNTVGVCFTTSGPGATNAITGIATAFMDSSPMVVISGQVPTSLLGKDSFQEIDITGATLSMTKHNYLVRNTKELVPTIKEAFRVANSGRKGPVLVDVPKDLFLAEMDFSGEDYDLCQIDDYMDYKSDFDLDDETNIKLLNEAIDIIKESKKPVIYAGGGVKSSDSEEILEKFATKIDTPVLNTLMGLGNIDRKNELSLGMVGMHGSRESNLALSNSDLVIAIGARFSDRVISKSSEFAKNAKIIHIDIDPSEISKNIESNVSLVGDVKLVLSLLIERVESKNNSNWKEEIKRFRKSEGVQTYEFHPQNILKKINEKYETLKKPTVVVTDVGQHQMWAAKYWNFKGNKSFITSAGLGTMGFGLGAAIGTKVGNVDKNVVLVTGDGSFRMNCNELATVANYNVPMLILLLNNRTLGMVRQWQKLFSNQRYSQTDINENVDYVKLVNAYNIDGYKVSSMEELGKALDMIDFNKPVFLQCDIDKDYDVYPIVAPNDALENLICN*

>CD630_15790 Clostridioides_difficile_630_NC_009089 sporulation-associated two-component sensor histidine kinase

MDTHNKYVNFIKNIPVPFLYCRIVKRQEDIEYRVEYISKGMGKVLQLEEGICDKNILDVLPVFKSKKYFKELFSNEVDCIKRYIPTLKNWINIKKQIIGDSYIILYFGKIVFDYRQIIDSFDKKEKVAYIKDEEGIYIDCSENLIPILNNNIKTTKDIFGKNDIEVWGENTGKLFRDDYREGVSSKKRFLQNLFEYEETFFMVEKYFLYDEDELLGTIGIVDNIIYSGYSNRNYNSKDLMKMIEHSIPENMFYKDVYGNYIGFNSGFLNLACMNKEELLGKNSYKISEEEALIDKIFESDKGVVENKKVVTFELNISMNDENKCIEITKRPFFDSYGSVIGIIGTARDISRRKRLEEEMDKTRMEFFANLSHELRTPINLISSSLQVIEKKEADLIESNDTLKRNLGIIKQNGNRILRLVNNVIDFTKMQSGYLDFKPEESDIIAFIEEICMSVADFASQNNIQLTFDTEIEEFSMLFDSEKLERIILNLLSNGIKYNKKDGKINIFLYVKDNVFNMKISDSGIGIPKEKIDKIFNRFEQIDNELSYRVKGSGIGLSLVKSLVELHEGSISLKSQLGIGSEFIVSLPVRSKNNIEKYNHKREISNELSKKLEIEFSDL*

>CD630_16230 Clostridioides_difficile_630_NC_009089 oxidoreductase

MSLKSLKIKENLYWVGSLDPDLRVFDIIMYTPYGTTYNSYVLKGTEKTVLFETVKDKHFDNYIERLNDLNIDFEKIDYIVVSHTEPDHAGSVEKLLDLAKNAKVVASETAIKYLKEIVNKDFEYVAVTDGDTLSIGDKTLEFFSVPMLHWPDTIYTYIKEDKTLVTCDSFGSHYSNDKIVNTLDENEEKDYLDALRYYYDCIMGPFKPSMVTAIEKIKDLDIDTVCPGHGPVLTENPRKIIDLYYNWSVNEQIKLEKEVTICYVSAHGYTKIMAEAIKAYIEKNSNYKVNLFDVIEHKQEDILAKIAVSQGVLFGTPTILGDALKPIWDILISLNPVLHGGKVASVFGSYGWSGEGIENAMERISQLRMTAVKPFAVNFKPSNEEIDKLHSYTGKFLDKLNSTFGSKKKTKKFKCVICNEVFEGDSAPSVCPVCGAKEDQFIEVEEDEVTFRKDTDEYFVIVGNGAAGFYAADAIRKRNKTCKITMISNEDELTYYRPALSDGINEELGSDFYMEDKDWYDKNNIVVILGTNVDKLDEVNKTIIVNDGAIKFDKLVIATGSRNFIPPIKGHDLENVFTLRNIKDLYSVKEALEKSKKVVVIGGGLLGLEAAWEFRLKGLEVVVVEAMDSILSKQLDKEGSKILEQCVRDTGIDVRLGVAVDGIEGDVKAQKVVFKDGDSVDCDMVVFSIGVRANTQMVQDTSVKIDRGIVVDKTLQTNVKDIYACGDVAQVGNISLAIWPSSVEMGKIAGANASGDNLTFESEVYPVSLDAMNVKVFSIGNIQNFDKEISSKDEGQRIYKKLFMKDGSLVGAILINDLSCTVKLIRLISEKGDFEDIMKADIL*

>CD630_16310 Clostridioides_difficile_630_NC_009089 superoxide dismutase

MKKKILIPVIMSLFIISQCITSFAFTPENNKFKVKPLPYAYDALEPYIDKETMKLHHDKHYQAYVDKLNAALEKYPELYNYSLCELLQNLDSLPKDIATTVRNNAGGAYNHKFFFDIMTPEKTIPSESLKEAIDRDFGSFEKFKQEFQKSALDVFGSGWAWLVATKDGKLSIMTTPNQDSPVSKNLTPIIGLDVWEHAYYLKYQNRRNEYIDNWFNVVNWNGALENYKNLKSQD*

>CD630_16490 Clostridioides_difficile_630_NC_009089 iron family ABC transporter ATP-binding protein

MIEIKNIFKRYKNKNVVDDVSFSIEKGKITSFIGPNGAGKSTVLSIVTRLIGGDGGEVIIEGKSLTNYSNKELAKKIAILKQSNNITLKLTIRELVGFGRFPYSEGNLTKEDENYIDEAIEYMKLTDIQHKYLDELSGGQRQRAYIAMVIAQDTEYILLDEPLNNLDMNHSVQMMKVLRSLCDELDKTIVLVMHDINFASCYSDNIVALKNGKVEKVGRTDEIVNEKVLEDIYEMNFNIKNINGNRICIYF*

>CD630_16550 Clostridioides_difficile_630_NC_009089 Na+/H+ antiporter NhaC-like portein

MRNKKINIIFLTTIMFIMSTVMVFAEEDIDTIALANAEKFGILTLIPPLVAIILAFITKNVIISLLIGILSGSFIIKASGINVFATFIQAFLDLVDRALVSLADPWNAGIILQVLAIGGVINLVAKMGGAKAIAEALAKRAKSAKGTQLITWFLGLLVFFDDYANSLIVGPMMRPVADKMKISREKLAFIIDATAAPVAGLAIISTWIGLEVGLIHDAFESISIDVDAFGIFLNTIPFRFYNILILAFIVISALLLKEFGPMRKAEIKSRSRKISIDLDEGVEELDDLAPKNGVKLSVWNAIIPIGTLIIVALASFYYSGYTSIMGGDDKALIQLFTNSPYSFEAIKEAFSASDASRALFQSALVASLVAIIMAVVKKIFTISEAIDVWIDGMKSLVITGVILILAWSLSSVIKELGTAKFLIHLLSGSLPPFLLPSLIFGLGAIISFATGTAYGTMGILMPLAIPLAYSLNPDMSYVIVSTSAVLTGAIFGDHCSPISDTTILSSMGAGCNHIDHVNTQMPYAIFTAVITIVFGYIPAGLGLPIYIVLPVAIAAIFVGIQIIGKKVDEAEIELVE*

>CD630_16570 Clostridioides_difficile_630_NC_009089 bifunctional glycine dehydrogenase/aminomethyl transferase

MNELKRVSLYNIHKELGAKLVEFAGWEMPLEYEGINKEHEKVRKSAGIFDVSHMGEVQIKGAESEKFIQNLVTNDISTLKINDIIYTPMCYENGGVVDDLLIYKFGEEDYLLVINAGNIDKDVAWIIKQSEGYNVDIKNISSEVSQLAIQGPKAEEILQKITDIDLNSIKFYKSIPSIIVCGCPCLVSRTGYTGEDGFEIYCKNKYVEIIWNEVLKVGGEDICPAGLGCRDTLRFEAALPLYGHEINEHISPIEGGLSIFVKTNKESFIGKSILSKEKESGAKRKLVGFEMQGKGMPRNGYDIRIGDKTVGFVTTGCASPTTGKILGMGIIDSEYAKVGNEIGIAIRKKVVPAVIVKKPFYKKQYKKDNIILNKENKFSYIPATSEDKSKMLKVVGLNSVDELFSDIPEEVKLKRDLNLEIGKSELEVSKIVKRLSEENLSLEDLTCFLGAGAYDHYIPSIIKHITSRSEFYTAYTPYQAEISQGTLQVVFEFQSMIAEITGMEIANASMYDGATAAIEACIMAMNQTRKSKIVVSKTIHPETLSVLRTYLQYKDCEIVEIDFCNEYGTTDIEKLKASVDKDTACVLIQTPNFFGIIEEMEEIEKITHENKAMLIMSVDPISLGVLKTPGEIGADIVVGEAQSLGNPLNFGGPYVGFLASKSKYTRKMPGRIVGQSLDVEGKIAYVLTLQTREQHVRREKATSNICSNQALNALVASIYMATMGKEGFKEVGMQSMKKAHYTYNKLVQTGKYKPIFKGKFFKEFAVQGNLNIETINDKLLEENILGGYNLEYNYPELKNSTLLCVTEKRSKEEIDKLVGIMEGL*

>CD630_16720 Clostridioides_difficile_630_NC_009089 two-component sensor histidine kinase

MKWKITRNFIFTIVFVAISVVIINIISILYVISTNSFFKVVDSGNNPEEFARSFEKDLYEKDGEFKLSKIGAEKLEKSNSWIQVLNDLGEEVYGVNVPKDTPKKYTPFQMVNNYKYIETKYVNFVLEKHLNKKHLNIIVGIPSRDISRIILTYSQNNIKKTLNKVIIITLVIDSVVALGVGYLFSRKLTKPISSVLWSIETMANGNYSLYLKDRGIYEEVFKNINMLADTLRVNEVERKENEELREEWLANITHDIKTPLASIQGYAEIINDKDYEFEEDEIQEYTEIIYNKSKYIKDLVDDLNLSTRLKNNTIVLDKKKINLVSLVRNIIIDILNDNRYKNRNIEFESNEDLIEVYIDSILFRRAITNLIFNSIVHNSEGTLISVEIVKKDNIEIIIKDNGIGISKSDLKHIFKKYYRGTNTGEMHKGSGLGMAISKEIIEIHKGKIYVSSEIGIGTKIIIEIKQN*

>CD630_16910 Clostridioides_difficile_630_NC_009089 thioredoxin-disulfide reductase

VSIMRYDIAIIGSGPAGLSAAINAKIRNKTIIMFGNDNLSNKLVKAPSIDNYLGFYDISGDELKDKFKSHIDSMDISIENKRINNIYAMGEYFTIMSGNDMYEATTVILATGVEYTRPIKGEEEFLGRGVGYCATCDAPLYRNKKVAVIGYNEESKEEANFLSELTSKTYFIPMYKKDNLMRSSDNLDDSIEVIHDRPVQIDGDKLVNKVSFKENHIEVDGVFVIKDSTAPSALVPGIEIDGIHIKVDNNMKTSIDGCFAAGDCVGKPYSYIKAAGQGQIAALNAVYYLDKLKRA*

>CD630_16950 Clostridioides_difficile_630_NC_009089 symporter protein

MEFQQIIAICIFLIVMAAIITEKVNRSVAAVGGALLMIIFNILTLDEGLSHIDFNTIGVLVGMMLFVAVVKNSGLFEYIAIWTAKKAKGDPWKIMICFAIITAILSAVLDNVTTVLLIGPMTIVITQILGLNPVPFLITQILASNIGGTATLIGDPPNIMIGSAANLSFMDFVINLGPAVIVILAITIICFRFIYGKELVVNERAKNAILKLDEKKSVKDKPLLIKSLILIAFILFGFMFHSTIHIDSSVVALTGASIMLLIGKQDVDEIMAGIEWSTILFFMGLFVVVGGLVEVGIINKLAQALIGLTEGHLVFTMLLILWLSAIVSSFLDNIPFVATLIPLILTMQAEGIDVMPLWWATSLGACLGGNGTLIGASANVVLAGIGNKHGHPISFKEYFKIGFPLMIISIIISTVYLIIKF*

>CD630_17000 Clostridioides_difficile_630_NC_009089 riboflavin biosynthesis bifunctional diaminohydroxyphosphoribosylaminopyrimidine deaminase /5-amino-6-(5-phosphoribosylamino)uracil reductase

VNFVNQKEKDIYYMKKAIELAKNGEGFVNPNPLVGCVIVKDSNIIGKGYHEKFGSNHAEVNAINSAKQSLKDSTLYVNLEPCSHYGKTPPCVDKIIQNKIKRVVISTLDPNPLVCGNGVKKLKDNNIDVTVGILEDEARDLNEAFFYHIKNKRPLCIVKSAVSLDGKIATKSLESKWISNESSRYLTHKYRNKYQSIMVGINTVLNDNPLLTCRLNQEKVSHPTRIVIDTHLKLPLNSNLVKDKTSKTIVFTCCKESIKLSMLKENNVETIISPSKNNLVDLEFVMYKLGELNIDSVLVEGGATLNDSLFRNKLVDKVKLFLSPKIIGGKDAPTFVSGEGINHLSDSTQLTINNVTLIDGDILIESDVLN*

>CDIF1296T_01789 Clostridioides_difficile_ATCC_9689__DSM_1296_strain_DSM1296_CP011968 thiamine biosynthesis protein ThiC

MLGEICLSVYTIIRVLSKRGKALFLFSKTITLILEDCNMNYTTQMDAARKGIITKEMEIVSQKEQVDVNELRELIANGQVVIPANKNHKSLSAEGVGKNLRTKINVNLGISRDCKDIEKELEKVRVAIDMKAEAIMDLSNYGKTREFREKVVEMSPAMIGSVPMYDAVGYLEKELKDITEEEFLNVIRQHAIDGVDFITIHAGLTRSVCQKIKNHERLTHIVSRGGSLLFAWMELNNKENPIYTNFDKILDICEEYDVTLSLGDACRPGCIKDSTDGVQIQELVVLGELTKRAWERNVQVMIEGPGHMAIDEIEANVVLEKRLCHGAPFYVLGPLVTDIAPGYDHITSAIGGALACAKGVDFLCYVTPAEHLRLPNLDDMKEGIIAAKIAAHAGDIAKNVKGAREWDNKMSKARADLDWCEMFRLAIDPEKAKRYRDESTPTHEDSCTMCGKMCSMRTVKKILNNEELNLI*

>CD630_17230 Clostridioides_difficile_630_NC_009089 acyltransferase

MNNYLKFATYQLIGFTSSIPRLIKIKKNPDKFSLKEKFEFMQKQAKKSLDIVNIELNIIGKETLPKEPLLFVVNHSSMLDSFILTASVERPIGCVIADEPVWRNIPIFKEWAKLLRCVYVNRKNNREGIKSIAQASQNILTGQSMAVFPEGDLTWIKEPNSLVSEFRSGALKIAYKAKCPIVPLVIKNSKDTYEGYQPIGKINSVPVEVEFLEPIYDHIENPRLKSSVLGENIKNKMINTIENFRKSNKTFKEF*

>CD630_17300 Clostridioides_difficile_630_NC_009089 ATP-binding protein

MADCNSCPSKGNCNSQSNCSIENNPNNKFGKIIGVMSGKGGVGKSTVTALLANKLNKMGYKVGILDSDITGPSIPRLMGVKNVKAYSDGSYIYPVENSNNIKVMSINLMIDDENEPVVWRGPLLGGVVKQFYTDVLWEELDYLLIDMPPGTGDVALTVMQSIPISGIVMVSVPQDLVSMIVSKAVNMAKKMNINVLGVIENMSYIQCPDCSKKIKLFEGESTEKFLDDLDLELLGELPMTKEIIDITHNGVTEISDDLDSILTNVVEKIK*

>CD630_17530 Clostridioides_difficile_630_NC_009089 multidrug family ABC transporter ATP-binding protein

MIKVDDLSFSYTDRDFLQNINFEVGKGEILGFLGPSGAGKSTLQKILIGMITNYGGSVIVNGVESKRHSNKFYENIGVDFEFPSLYEKLTAIENLKYFGSLYSKKLLSIDELLKSVGLENESNKRVSEYSKGMKSRLNFIKALLHNPDILFLDEPTSGLDPSNSKVMKDIILSEKSKGKTIILTTHNMLDATELCDRVAFIVNGKISALDTPHNLIMSKGAIKVRYTYFDNGEKTSECFLNNTANDKNLNMLIEKNKLLSIHSSEPTLNDIFIEITGRNLQ*

>CD630_17740 Clostridioides_difficile_630_NC_009089 amino acid family ABC transporter substrate-binding protein

MKNILKKVGIFTIMLGLLGGVVGCSKPDNEKDKDASKESKKEVVVGFDNTFVPMGFLDEKGDTVGFDVDLAKETFKRLGMEVKFQPIDWSMKETELNDSKTVDVLWNGYSITDERKKIVSYTEPYLQNKQIIVTLSDSKINSKADLKDKEVGTQQGSTALDAVEKDKDFMNSLKGGAPVLYDTYDKALRDLEIGRTSAVVGDEVLIRYYMGQKGEDKYKVLKDDFGLEDYVVATSKENPELCEKINETLKEMKKDGTFDKIYDKWFK*

>CD630_17760 Clostridioides_difficile_630_NC_009089 amino acid family ABC transporter ATP-binding protein

MLKIKNLNKSFKKNRVLKDISFELEEGQIGVLLGKSGAGKTTILRCINGLEEFDSGEIIIDNEVIKNKRDMAKIRGKIGMVFQNFNLFPHMTVLENIIESPVNVFKVPRKEAEERARELLRLVDLEDKLNSYPFELSGGQQQRVAIARSCALMPKVLCFDEPTSALDIDTIQRVVNIMNRLKDKGMTILIITHDVVFSNNVADKIISIKDGIVENVQIKEKIV*

>PCZ31_RS13230 Peptoclostridium_difficile_strain_Z31_NZ_CP013196 two-component sensor histidine kinase

IVLHFEGLRKKVIKNYFIIIIIMVTLFEGLFMFYIQNYYYDSVKQLLESEIKYADEYNAITMETTSFEKKVKNIFDKQPLTKNSEFGISIIDKDKNIILDQYGFKSKEKANYEDVNNALKDIKTKNLTPYTYRIPDTGEHVMSISLPLKVNNIIEGVVRYTVSLDAIDNAILKQATWLILAGIFILIIAILISLKFAETLIKPLRELKKFANELAVGNYNIKLEKMKIVDDEIGDLAQTFEHMAHEIDKSEKLKEEFISSVSHELRTPLTSIKGWSETLGYESITREELDLGLGIIQDETERLIKLVEELLDFSRLSSDRIKLHVDIVDVEGLIVGVVNQLKVKAAEKDISLLFEFENEFIENIQGDKNRLRQVLINLIQNSFKFTSQGGYIKVVASQDEEITTISVEDNGSGIEKQNLNKVLDKFFQEDYNKAGSGLGLAISNEIVKLHGGRMKIESEKNVGTKITFNIKNKFAKQA*

>CD630_18140 Clostridioides_difficile_630_NC_009089 phosphoglycerate mutase

MGNTFYIVRHGQTDWNILGKTQGHGNSDLTPQGIEQAKELSEDIGKYSIDYIFSSDLGRAMQTAQILGDKLNIEVQKTEALREMGFGVWEGLLIKEIQKDYSDIYATWRNEPHLVNIPEGETLKIIKERVDAFIKELNEKYDNKNIILVTHSITLRVMLLSFLESGMENIYRIKQDNTALNIVEFKDYGPVIVKMNDTSHIKNHVKINNSALE*

>CD630_18160 Clostridioides_difficile_630_NC_009089 cytidylate kinase

MGNLVIAVDGPAGAGKSTIAKIVAKKLNINYIDTGAMYRAVTYKCLKSGIDVNNEKEVIQIAENSDIDFKDNNIYLDKEVINEEIRTIEVSNNVSNVAKIKEVRQLMVEVQRKIGMKNSVILDGRDIGSYVFPDADYKFFLVATPEERGNRRYKELCNKGYNTTLEEVIEDIIRRDEIDSNREFAPLVKANDALEIDTTGKTIEEVVEEVVSKINL*

>QAE_RS0209090 Clostridioides_difficile_QCD_23m63_NZ_CM000660 cation transporter

MYILNLNTRETIEDFRDKFYVAENSYLILSAPKNLKLLKETLDIDEITFNDCLKFDEITKLDLFDNYDFLSLNTFELRDGEAVIEEVNMYLSDNFILVVVNEEHFLFEFVKNIILKNSQLEKNPVINLFKINYLILREVIKNGFESLEKVEELILQIEDEMMDNINKNHVSRISDVRGLTRIIVKNTRPLLYIGDRIVKENIRYLKYSNVKKYNLENFQGIDFGIDKLYSFALSTRELADKLLDIYSSRVGEKTNNLITKLTLLTAISAPLTIITGIYGMNFRYMPELNWIYGYPATLFFMLCIIFVGIIIFKIKNYCKLQMIFN*

>CD630_18350 Clostridioides_difficile_630_NC_009089 chorismate synthase

MSGIWGNNLKVSIFGESHGNAIGINIDGLPSGIELDLDKIDKEMKRRAPGKNSISTSRNESDIPEILSGYFNGRTTGTPLCAIIRNSDTRSKDYGELKNLMRPGHADFTGNVRYSGFNDYRGGGHFSGRITAPLVFCGAICKQILSQKGIEIGAHIKKIKNIEDMSFDYVNISKQQLSNLQTLELPLLDLSKEEAMKNTIIDAKNQGDSVGGIIECAVVGINVGLGNPFFDSVESTLSHLLFSVPAVKGVEFGLGFELADMYGSQSNDEMYYEGNQVKSKTNNNGGIIGGITTGMPIIFKVAIKPTPSISRQQNTVNIKDKKDDILYIKGRHDPCIVQRAIPVIEAVTAIGIFDLMKGR*

>CD630_18380 Clostridioides_difficile_630_NC_009089 shikimate kinase

MINKTKEKLILIGMPGSGKTTIGKLLAKEYNCSFCDMDDYIIQISQKSIAELFSEGEDIFRNYETQACRELSISDKTVISTGGGVIKKDVNMEILKETGIIIFIDRPIQKILEDININSRPLLKNGKDRLYNLYNERINLYKKFSDIEILNDKSLNNAVYNITNAVSENFKFDFKEK*

>CD630_18990 Clostridioides_difficile_630_NC_009089 dCMP deaminase

LNKKLENRCSWQEYFMRLCETVAERGTCDRAYVGAIIVNSENRIVSTGYNGSISGDKHCSEVGHEMRDGHCIRTIHAEQNALYYCAKEGISVKDCSIYVTHFPCLNCTKAIIQAGIKHIYYRTGYRIDEYAIKLLQSSNVLYTKL*

>CD630_19380 Clostridioides_difficile_630_NC_009089 acetyl-CoA carboxylase biotin carboxylase subunit

LIKKILVANRGDIAVRIIRTCKELGIKTVAIYSEIDKDCFHRYIADESICIGPNNISKSYNNIENIIYLALKLKCDAIHPGFGFLSENPEFAKQCEDNNIIFIGPTREQMILMGDKSRARETMMELNIPVVPGSESVLKTKEEALEVAREIGYPVMIKASSGGGGKGMRIVRKEEELFSNFDMASSEALAAFSNSDLYMEKFIENPRHIEVQVFGDKHSNAIHLGDRDCSMQRRNQKVIEESLSPYLSDEERQKLHKIAVDIVKGVGYIGAGTIEFIVDKDKNFYFIEMNTRIQVEHPVTEMVTNLDLIKLQISIANGDKIPFKQEDITFRGHAIECRINAEDSSKNFAPSPGKIESLNLPGGFGVRFDTFVYAGYTIPPLYDSMIGKLICWAETREECISRIYRALDEIIVEGINTNVEFQKALVTSEEFREDTHHTKFIEDVFMKKEFATL*

>CD630_19390 Clostridioides_difficile_630_NC_009089 acetyl-CoA carboxylase, biotin carboxyl carrier protein

MNINEIKELLKAIDSTNLEYVKLESSDLRLEVSKKAQSTSSPVLSVQQESVVDLSLEKPVVNDTPVTSNENLSVVVAPLMGTFYDSPSPDADSFVKVGDVVEEGDTLCILEAMKLMNEITSEIKGEIIEVLVSNEELVEYNQPLFKIKPL*

>CD630_19420 Clostridioides_difficile_630_NC_009089 transfer RNA (guanine-N(7)-)-methyltransferase

VRRRRKKGADEKLLSYTKYVLRDDIDKLKGKWNLKFRNDNPIHVEFGTGKGKFITTLAKQNPDINYIAMELKEEVLLKAVEKADASNLNNILFLWGDVSNILDYFEAKELSRIYINFCDPWPKNRWSKRRLTHSGFLEMYNRVLEDDGEIHFKTDNEKLFEFSLNEIAANNWLLKNISLDLGNSEYENNVTTEYEDKFMSQGMRIFRCEAKKRN*

>CD630_19470 Clostridioides_difficile_630_NC_009089 ABC transporter ATP-binding protein

MKITHNSLAVEAKNIIKEYKIGNTTTRVLKEVSLQVMKGEFVSIMGQSGSGKSTLLYILGGLDTPTSGKVYMNGADISHFNDEKMSIIRRRNIGFVFQFYNLIPNLNVEENIMLPLLLDGKNLKDYKNQLDEILDIVGLTDRRKHTPRELSGGQQQRVAIARALIGKPEILFADEPTGNLDSKTGIEIIDLLNKINRDNGQTIIMVTHSPEAAKSSSRTITVSDGLIV*

>CD630_19520 Clostridioides_difficile_630_NC_009089 LysR family transcriptional regulator

MNIKLELYKVFNAVVNNKSFSLAAKELFMSQPAVSQSIKQLEEQLDTLLFYRNNKGVKLTPEGKILSEHVTTALKLISSGEDRINKFKKLEYGSLKIGVGDTAARFFLLKYLEIFHKKYPHIHVSTINRTSRELISLLKDGNIDIAIINMPIEDDTLNIVECIEIHDIFVCANDYIEYKGRKISLEELNTLPLIMLENKANSRLYVNEYFLSKGIKLNPDIELGSHELLLEFAYINLGVSCVIEEFSIDYLENEKLFKLDIKEPIPKRNIGYCHLKDISLSLATKEFLSMISNNI*

>CD630_19550 Clostridioides_difficile_630_NC_009089 ABC transporter ATP-binding protein

MSILVTNNLVKHYGKGETKVKALNGVSIEIERDTFTAIVGTSGSGKSTLLNIIGGLDNPTSGDVIIKGKNISKIGKKDLTVFRRRNIGFIFQNYSLMPVLNTYDNIALPVTLDKGNHIDHEYIEMLMKTLGIWDKRLKFPSELSGGQQQRVAIARALANKPALILADEPTGNLDSKTTMEVVCLLKESSAKFHQTILMVTHNENIAQVCDSIIHIEDGVVVNTGGEVL*

>CD630_19760 Clostridioides_difficile_630_NC_009089 DNA mismatch repair protein MutL

MKNIINILDDLTINKIAAGEVVERPSSVVKELIENSIDAGANKISIDIIDGGKSLIKITDNGIGIPSSEVEKSFLRHATSKIKKIDDLYDLYSLGFRGEALASISAVSKLEMTTKTKDEIIGTKIYVEGGKIISKEPIGFTNGTTIIIKDIFFNTPARQKFLKSTHAETINISDLINKLAIGNPNIQFKYTNNNKQMLNTPGDGKLVNTIRSIYGKEITENIIDVEFKCNHFKMNGYIGNNNIYRSNKNLQHIYINKRFVKSKIIIDAITESYKSIIPIGKHAVCFLNIEVDPSCIDVNIHPNKLEIKFEKEQEVYIELRDFLKVKLIHSNLIGKYATYSDKKTQPRIAINSREKSTDYKLRNNDLLESTPKNSNITKGKDEVIEVVTLSSEKPINEFQSVSEVLNASVEDDVKNINYLSEDSANDNIQEEFQVDGIKNEGNYYLGDSIKDSEEEYLCSSKRKFSLYGYSVIGVVFNTYIILSKDDSMYLLDQHAAHERILYERYMEKFYRQDINMQILLDPVVIEVSNVDMLQIENNLELFMKFGFELEIFGNNHIMVRCVPTIFGVPETEKFILQIIDNIEEITSNYDLKGERFASMACRSAIKANDKIYDIEIKSLLEQLEKCENPFTCPHGRPIMVEISKTEIEKMFKRIM*

>CD630_20190 Clostridioides_difficile_630_NC_009089 drug resistance transporter

MTNSFKKDDNRKKITVLFVVIAMTFMATLDSSIINVALPVLASKLNVSLASIEWVIASYSIIICSTLLFFGRLGDIIGKSRVFQVGTILFTSASLLCGLSNSLTLLIVCRFIQGIGASAYMANNHGIITELFPKESRGKALGILVTAVAIGNMVGPSVGGFILSIFDWNVIFFINIPIGLIVIFLNTKFLPNSKKSSENMDKTGAILQFLGTTLFFSALISAQQTGLLNPYILIALLLSIIFIILFLILEKKHPQPLLDLEIFRNFKFSLNLICALTSFICIASSSILIPFYLQSTMKLPPIQAGLFMILSPLILAIFSPIFGNISDKIKSEKIILIGLLVMSFGFFLMSRLKESSALILFVIYILIISIGQAIFQPANNALIMSSCSRSKLGVVGSINSLVRNLGQVIGITISTTLLYNFMSIKAGYRVNDYVINNDKIFVFGMRNVYIIVTLVCLIGAILIGFYLFKYNKNEQ*

>CD630_20200 Clostridioides_difficile_630_NC_009089 chaperone protein

MDVEKMTLRVQKSLNEAYNEAVKNHNQQVDVIHLFSALINQEDGLIPNIIEKMNISIDSLRNTVNFEIDKLPKVYGEGADSQGVSATRKINEVLIKAESISKEFKDSYISVEHVMLAMMETESKSAVGKILKQYNINKNDFLNILSQVRGSQRVETQDPEGTYDALARYGTNLVDLAKKNKLDPVIGRDEEIRRIIRILSRRTKNNPVLIGEPGVGKTAIVEGLAERIVRGDVPEGLKDKIVFSLDMGALIAGAKYRGEFEERLKAVLKEVQSSDGKIILFIDEIHTIVGAGKTEGSMDAGNLIKPMLARGELNCIGATTFDEYRKYIEKDKALERRFQPVIAEEPTVEDTISILRGLKERFEIHHGVRIHDNAIVAAAKLSHRYIQDRFLPDKAIDLIDEAGAMIRSEIDSLPTELDVVRRKLFTLETEREALLKENDDKSKSRLDDIQKEIAELKSKNDEMTAKYEKEKSQILDIKNLKAQLDEAKGKAEKYEREYDFNKAAEVKYGEIPKLEEQIKQYEENMNDGSENSLLKEEVTEEEISSIVSKWTGIPVTKLVEGEREKLLKLEDELHKRVIGQDEAVTAVSNAVIRARAGLKDERKPIGSFIFLGPTGVGKTELAKTLARNLFDSEDNIIRIDMSEYMEKHAVSRLVGPPPGYVGYEEGGQLTEAVRRAPYSVILFDEIEKAHEDVFNMFLQILDDGRLTDNKGKTVDFKNTLIIMTSNIGSNYLLEAGGNITETTNNLVMNEMKHRFKPEFLNRVDDIIMFKPLDQENIKKIIDIFMKDLKNRLKEKDITIEVTNSAKDVMVREGYDPVYGARPLKRYIGNTLETIIAKKLIAGDVYNGCTIVIDGKDENIEVLVK*

>CD630_20240 Clostridioides_difficile_630_NC_009089 multidrug family ABC transporter ATP-binding protein

MISMDAITIKNLNKTYKDFSLQDISFSVPKGSVMGFVGENGAGKTTTLKAILNLISYDSGNIEIFGLDSKKNEKEIKEQIGVVFEGSNFHENLNTDHVSKIMSKIYKNWNDTLFKDYLKKLRVPDNKLIKEFSKGNKMKLSIAVALSHKPKLLILDEATSSLDPIVREEILDIFLDFIQDEEHSIILSSHITSDLDKIADYITFIHKGKIVFSENKDELIDTMGVLKCKPSDFDNLSREDYSYYRKSQFGYEVLLKDKHRFISRHPNCIVDNTSIEEIMLFYVRGDK*

>CD630_20300 Clostridioides_difficile_630_NC_009089 ornithine carbamoyltransferase

MNNISLKGKSFLTLKDFTKEEIRYLLTLSSYLKTKKKVGIKGDLLQGKNIALLFEKTSTRTRCSFEVAAHDEGAHVTYLGPNDCHMGKKESVSDTAKVLGRFYDGIEFRGFKQETVESLAKYSGVPVWNGLTDEYHPTQILADFLTVIENVDKDLNKVKFVYVGDARNNMGNSLMIGCAKMGIHFVALAPKELWPNEELVKEMKEIAKKSKGDIMLTENIDDVKGADVIYTDVWVSMGEEEQYEARINQLKDYQVNMDMIKKTKNENVIFLHCLPAFHDLETKVAQEIYEKFGLSELEVTNEVFNSKYSKVFEEAENRMHTIKAVMVSTMTDVNMNNNLI*

>CD630_20310 Clostridioides_difficile_630_NC_009089 acetylornithine aminotransferase

MNNDNTISKWNEYFIDTYNQPNFVIDYGEGSCFFDTNGNKYIDFTSGYGVSSLGYSNSNLKNALKEQVDKLLHTSNLYFNEPVLCSGEKIINSSGMAKVYFCNSGTEANETAFKIARKYSSDKYGNGRGTIISLKDSFHGRTMMSLMATGMDKYHKYFYPLPEGFKYVERNNIEDLKNNLDSTVCAIILEAIQGEGGVNVLEKDYVLEIVKICQEKDIVVIFDEVQCGIGRTGKLFGYEYFDVKPDIVTVAKGLGAGIPVGGVLVNKKLSKVLGKGDQGTTFGGNLLAMVAASVVLDEISKDGFYNEVLEKGNYIRKSIESFNNKVVLKTKGIGLMIGIETNIESSIIEEKARKKGLLILTAGKNVLRFLPPLTISYKEIDEALEILKDILLEIN*

>CD630_20480 Clostridioides_difficile_630_NC_009089 RpiR family transcriptional regulator

MEETNEMKDSKHLISNIQSQYTRLSKGQKLIAQYILNNYDKVAFMTACKLGETVGVSESTVVRFANALGYSGYPKLQAALQELIKNKLTTVQRVEMAHDYSDDFAILNKVLKSDIDNIRSTLEEIDERAFKEASNKLLRARKIYILGMRSSFVVAQYLGFYLDIILDNVHIIRMDMGDAFEQIVRINEEDVIVAISFPRYSKKSYQIVNYAKEKGAHVISLTDSLFAPVASLADNTLLVKSNMASFVDSLVPALSISNALAISVGMKEKEDIKQHFDDLEQIWKRYSVYE*

>CD630_20590 Clostridioides_difficile_630_NC_009089 glutamine--tRNA ligase

MSNETNSSNFIKNIIINDLETGKHDSIITRFPPEPNGYLHIGHAKSICLNFGLAKEFNGKANLRFDDTNPLKEDVEYVESIKEDVKWLGFDWNELNFASNYFDEMYKRALILIKKGKAYVCDLTQEEMREYRGTLTEPGKESPHRNRTIEENLDLFERMKNGEFKDGEKTLRAKIDMSSPNINLRDPIIYRISHSTHHNTGDKWCIYPMYAFAHPIEDAIEGITHSICTLEFEDQRPLYDWFVKECEMENIPRQIEFARLNINNTVMSKRKLKQLVDEGIVDGWDDPRVPTISGIRRKGYTAEALRNFCSEIGVSKVNSTVDSQMLDYFLRENLQPKAPLTMGVLRPLKLIITNYPEDKIEMLEIENNAKDESQGKRLVPFSRELYIEQDDFMEEPVKKYFRFFPGNEVRLKGAYFVKCTDVIKDENGNVVEIHGTYDPETKSGSGFTGRKVKSTIHWVDAKSAIPCEFRLFEPLILDDIPENEGKHFLEQINPNSLEILQGFVEPTQIKDAKPFDKFQFVRNGFFSIDNKYTTDEKFVFNRIVPLKSSFKPGK*

>CD630_20680 Clostridioides_difficile_630_NC_009089 ABC transporter ATP-binding protein

MLQVTGVGLRFGDKELFKDVNLKFTKGNCYGIIGANGAGKSTFLKILSGEIEPNTGSISITDKERMSVLKQDHFEYEEETVLNVVIRGHERLWNIMKEKDALYMKEDFNEEDGIKAAELEGEFAELDGWDAETNAEKILMGLGITKDMHYKQMKELVGGEKVKVLLAQSLFGKPEILLMDEPTNHLDFKSINWLNNFIMDLEESIVIIVSHDRHFLNQICTNIVDVDFGKIQMYVGNYDFWYESSQLALQLAKDQNKKTEEKIAQLKEFIARFSSNASKAKQATSRKKQLEKLEVEDIQPSRRRYPYVGFTPAREIGNEVLEVHNLTKTIDGVKVLDNVSFRLDRDDKVVFMGDEIATTALFNIVMGELEPDSGEYKWGVTTSQDYLPKNHNKFFDGVEYSLVDWLRQFSEEKSESFIRGFLGRMLFSGEEALKEAQVISGGEKVRCMLSKLMLSNANVLVLDDPTNHLDLESITSVNKGLEKFPGVLLFTSHDHEFISTIANRIIEITPNGIMDRKMDFDEYLESKEIQDQLAKMYGKDK*

>CD630_21060 Clostridioides_difficile_630_NC_009089 ATP-dependent RNA helicase

MNTFEQLKISSTLIDGLKKQDITSPTEVQSLVIGNIIQNKDLLINSQTGTGKTLAYLLPIFEKIDTSKRETQALILAPTHELVMQITNQVELLAKNAELSVTSLALIGEVNIQKQIKNIKAVKPHIVIGSCGRVLDLIKQKKLKSHNIKTIVLDEVDNLLNGKNITCIEDIIRTTLRDRQIIGCSASLTDSTIKICDKLMKEFEIIKTKEKSQINPNINHSYLLGEIRDKFTFLRKALAATNPKKAIVFVNNEKNIEVLVSKLNYHNYKAIGIFGNMEKEDRKNAINKFKLGKAKILITTDLSARGLDIVDVSHVFNLDFPKSKNEYLHRCGRTARGNRSGNTISIITKKELDIIKDLQKEFNIVITPKTLQNGELIDIIK*

>CD630_21160 Clostridioides_difficile_630_NC_009089 GTP-binding protein BipA

MSQKHKIINIAVIAHVDAGKSTLVDAFLSQSGVFRKNEVVKDCVMDSNDLEKERGITIYSKNCAINYEDYKINIVDTPGHSDFSSEVERVMKTVDTVILLVDASEGPMPQTRFVLQKSLEFGLKPILFINKIDKKDQRAEEVVNEVFDLFVDLNATDEQCEFPIIYGIAKQGIAKLEMDDDSEDLSPLFKTIVNHVEAYPNYDNEPLQFQISALAYDDYVGRLGIGRIYKGTLKNNTQVAICREDSVVSKGKVSKLSVYEGLKQVEVDEATSGEIVVIAGIPDISIGETICDLDSPLPMEMIKIEEPTLSMNFLVNDSPFVGKSGKFVTTRHLKDRLEKELEVNVGLKVEPLDTTDGYKVSGRGELHLSILLENMRREGYEVGVSKPEVLMHKEDGKLMEPIERVVVNCPEVYSGTIINELNMRKGMMESMSIEGDYVKIEFLAPTRGLLGYRSEFINATRGEGTLVRSFEKFEEFKGEIPSRGNGVLIAQGPGVTMGYSLNALSDRAVMFVDPGVEVYEGMIIGMNSRKDDMVVNPCKNKKMSNVRASGSDDAIKLSPPRIFTLEEALEFIEDDELVEITPDSIRLRKRFLNEHDRLRYNKSRQGK*

>CD630_21170 Clostridioides_difficile_630_NC_009089 thioredoxin reductase

MVDIIVIGAGPAGLTSAIYAMRAGLSVTVFEKNIYGGQVASTSEVENYPAVQKISGVEFSNNIYNQAVAQGVDIQFDEVEEINLEGKVKVVKTSSGEHKAKAVILANGVERRKLGCAGEQEFTGRGVSYCATCDGAFFKDKEVAIVGGGNTALEDALFLANNCTKVYLIHRRDSFRGEEVLEKSVKARENIEILYSHGVEKIEGEKTVSKIEVKNLKTEEKRTIDVSGIFIAIGLKPNNKMFENVLDLDEGGYIISDESCTTSVEGVYVAGDSRTKFLRQIITAASDGAIAAVQAANYINVE*

>CD630_21290 Clostridioides_difficile_630_NC_009089 membrane-associated peptidase

MTIIAALILFSIIVLIHELGHFIFAKRSGIKVNEFSIGMGPKIYSVKKDTEYSIRALPIGGYVSMEGEDEEQISPNSFGNKSILQRFSTIVAGPIFNIILAAILLVPVFLYIGSPTTKLGKIMPDTPAQAVGLQVGDKINKINGNSVKTWDEVANIINTSSGGELKLSITRDGSDKVVNVTPKNNNGKYEIGIQPQREKDFLGSIVNACKTTVDMTKQMLTFLGQMITGRVPGGIGNAVAGPVGVIGMVSDAARTGIINVVYLAAVISLNLGIVNLLPIPALDGWRILMLLLEAVRGGKKLDPNKEGMINVVGFGALMLFMLFITYKDILRLFQ*

>CD630_21350 Clostridioides_difficile_630_NC_009089 phosphatidate cytidylyltransferase

MLTRIIASLALVPLFLFVVYGGIPLYIAETAIVYIALHEFYKAFKIKDVHPIFIIGYLFSIYLAVKNIFNLPLEYTYAVIFILFLASIIYMLMGKNNVIDVSITFLGVFYIGVFLDFIIITINGFEKGSIYVWLIFVISFMTDIFAYFSGYLLGKHKLIPKVSPKKTIEGAIGGIIGSTLCCILFGYLFGIDLLQLAIIGSIGSVIAQLGDLFASSIKRYVGIKDYGKIIPGHGGILDRFDSVILVAPFVYSAIKFFIR*

>CD630_21360 Clostridioides_difficile_630_NC_009089 undecaprenyl pyrophosphate synthetase

MNNNIIYDIDLNNIPTHIAIIMDGNGRWAKARFLPRTAGHKAGVETIRDIVKECSKLGVKHLTLYAFSTENWKRPKLEVDTLMNLLSTYLRNEIAELHQNNVKVTAIGDISALPKTCIRELNSAKELTKNNTGVNLNLALNYGSRADIKNALIDIVKNCESGKIDINNIDEDIIKNYLSTKSIPDPDLVIRTSGEQRLSNFLLWEVAYSEFYFTDIHWPDFKKEELQKAIYVYQRRDRRFGGLK*

>CD630_21390 Clostridioides_difficile_630_NC_009089 elongation factor Ts

MANITAQMVKELRESTGAGMMDCKKALQEAEGNMEKAVDLLREKGLSKAAKKAGRVAAEGLVAIEMNDDNTVASMVEVNSETDFVAKNEDFKVFVKDAACMALATDKEDIASLLGETHKEGITLQEVLNNRVAKIGEKLDFRRFAKVVTNGQVAGYIHGGGKIGVLVEMETEARDAKVLELGKDVAMQVAAMNPKYVSRDEVDAEYIAHETEVLTQQALNEGKPANIVEKMVKGRLEKELKEVCLLEQTFVKNPDITVKQLVADVAKAVGSDIKVVKVVRFEVGEGIQKREENFAEEVAKQLK*

>CD630_21400 Clostridioides_difficile_630_NC_009089 30S ribosomal protein S2

MSVISMKQLLEAGVHFGHQTRRWNPKMAKYIFTERNGIYIIDLQKTVKKVEEAYKFTKEVAETGKPILFVGTKKQAQDAIKDEAERCGMYFVNERWLGGMLTNHKTIKTRINKLRELEKMEEEGVFNVLPKKEVIKLRAEKEKLEKYLGGIKDMPELPGAMFVVDPRKENIAIQEAHRLGIPVVGIVDTNCDPEQLDFAIPGNDDAIRAVKLITGAMATAVIEGRQGAEEEVAEDQE*

>CD630_22060 Clostridioides_difficile_630_NC_009089 aldehyde dehydrogenase

MDIKELVKMQRKYYNTGKTRDISFRIEQLKKLKLVVSQNEEKILLALKKDLNKSDFEGFMTEVGMFYSELNFAIKNIRKWSKIKRVKSSMVNFPSISKVVPQPYGVTLIMSPWNYPFQLALIPLVWSLAAGNCVILKPSEYSASTSSVVKDIVEDTFSKEYVAVVQGSQEESEKLLLERFDYIFFTGSTNVGKIIMKSASEHLTPITLELGGKSPCIILKDANIDLTAKRLTWGKLINAGQTCVAPDYVLVHEDRKNELIEKIKYYTNKYFGDNPCNNEQFPKIINQKHFNRILSLIDKDKIVYGGNYNKETLKIEPTIVDNVNWDDNIMKEEIFGPIFPILTYKDLDEVIQKIIQMPNPLALYIFTKNKYLENKLLEMIPAGGCCINDTVTHIATNYLPFGGIGESGMGSYHGKAGFDTFTHYKSVLKKLNLDVPIRYAPYDNKLIKVLKKIM*

>CD630_22090 Clostridioides_difficile_630_NC_009089 GTP-binding protein HflX

MDMAKKGITVGLNINNKSEDFNELMIELENLCSACDIDVVGSITQNAKQVNRAFYIGTGKVEEILNLIKKENIEIVIFYNELSTSQLKNLEEKLNCEIIDRTALILDIFAQRAKTREAKLQVEVASLKYMLPRLIGSNENLGRQSGGVGTKNRGSGEKKLELDRRRIEEKITSLNRELDDLKFQRETQRSMRRKSNLPNVALVGYTNAGKSSIMNKLVDIFKNSEEKKVFEKNMLFATLETSVRNIVLANNKEFLLSDTVGFVSNLPHDLVKAFRSTLEEACEADVLLHVIDISNPSYKSHIKVTEDTLKQIGADGIPMIHVYNKIDLIDVEVLDRILDSIDKEGIFVSVKKDINIDKMIKCICDSIFKDYVRCKFLIPYDKGHVVSYFNENTSIINTEYREDGAILDVECSNIEYNKYKKYALE*

>CD630_22100 Clostridioides_difficile_630_NC_009089 multidrug family ABC transporter ATP-binding protein/permease

MSRMGRGPMGKSMGAGQKANDFKGTMRKLIAYLSKFKISIILVIVFAIGSASFSIVGPKILGKATTKIFEGLVSKVSGGNVGIDFNAIGKILTFLLFLYLISALFSFIQGFIMSGISQKVSYNLRKEISAKLDRLPMKYFDTKTHGEILSRITNDIDTLNQSLNQSMTQLITSVTTMIGVLIMMLSISGIMTLVAVLILPISMFVISRIIKKSQKYFRYQQEYLGNVNGQVEETYSGQTIVKAFNREDEVIEEFDKLNDSLYNSAWKSQFLSGIMQPLMMFIGNLGYVMVSILGGWLAIKKTIEVGDIQSFIQYVRNFTQPMTQIAQVANLLQSTAAASERVFEFLEEEEEVQIVENAVSIDGLEGKIDFENVNFGYNPNKTIINDFSVNVKPGQKVAIVGPTGAGKTTIVKLLMRFYDVNSGSILIDGHNIKDFNRSELREMFGMVLQDTWLFSGSIMENIRYGKLNATDEEVIEAAKSAHVHRFIKTLPDGYKMKLNEEASNVSQGQKQLLTIARAILADPKILILDEATSSVDTRTEVLIQKAMDNLMEGRTSFVIAHRLSTIRDADMILVMNEGDIVEQGNHEELLKKGGFYANLYNSQFEEDEAM*

>CD630_22400 Clostridioides_difficile_630_NC_009089 N-acetylneuraminate lyase

MRTTDMKGIYSALLVSFDKEGNINEKGLRQIIRHNIDVCKVDGLYVGGSTGENFMLSTDEKKRIFEIAKDEVKEEIKLIAQVGSVNLKEAVELAKFTTDLGYDAISAVTPFYYKFDFEEIKHYYNTIINSVDNRLIIYSIPFLTGVDMSLDQFGELFENEKIIGVKFTAADFYLLERMRKTFPNKLIFAGFDEMMLPATVLGVDGAIGSTFNVNGVRARQIFELTKNEKISEALEVQHVTNDLITDILGNGLYQTIKLLLEEQGVEAGYCRQPMKEATDEMKSRAKEIYRKYF*

>CD630_22610 Clostridioides_difficile_630_NC_009089 M24 family peptidase

VNIKDRLSGLRKFMEEKNIDAYMIPSSDNHQSEYVGDYFKSREFISGFNGSAGTVIVTKDEAGLWTDGRYFIQAESQLEGSTIKLFKMGQEGCPTTDEYLYKNIPEGGTLGFDGRVISAREGATLAEKLSKKGIKIEYQYDLIDSIWPDRPALSDSKAFLLDVKYCGESFSSKLARLREKMSEKGTSTHVITTLDDIAWLFNIRGGDVKYNPVVLSYAVITLKEVYLFVDESKLNEEILNELAKENVQIKPYNDVYEFVKNIDKTEKVLLDGTKLSYTIYNNIPCEVEKVDEFNPVMFFKAQKNEVELENIRNSHVKDGVAFTKFMYWLKKNVGKMEITEISATQKLEDLRREQEGFFEPSFNTIAAYKEHAAMMHYSATPESNYKLEAEGLFLVDSGGQYYDGTTDITRTTVLGPISDELKLHFTSVARGMINLSKAKFLHGCRGYNLDILSRSCMWNMGIDYQCGTGHGIGFVLNVHEAPNGFRWRVVPERFDSAVLEEGMVTTNEPGIYIEGSHGIRTENEIVVRKAEKNFYGQFMEFEVVTLAPIDLDGIVPELMNKDEKDYLNWYHKLVYDKISPFLTDEEREWLKVYTRAI*

>CD630_23190 Clostridioides_difficile_630_NC_009089 ribulose-phosphate 3-epimerase

MSIICASIMCADQMKLKEELEALEEAGVKLLHCDVMDGIFVKNLAMGPELLKSISENTTIPLDIHLATETPDKYIDMMSYIKPKYISFHVESSTNVKADIQKLRNYGIGPVLAISPQTSVDKIEEYISLVEGILVMTVNPGFAGQKFNLSVLDKLDKLTEILKDYDNPPFIEVDGNINKDTIKLMNGKKVDIYVVGTSALFNDKPPISYKDKIEELKESIK*

>CD630_23240 Clostridioides_difficile_630_NC_009089 sugar-phosphate dehydrogenase

VKAAVLHGTNDMRFEDIEIKPCESDEVKIKVMAAGICGSDPPRVLKHWKYPVPAIPGHEFSGVIAEVGKDVKNVKVGDRVVAIPFIPCNECEYCKRGLFSLCDDHGMLGAKSFGAFAEYVNIKATNVLPIGDMDFEDAAMIEPLAVAMHGVLNIGVQVGDTVAVMGSGTMGQLVIQGLKIAGAGTIIAVDISDNKLRESKELGADIIINAKDINPVEKIKELTGGKGVDIALECAGSKITQEQCLLITKKKSKIGFLGIAYSDITLSEEAFENIFRKELELKGFWNSYSAPFPGQEWTKGINLVNEGKIKLKEMVSHRFSLEDTYKAFEMIRDRKEEFNKILILPQGVEK*

>CD630_23350 Clostridioides_difficile_630_NC_009089 inosine 5-monophosphate dehydrogenase

MAFYFDTPSHTFSEYLLVPGFSSTDCIPANVSLKTPVTKFKKGEEADIYMNIPLTSAIMQSVSDDKMAIALAKEGGISFIYGSQTIENEAAMVARVKSHKAGFVVSDSNIKPDNTLKDILDLKEKTGHSTVAVTEDGTSTGKLLGIVASRDYRISRMDLDTKVSEFMTPMSSIVYANKDVTLKEANNIIWDHKLNSLPVLDDNGNLMYMVFRKDYSSHKENPLELLDSSKRYVVGAGINTRDFAERVPALVEAGADVLCIDSSEGFSEWQKITLDFIREKYGDTVKVGAGNVVDREGFLYLAEAGADFVKVGIGGGSICITREQKGIGRGQATSIIEVAQARDEYFEKTGIYIPICSDGGIVYDHHITLALAMGADFIMLGRYFSRFDESPTNKVNINGSYMKEYWGEGSARARNWQRYDLGGDKKLSFEEGVDSYVPYAGSLKDNVTLSLSKVRSTMCNCGALSIPELQKNAKLTLVSSTSIVEGGAHDVMLKDNRNL*

>CD630_23470 Clostridioides_difficile_630_NC_009089 Xaa-Pro dipeptidase

MSRVKNVVELLETKGVDALYLTKKTNVNYISGFPDEEAYAVICKDGNFLVTDSRYMELAEKVCKDFEIINWHNFDRSVAKAVKSVCDKVGIKKLGFERTNIVFDKYEELKNLIEKDNGELIPTENIVETLRYVKDKDEIKNTRKACEIADKALEELIPHIKAGVSEIELATKLEYFMKMNGAQNIGFETILISGAKTSLLHGKPSDKIIEKGDFVLIDYGAMYNGYISDTTRTFIVGGASEKQLEIYNLVKEAQNVGVENMKAGVHATIPDAEIRKVVKKYEDYYYQGIGHGVGRDVHEEPFIGNYGDKIIEEGCIITMEPGIYFPGWGGVRIEDTVLITKNGPERLTKFPKDLMILDK*

>CD630_23550 Clostridioides_difficile_630_NC_009089 thioredoxin 2

MLDLDKATFEEEVLNAEGFVFVDFWSEGCEPCKALMPDVHKLAETYGDKIKFCKMDTTKARRLAIKQKVLGLPTMAIYKDGEKVDEVTKDDATVPNIENMIKKYL*

>CD630_23560 Clostridioides_difficile_630_NC_009089 thioredoxin reductase

MENVYDLVIIGSGPAGLAAGLYGARAKLKTLILEKDKTGGQIVITHEIANYPGSVPNATGPSLIARMVEQCKEFGAEMLRDNIVDTELDGDIKVLKGEKAEYRAKAVIIGTGATPRKIGCPGEKELTGKGVSYCATCDADFFEDFEVFVVGGGDSALEEAMYLTKFARKVTIVHRRQGFRCAKSVEEKAKANPKIEFLLDTVIEEIKGDGILESVVFKNKVTGETHEYFADEEDGTMGVFVFVGLDAQTDLFKGKVDMDEKGYIITDEDMRTNIPGVFAAGDCRSKTLRQVVTATNDGAIASIVAEKYIDEKFGN*

>CD630_23590 Clostridioides_difficile_630_NC_009089 HAD superfamily hydrolase

MYKLIALDIDGTILNTQKRITPEVFESIQEAKRAGAKVVITTGRPLPGVKELLNQLNLTDEGDYVICFNGAIIQEVKSEKIIHDVEMSLDDFDFIYNNVCKKYKTKIHINTMTNLITPNETPGKYTLHEAKLNNIEVKYIQKDKIDESIKICKIMIVDEPERLEEIIQQLPKNLFNKYTIVRSAPFYLEFLGKTTNKGTALKTLCTNLNIPIENAIAVGDEENDQHMIKYAGLGVAMGNARNSIKEIADYVTDTNNENGVAKVINKYILNKAI*

>CD630_23610 Clostridioides_difficile_630_NC_009089 nitrate/sulfonate/taurine ATP-binding protein

MKLSVRDINKTFVNNRVHTKVLEDISMDIDDGQFVCLLGPSGCGKTTLLTIIGGFQKSERGDVFINDKRVKKPGIDRAFIFQNYALFPWKTIRGNVLFPMKQQKIPKEKREEMLEELLVMSDLKGKEKLFPHQLSGGMKQRVAMIRALACNPEVLLMDEPLGAVDFQMRQNLQEELERIWIKKKITALMVTHDVDEAVYMSDRVIVMSRDKGRIIDDINIDIPRPRIRGSQKYEEYKNKLTDTLSKCYEV*

>CDIF1296T_02484 Clostridioides_difficile_ATCC_9689__DSM_1296_strain_DSM1296_CP011968 nicotinate-nucleotide pyrophosphorylase

MNYLIIDRMIQDALIEDVPSEDITTNSIVDENSKSTVDLICKQDGIIAGLGVFKRVFEILGDVDVKLYKNDGDRVKNREKIAVLTGSTRNLLVGERVALNYLQRMSGIATITSRYVEKLEGTNTKLLDSRKTIPNLRILDKYSVKVGGGCNHRFNLSDGILLKDNHIGAAGSVKKAVELARKNTSFVRKIEVEVETLDMVKEAIEANADIIMLDNMSLEMAKQAVDIINGRAIVEFSGNVNLDTIEDIGKIGVDVVSVGALTHSVKALDISMKNLRNI*

>CD630_23710 Clostridioides_difficile_630_NC_009089 L-aspartate oxidase

MNLEQDVLIVGSGVSGLYCALNLDKSLNVLVVSKSTIENNNTYLAQGGISTARNIDDIESFVEDTMKAGQYKNRVEAVQVLADESIENVGQIVEYGMPLDKENGEIDYTREGAHSVNRIVHSKDNTGEVVFKTLLKEAKTRENITLIEDAYLLDILKDGNKCIGARIFKSKKEIHVFSKIVVLATGGIGGLFKNSTNQRHLTGDGIAIALRNNIKIENLEYIQIHPTAFYEENNEGRRMLISESLRGEGAKLLNKNKERFVDELLPRDVVSKAIFEQMEKDKLPYVYLDATHLDSEYLINRFSFIYNECLARGTDITKECIKVSPAQHYFMGGIHVDLDSKTSMENLYAVGEISCTGVHGANRLASNSLLEGLVFSKRAAKNINSVIDNVKVKFIDVPDMDIDIEQVKKENKILVIKEIERTSEDFGDELFDY*

>CD630_23820 Clostridioides_difficile_630_NC_009089 pyridoxal phosphate-dependent transferase

MSQSMVGKHAMWPKENDVIFSISGRAQAAEKAFGMDNVINATIGALMDDSGKLITMKTVYEEYKALDNCEIGAYAALEGQPDYLEAVKKVFFRDYLPEGHIRVLASPGGSGAIKLAVWNYTNEGDEVLTSDWFWSPYVSIAEEANRKVVNYQLFDENRRFNFESFKEKFVNIAEKQGRVFTIINTPAHNPTGYSVADDEWDKILDLSKEVAKDKDKKIIFFVDSAYIDFAGDDDVCRKFFKKFSNLPENVLVLVGFSMSKGFTAYGMRMGAIICISSSEDVAEEFHYSCVHSCRANWSNCNRSAMAVLSNIVNDPKKFKEYEDEKEIYKNMLTRRADVFVKEAERVGLEILPYIAGFFVSIPCDNPKEVCEELTKHNLFAVPLKMGLRFAVCAVSEDKCKKAPSIIKEALESLEVKINN*

>CD630_24360 Clostridioides_difficile_630_NC_009089 divalent cation transporter

VGYMDRKLDASQDLLYEVKSLIDNNKVLELRELIEEYHIIDIFDIMENLEEDMKIQLFEVLPLDMASSILEEGSVEFFISILSKLDVEHSKNILELMSLGDMADKLSELEEEEREHIINLLNQENADYVKELLFYDEDSAGGTMTTGYISINKDMTALEAIDHMREEAEEAETIYYIYVVDDEEKLVGVLSLRELIIARDANIVEDLMSENIISVYVDEDREEAVRLVSKYNLIAIPVVDRQEKLKGIITVDDIIDVMEEEATEDMYKFAGSSEHEREVAEKENPTLREQIISALRGRLPWLIITLVGGLLASLILSNLDYIMNPVYAPLVFFIPVVIGMGGNIGTQSSSVTVITLSNKDLNFSNVVREGIVGIITGLLCSIITGIVIYFVMRKLDIVLIVSISLFINMVLGATIGAFMPVLLKKMDADPSTVSSPIISTALDITGIAVYFIITTALLSKIV*

>CD630_24390 Clostridioides_difficile_630_NC_009089 diacylglycerol kinase

MKPEKTRQGIIKAFNAAIEGILYTFKFERNMKIHYLGSVAVLIISLFFNFSKLEMIMLLMSICLVVVAEMFNTAIEKAVDLVTDEYHVLAKIAKDVAAGGVLVAALNSVVVGYILFYDKLTDISGILIYKIRESELHITLICILLVLIAVVVVKALTSTGTPLKGGMPSGHAALAFAIATAITLMTERVVASTLAYIMAVLVAQSRIEGKIHTFWETIAGALLGVLIAILVFQLGMFYN*

>CD630_24400 Clostridioides_difficile_630_NC_009089 rRNA maturation factor YbeY

MDLILDDRQDKLEVSEELIEKIKDIIIECLDYEGYDDNYEVSLSFVDNKEIHELNREYRGVDRVTDVLSFPLLSDDFEDVELEEESLGDIVVSLERALEQSIEYNHSFEREVCFLICHSMFHLLGYDHDTDENTKEMREKEEHILNKLNITRE*

>CD630_24480 Clostridioides_difficile_630_NC_009089 MiaB-like tRNA modifying protein

LKKVAFYTLGCKVNQYETEAMLELFEKDGYEQVNSEEYADVYVINTCTVTHMSDRKSRQYIRRVKKKNPDAIIAVVGCYSQVSPEEILDIEEVNLVMGTNDRRKIVEEIKKINSSKKVSTVDDIMKVKAFEEIEISQTNGKTRAFMKIQDGCDRFCTYCIIPYARGRVRSRDIDSIVDEVKKLANNGYKEVVLTGIHVASYGKDLKDRDIKLLDVIKQINQIEKIERIRLSSVEPILFTDEFVNEVLKMDKVCPHYHLSLQSGCDETLKRMNRRYTTLEYKTIVDRLRSKMPDVAITTDVIVGFPGETNEEFKKTYEFLKEIELSQMHIFKYSPRKGTPAATMENQVDPQMKHFRSEQLLNLSKVNFNKFATKFIGRELDVLFEQNIEGNKYEGLTSNYIRVVVESDKNIQGQILKVKINDVKDEYVEGILL*

>CD630_24500 Clostridioides_difficile_630_NC_009089 50S ribosomal protein L11 methyltransferase

MNNWIEVTIKTTTEAVEPITNILYEQGAGGAVIEDPKDFLFQKKNELDWDYVEEEVFKKNEEDDVLIKTYVSEEKNVMEFVEIIKQKVLGLKDFGIDIGEGSVSLYQVNEADWANAWKAYYKPTKVGQRVVVKPTWEDYAMQEGDLIIELDPGMAFGTGTHETTSMCIRELEKYVNKDSKVFDIGCGSGILAIAAAKLGAKEVVAVDLDEVAVKVAKENVLENKVEKSVSVMHGNLTDVIKDKADVIVANIIADIIKILAKDVQNFMKEDAIFISSGIILDKVEEVKESLIENGFEIVEVQKLGEWSAIVSKLKK*

>CD630_24560 Clostridioides_difficile_630_NC_009089 sugar family ABC transporter ATP-binding protein

MSEVILKNISKLYSNGFNAVKNINIDIKDKEFIVLVGPSGCGKSTTLRMIAGLEEISEGELYIGDKLVNDIEPKDRDIAMVFQNYALYPHLSVYENMAFALKLRKLPKDEIDKKVKEAAKILDLLPLLNKKPKTLSGGQRQRVALGRAIVRNPKVFLMDEPLSNLDAKLRTAMRTEITKLHQQLGTTFIYVTHDQVEAMTMADRIVVMKDGVVQQIATPQDVYDYPANIFVAGFIGAPQMNFIDVILIEENDEIYAQNEHIKLKLNKEKHYDLIKDNYINNEVVIGIRPEDIHVEDTFIKSNPDTCFKSRIEIKELMGAETYAHLKLGENTITIRFDSKNRINVGDDLILSVDNSRVHIFDKETTLAIR*

>CD630_24590 Clostridioides_difficile_630_NC_009089 ROK family glucokinase

MYYIGVDIGGTGIQAGVVDNYGKIIFRSECKTVIEKGFEGILNDIKIMIYKLLEDNKLTMSDIKSIGFGVPGFINKEGLVTCVNLKWNKKAFNKELKRRFPDVEIHGENDATVAALGEAKFGSMKGANVGVLYTLGTGVGGGIVINQKVFSGAHGLGSEIGHQIIGENYFNCNCGNNGCVETFCSATAIIKYSQKLIEEGEKSRILDLAEGNLENVNAKMVFDAYRENDLVAIKVINRFKEYLAKTFANTINSLDPEIISIGGGISKSSDIILDGIEDLVRKFVLYKTEDIATITCATLGSDAGIIGAAFL*

>CD630_24610 Clostridioides_difficile_630_NC_009089 chaperone DnaK

MGKIIGIDLGTTNSCVAVLEGGEAQIIANSEGMRTTPSVVAFTKDGERIVGEPAKRQAVTNADKTITSIKTHMGTDYKVNIDGKSYTPQEISAIILQKLKSDAESYLGQTVTEAVITVPAYFTDAQRQATKDAGRIAGLDVKRIINEPTAAALAYGMDKLDQEKKILVFDLGGGTFDVSILEIGDGTFEVLATAGNNRLGGDDFDQIVIDYLAEEFKKAEGVDLRNDKMALQRLKEAAEKAKKELSSTMSSNINLPFITATAEGPKHLNIDLSRAKFEELTRGLVEKTMEPTKTALQDAGLSTGDIDDVLLVGGSTRIPAVQEAVKKFIGKEPHKGINPDECVAAGASIQAGVLAGDVKDLLLLDVTPLSLGIETMGNVMTKIIERNTTIPTKKSQIFSTAADNQTAVDIHVLQGERSMAYDNTTLGRFQLTDIPPAQRGIPQIEVTFDIDANGIVNVSAKDLGTGKEQKITITSNTNLSEAEIEQKIKEAEMNAEADKQKKEKIEAFNQAESTIYQTEKTLNELGDKISSGEKEDIEKAIADLKAVKDNQDATAEELKKATDEVMTKFQKVSQEMYQKAAQEQQAAQGAEQAQDNGPKDDNVVDADFKEVDEDK*

>CD630_24640 Clostridioides_difficile_630_NC_009089 oxygen-independent coproporphyrinogen-III oxidase

MLGLYVHIPFCVKKCKYCDFNSYKMDIDSKKRYIEDLKIEMELYSNKLYKDNKYKNKECCSLNKNDKITSIFVGGGTPSILTSDEIREVFISIKEMFDIDENAEITIECNPGTLTLEKLKTMKEIGINRLSIGLQAIQEKHLNFIGRIHTYEEFEKNYKDALSVGFKNINIDLMYSLPNQTLCDWKETLEKVVHLNPTHISAYSLILEEGTELYNMYESNKFELIDENVDIEMYEYTINYLKSKGYNQYEISNYSKEGYNCEHNILYWECEHYIGIGAGASGYINENRYNNVESLEDYHLSLVKREKPIQENEILSEKDMIEEKIFMGLRMNKGIKFEDFKKKFGIDFREKYNKQIEMLLARKLINQSFEGIQLTQKGREISNSVFIEFME*

>CD630_24660 Clostridioides_difficile_630_NC_009089 tRNA-nucleotidyltransferase/poly(A) polymerase family protein

VIFMINIEIPKKVDYIIKELEKNGYEAYIVGGCVRDCLLERVPNDWDITTSARPEVVVELFEKTIPTGIQHGTVTVMIEHEPFEVTTYRIDGNYSDGRHPDSIEFTNNIVKDLSRRDFTINSIAYNSKTGLVDPFNGYEDIQNKYIRCVGNPVDRFEEDALRMLRAVRFSAQLNFKIAEGTKQSIHKKADLIKNVSIERIQTEFNKILVSDSSKLNLLKSTGLLKFIIPEICELEDVTQHNPYHIYDVQKHTLIATEVIEDELYLKLTMLFHDLGKKVTKTTDKNGVDHFYTHSRESVKIAKKILKRLKYDNYTINKVLILIQYHDYRIEPKRKIIKKLLNKLEDVGLFEDLIKVNWADTLAKNPKYAKQKILNLIECEKEFKHIINQKECFNLKDLAINGKDLISIGIKPGKDIGYILNKMLEIVINNPELNEKEILKEKALNIYTF*

>CD630_24670 Clostridioides_difficile_630_NC_009089 elongation factor 4

VDNKQSRTRNFSIIAHIDHGKSTLADRLIQQTGLVSERDMKSQLLDNMDLERERGITIKLQNIRLMYKAKDGNEYYLNLIDTPGHVDFNYEVSRSLAACEGALLVVDAAQGVEAQTLANVYLAIDQDLEILPIINKIDLPSARPEEVKNEIEDLIGLDSSEAPLISAKTGLNIEDVLEDIVKNVPPPKGDNEAPLKALIFDSYYDAYKGVVAYVRVFEGTVKKGMTIKMMNTNKKFEVTEVGVMAPGQTELSELSAGDVGYIAASIKDIRSCRVGDTITDSNNPTEEPLPGYKKATPMVYCGIYPGEGEKYENVRDALEKLQVNDAALEYEAETSAALGFGFRCGFLGLLHMEIMQERLEREFNLDIITTAPSVIYRVTKMDGEVVMIQNPANLPEPSEIKMIEEPIVKGDIIVPKDYVGVVMELCQERRGNMLNMEYIDERRVMLHYDLPLNEVVYDFFDALKSRTRGYGSLDYEVKGYVASTLVKLDILINKEQVDALSFIVHETRAFPRGKAMCEKLKGEIPRHQFAIPIQAAVGNKVIARETISALRKDVLAKCYGGDISRKKKLLEKQKEGKKRMRQIGSVEVPQKAFMSVLKLDE*

>CD630_25060 Clostridioides_difficile_630_NC_009089 major facilitator superfamily transporter

METQSNNKKGNLIIAIVMTGAFISSLSQTLLSTALPNIMSDFKITADIGQWLTTIYLLIAGIIVPTTAYLINRFSTRKLFITSMSIFSIGCIIALFSNNFSTMLIARVLQAMGSGSLMPLLQVIILYLCPEEKRGAAMSLVGITVGFAPAIGPTLSGWLVDSFGWHSLFLFLSPIAILDVILSFILLRNVGETQKLKLDIPSIVLSSLGFGGLLIGFTNQGNYGWTNIATYLPILIGIMSLILFTLRQLKSKEPFLELRVFKNKPFLISTILIMIVYASMMSATLMIPLYVQSVRGFSALSSGSLMLPGAILMVVLNPIAGRHLDKYGPHALSILGTGCLLLGTLSFAFLGRDTSLIHVSLMYCIRMIGISMVLMPLTTWGIKTLDRELISHATAINNTLRQISGAIGSAILITIMTSATKKAHMSSNMLSNIHGIDVAFSIAATLAFTGLIVSICFIKRHQIIRS*

>CD630_25140 Clostridioides_difficile_630_NC_009089 threonine dehydratase II

MMNVTLTDVKEARETIKNIVKRTDLLESVKLSEKTGANVFYKCENLQKTGSFKLRGACNKIASLTDEEKANGVIASSAGNHAQGVALGAKMTGIKSTIVMPATAPLAKVSATKGYGAEVVLNGAVYDDAYAKAVEIQKETGATFLHPFNDKYVIAGQGTISLEIFEQLDNKVDTILCPVGGGGIISGVAVAAKALNPNVKIVGVQTANIPSMKESIKNGKVTTAFNDTTIADGIAVKTPGDLTFEIINELVDEIVVVEETEIAESILFMMESQKIVSEGAGAVCTAAILSGKYVPAKDENVVCIISGGNIDINTLYRIIGVALAKEGRRYSFSTIMEDKPGNFAELTRIISENGGNILSANQGKLSAGEALGKQSAEFILETIDYDHIARIKKAIEEKGFKIIEL*

>CD630_25240 Clostridioides_difficile_630_NC_009089 nicotinic acid mononucleotide adenylyltransferase

MRSENLVKMAELKNTEKLEKFNRHKGKIKIGILGGTFDPIHYAHLATAEFIRDKYDIDKIIFIPSGNPPHKLCITTDKYDRYNMTLLATESNEDFLVSKVEIERKKRTYTIDTLKYLKKKYKNADIYFITGADAICSVEEWKDVKKNFELATFIAATRPGISLLRSQETIEKLTKKYNADIITVYVPSLDISSTYIREQLNEGKSIRYLVPENVENYLYENKLYQYGDD*

>CD630_25340 Clostridioides_difficile_630_NC_009089 ABC transporter ATP-binding protein

MKILYTENLSKHYGKGESLVRALDNVDLEINEGEFVAIIGKSGSGKSTLLHMIGGLDIPTSGKVYIDNKNIFTLKEEELAVFRRRKIGFIFQSYNLIPSLNVWENVVLPIGLDGREVDESFIKELLKSLGLENKHDVLPNTLSGGQQQRVAIARALATRPAIILADEPTGNLDSKTSDEVMSILKSMSKKYSQTLVMITHDDSIAQMADRVIFIEDGRVSKVGDKND*

>CD630_25740 Clostridioides_difficile_630_NC_009089 thiamine pyrophosphokinase

MKICIVLNGEIEDYEVTRDIIIKECYDCIICADGGANHTYKMEIMPDYILGDLDSVEEEKINFYKNKGVKFEKFPSKKDETDTELCLFLAKTLKANHIDFFGALGGRIDHTLANIKLLYYLKEDGIYSRILSDKEEMYIVENEEISLYGNPGDTISVIAINGDAKGVTLTGLEYPLDNYYMKYSVPIGISNVMLSNSCKIKVEQGCVLVVRNL*

>CD630_25750 Clostridioides_difficile_630_NC_009089 ribulose-phosphate 3-epimerase

MIKLAPSILSADFAKLLEDVRKVESAGCEYLHIDVMDGHFVPNITLGPLVVKSLKKENINMVFDAHLMIENPDQYIEEFVKAGCDIITVHQEACVHLHRTIQNIKSHGIKAGVVLNPATPVDTIKHVLPDLDMVLLMSVNPGFGGQSFIPCVLDKIKELKAIIDSQGLNIDIEVDGGISPKNVAEVVQAGANVIVAGSAIFGSDDIQETVNLFRKNASLEELV*

>CD630_25760 Clostridioides_difficile_630_NC_009089 ribosome biogenesis GTPase

MLEGKIIKGISGFYYVDTYNGIYECKARGIFRKQKITPLVGDRVKISIVDEDEKKGILEEIDSRDTELIRPPIANVDKALIVFAIKNPKPNLSLLDRFIVLAEKENLETVIILTKADLDDNDTLETVKNIYELSGYKVIPVSNITKLNIDKVKEELKENVVVFAGPSGVGKSSLLNEIDENFKLQTGVVSDKIKRGKHTTRHAELLKLEFGGMVADTPGFSSLALEDIEEVELKDYFIEFDKFNDCKFGSKCIHENEPNCAIKEAVTNGEISKERYDSYIQLLHEIRQNNSRRY*

>CD630_25770 Clostridioides_difficile_630_NC_009089 3-oxoacyl-ACP reductase

MKKKTVLITGGARGIGKAMSKAFAKEGYNVLVNFNKSENEAKELYTILNEKNFSVKLFKANISNREDVEDMVDYCIKEFGGLDVLVNNAGVSQDKLFTDITDEDWDNMMNINLKGSFYCSQVALKYMISEKKGNIINISSIWGISGASCEVHYSITKAGIIGMTKALAKEVGPSNIRVNSIAPGVINTDMLSGYNEEDIDALVEETPLMRLGTPEDIANCAIFLASDKSNFITGQVISPNGGFVI*

>CDIF1296T_02705 Clostridioides_difficile_ATCC_9689__DSM_1296_strain_DSM1296_CP011968 serine/threonine-protein kinase and phosphatase

VGDTILGNRYEIIRKIGDGGMAFVYEAKDRLLNRTVALKVLRPEFVDDDEFLTKFKREAEAVASLSHPNIVNVYDVGEDGKVHYIVMEFVDGKNLKEIIQDEGILDEYTALDITKQIAMALSAAHKKGIIHRDIKPHNILISNEGRVVKVADFGIAKAVSNSTMTNIGSIIGSVHYFSPEQAKGKFVTNNADLYSLGIVLYEMLIGKVPFRGDSPISIALQHINDDIDFTSEEKVRIPQSVRTTIKKLTEKSSADRYQTAEELIEDIEYIEKNIDLDFIKEYDDFATKKIDEKEINKVVNPTLAKPAPEKVVKPVEVADLDDDEDYYDDFYEEDDDEDEEEEEIMRAKKNQRPKSTPSKRTKKKKKKQESPKSRRRLKVIAAVLILILCAQVFLAYKFLFAGGFGNKSLTVPNLVNMTLEEAQSAVEKEGLYLSVKSEEYNSEVDENCIISQTPEGGSTNVKKGDTINVVVSKGSSQASVPNVVGLTLSNAKQLIEENNLKVGTVKYEYSSIYKEGTVLSQSPGAGSSRAQEGDEVNLYVSKGSEKSNTQTPTVPDKKPTTPENNTPTEPGSNSGNSGGNSGGSNSGNSGGNSGNNGSNSGGSNSGNSGGNSGGSNSGDNGGSSGGSNSGDNGGSSGGSNSGDNGDSSGGSNSGDNGGTSSGTGANIGKTE*

>CD630_25790 Clostridioides_difficile_630_NC_009089 phosphatase

MVYSCASHIGKIRKNNEDYCEGEVIDTEHGPIGIFAIADGMGGHKKGEVASKLAVENIIDFLKENLLQHDNVKIDYIDDILKQAYNNVNSIVHKKSMEDIEFEGMGTTLVTAIVYNNVLYVANVGDSRCYLLTDEKFDKITIDHSVVEELMMAHVITEEEARRHPQRNRITRAIGTDDMVVVDIFKKEIKKSDIILLATDGLTGFIDDEDIRDLILDYEYERTSNISEELISMANDVSGKDNVSVIVIKV*

>CD630_25840 Clostridioides_difficile_630_NC_009089 methionyl-tRNA formyltransferase

MKIVFMGTPDIAVPCLQKIIDENYEILGVVTQPDKPKGRGKKLGMSPVKELAIENNIPVYQPVKARDKEFIDKIKSLNPDVIVVVAFGQILPKEILEIPKLGCINVHVSLLPKYRGAAPINWVIINGEEKTGVTTMYMDEGLDTGDMILKTEVNLDENITAGELHDKMMNIGAETLKETLRLIEEGNAPREVQNHEEFSYAPIMNKSLGNIDFSKSAREIHNLVRGVNPWPSAYTTYNDVIMKVWKTKVLDEKSTKDVGTIIDVSKDGIKVSTIDNVLLIEEIQMPNKKRMLVGEYIKGNTIETGLVLG*

>CD630_25850 Clostridioides_difficile_630_NC_009089 peptide deformylase 2

MALRQIVQIGEPVLRKKSKKVEKIDEKIIQLLDDMAETMYDADGVGLAAPQVGILKRVVVIDIGEELIELINPEIIETSGEQIDEEGCLSVVGEAGNVRRPNYVKVRALNRNGETIELEGEELLARAFCHEIDHLDGILFVDKIEK*

>CD630_25860 Clostridioides_difficile_630_NC_009089 primosomal protein N

MKKYAKVIVRSNTIYTDNLFTYQIPVFLSDVIKIGHRILVPFGKGNKPTEAFVFQFTDSLDEKIKIKEIIDILDENPIFRKEDLELVYWMKNRYLCTYIECINLIYPKGYKLNNYKVVLLGESLSGLNDIELKEKISTLSDKNREIVEKVIDSKGKIKVDKLKYIPNLNSSLYTMNKNGIIKLCWEYKNHKNEKKVCYISLSLESDKIDDYIEQNKINVGSKQKEILSFLKNNENVEINDLLDLLNASKQSINSLSKKKLITLEFKDYYREPKSIYKSVLKSIKLNNEQQEAVDEIKSNMFVDDKKTYLIHGVTGSGKTEVYMEIIEFALNQGLDSIFLVPEIALTPQTIDRLKIRFGDLVGVFHSKLSEGEKHDVYKAVKAGKVRVLIGARSALFAPFNSLGLIIIDECHESSYKSEKNPKFNAIEVARFMALKNNITLILGSATPSIEEYYRAKSGEYKLINIKSRANDKPLPNIEVVDMKDELDKGNRSIFSMKLQKEIRYAIEENNQVILFLNRRGYANFVSCRKCGYVFQCENCDISLTYHKKSNTGRCHYCGYEKEIPKECPECKSTYVKPFGVGTQKIEEELKYIFPDIKTLRMDKDTTSKKGALDEILNKFKDKEADVLIGTQMLSKGLDFENVTLVGILSADMILNFPDFKSFETTFQLITQVSGRAGRADKEGKVVLQTYDTEHYAIKHAIEYDYEGFYEDEIKIRKAFGYSPFNNMLSVVVSGEDERLVIKNIKNMHASLIYLLEKRGINDLGFILGPNPCSISKINQNYRWQILFKDENIEINLLKGIIKYICITKRDLIFDKNINVSIDINPNSVL*

>CD630_25880 Clostridioides_difficile_630_NC_009089 guanylate kinase

MFVKKGLLLVVSGPSGAGKGTICKELLKENDTIKLSVSATTRKPRTGEVDGVNYFFISKEKFEEMIEKGEFLEYAQIYDNFYGTPKAAIMECLEKGQDVLLEIEMQGAKQIKEVCPEGVFIFVLPPSLEELKNRIVGRGTETEAEIEKRFSCAYEEIKMIKDYDYFIFNEDVKTSAKEIEGIISSEKNKVSRYKNIIIEKFKEEL*

>CD630_26130 Clostridioides_difficile_630_NC_009089 M24 family peptidase

MKAQRLNAVLEQMKKDDISQMLVSDPTSIFYLTGVLIHPGERLLALYLNLNGNNKLFINELFPVSEDLGVEMVWFNDTQNPVEIITEHIDKNATMGVDKNWPARFLLNLIELGGGSKFVNSSYIIDTLRMCKDEEEKELMRIASKLNDKAMEQLKATVSGELTEKQLVGKLSKIYEDLGTDGFSFDPIIGFGPNGANPHGEPGNALVKPGDAIILDIGCIKDNYCADMTRTVFYKEIPEKGREIFEIVLEANKRAEAIVKPGVRFCDIDAAARDYITEKGYGQYFTHRTGHSIGLEVHDKGDVSSINTDTVQPGMIFSIEPGIYLPGEFGVRIEDLVLVTEDGCEILNKHDKEICVVG*

>CDIF1296T_02745 Clostridioides_difficile_ATCC_9689__DSM_1296_strain_DSM1296_CP011968 isoleucyl-tRNA synthetase

VIYMAKFKPLVDSSVKQAEAQVFDYWKDINILEKTLEKGKDDPSFVFYEGPPTANGNPGVHHVLSRTLKDSVCRYKTMSGYQVKRKAGWDTHGLPVEIQVEKELGLTSKQQIEEYGIAEFNQKCRESVFSFEKQWRIMTERMAYEVDLDNPYITLDNNYIESVWWILNKFNKEGYIYEGHKILPYCPRCGTGLASHEVAQGYKEVKTNTVIAKFKKKDADEYFLAWTTTPWTLPSNVSLTVNADVDYLKVKKGDEVYYVSKPLADKVLGEDYEVLEEMKGKDLEGLEYEQLMPFVEVDKKAFFITVGDYVTTEDGTGIVHTAPAFGEDDYNTGKRYDLPVIQPVDETGKFTSTPWEGRFVMEDGLDVEIIKWLASENKLYSKEKVVHNYPHCWRCQTPLVYYAKPSWYIEMTKLKDKLIENNNGVKWFPSFVGEKRFGNWLENLNDWAISRTRYWGTPLPIWRCECGHTDSVGSRAELAEKAIEDVNPETVELHRPYVDDIHLKCEKCGKPMTRVTEVIDCWFDSGAMPFAQHHYPFENKENFDQLFPADYISEGIDQTRGWFYSLLAVSTFVLGKAPYKSVLVPDLVLDKDGKKMSKSRGNTVNPMELFDQYGADALRWYLLYVSPPWTPTKFDMDGLKEIQSKFIGTMKNVYNFFTLYANTDDINPTEFFVEYKDRPELDRWILSKFNNLMKDVEENLAIFELNKTLRMIQDFINDDLSNWYIRRSRRRFWATELTEDKKAVYNTTYELLHGLCRAIAPFAPYMSEEMYRNLTGEVSVHLAEYPKCNEELVDTKLEEKMDLAKNLVTLGRASREVERIKVRQPLQKVLVDGKFEDTISDVVDLIKEELNVKEVIFAKDLDEYMNFSLKPNFKEAGPLLGSKMNLFVGALSKLNAHETANKLEKGETLTVDLDGEAFEFNKDLVLIGITAKEGFNVSVENNLFVILDTKLTEELINEGYAREFISKVQQLRKSNNFEVLDNIVIEYCGDDEIAKAVDHFNEYIKSETLALEINRVDDKSLEEQNLNDHMTGIKVIKK*

>CD630_26230 Clostridioides_difficile_630_NC_009089 pyridoxal 5-phosphate-dependent protein

VIIMGSIKDNLSEIRNKIDKAAKKVDRDSKEITLLAVTKTVDVDLVNEAMREGITSVGENKPQELARKYDIIGDKLNWHLIGTLQTNKVKYIIDKVCMIHSLDRIALCEEIQKRAEQINREIDCLVQVNISKEESKHGLDREEVVDFVKEVSEKYKNIKIKGLMTMAPFIEDEDEIRGVFKGLKDLSKQIKELNIPNVGMDVLSMGMSHDYEIAIEEGATIVRVGTSIFGERNYNKK*

>CD630_26300 Clostridioides_difficile_630_NC_009089 glycerol-3-phosphate dehydrogenase

VVLNMEKVCVLGTGSWGSALALGLAKKGNDVSMWTRKEEQAKKINRTKENTDYLPGVLFPNNITISTDIEKTIKDCKVIVLAVPSQAVRSTCQKIKPFIKEGQVIVNVAKGLEKGTGLRLSQVCEEELPQNPYVILSGPSHAEEVARDIPTTVVVASKDLKIAQMIQDLFMSPKLRVYTNPDIVGVELGGALKNIIAFGAGICDGLGYGDNAKAALMTRGISEMSRLGIAMGANMSTFAGLSGIGDLIVTCTSMHSRNRRAGILIGKGMSLEDTLKEVKMVVEGITATEVAHDVAEKLDIDMPITNAIYSVIKKGSNPKEVGIELMMRSKKHEMEEVVLGDDI*

>CD630_26320 Clostridioides_difficile_630_NC_009089 GTP-binding protein Der

MSISRPVVAVVGRPNVGKSTIFNKFAGKRISIVENTPGVTRDRIFAEVEWLDKYFTLVDTGGIEPDSEDIILSQMRNQAMLAMDMSHVILFIVDGKAGITAADKEIAQLLRKTKKPVILVVNKIDSQSQFDNIYDFYELGFGTPFAVSGANSMGFGDLLDEIVENFPAGLDTEYEEDIIRVAITGKPNAGKSSILNKILGEERVIVSPIAGTTRDAIDTYFEKNGQKFLLIDTAGLRRKSKIYETIEKYSVIRAMSAVDRADVVLIVIDALEGVTEQDTKVAGIAHDEGKGCIFVINKWDLIEKDNKTMSNYTKDIKEKFPFMMYAPIVFVSAKTNQRMNKILDTVEYVSNEHSKRISTSALNEVIGEAVMLNQPPSDKGRRLKIYYGTQTDIRPPKITLFINDKDLTHFSYQRYLENKIRENFGFEGTSIKFEYRQKNKK*

>CD630_26420 Clostridioides_difficile_630_NC_009089 sporulation sigma factor SigG

MQVNKVEICGVNTSELPVLKNKQMKELLLQIKNGDEEARQQFVRGNLRLVLSVIKKFNNRGENIDDLFQIGCIGLIKAIDNFDLSQNVRFSTYAVPMIIGEIRRYLRDNNPIRVSRSLKDIAYKALQVRERLIRTNSKEPTVSEIAKELELEVESVVMALDAIQDPISLFDPVYQDNGDAIFVMDQVQDKKDTDENWLQEISLKEAIKKLNSREKLVLDLRFYKGRTQIEVADEIGISQAQVSRIEKNALKNMRKYV*

>CD630_26540 Clostridioides_difficile_630_NC_009089 phospho-N-acetylmuramoyl-pentapeptide- transferase

MMLGITELTYTALIAFLIVIIIGPIFIPMLRKFKFGQTVRDDGPQTHLAKNGTPTMGGIIMIVAILITGLTRVKVSHDMAVGLICIAGFGFIGFLDDFIKIKLKRSLGLKAYQKIILQVALSFYVAFYQYTSSSSASQLMIPFTDFVINVGILYIPIMMFIIVAIVNAVNLTDGLDGLASGVTLIVSVFFMLFASSIAGNTEVAVLAAATVGACLGFLGFNSYPARVFMGDTGSMALGGAVVAFSVLTNSVLIIPIIGGIYFAEALSVLIQVGYFKATRKRFFKMAPIHHHFEQCGWPETRVVFIFWIITVVLAWISIIAVF*

>CD630_26580 Clostridioides_difficile_630_NC_009089 rRNA small subunit methyltransferase

MEFHHVSVLLNECIENLNIKPDGVYVDCTMGGAGHSKEIVKKLSDKGLFIGFDQDKNAISTAKERLSEYESRVKFVHSNFENIKEELEKIGVYKIDGVLADLGVSSHQLDEADRGFSYMQDAPLDMRMDVRCEFSAYDVVNTYTEDELTKIIKDYGEDNWAKRIAKFIVEERANKPIETTGELVDVIKKAIPKKARIVGPHPAKRTFQAIRIEVNNELGVITKMINDASSIMNEGGRICIITFHSLEDRIVKNAFKHLASDCICPQHLPICQCDKESEVKIITRKPILPSEEEIEVNPRSRSAKLRVAEKI*

>CD630_26600 Clostridioides_difficile_630_NC_009089 M16 family peptidase

MEKIVNDILKEEVYYEKLQNGLDVYFMPKRGFMKKYAILATNYGSNDLEFVPIGEDKKIRVNEGIAHFLEHKMFEQPDGGDAFDKFSKLGVNANAFTNFTMTAYLFSATENFYESLEHLIDYVQTPYFTDENVEKEKGIIAQEIKMYNDDPDWNVYFNCLKAMYVNYPARIDIAGTVDSIYKITKEELYKCYNTFYNPGNMALFVVGDLDVEKVIDVTKKSNNYKVDRLSKSIERFYPEEPESVKEKEVIEKFPISMPMFNIGFKDSNVGLKGKELLRKEIVTDILVGMLFKKGSKLYEDLYMQGLINENFGAGFSSQVDYAFSIIAGDSKEPKKVKEIILDYIEKSKKEGLSKEEFERTKKKKIGSFIKCFDSINFIGNSFISYVFKDINLLDYLDIIKDITFEEVEERLKEHFKEEYCVISIVEPK*

>CD630_26620 Clostridioides_difficile_630_NC_009089 GTP-binding protein YchF

MKLGIVGLPNVGKSTLFNAITQAGAESANYPFCTIDPNVGVVSVPDERLNKLQELYNSEKIVPTAIEFCDIAGLVRGASKGEGLGNKFLSHIREVDAIVHVVRCFEDENVVHVDGSVDPLRDIETINLELIFSDIEILERRINKTQKAAKADKTLGSELDLLKSIMSTLEESKCVRTMEFTEDEQTFVNSLDLLTSKPVIYASNVSEDDLADNGENNKYVQQVKAFAETEDAEVVVVCAQIEAEISELDSAEEKKEFLETLGLEQSGLDKLIKSSYALLGLISFLTAGPKEVRAWTIKVGSKAPQAGGKIHSDIERGFIRAETIAFNDLVEHGTMAAAKEKGLVKLEGKEYIVKDGDVILFRFNV*

>CD630_26700 Clostridioides_difficile_630_NC_009089 ABC transporter ATP-binding protein

LKKEESLLKVEGLKKYFEVKSGLFDKEIKCVKAVDGISFDLKKGETLAIVGESGCGKSTAGRTILRLIEKTEGKVEFNGVDIYKLEKEELRKLRPKMQMIFQDPYSSLNPRLNVEQIISEAVIEHNLVPKNEIKDNILNVVESCGLSKYHLNRKPGEFSGGQRQRIGIARALALNPEFIVCDEPVSALDVSIQSQIINLLMDLQDKYKLSYLFISHDLSVVNHIAHRVCVMYLGSIIEIATKGELYLHSQHPYTKALMSAVPIQDPTIKKNRIILKGDIPSPTNPPSGCKFHTRCPYCKEICKKEIPVLKEISRNHFVACHLV*

>PCZ31_RS04475 Peptoclostridium_difficile_strain_Z31_NZ_CP013196 peptide ABC transporter ATP-binding protein

MNREKILEIKNLKQYFHLDKSTTVKAVDDISFDIYKGEIFGLVGESGSGKSTTGKTIIRLHESTGGEVIYKGNCISDKKTYKFIKKDVNKSMQIIFQDSTSSLNPRMTIADIISEPLKIQGICKNKTDRMNKVYEMLKLVGLDRSYANKYPSDFSGGQRQRIGIARALSVDPEFIIADEPIASLDVSIQAQIVNLFKKLQQEKNLTCLFIAHDLSMVRHISDRIGVMYNGKLVELADSNELYNNPIHPYTKSLLSAIPVPDPRYAKSRNRIEYNSNGYDCSNEKSLSWIEVSDGHFVYSSKSEINKYQQNLKVV*

>CD630_26710 Clostridioides_difficile_630_NC_009089 ABC transporter ATP-binding protein

MSKALIEVKDLKVYFHTDKGIVKSVNEVSFNINEGETIGIVGESGCGKSVTAMSLMKLLPTSKIEGGEIIFRGKDILKMNEDELMGIRGNEISMIFQEPMTSLNPAFTIGSQIIEGIMIHQDLSKEEAKKKVIDMIKLVEIPRAEEIYNSYPHELSGGMRQRIMIAMALSCNPKLLIADEPTTALDVTIQAQILDIMKNIKEKLNTSIMMITHDLGVVAEMCDKVLVMYAGKIIEVAEVVELFKNPKHPYTIGLLKSKPVLGKNKDKRLYSIPGQVPNPIGMPDSCYFSDRCEKVCDKCRTQIPPLIELNSGHSIACWLYKKEEI*

>CD630_26760 Clostridioides_difficile_630_NC_009089 acetoacetyl-CoA thiolase 2

MKDVVIVSAVRTPIGSFGGVFKNTSAVQLGTIAVKEAISRVGLNLSEIDEVIIGNVLQTGLGQNVARQIAINAGIPNSVPSYTVNKLCGSGLKSVQLAAQSITSGENDVVIAGGTENMSQAPYIVPTARFGSKMGNITMVDSMLTDGLIDAFNQYHMGITAENIATKFEFTREMQDKLALESQNKAENAIKNNRFKEEIVPVDVLIRRGKIETIDKDEYPKLGMTFEGLSKLKPAFKKDGTVTAGNASGINDGAAMLILMSQQKADELGIRPLAKIKSYASAGVEPEVMGTGPIPATRKALKKAGLSINDIDLIEANEAFAAQALAVKNELQIDSSKLNVNGGAIALGHPIGASGARILVTLIYEMQKRKVETGLATLCIGGGQGISMVVSR*

>CD630_26770 Clostridioides_difficile_630_NC_009089 acetyl-CoA--acetoacetyl-CoA transferase subunit alpha

MNKIVSIDEALSHVKDGDTIMVGGFMANGSPENLIDYLCSKNIKDLTLICNDTGFIDKGVGKMVVNKQFKKIIASHVGLNKETGRQMNNNETDVELVPQGTLAEQIRAAGYGLGGILTPTGIGTLVEEGKQKIVVDSKEYLLEKPIYADVALLFASKVDKAGNLVYKGSMNNFNNLMASAAKITIVESDEIVEIGDIDPNEVNTPGIFVNYIVEGGNL*

>CD630_26780 Clostridioides_difficile_630_NC_009089 succinyl CoA:3-oxoacid CoA-transferase subunit B

MDKLEMQEYIANRVSKELKDGAVVNLGIGLPTKVANYIPDNVNVILQSENGFLGLGSAEDGNSSDETIVNAGGQPVTILPGGCFFDSATSFGIIRGGHVDITVLGALQVDKYGNIANYMIPGKMVPGMGGAMDLVTGAKKVIVAMEHTSKGSAKILNNCTLPLTATNAVDLIVTEMGVMEVTSDGILLKEINPAFTLDDVISATEAPLILSDSLNGKISVV*

>CD630_26890 Clostridioides_difficile_630_NC_009089 lipoprotein

LKKVVIIGGGPAGMIAASTACEKGYDVTLIEKNHKLGKKLAITGKGRCNITNACEIEELIENVPTNGKFLYSAFYTFTNDDVISMFNNLGVKTKTERGKRVFPESDKAFDIVNALERQLKSKKVNILLNSKVEKIISKNNKIEKVILNDKKEIKCDSVVVATGGLSYPLTGSTGDGYKFAISQGHTIIDTKPSLIGIEVQESFTKDLEKLSLRNVEIRVFNSKQKKVYSDFGELEFTRFGLDGPIIKSASCRMKDTRKENYTILLDLKPALDEEKLDKRVQKDFQKYTNKKFEKALDDLLPKKLIPIIINLSEINPNTVVHQISREQRKNLVHLLKNLKFTVKRYRPIEEAIITSGGVKVNEINSSTMESKLVEGLFFAGEVIDIDAYTGGFNLQIAFSTGYLAGFNC*

>CD630_26910 Clostridioides_difficile_630_NC_009089 hypoxanthine phosphoribosyltransferase

MYKVTGKMLTEEQIKEKVYELGKKIEEDFKGEDLLVVGILKGASVFVSDLIRCIDLDVNIDFMSVTSYGNSTESSGTVKILKDLDVDIEGKNVLIVEDIIDSGLTLSNLVAALKTRNPKSLKLCTLLDKPQRRKANIPVDYVGFVIEDKFIVGYGIDYAEKYRNLPYIGIVEDVE*

>CD630_27080 Clostridioides_difficile_630_NC_009089 shikimate 5-dehydrogenase

MEISGRTGLFALIGTPVGHSKSPVMYNYSFKKLDLDYRYLAFDITVDKVKEALLAIKTFNIKGANVTMPCKSAVTEYMDELSPAARIIGACNTIVNDNGKLVGHITDGVGYVRNLKENGVEVKGKKITIMGAGGAATAIQVQCALDGAREISIFNPKDDFYKRAEQTVENIKKDVPECVVNLYDLEDTNKLYEEIESSDILTNATLIGMKPYDNETNIKDTSVLRKDLVVTDVVYNPKKTKMIEDAEANGCKAIGGLGMLLYQGAEAFNLYTGLEMPVEEVNELCFK*

>CD630_27140 Clostridioides_difficile_630_NC_009089 UDP-glucose 4-epimerase

MAVLVAGGAGYIGSHTAIELLESGYEVVIVDNLSNSNSIVVDRIKELSKKPVKFYNIDIRNKDEMHIVFKENNIESIIHFAALKAVGESVEKPIEYYSNNLISTLNLFELMREYGVKKFVFSSSATVYGDPHTCPILEDFPLSVTNPYGRTKLMIEQMLVDISKADKSLDIALLRYFNPVGAHKSGRIGEEPNGVPSNLMPYITKIAVGKLKELSVYGNDYPTHDGTGVRDYIHVLDLAAGHVKALQKLEENPGLVVYNLGTGKGYSVLDLVKAFSKASGKEIPYKIVGRRAGDVAMCYADSSKAEKELGWKAKYELEEMCEDSWRWQSMNPNGYEE*

>CD630_27390 Clostridioides_difficile_630_NC_009089 aspartate--tRNA ligase

LETLKGLKRTHYCGELREKNINEEVVLMGWVQKKRNLGGLVFVDLRDTSGLCQIVFDTDVDKEAFEKAEKLGAEFVIAVKGKVCERQSKNPNMPTGDIEIFATELRLLNKSETPPIYIKDDDDVSEALRLKYRYLDLRKPSMQRNLKLRHKVMNITRNYLSNNRFCEIETPFLIAPTPEGARDYLVPSRVNPGKFYALPQSPQLYKQLLMVSGMDRYFQIVKCFRDEDLRADRQPEFTQIDCEMSFVEQEDVMSMIEGLLEAIFKEVLDVELALPLPKMTYAEAMSKYGSDKPDTRFGYELTDISDVVCNCGFKVFADATQPGKSVRGINVKGKADDFTRKQISSLEEHAKTYRAKGLAWMKVGQEGVTSPIAKFFNEEEMNAILTRMNAEVGDLLLFVADKNSIVFDALGQVRLEVANRLNLLDKNVYNLLWVTEFPVFEEDEETGTFSAMHHPFTSPMDEDLDKLEEGDKSSLRAKAYDIVLNGYEIGGGSVRISNSDVQSRMFKALGFTEERANEKFGYLLEAFKYGTPPHAGLAFGLDRLVMLLAGTDNIREVIAFPKNQNAVCPMTNAPTLAEDEQLEELSIKVDIKDNE*

>CD630_27400 Clostridioides_difficile_630_NC_009089 histidine--tRNA ligase

MLTKAPRGTKDITPKEAYKWRYVENKFREICALYGYEEMVTPIFEHTELFKRSVGDTTDIVQKEMYSFKDKGDREITLKPEGTAGVVRAFIENKLYADTQPTKLFYVTPCFRYERPQAGRQRQFHQFGIEALGSDTPSMDAEIIALAVQFFNEVGLNDLVVSINSVGCPVCRKEYNALLKEYLDSKADILCDTCNERREKNPMRVIDCKNPTCKENIKDIPFIADHLCDDCKSHFDKLQEYLKEMNINFVIDKTIVRGLDYYRKTAFEIISNDIGAQSTVCGGGRYDGLVEQLGGPKGISGIGFGLGIERLLLTLEGNGIEIENPQSTDIFIVTIGEEANTRSFKLLKDLRQNHISADKDHIERSVKAQFKYSDKINSKFTIVIGDDELKNDTATLKNMKTSEQTTVKLSTLVEELKQKL*

>CD630_27410 Clostridioides_difficile_630_NC_009089 coproporphyrinogen III oxidase

LMLYVFLKGHDYKYEVAELIKLFTSEFKFKDSNANNNIESKHLINRLIYENGVLFSSTEYYENGNLKYESIQNIENMNLEFLDGFDSSKTIEEFLNSLDSESKRNFKKLCKENIKKSMFVVLKQVFNSYVPWGILTGIRPVKIVHNLMDKKLDDNSIRTILKDNYFIIDEKIDLALEIAKRERLFMYPIDKNKISLYVSIPFCPTRCVYCSFPSNSLKQFGHLKREYVYKLIEEIKGFAKVIKETKKEVETLYIGGGTPTTLDEEELDLLINSLFNELDLSKIKEFTVEAGRPDTITEQKLRVLKKHNVSRISINPQTMNDDTLVKIGREHRVSDLVDCFNMARRMGFDNINMDIILGLIDEDLNMVKNTLEEIKKLSPESLTVHTLAIKRASNLNINMDRYKEHLTQYEEMVKMIDLSMKYAKDMGLNPYYMYRQKHMLGNLENIGYVKEGYECIYNIQIMEEKQSNYALGAGAISKFVYVDEDRIERVENVKNVEQYIERVDEMIKRKKEEVYKNVD*

>CD630_27430 Clostridioides_difficile_630_NC_009089 D-tyrosyl-tRNA(Tyr) deacylase

MRAVVQRVSSSKVTVDENTIGQINKGLLVLLGVTHDDKSSDVDYMIDKILNLRIFEDENDKMNLSLMDIGGELLVVSQFTLYGDCRKGRRPGFSNAARPELANNLYEEFVKKAKDKGVTVGTGQFAAHMMVELTNDGPVTILLDSSKSF*

>CD630_27450 Clostridioides_difficile_630_NC_009089 adenine phosphoribosyltransferase

MDLKNFIRNIDDFPKPGIDFKDVTTLFKDGDAFKYAVDSIVEELKDKDVDLVIGPEARGFLMGTPVAYALGVGFVPIRKPGKLPGEVESYEYGLEYGTDTLEIHKDAIKKGQKVAIVDDLLATGGTMEAAAKLVEKLGGEVVSMQFLIELKFLNGREKLSNYDVNSLIKY*

>CD630_27470 Clostridioides_difficile_630_NC_009089 ubiquinone biosynthesis protein

LRISYRNLKRYREIGYVLIKYGFSFIVERLNIEGIAYKIPLFDPPEEIKNMTTGERMKRVLEELGPTYIKIGQILSTRKDLLDQDIIDEISKLRDDVEKFDSNIAIDIFKEEVGLSIEEIFLEFKEEPIAAASIGQVYEGVLKTGEEVIVKIQRPNIEKIIKSDLEILRTIANTLKDLKKDFNLDLVQMIEEFQTQLMRELDYTFEAINATKFSRIFKNSDEVYIPKVYSEYNTKKILVMEKVNGTKLSDVEKIRRLGYNTKTIVEIGVRSFFTQVLSHGFFHADPHPGNIFVVAKNKIAYIDFGMIGIIDNKTLNQLNEIALAGVEKNVDKIIYLLIEMDALNGEADIKGLRQDLLYLIHYYYDISIEKINVTDILNELFRFFRQYKIVMPAQFVTLAKTVITLEGTSRTLNTDFSFGSMGKEFMKHHYKSKFNPKNVVLSSRQNVEEILLDIKTIPKQLKAILKNIERNNIKMQIEDVKMTRLENCIIELTSQISLSLVLASIIVGSSLIIASPNIENNIWIKFTAIAGFFISFIIGLCLVIRSIRSKYKKD*

>CD630_27620 Clostridioides_difficile_630_NC_009089 undecaprenyl pyrophosphate synthetase

MRIPKHVGIIPDGNRRWASQNGMTKDKGYESGIDPGLESYRLCRDLGCEEVTFYGFTTDNTRRPSVQTKAFTKACIDAANILIDEGAALLVVGNTKSPMFPEELLPYTNRIKAKEGQIKANLLVNYGWYWDLNNISGVENVNKNNIQDYIQSFDVSRLDLIIRWGGRRRLSGFLPVQSIYADFYVIDNYWPDFKSDDIKDAFSWYDTQDVTLGG*

>CD630_27710 Clostridioides_difficile_630_NC_009089 UDP-glucose 6-dehydrogenase

MKIAIAGTGYVGLVTGVCLAEVGNENVVCVDVDEQKVKSMKLGIAPIYEEGLEELMVKNYNRGRIDYTTDYKKAYEDVDVILIAVGTPERSDGSANLDYIKAVAKQIAETVTKDTLVVIKSTVPIGTNEEIEDYINRNLKNDVKVELASNPEFLSQGTAVKDTLHASRIVIGVESEWAREILEEIYKPFNQPIIVTNRNSAEMIKYASNNFLALKISFMNDIANLCEIAGANIEDVALGMSYDDRIGSKFLKAGIGYGGSCFPKDTKALHWLSEHNGYELKTVKAAIEVNESQKLKLVRKASKLVESFQDMKVAVLGLTFKPDTDDIREAPSIPNIKYLLERGARVTVYDPIGVNNTKKIFGDSILYADSVDDAIEDAEICFIMTEWKDVLNYDIHKYKEKMKNPLVLDGRNCYKIKDMEELGIEYYSIGR*

>CDIF1296T_02918 Clostridioides_difficile_ATCC_9689__DSM_1296_strain_DSM1296_CP011968 phosphomannomutase/phosphoglycerate mutase

MDYKNNYEMWLNSPYFDEQTKNELLSIKDDEKEIQDRFYKNLEFGTGGLRGIIGAGTNRINIYTVRRATLGVLNYIMKTQGEEGKQKGIVIAHDSRYMSREFCIEVAKTLSAYGVKAYIFEELKPTPELSFAVRYLKCAMGIVITASHNPKEYNGYKVYDSDGGQICIDMANDIIAEVNKIDDYSTIKSIDFKEALSKNLITILDNEVDDEFIKAVKKQVLRQNIIDEYGKKLKIIYTPIHGTGNKPVRKVLNECGFENVMVVKEQELPDSNFSTVKYPNPEEKSVFNIAIEMAKNNGTDLIIGTDPDCDRVGIVVKDSSGEYVVLNGNQVGSLLVRYILESLVEENKLPKNNPTIIKTIVTSELGAKIAKAYNVDCLNTLTGFKFIGEKIKAFEESNDRSFIMGYEESYGYLIGTHARDKDGVVSSLMICEMAAYYSSKGMNLYEALIDTYNKFGYYKEDLKSVTLKGIDGIKKIKEMMLYFRSVKIDNVADVKVDKILDYKDSVDDLPKSDVLKFLLEDGSWIAIRPSGTEPKIKFYFGANSDNQEDVEFKLNNLISYILNVVDSI*

>CD630_28020 Clostridioides_difficile_630_NC_009089 queuine tRNA-ribosyltransferase

MYAVRYELIKTCKQSGARLGRLHTPHGIIETPIFMPVGTQATVKSMTPEELKEIGSQIILSNTYHLYMRPGHELIKRAGGLHKFMNWDKPILTDSGGFQVFSLGPLRKIKEEGVEFRSHLDGSKHFLTPEKAMEIQNALGSDIMMAFDECAPYPSDREYVKNSLERTTRWLKRCKDAHNNTDKQALFGIIQGGMYKDLREQSAKEITNIDLPGYAIGGLSVGEPKPLMYDVLEHTTPLMPKDKPRYLMGVGSPDDLVEGVIRGVDMFDCVLPTRIARNGTAMTSQGKVVVRNATYAEDFTPLDPECDCYACKNYSRAYIRHLIKANEILGARLITTHNLHFLLNLMKQIRQAIMEDRLLDFRNEFFAKYGYEI*

>CD630_28050 Clostridioides_difficile_630_NC_009089 Holliday junction ATP-dependent DNA helicase RuvB

MQGFEDENRIITSTMKMEDIDIENSLRPKTLEDYLGQEKSKEQLSIFIEAAKSRNEQLDHVLLYGPPGLGKTTLASIIANEMGVNLRITSGPAIERAGDLAAILTNLNENDVLFIDEIHRINRSVEEVLYPAMEDFCLDIIIGKGPSARSIRLDLPKFTLIGATTRAGMLTNPLRDRFGVICKLDYYTVDELSKIVLRSSSILDAEIQSNGALELAKRSRGTPRIANRLLKRVRDFAQVRADGKITDKVAKDALELLGVDSLGLDFVDEKLLMTIIEKFRGGPVGLDTLAASIGEDRNTIEDVYEPYLLQLGFINRGPRGRVAMPLAYEHLKIPYPNEK*

>CD630_28330 Clostridioides_difficile_630_NC_009089 calcium-transporting ATPase

MRYYNKPTKEVLKYLKTNPEIGLDDNEVEERKLRYGLNEFTIKEGRTFWDELGESLTEPMILILIGAAVISSFVGELHDALGILGAIFIGISIGIITEGKSKKAAHALSKLTENIEVKVLRNGKIIKISKNDLVPGDIVYIETGDMIPADGRLIQSINLKLREDMLTGESDDVAKNADAVLDMEVVYSKTEIIEQDAIPAKQVNMVFGGTLVAYGRGIMVVTHTGDKTEMGKIAQNLSNEDQQTPLQIKLGKLGAKIAGISGIIATLLCMFMIIQMQRKGMLILDTSSVLSFLQSLEPAKNAYMVCIALIVATVPEGLPTMINITLAITMQKMAKINALVTKKEACETIGSVSVICSDKTGTLTQNKMMVEVAYVDGKYISGGEYQSNSYFEQNCIVNSTADIEKEDNSFKYIGSATECALLLYHNDKNYNEMRKQTYLISQIPFSSEEKKMSTLIRQEDSDILLSKGAPEVLLKKCSYVQQGKNIVPITPKVEKSILDEIKKLQIKSMRTLGFAYKKMSNSKTEVAMTSEGELNLIGNSRSYMKEDNLVFSGFVGIVDPLREGVKDSIDKAFNAGVDVKMLTGDNINTATAIGNELGLLNDGKKAVEATYIDVLTDKELREEIKGISIVARSKPDTKMRIVSALQKSGEVVAVTGDGINDAPALSQADVGIAMGISGTEVSKNAADIILTDDSFSTIVEGIKWGRGIYENFQRFIQFQLTVNIVAFIIAIISQLTGKDMPFTTIQLLWVNIIMDGPPALALGLEPVRDYVLKRKPINRHSGIIARSMFVNIIINAILIITIVFTQSAFNILGATSEEQGTVIFSLFAFSALFNALNCREFGLNSTIPNFFKNKLALQIIVVTGIIQIIFTQVFQSFFNSVSLDFDMWIKIILFASTILLSNEFVKLILRTMKNSRTMNFNSKN*

>CD630_28340 Clostridioides_difficile_630_NC_009089 serine hydroxymethyltransferase

MLKTTKISDPELYKIVADELVRQEHNIEMIASESTAPTEVLELSGCVFTNKTEEGYPGARFQAGSEEADKLETLAIKRAKEVFGAEHVNVQPYSGSTANYCVYSSILKPNDTVLSMRLDQGGHLTHGSAVNFLHDIYKYEFYGVDPNTGRIDYDALEAKAKECRPKLIIAGASSYPRLIDYERISKVAKEVGAYFMVDMAHVAGLVAAKVIPSPVPYADFVSSSTTKTFCGPRSGIVLCKAEHAKKLDKGVFPGTLGSIHLNTVAAKAFSLLYLSTDKFKKIMEQVVVNAQTLASELISHGFSIVSGGTDNHIVMVDLRSKNLTGKQFEKALEYVGITVNKNVIPDDPQSPFVTSGVRIGLTSISQRGLKEKEVIQIAGIMNKVAENIDNKEVLDECKAEAQELISKFPLYPEGYFED*

>CD630_29700 Clostridioides_difficile_630_NC_009089 thiolase

MKKVFILGGLRSHIGLKNGIFQFVQPELLGASVLKDLIKKYEIDKIDEIICGNAVGTGGNIARLMTLTAGVSNEVPAFTVDMQCASAMMSIDIAFSKVKSGQCDLIIAGGFESSSLQPMRTYHKNDKRYNTNNPNYTVAQFSPDDNSQNSMLEGAERVAELYQIEKADLDFWVKESHKRAKEAREEKILEDIISPINNSTCDEGIRDKMSQRLLDRMPSILGKETITNAANSCLINDGASFIIICSKKYLEHVKKKPKAKIINTCTIGTDATLSPTSAIQAMDKLLEIESINYLDVSAVEFNEAFAVIDVLFQRKYPELIDRYNIFGGALAYGHPYGASGAIIALHLLKALEKTKGRYGICSIAAAGGLGSALLLERV*

>CD630_29850 Clostridioides_difficile_630_NC_009089 ABC transporter ATP-binding protein

MKILTVNNLSKVYGKKIIFNALNDINFSIEDGEFVGIMGPSGSGKTTLLNMISTIDKPTTGTMELKGKNPLLLRGEELALFRRRELGFVFQDFNLLDTLTIGENIVLPLTLDKVSVKEQDERLNEVSTILGIKDLLGKRTFEVSGGQAQRTAIARALINNPSILLADEPTGNLDSKSSKVVMELFQKINKENKVTTMMVTHDPLAASYCSRILFIKDGSIYNEIYKGSSREQFYQEIMDVLTLLGGDN*

>CD630_29940 Clostridioides_difficile_630_NC_009089 ribonucleoside-diphosphate reductase subunit beta

MTFNLHKIHNAVNWNREEDGFTQAFWEQNVKQFWLPEEISVSKDIKVWNELSNKEKELYKKVLGGLTLLDTKQGNNGIPSMMSLTENLQRKAVLSFMGTMEEIHAKSYSSIFMTLLSNLEIDELFEWIETEPTLQRKADLVLAQYENTTNQEGLYLSMVTSVFLESFLFYSGFFYPLYLSGQGKMVASGEIISLILRDESLHGKYIGLLAQEIYDSFDKMDKEMLEEKMYSILYSLMENEIEYTNVIYRESGLEKEVVNFLKYNANRALENLGFEKLYTVDAINPIVLNGLSTETKTHDFFSTKGNGYQKGVYEELEDEDFII*

>PCZ31_RS06740 Peptoclostridium_difficile_strain_Z31_NZ_CP013196 ribonucleotide-diphosphate reductase

IMNWIELNNQVIIKNEQGKYQLEKDKEALSSYIEEFIQPKLRKFNNLEERLKYLIDEGYYSKEVINQYSIDVIKNIYDMIDKHKFEFQSYMSANKFYQNYALKSNDGKEILETYNDKVLIVALTLGNGNKDLALNLADKIVKQEFQPATPTFLNAGRKRAGEMVSCFLLSVEDSTEGISYAISSSNHLSKIGGGVALNLSKLRASGESIKDIEGAAGGVVGVAKMLEQSFSYFNQMGARQGSGAVYLTVFHPDFELLMDTKKINADEKIRLATLSLGAIIPDKFMELAEKNEVAYAFYPHTVYKKYGVSLDEIEMDKWYDKLVNDSDIRKKEINPRQMLTKIAQMQQESGYPYVVYIDTANREHTLKDVGIIKMSNLCCEIFQYQTPSEIEGYGGKNEWGQDISCNLGSLNIANVMDNKTIESTVETAIRALSFVADSTDIKPVPTVSNSNSKSHSIGLGAMNLHGYLVRENILYTSDDAIDFSNVFFAMVRYYSIKASMKIAIERNQTFEGFDKSEYVKGRNSKVLSKYYEQSYLPKSEKVRALFEGIYIPTKEDWTKLLDEVKEKGIYNAYLMAVAPTQSISYVQNATSSIMPITEPVEVRTYGDSTTIYPMPFLTNDNMLYYQSAYRMDMRKVIDLVATVQNHVDQGISTTLFVTDEKTTRDIARHYIYAYKKGLKSLYYTRTKMTRDTHECLVCSV*

>CD630_29970 Clostridioides_difficile_630_NC_009089 iron family ABC transporter ATP-binding protein

MLKTNNLSVGYNNKVVISNINVEVKNGEILCLLGSNGAGKTTLLRSLSKLISPIKGEIYLNGVNINCISRKALSKKMALVLTNRLLGDLMTVQDIVNIGRYPYTGFFGSLSKKDLIMVDEALESVDALHLKKRYFDELSDGEKQKVLVARALVQEPEIIILDEPTTHLDIKHRLELINILKKLSKEKSISVILSLHEIDIALKSCDKVALIKNNKVIAYGQPEDVVDEDIINSLYELDDKNFNSLLGSVEISNKSKNEVFIIGGGGKATPIYRAFTKKGIGLYSGIIHENDIDYEIGRTMGIKMFTENPFEPISDESFDLAIRNLNDSKIIIDTGFSVGETNKRNIDIIKEALKLDKKVYSFRNRDESKKYYDSLDNKIEHIDKVSQIINSADINNLL*

>CD630_30090 Clostridioides_difficile_630_NC_009089 pyridine nucleotide-disulfide oxidoreductase

MKKTFDAIIIGFGKGGKTLAGDLANRGLKVALIEKSNKMYGGTCVNVACIPTKSLENSANSVKTKNINSWDEVQAEYEKAIDKKETLITKLREANYNKLNSNENVTIFTGMGTFIDEKTVQVKTENEIYELVADNIFINTGSRPFIPNIKGIENKNIVYDSESLMNLRTLPKKMTIIGAGFIGLEFAGIYSSFGAEVTILNSNNGILPNEDVEDSEEIIKLLAKRNVKIVNNANIKEIKEVSELAIVEYEVDGKSKELTSNMILVATGRKANTEGLGLENAGIELNERGFIKVSETLKTNKEHIWAIGDINGGPQFTYISLDDYRIVINQLFGDKTRTTNDRKNIPNSIFISPAFSRVGLNVKQAKEKGYEVLVAKMPVEAIPRAKQIGKADGFIKIVIDKKSNKILGASMICENSSEIIHLIQLAVDLEVEYTYLRDRVYAHPTMTEALNDILSPNMIKEV*

>CD630_30540 Clostridioides_difficile_630_NC_009089 two-component response regulator

MYRILLVEDDIDLSKEIALALEKWGFKVELIDDFEVVLDEFIDKKPDVVLLDVNLPLYNGFYWCEKIRAISNVPLIFLSSRDSDMDLIMGINNGADDYITKPFSIEILVTKINGIIRRVYNYSDSNSILYCEDLMFDVGKGIIKHKYKDKSIELTKNEIKILTLLLKNKNRVVSRESLMMTLWDNDEFVTDNALTVNMNRLRSKVKELGFDDFIKTKKGIGYIIQC*

>CD630_30560 Clostridioides_difficile_630_NC_009089 ABC transporter ATP-binding protein

MKEILKIKNISKDYGIKGFKTNVLKNISLTVNEGDFIAIMGPSGAGKTTLLNLMSTLDKQTSGEIILDGINISKVKNNELSKLRREKIGFIFQDYNLLDNMKLMDNIALPLALGKKKSKEIEAKVFSIAKKFGLENHLDKYPYQLSGGQKQRGAAARSLITNPTVIFADEPTGALDSKSAYELLESLEKINRENNATIIMITHDPLTASYSNEVYMINDGNIKCKLNKGNSRKEFYGKIMDMLASMGGEM*

>CD630_30650 Clostridioides_difficile_630_NC_009089 xylulose kinase

LEYVLGVDIGTSGTKTVLFDKLGNTIKSCTYEYPLIQEKSGWAEQDANDWWKAVVESIREVVQSSNISSECIKGIGLSGQMHGLVMLDNEGKTLRNSIIWCDQRTVKECEEITDLVGEERLIEITANPALTGFTASKILWVRNNEPDIYKNTNKILLPKDYIRYKLTGEYATEVSDASGMQLLDIRKRDWSDEVLEKLNIDKNLLGKVYESQEITGYVTRDVASLTGLKEGTIVVGGAGDQAAGAIGNGIVKDGVVSSTIGTSGVVFAYTKEPKIDKEGRVHTFCHAIPNTWHVMGVTQGAGLSLKWFKDNFCQSEVEVSNSLGEDVYEIINNQVSQVPTGCNGLLYLPYMMGERTPHLDPYARGVFFGLSPIHSKKEIARSIMEGVSYSLKDCMDIIENLNIDVNEVRASGGGGKSKVWRQMQADMFNQDVYTINSSEGPALGVAILALVGAGIYENIQKACDAIIKTSTKLEPISENVDTYNIYHRLYKRIYKSLKDDFKLLDEVVNAKINN*

>CD630_31230 Clostridioides_difficile_630_NC_009089 Fe-S binding domain-containing protein

MNMKYQHIIEVDKELCIGCGLCKNDCPVNNIIIENKKSVIKKQDCLMCGHCAAICPTKAITLTGFDEPPIELTNKPKLDSDELLMAIKSRRSIRKFKDKEVSSEIIKQIIEAGRYTPSAKNSQDVSYIVLDNKKSIYENEAVKFFRKIKPIANIAIKYSKEVEIDDNFFFKHAPIAIMIITKDKISGSLAASNMALMAESYGLGVLFSGYFSDVANNSPKLKKLLGLKRSNHVLTTLVIGYPDVKYRRTAQKEVATVRYL*

>CD630_31240 Clostridioides_difficile_630_NC_009089 6-phospho-beta-glucosidase

LTMSFKNDFLWGGATAANQCEGGYNEDERGLANVDVCPTGKDRTAVITGKLKMFDFDDEHYYPAKTGIDMYHNYKEDIKLFAEMGFKVYRMSIAWSRIFPKGDEETPNEKGLQFYENIFKECKKYKIEPLVTITHFDCPMHLVKKYGAWRNRKMIGFYEKLCNVIFRRYKGLVKYWLTFNEINMILHAPFMGAGICFEEGEDVEKIKYQAAHHELVASAIATKIAHEVDSNNLIGCMFAAGSVYPYSCNPNDVWEATKLDRENYFFVDVQSKGKYPNYALKYMEQKGITPEMEPGDIELLNKYTVDFISFSYYNSRCVRTDENADDMAEGNIFTSAKNPYLSYSQWGWPIDPLGLRITLNHVYDRYEKPLFIVENGLGAKDIADENGYVEDDYRIDYLREHIKAMHDAVSIDGVDLLGYTTWAPIDLVSAGTGEMEKRYGFIYVDRDNSGNGSLRRMKKKSFEWYKKVIASNGEDLY*

>CD630_31350 Clostridioides_difficile_630_NC_009089 fructose-1-6-bisphosphate aldolase

MLINMKEMLKVAQENQFAVPAFNIGSGQILKAVVQSANEKNAPVILAIHPNELSFLGDSFVASCIEEANKSKVPMVIHLDHGENKEQILRAIRCGFTSVMIDGSHLPYEENVAISREIVEIAKGLNVSVEGELGTIGTTGTSSEGGTDEIIYTDSKLAKDFVEKTGVDTLAIAIGTAHGIYPKGFKPELKLDLLKEIREVVDIPLVLHGGSSNPDEEIAQAVKLGVCKINISSDVKSAYYKKCRELLEQNPSLYEPDTIYPPCIKSAREVIEFKMNLFNAIDKLKCFYKNKN*

>CD630_31640 Clostridioides_difficile_630_NC_009089 ribonuclease R

MIPGLKESLLGLINESAYNPLKKEELAEIFDIHSSEMPMFYNFLNELEEDGYICFTKKGKIVSPNQMGYFVGKFVSHKKGFGFVESDVEYTQDLFISSDNINGAMHNDRVMAEIVVPATEEKRAEGRIVKVIKREITRIVGTFQSSKTFGFVTPDNKKFTKDIYIPKKHFSGAVDNDKVVCEITVWPQEDRKPEGKIIEILGQKGERGVEIDSIIREHGLPEEFPKKVLQEAEAVAVEIPQEEIKRRRDLRDLNIFTIDGDDAKDLDDAISIEVLPNGNFKLGVHIADVTHYVREKNKLDKEALKRATSVYLVDKVIPMLPKTLSNGVCSLNPFEDKLTLSIFMEIDHKGNVVKHEICESVINSKARMTYTEVSDILEKDDEKLKKTFEHVVEDFKNAEILARILMSRREKRGAIDFNFPEAKIILNGKGEVVDIKPYERRISNKIIEEFMLISNETIAEHFYWMGIPFVYRIHETPSLEKMEELSKFISTFGYIIKGDKEEVHPKALQGIIEKIKGKKEEGAISTIMLRSLKQAKYSPECSGHFGLAAKFYCHFTSPIRRYPDLQIHRIIKESLNNKISGKRQEQLTTIVDYASVQSSEKERKAELAERDVHDFYKALYMKDKVGQEFEGVVSSVTSFGIFVELDNTIEGLIRLANMNDDYYIYDESSYSVLGERTKKSYKIGDLVKIKVESVNVDFKEIDFEIVEKIEE*

>CD630_31710 Clostridioides_difficile_630_NC_009089 2,3-bisphosphoglycerate-independent phosphoglycerate mutase

MMKKPVALIIMDGFGYNKDVKGNAIAESKTPNLDRIKKEYPNTLINASGLDVGLPDGQMGNSEVGHTNIGAGRIVYQDLTRITKSIKDGDFFTNKVLCEAMDNAKENSLHVMGLLSDGGVHSHIDHLKAIIKMAKDKGVQKVYVHAFTDGRDTDPQSALEYAKEVQASMDEIGVGEFATVSGRYYAMDRDKRWERVELAYNAMVRGIGEKANSIEEAIQNSYDDGKNDEFIMPTVIMKDDKPVGSIKENDSIIFFNFRPDRARQITRALVCEEFDGFKREDIKNFFVCLTEYDITIENVHIAFGPQSLANTLGEYLAKNGKTQLRAAETEKYAHVTFFFNGGVEEPNKGEERLLIPSPKVATYDLKPEMSAYELTDKALDKLGEDKFDFIVLNFANPDMVGHTGSIEAAIKAVETVDTCVGKLIDKIVELGGSAIITADHGNAEYMLDPETGKTVTAHSINPVPFIVVGQEYESAKLLDGGRLSDIAPTILDMMKLEKPEEMTGHSLISK*

>CD630_31720 Clostridioides_difficile_630_NC_009089 triosephosphate isomerase

MRKPIIAGNWKMHKTIKEALEFVNEIKDKVNSDKVEAVICAPFTLLKDLKEATKGTNIKIGAQNMHFEEKGAFTGEVSPLMLKEIDMDYVVIGHSERRQYFNETDETVNKKVLKALEVGIDPILCVGETLEQREAGKTKDVCKVQVEKALENVLKDDLAKVVVAYEPIWAIGTGKTATAEDANDVISYIREVIKGLYGELANEVRIQYGGSVKPSNVAEIMGQSDIDGALVGGASLASNDYLDLVNF*

>CD630_31730 Clostridioides_difficile_630_NC_009089 phosphoglycerate kinase

MSMLNKKTIEDIDVCGKKVLVRCDFNVPLQDGVITDENRLNGALPTIQYLISKGAKVILCSHLGKPKGEAKPELSLAPVAKRLSEMLGKEVVFAADDNVVGENAKKATEKMENGDVVLLENTRYRKEETKNEENFSKELASLAEIFVNDAFGTAHRAHCSTVGAGEFLQERVCGYLIQKELKFLGEAVANPVRPFTAILGGAKVSDKLAVINELLEKVDNLIIGGGMAYTFLKAQGYEVGTSLLEIDKVEYAKEMMEKAKNKGVNLLLPVDVVMADHFAPDATPIVTEDANVKEDYMGLDMGPKTIANFVKTIKESKTVVWNGPMGVFEFENFANGTLSVARAMAELTDATTVIGGGDSAAAVNQLGFGDKMTHVSTGGGASLEFLEGKELPGIAALDNK*

>CD630_31740 Clostridioides_difficile_630_NC_009089 glyceraldehyde-3-phosphate dehydrogenase

MVKVAINGFGRIGRLALRKMMEQQDKFEVVAINDLTDAKMLAHLFKYDTAQGRFNGEIEVKEGAFVVNGKEIKVTAERNPADLPWAELGVDIVLECTGFFTSKDKAEAHIQAGAKKVVISAPATGDLKTIVFNTNSDILDGSETVISGASCTTNCLAPMAKVLNDKYGIEKGLMTTIHAYTNDQNTLDGPHPKGDLRRARAAAGNIVPNTTGAAKAIGLVIPSLKGKLDGAAQRVPVVTGSITELVCTLGKNVTVEEINAAMKEASNESFGYTEEMLVSSDIIGISYGSLFDATQTKVMEVDGKQLVKVVSWYDNEMSYTSQLIRTLGYFAQLAK*

>CD630_32010 Clostridioides_difficile_630_NC_009089 multidrug family ABC transporter ATP-binding protein

MIKVGELKFSYGKDKQILHGLNFEVKEGEIFGFLGPNGSGKSTTQKILNGVLKGYNGHVSIFGKEVKAYTESLYQKIGVLFEFPYLYTNLSAIDNLEYFSSFYPKNQRRDIYELLDLLEFKKEFINKPVSSYSKGMKQRISMARALVSNPKLLFLDEPTSGLDPSGAVLFRKIIEEERKKGTTIFLTTHNMLDADLMCNNVAFIADGKIMVIDKPKNLKMKNSNNKVEVEFVYNGNRDIEIVDIQELESGITFKYDEILSVHSKEPTLEEVFIKCTGRMLV*

>CD630_32030 Clostridioides_difficile_630_NC_009089 two-component response regulator

MAKILVVEDEKRMQNIIVEYMQKGGYTCITADDGVEALTILKSNNIDLMILDIMMPYLDGFSVCRVSREMTNIPIIILTAKGEEEDKLKGYEYGADDYITKPFSPKVLLAKVNALLRRYTTDIPKNSLSLGKIFIMVASRQVYVEDKLIDLTYKEFELLRLFMENPNQVFSREKLLNCIWGYDFEGNTRTVDTHIKTLRKKLGSEGHHIVTLIRSGYKFEVKE*

>CD630_32050 Clostridioides_difficile_630_NC_009089 nitroreductase

MISDSISKRRSIRKYKNQSISHETIEKIIEAGINAPSSKNRQPWRFVVITEKEKESMLKAMSKGIQNEINDNGLLPGSRQHIAGANYTVEIMKQAPVTIFILNILGKSPLEKLSPEERFYEMANMQSIGAAIQNMSLTAVELGLGSLWICDVYFAYRELCEWLNTDSQLVAAISLGYPDEEPSRRPRLQLSDVTEWR*

>CD630_32150 Clostridioides_difficile_630_NC_009089 glycine betaine/carnitine/choline ABC transporter ATP-binding protein

MTPIIQFKNIKKQYNDKTIIDNLNLDIEKGEFLTVIGSSGSGKTTLLKMINGLILPDGGNILINQTDIKNEDLIKLRRRIGYCVQGSVLFPHMTVEENISYVPNLLSKKNKLEVKSAVNKWMEIVGLPNDMKVRYPSELSGGQQQRVGIARALASSPEILLMDEPFGAVDEITRKQLQKEIKEIHKKTGVTIIFITHDIYEALILGTKTLVLNHGVVQQYDTPENILNTPANQFVDQLLNIRKSIIDDDNLKE*

>CD630_32170 Clostridioides_difficile_630_NC_009089 ABC transporter ATP-binding protein

MNNEFIILKGCKENNLNNISLKIPKRKITIFTGVSGSGKSSIVFETIAKESQRQLNERFSTFVRSFLPKYGEVKADCIENLSTPIIIDQSRLGGNSRSTLGTITDVNSFLRALYSRFGSEYIGNANMFSFNNIDGMCPHCHGLGKKLVPNMNQILDMNKSLNEGAILLSGFGVGSWHWNIFAESGFFDINKRICDYSEVELEKFLHGKAEKIKIENAGQTNMTYEGLMVKFNRLYLSREGEISEATKKKLSKLLIEDRCPICDGRRLNERVYRSLINGYNIADLTSMQIDELAEVIKSIDEPEAQPLIKGIIEKLNSIIDIGLGYLTLDRETSSLSGGESQRIKMVKYLNSNLVDLMYIFDEPSVGLHPKDVYKLNNLLKKLRDKGNTIIVVEHDPDVIKIADHIVDVGPKAGKYGGEVVYEGSYENLLTSGTLTGNALSKFLPIKESVREHSGYLEVKNCNKNNLKNVSIKMPKGVLTLVTGVAGAGKSTLIKDEFLKQNPSAVLIDQSPVSANSRSSLATYSGIMNNIRSIFSKTNGVNASLFSSNSEGACDNCKGSGIVEMNLAFMESIKSTCNVCEGKKFKKEVLEYRFQGKNIIEVLEMSVLEAIEFFNLKQIKTKLQCIEKMGIGYLTLGQTLDTLSGGECQRLKLASELNKESSVYILDEPTTGLHMADIENFISIIEEIVDNGNTVIIIEHNIDVIKRADWIIELGPEGGTKGGRVIFEGIPKQLCNSKISLTAKYIV*

>CD630_32190 Clostridioides_difficile_630_NC_009089 Hsp33-like chaperonin

MRDYVLRATSGNGQVRAFVATTRNTVEEARRLHETTKVATAALGRTLTATSIMGLMMKNDSDKLTVIIKGGGPIGTIIATSDSKGMVKGYVGNPQVEVEDYPNGKLNVAAAVGTEGVVKVIKDLGLREPYNGTYPLVSGEIAEDFTYYFAVSEQTPSVVALGVLTKEDEVEFAGGFIVQLMPDAEEETIAKLEENVAKLPSITNMLKEGKSPEDILNIVLDGLEPKILDTCEVGFMCECSKERVKTALVAIGKKSLAQIIEEDKKAEVGCQFCNKKYMYSEEELLEILKEM*

>CD630_32250 Clostridioides_difficile_630_NC_009089 4-hydroxy-tetrahydrodipicolinate synthase

MIFKGSAVALVTPFTKDNKVDFDKLGELVEYQIANGTDAIVSCGTTGEANTMTDEEQLATIKYVVEKVNKRVPVIAGSGSNDTMHSVNLSQEAEKLGVDALLIITPYYNKANKAGLKRHFETIANSVKLPIILYNVPGRTCVNISPSLIVELAKIDNIVAVKEASGDLGQVAEIASLVPDDFAIYSGNDDTILPLLSLGGQGVISVLANVCPQETHDLVAKFFEGDIEGSRKLQLGMDALIAALFIEVNPIPVKTAMNLLGFNVGDLRLPLAEMDPANLEVLKKELVNFGLKVNA*

>CD630_32310 Clostridioides_difficile_630_NC_009089 hypoxanthine phosphoribosyltransferase

MDIETKKWEVLYSEEKIKDKLRELGAIIEKDYKDKNLMVVSLLKGSFIFCADLVRNINLPLRVNFMTTSSYGNNEESTGRVKVVSDVTTDIAGYDVLVVDDITDSALTMDFVLKHLKAKNPASLKCCVLLDKPSRRKVDLVPDYCGFEIEDKFVVGYGFDFGDYYRNVPYIFNVTDEDR*

>CD630_32530 Clostridioides_difficile_630_NC_009089 folylpolyglutamate synthase

MKYEEALEYISQTNKFGIRLGLENIGKLLELLGNPQETLNIIHVAGTNGKGSVCSFVSNILRECGYKVGLYTSPYLETFTERIRVNGQNIPQDDVARIIELIKEKIEIMVKEGYAYPTEFEVVTAMAFYYYSEQKVDFVALEVGLGGRYDATNIITKSLVSVIVSISLDHTGILGDTIEKIAYEKAGIIKENGVVLVYDQTDEAKDVIKSVCKEKKAKYIEVDFDDINIKKSDINSQIYDCTVMKETYRDLEIKLIGEHQINNSILAISVIKYLKDINKLANINEESIRKGLINTKWPGRIEKIKENPIFIIDGAHNEDGAKSLAKALDKNFKGRKLTLLIGMLEDKDIDSVLEILLPHFNKVITTTPSNPRAINSDILREKVLKYVDDVTSKHEIEDAVNYTLETSSEDDIIISAGSLYMIGTVRTLVKKL*

>CD630_32560 Clostridioides_difficile_630_NC_009089 valine--tRNA ligase

MENTNLSKTYNPKDFEARLYKKWMDEGYFKSKPNPDKKPFTIMMPPPNITGQLHMGHALDHTLQDILIRWKRMDGYEAFWLPGTDHASIATEVKVVERIKKQEGKTKYEIGREEFLKRAWEWKDEFGGKISNQLKQLGDSCDWDKERFTMDEGCNEAVIEFFVSLYEKGHIYRGNRIINWCPDCKTTLSDAEVEHEEHDGNFYHIKYPLKDSEDFLEIATTRPETMIGDTGIAVNPEDDRYKHLIGKTAILPLVGRELPIVADSYVDLEFGTGAVKMTPAHDPNDFEVGLRHNLEQLNTMNEDGTMNEVCGKYEGMDRFECRKAIVADLKEQGYLIKIKEHNHNVGTCYRCHTVVEPRLSEQWFVKMEELAKPAIDILKKGELEFVPDKFDKTYLQWLENIRDWCISRQLWWGHQIPAYYCQECGEIVVARKMPDKCPKCGSTHFKQDEDALDTWFSSALWPFSTLGWPNKTEALDYYYPTSVLVTGYDIIFFWVVRMAFAGMFCMNEKPFDHVLVHGLVRDSQGRKMSKSLGNGIDPLEIIEQYGADALRFTLTTGNSPENDMRFYMERVEFARNFANKLWNASRFVFMNIDEDIIKNMTRESVKEDLTLADKWIISRANNIVKEATNNMDKFDLGIALQKIYDFTWSEYCDWYIEMVKPRLYGEDANAKSAALYTLTYVLEKILKLLHPYMPFITEEIYTHLPTVEGCIIVSEWPKYNEEDNMAEEEDMMNLLMEGIRSIRNVRAEMNVPPSKKAKLIIIPSEEKIEAIELGKDYFITLASASNVEIAKDKSNVPEDAVGVVIDGVEIFIPLNELVDFEKEIERLSKEKKKLEGEIKRVNGKLANQGFLAKAPESLIEEEKAKKEKFEEMIKSVEERLTNLESKIK*

>CD630_32610 Clostridioides_difficile_630_NC_009089 phosphate ABC transporter ATP-binding protein PstB

MELIDKIKMSVKDLDLFYGDKQALKKINMDIKENKVTALIGPSGCGKSTFIRTLNRMNDLIEDVTIKGNISVDGEDIYTSDDVINLRTKVGMVFQKPNPFPMSIYDNVAYGPRTHGLRDKKQLDKIVEESLKGAAIWDEVKDRLKSSALGLSGGQQQRICIARAIAMRPEVILMDEPTSALDPISTLKVEELIEDLKKDYTIVIVTHNMQQAARISDETAFFLNGEVIEFSDTKTMFTTPVDKRTEDYITGRFG*

>CD630_32830 Clostridioides_difficile_630_NC_009089 pyruvate formate-lyase

MNPLVINLQKCSIHDGPGIRSTVFFKGCPLECVWCHNPESQTYTKQVLYNEERCSKCEACINICPHKAIYKGETKICLDQDKCEFCETCLDYCVNNAREIVGQEYSVRDLVKEIEKDRIFYEESGGGVTLSGGEVMAQDMDFICGVINMCKSKGIHVAIDTCGYAKSENYERVAKCADLFLYDIKLIDEDKHIKFTGKSNDLILKNVKILSELGANINIRIPLIVGVNVDDENLEVKKMIEFLKPLNIQAVSLLPYHNIGKHKYDKIYKKYEGEELQRPSEEKLEEIKRLFEASNFNTKIGG*

>CD630_32840 Clostridioides_difficile_630_NC_009089 serine protease, HrtA family

MSRRKKGISLVILVAIISSILSSFLTIILVKDNLVSKSTGSSTPIVVNDDGKSQNIYQAVAEKATPSVVGITTTSVDTSNMFAIPTETQGVGTGIIVDSNGYILTNSHVISDGQATSVNVLFNDGSTTSGKVVWFDQQLDLAIVKVDKTGLTPAEFADSDKVKVGDISIAIGNPLGLDFQKTVTQGIISGLDRTIQTEKTNMTGLLQTDASINAGNSGGPLLNQKGQVIGINTAKASQAEGLGFAIPINTAKSIVEEVIKNGKYEKVTLGIKGTDVSNYEAATGTKLSTDKGVYVAEVISGSSAEKAGVKVGDIITKVGDTDITGMNDLNKKLYTFSKGASTKITVNRGGKAVTINVNF*

>CD630_32990 Clostridioides_difficile_630_NC_009089 major facilitator superfamily transporter

MKQKWIVLIIICIGVFMSTLDGSILNIANPTIAADFKINMSQIQWVVTAYMLVVTATMLFFGKLGDKVGSNRLYTLGFFIFTIGSFLCSMSNNLSTLISSRIFQAVGASILMATGLGIVSNAFPANEKGKAIGITGAVVGIGNMSGPVIGGIILEHFGWPSIFIINIPIGIIAVFLGIKFLPKPVLDEQNKSFDIPGLLLFASCTTLILLAMNEKGNTRLYLGITALIIFLLLALREVKFEQSFIDLPLFKNRNFTVGNIIGVACYFPQMAVSFLLPFYLEQLKNLSPMMAGYVMTVHPLIMVLIAPIAGSLSDKHGAKNILTASFSFMTISLVGMALLKADSPLYLLIVCLVIFGLGLGAFSSPNNSSILADVPPQKQGYGGSFLATIRNLSFALGTAFFSSFFAQSLTYNQKFKSHTSAYVIASNQSYWIAASVCFIGLILTVFFMRKTDKSIS*

>CDIF1296T_03417 Clostridioides_difficile_ATCC_9689__DSM_1296_strain_DSM1296_CP011968 ribosome biogenesis GTP-binding protein YsxC

MKIRSSEITMSAVNKSQYPAEGIPEIALAGRSNVGKSSIINTLLNRRNFARTSQTPGKTRTINFYLINNEFYFVDLPGYGYAKIAKSEKEKWGGIMERYLESRQELCSIFLLVDIRHEPTADDKLMYEWIKHFGYNCVVIATKADKISRGQYQKHISIIRKKLQMESSEKVIPVSSLKKTGVEELWEEIVNQYNQHGYEITVD*

>CD630_33040 Clostridioides_difficile_630_NC_009089 ATP-dependent protease ATP-binding subunit ClpX

MSKYEEKRQLKCSFCGKNQDQVRRLIAGPNVYICDECVELCDEIIQEEIEDTIDEDTTSLPKPKEMMEILNDYVIGQEKAKKALSVAVYNHYKRIYSKKSSSKDIEIQKSNILLLGPTGSGKTLLAQTLARTLNVPFAMADATSLTEAGYVGEDVENILLKLIQAADFDIEKAERGIIYIDEIDKIARKSENPSITRDVSGEGVQQALLKILEGTVANVPPQGGRKHPHQEFLKIDTTNVLFILGGAFDGLEKIIQKRGGDKTLGFGAKIESKKELDLGKLYEKVLPEDLLKYGIIPEFIGRIPVLATLELLDEDALMQILQEPKNALVKQYKKLLELDDVELEFEEGALRAIAKKAIERNTGARGLRSIVESVMMETMFEVPSRDNIKKVIVTEKSVNEDSVNPIIVLKDQEESA*

>CD630_33050 Clostridioides_difficile_630_NC_009089 ATP-dependent Clp protease proteolytic subunit 1

MALVPVVVEQTGRGERSYDIFSRLLKDRIIFLGDQVNDATAGLIVAQLLFLEAEDPDKDIHLYINSPGGSITSGMAIYDTMQYIKPDVSTICIGMAASMGAFLLAAGAKGKRLALPNSEIMIHQPLGGAQGQATDIEIHAKRILKIKETLNEILSERTGQPLEKIKMDTERDNFMSALEAKEYGLIDEVFTKRP*

>CD630_33590 Clostridioides_difficile_630_NC_009089 ABC transporter ATP-binding protein

MSLLKVTNLSQCFMDKSLYEKANFDLFKGEHIGVVGQNGTGKSTLIKILLGEVVPDSGEIKWQPNINIGHLDQYAEINRDTTISLYLHTAFEELYKIEKEMNLLYQKSAISENEQYLIKASDYQEQLIANNFYSIDNEINKIANGLGLDSIGMNRVVRELSGGQRAKVILAKLLLSNHDVLLLDEPTNFLDKEHVEWLSNYLNTFNGAFIVVSHDFDFLEKISTGILDIEFGMIKKYHGKYSEFLKQKSRLREDYIRRYQAQQKKIEKEETFIRKNKAGVNSKIARGRQKQLDKIERIAPPSFTGKPNIQFSEIEISAQNALTITNLEVGYYYSLLPKLNFSVDGGQKIVITGFNGIGKSTLLKTLVKDIPRISGDFQFSEQVKIGYYEQDLKWENPDKTPLQIVADKYPKLNTKEIRRHLARCGVKEEHVSRSVSTLSGGEQSKVKLCCMMLSPCNFLILDEPTNHFDAETKDALQNALKQFRGSIILVSHEEKFYKGWIDKVFNIEKQLV*

>AEC_RS0218755 Clostridioides_difficile_QCD_37x79_NZ_CM000658 23S rRNA (uracil-5-)-methyltransferase RumA

MLSKNEMYIVDIVDIGQGGVGVGKYNGITVFVEGGLIHDKVKVRINKVKKNYAEGDIVEIIEKSPFRVKRVCSDNLRDCGGCQIQDLDYNKQLEMKTNEVKQVLSRIGKLEDVEVHDTIGMDNPYRYRNKAQFPIQKNNGIPIIGFYKKKTHDVISTEKCVIQHEINDKIVKIIKTYIRAYKVSIYDEKTHTGLIRHLVTKIGFTTKEVMVVLVANGNKLPYLKELASVLKENIPGFKTLVVNVNQKDTNAILGRENIVIYGDGKINDYIGDLVFEISPLSFFQVNPVQTKVLYDKALEYANLNDTDTVFDIYCGIGTISLFLAQKAKKVYGIEIIDDAIKDAKINAKLNNIHNAEFYVGKAEEIVPKMYREGKTANVVVVDPPRKGCDEKVLETIVSMNPERIVYVSCNPSTLARDLDYLNVHGFKCVEAQPVDMFPHSTHVETVALLSKLDVDKHIDVEIKLDELDLTSAESKATYAQIKEYVLEKFGLKVSTLYIAQIKKKCGIELREHYNKSKKDNQAIPQCTPEKEEAIMDALRHFKMI*

>CD630_33940 Clostridioides_difficile_630_NC_009089 pyruvate kinase

MLNNVKKTKIVCTLGPASQSEEVLTQLMQNGLNVCRFNFSHGSHEEHKERIDMAKKVREKLNKPVAILLDTKGPEIRTGNFEDPEVFLEEGQKFTITMKDIMGTKEMCTVSYKGLAEDVKSGDSILIDDGLVGLRVKEINGEDIVCVVENSGIVKNHKGVNVPGVKINLPAITPKDISDIEFGISQGIDYIAASFVRKASDVLAIREVLENNNATDIQIISKIENQEGVENLDEILKVSDGIMVARGDLGVEIPTEEMPIVQKMMIKKCNELAKPVVTATQMLDSMIRNPRPTRAEVTDVANAIYDGTDAIMLSGETAAGKYPVEAVKMMATIAKRTEETLDYDRLLKENGTNNVTVTDAISHATCTTAVDLNASAIITSTSSGYTARMVSKFRPKSPIIATTNNEKTMNKLALTWGVYPVKSSVAGNTDEVIEKAIEAARQADYIDNGELVVITAGVPVGVSGTTNLIKVHVISEEIVQGIGVGTQTVEGKVRIIKNGNACVDFNEGDILVSTATDAEMNNYIEKAGAVVTENGGMTSHTAIVGINLDIPVIVSAADITNLVKDGEVVTVDASRGVVYRGSTRVL*

>CD630_33950 Clostridioides_difficile_630_NC_009089 6-phosphofructokinase

MKTIGLLTSGGDAPGMNAAIRAVVRSAIYYGCKVYGINRGYKGLLEEDLTEMNLSSVGDIIHRGGTILKSSRCEEFKTEEGRLKAVKILKKYKIDCLVVIGGDGSFAGAQKLSDLGFPAIGIPGTIDNDLAYTDYTIGFDTAMNTIIDAIGKIRDTSSSHERVNIVEVMGRHCGDLALYAGLAGGAETIIVPEVEITVDEVALRLKTTQKRGKRHSIIVLAEGVGSASDLEKELKKESGADLRVTVLGHVQRGGSPTVSDRILASRLGVRAVELLLDGKSARVVGIKENKIIDLEISEALAQKKVFDKEAYEMAKILSI*

>CD630_34080 Clostridioides_difficile_630_NC_009089 DNA mismatch repair ATPase MutS

MGIEYFSKLLESSTEKELLLSKKSNNISTARLISFLIVIAGFAIRFYNKNIVGIFVGVLSIIIFIALLVIHSKVKEEETYFKSKSEVLNKYIKRFGDGWKEFKIDGKEYLKDENSQAKDLDLFGRASLYQYICVANTSYGKNFLAKYLWNENPDENVILERQRAIKELLSKDDFSIHIQTLSNIIGKEQKNNSDSSIESFIEYGENKNVYIPKWMHIFTWGLPTATILSFIFCMLGFLPVLPVFLLFIIQLGFSGFGYPRLMQTLAPLFSFSRSIQVYEKMFEVLEKETFESAYLKELQDKLSKGSGVSRGVKQLNSIGNAVNLRYNQILYIIACGVLMWNYHCAEALERWKGIYGNQIRDWFESIGEFEALISLTVVSHVKENTCFPVIKYEDTPRLKVEEVYHPLIAEKSVIANSIQLNSQTCIITGSNMSGKTTFLRSIGVNLVLAYAGAPVCAKSFDATCMAIFTSMRIQDDVSQGISTFYAEILRIKSMIQYSLKELPMLVLVDEIFKGTNSADRIIGASEAVKKLSKPWVISMVTTHDFELCDLSGSGDVEVVNYHFSEYYVDDKIHFDYKIKDGRCKTTNAKQLMRMAGIL*

>CD630_34120 Clostridioides_difficile_630_NC_009089 excinuclease ABC subunit B

MDFKIKSDFKPTGDQPEAIKSIVDSINRNEKFSTLLGVTGSGKTFTMANIIQQVKKPTLIMAHNKTLAAQLYSEFKEFFPDNAVEYFVSYYDYYQPEAYVAHSDTYIEKDASINDEIDKLRHSATASILERRDTIIISSVSCIYGLGDPKDYKELMLSIRPGMQRDRDDVIKRLIEIQYERNDINFTRGTFRVRGDILEIFPASNDEKAIRIEFFGDEVDRITEIDYVTGKIVGTRNHVVIFPASHYVTTPERIEKAIVEIENELQEQIKFFKENDRLLEAQRIEQRTKYDIEMLKEIGFCQGIENYSRHITGRSEGERPYTLMDFFPDDYLIIVDEAHVTIPQVRGMYAGDRSRKTSLIENGFRLPSALDNRPLNFQEFEGNINQMLFVSATPGPYEIQHSETIAEQIIRPTGLLDPIVEVRPINNQIDDLVGEITKTIEKNERVLITTLTKKMSEDLTNYLKEIGIKVKYLHSDIVTLERTEIIRDLRLGKFDVLVGINLLREGLDIPEVSLIAILDADKEGFLRSETALIQTIGRAARNENGRVIMYADRITDSMQNAIDETKRRRDIQNLYNEEHNIIPKTIQKNIRDSIEATKVAEEEVVYGISDTDDKDEIRANIDKLKSEMMEAAQNLQFERAAELRDKVKQLEEKLEK*

>CD630_34170 Clostridioides_difficile_630_NC_009089 sugar family ABC transporter ATP-binding protein

MADIRLENVTKSYNKKNTVIENLNLTIEDGSFTVLVGPSGCGKSTTLRMIAGLEDITSGRLKIEENDVTKTDASKRDIAMVFQNYALYPHMTVRENIEFGLKNKKVEKEKREKLITEVIDVVGLREYLNVKPGNLSGGQRQRVALARAMVKNPKVFLMDEPLSNLDAKLRNQMRTELIQLHQKLKSTFVYVTHDQVEAMSMADKIVIMNKGKIMQIGSPKKIYQNPKNLFVAQFIGSPCMNILDIKNDIKFGFRPEKATLESINIDGKNDDVFSLFGKVVTKEVLGNETIYCLSVLNREIRVKTENDIFDIGRTLRISVLEKDLYYFDKNGERIEDVKVCKSMYSKIKGDIYEKAI*

>CD630_34210 Clostridioides_difficile_630_NC_009089 porphobilinogen deaminase

MNIVVGTRGSNLALIQTEWVINELKKKYPEISFEIKIIKTKGDLIQNVSLDKIGDKGLFVKEIEQQLLDGKIDIAVHSMKDMPSYLANGLKFAHTPKREDPRDVLILREGYKNLDDLPHGAVIGTGSKRRKFQLLKQRPDLNIVQVRGNVETRIRKIKDENMHGIVLAASGIIRANLQDKISSYLPVDVVIPAPAQGALAIEIRSNDSAIEGIVNSLKDENTEIQILAERGFLDGVNGSCHIPMAAYCEIIQDKIHLTGLYGDSEGKKVVIKSIDGDISSPRELGLKLAKLVLKEYENYEG*

>CD630_34490 Clostridioides_difficile_630_NC_009089 phosphosugar isomerase

MFKLEEQKLKDLGAIITTNEIKQQPELWLETYEIYKSNKEKLSRFIDTISNNHGQFRVIFTGAGTSAYIGNSILPYLKNKNDIRKYIFEAIPTTDIVSNPYDYLKKDIPTLLISFARSGNSPESLAALNLGNKIVDNFYHLAITCNPEGELAKMTKNDENNYLLLMPSKSNDEGFAMTGSFSCMMLSAMLIFDSLEDDVEKSYINAIIEMGRNVIDRKDEIHELINKDFDRVVYLGSGGLGGLTQEAQLKLLELTAGKISTVYDSPMGFRHGPKSFIDENTLVFEFVSNCLYTRKYDLDVLEEIKRDKIAKFTCAVSVENENNFSGTKFEFKEKYNKLPDVYLAMPYILFAQTIALFVSVKVGNKPDTPSATGTVNRVVKGVTIYEY*

>CD630_34590 Clostridioides_difficile_630_NC_009089 transketolase pyridine binding subunit

MSKMATREAYGKALVKLGQINDNVVVLDADLSKSTKTHDFYKSFPDRFFNMGIAEQNLIGAACGLSTAGKIPFASTFAMFATGRAFEIIRNSVCYPKLNVKICATHAGLTVGEDGASHESVEDIAIMRAIPNMTVLVPADGVETEKIIFEIAKYNGPVYVRLGRSSVPVLFDEDYKFEIGKGTVLREGKDVSIIACGIMVNEALLAQEKLQEEGISARVINMSSIKPIDKDLILESAKETNAIVTVEEHSIIGGLGSAVSEVVGESCPTIVKKVGIKDTFGESGTPNELLKKYELTCDDIIKTVKEAIIAKRM*

>CD630_34600 Clostridioides_difficile_630_NC_009089 transketolase thiamine diphosphate-binding subunit

MRDHKGLNEIARIIRRDIVSMIHRAKSGHPGGSLSVVEILTALYFDEMNVDSSNPKMEDRDRFVLSKGHAAPALYATLAEKGYFDKEELNGLRKIGRMLQGHPDMKGTPGVEISTGSLGQGFSVACGMAMASKLDNAPWNVYTLLGDGEVQEGIVWEAAMSAAHYKLDNLIAFLDNNGLQIDGDIESVMSLGSIVDKFKAFGWNVIEIDGHDFDQIFAALDIAKSTVKKPTMIVAKTIKGKGISFMENQAGWHGTAPSDEELEKALLELGGADNE*

>CD630_34660 Clostridioides_difficile_630_NC_009089 holo-ACP synthase

MNIFDIGVDIIEIDRIRKAVDKNNRFLEKIFTDREIEYFNSKNFKAESIAGNFAAKEAISKSIGTGIRVFNFKDIEVLRDEMGKPIVKTYNNLAKMCIDYNVLEIKVSISHSKDYAIANAITIIKD*

>CD630_34680 Clostridioides_difficile_630_NC_009089 ATP synthase subunit beta

MANVGKVVQIVGAVLDVKFDSEQSLPNLLNALVIKLGDKEIVAEVAQHIGDDTVRCIAMSATDGLVRGMEVVDTGGPISVPVGDETLGRIFNVLGKPVDGKPAPKSAPKLPIHRPAPAYDELETTAEILETGIKVVDLLAPYLKGGKIGLFGGAGVGKTVLIQELINNIAKQHGGISVFSGVGERTREGNDLYGEMSESGVINKTALVFGQMNEPPGARMRVALTGLTMAEHFRDEQGQDVLLFVDNIFRFTQAGSEVSALLGRMPSAVGYQPTLATEMGALQERITSTKKGSITSVQAVYVPADDLTDPAPATTFSHLDAKTVLSRQISSLGIYPAVDPLESTSRILDPSIVGKEHYEVARGVQSILQRYKELQDIIAILGMDELSDEDKLIVARARKIQRFLSQSFTVAEQFTGNPGQYVPVKETVRGFKEILEGKHDDLPESAFLFVGTIEDAVRKAKGSM*

>CD630_34700 Clostridioides_difficile_630_NC_009089 ATPase subunit alpha

MNLKPEEISSIIKQQIKNYENKVELTDTGSVLTVGDGIASVYGLEKAMSGELLEFPGEIYGMALNLEEEVVGAVILGDDSEIKEGDIVKRTGRIVEVPVGEALIGRVVNSLGQPIDGKGPIAYTKTRPVESEAPGIIDRRSVYEPLQTGIKSIDSMIPIGRGQRELIIGDRQTGKTSIVIDTILNQKGKDVICIYVAIGQKRSTIAQLVSSLEKGGALDYTIVVSATASESAPLQYIAPYAGAAMGEEFMYNGKHVLIVYDDLSKQAVAYREMSLLLRRPPGREAYPGDVFYLHSRLLERAAKLSDELGGGSMTALPIIETQAGDVSAYIPTNVISITDGQIYLQPELFYSGVRPAVDPGISVSRVGGSAQIKAMKKVAGTLKLAYSQYRELAAFSQFGSDLDEDTKKRLAQGERIVEILKQGEHQPIKVENQVMIIYAVINNHLEDIPIDNIARFESELYAFVDNNYPEISRKILGGEDFTHDLTDAINEFKEKFVVEV*

>CD630_34810 Clostridioides_difficile_630_NC_009089 protein-tyrosine phosphatase reductase

MNILIVCTGNTCRSPMAEAILRKAIKESGRSIEEYSISSAGISTANGMGASENSIEVLKEIGIDLSNHRSKVITKKLIDESDIILTMTKSHKEILVQAVPKCKEKVYTFKGFANKNEEDISDPFGGNLDIYRSTMREIMYSVNEIVKKI*

>CD630_34840 Clostridioides_difficile_630_NC_009089 peptide chain release factor 1

MLKKLEVLEDTYKDLSEKIGDPDVINDQKVWQKYIKEHADLEPIVMKYREYKSVLDSIKESKEILQEESDEELRELAKMELAEMEEKVAPLEEEIKILLLPKDPNDDKNVIVEIRGGAGGDEAALFAGDLFRMYSRYAERRRWKIELLSASDTGVGGYKEVSFMIKGKGAYSRLKYESGVHRVQRIPSTESGGRIHTSTSTVAVLPEVEDVEVEINPNDLRIDVFRSSGNGGQSVNTTDSAVRVTHIPTGEVVSCQDGKSQLKNKEQALKILKARLYDKALAEQHKDIAAERKSQVGTGDRSERIRTYNFPQGRISDHRINLTLYKLDAFLDGDIDEMIDALITVDQTEKMTAI*

>CD630_34880 Clostridioides_difficile_630_NC_009089 UTP--glucose-1-phosphate uridylyltransferase

MQVKVKKAVIPAAGLGTRFLPATKAQPKEMLPIVDKPTLQYIIEEAVASGIEEILIITGRNKKSIEDHFDKSVELELDLEKKGKKELLEVVQNISNMINIHYIRQKEPKGLGDAIYCARHFIGDEPFAVMLGDDIVDNDVPCLKQLTDAYEEYRTTILGVQKVNQEDTNKYGIIEAKNIEGRVYKVKDMVEKPESGKAPSNIAILGRYIITPEIFDILKDLPPGKGGEVQLTDALKILSKKEAMYAYNFEGKRYDVGDKLGFLEATVDFALKKEDLKEDFIKYLKHVCSEFDKNNNVLYNNTDEKIDLIEVNEARELEIQK*

>CD630_35010 Clostridioides_difficile_630_NC_009089 transcription-repair coupling factor

MNDVFLYPLQNSKEYKDIINCIKNTKGSLLVNGLLPVQKPHISYSIFNDLSRQMIFITSSDLEAKKVYEDLSFYMEDKVEYLGFQDIYFYHLDAKDRNEEAKKLKVLLKLANKEKIILVTSIEAVLRKYIPKQVLLDSVSHYKVGDSLDLEKLTEKLVSLGYERVSKIEGFGQFSIRGGIIDVFSLEYTNPIRMELFDDEIDSIRTFDVYSQKSIDKLQQFSITPSREFIYPEKTTDALVKLKKETTKNTDEGTFQNIDYISSKTYFEGVENYIDYIYPEENKSIFTYLADDAIVFINDITRLKERCENYINEFRENYKLNLERGLAIKNQGKLLYHYTDLEYLVKDKSVVLNSLLPKSINNFSIKSIINFESREVPTFNGKVDLLVEELNRLKYNGYKIILATNTLERANKLGKDLLDKGLETTISKDRDIEIKSSQVIIVPAHINSGFQYKSIKFVVITDNEMIGVYKRASKTSNKKVKKGKKIESFLDLSVGDYVVHENSGVGRYTGIEQITVNAIKKDYMKIVYQGGDNLYVPIDQMDKVQKYIGAEVEKVKLNKLGTNEWTKAKAKVKKEIEDMTKDLIELYAKREKIQGYKFSKDTPWQAEFESLFPYQETEDQLKAIEETKKDMESSKVMDRLVCGDVGYGKTEVAIRSIFKACMDQKQVAVLVPTTILAQQHYNTFKERFENYPLRVEVLSRFKTPKQQKQIIEDAKKGLVDILIGTHRIISKDINLPNLGLVVIDEEQRFGVKHKEALKKIKSTVDVLTLSATPIPRTLHMSLSGIRDMSVIEEPPQERHPVITYVTESKESVIQDEIERELSRGGQVFFVYNRVEHIEEMASMIQKLVPDARVAVAHGRMTSKSLENIILGFLNKDYDVLVCTTIIETGMDISNANTMIIYDADKMGLAQLYQLRGRVGRSSRQGYAYLLYEKDKTLSEIAEKRLKAIREFTEFGSGFKIAMRDLEIRGAGNILGSQQHGHMAVIGYDLYVKMLNDAIKKVKGEPIVEEIDVEIDLSVNAYIPDNYIKDELIKIEMYKKIASIENKEDMLDIQEELEDRFSDIPKPVQTLLTIAYIKSLCKILKIEKIRQLKDEILLVPITKYRTKQKIGYNIVTELEELLEKMCKVK*

>CD630_35020 Clostridioides_difficile_630_NC_009089 peptidyl-tRNA hydrolase

MYVVVGLGNPGKKYEKTRHNVGFDVIDILAKEYNISVTKIKHKALIGEGRVGTEKVLLVKPQTYMNLSGETLIDIYKYYKVDLSNIVVVYDDIDLEVGKIRIRKKGSGGTHNGMKSITKCLGSNDFPRVRVGVSKPEAGQDLADFVLSRFRKEESDNINEALEKAADAIDSIIRENIDMSMNKYNG*

>CD630_35050 Clostridioides_difficile_630_NC_009089 twitching motility protein PilT

MYIYDLLEQGIRLNASDIHITVGTNPVARVKGGFVKLSEQILTSEVTMQMAKDIAGESMFKVIEEHGEADFSASLKTGERFRVNAYRQKGNYAIAIRTITAEIPTFEKLGLPESIKSFTEKHKGLVLVTGPTGSGKSTTLASMINIINEKQQKHIITLEDPIEYVHHHKQSLVNQREVGTDTESFHSALRAILRQDPDVILIGEMRDPETVSIALTAAETGHLVFSTLHTVGAAKTIDRIVDMFQPSQQQQIKTQLSTVCEGVVSQQLLPTADGKGRIAAIELMFATPAIKNLIREGKTYQIPNMIQTGVKSGMKTMDQDLMELYKNGKITKDMALSRCTDQEFMTRMIGGVNYNGYYNR*

>CD630_35120 Clostridioides_difficile_630_NC_009089 type IV pilus transporter system

VAKKVRIGDKLVEKGYITEEQLKWALSEQKNSGKRLGEFLVQEGLIDSNLLISVLKELLDIESIFLEGTEIDTLATKMVPENICKRYTVFPFKIDGNKICLAMSDPQDREAVQDVRRMSGKDVEIFISSTEDINKAIGHAYAHSEINKAMTEYNKNRTGGVRETVILEEDVNAAPIVRLVNNILENAVRMEASDIHIEQSENYMRVRFRIDGMLREYMRMNSAPYKAVISRIKIMSDINISEKRIPQDGRIYLKVDNKPIDFRVSTMPTNRDEKIAMRVLDKSNFMVSKEVLGIDEHGSKIYDELINTPYGLILVVGPTGSGKTTTLYSMLNQLNTENRNLLTIEDPIEYELPGVNQSQINEKAGLTFASGLRAFMRQDPDIIMVGEIRDTETAEIAIRASLTGHLVLSTLHANTAVGAISRLLDMDVESFLITSSVLGVISQRLTRKICEHCKVSYEADIGEKKALGIDVNESVTIYRGKGCERCNNTGYKGRLGIFEMLEITPEIKELIDSSANQREILKMARKQGMVSLKEDIVKKVLNGKTTVEEMIRIILMTD*

>CD630_35140 Clostridioides_difficile_630_NC_009089 ribose-phosphate pyrophosphokinase

MNTSGSEIKIIAGNSSKELAQKIADYIGVSVLDCEVGTFSDGEICVNMNETVRGCDVFVVQSTNSPVNDNLMELLILIDALKRASAGRITAVIPYYGYARQDRKAKARDPITAKLVANLITAAGADRVLTMDLHAAQIQGYFDIPLDHLLGGTILANYFNEKKIEDLVVVSPDLGSVTRSRKFANTLNGEVPIAIIDKRRPKANVCEVMNLIGDVKGKNVILLDDMIDTAGTIVNAANALKEFGAKDVYACCTHGVLSGPAIERIANSEISELIVLDTIQLPEEKRIDKIKIKTVAPLFGDAIRMIFSNESVSKLF*

>CD630_35150 Clostridioides_difficile_630_NC_009089 bifunctional N-acetylglucosamine-1-phosphate uridyltransferase/glucosamine-1-phosphate acetyltransferase

MNFKAIILAAGKGTRMKSKYPKVIHKVCGKEMVNHIIDVSKKSGVKDTVVILGHEADVVKEKLAEEIIIAMQTEQLGTGHAVKMAKEYINDEDTIVVLCGDTPLIKEETLKRLFEYHIENKYHATVLTTRVGNPTGYGRIIRDKKGDLLKIVEQKDANSEEKMISEINSGIYCFNGKSLREALDLLNNNNSQGEYYLTDTAKIMRDKGLKVGAFAGSTIEELMGVNSRVELSKAEEIMRRRINESHMVNGVTIIDTNSTYIESDVMIGNDTIIYPGVMLQGKTRIGSDCIIGMNSSITNSEIGDGTEIKNSTIIDSKVGENSTVGPYAYLRPKSDLGNNVKIGDFVEVKNAIIEDGSKASHLSYIGDAHVGKNVNIGCGVVFVNYDGKNKFKSIVKDNAFIGSNSNLVAPVVVEEKGYIATGSTITHDVPDGALAIARERQVIKEGWVEKKNQKDDQSK*

>CD630_35200 Clostridioides_difficile_630_NC_009089 cation efflux protein

METRYEEANKITIQSILWNVVLTIIKVIAGVIGNSSAMIADGLHSASDIISSIGVLIGNYVSSRPGDREHNYGHEKAETLVSFVLSILLIFVSITIGIEAIKSLFNLDALSVPSILPLVVSVISILIKEYQYRITIKVAKKINSPALKADAWHHRSDALSSVAAFIGIGGSILGFKPLDPIASVVVAIFVAKVGISILISSVNELMDVSVDEEEIKELKFIVADTEGVKNLGDIKTRKHGAMAYVDLTICVDENLTVKQGHDIATKLEKHIIKHMEFVKGITVHVEPCTNCQGNKCNN*

>CD630_35230 Clostridioides_difficile_630_NC_009089 rRNA small subunit methyltransferase A

MDRLSSHNATKEVVQKYNFKFSKSLGQNFLIDSNIIDKILSGARITRGDNIIEVGPGIGTLTREMGKIAEKVVAIEIDRNLIPILKDTLSDLDNTEVVNQDILKVDIQELVKDKLNGGPVKLVANLPYYITTPIVMKFLEEDIPVTDIVVMVQKEVADRMNAIPGTKDYGALSIAVQYYCDTEIVAKAPRHMFIPQPNVDSTVIGLHVRDKRKYDVHNEDIFFKTVKASFGQRRKTLLNSLGGLGFLNKDEIREILKEANIDEKRRGETLSIEEFSVLSNIINTKVSSK*

>CD630_35300 Clostridioides_difficile_630_NC_009089 iron family ABC transporter ATP-binding protein

MSFLQVKNVGKSYGQVKVLKDISIDIEKGEFICLLGPSGCGKSTLLRIIAGLEDKHGGKIIINDKDMTNSPPESRNFGIVFQSYALFPNMNVYKNIAFGLENKNISKSNIDKKVKEVLEVVELSGYEKKYPSQLSGGQQQRVALARAIALEPDFLLLDEPLSALDAKVRLKLREQIRSLHRKLGITTIMVTHDQEEALCLADKMVVMNRGEIIQVGTPKEVYKNPETPFVADFIGTINFIDDGINKIAIRPEDIKVESNRDSKDKDIKVGEILDIEFRGFNYRITVEYRSKQMKLDVVSKVAEQMKLCIGSKINFKIPKEGIVQYKSEGCA*

>CD630_35390 Clostridioides_difficile_630_NC_009089 deoxyribonuclease

MLFDSHAHLNDESFDEDRDELIGSLKDKGVDLVVNPGADIETSITAIELAKKYDFIYSAVGVHPHDVSKLDDTAIETLRKLATENEKVVAIGEIGLDYYYDYSPREEQKEWFKKQIELANELKLPIIIHDRDAHGDTFEIIKNTKNPEIGCVLHCYSGNVELAREYVKMGCYISIPGTVTFKNNKKTREVVREIPLERLFIETDSPYMSPEPHRGKRNNPSQVSFVADKIAQEKGISYEEVCRVTKENAKKFFNIK*

>CD630_35400 Clostridioides_difficile_630_NC_009089 methionine--tRNA ligase

MSKPSFYVTTPIYYPSGGLHIGHTYSTVAADTIARFKRFCGYDVKFLTGTDEHGEKIQKKAIEQGMSEIEYLDGMIKDIKALWNTMDISYDDFIRTTEKRHTDIIQKIFTKLYEQGDIYKGEYEGRYCTPCESFWTESQLLEGNKCPDCGRETYLVKEESYFFRLSKYEDRLKELFKDDSFCFPAARKNEMVANFLDKGLEDLSVTRTTFDWGIKVPFDEKHVIYVWVDALCNYITALGYMTDNDEEFKKYWPANVQIVGKEIVRFHTIIWPALLMALGLEVPKQVFGHGWILFADDKMSKSKGNVVYPEPIIERYGIDTLKYFLLREFAFGQDGSYTHRNFVTRINYDLANDLGNLISRTVAMVEKYNNGIIPTAKVSTDFDADLKEQAVSTRENFEAEMDKMQFHEALESVWKLVRRTNKYIDETMPWALAKDETKKGELDTVLYNLCESIRIIATLINPIMNETANKIYEHIGIKGQDDITTWESTKTFGLIGENVKVFKGEPLFPRLDVEKEIEELTKMFSGKPPVEEKPLEHKEEITIDDLDKIELRVGKIISCEKHPKANKLLVSQVKIGPETRQIVSGIAEYYKPEDLVGKEVTVVCNLKPVKLRGVESQGMILAAGDDGEPYVLPFTQGAKDGCEVR*

>CD630_35430 Clostridioides_difficile_630_NC_009089 nicotinate phosphribosyltransferase

MRNLTLLTDLYQLTMLNGYFEKNIHEDIVVFDMFFRKNACDGGYTIVCGIDQVVEYIDNLHFSDEDLEYLKNLNLFSDKFLKFLKEFKFTGDIYAVEEGTIMFPNEPLITVKAPLYQAQLIETALLTIVNFQSLIATKASRVCFAAQGDPVFEFGLRRAQGPDAGIYGARAAVVGGCAGTANVLAGKMFDIPIIGTQAHSWVQKFDNELEAFQAYADVYPDKCLLLVDTYDVLNSGVPNAIKVFKNISEKGYKPMGIRLDSGDLAYLSKEAKKQLDNAGFSDISITASNDLDEYTITSLKAEGATINSWGVGTKLITSFDSPSLGGVYKLAASCEKGVLEPKIKISENPEKINNPGYKKVIRIYNEDNKAEADLIMLHDEVIDESKPLEIFHPTYTWKTKVFTNYKVKELLKPLYIKGRCKYNKKAVLEIKNHVQYELSTIWEQYKRLSKPHIYKVDLSRNLWYLKTQMIDSKKVL*

>CD630_35440 Clostridioides_difficile_630_NC_009089 AsnC/Lrp family transcriptional regulator

MDVTDYRIIEILQDDGRISMKDLGKIVGLTSPAVSERVKRLEESGVIEGYKAIVNPDSLGRVIKAFIHISLPSNGYTEFIESAAKDPRIVECHHITGDDCLLLKVIVKDMYELENVIDTIKKIGSTKTSVILSTPIQAKSIL*

>CD630_35520 Clostridioides_difficile_630_NC_009089 lysine--tRNA ligase

MKNNQQSNEEAQIQEDLSEVLQVRRDKLKKLQESGRDPFKESRYDRTHYSMDIKDNFDSLEGKTTKIAGRIMSKRIQGKAGFIDIQDQEGRIQSYVRLDAIGEEEYSVFSTYDIGDIVGIEGEIFKTKKGEISVKAKSVVLLCKSLQVLPEKYHGLKDQELRYRQRYVDLIVNPEVKNAFLIRTKALKALRAYLDDRGFLEVETPILNTIAGGANARPFITNHNTLHIPMYLRIANELYLKRLIVGGFDKVYEMGRMFRNEGMDLKHNPEYTAIELYQAYADYTDMMEITENVIAHMAEVATGSMIVNYQGTEINFTPPWKRMSMEDCVKEYSGVDFSTINTDEEALEVAREKGIEIKPGMRRGEVINAFFEEFGEDKLIQPTFITHHPVEVSPLSKRNVEDPRRTDRFEAFANKWELANAFSELNDPIDQKGRFIDQLRKRELGDDEAFEMDEDFLKALEVGLPPTGGLGIGIDRVIMLLTNSPSIRDVLLFPTMKLIDNNSNKEEEN*

>CD630_35550 Clostridioides_difficile_630_NC_009089 pantothenate kinase

MLLVFDVGNTNMVLGIYKGDKLVNYWRIKTDREKTSDEYGILISNLFDYDNVNISDIDDVIISSVVPNVMHSLENFCIKYCKKQPLIVGPGIKTGLNIKYDNPKQVGADRIVNAVAGIEKYGAPSILVDFGTATTFCAISEKGEYLGGTIAPGIKISSEALFQSASKLPRVELAKPGMTICKSTVSAMQSGIIYGYVGLVDKIISIMKKELNCDDVKVIATGGLAKLIASETKSIDYVDGFLTLEGLRIIYEKNQE*

>CD630_35590 Clostridioides_difficile_630_NC_009089 ATP-dependent zinc metalloprotease FtsH

MYERRASILNKLLKGAGFYLLVFIIIVGIVQFSGKPTEKIKDLKFSEVYRELTDENISRLYFVNQTSVEGTIKDTNTKFKSYVPTEVMGNKLADEVLDQAKAGKLTFGGEAKPSTPWFVEMLPTLLLIFFMVIIWFVFMNQSQGGGGKVMSFGKSKAKVHKDDEKTRVTFKDVAGLDEEKEDLQEVVDFLKNPKKYIELGARIPKGMLMVGPPGTGKTYLSRAVAGEAGVPFFSISGSDFVEMFVGVGASRVRDLFEQAKKSAPAIIFIDEIDAVGRKRGAGLGGGHDEREQTLNQLLVEMDGFGVNQGIIIMAATNRPDILDPALLRPGRFDRQVVVGTPDVKGREAIFKVHSRNKPLSDDVKMDVLARRTPGFTPADIENLMNEAAILTARKREKKIKMETIEEAITKVIAGVAKKSKVISEKERRLTAYHEGGHAVCAHVLEEVSPVHQVTIVPRGRAGGFTMQLPVEDKFYATKNEMKENIVVLLGGRVAEELVLKDVSTGASNDLERVTATARSMVTKYGMSSKLGPMSFDSDDEVFLGNSFSSKRNYSEEVAFEIDQETKRIVDGAYDKTRSILQENMDRLEYVAQALLIYETLDAEQFVKAFNKELPLNEIENAVTEENSSKEVEEQLTIKLEKDEEERNNVIDINKNLEDKSDKDK*

>CD630_35600 Clostridioides_difficile_630_NC_009089 tRNA(Ile)-lysidine synthase

MIFDKVLSTINKHNLIQKGDKIVLGLSGGPDSVCLLHVLNRLKKDFNIEIYAAHLNHQIRGIEAQKDALYVSKLCEDMGIIFFVKSINVPKYCENEGLSLEEGARKLRYEMFYEIKDKIKANKIAIGHNLNDQAETVMMRIMRGTGLKGLKGIDYIRDNCIIRPILDVERNEIEEYCEAYNLNPRIDKTNLENIYTRNKIRLDLLPYMKDNFNSNVIESIVRMSNSLKSDNDYIEKEAEAKFREVSNIKEKGFVEINLDDFVCLHDAIKVRVLRNSIKHILGDTNFVDQRHIEDIMSLEDNSKVNKMLTLPRNIFVYRKKDSIILTNEEIVNEEIEFYYNVPSNGFIKIKELKQIIETQVMSIDRYKSMKLDNSSKGFDFNKVKGGIVIRSRRQGDKIKLAMGSKKVKDLFIDLKIPREERCKIPIITDSEGIICVGDYKISENYKIDENTKEVLKINFNKL*

>AEC_RS0219720 Clostridioides_difficile_QCD_37x79_NZ_CM000658 methionine gamma-lyase

MSNNINQDLETKIIHWGHSADPTTGALATPICQTATFAAKTVEHFEELCMTWGYVYTRECNPTLTELEAKLAMLENAESAISSTSGMGAITSTILALVKSGDHIVSSDGIFSHTKLFMSELLSKFGVEVTFVDAVNPQNVKEAMRPNTKIVYIESPLNPSLDLVDIKTIAEIAHENKSLAIVDSTFGTPIVQRPIDLGADLVIHSLTKFINGHGDTLGGAVAGSKELIDLVRWPSLCCFTGASLPPMSAWMILRGMKTLDMRMKKHCENGLAVAEFLEEEENVELVKYPALKSHPQYELCKTQMNGLGGGVVSFKLKDGINGLTRDQASRKLMNSLELATIATSLGEEHTLVQMNGENLIRIAVGLESSNDIINDFKQANKKIKIKLHE*

>CD630_35850 Clostridioides_difficile_630_NC_009089 ABC transporter ATP-binding protein

MAFLKIEDLCKVYGKNENKVTALDHVSLTIEKGEFTAIIGSSGSGKSTLLHSIAGVDVPTSGKIYLEGQDVYGQSNEKLAIFRRRQVGLIYQFHNLIPTLNVVENITLPILMDKRKVNQERLNDLLELLGLKERKTHLPNQLSGGQQQRVAIGRALMNAPAVMLADEPTGSLDSKNGQEIIQLLKESHSKYHQTLIIVTHDENIALQADRIICISDGKVVRDERKVNR*

>CD630_35860 Clostridioides_difficile_630_NC_009089 two-component sensor histidine kinase

MFRNREFRKFAILFLLITILTVALGFAISIMTGILSIVSATTFGIAFFVFTKNRYKSIAQISEQINLVLHNANHLYIAESDEGELSILQSEITKMTLRIREQNYALKKEKEHLADSLADIAHQLRTPLTSVTLILSLLENTSDEDERKELIRETEELLIRMDWLITSLLKLSRLDAGIVVFQKEQIDVNNLISSALHQLLIPMELHNITLHIDIPKGVRILGDLNWLSEAIQNIFKNCMESVGDNGKIDIICEDNFLFTQLTIHDNGAGFKKEDLPCLFNRFYRGKNSSTAGYGIGLALCKTIIMRQGGTITAQNHPQGGAIFVIRFPK*

>CD630_35890 Clostridioides_difficile_630_NC_009089 carbamoyl-phosphate synthase small subunit

MKARLILEDGTVFIGKAFGYLEESVGEVVFNTSMIGYGEVLTDPSYYGQIVTMTYPLVGNYGINLSSAESEKVQVKGFIVREKSDSPSNFRCEIDIDQYLKQNKVIGLEGIDTRALTKILRNNGTMKGIITLEDSKLEDVKHKLDKFSNTEAVRTVTRKEVEHIKGNGPKVAVMDFGVKRNILRSFIARGCDITIFPATTSPEDVLSINPDLIFLSNGPGDPEDLEDVIENIKALIGKKPIVGICLGHQLLALALGGKTAKLKFGHRGGNHPVKDLEEGKVFITSQNHGYYVSEVPEQMKVTHINLNDNTVEGMRHEKLDVYSVQYHPEACPGPKDNDYIFDKFLELVK*

>CD630_36080 Clostridioides_difficile_630_NC_009089 ABC transporter ATP-binding protein

MLEIKNLSFNVESNNEELGIINDVSLSFERGKLIVITGPNGGGKSTIAKLIMGIEKATSGQIILDGEDITNLSITERAKKGIGYAFQQPPRIKGMTVENLLTLAHGKPLSTDVCCQYLTDVGLCSKDYLNREVDNSLSGGEMKRIEIATLFARDLKVSIFDEPEAGIDLWSFGKLNESFKKIHEESNQTIIIISHQERILELADEIIVLQDGSVKSHGTKEAILPEIMCQVNSSCELMKDMN*

>CD630_36540 Clostridioides_difficile_630_NC_009089 DNA replication protein DnaC

MNEDKIRKILAKYAKRRDDNELLLEHRKNEVYNRIPEIKSIDDEISKIGLSLAKIVLLNPKSKDEIVKKTKENIESLKVKKERLLAESNIPLDYLEIKFQCISCKDKGFLPNGEKCSCLKQEIVNEAYKMSNLDRILSQENFSNFNLNIFSPKKGSDGEISPRENMLNNLSICENFVHDFKKDNSENLLFYGSTGLGKTYMCNCIAKELLDKGNVVIYQTSFRILDILEDYKFRRDTNNQISEDNYKNLFDCDLLIIDDLGTELNNSFTSGEIFNIVNTRLVAGKKIIISTNLTPSQIGNTYTQRTLSRILDKFRILEFTGDDLRWERFK*

>CD630_36550 Clostridioides_difficile_630_NC_009089 adenylosuccinate synthetase

MKTVAIVGSQWGDEGKGKVIDYLATQADVVVRGQGGNNAGHTLVVEGKKYALHLIPSGVLNPNTVNIIGNGIVFDPKGFLEELEMFKTDNISTENIKISDRAHVIFPYHKELDALSEEARGDLKIGTTKKGIGPCYMDKTERSGIRICDLMDKDKFAIKLKAQIDAKNEIVKNIYGKEELFDFETIYNEYLGYAEQIRKYVADTSVIVYDAVRAGKKVLFEGAQGTLLDLDLGTYPFVTSSHPTSGGFAIGAGIGPNMIKDVVGIVKAYTTRVGEGPFVTEQINETGDKIREQGHEFGVTTGRPRRCGWFDAVIVKYAARVNGLTSISFMLLDVLTGFDKIKVCTSYKMGDKIITDFPASLDDLAKCEPVYEELDGWNEDITQIDNFDDLPENAKKYVAKIEELVGVSVDMVSVGPNRAQTIIRRNIFA*

>CD630_36700 Clostridioides_difficile_630_NC_009089 cysteine desulfurase

MIYLDNAATTYPKPERVYNAVLDCMKNYCANPGRAGHKLAMRAAREIYDTRENIAKLFNVSNPMNIVFTSNATDSLNLAIKGVLQEGDHVITTSMEHNSVIRPIKALEKRGIENTVVKCDYEGFLDYEDLEKSIKSNTKLIVTTHASNVCGTLIDIKKVGEIAKKHNILFLVDASQTAGVYDIDVNECNIDMLAMPGHKCLFGPQGTGILYVREGLNLNILKEGGTGSKSEEIVQPELFPDKYESGTHNTPGIAGLNQGILFIFERGINNIRQHEEELCQYMIDKLEEVPDIKIYGPKDSKKRASVIALNIGDMDSGEVTFLLDSDYNIATRSGIHCSPLAHTTLGTLKQGAVRFSIGYFNTKDEIDKAVEALKKISKNK*

>CD630_36720 Clostridioides_difficile_630_NC_009089 sporulation initiation inhibitor

MGKVIAVFNQKGGVGKTTTNVNLSASLGTLGKKILVLDLDPQGNTTSGYGINKNEVENTIYEIMLDGLHIKEAIISTEFENIDVVPSATELSGAEIELTSKTNREYILKNSIKAVIDEYDYIFLDCPPSLGMLTINCLTAVDSVLIPIQCEYYALEGVSQLMETIKLVKSRLNADIEIQGVVLSMFDGRANLSIQVVEEVKKYFKGSVYTTLIPRNVRLAEAPSHGKPVIYYDKRCRGSVAYLELAEEFIDLEEEEW*

>CD630_36750 Clostridioides_difficile_630_NC_009089 tRNA uridine 5-carboxymethylaminomethyl modification protein GidA

MIKFEAGKYDVIVVGAGHAGCEAALATARMGYKTLIITMSLDSIALMPCNPSIGGTGKGQLVKEIDALGGQMGLNIDKTYIQSRMLNTAKGPAVHSLRAQADKFKYHEEMKKTLEDEPNLDIAMDEVVEILHEGNVVIGVGTKLGCSFKSKAVILATGVYLNSKIYMGEVAFYEGPNALGYAKYLTDSLVELGLRMRRFKTGTPARVHRDSIDFSVMSLQEGDEKVTPFSFMNENIEKKQEPCYLTRTTEETQKVILDNLKRSAMYSGVIESTGPRYCPSIEDKVVRFSDKTSHQLFIEPEGLNTKEMYIQGISTSLPFEVQLDMYKTIKGLENCKIMRPAYAIEYDCVDPTQLKISLEIKGVENLFSAGQFNGTSGYEEAAAQGLMAGINAVRKIEGKEPFVLDRSEAYIGVLLDDLVTKGTNEPYRMMTSRAEYRLYLRQDNADMRLTQKGYDIGLVKKDRYERFLNKKAAVEKEFERLKNERVTPKEVNSLLEEKGATPIKVGISLYEFLKRPEVTYELLEELGKGAGDDVSREVKEQCVIITKYEGYIEKQLKQIDQFKKLENKKLDEKINYSSIEGLRLEARQKLDDIKPISIGQASRISGVSPADISVLLIYLEQIRRTRGGKGE*

>CD630_36760 Clostridioides_difficile_630_NC_009089 tRNA modification GTPase TrmE

LFIDDTIAAIATAPGEGGIGILRISGEKALKVAEEIFKSMSGKSIEEYNKRTLIYGNIVDNENIIDEVLLAYMKGPNSYTGEDVIEINCHGGFISVKKILELILSKDVRLAEAGEFTKRAFLNGRIDLSQAEAVIDVIKAKTDIAHEVAQNQLEGSLSKKIRELRDKVTEILAHVEVAIDYPEEDIEHITYQTLKEKTDELKKDIKKLYDTAESGKILREGLKTVIVGKPNVGKSSLLNSILGENRAIVTDIPGTTRDVIEEFVNIKGIPLKIVDTAGIRDTDDIVEKIGVEKSKESFTSADLIVMVLDASRKLSEEDIEILEKLKDKQTIVLLNKNDLKQEIEEEKILKYVENNSIIKISALQQEGIEELQDKIESMVYKGSIKNNSSLVVTNSRHKDALSKAYKSATDALIALEQSMPFDFVEVDLKNIWDYLGYINGDTVTEDLLDNIFHNFCIGK*
