# Supplementary figures and images for: Core-genome-mediated promising alternative drug and multi-epitope vaccine targets prioritization against infectious Clostridium difficile
Source: PLoS One. 2024 Jan 19;19(1):e0293731. doi: 10.1371/journal.pone.0293731 (PMC10798517; doi:10.1371/journal.pone.0293731)

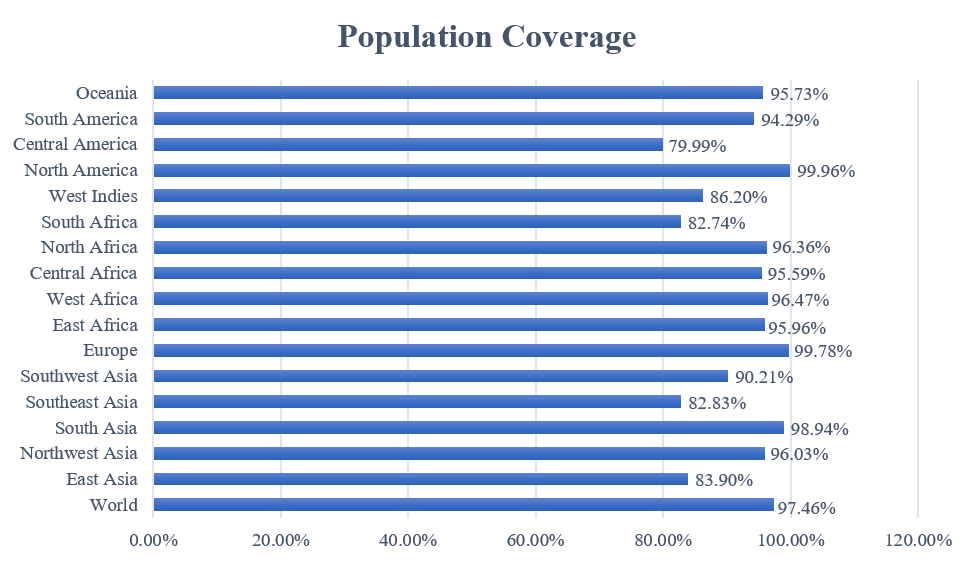

Supplement: S1 Fig — (TIF) [file pone.0293731.s004.tif]

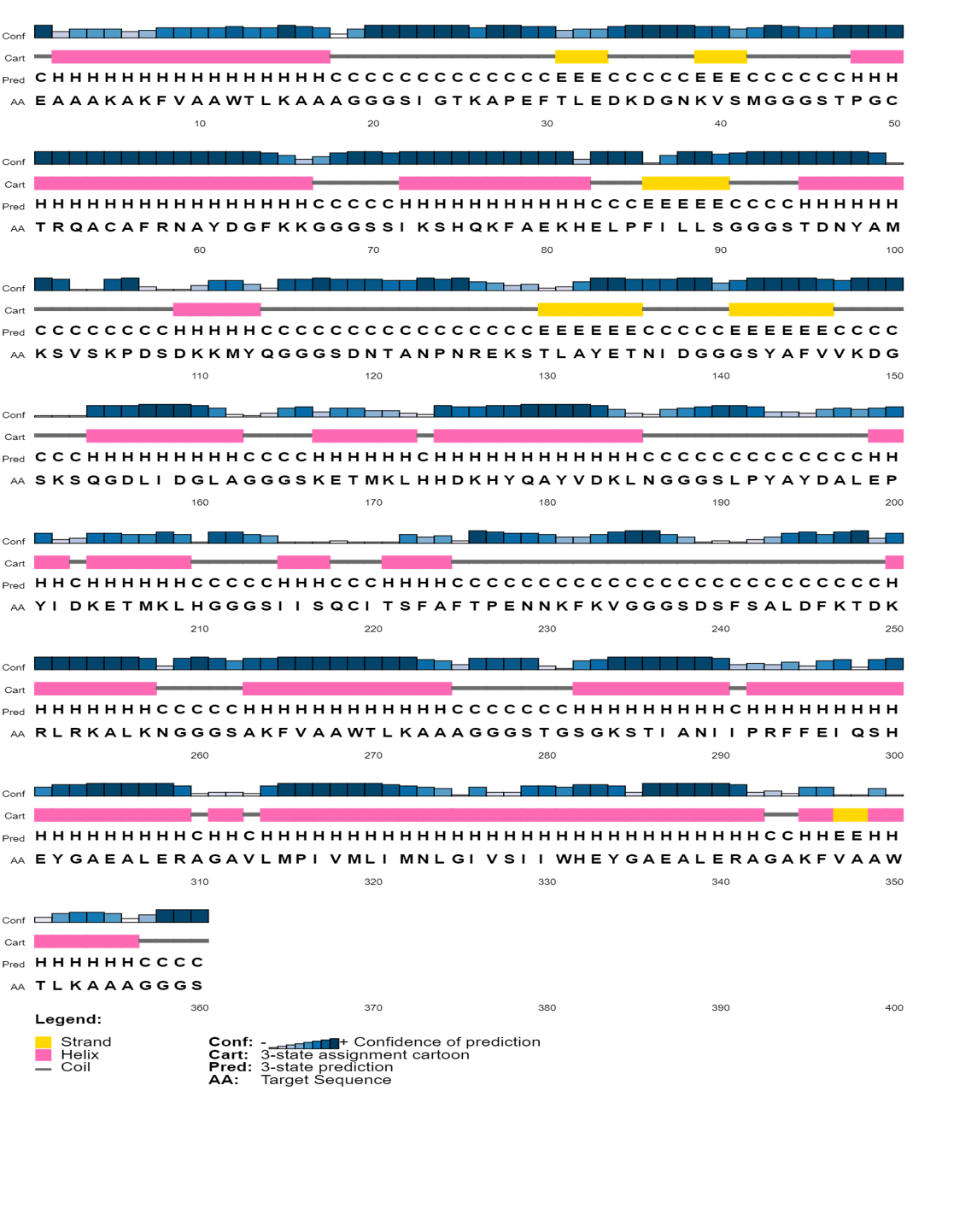

Supplement: S2 Fig — (TIF) [file pone.0293731.s005.tif]

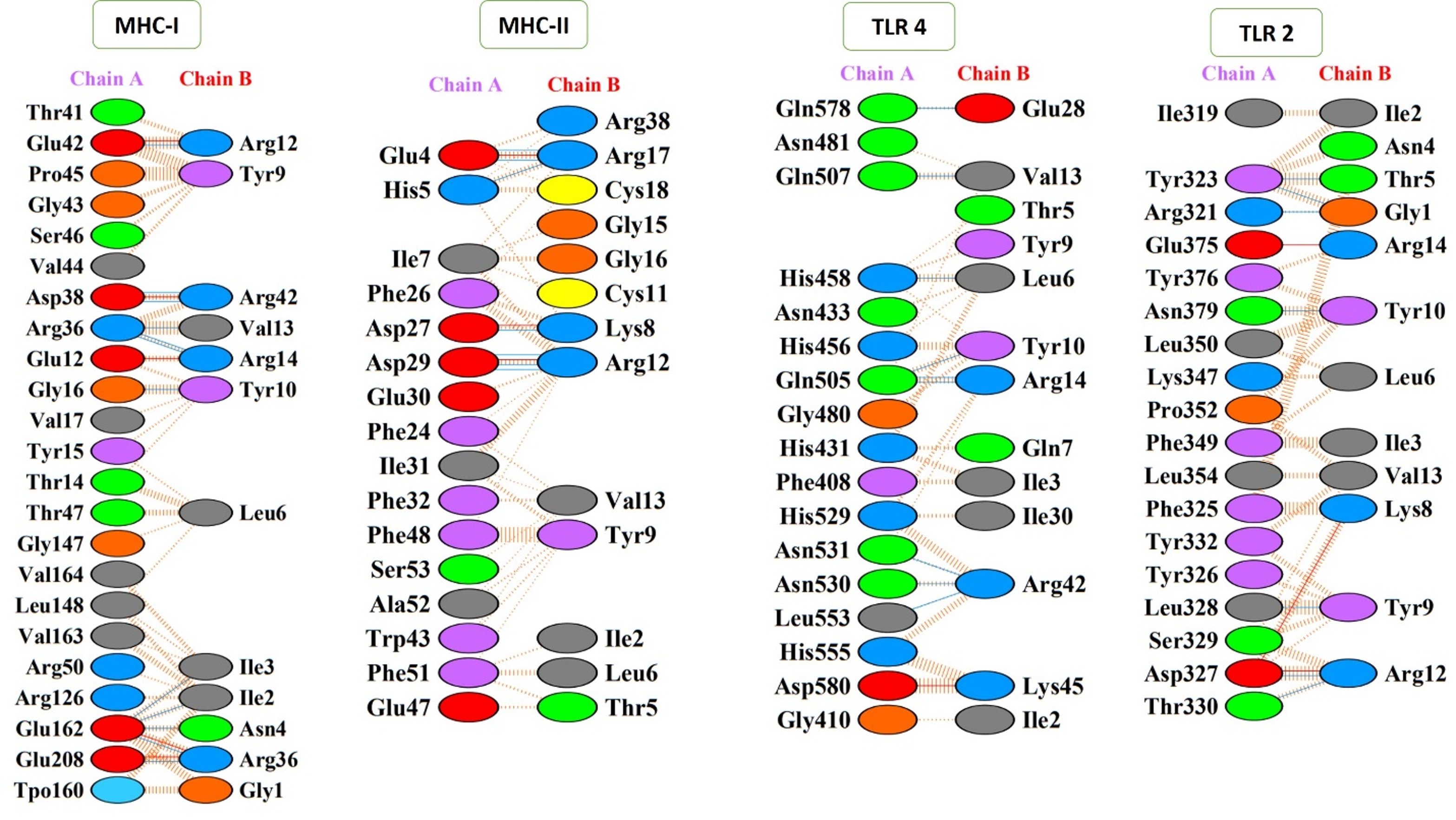

Supplement: S3 Fig — (TIF) [file pone.0293731.s006.tif]

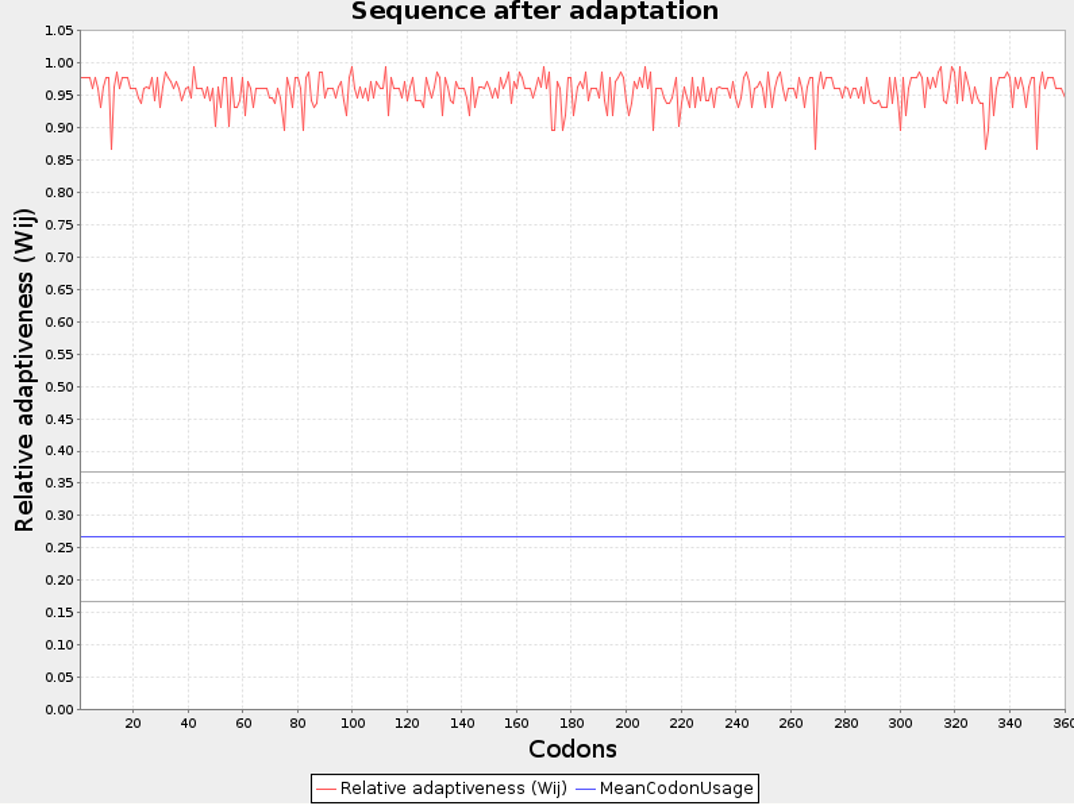

Supplement: S4 Fig — (TIF) [file pone.0293731.s007.tif]

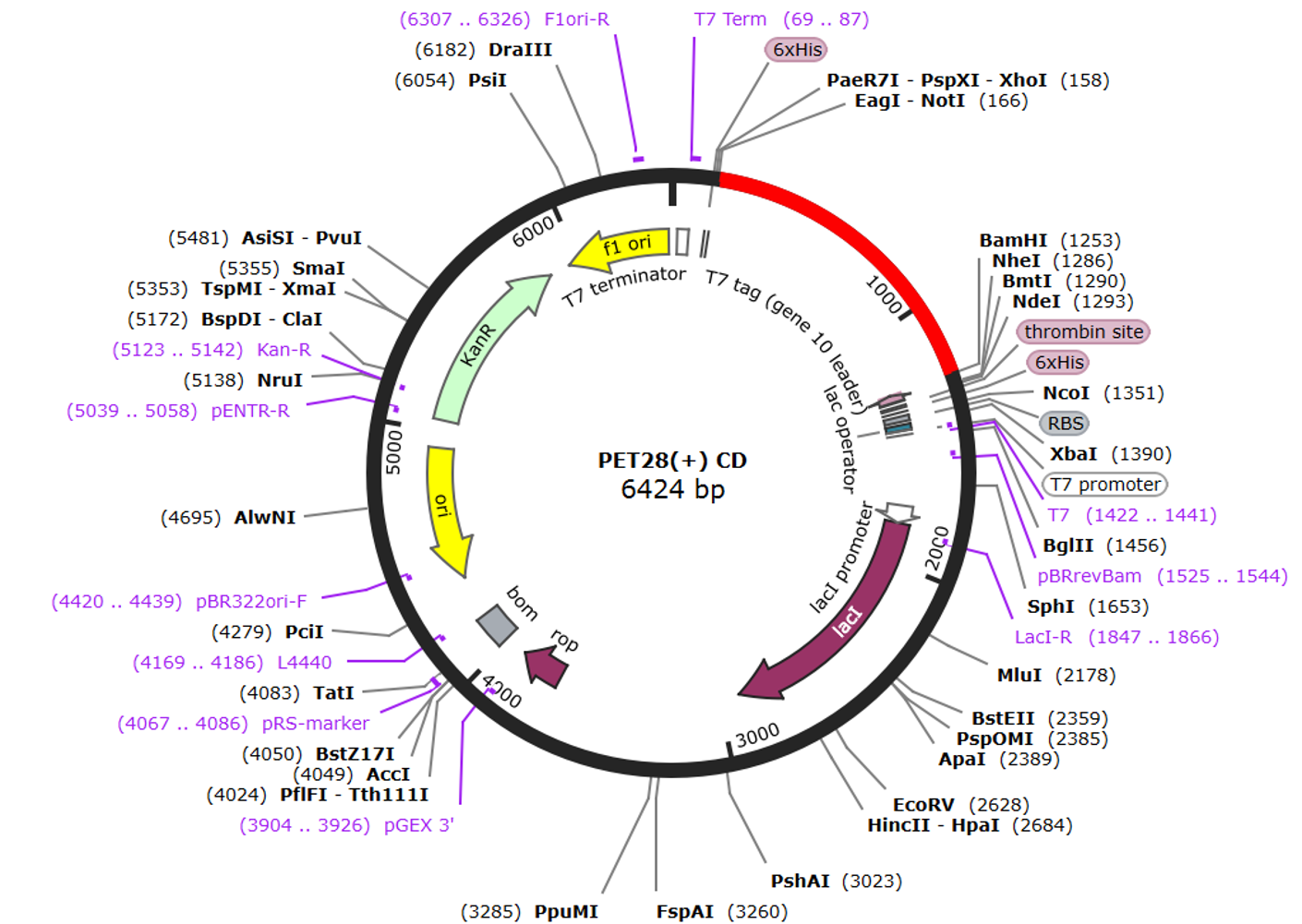

Supplement: S5 Fig — (TIF) [file pone.0293731.s008.tif]

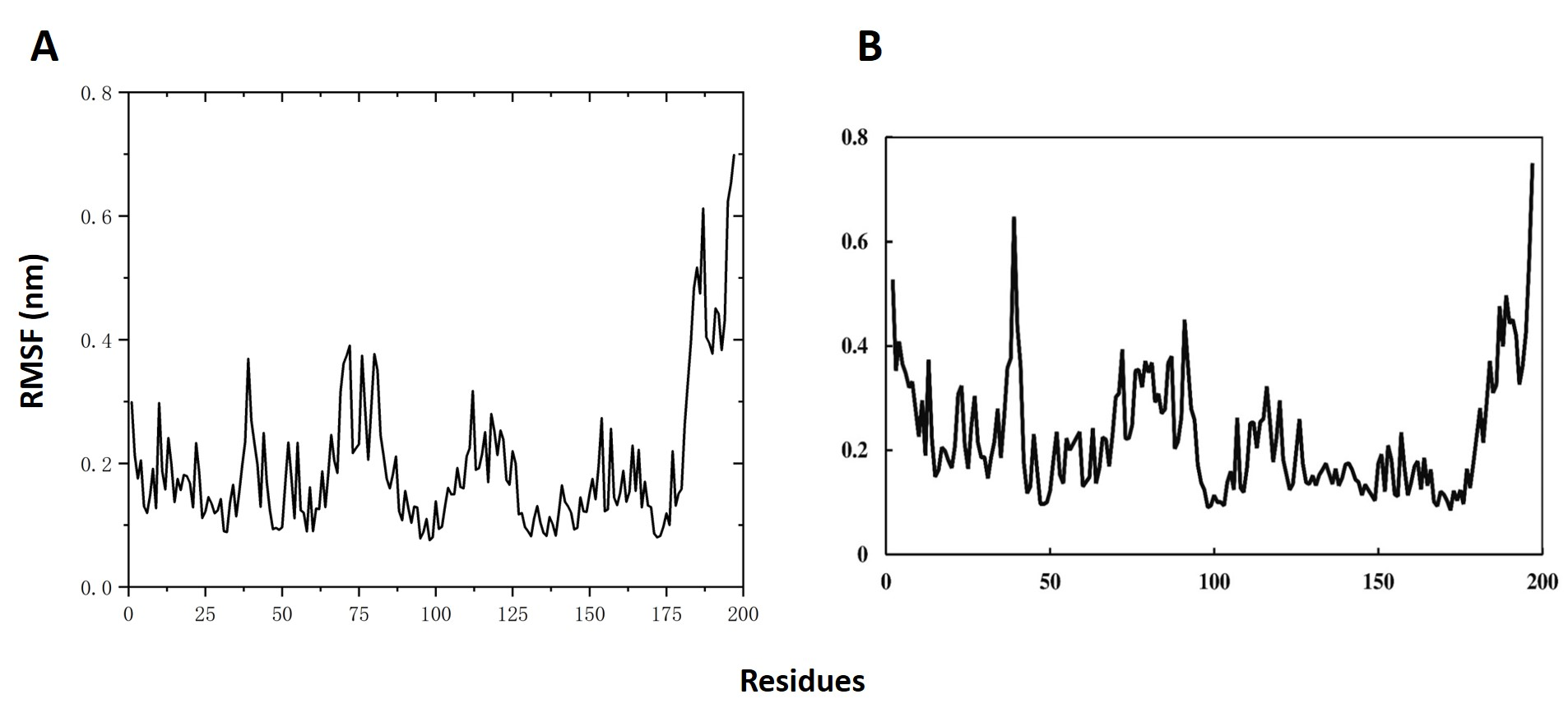

Supplement: S6 Fig — The individual RMSF graphs of protein (A) and protein-C7 complex (B). (TIF) [file pone.0293731.s009.tif]
