# Supplementary material for: Core-genome-mediated promising alternative drug and multi-epitope vaccine targets prioritization against infectious Clostridium difficile
Source: PLoS One. 2024 Jan 19;19(1):e0293731. doi: 10.1371/journal.pone.0293731 (PMC10798517; doi:10.1371/journal.pone.0293731)
Supplement: S1 Table — (DOCX) [file pone.0293731.s010.docx]

**S1 Table.** Overlapping B-cell, MHC-I, and MHC-II epitopes prediction using IEDB analysis resource and ABCPred server.

| Protein IDs | B Cell Epitopes | ABCpred Score | MHC-I Epitopes | 1c50 | MHC-II Epitopes | Ic50 |
| --- | --- | --- | --- | --- | --- | --- |
| CD630_18220 | IGTKAPEFTLEDKDGNKVSM | 0.88 | NKVSMSDFK | 75.21 | NKVSMSDFKGKKVVV | 102.1 |
|  | TPGCTRQACAFRNAYDGFKK | 0.84 | AFRNAYDGFK | 13.9 | ACAFRNAYDGFKKED | 10 |
|  | SIKSHQKFAEKHELPFILLS | 0.82 | AEKHELPFIL | 45 | EKHELPFILLSDPDL | 39 |
| CD630_27870 | TDNYAMKSVSKPDSDKKMYQ | 0.89 | LTDTDNYAM | 92.77 | DKTLLTDTDNYAMKS | 73 |
|  | DNTANPNREKSTLAYETNID | 0.88 | TLAYETNIDA | 32.4 | TLAYETNIDAYYLYE | 177.10 |
|  | YAFVVKDGSKSQGDLIDGLA | 0.87 | YAFVVKDGSK | 91.19 | DLKYAFVVKDGSKSQ | 46.6 |
| CD630_16310 | KETMKLHHDKHYQAYVDKLN | 0.84 | KETMKLHHDK | 55.7 | KETMKLHHDKHYQAY | 152.90 |
|  | LPYAYDALEPYIDKETMKLH | 0.81 | FKVKPLPYAY | 122.74 | NNKFKVKPLPYAYDA | 47.30 |
|  | IISQCITSFAFTPENNKFKV | 0.81 | FTPENNKFKV | 135.5 | TSFAFTPENNKFKVK | 22.50 |
| CD630_10170 | DSFSALDFKTDKRLRKALKN | 0.96 | DSFSALDFK | 9.23 | PEVYVFDDSFSALDF | 4.70 |
|  | TGSGKSTIANIIPRFFEIQS | 0.91 | GSTGSGKSTI | 52.91 | IGSTGSGKSTIANII | 145.6 |
|  | AVLMPIVMLIMNLGIVSIIW | 0.87 | FAVLMPIVM | 7.31 | AVLMPIVMLIMNLGI | 90.90 |
