# Supplementary material for: Core-genome-mediated promising alternative drug and multi-epitope vaccine targets prioritization against infectious Clostridium difficile
Source: PLoS One. 2024 Jan 19;19(1):e0293731. doi: 10.1371/journal.pone.0293731 (PMC10798517; doi:10.1371/journal.pone.0293731)
Supplement: S2 Table — (DOCX) [file pone.0293731.s011.docx]

**S2 Table.** Immunogenicity analysis of non-human homologous overlapping epitopes.

| Protein IDs | B-Cell Epitopes | Human Homology (E value> 0.05) | ABCPred Score | Antigenecity Score | Allergenicity  AllerTOP2.0 | ToxinPred |
| --- | --- | --- | --- | --- | --- | --- |
| CD630_18220 | IGTKAPEFTLEDKDGNKVSM | 4.7 | 0.88 | 1.6744 | Non-Allergen | Non-Toxin |
|  | TPGCTRQACAFRNAYDGFKK | 18 | 0.84 | 0.5882 | Non-Allergen | Non-Toxin |
|  | SIKSHQKFAEKHELPFILLS | 13 | 0.82 | 0.6647 | Non-Allergen | Non-Toxin |
| CD630_27870 | TDNYAMKSVSKPDSDKKMYQ | 4.7 | 0.89 | 0.4628 | Non-Allergen | Non-Toxin |
|  | DNTANPNREKSTLAYETNID | 0.84 | 0.88 | 0.8424 | Non-Allergen | Non-Toxin |
|  | YAFVVKDGSKSQGDLIDGLA | 9.2 | 0.87 | 0.5308 | Non-Allergen | Non-Toxin |
| CD630_16310 | KETMKLHHDKHYQAYVDKLN | 2.3 | 0.84 | 0.4809 | Non-Allergen | Non-Toxin |
|  | LPYAYDALEPYIDKETMKLH | 0.83 | 0.81 | 0.5648 | Non-Allergen | Non-Toxin |
|  | IISQCITSFAFTPENNKFKV | 3.3 | 0.81 | 0.5538 | Non-Allergen | Non-Toxin |
| CD630_10170 | DSFSALDFKTDKRLRKALKN | 0.15 | 0.96 | 0.5703 | Non-Allergen | Non-Toxin |
|  | TGSGKSTIANIIPRFFEIQS | 2.3 | 0.91 | 1.2061 | Non-Allergen | Non-Toxin |
|  | AVLMPIVMLIMNLGIVSIIW | 3.3 | 0.87 | 0.8525 | Non-Allergen | Non-Toxin |
