# Supplementary material for: Core-genome-mediated promising alternative drug and multi-epitope vaccine targets prioritization against infectious Clostridium difficile
Source: PLoS One. 2024 Jan 19;19(1):e0293731. doi: 10.1371/journal.pone.0293731 (PMC10798517; doi:10.1371/journal.pone.0293731)
Supplement: S3 Table — (DOCX) [file pone.0293731.s012.docx]

**S3 Table.** Prioritized MHC-I and MHC-II epitopes with the respective alleles.

| **Protein IDs** | **MHC-I Epitopes** | **Alleles** | **MHC-II Epitopes** | **Alleles** |
| --- | --- | --- | --- | --- |
| CD630_18220 | NKVSMSDFK | HLA-A*68:02 | NKVSMSDFKGKKVVV | HLA-DRB1*07:01 |
|  | AFRNAYDGFK | HLA-A*30:01 | ACAFRNAYDGFKKED | HLA-DRB5*01:01 |
|  | AEKHELPFIL | HLA-B*40:01 | EKHELPFILLSDPDL | HLA-DRB1*04:05 |
|  |  |  |  | HLA-DQA1*05:01 |
|  |  |  |  | HLA-DQB1*02:01 |
|  |  |  |  | HLA-DPA1*01:03 |
|  |  |  |  | HLA-DPB1*02:01 |
|  |  |  |  | HLA-DRB4*01:01 |
|  |  |  |  | HLA-DRB1*01:01 |
| CD630_27870 | LTDTDNYAM | HLA-B*35:01 | DKTLLTDTDNYAMKS | HLA-DRB1*03:01 |
|  |  | HLA-A*01:01 |  |  |
|  |  | HLA-A*68:01 |  |  |
|  |  | HLA-A*11:01 |  |  |
|  | TLAYETNIDA | HLA-A*30:02 | TLAYETNIDAYYLYE | HLA-DRB3*01:01 |
|  | YAFVVKDGSK | HLA-A*68:01 | DLKYAFVVKDGSKSQ | HLA-DRB5*01:01 |
|  |  | HLA-A*02:06 |  |  |
| CD630_16310 | KETMKLHHDK | HLA-A*11:01 | KETMKLHHDKHYQAY | HLA-DRB3*01:01 |
|  |  | HLA-A*03:01 |  |  |
|  |  | HLA-A*68:01 |  |  |
|  | FKVKPLPYAY | HLA-A*30:02 | NNKFKVKPLPYAYDA | HLA-DRB1*07:01 |
|  |  |  |  | HLA-DRB4*01:01 |
|  |  |  |  | HLA-DRB5*01:01 |
|  | FTPENNKFKV | HLA-A*68:02 | TSFAFTPENNKFKVK | HLA-DRB5*01:01 |
|  |  | HLA-A*02:06 |  |  |
| CD630_10170 | DSFSALDFK | HLA-A*68:01 | PEVYVFDDSFSALDF | HLA-DRB3*01:01 |
|  |  | HLA-A*68:01 |  |  |
|  |  | HLA-A*11:01 |  |  |
|  | GSTGSGKSTI | HLA-A*23:01 | IGSTGSGKSTIANII | HLA-DRB1*07:01 |
|  | FAVLMPIVM | HLA-B*35:01 | AVLMPIVMLIMNLGI | HLA-DRB4*01:01 |
