# Supplementary material for: Core-genome-mediated promising alternative drug and multi-epitope vaccine targets prioritization against infectious Clostridium difficile
Source: PLoS One. 2024 Jan 19;19(1):e0293731. doi: 10.1371/journal.pone.0293731 (PMC10798517; doi:10.1371/journal.pone.0293731)
Supplement: S4 Table — (DOCX) [file pone.0293731.s013.docx]

**S4 Table.** Molecular interactions between receptor molecules (Chain A) and vaccine construct (Chain B).

| **MHC-I-Vaccine Construct** | | |
| --- | --- | --- |
| 1. GLY 16 A <--> TYR 10 B  2. ARG 36 A <--> VAL 13 B  3. ARG 36 A <--> ARG 14 B  4. ARG 36 A <--> ARG 14 B  5. ASP 38 A <--> ARG 42 B  6. ASP 38 A <--> ARG 42 B  7. GLU 42 A <--> ARG 12 B  8. GLU 162 A <--> ILE 2 B  9. GLU 162 A <--> ILE 3 B  10. GLU 162 A <--> ASN 4 B  11. GLU 162 A <--> ARG 36 B  12. GLU 208 A <--> ARG 36 B | | |
| Number of hydrogen bonds: | 12 | |
| Number of salt bridges: | 5 | |
| Number of non-bonded contacts: | 146 | |
| **MHC-II-Vaccine Construct** | | |
| 1. GLU 4 A <--> ARG 17 B  2. GLU 4 A <--> ARG 17 B  3. HIS 5 A <--> ARG 17 B  4. ASP 27 A <--> LYS 8 B  5. ASP 29 A <--> ARG 12 B  6. ASP 29 A <--> ARG 12 B  7. ASP 29 A <--> ARG 12 B | | |
| Number of hydrogen bonds: | | 7 |
| Number of salt bridges: | | 3 |
| Number of non-bonded contacts: | | 148 |
| **TLR2-Vaccine Construct** | | |
| 1.ARG 321 A <--> 1 N GLY 1 B  2.TYR 323 A <--> 5 O GLY 1 B  3.TYR 323 A <--> 35 N THR 5 B  4.ASP 327 A <--> 122 NH1 ARG 12 B  5.LEU 328 A <--> 88 OH TYR 9 B  6.THR 330 A <--> 125 NH2 ARG 12 B  7.ASN 379 A <--> 102 OH TYR 10 B | | |
| Number of hydrogen bonds: | | 7 |
| Number of salt bridges: | | 3 |
| Number of non-bonded contacts: | | 118 |
| **TLR4-Vaccine Construct** | | |
| 1. HIS 458 A <--> LEU 6 B  2. GLN 505 A <--> TYR 10 B  3. GLN 505 A <--> ARG 14 B  4. GLN 505 A <--> ARG 14 B  5. GLN 507 A <--> VAL 13 B  6. ASN 530 A <--> ARG 42 B  7. ASN 531 A <--> ARG 42 B  8. LEU 553 A <--> ARG 42 B  9. GLN 578 A <--> GLU 28 B | | |
| Number of hydrogen bonds: | | 9 |
| Number of salt bridges: | | 1 |
| Number of non-bonded contacts: | | 117 |
