# Supplementary material for: Core-genome-mediated promising alternative drug and multi-epitope vaccine targets prioritization against infectious Clostridium difficile
Source: PLoS One. 2024 Jan 19;19(1):e0293731. doi: 10.1371/journal.pone.0293731 (PMC10798517; doi:10.1371/journal.pone.0293731)
Supplement: S5 Table — (DOCX) [file pone.0293731.s014.docx]

**S5 Table.** Codon Optimization of vaccine construct.

| **Vaccine Peptide Sequence** | **Improved DNA** | **GC Content** | **CAI Index** |
| --- | --- | --- | --- |
| EAAAKGIINTLQKYYCRVRGGRCAVLSCLPKEEQIGKCSTRGRKCCRRKKEAAAKAKFVAAWTLKAAAGGGSIGTKAPEFTLEDKDGNKVSMGGGSTPGCTRQACAFRNAYDGFKKGGGSSIKSHQKFAEKHELPFILLSGGGSTDNYAMKSVSKPDSDKKMYQGGGSDNTANPNREKSTLAYETNIDGGGSYAFVVKDGSKSQGDLIDGLAGGGSKETMKLHHDKHYQAYVDKLNGGGSLPYAYDALEPYIDKETMKLHGGGSIISQCITSFAFTPENNKFKVGGGSDSFSALDFKTDKRLRKALKNGGGSAKFVAAWTLKAAAGGGSTGSGKSTIANIIPRFFEIQSHEYGAEALERAGAVLMPIVMLIMNLGIVSIIWHEYGAEALERAGAKFVAAWTLKAAAGGGS | GAGGCCGCCGCCAAGGCCAAGTTCGTGGCCGCCTGGACCCTGAAGGCCGCCGCCGGCGGCGGCAGCATCGGCACCAAGGCCCCCGAGTTCACCCTGGAGGACAAGGACGGCAACAAGGTGAGCATGGGCGGCGGCAGCACCCCCGGCTGCACCCGCCAGGCCTGCGCCTTCCGCAACGCCTACGACGGCTTCAAGAAGGGCGGCGGCAGCAGCATCAAGAGCCACCAGAAGTTCGCCGAGAAGCACGAGCTGCCCTTCATCCTGCTGAGCGGCGGCGGCAGCACCGACAACTACGCCATGAAGAGCGTGAGCAAGCCCGACAGCGACAAGAAGATGTACCAGGGCGGCGGCAGCGACAACACCGCCAACCCCAACCGCGAGAAGAGCACCCTGGCCTACGAGACCAACATCGACGGCGGCGGCAGCTACGCCTTCGTGGTGAAGGACGGCAGCAAGAGCCAGGGCGACCTGATCGACGGCCTGGCCGGCGGCGGCAGCAAGGAGACCATGAAGCTGCACCACGACAAGCACTACCAGGCCTACGTGGACAAGCTGAACGGCGGCGGCAGCCTGCCCTACGCCTACGACGCCCTGGAGCCCTACATCGACAAGGAGACCATGAAGCTGCACGGCGGCGGCAGCATCATCAGCCAGTGCATCACCAGCTTCGCCTTCACCCCCGAGAACAACAAGTTCAAGGTGGGCGGCGGCAGCGACAGCTTCAGCGCCCTGGACTTCAAGACCGACAAGCGCCTGCGCAAGGCCCTGAAGAACGGCGGCGGCAGCGCCAAGTTCGTGGCCGCCTGGACCCTGAAGGCCGCCGCCGGCGGCGGCAGCACCGGCAGCGGCAAGAGCACCATCGCCAACATCATCCCCCGCTTCTTCGAGATCCAGAGCCACGAGTACGGCGCCGAGGCCCTGGAGCGCGCCGGCGCCGTGCTGATGCCCATCGTGATGCTGATCATGAACCTGGGCATCGTGAGCATCATCTGGCACGAGTACGGCGCCGAGGCCCTGGAGCGCGCCGGCGCCAAGTTCGTGGCCGCCTGGACCCTGAAGGCCGCCGCCGGCGGCGGCAGC | 67.96 | 0.95 |
