# Supplementary material for: Core-genome-mediated promising alternative drug and multi-epitope vaccine targets prioritization against infectious Clostridium difficile
Source: PLoS One. 2024 Jan 19;19(1):e0293731. doi: 10.1371/journal.pone.0293731 (PMC10798517; doi:10.1371/journal.pone.0293731)
Supplement: S6 Table — (DOCX) [file pone.0293731.s015.docx]

**S6 Table.** Druggability analysis of shortlisted non-human homologous pathogenic essential proteins.

| **Gene symbols** | **UniprotKB** | **PDB homolog ID's**  **(%Identity)** | **Protein Name** | **PockDrug score > 0.5 (Residues in pocket)** | **QMEAN > −4** | **ERRAT Quality Factor** | **Molecular weight (Kilo-daltons)** | **Node degree (K)≥5 (STRING analysis)** | **Ramachandran score PROCHECK Results** |
| --- | --- | --- | --- | --- | --- | --- | --- | --- | --- |
| CD630_00220 | Q18CA6 | 2xex  (38%) | elongation factor G | 0.81(64) | 0.76 | 94.8505 | 72.04 | 10 | 89.8% |
| CD630_00360 | Q18CB6 | 1ni4  (37%) | acetoin dehydrogenase E1 component subunit alpha | 1.0 (18) | 0.77 | 88.6731 | 35.67 | 9.09 | 92.9% |
| CD630_00370 | Q18CC3 | 1ik6  (50%) | acetoin dehydrogenase E1 component subunit beta | 0.96(115) | 0.74 | 91.4684 | 35.63 | 8.73 | 90.7% |
| CD630_00520 | Q18CD5 | 1u0b  (50%) | cysteine--tRNA synthetase | 0.77 (15) | 0.76 | 95.7494 | 53.93 | 6.18 | 90.5 |
| CD630_00910 | Q18CH9 | 5g41  62.96% | adenylate kinase | 0.76(52) | 0.88 | 94.1176 | 23.7 | 6.55 | 96.7% |
| CD630_01010 | Q18CI9 | 4mki  61.92% | cobalt ABC transporter ATP-binding protein | 0.99(15) | 0.84 | 96.1749 | 32.02 | 6.73 | 91% |
| CD630_01200 | Q18CK9 | 4amv (47.5%) | glucosamine--fructose-6-phosphate aminotransferase | 0.97(109) | 0.77 | 94.9622 | 67.43 | 5.64 | 91.5% |
| CD630_01840 | Q18CS8 | 6ypo  (48.3%) | aspartate carbamoyltransferase | 0.92(14) | 0.75 | 90.2746 | 34.7 | 7.45 | 90.7% |
| CD630_01980 | Q18CT8 | 5tw7  (53.65%) | GMP synthase | 0.92(45) | 0.76 | 91.3442 | 57.33 | 6 | 90.5% |
| CD630_02180 | Q18CV6 | 1o4v  (48%) | 5-(carboxyamino)imidazole ribonucleotide mutase | 0.74(46) | 0.77 | 100 | 17.12 | 9.09 | 89.2% |
| CD630_02190 | Q18CV5 | 2z02  (54.59%) | phosphoribosylaminoimidazolesuccinocarboxamide synthase | 0.97(15) | 0.83 | 88.8889 | 26.7 | 8.91 | 91.1% |
| CD630_02230 | Q18CW0 | 4ehi  (43.54%) | bifunctional phosphoribosylaminoimidazolecarboxamide formyltransferase/IMP cyclohydrolase | 0.86(122) | 0.71 | 91.0042 | 57.11 | 10 | 90.9% |
| CD630_03270 | Q18D67 | 3gfo  (41.7%) | cobalt ABC transporter ATP-binding protein CbiO | 1.0(14) | 0.76 | 93.722 | 31.36 | 8 | 92% |
| CD630_03940 | Q188H7 | 7jp2  (39.63%) | D-lactate dehydrogenase | 0.88(53) | 0.74 | 97.0497 | 36.52 | 8 | 90.8% |
| CD630_04590 | Q188Q2 | 5lj6  (39.56%) | ABC transporter ATP-binding protein | 0.81(52) | 0.72 | 95.0472 | 25.37 | 8.18 | 91.7% |
| CD630_04840 | Q188S6 | 5lj6  (43%) | ABC transporter ATP-binding protein | 0.58(78) | 0.75 | 92.2705 | 24.29 | 6.91 | 93.8% |
| CD630_07000 | Q189P6 | 2rhq  37.61% | phenylalanine--tRNA ligase subunit beta | 0.99(22) | 0.72 | 93.3884 | 89.24 | 9.45 | 88.4% |
| CD630_07230 | Q189R5 | 6uzi  (41.73%) | bifunctional carbon monoxide dehydrogenase/acetyl-CoA synthase dihydrolipoyl dehydrogenase subunit | 0.97(57) | 0.79 | 91.2442 | 49.14 | 6 | 92.2% |
| CD630_07500 | Q189U7 | 4i62  36% | amino acid family ABC transporter substrate-binding protein | 0.97(23) | 0.75 | 99.1031 | 29.54 | 6 | 93.5% |
| CD630_07520 | Q189V0 | 2ouk  65.42% | amino acid family ABC transporter ATP-binding protein | 0.96(14) | 0.83 | 96.1039 | 26.62 | 5.64 | 91.2% |
| CD630_07530 | Q189U9 | 4r5f  37% | cysteine desulfurase | 0.53(49) | 0.75 | 90.7483 | 42.74 | 6 | 91.9% |
| CD630_07560 | Q189V2 | 5j62  100% | nitroreductase | 0.98(24) | 0.87 | 97.9381 | 23.75 | 5.09 | 93.7% |
| CD630_08180 | Q18A15 | 4ze5  44.4% | 6-phospho-beta-glucosidase | 0.78(48) | 0.75 | 93.054 | 54.66 | 7.09 | 88% |
| CD630_08340 | Q18A33 | 6lky  54.28% | Isocitrate dehydrogenase | 0.71(45) | 0.82 | 96.7033 | 36.12 | 7.09 | 91% |
| CD630_08560 | Q18A52 | 4fwi  38% | ABC transporter ATP-binding protein | 0.94(42) | 0.69 | 96.7949 | 37.79 | 7.45 | 88.6% |
| CD630_08820 | Q18A75 | 6shn  43% | glucose-1-phosphate adenylyltransferase | 0.98(16) | 0.73 | 90.798 | 43.42 | 6.18 | 86.7% |
| CD630_09000 | Q18A90 | 7ahe  38.67% | glycine betaine/carnitine/choline ABC transporter ATP-binding protein | 0.99(19) | 0.64 | 91.9162 | 42.38 | 7.09 | 87.5% |
| CD630_09920 | Q18AJ0 | 3udo  49.14% | 3-isopropylmalate dehydrogenase | 1.0(14) | 0.79 | 95.9712 | 39.37 | 9.09 | 90% |
| CD630_09940 | Q18AJ4 | 2dr1  36.26% | serine-pyruvate aminotransferase | 0.99(14) | 0.78 | 90.1876 | 40.05 | 6.18 | 88.8% |
| CD630_10050 | Q18AK8 | 5cee  60.77% | NAD-dependent malic enzyme | 0.95(24) | 0.81 | 93.4228 | 42.72 | 8.18 | 87.5% |
| CD630_11820 | Q18B45 | 4jro  58.30% | 3-oxoacyl-ACP reductase | 0.95(32) | 0.82 | 98.7408 | 26.76 | 5.82 | 92.4% |
| CD630_11840 | Q18B43 | 4ls5  49.51% | 3-oxoacyl-ACP synthase | 1.0(30) | 0.81 | 85.2174 | 44.05 | 8.73 | 91.4% |
| CD630_12240 | Q18B85 | 3fuc  48.89% | purine nucleoside phosphorylase | 0.96(52) | 0.78 | 91.5475 | 29.32 | 5.64 | 92.3% |
| CD630_12790 | Q18BE4 | 4r5f  49.21% | cysteine desulfurase | 0.84(171) | 0.79 | 97.9564 | 43.82 | 6 | 90.9% |
| CD630_13090 | Q18BH4 | 3jcj  53.37% | translation initiation factor IF-2 | 0.80 (15) | 0.62 | 83.7587 | 69.72 | 8.55 | 91.8% |
| CD630_14450 | Q18BW6 | 6qur  64.21% | para-aminobenzoate/anthranilate synthase glutamine amidotransferase component II | 0.97(14) | 0.84 | 86.7816 | 21.88 | 7.27 | 92.6% |
| CD630_15210 | Q18C40 | 1h3e  45.64% | tyrosine--tRNA ligase | 1.0(14) | 0.74 | 97.9566 | 45.74 | 8.73 | 92.6% |
| CD630_15280 | Q18C48 | 5lj6 46.40% | ABC transporter ATP-binding protein | 0.61(84) | 0.77 | 98.1352 | 25.4 | 7.64 | 92.1% |
| CD630_15320 | Q18C53 | 5lj6  37.55% | ABC transporter ATP-binding protein | 0.95(34) | 0.72 | 92.6606 | 28.24 | 6.36 | 92.0% |
| CD630_15370 | Q18C58 | 4ylf  59.74% | oxidoreductase | 0.88(39) | 0.76 | 95.9732 | 49.6 | 7.64 | 88.7% |
| CD630_15660 | Q186C0 | 1t9c  41.37% | acetolactate synthase large subunit | 0.87(85) | 0.75 | 92.209 | 62.43 | 9.09 | 88.0% |
| CD630_16570 | Q186L1 | 1v5v  38.42% | bifunctional glycine dehydrogenase/aminomethyl transferase | 0.61(31) | 0.71 | 94.8034 | 91.92 | 8.18 | 87.5% |
| CD630_16910 | Q186P9 | 6gnc  60.00% | thioredoxin-disulfide reductase | 0.79(33) | 0.82 | 97.3166 | 32.6 | 7.27 | 91.6% |
| CD630_17530 | Q186W1 | 4yer  36.65% | multidrug family ABC transporter ATP-binding protein | 0.8 (132) | 0.65 | 88.0998 | 31.45 | 5.27 | 89.5% |
| CD630_17760 | Q186Y5 | 2ouk  (48.2%) | amino acid family ABC transporter ATP-binding protein | 0.95(26) | 0.75 | 93.4426 | 25.23 | 5.64 | 89.1% |
| CD630_18050 | Q187B5 | 6nu7  35.56% | sucrose-6-phosphate hydrolase | 1.0(15) | 0.72 | 74.9739 | 57.66 | 10 | 84.6% |
| CD630_18350 | Q187E5 | 1umf  39.07% | chorismate synthase | 0.81(95) | 0.66 | 87.4443 | 39.04 | 8.91 | 85.1% |
| CD630_19380 | Q187P8 | 2vpq  55.28% | acetyl-CoA carboxylase biotin carboxylase subunit | 0.82 (14) | 0.81 | 94.8956 | 51.33 | 6.91 | 91.2% |
| CD630_19470 | Q187Q7 | 5lj6  41.63% | ABC transporter ATP-binding protein | 1.0(17) | 0.75 | 97.6526 | 25.26 | 6.18 | 93.3% |
| CD630_19550 | Q187R6 | 5lj6  42.67% | ABC transporter ATP-binding protein | 0.94(36) | 0.71 | 97.5962 | 24.92 | 7.64 | 91.4% |
| CD630_20310 | Q187Z9 | 2e54  48.55% | acetylornithine aminotransferase | 1.0(14) | 0.81 | 93.0201 | 43.79 | 7.27 | 88.3% |
| CD630_20590 | Q188C8 | 5bnz  58.21% | glutamine--tRNA ligase | 0.6(32) | 0.82 | 87.5696 | 64.29 | 7.82 | 92.0% |
| CD630_21160 | Q185Q6 | 4zcm  50.83% | GTP-binding protein BipA | 0.65(45) | 0.70 | 87.2727 | 68.04 | 6.91 | 87.1% |
| CD630_21170 | Q185Q5 | 7jyp  41.28% | thioredoxin reductase | 0.75(64) | 0.77 | 96.8804 | 32.57 | 6 | 90.2% |
| CD630_22060 | Q185Z3 | 6k10  44.47% | aldehyde dehydrogenase | 0.92(84) | 0.81 | 92.0135 | 51.9 | 8.55 | 94.6% |
| CD630_23560 | Q185M7 | 5uth  45.57% | thioredoxin reductase | 0.92(59) | 0.75 | 92.6544 | 34.15 | 6.18 | 87.7% |
| CD630_23710 | Q185P0 | 1knp  39.06% | L-aspartate oxidase | 1.0(15) | 0.74 | 93.0108 | 48.54 | 6 | 87.4% |
| CD630_23820 | Q181W7 | 3rq1  44.69% | pyridoxal phosphate-dependent transferase | 0.82(36) | 0.80 | 90.8416 | 47.31 | 7.27 | 93.1% |
| CD630_24610 | Q182E8 | 7kzi  59.76% | chaperone DnaK | 0.94(49) | 0.73 | 94.2804 | 66.48 | 7.82 | 92.2% |
| CD630_24670 | Q182F4 | 3jce  58.14% | elongation factor 4 | 0.56(10) | 0.75 | 80.7018 | 67.31 | 10 | 83% |
| CD630_25140 | Q182J9 | 6lut  39.87% | threonine dehydratase II | 0.91(14) | 0.76 | 85.6905 | 42.99 | 5.64 | 93.4% |
| CD630_25240 | Q182L2 | 5deo  39.60% | nicotinic acid mononucleotide adenylyltransferase | 0.97(18) | 0.68 | 92.3885 | 26.69 | 7.82 | 90.5% |
| CD630_25340 | Q182M1 | 5lj6  46.46% | ABC transporter ATP-binding protein | 0.93(15) | 0.74 | 97.2093 | 25.15 | 7.64 | 92.0% |
| CD630_25770 | Q182R6 | 3osu  40.00% | 3-oxoacyl-ACP reductase | 0.87(30) | 0.77 | 91.3183 | 26.74 | 5.27 | 91.9% |
| CD630_25840 | Q182S2 | 4iqf  45.31% | methionyl-tRNA formyltransferase | 0.99(29) | 0.77 | 93.9394 | 34.46 | 6.73 | 86.9% |
| CD630_25880 | Q182S8 | 3tau  48.00% | guanylate kinase | 0.98(18) | 0.72 | 89.5013 | 23.37 | 5.82 | 90.8% |
| CD630_26300 | Q182W5 | 1z82  42.11% | glycerol-3-phosphate dehydrogenase | 0.92(45) | 0.76 | 96.9793 | 36.96 | 6.18 | 93.7% |
| CD630_26700 | Q183A4 | 4fwi  36.01% | ABC transporter ATP-binding protein | 0.94(38) | 0.67 | 92.926 | 36.43 | 7.64 | 89.4% |
| CD630_26710 | Q183A5 | 4fwi  37.42% | ABC transporter ATP-binding protein | 1.0(33) | 0.71 | 94.1935 | 36.27 | 7.45 | 85.7% |
| CD630_26910 | Q183C4 | 2geb  61.63% | hypoxanthine phosphoribosyltransferase | 0.97(20) | 0.83 | 92.6493 | 19.58 | 8.18 | 93.5% |
| CD630_27140 | Q183E8 | 1kvq  55.19% | UDP-glucose 4-epimerase | 0.84(103) | 0.81 | 96.0184 | 37.48 | 6 | 91.8% |
| CD630_27390 | Q183H4 | 6wom  54.15% | aspartate--tRNA ligase | 0.99(18) | 0.76 | 93.5739 | 67.5 | 9.82 | 91.0% |
| CD630_27710 | Q183L0 | 2y0e  43.49% | UDP-glucose 6-dehydrogenase | 1.0(33) | 0.78 | 97.981 | 48.13 | 6.73 | 91.4% |
| CD630_28050 | Q183N8 | 6blb  61.21% | Holliday junction ATP-dependent DNA helicase RuvB | 0.31(19) | 0.83 | 100 | 37.78 | 5.27 | 94.9% |
| CD630_28170 | Q183Q0 | 6quz  40.31% | multidrug family ABC transporter ATP-binding protein/permease | 0.91(25) | 0.73 | 95.1957 | 66.73 | 6.73 | 92.3% |
| CD630_28180 | Q183Q4 | 6quz  37.96% | multidrug family ABC transporter ATP-binding protein/permease | 1.0(22) | 0.70 | 96.3504 | 64.31 | 6.18 | 94.8% |
| CD630_28430 | Q183S7 | 5gxf  38.53% | zinc-binding dehydrogenase | 0.87(39) | 0.75 | 95.0637 | 36.08 | 9.82 | 92.1% |
| CD630_29550 | Q184E3 | 3vr4  72.59% | V-type ATP synthase subunit B | 1.0(14) | 0.86 | 97.2727 | 50.57 | 9.64 | 92.3% |
| CD630_29850 | Q184H5 | 5lj6  40.63% | ABC transporter ATP-binding protein | 0.86(15) | 0.73 | 93.3333 | 27.53 | 6.91 | 91.5% |
| CD630_29940 | Q184I6 | 6qo5  54.60% | ribonucleoside-diphosphate reductase subunit beta | 0.59(19) | 0.81 | 99.8214 | 37.48 | 8.73 | 97.4% |
| CD630_30090 | Q184K0 | 6kgy  42.37% | pyridine nucleotide-disulfide oxidoreductase | 0.81(54) | 0.76 | 93.3025 | 51.2 | 6.73 | 86.6% |
| CD630_30560 | Q184P3 | 4dde  52% | ABC transporter ATP-binding protein | 0.82(77) | 0.81 | 91.1379 | 66.13 | 6.73 | 90.6% |
| CD630_30910 | Q184S7 | 5brp  52.82% | trehalose-6-phosphate hydrolase | 0.94(15) | 0.82 | 91.1927 | 66.13 | 8 | 89.1% |
| CD630_30950 | Q184T2 | 4dde  52.03% | 6-phospho-beta-glucosidase | 0.82(77) | 0.81 | 91.1379 | 54.91 | 9.82 | 90.6% |
| CD630_31240 | Q184W2 | 4ipl  53.13% | 6-phospho-beta-glucosidase | 0.81(49) | 0.78 | 89.0811 | 55.12 | 9.64 | 90.1% |
| CD630_32170 | Q17ZW1 | 3zqj  38.39% | ABC transporter ATP-binding protein | 0.98(14) | 0.63 | 85.0914 | 82.74 | 8.73 | 86.7% |
| CD630_32310 | Q17ZX6 | 3o7m  45.45% | hypoxanthine phosphoribosyltransferase | 0.92(27) | 0.77 | 93.6923 | 20.49 | 8.36 | 91.5% |
| CD630_32560 | Q180A1 | 1ivs  45.61% | valine--tRNA ligase | 1.0(16) | 0.72 | 91.8391 | 103.01 | 8.91 | 86.9% |
| CD630_32610 | Q180A5 | 4u00  36.40% | phosphate ABC transporter ATP-binding protein PstB | 0.98(14) | 0.67 | 86.0759 | 28.55 | 5.45 | 89.1% |
| CD630_34170 | Q180R5 | 1q12  45.37% | sugar family ABC transporter ATP-binding protein | 0.99(20) | 0.69 | 90 | 40.54 | 5.27 | 84.6% |
| CD630_34590 | Q180V8 | 6yaj  66.24% | transketolase pyridine binding subunit | 0.84(15) | 0.84 | 90.9699 | 33.85 | 7.45 | 90.9% |
| CD630_34680 | Q180W5 | 5hkk  75.22% | ATP synthase subunit beta | 1.0(15) | 0.81 | 95.881 | 49.8 | 7.45 | 91.8% |
| CD630_34880 | Q180Y5 | 5j49  48.62% | UTP--glucose-1-phosphate uridylyltransferase | 0.5(128) | 0.76 | 94.9866 | 36.37 | 6.36 | 93.3% |
| CD630_35150 | Q181B4 | 1hm9  47.22% | bifunctional N-acetylglucosamine-1-phosphate uridyltransferase/glucosamine-1-phosphate acetyltransferase | 0.85(15) | 0.79 | 83.0445 | 50.37 | 7.09 | 89.4% |
| CD630_35300 | Q181C9 | 1q12  42.77% | iron family ABC transporter ATP-binding protein | 0.94(24) | 0.68 | 91.1576 | 37.15 | 6.55 | 80.3% |
| CD630_35400 | Q181D9 | 4qrd  54.04% | methionine--tRNA ligase | 0.89(37) | 0.78 | 91.9588 | 74.11 | 8.91 | 92.1% |
| CD630_35520 | Q181F2 | 3a74  58.39% | lysine--tRNA ligase | 0.91(22) | 0.82 | 93.1478 | 58.47 | 7.27 | 91.7% |
| CD630_35850 | Q181I7 | 5lj6  43.75% | ABC transporter ATP-binding protein | 1.0(14) | 0.75 | 98.5882 | 25.39 | 8.91 | 92.0% |
| CD630_35890 | Q181J1 | 1a9x  48.14% | carbamoyl-phosphate synthase small subunit | 0.7(15) | 0.81 | 90.0293 | 38.77 | 8.36 | 89.7% |
