# Supplementary material for: Core-genome-mediated promising alternative drug and multi-epitope vaccine targets prioritization against infectious Clostridium difficile
Source: PLoS One. 2024 Jan 19;19(1):e0293731. doi: 10.1371/journal.pone.0293731 (PMC10798517; doi:10.1371/journal.pone.0293731)
Supplement: S7 Table — (DOCX) [file pone.0293731.s016.docx]

**S7 Table.** Top 10 hit molecules obtained from virtual screening using Pharmit Server.

| **Compounds** | **(MolPort IDs)** | **Formula** | **Structure** |
| --- | --- | --- | --- |
| C1 | MolPort-044-559-927 | C17H20N4O9S | 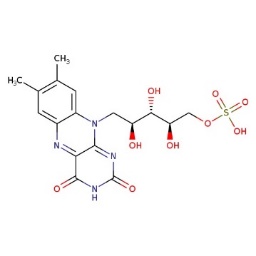 |
| C2 | MolPort-044-724-190 | C17H21N4O9P | 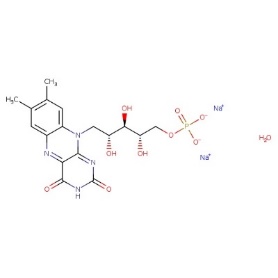 |
| C3 | MolPort-003-939-021 | C17H20N4NaO9P | 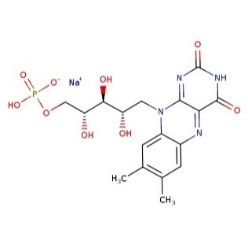 |
| C4 | MolPort-021-783-318 | C17H20N4NaO9P | 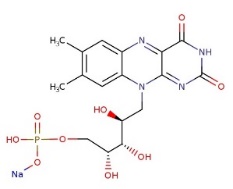 |
| C5 | MolPort-039-136-733 | C17H20N4NaO9P | 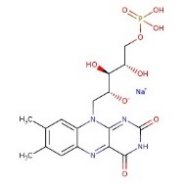 |
| C6 | MolPort-003-934-329 | C17H20N4O6 | 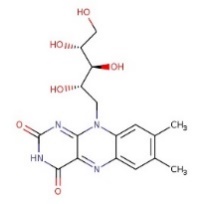 |
| C7 | MolPort-001-785-965 | C17H20N4O6 | 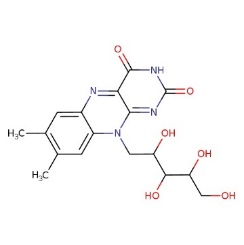 |
| C8 | MolPort-004-964-255 | C17H20N4NaO9P | 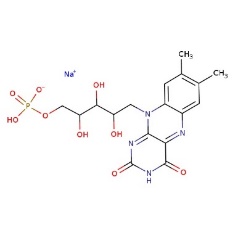 |
| C9 | MolPort-003-666-643 | C17H20N4O6 | 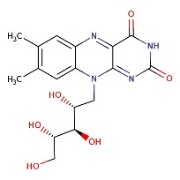 |
| C10 | MolPort-044-561-302 | C18H23N5O6 | 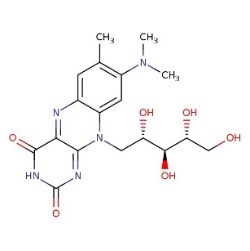 |
