# Supplementary material for: Core-genome-mediated promising alternative drug and multi-epitope vaccine targets prioritization against infectious Clostridium difficile
Source: PLoS One. 2024 Jan 19;19(1):e0293731. doi: 10.1371/journal.pone.0293731 (PMC10798517; doi:10.1371/journal.pone.0293731)
Supplement: S8 Table — (DOCX) [file pone.0293731.s017.docx]

**S8 Table.** CB-Dock energy, physicochemical, pharmacokinetics, and medicinal chemistry properties prediction of the molecules obtained from virtual screening using CB-Dock and SwissADME servers.

|  | **Compounds**  **(MolPort IDs)** | **Molecular Weight (g/mol)** | **CB-Dock**  **(Vina score)** | **H-Bond Acceptors** | **H-Bond Donors** | **TPSA**  **(Å2)** | **Consensus**  **Log P_o/w_** | **Molar Refractivity** | **GI**  **Absorption** | **Log S** | **BBB**  **Permeant** | **P-gp**  **Substrate** | **Log Kp (skin permeation) cm/s** | **Drug-likeness based on Lipinski rule** | **Bioavailability**  **Score** | **Synthetic accessibility** |
| --- | --- | --- | --- | --- | --- | --- | --- | --- | --- | --- | --- | --- | --- | --- | --- | --- |
| C1 | MolPort-044-559-927 | 456.43 | -8.8 | 11 | 5 | 213.31 | -0.82 | 107.45 | Low | -1.38  Very soluble | No | No | -10.39 | Yes  1 violation: N or O >10 | 0.11 | 4.23 |
| C2 | MolPort-044-724-190 | 456.34 | -7.8 | 11 | 6 | 217.90 | -1.03 | 107.90 | low | -0.93  Very soluble | No | No | -10.90 | No  2 violations: N or O >10, NH or OH >5 | 0.11 | 4.41 |
| C3 | MolPort-003-939-021 | 478.33 | -8.5 | 11 | 5 | 220.73 | -4.52 | 106.37 | Low | -1.05  Very soluble | No | Yes | -11.04 | Yes  1 violation: N or O >10 | 0.11 | 4.42 |
| C4 | MolPort-021-783-318 | 478.33 | -8.8 | 11 | 5 | 206.90 | -1.15 | 106.77 | Low | -1.42  Very soluble | No | Yes | -10.55 | Yes  1 violation: N or O >10 | 0.11 | 4.50 |
| C5 | MolPort-039-136-733 | 478.33 | -8.7 | 11 | 5 | 220.73 | -3.41 | 106.37 | Low | -1.05  Very soluble | No | Yes | -11.04 | Yes  1 violation: N or O >10 | 0.11 | 4.42 |
| C6 | MolPort-003-934-329 | 376.36 | -9.5 | 8 | 5 | 161.56 | -0.19 | 96.99 | Low | -1.31  Very soluble | No | No | -9.63 | Yes | 0.55 | 3.84 |
| C7 | MolPort-001-785-965 | 376.36 | -9.3 | 8 | 5 | 161.56 | -0.40 | 96.99 | Low | -1.31  Very soluble | No | No | -9.63 | Yes | 0.55 | 3.84 |
| C8 | MolPort-004-964-255 | 478.33 | -8.4 | 11 | 5 | 220.73 | -4.53 | 106.37 | Low | -1.05  Very soluble | No | Yes | -11.04 | Yes  1 violation: N or O >10 | 0.11 | 4.42 |
| C9 | MolPort-003-666-643 | 376.36 | -10 | 8 | 5 | 161.56 | -0.42 | 96.99 | Low | -1.31  Very soluble | No | No | -9.63 | Yes | 0.55 | 3.84 |
| C10 | MolPort-044-561-302 | 405.41 | -8.1 | 8 | 5 | 164.80 | -0.53 | 106.23 | Low | -1.24  Very soluble | No | No | -9.98 | Yes  1 violation: N or O >10 | 0.55 | 4.07 |
